# A novel defined risk signature of cuproptosis-related long non-coding RNA for predicting prognosis, immune infiltration, and immunotherapy response in lung adenocarcinoma

**DOI:** 10.3389/fphar.2023.1146840

**Published:** 2023-08-21

**Authors:** Chao Ma, Feng Li, Zhuoyu Gu, Yang Yang, Yu Qi

**Affiliations:** Department of Thoracic Surgery, The First Affiliated Hospital of Zhengzhou University, Zhengzhou, China

**Keywords:** lung adenocarcinoma, lncRNA, signature, cuproptosis, copper, tumor microenvironment

## Abstract

**Background:** Cuproptosis is a newly discovered non-apoptotic form of cell death that may be related to the development of tumors. Nonetheless, the potential role of cuproptosis-related lncRNAs in tumor immunity formation and patient-tailored treatment optimization of lung adenocarcinoma (LUAD) is still unclear.

**Methods:** RNA sequencing and survival data of LUAD patients were downloaded from The Cancer Genome Atlas (TCGA) database for model training. The patients with LUAD in GSE29013, GSE30219, GSE31210, GSE37745, and GSE50081 were used for validation. The proofed cuproptosis-related genes were extracted from the previous studies. The Pearson correlation was applied to select cuproptosis-related lncRNAs. We chose differentially expressed cuproptosis-related lncRNAs in the tumor and normal tissues and allowed them to go to a Cox regression and a LASSO regression for a lncRNA signature that predicts the LUAD prognosis. Kaplan–Meier estimator, Cox model, ROC, tAUC, PCA, nomogram predictor, decision curve analysis, and real-time PCR were further deployed to confirm the model’s accuracy. We examined this model’s link to other regulated cell death forms. Applying TMB, immune-related signatures, and TIDE demonstrated the immunotherapeutic capabilities of signatures. We evaluated the relationship of our signature to anticancer drug sensitivity. GSEA, immune infiltration analysis, and function experiments further investigated the functional mechanisms of the signature and the role of immune cells in the prognostic power of the signature.

**Results:** An eight-lncRNA signature (TSPOAP1-AS1, AC107464.3, AC006449.7, LINC00324, COLCA1, HAGLR, MIR4435-2HG, and NKILA) was built and demonstrated owning prognostic power by applied to the validation cohort. Each signature gene was confirmed differentially expressed in the real world by real-time PCR. The eight-lncRNA signature correlated with 2321/3681 (63.05%) apoptosis-related genes, 11/20 (55.00%) necroptosis-related genes, 34/50 (68.00%) pyroptosis-related genes, and 222/380 (58.42%) ferroptosis-related genes. Immunotherapy analysis suggested that our signature may have utility in predicting immunotherapy efficacy in patients with LUAD. Mast cells were identified as key players that support the predicting capacity of the eight-lncRNA signature through the immune infiltrating analysis.

**Conclusion:** In this study, an eight-lncRNA signature linked to cuproptosis was identified, which may improve LUAD management strategies. This signature may possess the ability to predict the effect of LUAD immunotherapy. In addition, infiltrating mast cells may affect the signature’s prognostic power.

## Introduction

Lung cancer is one of the most common malignant tumors affecting human health and quality of life, and it has the highest mortality rate among all cancers (Siegel et al., 2021). Nearly 80%–85% of lung cancer cases are non-small cell lung cancers (NSCLC) (Siegel et al., 2021). The 5-year survival rate for lung cancer in the United States is approximately 20%. Lung adenocarcinoma (LUAD), accounting for nearly 40% of NSCLC, has surpassed lung squamous cell carcinoma (LUSC) in incidence as the most common histological subtype of lung cancer (Siegel et al., 2021). It has been shown that early diagnosis and the advent of new treatments have prolonged the survival of people with LUAD (Spella and Stathopoulos, 2021). Due to LUAD’s insidious onset, patients with this disease have lymph node metastasis at screening, and the postoperative recurrence rate is high, leading to poor outcomes (Spella and Stathopoulos, 2021). Despite significant breakthroughs in diagnostic and remedial technologies, there is still a low long-term survival rate in LUAD patients (Spella and Stathopoulos, 2021). To improve the clinical outcome, new therapeutic targets need to be identified. It is, therefore, urgent and imperative that a new prognostic model for a feasible targeted biomarker be developed.

Programmed cell death (PCD) is crucial for tissue homeostasis and animal development. PCDs, such as apoptosis, necrosis, pyroptosis, and ferroptosis, are demonstrated to be critical for tumorigenesis, progression, and metastasis (Liu et al., 2022). One recently study published in “Science” have revealed cuproptosis as a new type of PCD, distinct from ferroptosis and apoptosis (Tsvetkov et al., 2022). Tsvetkov et al. proposed that cuproptosis requires mitochondrial respiration but that it is less affected by ATP produced by glycolysis (Tsvetkov et al., 2022). Their results show that copper seems to be closely linked to the TCA cycle, implying a close connection between cuproptosis and mitochondrial metabolism (Tsvetkov et al., 2022). It remains unclear how cuproptosis contributes to tumor initiation, progression, and immunity (Chen et al., 2022). It has been demonstrated that cuproptosis-related genes are expressed aberrantly and impact outcomes in clear cell renal cell carcinoma and hepatocellular carcinoma (Bian et al., 2022; Zhang et al., 2022). A recent study examined the significance of cuproptosis-related genes in immune infiltration and melanoma prognosis (Lv et al., 2022). Understanding the heterogeneity of LUAD may be improved by determining the molecular profiles of genes associated with cuproptosis. Long non-coding RNAs (lncRNAs), which are transcripts >200 nucleotides in length but not translated into protein, regulate gene expression at multiple levels and participate in various biological processes, especially cell death mechanisms (Alishahi et al., 2019). Therefore, the relationship between lncRNA and cuproptosis has potential significance for the clinical research of LUAD. Because RCD is more immuno-targeted than others (Liu et al., 2022), we need to understand how cuproptosis is initiated, propagated, and executed in LUAD cells, which have important implications for possible combined diagnostic and therapeutic interventions.

This work aims to fill the gap in studying cuproptosis-related lncRNA and LUAD prognosis. Using experimentally confirmed cuproptosis-related genes, we constructed a lncRNA signature to predict LUAD outcomes and further demonstrated its prognostic value. Moreover, we examined the correlation between the signature and other RCDs, and how each gene was expressed in the real world. More importantly, we investigated this model’s immunotherapeutic potential and anticancer drug selection value. For functional exploration, we performed gene set enrichment analysis (GSEA) to reveal the pathways in which the signatures reside. The final analysis of tumor-infiltrating immune cells provided the basis for further investigation of lncRNA signatures.

## Materials and methods

### Data selection and preprocessing

The project TCGA-LUAD contains LUAD samples and their accordingly clinical data. We downloaded the RNA-seq data (type: HTSeq - Counts) and clinical phenotype of patients in the project TCGA-LUAD on the GDC Xena Hub (https://gdc.xenahubs.net). All the data available in GDC Xena Hub is sourced exclusively from the official GDC pipeline (https://docs.gdc.cancer.gov/Data/Bioinformatics_Pipelines/Expression_mRNA_Pipeline/). It is worth noting that GDC Xena Hub carries out log2 (count+1) processing exclusively. To get more LUAD samples, we searched the Gene Expression Omnibus (GEO) (Clough and Barrett, 2016) database (https://www.ncbi.nlm.nih.gov/geo/) using the keyword “lung adenocarcinoma” and applying the following screening criteria: 1) total RNA expression data is available; 2) total LUAD cases that contain survival data are greater than 80. Finally, datasets named GSE29013, GSE30219, GSE31210, GSE37745, and GSE50081 were selected, and their gene expression matrix and clinical parameters were retrieved from GEO online portal. These GEO datasets are all generated using Platform GPL570, and proper protocols were followed for data collection and generation. Additional details can be found at https://www.ncbi.nlm.nih.gov/geo/query/acc.cgi?acc=GPL570. For the datasets selected above, only the tumor sample that contained gene expression and survival data were collected. Eligible patients in TCGA-LUAD were collected and served as the training cohort, and the downloaded data were directly used in our analysis without preprocessing. For the preprocessing of GSE29013, GSE30219, GSE31210, GSE37745, and GSE50081, we used the R package inSilicoMerging to merge them (Taminau et al., 2012), and then we adopted the method that developed by Johnson et al. (Johnson et al., 2007) to remove the batch effect and finally obtained the data matrix treated as a validation cohort.

### Screening of experimental proofed cuproptosis-related genes

We screened cuproptosis-related genes according to the following criteria: 1) Search the PubMed (https://pubmed.ncbi.nlm.nih.gov/) using the keyword “copper induced cell death pathway cancer”; 2) Studies unrelated to the search topic or with only Silico analysis were manually excluded; 3) The genes involved in wet experiment confirmed copper-induced cell death pathways in each study were collected, and unique ones were taken.

### Construction and validation of the prognosis model

LncRNA expression data were categorized depending on the annotations provided by the GENCODE project (Derrien et al., 2012). Cuproptosis-related lncRNAs were identified by comparing cuproptosis-related genes and lncRNAs using the limma R package with the following criteria: | Pearson correlation coefficient | > 0.3 and *p* < 0.05. For the selection of differentially expressed ones form the cuproptosis-related lncRNAs in normal and tumor tissue, we adopted the limma R package and applied false discovery rate <0.05.

Then, these screened lncRNAs were subjected to univariate Cox regression model for choosing the ones with potential prognostic power. The “survival” R package helped to build Cox models, and the cuproptosis-related lncRNA with a *p*-value less than 0.05 were selected. As a precaution against overfitting, we used the R package “glmnet” to combine Cox proportional hazards regression and the least absolute shrinkage and selection operator (LASSO) for the selected lncRNAs. We used 10-fold cross-validation to pick the optimal lambda (Tibshirani, 1997; Sauerbrei et al., 2007; Friedman et al., 2010; Goeman, 2010). The following formula was used to calculate each patient’s risk score:

$$Risk\;score=\sum_{i}^{n}Expi*\beta i$$

with βi indicating the coefficient, Expi representing the relative expression levels of each lncRNA standardized by z-score, and n indexing each hub lncRNA in the signature.

Patients in each cohort and subtypes were divided into low-risk groups and high-risk groups based on the best cutoff defined by the “surv_cutpoint ()” function of the “survminer” R package. The prognostic value of the signature was assessed in LUAD patients in the training and validation cohorts in Kaplan–Meier curve, univariate Cox analysis, and multivariable Cox analysis, the receiver operating characteristic (ROC) curve (Cao and Lopez-de-Ullibarri, 2019), time-dependent AUC (tAUC) analysis, and principal component analysis (PCA). The ROC curves and the tAUC analysis were made possible with the help of the “timeROC” and “survival” R packages. The “scatterplot3D” R package was used to evaluate the distribution of patients with different risk scores by PCA. Moreover, using the R packages “survival”, “survminer,” “rms” and “regplot”, we built nomograms predicting overall survival at 1, 3, and 5 years and applied calibration curves to evaluate model predictions versus actual outcomes consistency between. The R package “ggDCA” carried out the decision curve analysis to assess the nomogram accuracy for LUAD outcomes.

### Validation of the signature genes’ expression profile using the real-time PCR

The LUAD cell line A549 and the human lung fibroblasts cell line WI-38 were provided by Jiangsu KeyGEN BioTECH Co., Ltd. (Nanjing, China). A549 cells were grown in 90% RPMI-1640 (Thermo, United States) containing 10% fetal bovine serum (FBS), while WI-38 cells were grown in a 90% DMEM medium (Thermo, United States) containing 10% FBS. In addition, all cell media were supplemented with penicillin (100 U/mL) and streptomycin (100 U/mL) at 37°C in a 5% CO_2_ incubator. We isolated total RNA with TRIzol reagent (Invitrogen, United States). First-strand cDNA was synthesized from 1 μg total RNA using a PrimeScript™ 1st strand cDNA Synthesis Kit (Takara Bio, Japan). The One Step TB Green™ PrimeScript™ RT-PCR Kit II was used for real-time PCR (Takara Bio, Japan). The cycling conditions were 30 s of polymerase activation at 95°C followed by 40 cycles at 95°C for 5 s and 60°C for 30 s. GAPDH was used as an internal loading control. The relative level was calculated by the relative quantification 2^−ΔΔCT^ method.

### GSEA

For GSEA, we obtained the GSEA software (version 4.2.3) from the GSEA website (http://www.gsea-msigdb.org/gsea/downloads.jsp) (Subramanian et al., 2005). Patients were divided into low-risk groups and high-risk groups based on the best cutoff defined by the “surv_cutpoint ()” function of the “survminer” R package. We downloaded “c2.cp.kegg.v7.4.symbols.gmt” from the Molecular Signatures Database (Liberzon et al., 2011) (http://www.gsea-msigdb.org/gsea/downloads.jsp) to equip the GSEA software to evaluate related molecular mechanisms and pathways based on gene expression profiles and groupings. The parameter was as follows: the minimum gene set to 5; the maximum gene set to 5000; one thousand resamplings; *p*-value < 0.05 and FDR <0.25 were considered statistically significant.

### Correlations between the cuproptosis-related lncRNA signature and apoptosis, necroptosis, pyroptosis, and ferroptosis

For better knowing the interactions between our cuproptosis-related lncRNA signature and other types of “cell death”, we adopted the Pearson analysis. Apoptosis, necroptosis, and pyroptosis-related genes were extracted from the GeneCard and GSEA online databases, respectively, by applying the following steps: 1) search the GeneCard using the corresponding keyword; 2) search the GSEA using the corresponding keyword: 3) merge the above results and take the unique genes. FerrDb is the first database dedicated to ferroptosis regulators and ferroptosis-related diseases (Zhou and Bao, 2020). Ferroptosis-related genes were obtained from FerrDb (http://www.datjar.com:40013/bt2104/).

### Correlations between the lncRNA signature and immunotherapy

As part of the “gsva” R package, the “single-sample gene set enrichment analysis (ssGSEA)” function was used to assess 13 immune-related pathways. The “maftools” was used to identify the top 20 mutated genes and visualize mutations and their frequencies in all samples of the training cohort. Patients were divided into low-risk groups and high-risk groups based on the best cutoff defined by the “surv_cutpoint ()” function of the “survminer” R package. We applied the chi-square test to assess differences in gene mutation frequencies in low and high risk LUAD populations.

Tumor mutational burden (TMB) is an emerging therapeutic measure of immunotherapy sensitivity. TMB is defined as the frequency of certain mutations within a tumor’s genes (Chalmers et al., 2017). The TMB rank score of each case with LUAD was obtained as previously described (Chalmers et al., 2017). We deployed the Pearson coefficient together with the Wilcoxon rank-sum to calculate the connections between the risk score and TMB. Then, we selected CD274 (Fabrizio et al., 2018), CTLA4 (Rowshanravan et al., 2018), HAVCR2 (Holderried et al., 2019), IDO1 (Platten et al., 2014), LAG3 (Andrews et al., 2017), PDCD1 (Ribas and Wolchok, 2018), CD8A (Raskov et al., 2021), CXCL10 (Shi et al., 2021), CXCL9 (Liang et al., 2021), GZMA (Inoue et al., 2016), GZMB (Hurkmans et al., 2020), IFNG (Jorgovanovic et al., 2020), PRF1 (Fan et al., 2021), TBX2 (Li et al., 2021), and TNF (Freeman et al., 2021) from previous studies as immune relevant signatures, and used the Pearson and Wilcoxon rank-sum analysis to measure the correlations between our signature and the above listed immune signatures. To further test whether our lncRNA signature could guide immunotherapy, we deployed the Kaplan–Meier estimator to test the prognostic sensitivity of individual immune relevant signatures in high and low-risk patients, respectively. Tumor Immune Dysfunction and Exclusion (TIDE) is a computational framework that integrates T cell dysfunction expression signatures and T cell exclusion to model tumor immune evasion. Using TIDE, tumor immune evasion can be modeled in two ways and can be used to predict immunotherapy outcomes (Jiang et al., 2018; Fu et al., 2020; Chen Y. et al., 2021). More importantly, we tested if our signature correlated with the TIDE.

### PDCD1 and PRF1 expression detection and colony-formation assays

A549 cells were stably infected with a virus carrying either MIR4435-2HG, NKILA and their respective controls and seeded in 6-well plates (2 × 10^5^ cells/well) containing culture medium with 10% FBS for indicated time points.

48 h after transfection with lentivirus, the expressions of PDCD1 and PRF1 in the MIR4435-2HG and NKILA overexpression groups and their control groups were detected by real-time PCR. PDCD1 primer sequence is as follows: Forward: GTG​TCA​CAC​AAC​TGC​CCA​AC, Reverse: CTG​CCC​TTC​TCT​CTG​TCA​CC. PRF1 primer sequence is as follows: Forward: ACT​CAC​AGG​CAG​CCA​ACT​TT, Reverse: GGG​TGC​CGT​AGT​TGG​AGA​TA.

For the colony-formation assay, MIR4435-2HG-overexpressing and NKILA-overexpressing A549 cells (1000 cells/well) were seeded in 6-well plates and cultured under standard conditions. After 7∼10 days, cells were fixed with methanol and stained with 1% crystal violet. The number of colonies was manually counted using free ImageJ software (version 1.53e, National Institutes of Health, Bethesda, MD, USA).

### Drug sensitivity and the signature

Half-maximal inhibitory concentration (IC50) data for anticancer drugs were obtained from the Genomics of Cancer Drug Sensitivity (GDSC) database (https://www.cancerrxgene.org/) (Geeleher et al., 2014a). The drug distribution in the high and low risk score groups was analyzed and visualized using the “pRRophetic” R package (Geeleher et al., 2014b). The difference and correlation in the IC50 between the different risk groups was tested by the Wilcoxon test and the Spearman’s rank correlation coefficient, respectively.

### Relationships between the TICs and our signature

TICs play an essential role in the development and progression of cancer (Ye et al., 2019). We invoked the “CIBERSORT” R package developed by Newman and colleagues (Newman et al., 2015) to assess the abundance of 22 TICs using gene expression data from the training cohort. To better illustrate a clear portrait of the lncRNA signature, we first used the Pearson test and Wilcoxon rank-sum analysis to assess the signatures’ correlations with 22 TICs. Next, we applied the univariate Cox model and Kaplan-Meier curve to determine the TICs that had prognostic power. Finally, combining the above results, we inferred candidate TICs may contribute to the prognostic power of the lncRNA signature.

### Statistical analysis

We employed a Wilcox test to compare gene expression levels between tumor and non-tumor tissues. To evaluate the predictive power of our survival model for patient survival, we utilized the tAUC. The overall survival rates of each group were compared using a Kaplan-Meier analysis in conjunction with a log-rank test. To identify molecules associated with prognosis, Cox regression of overall survival was performed using univariate data. We analyzed all statistics using R software, and we performed two-sided t-tests for all statistical tests unless otherwise stated. Statistical significance was defined as *p* < 0.05.

## Results

### Patient characteristics

**FIGURE 1:**
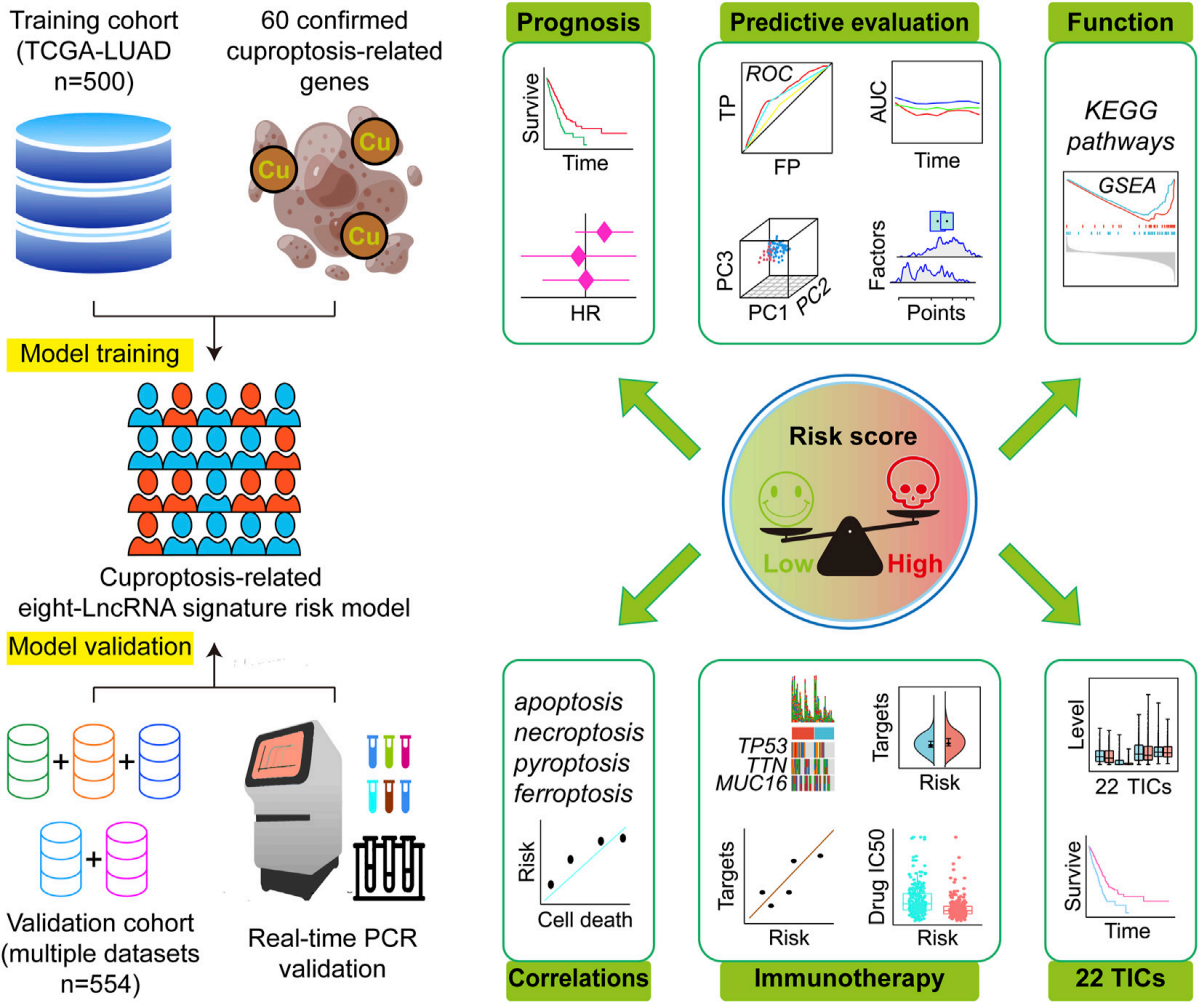
Research design and analysis process. TCGA, The Cancer Genome Atlas; LUAD, lung adenocarcinoma; HR, hazard ratio; ROC, receiver operating characteristic; TP, true positive rate; FP, false positive rate; AUC, area under the ROC curve; PC, principal component; KEGG, Kyoto Encyclopedia of Genes and Genomes; GSEA, Gene Set Enrichment Analysis; IC50, half maximal inhibitory concentration; TICs, tumor-infiltrating immune cells.

**FIGURE 2:**
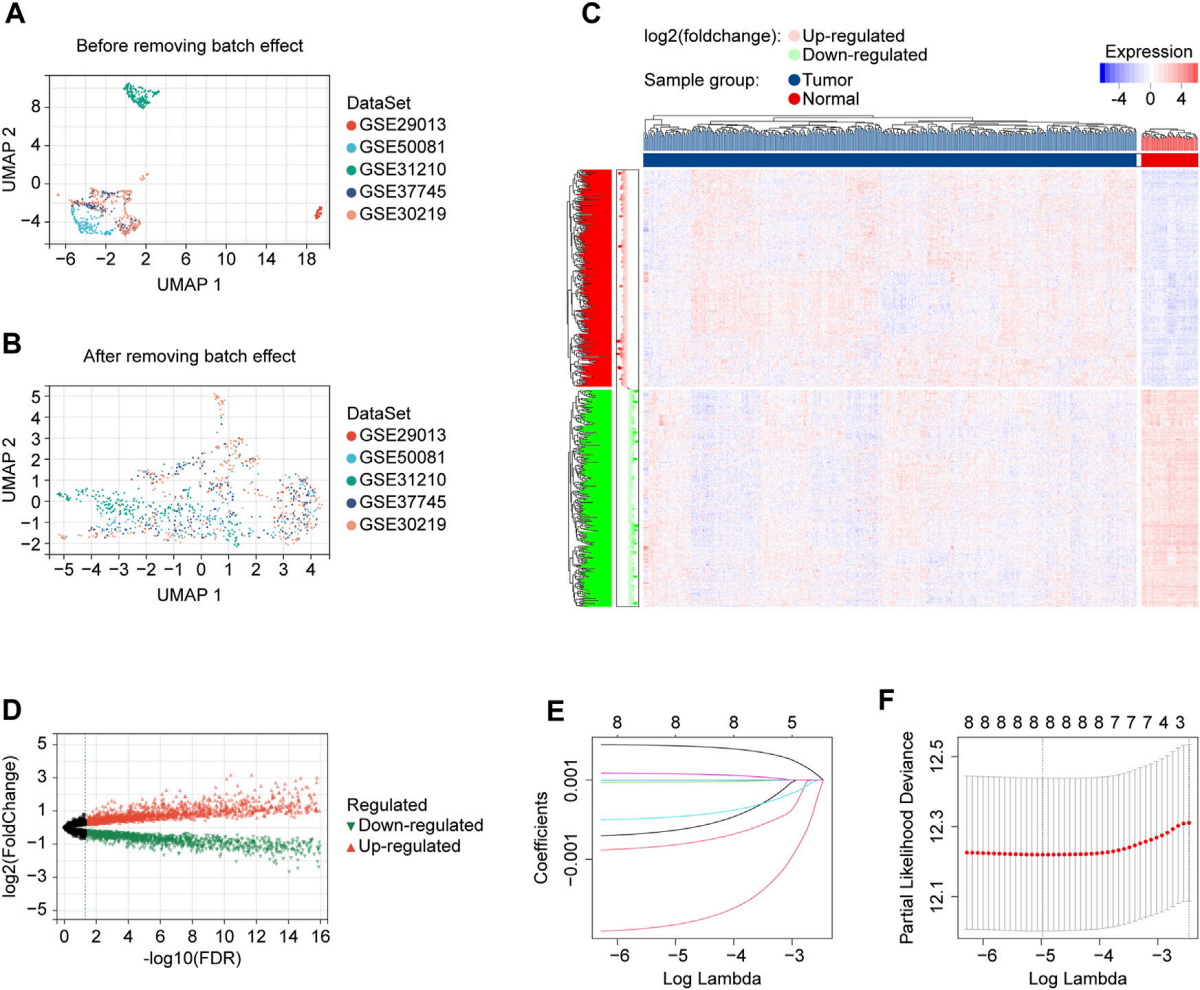
Data preprocessing and risk model development. **(A)** UMAP plot showing validation cohort merged before batch effect removing, displaying that the samples of each dataset are separated from each other. **(B)** UMAP plot showing validation cohort merged after batch effect removing, displaying that samples of each dataset are clustered and intertwined with each other. **(C)** The heatmap for identifying differentially expressed lncRNAs in normal and tumor tissues. **(D)** The volcano plot for identifying differentially expressed lncRNAs in normal and tumor tissues. **(E)** LASSO coefficient profiles of prognostic lncRNAs enrolled. **(F)** LASSO regression with ten-fold cross-validation obtained eight prognostic genes using the minimum Lambda (0.006898922). UMAP, Uniform Manifold Approximation and Projection; LASSO, least absolute shrinkage and selection operator; FDR, false discovery rate.

A total of 1054 LUAD cases with available survival data were included in the present study. The flowchart of this study is shown in Figure 1. We included 500 patients from the TCGA project for the risk model construction. We downloaded five validation datasets (GSE29013, GSE30219, GSE31210, GSE37745, and GSE50081) from GEO. According to our selection criteria, data from the five GEO datasets contained 554 pieces of usable LUAD information and was used to validate the gene signature we built. The patient information used in this study is listed in Table 1. We merged five validation datasets into one cohort to better process our study. Batch effects are nonbiological differences between two or more datasets. We removed the bias caused by batch effects and made the transcriptional profiles more similar based on our methods stated before (Figures 2A, B).

**TABLE 1:** Clinical characteristics of patients enrolled in the study.

Characteristics	Training cohort (TCGA-LUAD, *n* = 500)	Validation cohort (GSE29013, GSE30219, GSE31210, GSE37745, and GSE50081, *n* = 554)
Age		
<65	219 (43.8%)	315 (56.86%)
≥65	271 (54.2%)	239 (43.14%)
Unknown	10 (2%)	0
Gender		
Female	270 (54%)	265 (47.83%)
Male	230 (46%)	289 (52.17%)
Race		
White	386 (77.2%)	NA
Non-White	60 (12%)	NA
Unknown	54 (10.8%)	NA
Ethnicity		
Hispanic or Latino	7 (1.4%)	NA
Non-Hispanic or Latino	381 (76.2%)	NA
Unknown	112 (22.4%)	NA
Tumor stage		
Stage I	268 (53.6%)	339 (61.19%)
Stage II	119 (23.8%)	108 (19.49%)
Stage III	80 (16%)	21 (3.79%)
Stage IV	25 (5%)	4 (0.72%)
Unknown	8 (1.6%)	82 (14.8%)
Prior malignancy		
Yes	79 (15.8%)	NA
No	421 (84.2%)	NA
Tissue origin		
Upper lobe lung	291 (58.2%)	NA
Non-upper lobe lung	209 (41.8%)	NA
Smoking history		
Ever	415 (83%)	216 (38.99%)
Never	71 (14.2%)	139 (25.09%)
Unknown	14 (2.8%)	199 (35.92%)
Vital status		
Alive	318 (63.6%)	348 (62.82%)
Dead	182 (36.4%)	206 (37.18%)

### A cuproptosis-related eight-lncRNA signature generated

We got 60 cuproptosis-related genes following our criteria, shown in Table 2. We used the Pearson algorithm to detect lncRNAs that significantly associated with cuproptosis-related genes in the training cohort samples, and finally 8277 unique lncRNAs were determined (Supplementary Table S1; Supplementary Figure S1). From the 8277 lncRNAs, the “limma” R language package identified 3261 of them differentially expressed in normal and tumor tissues (Figures 2C, D). Then, we used the expression profiles of the 3261 lncRNAs in the training cohort to assess for candidates with prognostic ability. And 9 lncRNAs were identified having potential prognostic ability (Supplementary Table S2). By utilizing the “glmnet” R package (version 4.1.2, New Zealand), LASSO Cox regression analysis was carried out to narrow the scope of applicant genes and develop a predictive model. Finally, the optimal lambda was selected as 0.006898922, at which point 8 lncRNAs and their coefficients were preserved (Figures 2E, F; Table 3). In addition, we displayed the correlations between each of the 8 lncRNAs and the 60 cuproptosis-related genes, which are shown in Supplementary Figure S2A.

**TABLE 2:** The cuproptosis-related genes that extracted from the previous research.

ID	Description	Evidence with cuproptosis (PMID)
AFP	alpha fetoprotein	80265
ANKRD9	ankyrin repeat domain 9	31337707
AOC2	amine oxidase copper containing 2	20013028
AOC3	amine oxidase copper containing 3	34318901
APLP2	amyloid beta precursor like protein 2	28197076
APOA4	apolipoprotein A4	30959835
ATG9A	autophagy related 9A	32610138
ATOX1	antioxidant 1 copper chaperone	27472369, 31898157
ATP7A	ATPase copper transporting alpha	21221114
ATP7B	ATPase copper transporting beta	17268820, 34831341, 34209820
CCDC22	coiled-coil domain containing 22	25355947
CCS	copper chaperone for superoxide dismutase	22387373
CDKN2A	cyclin dependent kinase inhibitor 2A	35298263, 32875942
CNNM1	cyclin and CBS domain divalent metal cation transport mediator 1	27251091
COMMD1	copper metabolism domain containing 1	33482423
COX17	cytochrome c oxidase copper chaperone COX17	15142040
CP	ceruloplasmin	12055353
CUTC	cutC copper transporter	16182249
DBH	dopamine beta-hydroxylase	6998654
DLAT	dihydrolipoamide S-acetyltransferase	35298263
DLD	dihydrolipoamide dehydrogenase	35298263
F5	coagulation factor V	6490642
F8	coagulation factor VIII	8602593
FDX1	ferredoxin 1	35298263
GCSH	glycine cleavage system protein H	35298263
GLS	glutaminase	35298263
GPC1	glypican 1	34224365
HEPH	hephaestin	29659961
LIAS	lipoic acid synthetase	35298263
LIPT1	lipoyltransferase 1	35298263
LIPT2	lipoyl (octanoyl) transferase 2	35298263
LOX	lysyl oxidase	9355764
LOXL1	lysyl oxidase like 1	32253563
LOXL2	lysyl oxidase like 2	30320382
LOXL3	lysyl oxidase like 3	31340433
LOXL4	lysyl oxidase like 4	14551188
METTL17	methyltransferase like 17	31487196
MOXD1	monooxygenase DBH like 1	15337741
MOXD2P	monooxygenase DBH like 2, pseudogene	17642472
MPC1	mitochondrial pyruvate carrier 1	35298263
MT1DP	metallothionein 1D, pseudogene	29507753
MT1X	metallothionein 1X	33559028
MT3	metallothionein 3	32843100
MT4	metallothionein 4	14627437
MT-CO3	mitochondrially encoded cytochrome c oxidase III	34504870
MTF1	metal regulatory transcription factor 1	35298263
PAM	peptidylglycine alpha-amidating monooxygenase	20648645
PDHA1	pyruvate dehydrogenase E1 subunit alpha 1	35298263
PDHB	pyruvate dehydrogenase E1 subunit beta	35298263
RNF7	ring finger protein 7	10082581
S100A5	S100 calcium binding protein A5	10882717
SCO2	synthesis of cytochrome C oxidase 2	15229189
SLC11A2	solute carrier family 11 member 2	31804182
SLC31A1	solute carrier family 31 member 1	30706544
SLC31A2	solute carrier family 31 member 2	24703712
SNAI3	snail family transcriptional repressor 3	12579345
SOD1	superoxide dismutase 1	32517371
STEAP2	STEAP2 metalloreductase	29674723
STEAP3	STEAP3 metalloreductase	34741044
TYR	tyrosinase	29473882

**TABLE 3:** Prognostic lncRNAs obtained from the LASSO Cox regression model and their primer sequences.

Gene symbol	Coefficient	Sequence (5' - 3′)	
		Forward	Reverse
TSPOAP1-AS1	−0.003636899	TGA​CAC​CTT​GAC​CAG​CGA​ACA​C	CAG​GCT​GTG​GTC​GTC​TAT​CTC​C
AC107464.3	−0.001626257	GAT​GTC​CGC​AGG​GCA​AGA​GAA​T	ATC​ACA​ATG​GCC​GCA​GGA​AGA​G
AC006449.7	−0.00125717	GGA​GTG​CTG​CGT​GTG​AGT​TAC​C	ACC​ACG​AGG​TGC​TCA​CGA​ACA
LINC00324	−0.000923428	CGG​AGG​CAG​GAA​GTG​TCA​AGA​T	TCA​AGG​AAG​TGG​GAG​GGA​GTG​G
COLCA1	−4.91E-05	TGC​GTG​CCC​TTG​GTC​TGG​AA	GGG​TAA​CTC​GGC​TGC​TTC​TCC​T
HAGLR	−4.93E-06	GGA​CCC​TTC​ACC​TGC​CTC​TAC​T	GCC​AGG​TCC​AGC​ATG​AAA​CAG​A
MIR4435-2HG	0.000156045	TGC​CAG​GAC​ACA​GCC​ATC​TAA	CCC​TTC​TAC​CCT​ACC​TCA​GCA​T
NKILA	0.000863014	CGA​CCA​GGA​AAG​ACG​GGA​ACT​C	GGC​GGC​GAC​AAT​ACA​CCA​GT

### The eight-lncRNA signature proven to have stable prognostic capacity

**FIGURE 3:**
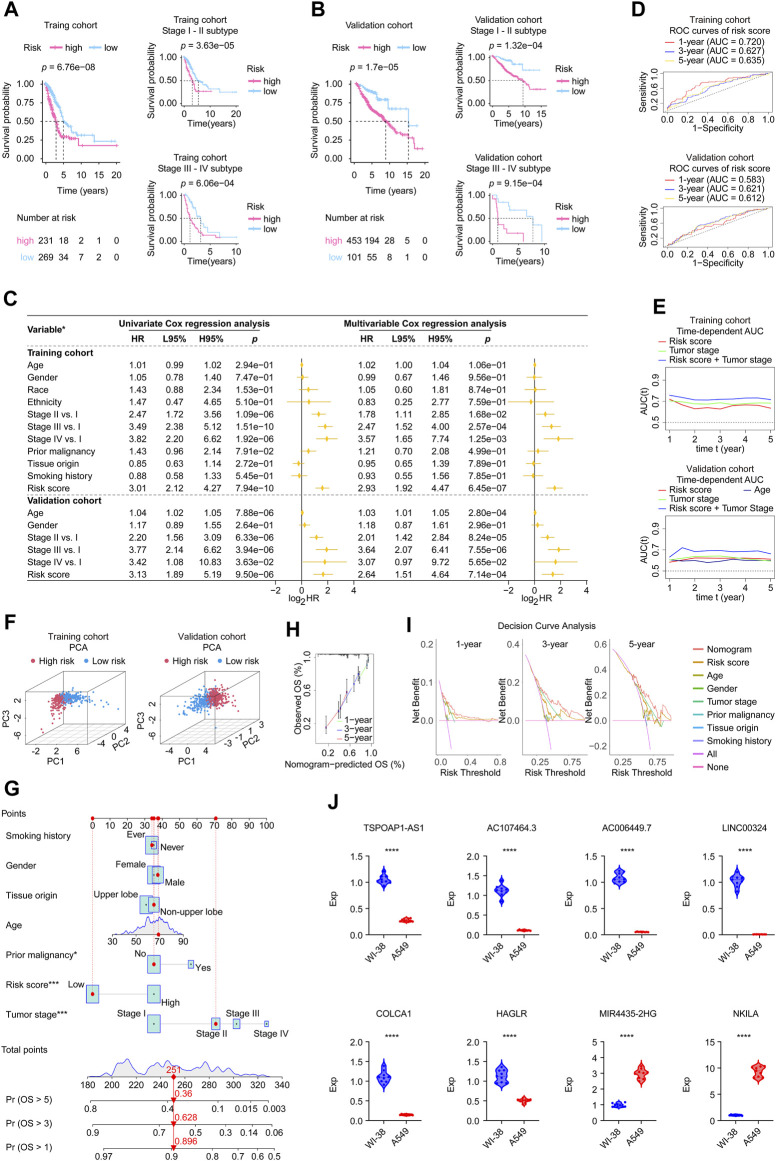
Validation of the eight-lncRNA signature using the involved studied cohorts and real-time PCR experiment. **(A,B)** Kaplan-Meier analysis was performed in the whole and subtypes of training and validation cohorts. Patients in each cohort and subtypes were divided into low-risk groups and high-risk groups based on the best cutoff defined by the “surv_cutpoint ()” function of the “survminer” R package. The log-rank test with a *p-*value < 0.05 suggests the survival difference is significant. The bottom part displays the number of patients at risk. **(C)** Univariate and multivariable Cox proportional hazards models. *: the variables involved in the studied cohorts, explains as follows: Gender: male vs. female; Race: white vs. non-white; Ethnicity: Hispanic or Latino vs. non-Hispanic or Latino; Prior malignancy: yes vs. no; Tumor origin: upper lobe lung vs. non-upper lobe lung; Smoking history: ever vs. never. **(D)** ROC curves. The ROC curves valued the accuracy for LUAD outcome prediction of our signature at 1-, 3-, and 5-year, respectively. **(E)** The tAUC analyses compared our signature’s prognostic ability with other available clinical characteristics. The larger the AUC, the stronger the model’s predictive ability. **(F)** Principal component analysis scatter plot. **(G)** The nomogram, a quantitative model for predicting clinical prognosis, to predict 1-year, 3-year, and 5-year OS in the LUAD patients of the TCGA-LUAD cohort using seven factors, including age, grade, tumor stage, risk score, smoking history, tissue origin, and prior malignancy. **(H)** The calibration curves indicated that the nomogram accurately predicted the 1-, 3-, and 5-year OS of LUAD patients in the TCGA cohort. **(I)** Decision curve analysis. The curves assessed the nomogram prediction accuracy for LUAD outcomes at 1-, 3-, and 5-year, respectively. **(J)** The expression levels of the 8 signature genes in A549 (*n* = 9) and WI-38 (*n* = 9) cell lines detected by real-time PCR. Data were means ± STD. ****: *p-*value < 0.0001; HR, hazard ratio; L95%, 95% confidence interval lower; H95%, 95% confidence interval higher; HR, hazard ratio; ROC, receiver operating characteristic; AUC, area under the ROC curve; tAUC, time-dependent AUC; TCGA, The Cancer Genome Atlas; LUAD, lung adenocarcinoma; PCA, Principal components analysis; OS, overall survival; Exp, relative expression.

In Supplementary Figures S2B, C, the upper parts showed the patients sorted by increasing risk score, the scatter plot in the middle showed the survival status of the LUADs, and the heatmap in the lower part showed the relative expression levels of eight hub lncRNAs. Based on a risk score calculator we developed, a risk score was calculated for each LUAD case enrolled. Kaplan-Meier estimator suggested that high risk patients had a worse survival in the TCGA cohort (log-rank test, *p* < 0.001, Figure 3A) and in the validaiton cohort (log-rank test, *p* < 0.001, Figure 3B) than low risk patients. In addition, the tumor stage subtype analysis suggested that our risk score can precisely predict the patients’ outcomes, where the higher risk score forebode worse disease ends (Figures 3A, B). In addition, in Supplementary Figure S3A, we displayed each eight lncRNA’s prognosis ability in the form of Kaplan-Meier curves using the two cohorts’ data, showing that the MIR4435-2HG and NKILA performed stable unfavorable impact on LUAD patients, while TSPOAP1-AS1, AC107464.3, AC006449.7, LINC00324, COLCA1, and HAGLR helped the prognosis improvement of LUADs.

Subsequently, we assessed the independent prognostic value of our gene signature. Univariate and multivariate Cox regression analyses were performed to evaluate whether signature risk score was independent of other clinical parameters (age, gender, race, ethnicity, tumor stage, tumor origin, etc.) as prognostic factors for LUAD patients (Figure 3C). Univariate Cox regression analysis showed that in training and validation cohorts, risk scores were significantly correlated with overall survival. Multivariate Cox regression analysis showed that risk scores were independently associated with survival in the training cohort (HR = 2.93, 95% CI: 1.92–4.47, *p* = 6.45e-07) and validation cohort (HR = 2.64, 95% CI: 1.51–4.64, *p* = 7.14e-04). In addition, the age of validation cohort also showed independent prognostic value, however, its significance did not show a consistence in the two cohorts. Moreover, each gene’s univariate Cox regression was shown in the chart exhibited in Supplementary Figure S3B. These results further confirmed the high predictive accuracy of our gene signature, suggesting that the model could be independently used to predict the prognosis of LUAD patients.

Then we applied ROC analysis (Figure 3D) and time-dependent AUC (Figure 3E) to verify the prognostic ability of our lncRNA signature. The ROC curve for assessing the predictive strength of the gene prognostic signature exhibited an AUC of 0.720, 0.627, and 0.635 at 1, 3, and 5 years, respectively, suggesting that the risk signature would have a tolerable predictive capacity in the training database. Concurrently, this signature displayed similar results for the validation cohort in ROC curve analysis at 1, 3, and 5 years, with AUCs of 0.583, 0.621, and 0.612, respectively. According to the time-dependent AUC performed in the training cohort (Figure 3E), our risk score was close compared to tumor stage, which is regarded as the prognosis gold standard. The AUC was generally greater than 0.7 when we combined the risk score and tumor stage. Consistently, the AUC performance of the risk score combined with the tumor stage outperformed all remaining factors at all time points, suggesting that our risk score is an excellent complement to the tumor stage (Figure 3E). PCA results showed significant heterogeneity between high-risk and low-risk patients in the training and validation cohorts (Figure 3F), which certified the superior discrimination of the risk score model. Moreover, we constructed a nomogram using seven factors, including age, grade, tumor, etc., a quantitative model for predicting clinical outcomes, predicting 1-, 3-, and 5-year overall survival in the TCGA-LUAD cohort population (Figure 3G). The calibration curves confirmed that the nomogram accurately predicted the 1-, 3-, and 5-year overall survival of LUAD patients in the TCGA cohort (Figure 3H). Decision curve analysis assessed the nomogram accuracy for LUAD outcomes at 1-, 3-, and 5-year, respectively, and the results confirmed that the nomogram is superior to other factors (Figure 3I).

### The expression level of each gene of the signature was validated by real-time PCR

To better understand the real-world expression pattern of each gene in the gene signature, we applied the real-time PCR to detect the above genes in human LUAD cell lines (*n* = 9) and human normal cell lines (*n* = 9) difference in expression. Table 3 shows the primer sequences for the 8 genes, TSPOAP1-AS1, AC107464.3, AC006449.7, LINC00324, COLCA1, HAGLR, MIR4435-2HG, and NKILA. Notably, all genes were differentially expressed in tumor and normal cell lines (Figure 3J). Specifically, genes of MIR4435-2HG and NKILA were highly expressed in LUAD cell lines, while the remaining were under-expressed in LUAD cell lines. The upregulated genes in LUAD like MIR4435-2HG and NKILA were also showed having unfavorable prognosis powerful in Supplementary Figure S3, and the downregulated genes in LUAD like TSPOAP1-AS1, AC107464.3, AC006449.7, LINC00324, COLCA1, and HAGLR displayed owning protectable function for the LUAD outcomes, which further proved the validity of the gene signatures we found and provided clues for deeper research.

### GSEA determined the mechanisms of the prognosis signature

**FIGURE 4:**
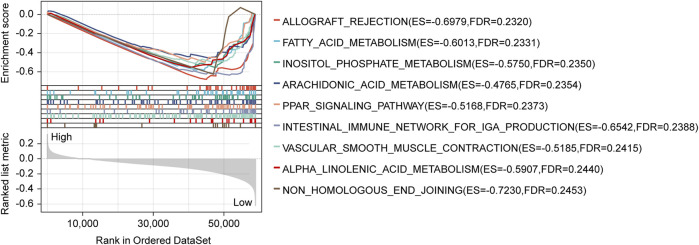
GSEA analysis with the KEGG gene set as the background identified relevant pathways of our signature. The significance threshold of this analysis was set as: *p-*value < 0.05, and FDR <0.25. GSEA, Gene Set Enrichment Analysis; KEGG, Kyoto Encyclopedia of Genes and Genomes; FDR, false discovery rate.

The GSEA analysis performed based on risk scores discovered significantly enriched gene sets in the lncRNA signature, which the top ten items were primarily related to allograft rejection, fatty acid metabolism, inositol phosphate metabolism, arachidonic acid metabolism, PPAR signaling pathway, intestinal immune network for IgA production, vascular smooth muscle contraction, alpha-Linolenic acid metabolism, and non-homologous end-joining (Figure 4; Supplementary Table S3).

### The eight-lncRNA signature’s relationships with apoptosis, necroptosis, pyroptosis, and ferroptosis

We got apoptosis, necroptosis, pyroptosis, and ferroptosis genes following our criteria, shown in Supplementary Table S4. The Pearson coefficient examined the relationships between our prognosis model and apoptosis, necroptosis, pyroptosis, and ferroptosis-related genes, respectively (Supplementary Table S4; Supplementary Figure S4). The analysis showed that SELENBP1, METTL7A, ANLN, SARM1, GGA2, GAPDH, MOAP1, CTSH, RBP5, and CEP55 were the top ten correlated apoptosis-related genes, and overall, 2321/3681 (63.05%) genes significantly linked with the lncRNA signature. RIPK3, FADD, TRPM7, CYLD, TLR3, IPMK, ZBP1, TNF, FASLG, and PGLYRP1 were discovered as the top necroptosis-related that correlated with the eight-lncRNA signature. As a whole, there were 11/20 (55.00%) necroptosis-related genes correlated with the signature pronouncedly. Moreover, the Pearson test found the top pyroptosis-related genes that correlated with our signature are NLRP1, CARD8, GSDMB, CYCS, IRF2, GSDMD, IL1A, NLRP6, CASP1, and CHMP2B. In total, 34/50 (68.00%) pyroptosis-related genes correlated with our signature. The examination found SLC2A1, RRM2, AURKA, MDM4, PLA2G6, DUOX1, GLS2, NRAS, HIF1A, and SIRT3 were top ferroptosis-related genes that correlated with our signature. To sum up, 222/380 (58.42%) ferroptosis-related genes correlated with our signature.

### The role of risk score participating in immunotherapy

**FIGURE 5:**
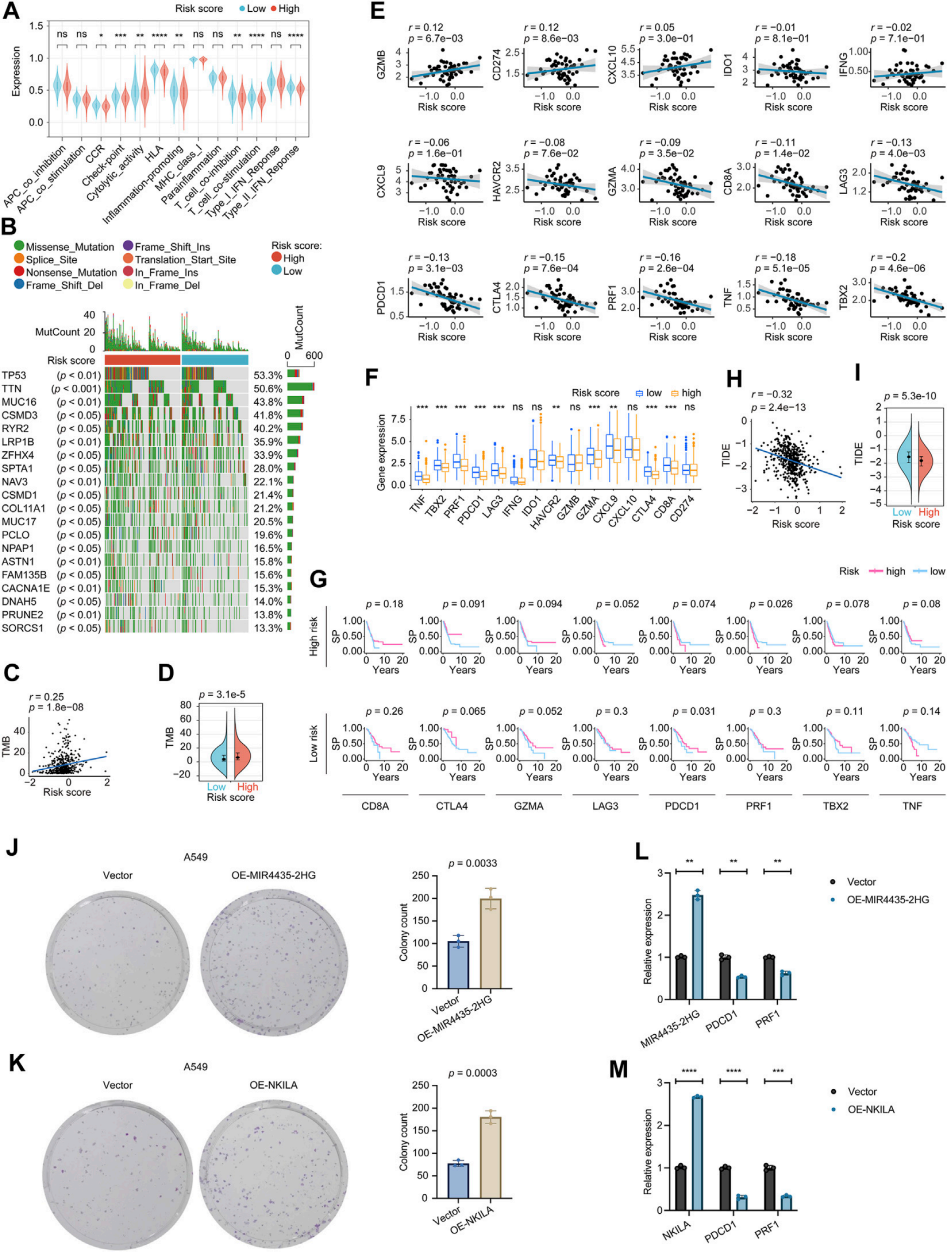
Determination of the relationship between the eight-lncRNA signature and immunotherapy. **(A)** The immune function distribution differences in the high and low risk score populations of the LUAD. **(B)** The waterfall plot shows the top 20 genes mutated in the LUAD, and their difference in the high and low risk groups. **(C)** The correlation between TMB and the signature tested by the Pearson coefficient. **(D)** The TMB difference in the high and low-risk patients tested by the Wilcoxon rank-sum. **(E)** The Pearson coefficient evaluated the correlations between the signature and the immune relevant signatures. **(F)** The Wilcoxon rank-sum revealed the distribution differences of the immune relevant signatures in high and low-risk patients. **(G)** The Kaplan–Meier estimator measured the immune relevant signatures’ prognosis sensitive zone by testing in high and low-risk groups, respectively. **(H)** The correlation between TIDE and the signature tested by the Pearson coefficient. **(I)** The TIDE difference in the high and low-risk patients tested by the Wilcoxon rank-sum. *p-*value < 0.05 was considered statistically significant. **(J)** Colony-forming abilities of OE-MIR4435-2HG manipulated A549 cells were measured by performing colony-formation assays. The left two panels: representative photomicrographs. Data presented as the mean ± SD of three independent experiments. **(K)** Colony-forming abilities of OE-NKILA manipulated A549 cells were measured by performing colony-formation assays. The left two panels: representative photomicrographs. Data presented as the mean ± SD of three independent experiments. **(L)** Effect of MIR4435-2HG overexpression on PDCD1 and PRF1 expression. Data presented as the mean ± SD of three independent experiments. **(M)** Effect of NKILA overexpression on PDCD1 and PRF1 expression. Data presented as the mean ± SD of three independent experiments. TMB, Tumor mutational burden; TIDE, Tumor Immune Dysfunction and Exclusion. ns: *p-*value > 0.05; *: *p-*value < 0.05; **: *p*-value < 0.01; ***: *p*-value < 0.001; ****: *p-*value < 0.0001.

As for the related immune functions, the scores for the CCR, Check−point, Cytolytic_activity, HLA, Inflammation−promoting, T_cell_co−inhibition, T_cell_co−stimulation, and Type_II_IFN_Reponse were lower in the high-risk group than in the low-risk group (Figure 5A). These findings revealed that the eight-lncRNA signature might be associated with the immunological status of LUADs. We thoroughly explored the mutation characteristics of all tumor samples in the TCGA-LUAD cohort. As top 20 mutated shown in Figure 5B, gene TP53 mutated most frequently approximately accounting for 53.3% in the cohort, followed by TTN (50.6%) and MUC16 (43.8%). Among the alterations, missense mutation was the most common variant classification. Interestingly, we noticed that the mutation distribution of these 20 genes in the high and low risk groups was statistically significant (*p* < 0.05).

We explored the TMB difference among the risk score, and the Pearson coefficient found that the TMB was positively correlated with the risk score (Figure 5C). Moreover, the Wilcoxon test displayed that the higher risk score group had a higher TMB (Figure 5D). According to clinical trials and preclinical studies, the immune checkpoint blockade offers more durable clinical benefits, including treatment responses and long-term survival, to patients with higher TMB (Samstein et al., 2019; Stenzinger et al., 2019). Our results here demonstrated that our high risk LUADs might expect more responses from immunotherapy to a certain extent.

We noticed that 10/15 of the checkpoint relevant signatures, GZMB, CD274, GZMA, CD8A, LAG3, PDCD1, CTLA4, PRF1, TNF, and TBX2 correlated with the eight-lncRNA signature by the Pearson coefficient (Figure 5E). The Wilcoxon examination found TNF, TBX2, PRF1, PDCD1, LAG3, HAVCR2, GZMA, CXCL9, CTLA4, and CD8A, were expressed differently between high and low-risk groups significantly (Figure 5F). Incorporating the Wilcoxon and Pearson analyses, eight genes, including CD8A, CTLA4, GZMA, LAG3, PDCD1, PRF1, TBX2, and TNF were closely connected to the eight-lncRNA signature. Subsequently, we focused on the eight identified checkpoint relevant signatures. We tested these signatures’ prognosis roles in the high and low-risk groups to see the “comfort risk score zone” for potential immunotherapy. As shown in Figure 5G, CD8A, CTLA4, GZMA, LAG3, TBX2, and TNF did not impact the prognosis in neither high nor low-risk LUADs, hinting that these checkpoint genes were incapable of utilizing the risk score for influencing the LUAD prognosis. Notably, PDCD1 could protect the prognosis more in the low risk-groups than that in the high-risk group, and PRF1 impacted the LUAD survival probability more in the high-risk group than that in its low-risk group, which implied PDCD1 and PRF1 target therapies might maximize their influences in their specific risk score zone, respectively. These results suggested that our risk score system could potentially guide immunotherapy choices based on each checkpoint’s “comfort risk score zone”, however, more clinical data are needed to support our conclusions.

Following that, we examined the potential clinical efficacy of immunotherapy based on the risk score subgroups using the TIDE. As a surrogate biomarker, TIDE scores can provide insight into whether a NSCLC patient will respond to immune checkpoint blockades, including anti-PD1 and anti-CTLA4, if therapy is initiated. Higher TIDE prediction scores represent a higher likelihood of immune evasion, suggesting that patients are less likely to benefit from immunotherapy (Jiang et al., 2018; Fu et al., 2020; Chen Y. et al., 2021). According to our results, high-risk patients had lower TIDE scores than low-risk patients, indicating that immunotherapy was more beneficial for them (Figures 5H, I), which corresponded with our “TMB difference” findings above. The TIDE results were consistent with the “comfort risk score zone” of PRF1 we mentioned and could potentially guide the planning of the anti-PD1 (anti-PDCD1) strategy for LUAD treatment based on the risk score.

The bioinformatics analysis results of PDCD1 and PRF1 are exciting to us. The role of two of the eight genes in our model, MIR4435-2HG and NKILA, negatively affecting prognosis in LUAD was also of interest. Using a colony formation assay, we further examined the effect of MIR4435-2HG and NKILA on long-term growth (7∼10 days) of A549 cells. As shown in Figures 5J, K, the numbers of colonies of the A549 cell line increased following the overexpression of MIR4435-2HG and NKILA, respectively. These results indicated that MIR4435-2HG and NKILA might be deeply involved in LUAD progression by inducing the growth and motility of cells. The real-time PCR results (Figures 5L, M) showed that the immune checkpoints PDCD1 and PRF1 were downregulated after overexpression of MIR4435-2HG or NKILA, suggesting that PDCD1 and PRF1 had a mutual regulatory relationship with MIR4435-2HG or NKILA, respectively.

### Risk score and anticancer drug sensitivity

**FIGURE 6:**
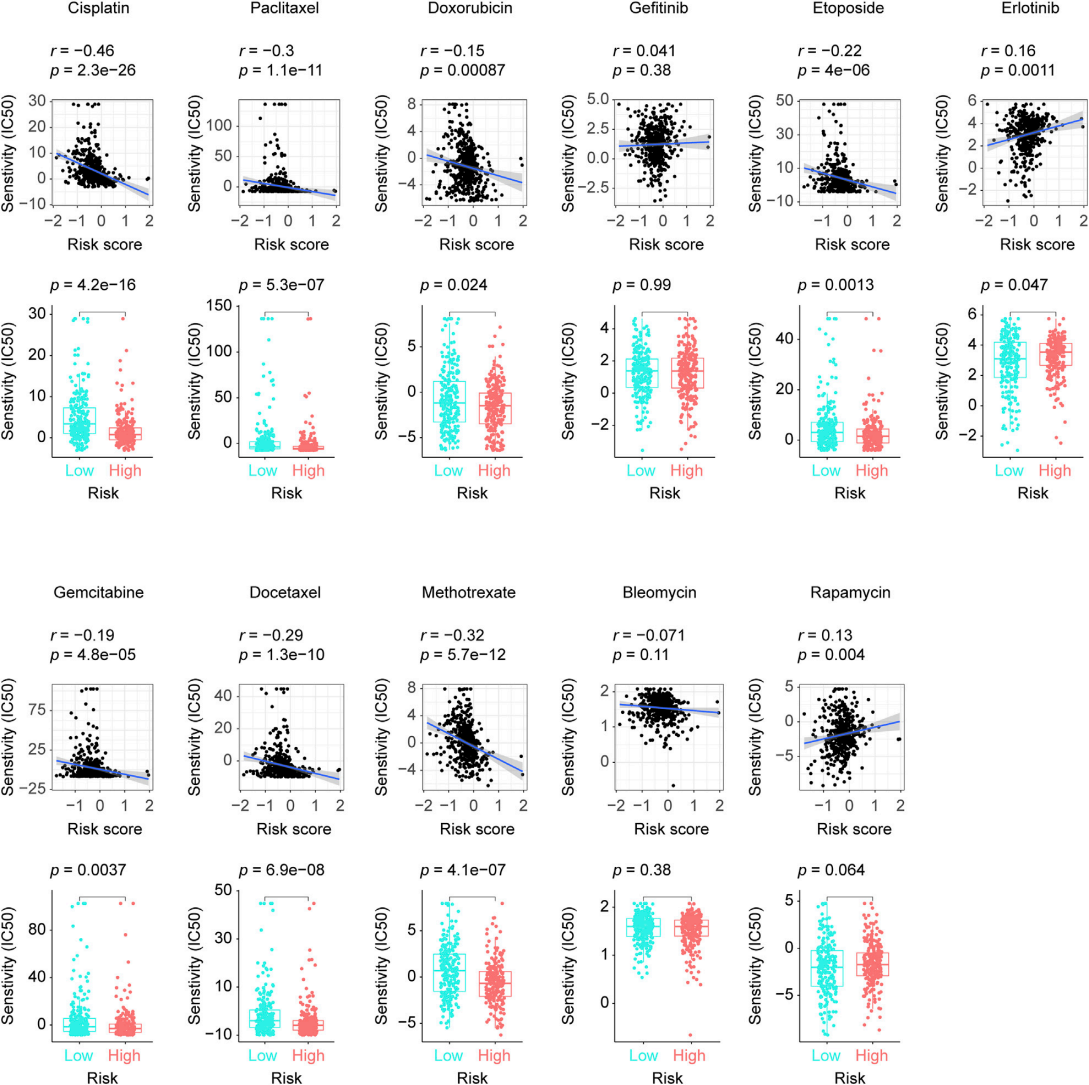
The disparities in our selected anticancer drugs’ responses between groups with low and high risk scores. The difference and correlation in the IC50 between the different risk groups was analyzed by the Wilcoxon test (lower plots) and the Spearman’s rank correlation coefficient, respectively (upper plots). IC50: half maximal inhibitory concentration.

We screened out 8 chemotherapy drugs (cisplatin, paclitaxel, doxorubicin, etoposide, gemcitabine, docetaxel, methotrexate, and bleomycin), 2 epidermal growth factor receptor (EGFR) inhibitors (gefitinib, erlotinib), and 1 immunosuppressive drug (rapamycin) from the GDSC database, which had been used in LUAD clinical practice. By adopting “pRRophetic” package, we found 7 chemotherapy drugs (cisplatin, paclitaxel, doxorubicin, etoposide, gemcitabine, docetaxel, and methotrexate) and 1 EGFR inhibitor (erlotinib) showed significantly sensitivity differences in the high and low-risk patients, while, Bleomycin, gefitinib, and rapamycin exhibited no correlation with our risk score model. As specifically shown in Figure 6, cisplatin, paclitaxel, doxorubicin, etoposide, gemcitabine, docetaxel, and methotrexate received more feedback from the high-risk patients, otherwise, erlotinib got a more intensive response in the low-risk LUADs, which hinted the eight-lncRNA signature’s potential usage in the clinical practice.

### Mast cells’ vital roles in the eight-lncRNA signature’s prediction ability

**FIGURE 7:**
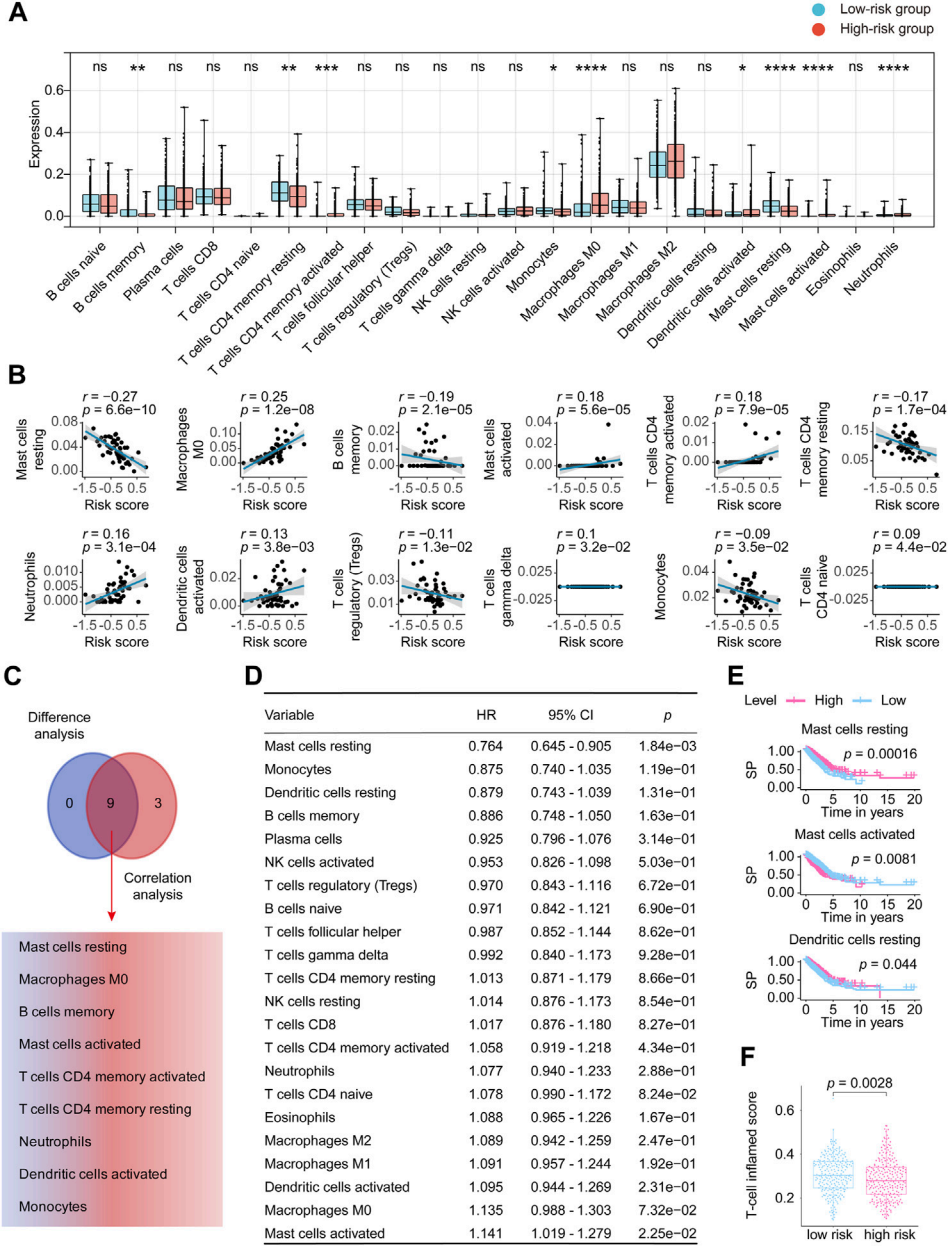
Comprehensive analysis determined the relationship between 22 TICs and the eight-lncRNA signature. **(A)** Patients were divided into low-risk groups and high-risk groups based on the best cutoff defined by the “surv_cutpoint ()” function of the “survminer” R package. The Wilcoxon rank-sum was applied to detect the difference in TIC distribution between the high and low-risk groups. **(B)** The Pearson coefficient examined the correlations between TICs and our signature. Here, we only plotted the TIC correlation with a *p-*value < 0.05. **(C)** The results of Wilcoxon’s rank-sum test and the Pearson coefficient were intersected to determine stable and critical TICs. **(D)** We performed Cox analysis to determine the survival predicting power for the 22 TICs. **(E)** Kaplan-Meier estimators were plotted to check the TICs that can differ the survival possibility between low and high-risk groups. Only showed Kaplan-Meier curves with *p-*values < 0.05. **(F)** The distribution of T-cell inflamed scores of the high- and low -risk groups is displayed in the form of a box plot. Significant differences were detected by the Wilcoxon test. HR, hazard ratio; TIC, tumor-infiltrating immune cell; *p*-value < 0.05 is considered significant; ns: *p-*value > 0.05; *: *p-*value < 0.05; **: *p-*value < 0.01; ***: *p-*value < 0.001; ****: *p*-value < 0.0001.

Supplementary Figure S5A details the distribution of the 22 TICs in each patient and high and low-risk groups. Supplementary Figure S5B shows the mutual internal relationships of the 22 TICs in the LUADs. As shown in Figure 7A, the Wilcoxon rank sum test identified 9 TICs related to the risk score. The Pearson coefficient (Figure 7B; Supplementary Table S5) found 12 TICs closely linked to our signature. In summary (Figure 7C), a total of 9 TICs are significantly related to the lncRNA signature, which included Mast cells resting, Macrophages M0, B cells memory, Mast cells activated, T cells CD4 memory activated, T cells CD4 memory resting, Neutrophils, Dendritic cells activated, and Monocytes. Specifically, our signature was positively correlated with Macrophages M0, Mast cells activated, T cells CD4 memory activated, Neutrophils, Dendritic cells activated, and negatively correlated with the rest.

We then constructed the Cox analysis (Figure 7D) and Kaplan–Meier curve (Figure 7E) to assess the survival predictive power of each of the 22 TICs. Cox analysis can tell that Mast cells resting (HR = 0.764, 95% CI = 0.645–0.905, *p* = 1.84e-03) and Mast cells activated (HR = 1.141, 95% CI = 1.019–1.279, *p* = 2.25e-02) significantly affected the LUAD prognosis. Furthermore, Mast cells resting, Mast cells activated, and Dendritic cells resting significantly impacted the survival possibility of LUAD in the constructed Kaplan-Meier analysis (Figure 7E; Supplementary Table S6). On the whole, the survival predictive analysis suggested the Mast cells resting and Mast cells activated pronounced closely connected to LUAD outcomes. T-cell related score were obtained from the 22 TICs and combined as T-cell inflamed score. Figure 7F revealed a disparity in the T-cell inflamed score between the high-risk and low-risk score groups, which was unexpected. However, no statistical difference was observed after conducting a correlation test between the T-cell inflamed and risk scores (Supplementary Figure S5C). The KM analysis of the T-cell inflamed score indicated that it had no impact on the prognosis of LUAD (Supplementary Figure S5D).

To sum, our study in this section revealed that mast cells, which include the mast cells resting and mast cells activated, significantly correlated with the eight-lncRNA signature and bounded up with the LUAD outcomes, insinuating that mast cells infiltration might play an important part in our discovered signature.

## Discussion

Programmed cell death has dramatically expanded the anticancer arsenal. Since ferroptosis, necroptosis, and pyroptosis were discovered, we have better-understood carcinogenesis mechanisms and clinical assessment (Lelievre et al., 2020; Ge et al., 2022). Copper plays an essential cofactor in multiple biological processes during tumor metastasis (Lelievre et al., 2020; Ge et al., 2022). A recent scientific study proposes a new form of programmed cell death: cuproptosis. Cuproptosis leads to protein aggregation, iron-sulfur cluster protein loss, and proteotoxic stress, ultimately leading to cell death (Tsvetkov et al., 2022). Studies have shown that cuproptosis affects the immune microenvironment of various tumors, such as sarcoma (Chu et al., 2022) and melanoma (Lv et al., 2022), thereby affecting the treatment effect and prognosis of patients. Multiple studies have shown that modulating lncRNAs may become a new therapeutic approach for human cancers (Bhan et al., 2017; Peng et al., 2017). However, no studies have focused on systematically assessing cuproptosis-related lncRNA signatures and their association with overall survival in LUAD patients. In the present research, we innovatively adopt the novel cuproptosis concept to establish a cuproptosis-related eight-lncRNA signature predicting LUAD outcomes by digging TCGA and GEO databases (with a total sample size exceeding 1000 cases). A key novelty was the use of comprehensive bioinformatics analysis, such as LASSO regression, Kaplan-Meier curves, Cox models, ROC curves, tAUC, nomogram, and decision curve analysis, as well as the validation in a large independent cohort. Most importantly, we detailed the expression pattern of the signature genes in real world, the role of risk score participating in immunotherapy and distinguishing ability for some anticancer-drug sensitive. The immune infiltration analysis speculated that mast cells may contribute to the predictive power of the signature.

Our signature contains eight lncRNAs (Table 3), which were TSPOAP1-AS1, AC107464.3, AC006449.7, LINC00324, COLCA1, HAGLR, MIR4435-2HG, and NKILA. In our research, MIR4435-2HG and NKILA adversely affected LUAD outcomes, while the remaining genes displayed favorable impacts (Supplementary Figure S3). We performed real-time PCR validation and found that all genes were significantly differentially expressed in LUAD and normal cell lines. In addition, the impact of MIR4435-2HG and NKILA on the extended growth (7∼10 days) of A549 cells was verified through a colony formation assay. Overexpression of MIR4435-2HG and NKILA resulted in an increase in the number of A549 cell line colonies, suggesting their potential involvement in promoting cell growth and motility and their potential significance in the progression of LUAD. There is a strong expression of MIR4435-2HG in lung cancer tissues and tumor cell lines (Zhong et al., 2022). In addition, MIR4435-2HG is involved in the mechanism of action of anticancer drugs, including cisplatin for the treatment of non-small cell lung cancer and colon cancer, and carboplatin for the treatment of triple-negative breast cancer (Zhong et al., 2022). Clinically, NKILA overexpression in tumor-specific CTL and TH1 cells correlates with shorter patient survival and increased apoptosis (Huang et al., 2018; Dai et al., 2021). The NKILA lncRNA stimulates activation-induced cell death in T cells, promoting tumor immunoevasion (Huang et al., 2018). In our wet-experiments, we also confirmed that the MIR4435-2HG and NKILA were up-expressed in LUAD cell lines, which are consistently with their prognosis function found (Supplementary Figure S3) in this study and the previous findings shown above. We uncovered that TSPOAP1-AS1, AC107464.3, AC006449.7, LINC00324, COLCA1, and HAGLR were down-expressed in LUAD cell lines and displayed them having favorable impacts on the LUAD outcomes, which also confirmed the gene signature components’ power. In exceptional cases, the relationship between the differential expression pattern of specific genes and its impact on the prognosis of LUAD is not static, which is related to their regulatory status in the disease, and more research is needed to explore. We found that 8 genes were differentially expressed in the real world, further corroborating the gene signature’s validity and coming forward for further work.

The way of regulated cell death of cuproptosis, apoptosis, necroptosis, pyroptosis, and ferroptosis varies, but they are also somewhat related (Chen X. et al., 2021; Tsvetkov et al., 2022). Apoptosis is mediated by proteolytic enzymes called caspases, which trigger cell death by cleaving specific proteins in the cytoplasm and nucleus. Beyond classical apoptosis, several forms of regulated cell death have been identified. In terms of the exact mechanism by which copper ions cause cell death, several hypotheses have been proposed, including the induction of apoptosis, caspase-independent cell death, the induction of reactive oxygen species, and inhibition of the ubiquitin-proteasome system (Elmore, 2007; Li et al., 2022). Necroptosis is an alternative mode of regulated cell death mimicking features of apoptosis and necrosis (Dhuriya and Sharma, 2018). Ferroptosis is a type of programmed cell death dependent on iron and characterized by the accumulation of lipid peroxides, and is genetically and biochemically distinct from other forms of regulated cell death (Ma et al., 2021). Pharmacological or genetic inhibition of apoptosis (using the caspase inhibitor Z-VAD-FMK or double knockout of BAK and BAX), ferroptosis (using ferrostatin-1 and liproxstatin-1), and necroptosis (using necrostatin-1) failed to suppress cell death induced by the ES–Cu complex in multiple cancer cell lines (Tang et al., 2022). Pyroptosis represents a form of cell death that is triggered by proinflammatory signals and associated with inflammation. Inhibition of cuproptosis has been shown to be involved in mediating inflammation, resulting in the excessive survival and proliferation of a variety of immune cells, such as fibroblast-like synoviocytes, effector T cells, and macrophages (Zhao et al., 2022). From our research, these regulated cell deaths seem to be somewhat related, such as our cuproptosis-related signature correlated with 2321/3681 (63.05%) apoptosis-related genes, 11/20 (55.00%) necroptosis-related genes, 34/50 (68.00%) pyroptosis-related genes, and 222/380 (58.42%) ferroptosis-related genes. Our discovery may provide potential explanations and inspirations for further research of cell death-related tumor mechanisms.

Continued innovation and advances in cancer immunotherapy have extended survival for some patients with deadly cancers (Vanneman and Dranoff, 2012; Waldman et al., 2020). The approach is revolutionizing the field of oncology as more and more cancer patients become eligible for immuno-based therapies (Vanneman and Dranoff, 2012; Waldman et al., 2020). Targeted strategies inhibit tumor progression by interfering with crucial molecular pathways, while immunotherapy produces durable and effective tumor destruction by stimulating the host’s own response (Vanneman and Dranoff, 2012; Waldman et al., 2020). To maximize the benefits of immunotherapy, it is crucial to determine whether a specific biomarker is suitable for the host (Suresh et al., 2018). This study gives hints about which immunotherapy targets to use or under what circumstances to apply. We first found that our risk score was associated with TMB, suggesting that our signature appeared to guide immunotherapy. Next, we followed the trail and found eight checkpoints, including CD8A, CTLA4, GZMA, LAG3, PDCD1, PRF1, TBX2, and TNF related to our eight-lncRNA signature score. We analyzed the sensitivity of each target in different risk score intervals and found that the targeting of PDCD1 was more effective in low-risk patients and triggering for PRF1 can benefit the high-risk group more. Our findings hinted that PDCD1 and PRF1 target therapies might maximize their influences in their specific lncRNA signature risk score zone, respectively. Interestingly, Voli and colleagues stated a strong correlation between PD-L1 expression and intracellular copper levels, noting that copper dysregulators inhibit PD-L1 *in vitro* and *in vivo*, resulting in increased tumor-infiltrating lymphocytes (Voli et al., 2018). In a recent study, Zeng demonstrated that FOXO1, a gene significantly associated with PDCD1, downregulated copper homeostasis is a novel indicator of breast cancer prognosis and immune response (Zeng et al., 2022). The TIDE database related analysis confirmed the above points of our view. Additionally, the real-time PCR results **(** Figures 5L, M) showed that the immune checkpoints PDCD1 and PRF1 were downregulated after overexpression of MIR4435-2HG or NKILA, suggesting that PDCD1 and PRF1 had a mutual regulatory relationship with MIR4435-2HG or NKILA, respectively. The above evidence affirmed that our risk score could bring hope to precisely targeted therapy.

In the section on TICs analysis of our work, we concluded that the mast cells might help our signature bring off secure prognostic power. Mast cell is known for its involvement in allergic disorders, but in recent years, accumulating evidence has shed light on its involvement in cancer, including LUAD (Stoyanov et al., 2012; Bo et al., 2018; Salamon et al., 2020). Mast cells are involved in disease processes characterized by inflammation and tissue remodeling, and their activated forms promote lung health through innate and adaptive immune responses to respiratory pathogens (Virk et al., 2016). Mast cells promote angiogenesis and secrete growth factors, including VEGF, evidence that mast cells are associated with poor prognosis in NSCLC (Virk et al., 2016). Since the immune microenvironment plays a vital part in the malignancy progression, mast cells, as a critical stromal component of the immune system, are undoubtedly a key regulator for maintaining tissue homeostasis. Also, our study examined the correlation between the T-cell inflamed score and the risk score and observed significant variations in the T-cell inflamed score between the high and low-risk score groups. These findings suggest that T-cell research may emerge as a promising avenue for future LUAD studies. There is no dispute that the current findings are far from sufficient, and further research should pay close attention to the parts of mast cells in tumor microenvironment remodeling and tumor immunity.

This study has certain limitations. We generated this eight-lncRNA signature from publicly accessible data. Although it has been confirmed to have stable prognosis ability through applied to another large independent cohort, its clinical applicability needs further confirmation with more parameters. Furthermore, there are still no wet-lab experimental facts to hold up the eight lncRNAs’ parts in cuproptosis-related mechanisms. Therefore, more research, which focuses *in vivo* and *in vitro*, is urgently needed to reveal more clues that support the signature’s promising future.

## Conclusion

The present research constructed a novel and capable cuproptosis-related eight-lncRNA prognostic signature for LUAD. By applying the signature to independent cohort, its stability and broad applicability was validated. The signature owns the potential ability to undertake the role of precise immunotherapy and anticancer drug selection. The immune infiltration analysis hinted that the mast cells might help the signature to maintain its predictive power. Our work may promote the evolution of diagnosis and treatment of LUAD.

## Novelty & impact statements

In the present research, we adopt the novel concept to establish a cuproptosis-related eight-lncRNA signature predicting LUAD outcomes by digging TCGA and GEO databases (with a total sample size exceeding 1000 cases). Most importantly, we detailed the expression pattern of the signature genes in the real world, the role of risk score participating in immunotherapy, and the model’s distinguishing power for anticancer drugs. The mast cells may contribute to the predictive power of the signature.

## Data Availability

Publicly available datasets were analyzed in this study. This data can be found here: The following publicly available datasets were used in this study: TCGA-LUAD: https://gdc.xenahubs.net; GSE29013, GSE30219, GSE31210, GSE37745, and GSE50081: https://www.ncbi.nlm.nih.gov/geo/; The data of real-time PCR can be reached by contacting the corresponding authors.

## Abbreviations

LUAD, lung adenocarcinoma; LASSO, least absolute shrinkage and selection operator Cox regression model; TCGA, The Cancer Genome Atlas; ROC, receiver operating characteristic; AUC, Area under the ROC curve; tAUC, Time-dependent AUC; GSEA, Gene Set Enrichment Analysis; TICs, tumor-infiltrating immune cells; HR, hazard ratio; CI, confidence interval; FDR, false discovery rate; TIDE, Tumor Immune Dysfunction and Exclusion; PC, principal component; KEGG, Kyoto Encyclopedia of Genes and Genomes; IC50, half maximal inhibitory concentration; UMAP, Uniform Manifold Approximation and Projection; PCA, Principal components analysis; OS, overall survival; TMB, Tumor mutational burden.
