# Supplementary material for: A novel defined risk signature of cuproptosis-related long non-coding RNA for predicting prognosis, immune infiltration, and immunotherapy response in lung adenocarcinoma
Source: Front Pharmacol. 2023 Aug 21;14:1146840. doi: 10.3389/fphar.2023.1146840 (PMC10475834; doi:10.3389/fphar.2023.1146840)
Supplement: Supplementary file 1 [file DataSheet1.PDF]

Figure S1

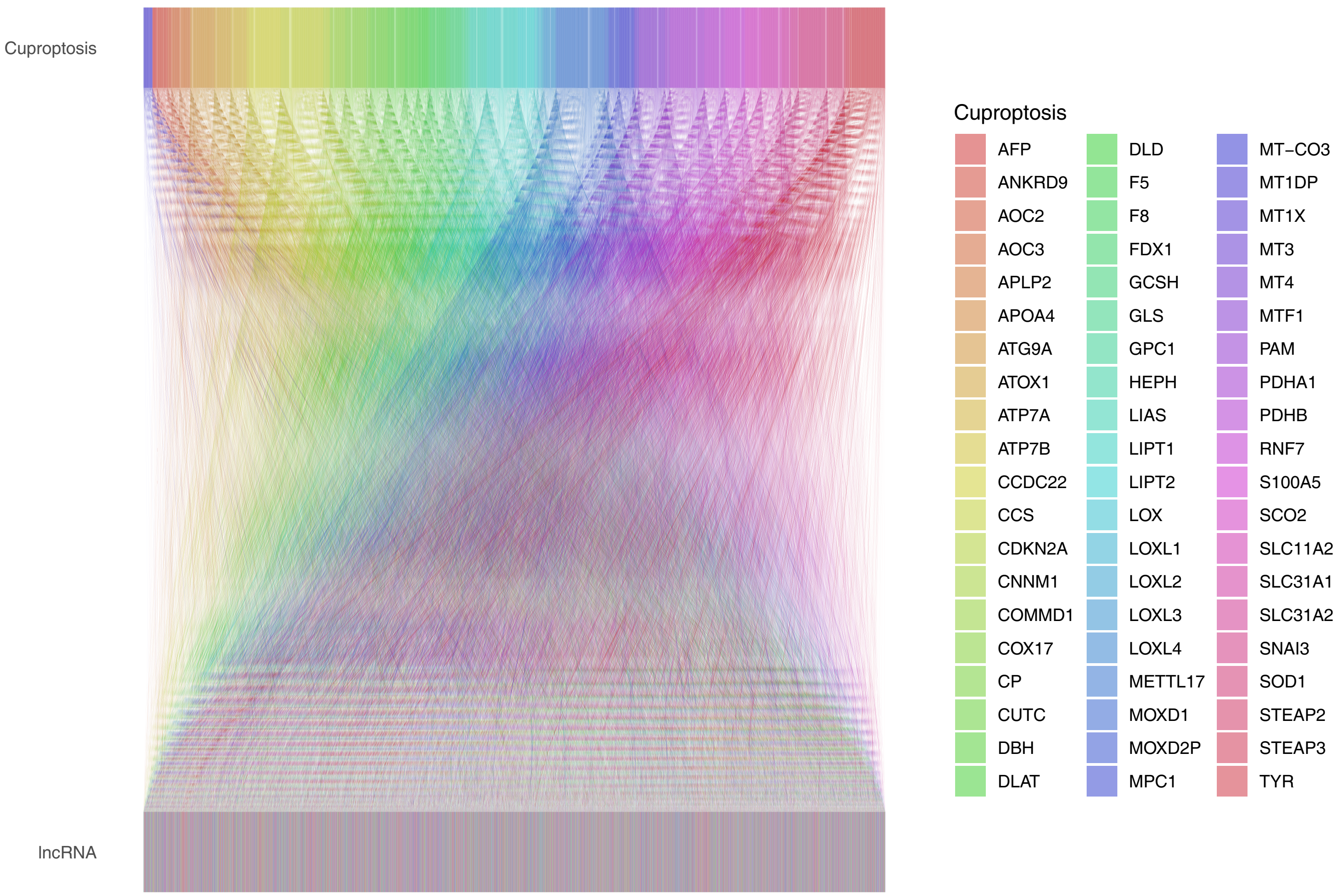
Cuproptosis
lncRNA
Cuproptosis
AFP
ANKRD9
AOC2
AOC3
APLP2
APOA4
ATG9A
ATOX1
ATP7A
ATP7B
CCDC22
CCS
CDKN2A
CNNM1
COMMD1
COX17
CP
CUTC
DBH
DLAT
DLD
F5
F8
FDX1
GCSH
GLS
GPC1
HEPH
LIAS
LIPT1
LIPT2
LOX
LOXL1
LOXL2
LOXL3
LOXL4
METTL17
MOXD1
MOXD2P
MPC1
MT−CO3
MT1DP
MT1X
MT3
MT4
MTF1
PAM
PDHA1
PDHB
RNF7
S100A5
SCO2
SLC11A2
SLC31A1
SLC31A2
SNAI3
SOD1
STEAP2
STEAP3
TYR

Figure S2

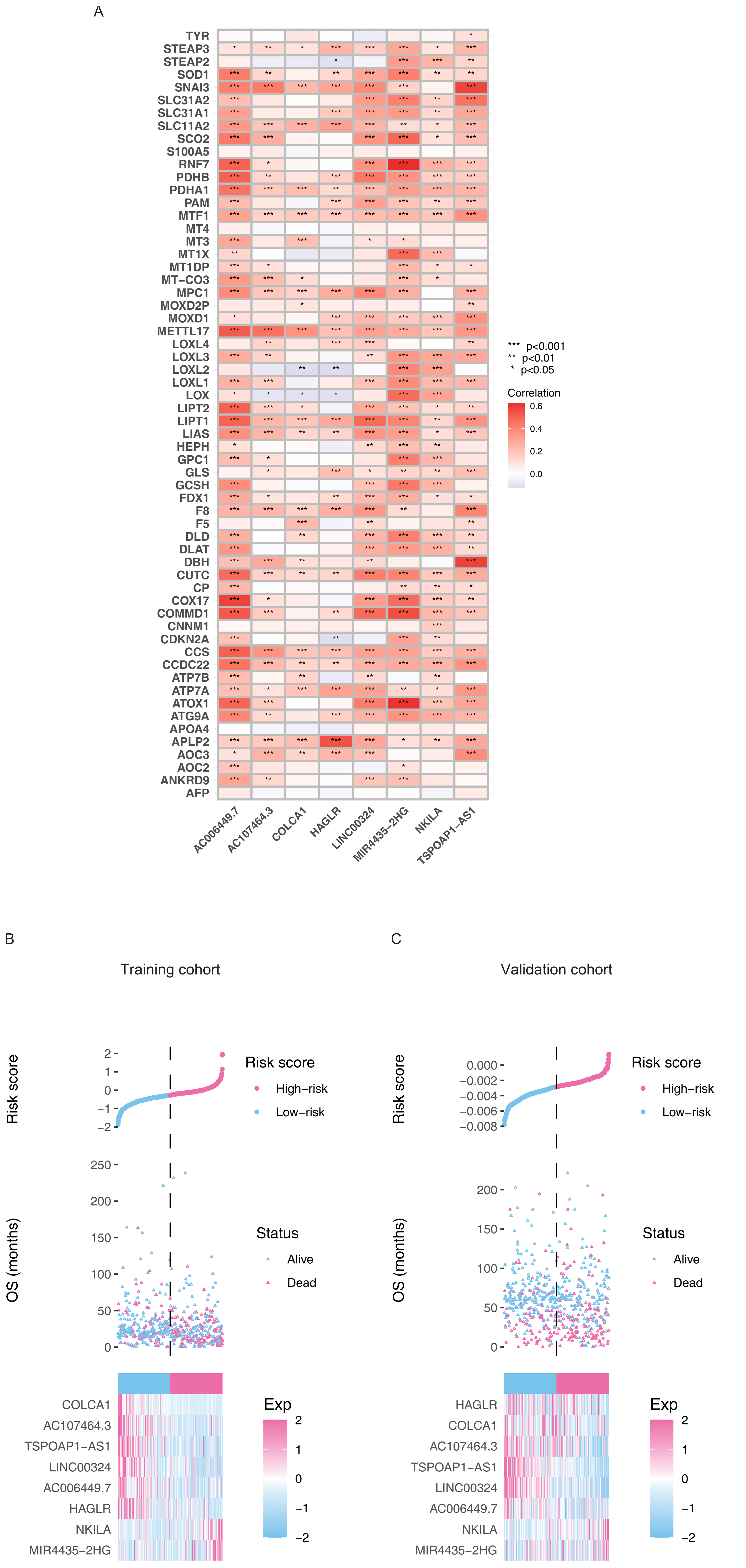
A
TYR
STEAP3
STEAP2
SOD1
SNAI3
SLC31A2
SLC31A1
SLC11A2
SCO2
S100A5
RNF7
PDHB
PDHA1
PAM
MTF1
MT4
MT3
MT1X
MT1DP
MT-CO3
MPC1
MOXD2P
MOXD1
METTL17
LOXL4
LOXL3
LOXL2
LOXL1
LOX
LIPT2
LIPT1
LIAS
HEPH
GPC1
GLS
GCSH
FDX1
F8
F5
DLD
DLAT
DBH
CUTC
CP
COX17
COMMD1
CNNM1
CDKN2A
CCS
CCDC22
ATP7B
ATP7A
ATOX1
ATG9A
APOA4
APLP2
AOC3
AOC2
ANKRD9
AFP
AC006449.7
AC107464.3
COLCA1
HAGLR
LINC00324
MIR4435-2HG
NKILA
TSPOAP1-AS1
*** p<0.001
** p<0.01
* p<0.05
Correlation
0.6
0.4
0.2
0.0
B
Training cohort
Risk score
Risk score
High-risk
Low-risk
OS (months)
Status
Alive
Dead
COLCA1
AC107464.3
TSPOAP1-AS1
LINC00324
AC006449.7
HAGLR
NKILA
MIR4435-2HG
Exp
C
Validation cohort
Risk score
Risk score
High-risk
Low-risk
OS (months)
Status
Alive
Dead
HAGLR
COLCA1
AC107464.3
TSPOAP1-AS1
LINC00324
AC006449.7
NKILA
MIR4435-2HG
Exp

Figure S3

A

Kaplan–Meier estimator

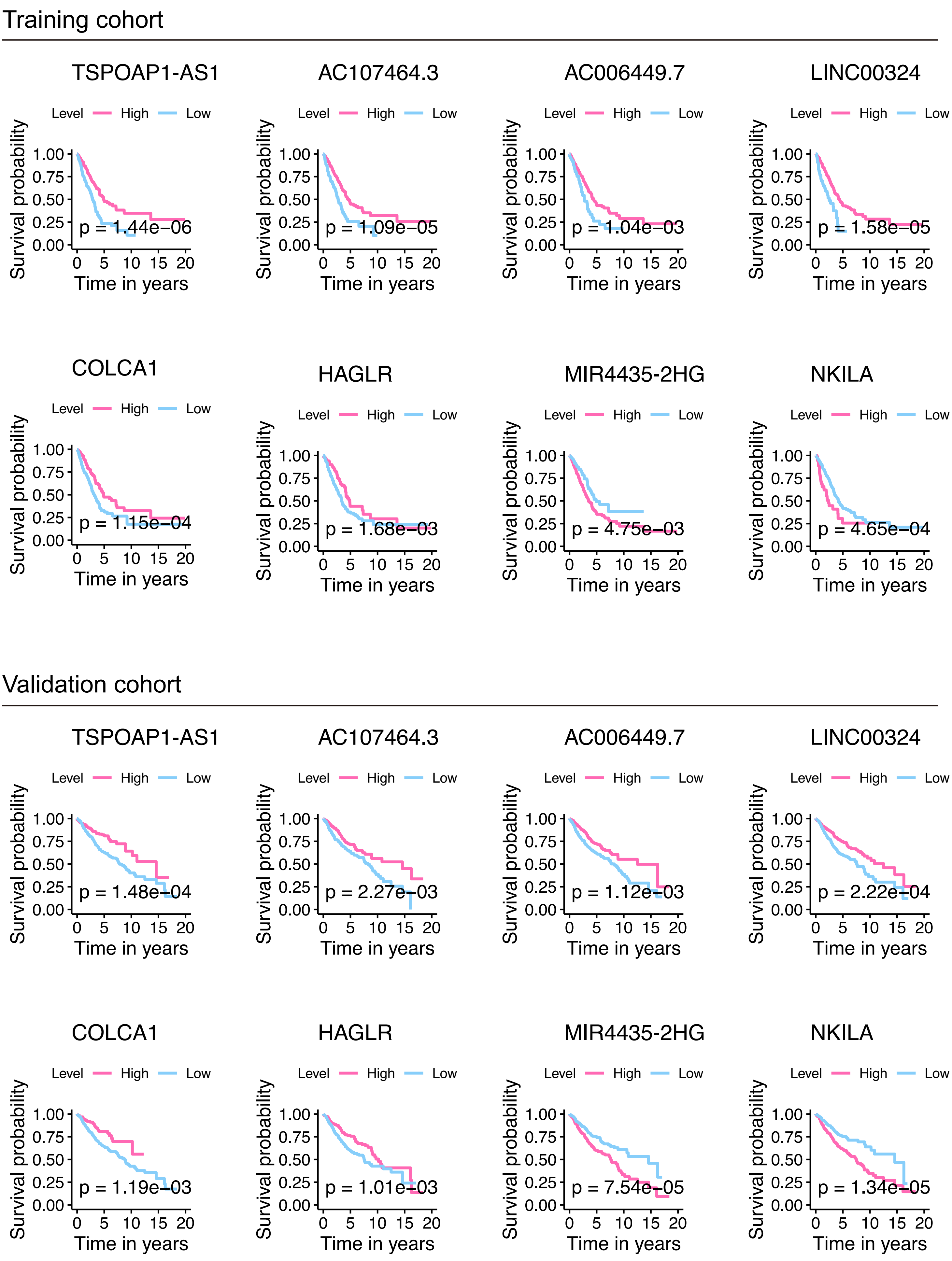

B

Univariate Cox regression

Variable	HR	L95%	H95%	p
**Training cohort**				
AC006449.7	0.9984	0.9968	0.9999	3.85e−02
AC107464.3	0.9931	0.9876	0.9987	1.52e−02
COLCA1	0.9998	0.9997	1.0000	1.03e−02
HAGLR	0.9999	0.9999	1.0000	1.76e−02
LINC00324	0.9979	0.9965	0.9994	5.81e−03
MIR4435−2HG	1.0002	1.0000	1.0003	3.56e−02
NKILA	1.0010	1.0005	1.0015	1.41e−04
TSPOAP1−AS1	0.9934	0.9892	0.9976	2.19e−03
**Validation cohort**				
AC006449.7	0.3308	0.1354	0.8086	1.53e−02
AC107464.3	0.2843	0.1072	0.7541	1.15e−02
COLCA1	0.7935	0.6802	0.9257	3.27e−03
HAGLR	0.7776	0.6421	0.9416	1.00e−02
LINC00324	0.7202	0.5399	0.9607	2.56e−02
MIR4435−2HG	2.4731	1.5598	3.9210	1.18e−04
NKILA	1.4194	1.0956	1.8389	8.03e−03
TSPOAP1−AS1	0.3438	0.1801	0.6564	1.21e−03

Figure S4

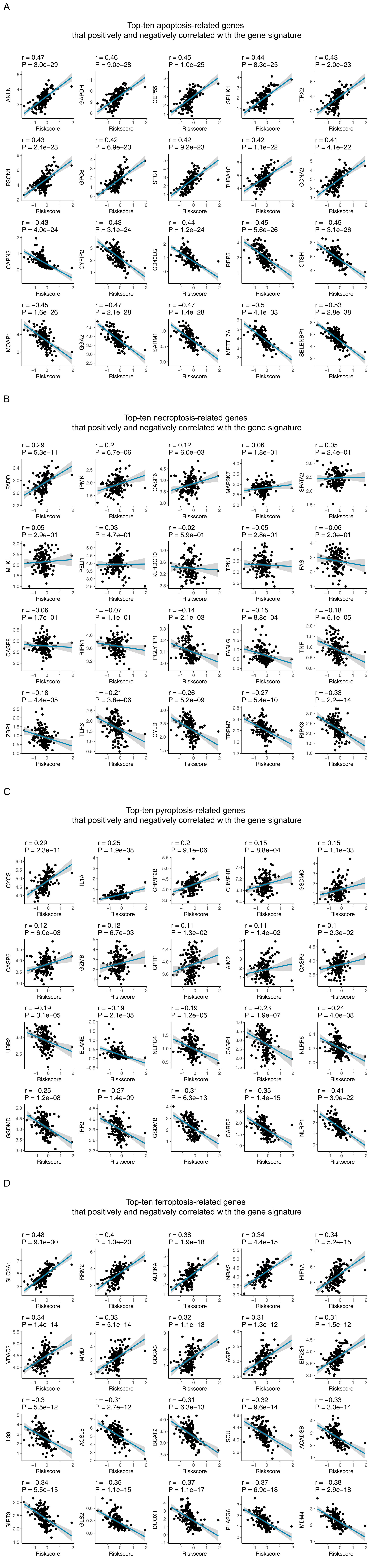
A
Top-ten apoptosis-related genes
that positively and negatively correlated with the gene signature
ANLN r = 0.47 P = 3.0e−29
GAPDH r = 0.46 P = 9.0e−28
CEP55 r = 0.45 P = 1.0e−25
SPHK1 r = 0.44 P = 8.3e−25
TPX2 r = 0.43 P = 2.0e−23
FSCN1 r = 0.43 P = 2.4e−23
GPC6 r = 0.42 P = 6.9e−23
STC1 r = 0.42 P = 9.2e−23
TUBA1C r = 0.42 P = 1.1e−22
CCNA2 r = 0.41 P = 4.1e−22
CAPN3 r = −0.43 P = 4.0e−24
CYFIP2 r = −0.43 P = 3.1e−24
CD40LG r = −0.44 P = 1.2e−24
RBP5 r = −0.45 P = 5.6e−26
CTSH r = −0.45 P = 3.1e−26
MOAP1 r = −0.45 P = 1.6e−26
GGA2 r = −0.47 P = 2.1e−28
SARM1 r = −0.47 P = 1.4e−28
METTL7A r = −0.5 P = 4.1e−33
SELENBP1 r = −0.53 P = 2.8e−38
Riskscore
B
Top-ten necroptosis-related genes
that positively and negatively correlated with the gene signature
FADD r = 0.29 P = 5.3e−11
IPMK r = 0.2 P = 6.7e−06
CASP6 r = 0.12 P = 6.0e−03
MAP3K7 r = 0.06 P = 1.8e−01
SPATA2 r = 0.05 P = 2.4e−01
MLKL r = 0.05 P = 2.9e−01
PELI1 r = 0.03 P = 4.7e−01
KLHDC10 r = −0.02 P = 5.9e−01
ITPK1 r = −0.05 P = 2.8e−01
FAS r = −0.06 P = 2.0e−01
CASP8 r = −0.06 P = 1.7e−01
RIPK1 r = −0.07 P = 1.1e−01
PGLYRP1 r = −0.14 P = 2.1e−03
FASLG r = −0.15 P = 8.8e−04
TNF r = −0.18 P = 5.1e−05
ZBP1 r = −0.18 P = 4.4e−05
TLR3 r = −0.21 P = 3.8e−06
CYLD r = −0.26 P = 5.2e−09
TRPM7 r = −0.27 P = 5.4e−10
RIPK3 r = −0.33 P = 2.2e−14
Riskscore
C
Top-ten pyroptosis-related genes
that positively and negatively correlated with the gene signature
CYCS r = 0.29 P = 2.3e−11
IL1A r = 0.25 P = 1.9e−08
CHMP2B r = 0.2 P = 9.1e−06
CHMP4B r = 0.15 P = 8.8e−04
GSDMC r = 0.15 P = 1.1e−03
CASP6 r = 0.12 P = 6.0e−03
GZMB r = 0.12 P = 6.7e−03
CPTP r = 0.11 P = 1.3e−02
AIM2 r = 0.11 P = 1.4e−02
CASP3 r = 0.1 P = 2.3e−02
UBR2 r = −0.19 P = 3.1e−05
ELANE r = −0.19 P = 2.1e−05
NLRC4 r = −0.19 P = 1.2e−05
CASP1 r = −0.23 P = 1.9e−07
NLRP6 r = −0.24 P = 4.0e−08
GSDMD r = −0.25 P = 1.2e−08
IRF2 r = −0.27 P = 1.4e−09
GSDMB r = −0.31 P = 6.3e−13
CARD8 r = −0.35 P = 1.4e−15
NLRP1 r = −0.41 P = 3.9e−22
Riskscore
D
Top-ten ferroptosis-related genes
that positively and negatively correlated with the gene signature
SLC2A1 r = 0.48 P = 9.1e−30
RRM2 r = 0.4 P = 1.3e−20
AURKA r = 0.38 P = 1.9e−18
NRAS r = 0.34 P = 4.4e−15
HIF1A r = 0.34 P = 5.2e−15
VDAC2 r = 0.34 P = 1.4e−14
MMD r = 0.33 P = 5.1e−14
CDCA3 r = 0.32 P = 1.1e−13
AGPS r = 0.31 P = 1.3e−12
EIF2S1 r = 0.31 P = 1.5e−12
IL33 r = −0.3 P = 5.5e−12
ACSL5 r = −0.31 P = 2.7e−12
BCAT2 r = −0.31 P = 6.3e−13
ISCU r = −0.32 P = 9.6e−14
ACADSB r = −0.33 P = 3.0e−14
SIRT3 r = −0.34 P = 5.5e−15
GLS2 r = −0.35 P = 1.1e−15
DUOX1 r = −0.37 P = 1.1e−17
PLA2G6 r = −0.37 P = 6.9e−18
MDM4 r = −0.38 P = 2.9e−18
Riskscore

Figure S5

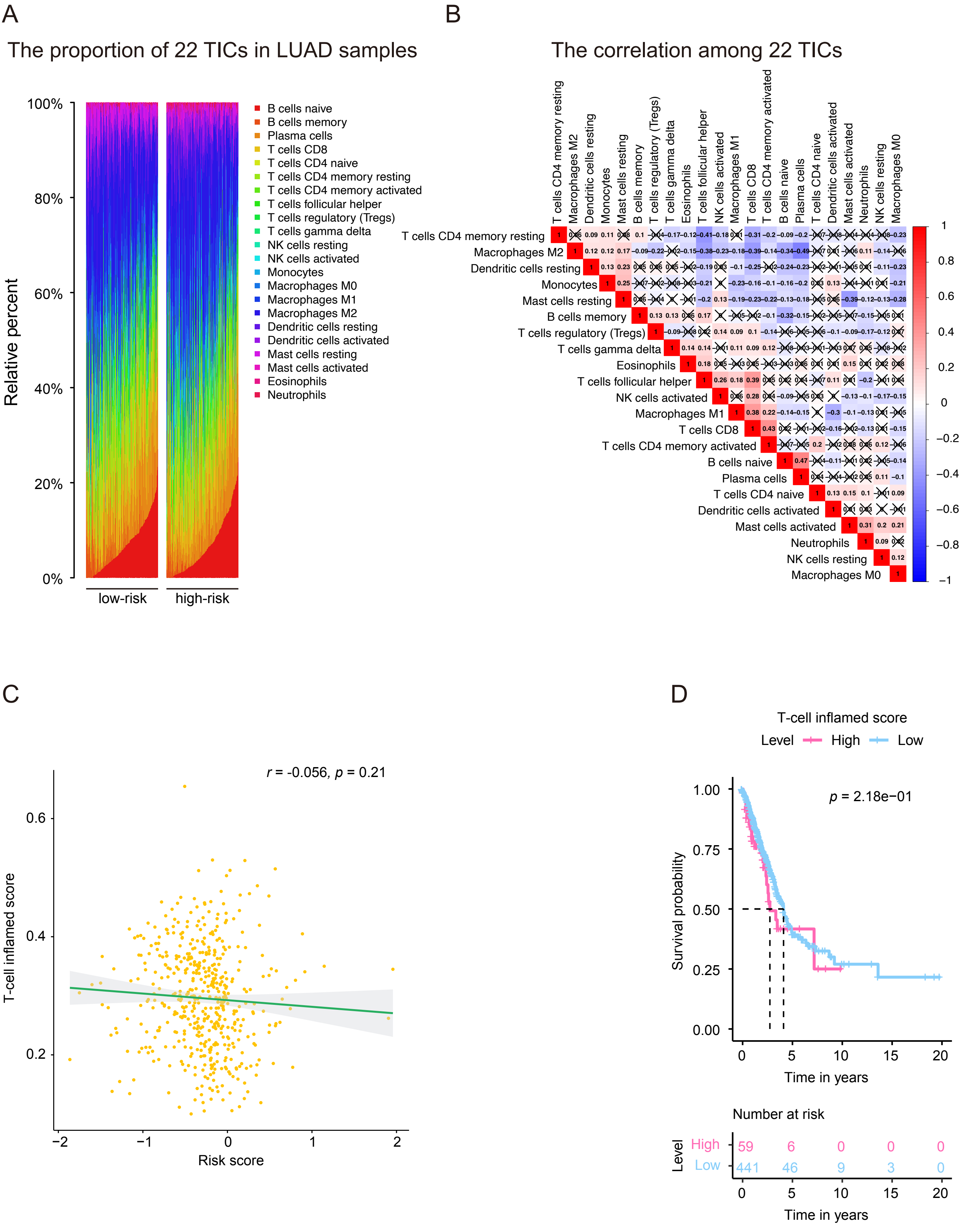
A
The proportion of 22 TICs in LUAD samples
100%
80%
60%
40%
20%
0%
Relative percent
low-risk
high-risk
B cells naive
B cells memory
Plasma cells
T cells CD8
T cells CD4 naive
T cells CD4 memory resting
T cells CD4 memory activated
T cells follicular helper
T cells regulatory (Tregs)
T cells gamma delta
NK cells resting
NK cells activated
Monocytes
Macrophages M0
Macrophages M1
Macrophages M2
Dendritic cells resting
Dendritic cells activated
Mast cells resting
Mast cells activated
Eosinophils
Neutrophils
B
The correlation among 22 TICs
T cells CD4 memory resting
Macrophages M2
Dendritic cells resting
Monocytes
Mast cells resting
B cells memory
T cells regulatory (Tregs)
T cells gamma delta
Eosinophils
T cells follicular helper
NK cells activated
Macrophages M1
T cells CD8
T cells CD4 memory activated
B cells naive
Plasma cells
T cells CD4 naive
Dendritic cells activated
Mast cells activated
Neutrophils
NK cells resting
Macrophages M0
1
0.8
0.6
0.4
0.2
0
−0.2
−0.4
−0.6
−0.8
−1
C
r = -0.056, p = 0.21
T-cell inflamed score
0.6
0.4
0.2
−2
−1
0
1
2
Risk score
D
T-cell inflamed score
Level
High
Low
p = 2.18e−01
Survival probability
1.00
0.75
0.50
0.25
0.00
0
5
10
15
20
Time in years
Number at risk
Level
High 59 6 0 0 0
Low 441 46 9 3 0
Time in years

Table S1. LncRNAs that were significantly associated with the cuproptosis-related genes were determined using the Pearson algorithm (cor.>0.3 or <-0.3, p-value<0.05).

Cuproptosis-related genes	LncRNAs	Cor.	P-value	Regulation
AFP	AC004241.2	0.306509016	2.45E-12	postive
AFP	AC004862.1	0.976517345	0	postive
AFP	AC004870.3	0.466997889	1.89E-28	postive
AFP	AC005229.4	0.305727406	2.81E-12	postive
AFP	AC005280.1	0.656249187	6.64E-63	postive
AFP	AC006205.2	0.440837689	3.45E-25	postive
AFP	AC006357.1	0.308386852	1.78E-12	postive
AFP	AC007100.1	0.938034625	2.39E-231	postive
AFP	AC007298.2	0.587919114	8.30E-48	postive
AFP	AC008154.2	0.311459056	1.04E-12	postive
AFP	AC008267.5	0.370689228	9.86E-18	postive
AFP	AC008443.3	0.35361775	3.57E-16	postive
AFP	AC008601.1	0.351413281	5.59E-16	postive
AFP	AC008752.2	0.319308106	2.59E-13	postive
AFP	AC008760.2	0.367102209	2.14E-17	postive
AFP	AC009403.1	0.51565762	2.58E-35	postive
AFP	AC009407.1	0.454135447	8.23E-27	postive
AFP	AC010737.1	0.511259543	1.20E-34	postive
AFP	AC010761.6	0.40155502	8.51E-21	postive
AFP	AC010907.2	0.445985752	8.28E-26	postive
AFP	AC012087.2	0.373874474	4.93E-18	postive
AFP	AC012146.1	0.661307391	3.51E-64	postive
AFP	AC012409.1	0.531139817	9.64E-38	postive
AFP	AC012467.2	0.419451106	1.00E-22	postive
AFP	AC012676.1	0.580244355	2.49E-46	postive
AFP	AC013509.1	0.36362706	4.47E-17	postive
AFP	AC015540.1	0.379160858	1.53E-18	postive
AFP	AC015917.2	0.863556708	3.43E-150	postive
AFP	AC016044.1	0.587467074	1.02E-47	postive
AFP	AC016550.2	0.451449748	1.77E-26	postive
AFP	AC017091.1	0.317516459	3.57E-13	postive
AFP	AC018690.1	0.33915009	6.34E-15	postive
AFP	AC018904.2	0.496603783	1.70E-32	postive
AFP	AC019185.1	0.720193553	4.18E-81	postive
AFP	AC020743.3	0.310286204	1.28E-12	postive
AFP	AC021074.3	0.982161559	0	postive
AFP	AC021146.12	0.367590682	1.92E-17	postive
AFP	AC022816.1	0.947551457	7.46E-249	postive
AFP	AC023090.1	0.374867637	3.96E-18	postive
AFP	AC025254.1	0.871142023	6.10E-156	postive
AFP	AC026367.3	0.314889866	5.69E-13	postive
AFP	AC026462.3	0.89981998	1.56E-181	postive
AFP	AC026462.4	0.330686724	3.19E-14	postive
AFP	AC027237.4	0.400818154	1.02E-20	postive
AFP	AC061961.2	0.880555766	1.32E-163	postive

AFP	AC061975.6	0.455224027	6.02E-27	postive
AFP	AC064834.1	0.373615524	5.22E-18	postive
AFP	AC067930.3	0.873716816	5.62E-158	postive
AFP	AC068506.1	0.746918509	2.61E-90	postive
AFP	AC068631.1	0.471571409	4.76E-29	postive
AFP	AC073107.1	0.510277495	1.68E-34	postive
AFP	AC073111.1	0.430710227	5.32E-24	postive
AFP	AC073365.1	0.661291537	3.54E-64	postive
AFP	AC073842.1	0.655850076	8.35E-63	postive
AFP	AC074237.1	0.43253712	3.27E-24	postive
AFP	AC078788.2	0.308840034	1.64E-12	postive
AFP	AC079921.2	0.65076163	1.51E-61	postive
AFP	AC083902.1	0.410473669	9.61E-22	postive
AFP	AC084036.1	0.605234396	2.74E-51	postive
AFP	AC084082.1	0.395468259	3.63E-20	postive
AFP	AC084125.4	0.48262793	1.55E-30	postive
AFP	AC084781.1	0.378271233	1.87E-18	postive
AFP	AC084781.2	0.379157141	1.53E-18	postive
AFP	AC087241.2	0.424342442	2.83E-23	postive
AFP	AC087482.1	0.610507042	2.16E-52	postive
AFP	AC090164.3	0.47784985	6.92E-30	postive
AFP	AC090515.5	0.324673196	9.74E-14	postive
AFP	AC090772.1	0.333135425	2.01E-14	postive
AFP	AC091057.1	0.483218625	1.29E-30	postive
AFP	AC091133.3	0.373978446	4.82E-18	postive
AFP	AC091153.3	0.317850734	3.36E-13	postive
AFP	AC092295.1	0.394921506	4.13E-20	postive
AFP	AC092343.1	0.462024076	8.30E-28	postive
AFP	AC092490.1	0.474065135	2.22E-29	postive
AFP	AC092580.2	0.405915966	2.95E-21	postive
AFP	AC092925.1	0.999895665	0	postive
AFP	AC092941.2	0.486922	3.97E-31	postive
AFP	AC093484.2	0.605350421	2.59E-51	postive
AFP	AC093582.1	0.344401964	2.27E-15	postive
AFP	AC096536.2	0.324660839	9.76E-14	postive
AFP	AC097468.1	0.305258024	3.04E-12	postive
AFP	AC097501.1	0.568435742	3.93E-44	postive
AFP	AC097512.1	0.543017537	1.09E-39	postive
AFP	AC099552.1	0.99577312	0	postive
AFP	AC099552.3	0.992375499	0	postive
AFP	AC100839.2	0.39907536	1.54E-20	postive
AFP	AC100847.1	0.431143576	4.74E-24	postive
AFP	AC100872.2	0.386153287	3.16E-19	postive
AFP	AC103746.1	0.640088217	5.50E-59	postive
AFP	AC104051.2	0.979545205	0	postive
AFP	AC104078.1	0.445987296	8.27E-26	postive
AFP	AC105105.2	0.793754694	1.32E-109	postive
AFP	AC106874.1	0.37555612	3.41E-18	postive
AFP	AC108517.1	0.859328797	3.90E-147	postive
AFP	AC108704.1	0.359550307	1.05E-16	postive

AFP	AC109927.1	0.349765278	7.80E-16	postive
AFP	AC112219.2	0.317325551	3.69E-13	postive
AFP	AC114485.1	0.877043081	1.13E-160	postive
AFP	AC115618.1	0.524241131	1.21E-36	postive
AFP	AC115618.2	0.304730376	3.33E-12	postive
AFP	AC115619.1	0.994783183	0	postive
AFP	AC116348.3	0.31638644	4.36E-13	postive
AFP	AC116348.5	0.516429803	1.96E-35	postive
AFP	AC116609.2	0.390571583	1.14E-19	postive
AFP	AC117386.2	0.347084504	1.33E-15	postive
AFP	AC117394.2	0.431553577	4.25E-24	postive
AFP	AC121761.1	0.351529221	5.46E-16	postive
AFP	AC122108.3	0.671727892	6.84E-67	postive
AFP	AC124947.2	0.380239806	1.20E-18	postive
AFP	AC125616.1	0.485120118	7.05E-31	postive
AFP	AC126323.1	0.60332887	6.77E-51	postive
AFP	AC126323.3	0.877845026	2.46E-161	postive
AFP	AC126763.1	0.323107336	1.30E-13	postive
AFP	AC131009.1	0.383780342	5.42E-19	postive
AFP	AC131009.3	0.33844113	7.28E-15	postive
AFP	AC140912.1	0.34998093	7.47E-16	postive
AFP	AC145207.1	0.483948446	1.02E-30	postive
AFP	AC145423.2	0.303902324	3.83E-12	postive
AFP	AC232271.1	0.314036615	6.62E-13	postive
AFP	AC244250.1	0.352025053	4.94E-16	postive
AFP	AC245452.5	0.376891812	2.54E-18	postive
AFP	AC246785.3	0.491844583	8.11E-32	postive
AFP	ADCY6-DT	0.601038731	2.00E-50	postive
AFP	AF196972.1	0.315089864	5.49E-13	postive
AFP	AF240627.1	0.306541952	2.44E-12	postive
AFP	AL008723.2	0.302495762	4.86E-12	postive
AFP	AL009179.1	0.305957962	2.70E-12	postive
AFP	AL024507.3	0.3780181	1.98E-18	postive
AFP	AL031727.2	0.340835969	4.57E-15	postive
AFP	AL032821.1	0.358704255	1.25E-16	postive
AFP	AL049543.1	0.504325325	1.29E-33	postive
AFP	AL117329.1	0.35180119	5.17E-16	postive
AFP	AL138826.1	0.921830777	3.97E-207	postive
AFP	AL157400.2	0.317874724	3.34E-13	postive
AFP	AL157896.1	0.964993186	1.29E-291	postive
AFP	AL158071.4	0.4853493	6.55E-31	postive
AFP	AL158850.1	0.331567259	2.70E-14	postive
AFP	AL161645.1	0.458024101	2.68E-27	postive
AFP	AL161938.1	0.359461682	1.07E-16	postive
AFP	AL162411.1	0.584959875	3.11E-47	postive
AFP	AL163953.1	0.312536441	8.62E-13	postive
AFP	AL353622.2	0.336005787	1.16E-14	postive
AFP	AL353708.1	0.317496297	3.58E-13	postive
AFP	AL354811.1	0.323862049	1.13E-13	postive
AFP	AL355096.1	0.452460741	1.33E-26	postive

AFP	AL355102.4	0.793989639	1.03E-109	postive
AFP	AL356608.1	0.323087542	1.30E-13	postive
AFP	AL356652.1	0.729983895	2.39E-84	postive
AFP	AL359878.2	0.326042113	7.57E-14	postive
AFP	AL390038.1	0.33674794	1.01E-14	postive
AFP	AL391095.2	0.349445497	8.32E-16	postive
AFP	AL391095.3	0.924549084	8.38E-211	postive
AFP	AL591848.2	0.363143584	4.95E-17	postive
AFP	AL592494.1	0.964349925	1.10E-289	postive
AFP	AL596211.1	0.684520679	2.25E-70	postive
AFP	AL929236.1	0.314961764	5.62E-13	postive
AFP	AP001830.2	0.346971184	1.36E-15	postive
AFP	AP005264.4	0.914033469	2.79E-197	postive
AFP	AP005264.5	0.725653762	6.75E-83	postive
AFP	AP005329.2	0.659119595	1.26E-63	postive
AFP	APOC1P1	0.783542565	5.44E-105	postive
AFP	ARAP1-AS1	0.664657113	4.85E-65	postive
AFP	ASMTL-AS1	0.303834516	3.87E-12	postive
AFP	B3GALT1-AS1	0.336826883	9.93E-15	postive
AFP	BCAR3-AS1	0.677581009	1.84E-68	postive
AFP	C3P1	0.751549436	5.05E-92	postive
AFP	C5orf34-AS1	0.815270833	3.06E-120	postive
AFP	CERNA1	0.31055715	1.22E-12	postive
AFP	CPS1-IT1	0.339877615	5.51E-15	postive
AFP	CSE1L-AS1	0.340907433	4.51E-15	postive
AFP	CTD-2024D23.1	0.328343358	4.94E-14	postive
AFP	CTD-2240J17.1	0.403561433	5.24E-21	postive
AFP	CU633904.2	0.344277123	2.33E-15	postive
AFP	DHRSX-IT1	0.510557102	1.53E-34	postive
AFP	DLGAP4-AS1	0.310485328	1.23E-12	postive
AFP	DRAIC	0.420308571	8.03E-23	postive
AFP	ELOVL2-AS1	0.817086679	3.34E-121	postive
AFP	EMSLR	0.348928089	9.23E-16	postive
AFP	F11-AS1	0.540865366	2.48E-39	postive
AFP	FAM99B	0.326891941	6.47E-14	postive
AFP	FAR2P4	0.375078638	3.78E-18	postive
AFP	GCC2-AS1	0.303529446	4.08E-12	postive
AFP	GOLGA2P11	0.550524118	5.80E-41	postive
AFP	GOLGA8M	0.339545601	5.88E-15	postive
AFP	GTF2IRD2P1	0.345719934	1.75E-15	postive
AFP	H2BP1	0.302027424	5.26E-12	postive
AFP	HERC2P3	0.58428391	4.20E-47	postive
AFP	HULC	0.402682549	6.48E-21	postive
AFP	KCNIP2-AS1	0.368008515	1.76E-17	postive
AFP	KDM4A-AS1	0.305178134	3.08E-12	postive
AFP	KTN1-AS1	0.307683934	2.00E-12	postive
AFP	LGR4-AS1	0.553845132	1.55E-41	postive
AFP	LINC00106	0.349700075	7.90E-16	postive
AFP	LINC00189	0.49848242	9.15E-33	postive
AFP	LINC00473	0.361346079	7.23E-17	postive

AFP	LINC00533	0.963786504	5.11E-288	postive
AFP	LINC00602	0.420685272	7.29E-23	postive
AFP	LINC00870	0.353138901	3.94E-16	postive
AFP	LINC00898	0.494096909	3.89E-32	postive
AFP	LINC00992	0.813769202	1.87E-119	postive
AFP	LINC01136	0.44197008	2.52E-25	postive
AFP	LINC01234	0.472294837	3.82E-29	postive
AFP	LINC01273	0.40200109	7.64E-21	postive
AFP	LINC01399	0.39544051	3.66E-20	postive
AFP	LINC01447	0.526009794	6.34E-37	postive
AFP	LINC01465	0.300307587	7.02E-12	postive
AFP	LINC01545	0.367247845	2.07E-17	postive
AFP	LINC01560	0.448327328	4.29E-26	postive
AFP	LINC01608	0.399401175	1.43E-20	postive
AFP	LINC01619	0.331258779	2.86E-14	postive
AFP	LINC01632	0.955832908	5.54E-267	postive
AFP	LINC01639	0.377174352	2.38E-18	postive
AFP	LINC01647	0.702536353	1.36E-75	postive
AFP	LINC01687	0.570390941	1.72E-44	postive
AFP	LINC01809	0.313487011	7.29E-13	postive
AFP	LINC01816	0.682181736	1.01E-69	postive
AFP	LINC01827	0.689359198	9.75E-72	postive
AFP	LINC01887	0.40877534	1.46E-21	postive
AFP	LINC02032	0.670267817	1.67E-66	postive
AFP	LINC02136	0.486644106	4.33E-31	postive
AFP	LINC02147	0.497708398	1.18E-32	postive
AFP	LINC02151	0.494354127	3.57E-32	postive
AFP	LINC02197	0.678963922	7.72E-69	postive
AFP	LINC02206	0.458863689	2.10E-27	postive
AFP	LINC02308	0.347372947	1.26E-15	postive
AFP	LINC02370	0.714764647	2.29E-79	postive
AFP	LINC02429	0.566516435	8.77E-44	postive
AFP	LINC02496	0.398062994	1.96E-20	postive
AFP	LINC02525	0.592281893	1.15E-48	postive
AFP	LINC02532	0.480719772	2.83E-30	postive
AFP	LINC02701	0.515937156	2.34E-35	postive
AFP	LINC02799	0.458045771	2.66E-27	postive
AFP	LPGAT1-AS1	0.360791544	8.12E-17	postive
AFP	MIR122HG	0.998240742	0	postive
AFP	MSH5-SAPCD1	0.509550051	2.16E-34	postive
AFP	NF1P10	0.317336419	3.68E-13	postive
AFP	OGFRP1	0.370313023	1.07E-17	postive
AFP	OR8G3P	0.316351891	4.39E-13	postive
AFP	PAQR9-AS1	0.999733384	0	postive
AFP	PDCD4-AS1	0.358176032	1.40E-16	postive
AFP	PDCD6IPP1	0.326406072	7.08E-14	postive
AFP	PLGLA	0.566838221	7.67E-44	postive
AFP	PPY2P	0.424340237	2.84E-23	postive
AFP	PROSER2-AS1	0.669648857	2.43E-66	postive
AFP	PXN-AS1	0.415880681	2.48E-22	postive

AFP	RMST	0.502290809	2.56E-33	postive
AFP	RP11-392O1.4	0.471080347	5.53E-29	postive
AFP	RP11-552M14.1	0.549654554	8.17E-41	postive
AFP	RP11-63K6.7	0.452768489	1.22E-26	postive
AFP	RP11-855A2.5	0.433768537	2.35E-24	postive
AFP	RP1-90G24.6	0.358692282	1.26E-16	postive
AFP	RP3-323A16.1	0.375489816	3.46E-18	postive
AFP	RP5-968D22.3	0.44877781	3.78E-26	postive
AFP	SAPCD1-AS1	0.789461362	1.24E-107	postive
AFP	SELENOOLP	0.3377778	8.27E-15	postive
AFP	SLC12A9-AS1	0.481622657	2.13E-30	postive
AFP	SLC5A4-AS1	0.319017638	2.72E-13	postive
AFP	SNHG14	0.408320944	1.64E-21	postive
AFP	SNHG20	0.347898656	1.13E-15	postive
AFP	SNHG21	0.37436516	4.43E-18	postive
AFP	SNHG4	0.310981618	1.13E-12	postive
AFP	TAAR3P	0.414749017	3.30E-22	postive
AFP	THRB-AS1	0.588547025	6.26E-48	postive
AFP	TMC3-AS1	0.367399516	2.00E-17	postive
AFP	TPM3P9	0.461226703	1.05E-27	postive
AFP	U91324.1	0.610250673	2.44E-52	postive
AFP	UPK1A-AS1	0.942048583	2.28E-238	postive
AFP	UXT-AS1	0.431097808	4.80E-24	postive
AFP	VPS9D1-AS1	0.372739214	6.32E-18	postive
AFP	WHAMMP3	0.31686242	4.01E-13	postive
AFP	Z83851.4	0.437858118	7.78E-25	postive
AFP	Z97056.1	0.310711955	1.19E-12	postive
AFP	ZBTB46-AS1	0.365543253	2.98E-17	postive
AFP	ZFHX4-AS1	0.522343594	2.39E-36	postive
ANKRD9	AC000068.2	0.311956051	9.54E-13	postive
ANKRD9	AC002398.1	0.301190995	6.06E-12	postive
ANKRD9	AC002467.1	0.427657558	1.19E-23	postive
ANKRD9	AC003102.1	0.364468227	3.74E-17	postive
ANKRD9	AC004447.2	0.305721164	2.81E-12	postive
ANKRD9	AC004596.1	0.341351329	4.13E-15	postive
ANKRD9	AC004801.6	0.342770013	3.13E-15	postive
ANKRD9	AC004803.1	0.308800582	1.65E-12	postive
ANKRD9	AC004812.2	0.301771965	5.49E-12	postive
ANKRD9	AC004893.11	0.340931699	4.49E-15	postive
ANKRD9	AC004918.1	0.327170524	6.14E-14	postive
ANKRD9	AC005034.4	0.406452811	2.59E-21	postive
ANKRD9	AC005034.6	0.300943894	6.31E-12	postive
ANKRD9	AC005229.4	0.338031603	7.88E-15	postive
ANKRD9	AC005237.1	0.430734937	5.28E-24	postive
ANKRD9	AC005261.2	0.336230239	1.11E-14	postive
ANKRD9	AC005261.4	0.379409699	1.45E-18	postive
ANKRD9	AC005520.4	0.353006505	4.05E-16	postive
ANKRD9	AC005696.1	0.399659568	1.34E-20	postive
ANKRD9	AC006001.2	0.321416958	1.77E-13	postive
ANKRD9	AC006126.4	0.315787414	4.85E-13	postive

ANKRD9	AC006238.1	0.331111112	2.94E-14	postive
ANKRD9	AC006252.1	0.303627724	4.01E-12	postive
ANKRD9	AC006449.2	0.329279354	4.15E-14	postive
ANKRD9	AC006449.7	0.304956957	3.20E-12	postive
ANKRD9	AC006547.1	0.304685725	3.35E-12	postive
ANKRD9	AC006942.1	0.301523722	5.73E-12	postive
ANKRD9	AC007114.1	0.301094146	6.15E-12	postive
ANKRD9	AC007182.1	0.320122849	2.23E-13	postive
ANKRD9	AC007228.9	0.508305064	3.32E-34	postive
ANKRD9	AC007383.2	0.527043665	4.35E-37	postive
ANKRD9	AC007406.3	0.328280231	5.00E-14	postive
ANKRD9	AC008280.3	0.308892685	1.63E-12	postive
ANKRD9	AC008438.1	0.382438133	7.34E-19	postive
ANKRD9	AC008443.1	0.408543573	1.55E-21	postive
ANKRD9	AC008610.1	0.367700394	1.88E-17	postive
ANKRD9	AC008622.2	0.401592289	8.43E-21	postive
ANKRD9	AC008735.4	0.312870669	8.13E-13	postive
ANKRD9	AC008750.4	0.306339644	2.53E-12	postive
ANKRD9	AC008915.3	0.340841356	4.57E-15	postive
ANKRD9	AC009065.3	0.421797025	5.48E-23	postive
ANKRD9	AC009065.5	0.327506664	5.77E-14	postive
ANKRD9	AC009065.6	0.421113139	6.53E-23	postive
ANKRD9	AC009065.9	0.308954617	1.61E-12	postive
ANKRD9	AC009118.3	0.313657135	7.08E-13	postive
ANKRD9	AC009779.3	0.397280749	2.36E-20	postive
ANKRD9	AC009955.4	0.301867646	5.40E-12	postive
ANKRD9	AC010136.1	0.316439868	4.32E-13	postive
ANKRD9	AC010273.3	0.391100199	1.01E-19	postive
ANKRD9	AC010307.2	0.375303167	3.60E-18	postive
ANKRD9	AC010307.4	0.410489361	9.57E-22	postive
ANKRD9	AC010326.4	0.325494727	8.38E-14	postive
ANKRD9	AC010326.5	0.32365854	1.17E-13	postive
ANKRD9	AC010503.5	0.420172656	8.32E-23	postive
ANKRD9	AC010809.1	0.305983315	2.69E-12	postive
ANKRD9	AC010969.2	0.323777499	1.15E-13	postive
ANKRD9	AC010997.4	0.309634678	1.43E-12	postive
ANKRD9	AC011472.2	0.381977634	8.15E-19	postive
ANKRD9	AC011978.2	0.349800919	7.74E-16	postive
ANKRD9	AC012063.1	0.357211968	1.71E-16	postive
ANKRD9	AC012306.2	0.388387224	1.89E-19	postive
ANKRD9	AC012313.1	0.390264673	1.23E-19	postive
ANKRD9	AC012313.9	0.333958699	1.72E-14	postive
ANKRD9	AC012442.2	0.302301984	5.02E-12	postive
ANKRD9	AC012510.1	0.322552452	1.44E-13	postive
ANKRD9	AC012603.1	0.341102373	4.34E-15	postive
ANKRD9	AC015726.1	0.34555442	1.81E-15	postive
ANKRD9	AC015818.3	0.301018254	6.23E-12	postive
ANKRD9	AC015922.2	0.414998568	3.10E-22	postive
ANKRD9	AC015922.3	0.442867787	1.97E-25	postive
ANKRD9	AC016065.1	0.309171764	1.55E-12	postive

ANKRD9	AC016727.1	0.376611768	2.70E-18	postive
ANKRD9	AC016876.2	0.316037587	4.64E-13	postive
ANKRD9	AC016876.3	0.332904132	2.10E-14	postive
ANKRD9	AC018413.1	0.447652724	5.19E-26	postive
ANKRD9	AC018647.2	0.325730039	8.02E-14	postive
ANKRD9	AC018809.2	0.307306158	2.14E-12	postive
ANKRD9	AC020663.2	0.358744016	1.24E-16	postive
ANKRD9	AC020915.3	0.351621304	5.36E-16	postive
ANKRD9	AC020978.4	0.344324999	2.31E-15	postive
ANKRD9	AC022007.1	0.338152903	7.69E-15	postive
ANKRD9	AC022306.2	0.313481454	7.30E-13	postive
ANKRD9	AC023024.1	0.317129714	3.82E-13	postive
ANKRD9	AC023421.1	0.385883067	3.36E-19	postive
ANKRD9	AC023509.3	0.419239716	1.06E-22	postive
ANKRD9	AC024267.7	0.373712284	5.11E-18	postive
ANKRD9	AC026369.2	0.334436959	1.57E-14	postive
ANKRD9	AC026471.1	0.34914952	8.83E-16	postive
ANKRD9	AC027097.1	0.310382078	1.26E-12	postive
ANKRD9	AC027288.1	0.315508937	5.10E-13	postive
ANKRD9	AC027307.2	0.338243322	7.56E-15	postive
ANKRD9	AC027575.3	0.39070791	1.11E-19	postive
ANKRD9	AC027601.1	0.362823895	5.30E-17	postive
ANKRD9	AC027644.3	0.375274764	3.62E-18	postive
ANKRD9	AC027763.2	0.357992872	1.45E-16	postive
ANKRD9	AC036176.1	0.354369415	3.06E-16	postive
ANKRD9	AC040173.2	0.319845354	2.35E-13	postive
ANKRD9	AC046134.2	0.303649733	4.00E-12	postive
ANKRD9	AC064802.1	0.331719652	2.62E-14	postive
ANKRD9	AC064807.4	0.311122317	1.10E-12	postive
ANKRD9	AC067750.1	0.363435006	4.66E-17	postive
ANKRD9	AC067852.2	0.442902228	1.95E-25	postive
ANKRD9	AC068338.2	0.379797381	1.33E-18	postive
ANKRD9	AC068473.5	0.359557736	1.05E-16	postive
ANKRD9	AC068888.1	0.399834053	1.29E-20	postive
ANKRD9	AC073073.2	0.30384237	3.87E-12	postive
ANKRD9	AC073575.4	0.303215629	4.30E-12	postive
ANKRD9	AC073896.4	0.318630534	2.92E-13	postive
ANKRD9	AC078846.1	0.319423044	2.53E-13	postive
ANKRD9	AC078909.1	0.316220647	4.49E-13	postive
ANKRD9	AC079834.2	0.334132494	1.66E-14	postive
ANKRD9	AC080112.2	0.358311876	1.36E-16	postive
ANKRD9	AC087269.1	0.303175441	4.33E-12	postive
ANKRD9	AC087741.2	0.388765592	1.73E-19	postive
ANKRD9	AC090114.2	0.327264169	6.04E-14	postive
ANKRD9	AC090587.1	0.315046771	5.53E-13	postive
ANKRD9	AC090617.5	0.349310374	8.55E-16	postive
ANKRD9	AC090772.3	0.380911371	1.04E-18	postive
ANKRD9	AC092115.4	0.334907042	1.43E-14	postive
ANKRD9	AC092123.1	0.307092318	2.22E-12	postive
ANKRD9	AC092171.2	0.409740705	1.15E-21	postive

ANKRD9	AC092368.3	0.305305598	3.02E-12	postive
ANKRD9	AC093157.1	0.317080021	3.85E-13	postive
ANKRD9	AC093248.1	0.333242716	1.97E-14	postive
ANKRD9	AC093249.6	0.338398754	7.34E-15	postive
ANKRD9	AC093523.2	0.32757348	5.70E-14	postive
ANKRD9	AC093585.1	0.33733034	9.01E-15	postive
ANKRD9	AC093620.1	0.300284233	7.05E-12	postive
ANKRD9	AC093627.4	0.310317601	1.27E-12	postive
ANKRD9	AC093627.5	0.338220392	7.59E-15	postive
ANKRD9	AC093797.1	0.319920274	2.31E-13	postive
ANKRD9	AC096677.1	0.438447935	6.63E-25	postive
ANKRD9	AC096734.2	0.329572806	3.93E-14	postive
ANKRD9	AC096772.6	0.467853122	1.46E-28	postive
ANKRD9	AC097468.3	0.421483071	5.94E-23	postive
ANKRD9	AC098820.2	0.353598939	3.59E-16	postive
ANKRD9	AC098820.4	0.368510373	1.58E-17	postive
ANKRD9	AC099778.1	0.316320918	4.41E-13	postive
ANKRD9	AC100810.1	0.418033424	1.44E-22	postive
ANKRD9	AC103764.1	0.3175345	3.55E-13	postive
ANKRD9	AC104113.1	0.328603575	4.71E-14	postive
ANKRD9	AC104411.2	0.311587847	1.02E-12	postive
ANKRD9	AC104667.2	0.320660266	2.03E-13	postive
ANKRD9	AC104825.1	0.331047748	2.98E-14	postive
ANKRD9	AC105398.2	0.337912964	8.06E-15	postive
ANKRD9	AC105399.1	0.3055669	2.88E-12	postive
ANKRD9	AC106886.3	0.30775552	1.98E-12	postive
ANKRD9	AC107214.2	0.333455128	1.89E-14	postive
ANKRD9	AC107375.1	0.324689802	9.71E-14	postive
ANKRD9	AC107982.3	0.34473573	2.13E-15	postive
ANKRD9	AC108860.2	0.332042278	2.47E-14	postive
ANKRD9	AC109322.1	0.311916764	9.61E-13	postive
ANKRD9	AC112220.2	0.363233599	4.86E-17	postive
ANKRD9	AC113133.1	0.350434432	6.82E-16	postive
ANKRD9	AC114271.1	0.30743187	2.09E-12	postive
ANKRD9	AC114341.1	0.459975626	1.51E-27	postive
ANKRD9	AC114810.1	0.390663875	1.12E-19	postive
ANKRD9	AC120049.1	0.313251328	7.60E-13	postive
ANKRD9	AC120053.1	0.3450905	1.98E-15	postive
ANKRD9	AC121757.1	0.31313957	7.75E-13	postive
ANKRD9	AC123768.2	0.30984757	1.38E-12	postive
ANKRD9	AC124242.1	0.363210425	4.88E-17	postive
ANKRD9	AC125257.1	0.354319548	3.10E-16	postive
ANKRD9	AC127537.1	0.36000764	9.56E-17	postive
ANKRD9	AC129492.2	0.376021148	3.07E-18	postive
ANKRD9	AC129507.2	0.332742249	2.16E-14	postive
ANKRD9	AC129507.3	0.394384426	4.69E-20	postive
ANKRD9	AC132008.2	0.309492922	1.47E-12	postive
ANKRD9	AC133528.2	0.421356122	6.14E-23	postive
ANKRD9	AC134043.2	0.489428347	1.77E-31	postive
ANKRD9	AC134878.2	0.358371228	1.34E-16	postive

ANKRD9	AC135050.1	0.323616825	1.18E-13	postive
ANKRD9	AC135050.5	0.303208141	4.31E-12	postive
ANKRD9	AC135050.6	0.415254928	2.91E-22	postive
ANKRD9	AC137932.1	0.50141808	3.43E-33	postive
ANKRD9	AC137932.2	0.429082591	8.18E-24	postive
ANKRD9	AC137932.3	0.322956858	1.33E-13	postive
ANKRD9	AC138696.2	0.327638704	5.63E-14	postive
ANKRD9	AC139149.1	0.377856339	2.05E-18	postive
ANKRD9	AC139768.1	0.347346464	1.27E-15	postive
ANKRD9	AC139887.1	0.308041753	1.88E-12	postive
ANKRD9	AC139887.2	0.383788427	5.41E-19	postive
ANKRD9	AC142472.1	0.365283614	3.15E-17	postive
ANKRD9	AC145124.1	0.304234952	3.62E-12	postive
ANKRD9	AC147651.1	0.352410707	4.57E-16	postive
ANKRD9	AC211433.2	0.320469357	2.10E-13	postive
ANKRD9	AC231981.1	0.31060125	1.21E-12	postive
ANKRD9	AC244090.3	0.34086806	4.54E-15	postive
ANKRD9	AC245140.2	0.329785488	3.77E-14	postive
ANKRD9	ACVR2B-AS1	0.351821256	5.15E-16	postive
ANKRD9	ADGRD1-AS1	0.333290643	1.95E-14	postive
ANKRD9	ADIRF-AS1	0.317793171	3.39E-13	postive
ANKRD9	AF131216.4	0.301170794	6.08E-12	postive
ANKRD9	AFG3L1P	0.357276629	1.69E-16	postive
ANKRD9	AL020996.1	0.336391907	1.08E-14	postive
ANKRD9	AL021392.1	0.383613083	5.63E-19	postive
ANKRD9	AL022328.1	0.333804428	1.77E-14	postive
ANKRD9	AL022328.2	0.311700642	9.98E-13	postive
ANKRD9	AL023284.4	0.306627441	2.40E-12	postive
ANKRD9	AL031673.1	0.304589081	3.41E-12	postive
ANKRD9	AL031775.1	0.387908393	2.11E-19	postive
ANKRD9	AL031847.1	0.367137623	2.12E-17	postive
ANKRD9	AL031963.3	0.302297306	5.03E-12	postive
ANKRD9	AL033397.1	0.347353463	1.26E-15	postive
ANKRD9	AL035587.1	0.303720709	3.95E-12	postive
ANKRD9	AL035661.1	0.351374361	5.64E-16	postive
ANKRD9	AL049796.1	0.30752508	2.06E-12	postive
ANKRD9	AL049840.4	0.354513083	2.98E-16	postive
ANKRD9	AL049840.5	0.354296611	3.11E-16	postive
ANKRD9	AL049840.6	0.415796903	2.53E-22	postive
ANKRD9	AL050309.1	0.304553093	3.43E-12	postive
ANKRD9	AL096828.3	0.30385441	3.86E-12	postive
ANKRD9	AL117209.1	0.412550084	5.73E-22	postive
ANKRD9	AL117332.1	0.333490972	1.88E-14	postive
ANKRD9	AL117336.2	0.304714478	3.33E-12	postive
ANKRD9	AL118506.1	0.331632281	2.67E-14	postive
ANKRD9	AL118508.1	0.571325551	1.16E-44	postive
ANKRD9	AL118558.3	0.425855471	1.91E-23	postive
ANKRD9	AL118558.4	0.348567427	9.92E-16	postive
ANKRD9	AL121603.2	0.300171754	7.18E-12	postive
ANKRD9	AL121839.2	0.370390673	1.05E-17	postive

ANKRD9	AL121906.2	0.365711824	2.87E-17	postive
ANKRD9	AL121944.2	0.351562203	5.43E-16	postive
ANKRD9	AL122010.1	0.423254457	3.76E-23	postive
ANKRD9	AL122023.3	0.441691864	2.72E-25	postive
ANKRD9	AL132712.1	0.406958612	2.29E-21	postive
ANKRD9	AL132857.1	0.304951875	3.20E-12	postive
ANKRD9	AL133153.2	0.343257821	2.85E-15	postive
ANKRD9	AL133355.1	0.371395818	8.46E-18	postive
ANKRD9	AL133367.1	0.330980259	3.02E-14	postive
ANKRD9	AL133520.1	0.326661775	6.75E-14	postive
ANKRD9	AL135925.1	0.374003172	4.79E-18	postive
ANKRD9	AL136040.1	0.315586783	5.03E-13	postive
ANKRD9	AL136295.7	0.303447257	4.14E-12	postive
ANKRD9	AL137127.1	0.390237421	1.23E-19	postive
ANKRD9	AL138756.1	0.395996319	3.21E-20	postive
ANKRD9	AL139011.1	0.347243437	1.29E-15	postive
ANKRD9	AL139246.5	0.344885668	2.06E-15	postive
ANKRD9	AL139289.1	0.335581387	1.26E-14	postive
ANKRD9	AL139384.1	0.327487654	5.79E-14	postive
ANKRD9	AL158071.3	0.374584674	4.22E-18	postive
ANKRD9	AL158212.3	0.331918304	2.53E-14	postive
ANKRD9	AL160006.1	0.360532578	8.57E-17	postive
ANKRD9	AL160313.2	0.367197614	2.09E-17	postive
ANKRD9	AL161421.1	0.349489151	8.25E-16	postive
ANKRD9	AL161452.1	0.321025547	1.90E-13	postive
ANKRD9	AL161729.3	0.308560493	1.72E-12	postive
ANKRD9	AL161772.1	0.343538898	2.69E-15	postive
ANKRD9	AL162258.2	0.322667338	1.41E-13	postive
ANKRD9	AL162274.2	0.311293489	1.07E-12	postive
ANKRD9	AL162377.1	0.331358185	2.81E-14	postive
ANKRD9	AL162497.1	0.311096511	1.11E-12	postive
ANKRD9	AL162595.1	0.309123159	1.56E-12	postive
ANKRD9	AL353743.1	0.377336181	2.30E-18	postive
ANKRD9	AL353997.2	0.347798868	1.16E-15	postive
ANKRD9	AL354718.3	0.359667064	1.03E-16	postive
ANKRD9	AL354892.2	0.308556434	1.72E-12	postive
ANKRD9	AL354920.1	0.309225709	1.54E-12	postive
ANKRD9	AL355001.2	0.310916127	1.14E-12	postive
ANKRD9	AL355574.1	0.333486346	1.88E-14	postive
ANKRD9	AL355802.3	0.385205787	3.92E-19	postive
ANKRD9	AL356488.3	0.361220566	7.42E-17	postive
ANKRD9	AL358472.3	0.356139093	2.13E-16	postive
ANKRD9	AL358852.1	0.309126553	1.56E-12	postive
ANKRD9	AL359921.2	0.323018504	1.32E-13	postive
ANKRD9	AL365181.2	0.372857591	6.16E-18	postive
ANKRD9	AL365181.3	0.378535369	1.76E-18	postive
ANKRD9	AL365330.1	0.337154112	9.33E-15	postive
ANKRD9	AL390198.1	0.31663184	4.18E-13	postive
ANKRD9	AL391121.1	0.452454453	1.33E-26	postive
ANKRD9	AL391427.1	0.399390595	1.43E-20	postive

ANKRD9	AL391684.1	0.309054159	1.58E-12	postive
ANKRD9	AL391834.2	0.322403815	1.48E-13	postive
ANKRD9	AL392172.1	0.317593995	3.52E-13	postive
ANKRD9	AL441992.2	0.345653022	1.77E-15	postive
ANKRD9	AL442224.1	0.322844199	1.36E-13	postive
ANKRD9	AL445645.1	0.326325749	7.18E-14	postive
ANKRD9	AL450998.2	0.307756721	1.98E-12	postive
ANKRD9	AL451050.2	0.312232273	9.09E-13	postive
ANKRD9	AL451069.2	0.398919768	1.60E-20	postive
ANKRD9	AL451085.2	0.347676072	1.19E-15	postive
ANKRD9	AL451085.3	0.484823239	7.74E-31	postive
ANKRD9	AL451165.2	0.393310438	6.03E-20	postive
ANKRD9	AL512622.1	0.318340042	3.08E-13	postive
ANKRD9	AL513165.1	0.313814952	6.88E-13	postive
ANKRD9	AL583722.3	0.372436857	6.75E-18	postive
ANKRD9	AL583810.1	0.339515918	5.91E-15	postive
ANKRD9	AL589642.2	0.364546729	3.68E-17	postive
ANKRD9	AL589765.6	0.302224725	5.09E-12	postive
ANKRD9	AL590617.2	0.337877483	8.11E-15	postive
ANKRD9	AL590666.2	0.364315976	3.86E-17	postive
ANKRD9	AL592071.1	0.344700278	2.14E-15	postive
ANKRD9	AL592211.1	0.335220718	1.35E-14	postive
ANKRD9	AL604028.1	0.306203329	2.59E-12	postive
ANKRD9	AL645941.1	0.302609865	4.77E-12	postive
ANKRD9	AL671710.1	0.331128594	2.93E-14	postive
ANKRD9	AL731533.2	0.352100319	4.87E-16	postive
ANKRD9	AL731569.1	0.304684824	3.35E-12	postive
ANKRD9	AL731577.2	0.348009966	1.11E-15	postive
ANKRD9	AL807752.5	0.395734351	3.41E-20	postive
ANKRD9	AL928654.1	0.348236432	1.06E-15	postive
ANKRD9	AL928654.2	0.302153092	5.15E-12	postive
ANKRD9	ALOX12-AS1	0.323415093	1.23E-13	postive
ANKRD9	ALOX12P2	0.359213093	1.13E-16	postive
ANKRD9	AP000254.2	0.377363505	2.28E-18	postive
ANKRD9	AP000470.1	0.397556544	2.21E-20	postive
ANKRD9	AP000688.1	0.494013748	4.00E-32	postive
ANKRD9	AP001062.1	0.363201688	4.89E-17	postive
ANKRD9	AP001107.1	0.309590586	1.44E-12	postive
ANKRD9	AP001160.2	0.306929862	2.28E-12	postive
ANKRD9	AP001160.3	0.423912509	3.17E-23	postive
ANKRD9	AP001318.2	0.300449068	6.86E-12	postive
ANKRD9	AP001453.4	0.357763932	1.52E-16	postive
ANKRD9	AP001542.3	0.308150157	1.85E-12	postive
ANKRD9	AP001972.1	0.444788207	1.16E-25	postive
ANKRD9	AP002360.2	0.323130011	1.29E-13	postive
ANKRD9	AP002498.1	0.397342719	2.33E-20	postive
ANKRD9	AP002748.4	0.38846907	1.86E-19	postive
ANKRD9	AP002761.4	0.32244661	1.46E-13	postive
ANKRD9	AP002807.1	0.348213183	1.07E-15	postive
ANKRD9	AP003068.2	0.337456579	8.80E-15	postive

ANKRD9	AP003119.1	0.308077522	1.87E-12	postive
ANKRD9	AP003486.1	0.325908646	7.76E-14	postive
ANKRD9	AP003559.1	0.324089366	1.08E-13	postive
ANKRD9	AP006621.3	0.345979242	1.66E-15	postive
ANKRD9	ARMCX5-GPRASP2	0.315515483	5.09E-13	postive
ANKRD9	ARRDC1-AS1	0.482915567	1.42E-30	postive
ANKRD9	ASB16-AS1	0.372519046	6.63E-18	postive
ANKRD9	ASH1L-AS1	0.326676716	6.73E-14	postive
ANKRD9	ATP2C2-AS1	0.30181771	5.45E-12	postive
ANKRD9	ATP8B5P	0.301259462	5.99E-12	postive
ANKRD9	ATXN2-AS	0.315028731	5.55E-13	postive
ANKRD9	BACE1-AS	0.374467642	4.33E-18	postive
ANKRD9	BAIAP2-DT	0.380614777	1.11E-18	postive
ANKRD9	BARX1-DT	0.344987437	2.02E-15	postive
ANKRD9	BTNL10	0.341870603	3.74E-15	postive
ANKRD9	BX255925.1	0.321755563	1.66E-13	postive
ANKRD9	BX284668.5	0.364045101	4.09E-17	postive
ANKRD9	BX322557.10	0.383172471	6.22E-19	postive
ANKRD9	BX324167.1	0.30511151	3.12E-12	postive
ANKRD9	BX537318.1	0.354422012	3.03E-16	postive
ANKRD9	BX539320.1	0.464377548	4.14E-28	postive
ANKRD9	BX649601.1	0.301502749	5.75E-12	postive
ANKRD9	BX649632.1	0.425290292	2.21E-23	postive
ANKRD9	C15orf56	0.360311753	8.97E-17	postive
ANKRD9	C18orf65	0.351930213	5.04E-16	postive
ANKRD9	C1orf229	0.341537834	3.99E-15	postive
ANKRD9	C22orf46	0.315276917	5.31E-13	postive
ANKRD9	C2CD4D-AS1	0.427774021	1.16E-23	postive
ANKRD9	C5orf66	0.33159731	2.69E-14	postive
ANKRD9	C9orf163	0.376314172	2.88E-18	postive
ANKRD9	CA5BP1	0.319426037	2.53E-13	postive
ANKRD9	CATIP-AS1	0.349770993	7.79E-16	postive
ANKRD9	CATSPER2P1	0.366286208	2.54E-17	postive
ANKRD9	CCDC183-AS1	0.319324116	2.58E-13	postive
ANKRD9	CCNT2-AS1	0.344682364	2.15E-15	postive
ANKRD9	CD27-AS1	0.321460083	1.75E-13	postive
ANKRD9	CDC37L1-DT	0.323551117	1.20E-13	postive
ANKRD9	CENPBD1P1	0.407863359	1.83E-21	postive
ANKRD9	CHL1-AS2	0.325039077	9.11E-14	postive
ANKRD9	CIDECP1	0.367105867	2.13E-17	postive
ANKRD9	CKMT2-AS1	0.315097429	5.49E-13	postive
ANKRD9	CNNM3-DT	0.301654372	5.60E-12	postive
ANKRD9	COA6-AS1	0.345051664	2.00E-15	postive
ANKRD9	CRNDE	0.33771418	8.37E-15	postive
ANKRD9	CRYZL2P	0.38926822	1.54E-19	postive
ANKRD9	CTBP1-DT	0.45380581	9.05E-27	postive
ANKRD9	CTD-2132N18.4	0.320467331	2.10E-13	postive
ANKRD9	CTD-2134A5.3	0.344218967	2.36E-15	postive
ANKRD9	CUTALP	0.326504249	6.95E-14	postive
ANKRD9	DAAM2-AS1	0.305135833	3.10E-12	postive

ANKRD9	DANCR	0.384546295	4.56E-19	postive
ANKRD9	DARS-AS1	0.368629569	1.54E-17	postive
ANKRD9	DCST1-AS1	0.406156047	2.79E-21	postive
ANKRD9	DGCR11	0.321326004	1.79E-13	postive
ANKRD9	DHRS4-AS1	0.392381496	7.49E-20	postive
ANKRD9	DICER1-AS1	0.334576625	1.53E-14	postive
ANKRD9	DLG5-AS1	0.35442105	3.03E-16	postive
ANKRD9	DLGAP1-AS1	0.407073778	2.22E-21	postive
ANKRD9	DM1-AS	0.356233148	2.09E-16	postive
ANKRD9	DSCAM-AS1	0.437018169	9.78E-25	postive
ANKRD9	DSTNP2	0.330195838	3.50E-14	postive
ANKRD9	EBLN3P	0.407941874	1.80E-21	postive
ANKRD9	EDRF1-DT	0.336059984	1.15E-14	postive
ANKRD9	EIF3J-DT	0.370417377	1.05E-17	postive
ANKRD9	ELF3-AS1	0.395311635	3.77E-20	postive
ANKRD9	EMC1-AS1	0.305207876	3.07E-12	postive
ANKRD9	EP400P1	0.307967896	1.91E-12	postive
ANKRD9	FAHD2CP	0.308014583	1.89E-12	postive
ANKRD9	FAM157B	0.320690464	2.01E-13	postive
ANKRD9	FAM86B3P	0.334715133	1.49E-14	postive
ANKRD9	FAM86C2P	0.363335507	4.75E-17	postive
ANKRD9	FAM86EP	0.380930529	1.03E-18	postive
ANKRD9	FAM86JP	0.43173173	4.05E-24	postive
ANKRD9	FBXL19-AS1	0.32430031	1.04E-13	postive
ANKRD9	FGD5-AS1	0.379441403	1.44E-18	postive
ANKRD9	FOXD2-AS1	0.309925224	1.36E-12	postive
ANKRD9	FRG1HP	0.37754819	2.19E-18	postive
ANKRD9	GAS5-AS1	0.345308824	1.90E-15	postive
ANKRD9	GATA2-AS1	0.383555089	5.70E-19	postive
ANKRD9	GLIS2-AS1	0.326350576	7.15E-14	postive
ANKRD9	GMDS-DT	0.304875374	3.24E-12	postive
ANKRD9	GNRHR2	0.329646099	3.87E-14	postive
ANKRD9	GOLGA2P7	0.311272784	1.08E-12	postive
ANKRD9	GPRC5D-AS1	0.387089121	2.55E-19	postive
ANKRD9	GTF2IP1	0.379802613	1.33E-18	postive
ANKRD9	GUCY2EP	0.305072969	3.14E-12	postive
ANKRD9	H1FX-AS1	0.302782409	4.63E-12	postive
ANKRD9	H2BC20P	0.336744938	1.01E-14	postive
ANKRD9	HAR1A	0.329201261	4.21E-14	postive
ANKRD9	HCG18	0.348280644	1.05E-15	postive
ANKRD9	HCG25	0.305020747	3.17E-12	postive
ANKRD9	HEIH	0.348824068	9.42E-16	postive
ANKRD9	HMGB1P5	0.303013043	4.45E-12	postive
ANKRD9	HPN-AS1	0.344155609	2.38E-15	postive
ANKRD9	IL6R-AS1	0.365716764	2.87E-17	postive
ANKRD9	INTS6-AS1	0.305864052	2.74E-12	postive
ANKRD9	IQCH-AS1	0.300185667	7.17E-12	postive
ANKRD9	ITFG2-AS1	0.331488913	2.74E-14	postive
ANKRD9	JMJD1C-AS1	0.327546161	5.73E-14	postive
ANKRD9	KIAA1671-AS1	0.31592037	4.74E-13	postive

ANKRD9	KMT2E-AS1	0.303991184	3.77E-12	postive
ANKRD9	KRT7-AS	0.301908843	5.37E-12	postive
ANKRD9	LINC00160	0.396782466	2.66E-20	postive
ANKRD9	LINC00205	0.46394126	4.71E-28	postive
ANKRD9	LINC002481	0.411427303	7.58E-22	postive
ANKRD9	LINC00315	0.321951869	1.60E-13	postive
ANKRD9	LINC00467	0.404671765	4.00E-21	postive
ANKRD9	LINC00476	0.383713753	5.50E-19	postive
ANKRD9	LINC00637	0.351293473	5.73E-16	postive
ANKRD9	LINC00638	0.425357372	2.18E-23	postive
ANKRD9	LINC00680	0.301513689	5.74E-12	postive
ANKRD9	LINC00682	0.443038039	1.88E-25	postive
ANKRD9	LINC00863	0.563254828	3.39E-43	postive
ANKRD9	LINC00909	0.376822147	2.58E-18	postive
ANKRD9	LINC00933	0.357045404	1.77E-16	postive
ANKRD9	LINC00940	0.332923641	2.09E-14	postive
ANKRD9	LINC00963	0.451271929	1.87E-26	postive
ANKRD9	LINC01011	0.390515958	1.16E-19	postive
ANKRD9	LINC01128	0.333839361	1.76E-14	postive
ANKRD9	LINC01145	0.372640954	6.45E-18	postive
ANKRD9	LINC01220	0.37400338	4.79E-18	postive
ANKRD9	LINC01270	0.382085826	7.95E-19	postive
ANKRD9	LINC01271	0.36902946	1.41E-17	postive
ANKRD9	LINC01278	0.350194433	7.15E-16	postive
ANKRD9	LINC01342	0.358117314	1.42E-16	postive
ANKRD9	LINC01508	0.349602454	8.06E-16	postive
ANKRD9	LINC01564	0.338546341	7.13E-15	postive
ANKRD9	LINC01655	0.339386088	6.06E-15	postive
ANKRD9	LINC01719	0.371280086	8.68E-18	postive
ANKRD9	LINC01752	0.450527838	2.30E-26	postive
ANKRD9	LINC01770	0.345487967	1.83E-15	postive
ANKRD9	LINC01814	0.30196732	5.31E-12	postive
ANKRD9	LINC01881	0.32359429	1.19E-13	postive
ANKRD9	LINC01924	0.334744288	1.48E-14	postive
ANKRD9	LINC01932	0.448749662	3.81E-26	postive
ANKRD9	LINC01977	0.322183755	1.54E-13	postive
ANKRD9	LINC02012	0.330054302	3.59E-14	postive
ANKRD9	LINC02014	0.315190357	5.40E-13	postive
ANKRD9	LINC02026	0.303571727	4.05E-12	postive
ANKRD9	LINC02122	0.384263087	4.86E-19	postive
ANKRD9	LINC02198	0.36428888	3.89E-17	postive
ANKRD9	LINC02257	0.389237836	1.55E-19	postive
ANKRD9	LINC02280	0.374334689	4.46E-18	postive
ANKRD9	LINC02298	0.511561258	1.08E-34	postive
ANKRD9	LINC02437	0.502984903	2.03E-33	postive
ANKRD9	LINC02469	0.318388067	3.05E-13	postive
ANKRD9	LINC02482	0.308656918	1.69E-12	postive
ANKRD9	LINC02585	0.352238104	4.73E-16	postive
ANKRD9	LINC02749	0.374640268	4.17E-18	postive
ANKRD9	LINC02818	0.310568646	1.22E-12	postive

ANKRD9	MAN1B1-DT	0.406069799	2.84E-21	postive
ANKRD9	MAPKAPK5-AS1	0.399345288	1.45E-20	postive
ANKRD9	MCF2L-AS1	0.321937963	1.61E-13	postive
ANKRD9	MED4-AS1	0.322474494	1.46E-13	postive
ANKRD9	MIF-AS1	0.31080205	1.17E-12	postive
ANKRD9	MINCR	0.313404257	7.40E-13	postive
ANKRD9	MIR200CHG	0.404334045	4.34E-21	postive
ANKRD9	MIR22HG	0.425201962	2.27E-23	postive
ANKRD9	MIR3936HG	0.30198158	5.30E-12	postive
ANKRD9	MIR762HG	0.364281126	3.89E-17	postive
ANKRD9	MRPL20-AS1	0.425597959	2.04E-23	postive
ANKRD9	MYO16-AS1	0.517453272	1.37E-35	postive
ANKRD9	MZF1-AS1	0.324797858	9.52E-14	postive
ANKRD9	NACA4P	0.320588944	2.05E-13	postive
ANKRD9	NAPA-AS1	0.33550805	1.28E-14	postive
ANKRD9	NBR2	0.333473687	1.88E-14	postive
ANKRD9	NDUFB2-AS1	0.356163256	2.12E-16	postive
ANKRD9	NDUFV2-AS1	0.351914362	5.05E-16	postive
ANKRD9	NIFK-AS1	0.360489498	8.64E-17	postive
ANKRD9	NKX2-1-AS1	0.303195551	4.32E-12	postive
ANKRD9	NOP14-AS1	0.368998415	1.42E-17	postive
ANKRD9	NORAD	0.370079223	1.13E-17	postive
ANKRD9	NRAV	0.360672695	8.32E-17	postive
ANKRD9	NRSN2-AS1	0.530269858	1.33E-37	postive
ANKRD9	NUDT16P1	0.384191361	4.94E-19	postive
ANKRD9	NUTM2A-AS1	0.453049085	1.12E-26	postive
ANKRD9	NUTM2B-AS1	0.306518491	2.45E-12	postive
ANKRD9	OIP5-AS1	0.504162392	1.36E-33	postive
ANKRD9	OLMALINC	0.361915649	6.41E-17	postive
ANKRD9	OTUD6B-AS1	0.350538033	6.68E-16	postive
ANKRD9	PAXIP1-AS1	0.362511962	5.66E-17	postive
ANKRD9	PCAT6	0.368978818	1.43E-17	postive
ANKRD9	PDIA3P1	0.444087076	1.40E-25	postive
ANKRD9	PGAM1P8	0.306677955	2.38E-12	postive
ANKRD9	PIPSL	0.322044228	1.58E-13	postive
ANKRD9	PITPNA-AS1	0.423108374	3.90E-23	postive
ANKRD9	PMS2CL	0.331901317	2.54E-14	postive
ANKRD9	POLR2J4	0.317695889	3.45E-13	postive
ANKRD9	PPP1R26-AS1	0.376346934	2.86E-18	postive
ANKRD9	PPP3CB-AS1	0.337443067	8.82E-15	postive
ANKRD9	PPP4R1-AS1	0.321446263	1.76E-13	postive
ANKRD9	PRKAG2-AS1	0.335844606	1.20E-14	postive
ANKRD9	PRKCZ-AS1	0.358413746	1.33E-16	postive
ANKRD9	PRR34-AS1	0.31760383	3.51E-13	postive
ANKRD9	PRRT3-AS1	0.31332332	7.50E-13	postive
ANKRD9	PSMA3-AS1	0.307728202	1.99E-12	postive
ANKRD9	PSMG3-AS1	0.317061758	3.87E-13	postive
ANKRD9	PTOV1-AS1	0.417738603	1.55E-22	postive
ANKRD9	PXN-AS1	0.347880325	1.14E-15	postive
ANKRD9	RAB11B-AS1	0.371861276	7.65E-18	postive

ANKRD9	RALY-AS1	0.320592517	2.05E-13	postive
ANKRD9	RARA-AS1	0.470076819	7.49E-29	postive
ANKRD9	RASA4CP	0.417488814	1.65E-22	postive
ANKRD9	RASSF10-DT	0.300172926	7.18E-12	postive
ANKRD9	RBM15-AS1	0.42741951	1.27E-23	postive
ANKRD9	RBPMS-AS1	0.310292542	1.28E-12	postive
ANKRD9	RNF216P1	0.318536471	2.97E-13	postive
ANKRD9	RNU6ATAC35P	0.335859114	1.20E-14	postive
ANKRD9	RP11-101E13.5	0.419098945	1.09E-22	postive
ANKRD9	RP11-104N10.2	0.300587609	6.70E-12	postive
ANKRD9	RP11-10C24.1	0.310290286	1.28E-12	postive
ANKRD9	RP11-10C24.2	0.327711655	5.56E-14	postive
ANKRD9	RP11-175O19.4	0.346509688	1.50E-15	postive
ANKRD9	RP11-226L15.5	0.361240247	7.39E-17	postive
ANKRD9	RP1-122P22.2	0.340032583	5.35E-15	postive
ANKRD9	RP11-2B6.3	0.313044912	7.88E-13	postive
ANKRD9	RP11-429J17.8	0.310388486	1.25E-12	postive
ANKRD9	RP11-435O5.2	0.386412808	2.98E-19	postive
ANKRD9	RP11-465N4.5	0.393456879	5.83E-20	postive
ANKRD9	RP11-529E10.6	0.353885715	3.38E-16	postive
ANKRD9	RP11-539I5.1	0.332175983	2.41E-14	postive
ANKRD9	RP11-672A2.1	0.313458011	7.33E-13	postive
ANKRD9	RP4-545L17.11	0.322111728	1.56E-13	postive
ANKRD9	RP4-737E23.7	0.373697917	5.12E-18	postive
ANKRD9	RPARP-AS1	0.429676951	6.99E-24	postive
ANKRD9	RPL13P5	0.317131157	3.82E-13	postive
ANKRD9	RPS10P7	0.395401245	3.69E-20	postive
ANKRD9	RUSC1-AS1	0.310009343	1.34E-12	postive
ANKRD9	SCAMP1-AS1	0.309217262	1.54E-12	postive
ANKRD9	SCAND2P	0.301388314	5.86E-12	postive
ANKRD9	SCAT8	0.334679987	1.50E-14	postive
ANKRD9	SDCBP2-AS1	0.335822229	1.20E-14	postive
ANKRD9	SDHAP1	0.321097172	1.87E-13	postive
ANKRD9	SEMA3B-AS1	0.313471577	7.31E-13	postive
ANKRD9	SERHL	0.331843643	2.56E-14	postive
ANKRD9	SGMS1-AS1	0.425379773	2.16E-23	postive
ANKRD9	SLC25A25-AS1	0.372922539	6.07E-18	postive
ANKRD9	SLC44A3-AS1	0.302947237	4.50E-12	postive
ANKRD9	SMG7-AS1	0.350030455	7.39E-16	postive
ANKRD9	SNAI3-AS1	0.314829714	5.75E-13	postive
ANKRD9	SNHG11	0.41125549	7.91E-22	postive
ANKRD9	SNHG17	0.319682509	2.42E-13	postive
ANKRD9	SNHG28	0.311915455	9.61E-13	postive
ANKRD9	SNHG32	0.403169387	5.76E-21	postive
ANKRD9	SNHG7	0.426031599	1.82E-23	postive
ANKRD9	SNHG8	0.429034003	8.29E-24	postive
ANKRD9	SPINT1-AS1	0.391013237	1.03E-19	postive
ANKRD9	SREBF2-AS1	0.331801188	2.58E-14	postive
ANKRD9	SRP14-AS1	0.42859467	9.31E-24	postive
ANKRD9	ST20-AS1	0.333878793	1.74E-14	postive

ANKRD9	STARD7-AS1	0.438810925	6.00E-25	postive
ANKRD9	STPG3-AS1	0.302010771	5.27E-12	postive
ANKRD9	STX17-AS1	0.44761922	5.24E-26	postive
ANKRD9	SUCLA2-AS1	0.379033027	1.58E-18	postive
ANKRD9	SUCLG2-AS1	0.386694741	2.79E-19	postive
ANKRD9	SVIL-AS1	0.340002257	5.38E-15	postive
ANKRD9	TAF1A-AS1	0.365448531	3.04E-17	postive
ANKRD9	TAPT1-AS1	0.365789255	2.83E-17	postive
ANKRD9	TDRKH-AS1	0.332444959	2.29E-14	postive
ANKRD9	THAP9-AS1	0.322833364	1.36E-13	postive
ANKRD9	TMEM161B-AS1	0.32177505	1.65E-13	postive
ANKRD9	TMEM198B	0.347828641	1.15E-15	postive
ANKRD9	TMEM202-AS1	0.36056478	8.51E-17	postive
ANKRD9	TMEM44-AS1	0.336644868	1.03E-14	postive
ANKRD9	TMEM92-AS1	0.338778039	6.82E-15	postive
ANKRD9	TOB1-AS1	0.310539902	1.22E-12	postive
ANKRD9	TP73-AS1	0.326681568	6.73E-14	postive
ANKRD9	TRAM2-AS1	0.419697498	9.40E-23	postive
ANKRD9	TRIM52-AS1	0.358795631	1.23E-16	postive
ANKRD9	TSTD3	0.338052154	7.84E-15	postive
ANKRD9	TTC39A-AS1	0.348382938	1.03E-15	postive
ANKRD9	TUBBP5	0.31061329	1.21E-12	postive
ANKRD9	TUG1	0.396429716	2.89E-20	postive
ANKRD9	U47924.2	0.351410537	5.60E-16	postive
ANKRD9	U62317.3	0.338084555	7.80E-15	postive
ANKRD9	U91328.1	0.374977004	3.87E-18	postive
ANKRD9	UBE2D3-AS1	0.338607811	7.05E-15	postive
ANKRD9	UBR5-AS1	0.367989089	1.77E-17	postive
ANKRD9	UGDH-AS1	0.308019177	1.89E-12	postive
ANKRD9	UPF3AP2	0.347911178	1.13E-15	postive
ANKRD9	URAHP	0.325586553	8.23E-14	postive
ANKRD9	USP46-AS1	0.351749497	5.22E-16	postive
ANKRD9	VASH1-AS1	0.34782923	1.15E-15	postive
ANKRD9	VPS13B-DT	0.360254097	9.08E-17	postive
ANKRD9	WAC-AS1	0.540491962	2.86E-39	postive
ANKRD9	WASH3P	0.365624949	2.93E-17	postive
ANKRD9	Z92544.2	0.316450083	4.31E-13	postive
ANKRD9	Z97634.1	0.370542193	1.02E-17	postive
ANKRD9	Z97653.1	0.337848178	8.16E-15	postive
ANKRD9	ZBTB11-AS1	0.377047813	2.45E-18	postive
ANKRD9	ZNF205-AS1	0.349687356	7.92E-16	postive
ANKRD9	ZNF213-AS1	0.3840242	5.13E-19	postive
ANKRD9	ZNF252P	0.37821562	1.89E-18	postive
ANKRD9	ZNF337-AS1	0.380117643	1.24E-18	postive
ANKRD9	ZNF503-AS2	0.323234881	1.27E-13	postive
ANKRD9	ZNF767P	0.301040742	6.21E-12	postive
ANKRD9	ZNRD2-AS1	0.328878774	4.47E-14	postive
ANKRD9	ZSCAN16-AS1	0.372174038	7.14E-18	postive
AOC2	AC002116.2	0.33012673	3.54E-14	postive
AOC2	AC002519.1	0.315441038	5.16E-13	postive

AOC2	AC003973.1	0.471495127	4.87E-29	postive
AOC2	AC004066.2	0.360601306	8.45E-17	postive
AOC2	AC004156.1	0.39783031	2.08E-20	postive
AOC2	AC004263.1	0.321364786	1.78E-13	postive
AOC2	AC004448.2	0.411600645	7.26E-22	postive
AOC2	AC004771.3	0.410359292	9.89E-22	postive
AOC2	AC004877.1	0.318962465	2.75E-13	postive
AOC2	AC004890.2	0.304254842	3.61E-12	postive
AOC2	AC005288.1	0.32348421	1.21E-13	postive
AOC2	AC005534.1	0.357613199	1.57E-16	postive
AOC2	AC006042.3	0.460588945	1.27E-27	postive
AOC2	AC006116.10	0.375962899	3.11E-18	postive
AOC2	AC007204.2	0.382157891	7.82E-19	postive
AOC2	AC007638.1	0.300398591	6.92E-12	postive
AOC2	AC007786.2	0.566272927	9.71E-44	postive
AOC2	AC007786.3	0.509611243	2.12E-34	postive
AOC2	AC008060.1	0.55217605	3.01E-41	postive
AOC2	AC008060.3	0.712378002	1.30E-78	postive
AOC2	AC008060.4	0.691118446	3.06E-72	postive
AOC2	AC008556.1	0.316102641	4.59E-13	postive
AOC2	AC008743.1	0.502918586	2.07E-33	postive
AOC2	AC009404.1	0.333475195	1.88E-14	postive
AOC2	AC010722.1	0.315554786	5.06E-13	postive
AOC2	AC011008.1	0.3092325	1.53E-12	postive
AOC2	AC011247.2	0.358793847	1.23E-16	postive
AOC2	AC011396.2	0.381185453	9.73E-19	postive
AOC2	AC012254.1	0.361776638	6.60E-17	postive
AOC2	AC012313.1	0.331585418	2.69E-14	postive
AOC2	AC012360.3	0.306005528	2.67E-12	postive
AOC2	AC012462.1	0.665818226	2.43E-65	postive
AOC2	AC012462.2	0.608066798	7.03E-52	postive
AOC2	AC012668.1	0.484446806	8.73E-31	postive
AOC2	AC012668.2	0.590534893	2.55E-48	postive
AOC2	AC012668.3	0.641957278	1.99E-59	postive
AOC2	AC013472.2	0.345879901	1.70E-15	postive
AOC2	AC015813.1	0.320305661	2.16E-13	postive
AOC2	AC015910.1	0.512953081	6.64E-35	postive
AOC2	AC018521.4	0.32556489	8.27E-14	postive
AOC2	AC018521.7	0.307156424	2.20E-12	postive
AOC2	AC018635.2	0.530646322	1.16E-37	postive
AOC2	AC019069.1	0.344839173	2.08E-15	postive
AOC2	AC024563.1	0.307464258	2.08E-12	postive
AOC2	AC025165.1	0.303533768	4.08E-12	postive
AOC2	AC026367.2	0.318399454	3.04E-13	postive
AOC2	AC026471.1	0.37472089	4.09E-18	postive
AOC2	AC026471.4	0.418070833	1.42E-22	postive
AOC2	AC060780.1	0.333013434	2.06E-14	postive
AOC2	AC061709.2	0.333675439	1.81E-14	postive
AOC2	AC067852.3	0.323437388	1.22E-13	postive
AOC2	AC068039.4	0.370993718	9.23E-18	postive

AOC2	AC068418.2	0.657201263	3.83E-63	postive
AOC2	AC068672.1	0.394989993	4.06E-20	postive
AOC2	AC069222.1	0.302571141	4.80E-12	postive
AOC2	AC069503.3	0.354747839	2.84E-16	postive
AOC2	AC073389.1	0.30106804	6.18E-12	postive
AOC2	AC073389.3	0.334132305	1.66E-14	postive
AOC2	AC073569.2	0.300296487	7.04E-12	postive
AOC2	AC073655.2	0.300671985	6.61E-12	postive
AOC2	AC074117.1	0.332337413	2.34E-14	postive
AOC2	AC084864.1	0.300599542	6.69E-12	postive
AOC2	AC087289.1	0.443297095	1.75E-25	postive
AOC2	AC087318.1	0.30506252	3.14E-12	postive
AOC2	AC089983.1	0.340578699	4.81E-15	postive
AOC2	AC090283.1	0.620768912	1.34E-54	postive
AOC2	AC090844.2	0.309474313	1.47E-12	postive
AOC2	AC092070.4	0.307692607	2.00E-12	postive
AOC2	AC092295.2	0.33016822	3.51E-14	postive
AOC2	AC092364.1	0.66080674	4.71E-64	postive
AOC2	AC092650.1	0.31796975	3.29E-13	postive
AOC2	AC092919.2	0.321723059	1.67E-13	postive
AOC2	AC106782.5	0.342922659	3.04E-15	postive
AOC2	AC106820.2	0.350422547	6.83E-16	postive
AOC2	AC107294.2	0.36668194	2.34E-17	postive
AOC2	AC111198.1	0.438172698	7.14E-25	postive
AOC2	AC112484.1	0.537993831	7.40E-39	postive
AOC2	AC112484.3	0.395005387	4.05E-20	postive
AOC2	AC113208.4	0.327437503	5.85E-14	postive
AOC2	AC114956.1	0.307819826	1.96E-12	postive
AOC2	AC117490.2	0.304801239	3.29E-12	postive
AOC2	AC120114.1	0.341717539	3.85E-15	postive
AOC2	AC121758.2	0.411025446	8.38E-22	postive
AOC2	AC124045.1	0.33846037	7.25E-15	postive
AOC2	AC124944.2	0.324095557	1.08E-13	postive
AOC2	AC131025.1	0.366269343	2.55E-17	postive
AOC2	AC133528.1	0.61888196	3.46E-54	postive
AOC2	AC135050.3	0.318806918	2.83E-13	postive
AOC2	AC138150.2	0.328084318	5.19E-14	postive
AOC2	AC139100.2	0.322253304	1.52E-13	postive
AOC2	AC140125.2	0.334089992	1.68E-14	postive
AOC2	AC145207.1	0.331293365	2.84E-14	postive
AOC2	AC246787.2	0.474956869	1.69E-29	postive
AOC2	AF038458.2	0.426254093	1.72E-23	postive
AOC2	AF038458.3	0.459331404	1.83E-27	postive
AOC2	AL021937.1	0.355740101	2.31E-16	postive
AOC2	AL021937.4	0.30057041	6.72E-12	postive
AOC2	AL022322.1	0.311396987	1.05E-12	postive
AOC2	AL031727.2	0.302827386	4.59E-12	postive
AOC2	AL034417.3	0.307245778	2.16E-12	postive
AOC2	AL035458.2	0.312157014	9.21E-13	postive
AOC2	AL049795.1	0.329601315	3.91E-14	postive

AOC2	AL079307.1	0.445475514	9.55E-26	postive
AOC2	AL121829.1	0.411385333	7.66E-22	postive
AOC2	AL132640.3	0.566314517	9.54E-44	postive
AOC2	AL132780.2	0.3710394	9.14E-18	postive
AOC2	AL133387.2	0.368290357	1.65E-17	postive
AOC2	AL137786.1	0.440033905	4.30E-25	postive
AOC2	AL157400.3	0.31671419	4.11E-13	postive
AOC2	AL158070.1	0.314042101	6.61E-13	postive
AOC2	AL160006.1	0.30012926	7.24E-12	postive
AOC2	AL160314.2	0.318629512	2.92E-13	postive
AOC2	AL162741.1	0.307874671	1.94E-12	postive
AOC2	AL354710.2	0.375023673	3.83E-18	postive
AOC2	AL354989.1	0.380390364	1.16E-18	postive
AOC2	AL355432.1	0.320346634	2.14E-13	postive
AOC2	AL355574.1	0.302611608	4.77E-12	postive
AOC2	AL358334.3	0.304321884	3.57E-12	postive
AOC2	AL365181.4	0.379071343	1.56E-18	postive
AOC2	AL390195.3	0.332255167	2.37E-14	postive
AOC2	AL445231.1	0.336306989	1.10E-14	postive
AOC2	AL451074.2	0.399481048	1.40E-20	postive
AOC2	AL590708.1	0.322872015	1.35E-13	postive
AOC2	AL592435.1	0.370938379	9.35E-18	postive
AOC2	AL645608.6	0.311677943	1.00E-12	postive
AOC2	AL807752.4	0.36384732	4.27E-17	postive
AOC2	AOC4P	0.367011169	2.18E-17	postive
AOC2	AP000446.1	0.33192651	2.52E-14	postive
AOC2	AP001469.2	0.310306002	1.27E-12	postive
AOC2	AP001574.1	0.339029003	6.49E-15	postive
AOC2	AP001767.3	0.356943542	1.81E-16	postive
AOC2	AP003472.2	0.450390694	2.40E-26	postive
AOC2	BCDIN3D-AS1	0.360119901	9.34E-17	postive
AOC2	BOLA2P2	0.343746168	2.59E-15	postive
AOC2	BSN-DT	0.301414279	5.83E-12	postive
AOC2	BX322562.1	0.300587494	6.70E-12	postive
AOC2	BX322639.1	0.379052601	1.57E-18	postive
AOC2	C11orf72	0.313755689	6.95E-13	postive
AOC2	CCDC18-AS1	0.32885551	4.49E-14	postive
AOC2	CCDC84-DT	0.368169187	1.70E-17	postive
AOC2	CD2BP2-DT	0.366661337	2.35E-17	postive
AOC2	CR559946.1	0.312535972	8.62E-13	postive
AOC2	CTBP1-DT	0.32580584	7.91E-14	postive
AOC2	CYP4A22-AS1	0.305791381	2.77E-12	postive
AOC2	DDX11L1	0.495469304	2.48E-32	postive
AOC2	DNAJC27-AS1	0.314909425	5.67E-13	postive
AOC2	DNM1P47	0.396415303	2.90E-20	postive
AOC2	FAM86HP	0.32460879	9.86E-14	postive
AOC2	FAM90A27P	0.717673314	2.72E-80	postive
AOC2	FBXL19-AS1	0.370680159	9.88E-18	postive
AOC2	FER1L4	0.376119484	3.01E-18	postive
AOC2	FGF13-AS1	0.316389127	4.36E-13	postive

AOC2	FP475955.3	0.361510915	6.98E-17	postive
AOC2	GAPDHP72	0.389646097	1.41E-19	postive
AOC2	GLCCI1-DT	0.357145981	1.73E-16	postive
AOC2	GLYATL1P2	0.330772575	3.14E-14	postive
AOC2	GRM8-AS1	0.367674591	1.89E-17	postive
AOC2	HERC2P5	0.313434882	7.36E-13	postive
AOC2	HSD17B3-AS1	0.305780702	2.78E-12	postive
AOC2	INTS6-AS1	0.307252733	2.16E-12	postive
AOC2	KDM2B-DT	0.367449761	1.98E-17	postive
AOC2	LAMTOR5-AS1	0.369018374	1.42E-17	postive
AOC2	LINC00202-1	0.339224225	6.25E-15	postive
AOC2	LINC00307	0.44044733	3.84E-25	postive
AOC2	LINC00334	0.40668734	2.45E-21	postive
AOC2	LINC00359	0.319078295	2.69E-13	postive
AOC2	LINC00471	0.301578001	5.67E-12	postive
AOC2	LINC00607	0.476607164	1.02E-29	postive
AOC2	LINC00663	0.348721574	9.62E-16	postive
AOC2	LINC01054	0.405032459	3.66E-21	postive
AOC2	LINC01100	0.414558299	3.46E-22	postive
AOC2	LINC01115	0.337604424	8.55E-15	postive
AOC2	LINC01166	0.330983048	3.02E-14	postive
AOC2	LINC01320	0.307820642	1.96E-12	postive
AOC2	LINC01399	0.375196677	3.69E-18	postive
AOC2	LINC01518	0.331631633	2.67E-14	postive
AOC2	LINC01563	0.745235968	1.07E-89	postive
AOC2	LINC01627	0.460440004	1.32E-27	postive
AOC2	LINC01646	0.772445438	2.98E-100	postive
AOC2	LINC01798	0.399858373	1.28E-20	postive
AOC2	LINC01892	0.46673997	2.05E-28	postive
AOC2	LINC01968	0.339371776	6.08E-15	postive
AOC2	LINC01979	0.704949434	2.54E-76	postive
AOC2	LINC02029	0.545539636	4.09E-40	postive
AOC2	LINC02094	0.373391	5.48E-18	postive
AOC2	LINC02208	0.648739996	4.70E-61	postive
AOC2	LINC02210	0.307292213	2.14E-12	postive
AOC2	LINC02215	0.300647482	6.63E-12	postive
AOC2	LINC02216	0.515700141	2.54E-35	postive
AOC2	LINC02340	0.388450157	1.86E-19	postive
AOC2	LINC02368	0.76494566	3.36E-97	postive
AOC2	LINC02369	0.588322974	6.92E-48	postive
AOC2	LINC02385	0.7684041	1.36E-98	postive
AOC2	LINC02571	0.317887965	3.34E-13	postive
AOC2	LNCOC1	0.520984912	3.89E-36	postive
AOC2	LRRC3-DT	0.300080752	7.30E-12	postive
AOC2	MEIS1-AS3	0.778164107	1.17E-102	postive
AOC2	METTL14-DT	0.326448882	7.02E-14	postive
AOC2	MGAT4EP	0.526047578	6.26E-37	postive
AOC2	MUC20-OT1	0.325881909	7.80E-14	postive
AOC2	NCBP2-AS1	0.30443281	3.50E-12	postive
AOC2	NRXN2-AS1	0.342948677	3.02E-15	postive

AOC2	OR2S1P	0.323525577	1.20E-13 postive
AOC2	PCOLCE-AS1	0.33965348	5.75E-15 postive
AOC2	PDZK1P1	0.306044705	2.66E-12 postive
AOC2	PRKAR2A-AS1	0.368775377	1.49E-17 postive
AOC2	PRSS42P	0.351781274	5.19E-16 postive
AOC2	PRSS46P	0.352606427	4.39E-16 postive
AOC2	RDM1P5	0.365101918	3.27E-17 postive
AOC2	RFPL3S	0.328003209	5.26E-14 postive
AOC2	RP11-389O22.1	0.345948309	1.67E-15 postive
AOC2	RP3-461P17.10	0.319991952	2.29E-13 postive
AOC2	RP5-1125A11.7	0.337961618	7.98E-15 postive
AOC2	RPL32P3	0.464149864	4.43E-28 postive
AOC2	RPS4XP16	0.310238522	1.29E-12 postive
AOC2	RUNDC3A-AS1	0.38178815	8.50E-19 postive
AOC2	SDR16C6P	0.361518847	6.97E-17 postive
AOC2	SH3BP5-AS1	0.307850674	1.95E-12 postive
AOC2	SLC16A1-AS1	0.398123396	1.94E-20 postive
AOC2	SLC47A1P2	0.420561231	7.53E-23 postive
AOC2	SP2-AS1	0.325452804	8.44E-14 postive
AOC2	SPAG5-AS1	0.302203329	5.11E-12 postive
AOC2	SRRM2-AS1	0.315453466	5.15E-13 postive
AOC2	SSPO	0.511271011	1.19E-34 postive
AOC2	SUMO2P17	0.353902758	3.37E-16 postive
AOC2	TPI1P2	0.702963513	1.01E-75 postive
AOC2	U73166.1	0.304247629	3.61E-12 postive
AOC2	WARS2-AS1	0.321075094	1.88E-13 postive
AOC2	YEATS2-AS1	0.373703554	5.12E-18 postive
AOC2	Z82198.1	0.342982921	3.00E-15 postive
AOC2	ZNF30-AS1	0.396334815	2.96E-20 postive
AOC3	A2M-AS1	0.411742933	7.01E-22 postive
AOC3	AC000403.1	0.378324356	1.85E-18 postive
AOC3	AC002091.2	0.322840802	1.36E-13 postive
AOC3	AC004112.1	0.308446229	1.76E-12 postive
AOC3	AC004540.1	0.318211046	3.15E-13 postive
AOC3	AC004865.2	0.36634437	2.51E-17 postive
AOC3	AC004893.11	0.352220469	4.75E-16 postive
AOC3	AC004908.1	0.381725218	8.62E-19 postive
AOC3	AC004921.1	0.321485743	1.74E-13 postive
AOC3	AC004947.1	0.476077755	1.20E-29 postive
AOC3	AC005034.6	0.309133552	1.56E-12 postive
AOC3	AC005180.1	0.560126312	1.23E-42 postive
AOC3	AC005180.2	0.575228992	2.19E-45 postive
AOC3	AC005264.1	0.321599665	1.71E-13 postive
AOC3	AC005332.4	0.309211644	1.54E-12 postive
AOC3	AC005674.2	0.322502594	1.45E-13 postive
AOC3	AC005740.4	0.331186493	2.90E-14 postive
AOC3	AC005845.1	0.301741912	5.52E-12 postive
AOC3	AC005884.1	0.537412576	9.22E-39 postive
AOC3	AC005901.1	0.461564625	9.50E-28 postive
AOC3	AC006033.2	0.440843952	3.44E-25 postive

AOC3	AC006126.4	0.451607534	1.70E-26	postive
AOC3	AC007406.3	0.446687188	6.80E-26	postive
AOC3	AC007406.5	0.300868899	6.39E-12	postive
AOC3	AC007566.1	0.323054593	1.31E-13	postive
AOC3	AC007671.1	0.596732599	1.49E-49	postive
AOC3	AC007686.3	0.332970752	2.07E-14	postive
AOC3	AC007728.2	0.305735852	2.80E-12	postive
AOC3	AC007861.1	0.365420063	3.06E-17	postive
AOC3	AC007998.3	0.455606489	5.39E-27	postive
AOC3	AC008124.1	0.350391736	6.87E-16	postive
AOC3	AC008494.3	0.324713672	9.67E-14	postive
AOC3	AC008669.1	0.376304749	2.89E-18	postive
AOC3	AC008758.4	0.392631902	7.07E-20	postive
AOC3	AC008764.2	0.453685694	9.36E-27	postive
AOC3	AC008957.1	0.331766796	2.60E-14	postive
AOC3	AC009093.2	0.322958646	1.33E-13	postive
AOC3	AC009093.3	0.346797263	1.41E-15	postive
AOC3	AC009120.3	0.30168708	5.57E-12	postive
AOC3	AC009126.1	0.313496661	7.28E-13	postive
AOC3	AC009133.3	0.315672637	4.95E-13	postive
AOC3	AC009506.2	0.301677343	5.58E-12	postive
AOC3	AC009806.1	0.426744932	1.51E-23	postive
AOC3	AC009831.1	0.362558145	5.60E-17	postive
AOC3	AC009962.1	0.3537479	3.48E-16	postive
AOC3	AC010175.1	0.401503167	8.62E-21	postive
AOC3	AC010247.1	0.347258438	1.29E-15	postive
AOC3	AC010260.1	0.310361079	1.26E-12	postive
AOC3	AC010776.3	0.491744153	8.38E-32	postive
AOC3	AC010931.1	0.319267568	2.60E-13	postive
AOC3	AC010976.2	0.525412342	7.88E-37	postive
AOC3	AC010998.1	0.32565574	8.13E-14	postive
AOC3	AC011365.3	0.452962247	1.15E-26	postive
AOC3	AC011466.1	0.301296848	5.95E-12	postive
AOC3	AC011472.1	0.354545562	2.96E-16	postive
AOC3	AC011477.3	0.349003275	9.09E-16	postive
AOC3	AC011899.2	0.503104987	1.95E-33	postive
AOC3	AC011899.3	0.351459832	5.54E-16	postive
AOC3	AC012313.5	0.311413043	1.05E-12	postive
AOC3	AC012313.6	0.356869859	1.83E-16	postive
AOC3	AC012409.3	0.389599318	1.43E-19	postive
AOC3	AC013553.3	0.574289614	3.28E-45	postive
AOC3	AC015726.1	0.37902901	1.58E-18	postive
AOC3	AC015802.3	0.3349735	1.42E-14	postive
AOC3	AC015819.1	0.304542977	3.43E-12	postive
AOC3	AC015871.3	0.327398549	5.89E-14	postive
AOC3	AC015922.2	0.34599881	1.66E-15	postive
AOC3	AC016885.2	0.411120459	8.18E-22	postive
AOC3	AC018521.6	0.375275383	3.62E-18	postive
AOC3	AC018529.1	0.332605003	2.22E-14	postive
AOC3	AC018647.1	0.648301271	6.01E-61	postive

AOC3	AC018697.1	0.345617538	1.79E-15	postive
AOC3	AC018755.4	0.448383899	4.22E-26	postive
AOC3	AC018809.2	0.318904341	2.78E-13	postive
AOC3	AC019193.2	0.447370571	5.62E-26	postive
AOC3	AC021016.2	0.476655776	1.00E-29	postive
AOC3	AC022098.1	0.499016175	7.66E-33	postive
AOC3	AC022164.1	0.441666894	2.74E-25	postive
AOC3	AC022613.2	0.387243069	2.46E-19	postive
AOC3	AC022915.2	0.385114678	4.00E-19	postive
AOC3	AC024075.1	0.428478022	9.60E-24	postive
AOC3	AC024075.2	0.353446085	3.70E-16	postive
AOC3	AC024075.3	0.359311368	1.11E-16	postive
AOC3	AC024132.4	0.508216836	3.42E-34	postive
AOC3	AC024337.2	0.49158828	8.81E-32	postive
AOC3	AC025279.1	0.422507398	4.56E-23	postive
AOC3	AC025470.2	0.40050181	1.10E-20	postive
AOC3	AC025822.2	0.314103707	6.54E-13	postive
AOC3	AC026369.2	0.429866319	6.65E-24	postive
AOC3	AC026369.3	0.420586077	7.48E-23	postive
AOC3	AC026391.1	0.373343187	5.54E-18	postive
AOC3	AC026780.2	0.375281893	3.62E-18	postive
AOC3	AC026904.2	0.527153337	4.18E-37	postive
AOC3	AC026979.3	0.326812525	6.57E-14	postive
AOC3	AC026992.1	0.305360189	2.99E-12	postive
AOC3	AC026992.2	0.342933812	3.03E-15	postive
AOC3	AC027237.5	0.30878727	1.66E-12	postive
AOC3	AC027348.1	0.349034007	9.03E-16	postive
AOC3	AC027601.3	0.453548441	9.74E-27	postive
AOC3	AC036108.3	0.308259942	1.82E-12	postive
AOC3	AC037459.2	0.359626967	1.03E-16	postive
AOC3	AC044810.3	0.31319585	7.68E-13	postive
AOC3	AC048382.5	0.357112154	1.74E-16	postive
AOC3	AC053503.3	0.336705958	1.02E-14	postive
AOC3	AC055720.2	0.325464645	8.42E-14	postive
AOC3	AC060234.2	0.31565279	4.97E-13	postive
AOC3	AC060766.6	0.394479694	4.58E-20	postive
AOC3	AC060766.7	0.3492984	8.57E-16	postive
AOC3	AC064805.1	0.384748923	4.35E-19	postive
AOC3	AC068700.1	0.301223572	6.02E-12	postive
AOC3	AC068870.2	0.317090502	3.85E-13	postive
AOC3	AC069209.1	0.406320084	2.68E-21	postive
AOC3	AC079467.1	0.301160339	6.09E-12	postive
AOC3	AC080038.2	0.428167321	1.04E-23	postive
AOC3	AC083964.1	0.300817266	6.45E-12	postive
AOC3	AC087477.2	0.440372724	3.92E-25	postive
AOC3	AC087477.5	0.45919934	1.90E-27	postive
AOC3	AC087500.1	0.355339585	2.51E-16	postive
AOC3	AC087500.2	0.325654536	8.13E-14	postive
AOC3	AC087521.1	0.459528916	1.73E-27	postive
AOC3	AC087854.1	0.390635935	1.12E-19	postive

AOC3	AC090044.1	0.413344316	4.70E-22	postive
AOC3	AC090136.3	0.450798485	2.13E-26	postive
AOC3	AC090559.1	0.507598656	4.22E-34	postive
AOC3	AC090825.1	0.34431188	2.31E-15	postive
AOC3	AC091057.3	0.302445903	4.90E-12	postive
AOC3	AC092078.2	0.304728951	3.33E-12	postive
AOC3	AC092134.1	0.314124517	6.52E-13	postive
AOC3	AC092354.2	0.311595506	1.02E-12	postive
AOC3	AC092375.2	0.334902115	1.44E-14	postive
AOC3	AC092376.2	0.459134596	1.94E-27	postive
AOC3	AC092384.1	0.420495679	7.66E-23	postive
AOC3	AC093010.3	0.350353704	6.93E-16	postive
AOC3	AC093110.1	0.540679326	2.66E-39	postive
AOC3	AC093278.2	0.718934907	1.07E-80	postive
AOC3	AC093390.1	0.314541142	6.05E-13	postive
AOC3	AC093484.2	0.30225543	5.06E-12	postive
AOC3	AC093495.1	0.366967823	2.20E-17	postive
AOC3	AC093510.2	0.384979298	4.13E-19	postive
AOC3	AC093726.1	0.41454948	3.47E-22	postive
AOC3	AC095050.1	0.495660277	2.33E-32	postive
AOC3	AC096531.2	0.353840804	3.41E-16	postive
AOC3	AC096733.3	0.378298509	1.86E-18	postive
AOC3	AC096772.6	0.391142373	1.00E-19	postive
AOC3	AC096921.2	0.422023708	5.17E-23	postive
AOC3	AC100803.3	0.486295618	4.84E-31	postive
AOC3	AC103563.7	0.305104958	3.12E-12	postive
AOC3	AC103858.1	0.357708285	1.54E-16	postive
AOC3	AC104083.1	0.513110256	6.29E-35	postive
AOC3	AC104211.2	0.568935794	3.18E-44	postive
AOC3	AC104237.2	0.380785988	1.06E-18	postive
AOC3	AC104237.3	0.444292862	1.33E-25	postive
AOC3	AC104260.1	0.408663564	1.50E-21	postive
AOC3	AC104532.2	0.307054171	2.23E-12	postive
AOC3	AC104758.1	0.327510255	5.77E-14	postive
AOC3	AC104825.1	0.39422417	4.87E-20	postive
AOC3	AC104836.1	0.332273944	2.36E-14	postive
AOC3	AC104938.1	0.36259456	5.56E-17	postive
AOC3	AC104984.2	0.365374939	3.09E-17	postive
AOC3	AC105001.1	0.474025442	2.25E-29	postive
AOC3	AC105020.6	0.376504627	2.76E-18	postive
AOC3	AC105206.2	0.365954692	2.73E-17	postive
AOC3	AC106881.1	0.554105933	1.40E-41	postive
AOC3	AC107959.1	0.579812579	3.01E-46	postive
AOC3	AC108134.3	0.463585417	5.23E-28	postive
AOC3	AC108488.1	0.351213579	5.82E-16	postive
AOC3	AC110995.1	0.360874827	7.98E-17	postive
AOC3	AC112722.1	0.340838949	4.57E-15	postive
AOC3	AC115618.2	0.304989485	3.18E-12	postive
AOC3	AC116312.1	0.363469796	4.62E-17	postive
AOC3	AC116366.2	0.30876499	1.66E-12	postive

AOC3	AC119424.1	0.379974253	1.28E-18	postive
AOC3	AC120049.1	0.494901274	2.99E-32	postive
AOC3	AC120498.2	0.489498841	1.73E-31	postive
AOC3	AC120498.6	0.380054043	1.25E-18	postive
AOC3	AC122129.1	0.48177573	2.03E-30	postive
AOC3	AC126177.3	0.447381421	5.60E-26	postive
AOC3	AC126755.1	0.310170401	1.30E-12	postive
AOC3	AC127521.1	0.305958568	2.70E-12	postive
AOC3	AC129507.1	0.305314623	3.01E-12	postive
AOC3	AC131097.3	0.336111687	1.14E-14	postive
AOC3	AC135012.3	0.635195988	7.60E-58	postive
AOC3	AC135178.2	0.538948044	5.15E-39	postive
AOC3	AC135782.2	0.304992522	3.18E-12	postive
AOC3	AC138028.4	0.318161877	3.18E-13	postive
AOC3	AC138207.6	0.308846464	1.64E-12	postive
AOC3	AC138356.1	0.331762148	2.60E-14	postive
AOC3	AC138932.1	0.358896036	1.21E-16	postive
AOC3	AC142381.3	0.318110462	3.21E-13	postive
AOC3	AC145098.1	0.405004421	3.69E-21	postive
AOC3	AC147067.2	0.414695873	3.35E-22	postive
AOC3	AC241952.2	0.311364964	1.06E-12	postive
AOC3	AC243829.4	0.301476407	5.77E-12	postive
AOC3	AC245884.11	0.309472834	1.47E-12	postive
AOC3	ACOXL-AS1	0.307463233	2.08E-12	postive
AOC3	ACTA2-AS1	0.414969989	3.12E-22	postive
AOC3	ADAMTS7P1	0.408788671	1.46E-21	postive
AOC3	ADAMTS7P3	0.653015772	4.22E-62	postive
AOC3	ADAMTS9-AS2	0.308741307	1.67E-12	postive
AOC3	ADGRE4P	0.385427364	3.73E-19	postive
AOC3	ADPGK-AS1	0.315746357	4.89E-13	postive
AOC3	AF001548.1	0.334239361	1.63E-14	postive
AOC3	AF111167.2	0.313499284	7.28E-13	postive
AOC3	AF111169.3	0.341838005	3.76E-15	postive
AOC3	AF131215.5	0.372101911	7.26E-18	postive
AOC3	AGAP11	0.304634226	3.38E-12	postive
AOC3	AL009178.2	0.535805733	1.69E-38	postive
AOC3	AL021026.1	0.316559821	4.23E-13	postive
AOC3	AL021368.2	0.30861881	1.71E-12	postive
AOC3	AL023653.1	0.315892123	4.76E-13	postive
AOC3	AL031846.2	0.43194638	3.83E-24	postive
AOC3	AL034397.3	0.309302311	1.52E-12	postive
AOC3	AL035409.1	0.41966855	9.47E-23	postive
AOC3	AL035530.1	0.344473696	2.24E-15	postive
AOC3	AL035530.2	0.306311508	2.54E-12	postive
AOC3	AL035587.1	0.348152456	1.08E-15	postive
AOC3	AL049569.1	0.398787171	1.65E-20	postive
AOC3	AL049796.1	0.314081531	6.57E-13	postive
AOC3	AL049840.4	0.33595122	1.17E-14	postive
AOC3	AL049840.5	0.346509254	1.50E-15	postive
AOC3	AL049871.1	0.420369954	7.91E-23	postive

AOC3	AL078581.2	0.366049326	2.67E-17	postive
AOC3	AL109741.1	0.567859697	5.00E-44	postive
AOC3	AL122035.1	0.334564273	1.53E-14	postive
AOC3	AL133297.2	0.346664828	1.45E-15	postive
AOC3	AL133355.1	0.426833452	1.48E-23	postive
AOC3	AL133371.2	0.371655754	8.00E-18	postive
AOC3	AL133406.2	0.305567803	2.88E-12	postive
AOC3	AL135960.1	0.563449377	3.13E-43	postive
AOC3	AL136084.3	0.414511158	3.50E-22	postive
AOC3	AL136368.1	0.324656121	9.77E-14	postive
AOC3	AL136369.1	0.331258941	2.86E-14	postive
AOC3	AL136982.1	0.376217782	2.94E-18	postive
AOC3	AL137003.1	0.361312918	7.28E-17	postive
AOC3	AL138995.1	0.300242904	7.10E-12	postive
AOC3	AL139231.1	0.515582704	2.65E-35	postive
AOC3	AL139339.1	0.385165088	3.96E-19	postive
AOC3	AL157394.1	0.337916677	8.05E-15	postive
AOC3	AL157895.2	0.453348804	1.03E-26	postive
AOC3	AL157935.3	0.304380192	3.53E-12	postive
AOC3	AL158211.1	0.33334112	1.93E-14	postive
AOC3	AL158212.3	0.303273124	4.26E-12	postive
AOC3	AL160153.1	0.364422667	3.78E-17	postive
AOC3	AL160313.2	0.324643108	9.80E-14	postive
AOC3	AL353593.1	0.334125146	1.66E-14	postive
AOC3	AL354861.3	0.559386798	1.66E-42	postive
AOC3	AL355803.1	0.49289213	5.76E-32	postive
AOC3	AL356056.2	0.313844754	6.85E-13	postive
AOC3	AL356599.1	0.347932249	1.13E-15	postive
AOC3	AL357054.4	0.428028	1.08E-23	postive
AOC3	AL357146.1	0.354149722	3.21E-16	postive
AOC3	AL359532.1	0.345730191	1.75E-15	postive
AOC3	AL360181.2	0.308115387	1.86E-12	postive
AOC3	AL390755.1	0.494324488	3.61E-32	postive
AOC3	AL391807.1	0.406067377	2.85E-21	postive
AOC3	AL391834.2	0.329757167	3.79E-14	postive
AOC3	AL445307.1	0.499664488	6.17E-33	postive
AOC3	AL445423.1	0.705579855	1.63E-76	postive
AOC3	AL445426.1	0.484399045	8.86E-31	postive
AOC3	AL450326.1	0.33356121	1.85E-14	postive
AOC3	AL512791.1	0.339048257	6.47E-15	postive
AOC3	AL583856.2	0.331267237	2.86E-14	postive
AOC3	AL590226.1	0.63197185	4.18E-57	postive
AOC3	AL591686.1	0.429294171	7.74E-24	postive
AOC3	AL606469.1	0.573203519	5.22E-45	postive
AOC3	AL645728.1	0.309235051	1.53E-12	postive
AOC3	AL662844.4	0.326417377	7.06E-14	postive
AOC3	AL691432.2	0.303648199	4.00E-12	postive
AOC3	AL731567.1	0.311417593	1.05E-12	postive
AOC3	AL731577.2	0.362835125	5.28E-17	postive
AOC3	AOC4P	0.494450418	3.46E-32	postive

AOC3	AP000438.1	0.455314181	5.86E-27	postive
AOC3	AP000766.1	0.304170199	3.66E-12	postive
AOC3	AP000915.1	0.310578971	1.21E-12	postive
AOC3	AP001056.1	0.319348949	2.57E-13	postive
AOC3	AP001094.2	0.400913582	9.93E-21	postive
AOC3	AP001107.5	0.324860049	9.41E-14	postive
AOC3	AP001189.1	0.631651378	4.94E-57	postive
AOC3	AP001189.3	0.694961417	2.37E-73	postive
AOC3	AP001330.4	0.301617736	5.64E-12	postive
AOC3	AP001486.2	0.333106337	2.02E-14	postive
AOC3	AP002761.3	0.352684747	4.32E-16	postive
AOC3	AP002840.2	0.364470255	3.74E-17	postive
AOC3	AP003071.4	0.381347299	9.39E-19	postive
AOC3	AP003355.2	0.392484591	7.32E-20	postive
AOC3	AP003486.1	0.377840147	2.06E-18	postive
AOC3	AP003557.1	0.310253491	1.28E-12	postive
AOC3	ATP2B1-AS1	0.375311121	3.59E-18	postive
AOC3	ATXN1-AS1	0.308357079	1.78E-12	postive
AOC3	BCRP3	0.300378363	6.94E-12	postive
AOC3	BTN2A3P	0.325356623	8.59E-14	postive
AOC3	C17orf102	0.303111944	4.38E-12	postive
AOC3	C21orf62-AS1	0.330066213	3.58E-14	postive
AOC3	C5orf64	0.339261253	6.21E-15	postive
AOC3	CACNA1C-AS1	0.305937038	2.71E-12	postive
AOC3	CADM3-AS1	0.463107001	6.03E-28	postive
AOC3	CARD8-AS1	0.347044946	1.34E-15	postive
AOC3	CASP17P	0.410560837	9.41E-22	postive
AOC3	CENPBD1P1	0.332771505	2.15E-14	postive
AOC3	CEP250-AS1	0.312103414	9.30E-13	postive
AOC3	CHIAP2	0.347381023	1.26E-15	postive
AOC3	COX10-AS1	0.309589394	1.44E-12	postive
AOC3	CSPG4P13	0.305525239	2.90E-12	postive
AOC3	CTC-205M6.5	0.332698172	2.18E-14	postive
AOC3	CTC-786C10.1	0.355654107	2.36E-16	postive
AOC3	DACT3-AS1	0.423575149	3.46E-23	postive
AOC3	DNM1P46	0.318247163	3.13E-13	postive
AOC3	DNM3OS	0.323411207	1.23E-13	postive
AOC3	EIF3J-DT	0.32426799	1.05E-13	postive
AOC3	EPB41L4A-DT	0.409032782	1.37E-21	postive
AOC3	FAM66C	0.331716661	2.63E-14	postive
AOC3	FENDRR	0.627912919	3.47E-56	postive
AOC3	FGD5-AS1	0.337023651	9.56E-15	postive
AOC3	FRG1HP	0.305314358	3.01E-12	postive
AOC3	GGTA1P	0.519302161	7.09E-36	postive
AOC3	GRPEL2-AS1	0.334404658	1.58E-14	postive
AOC3	GTF2IP4	0.337211303	9.22E-15	postive
AOC3	GVINP1	0.411575351	7.31E-22	postive
AOC3	HCG11	0.330848244	3.09E-14	postive
AOC3	HECW2-AS1	0.307818574	1.96E-12	postive
AOC3	HID1-AS1	0.709607671	9.46E-78	postive

AOC3	HLA-DQB1-AS1	0.314161387	6.47E-13	postive
AOC3	HLX-AS1	0.569247783	2.79E-44	postive
AOC3	IGFBP7-AS1	0.380927568	1.03E-18	postive
AOC3	IL10RB-DT	0.300895273	6.36E-12	postive
AOC3	IRF1-AS1	0.32208083	1.56E-13	postive
AOC3	ITGA9-AS1	0.44466917	1.19E-25	postive
AOC3	JAZF1-AS1	0.320044064	2.26E-13	postive
AOC3	KIAA1671-AS1	0.302008573	5.28E-12	postive
AOC3	L3MBTL2-AS1	0.370776723	9.68E-18	postive
AOC3	LANCL1-AS1	0.353301157	3.81E-16	postive
AOC3	LBX1-AS1	0.332282791	2.36E-14	postive
AOC3	LIMD1-AS1	0.336299874	1.10E-14	postive
AOC3	LINC00032	0.420911812	6.88E-23	postive
AOC3	LINC00092	0.359953356	9.67E-17	postive
AOC3	LINC00163	0.557593094	3.43E-42	postive
AOC3	LINC00310	0.449036772	3.51E-26	postive
AOC3	LINC00469	0.348019008	1.11E-15	postive
AOC3	LINC00551	0.403588866	5.21E-21	postive
AOC3	LINC00656	0.431612571	4.18E-24	postive
AOC3	LINC00663	0.319339557	2.57E-13	postive
AOC3	LINC00670	0.420321029	8.01E-23	postive
AOC3	LINC00702	0.534786548	2.48E-38	postive
AOC3	LINC00844	0.394559391	4.50E-20	postive
AOC3	LINC00863	0.345259038	1.92E-15	postive
AOC3	LINC00891	0.330143991	3.53E-14	postive
AOC3	LINC00892	0.369825934	1.19E-17	postive
AOC3	LINC00899	0.35085109	6.27E-16	postive
AOC3	LINC00921	0.49824758	9.89E-33	postive
AOC3	LINC00924	0.521491305	3.25E-36	postive
AOC3	LINC00926	0.302196997	5.11E-12	postive
AOC3	LINC00968	0.784349197	2.40E-105	postive
AOC3	LINC00987	0.626946773	5.72E-56	postive
AOC3	LINC00996	0.389646059	1.41E-19	postive
AOC3	LINC01081	0.528044806	3.02E-37	postive
AOC3	LINC01082	0.406653273	2.47E-21	postive
AOC3	LINC01128	0.331602559	2.68E-14	postive
AOC3	LINC01140	0.445920732	8.43E-26	postive
AOC3	LINC01150	0.439558847	4.89E-25	postive
AOC3	LINC01197	0.539059422	4.94E-39	postive
AOC3	LINC01228	0.326707155	6.69E-14	postive
AOC3	LINC01352	0.742771267	8.32E-89	postive
AOC3	LINC01394	0.33859903	7.06E-15	postive
AOC3	LINC01412	0.470248872	7.11E-29	postive
AOC3	LINC01447	0.351278775	5.75E-16	postive
AOC3	LINC01624	0.431129419	4.76E-24	postive
AOC3	LINC01645	0.35400906	3.30E-16	postive
AOC3	LINC01678	0.372378109	6.83E-18	postive
AOC3	LINC01779	0.315592001	5.02E-13	postive
AOC3	LINC01797	0.35186352	5.11E-16	postive
AOC3	LINC01798	0.399475705	1.40E-20	postive

AOC3	LINC01836	0.362547425	5.61E-17	postive
AOC3	LINC01852	0.422191053	4.95E-23	postive
AOC3	LINC01915	0.332186997	2.40E-14	postive
AOC3	LINC01936	0.847511368	4.28E-139	postive
AOC3	LINC01945	0.50420131	1.34E-33	postive
AOC3	LINC01963	0.363906358	4.21E-17	postive
AOC3	LINC01985	0.363283424	4.81E-17	postive
AOC3	LINC01996	0.341871387	3.74E-15	postive
AOC3	LINC02016	0.495276766	2.64E-32	postive
AOC3	LINC02033	0.338731385	6.88E-15	postive
AOC3	LINC02035	0.35811959	1.42E-16	postive
AOC3	LINC02073	0.331520764	2.72E-14	postive
AOC3	LINC02104	0.312645073	8.46E-13	postive
AOC3	LINC02126	0.515224344	3.00E-35	postive
AOC3	LINC02147	0.355172773	2.60E-16	postive
AOC3	LINC02202	0.506579443	5.99E-34	postive
AOC3	LINC02256	0.327179012	6.13E-14	postive
AOC3	LINC02273	0.377875075	2.04E-18	postive
AOC3	LINC02285	0.361400574	7.14E-17	postive
AOC3	LINC02371	0.315157649	5.43E-13	postive
AOC3	LINC02391	0.336520415	1.05E-14	postive
AOC3	LINC02408	0.358285758	1.37E-16	postive
AOC3	LINC02519	0.514189011	4.31E-35	postive
AOC3	LINC02580	0.46312812	5.99E-28	postive
AOC3	LINC02693	0.317586325	3.52E-13	postive
AOC3	LINC02705	0.375631036	3.35E-18	postive
AOC3	LINC02752	0.443967189	1.45E-25	postive
AOC3	LINC02785	0.315010612	5.57E-13	postive
AOC3	LINC02828	0.313537103	7.23E-13	postive
AOC3	LINC02829	0.430686851	5.35E-24	postive
AOC3	LMO7DN	0.313854853	6.83E-13	postive
AOC3	LMO7DN-IT1	0.319446521	2.52E-13	postive
AOC3	LPAL2	0.399293301	1.46E-20	postive
AOC3	LRRC37A17P	0.428700804	9.05E-24	postive
AOC3	LRRC8C-DT	0.382900414	6.61E-19	postive
AOC3	MAGI2-AS3	0.463300044	5.69E-28	postive
AOC3	MAGOH2P	0.344107765	2.41E-15	postive
AOC3	MAP2K4P1	0.310897763	1.15E-12	postive
AOC3	MBL1P	0.388171758	1.99E-19	postive
AOC3	MBNL1-AS1	0.373845257	4.96E-18	postive
AOC3	MIR1-1HG-AS1	0.486616518	4.37E-31	postive
AOC3	MIR22HG	0.43716943	9.38E-25	postive
AOC3	MIR497HG	0.60369913	5.68E-51	postive
AOC3	MMADHC-DT	0.34458552	2.19E-15	postive
AOC3	MRGPRF-AS1	0.428207244	1.03E-23	postive
AOC3	MTMR9LP	0.354402161	3.04E-16	postive
AOC3	NAPSB	0.414121545	3.87E-22	postive
AOC3	NBPF25P	0.318250571	3.13E-13	postive
AOC3	NORAD	0.3749104	3.93E-18	postive
AOC3	NR2F2-AS1	0.361890581	6.45E-17	postive

AOC3	NR4A1AS	0.365088539	3.28E-17	postive
AOC3	OR14L1P	0.304770208	3.30E-12	postive
AOC3	OR52K3P	0.34755593	1.21E-15	postive
AOC3	OR7E47P	0.652046248	7.31E-62	postive
AOC3	OR7E94P	0.469928596	7.84E-29	postive
AOC3	PAN3-AS1	0.302957171	4.50E-12	postive
AOC3	PAXIP1-AS2	0.470479081	6.63E-29	postive
AOC3	PCAT19	0.370583218	1.01E-17	postive
AOC3	PCED1B-AS1	0.337049332	9.52E-15	postive
AOC3	PGM5-AS1	0.307828811	1.96E-12	postive
AOC3	PKNOX2-AS1	0.365146628	3.24E-17	postive
AOC3	PLAC9P1	0.367445068	1.98E-17	postive
AOC3	PPP3CB-AS1	0.323487954	1.21E-13	postive
AOC3	PRR34	0.363386391	4.70E-17	postive
AOC3	PTPRD-AS1	0.507662795	4.13E-34	postive
AOC3	RAP2C-AS1	0.361468302	7.04E-17	postive
AOC3	RP11-136H19.1	0.520400836	4.79E-36	postive
AOC3	RP11-180N14.1	0.400678099	1.05E-20	postive
AOC3	RP11-182J1.1	0.406522757	2.55E-21	postive
AOC3	RP11-229P13.19	0.312781484	8.26E-13	postive
AOC3	RP1-122P22.2	0.403872542	4.86E-21	postive
AOC3	RP11-327L3.3	0.36685765	2.25E-17	postive
AOC3	RP11-346C20.4	0.303812043	3.89E-12	postive
AOC3	RP11-394O4.5	0.505276046	9.33E-34	postive
AOC3	RP11-401P9.4	0.39657153	2.80E-20	postive
AOC3	RP11-514D23.3	0.445604987	9.21E-26	postive
AOC3	RP11-554D14.8	0.328697022	4.63E-14	postive
AOC3	RP1-167F1.2	0.337103172	9.42E-15	postive
AOC3	RP11-876N24.4	0.323642398	1.18E-13	postive
AOC3	RP13-36G14.4	0.314346714	6.26E-13	postive
AOC3	RP13-514E23.1	0.336064074	1.15E-14	postive
AOC3	RP4-575N6.5	0.53691048	1.11E-38	postive
AOC3	RP5-1042I8.7	0.358714554	1.25E-16	postive
AOC3	RPL23AP53	0.389838917	1.35E-19	postive
AOC3	RRN3P2	0.390618903	1.13E-19	postive
AOC3	RXYLT1-AS1	0.331453024	2.76E-14	postive
AOC3	SCAND2P	0.356342968	2.04E-16	postive
AOC3	SEC22B4P	0.329579467	3.92E-14	postive
AOC3	SH3BP5-AS1	0.314038105	6.62E-13	postive
AOC3	SH3RF3-AS1	0.468286051	1.29E-28	postive
AOC3	SIGLEC17P	0.543156936	1.03E-39	postive
AOC3	SIGLEC22P	0.46875829	1.12E-28	postive
AOC3	SLC6A1-AS1	0.33902722	6.50E-15	postive
AOC3	SMIM25	0.481589867	2.15E-30	postive
AOC3	SNAI3-AS1	0.309602147	1.44E-12	postive
AOC3	ST20-AS1	0.347959081	1.12E-15	postive
AOC3	STRA6LP	0.342901815	3.05E-15	postive
AOC3	TBC1D27P	0.311868381	9.69E-13	postive
AOC3	TBX2-AS1	0.4831578	1.31E-30	postive
AOC3	TBX5-AS1	0.828550247	1.58E-127	postive

AOC3	TCAF2P1	0.319496625	2.50E-13	postive
AOC3	TMEM198B	0.311719234	9.95E-13	postive
AOC3	TP73-AS1	0.352530399	4.46E-16	postive
AOC3	TSPOAP1-AS1	0.379513834	1.42E-18	postive
AOC3	U62631.1	0.332164356	2.41E-14	postive
AOC3	WDFY3-AS2	0.361826845	6.53E-17	postive
AOC3	WNT5A-AS1	0.309100149	1.57E-12	postive
AOC3	WWC2-AS2	0.348231636	1.06E-15	postive
AOC3	Z69706.1	0.312087419	9.32E-13	postive
AOC3	Z84492.1	0.383965324	5.20E-19	postive
AOC3	Z94057.1	0.325814969	7.89E-14	postive
AOC3	Z97989.1	0.491037476	1.05E-31	postive
AOC3	ZEB2-AS1	0.384497866	4.61E-19	postive
AOC3	ZNF582-AS1	0.331398373	2.79E-14	postive
APLP2	AATBC	0.389071387	1.62E-19	postive
APLP2	ABCC6P1	0.307725814	1.99E-12	postive
APLP2	ABCC6P2	0.393356376	5.97E-20	postive
APLP2	ABHD11-AS1	0.315642265	4.98E-13	postive
APLP2	AC000403.1	0.345941928	1.67E-15	postive
APLP2	AC002401.2	0.305974666	2.69E-12	postive
APLP2	AC003070.1	0.3872421	2.46E-19	postive
APLP2	AC004067.1	0.365393686	3.07E-17	postive
APLP2	AC004233.1	0.345055544	2.00E-15	postive
APLP2	AC004596.1	0.300821927	6.44E-12	postive
APLP2	AC004771.2	0.386677492	2.80E-19	postive
APLP2	AC004846.1	0.358075548	1.43E-16	postive
APLP2	AC004893.11	0.381188266	9.73E-19	postive
APLP2	AC004943.2	0.357932757	1.47E-16	postive
APLP2	AC004951.1	0.322545922	1.44E-13	postive
APLP2	AC004980.1	0.304274199	3.59E-12	postive
APLP2	AC004982.1	0.333103785	2.02E-14	postive
APLP2	AC004982.2	0.439562337	4.89E-25	postive
APLP2	AC005034.4	0.354721801	2.85E-16	postive
APLP2	AC005034.6	0.326296556	7.22E-14	postive
APLP2	AC005041.3	0.340513342	4.87E-15	postive
APLP2	AC005288.1	0.407820038	1.85E-21	postive
APLP2	AC005332.3	0.358822055	1.22E-16	postive
APLP2	AC005332.6	0.539658363	3.93E-39	postive
APLP2	AC005479.1	0.365320659	3.12E-17	postive
APLP2	AC005498.3	0.315313118	5.28E-13	postive
APLP2	AC005520.4	0.327180863	6.13E-14	postive
APLP2	AC005562.1	0.486515495	4.52E-31	postive
APLP2	AC005670.3	0.430788448	5.21E-24	postive
APLP2	AC005790.1	0.302367661	4.97E-12	postive
APLP2	AC006001.3	0.337933017	8.03E-15	postive
APLP2	AC006126.4	0.301506424	5.74E-12	postive
APLP2	AC006213.2	0.338222151	7.59E-15	postive
APLP2	AC006449.2	0.319612159	2.45E-13	postive
APLP2	AC007228.9	0.313290669	7.55E-13	postive
APLP2	AC007292.2	0.325945244	7.71E-14	postive

APLP2	AC007405.4	0.32004599	2.26E-13	postive
APLP2	AC007485.1	0.323889539	1.12E-13	postive
APLP2	AC007613.1	0.308664221	1.69E-12	postive
APLP2	AC007663.4	0.327905154	5.36E-14	postive
APLP2	AC008105.2	0.325428384	8.48E-14	postive
APLP2	AC008115.4	0.324591781	9.89E-14	postive
APLP2	AC008124.1	0.331413124	2.78E-14	postive
APLP2	AC008268.1	0.316002194	4.67E-13	postive
APLP2	AC008494.3	0.363120697	4.97E-17	postive
APLP2	AC008655.2	0.348395806	1.03E-15	postive
APLP2	AC008663.2	0.301617051	5.64E-12	postive
APLP2	AC008669.1	0.310305221	1.27E-12	postive
APLP2	AC008758.4	0.392063281	8.07E-20	postive
APLP2	AC008760.1	0.345235476	1.93E-15	postive
APLP2	AC008763.1	0.316775115	4.07E-13	postive
APLP2	AC008764.2	0.45350178	9.87E-27	postive
APLP2	AC008771.1	0.445843495	8.61E-26	postive
APLP2	AC008915.3	0.319138165	2.67E-13	postive
APLP2	AC008966.1	0.40562782	3.17E-21	postive
APLP2	AC009041.1	0.365229503	3.18E-17	postive
APLP2	AC009041.3	0.43660383	1.09E-24	postive
APLP2	AC009090.3	0.349381091	8.43E-16	postive
APLP2	AC009113.1	0.354808851	2.80E-16	postive
APLP2	AC009120.3	0.303664155	3.99E-12	postive
APLP2	AC009126.1	0.32638882	7.10E-14	postive
APLP2	AC009133.1	0.301935439	5.34E-12	postive
APLP2	AC009237.14	0.463558054	5.27E-28	postive
APLP2	AC009237.3	0.321593944	1.71E-13	postive
APLP2	AC009336.1	0.382019439	8.07E-19	postive
APLP2	AC009506.2	0.393955939	5.18E-20	postive
APLP2	AC009779.3	0.367647671	1.90E-17	postive
APLP2	AC009831.1	0.323476227	1.21E-13	postive
APLP2	AC010226.1	0.346989942	1.36E-15	postive
APLP2	AC010245.2	0.309566973	1.45E-12	postive
APLP2	AC010503.5	0.407239096	2.14E-21	postive
APLP2	AC010519.1	0.332632213	2.21E-14	postive
APLP2	AC010761.4	0.385527727	3.64E-19	postive
APLP2	AC010969.2	0.385493948	3.67E-19	postive
APLP2	AC011346.1	0.327459318	5.82E-14	postive
APLP2	AC011374.2	0.300506421	6.79E-12	postive
APLP2	AC011442.1	0.303782893	3.91E-12	postive
APLP2	AC011472.1	0.318467203	3.01E-13	postive
APLP2	AC011476.3	0.316905427	3.98E-13	postive
APLP2	AC011477.1	0.301777228	5.49E-12	postive
APLP2	AC011477.2	0.360220313	9.14E-17	postive
APLP2	AC011477.3	0.406843669	2.35E-21	postive
APLP2	AC011511.2	0.415522892	2.72E-22	postive
APLP2	AC011511.5	0.370748045	9.74E-18	postive
APLP2	AC012306.2	0.321288952	1.81E-13	postive
APLP2	AC012313.5	0.404318556	4.36E-21	postive

APLP2	AC012618.3	0.344009582	2.45E-15	postive
APLP2	AC013275.1	0.342122327	3.56E-15	postive
APLP2	AC013356.2	0.393468005	5.81E-20	postive
APLP2	AC015871.3	0.326308214	7.21E-14	postive
APLP2	AC015922.2	0.436336939	1.18E-24	postive
APLP2	AC015922.3	0.350192281	7.16E-16	postive
APLP2	AC016582.3	0.313261156	7.59E-13	postive
APLP2	AC016727.1	0.322568326	1.43E-13	postive
APLP2	AC016747.1	0.387239018	2.46E-19	postive
APLP2	AC018521.5	0.30577161	2.78E-12	postive
APLP2	AC018521.6	0.344799598	2.10E-15	postive
APLP2	AC018647.2	0.328134666	5.14E-14	postive
APLP2	AC018809.2	0.478278975	6.05E-30	postive
APLP2	AC019131.2	0.303675312	3.98E-12	postive
APLP2	AC019193.2	0.34586169	1.70E-15	postive
APLP2	AC020915.3	0.317143085	3.81E-13	postive
APLP2	AC020978.4	0.325720098	8.03E-14	postive
APLP2	AC021016.2	0.375381935	3.54E-18	postive
APLP2	AC021148.1	0.30598742	2.68E-12	postive
APLP2	AC021483.2	0.309639274	1.43E-12	postive
APLP2	AC022007.1	0.304154769	3.67E-12	postive
APLP2	AC022098.1	0.334986835	1.41E-14	postive
APLP2	AC022150.2	0.370761102	9.71E-18	postive
APLP2	AC022211.3	0.329702686	3.83E-14	postive
APLP2	AC022400.1	0.384116038	5.02E-19	postive
APLP2	AC022915.2	0.316911635	3.97E-13	postive
APLP2	AC023509.1	0.302260928	5.06E-12	postive
APLP2	AC023509.2	0.308963856	1.61E-12	postive
APLP2	AC024075.1	0.431336848	4.50E-24	postive
APLP2	AC024075.2	0.361372339	7.19E-17	postive
APLP2	AC024075.3	0.418894387	1.15E-22	postive
APLP2	AC024909.1	0.387183478	2.49E-19	postive
APLP2	AC024941.2	0.355997801	2.19E-16	postive
APLP2	AC025171.1	0.332786125	2.15E-14	postive
APLP2	AC025171.2	0.367260555	2.06E-17	postive
APLP2	AC025171.5	0.308166808	1.84E-12	postive
APLP2	AC025287.3	0.308820126	1.65E-12	postive
APLP2	AC026150.2	0.301824816	5.44E-12	postive
APLP2	AC026471.1	0.365286404	3.14E-17	postive
APLP2	AC026691.1	0.305812448	2.76E-12	postive
APLP2	AC027601.1	0.353856405	3.40E-16	postive
APLP2	AC037459.2	0.301904212	5.37E-12	postive
APLP2	AC040977.1	0.330319955	3.41E-14	postive
APLP2	AC044802.2	0.325872489	7.81E-14	postive
APLP2	AC055720.2	0.381457715	9.16E-19	postive
APLP2	AC060766.7	0.364414894	3.78E-17	postive
APLP2	AC060780.1	0.321301768	1.80E-13	postive
APLP2	AC067852.2	0.496348269	1.85E-32	postive
APLP2	AC067852.3	0.324761654	9.58E-14	postive
APLP2	AC068580.1	0.388799373	1.72E-19	postive

APLP2	AC068580.3	0.475374813	1.49E-29	postive
APLP2	AC068594.1	0.417647558	1.59E-22	postive
APLP2	AC068870.2	0.317572826	3.53E-13	postive
APLP2	AC069503.1	0.333715018	1.80E-14	postive
APLP2	AC073073.2	0.333348816	1.93E-14	postive
APLP2	AC073254.1	0.4129859	5.14E-22	postive
APLP2	AC073508.3	0.313390473	7.42E-13	postive
APLP2	AC079807.1	0.335506141	1.28E-14	postive
APLP2	AC080112.2	0.388220718	1.97E-19	postive
APLP2	AC083964.1	0.430205315	6.08E-24	postive
APLP2	AC084809.1	0.38044437	1.15E-18	postive
APLP2	AC087521.3	0.341463172	4.05E-15	postive
APLP2	AC090559.1	0.438659303	6.26E-25	postive
APLP2	AC090587.2	0.335848143	1.20E-14	postive
APLP2	AC091563.1	0.453686587	9.36E-27	postive
APLP2	AC091849.2	0.375387386	3.54E-18	postive
APLP2	AC092070.2	0.408870089	1.43E-21	postive
APLP2	AC092354.2	0.301027852	6.22E-12	postive
APLP2	AC092368.3	0.558629167	2.26E-42	postive
APLP2	AC092614.1	0.301562827	5.69E-12	postive
APLP2	AC092718.5	0.313965297	6.70E-13	postive
APLP2	AC092807.3	0.351458716	5.54E-16	postive
APLP2	AC093010.3	0.339742085	5.66E-15	postive
APLP2	AC093227.1	0.31263919	8.46E-13	postive
APLP2	AC093278.2	0.325603974	8.21E-14	postive
APLP2	AC093525.7	0.351733613	5.24E-16	postive
APLP2	AC093627.4	0.376225518	2.94E-18	postive
APLP2	AC093627.5	0.355720449	2.32E-16	postive
APLP2	AC093726.1	0.476448762	1.07E-29	postive
APLP2	AC093726.2	0.341014227	4.42E-15	postive
APLP2	AC093752.1	0.342851258	3.08E-15	postive
APLP2	AC093752.3	0.350791315	6.34E-16	postive
APLP2	AC096536.2	0.365889868	2.77E-17	postive
APLP2	AC096677.1	0.329786076	3.77E-14	postive
APLP2	AC096733.3	0.357740619	1.53E-16	postive
APLP2	AC096751.1	0.319672656	2.42E-13	postive
APLP2	AC096772.6	0.448449677	4.15E-26	postive
APLP2	AC097359.2	0.389267214	1.54E-19	postive
APLP2	AC097639.1	0.360466456	8.69E-17	postive
APLP2	AC098484.1	0.391659996	8.87E-20	postive
APLP2	AC100786.1	0.395221659	3.85E-20	postive
APLP2	AC100860.1	0.306384736	2.51E-12	postive
APLP2	AC100861.1	0.322114839	1.56E-13	postive
APLP2	AC103719.1	0.342793215	3.12E-15	postive
APLP2	AC104083.1	0.427835195	1.14E-23	postive
APLP2	AC104109.2	0.368783005	1.49E-17	postive
APLP2	AC104117.3	0.330771302	3.14E-14	postive
APLP2	AC104564.3	0.308170359	1.84E-12	postive
APLP2	AC104794.3	0.394689357	4.36E-20	postive
APLP2	AC104819.3	0.41792654	1.48E-22	postive

APLP2	AC104825.1	0.311832617	9.75E-13 postive
APLP2	AC104938.1	0.364517845	3.70E-17 postive
APLP2	AC105206.2	0.353970954	3.32E-16 postive
APLP2	AC105942.1	0.387463883	2.34E-19 postive
APLP2	AC106739.1	0.454269095	7.92E-27 postive
APLP2	AC106782.5	0.314030923	6.62E-13 postive
APLP2	AC106786.2	0.427453211	1.26E-23 postive
APLP2	AC106820.5	0.333962269	1.72E-14 postive
APLP2	AC107884.2	0.315319547	5.27E-13 postive
APLP2	AC107959.1	0.331404081	2.79E-14 postive
APLP2	AC108010.1	0.323409748	1.23E-13 postive
APLP2	AC108134.1	0.338394341	7.34E-15 postive
APLP2	AC108134.2	0.372750285	6.30E-18 postive
APLP2	AC108156.1	0.300671668	6.61E-12 postive
APLP2	AC110079.1	0.405671515	3.14E-21 postive
APLP2	AC110285.3	0.415160229	2.98E-22 postive
APLP2	AC110995.1	0.325769188	7.96E-14 postive
APLP2	AC112220.2	0.339239942	6.23E-15 postive
APLP2	AC114271.1	0.320716539	2.00E-13 postive
APLP2	AC114490.1	0.356299634	2.06E-16 postive
APLP2	AC115618.2	0.437308694	9.04E-25 postive
APLP2	AC121338.2	0.359070953	1.16E-16 postive
APLP2	AC122129.1	0.355341095	2.51E-16 postive
APLP2	AC123912.1	0.397003804	2.53E-20 postive
APLP2	AC125257.1	0.386889778	2.67E-19 postive
APLP2	AC126755.1	0.324418344	1.02E-13 postive
APLP2	AC126773.4	0.305593963	2.87E-12 postive
APLP2	AC127521.1	0.380667404	1.09E-18 postive
APLP2	AC135050.5	0.338026807	7.88E-15 postive
APLP2	AC135050.6	0.363280746	4.81E-17 postive
APLP2	AC138207.1	0.309786032	1.39E-12 postive
APLP2	AC138207.4	0.307348577	2.12E-12 postive
APLP2	AC138207.5	0.36131035	7.28E-17 postive
APLP2	AC138207.6	0.310192933	1.30E-12 postive
APLP2	AC138207.8	0.545989505	3.43E-40 postive
APLP2	AC138932.1	0.365644706	2.91E-17 postive
APLP2	AC138956.2	0.310534749	1.22E-12 postive
APLP2	AC139795.2	0.35089547	6.21E-16 postive
APLP2	AC141586.1	0.442072237	2.45E-25 postive
APLP2	AC145124.1	0.345388345	1.87E-15 postive
APLP2	AC226118.1	0.441809864	2.64E-25 postive
APLP2	AC241952.2	0.381895635	8.30E-19 postive
APLP2	AC242426.2	0.307431327	2.09E-12 postive
APLP2	AC254562.3	0.356073887	2.16E-16 postive
APLP2	ADGRE4P	0.317412943	3.63E-13 postive
APLP2	ADGRF5P1	0.473151914	2.94E-29 postive
APLP2	AF111167.2	0.47969292	3.90E-30 postive
APLP2	AFAP1-AS1	0.339273115	6.19E-15 postive
APLP2	AL020996.1	0.355697157	2.33E-16 postive
APLP2	AL021707.3	0.319852156	2.34E-13 postive

APLP2	AL022476.1	0.318417854	3.03E-13	postive
APLP2	AL031282.1	0.380327413	1.18E-18	postive
APLP2	AL031282.2	0.363628039	4.47E-17	postive
APLP2	AL031714.1	0.317336093	3.68E-13	postive
APLP2	AL031717.1	0.338867078	6.70E-15	postive
APLP2	AL034550.1	0.314781982	5.80E-13	postive
APLP2	AL035587.1	0.331659506	2.65E-14	postive
APLP2	AL035661.1	0.345828971	1.71E-15	postive
APLP2	AL049597.2	0.3144813	6.12E-13	postive
APLP2	AL049840.4	0.394249333	4.84E-20	postive
APLP2	AL049840.5	0.365560625	2.97E-17	postive
APLP2	AL050309.1	0.341886985	3.72E-15	postive
APLP2	AL078581.2	0.454800527	6.80E-27	postive
APLP2	AL078644.2	0.403851247	4.88E-21	postive
APLP2	AL109936.3	0.369429929	1.29E-17	postive
APLP2	AL118506.1	0.387773749	2.18E-19	postive
APLP2	AL121772.3	0.331935022	2.52E-14	postive
APLP2	AL121895.2	0.331417762	2.78E-14	postive
APLP2	AL122035.1	0.365539444	2.98E-17	postive
APLP2	AL132657.1	0.369344222	1.32E-17	postive
APLP2	AL133355.1	0.428066042	1.07E-23	postive
APLP2	AL133371.2	0.321012192	1.90E-13	postive
APLP2	AL133406.2	0.305234752	3.05E-12	postive
APLP2	AL135818.1	0.35050859	6.71E-16	postive
APLP2	AL135818.2	0.327486912	5.79E-14	postive
APLP2	AL137003.1	0.429126768	8.09E-24	postive
APLP2	AL137026.1	0.370294913	1.07E-17	postive
APLP2	AL137058.2	0.321045802	1.89E-13	postive
APLP2	AL138756.1	0.370608946	1.00E-17	postive
APLP2	AL139011.1	0.308939778	1.61E-12	postive
APLP2	AL157394.1	0.303945228	3.80E-12	postive
APLP2	AL157700.1	0.325000649	9.17E-14	postive
APLP2	AL157895.2	0.324516697	1.00E-13	postive
APLP2	AL160006.1	0.322867355	1.36E-13	postive
APLP2	AL161785.1	0.375612299	3.36E-18	postive
APLP2	AL162231.2	0.362083992	6.19E-17	postive
APLP2	AL162431.1	0.326573697	6.86E-14	postive
APLP2	AL162595.1	0.362036568	6.25E-17	postive
APLP2	AL162727.2	0.312995043	7.95E-13	postive
APLP2	AL353593.1	0.506727768	5.69E-34	postive
APLP2	AL356235.1	0.360454285	8.71E-17	postive
APLP2	AL356481.1	0.326938444	6.41E-14	postive
APLP2	AL356599.1	0.306125152	2.62E-12	postive
APLP2	AL357033.3	0.385171819	3.95E-19	postive
APLP2	AL357033.4	0.357828737	1.50E-16	postive
APLP2	AL360181.2	0.373979983	4.82E-18	postive
APLP2	AL365330.1	0.332346329	2.33E-14	postive
APLP2	AL391121.1	0.386684476	2.80E-19	postive
APLP2	AL391832.3	0.317468393	3.60E-13	postive
APLP2	AL391834.2	0.32781715	5.45E-14	postive

APLP2	AL445423.1	0.307089893	2.22E-12	postive
APLP2	AL450326.1	0.454676863	7.04E-27	postive
APLP2	AL451042.1	0.390764964	1.09E-19	postive
APLP2	AL451042.2	0.462873017	6.46E-28	postive
APLP2	AL513477.1	0.32764197	5.63E-14	postive
APLP2	AL589765.4	0.345060595	1.99E-15	postive
APLP2	AL589765.5	0.316488961	4.28E-13	postive
APLP2	AL591895.1	0.371026759	9.17E-18	postive
APLP2	AL603839.2	0.309166309	1.55E-12	postive
APLP2	AL604028.1	0.358311632	1.36E-16	postive
APLP2	AL627309.5	0.311300152	1.07E-12	postive
APLP2	AL645728.1	0.326584502	6.85E-14	postive
APLP2	AL669831.1	0.402428207	6.89E-21	postive
APLP2	AL671710.1	0.301168242	6.08E-12	postive
APLP2	AL691432.2	0.411640275	7.19E-22	postive
APLP2	AL691447.2	0.324383213	1.03E-13	postive
APLP2	AL731577.2	0.400398196	1.12E-20	postive
APLP2	AL732314.8	0.321666759	1.69E-13	postive
APLP2	ALOX12-AS1	0.345282614	1.91E-15	postive
APLP2	AMZ2P1	0.385564383	3.61E-19	postive
APLP2	ANO7L1	0.388444991	1.87E-19	postive
APLP2	AOC4P	0.376212138	2.95E-18	postive
APLP2	AP000254.2	0.350111305	7.27E-16	postive
APLP2	AP000295.10	0.321849681	1.63E-13	postive
APLP2	AP000317.1	0.334395788	1.58E-14	postive
APLP2	AP000347.1	0.336460131	1.07E-14	postive
APLP2	AP000439.1	0.484344245	9.01E-31	postive
APLP2	AP000442.1	0.368503684	1.58E-17	postive
APLP2	AP000759.1	0.353768672	3.46E-16	postive
APLP2	AP000766.1	0.355034436	2.67E-16	postive
APLP2	AP000808.1	0.360060458	9.46E-17	postive
APLP2	AP000866.1	0.445399109	9.75E-26	postive
APLP2	AP000866.2	0.440425439	3.86E-25	postive
APLP2	AP000866.5	0.366971614	2.20E-17	postive
APLP2	AP001107.1	0.413953999	4.03E-22	postive
APLP2	AP001107.9	0.305088947	3.13E-12	postive
APLP2	AP001160.3	0.334668887	1.50E-14	postive
APLP2	AP001267.3	0.365169315	3.22E-17	postive
APLP2	AP001271.1	0.364416527	3.78E-17	postive
APLP2	AP001318.2	0.396010428	3.19E-20	postive
APLP2	AP001486.2	0.329423672	4.04E-14	postive
APLP2	AP001816.1	0.320762364	1.99E-13	postive
APLP2	AP001922.5	0.313186929	7.69E-13	postive
APLP2	AP001922.6	0.320134455	2.23E-13	postive
APLP2	AP002761.3	0.305568053	2.88E-12	postive
APLP2	AP002761.4	0.318400787	3.04E-13	postive
APLP2	AP002840.2	0.499809958	5.88E-33	postive
APLP2	AP003068.2	0.411374063	7.68E-22	postive
APLP2	AP003392.1	0.371210324	8.81E-18	postive
APLP2	AP003392.4	0.332417273	2.30E-14	postive

APLP2	AP003392.5	0.363600501	4.50E-17	postive
APLP2	AP003486.1	0.501804852	3.01E-33	postive
APLP2	AP003498.1	0.36066976	8.33E-17	postive
APLP2	AP005717.1	0.311670093	1.00E-12	postive
APLP2	AP006623.1	0.305947708	2.70E-12	postive
APLP2	APTR	0.429976845	6.46E-24	postive
APLP2	ARHGAP27P1-BPTFP1-KPNA2P3	0.396391545	2.92E-20	postive
APLP2	ARHGEF26-AS1	0.310997207	1.13E-12	postive
APLP2	ARMCX5-GPRASP2	0.39006671	1.28E-19	postive
APLP2	ASB16-AS1	0.373372251	5.50E-18	postive
APLP2	ATXN1-AS1	0.434780559	1.79E-24	postive
APLP2	BACE1-AS	0.348639096	9.78E-16	postive
APLP2	BAIAP2-DT	0.595550881	2.57E-49	postive
APLP2	BDNF-AS	0.442406005	2.24E-25	postive
APLP2	BMS1P20	0.391819957	8.54E-20	postive
APLP2	BTN2A3P	0.321068542	1.88E-13	postive
APLP2	BX322557.10	0.375435665	3.50E-18	postive
APLP2	BX537318.1	0.302996525	4.47E-12	postive
APLP2	C15orf56	0.386692147	2.79E-19	postive
APLP2	C21orf62-AS1	0.401856167	7.91E-21	postive
APLP2	C22orf46	0.484702675	8.04E-31	postive
APLP2	CA5BP1	0.355622929	2.37E-16	postive
APLP2	CBR3-AS1	0.330529724	3.28E-14	postive
APLP2	CCDC84-DT	0.322522939	1.44E-13	postive
APLP2	CCNT2-AS1	0.376838155	2.57E-18	postive
APLP2	CCT6P3	0.358175913	1.40E-16	postive
APLP2	CD2BP2-DT	0.314383479	6.22E-13	postive
APLP2	CD81-AS1	0.304122389	3.69E-12	postive
APLP2	CEBPA-DT	0.312501688	8.67E-13	postive
APLP2	CENPBD1P1	0.42214134	5.01E-23	postive
APLP2	CFL1P1	0.356107165	2.15E-16	postive
APLP2	CKMT2-AS1	0.38475093	4.35E-19	postive
APLP2	CLUHP3	0.329030919	4.35E-14	postive
APLP2	COX10-AS1	0.436881855	1.01E-24	postive
APLP2	CRNDE	0.319733403	2.39E-13	postive
APLP2	CROCCP3	0.301177798	6.07E-12	postive
APLP2	CTBP1-DT	0.43043655	5.72E-24	postive
APLP2	CTC-487M23.5	0.344673935	2.15E-15	postive
APLP2	CTD-2245F17.3	0.478893166	5.00E-30	postive
APLP2	CTD-2554C21.3	0.303963475	3.79E-12	postive
APLP2	CUTALP	0.335666719	1.24E-14	postive
APLP2	DGCR11	0.386333292	3.03E-19	postive
APLP2	DNMBP-AS1	0.375516279	3.44E-18	postive
APLP2	DOCK9-DT	0.433184567	2.75E-24	postive
APLP2	DPP4-DT	0.364739589	3.53E-17	postive
APLP2	DPP9-AS1	0.301883357	5.39E-12	postive
APLP2	DSTNP2	0.485430469	6.38E-31	postive
APLP2	EBLN3P	0.34122726	4.24E-15	postive
APLP2	EIF1B-AS1	0.337677053	8.43E-15	postive

APLP2	EIF3J-DT	0.417200551	1.78E-22	postive
APLP2	ELOA-AS1	0.325157686	8.91E-14	postive
APLP2	EMC1-AS1	0.357313715	1.67E-16	postive
APLP2	ENTPD3-AS1	0.320544077	2.07E-13	postive
APLP2	EP300-AS1	0.385739961	3.47E-19	postive
APLP2	EPB41L4A-DT	0.496077005	2.03E-32	postive
APLP2	ERVE-1	0.42273264	4.30E-23	postive
APLP2	ERVK13-1	0.330683028	3.19E-14	postive
APLP2	FAHD2CP	0.350361683	6.92E-16	postive
APLP2	FAM111A-DT	0.458470722	2.35E-27	postive
APLP2	FAM157B	0.306769264	2.35E-12	postive
APLP2	FAM157C	0.344121057	2.40E-15	postive
APLP2	FAM160A1-DT	0.437844219	7.81E-25	postive
APLP2	FAM66B	0.336541851	1.05E-14	postive
APLP2	FAM86C2P	0.359448365	1.07E-16	postive
APLP2	FAM86DP	0.425559895	2.06E-23	postive
APLP2	FAM86EP	0.36455149	3.68E-17	postive
APLP2	FGD5-AS1	0.414190366	3.80E-22	postive
APLP2	FGF7P3	0.336675602	1.02E-14	postive
APLP2	FP325335.1	0.383699581	5.52E-19	postive
APLP2	FRG1HP	0.338472029	7.23E-15	postive
APLP2	GGT3P	0.305685135	2.83E-12	postive
APLP2	GGTA1P	0.348856195	9.36E-16	postive
APLP2	GHET1	0.315632206	4.99E-13	postive
APLP2	GNRHR2	0.39662881	2.76E-20	postive
APLP2	GOLGA2P10	0.333769098	1.78E-14	postive
APLP2	GS1-124K5.3	0.327454291	5.83E-14	postive
APLP2	GS1-358P8.4	0.381143556	9.83E-19	postive
APLP2	GTF2IP1	0.450188517	2.54E-26	postive
APLP2	GTF2IP4	0.506931638	5.31E-34	postive
APLP2	GUSBP1	0.393601047	5.63E-20	postive
APLP2	HAGLR	0.546764517	2.54E-40	postive
APLP2	HCG11	0.37261928	6.48E-18	postive
APLP2	HCG18	0.344165765	2.38E-15	postive
APLP2	HEIH	0.347690935	1.18E-15	postive
APLP2	HLA-DRB6	0.325697784	8.07E-14	postive
APLP2	HLX-AS1	0.301497173	5.75E-12	postive
APLP2	IL10RB-DT	0.474278545	2.08E-29	postive
APLP2	IL6R-AS1	0.364590754	3.65E-17	postive
APLP2	ITGA9-AS1	0.465220809	3.22E-28	postive
APLP2	JPX	0.311908445	9.62E-13	postive
APLP2	KIAA1671-AS1	0.371059183	9.10E-18	postive
APLP2	KRTAP5-AS1	0.309615926	1.44E-12	postive
APLP2	L3MBTL2-AS1	0.414939848	3.15E-22	postive
APLP2	LINC00205	0.30416828	3.66E-12	postive
APLP2	LINC00294	0.353948146	3.34E-16	postive
APLP2	LINC00339	0.394069862	5.05E-20	postive
APLP2	LINC00398	0.308126809	1.86E-12	postive
APLP2	LINC00467	0.316393905	4.36E-13	postive
APLP2	LINC00571	0.31097365	1.13E-12	postive

APLP2	LINC00638	0.317555839	3.54E-13	postive
APLP2	LINC00663	0.444572365	1.23E-25	postive
APLP2	LINC00667	0.390298034	1.22E-19	postive
APLP2	LINC00674	0.556521734	5.29E-42	postive
APLP2	LINC00863	0.337616672	8.53E-15	postive
APLP2	LINC00886	0.303729871	3.94E-12	postive
APLP2	LINC00899	0.325595275	8.22E-14	postive
APLP2	LINC00909	0.300267401	7.07E-12	postive
APLP2	LINC00910	0.43399433	2.21E-24	postive
APLP2	LINC00921	0.363522201	4.57E-17	postive
APLP2	LINC00933	0.331018832	2.99E-14	postive
APLP2	LINC00957	0.430267688	5.98E-24	postive
APLP2	LINC00997	0.359525252	1.06E-16	postive
APLP2	LINC01001	0.30293232	4.51E-12	postive
APLP2	LINC01011	0.336861539	9.86E-15	postive
APLP2	LINC01128	0.456572747	4.08E-27	postive
APLP2	LINC01135	0.324428034	1.02E-13	postive
APLP2	LINC01150	0.37615929	2.98E-18	postive
APLP2	LINC01184	0.453095043	1.11E-26	postive
APLP2	LINC01278	0.456408584	4.27E-27	postive
APLP2	LINC01336	0.300861641	6.40E-12	postive
APLP2	LINC01352	0.333433219	1.90E-14	postive
APLP2	LINC01426	0.445534044	9.39E-26	postive
APLP2	LINC01547	0.332438738	2.29E-14	postive
APLP2	LINC01558	0.347330563	1.27E-15	postive
APLP2	LINC01578	0.428911958	8.56E-24	postive
APLP2	LINC01772	0.341911123	3.71E-15	postive
APLP2	LINC01783	0.330055674	3.59E-14	postive
APLP2	LINC01852	0.523426335	1.62E-36	postive
APLP2	LINC01881	0.364419463	3.78E-17	postive
APLP2	LINC01948	0.338690799	6.93E-15	postive
APLP2	LINC02035	0.383938977	5.23E-19	postive
APLP2	LINC02038	0.393393236	5.91E-20	postive
APLP2	LINC02210	0.365985056	2.71E-17	postive
APLP2	LINC02256	0.320188351	2.21E-13	postive
APLP2	LINC02361	0.341690728	3.87E-15	postive
APLP2	LINC02416	0.330676271	3.19E-14	postive
APLP2	LINC02519	0.34314307	2.91E-15	postive
APLP2	LINC02569	0.34535589	1.88E-15	postive
APLP2	LINC02777	0.301143719	6.10E-12	postive
APLP2	LINC02861	0.37313901	5.79E-18	postive
APLP2	LPAL2	0.313317003	7.51E-13	postive
APLP2	LPP-AS2	0.300512301	6.79E-12	postive
APLP2	LRRC37A16P	0.499924727	5.66E-33	postive
APLP2	LRRC37A17P	0.44493264	1.11E-25	postive
APLP2	LRRC37BP1	0.530748165	1.11E-37	postive
APLP2	LRRK2-DT	0.300765503	6.50E-12	postive
APLP2	MAP2K4P1	0.36784624	1.82E-17	postive
APLP2	MAP4K3-DT	0.362482177	5.69E-17	postive
APLP2	MATN1-AS1	0.311391891	1.05E-12	postive

APLP2	MED14OS	0.341081249	4.36E-15	postive
APLP2	MID1IP1-AS1	0.348834052	9.40E-16	postive
APLP2	MIR22HG	0.357455099	1.62E-16	postive
APLP2	MKLN1-AS	0.368011689	1.76E-17	postive
APLP2	MRPL20-AS1	0.454195659	8.09E-27	postive
APLP2	MRPS31P4	0.33626763	1.11E-14	postive
APLP2	NAPSB	0.305133801	3.10E-12	postive
APLP2	NARF-AS1	0.311022587	1.12E-12	postive
APLP2	NBPF25P	0.491377574	9.44E-32	postive
APLP2	NBPF8	0.334041341	1.69E-14	postive
APLP2	NBR2	0.361581431	6.88E-17	postive
APLP2	NCMAP-DT	0.328471429	4.82E-14	postive
APLP2	NDUFA6-DT	0.425877454	1.90E-23	postive
APLP2	NIFK-AS1	0.390235171	1.23E-19	postive
APLP2	NINJ2-AS1	0.317327029	3.69E-13	postive
APLP2	NKAPP1	0.314319963	6.29E-13	postive
APLP2	NKX2-1-AS1	0.398702277	1.69E-20	postive
APLP2	NOP14-AS1	0.456531708	4.13E-27	postive
APLP2	NOP53-AS1	0.357458696	1.62E-16	postive
APLP2	NORAD	0.497793092	1.15E-32	postive
APLP2	NPEPPSP1	0.30415403	3.67E-12	postive
APLP2	NR2F1-AS1	0.309932684	1.36E-12	postive
APLP2	NUDT16P1	0.346901131	1.38E-15	postive
APLP2	OGFRP1	0.326895144	6.47E-14	postive
APLP2	OIP5-AS1	0.351424431	5.58E-16	postive
APLP2	OTUD6B-AS1	0.319406944	2.54E-13	postive
APLP2	PAXBP1-AS1	0.31860245	2.93E-13	postive
APLP2	PAXIP1-AS2	0.557987502	2.93E-42	postive
APLP2	PDIA3P1	0.309093321	1.57E-12	postive
APLP2	PGAM1P8	0.328112128	5.16E-14	postive
APLP2	PICART1	0.386894685	2.67E-19	postive
APLP2	PIK3CD-AS2	0.313969127	6.70E-13	postive
APLP2	PINK1-AS	0.465163634	3.27E-28	postive
APLP2	PKD1P6	0.33670194	1.02E-14	postive
APLP2	PLBD1-AS1	0.442673077	2.08E-25	postive
APLP2	PLEKHM1P1	0.501533102	3.30E-33	postive
APLP2	PMS2CL	0.327536038	5.74E-14	postive
APLP2	PMS2P4	0.431565594	4.23E-24	postive
APLP2	POLR2J4	0.341046075	4.39E-15	postive
APLP2	PPP3CB-AS1	0.365594955	2.95E-17	postive
APLP2	PRDM16-DT	0.306159064	2.61E-12	postive
APLP2	PRKAR2A-AS1	0.352944493	4.10E-16	postive
APLP2	PSMA3-AS1	0.302082584	5.21E-12	postive
APLP2	PSMG3-AS1	0.307470459	2.08E-12	postive
APLP2	PTENP1	0.359514768	1.06E-16	postive
APLP2	PTOV1-AS1	0.343496039	2.72E-15	postive
APLP2	RAP2C-AS1	0.328294383	4.99E-14	postive
APLP2	RARA-AS1	0.403714348	5.05E-21	postive
APLP2	RASA4CP	0.310217852	1.29E-12	postive
APLP2	RNF126P1	0.310306548	1.27E-12	postive

APLP2	RNF157-AS1	0.331229131	2.88E-14	postive
APLP2	RNF216P1	0.391213959	9.84E-20	postive
APLP2	RNFT1-DT	0.33388108	1.74E-14	postive
APLP2	RP11-101E13.5	0.320953642	1.92E-13	postive
APLP2	RP11-10C24.1	0.333978979	1.71E-14	postive
APLP2	RP11-159G9.5	0.390897025	1.06E-19	postive
APLP2	RP11-164P12.4	0.475792528	1.31E-29	postive
APLP2	RP11-175O19.4	0.384879391	4.22E-19	postive
APLP2	RP11-206L10.5	0.323154188	1.29E-13	postive
APLP2	RP11-226L15.5	0.309948491	1.35E-12	postive
APLP2	RP1-122P22.2	0.421027879	6.68E-23	postive
APLP2	RP11-282O18.3	0.356110391	2.14E-16	postive
APLP2	RP11-337C18.9	0.368406692	1.61E-17	postive
APLP2	RP11-345J18.2	0.335570649	1.26E-14	postive
APLP2	RP11-462G12.2	0.384208723	4.92E-19	postive
APLP2	RP11-539I5.1	0.379204401	1.52E-18	postive
APLP2	RP11-876N24.4	0.361802503	6.57E-17	postive
APLP2	RP11-876N24.5	0.342936744	3.03E-15	postive
APLP2	RP13-36G14.4	0.345553652	1.81E-15	postive
APLP2	RP5-1042I8.7	0.482826798	1.46E-30	postive
APLP2	RP5-1125A11.6	0.327059952	6.27E-14	postive
APLP2	RP5-1139B12.2	0.505533973	8.55E-34	postive
APLP2	RPARP-AS1	0.384151354	4.98E-19	postive
APLP2	RPL13P5	0.491713791	8.46E-32	postive
APLP2	RPL23AP53	0.397388948	2.30E-20	postive
APLP2	RPL23AP7	0.401675972	8.27E-21	postive
APLP2	RPL23AP82	0.389993154	1.31E-19	postive
APLP2	RPS10P7	0.352332923	4.64E-16	postive
APLP2	RPS18P9	0.313802161	6.90E-13	postive
APLP2	RRN3P3	0.400338878	1.14E-20	postive
APLP2	RRP7BP	0.326917926	6.44E-14	postive
APLP2	RTCA-AS1	0.3397247	5.68E-15	postive
APLP2	SAP30L-AS1	0.311445989	1.04E-12	postive
APLP2	SBDSP1	0.411482341	7.48E-22	postive
APLP2	SCAND2P	0.435430515	1.50E-24	postive
APLP2	SDAD1P1	0.428865427	8.66E-24	postive
APLP2	SEC22B4P	0.345357104	1.88E-15	postive
APLP2	SEC24B-AS1	0.327909274	5.36E-14	postive
APLP2	SEPTIN7-AS1	0.358838546	1.22E-16	postive
APLP2	SEPTIN9-DT	0.434931036	1.72E-24	postive
APLP2	SERTAD4-AS1	0.394293544	4.79E-20	postive
APLP2	SFTA1P	0.356016257	2.19E-16	postive
APLP2	SGMS1-AS1	0.351711763	5.26E-16	postive
APLP2	SH3GL1P1	0.309584328	1.44E-12	postive
APLP2	SIGLEC17P	0.362780243	5.35E-17	postive
APLP2	SIGLEC22P	0.384804702	4.30E-19	postive
APLP2	SLC44A3-AS1	0.361999864	6.30E-17	postive
APLP2	SLC9A7P1	0.300573155	6.72E-12	postive
APLP2	SMG7-AS1	0.338859821	6.71E-15	postive
APLP2	SMIM15-AS1	0.346711632	1.44E-15	postive

APLP2	SNAI3-AS1	0.339273913	6.19E-15	postive
APLP2	SNHG16	0.308634813	1.70E-12	postive
APLP2	SNHG31	0.359463079	1.07E-16	postive
APLP2	SP2-AS1	0.344361166	2.29E-15	postive
APLP2	SSR4P1	0.30373445	3.94E-12	postive
APLP2	ST20-AS1	0.393905946	5.24E-20	postive
APLP2	ST3GAL5-AS1	0.417908323	1.48E-22	postive
APLP2	STAG3L2	0.331534237	2.72E-14	postive
APLP2	STAG3L3	0.327630518	5.64E-14	postive
APLP2	SVIL2P	0.345577761	1.80E-15	postive
APLP2	SVIL-AS1	0.402998725	6.01E-21	postive
APLP2	SYNPR-AS1	0.356585528	1.94E-16	postive
APLP2	TBX5-AS1	0.382746567	6.85E-19	postive
APLP2	THAP9-AS1	0.302434079	4.91E-12	postive
APLP2	TMEM99	0.318951164	2.76E-13	postive
APLP2	TMEM9B-AS1	0.314743817	5.84E-13	postive
APLP2	TNFRSF10A-AS1	0.32477445	9.56E-14	postive
APLP2	TNRC6C-AS1	0.334251968	1.62E-14	postive
APLP2	TP53TG1	0.302439031	4.91E-12	postive
APLP2	TP73-AS1	0.537942227	7.55E-39	postive
APLP2	TRAM2-AS1	0.345575205	1.80E-15	postive
APLP2	TSTD3	0.32372531	1.16E-13	postive
APLP2	TTC28-AS1	0.37563468	3.35E-18	postive
APLP2	TUG1	0.412094597	6.42E-22	postive
APLP2	UBA6-AS1	0.335307375	1.33E-14	postive
APLP2	USP27X-AS1	0.380763224	1.07E-18	postive
APLP2	WAC-AS1	0.37102825	9.16E-18	postive
APLP2	WARS2-AS1	0.335367775	1.31E-14	postive
APLP2	WASH3P	0.304314975	3.57E-12	postive
APLP2	WDFY3-AS2	0.47102942	5.61E-29	postive
APLP2	WNT5A-AS1	0.377984814	1.99E-18	postive
APLP2	WWC2-AS2	0.348144531	1.08E-15	postive
APLP2	Z92544.2	0.30945211	1.48E-12	postive
APLP2	Z98884.1	0.348963181	9.16E-16	postive
APLP2	ZBED5-AS1	0.320177853	2.21E-13	postive
APLP2	ZBTB11-AS1	0.444606841	1.22E-25	postive
APLP2	ZBTB44-DT	0.461044803	1.11E-27	postive
APLP2	ZNF137P	0.354896026	2.75E-16	postive
APLP2	ZNF205-AS1	0.344095442	2.41E-15	postive
APLP2	ZNF252P	0.352254419	4.72E-16	postive
APLP2	ZNF321P	0.309780352	1.39E-12	postive
APLP2	ZNF542P	0.339706453	5.70E-15	postive
APLP2	ZNF56	0.378058845	1.96E-18	postive
APLP2	ZNF561-AS1	0.322259908	1.51E-13	postive
APLP2	ZNF658B	0.36197521	6.33E-17	postive
APLP2	ZNF702P	0.422686073	4.35E-23	postive
APLP2	ZNF788P	0.342767269	3.13E-15	postive
APLP2	ZNF815P	0.382556756	7.15E-19	postive
APLP2	ZNRD2-AS1	0.313157705	7.73E-13	postive
APLP2	ZNRF2P1	0.324632764	9.81E-14	postive

APOA4	AC006369.1	0.307999413	1.90E-12	postive
APOA4	AC007218.1	0.336399976	1.08E-14	postive
APOA4	AC051649.1	0.313814775	6.88E-13	postive
APOA4	AC122138.1	0.3318854	2.54E-14	postive
APOA4	AL034346.1	0.300293595	7.04E-12	postive
APOA4	AP002833.1	0.337510882	8.71E-15	postive
APOA4	KIRREL3-AS2	0.369535846	1.27E-17	postive
APOA4	LINC00824	0.310542187	1.22E-12	postive
APOA4	LINC01243	0.311036061	1.12E-12	postive
APOA4	LINC01485	0.30372864	3.94E-12	postive
APOA4	LINC01516	0.303410211	4.16E-12	postive
APOA4	MIR124-2HG	0.342171655	3.52E-15	postive
APOA4	Z82249.1	0.369465315	1.29E-17	postive
ATG9A	A2M-AS1	0.300600144	6.69E-12	postive
ATG9A	ABALON	0.36991654	1.17E-17	postive
ATG9A	AC000068.1	0.339390037	6.06E-15	postive
ATG9A	AC000068.2	0.36996483	1.15E-17	postive
ATG9A	AC000403.1	0.333376734	1.92E-14	postive
ATG9A	AC002310.1	0.321872862	1.63E-13	postive
ATG9A	AC002398.1	0.392922154	6.60E-20	postive
ATG9A	AC002467.1	0.40749392	2.01E-21	postive
ATG9A	AC002550.2	0.302862248	4.57E-12	postive
ATG9A	AC003070.1	0.31289847	8.09E-13	postive
ATG9A	AC004156.1	0.300594586	6.69E-12	postive
ATG9A	AC004221.1	0.300525096	6.77E-12	postive
ATG9A	AC004241.1	0.366826347	2.26E-17	postive
ATG9A	AC004241.2	0.325590631	8.23E-14	postive
ATG9A	AC004241.3	0.32784179	5.42E-14	postive
ATG9A	AC004447.2	0.419478086	9.94E-23	postive
ATG9A	AC004466.1	0.330134465	3.54E-14	postive
ATG9A	AC004471.2	0.321336741	1.79E-13	postive
ATG9A	AC004477.3	0.40863963	1.51E-21	postive
ATG9A	AC004596.1	0.48612545	5.11E-31	postive
ATG9A	AC004707.2	0.303521664	4.08E-12	postive
ATG9A	AC004771.2	0.415675858	2.61E-22	postive
ATG9A	AC004771.4	0.329503656	3.98E-14	postive
ATG9A	AC004801.6	0.467435836	1.66E-28	postive
ATG9A	AC004803.1	0.417341021	1.71E-22	postive
ATG9A	AC004812.2	0.417811528	1.52E-22	postive
ATG9A	AC004846.1	0.342184438	3.51E-15	postive
ATG9A	AC004893.11	0.437508979	8.56E-25	postive
ATG9A	AC004918.1	0.311143455	1.10E-12	postive
ATG9A	AC004918.5	0.301459398	5.79E-12	postive
ATG9A	AC004943.2	0.475081341	1.63E-29	postive
ATG9A	AC004951.1	0.352507841	4.48E-16	postive
ATG9A	AC004980.1	0.327436725	5.85E-14	postive
ATG9A	AC005005.3	0.344026972	2.45E-15	postive
ATG9A	AC005034.4	0.617524365	6.81E-54	postive
ATG9A	AC005034.6	0.501404683	3.45E-33	postive
ATG9A	AC005037.1	0.307868219	1.94E-12	postive

ATG9A	AC005041.3	0.430733381	5.28E-24	postive
ATG9A	AC005229.4	0.385171216	3.95E-19	postive
ATG9A	AC005253.1	0.348502764	1.01E-15	postive
ATG9A	AC005261.2	0.386383245	3.00E-19	postive
ATG9A	AC005261.3	0.337741815	8.33E-15	postive
ATG9A	AC005261.4	0.45311604	1.10E-26	postive
ATG9A	AC005288.1	0.598349382	7.03E-50	postive
ATG9A	AC005324.5	0.32808135	5.19E-14	postive
ATG9A	AC005332.4	0.314448048	6.15E-13	postive
ATG9A	AC005332.6	0.399788853	1.30E-20	postive
ATG9A	AC005387.1	0.38899821	1.64E-19	postive
ATG9A	AC005387.2	0.334622006	1.51E-14	postive
ATG9A	AC005498.3	0.311413137	1.05E-12	postive
ATG9A	AC005562.1	0.581897338	1.21E-46	postive
ATG9A	AC005586.1	0.380777481	1.07E-18	postive
ATG9A	AC005670.3	0.525448046	7.78E-37	postive
ATG9A	AC005696.1	0.376373612	2.84E-18	postive
ATG9A	AC005840.2	0.313885726	6.80E-13	postive
ATG9A	AC005840.4	0.311404295	1.05E-12	postive
ATG9A	AC006001.2	0.402855565	6.22E-21	postive
ATG9A	AC006064.2	0.322752623	1.38E-13	postive
ATG9A	AC006064.3	0.345363943	1.88E-15	postive
ATG9A	AC006126.4	0.441627741	2.77E-25	postive
ATG9A	AC006206.2	0.317757706	3.41E-13	postive
ATG9A	AC006213.1	0.364066295	4.07E-17	postive
ATG9A	AC006213.2	0.326023066	7.60E-14	postive
ATG9A	AC006213.4	0.334503524	1.55E-14	postive
ATG9A	AC006213.5	0.30498062	3.19E-12	postive
ATG9A	AC006252.1	0.379790925	1.33E-18	postive
ATG9A	AC006333.1	0.368272986	1.66E-17	postive
ATG9A	AC006449.2	0.438658819	6.26E-25	postive
ATG9A	AC006449.7	0.395696548	3.44E-20	postive
ATG9A	AC006504.5	0.372766886	6.28E-18	postive
ATG9A	AC006547.1	0.463715705	5.03E-28	postive
ATG9A	AC007032.1	0.301434662	5.81E-12	postive
ATG9A	AC007066.2	0.326862899	6.50E-14	postive
ATG9A	AC007228.9	0.413107523	4.98E-22	postive
ATG9A	AC007292.1	0.346107215	1.62E-15	postive
ATG9A	AC007292.2	0.360428234	8.76E-17	postive
ATG9A	AC007342.4	0.330583329	3.25E-14	postive
ATG9A	AC007383.2	0.548148034	1.48E-40	postive
ATG9A	AC007406.3	0.386726609	2.77E-19	postive
ATG9A	AC007406.5	0.507954189	3.74E-34	postive
ATG9A	AC007541.1	0.323700532	1.16E-13	postive
ATG9A	AC007663.4	0.340297029	5.08E-15	postive
ATG9A	AC008014.1	0.344642284	2.17E-15	postive
ATG9A	AC008105.1	0.362167207	6.08E-17	postive
ATG9A	AC008124.1	0.415832941	2.51E-22	postive
ATG9A	AC008280.3	0.357272967	1.69E-16	postive
ATG9A	AC008393.1	0.344070215	2.43E-15	postive

ATG9A	AC008438.1	0.433345122	2.63E-24	postive
ATG9A	AC008443.1	0.484725108	7.99E-31	postive
ATG9A	AC008494.3	0.346282877	1.57E-15	postive
ATG9A	AC008622.2	0.402690758	6.47E-21	postive
ATG9A	AC008735.1	0.342981223	3.00E-15	postive
ATG9A	AC008735.2	0.36487388	3.43E-17	postive
ATG9A	AC008735.4	0.373988177	4.81E-18	postive
ATG9A	AC008764.2	0.36628002	2.55E-17	postive
ATG9A	AC008771.1	0.327578758	5.70E-14	postive
ATG9A	AC008915.3	0.39341264	5.89E-20	postive
ATG9A	AC008966.1	0.328802165	4.54E-14	postive
ATG9A	AC009061.2	0.371465643	8.33E-18	postive
ATG9A	AC009065.3	0.361054125	7.68E-17	postive
ATG9A	AC009065.5	0.313300691	7.53E-13	postive
ATG9A	AC009065.6	0.343069756	2.95E-15	postive
ATG9A	AC009065.9	0.417585386	1.61E-22	postive
ATG9A	AC009113.1	0.390677796	1.11E-19	postive
ATG9A	AC009118.3	0.334229484	1.63E-14	postive
ATG9A	AC009126.1	0.457028642	3.57E-27	postive
ATG9A	AC009226.1	0.345124427	1.97E-15	postive
ATG9A	AC009237.14	0.500770945	4.26E-33	postive
ATG9A	AC009309.2	0.311176062	1.09E-12	postive
ATG9A	AC009318.2	0.382284202	7.60E-19	postive
ATG9A	AC009318.3	0.339372779	6.08E-15	postive
ATG9A	AC009404.1	0.341422228	4.08E-15	postive
ATG9A	AC009506.2	0.410809327	8.84E-22	postive
ATG9A	AC009690.2	0.381542042	8.98E-19	postive
ATG9A	AC009779.3	0.538958667	5.13E-39	postive
ATG9A	AC009831.1	0.505010348	1.02E-33	postive
ATG9A	AC009948.3	0.416446438	2.15E-22	postive
ATG9A	AC009961.1	0.366755342	2.30E-17	postive
ATG9A	AC010136.1	0.300336849	6.99E-12	postive
ATG9A	AC010271.1	0.337501947	8.72E-15	postive
ATG9A	AC010273.2	0.301687845	5.57E-12	postive
ATG9A	AC010319.4	0.376912479	2.52E-18	postive
ATG9A	AC010326.4	0.372953898	6.03E-18	postive
ATG9A	AC010326.5	0.364680648	3.58E-17	postive
ATG9A	AC010442.1	0.417434589	1.67E-22	postive
ATG9A	AC010503.5	0.508527197	3.07E-34	postive
ATG9A	AC010504.1	0.337304493	9.06E-15	postive
ATG9A	AC010618.3	0.326378377	7.11E-14	postive
ATG9A	AC010643.1	0.30837531	1.78E-12	postive
ATG9A	AC010680.2	0.373445534	5.41E-18	postive
ATG9A	AC010761.1	0.307630809	2.02E-12	postive
ATG9A	AC010761.4	0.380578501	1.12E-18	postive
ATG9A	AC010761.6	0.306358225	2.52E-12	postive
ATG9A	AC010809.1	0.409303275	1.28E-21	postive
ATG9A	AC010809.2	0.320884646	1.94E-13	postive
ATG9A	AC010864.1	0.30549565	2.92E-12	postive
ATG9A	AC010894.2	0.307631839	2.02E-12	postive

ATG9A	AC010969.2	0.474708594	1.83E-29	postive
ATG9A	AC010997.4	0.3273501	5.94E-14	postive
ATG9A	AC011450.1	0.326869142	6.50E-14	postive
ATG9A	AC011451.1	0.31569914	4.93E-13	postive
ATG9A	AC011472.1	0.377725583	2.11E-18	postive
ATG9A	AC011472.2	0.368260065	1.67E-17	postive
ATG9A	AC011476.3	0.384291091	4.83E-19	postive
ATG9A	AC011477.2	0.324970298	9.22E-14	postive
ATG9A	AC011498.6	0.332430713	2.29E-14	postive
ATG9A	AC011676.1	0.331711237	2.63E-14	postive
ATG9A	AC011731.1	0.344712527	2.14E-15	postive
ATG9A	AC011921.1	0.31571872	4.91E-13	postive
ATG9A	AC011978.2	0.375900341	3.16E-18	postive
ATG9A	AC012063.1	0.539226132	4.63E-39	postive
ATG9A	AC012073.1	0.421764791	5.52E-23	postive
ATG9A	AC012184.3	0.309242992	1.53E-12	postive
ATG9A	AC012306.2	0.52839566	2.65E-37	postive
ATG9A	AC012313.1	0.496066501	2.04E-32	postive
ATG9A	AC012313.2	0.319533589	2.48E-13	postive
ATG9A	AC012313.5	0.342436169	3.34E-15	postive
ATG9A	AC012313.8	0.346066797	1.63E-15	postive
ATG9A	AC012360.3	0.372702826	6.37E-18	postive
ATG9A	AC012442.2	0.404942971	3.75E-21	postive
ATG9A	AC012442.3	0.342894926	3.06E-15	postive
ATG9A	AC012467.2	0.473812597	2.40E-29	postive
ATG9A	AC012510.1	0.521571907	3.15E-36	postive
ATG9A	AC012603.1	0.397370455	2.31E-20	postive
ATG9A	AC012676.1	0.417636813	1.59E-22	postive
ATG9A	AC013356.2	0.306746111	2.36E-12	postive
ATG9A	AC015726.1	0.307119835	2.21E-12	postive
ATG9A	AC015802.4	0.31996744	2.30E-13	postive
ATG9A	AC015819.1	0.305701826	2.82E-12	postive
ATG9A	AC015871.3	0.332899087	2.10E-14	postive
ATG9A	AC015922.2	0.546707744	2.60E-40	postive
ATG9A	AC015922.3	0.481558628	2.17E-30	postive
ATG9A	AC016027.1	0.377115862	2.41E-18	postive
ATG9A	AC016065.1	0.432643781	3.18E-24	postive
ATG9A	AC016394.2	0.308118045	1.86E-12	postive
ATG9A	AC016722.2	0.322608327	1.42E-13	postive
ATG9A	AC016727.1	0.369141815	1.38E-17	postive
ATG9A	AC016747.1	0.389776181	1.37E-19	postive
ATG9A	AC016876.2	0.372404039	6.80E-18	postive
ATG9A	AC016876.3	0.409988666	1.08E-21	postive
ATG9A	AC016888.1	0.37888195	1.63E-18	postive
ATG9A	AC017074.1	0.365345487	3.11E-17	postive
ATG9A	AC018413.1	0.338599026	7.06E-15	postive
ATG9A	AC018521.5	0.360129827	9.32E-17	postive
ATG9A	AC018521.6	0.407148259	2.18E-21	postive
ATG9A	AC018645.3	0.341987974	3.65E-15	postive
ATG9A	AC018647.2	0.471689824	4.59E-29	postive

ATG9A	AC018653.3	0.412049296	6.49E-22	postive
ATG9A	AC018690.1	0.413343515	4.70E-22	postive
ATG9A	AC018766.4	0.349550841	8.14E-16	postive
ATG9A	AC018809.2	0.417080152	1.83E-22	postive
ATG9A	AC018904.1	0.360642044	8.37E-17	postive
ATG9A	AC019077.1	0.311834093	9.75E-13	postive
ATG9A	AC019131.2	0.317561081	3.54E-13	postive
ATG9A	AC019205.1	0.331005905	3.00E-14	postive
ATG9A	AC019294.2	0.312177663	9.18E-13	postive
ATG9A	AC020558.2	0.334487019	1.55E-14	postive
ATG9A	AC020661.1	0.377274801	2.33E-18	postive
ATG9A	AC020663.2	0.34286456	3.07E-15	postive
ATG9A	AC020904.2	0.311490494	1.04E-12	postive
ATG9A	AC020915.1	0.382289598	7.59E-19	postive
ATG9A	AC020915.2	0.312753355	8.30E-13	postive
ATG9A	AC020915.3	0.54190782	1.66E-39	postive
ATG9A	AC021016.2	0.372639723	6.46E-18	postive
ATG9A	AC021016.3	0.384889753	4.21E-19	postive
ATG9A	AC021148.1	0.356128471	2.14E-16	postive
ATG9A	AC021224.1	0.346126872	1.61E-15	postive
ATG9A	AC021483.2	0.319543706	2.48E-13	postive
ATG9A	AC022007.1	0.359070643	1.16E-16	postive
ATG9A	AC022098.1	0.433948381	2.24E-24	postive
ATG9A	AC022150.2	0.472223002	3.90E-29	postive
ATG9A	AC022167.2	0.396961777	2.55E-20	postive
ATG9A	AC022211.3	0.499159743	7.30E-33	postive
ATG9A	AC022306.2	0.322082035	1.56E-13	postive
ATG9A	AC022364.1	0.405174465	3.54E-21	postive
ATG9A	AC022400.1	0.363410854	4.68E-17	postive
ATG9A	AC022400.5	0.318986959	2.74E-13	postive
ATG9A	AC022613.2	0.452294675	1.39E-26	postive
ATG9A	AC022916.2	0.351997865	4.97E-16	postive
ATG9A	AC023024.1	0.39285596	6.71E-20	postive
ATG9A	AC023043.4	0.37769848	2.12E-18	postive
ATG9A	AC023509.1	0.302114811	5.18E-12	postive
ATG9A	AC023509.3	0.348112332	1.09E-15	postive
ATG9A	AC023790.2	0.311510676	1.03E-12	postive
ATG9A	AC024075.1	0.321440593	1.76E-13	postive
ATG9A	AC024075.2	0.33585771	1.20E-14	postive
ATG9A	AC024075.3	0.315575616	5.04E-13	postive
ATG9A	AC024267.4	0.317193892	3.78E-13	postive
ATG9A	AC024267.7	0.41871544	1.21E-22	postive
ATG9A	AC024575.2	0.362882182	5.23E-17	postive
ATG9A	AC024896.1	0.422462046	4.61E-23	postive
ATG9A	AC025048.2	0.3698783	1.18E-17	postive
ATG9A	AC025171.1	0.343150854	2.91E-15	postive
ATG9A	AC025181.2	0.362709475	5.43E-17	postive
ATG9A	AC025262.1	0.318391715	3.05E-13	postive
ATG9A	AC025580.2	0.308812939	1.65E-12	postive
ATG9A	AC025682.2	0.372240615	7.04E-18	postive

ATG9A	AC025857.2	0.32117508	1.84E-13	postive
ATG9A	AC026367.3	0.324431589	1.02E-13	postive
ATG9A	AC026369.2	0.360778113	8.14E-17	postive
ATG9A	AC026401.3	0.465802273	2.71E-28	postive
ATG9A	AC026471.1	0.438358365	6.79E-25	postive
ATG9A	AC026691.1	0.367886455	1.80E-17	postive
ATG9A	AC026803.2	0.344595895	2.19E-15	postive
ATG9A	AC027097.1	0.36577121	2.84E-17	postive
ATG9A	AC027237.5	0.367285014	2.05E-17	postive
ATG9A	AC027307.2	0.468626284	1.16E-28	postive
ATG9A	AC027601.1	0.377982348	1.99E-18	postive
ATG9A	AC027601.2	0.354863743	2.77E-16	postive
ATG9A	AC027644.3	0.309591126	1.44E-12	postive
ATG9A	AC027796.4	0.31290741	8.07E-13	postive
ATG9A	AC032044.1	0.30131474	5.93E-12	postive
ATG9A	AC034102.6	0.301970725	5.31E-12	postive
ATG9A	AC034236.2	0.327095328	6.23E-14	postive
ATG9A	AC036176.1	0.310610618	1.21E-12	postive
ATG9A	AC037459.2	0.424091813	3.02E-23	postive
ATG9A	AC040970.1	0.304856631	3.26E-12	postive
ATG9A	AC040977.1	0.396180198	3.07E-20	postive
ATG9A	AC046134.2	0.364354411	3.83E-17	postive
ATG9A	AC046143.2	0.325655199	8.13E-14	postive
ATG9A	AC048382.1	0.309336408	1.51E-12	postive
ATG9A	AC048382.2	0.318080859	3.22E-13	postive
ATG9A	AC048382.5	0.316871391	4.00E-13	postive
ATG9A	AC051619.6	0.35125227	5.78E-16	postive
ATG9A	AC053503.4	0.3828204	6.74E-19	postive
ATG9A	AC055720.2	0.350080444	7.32E-16	postive
ATG9A	AC060766.7	0.333527966	1.86E-14	postive
ATG9A	AC060780.1	0.451568659	1.71E-26	postive
ATG9A	AC064807.1	0.38637134	3.00E-19	postive
ATG9A	AC064836.3	0.392341824	7.56E-20	postive
ATG9A	AC067750.1	0.394681968	4.37E-20	postive
ATG9A	AC067838.1	0.337576139	8.60E-15	postive
ATG9A	AC067852.2	0.728945744	5.35E-84	postive
ATG9A	AC067930.4	0.309978021	1.35E-12	postive
ATG9A	AC068228.2	0.308838938	1.64E-12	postive
ATG9A	AC068338.2	0.398889419	1.61E-20	postive
ATG9A	AC068338.3	0.312210305	9.13E-13	postive
ATG9A	AC068473.5	0.40528777	3.44E-21	postive
ATG9A	AC068580.1	0.368201314	1.69E-17	postive
ATG9A	AC068580.3	0.454876195	6.65E-27	postive
ATG9A	AC068790.9	0.307044306	2.24E-12	postive
ATG9A	AC068831.2	0.376157571	2.98E-18	postive
ATG9A	AC068870.2	0.431964863	3.81E-24	postive
ATG9A	AC068888.1	0.346736458	1.43E-15	postive
ATG9A	AC069185.1	0.348479106	1.01E-15	postive
ATG9A	AC069307.1	0.326202187	7.35E-14	postive
ATG9A	AC069544.1	0.318271921	3.11E-13	postive

ATG9A	AC072061.1	0.320733256	2.00E-13	postive
ATG9A	AC073046.1	0.450713864	2.19E-26	postive
ATG9A	AC073073.2	0.427653322	1.19E-23	postive
ATG9A	AC073195.2	0.303186284	4.32E-12	postive
ATG9A	AC073254.1	0.459281645	1.85E-27	postive
ATG9A	AC073508.3	0.45643192	4.25E-27	postive
ATG9A	AC073569.2	0.518392123	9.80E-36	postive
ATG9A	AC073575.4	0.352847525	4.18E-16	postive
ATG9A	AC073611.1	0.345033326	2.01E-15	postive
ATG9A	AC073869.1	0.434872967	1.75E-24	postive
ATG9A	AC073896.2	0.372188541	7.12E-18	postive
ATG9A	AC073896.3	0.411599799	7.26E-22	postive
ATG9A	AC073896.4	0.397402885	2.30E-20	postive
ATG9A	AC074117.1	0.444620788	1.21E-25	postive
ATG9A	AC078785.1	0.316408565	4.35E-13	postive
ATG9A	AC079305.2	0.30351579	4.09E-12	postive
ATG9A	AC079807.1	0.485914153	5.47E-31	postive
ATG9A	AC079834.2	0.377729914	2.11E-18	postive
ATG9A	AC079848.1	0.314193787	6.44E-13	postive
ATG9A	AC079922.2	0.332946874	2.08E-14	postive
ATG9A	AC080023.1	0.307840081	1.95E-12	postive
ATG9A	AC080112.2	0.544639491	5.80E-40	postive
ATG9A	AC083799.1	0.380250516	1.20E-18	postive
ATG9A	AC083862.2	0.390326825	1.21E-19	postive
ATG9A	AC083964.1	0.357253737	1.69E-16	postive
ATG9A	AC084018.1	0.301987684	5.30E-12	postive
ATG9A	AC084018.2	0.308126371	1.86E-12	postive
ATG9A	AC084125.4	0.335142925	1.37E-14	postive
ATG9A	AC084809.1	0.352372935	4.60E-16	postive
ATG9A	AC087289.1	0.337616307	8.53E-15	postive
ATG9A	AC087289.2	0.409586181	1.20E-21	postive
ATG9A	AC087289.5	0.406839847	2.36E-21	postive
ATG9A	AC087501.4	0.349097343	8.92E-16	postive
ATG9A	AC087521.3	0.345104195	1.98E-15	postive
ATG9A	AC087645.2	0.380203045	1.21E-18	postive
ATG9A	AC087741.1	0.301275359	5.97E-12	postive
ATG9A	AC087741.2	0.318399287	3.04E-13	postive
ATG9A	AC090114.2	0.400160023	1.19E-20	postive
ATG9A	AC090198.1	0.33423814	1.63E-14	postive
ATG9A	AC090229.1	0.352278082	4.69E-16	postive
ATG9A	AC090425.2	0.314247744	6.38E-13	postive
ATG9A	AC090515.3	0.403893177	4.83E-21	postive
ATG9A	AC090587.1	0.372192613	7.12E-18	postive
ATG9A	AC090587.2	0.392389844	7.48E-20	postive
ATG9A	AC090589.3	0.306861556	2.31E-12	postive
ATG9A	AC090617.5	0.385470955	3.69E-19	postive
ATG9A	AC091057.1	0.376562518	2.73E-18	postive
ATG9A	AC091271.1	0.317199611	3.77E-13	postive
ATG9A	AC091729.3	0.384567568	4.53E-19	postive
ATG9A	AC092070.2	0.378599822	1.74E-18	postive

ATG9A	AC092115.4	0.300107122	7.26E-12	postive
ATG9A	AC092118.2	0.3506967	6.46E-16	postive
ATG9A	AC092123.1	0.315721595	4.91E-13	postive
ATG9A	AC092171.2	0.498530283	9.00E-33	postive
ATG9A	AC092295.2	0.502038169	2.79E-33	postive
ATG9A	AC092354.2	0.301292785	5.95E-12	postive
ATG9A	AC092368.3	0.541719922	1.79E-39	postive
ATG9A	AC092614.1	0.415057371	3.05E-22	postive
ATG9A	AC092687.3	0.335083454	1.39E-14	postive
ATG9A	AC092718.2	0.309577419	1.44E-12	postive
ATG9A	AC092718.4	0.346410964	1.53E-15	postive
ATG9A	AC092718.5	0.321550645	1.72E-13	postive
ATG9A	AC092720.1	0.342269825	3.45E-15	postive
ATG9A	AC092747.4	0.372389928	6.82E-18	postive
ATG9A	AC092803.1	0.352470739	4.51E-16	postive
ATG9A	AC092910.3	0.340720007	4.68E-15	postive
ATG9A	AC092953.2	0.302779223	4.63E-12	postive
ATG9A	AC093010.3	0.318820604	2.82E-13	postive
ATG9A	AC093227.1	0.348188975	1.07E-15	postive
ATG9A	AC093249.6	0.329578258	3.92E-14	postive
ATG9A	AC093278.2	0.322258604	1.52E-13	postive
ATG9A	AC093297.2	0.439540714	4.92E-25	postive
ATG9A	AC093382.1	0.318822014	2.82E-13	postive
ATG9A	AC093627.5	0.409830589	1.13E-21	postive
ATG9A	AC093673.1	0.372703939	6.37E-18	postive
ATG9A	AC093726.1	0.323193037	1.28E-13	postive
ATG9A	AC093752.1	0.334309984	1.61E-14	postive
ATG9A	AC093752.3	0.361030718	7.72E-17	postive
ATG9A	AC096536.2	0.419522863	9.82E-23	postive
ATG9A	AC096677.1	0.39482529	4.23E-20	postive
ATG9A	AC096733.3	0.410988651	8.46E-22	postive
ATG9A	AC096772.6	0.56385289	2.65E-43	postive
ATG9A	AC096992.2	0.336434874	1.07E-14	postive
ATG9A	AC097359.2	0.359608128	1.04E-16	postive
ATG9A	AC097468.3	0.559823785	1.39E-42	postive
ATG9A	AC097639.1	0.355716984	2.32E-16	postive
ATG9A	AC098484.1	0.47835738	5.91E-30	postive
ATG9A	AC098820.2	0.46751293	1.62E-28	postive
ATG9A	AC098820.4	0.327111222	6.21E-14	postive
ATG9A	AC099343.4	0.363864127	4.25E-17	postive
ATG9A	AC099568.2	0.301244357	6.00E-12	postive
ATG9A	AC099778.1	0.419508987	9.86E-23	postive
ATG9A	AC099850.2	0.395944251	3.25E-20	postive
ATG9A	AC099850.4	0.348821757	9.43E-16	postive
ATG9A	AC100803.3	0.378424157	1.80E-18	postive
ATG9A	AC100810.1	0.383806602	5.39E-19	postive
ATG9A	AC100847.1	0.337591011	8.57E-15	postive
ATG9A	AC100861.1	0.470834181	5.96E-29	postive
ATG9A	AC102953.2	0.337260637	9.14E-15	postive
ATG9A	AC103974.1	0.302310512	5.01E-12	postive

ATG9A	AC104109.2	0.368520331	1.58E-17	postive
ATG9A	AC104113.1	0.414441723	3.57E-22	postive
ATG9A	AC104411.2	0.355928604	2.23E-16	postive
ATG9A	AC104521.1	0.307594828	2.04E-12	postive
ATG9A	AC104532.2	0.321304579	1.80E-13	postive
ATG9A	AC104561.1	0.315704152	4.93E-13	postive
ATG9A	AC104564.3	0.34404243	2.44E-15	postive
ATG9A	AC104667.2	0.314170053	6.46E-13	postive
ATG9A	AC104794.3	0.375504737	3.44E-18	postive
ATG9A	AC104825.1	0.369898267	1.17E-17	postive
ATG9A	AC105001.1	0.306880413	2.30E-12	postive
ATG9A	AC105020.6	0.362333336	5.87E-17	postive
ATG9A	AC105036.3	0.442121969	2.42E-25	postive
ATG9A	AC105206.2	0.385440047	3.72E-19	postive
ATG9A	AC105219.1	0.352658357	4.34E-16	postive
ATG9A	AC105285.1	0.317575517	3.53E-13	postive
ATG9A	AC106739.1	0.473386674	2.74E-29	postive
ATG9A	AC106782.5	0.304672594	3.36E-12	postive
ATG9A	AC106786.2	0.363790218	4.32E-17	postive
ATG9A	AC106795.1	0.407764237	1.88E-21	postive
ATG9A	AC106820.5	0.348269232	1.05E-15	postive
ATG9A	AC107027.3	0.432512403	3.29E-24	postive
ATG9A	AC107068.1	0.421612019	5.75E-23	postive
ATG9A	AC107214.1	0.352712678	4.30E-16	postive
ATG9A	AC107214.2	0.362029225	6.26E-17	postive
ATG9A	AC107375.1	0.325788673	7.93E-14	postive
ATG9A	AC107959.1	0.31534639	5.25E-13	postive
ATG9A	AC107959.3	0.412439289	5.89E-22	postive
ATG9A	AC107982.3	0.418010208	1.45E-22	postive
ATG9A	AC108010.1	0.316455209	4.31E-13	postive
ATG9A	AC108134.1	0.324467388	1.01E-13	postive
ATG9A	AC108488.1	0.395997299	3.20E-20	postive
ATG9A	AC108860.2	0.30156276	5.69E-12	postive
ATG9A	AC109322.1	0.328260037	5.02E-14	postive
ATG9A	AC109347.1	0.430954546	4.98E-24	postive
ATG9A	AC109449.1	0.33568579	1.24E-14	postive
ATG9A	AC109454.2	0.326591991	6.84E-14	postive
ATG9A	AC109460.1	0.37138579	8.48E-18	postive
ATG9A	AC109460.2	0.364006227	4.13E-17	postive
ATG9A	AC110079.1	0.451550905	1.72E-26	postive
ATG9A	AC112220.2	0.504304498	1.30E-33	postive
ATG9A	AC112236.2	0.369304449	1.33E-17	postive
ATG9A	AC112694.1	0.351496887	5.50E-16	postive
ATG9A	AC113189.4	0.328582882	4.73E-14	postive
ATG9A	AC114271.1	0.520617795	4.44E-36	postive
ATG9A	AC114341.1	0.399790605	1.30E-20	postive
ATG9A	AC114490.1	0.358742903	1.24E-16	postive
ATG9A	AC114730.1	0.323972542	1.11E-13	postive
ATG9A	AC114730.2	0.323342717	1.24E-13	postive
ATG9A	AC114810.1	0.361577884	6.88E-17	postive

ATG9A	AC114956.2	0.316289501	4.44E-13	postive
ATG9A	AC115618.1	0.410975748	8.48E-22	postive
ATG9A	AC115618.2	0.524755396	1.00E-36	postive
ATG9A	AC116447.1	0.354286631	3.12E-16	postive
ATG9A	AC116913.1	0.418226069	1.37E-22	postive
ATG9A	AC116914.2	0.33248511	2.27E-14	postive
ATG9A	AC118754.1	0.30039816	6.92E-12	postive
ATG9A	AC120053.1	0.343087442	2.94E-15	postive
ATG9A	AC120114.1	0.302275223	5.04E-12	postive
ATG9A	AC121338.2	0.365340877	3.11E-17	postive
ATG9A	AC121757.1	0.327488947	5.79E-14	postive
ATG9A	AC121761.1	0.302005545	5.28E-12	postive
ATG9A	AC123768.2	0.312724576	8.34E-13	postive
ATG9A	AC124016.2	0.353992286	3.31E-16	postive
ATG9A	AC124283.2	0.398886031	1.61E-20	postive
ATG9A	AC125257.1	0.497947572	1.09E-32	postive
ATG9A	AC125437.1	0.356307137	2.06E-16	postive
ATG9A	AC125494.1	0.435092935	1.65E-24	postive
ATG9A	AC125494.2	0.336071332	1.15E-14	postive
ATG9A	AC125807.2	0.433232704	2.71E-24	postive
ATG9A	AC126755.1	0.375485054	3.46E-18	postive
ATG9A	AC127024.6	0.324044705	1.09E-13	postive
ATG9A	AC127502.2	0.310798624	1.17E-12	postive
ATG9A	AC127521.1	0.321274943	1.81E-13	postive
ATG9A	AC127537.1	0.35280877	4.21E-16	postive
ATG9A	AC129510.1	0.303947874	3.80E-12	postive
ATG9A	AC130371.2	0.348656956	9.74E-16	postive
ATG9A	AC131009.3	0.4197779	9.20E-23	postive
ATG9A	AC131097.3	0.310523883	1.23E-12	postive
ATG9A	AC131159.2	0.318490089	2.99E-13	postive
ATG9A	AC131235.3	0.32591518	7.75E-14	postive
ATG9A	AC132008.2	0.425753725	1.96E-23	postive
ATG9A	AC132872.1	0.344070779	2.43E-15	postive
ATG9A	AC132872.3	0.352892639	4.14E-16	postive
ATG9A	AC132938.4	0.424699187	2.58E-23	postive
ATG9A	AC133528.2	0.478211959	6.18E-30	postive
ATG9A	AC133552.2	0.327671806	5.60E-14	postive
ATG9A	AC134878.2	0.328702166	4.62E-14	postive
ATG9A	AC135050.1	0.548458396	1.31E-40	postive
ATG9A	AC135050.4	0.317177178	3.79E-13	postive
ATG9A	AC135050.5	0.349848823	7.67E-16	postive
ATG9A	AC135050.6	0.365784457	2.83E-17	postive
ATG9A	AC135178.4	0.393692847	5.51E-20	postive
ATG9A	AC135178.6	0.360386195	8.83E-17	postive
ATG9A	AC135983.3	0.322043214	1.58E-13	postive
ATG9A	AC136475.2	0.360616746	8.42E-17	postive
ATG9A	AC137630.1	0.307583957	2.04E-12	postive
ATG9A	AC137932.1	0.301392908	5.85E-12	postive
ATG9A	AC137932.3	0.327568026	5.71E-14	postive
ATG9A	AC138028.4	0.325894992	7.78E-14	postive

ATG9A	AC138028.6	0.337295475	9.08E-15	postive
ATG9A	AC138035.1	0.383093662	6.33E-19	postive
ATG9A	AC138207.4	0.379473801	1.43E-18	postive
ATG9A	AC138207.8	0.363410435	4.68E-17	postive
ATG9A	AC138696.2	0.326302142	7.22E-14	postive
ATG9A	AC138932.1	0.391279096	9.69E-20	postive
ATG9A	AC138956.2	0.3104262	1.25E-12	postive
ATG9A	AC139149.1	0.361704423	6.70E-17	postive
ATG9A	AC139256.3	0.350169822	7.19E-16	postive
ATG9A	AC139530.1	0.395905655	3.27E-20	postive
ATG9A	AC139768.1	0.412407098	5.94E-22	postive
ATG9A	AC139795.2	0.345436013	1.85E-15	postive
ATG9A	AC139887.1	0.30725482	2.16E-12	postive
ATG9A	AC139887.2	0.444809164	1.15E-25	postive
ATG9A	AC141586.1	0.548066302	1.53E-40	postive
ATG9A	AC142381.3	0.428862963	8.67E-24	postive
ATG9A	AC142472.1	0.338372012	7.37E-15	postive
ATG9A	AC145098.1	0.351006228	6.07E-16	postive
ATG9A	AC145207.5	0.383897448	5.28E-19	postive
ATG9A	AC145285.6	0.355315962	2.52E-16	postive
ATG9A	AC145423.3	0.307505776	2.07E-12	postive
ATG9A	AC211433.2	0.303841649	3.87E-12	postive
ATG9A	AC231981.1	0.325989194	7.65E-14	postive
ATG9A	AC232271.1	0.307266775	2.15E-12	postive
ATG9A	AC233992.3	0.325143076	8.94E-14	postive
ATG9A	AC242376.2	0.313589382	7.16E-13	postive
ATG9A	AC244669.1	0.328292136	4.99E-14	postive
ATG9A	AC245052.4	0.351836167	5.13E-16	postive
ATG9A	AC245060.2	0.319792238	2.37E-13	postive
ATG9A	AC245140.2	0.418287408	1.35E-22	postive
ATG9A	AC245297.4	0.31395205	6.72E-13	postive
ATG9A	AC245452.1	0.358984008	1.18E-16	postive
ATG9A	ACVR2B-AS1	0.350176726	7.18E-16	postive
ATG9A	AD000671.3	0.370855233	9.52E-18	postive
ATG9A	ADGRD1-AS1	0.321084785	1.88E-13	postive
ATG9A	AF001548.2	0.323174302	1.28E-13	postive
ATG9A	AF131216.4	0.344955027	2.04E-15	postive
ATG9A	AFG3L1P	0.417106071	1.82E-22	postive
ATG9A	AGBL5-IT1	0.307199481	2.18E-12	postive
ATG9A	AL008726.1	0.343690658	2.61E-15	postive
ATG9A	AL008729.1	0.316427541	4.33E-13	postive
ATG9A	AL009178.2	0.37211067	7.24E-18	postive
ATG9A	AL009179.1	0.324849102	9.43E-14	postive
ATG9A	AL020996.1	0.427696176	1.18E-23	postive
ATG9A	AL021392.1	0.312744873	8.31E-13	postive
ATG9A	AL021707.3	0.356779048	1.87E-16	postive
ATG9A	AL021878.2	0.306531052	2.44E-12	postive
ATG9A	AL022238.3	0.308687786	1.69E-12	postive
ATG9A	AL022328.1	0.305183602	3.08E-12	postive
ATG9A	AL022328.2	0.319892125	2.33E-13	postive

ATG9A	AL022344.1	0.314619588	5.97E-13	postive
ATG9A	AL023284.4	0.38997529	1.31E-19	postive
ATG9A	AL023803.2	0.320776557	1.98E-13	postive
ATG9A	AL024498.1	0.305682523	2.83E-12	postive
ATG9A	AL024507.3	0.403486347	5.34E-21	postive
ATG9A	AL031055.1	0.343103959	2.93E-15	postive
ATG9A	AL031123.2	0.392309668	7.62E-20	postive
ATG9A	AL031282.1	0.426497365	1.61E-23	postive
ATG9A	AL031282.2	0.409643948	1.18E-21	postive
ATG9A	AL031600.1	0.30882443	1.65E-12	postive
ATG9A	AL031714.1	0.340529492	4.85E-15	postive
ATG9A	AL031775.1	0.433336268	2.64E-24	postive
ATG9A	AL031985.3	0.395333722	3.75E-20	postive
ATG9A	AL034550.1	0.303517128	4.09E-12	postive
ATG9A	AL035252.4	0.309167278	1.55E-12	postive
ATG9A	AL035587.1	0.485715026	5.83E-31	postive
ATG9A	AL049597.2	0.481864259	1.97E-30	postive
ATG9A	AL049779.1	0.300443502	6.87E-12	postive
ATG9A	AL049796.1	0.417874902	1.50E-22	postive
ATG9A	AL049840.4	0.518084676	1.09E-35	postive
ATG9A	AL049840.5	0.514436329	3.96E-35	postive
ATG9A	AL049840.6	0.482825357	1.46E-30	postive
ATG9A	AL049871.1	0.307419933	2.10E-12	postive
ATG9A	AL050309.1	0.441872156	2.59E-25	postive
ATG9A	AL078581.2	0.405010083	3.68E-21	postive
ATG9A	AL078644.2	0.454991052	6.44E-27	postive
ATG9A	AL080250.1	0.319073685	2.70E-13	postive
ATG9A	AL096828.3	0.32025623	2.18E-13	postive
ATG9A	AL109811.1	0.324112713	1.08E-13	postive
ATG9A	AL117209.1	0.363798646	4.31E-17	postive
ATG9A	AL117327.1	0.323117554	1.30E-13	postive
ATG9A	AL117332.1	0.402901187	6.15E-21	postive
ATG9A	AL117336.2	0.376260153	2.92E-18	postive
ATG9A	AL118506.1	0.498920476	7.91E-33	postive
ATG9A	AL118558.3	0.351895526	5.07E-16	postive
ATG9A	AL121603.2	0.371806297	7.74E-18	postive
ATG9A	AL121894.2	0.356851536	1.84E-16	postive
ATG9A	AL121906.2	0.313937117	6.74E-13	postive
ATG9A	AL121944.2	0.526846948	4.67E-37	postive
ATG9A	AL122010.1	0.480469151	3.06E-30	postive
ATG9A	AL122023.3	0.387231082	2.47E-19	postive
ATG9A	AL122035.1	0.347708858	1.18E-15	postive
ATG9A	AL132712.1	0.311475674	1.04E-12	postive
ATG9A	AL133215.2	0.331122865	2.94E-14	postive
ATG9A	AL133216.2	0.335756949	1.22E-14	postive
ATG9A	AL133338.1	0.345897756	1.69E-15	postive
ATG9A	AL133355.1	0.414571546	3.45E-22	postive
ATG9A	AL133367.1	0.323572062	1.19E-13	postive
ATG9A	AL133406.2	0.308900796	1.62E-12	postive
ATG9A	AL133520.1	0.353448621	3.70E-16	postive

ATG9A	AL135791.1	0.301693893	5.56E-12	postive
ATG9A	AL135925.1	0.416662047	2.04E-22	postive
ATG9A	AL136040.1	0.388749089	1.74E-19	postive
ATG9A	AL136084.3	0.304111079	3.70E-12	postive
ATG9A	AL136295.7	0.326672846	6.74E-14	postive
ATG9A	AL136368.1	0.310165471	1.30E-12	postive
ATG9A	AL137058.2	0.370394127	1.05E-17	postive
ATG9A	AL137127.1	0.414761889	3.29E-22	postive
ATG9A	AL137186.2	0.356956106	1.80E-16	postive
ATG9A	AL138724.2	0.307903702	1.93E-12	postive
ATG9A	AL138756.1	0.447375879	5.61E-26	postive
ATG9A	AL138762.2	0.363628251	4.47E-17	postive
ATG9A	AL138781.1	0.31200633	9.46E-13	postive
ATG9A	AL138921.1	0.334454593	1.56E-14	postive
ATG9A	AL138921.2	0.326158042	7.41E-14	postive
ATG9A	AL139011.1	0.421413863	6.05E-23	postive
ATG9A	AL139035.1	0.439418642	5.08E-25	postive
ATG9A	AL139246.5	0.323451122	1.22E-13	postive
ATG9A	AL139287.1	0.381214277	9.67E-19	postive
ATG9A	AL139384.1	0.393157443	6.25E-20	postive
ATG9A	AL139423.1	0.325005193	9.17E-14	postive
ATG9A	AL157832.1	0.318863266	2.80E-13	postive
ATG9A	AL158212.3	0.468001216	1.40E-28	postive
ATG9A	AL158835.2	0.33606117	1.15E-14	postive
ATG9A	AL160006.1	0.470225598	7.16E-29	postive
ATG9A	AL160313.2	0.354567761	2.94E-16	postive
ATG9A	AL161421.1	0.389523631	1.46E-19	postive
ATG9A	AL161452.1	0.387978201	2.08E-19	postive
ATG9A	AL161729.1	0.311983606	9.50E-13	postive
ATG9A	AL162231.2	0.372584495	6.53E-18	postive
ATG9A	AL162258.2	0.327919826	5.35E-14	postive
ATG9A	AL162274.2	0.37612723	3.00E-18	postive
ATG9A	AL162377.1	0.403474652	5.35E-21	postive
ATG9A	AL162431.1	0.349364171	8.45E-16	postive
ATG9A	AL162595.1	0.436546464	1.11E-24	postive
ATG9A	AL162741.1	0.318434305	3.02E-13	postive
ATG9A	AL353194.1	0.352865348	4.17E-16	postive
ATG9A	AL353593.1	0.447963508	4.75E-26	postive
ATG9A	AL353708.1	0.406034479	2.87E-21	postive
ATG9A	AL353743.1	0.382226741	7.70E-19	postive
ATG9A	AL353796.1	0.411397777	7.64E-22	postive
ATG9A	AL354696.1	0.322854196	1.36E-13	postive
ATG9A	AL354696.2	0.375278473	3.62E-18	postive
ATG9A	AL354740.1	0.319944681	2.30E-13	postive
ATG9A	AL354892.2	0.435434399	1.50E-24	postive
ATG9A	AL354920.1	0.342711385	3.17E-15	postive
ATG9A	AL354928.1	0.366920568	2.22E-17	postive
ATG9A	AL355001.2	0.370522754	1.02E-17	postive
ATG9A	AL355353.2	0.311460037	1.04E-12	postive
ATG9A	AL355574.1	0.427335104	1.30E-23	postive

ATG9A	AL355802.3	0.422450154	4.63E-23	postive
ATG9A	AL355803.1	0.375794322	3.23E-18	postive
ATG9A	AL356019.2	0.345209566	1.94E-15	postive
ATG9A	AL356299.3	0.341473959	4.04E-15	postive
ATG9A	AL356481.1	0.439320993	5.22E-25	postive
ATG9A	AL356481.3	0.32788552	5.38E-14	postive
ATG9A	AL356488.3	0.364210972	3.95E-17	postive
ATG9A	AL357033.3	0.301866942	5.40E-12	postive
ATG9A	AL357079.1	0.313398583	7.41E-13	postive
ATG9A	AL357140.2	0.309974174	1.35E-12	postive
ATG9A	AL358472.2	0.3643775	3.81E-17	postive
ATG9A	AL358472.3	0.416830133	1.95E-22	postive
ATG9A	AL358852.1	0.324549301	9.97E-14	postive
ATG9A	AL359091.3	0.343469595	2.73E-15	postive
ATG9A	AL359198.1	0.317462008	3.60E-13	postive
ATG9A	AL359532.1	0.388241556	1.96E-19	postive
ATG9A	AL359643.3	0.494194902	3.77E-32	postive
ATG9A	AL359711.2	0.357270762	1.69E-16	postive
ATG9A	AL359715.3	0.393935476	5.21E-20	postive
ATG9A	AL359921.2	0.397937139	2.02E-20	postive
ATG9A	AL360181.2	0.401298686	9.05E-21	postive
ATG9A	AL360270.1	0.383947832	5.22E-19	postive
ATG9A	AL360270.3	0.300796796	6.47E-12	postive
ATG9A	AL365203.2	0.363709231	4.39E-17	postive
ATG9A	AL365330.1	0.386933731	2.64E-19	postive
ATG9A	AL390719.1	0.33269043	2.18E-14	postive
ATG9A	AL390728.4	0.354627242	2.91E-16	postive
ATG9A	AL390955.2	0.317009981	3.90E-13	postive
ATG9A	AL391121.1	0.537133987	1.02E-38	postive
ATG9A	AL391244.2	0.395976197	3.22E-20	postive
ATG9A	AL391684.1	0.319984575	2.29E-13	postive
ATG9A	AL391825.1	0.328274096	5.01E-14	postive
ATG9A	AL391832.2	0.342723619	3.16E-15	postive
ATG9A	AL391832.3	0.303939971	3.80E-12	postive
ATG9A	AL391834.2	0.311181407	1.09E-12	postive
ATG9A	AL391988.1	0.326079583	7.52E-14	postive
ATG9A	AL441992.2	0.405540431	3.24E-21	postive
ATG9A	AL445222.2	0.332483821	2.27E-14	postive
ATG9A	AL449106.1	0.304727508	3.33E-12	postive
ATG9A	AL450326.1	0.548615578	1.23E-40	postive
ATG9A	AL450998.2	0.392869523	6.69E-20	postive
ATG9A	AL451042.1	0.318069671	3.23E-13	postive
ATG9A	AL451042.2	0.370492013	1.03E-17	postive
ATG9A	AL451050.2	0.327244084	6.06E-14	postive
ATG9A	AL451085.3	0.430689061	5.35E-24	postive
ATG9A	AL451165.2	0.343550486	2.69E-15	postive
ATG9A	AL512408.1	0.330049229	3.59E-14	postive
ATG9A	AL512413.1	0.304004593	3.76E-12	postive
ATG9A	AL512625.1	0.361130565	7.56E-17	postive
ATG9A	AL512770.1	0.361868798	6.47E-17	postive

ATG9A	AL513165.1	0.422343254	4.76E-23	postive
ATG9A	AL513477.1	0.306786059	2.34E-12	postive
ATG9A	AL513550.1	0.355876764	2.25E-16	postive
ATG9A	AL583722.3	0.346026254	1.65E-15	postive
ATG9A	AL589765.4	0.458020604	2.68E-27	postive
ATG9A	AL589765.5	0.310109166	1.32E-12	postive
ATG9A	AL590133.1	0.318466793	3.01E-13	postive
ATG9A	AL590369.1	0.373761243	5.05E-18	postive
ATG9A	AL590617.2	0.38386016	5.32E-19	postive
ATG9A	AL590666.2	0.340217705	5.16E-15	postive
ATG9A	AL591684.2	0.31077912	1.17E-12	postive
ATG9A	AL592071.1	0.327987301	5.28E-14	postive
ATG9A	AL592148.3	0.312422271	8.79E-13	postive
ATG9A	AL592166.1	0.379875355	1.31E-18	postive
ATG9A	AL596094.1	0.367226549	2.08E-17	postive
ATG9A	AL596202.1	0.316294061	4.43E-13	postive
ATG9A	AL603839.2	0.386497651	2.92E-19	postive
ATG9A	AL604028.1	0.413747886	4.25E-22	postive
ATG9A	AL606489.1	0.319735712	2.39E-13	postive
ATG9A	AL606491.1	0.329786125	3.77E-14	postive
ATG9A	AL606760.2	0.308438151	1.76E-12	postive
ATG9A	AL606834.1	0.393858727	5.30E-20	postive
ATG9A	AL627309.5	0.308553523	1.73E-12	postive
ATG9A	AL645568.1	0.356870645	1.83E-16	postive
ATG9A	AL645608.1	0.309515141	1.46E-12	postive
ATG9A	AL645608.7	0.320242422	2.18E-13	postive
ATG9A	AL645728.1	0.345028408	2.01E-15	postive
ATG9A	AL645933.3	0.305644864	2.85E-12	postive
ATG9A	AL645940.1	0.337278563	9.10E-15	postive
ATG9A	AL645941.1	0.325806473	7.91E-14	postive
ATG9A	AL662797.2	0.333854961	1.75E-14	postive
ATG9A	AL669831.1	0.360490638	8.64E-17	postive
ATG9A	AL671710.1	0.43991382	4.44E-25	postive
ATG9A	AL691432.2	0.334795842	1.46E-14	postive
ATG9A	AL731566.2	0.343572805	2.67E-15	postive
ATG9A	AL731569.1	0.326408921	7.07E-14	postive
ATG9A	AL731571.1	0.411408169	7.62E-22	postive
ATG9A	AL731577.2	0.592447005	1.07E-48	postive
ATG9A	AL732314.8	0.380625501	1.10E-18	postive
ATG9A	AL732372.2	0.358356453	1.35E-16	postive
ATG9A	AL807752.5	0.334703524	1.49E-14	postive
ATG9A	AL928654.1	0.315171392	5.41E-13	postive
ATG9A	ALG1L13P	0.349395757	8.40E-16	postive
ATG9A	ALOX12-AS1	0.465222506	3.22E-28	postive
ATG9A	ALOX12P2	0.316265543	4.46E-13	postive
ATG9A	AMZ2P1	0.397903648	2.04E-20	postive
ATG9A	ANO7L1	0.40558011	3.21E-21	postive
ATG9A	ANP32AP1	0.31521142	5.38E-13	postive
ATG9A	AP000254.2	0.370576433	1.01E-17	postive
ATG9A	AP000442.1	0.450457533	2.35E-26	postive

ATG9A	AP000487.1	0.356252296	2.08E-16	postive
ATG9A	AP000759.1	0.508122147	3.53E-34	postive
ATG9A	AP000769.1	0.326516734	6.94E-14	postive
ATG9A	AP000781.1	0.310791201	1.17E-12	postive
ATG9A	AP000808.1	0.427666692	1.19E-23	postive
ATG9A	AP000894.4	0.315939119	4.72E-13	postive
ATG9A	AP001107.1	0.466291768	2.34E-28	postive
ATG9A	AP001160.1	0.340766292	4.63E-15	postive
ATG9A	AP001160.2	0.30822423	1.83E-12	postive
ATG9A	AP001160.3	0.463961173	4.68E-28	postive
ATG9A	AP001189.3	0.329117239	4.28E-14	postive
ATG9A	AP001318.2	0.494135906	3.84E-32	postive
ATG9A	AP001330.4	0.306078845	2.64E-12	postive
ATG9A	AP001372.2	0.390861139	1.07E-19	postive
ATG9A	AP001453.2	0.335622458	1.25E-14	postive
ATG9A	AP001453.4	0.489194947	1.91E-31	postive
ATG9A	AP001462.1	0.371921579	7.55E-18	postive
ATG9A	AP001469.2	0.302618299	4.76E-12	postive
ATG9A	AP001469.3	0.339740445	5.66E-15	postive
ATG9A	AP001486.2	0.331074218	2.96E-14	postive
ATG9A	AP001505.1	0.428414853	9.76E-24	postive
ATG9A	AP001527.2	0.374185764	4.60E-18	postive
ATG9A	AP001542.3	0.323028774	1.32E-13	postive
ATG9A	AP001816.1	0.391372464	9.48E-20	postive
ATG9A	AP001922.6	0.331098835	2.95E-14	postive
ATG9A	AP002360.2	0.30509546	3.13E-12	postive
ATG9A	AP002387.2	0.342548618	3.27E-15	postive
ATG9A	AP002748.4	0.349939679	7.53E-16	postive
ATG9A	AP002761.3	0.32961175	3.90E-14	postive
ATG9A	AP002761.4	0.405102608	3.60E-21	postive
ATG9A	AP002807.1	0.358324762	1.36E-16	postive
ATG9A	AP002840.2	0.334150818	1.66E-14	postive
ATG9A	AP003068.1	0.387490605	2.32E-19	postive
ATG9A	AP003068.2	0.432110818	3.66E-24	postive
ATG9A	AP003352.1	0.306523211	2.45E-12	postive
ATG9A	AP003392.5	0.313644282	7.09E-13	postive
ATG9A	AP003486.1	0.447558551	5.33E-26	postive
ATG9A	AP003498.1	0.304177355	3.65E-12	postive
ATG9A	AP003680.1	0.360097141	9.38E-17	postive
ATG9A	AP003721.4	0.348819038	9.43E-16	postive
ATG9A	AP004609.5	0.308999474	1.60E-12	postive
ATG9A	AP005329.1	0.326900231	6.46E-14	postive
ATG9A	AP005432.2	0.306475167	2.47E-12	postive
ATG9A	AP005482.4	0.447770682	5.02E-26	postive
ATG9A	AP005717.1	0.317976794	3.28E-13	postive
ATG9A	AP006621.3	0.357393611	1.65E-16	postive
ATG9A	ARHGAP5-AS1	0.314277257	6.34E-13	postive
ATG9A	ARHGEF35-AS1	0.326604972	6.82E-14	postive
ATG9A	ARMCX5-GPRASP2	0.503924401	1.48E-33	postive
ATG9A	ARRDC1-AS1	0.506624189	5.89E-34	postive

ATG9A	ASB16-AS1	0.491492519	9.09E-32	postive
ATG9A	ASH1L-AS1	0.423153952	3.86E-23	postive
ATG9A	ATF4P4	0.352229627	4.74E-16	postive
ATG9A	ATP2B1-AS1	0.334555816	1.53E-14	postive
ATG9A	ATXN1-AS1	0.388904109	1.68E-19	postive
ATG9A	BAALC-AS1	0.378292804	1.86E-18	postive
ATG9A	BACE1-AS	0.438668865	6.24E-25	postive
ATG9A	BAIAP2-DT	0.518986753	7.94E-36	postive
ATG9A	BCDIN3D-AS1	0.392741312	6.89E-20	postive
ATG9A	BDNF-AS	0.306595657	2.42E-12	postive
ATG9A	BEND3P1	0.360741909	8.20E-17	postive
ATG9A	BMS1P20	0.472446319	3.65E-29	postive
ATG9A	BTG3-AS1	0.341534416	3.99E-15	postive
ATG9A	BTN2A3P	0.419306452	1.04E-22	postive
ATG9A	BX255925.1	0.30373095	3.94E-12	postive
ATG9A	BX322557.10	0.449649745	2.95E-26	postive
ATG9A	BX537318.1	0.373678079	5.14E-18	postive
ATG9A	BX539320.1	0.315974631	4.69E-13	postive
ATG9A	C10orf25	0.393933499	5.21E-20	postive
ATG9A	C15orf56	0.33901529	6.51E-15	postive
ATG9A	C17orf82	0.305409087	2.96E-12	postive
ATG9A	C18orf65	0.347112015	1.33E-15	postive
ATG9A	C1orf220	0.3025013	4.86E-12	postive
ATG9A	C1orf229	0.338826366	6.75E-15	postive
ATG9A	C1RL-AS1	0.375670035	3.32E-18	postive
ATG9A	C22orf46	0.515591434	2.64E-35	postive
ATG9A	C2CD4D-AS1	0.450146986	2.57E-26	postive
ATG9A	C2orf27A	0.321132882	1.86E-13	postive
ATG9A	C5orf66	0.406467896	2.58E-21	postive
ATG9A	C6orf99	0.300993047	6.26E-12	postive
ATG9A	C9orf163	0.334026337	1.70E-14	postive
ATG9A	CA5BP1	0.550745803	5.31E-41	postive
ATG9A	CACTIN-AS1	0.372902906	6.09E-18	postive
ATG9A	CAPN10-DT	0.398237146	1.88E-20	postive
ATG9A	CATSPER2P1	0.448258022	4.38E-26	postive
ATG9A	CCDC183-AS1	0.431045438	4.86E-24	postive
ATG9A	CCDC84-DT	0.309099317	1.57E-12	postive
ATG9A	CCNT2-AS1	0.472500892	3.59E-29	postive
ATG9A	CCT6P1	0.369419499	1.30E-17	postive
ATG9A	CD27-AS1	0.479553639	4.07E-30	postive
ATG9A	CD99P1	0.420157596	8.35E-23	postive
ATG9A	CDC37L1-DT	0.302570622	4.80E-12	postive
ATG9A	CENPBD1P1	0.67048245	1.46E-66	postive
ATG9A	CERNA1	0.313401675	7.40E-13	postive
ATG9A	CFL1P1	0.352613097	4.38E-16	postive
ATG9A	CHKB-CPT1B	0.301631728	5.62E-12	postive
ATG9A	CHROMR	0.37842494	1.80E-18	postive
ATG9A	CIDECP1	0.555695754	7.38E-42	postive
ATG9A	CIRBP-AS1	0.388847085	1.70E-19	postive
ATG9A	CKMT2-AS1	0.438864309	5.92E-25	postive

ATG9A	CNNM3-DT	0.388305822	1.93E-19	postive
ATG9A	COX10-AS1	0.45404806	8.44E-27	postive
ATG9A	CPNE8-AS1	0.30904408	1.58E-12	postive
ATG9A	CR381653.1	0.328765411	4.57E-14	postive
ATG9A	CROCCP2	0.375428387	3.50E-18	postive
ATG9A	CRYBB2P1	0.406552144	2.53E-21	postive
ATG9A	CRYZL2P	0.52671455	4.91E-37	postive
ATG9A	CTA-14H9.5	0.402013173	7.62E-21	postive
ATG9A	CTA-989H11.1	0.381829442	8.42E-19	postive
ATG9A	CTBP1-AS	0.319018911	2.72E-13	postive
ATG9A	CTBP1-DT	0.597020822	1.30E-49	postive
ATG9A	CTC-205M6.5	0.414605481	3.42E-22	postive
ATG9A	CTC-487M23.5	0.324475819	1.01E-13	postive
ATG9A	CTC-786C10.1	0.391007631	1.03E-19	postive
ATG9A	CTD-2132N18.4	0.326129002	7.45E-14	postive
ATG9A	CTD-2245F17.3	0.371014852	9.19E-18	postive
ATG9A	CTD-2574D22.4	0.364964253	3.37E-17	postive
ATG9A	CUTALP	0.388774704	1.73E-19	postive
ATG9A	DACT3-AS1	0.330350182	3.40E-14	postive
ATG9A	DANCR	0.361841926	6.51E-17	postive
ATG9A	DARS-AS1	0.383349594	5.98E-19	postive
ATG9A	DBIL5P	0.400889268	9.99E-21	postive
ATG9A	DCST1-AS1	0.310739758	1.18E-12	postive
ATG9A	DDN-AS1	0.345565708	1.80E-15	postive
ATG9A	DDX11-AS1	0.340819458	4.59E-15	postive
ATG9A	DDX39B-AS1	0.337523198	8.69E-15	postive
ATG9A	DGCR11	0.507640521	4.16E-34	postive
ATG9A	DHCR24-DT	0.316381242	4.37E-13	postive
ATG9A	DHRS4-AS1	0.422526194	4.54E-23	postive
ATG9A	DLEU1	0.300961117	6.29E-12	postive
ATG9A	DLGAP1-AS1	0.333900217	1.74E-14	postive
ATG9A	DM1-AS	0.365127258	3.25E-17	postive
ATG9A	DNAH10OS	0.334624117	1.51E-14	postive
ATG9A	DNAJC3-DT	0.390578294	1.14E-19	postive
ATG9A	DNMBP-AS1	0.363858937	4.26E-17	postive
ATG9A	DOCK9-DT	0.336439282	1.07E-14	postive
ATG9A	DPP9-AS1	0.318145012	3.19E-13	postive
ATG9A	DPY19L2P3	0.366962977	2.20E-17	postive
ATG9A	DSG2-AS1	0.34077021	4.63E-15	postive
ATG9A	DSTNP2	0.54446475	6.21E-40	postive
ATG9A	EBLN3P	0.521750513	2.96E-36	postive
ATG9A	ECI2-DT	0.373969711	4.83E-18	postive
ATG9A	EDRF1-DT	0.372378901	6.83E-18	postive
ATG9A	EIF1B-AS1	0.324033523	1.10E-13	postive
ATG9A	EIF2AK3-DT	0.404129179	4.57E-21	postive
ATG9A	EIF3J-DT	0.520851197	4.08E-36	postive
ATG9A	ELF3-AS1	0.453312762	1.04E-26	postive
ATG9A	ELOA-AS1	0.377648988	2.14E-18	postive
ATG9A	EMC1-AS1	0.434203627	2.09E-24	postive
ATG9A	EMC3-AS1	0.360129433	9.32E-17	postive

ATG9A	ENTPD3-AS1	0.318961191	2.75E-13	postive
ATG9A	EP400P1	0.408273711	1.66E-21	postive
ATG9A	EPB41L4A-DT	0.413789807	4.20E-22	postive
ATG9A	ERICD	0.392696618	6.96E-20	postive
ATG9A	FAHD2CP	0.390198137	1.25E-19	postive
ATG9A	FAM111A-DT	0.435941382	1.31E-24	postive
ATG9A	FAM157B	0.386060036	3.23E-19	postive
ATG9A	FAM157C	0.328314593	4.97E-14	postive
ATG9A	FAM160A1-DT	0.302852188	4.58E-12	postive
ATG9A	FAM21EP	0.330964879	3.03E-14	postive
ATG9A	FAM21FP	0.33771913	8.36E-15	postive
ATG9A	FAM66B	0.312667589	8.42E-13	postive
ATG9A	FAM66C	0.321511276	1.74E-13	postive
ATG9A	FAM66D	0.317062118	3.87E-13	postive
ATG9A	FAM83C-AS1	0.355554483	2.40E-16	postive
ATG9A	FAM86B3P	0.332653935	2.20E-14	postive
ATG9A	FAM86C2P	0.463186724	5.89E-28	postive
ATG9A	FAM86DP	0.435600807	1.43E-24	postive
ATG9A	FAM86EP	0.508426235	3.18E-34	postive
ATG9A	FAM86FP	0.35584132	2.27E-16	postive
ATG9A	FAM86HP	0.377074203	2.44E-18	postive
ATG9A	FAM86JP	0.51186962	9.68E-35	postive
ATG9A	FBXL19-AS1	0.386898689	2.66E-19	postive
ATG9A	FGD5-AS1	0.54490527	5.23E-40	postive
ATG9A	FLVCR1-DT	0.330444471	3.34E-14	postive
ATG9A	FOXD2-AS1	0.446619666	6.93E-26	postive
ATG9A	FRG1HP	0.503510682	1.70E-33	postive
ATG9A	FZD4-DT	0.378419898	1.81E-18	postive
ATG9A	GASAL1	0.312867618	8.13E-13	postive
ATG9A	GATA2-AS1	0.38019465	1.22E-18	postive
ATG9A	GBAP1	0.319692158	2.41E-13	postive
ATG9A	GCC2-AS1	0.400873853	1.00E-20	postive
ATG9A	GEMIN7-AS1	0.311055947	1.12E-12	postive
ATG9A	GHET1	0.311242109	1.08E-12	postive
ATG9A	GLIDR	0.344720427	2.13E-15	postive
ATG9A	GLUD1P3	0.330060288	3.59E-14	postive
ATG9A	GMDS-DT	0.303835057	3.87E-12	postive
ATG9A	GNRHR2	0.458722296	2.18E-27	postive
ATG9A	GOLGA2P7	0.331129842	2.93E-14	postive
ATG9A	GOLGA6L5P	0.318973368	2.75E-13	postive
ATG9A	GPRC5D-AS1	0.493100679	5.39E-32	postive
ATG9A	GS1-124K5.3	0.323631651	1.18E-13	postive
ATG9A	GS1-358P8.4	0.325410178	8.51E-14	postive
ATG9A	GSEC	0.305682412	2.83E-12	postive
ATG9A	GTF2IP1	0.509505776	2.19E-34	postive
ATG9A	GTF2IP4	0.495354827	2.57E-32	postive
ATG9A	GTSE1-DT	0.330045737	3.60E-14	postive
ATG9A	GUSBP1	0.370426633	1.04E-17	postive
ATG9A	GUSBP2	0.33261417	2.22E-14	postive
ATG9A	GUSBP5	0.342218055	3.49E-15	postive

ATG9A	H1FX-AS1	0.300457799	6.85E-12	postive
ATG9A	H2AZ2P1	0.327737901	5.53E-14	postive
ATG9A	H2BC19P	0.37114954	8.93E-18	postive
ATG9A	H2BC20P	0.36941202	1.30E-17	postive
ATG9A	HCG11	0.469555144	8.77E-29	postive
ATG9A	HCG18	0.528904118	2.20E-37	postive
ATG9A	HCG25	0.411070497	8.29E-22	postive
ATG9A	HEIH	0.4402298	4.07E-25	postive
ATG9A	HERC2P2	0.300712184	6.56E-12	postive
ATG9A	HERC2P9	0.318050099	3.24E-13	postive
ATG9A	HM13-IT1	0.384983649	4.12E-19	postive
ATG9A	HMGB1P5	0.508536863	3.06E-34	postive
ATG9A	HSP90AB2P	0.363158044	4.94E-17	postive
ATG9A	HYI-AS1	0.306618829	2.41E-12	postive
ATG9A	IDH1-AS1	0.307817546	1.96E-12	postive
ATG9A	IER3-AS1	0.313157069	7.73E-13	postive
ATG9A	IL10RB-DT	0.393874058	5.28E-20	postive
ATG9A	ILF3-DT	0.41621294	2.28E-22	postive
ATG9A	INE1	0.307904511	1.93E-12	postive
ATG9A	INTS6-AS1	0.330469288	3.32E-14	postive
ATG9A	IQCH-AS1	0.403370502	5.49E-21	postive
ATG9A	ITFG2-AS1	0.328596418	4.71E-14	postive
ATG9A	ITGA9-AS1	0.307663218	2.01E-12	postive
ATG9A	ITPR1-DT	0.302846256	4.58E-12	postive
ATG9A	JPX	0.376500277	2.77E-18	postive
ATG9A	KCNIP2-AS1	0.38529117	3.84E-19	postive
ATG9A	KDM4A-AS1	0.303212859	4.30E-12	postive
ATG9A	KDM7A-DT	0.310607954	1.21E-12	postive
ATG9A	KIF9-AS1	0.385580164	3.60E-19	postive
ATG9A	KRT18P55	0.339185303	6.30E-15	postive
ATG9A	KRT8P12	0.457189773	3.41E-27	postive
ATG9A	KRTAP5-AS1	0.310113624	1.32E-12	postive
ATG9A	KTN1-AS1	0.353314429	3.80E-16	postive
ATG9A	L3MBTL2-AS1	0.318481069	3.00E-13	postive
ATG9A	LBX2-AS1	0.429570723	7.19E-24	postive
ATG9A	LENG8-AS1	0.37357725	5.26E-18	postive
ATG9A	LINC00115	0.358912414	1.20E-16	postive
ATG9A	LINC00174	0.318754854	2.86E-13	postive
ATG9A	LINC00205	0.457966916	2.72E-27	postive
ATG9A	LINC00235	0.307191027	2.18E-12	postive
ATG9A	LINC002481	0.301324527	5.92E-12	postive
ATG9A	LINC00265	0.303399244	4.17E-12	postive
ATG9A	LINC00294	0.449219834	3.34E-26	postive
ATG9A	LINC00315	0.3377471	8.32E-15	postive
ATG9A	LINC00339	0.442046903	2.47E-25	postive
ATG9A	LINC00346	0.332466454	2.28E-14	postive
ATG9A	LINC00449	0.382152894	7.83E-19	postive
ATG9A	LINC00467	0.378588267	1.74E-18	postive
ATG9A	LINC00471	0.356754703	1.88E-16	postive
ATG9A	LINC00476	0.348653902	9.75E-16	postive

ATG9A	LINC00511	0.350851255	6.27E-16	postive
ATG9A	LINC00526	0.344934782	2.04E-15	postive
ATG9A	LINC00562	0.305282167	3.03E-12	postive
ATG9A	LINC00623	0.376748088	2.62E-18	postive
ATG9A	LINC00630	0.38404449	5.10E-19	postive
ATG9A	LINC00638	0.306729556	2.36E-12	postive
ATG9A	LINC00654	0.368737215	1.50E-17	postive
ATG9A	LINC00665	0.317506751	3.57E-13	postive
ATG9A	LINC00667	0.458910775	2.07E-27	postive
ATG9A	LINC00674	0.360780491	8.13E-17	postive
ATG9A	LINC00680	0.407234978	2.14E-21	postive
ATG9A	LINC00847	0.388713152	1.75E-19	postive
ATG9A	LINC00857	0.465046338	3.39E-28	postive
ATG9A	LINC00863	0.556661823	5.00E-42	postive
ATG9A	LINC00869	0.350379394	6.89E-16	postive
ATG9A	LINC00888	0.392358285	7.53E-20	postive
ATG9A	LINC00899	0.327403959	5.88E-14	postive
ATG9A	LINC00909	0.491071322	1.04E-31	postive
ATG9A	LINC00910	0.415902921	2.47E-22	postive
ATG9A	LINC00921	0.421003573	6.72E-23	postive
ATG9A	LINC00933	0.419642194	9.53E-23	postive
ATG9A	LINC00957	0.452388643	1.36E-26	postive
ATG9A	LINC00963	0.585234224	2.76E-47	postive
ATG9A	LINC00997	0.448969711	3.58E-26	postive
ATG9A	LINC01001	0.390729773	1.10E-19	postive
ATG9A	LINC01006	0.344669245	2.15E-15	postive
ATG9A	LINC01011	0.462059939	8.21E-28	postive
ATG9A	LINC01106	0.315200996	5.39E-13	postive
ATG9A	LINC01124	0.3140465	6.61E-13	postive
ATG9A	LINC01128	0.591288208	1.81E-48	postive
ATG9A	LINC01134	0.361570218	6.89E-17	postive
ATG9A	LINC01137	0.402274767	7.15E-21	postive
ATG9A	LINC01138	0.399185418	1.50E-20	postive
ATG9A	LINC01144	0.302279257	5.04E-12	postive
ATG9A	LINC01145	0.478011047	6.58E-30	postive
ATG9A	LINC01184	0.46035295	1.36E-27	postive
ATG9A	LINC01220	0.336290333	1.10E-14	postive
ATG9A	LINC01270	0.382569818	7.13E-19	postive
ATG9A	LINC01271	0.361549279	6.92E-17	postive
ATG9A	LINC01278	0.529239776	1.94E-37	postive
ATG9A	LINC01376	0.380171995	1.22E-18	postive
ATG9A	LINC01521	0.39789627	2.04E-20	postive
ATG9A	LINC01534	0.310530188	1.22E-12	postive
ATG9A	LINC01560	0.425705209	1.99E-23	postive
ATG9A	LINC01578	0.366752427	2.30E-17	postive
ATG9A	LINC01679	0.33412881	1.66E-14	postive
ATG9A	LINC01703	0.30461269	3.39E-12	postive
ATG9A	LINC01719	0.369490699	1.28E-17	postive
ATG9A	LINC01772	0.306567127	2.43E-12	postive
ATG9A	LINC01806	0.436262424	1.20E-24	postive

ATG9A	LINC01881	0.550990523	4.82E-41	postive
ATG9A	LINC01963	0.360468247	8.68E-17	postive
ATG9A	LINC02012	0.378354472	1.83E-18	postive
ATG9A	LINC02014	0.314105297	6.54E-13	postive
ATG9A	LINC02026	0.319356353	2.56E-13	postive
ATG9A	LINC02035	0.466107112	2.47E-28	postive
ATG9A	LINC02193	0.352614955	4.38E-16	postive
ATG9A	LINC02210	0.494198188	3.76E-32	postive
ATG9A	LINC02256	0.320669291	2.02E-13	postive
ATG9A	LINC02298	0.32931081	4.13E-14	postive
ATG9A	LINC02361	0.306500401	2.46E-12	postive
ATG9A	LINC02449	0.317235294	3.75E-13	postive
ATG9A	LINC02569	0.312217094	9.12E-13	postive
ATG9A	LINC02585	0.391240182	9.78E-20	postive
ATG9A	LINC02604	0.311853995	9.71E-13	postive
ATG9A	LINC02693	0.457285114	3.32E-27	postive
ATG9A	LINC02709	0.311965247	9.53E-13	postive
ATG9A	LINC02818	0.338597406	7.06E-15	postive
ATG9A	LLPH-DT	0.334107511	1.67E-14	postive
ATG9A	LMCD1-AS1	0.328759397	4.57E-14	postive
ATG9A	LOH12CR2	0.366690327	2.33E-17	postive
ATG9A	LOXL1-AS1	0.40223547	7.22E-21	postive
ATG9A	LPP-AS2	0.360933912	7.88E-17	postive
ATG9A	LRRC37A16P	0.341687226	3.87E-15	postive
ATG9A	LRRC37A17P	0.519824025	5.89E-36	postive
ATG9A	LRRC37A6P	0.315287342	5.30E-13	postive
ATG9A	LRRC37BP1	0.58973349	3.66E-48	postive
ATG9A	LSP1P4	0.395355114	3.73E-20	postive
ATG9A	MAFG-DT	0.465744871	2.75E-28	postive
ATG9A	MAN1B1-DT	0.418971933	1.13E-22	postive
ATG9A	MAP2K4P1	0.417075886	1.83E-22	postive
ATG9A	MAP3K14-AS1	0.477096693	8.74E-30	postive
ATG9A	MAP3K2-DT	0.305643283	2.85E-12	postive
ATG9A	MAP4K3-DT	0.451006825	2.01E-26	postive
ATG9A	MAPKAPK5-AS1	0.525339501	8.09E-37	postive
ATG9A	MCM3AP-AS1	0.397223879	2.40E-20	postive
ATG9A	MCPH1-AS1	0.369688342	1.22E-17	postive
ATG9A	MED14OS	0.354150956	3.20E-16	postive
ATG9A	MED8-AS1	0.326268384	7.26E-14	postive
ATG9A	MFF-DT	0.40377856	4.97E-21	postive
ATG9A	MIATNB	0.375195188	3.69E-18	postive
ATG9A	MIF-AS1	0.354396959	3.05E-16	postive
ATG9A	MIPEPP3	0.342604921	3.24E-15	postive
ATG9A	MIR210HG	0.329416232	4.04E-14	postive
ATG9A	MIR22HG	0.402067596	7.52E-21	postive
ATG9A	MIR34AHG	0.308471461	1.75E-12	postive
ATG9A	MIR4435-2HG	0.355527034	2.42E-16	postive
ATG9A	MIR4453HG	0.375313726	3.59E-18	postive
ATG9A	MIR4713HG	0.362028214	6.26E-17	postive
ATG9A	MIR762HG	0.392800858	6.79E-20	postive

ATG9A	MKLN1-AS	0.453482996	9.92E-27	postive
ATG9A	MMADHC-DT	0.338237386	7.57E-15	postive
ATG9A	MMP25-AS1	0.332179361	2.41E-14	postive
ATG9A	MRPL20-AS1	0.603684624	5.72E-51	postive
ATG9A	MRPL45P2	0.352956717	4.09E-16	postive
ATG9A	MRPS31P4	0.378627257	1.73E-18	postive
ATG9A	MRPS31P5	0.311481862	1.04E-12	postive
ATG9A	MSH5-SAPCD1	0.314008169	6.65E-13	postive
ATG9A	MSL3P1	0.47320881	2.89E-29	postive
ATG9A	MTMR9LP	0.310398643	1.25E-12	postive
ATG9A	MUC20-OT1	0.337531076	8.67E-15	postive
ATG9A	MZF1-AS1	0.349524819	8.19E-16	postive
ATG9A	NACA4P	0.346034167	1.64E-15	postive
ATG9A	NAPA-AS1	0.399662097	1.34E-20	postive
ATG9A	NBPF25P	0.487318415	3.49E-31	postive
ATG9A	NBPF8	0.380685292	1.09E-18	postive
ATG9A	NBR2	0.5122308	8.54E-35	postive
ATG9A	NCK1-DT	0.35082008	6.31E-16	postive
ATG9A	NDUFB2-AS1	0.473838596	2.38E-29	postive
ATG9A	NDUFV2-AS1	0.31475289	5.83E-13	postive
ATG9A	NIFK-AS1	0.564213384	2.28E-43	postive
ATG9A	NINJ2-AS1	0.400342406	1.14E-20	postive
ATG9A	NNT-AS1	0.347340726	1.27E-15	postive
ATG9A	NOP14-AS1	0.600836416	2.20E-50	postive
ATG9A	NOP53-AS1	0.401921798	7.79E-21	postive
ATG9A	NORAD	0.636617884	3.56E-58	postive
ATG9A	NPEPPSP1	0.340580702	4.81E-15	postive
ATG9A	NPIPP1	0.309826691	1.38E-12	postive
ATG9A	NRAV	0.518929603	8.10E-36	postive
ATG9A	NRSN2-AS1	0.460961023	1.13E-27	postive
ATG9A	NUDT16P1	0.517929862	1.16E-35	postive
ATG9A	NUP50-DT	0.318869659	2.80E-13	postive
ATG9A	NUTM2A-AS1	0.61596982	1.48E-53	postive
ATG9A	NUTM2B-AS1	0.379794253	1.33E-18	postive
ATG9A	OGFR-AS1	0.332033095	2.47E-14	postive
ATG9A	OGFRP1	0.439330482	5.21E-25	postive
ATG9A	OIP5-AS1	0.612837327	6.92E-53	postive
ATG9A	OLMALINC	0.397847065	2.07E-20	postive
ATG9A	OR2A1-AS1	0.308691983	1.68E-12	postive
ATG9A	OR7E14P	0.402018656	7.61E-21	postive
ATG9A	OSER1-DT	0.391752883	8.68E-20	postive
ATG9A	OSGEPL1-AS1	0.302578311	4.79E-12	postive
ATG9A	OTUD6B-AS1	0.485668974	5.92E-31	postive
ATG9A	PARD6G-AS1	0.300782161	6.49E-12	postive
ATG9A	PARGP1	0.311435328	1.05E-12	postive
ATG9A	PAXIP1-AS1	0.330026734	3.61E-14	postive
ATG9A	PAXIP1-AS2	0.39376187	5.42E-20	postive
ATG9A	PCAT6	0.373000661	5.97E-18	postive
ATG9A	PCBP1-AS1	0.412675737	5.55E-22	postive
ATG9A	PCOTH	0.310223119	1.29E-12	postive

ATG9A	PDCD4-AS1	0.38415972	4.97E-19	postive
ATG9A	PDIA3P1	0.469251469	9.61E-29	postive
ATG9A	PDPK2P	0.375678471	3.32E-18	postive
ATG9A	PGAM1P8	0.421795161	5.48E-23	postive
ATG9A	PI4KAP2	0.330888185	3.07E-14	postive
ATG9A	PICART1	0.379283032	1.49E-18	postive
ATG9A	PINK1-AS	0.558657493	2.23E-42	postive
ATG9A	PIPSL	0.44574975	8.84E-26	postive
ATG9A	PITPNA-AS1	0.408057081	1.75E-21	postive
ATG9A	PITRM1-AS1	0.339978666	5.40E-15	postive
ATG9A	PKD1P6	0.418762593	1.19E-22	postive
ATG9A	PLBD1-AS1	0.333105551	2.02E-14	postive
ATG9A	PLEKHA8P1	0.571222226	1.21E-44	postive
ATG9A	PMS2CL	0.517978242	1.14E-35	postive
ATG9A	PMS2P3	0.391769685	8.64E-20	postive
ATG9A	PMS2P4	0.424631796	2.63E-23	postive
ATG9A	POLH-AS1	0.38600331	3.27E-19	postive
ATG9A	POLR2J4	0.47055647	6.48E-29	postive
ATG9A	PPP1R12A-AS1	0.317546689	3.55E-13	postive
ATG9A	PPP1R26-AS1	0.432214745	3.56E-24	postive
ATG9A	PPP3CB-AS1	0.372081837	7.29E-18	postive
ATG9A	PPT2-EGFL8	0.457305316	3.30E-27	postive
ATG9A	PRC1-AS1	0.303761353	3.92E-12	postive
ATG9A	PRELID1P1	0.37705958	2.44E-18	postive
ATG9A	PRKAR2A-AS1	0.374048048	4.74E-18	postive
ATG9A	PRKCZ-AS1	0.355000996	2.69E-16	postive
ATG9A	PRORSD1P	0.372047544	7.34E-18	postive
ATG9A	PRR34	0.307983576	1.90E-12	postive
ATG9A	PSMA3-AS1	0.313969794	6.70E-13	postive
ATG9A	PSMG3-AS1	0.307489245	2.07E-12	postive
ATG9A	PTENP1	0.44814427	4.52E-26	postive
ATG9A	PTMAP5	0.320990874	1.91E-13	postive
ATG9A	PTOV1-AS1	0.579114744	4.08E-46	postive
ATG9A	PTOV1-AS2	0.348397045	1.03E-15	postive
ATG9A	PXN-AS1	0.436654033	1.08E-24	postive
ATG9A	RAB11B-AS1	0.350601335	6.59E-16	postive
ATG9A	RAB30-DT	0.332204201	2.40E-14	postive
ATG9A	RAB4B-EGLN2	0.330076113	3.57E-14	postive
ATG9A	RALY-AS1	0.389846366	1.35E-19	postive
ATG9A	RAP2C-AS1	0.326165577	7.40E-14	postive
ATG9A	RARA-AS1	0.521470914	3.27E-36	postive
ATG9A	RASA4CP	0.463906899	4.76E-28	postive
ATG9A	RASAL2-AS1	0.394755732	4.30E-20	postive
ATG9A	RASSF1-AS1	0.32339622	1.23E-13	postive
ATG9A	RBM15-AS1	0.450022643	2.66E-26	postive
ATG9A	RHPN1-AS1	0.347375062	1.26E-15	postive
ATG9A	RNASEH1-AS1	0.381633525	8.80E-19	postive
ATG9A	RNF216P1	0.541097941	2.27E-39	postive
ATG9A	ROCK1P1	0.345235332	1.93E-15	postive
ATG9A	RP11-101E13.5	0.487991139	2.82E-31	postive

ATG9A	RP11-104N10.2	0.308464285	1.75E-12	postive
ATG9A	RP11-10C24.1	0.464401154	4.11E-28	postive
ATG9A	RP11-10C24.2	0.33596987	1.17E-14	postive
ATG9A	RP11-153F5.7	0.332939761	2.08E-14	postive
ATG9A	RP11-159G9.5	0.379218122	1.51E-18	postive
ATG9A	RP11-15N24.4	0.36301609	5.09E-17	postive
ATG9A	RP11-164P12.4	0.378122578	1.93E-18	postive
ATG9A	RP11-175O19.4	0.61493692	2.46E-53	postive
ATG9A	RP11-199F11.2	0.345102862	1.98E-15	postive
ATG9A	RP11-206L10.5	0.311021564	1.12E-12	postive
ATG9A	RP11-226L15.5	0.506182703	6.85E-34	postive
ATG9A	RP1-122P22.2	0.504088808	1.40E-33	postive
ATG9A	RP11-282O18.3	0.496750368	1.62E-32	postive
ATG9A	RP11-337C18.9	0.341849316	3.75E-15	postive
ATG9A	RP11-435O5.2	0.435095732	1.64E-24	postive
ATG9A	RP11-439L18.2	0.386382451	3.00E-19	postive
ATG9A	RP11-465N4.5	0.386324439	3.04E-19	postive
ATG9A	RP11-529E10.6	0.391053615	1.02E-19	postive
ATG9A	RP11-539I5.1	0.408449321	1.59E-21	postive
ATG9A	RP11-540B6.6	0.402506142	6.77E-21	postive
ATG9A	RP11-61L19.3	0.355237422	2.57E-16	postive
ATG9A	RP11-658F2.8	0.352354928	4.62E-16	postive
ATG9A	RP11-706O15.1	0.400224043	1.17E-20	postive
ATG9A	RP11-86H7.7	0.410387268	9.82E-22	postive
ATG9A	RP11-876N24.4	0.308722514	1.68E-12	postive
ATG9A	RP11-876N24.5	0.347624129	1.20E-15	postive
ATG9A	RP3-355L5.5	0.356764583	1.87E-16	postive
ATG9A	RP4-545L17.11	0.344590998	2.19E-15	postive
ATG9A	RP5-1039K5.19	0.335355091	1.32E-14	postive
ATG9A	RP5-1042I8.7	0.31918491	2.64E-13	postive
ATG9A	RP5-1112D6.4	0.372783577	6.26E-18	postive
ATG9A	RP5-1125A11.6	0.437249922	9.18E-25	postive
ATG9A	RP5-1125A11.7	0.383988406	5.17E-19	postive
ATG9A	RP5-1139B12.2	0.431518643	4.29E-24	postive
ATG9A	RP9P	0.39017101	1.25E-19	postive
ATG9A	RPARP-AS1	0.371494529	8.28E-18	postive
ATG9A	RPL13AP7	0.314142061	6.50E-13	postive
ATG9A	RPL13P5	0.57983389	2.98E-46	postive
ATG9A	RPL23AP53	0.445134088	1.05E-25	postive
ATG9A	RPL23AP7	0.490861771	1.12E-31	postive
ATG9A	RPL23AP82	0.491107519	1.03E-31	postive
ATG9A	RPL32P3	0.353617813	3.57E-16	postive
ATG9A	RPL6P27	0.350883086	6.23E-16	postive
ATG9A	RPS10P7	0.400267468	1.16E-20	postive
ATG9A	RPS18P9	0.427463757	1.25E-23	postive
ATG9A	RPS4XP16	0.372664375	6.42E-18	postive
ATG9A	RRN3P3	0.49599482	2.08E-32	postive
ATG9A	RRP7BP	0.445130387	1.05E-25	postive
ATG9A	RUSC1-AS1	0.342274157	3.45E-15	postive
ATG9A	SAMD12-AS1	0.302880748	4.55E-12	postive

ATG9A	SBDSP1	0.494784302	3.10E-32	postive
ATG9A	SBF2-AS1	0.387285586	2.44E-19	postive
ATG9A	SCAMP1-AS1	0.34814597	1.08E-15	postive
ATG9A	SCAND2P	0.45439587	7.64E-27	postive
ATG9A	SDAD1P1	0.414115676	3.87E-22	postive
ATG9A	SDCBP2-AS1	0.353036519	4.02E-16	postive
ATG9A	SDHAP1	0.458378357	2.41E-27	postive
ATG9A	SEC24B-AS1	0.330463853	3.32E-14	postive
ATG9A	SEMA3F-AS1	0.300259596	7.08E-12	postive
ATG9A	SEPSECS-AS1	0.368236994	1.67E-17	postive
ATG9A	SEPTIN4-AS1	0.344141291	2.39E-15	postive
ATG9A	SEPTIN7P2	0.349066196	8.98E-16	postive
ATG9A	SERHL	0.418631725	1.23E-22	postive
ATG9A	SGMS1-AS1	0.400230493	1.17E-20	postive
ATG9A	SH3GL1P1	0.358714001	1.25E-16	postive
ATG9A	SH3RF3-AS1	0.352809956	4.21E-16	postive
ATG9A	SHLD2P1	0.346959051	1.37E-15	postive
ATG9A	SLC25A25-AS1	0.333162037	2.00E-14	postive
ATG9A	SLC25A5-AS1	0.322136774	1.55E-13	postive
ATG9A	SLC2A1-AS1	0.328024901	5.24E-14	postive
ATG9A	SLC44A3-AS1	0.409683223	1.17E-21	postive
ATG9A	SLC9A7P1	0.331954263	2.51E-14	postive
ATG9A	SLX1A-SULT1A3	0.334887012	1.44E-14	postive
ATG9A	SMG7-AS1	0.452885923	1.18E-26	postive
ATG9A	SNAI3-AS1	0.426125416	1.78E-23	postive
ATG9A	SNHG1	0.324236022	1.06E-13	postive
ATG9A	SNHG11	0.518426226	9.69E-36	postive
ATG9A	SNHG12	0.338178163	7.66E-15	postive
ATG9A	SNHG16	0.505652013	8.21E-34	postive
ATG9A	SNHG17	0.402160294	7.35E-21	postive
ATG9A	SNHG20	0.412816071	5.36E-22	postive
ATG9A	SNHG21	0.373662053	5.16E-18	postive
ATG9A	SNHG28	0.388081709	2.03E-19	postive
ATG9A	SNHG3	0.300524014	6.77E-12	postive
ATG9A	SNHG31	0.306947369	2.28E-12	postive
ATG9A	SNHG32	0.399835457	1.29E-20	postive
ATG9A	SNHG7	0.43986537	4.50E-25	postive
ATG9A	SNHG8	0.362057752	6.22E-17	postive
ATG9A	SP2-AS1	0.357574174	1.59E-16	postive
ATG9A	SPAG5-AS1	0.39948987	1.40E-20	postive
ATG9A	SPINT1-AS1	0.376722596	2.63E-18	postive
ATG9A	SREBF2-AS1	0.471461067	4.92E-29	postive
ATG9A	SRP14-AS1	0.33496106	1.42E-14	postive
ATG9A	SSR4P1	0.363409731	4.68E-17	postive
ATG9A	ST20-AS1	0.520817984	4.13E-36	postive
ATG9A	STAG3L1	0.332781211	2.15E-14	postive
ATG9A	STAG3L2	0.423762827	3.29E-23	postive
ATG9A	STAG3L3	0.402698131	6.46E-21	postive
ATG9A	STAG3L4	0.332896635	2.10E-14	postive
ATG9A	STAG3L5P	0.339054774	6.46E-15	postive

ATG9A	STAG3L5P-PVRIG2P-PILRB	0.358519022	1.30E-16	postive
ATG9A	STARD7-AS1	0.66075341	4.86E-64	postive
ATG9A	STK4-AS1	0.383914386	5.26E-19	postive
ATG9A	STX17-AS1	0.344467251	2.24E-15	postive
ATG9A	STX18-AS1	0.461666126	9.22E-28	postive
ATG9A	STXBP5-AS1	0.311873742	9.68E-13	postive
ATG9A	SUCLA2-AS1	0.380993799	1.02E-18	postive
ATG9A	SUCLG2-AS1	0.405551316	3.23E-21	postive
ATG9A	SUMO2P17	0.442531734	2.16E-25	postive
ATG9A	SUZ12P1	0.392371038	7.51E-20	postive
ATG9A	SVIL2P	0.343671869	2.62E-15	postive
ATG9A	SVIL-AS1	0.626168935	8.54E-56	postive
ATG9A	SYS1-DBNDD2	0.359970869	9.63E-17	postive
ATG9A	TAF1A-AS1	0.355278507	2.54E-16	postive
ATG9A	TAPT1-AS1	0.430936003	5.01E-24	postive
ATG9A	TBC1D8-AS1	0.389883477	1.34E-19	postive
ATG9A	TDRKH-AS1	0.341708808	3.86E-15	postive
ATG9A	TEKT4P2	0.33566474	1.24E-14	postive
ATG9A	THAP9-AS1	0.481367657	2.31E-30	postive
ATG9A	TMCC1-AS1	0.311190267	1.09E-12	postive
ATG9A	TMED2-DT	0.347685943	1.18E-15	postive
ATG9A	TMEM147-AS1	0.350282369	7.03E-16	postive
ATG9A	TMEM198B	0.410301961	1.00E-21	postive
ATG9A	TMEM202-AS1	0.445675225	9.03E-26	postive
ATG9A	TMEM44-AS1	0.345386969	1.87E-15	postive
ATG9A	TMEM99	0.41923158	1.06E-22	postive
ATG9A	TMEM9B-AS1	0.319893945	2.33E-13	postive
ATG9A	TNFRSF10A-AS1	0.338511558	7.18E-15	postive
ATG9A	TNRC6C-AS1	0.393599888	5.63E-20	postive
ATG9A	TOB1-AS1	0.436551807	1.11E-24	postive
ATG9A	TOLLIP-AS1	0.341038942	4.39E-15	postive
ATG9A	TONSL-AS1	0.392976768	6.52E-20	postive
ATG9A	TP53TG1	0.301117038	6.13E-12	postive
ATG9A	TP73-AS1	0.499796663	5.91E-33	postive
ATG9A	TPM3P9	0.394863956	4.19E-20	postive
ATG9A	TPT1-AS1	0.398971674	1.58E-20	postive
ATG9A	TPTEP2	0.321734324	1.67E-13	postive
ATG9A	TRAM2-AS1	0.458566167	2.29E-27	postive
ATG9A	TRAPPC12-AS1	0.509883163	1.93E-34	postive
ATG9A	TRIM52-AS1	0.356235581	2.09E-16	postive
ATG9A	TSC22D1-AS1	0.310131695	1.31E-12	postive
ATG9A	TSPY26P	0.34952153	8.19E-16	postive
ATG9A	TSTD3	0.445378588	9.81E-26	postive
ATG9A	TTC28-AS1	0.48499819	7.32E-31	postive
ATG9A	TTC41P	0.314461286	6.14E-13	postive
ATG9A	TTN-AS1	0.358858569	1.21E-16	postive
ATG9A	TTTY15	0.315956327	4.71E-13	postive
ATG9A	TUBBP1	0.332274725	2.36E-14	postive
ATG9A	TUBBP5	0.330898507	3.06E-14	postive
ATG9A	TUG1	0.630789192	7.77E-57	postive

ATG9A	U47924.2	0.379307847	1.48E-18	postive
ATG9A	U91328.1	0.404983474	3.71E-21	postive
ATG9A	UBA6-AS1	0.55497414	9.86E-42	postive
ATG9A	UBAC2-AS1	0.321192327	1.84E-13	postive
ATG9A	UBE2D3-AS1	0.462977491	6.26E-28	postive
ATG9A	UBE2Q1-AS1	0.320148691	2.22E-13	postive
ATG9A	UBE2Q2P1	0.348456999	1.01E-15	postive
ATG9A	UBR5-AS1	0.357359184	1.66E-16	postive
ATG9A	UGDH-AS1	0.376247277	2.92E-18	postive
ATG9A	UPF3AP2	0.41412468	3.86E-22	postive
ATG9A	URAHP	0.33650057	1.06E-14	postive
ATG9A	URB1-AS1	0.308334473	1.79E-12	postive
ATG9A	USP27X-AS1	0.403963495	4.75E-21	postive
ATG9A	USP46-AS1	0.485720453	5.82E-31	postive
ATG9A	UXT-AS1	0.38841112	1.88E-19	postive
ATG9A	VASH1-AS1	0.378833702	1.65E-18	postive
ATG9A	VDAC1P8	0.317821409	3.38E-13	postive
ATG9A	VPS13B-DT	0.309900769	1.37E-12	postive
ATG9A	VPS33B-DT	0.314769446	5.81E-13	postive
ATG9A	WAC-AS1	0.583791226	5.23E-47	postive
ATG9A	WARS2-AS1	0.436006439	1.29E-24	postive
ATG9A	WASH3P	0.491303911	9.66E-32	postive
ATG9A	WASIR2	0.317701571	3.45E-13	postive
ATG9A	WHAMMP3	0.346809473	1.41E-15	postive
ATG9A	WNT5A-AS1	0.329816179	3.75E-14	postive
ATG9A	WWC2-AS2	0.366844928	2.26E-17	postive
ATG9A	Z68871.1	0.301215346	6.03E-12	postive
ATG9A	Z83851.4	0.434986148	1.69E-24	postive
ATG9A	Z92544.2	0.384600124	4.50E-19	postive
ATG9A	Z95115.1	0.383644032	5.59E-19	postive
ATG9A	Z97634.1	0.393599565	5.64E-20	postive
ATG9A	Z98884.2	0.352042789	4.92E-16	postive
ATG9A	Z99572.1	0.406859683	2.34E-21	postive
ATG9A	ZBED5-AS1	0.355870276	2.25E-16	postive
ATG9A	ZBTB11-AS1	0.5341524	3.14E-38	postive
ATG9A	ZBTB44-DT	0.311142214	1.10E-12	postive
ATG9A	ZEB1-AS1	0.349211556	8.72E-16	postive
ATG9A	ZNF137P	0.426970859	1.43E-23	postive
ATG9A	ZNF205-AS1	0.370083382	1.12E-17	postive
ATG9A	ZNF213-AS1	0.387466972	2.34E-19	postive
ATG9A	ZNF236-DT	0.305647145	2.84E-12	postive
ATG9A	ZNF252P	0.470648543	6.30E-29	postive
ATG9A	ZNF271P	0.448631904	3.94E-26	postive
ATG9A	ZNF286B	0.306558328	2.43E-12	postive
ATG9A	ZNF337-AS1	0.370168219	1.10E-17	postive
ATG9A	ZNF341-AS1	0.389737435	1.39E-19	postive
ATG9A	ZNF37BP	0.315355829	5.24E-13	postive
ATG9A	ZNF436-AS1	0.325338908	8.62E-14	postive
ATG9A	ZNF503-AS2	0.488951518	2.07E-31	postive
ATG9A	ZNF528-AS1	0.307776778	1.97E-12	postive

ATG9A	ZNF529-AS1	0.395439508	3.66E-20	postive
ATG9A	ZNF542P	0.368081195	1.73E-17	postive
ATG9A	ZNF56	0.343044669	2.97E-15	postive
ATG9A	ZNF561-AS1	0.401069435	9.56E-21	postive
ATG9A	ZNF649-AS1	0.311545822	1.03E-12	postive
ATG9A	ZNF667-AS1	0.324740436	9.62E-14	postive
ATG9A	ZNF674-AS1	0.316041515	4.64E-13	postive
ATG9A	ZNF687-AS1	0.407647786	1.93E-21	postive
ATG9A	ZNF767P	0.359101059	1.15E-16	postive
ATG9A	ZNF790-AS1	0.302382023	4.95E-12	postive
ATG9A	ZNF815P	0.396483701	2.86E-20	postive
ATG9A	ZNNT1	0.318245139	3.13E-13	postive
ATG9A	ZNRD1ASP	0.321230272	1.83E-13	postive
ATG9A	ZNRD2-AS1	0.462896863	6.41E-28	postive
ATG9A	ZNRF2P1	0.371837137	7.69E-18	postive
ATG9A	ZNRF2P2	0.308262792	1.81E-12	postive
ATG9A	ZSCAN12P1	0.415126646	3.00E-22	postive
ATG9A	ZSCAN16-AS1	0.313168502	7.71E-13	postive
ATOX1	A1BG-AS1	0.314626917	5.96E-13	postive
ATOX1	A2M-AS1	0.314360792	6.25E-13	postive
ATOX1	ABHD11-AS1	0.318777168	2.84E-13	postive
ATOX1	AC000068.1	0.325912334	7.75E-14	postive
ATOX1	AC002091.1	0.309366489	1.50E-12	postive
ATOX1	AC002116.2	0.403936723	4.78E-21	postive
ATOX1	AC002310.1	0.300939208	6.32E-12	postive
ATOX1	AC002398.1	0.30014018	7.22E-12	postive
ATOX1	AC002467.1	0.54901338	1.05E-40	postive
ATOX1	AC002470.1	0.361535438	6.94E-17	postive
ATOX1	AC004241.1	0.486155937	5.07E-31	postive
ATOX1	AC004241.3	0.337114083	9.40E-15	postive
ATOX1	AC004447.2	0.302165397	5.14E-12	postive
ATOX1	AC004477.1	0.331353234	2.81E-14	postive
ATOX1	AC004540.1	0.315336227	5.26E-13	postive
ATOX1	AC004585.1	0.416020433	2.39E-22	postive
ATOX1	AC004596.1	0.302616125	4.76E-12	postive
ATOX1	AC004771.2	0.305458141	2.94E-12	postive
ATOX1	AC004801.6	0.370773065	9.69E-18	postive
ATOX1	AC004812.2	0.369526625	1.27E-17	postive
ATOX1	AC004854.2	0.324595408	9.88E-14	postive
ATOX1	AC004865.2	0.341132881	4.31E-15	postive
ATOX1	AC004921.1	0.422515704	4.55E-23	postive
ATOX1	AC004943.2	0.331297529	2.84E-14	postive
ATOX1	AC004988.1	0.325122346	8.97E-14	postive
ATOX1	AC005034.4	0.343834074	2.54E-15	postive
ATOX1	AC005037.1	0.312439545	8.77E-13	postive
ATOX1	AC005050.1	0.319467875	2.51E-13	postive
ATOX1	AC005076.2	0.366167152	2.61E-17	postive
ATOX1	AC005229.4	0.370273646	1.08E-17	postive
ATOX1	AC005261.4	0.340205418	5.17E-15	postive
ATOX1	AC005332.4	0.371450398	8.36E-18	postive

ATOX1	AC005498.2	0.304770253	3.30E-12	postive
ATOX1	AC005520.4	0.383427944	5.87E-19	postive
ATOX1	AC005562.1	0.378478608	1.78E-18	postive
ATOX1	AC005670.3	0.368610255	1.55E-17	postive
ATOX1	AC005837.1	0.30043762	6.87E-12	postive
ATOX1	AC006001.2	0.354884449	2.76E-16	postive
ATOX1	AC006033.2	0.358950805	1.19E-16	postive
ATOX1	AC006122.1	0.307263606	2.16E-12	postive
ATOX1	AC006213.1	0.354342504	3.08E-16	postive
ATOX1	AC006333.1	0.417650791	1.58E-22	postive
ATOX1	AC006449.7	0.488943401	2.07E-31	postive
ATOX1	AC006504.5	0.323817863	1.14E-13	postive
ATOX1	AC006547.1	0.31530098	5.29E-13	postive
ATOX1	AC007032.1	0.359555715	1.05E-16	postive
ATOX1	AC007114.1	0.337672039	8.44E-15	postive
ATOX1	AC007228.9	0.303722007	3.95E-12	postive
ATOX1	AC007383.2	0.35099201	6.09E-16	postive
ATOX1	AC007728.2	0.338508076	7.18E-15	postive
ATOX1	AC007773.1	0.374034684	4.76E-18	postive
ATOX1	AC007998.3	0.418494326	1.28E-22	postive
ATOX1	AC008014.1	0.413921168	4.06E-22	postive
ATOX1	AC008035.1	0.351077256	5.99E-16	postive
ATOX1	AC008083.3	0.316512605	4.27E-13	postive
ATOX1	AC008105.2	0.340835283	4.57E-15	postive
ATOX1	AC008267.5	0.336196747	1.12E-14	postive
ATOX1	AC008393.1	0.379930206	1.29E-18	postive
ATOX1	AC008438.1	0.44254207	2.16E-25	postive
ATOX1	AC008443.1	0.483870098	1.05E-30	postive
ATOX1	AC008443.5	0.414352717	3.65E-22	postive
ATOX1	AC008494.3	0.513212952	6.07E-35	postive
ATOX1	AC008608.2	0.450192518	2.53E-26	postive
ATOX1	AC008610.1	0.354653158	2.89E-16	postive
ATOX1	AC008747.1	0.31483411	5.75E-13	postive
ATOX1	AC008750.1	0.317671495	3.47E-13	postive
ATOX1	AC008758.4	0.324560183	9.95E-14	postive
ATOX1	AC008771.1	0.429013741	8.33E-24	postive
ATOX1	AC008915.3	0.515680093	2.56E-35	postive
ATOX1	AC008946.1	0.356045722	2.17E-16	postive
ATOX1	AC008966.1	0.438657357	6.26E-25	postive
ATOX1	AC009005.1	0.300604799	6.68E-12	postive
ATOX1	AC009061.2	0.478087828	6.42E-30	postive
ATOX1	AC009065.9	0.305298721	3.02E-12	postive
ATOX1	AC009126.1	0.456535559	4.12E-27	postive
ATOX1	AC009171.2	0.376538605	2.74E-18	postive
ATOX1	AC009237.14	0.387808545	2.16E-19	postive
ATOX1	AC009275.1	0.32209472	1.56E-13	postive
ATOX1	AC009309.2	0.37508573	3.78E-18	postive
ATOX1	AC009318.2	0.306062124	2.65E-12	postive
ATOX1	AC009403.1	0.316457208	4.31E-13	postive
ATOX1	AC009506.2	0.343883873	2.52E-15	postive

ATOX1	AC009690.2	0.33513855	1.37E-14	postive
ATOX1	AC009779.3	0.44682068	6.55E-26	postive
ATOX1	AC009831.1	0.441112725	3.20E-25	postive
ATOX1	AC010173.1	0.381042726	1.01E-18	postive
ATOX1	AC010186.2	0.370529663	1.02E-17	postive
ATOX1	AC010226.1	0.413719135	4.28E-22	postive
ATOX1	AC010247.1	0.345492624	1.83E-15	postive
ATOX1	AC010280.2	0.348460527	1.01E-15	postive
ATOX1	AC010319.4	0.301580918	5.67E-12	postive
ATOX1	AC010320.2	0.325017548	9.14E-14	postive
ATOX1	AC010359.2	0.300304767	7.03E-12	postive
ATOX1	AC010442.1	0.354915408	2.74E-16	postive
ATOX1	AC010504.1	0.447616203	5.24E-26	postive
ATOX1	AC010531.6	0.318420269	3.03E-13	postive
ATOX1	AC010655.4	0.346041266	1.64E-15	postive
ATOX1	AC010894.2	0.330882881	3.07E-14	postive
ATOX1	AC010913.1	0.306521543	2.45E-12	postive
ATOX1	AC010969.2	0.345845189	1.71E-15	postive
ATOX1	AC011374.2	0.380854286	1.05E-18	postive
ATOX1	AC011451.1	0.38561986	3.57E-19	postive
ATOX1	AC011603.3	0.350439684	6.81E-16	postive
ATOX1	AC011731.1	0.305738353	2.80E-12	postive
ATOX1	AC011899.2	0.323267766	1.26E-13	postive
ATOX1	AC012063.1	0.333288423	1.95E-14	postive
ATOX1	AC012146.1	0.337268874	9.12E-15	postive
ATOX1	AC012184.3	0.360738427	8.21E-17	postive
ATOX1	AC012306.2	0.406045864	2.86E-21	postive
ATOX1	AC012360.2	0.363509127	4.58E-17	postive
ATOX1	AC012442.3	0.408830623	1.44E-21	postive
ATOX1	AC012603.1	0.413540675	4.47E-22	postive
ATOX1	AC012615.1	0.330307762	3.42E-14	postive
ATOX1	AC012640.4	0.415906575	2.46E-22	postive
ATOX1	AC015819.1	0.442546663	2.15E-25	postive
ATOX1	AC015912.3	0.305236289	3.05E-12	postive
ATOX1	AC015922.3	0.330683923	3.19E-14	postive
ATOX1	AC015982.2	0.415668937	2.62E-22	postive
ATOX1	AC016027.1	0.42477457	2.53E-23	postive
ATOX1	AC016065.1	0.384886379	4.22E-19	postive
ATOX1	AC016394.2	0.420327965	7.99E-23	postive
ATOX1	AC016405.3	0.364434992	3.77E-17	postive
ATOX1	AC016727.1	0.310642954	1.20E-12	postive
ATOX1	AC016747.1	0.445142896	1.05E-25	postive
ATOX1	AC016876.2	0.417661486	1.58E-22	postive
ATOX1	AC016876.3	0.438415174	6.69E-25	postive
ATOX1	AC016888.1	0.403716607	5.05E-21	postive
ATOX1	AC018521.5	0.564091849	2.40E-43	postive
ATOX1	AC018521.6	0.319842714	2.35E-13	postive
ATOX1	AC019069.1	0.395681339	3.45E-20	postive
ATOX1	AC020663.2	0.35754167	1.60E-16	postive
ATOX1	AC020904.2	0.318640479	2.91E-13	postive

ATOX1	AC020910.5	0.386285416	3.06E-19	postive
ATOX1	AC020915.3	0.41031107	1.00E-21	postive
ATOX1	AC021016.2	0.335275022	1.34E-14	postive
ATOX1	AC021028.1	0.359292285	1.11E-16	postive
ATOX1	AC021188.1	0.359703312	1.02E-16	postive
ATOX1	AC022092.1	0.417387947	1.69E-22	postive
ATOX1	AC022098.1	0.348743852	9.58E-16	postive
ATOX1	AC022101.1	0.331423116	2.78E-14	postive
ATOX1	AC022167.4	0.375497742	3.45E-18	postive
ATOX1	AC022217.4	0.311845607	9.73E-13	postive
ATOX1	AC022509.4	0.311444376	1.04E-12	postive
ATOX1	AC022613.2	0.331793252	2.59E-14	postive
ATOX1	AC022706.1	0.369339562	1.32E-17	postive
ATOX1	AC022915.1	0.302426977	4.92E-12	postive
ATOX1	AC022916.2	0.316328935	4.41E-13	postive
ATOX1	AC023043.1	0.344482183	2.24E-15	postive
ATOX1	AC023509.3	0.328752636	4.58E-14	postive
ATOX1	AC023813.3	0.343399554	2.77E-15	postive
ATOX1	AC024243.1	0.323640289	1.18E-13	postive
ATOX1	AC024267.7	0.309126524	1.56E-12	postive
ATOX1	AC024575.2	0.361285728	7.32E-17	postive
ATOX1	AC025048.2	0.318048179	3.24E-13	postive
ATOX1	AC025048.4	0.462371517	7.49E-28	postive
ATOX1	AC025181.2	0.307241243	2.16E-12	postive
ATOX1	AC025822.2	0.318121569	3.20E-13	postive
ATOX1	AC025857.2	0.310563395	1.22E-12	postive
ATOX1	AC026304.1	0.386573601	2.87E-19	postive
ATOX1	AC026401.3	0.332852119	2.12E-14	postive
ATOX1	AC026471.1	0.393066506	6.38E-20	postive
ATOX1	AC026691.1	0.402772976	6.34E-21	postive
ATOX1	AC027097.1	0.334256101	1.62E-14	postive
ATOX1	AC027307.2	0.362298397	5.92E-17	postive
ATOX1	AC027319.1	0.365200384	3.20E-17	postive
ATOX1	AC027559.1	0.344579059	2.19E-15	postive
ATOX1	AC027644.3	0.345665059	1.77E-15	postive
ATOX1	AC027682.6	0.327653266	5.62E-14	postive
ATOX1	AC034199.1	0.487924127	2.88E-31	postive
ATOX1	AC034236.2	0.347937699	1.13E-15	postive
ATOX1	AC036176.1	0.418403293	1.31E-22	postive
ATOX1	AC040970.1	0.356106348	2.15E-16	postive
ATOX1	AC040977.1	0.300004897	7.39E-12	postive
ATOX1	AC046134.2	0.458156006	2.57E-27	postive
ATOX1	AC046143.1	0.34855701	9.94E-16	postive
ATOX1	AC046143.2	0.319346511	2.57E-13	postive
ATOX1	AC048382.5	0.300557289	6.74E-12	postive
ATOX1	AC051619.6	0.313182005	7.69E-13	postive
ATOX1	AC053503.4	0.364086918	4.06E-17	postive
ATOX1	AC060766.7	0.340810743	4.59E-15	postive
ATOX1	AC061992.2	0.302101165	5.19E-12	postive
ATOX1	AC064807.1	0.425959986	1.86E-23	postive

ATOX1	AC067852.2	0.516086769	2.22E-35	postive
ATOX1	AC068338.2	0.459800093	1.59E-27	postive
ATOX1	AC068580.3	0.321785724	1.65E-13	postive
ATOX1	AC068620.1	0.314703843	5.88E-13	postive
ATOX1	AC068790.8	0.377873519	2.04E-18	postive
ATOX1	AC068870.2	0.431584044	4.21E-24	postive
ATOX1	AC069209.1	0.328071428	5.20E-14	postive
ATOX1	AC069213.1	0.306553568	2.43E-12	postive
ATOX1	AC069307.1	0.32938645	4.07E-14	postive
ATOX1	AC073332.1	0.342129166	3.55E-15	postive
ATOX1	AC073508.3	0.361200257	7.45E-17	postive
ATOX1	AC073529.1	0.372358067	6.86E-18	postive
ATOX1	AC073569.2	0.329296405	4.14E-14	postive
ATOX1	AC073611.1	0.50783103	3.90E-34	postive
ATOX1	AC073896.2	0.510338623	1.65E-34	postive
ATOX1	AC073896.4	0.310355795	1.26E-12	postive
ATOX1	AC074044.1	0.325921291	7.74E-14	postive
ATOX1	AC078883.1	0.349237985	8.67E-16	postive
ATOX1	AC079209.1	0.359456525	1.07E-16	postive
ATOX1	AC079807.1	0.302623419	4.76E-12	postive
ATOX1	AC079848.1	0.337540169	8.66E-15	postive
ATOX1	AC079922.2	0.433452057	2.56E-24	postive
ATOX1	AC080013.5	0.300675637	6.60E-12	postive
ATOX1	AC080112.2	0.425240146	2.24E-23	postive
ATOX1	AC083799.1	0.478390587	5.85E-30	postive
ATOX1	AC083843.1	0.301164782	6.08E-12	postive
ATOX1	AC083862.2	0.358590361	1.28E-16	postive
ATOX1	AC084757.3	0.332510004	2.26E-14	postive
ATOX1	AC084809.1	0.320065935	2.25E-13	postive
ATOX1	AC087521.3	0.320319352	2.15E-13	postive
ATOX1	AC087627.1	0.31123183	1.08E-12	postive
ATOX1	AC087645.2	0.393699413	5.50E-20	postive
ATOX1	AC087672.2	0.341446279	4.06E-15	postive
ATOX1	AC087741.2	0.305106315	3.12E-12	postive
ATOX1	AC090114.2	0.313528863	7.24E-13	postive
ATOX1	AC090125.1	0.302414255	4.93E-12	postive
ATOX1	AC090204.1	0.368227881	1.68E-17	postive
ATOX1	AC090409.1	0.341391846	4.10E-15	postive
ATOX1	AC090515.3	0.386411546	2.98E-19	postive
ATOX1	AC090559.1	0.335518163	1.28E-14	postive
ATOX1	AC090587.1	0.486532332	4.49E-31	postive
ATOX1	AC090617.5	0.385311644	3.83E-19	postive
ATOX1	AC090825.1	0.405920132	2.95E-21	postive
ATOX1	AC090912.1	0.432260133	3.52E-24	postive
ATOX1	AC090970.2	0.313888366	6.79E-13	postive
ATOX1	AC091271.1	0.314830627	5.75E-13	postive
ATOX1	AC091390.5	0.352317366	4.66E-16	postive
ATOX1	AC091729.3	0.425505533	2.09E-23	postive
ATOX1	AC091982.1	0.307417663	2.10E-12	postive
ATOX1	AC091982.3	0.501108024	3.81E-33	postive

ATOX1	AC092115.4	0.326933924	6.42E-14	postive
ATOX1	AC092295.2	0.350802607	6.33E-16	postive
ATOX1	AC092354.1	0.305888392	2.73E-12	postive
ATOX1	AC092354.2	0.342049147	3.61E-15	postive
ATOX1	AC092368.3	0.438277388	6.94E-25	postive
ATOX1	AC092718.4	0.364500647	3.72E-17	postive
ATOX1	AC092903.2	0.350035032	7.39E-16	postive
ATOX1	AC092910.3	0.326749158	6.64E-14	postive
ATOX1	AC093157.1	0.377500369	2.22E-18	postive
ATOX1	AC093520.1	0.338580132	7.08E-15	postive
ATOX1	AC093627.5	0.306040289	2.66E-12	postive
ATOX1	AC093627.6	0.304426015	3.50E-12	postive
ATOX1	AC093673.1	0.525779405	6.90E-37	postive
ATOX1	AC093726.1	0.346253298	1.57E-15	postive
ATOX1	AC096536.2	0.327968216	5.30E-14	postive
ATOX1	AC096677.1	0.328226244	5.05E-14	postive
ATOX1	AC096733.3	0.349684329	7.93E-16	postive
ATOX1	AC097359.2	0.344304698	2.32E-15	postive
ATOX1	AC097468.3	0.377345459	2.29E-18	postive
ATOX1	AC098484.2	0.329430008	4.03E-14	postive
ATOX1	AC098487.1	0.437966494	7.56E-25	postive
ATOX1	AC098820.2	0.342780047	3.13E-15	postive
ATOX1	AC099066.2	0.364463909	3.74E-17	postive
ATOX1	AC099343.4	0.415120251	3.01E-22	postive
ATOX1	AC099522.1	0.304929241	3.22E-12	postive
ATOX1	AC099522.2	0.314187619	6.44E-13	postive
ATOX1	AC099548.2	0.412646104	5.59E-22	postive
ATOX1	AC099778.1	0.325004045	9.17E-14	postive
ATOX1	AC099850.2	0.414024185	3.96E-22	postive
ATOX1	AC100810.1	0.394269375	4.82E-20	postive
ATOX1	AC100849.1	0.30722244	2.17E-12	postive
ATOX1	AC100861.1	0.421959364	5.25E-23	postive
ATOX1	AC103760.1	0.318356468	3.07E-13	postive
ATOX1	AC104109.2	0.385086635	4.03E-19	postive
ATOX1	AC104113.1	0.32929924	4.13E-14	postive
ATOX1	AC104653.1	0.48295496	1.40E-30	postive
ATOX1	AC104667.2	0.364729259	3.54E-17	postive
ATOX1	AC104794.3	0.31514201	5.44E-13	postive
ATOX1	AC105036.3	0.349120282	8.88E-16	postive
ATOX1	AC105285.1	0.355946193	2.22E-16	postive
ATOX1	AC105942.1	0.345053072	2.00E-15	postive
ATOX1	AC106786.2	0.311308075	1.07E-12	postive
ATOX1	AC106791.1	0.329222732	4.19E-14	postive
ATOX1	AC106795.1	0.594975003	3.36E-49	postive
ATOX1	AC106897.2	0.30809127	1.87E-12	postive
ATOX1	AC107032.2	0.388693489	1.76E-19	postive
ATOX1	AC107214.1	0.364310721	3.87E-17	postive
ATOX1	AC107294.2	0.338921171	6.63E-15	postive
ATOX1	AC107959.3	0.313291956	7.55E-13	postive
ATOX1	AC108047.1	0.313122389	7.77E-13	postive

ATOX1	AC108488.1	0.342357978	3.40E-15	postive
ATOX1	AC109322.1	0.344756773	2.12E-15	postive
ATOX1	AC109347.1	0.467831445	1.47E-28	postive
ATOX1	AC109454.2	0.331082543	2.96E-14	postive
ATOX1	AC110995.1	0.412990856	5.13E-22	postive
ATOX1	AC112220.2	0.415315943	2.86E-22	postive
ATOX1	AC112715.1	0.374731646	4.08E-18	postive
ATOX1	AC114271.1	0.509155533	2.48E-34	postive
ATOX1	AC115618.1	0.332817604	2.13E-14	postive
ATOX1	AC115618.2	0.318904405	2.78E-13	postive
ATOX1	AC116407.2	0.351438832	5.56E-16	postive
ATOX1	AC116563.1	0.359633567	1.03E-16	postive
ATOX1	AC116913.1	0.404555487	4.12E-21	postive
ATOX1	AC118553.1	0.300131585	7.23E-12	postive
ATOX1	AC119044.1	0.306390521	2.50E-12	postive
ATOX1	AC124016.2	0.384829407	4.27E-19	postive
ATOX1	AC124248.1	0.314277206	6.34E-13	postive
ATOX1	AC124947.1	0.320431406	2.11E-13	postive
ATOX1	AC125257.1	0.362136001	6.12E-17	postive
ATOX1	AC130343.2	0.313316894	7.51E-13	postive
ATOX1	AC131097.3	0.307759148	1.98E-12	postive
ATOX1	AC131235.3	0.314069927	6.58E-13	postive
ATOX1	AC132008.2	0.361300971	7.29E-17	postive
ATOX1	AC132938.4	0.38445695	4.65E-19	postive
ATOX1	AC133552.2	0.345231967	1.93E-15	postive
ATOX1	AC133552.5	0.321074524	1.88E-13	postive
ATOX1	AC134312.5	0.300819747	6.45E-12	postive
ATOX1	AC135048.1	0.372899902	6.10E-18	postive
ATOX1	AC135048.3	0.348577692	9.90E-16	postive
ATOX1	AC135050.1	0.39969026	1.33E-20	postive
ATOX1	AC136604.3	0.499529879	6.46E-33	postive
ATOX1	AC138207.2	0.348414908	1.02E-15	postive
ATOX1	AC138207.5	0.346180498	1.60E-15	postive
ATOX1	AC138207.8	0.301368503	5.88E-12	postive
ATOX1	AC138696.2	0.335303544	1.33E-14	postive
ATOX1	AC139768.1	0.375300719	3.60E-18	postive
ATOX1	AC139795.3	0.354286677	3.12E-16	postive
ATOX1	AC141586.1	0.347454734	1.24E-15	postive
ATOX1	AC144652.1	0.301988478	5.29E-12	postive
ATOX1	AC145098.1	0.433931071	2.25E-24	postive
ATOX1	AC147067.1	0.401330121	8.98E-21	postive
ATOX1	AC147651.4	0.519120286	7.57E-36	postive
ATOX1	AC243919.1	0.415632519	2.64E-22	postive
ATOX1	AC244090.1	0.432518224	3.28E-24	postive
ATOX1	AC244669.1	0.506723991	5.70E-34	postive
ATOX1	AC245140.2	0.32236992	1.48E-13	postive
ATOX1	AC245297.4	0.345814759	1.72E-15	postive
ATOX1	AD000671.3	0.3598765	9.83E-17	postive
ATOX1	ADAMTS7P4	0.361138778	7.55E-17	postive
ATOX1	AF131216.4	0.415590838	2.67E-22	postive

ATOX1	AF274858.1	0.351957854	5.01E-16	postive
ATOX1	AGAP2-AS1	0.38163573	8.80E-19	postive
ATOX1	AL008726.1	0.309014356	1.59E-12	postive
ATOX1	AL009178.2	0.321989446	1.59E-13	postive
ATOX1	AL020996.1	0.312496967	8.68E-13	postive
ATOX1	AL023581.2	0.389011054	1.64E-19	postive
ATOX1	AL024498.1	0.415084043	3.03E-22	postive
ATOX1	AL024507.3	0.399226004	1.49E-20	postive
ATOX1	AL031055.1	0.424949608	2.42E-23	postive
ATOX1	AL031775.1	0.328670875	4.65E-14	postive
ATOX1	AL031846.1	0.35780692	1.51E-16	postive
ATOX1	AL035446.1	0.317949469	3.30E-13	postive
ATOX1	AL035587.1	0.331930478	2.52E-14	postive
ATOX1	AL035661.1	0.300300186	7.03E-12	postive
ATOX1	AL049597.2	0.52323674	1.73E-36	postive
ATOX1	AL049840.6	0.323788384	1.15E-13	postive
ATOX1	AL050309.1	0.487639871	3.15E-31	postive
ATOX1	AL078581.2	0.37893799	1.61E-18	postive
ATOX1	AL078590.2	0.38367855	5.55E-19	postive
ATOX1	AL078644.2	0.369496686	1.28E-17	postive
ATOX1	AL096828.3	0.339755615	5.64E-15	postive
ATOX1	AL117332.1	0.470203312	7.21E-29	postive
ATOX1	AL117336.2	0.332229699	2.38E-14	postive
ATOX1	AL118506.1	0.34749534	1.23E-15	postive
ATOX1	AL118516.1	0.326422355	7.06E-14	postive
ATOX1	AL118558.3	0.424239158	2.91E-23	postive
ATOX1	AL121603.2	0.337747175	8.32E-15	postive
ATOX1	AL121944.2	0.499096434	7.46E-33	postive
ATOX1	AL122023.3	0.3428919	3.06E-15	postive
ATOX1	AL122035.1	0.315568897	5.05E-13	postive
ATOX1	AL133215.2	0.328813561	4.53E-14	postive
ATOX1	AL133338.1	0.351040241	6.03E-16	postive
ATOX1	AL133467.1	0.33864346	7.00E-15	postive
ATOX1	AL133520.1	0.304494104	3.46E-12	postive
ATOX1	AL135744.1	0.376531967	2.75E-18	postive
ATOX1	AL136040.1	0.357253248	1.69E-16	postive
ATOX1	AL137186.2	0.392540949	7.22E-20	postive
ATOX1	AL138724.2	0.478656847	5.38E-30	postive
ATOX1	AL138762.2	0.326685693	6.72E-14	postive
ATOX1	AL139035.1	0.349089088	8.94E-16	postive
ATOX1	AL139220.2	0.337798131	8.24E-15	postive
ATOX1	AL139260.1	0.364208397	3.95E-17	postive
ATOX1	AL139339.1	0.476278421	1.13E-29	postive
ATOX1	AL139352.1	0.365072296	3.29E-17	postive
ATOX1	AL139384.1	0.343660554	2.63E-15	postive
ATOX1	AL157394.1	0.302495439	4.86E-12	postive
ATOX1	AL160313.2	0.305850133	2.75E-12	postive
ATOX1	AL161421.1	0.354478449	3.00E-16	postive
ATOX1	AL161729.1	0.320881374	1.95E-13	postive
ATOX1	AL162231.2	0.457668757	2.97E-27	postive

ATOX1	AL162377.1	0.39618256	3.07E-20	postive
ATOX1	AL162424.1	0.305865802	2.74E-12	postive
ATOX1	AL353708.1	0.432562641	3.25E-24	postive
ATOX1	AL354740.1	0.424961728	2.41E-23	postive
ATOX1	AL354920.1	0.536852541	1.14E-38	postive
ATOX1	AL355001.2	0.497622977	1.22E-32	postive
ATOX1	AL356481.1	0.415955467	2.43E-22	postive
ATOX1	AL356599.1	0.354316051	3.10E-16	postive
ATOX1	AL357054.4	0.323635165	1.18E-13	postive
ATOX1	AL357140.2	0.320926034	1.93E-13	postive
ATOX1	AL357874.1	0.3127444	8.31E-13	postive
ATOX1	AL357992.1	0.363670143	4.43E-17	postive
ATOX1	AL358472.2	0.426414685	1.65E-23	postive
ATOX1	AL358472.3	0.332757578	2.16E-14	postive
ATOX1	AL358852.1	0.380317353	1.18E-18	postive
ATOX1	AL359091.3	0.343847027	2.53E-15	postive
ATOX1	AL359182.1	0.321756336	1.66E-13	postive
ATOX1	AL359232.1	0.323476254	1.21E-13	postive
ATOX1	AL359513.1	0.334808352	1.46E-14	postive
ATOX1	AL359532.1	0.363335241	4.75E-17	postive
ATOX1	AL359643.3	0.332135217	2.43E-14	postive
ATOX1	AL359711.2	0.365083622	3.28E-17	postive
ATOX1	AL359715.3	0.347088205	1.33E-15	postive
ATOX1	AL359962.1	0.329426115	4.04E-14	postive
ATOX1	AL390198.1	0.304493013	3.46E-12	postive
ATOX1	AL390955.2	0.489557273	1.70E-31	postive
ATOX1	AL391069.2	0.441159313	3.15E-25	postive
ATOX1	AL391832.2	0.355799168	2.29E-16	postive
ATOX1	AL391832.3	0.437813936	7.88E-25	postive
ATOX1	AL392172.1	0.381982779	8.14E-19	postive
ATOX1	AL441992.2	0.585725462	2.21E-47	postive
ATOX1	AL445231.1	0.323378062	1.24E-13	postive
ATOX1	AL450326.1	0.331009437	3.00E-14	postive
ATOX1	AL450468.1	0.305785045	2.78E-12	postive
ATOX1	AL450998.2	0.347562757	1.21E-15	postive
ATOX1	AL451074.6	0.31167376	1.00E-12	postive
ATOX1	AL451085.3	0.352606336	4.39E-16	postive
ATOX1	AL451165.2	0.393180554	6.22E-20	postive
ATOX1	AL512625.1	0.354960685	2.72E-16	postive
ATOX1	AL513550.1	0.405647911	3.15E-21	postive
ATOX1	AL583722.1	0.343687555	2.62E-15	postive
ATOX1	AL589843.1	0.320982277	1.91E-13	postive
ATOX1	AL590617.2	0.405205343	3.51E-21	postive
ATOX1	AL590764.1	0.416914633	1.91E-22	postive
ATOX1	AL592166.1	0.335200902	1.36E-14	postive
ATOX1	AL596094.1	0.329370229	4.08E-14	postive
ATOX1	AL604028.1	0.305275598	3.03E-12	postive
ATOX1	AL606491.1	0.331395837	2.79E-14	postive
ATOX1	AL645568.1	0.361170327	7.50E-17	postive
ATOX1	AL645728.1	0.358219017	1.39E-16	postive

ATOX1	AL645933.3	0.342260219	3.46E-15	postive
ATOX1	AL662844.4	0.312926556	8.05E-13	postive
ATOX1	AL732314.8	0.334145106	1.66E-14	postive
ATOX1	AL928654.1	0.301717695	5.54E-12	postive
ATOX1	ALOX12-AS1	0.373685114	5.14E-18	postive
ATOX1	AMZ2P1	0.451814243	1.60E-26	postive
ATOX1	ANO7L1	0.369829537	1.19E-17	postive
ATOX1	AP000317.1	0.322692676	1.40E-13	postive
ATOX1	AP000320.1	0.302577024	4.79E-12	postive
ATOX1	AP000442.1	0.335608408	1.25E-14	postive
ATOX1	AP000569.1	0.312731083	8.33E-13	postive
ATOX1	AP000692.2	0.324476016	1.01E-13	postive
ATOX1	AP000695.1	0.34070505	4.69E-15	postive
ATOX1	AP000695.2	0.341568291	3.96E-15	postive
ATOX1	AP000759.1	0.327284963	6.02E-14	postive
ATOX1	AP000769.1	0.327723851	5.54E-14	postive
ATOX1	AP001160.2	0.329954827	3.66E-14	postive
ATOX1	AP001160.3	0.39068248	1.11E-19	postive
ATOX1	AP001330.4	0.37496957	3.88E-18	postive
ATOX1	AP001347.1	0.342452565	3.33E-15	postive
ATOX1	AP001372.2	0.382155279	7.83E-19	postive
ATOX1	AP001434.1	0.30262879	4.75E-12	postive
ATOX1	AP001453.4	0.338295262	7.48E-15	postive
ATOX1	AP001816.1	0.555054102	9.55E-42	postive
ATOX1	AP001922.5	0.428165751	1.04E-23	postive
ATOX1	AP002360.2	0.327480571	5.80E-14	postive
ATOX1	AP002387.2	0.329588925	3.92E-14	postive
ATOX1	AP002748.4	0.381900379	8.29E-19	postive
ATOX1	AP002761.2	0.359631107	1.03E-16	postive
ATOX1	AP002954.1	0.400449482	1.11E-20	postive
ATOX1	AP003721.4	0.350175573	7.18E-16	postive
ATOX1	AP004609.5	0.375061077	3.80E-18	postive
ATOX1	AP005019.1	0.371923315	7.55E-18	postive
ATOX1	AP005329.1	0.307722901	1.99E-12	postive
ATOX1	AP005432.2	0.305052377	3.15E-12	postive
ATOX1	AP006621.3	0.383992602	5.17E-19	postive
ATOX1	AP007216.2	0.303472588	4.12E-12	postive
ATOX1	ARF4-AS1	0.387291641	2.43E-19	postive
ATOX1	ARMCX5-GPRASP2	0.459285535	1.85E-27	postive
ATOX1	ARRDC1-AS1	0.503439622	1.74E-33	postive
ATOX1	ASB16-AS1	0.40077982	1.03E-20	postive
ATOX1	ASH1L-AS1	0.321778866	1.65E-13	postive
ATOX1	ATF4P4	0.301033808	6.22E-12	postive
ATOX1	ATP2A1-AS1	0.324459345	1.01E-13	postive
ATOX1	ATXN1-AS1	0.330345189	3.40E-14	postive
ATOX1	ATXN2-AS	0.328958928	4.41E-14	postive
ATOX1	AZIN1-AS1	0.316770863	4.07E-13	postive
ATOX1	B4GALT1-AS1	0.429918858	6.56E-24	postive
ATOX1	BAALC-AS1	0.390690745	1.11E-19	postive
ATOX1	BCDIN3D-AS1	0.314831158	5.75E-13	postive

ATOX1	BHLHE40-AS1	0.489046989	2.00E-31	postive
ATOX1	BMS1P20	0.377628089	2.15E-18	postive
ATOX1	BTG3-AS1	0.343661335	2.63E-15	postive
ATOX1	BTN2A3P	0.316317044	4.42E-13	postive
ATOX1	BVES-AS1	0.350391958	6.87E-16	postive
ATOX1	BX284668.5	0.319777386	2.38E-13	postive
ATOX1	BX537318.1	0.337018496	9.57E-15	postive
ATOX1	BX640514.2	0.301723411	5.54E-12	postive
ATOX1	C10orf25	0.365914446	2.75E-17	postive
ATOX1	C18orf65	0.351890266	5.08E-16	postive
ATOX1	C21orf62-AS1	0.338234172	7.57E-15	postive
ATOX1	C22orf24	0.305094198	3.13E-12	postive
ATOX1	C2orf27A	0.366805452	2.27E-17	postive
ATOX1	C5orf66	0.323285117	1.26E-13	postive
ATOX1	C6orf99	0.389973078	1.31E-19	postive
ATOX1	C8orf37-AS1	0.341126495	4.32E-15	postive
ATOX1	CA5BP1	0.443092075	1.85E-25	postive
ATOX1	CARD8-AS1	0.532997346	4.84E-38	postive
ATOX1	CASP17P	0.41350825	4.51E-22	postive
ATOX1	CATIP-AS1	0.301508134	5.74E-12	postive
ATOX1	CATSPER2P1	0.377041907	2.45E-18	postive
ATOX1	CCNT2-AS1	0.348796553	9.48E-16	postive
ATOX1	CCR5AS	0.368396908	1.62E-17	postive
ATOX1	CD27-AS1	0.450445714	2.36E-26	postive
ATOX1	CD99P1	0.325914354	7.75E-14	postive
ATOX1	CDC37L1-DT	0.377171141	2.38E-18	postive
ATOX1	CENPBD1P1	0.450455378	2.35E-26	postive
ATOX1	CEP250-AS1	0.364383044	3.81E-17	postive
ATOX1	CEP83-DT	0.313463928	7.32E-13	postive
ATOX1	CFL1P1	0.321056485	1.88E-13	postive
ATOX1	CHKB-DT	0.418341918	1.33E-22	postive
ATOX1	CHROMR	0.380823967	1.06E-18	postive
ATOX1	CIDECP1	0.553891447	1.52E-41	postive
ATOX1	CKMT2-AS1	0.363041354	5.06E-17	postive
ATOX1	COA6-AS1	0.328342315	4.94E-14	postive
ATOX1	CPNE8-AS1	0.354816952	2.80E-16	postive
ATOX1	CR381653.1	0.318179776	3.17E-13	postive
ATOX1	CRYBB2P1	0.331259603	2.86E-14	postive
ATOX1	CRYZL2P	0.371853888	7.66E-18	postive
ATOX1	CTA-989H11.1	0.33310447	2.02E-14	postive
ATOX1	CTBP1-DT	0.449017948	3.53E-26	postive
ATOX1	CTD-2521M24.9	0.417984322	1.45E-22	postive
ATOX1	CYTOR	0.608339334	6.17E-52	postive
ATOX1	DANCR	0.33642822	1.07E-14	postive
ATOX1	DARS-AS1	0.424970139	2.41E-23	postive
ATOX1	DBIL5P	0.3126718	8.42E-13	postive
ATOX1	DCST1-AS1	0.363954079	4.17E-17	postive
ATOX1	DEC1	0.332016856	2.48E-14	postive
ATOX1	DEPDC1-AS1	0.358793878	1.23E-16	postive
ATOX1	DGCR11	0.310920342	1.14E-12	postive

ATOX1	DHCR24-DT	0.318089853	3.22E-13	postive
ATOX1	DHRS4-AS1	0.386891588	2.67E-19	postive
ATOX1	DLG1-AS1	0.336225879	1.11E-14	postive
ATOX1	DLGAP1-AS1	0.309034255	1.59E-12	postive
ATOX1	DNAJC3-DT	0.31515106	5.43E-13	postive
ATOX1	DPH6-DT	0.432542322	3.26E-24	postive
ATOX1	DTNB-AS1	0.301152363	6.09E-12	postive
ATOX1	DUBR	0.323930575	1.12E-13	postive
ATOX1	DUXAP8	0.325806101	7.91E-14	postive
ATOX1	DUXAP9	0.33861619	7.03E-15	postive
ATOX1	EBLN3P	0.383622767	5.62E-19	postive
ATOX1	EIF1B-AS1	0.412787287	5.40E-22	postive
ATOX1	EIF2AK3-DT	0.305764327	2.79E-12	postive
ATOX1	EIF3J-DT	0.409700446	1.16E-21	postive
ATOX1	ELF3-AS1	0.339430072	6.01E-15	postive
ATOX1	ELOA-AS1	0.316880314	3.99E-13	postive
ATOX1	EMC1-AS1	0.431314175	4.53E-24	postive
ATOX1	ENTPD3-AS1	0.479576655	4.04E-30	postive
ATOX1	EPB41L4A-AS1	0.343373044	2.78E-15	postive
ATOX1	EPB41L4A-DT	0.401154004	9.37E-21	postive
ATOX1	EPCAM-DT	0.30104641	6.20E-12	postive
ATOX1	EPHA1-AS1	0.310378909	1.26E-12	postive
ATOX1	ERICH6-AS1	0.315601665	5.02E-13	postive
ATOX1	ETV7-AS1	0.428411922	9.77E-24	postive
ATOX1	EXOC3-AS1	0.398917099	1.60E-20	postive
ATOX1	EXOSC10-AS1	0.457301175	3.30E-27	postive
ATOX1	FAHD2CP	0.335778692	1.21E-14	postive
ATOX1	FAM111A-DT	0.458507529	2.32E-27	postive
ATOX1	FAM21FP	0.379647614	1.37E-18	postive
ATOX1	FAM86C2P	0.370593509	1.01E-17	postive
ATOX1	FAM86DP	0.384196378	4.93E-19	postive
ATOX1	FAM86EP	0.447860416	4.89E-26	postive
ATOX1	FAM86JP	0.446645977	6.88E-26	postive
ATOX1	FGD5-AS1	0.516127245	2.18E-35	postive
ATOX1	FGF7P6	0.314974071	5.61E-13	postive
ATOX1	FO393418.1	0.338009973	7.91E-15	postive
ATOX1	FOXD3-AS1	0.352359361	4.62E-16	postive
ATOX1	FRG1HP	0.529174947	1.99E-37	postive
ATOX1	FZD4-DT	0.379698874	1.36E-18	postive
ATOX1	GAPLINC	0.37008617	1.12E-17	postive
ATOX1	GASAL1	0.348727733	9.61E-16	postive
ATOX1	GBAP1	0.316165055	4.54E-13	postive
ATOX1	GBP1P1	0.41353073	4.48E-22	postive
ATOX1	GCC2-AS1	0.349823243	7.71E-16	postive
ATOX1	GGTA1P	0.420768648	7.14E-23	postive
ATOX1	GIHCG	0.370685983	9.87E-18	postive
ATOX1	GLIDR	0.438977325	5.74E-25	postive
ATOX1	GNRHR2	0.346346006	1.55E-15	postive
ATOX1	GPRC5D-AS1	0.409724272	1.16E-21	postive
ATOX1	GSEC	0.34519686	1.94E-15	postive

ATOX1	GTF2IP1	0.310562335	1.22E-12	postive
ATOX1	GTF2IRD1P1	0.328143891	5.13E-14	postive
ATOX1	H2AZ2P1	0.306532135	2.44E-12	postive
ATOX1	HCG18	0.355012895	2.69E-16	postive
ATOX1	HCG25	0.400854388	1.01E-20	postive
ATOX1	HCP5	0.545402708	4.31E-40	postive
ATOX1	HECW2-AS1	0.328867728	4.48E-14	postive
ATOX1	HEIH	0.529117186	2.03E-37	postive
ATOX1	HLA-DRB6	0.416282806	2.24E-22	postive
ATOX1	HLA-J	0.318169238	3.17E-13	postive
ATOX1	HLA-L	0.450128981	2.58E-26	postive
ATOX1	HMGB1P5	0.43720239	9.30E-25	postive
ATOX1	HOMER3-AS1	0.371984902	7.45E-18	postive
ATOX1	HSD11B1-AS1	0.308631341	1.70E-12	postive
ATOX1	IDH1-AS1	0.351103339	5.95E-16	postive
ATOX1	IL10RB-DT	0.379247891	1.50E-18	postive
ATOX1	IL6R-AS1	0.301093312	6.16E-12	postive
ATOX1	ILF3-DT	0.397255997	2.38E-20	postive
ATOX1	IPO9-AS1	0.300714968	6.56E-12	postive
ATOX1	IQCH-AS1	0.39840848	1.81E-20	postive
ATOX1	IRF1-AS1	0.325578858	8.25E-14	postive
ATOX1	ITFG2-AS1	0.324461242	1.01E-13	postive
ATOX1	JPX	0.386429799	2.96E-19	postive
ATOX1	KDM7A-DT	0.403177076	5.75E-21	postive
ATOX1	KMT2E-AS1	0.42858987	9.32E-24	postive
ATOX1	KRT8P12	0.455405682	5.71E-27	postive
ATOX1	KTN1-AS1	0.439403332	5.11E-25	postive
ATOX1	L3MBTL4-AS1	0.360598689	8.45E-17	postive
ATOX1	LBX2-AS1	0.356097376	2.15E-16	postive
ATOX1	LCT-AS1	0.382056964	8.00E-19	postive
ATOX1	LINC00211	0.321918214	1.61E-13	postive
ATOX1	LINC00239	0.342580599	3.25E-15	postive
ATOX1	LINC00324	0.425477079	2.11E-23	postive
ATOX1	LINC00339	0.482570867	1.58E-30	postive
ATOX1	LINC00346	0.303400693	4.17E-12	postive
ATOX1	LINC00467	0.453421003	1.01E-26	postive
ATOX1	LINC00476	0.446964635	6.29E-26	postive
ATOX1	LINC00511	0.428040004	1.08E-23	postive
ATOX1	LINC00525	0.348876441	9.32E-16	postive
ATOX1	LINC00526	0.432474242	3.32E-24	postive
ATOX1	LINC00528	0.310201454	1.30E-12	postive
ATOX1	LINC00539	0.349359471	8.46E-16	postive
ATOX1	LINC00582	0.319396041	2.54E-13	postive
ATOX1	LINC00623	0.358083699	1.43E-16	postive
ATOX1	LINC00630	0.308492508	1.74E-12	postive
ATOX1	LINC00654	0.323657716	1.17E-13	postive
ATOX1	LINC00667	0.401361922	8.91E-21	postive
ATOX1	LINC00680	0.301234292	6.01E-12	postive
ATOX1	LINC00847	0.560948886	8.76E-43	postive
ATOX1	LINC00857	0.3194772	2.51E-13	postive

ATOX1	LINC00869	0.323673899	1.17E-13	postive
ATOX1	LINC00884	0.344766665	2.11E-15	postive
ATOX1	LINC00887	0.305217644	3.06E-12	postive
ATOX1	LINC00888	0.495767467	2.25E-32	postive
ATOX1	LINC00892	0.315339855	5.25E-13	postive
ATOX1	LINC00900	0.32703709	6.30E-14	postive
ATOX1	LINC00909	0.418575009	1.25E-22	postive
ATOX1	LINC00910	0.316059639	4.62E-13	postive
ATOX1	LINC00921	0.345808415	1.72E-15	postive
ATOX1	LINC00933	0.339998738	5.38E-15	postive
ATOX1	LINC00944	0.34689088	1.39E-15	postive
ATOX1	LINC00957	0.321582993	1.71E-13	postive
ATOX1	LINC00963	0.465204911	3.23E-28	postive
ATOX1	LINC01003	0.325366576	8.58E-14	postive
ATOX1	LINC01011	0.38667412	2.80E-19	postive
ATOX1	LINC01023	0.435484909	1.48E-24	postive
ATOX1	LINC01063	0.353778408	3.46E-16	postive
ATOX1	LINC01093	0.368772475	1.49E-17	postive
ATOX1	LINC01094	0.50577763	7.87E-34	postive
ATOX1	LINC01127	0.348117003	1.09E-15	postive
ATOX1	LINC01128	0.390078038	1.28E-19	postive
ATOX1	LINC01132	0.321685872	1.68E-13	postive
ATOX1	LINC01137	0.514179997	4.33E-35	postive
ATOX1	LINC01138	0.330697977	3.18E-14	postive
ATOX1	LINC01145	0.312956565	8.01E-13	postive
ATOX1	LINC01150	0.349836464	7.69E-16	postive
ATOX1	LINC01184	0.5359377	1.61E-38	postive
ATOX1	LINC01220	0.376022222	3.07E-18	postive
ATOX1	LINC01232	0.367120883	2.13E-17	postive
ATOX1	LINC01273	0.338687244	6.94E-15	postive
ATOX1	LINC01278	0.407782388	1.87E-21	postive
ATOX1	LINC01281	0.350314871	6.98E-16	postive
ATOX1	LINC01303	0.308800699	1.65E-12	postive
ATOX1	LINC01358	0.317668354	3.47E-13	postive
ATOX1	LINC01366	0.301424466	5.82E-12	postive
ATOX1	LINC01410	0.301679449	5.58E-12	postive
ATOX1	LINC01506	0.4228425	4.18E-23	postive
ATOX1	LINC01560	0.383957452	5.21E-19	postive
ATOX1	LINC01585	0.317658902	3.48E-13	postive
ATOX1	LINC01588	0.399904543	1.26E-20	postive
ATOX1	LINC01635	0.330193503	3.50E-14	postive
ATOX1	LINC01666	0.308011162	1.89E-12	postive
ATOX1	LINC01675	0.302970298	4.49E-12	postive
ATOX1	LINC01678	0.370855948	9.51E-18	postive
ATOX1	LINC01679	0.304147212	3.67E-12	postive
ATOX1	LINC01684	0.343832323	2.54E-15	postive
ATOX1	LINC01686	0.428956733	8.46E-24	postive
ATOX1	LINC01704	0.36609276	2.65E-17	postive
ATOX1	LINC01715	0.343657694	2.63E-15	postive
ATOX1	LINC01775	0.307194888	2.18E-12	postive

ATOX1	LINC01806	0.321717312	1.67E-13	postive
ATOX1	LINC01841	0.403814302	4.93E-21	postive
ATOX1	LINC01843	0.305677575	2.83E-12	postive
ATOX1	LINC01857	0.365326707	3.12E-17	postive
ATOX1	LINC01871	0.342088867	3.58E-15	postive
ATOX1	LINC01888	0.330245792	3.46E-14	postive
ATOX1	LINC01943	0.52362089	1.51E-36	postive
ATOX1	LINC02000	0.329379607	4.07E-14	postive
ATOX1	LINC02014	0.363967217	4.16E-17	postive
ATOX1	LINC02021	0.308199851	1.83E-12	postive
ATOX1	LINC02026	0.331552377	2.71E-14	postive
ATOX1	LINC02041	0.307333994	2.13E-12	postive
ATOX1	LINC02062	0.367612533	1.91E-17	postive
ATOX1	LINC02084	0.31267322	8.41E-13	postive
ATOX1	LINC02099	0.403906789	4.82E-21	postive
ATOX1	LINC02132	0.322196603	1.53E-13	postive
ATOX1	LINC02172	0.339573915	5.84E-15	postive
ATOX1	LINC02195	0.314328982	6.28E-13	postive
ATOX1	LINC02210	0.302086026	5.21E-12	postive
ATOX1	LINC02285	0.498490706	9.12E-33	postive
ATOX1	LINC02298	0.390862749	1.07E-19	postive
ATOX1	LINC02325	0.300513738	6.78E-12	postive
ATOX1	LINC02328	0.344374407	2.28E-15	postive
ATOX1	LINC02356	0.324630298	9.82E-14	postive
ATOX1	LINC02361	0.366461168	2.45E-17	postive
ATOX1	LINC02391	0.377818903	2.06E-18	postive
ATOX1	LINC02450	0.366670395	2.34E-17	postive
ATOX1	LINC02513	0.337208579	9.23E-15	postive
ATOX1	LINC02528	0.329350817	4.09E-14	postive
ATOX1	LINC02550	0.303578122	4.05E-12	postive
ATOX1	LINC02585	0.307235337	2.17E-12	postive
ATOX1	LINC02609	0.352402369	4.58E-16	postive
ATOX1	LINC02611	0.500576594	4.55E-33	postive
ATOX1	LINC02637	0.4210645	6.62E-23	postive
ATOX1	LINC02693	0.358565189	1.29E-16	postive
ATOX1	LINC02709	0.344621699	2.18E-15	postive
ATOX1	LINC02724	0.356424789	2.01E-16	postive
ATOX1	LINC02773	0.419718035	9.35E-23	postive
ATOX1	LINC02777	0.364626593	3.62E-17	postive
ATOX1	LINC02802	0.301893865	5.38E-12	postive
ATOX1	LINC02828	0.351212738	5.82E-16	postive
ATOX1	LIPE-AS1	0.44104573	3.26E-25	postive
ATOX1	LMCD1-AS1	0.303637626	4.01E-12	postive
ATOX1	LNCTAM34A	0.314051478	6.60E-13	postive
ATOX1	LOH12CR2	0.366684184	2.33E-17	postive
ATOX1	LOXL1-AS1	0.346444148	1.52E-15	postive
ATOX1	LPP-AS2	0.394975461	4.08E-20	postive
ATOX1	LRRC37BP1	0.313944922	6.73E-13	postive
ATOX1	LRRC8C-DT	0.306172043	2.60E-12	postive
ATOX1	LSP1P4	0.43409167	2.15E-24	postive

ATOX1	MACORIS	0.434347784	2.01E-24	postive
ATOX1	MAFG-DT	0.368444291	1.60E-17	postive
ATOX1	MAN1B1-DT	0.403259066	5.64E-21	postive
ATOX1	MAP2K4P1	0.406187847	2.76E-21	postive
ATOX1	MAP3K14-AS1	0.354732051	2.85E-16	postive
ATOX1	MAP3K2-DT	0.435175341	1.61E-24	postive
ATOX1	MAP4K3-DT	0.479068139	4.74E-30	postive
ATOX1	MAPKAPK5-AS1	0.582251012	1.03E-46	postive
ATOX1	MDS2	0.323934021	1.12E-13	postive
ATOX1	MED14OS	0.519424803	6.79E-36	postive
ATOX1	MIATNB	0.374389199	4.40E-18	postive
ATOX1	MIPEPP3	0.340412428	4.97E-15	postive
ATOX1	MIR155HG	0.367784745	1.84E-17	postive
ATOX1	MIR193BHG	0.378583133	1.74E-18	postive
ATOX1	MIR210HG	0.300642084	6.64E-12	postive
ATOX1	MIR22HG	0.417058041	1.84E-22	postive
ATOX1	MIR4435-2HG	0.616665981	1.04E-53	postive
ATOX1	MIR497HG	0.311892739	9.65E-13	postive
ATOX1	MIR583HG	0.334513761	1.55E-14	postive
ATOX1	MIR762HG	0.42034999	7.95E-23	postive
ATOX1	MKLN1-AS	0.424504481	2.72E-23	postive
ATOX1	MMADHC-DT	0.433226896	2.72E-24	postive
ATOX1	MRPL20-AS1	0.439723777	4.68E-25	postive
ATOX1	MRPL45P2	0.349766264	7.80E-16	postive
ATOX1	MRPS31P4	0.341825504	3.77E-15	postive
ATOX1	MSC-AS1	0.346638753	1.46E-15	postive
ATOX1	MYLK-AS1	0.368748838	1.50E-17	postive
ATOX1	NACA4P	0.345542302	1.81E-15	postive
ATOX1	NAIPP1	0.462426126	7.37E-28	postive
ATOX1	NAIPP3	0.346801077	1.41E-15	postive
ATOX1	NAPA-AS1	0.404069411	4.63E-21	postive
ATOX1	NAPSB	0.352804771	4.22E-16	postive
ATOX1	NBR2	0.360860064	8.00E-17	postive
ATOX1	NCF1B	0.405501037	3.27E-21	postive
ATOX1	NCK1-DT	0.552485363	2.66E-41	postive
ATOX1	NDUFB2-AS1	0.413229269	4.83E-22	postive
ATOX1	NEBL-AS1	0.308294696	1.80E-12	postive
ATOX1	NIFK-AS1	0.467507352	1.62E-28	postive
ATOX1	NKAPP1	0.401717255	8.18E-21	postive
ATOX1	NOP14-AS1	0.465609954	2.87E-28	postive
ATOX1	NORAD	0.309160033	1.55E-12	postive
ATOX1	NRAV	0.574769659	2.67E-45	postive
ATOX1	NRSN2-AS1	0.472088759	4.07E-29	postive
ATOX1	NUDT16P1	0.526982169	4.45E-37	postive
ATOX1	NUP50-DT	0.48229014	1.73E-30	postive
ATOX1	NUTM2A-AS1	0.478374632	5.88E-30	postive
ATOX1	OGFRP1	0.338986768	6.55E-15	postive
ATOX1	OIP5-AS1	0.376968025	2.49E-18	postive
ATOX1	OLMALINC	0.335075495	1.39E-14	postive
ATOX1	OSER1-DT	0.547256636	2.09E-40	postive

ATOX1	OTOAP1	0.343870375	2.52E-15	postive
ATOX1	OTUD6B-AS1	0.36291637	5.19E-17	postive
ATOX1	PAXIP1-AS2	0.370837784	9.55E-18	postive
ATOX1	PCBP1-AS1	0.415695352	2.60E-22	postive
ATOX1	PCCA-DT	0.465750073	2.75E-28	postive
ATOX1	PCED1B-AS1	0.474189591	2.14E-29	postive
ATOX1	PDCD4-AS1	0.418263198	1.36E-22	postive
ATOX1	PINK1-AS	0.354999809	2.69E-16	postive
ATOX1	PITPNA-AS1	0.439283884	5.28E-25	postive
ATOX1	PLEKHA8P1	0.451013612	2.01E-26	postive
ATOX1	PMS2P4	0.517719316	1.24E-35	postive
ATOX1	POLR2J4	0.417072998	1.83E-22	postive
ATOX1	PPP1R12A-AS1	0.364235087	3.93E-17	postive
ATOX1	PRELID1P1	0.545283645	4.52E-40	postive
ATOX1	PRICKLE2-DT	0.305418347	2.96E-12	postive
ATOX1	PRKAR2A-AS1	0.336915877	9.76E-15	postive
ATOX1	PRR34	0.418765784	1.19E-22	postive
ATOX1	PRR34-AS1	0.418871126	1.16E-22	postive
ATOX1	PSMB8-AS1	0.583178465	6.86E-47	postive
ATOX1	PTOV1-AS1	0.307928713	1.92E-12	postive
ATOX1	PXN-AS1	0.450560128	2.28E-26	postive
ATOX1	RAB11B-AS1	0.468789815	1.11E-28	postive
ATOX1	RAB30-DT	0.326381124	7.11E-14	postive
ATOX1	RABGAP1L-DT	0.331857697	2.56E-14	postive
ATOX1	RAET1E-AS1	0.302158106	5.15E-12	postive
ATOX1	RALY-AS1	0.351580881	5.41E-16	postive
ATOX1	RARA-AS1	0.427868854	1.13E-23	postive
ATOX1	RASAL2-AS1	0.425098996	2.33E-23	postive
ATOX1	RASSF1-AS1	0.346616064	1.46E-15	postive
ATOX1	RBM15-AS1	0.551851514	3.43E-41	postive
ATOX1	RBM26-AS1	0.328861593	4.49E-14	postive
ATOX1	RNASEH1-AS1	0.438586358	6.38E-25	postive
ATOX1	RNF216P1	0.520671773	4.35E-36	postive
ATOX1	RP11-101E13.5	0.360719674	8.24E-17	postive
ATOX1	RP11-10C24.1	0.397445664	2.27E-20	postive
ATOX1	RP11-160O5.1	0.317668848	3.47E-13	postive
ATOX1	RP11-2B6.3	0.319614809	2.45E-13	postive
ATOX1	RP11-314C16.1	0.342781481	3.13E-15	postive
ATOX1	RP11-435O5.2	0.301358916	5.89E-12	postive
ATOX1	RP11-514D23.3	0.313731796	6.98E-13	postive
ATOX1	RP11-541P9.3	0.339976333	5.40E-15	postive
ATOX1	RP11-556E13.1	0.430267446	5.98E-24	postive
ATOX1	RP11-58E21.3	0.399760635	1.31E-20	postive
ATOX1	RP11-86H7.7	0.368981252	1.43E-17	postive
ATOX1	RP11-876N24.4	0.317696284	3.45E-13	postive
ATOX1	RP11-876N24.5	0.326670771	6.74E-14	postive
ATOX1	RP4-545L17.11	0.429704485	6.94E-24	postive
ATOX1	RP5-968J1.1	0.340954343	4.47E-15	postive
ATOX1	RPARP-AS1	0.303758939	3.92E-12	postive
ATOX1	RPL12P38	0.303756384	3.93E-12	postive

ATOX1	RPL13AP3	0.318265601	3.12E-13	postive
ATOX1	RPL13AP7	0.374003678	4.79E-18	postive
ATOX1	RPL23AP7	0.521274775	3.51E-36	postive
ATOX1	RPL23AP82	0.604689993	3.55E-51	postive
ATOX1	RPLP1P6	0.492283276	7.03E-32	postive
ATOX1	RPS18P9	0.421798508	5.48E-23	postive
ATOX1	RRN3P3	0.379000521	1.59E-18	postive
ATOX1	SAP30-DT	0.397059359	2.49E-20	postive
ATOX1	SBDSP1	0.475467891	1.44E-29	postive
ATOX1	SBF2-AS1	0.475724734	1.33E-29	postive
ATOX1	SCAMP1-AS1	0.576345242	1.35E-45	postive
ATOX1	SDHAP1	0.304310955	3.57E-12	postive
ATOX1	SEC24B-AS1	0.306780312	2.34E-12	postive
ATOX1	SEPTIN4-AS1	0.486234804	4.94E-31	postive
ATOX1	SERPINB9P1	0.338253528	7.55E-15	postive
ATOX1	SLC25A34-AS1	0.373568055	5.27E-18	postive
ATOX1	SLC25A5-AS1	0.342253137	3.47E-15	postive
ATOX1	SLC44A3-AS1	0.347696047	1.18E-15	postive
ATOX1	SMIM25	0.426340634	1.68E-23	postive
ATOX1	SMIM2-AS1	0.371164294	8.90E-18	postive
ATOX1	SMPD5	0.310055401	1.33E-12	postive
ATOX1	SNAI3-AS1	0.389107009	1.60E-19	postive
ATOX1	SNHG11	0.491489757	9.10E-32	postive
ATOX1	SNHG15	0.37703206	2.46E-18	postive
ATOX1	SNHG16	0.380264782	1.20E-18	postive
ATOX1	SNHG17	0.340291711	5.08E-15	postive
ATOX1	SNHG28	0.342273692	3.45E-15	postive
ATOX1	SNHG29	0.33808597	7.79E-15	postive
ATOX1	SNHG30	0.377948306	2.01E-18	postive
ATOX1	SNHG32	0.363599192	4.50E-17	postive
ATOX1	SNHG5	0.383739366	5.47E-19	postive
ATOX1	SNHG6	0.335968947	1.17E-14	postive
ATOX1	SNHG8	0.35337772	3.75E-16	postive
ATOX1	SOCAR	0.40604611	2.86E-21	postive
ATOX1	SP2-AS1	0.370148055	1.11E-17	postive
ATOX1	SPATA41	0.346937265	1.37E-15	postive
ATOX1	SPINT1-AS1	0.346965529	1.37E-15	postive
ATOX1	SRP14-AS1	0.326658904	6.76E-14	postive
ATOX1	SSR4P1	0.351808867	5.16E-16	postive
ATOX1	ST20-AS1	0.371312315	8.62E-18	postive
ATOX1	STAG3L2	0.337651469	8.47E-15	postive
ATOX1	STAG3L4	0.486003954	5.32E-31	postive
ATOX1	STARD7-AS1	0.484074162	9.82E-31	postive
ATOX1	STK4-AS1	0.505211312	9.54E-34	postive
ATOX1	STX17-AS1	0.386533739	2.90E-19	postive
ATOX1	STX18-AS1	0.330881247	3.07E-14	postive
ATOX1	STXBP5-AS1	0.371993668	7.43E-18	postive
ATOX1	SUCLA2-AS1	0.330221143	3.48E-14	postive
ATOX1	SUMO2P17	0.421463978	5.97E-23	postive
ATOX1	SVIL-AS1	0.536594514	1.26E-38	postive

ATOX1	SYS1-DBNDD2	0.328420875	4.87E-14	postive
ATOX1	TAF1A-AS1	0.442057059	2.46E-25	postive
ATOX1	TDRKH-AS1	0.38059866	1.11E-18	postive
ATOX1	TEKT4P2	0.332762367	2.16E-14	postive
ATOX1	TFAP2A-AS1	0.312520167	8.64E-13	postive
ATOX1	THAP7-AS1	0.318924611	2.77E-13	postive
ATOX1	THAP9-AS1	0.327032289	6.30E-14	postive
ATOX1	TIPARP-AS1	0.327598648	5.68E-14	postive
ATOX1	TM4SF19-AS1	0.379086804	1.56E-18	postive
ATOX1	TMC3-AS1	0.368924618	1.44E-17	postive
ATOX1	TMCC1-AS1	0.332695651	2.18E-14	postive
ATOX1	TMEM161B-AS1	0.319839944	2.35E-13	postive
ATOX1	TMEM191A	0.344228455	2.35E-15	postive
ATOX1	TMEM202-AS1	0.376193083	2.96E-18	postive
ATOX1	TMEM26-AS1	0.363063681	5.04E-17	postive
ATOX1	TMEM44-AS1	0.43390384	2.27E-24	postive
ATOX1	TMEM99	0.410862746	8.73E-22	postive
ATOX1	TMPO-AS1	0.352011428	4.95E-16	postive
ATOX1	TOB1-AS1	0.426548703	1.59E-23	postive
ATOX1	TP53TG1	0.585819783	2.12E-47	postive
ATOX1	TPI1P2	0.31062863	1.20E-12	postive
ATOX1	TPRG1-AS1	0.399967614	1.25E-20	postive
ATOX1	TRAF3IP2-AS1	0.306471265	2.47E-12	postive
ATOX1	TRAM2-AS1	0.505637996	8.25E-34	postive
ATOX1	TRBV11-2	0.370201164	1.10E-17	postive
ATOX1	TRG-AS1	0.343472055	2.73E-15	postive
ATOX1	TRIM52-AS1	0.554139777	1.38E-41	postive
ATOX1	TSPOAP1-AS1	0.302129234	5.17E-12	postive
ATOX1	TSTD3	0.480990581	2.60E-30	postive
ATOX1	TTC28-AS1	0.374360977	4.43E-18	postive
ATOX1	TUBBP1	0.309337372	1.51E-12	postive
ATOX1	TUG1	0.336557287	1.05E-14	postive
ATOX1	TYMSOS	0.305494602	2.92E-12	postive
ATOX1	U47924.2	0.35707862	1.76E-16	postive
ATOX1	U62317.1	0.333115104	2.02E-14	postive
ATOX1	U62317.2	0.310176228	1.30E-12	postive
ATOX1	UBA6-AS1	0.554211921	1.34E-41	postive
ATOX1	UBAC2-AS1	0.353057703	4.01E-16	postive
ATOX1	UBE2D3-AS1	0.54420584	6.86E-40	postive
ATOX1	UBL7-AS1	0.433349333	2.63E-24	postive
ATOX1	UGDH-AS1	0.300520814	6.78E-12	postive
ATOX1	URB1-AS1	0.428006563	1.09E-23	postive
ATOX1	USP27X-AS1	0.387893832	2.12E-19	postive
ATOX1	USP30-AS1	0.483240044	1.28E-30	postive
ATOX1	USP46-AS1	0.412570972	5.70E-22	postive
ATOX1	UXT-AS1	0.419719145	9.34E-23	postive
ATOX1	VPS13B-DT	0.307677218	2.01E-12	postive
ATOX1	VPS33B-DT	0.314601865	5.99E-13	postive
ATOX1	WAC-AS1	0.397945828	2.02E-20	postive
ATOX1	WAKMAR2	0.361362027	7.20E-17	postive

ATOX1	WASH3P	0.396943382	2.56E-20	postive
ATOX1	WEE2-AS1	0.357095644	1.75E-16	postive
ATOX1	WWTR1-AS1	0.321250414	1.82E-13	postive
ATOX1	YTHDF3-AS1	0.342593479	3.24E-15	postive
ATOX1	Z83851.4	0.370300391	1.07E-17	postive
ATOX1	Z95114.1	0.329421075	4.04E-14	postive
ATOX1	Z97634.1	0.311089031	1.11E-12	postive
ATOX1	ZBED5-AS1	0.375462328	3.48E-18	postive
ATOX1	ZBTB11-AS1	0.385863762	3.37E-19	postive
ATOX1	ZFAS1	0.397952551	2.02E-20	postive
ATOX1	ZFPM2-AS1	0.359952273	9.67E-17	postive
ATOX1	ZNF205-AS1	0.344693159	2.14E-15	postive
ATOX1	ZNF213-AS1	0.334812912	1.46E-14	postive
ATOX1	ZNF236-DT	0.406160706	2.78E-21	postive
ATOX1	ZNF252P	0.38169742	8.68E-19	postive
ATOX1	ZNF426-DT	0.316477695	4.29E-13	postive
ATOX1	ZNF433-AS1	0.360431836	8.75E-17	postive
ATOX1	ZNF503-AS2	0.385954586	3.30E-19	postive
ATOX1	ZNF529-AS1	0.468475982	1.21E-28	postive
ATOX1	ZNF542P	0.305949217	2.70E-12	postive
ATOX1	ZNF561-AS1	0.66205674	2.26E-64	postive
ATOX1	ZNF687-AS1	0.475545066	1.41E-29	postive
ATOX1	ZNF788P	0.366644109	2.35E-17	postive
ATOX1	ZNF815P	0.40514677	3.56E-21	postive
ATOX1	ZNRD2-AS1	0.319617654	2.44E-13	postive
ATOX1	ZSCAN16-AS1	0.559715469	1.45E-42	postive
ATP7A	A2M-AS1	0.403163406	5.77E-21	postive
ATP7A	AC000403.1	0.375888452	3.17E-18	postive
ATP7A	AC002091.2	0.360970246	7.82E-17	postive
ATP7A	AC004066.2	0.304023194	3.75E-12	postive
ATP7A	AC004067.1	0.327659581	5.61E-14	postive
ATP7A	AC004112.1	0.423676381	3.37E-23	postive
ATP7A	AC004381.1	0.330243722	3.46E-14	postive
ATP7A	AC004477.3	0.315813854	4.83E-13	postive
ATP7A	AC004584.3	0.326156725	7.41E-14	postive
ATP7A	AC004596.1	0.386127454	3.18E-19	postive
ATP7A	AC004771.2	0.457997483	2.70E-27	postive
ATP7A	AC004771.4	0.300829394	6.43E-12	postive
ATP7A	AC004893.11	0.437586054	8.38E-25	postive
ATP7A	AC004918.5	0.440959807	3.33E-25	postive
ATP7A	AC004943.2	0.43777308	7.96E-25	postive
ATP7A	AC004951.1	0.394646317	4.41E-20	postive
ATP7A	AC004982.1	0.311243972	1.08E-12	postive
ATP7A	AC004982.2	0.335921868	1.18E-14	postive
ATP7A	AC005034.4	0.394509113	4.55E-20	postive
ATP7A	AC005034.6	0.490789661	1.14E-31	postive
ATP7A	AC005083.1	0.363088883	5.01E-17	postive
ATP7A	AC005154.4	0.343704406	2.61E-15	postive
ATP7A	AC005225.1	0.330785055	3.13E-14	postive
ATP7A	AC005253.1	0.337191242	9.26E-15	postive

ATP7A	AC005288.1	0.500610257	4.50E-33	postive
ATP7A	AC005332.6	0.40996777	1.09E-21	postive
ATP7A	AC005479.1	0.301706859	5.55E-12	postive
ATP7A	AC005498.3	0.32267934	1.40E-13	postive
ATP7A	AC005520.4	0.300885405	6.37E-12	postive
ATP7A	AC005562.1	0.451734339	1.64E-26	postive
ATP7A	AC005670.3	0.552124619	3.08E-41	postive
ATP7A	AC005740.4	0.308219387	1.83E-12	postive
ATP7A	AC006001.2	0.310092882	1.32E-12	postive
ATP7A	AC006001.3	0.314609376	5.98E-13	postive
ATP7A	AC006033.2	0.346247669	1.58E-15	postive
ATP7A	AC006042.3	0.306115547	2.62E-12	postive
ATP7A	AC006160.1	0.307985533	1.90E-12	postive
ATP7A	AC006213.1	0.370331018	1.07E-17	postive
ATP7A	AC006213.2	0.344170911	2.38E-15	postive
ATP7A	AC006333.1	0.306359937	2.52E-12	postive
ATP7A	AC006449.2	0.32651139	6.94E-14	postive
ATP7A	AC006547.1	0.303294007	4.25E-12	postive
ATP7A	AC007285.1	0.329518141	3.97E-14	postive
ATP7A	AC007342.4	0.341200813	4.26E-15	postive
ATP7A	AC007342.5	0.331698466	2.64E-14	postive
ATP7A	AC007389.1	0.305130615	3.11E-12	postive
ATP7A	AC007406.5	0.33420553	1.64E-14	postive
ATP7A	AC007566.1	0.437724993	8.07E-25	postive
ATP7A	AC007637.1	0.323654763	1.17E-13	postive
ATP7A	AC007663.4	0.38760626	2.26E-19	postive
ATP7A	AC007728.3	0.321843743	1.63E-13	postive
ATP7A	AC007780.1	0.351105591	5.95E-16	postive
ATP7A	AC008115.3	0.313246028	7.61E-13	postive
ATP7A	AC008115.4	0.305161614	3.09E-12	postive
ATP7A	AC008124.1	0.385784186	3.44E-19	postive
ATP7A	AC008393.1	0.399915818	1.26E-20	postive
ATP7A	AC008443.1	0.344429733	2.26E-15	postive
ATP7A	AC008494.2	0.36709882	2.14E-17	postive
ATP7A	AC008494.3	0.362642329	5.50E-17	postive
ATP7A	AC008537.2	0.361474624	7.03E-17	postive
ATP7A	AC008655.2	0.322749804	1.39E-13	postive
ATP7A	AC008663.2	0.409253843	1.30E-21	postive
ATP7A	AC008669.1	0.322085112	1.56E-13	postive
ATP7A	AC008764.2	0.431857326	3.92E-24	postive
ATP7A	AC008770.3	0.34962024	8.03E-16	postive
ATP7A	AC008771.1	0.468846315	1.09E-28	postive
ATP7A	AC008840.1	0.349160437	8.81E-16	postive
ATP7A	AC008906.1	0.348663391	9.73E-16	postive
ATP7A	AC008937.3	0.409186938	1.32E-21	postive
ATP7A	AC008966.1	0.435529775	1.46E-24	postive
ATP7A	AC009041.3	0.419896631	8.93E-23	postive
ATP7A	AC009053.3	0.368009481	1.76E-17	postive
ATP7A	AC009090.1	0.306255538	2.56E-12	postive
ATP7A	AC009090.3	0.323367158	1.24E-13	postive

ATP7A	AC009113.1	0.353278882	3.83E-16	postive
ATP7A	AC009120.3	0.376502658	2.76E-18	postive
ATP7A	AC009126.1	0.347028565	1.35E-15	postive
ATP7A	AC009163.7	0.363689469	4.41E-17	postive
ATP7A	AC009237.14	0.390133488	1.26E-19	postive
ATP7A	AC009506.2	0.38366094	5.57E-19	postive
ATP7A	AC009779.3	0.368378148	1.62E-17	postive
ATP7A	AC009831.1	0.342270024	3.45E-15	postive
ATP7A	AC009961.1	0.325291792	8.69E-14	postive
ATP7A	AC010226.1	0.433278066	2.68E-24	postive
ATP7A	AC010260.1	0.333901986	1.74E-14	postive
ATP7A	AC010273.2	0.382214745	7.72E-19	postive
ATP7A	AC010273.3	0.331898993	2.54E-14	postive
ATP7A	AC010834.3	0.322350806	1.49E-13	postive
ATP7A	AC010969.2	0.306384162	2.51E-12	postive
ATP7A	AC011294.1	0.332073819	2.46E-14	postive
ATP7A	AC011346.1	0.387852775	2.14E-19	postive
ATP7A	AC011476.3	0.331026484	2.99E-14	postive
ATP7A	AC011477.2	0.341293296	4.18E-15	postive
ATP7A	AC011477.3	0.39610479	3.12E-20	postive
ATP7A	AC011479.4	0.314470737	6.13E-13	postive
ATP7A	AC011510.1	0.309209928	1.54E-12	postive
ATP7A	AC011726.3	0.348697321	9.67E-16	postive
ATP7A	AC011899.2	0.320153049	2.22E-13	postive
ATP7A	AC011912.1	0.382289601	7.59E-19	postive
ATP7A	AC011921.1	0.321548572	1.72E-13	postive
ATP7A	AC012063.1	0.353597976	3.59E-16	postive
ATP7A	AC012306.2	0.308823204	1.65E-12	postive
ATP7A	AC012313.5	0.460483847	1.30E-27	postive
ATP7A	AC012414.4	0.304388596	3.53E-12	postive
ATP7A	AC012467.2	0.309364697	1.50E-12	postive
ATP7A	AC013356.2	0.309114625	1.57E-12	postive
ATP7A	AC015849.3	0.326162634	7.40E-14	postive
ATP7A	AC015871.3	0.311983001	9.50E-13	postive
ATP7A	AC015922.2	0.374049536	4.74E-18	postive
ATP7A	AC016722.2	0.408996527	1.39E-21	postive
ATP7A	AC016727.1	0.365718156	2.87E-17	postive
ATP7A	AC016747.1	0.392626792	7.08E-20	postive
ATP7A	AC017060.1	0.308472115	1.75E-12	postive
ATP7A	AC018410.1	0.316631445	4.18E-13	postive
ATP7A	AC018521.5	0.300844964	6.42E-12	postive
ATP7A	AC018521.6	0.399882253	1.27E-20	postive
ATP7A	AC018553.1	0.30695418	2.27E-12	postive
ATP7A	AC018645.3	0.341965561	3.67E-15	postive
ATP7A	AC018647.1	0.310612999	1.21E-12	postive
ATP7A	AC018647.2	0.40650684	2.56E-21	postive
ATP7A	AC018752.1	0.356667825	1.91E-16	postive
ATP7A	AC018809.2	0.508901874	2.70E-34	postive
ATP7A	AC019205.1	0.322005197	1.59E-13	postive
ATP7A	AC020904.2	0.317856722	3.35E-13	postive

ATP7A	AC020915.3	0.303062177	4.42E-12	postive
ATP7A	AC020978.1	0.360467881	8.68E-17	postive
ATP7A	AC021016.2	0.314347756	6.26E-13	postive
ATP7A	AC021086.1	0.350222327	7.11E-16	postive
ATP7A	AC021148.1	0.343862508	2.53E-15	postive
ATP7A	AC021224.1	0.316434788	4.32E-13	postive
ATP7A	AC021483.2	0.314381843	6.23E-13	postive
ATP7A	AC022098.1	0.342989461	3.00E-15	postive
ATP7A	AC022217.4	0.33118626	2.90E-14	postive
ATP7A	AC022400.6	0.303382196	4.18E-12	postive
ATP7A	AC022509.3	0.393698335	5.51E-20	postive
ATP7A	AC022613.2	0.311517366	1.03E-12	postive
ATP7A	AC022916.2	0.333204511	1.98E-14	postive
ATP7A	AC023115.1	0.336570713	1.04E-14	postive
ATP7A	AC024075.1	0.468936883	1.06E-28	postive
ATP7A	AC024075.3	0.456624396	4.02E-27	postive
ATP7A	AC024267.4	0.362642289	5.50E-17	postive
ATP7A	AC025048.6	0.33548193	1.28E-14	postive
ATP7A	AC025171.1	0.39926467	1.47E-20	postive
ATP7A	AC025171.2	0.433782888	2.34E-24	postive
ATP7A	AC025287.3	0.370231183	1.09E-17	postive
ATP7A	AC026150.2	0.391155129	9.97E-20	postive
ATP7A	AC026471.1	0.440740578	3.54E-25	postive
ATP7A	AC026691.1	0.359309953	1.11E-16	postive
ATP7A	AC026801.2	0.335941114	1.18E-14	postive
ATP7A	AC026904.2	0.319789965	2.37E-13	postive
ATP7A	AC037459.2	0.308588566	1.71E-12	postive
ATP7A	AC040168.1	0.350277013	7.04E-16	postive
ATP7A	AC044802.2	0.300256408	7.08E-12	postive
ATP7A	AC044860.1	0.310338553	1.27E-12	postive
ATP7A	AC048341.1	0.316757107	4.08E-13	postive
ATP7A	AC060766.6	0.346773064	1.42E-15	postive
ATP7A	AC060766.7	0.440048213	4.28E-25	postive
ATP7A	AC060780.1	0.396935309	2.57E-20	postive
ATP7A	AC063950.1	0.325999783	7.63E-14	postive
ATP7A	AC066613.1	0.327884577	5.38E-14	postive
ATP7A	AC067750.1	0.311151958	1.10E-12	postive
ATP7A	AC067852.2	0.423352474	3.66E-23	postive
ATP7A	AC067852.3	0.384737228	4.36E-19	postive
ATP7A	AC068580.1	0.371538542	8.20E-18	postive
ATP7A	AC068580.3	0.456725024	3.90E-27	postive
ATP7A	AC068790.9	0.320486329	2.09E-13	postive
ATP7A	AC068888.1	0.304211391	3.63E-12	postive
ATP7A	AC073046.1	0.430911542	5.04E-24	postive
ATP7A	AC073073.2	0.41547647	2.75E-22	postive
ATP7A	AC073254.1	0.432007293	3.76E-24	postive
ATP7A	AC073569.2	0.356438104	2.00E-16	postive
ATP7A	AC073896.3	0.35071135	6.45E-16	postive
ATP7A	AC074194.1	0.345208182	1.94E-15	postive
ATP7A	AC078785.1	0.346308935	1.56E-15	postive

ATP7A	AC079807.1	0.398389028	1.82E-20	postive
ATP7A	AC079848.1	0.34845371	1.01E-15	postive
ATP7A	AC079921.1	0.309507124	1.46E-12	postive
ATP7A	AC080112.2	0.367444405	1.98E-17	postive
ATP7A	AC083798.2	0.318525366	2.98E-13	postive
ATP7A	AC083843.1	0.311831236	9.75E-13	postive
ATP7A	AC083964.1	0.337011447	9.58E-15	postive
ATP7A	AC084809.1	0.323911363	1.12E-13	postive
ATP7A	AC087500.1	0.300042547	7.34E-12	postive
ATP7A	AC087854.1	0.316789275	4.06E-13	postive
ATP7A	AC090114.2	0.302990694	4.47E-12	postive
ATP7A	AC090115.1	0.300679226	6.60E-12	postive
ATP7A	AC090198.1	0.334106037	1.67E-14	postive
ATP7A	AC090559.1	0.441783761	2.66E-25	postive
ATP7A	AC090579.1	0.317120921	3.83E-13	postive
ATP7A	AC090587.2	0.367013891	2.18E-17	postive
ATP7A	AC090607.1	0.300589543	6.70E-12	postive
ATP7A	AC090825.1	0.325765729	7.97E-14	postive
ATP7A	AC091057.3	0.380349583	1.17E-18	postive
ATP7A	AC091180.3	0.348825234	9.42E-16	postive
ATP7A	AC091563.1	0.402550842	6.69E-21	postive
ATP7A	AC091982.1	0.416159889	2.31E-22	postive
ATP7A	AC092070.2	0.465142328	3.29E-28	postive
ATP7A	AC092368.3	0.425070509	2.34E-23	postive
ATP7A	AC092376.2	0.362259687	5.96E-17	postive
ATP7A	AC092614.1	0.373835213	4.97E-18	postive
ATP7A	AC092634.3	0.378166741	1.91E-18	postive
ATP7A	AC092718.5	0.312892018	8.10E-13	postive
ATP7A	AC092802.3	0.319109132	2.68E-13	postive
ATP7A	AC092944.1	0.319253275	2.61E-13	postive
ATP7A	AC092953.2	0.329435698	4.03E-14	postive
ATP7A	AC093010.3	0.394516508	4.54E-20	postive
ATP7A	AC093227.1	0.330864985	3.08E-14	postive
ATP7A	AC093278.2	0.3171339	3.82E-13	postive
ATP7A	AC093297.2	0.317011442	3.90E-13	postive
ATP7A	AC093495.1	0.316287351	4.44E-13	postive
ATP7A	AC093525.7	0.310503047	1.23E-12	postive
ATP7A	AC093535.1	0.330692114	3.18E-14	postive
ATP7A	AC093627.5	0.40188747	7.85E-21	postive
ATP7A	AC093726.1	0.439756201	4.64E-25	postive
ATP7A	AC093752.1	0.44792198	4.81E-26	postive
ATP7A	AC093752.3	0.370492875	1.03E-17	postive
ATP7A	AC093864.1	0.318956799	2.75E-13	postive
ATP7A	AC095055.1	0.301000842	6.25E-12	postive
ATP7A	AC096677.1	0.317938457	3.31E-13	postive
ATP7A	AC096733.3	0.439644392	4.78E-25	postive
ATP7A	AC096751.1	0.323170596	1.28E-13	postive
ATP7A	AC096772.6	0.375788179	3.24E-18	postive
ATP7A	AC096992.2	0.343099336	2.94E-15	postive
ATP7A	AC097359.2	0.438700721	6.19E-25	postive

ATP7A	AC097376.3	0.40107495	9.55E-21	postive
ATP7A	AC098484.1	0.515300737	2.92E-35	postive
ATP7A	AC098679.2	0.348869309	9.34E-16	postive
ATP7A	AC099343.4	0.388403939	1.88E-19	postive
ATP7A	AC099794.1	0.307079848	2.22E-12	postive
ATP7A	AC100786.1	0.33789817	8.08E-15	postive
ATP7A	AC100849.1	0.325531976	8.32E-14	postive
ATP7A	AC103719.1	0.302692902	4.70E-12	postive
ATP7A	AC103974.1	0.32547889	8.40E-14	postive
ATP7A	AC104024.2	0.303631243	4.01E-12	postive
ATP7A	AC104083.1	0.526790849	4.77E-37	postive
ATP7A	AC104109.2	0.44731364	5.71E-26	postive
ATP7A	AC104109.4	0.352273246	4.70E-16	postive
ATP7A	AC104123.1	0.310513244	1.23E-12	postive
ATP7A	AC104232.1	0.36486731	3.44E-17	postive
ATP7A	AC104532.2	0.322817217	1.37E-13	postive
ATP7A	AC104596.1	0.359141055	1.15E-16	postive
ATP7A	AC104794.3	0.331025851	2.99E-14	postive
ATP7A	AC104819.3	0.462539564	7.13E-28	postive
ATP7A	AC104825.1	0.359434471	1.08E-16	postive
ATP7A	AC104938.1	0.322918998	1.34E-13	postive
ATP7A	AC105020.6	0.330089278	3.57E-14	postive
ATP7A	AC105036.3	0.34000123	5.38E-15	postive
ATP7A	AC105206.2	0.416285585	2.24E-22	postive
ATP7A	AC105285.1	0.359226393	1.13E-16	postive
ATP7A	AC105935.1	0.326637853	6.78E-14	postive
ATP7A	AC105942.1	0.353067966	4.00E-16	postive
ATP7A	AC106739.1	0.383977256	5.18E-19	postive
ATP7A	AC106786.2	0.509775643	2.00E-34	postive
ATP7A	AC106820.5	0.331615348	2.68E-14	postive
ATP7A	AC106881.1	0.360479842	8.66E-17	postive
ATP7A	AC107027.3	0.42612259	1.78E-23	postive
ATP7A	AC107294.2	0.315734752	4.90E-13	postive
ATP7A	AC107884.1	0.391340042	9.55E-20	postive
ATP7A	AC107959.1	0.362531284	5.63E-17	postive
ATP7A	AC108010.1	0.392041655	8.11E-20	postive
ATP7A	AC108134.2	0.391973684	8.24E-20	postive
ATP7A	AC108156.1	0.417463074	1.66E-22	postive
ATP7A	AC108449.2	0.378572371	1.75E-18	postive
ATP7A	AC108463.2	0.309960787	1.35E-12	postive
ATP7A	AC108693.2	0.333771616	1.78E-14	postive
ATP7A	AC109347.1	0.390752381	1.09E-19	postive
ATP7A	AC109454.2	0.354470572	3.00E-16	postive
ATP7A	AC110079.1	0.391583243	9.03E-20	postive
ATP7A	AC110995.1	0.381400232	9.28E-19	postive
ATP7A	AC112220.2	0.324337064	1.04E-13	postive
ATP7A	AC112694.1	0.36553822	2.98E-17	postive
ATP7A	AC114271.1	0.318986063	2.74E-13	postive
ATP7A	AC114488.2	0.393238575	6.13E-20	postive
ATP7A	AC114763.1	0.31489286	5.69E-13	postive

ATP7A	AC114811.2	0.348802961	9.46E-16	postive
ATP7A	AC114956.2	0.403397188	5.45E-21	postive
ATP7A	AC115618.2	0.447163407	5.95E-26	postive
ATP7A	AC116366.2	0.330896323	3.06E-14	postive
ATP7A	AC116535.2	0.309560054	1.45E-12	postive
ATP7A	AC118344.1	0.300894813	6.36E-12	postive
ATP7A	AC118553.1	0.302767902	4.64E-12	postive
ATP7A	AC119150.1	0.345865787	1.70E-15	postive
ATP7A	AC120114.1	0.371003572	9.21E-18	postive
ATP7A	AC121338.2	0.395535632	3.57E-20	postive
ATP7A	AC122129.1	0.389518912	1.46E-19	postive
ATP7A	AC123912.1	0.347890213	1.14E-15	postive
ATP7A	AC124312.2	0.303329673	4.22E-12	postive
ATP7A	AC125257.1	0.359121937	1.15E-16	postive
ATP7A	AC125494.1	0.327616072	5.66E-14	postive
ATP7A	AC127502.1	0.433851783	2.30E-24	postive
ATP7A	AC133552.2	0.320311703	2.16E-13	postive
ATP7A	AC134775.1	0.328310936	4.97E-14	postive
ATP7A	AC135050.5	0.387745952	2.19E-19	postive
ATP7A	AC135050.6	0.323931113	1.12E-13	postive
ATP7A	AC135178.4	0.344197656	2.37E-15	postive
ATP7A	AC135983.3	0.360972705	7.81E-17	postive
ATP7A	AC136604.2	0.30936506	1.50E-12	postive
ATP7A	AC137630.1	0.308129368	1.86E-12	postive
ATP7A	AC138028.2	0.318687943	2.89E-13	postive
ATP7A	AC138035.1	0.414512934	3.50E-22	postive
ATP7A	AC138207.4	0.321474714	1.75E-13	postive
ATP7A	AC138207.5	0.325019529	9.14E-14	postive
ATP7A	AC138207.6	0.413591983	4.41E-22	postive
ATP7A	AC138207.8	0.459425766	1.78E-27	postive
ATP7A	AC138932.1	0.354920708	2.74E-16	postive
ATP7A	AC138956.1	0.363997576	4.13E-17	postive
ATP7A	AC138956.2	0.421898657	5.34E-23	postive
ATP7A	AC139795.1	0.448711781	3.85E-26	postive
ATP7A	AC139795.2	0.518605677	9.09E-36	postive
ATP7A	AC141586.1	0.422019345	5.17E-23	postive
ATP7A	AC144450.1	0.300830839	6.43E-12	postive
ATP7A	AC145098.1	0.364265939	3.91E-17	postive
ATP7A	AC145124.1	0.309753946	1.40E-12	postive
ATP7A	AC145285.2	0.351030259	6.04E-16	postive
ATP7A	AC145285.3	0.32575018	7.99E-14	postive
ATP7A	AC226118.1	0.428467356	9.62E-24	postive
ATP7A	AC234775.3	0.497705252	1.18E-32	postive
ATP7A	AC241952.2	0.414796712	3.26E-22	postive
ATP7A	AC242426.2	0.390443959	1.18E-19	postive
ATP7A	AC243919.2	0.300773487	6.50E-12	postive
ATP7A	AC245060.2	0.324154203	1.07E-13	postive
ATP7A	AC245140.2	0.331976519	2.50E-14	postive
ATP7A	AC245452.1	0.31703723	3.88E-13	postive
ATP7A	AC254633.1	0.320908017	1.94E-13	postive

ATP7A	ADAMTS9-AS2	0.390290467	1.22E-19	postive
ATP7A	ADGRD1-AS1	0.302326666	5.00E-12	postive
ATP7A	ADGRE4P	0.337096823	9.43E-15	postive
ATP7A	ADGRF5P1	0.374445374	4.35E-18	postive
ATP7A	ADNP-AS1	0.394091014	5.02E-20	postive
ATP7A	ADPGK-AS1	0.314623638	5.97E-13	postive
ATP7A	AF001548.1	0.338948316	6.60E-15	postive
ATP7A	AF111167.2	0.437871982	7.75E-25	postive
ATP7A	AL020996.1	0.352849748	4.18E-16	postive
ATP7A	AL021878.2	0.311254282	1.08E-12	postive
ATP7A	AL022157.1	0.393691463	5.52E-20	postive
ATP7A	AL023881.1	0.332807618	2.14E-14	postive
ATP7A	AL031282.1	0.364081863	4.06E-17	postive
ATP7A	AL031282.2	0.318566653	2.95E-13	postive
ATP7A	AL031320.2	0.373731477	5.09E-18	postive
ATP7A	AL031717.1	0.314031395	6.62E-13	postive
ATP7A	AL031775.1	0.337318039	9.04E-15	postive
ATP7A	AL031846.2	0.330559882	3.26E-14	postive
ATP7A	AL034550.1	0.310155861	1.31E-12	postive
ATP7A	AL035530.2	0.375504745	3.44E-18	postive
ATP7A	AL035587.1	0.392995693	6.49E-20	postive
ATP7A	AL035661.1	0.441390945	2.96E-25	postive
ATP7A	AL049597.2	0.333695923	1.81E-14	postive
ATP7A	AL049779.1	0.370619989	1.00E-17	postive
ATP7A	AL049796.1	0.380161709	1.22E-18	postive
ATP7A	AL049840.2	0.326426295	7.05E-14	postive
ATP7A	AL049840.4	0.431371734	4.46E-24	postive
ATP7A	AL049840.5	0.395790815	3.37E-20	postive
ATP7A	AL050309.1	0.452459342	1.33E-26	postive
ATP7A	AL050343.2	0.319447729	2.52E-13	postive
ATP7A	AL078581.2	0.456235175	4.49E-27	postive
ATP7A	AL078644.2	0.399749275	1.31E-20	postive
ATP7A	AL109804.1	0.333109844	2.02E-14	postive
ATP7A	AL109930.1	0.317993492	3.27E-13	postive
ATP7A	AL117327.1	0.408695859	1.49E-21	postive
ATP7A	AL118506.1	0.385303358	3.83E-19	postive
ATP7A	AL118558.1	0.305819617	2.76E-12	postive
ATP7A	AL121772.3	0.316654262	4.16E-13	postive
ATP7A	AL122035.1	0.571590159	1.04E-44	postive
ATP7A	AL122035.2	0.434272284	2.05E-24	postive
ATP7A	AL132657.1	0.346880722	1.39E-15	postive
ATP7A	AL132780.1	0.343367664	2.79E-15	postive
ATP7A	AL132780.2	0.326266794	7.26E-14	postive
ATP7A	AL132800.1	0.363434136	4.66E-17	postive
ATP7A	AL132989.1	0.307843231	1.95E-12	postive
ATP7A	AL133227.1	0.316678007	4.14E-13	postive
ATP7A	AL133355.1	0.389049539	1.62E-19	postive
ATP7A	AL133371.2	0.413703923	4.29E-22	postive
ATP7A	AL133406.2	0.355784211	2.29E-16	postive
ATP7A	AL133415.1	0.337335066	9.01E-15	postive

ATP7A	AL135791.1	0.358051804	1.44E-16	postive
ATP7A	AL137003.1	0.413862626	4.12E-22	postive
ATP7A	AL137058.2	0.316020539	4.66E-13	postive
ATP7A	AL137779.2	0.319130364	2.67E-13	postive
ATP7A	AL137856.1	0.300809434	6.46E-12	postive
ATP7A	AL138899.1	0.305634886	2.85E-12	postive
ATP7A	AL138921.2	0.32361089	1.18E-13	postive
ATP7A	AL139011.1	0.350171721	7.19E-16	postive
ATP7A	AL139397.1	0.317981562	3.28E-13	postive
ATP7A	AL157394.1	0.352666369	4.34E-16	postive
ATP7A	AL157700.1	0.344955774	2.04E-15	postive
ATP7A	AL157871.5	0.334788423	1.47E-14	postive
ATP7A	AL158212.3	0.338491654	7.21E-15	postive
ATP7A	AL160006.1	0.371342945	8.56E-18	postive
ATP7A	AL161785.1	0.394374814	4.70E-20	postive
ATP7A	AL162231.2	0.439248811	5.33E-25	postive
ATP7A	AL162595.1	0.423064864	3.95E-23	postive
ATP7A	AL162727.2	0.317311555	3.70E-13	postive
ATP7A	AL352984.1	0.318831516	2.82E-13	postive
ATP7A	AL353593.1	0.410278115	1.01E-21	postive
ATP7A	AL353625.1	0.315144111	5.44E-13	postive
ATP7A	AL353743.1	0.332651399	2.20E-14	postive
ATP7A	AL353804.1	0.322637831	1.41E-13	postive
ATP7A	AL354696.2	0.313002966	7.94E-13	postive
ATP7A	AL354740.1	0.337818771	8.21E-15	postive
ATP7A	AL354920.1	0.364164649	3.99E-17	postive
ATP7A	AL355312.2	0.303749211	3.93E-12	postive
ATP7A	AL356019.2	0.32287637	1.35E-13	postive
ATP7A	AL356235.1	0.695646487	1.50E-73	postive
ATP7A	AL356481.1	0.377371892	2.28E-18	postive
ATP7A	AL356599.1	0.500070188	5.39E-33	postive
ATP7A	AL357033.3	0.407835272	1.84E-21	postive
ATP7A	AL357033.4	0.332516506	2.26E-14	postive
ATP7A	AL357140.2	0.327868135	5.40E-14	postive
ATP7A	AL359076.1	0.355141093	2.62E-16	postive
ATP7A	AL359220.1	0.340342961	5.03E-15	postive
ATP7A	AL359921.1	0.334425198	1.57E-14	postive
ATP7A	AL390195.3	0.327018276	6.32E-14	postive
ATP7A	AL390728.4	0.311104078	1.11E-12	postive
ATP7A	AL391121.1	0.326069943	7.53E-14	postive
ATP7A	AL391832.3	0.382391194	7.42E-19	postive
ATP7A	AL391834.1	0.309320209	1.51E-12	postive
ATP7A	AL391834.2	0.323763957	1.15E-13	postive
ATP7A	AL450263.1	0.307684285	2.00E-12	postive
ATP7A	AL450326.1	0.502963461	2.04E-33	postive
ATP7A	AL450998.3	0.330669118	3.20E-14	postive
ATP7A	AL451042.1	0.303388568	4.18E-12	postive
ATP7A	AL451042.2	0.377666273	2.14E-18	postive
ATP7A	AL512791.1	0.347421428	1.25E-15	postive
ATP7A	AL513327.1	0.307358751	2.12E-12	postive

ATP7A	AL513550.1	0.378551703	1.75E-18	postive
ATP7A	AL590385.2	0.31757246	3.53E-13	postive
ATP7A	AL590729.1	0.3057443	2.80E-12	postive
ATP7A	AL591043.2	0.326559809	6.88E-14	postive
ATP7A	AL591684.2	0.367225011	2.08E-17	postive
ATP7A	AL603756.1	0.324471213	1.01E-13	postive
ATP7A	AL603839.2	0.333521741	1.87E-14	postive
ATP7A	AL604028.1	0.363010799	5.09E-17	postive
ATP7A	AL606760.1	0.382345374	7.50E-19	postive
ATP7A	AL606834.1	0.319641347	2.43E-13	postive
ATP7A	AL606834.2	0.337564955	8.62E-15	postive
ATP7A	AL627171.3	0.320076119	2.25E-13	postive
ATP7A	AL627309.5	0.367760645	1.85E-17	postive
ATP7A	AL645728.1	0.308242228	1.82E-12	postive
ATP7A	AL662844.3	0.346781147	1.42E-15	postive
ATP7A	AL669831.1	0.490995627	1.07E-31	postive
ATP7A	AL683813.2	0.326984654	6.36E-14	postive
ATP7A	AL691432.2	0.323911644	1.12E-13	postive
ATP7A	AL691447.2	0.314504741	6.09E-13	postive
ATP7A	AL731563.4	0.340137467	5.24E-15	postive
ATP7A	AL731566.2	0.301229111	6.02E-12	postive
ATP7A	AL731577.2	0.472546097	3.54E-29	postive
ATP7A	AL732314.8	0.34776042	1.17E-15	postive
ATP7A	ALKBH3-AS1	0.303809514	3.89E-12	postive
ATP7A	ALOX12-AS1	0.383176399	6.21E-19	postive
ATP7A	AMZ2P1	0.437420734	8.76E-25	postive
ATP7A	AP000317.1	0.370187741	1.10E-17	postive
ATP7A	AP000317.2	0.351523894	5.47E-16	postive
ATP7A	AP000347.1	0.347557096	1.21E-15	postive
ATP7A	AP000439.1	0.386664705	2.81E-19	postive
ATP7A	AP000442.1	0.368745552	1.50E-17	postive
ATP7A	AP000487.1	0.352183137	4.78E-16	postive
ATP7A	AP000692.1	0.340667721	4.72E-15	postive
ATP7A	AP000759.1	0.368898027	1.45E-17	postive
ATP7A	AP000766.1	0.393092276	6.35E-20	postive
ATP7A	AP000781.1	0.312262783	9.04E-13	postive
ATP7A	AP000787.1	0.313838136	6.85E-13	postive
ATP7A	AP000808.1	0.356347532	2.04E-16	postive
ATP7A	AP000866.1	0.396949593	2.56E-20	postive
ATP7A	AP000873.2	0.303457816	4.13E-12	postive
ATP7A	AP001001.1	0.307786873	1.97E-12	postive
ATP7A	AP001107.1	0.389376456	1.51E-19	postive
ATP7A	AP001160.3	0.316226937	4.49E-13	postive
ATP7A	AP001189.3	0.313681916	7.05E-13	postive
ATP7A	AP001318.2	0.311523075	1.03E-12	postive
ATP7A	AP001330.4	0.327851076	5.42E-14	postive
ATP7A	AP001372.2	0.38017532	1.22E-18	postive
ATP7A	AP001432.1	0.385850398	3.38E-19	postive
ATP7A	AP001462.1	0.309187732	1.55E-12	postive
ATP7A	AP001486.2	0.360597681	8.45E-17	postive

ATP7A	AP001793.1	0.301144668	6.10E-12	postive
ATP7A	AP001922.5	0.318011277	3.26E-13	postive
ATP7A	AP002840.2	0.444979318	1.10E-25	postive
ATP7A	AP002957.1	0.320299517	2.16E-13	postive
ATP7A	AP003059.1	0.354208784	3.17E-16	postive
ATP7A	AP003068.2	0.318789055	2.84E-13	postive
ATP7A	AP003355.2	0.338242391	7.56E-15	postive
ATP7A	AP003392.1	0.316682064	4.14E-13	postive
ATP7A	AP003392.4	0.363413284	4.68E-17	postive
ATP7A	AP003392.5	0.338471857	7.23E-15	postive
ATP7A	AP003486.1	0.501604374	3.22E-33	postive
ATP7A	AP003498.1	0.337058071	9.50E-15	postive
ATP7A	AP003721.4	0.380303861	1.19E-18	postive
ATP7A	AP005717.1	0.336134388	1.13E-14	postive
ATP7A	AP4B1-AS1	0.334245752	1.63E-14	postive
ATP7A	APTR	0.353860134	3.40E-16	postive
ATP7A	ARHGEF26-AS1	0.343648872	2.64E-15	postive
ATP7A	ARMCX5-GPRASP2	0.563393789	3.21E-43	postive
ATP7A	ASB16-AS1	0.415923806	2.45E-22	postive
ATP7A	ATXN1-AS1	0.439393048	5.12E-25	postive
ATP7A	BAALC-AS1	0.369358104	1.32E-17	postive
ATP7A	BAIAP2-DT	0.388529015	1.83E-19	postive
ATP7A	BCDIN3D-AS1	0.356548697	1.96E-16	postive
ATP7A	BDNF-AS	0.528475768	2.57E-37	postive
ATP7A	BMS1P1	0.357167997	1.72E-16	postive
ATP7A	BMS1P20	0.387800377	2.17E-19	postive
ATP7A	BTG3-AS1	0.317891112	3.33E-13	postive
ATP7A	BTN2A3P	0.42927125	7.78E-24	postive
ATP7A	BX322557.10	0.471820949	4.41E-29	postive
ATP7A	BX537318.1	0.321892976	1.62E-13	postive
ATP7A	C1orf147	0.309712619	1.41E-12	postive
ATP7A	C1RL-AS1	0.328150956	5.12E-14	postive
ATP7A	C21orf62-AS1	0.470859948	5.91E-29	postive
ATP7A	C22orf46	0.489860092	1.54E-31	postive
ATP7A	C5orf66	0.300784494	6.48E-12	postive
ATP7A	CA5BP1	0.390307671	1.21E-19	postive
ATP7A	CASC2	0.322580535	1.43E-13	postive
ATP7A	CASP16P	0.302158446	5.14E-12	postive
ATP7A	CATSPER2P1	0.314181486	6.45E-13	postive
ATP7A	CCDC144B	0.301405378	5.84E-12	postive
ATP7A	CCDC84-DT	0.319836177	2.35E-13	postive
ATP7A	CCNT2-AS1	0.334484671	1.55E-14	postive
ATP7A	CD27-AS1	0.320722484	2.00E-13	postive
ATP7A	CD2BP2-DT	0.322445416	1.46E-13	postive
ATP7A	CD81-AS1	0.302255414	5.06E-12	postive
ATP7A	CENPBD1P1	0.401461763	8.70E-21	postive
ATP7A	CERNA1	0.31887997	2.79E-13	postive
ATP7A	CFL1P1	0.410324933	9.97E-22	postive
ATP7A	CHROMR	0.359405602	1.08E-16	postive
ATP7A	CKMT2-AS1	0.437808821	7.89E-25	postive

ATP7A	COX10-AS1	0.454819206	6.76E-27	postive
ATP7A	CRYZL2P	0.331677843	2.65E-14	postive
ATP7A	CTB-119C2.1	0.374444302	4.35E-18	postive
ATP7A	CTBP1-DT	0.450847054	2.10E-26	postive
ATP7A	CTC-205M6.5	0.313234322	7.62E-13	postive
ATP7A	CTC-487M23.5	0.516564635	1.87E-35	postive
ATP7A	CTC-487M23.7	0.35993088	9.71E-17	postive
ATP7A	CTD-2245F17.3	0.515896185	2.37E-35	postive
ATP7A	CTD-2574D22.4	0.385101752	4.01E-19	postive
ATP7A	CUTALP	0.401337486	8.97E-21	postive
ATP7A	CYP2F2P	0.311508619	1.03E-12	postive
ATP7A	DACT3-AS1	0.332555207	2.24E-14	postive
ATP7A	DENND10P1	0.326889931	6.47E-14	postive
ATP7A	DHRS4-AS1	0.312053656	9.38E-13	postive
ATP7A	DLEU7-AS1	0.327151787	6.17E-14	postive
ATP7A	DNM1P51	0.30818487	1.84E-12	postive
ATP7A	DNM3OS	0.326861126	6.51E-14	postive
ATP7A	DNMBP-AS1	0.467910181	1.44E-28	postive
ATP7A	DPH6-DT	0.306778248	2.34E-12	postive
ATP7A	DPY19L2P3	0.402328081	7.06E-21	postive
ATP7A	DSTNP2	0.487353376	3.45E-31	postive
ATP7A	EAF1-AS1	0.348881309	9.32E-16	postive
ATP7A	EBLN3P	0.469020778	1.03E-28	postive
ATP7A	ECI2-DT	0.372394401	6.81E-18	postive
ATP7A	EDRF1-DT	0.301098225	6.15E-12	postive
ATP7A	EIF1B-AS1	0.478768689	5.20E-30	postive
ATP7A	EIF2AK3-DT	0.33907242	6.44E-15	postive
ATP7A	EIF3J-DT	0.467713933	1.53E-28	postive
ATP7A	ELOA-AS1	0.34889888	9.28E-16	postive
ATP7A	EMC1-AS1	0.364237483	3.93E-17	postive
ATP7A	ENO1-IT1	0.363322594	4.77E-17	postive
ATP7A	ENTPD1-AS1	0.398515749	1.76E-20	postive
ATP7A	EP300-AS1	0.320618438	2.04E-13	postive
ATP7A	EPB41L4A-DT	0.470130451	7.37E-29	postive
ATP7A	ERVE-1	0.49753576	1.25E-32	postive
ATP7A	ERVK13-1	0.393697581	5.51E-20	postive
ATP7A	FAHD2CP	0.343570397	2.68E-15	postive
ATP7A	FAM111A-DT	0.537096158	1.04E-38	postive
ATP7A	FAM157B	0.376315456	2.88E-18	postive
ATP7A	FAM157C	0.332890087	2.10E-14	postive
ATP7A	FAM160A1-DT	0.506330406	6.52E-34	postive
ATP7A	FAM21FP	0.309622692	1.43E-12	postive
ATP7A	FAM66B	0.351146196	5.90E-16	postive
ATP7A	FAM66C	0.366854814	2.25E-17	postive
ATP7A	FAM66D	0.36664247	2.36E-17	postive
ATP7A	FAM86C2P	0.362452151	5.73E-17	postive
ATP7A	FAM86DP	0.41618639	2.30E-22	postive
ATP7A	FAM86EP	0.381059338	1.00E-18	postive
ATP7A	FAM87B	0.326325035	7.19E-14	postive
ATP7A	FGD5-AS1	0.490905898	1.10E-31	postive

ATP7A	FGF13-AS1	0.34116427	4.29E-15	postive
ATP7A	FGF7P3	0.44358076	1.62E-25	postive
ATP7A	FHAD1-AS1	0.3359746	1.17E-14	postive
ATP7A	FRG1HP	0.431559542	4.24E-24	postive
ATP7A	FTH1P10	0.309421787	1.48E-12	postive
ATP7A	GAS5-AS1	0.35147073	5.53E-16	postive
ATP7A	GGTA1P	0.34880217	9.46E-16	postive
ATP7A	GHET1	0.356103328	2.15E-16	postive
ATP7A	GLCCI1-DT	0.30388717	3.84E-12	postive
ATP7A	GLIDR	0.33237079	2.32E-14	postive
ATP7A	GMDS-DT	0.409095014	1.35E-21	postive
ATP7A	GNG12-AS1	0.414936451	3.15E-22	postive
ATP7A	GNRHR2	0.352884579	4.15E-16	postive
ATP7A	GOLGA2P10	0.312712831	8.36E-13	postive
ATP7A	GOLGA2P7	0.342239071	3.48E-15	postive
ATP7A	GPRC5D-AS1	0.360712425	8.25E-17	postive
ATP7A	GS1-124K5.3	0.471435237	4.96E-29	postive
ATP7A	GS1-358P8.4	0.580450903	2.28E-46	postive
ATP7A	GTF2IP1	0.41298955	5.13E-22	postive
ATP7A	GTF2IP4	0.457779866	2.87E-27	postive
ATP7A	GUSBP1	0.427389999	1.28E-23	postive
ATP7A	GVINP1	0.309741065	1.40E-12	postive
ATP7A	H3P37	0.331407232	2.78E-14	postive
ATP7A	HAGLR	0.309086696	1.57E-12	postive
ATP7A	HCFC1-AS1	0.305927101	2.71E-12	postive
ATP7A	HCG11	0.40273126	6.41E-21	postive
ATP7A	HCG18	0.42802514	1.08E-23	postive
ATP7A	HCG25	0.346505847	1.50E-15	postive
ATP7A	HECW2-AS1	0.308803314	1.65E-12	postive
ATP7A	HEIH	0.436006435	1.29E-24	postive
ATP7A	HEXA-AS1	0.328236517	5.04E-14	postive
ATP7A	HSP90AB2P	0.410799929	8.86E-22	postive
ATP7A	IL10RB-DT	0.435476519	1.48E-24	postive
ATP7A	IL6R-AS1	0.357780473	1.52E-16	postive
ATP7A	INE1	0.331300389	2.84E-14	postive
ATP7A	IQCH-AS1	0.412556209	5.72E-22	postive
ATP7A	IRF1-AS1	0.307348857	2.12E-12	postive
ATP7A	ITFG1-AS1	0.32805941	5.21E-14	postive
ATP7A	ITGA9-AS1	0.434614385	1.87E-24	postive
ATP7A	JAZF1-AS1	0.366838317	2.26E-17	postive
ATP7A	JPX	0.375684459	3.31E-18	postive
ATP7A	KB-1507C5.4	0.306413436	2.49E-12	postive
ATP7A	KDM7A-DT	0.3104673	1.24E-12	postive
ATP7A	KIAA1671-AS1	0.421150248	6.47E-23	postive
ATP7A	L3MBTL2-AS1	0.421355149	6.14E-23	postive
ATP7A	L3MBTL4-AS1	0.318485159	3.00E-13	postive
ATP7A	LAMTOR5-AS1	0.303185479	4.32E-12	postive
ATP7A	LIFR-AS1	0.323248541	1.26E-13	postive
ATP7A	LINC00205	0.312213193	9.12E-13	postive
ATP7A	LINC00294	0.367582089	1.93E-17	postive

ATP7A	LINC00310	0.315023715	5.56E-13	postive
ATP7A	LINC00324	0.304150949	3.67E-12	postive
ATP7A	LINC00339	0.395839431	3.33E-20	postive
ATP7A	LINC00398	0.378043803	1.96E-18	postive
ATP7A	LINC00471	0.392598825	7.12E-20	postive
ATP7A	LINC00476	0.326828463	6.55E-14	postive
ATP7A	LINC00571	0.488224128	2.61E-31	postive
ATP7A	LINC00578	0.324762026	9.58E-14	postive
ATP7A	LINC00630	0.480534994	2.99E-30	postive
ATP7A	LINC00641	0.309846742	1.38E-12	postive
ATP7A	LINC00649	0.305150984	3.10E-12	postive
ATP7A	LINC00654	0.428695186	9.06E-24	postive
ATP7A	LINC00663	0.428062016	1.07E-23	postive
ATP7A	LINC00667	0.450486139	2.33E-26	postive
ATP7A	LINC00674	0.479828434	3.74E-30	postive
ATP7A	LINC00847	0.408927867	1.41E-21	postive
ATP7A	LINC00863	0.470482204	6.63E-29	postive
ATP7A	LINC00899	0.325260811	8.74E-14	postive
ATP7A	LINC00909	0.342724694	3.16E-15	postive
ATP7A	LINC00910	0.350249105	7.08E-16	postive
ATP7A	LINC00920	0.316399322	4.35E-13	postive
ATP7A	LINC00921	0.354008865	3.30E-16	postive
ATP7A	LINC00933	0.320535039	2.07E-13	postive
ATP7A	LINC00950	0.394570237	4.49E-20	postive
ATP7A	LINC00957	0.358652847	1.27E-16	postive
ATP7A	LINC00963	0.369058732	1.40E-17	postive
ATP7A	LINC00987	0.345613454	1.79E-15	postive
ATP7A	LINC01011	0.341874422	3.73E-15	postive
ATP7A	LINC01016	0.31249012	8.69E-13	postive
ATP7A	LINC01094	0.320409478	2.12E-13	postive
ATP7A	LINC01128	0.456776177	3.84E-27	postive
ATP7A	LINC01135	0.386976504	2.62E-19	postive
ATP7A	LINC01150	0.305430359	2.95E-12	postive
ATP7A	LINC01184	0.493522442	4.69E-32	postive
ATP7A	LINC01278	0.596547833	1.62E-49	postive
ATP7A	LINC01352	0.344551024	2.21E-15	postive
ATP7A	LINC01376	0.306286573	2.55E-12	postive
ATP7A	LINC01422	0.305668314	2.83E-12	postive
ATP7A	LINC01426	0.334568481	1.53E-14	postive
ATP7A	LINC01534	0.414174779	3.81E-22	postive
ATP7A	LINC01547	0.33199906	2.49E-14	postive
ATP7A	LINC01550	0.317161519	3.80E-13	postive
ATP7A	LINC01560	0.306113805	2.63E-12	postive
ATP7A	LINC01569	0.337998109	7.93E-15	postive
ATP7A	LINC01578	0.35635526	2.04E-16	postive
ATP7A	LINC01684	0.30659404	2.42E-12	postive
ATP7A	LINC01719	0.335866058	1.19E-14	postive
ATP7A	LINC01772	0.338446691	7.27E-15	postive
ATP7A	LINC01778	0.33715303	9.33E-15	postive
ATP7A	LINC01852	0.383537159	5.73E-19	postive

ATP7A	LINC01877	0.333027504	2.05E-14	postive
ATP7A	LINC01881	0.507028362	5.13E-34	postive
ATP7A	LINC01936	0.328044267	5.22E-14	postive
ATP7A	LINC01948	0.423021138	3.99E-23	postive
ATP7A	LINC01963	0.332316503	2.35E-14	postive
ATP7A	LINC02019	0.319409688	2.54E-13	postive
ATP7A	LINC02035	0.431675698	4.11E-24	postive
ATP7A	LINC02210	0.390438854	1.18E-19	postive
ATP7A	LINC02234	0.337606927	8.55E-15	postive
ATP7A	LINC02256	0.470874124	5.88E-29	postive
ATP7A	LINC02331	0.302581827	4.79E-12	postive
ATP7A	LINC02361	0.305571323	2.88E-12	postive
ATP7A	LINC02416	0.315366803	5.23E-13	postive
ATP7A	LINC02447	0.311367775	1.06E-12	postive
ATP7A	LINC02519	0.396434113	2.89E-20	postive
ATP7A	LINC02569	0.471153726	5.41E-29	postive
ATP7A	LINC02611	0.330231392	3.47E-14	postive
ATP7A	LINC02625	0.368221329	1.68E-17	postive
ATP7A	LINC02637	0.330679927	3.19E-14	postive
ATP7A	LINC02693	0.403703227	5.06E-21	postive
ATP7A	LINC02705	0.350740754	6.41E-16	postive
ATP7A	LINC02724	0.328588434	4.72E-14	postive
ATP7A	LINC02861	0.367098444	2.14E-17	postive
ATP7A	LMCD1-AS1	0.336905358	9.78E-15	postive
ATP7A	LNCTAM34A	0.322666024	1.41E-13	postive
ATP7A	LPP-AS2	0.345999925	1.66E-15	postive
ATP7A	LRRC37A16P	0.463990839	4.64E-28	postive
ATP7A	LRRC37A17P	0.599261553	4.60E-50	postive
ATP7A	LRRC37BP1	0.440771703	3.51E-25	postive
ATP7A	LRRC8C-DT	0.332017927	2.48E-14	postive
ATP7A	LYPLAL1-DT	0.354720144	2.85E-16	postive
ATP7A	MAGI2-AS3	0.419713446	9.36E-23	postive
ATP7A	MAP2K4P1	0.529202277	1.97E-37	postive
ATP7A	MAP4K3-DT	0.364606128	3.63E-17	postive
ATP7A	MATN1-AS1	0.303847213	3.87E-12	postive
ATP7A	MCM3AP-AS1	0.388404798	1.88E-19	postive
ATP7A	MED14OS	0.388696664	1.76E-19	postive
ATP7A	MEF2C-AS1	0.373111249	5.82E-18	postive
ATP7A	METTL14-DT	0.302372728	4.96E-12	postive
ATP7A	MFF-DT	0.464627413	3.84E-28	postive
ATP7A	MIATNB	0.311702017	9.98E-13	postive
ATP7A	MID1IP1-AS1	0.385291216	3.84E-19	postive
ATP7A	MIF-AS1	0.309531781	1.46E-12	postive
ATP7A	MIR22HG	0.328662573	4.66E-14	postive
ATP7A	MIR29B2CHG	0.311418256	1.05E-12	postive
ATP7A	MIR34AHG	0.408647312	1.51E-21	postive
ATP7A	MIR4453HG	0.321733976	1.67E-13	postive
ATP7A	MKLN1-AS	0.376695258	2.65E-18	postive
ATP7A	MKNK1-AS1	0.40403507	4.67E-21	postive
ATP7A	MMADHC-DT	0.327127521	6.19E-14	postive

ATP7A	MRPL20-AS1	0.383007476	6.46E-19	postive
ATP7A	MRPS31P4	0.416739478	2.00E-22	postive
ATP7A	MRPS31P5	0.401176019	9.32E-21	postive
ATP7A	NBPF25P	0.478354545	5.91E-30	postive
ATP7A	NBPF8	0.360045707	9.48E-17	postive
ATP7A	NBR2	0.40754018	1.98E-21	postive
ATP7A	NCK1-DT	0.319241929	2.62E-13	postive
ATP7A	NDUFA6-DT	0.408061641	1.74E-21	postive
ATP7A	NIFK-AS1	0.384622595	4.48E-19	postive
ATP7A	NINJ2-AS1	0.30562984	2.85E-12	postive
ATP7A	NKAPP1	0.444662244	1.20E-25	postive
ATP7A	NNT-AS1	0.361786262	6.59E-17	postive
ATP7A	NOP14-AS1	0.388595547	1.80E-19	postive
ATP7A	NOP53-AS1	0.324785208	9.54E-14	postive
ATP7A	NORAD	0.535578149	1.84E-38	postive
ATP7A	NPTN-IT1	0.325337045	8.62E-14	postive
ATP7A	NR2F1-AS1	0.375989796	3.10E-18	postive
ATP7A	NR2F2-AS1	0.338639654	7.00E-15	postive
ATP7A	NUDT16P1	0.340020389	5.36E-15	postive
ATP7A	NUTM2A-AS1	0.430056607	6.32E-24	postive
ATP7A	NUTM2B-AS1	0.304500451	3.46E-12	postive
ATP7A	OGFRP1	0.318725834	2.87E-13	postive
ATP7A	OIP5-AS1	0.502905438	2.08E-33	postive
ATP7A	OR2A1-AS1	0.332601093	2.22E-14	postive
ATP7A	OSER1-DT	0.311341124	1.06E-12	postive
ATP7A	OTUD6B-AS1	0.480978012	2.61E-30	postive
ATP7A	PARGP1	0.368091017	1.73E-17	postive
ATP7A	PAXBP1-AS1	0.401171273	9.33E-21	postive
ATP7A	PAXIP1-AS2	0.606896223	1.24E-51	postive
ATP7A	PCBP1-AS1	0.350482787	6.75E-16	postive
ATP7A	PDIA3P1	0.368672922	1.52E-17	postive
ATP7A	PEF1-AS1	0.309963704	1.35E-12	postive
ATP7A	PICART1	0.308720665	1.68E-12	postive
ATP7A	PINK1-AS	0.446672752	6.83E-26	postive
ATP7A	PIPSL	0.343104849	2.93E-15	postive
ATP7A	PITRM1-AS1	0.306154704	2.61E-12	postive
ATP7A	PKD1P6	0.320608609	2.04E-13	postive
ATP7A	PLBD1-AS1	0.399351473	1.44E-20	postive
ATP7A	PLEKHA8P1	0.371911496	7.56E-18	postive
ATP7A	PLEKHM1P1	0.392455767	7.36E-20	postive
ATP7A	PMS2CL	0.393673319	5.54E-20	postive
ATP7A	PMS2P3	0.303477119	4.12E-12	postive
ATP7A	PMS2P4	0.454237718	7.99E-27	postive
ATP7A	POLR2J4	0.330908419	3.06E-14	postive
ATP7A	PPP3CB-AS1	0.417828179	1.51E-22	postive
ATP7A	PRELID1P1	0.344856591	2.08E-15	postive
ATP7A	PRKAR2A-AS1	0.420285411	8.08E-23	postive
ATP7A	PRR34	0.412605322	5.65E-22	postive
ATP7A	PSMA3-AS1	0.353161857	3.92E-16	postive
ATP7A	PTENP1	0.505675007	8.15E-34	postive

ATP7A	PTOV1-AS1	0.335675112	1.24E-14	postive
ATP7A	RAP2C-AS1	0.643781399	7.34E-60	postive
ATP7A	RARA-AS1	0.317316352	3.70E-13	postive
ATP7A	RASSF10-DT	0.302800968	4.62E-12	postive
ATP7A	RBM26-AS1	0.341918529	3.70E-15	postive
ATP7A	RBM5-AS1	0.347791947	1.16E-15	postive
ATP7A	RDM1P5	0.339809418	5.58E-15	postive
ATP7A	RFX3-AS1	0.309643738	1.43E-12	postive
ATP7A	RNASEH2B-AS1	0.349596447	8.07E-16	postive
ATP7A	RNF157-AS1	0.324782229	9.55E-14	postive
ATP7A	RNF213-AS1	0.313609048	7.14E-13	postive
ATP7A	RNF216P1	0.389876606	1.34E-19	postive
ATP7A	RP11-10C24.1	0.371529343	8.22E-18	postive
ATP7A	RP11-159G9.5	0.597016108	1.31E-49	postive
ATP7A	RP11-164P12.4	0.584841522	3.28E-47	postive
ATP7A	RP11-165J3.6	0.323686129	1.17E-13	postive
ATP7A	RP11-175O19.4	0.359891843	9.79E-17	postive
ATP7A	RP11-182J1.1	0.30878777	1.66E-12	postive
ATP7A	RP11-206L10.3	0.353304281	3.81E-16	postive
ATP7A	RP11-206L10.5	0.387626929	2.25E-19	postive
ATP7A	RP11-226L15.5	0.326122065	7.46E-14	postive
ATP7A	RP1-122P22.2	0.510012016	1.84E-34	postive
ATP7A	RP11-282O18.3	0.379569745	1.40E-18	postive
ATP7A	RP11-337C18.9	0.429661204	7.02E-24	postive
ATP7A	RP11-439L18.2	0.350140514	7.23E-16	postive
ATP7A	RP11-462G12.2	0.393853633	5.31E-20	postive
ATP7A	RP11-465K1.2	0.359233102	1.12E-16	postive
ATP7A	RP11-467J12.4	0.333799007	1.77E-14	postive
ATP7A	RP11-524D16__A.3	0.393145345	6.27E-20	postive
ATP7A	RP11-539I5.1	0.479470394	4.18E-30	postive
ATP7A	RP11-540B6.6	0.317910951	3.32E-13	postive
ATP7A	RP11-58E21.3	0.314542868	6.05E-13	postive
ATP7A	RP11-86H7.7	0.356010242	2.19E-16	postive
ATP7A	RP11-876N24.4	0.4164553	2.15E-22	postive
ATP7A	RP11-876N24.5	0.452125532	1.46E-26	postive
ATP7A	RP11-89K10.1	0.340103244	5.27E-15	postive
ATP7A	RP13-36G14.4	0.431745909	4.04E-24	postive
ATP7A	RP1-93H18.1	0.321988069	1.59E-13	postive
ATP7A	RP3-337H4.10	0.34667355	1.45E-15	postive
ATP7A	RP3-368A4.5	0.431476494	4.34E-24	postive
ATP7A	RP5-1042I8.7	0.409838542	1.13E-21	postive
ATP7A	RP5-1125A11.6	0.314669449	5.92E-13	postive
ATP7A	RP5-1139B12.2	0.358666034	1.26E-16	postive
ATP7A	RPARP-AS1	0.308538938	1.73E-12	postive
ATP7A	RPL13P5	0.495344403	2.58E-32	postive
ATP7A	RPL23AP53	0.468956951	1.05E-28	postive
ATP7A	RPL23AP7	0.368379212	1.62E-17	postive
ATP7A	RPL23AP82	0.452992918	1.14E-26	postive
ATP7A	RPL32P3	0.346235832	1.58E-15	postive
ATP7A	RPS10P7	0.360245001	9.10E-17	postive

ATP7A	RPS4XP16	0.371030825	9.16E-18	postive
ATP7A	RRN3P3	0.347266937	1.29E-15	postive
ATP7A	RTCA-AS1	0.392295549	7.65E-20	postive
ATP7A	RXYLT1-AS1	0.316897668	3.98E-13	postive
ATP7A	SAMD12-AS1	0.371959213	7.49E-18	postive
ATP7A	SAP30L-AS1	0.391982776	8.22E-20	postive
ATP7A	SBDSP1	0.418398317	1.31E-22	postive
ATP7A	SBF2-AS1	0.305121184	3.11E-12	postive
ATP7A	SCAMP1-AS1	0.320831066	1.96E-13	postive
ATP7A	SCAND2P	0.467758949	1.51E-28	postive
ATP7A	SDAD1P1	0.457062956	3.54E-27	postive
ATP7A	SDCBP2-AS1	0.315803914	4.84E-13	postive
ATP7A	SEC22B4P	0.422325511	4.78E-23	postive
ATP7A	SEC24B-AS1	0.393994772	5.14E-20	postive
ATP7A	SEPTIN7-AS1	0.571378953	1.13E-44	postive
ATP7A	SEPTIN7P2	0.342977291	3.01E-15	postive
ATP7A	SGMS1-AS1	0.401058834	9.59E-21	postive
ATP7A	SGOL1-AS1	0.310108459	1.32E-12	postive
ATP7A	SH3RF3-AS1	0.333504149	1.87E-14	postive
ATP7A	SIGLEC22P	0.390131698	1.26E-19	postive
ATP7A	SLC25A5-AS1	0.342696798	3.18E-15	postive
ATP7A	SLC44A3-AS1	0.376376795	2.84E-18	postive
ATP7A	SLC5A4-AS1	0.37819753	1.90E-18	postive
ATP7A	SLC9A7P1	0.367096247	2.14E-17	postive
ATP7A	SMC5-AS1	0.363228449	4.86E-17	postive
ATP7A	SMG7-AS1	0.443287599	1.75E-25	postive
ATP7A	SMIM15-AS1	0.308167771	1.84E-12	postive
ATP7A	SNAI3-AS1	0.317444279	3.61E-13	postive
ATP7A	SNHG16	0.349411057	8.38E-16	postive
ATP7A	SNHG31	0.529297608	1.90E-37	postive
ATP7A	SP2-AS1	0.392146348	7.92E-20	postive
ATP7A	SPAG5-AS1	0.33291057	2.10E-14	postive
ATP7A	SREBF2-AS1	0.310543283	1.22E-12	postive
ATP7A	SSR4P1	0.325542704	8.30E-14	postive
ATP7A	ST20-AS1	0.306341123	2.53E-12	postive
ATP7A	ST3GAL5-AS1	0.326742243	6.65E-14	postive
ATP7A	STAG3L1	0.318470936	3.00E-13	postive
ATP7A	STAG3L2	0.379282177	1.49E-18	postive
ATP7A	STAG3L3	0.394103978	5.01E-20	postive
ATP7A	STARD7-AS1	0.360724163	8.23E-17	postive
ATP7A	STX18-AS1	0.413717685	4.28E-22	postive
ATP7A	SUCLG2-AS1	0.369304157	1.33E-17	postive
ATP7A	SUMO2P17	0.315727056	4.91E-13	postive
ATP7A	SVIL2P	0.398547973	1.75E-20	postive
ATP7A	SVIL-AS1	0.469156574	9.89E-29	postive
ATP7A	SYNPR-AS1	0.306316287	2.54E-12	postive
ATP7A	TBC1D8-AS1	0.31218551	9.17E-13	postive
ATP7A	TBX5-AS1	0.418786824	1.19E-22	postive
ATP7A	TCAF2P1	0.326618448	6.81E-14	postive
ATP7A	THAP9-AS1	0.347338044	1.27E-15	postive

ATP7A	TMEM161B-AS1	0.403570483	5.23E-21	postive
ATP7A	TMEM202-AS1	0.31798637	3.28E-13	postive
ATP7A	TMEM231P1	0.338851755	6.72E-15	postive
ATP7A	TMEM51-AS1	0.311791085	9.82E-13	postive
ATP7A	TMEM99	0.315369666	5.23E-13	postive
ATP7A	TMEM9B-AS1	0.447079389	6.09E-26	postive
ATP7A	TNFRSF10A-AS1	0.326395752	7.09E-14	postive
ATP7A	TP73-AS1	0.441272587	3.06E-25	postive
ATP7A	TPT1-AS1	0.305733511	2.80E-12	postive
ATP7A	TRAF3IP2-AS1	0.40563373	3.16E-21	postive
ATP7A	TRAM2-AS1	0.364570356	3.66E-17	postive
ATP7A	TSC22D1-AS1	0.315152817	5.43E-13	postive
ATP7A	TSPOAP1-AS1	0.342264863	3.46E-15	postive
ATP7A	TSTD3	0.366493353	2.43E-17	postive
ATP7A	TTC28-AS1	0.408511549	1.56E-21	postive
ATP7A	TTN-AS1	0.411968432	6.63E-22	postive
ATP7A	TUG1	0.473256961	2.85E-29	postive
ATP7A	U52111.1	0.302920445	4.52E-12	postive
ATP7A	U73169.1	0.319480402	2.51E-13	postive
ATP7A	UBA6-AS1	0.418867437	1.16E-22	postive
ATP7A	UBE2D3-AS1	0.373558847	5.28E-18	postive
ATP7A	UBE2Q2P2	0.387605946	2.26E-19	postive
ATP7A	UGDH-AS1	0.443821891	1.51E-25	postive
ATP7A	USP27X-AS1	0.496601646	1.71E-32	postive
ATP7A	USP46-AS1	0.401619254	8.38E-21	postive
ATP7A	UXT-AS1	0.338599747	7.06E-15	postive
ATP7A	VDAC1P8	0.347692658	1.18E-15	postive
ATP7A	WAC-AS1	0.421638537	5.71E-23	postive
ATP7A	WARS2-AS1	0.397704383	2.14E-20	postive
ATP7A	WDFY3-AS2	0.479409443	4.26E-30	postive
ATP7A	WEE2-AS1	0.316018723	4.66E-13	postive
ATP7A	WNT5A-AS1	0.41131553	7.80E-22	postive
ATP7A	Z68871.1	0.595963212	2.13E-49	postive
ATP7A	Z97353.2	0.339546098	5.88E-15	postive
ATP7A	Z97634.1	0.335730777	1.23E-14	postive
ATP7A	Z97989.1	0.39069979	1.11E-19	postive
ATP7A	Z99572.1	0.37646064	2.79E-18	postive
ATP7A	ZBTB11-AS1	0.395987476	3.21E-20	postive
ATP7A	ZBTB44-DT	0.331841881	2.56E-14	postive
ATP7A	ZKSCAN7-AS1	0.367219391	2.08E-17	postive
ATP7A	ZNF137P	0.380080137	1.25E-18	postive
ATP7A	ZNF205-AS1	0.389517854	1.46E-19	postive
ATP7A	ZNF213-AS1	0.30353088	4.08E-12	postive
ATP7A	ZNF252P	0.484893367	7.57E-31	postive
ATP7A	ZNF32-AS2	0.305184865	3.08E-12	postive
ATP7A	ZNF37BP	0.323327754	1.25E-13	postive
ATP7A	ZNF436-AS1	0.328817189	4.52E-14	postive
ATP7A	ZNF56	0.30574495	2.80E-12	postive
ATP7A	ZNF561-AS1	0.349002498	9.09E-16	postive
ATP7A	ZNF658B	0.534348025	2.92E-38	postive

ATP7A	ZNF702P	0.317333099	3.68E-13	postive
ATP7A	ZNF767P	0.34355987	2.68E-15	postive
ATP7A	ZNF815P	0.350772909	6.37E-16	postive
ATP7A	ZNRD1ASP	0.380244589	1.20E-18	postive
ATP7A	ZNRD2-AS1	0.319523258	2.49E-13	postive
ATP7A	ZNRF2P1	0.379691954	1.36E-18	postive
ATP7A	ZNRF2P2	0.38594949	3.31E-19	postive
ATP7A	ZRANB2-AS2	0.310855583	1.16E-12	postive
ATP7B	A2ML1-AS1	0.328627365	4.69E-14	postive
ATP7B	AC002511.1	0.315573606	5.04E-13	postive
ATP7B	AC004009.1	0.337550842	8.64E-15	postive
ATP7B	AC004596.1	0.315966117	4.70E-13	postive
ATP7B	AC004803.1	0.300871453	6.39E-12	postive
ATP7B	AC004923.1	0.329007849	4.37E-14	postive
ATP7B	AC005082.1	0.36536014	3.10E-17	postive
ATP7B	AC005288.1	0.359143754	1.14E-16	postive
ATP7B	AC005828.4	0.301130246	6.12E-12	postive
ATP7B	AC007098.1	0.368231929	1.68E-17	postive
ATP7B	AC007255.1	0.375342577	3.57E-18	postive
ATP7B	AC007365.1	0.304841569	3.26E-12	postive
ATP7B	AC007681.1	0.358276934	1.37E-16	postive
ATP7B	AC007922.4	0.367442602	1.99E-17	postive
ATP7B	AC007996.1	0.324685781	9.72E-14	postive
ATP7B	AC008124.1	0.303161483	4.34E-12	postive
ATP7B	AC008443.3	0.355600046	2.38E-16	postive
ATP7B	AC008514.1	0.369232626	1.35E-17	postive
ATP7B	AC010857.1	0.328056314	5.21E-14	postive
ATP7B	AC011005.4	0.30727041	2.15E-12	postive
ATP7B	AC011298.1	0.315329125	5.26E-13	postive
ATP7B	AC011450.1	0.307527674	2.06E-12	postive
ATP7B	AC012363.2	0.348481928	1.01E-15	postive
ATP7B	AC012467.2	0.339629008	5.78E-15	postive
ATP7B	AC013565.1	0.42697319	1.43E-23	postive
ATP7B	AC015522.1	0.305460376	2.94E-12	postive
ATP7B	AC015849.5	0.318737094	2.86E-13	postive
ATP7B	AC016825.1	0.323904876	1.12E-13	postive
ATP7B	AC016868.1	0.402492252	6.79E-21	postive
ATP7B	AC020779.2	0.354671319	2.88E-16	postive
ATP7B	AC021092.1	0.332005722	2.49E-14	postive
ATP7B	AC022031.1	0.320453225	2.10E-13	postive
ATP7B	AC024230.1	0.339635166	5.77E-15	postive
ATP7B	AC026412.3	0.311806179	9.80E-13	postive
ATP7B	AC046143.1	0.346256304	1.57E-15	postive
ATP7B	AC046168.1	0.371806043	7.74E-18	postive
ATP7B	AC061975.6	0.316029858	4.65E-13	postive
ATP7B	AC067960.1	0.358987841	1.18E-16	postive
ATP7B	AC068756.1	0.313909669	6.77E-13	postive
ATP7B	AC068790.9	0.34967702	7.94E-16	postive
ATP7B	AC068831.2	0.308067271	1.88E-12	postive
ATP7B	AC069285.2	0.327665546	5.60E-14	postive

ATP7B	AC073046.1	0.376216479	2.94E-18	postive
ATP7B	AC073529.1	0.315880968	4.77E-13	postive
ATP7B	AC074135.1	0.300537256	6.76E-12	postive
ATP7B	AC079807.1	0.320821884	1.97E-13	postive
ATP7B	AC092447.8	0.3192599	2.61E-13	postive
ATP7B	AC092687.3	0.340448562	4.93E-15	postive
ATP7B	AC093802.1	0.329581815	3.92E-14	postive
ATP7B	AC103702.2	0.350832423	6.29E-16	postive
ATP7B	AC106872.9	0.320054543	2.26E-13	postive
ATP7B	AC108676.1	0.321562013	1.72E-13	postive
ATP7B	AC111149.2	0.31113916	1.10E-12	postive
ATP7B	AC124947.2	0.394721176	4.33E-20	postive
ATP7B	AC125494.1	0.303768593	3.92E-12	postive
ATP7B	AC127526.2	0.350269727	7.05E-16	postive
ATP7B	AC128707.1	0.379085011	1.56E-18	postive
ATP7B	AC131097.2	0.370898073	9.43E-18	postive
ATP7B	AC131235.3	0.30873074	1.67E-12	postive
ATP7B	AC138761.1	0.324845241	9.44E-14	postive
ATP7B	ACVR2B-AS1	0.327163378	6.15E-14	postive
ATP7B	AF064860.2	0.37105651	9.11E-18	postive
ATP7B	AF233439.1	0.317105369	3.84E-13	postive
ATP7B	AL008721.1	0.307967404	1.91E-12	postive
ATP7B	AL022326.1	0.336915777	9.76E-15	postive
ATP7B	AL035458.2	0.319933882	2.31E-13	postive
ATP7B	AL049838.1	0.324658388	9.77E-14	postive
ATP7B	AL117329.1	0.366329085	2.52E-17	postive
ATP7B	AL121761.1	0.365010288	3.33E-17	postive
ATP7B	AL121790.2	0.33571902	1.23E-14	postive
ATP7B	AL133215.2	0.305840782	2.75E-12	postive
ATP7B	AL135878.1	0.321475372	1.75E-13	postive
ATP7B	AL137058.2	0.302404636	4.94E-12	postive
ATP7B	AL139035.1	0.31778324	3.40E-13	postive
ATP7B	AL139082.1	0.385541387	3.63E-19	postive
ATP7B	AL139383.1	0.38870413	1.76E-19	postive
ATP7B	AL158206.1	0.392223275	7.78E-20	postive
ATP7B	AL161668.4	0.32138473	1.78E-13	postive
ATP7B	AL161719.1	0.415825991	2.52E-22	postive
ATP7B	AL161729.1	0.319430052	2.53E-13	postive
ATP7B	AL161891.1	0.41153851	7.38E-22	postive
ATP7B	AL354892.2	0.31226293	9.04E-13	postive
ATP7B	AL354993.2	0.33669182	1.02E-14	postive
ATP7B	AL358473.1	0.33810753	7.76E-15	postive
ATP7B	AL359711.2	0.301226134	6.02E-12	postive
ATP7B	AL390964.1	0.325451862	8.44E-14	postive
ATP7B	AL391152.1	0.312670591	8.42E-13	postive
ATP7B	AL592546.2	0.338930711	6.62E-15	postive
ATP7B	AL731577.2	0.321965085	1.60E-13	postive
ATP7B	AL928654.1	0.308614541	1.71E-12	postive
ATP7B	AP000977.1	0.304269101	3.60E-12	postive
ATP7B	AP002754.2	0.321060879	1.88E-13	postive

ATP7B	AP003555.2	0.314957169	5.62E-13	postive
ATP7B	ATXN8OS	0.331556538	2.71E-14	postive
ATP7B	B3GALT5-AS1	0.393787653	5.39E-20	postive
ATP7B	BOLA2P2	0.308934049	1.62E-12	postive
ATP7B	BOLA3-AS1	0.359935189	9.71E-17	postive
ATP7B	CASC11	0.306615248	2.41E-12	postive
ATP7B	CENPBD1P1	0.301505213	5.74E-12	postive
ATP7B	DCXR-DT	0.320897775	1.94E-13	postive
ATP7B	DHRS4-AS1	0.355046427	2.67E-16	postive
ATP7B	DPY19L2P3	0.321636407	1.70E-13	postive
ATP7B	EIF2AK3-DT	0.302671408	4.72E-12	postive
ATP7B	EIPR1-IT1	0.350425713	6.83E-16	postive
ATP7B	ELF3-AS1	0.367510222	1.96E-17	postive
ATP7B	FAM3D-AS1	0.316393253	4.36E-13	postive
ATP7B	FGD5P1	0.404204485	4.48E-21	postive
ATP7B	FMO6P	0.328859939	4.49E-14	postive
ATP7B	GACAT3	0.336294034	1.10E-14	postive
ATP7B	H19	0.316509711	4.27E-13	postive
ATP7B	HERC2P5	0.304121677	3.69E-12	postive
ATP7B	HERC2P8	0.359825069	9.93E-17	postive
ATP7B	HOXA10-AS	0.310103581	1.32E-12	postive
ATP7B	HOXA-AS3	0.3641576	4.00E-17	postive
ATP7B	LINC00303	0.30344332	4.14E-12	postive
ATP7B	LINC00377	0.347725947	1.17E-15	postive
ATP7B	LINC00449	0.401735714	8.15E-21	postive
ATP7B	LINC00567	0.344018296	2.45E-15	postive
ATP7B	LINC00652	0.413862255	4.13E-22	postive
ATP7B	LINC00871	0.341628456	3.92E-15	postive
ATP7B	LINC00954	0.345738434	1.74E-15	postive
ATP7B	LINC01219	0.318662165	2.90E-13	postive
ATP7B	LINC01419	0.329372164	4.08E-14	postive
ATP7B	LINC01521	0.314517472	6.08E-13	postive
ATP7B	LINC01560	0.310865428	1.15E-12	postive
ATP7B	LINC01606	0.354849553	2.78E-16	postive
ATP7B	LINC01639	0.309795358	1.39E-12	postive
ATP7B	LINC01754	0.32015577	2.22E-13	postive
ATP7B	LINC01819	0.334758993	1.48E-14	postive
ATP7B	LINC01881	0.312900986	8.08E-13	postive
ATP7B	LINC01887	0.336738552	1.01E-14	postive
ATP7B	LINC02020	0.307833353	1.95E-12	postive
ATP7B	LINC02035	0.305959418	2.70E-12	postive
ATP7B	LINC02037	0.327340976	5.95E-14	postive
ATP7B	LINC02068	0.360666149	8.33E-17	postive
ATP7B	LINC02163	0.360711999	8.25E-17	postive
ATP7B	LINC02253	0.320835609	1.96E-13	postive
ATP7B	LINC02381	0.507459344	4.43E-34	postive
ATP7B	LINC02387	0.339652482	5.76E-15	postive
ATP7B	LINC02438	0.395812819	3.35E-20	postive
ATP7B	LINC02623	0.314590594	6.00E-13	postive
ATP7B	LINC02763	0.356377931	2.03E-16	postive

ATP7B	LRP4-AS1	0.337720109	8.36E-15	postive
ATP7B	MIPEPP3	0.398635377	1.71E-20	postive
ATP7B	MRPL23-AS1	0.406376118	2.64E-21	postive
ATP7B	MRPS31P4	0.385554215	3.62E-19	postive
ATP7B	MRPS31P5	0.311948044	9.56E-13	postive
ATP7B	MYCL-AS1	0.39761323	2.19E-20	postive
ATP7B	OR7E14P	0.366087609	2.65E-17	postive
ATP7B	PPY2P	0.350354422	6.93E-16	postive
ATP7B	PSMD10P2	0.320542121	2.07E-13	postive
ATP7B	RAB6C-AS1	0.322665219	1.41E-13	postive
ATP7B	RN7SL832P	0.305774923	2.78E-12	postive
ATP7B	RNASEH1-AS1	0.348879749	9.32E-16	postive
ATP7B	RP1-122P22.2	0.301250023	6.00E-12	postive
ATP7B	RP11-470E16.2	0.316687569	4.13E-13	postive
ATP7B	RP11-815I9.4	0.378037596	1.97E-18	postive
ATP7B	SNHG11	0.305106654	3.12E-12	postive
ATP7B	SOX21-AS1	0.357842108	1.50E-16	postive
ATP7B	SOX2-OT	0.363857248	4.26E-17	postive
ATP7B	STARD7-AS1	0.341053641	4.38E-15	postive
ATP7B	TBC1D8-AS1	0.347759485	1.17E-15	postive
ATP7B	TMPO-AS1	0.303353597	4.20E-12	postive
ATP7B	TPRXL	0.358284005	1.37E-16	postive
ATP7B	TSPY26P	0.383630959	5.61E-19	postive
ATP7B	TTC41P	0.332112281	2.44E-14	postive
ATP7B	UBAC2-AS1	0.318324447	3.08E-13	postive
ATP7B	Z99572.1	0.330466982	3.32E-14	postive
ATP7B	ZEB2P1	0.313466793	7.32E-13	postive
ATP7B	ZNF137P	0.301453645	5.79E-12	postive
ATP7B	ZNF286B	0.314127282	6.51E-13	postive
ATP7B	ZNRF2P2	0.319193955	2.64E-13	postive
CCDC22	A1BG-AS1	0.303995702	3.77E-12	postive
CCDC22	A2M-AS1	0.34712337	1.32E-15	postive
CCDC22	ABALON	0.368649489	1.53E-17	postive
CCDC22	AC000068.1	0.42195592	5.26E-23	postive
CCDC22	AC000068.2	0.391672686	8.84E-20	postive
CCDC22	AC002091.1	0.322805337	1.37E-13	postive
CCDC22	AC002091.2	0.340090914	5.29E-15	postive
CCDC22	AC002116.2	0.428363325	9.89E-24	postive
CCDC22	AC002310.1	0.423432245	3.59E-23	postive
CCDC22	AC002398.1	0.36020497	9.17E-17	postive
CCDC22	AC002401.2	0.337988113	7.94E-15	postive
CCDC22	AC002467.1	0.403480036	5.34E-21	postive
CCDC22	AC002470.1	0.445563516	9.31E-26	postive
CCDC22	AC002550.2	0.300635402	6.65E-12	postive
CCDC22	AC003070.1	0.316084129	4.60E-13	postive
CCDC22	AC003101.2	0.328724059	4.60E-14	postive
CCDC22	AC003102.1	0.380338128	1.18E-18	postive
CCDC22	AC003956.1	0.386969539	2.62E-19	postive
CCDC22	AC004067.1	0.331181197	2.90E-14	postive
CCDC22	AC004156.1	0.374969742	3.88E-18	postive

CCDC22	AC004233.1	0.302293539	5.03E-12	postive
CCDC22	AC004241.1	0.430030931	6.37E-24	postive
CCDC22	AC004241.3	0.323177814	1.28E-13	postive
CCDC22	AC004253.1	0.326225496	7.32E-14	postive
CCDC22	AC004263.1	0.300016324	7.37E-12	postive
CCDC22	AC004447.2	0.477439329	7.86E-30	postive
CCDC22	AC004471.2	0.314063816	6.59E-13	postive
CCDC22	AC004477.1	0.352455031	4.53E-16	postive
CCDC22	AC004477.3	0.344916899	2.05E-15	postive
CCDC22	AC004540.1	0.318503983	2.99E-13	postive
CCDC22	AC004596.1	0.463719159	5.03E-28	postive
CCDC22	AC004771.2	0.524628478	1.05E-36	postive
CCDC22	AC004771.4	0.470417207	6.76E-29	postive
CCDC22	AC004801.6	0.351006814	6.07E-16	postive
CCDC22	AC004803.1	0.373788464	5.02E-18	postive
CCDC22	AC004812.2	0.470593868	6.41E-29	postive
CCDC22	AC004825.2	0.325258386	8.75E-14	postive
CCDC22	AC004854.2	0.301693093	5.56E-12	postive
CCDC22	AC004865.2	0.303438153	4.14E-12	postive
CCDC22	AC004890.2	0.328133516	5.14E-14	postive
CCDC22	AC004893.11	0.422321109	4.79E-23	postive
CCDC22	AC004943.2	0.439995829	4.34E-25	postive
CCDC22	AC004973.1	0.355938579	2.22E-16	postive
CCDC22	AC004980.1	0.352855046	4.17E-16	postive
CCDC22	AC005005.3	0.48117917	2.45E-30	postive
CCDC22	AC005034.4	0.438503453	6.53E-25	postive
CCDC22	AC005034.5	0.342898553	3.05E-15	postive
CCDC22	AC005034.6	0.40534748	3.39E-21	postive
CCDC22	AC005041.3	0.379313708	1.48E-18	postive
CCDC22	AC005076.2	0.364926525	3.39E-17	postive
CCDC22	AC005225.3	0.328563927	4.74E-14	postive
CCDC22	AC005229.4	0.453995336	8.57E-27	postive
CCDC22	AC005253.1	0.416924247	1.90E-22	postive
CCDC22	AC005261.4	0.323123307	1.29E-13	postive
CCDC22	AC005288.1	0.553262854	1.96E-41	postive
CCDC22	AC005332.1	0.356872128	1.83E-16	postive
CCDC22	AC005332.4	0.463640238	5.15E-28	postive
CCDC22	AC005387.1	0.409137095	1.34E-21	postive
CCDC22	AC005387.2	0.379764211	1.34E-18	postive
CCDC22	AC005498.3	0.308033226	1.89E-12	postive
CCDC22	AC005519.1	0.35204322	4.92E-16	postive
CCDC22	AC005520.4	0.355161215	2.61E-16	postive
CCDC22	AC005562.1	0.508268541	3.36E-34	postive
CCDC22	AC005586.1	0.420563114	7.53E-23	postive
CCDC22	AC005625.1	0.37995714	1.28E-18	postive
CCDC22	AC005670.3	0.546065346	3.33E-40	postive
CCDC22	AC005695.1	0.327378199	5.91E-14	postive
CCDC22	AC005837.1	0.432500973	3.30E-24	postive
CCDC22	AC005840.4	0.30285734	4.57E-12	postive
CCDC22	AC005911.1	0.35293101	4.11E-16	postive

CCDC22	AC006001.2	0.385351992	3.79E-19	postive
CCDC22	AC006001.3	0.372030678	7.37E-18	postive
CCDC22	AC006126.4	0.314456274	6.14E-13	postive
CCDC22	AC006213.1	0.351570906	5.42E-16	postive
CCDC22	AC006252.1	0.439690569	4.72E-25	postive
CCDC22	AC006254.1	0.308547715	1.73E-12	postive
CCDC22	AC006333.1	0.399921006	1.26E-20	postive
CCDC22	AC006441.4	0.354728835	2.85E-16	postive
CCDC22	AC006449.2	0.490477414	1.26E-31	postive
CCDC22	AC006449.3	0.307320024	2.13E-12	postive
CCDC22	AC006449.5	0.40092173	9.91E-21	postive
CCDC22	AC006449.7	0.450486941	2.33E-26	postive
CCDC22	AC006504.5	0.300126478	7.24E-12	postive
CCDC22	AC006547.1	0.456984639	3.62E-27	postive
CCDC22	AC006942.1	0.352953761	4.09E-16	postive
CCDC22	AC007032.1	0.371115759	8.99E-18	postive
CCDC22	AC007066.2	0.330390326	3.37E-14	postive
CCDC22	AC007098.1	0.332484323	2.27E-14	postive
CCDC22	AC007114.1	0.393477663	5.80E-20	postive
CCDC22	AC007292.1	0.4356113	1.43E-24	postive
CCDC22	AC007292.2	0.429969527	6.47E-24	postive
CCDC22	AC007383.2	0.374037303	4.76E-18	postive
CCDC22	AC007406.5	0.384876262	4.23E-19	postive
CCDC22	AC007422.1	0.32763195	5.64E-14	postive
CCDC22	AC007541.1	0.429457383	7.41E-24	postive
CCDC22	AC007663.4	0.405864492	2.99E-21	postive
CCDC22	AC007686.3	0.328727066	4.60E-14	postive
CCDC22	AC007881.3	0.335298528	1.33E-14	postive
CCDC22	AC008105.1	0.340215783	5.16E-15	postive
CCDC22	AC008105.2	0.38168321	8.70E-19	postive
CCDC22	AC008124.1	0.400406438	1.12E-20	postive
CCDC22	AC008267.5	0.46856209	1.18E-28	postive
CCDC22	AC008280.3	0.301703541	5.55E-12	postive
CCDC22	AC008393.1	0.432670699	3.15E-24	postive
CCDC22	AC008438.1	0.433137711	2.78E-24	postive
CCDC22	AC008443.1	0.471332542	5.12E-29	postive
CCDC22	AC008443.5	0.378354998	1.83E-18	postive
CCDC22	AC008494.3	0.421241509	6.32E-23	postive
CCDC22	AC008543.1	0.35882583	1.22E-16	postive
CCDC22	AC008608.2	0.358360091	1.35E-16	postive
CCDC22	AC008622.2	0.329396583	4.06E-14	postive
CCDC22	AC008655.2	0.316519717	4.26E-13	postive
CCDC22	AC008734.1	0.361485783	7.02E-17	postive
CCDC22	AC008735.1	0.361415211	7.12E-17	postive
CCDC22	AC008735.2	0.364157665	4.00E-17	postive
CCDC22	AC008735.4	0.339240889	6.23E-15	postive
CCDC22	AC008747.1	0.388444144	1.87E-19	postive
CCDC22	AC008764.2	0.348306289	1.05E-15	postive
CCDC22	AC008764.6	0.315614669	5.00E-13	postive
CCDC22	AC008764.8	0.325296295	8.69E-14	postive

CCDC22	AC008771.1	0.339831271	5.56E-15	postive
CCDC22	AC008781.1	0.338596757	7.06E-15	postive
CCDC22	AC008870.3	0.320038105	2.27E-13	postive
CCDC22	AC008915.3	0.448679195	3.89E-26	postive
CCDC22	AC008946.1	0.395483325	3.62E-20	postive
CCDC22	AC008966.1	0.356046106	2.17E-16	postive
CCDC22	AC008982.2	0.326756178	6.63E-14	postive
CCDC22	AC009005.1	0.320103875	2.24E-13	postive
CCDC22	AC009041.3	0.349624814	8.02E-16	postive
CCDC22	AC009053.3	0.384834308	4.27E-19	postive
CCDC22	AC009061.2	0.452465804	1.33E-26	postive
CCDC22	AC009065.5	0.38173823	8.60E-19	postive
CCDC22	AC009065.9	0.376910295	2.53E-18	postive
CCDC22	AC009107.2	0.307868092	1.94E-12	postive
CCDC22	AC009113.1	0.433535092	2.50E-24	postive
CCDC22	AC009118.2	0.349568748	8.11E-16	postive
CCDC22	AC009118.3	0.306565003	2.43E-12	postive
CCDC22	AC009121.3	0.314712339	5.87E-13	postive
CCDC22	AC009121.4	0.332228042	2.38E-14	postive
CCDC22	AC009126.1	0.54099913	2.36E-39	postive
CCDC22	AC009159.3	0.319265786	2.60E-13	postive
CCDC22	AC009163.7	0.381101825	9.92E-19	postive
CCDC22	AC009171.2	0.365481514	3.02E-17	postive
CCDC22	AC009237.14	0.479274808	4.44E-30	postive
CCDC22	AC009309.2	0.484738212	7.95E-31	postive
CCDC22	AC009403.1	0.301932518	5.34E-12	postive
CCDC22	AC009404.1	0.357056903	1.76E-16	postive
CCDC22	AC009495.3	0.335814011	1.21E-14	postive
CCDC22	AC009506.2	0.437080221	9.61E-25	postive
CCDC22	AC009570.1	0.316210649	4.50E-13	postive
CCDC22	AC009630.2	0.413791691	4.20E-22	postive
CCDC22	AC009690.2	0.460029761	1.49E-27	postive
CCDC22	AC009779.3	0.524596799	1.06E-36	postive
CCDC22	AC009812.1	0.332191026	2.40E-14	postive
CCDC22	AC009831.1	0.53374473	3.66E-38	postive
CCDC22	AC009948.3	0.300046464	7.34E-12	postive
CCDC22	AC009961.1	0.310371248	1.26E-12	postive
CCDC22	AC010203.2	0.308768179	1.66E-12	postive
CCDC22	AC010226.1	0.359003054	1.18E-16	postive
CCDC22	AC010271.1	0.321608008	1.71E-13	postive
CCDC22	AC010273.2	0.344235714	2.35E-15	postive
CCDC22	AC010319.4	0.470761279	6.09E-29	postive
CCDC22	AC010319.5	0.368664183	1.53E-17	postive
CCDC22	AC010320.2	0.353194236	3.90E-16	postive
CCDC22	AC010359.2	0.338780102	6.81E-15	postive
CCDC22	AC010422.1	0.306370417	2.51E-12	postive
CCDC22	AC010422.2	0.360964852	7.83E-17	postive
CCDC22	AC010442.1	0.502302308	2.55E-33	postive
CCDC22	AC010463.3	0.361925918	6.40E-17	postive
CCDC22	AC010503.5	0.366485054	2.44E-17	postive

CCDC22	AC010504.1	0.448230317	4.41E-26	postive
CCDC22	AC010531.6	0.304514896	3.45E-12	postive
CCDC22	AC010618.3	0.366351711	2.51E-17	postive
CCDC22	AC010643.1	0.323842111	1.13E-13	postive
CCDC22	AC010655.4	0.39153147	9.14E-20	postive
CCDC22	AC010729.1	0.338122257	7.74E-15	postive
CCDC22	AC010761.1	0.331344578	2.82E-14	postive
CCDC22	AC010761.4	0.435180189	1.61E-24	postive
CCDC22	AC010761.6	0.347470643	1.24E-15	postive
CCDC22	AC010834.2	0.338837732	6.74E-15	postive
CCDC22	AC010864.1	0.352120861	4.85E-16	postive
CCDC22	AC010883.1	0.30943081	1.48E-12	postive
CCDC22	AC010894.2	0.441568191	2.82E-25	postive
CCDC22	AC010904.2	0.324001829	1.10E-13	postive
CCDC22	AC010907.2	0.301788835	5.48E-12	postive
CCDC22	AC010913.1	0.418641601	1.23E-22	postive
CCDC22	AC010969.2	0.412609395	5.65E-22	postive
CCDC22	AC011445.1	0.342080261	3.59E-15	postive
CCDC22	AC011450.1	0.437471898	8.64E-25	postive
CCDC22	AC011451.1	0.36047268	8.68E-17	postive
CCDC22	AC011472.1	0.387611323	2.26E-19	postive
CCDC22	AC011477.2	0.338113018	7.75E-15	postive
CCDC22	AC011498.1	0.313320673	7.51E-13	postive
CCDC22	AC011498.3	0.320323176	2.15E-13	postive
CCDC22	AC011498.6	0.318489751	2.99E-13	postive
CCDC22	AC011509.2	0.311493312	1.03E-12	postive
CCDC22	AC011511.1	0.365710736	2.87E-17	postive
CCDC22	AC011603.3	0.300731883	6.54E-12	postive
CCDC22	AC011731.1	0.315144566	5.44E-13	postive
CCDC22	AC011899.2	0.313266295	7.58E-13	postive
CCDC22	AC011921.1	0.381807103	8.46E-19	postive
CCDC22	AC012063.1	0.489027742	2.02E-31	postive
CCDC22	AC012073.1	0.371610319	8.08E-18	postive
CCDC22	AC012184.3	0.456278782	4.44E-27	postive
CCDC22	AC012306.2	0.505839158	7.70E-34	postive
CCDC22	AC012313.1	0.420220389	8.22E-23	postive
CCDC22	AC012313.2	0.320195649	2.20E-13	postive
CCDC22	AC012313.9	0.351084182	5.98E-16	postive
CCDC22	AC012360.2	0.315560472	5.05E-13	postive
CCDC22	AC012360.3	0.440288659	4.01E-25	postive
CCDC22	AC012363.3	0.334444443	1.57E-14	postive
CCDC22	AC012442.2	0.316176595	4.53E-13	postive
CCDC22	AC012451.1	0.34255541	3.27E-15	postive
CCDC22	AC012467.2	0.507048898	5.10E-34	postive
CCDC22	AC012510.1	0.331226471	2.88E-14	postive
CCDC22	AC012555.1	0.338849648	6.72E-15	postive
CCDC22	AC012603.1	0.30584136	2.75E-12	postive
CCDC22	AC012615.1	0.386538474	2.89E-19	postive
CCDC22	AC012615.6	0.339901689	5.48E-15	postive
CCDC22	AC012618.3	0.348763804	9.54E-16	postive

CCDC22	AC012640.4	0.346043402	1.64E-15	postive
CCDC22	AC012645.3	0.322847576	1.36E-13	postive
CCDC22	AC013565.1	0.302761823	4.65E-12	postive
CCDC22	AC015802.3	0.326011992	7.61E-14	postive
CCDC22	AC015802.4	0.369124635	1.38E-17	postive
CCDC22	AC015819.1	0.368114087	1.72E-17	postive
CCDC22	AC015909.2	0.306940252	2.28E-12	postive
CCDC22	AC015912.3	0.304158393	3.67E-12	postive
CCDC22	AC015917.2	0.333272628	1.96E-14	postive
CCDC22	AC015922.2	0.340420974	4.96E-15	postive
CCDC22	AC015982.2	0.376054712	3.05E-18	postive
CCDC22	AC016027.1	0.382554552	7.15E-19	postive
CCDC22	AC016065.1	0.481480349	2.23E-30	postive
CCDC22	AC016355.1	0.331222217	2.88E-14	postive
CCDC22	AC016394.2	0.41535028	2.84E-22	postive
CCDC22	AC016394.3	0.319982453	2.29E-13	postive
CCDC22	AC016405.3	0.362536143	5.63E-17	postive
CCDC22	AC016582.3	0.313319399	7.51E-13	postive
CCDC22	AC016590.3	0.348857637	9.36E-16	postive
CCDC22	AC016722.2	0.317491098	3.58E-13	postive
CCDC22	AC016727.1	0.391609139	8.97E-20	postive
CCDC22	AC016747.1	0.387276366	2.44E-19	postive
CCDC22	AC016876.2	0.477330021	8.13E-30	postive
CCDC22	AC016876.3	0.467723096	1.52E-28	postive
CCDC22	AC016888.1	0.430594117	5.48E-24	postive
CCDC22	AC017083.1	0.317147442	3.81E-13	postive
CCDC22	AC018521.5	0.359758072	1.01E-16	postive
CCDC22	AC018521.6	0.390722704	1.10E-19	postive
CCDC22	AC018647.2	0.406366317	2.65E-21	postive
CCDC22	AC018653.3	0.362435926	5.75E-17	postive
CCDC22	AC018682.2	0.356684883	1.90E-16	postive
CCDC22	AC018690.1	0.388660897	1.78E-19	postive
CCDC22	AC018695.4	0.306532369	2.44E-12	postive
CCDC22	AC018766.4	0.349718997	7.87E-16	postive
CCDC22	AC018809.2	0.354320007	3.10E-16	postive
CCDC22	AC019069.1	0.342640899	3.21E-15	postive
CCDC22	AC019077.1	0.331667976	2.65E-14	postive
CCDC22	AC019131.1	0.338081689	7.80E-15	postive
CCDC22	AC019131.2	0.312411506	8.81E-13	postive
CCDC22	AC019205.1	0.396115041	3.12E-20	postive
CCDC22	AC019294.2	0.398700068	1.69E-20	postive
CCDC22	AC020558.2	0.337916149	8.05E-15	postive
CCDC22	AC020663.2	0.341469983	4.04E-15	postive
CCDC22	AC020904.2	0.432313749	3.47E-24	postive
CCDC22	AC020907.2	0.369857801	1.18E-17	postive
CCDC22	AC020915.3	0.388309749	1.93E-19	postive
CCDC22	AC020934.2	0.333306991	1.94E-14	postive
CCDC22	AC021148.1	0.354380439	3.06E-16	postive
CCDC22	AC021613.1	0.360257136	9.07E-17	postive
CCDC22	AC021683.3	0.301822516	5.44E-12	postive

CCDC22	AC021755.2	0.327725762	5.54E-14	postive
CCDC22	AC022007.1	0.357858661	1.49E-16	postive
CCDC22	AC022098.1	0.465787982	2.72E-28	postive
CCDC22	AC022098.3	0.313087206	7.82E-13	postive
CCDC22	AC022150.2	0.42463943	2.62E-23	postive
CCDC22	AC022167.2	0.405868661	2.99E-21	postive
CCDC22	AC022211.3	0.530981964	1.02E-37	postive
CCDC22	AC022364.1	0.365351093	3.10E-17	postive
CCDC22	AC022382.1	0.302790715	4.62E-12	postive
CCDC22	AC022400.1	0.311280107	1.07E-12	postive
CCDC22	AC022415.2	0.367155057	2.11E-17	postive
CCDC22	AC022509.1	0.330151197	3.52E-14	postive
CCDC22	AC022509.2	0.330645607	3.21E-14	postive
CCDC22	AC022509.3	0.316828073	4.03E-13	postive
CCDC22	AC022509.4	0.362685354	5.45E-17	postive
CCDC22	AC022613.2	0.321694021	1.68E-13	postive
CCDC22	AC022916.2	0.508122711	3.53E-34	postive
CCDC22	AC023157.3	0.308006181	1.90E-12	postive
CCDC22	AC023509.3	0.354823231	2.79E-16	postive
CCDC22	AC023813.3	0.316157575	4.54E-13	postive
CCDC22	AC023983.2	0.380115953	1.24E-18	postive
CCDC22	AC024060.2	0.304582832	3.41E-12	postive
CCDC22	AC024075.2	0.337680525	8.43E-15	postive
CCDC22	AC024243.1	0.32104332	1.89E-13	postive
CCDC22	AC024267.7	0.3976513	2.17E-20	postive
CCDC22	AC024361.2	0.420147397	8.37E-23	postive
CCDC22	AC024575.2	0.497574535	1.24E-32	postive
CCDC22	AC024587.2	0.369726794	1.21E-17	postive
CCDC22	AC025048.2	0.447307184	5.72E-26	postive
CCDC22	AC025171.1	0.384784582	4.31E-19	postive
CCDC22	AC025181.2	0.364089025	4.05E-17	postive
CCDC22	AC025262.1	0.336517299	1.05E-14	postive
CCDC22	AC025265.1	0.307898807	1.93E-12	postive
CCDC22	AC025287.2	0.30419137	3.65E-12	postive
CCDC22	AC025682.2	0.401383133	8.87E-21	postive
CCDC22	AC025857.2	0.329490132	3.99E-14	postive
CCDC22	AC026254.2	0.300890275	6.37E-12	postive
CCDC22	AC026304.1	0.41170293	7.08E-22	postive
CCDC22	AC026367.2	0.300694808	6.58E-12	postive
CCDC22	AC026367.3	0.333121624	2.01E-14	postive
CCDC22	AC026401.3	0.393716592	5.48E-20	postive
CCDC22	AC026471.1	0.52653947	5.23E-37	postive
CCDC22	AC026471.4	0.379746764	1.34E-18	postive
CCDC22	AC026691.1	0.467411757	1.67E-28	postive
CCDC22	AC026801.2	0.305735817	2.80E-12	postive
CCDC22	AC027097.1	0.389654905	1.41E-19	postive
CCDC22	AC027097.2	0.422274483	4.84E-23	postive
CCDC22	AC027307.2	0.393077757	6.37E-20	postive
CCDC22	AC027307.3	0.302561352	4.81E-12	postive
CCDC22	AC027559.1	0.318905684	2.78E-13	postive

CCDC22	AC027601.2	0.432236923	3.54E-24	postive
CCDC22	AC027601.3	0.306993005	2.26E-12	postive
CCDC22	AC027644.3	0.385858017	3.38E-19	postive
CCDC22	AC027682.6	0.340657414	4.73E-15	postive
CCDC22	AC027702.2	0.317050007	3.88E-13	postive
CCDC22	AC027796.4	0.320864955	1.95E-13	postive
CCDC22	AC034102.6	0.406634892	2.48E-21	postive
CCDC22	AC034236.2	0.350638983	6.54E-16	postive
CCDC22	AC036176.1	0.402174356	7.33E-21	postive
CCDC22	AC037459.2	0.420720209	7.23E-23	postive
CCDC22	AC040162.3	0.375808479	3.22E-18	postive
CCDC22	AC040169.1	0.332014239	2.48E-14	postive
CCDC22	AC040970.1	0.385899578	3.35E-19	postive
CCDC22	AC046134.2	0.474822318	1.76E-29	postive
CCDC22	AC046143.2	0.332398036	2.31E-14	postive
CCDC22	AC048382.5	0.310122568	1.31E-12	postive
CCDC22	AC053503.1	0.482009606	1.88E-30	postive
CCDC22	AC053503.4	0.370307288	1.07E-17	postive
CCDC22	AC060766.7	0.349261964	8.63E-16	postive
CCDC22	AC060780.1	0.470171344	7.28E-29	postive
CCDC22	AC063943.2	0.329465899	4.01E-14	postive
CCDC22	AC064807.1	0.525500001	7.63E-37	postive
CCDC22	AC064836.3	0.388408477	1.88E-19	postive
CCDC22	AC067750.1	0.381488992	9.09E-19	postive
CCDC22	AC067852.2	0.686385189	6.77E-71	postive
CCDC22	AC067930.4	0.330609602	3.23E-14	postive
CCDC22	AC067960.1	0.354336709	3.09E-16	postive
CCDC22	AC068025.1	0.393830009	5.34E-20	postive
CCDC22	AC068338.2	0.427161122	1.36E-23	postive
CCDC22	AC068338.3	0.303168338	4.34E-12	postive
CCDC22	AC068473.5	0.412045085	6.50E-22	postive
CCDC22	AC068580.1	0.390001915	1.30E-19	postive
CCDC22	AC068580.3	0.439381194	5.14E-25	postive
CCDC22	AC068620.1	0.32993836	3.67E-14	postive
CCDC22	AC068790.9	0.373051244	5.90E-18	postive
CCDC22	AC068831.2	0.51434538	4.08E-35	postive
CCDC22	AC068831.3	0.383102397	6.32E-19	postive
CCDC22	AC068870.2	0.388546391	1.82E-19	postive
CCDC22	AC069185.1	0.370825795	9.58E-18	postive
CCDC22	AC069224.2	0.321058471	1.88E-13	postive
CCDC22	AC069285.2	0.360910608	7.92E-17	postive
CCDC22	AC069307.1	0.331268931	2.86E-14	postive
CCDC22	AC069544.1	0.337992451	7.93E-15	postive
CCDC22	AC072061.1	0.374652223	4.16E-18	postive
CCDC22	AC073046.1	0.369105101	1.39E-17	postive
CCDC22	AC073073.2	0.427173857	1.35E-23	postive
CCDC22	AC073195.2	0.454958208	6.50E-27	postive
CCDC22	AC073254.1	0.376436714	2.80E-18	postive
CCDC22	AC073389.1	0.328146295	5.13E-14	postive
CCDC22	AC073508.3	0.418250379	1.36E-22	postive

CCDC22	AC073517.1	0.365560063	2.97E-17	postive
CCDC22	AC073529.1	0.462389643	7.45E-28	postive
CCDC22	AC073569.2	0.426488106	1.62E-23	postive
CCDC22	AC073575.4	0.317966322	3.29E-13	postive
CCDC22	AC073611.1	0.333675492	1.81E-14	postive
CCDC22	AC073842.1	0.319342805	2.57E-13	postive
CCDC22	AC073842.2	0.373842318	4.96E-18	postive
CCDC22	AC073863.1	0.3266522	6.76E-14	postive
CCDC22	AC073869.1	0.537293705	9.64E-39	postive
CCDC22	AC073896.2	0.441334254	3.01E-25	postive
CCDC22	AC073896.3	0.400701141	1.04E-20	postive
CCDC22	AC073896.4	0.33528253	1.33E-14	postive
CCDC22	AC074011.1	0.383598631	5.65E-19	postive
CCDC22	AC074029.3	0.323100731	1.30E-13	postive
CCDC22	AC074117.1	0.499091472	7.47E-33	postive
CCDC22	AC078785.1	0.469694507	8.41E-29	postive
CCDC22	AC078860.2	0.302138508	5.16E-12	postive
CCDC22	AC079145.1	0.357163576	1.73E-16	postive
CCDC22	AC079174.2	0.358575122	1.29E-16	postive
CCDC22	AC079305.2	0.305817038	2.76E-12	postive
CCDC22	AC079414.3	0.352236555	4.73E-16	postive
CCDC22	AC079447.1	0.312164178	9.20E-13	postive
CCDC22	AC079807.1	0.551636078	3.73E-41	postive
CCDC22	AC079848.1	0.430475615	5.66E-24	postive
CCDC22	AC079921.2	0.322946133	1.34E-13	postive
CCDC22	AC079922.2	0.533162834	4.55E-38	postive
CCDC22	AC080013.5	0.381887591	8.31E-19	postive
CCDC22	AC080112.2	0.496162688	1.97E-32	postive
CCDC22	AC080129.2	0.311997863	9.47E-13	postive
CCDC22	AC083799.1	0.415246348	2.91E-22	postive
CCDC22	AC083862.2	0.392371108	7.51E-20	postive
CCDC22	AC083880.1	0.30925057	1.53E-12	postive
CCDC22	AC083964.1	0.301110758	6.14E-12	postive
CCDC22	AC084018.1	0.320948307	1.92E-13	postive
CCDC22	AC084018.2	0.337679761	8.43E-15	postive
CCDC22	AC084036.1	0.302415769	4.93E-12	postive
CCDC22	AC084125.2	0.349925912	7.55E-16	postive
CCDC22	AC084125.4	0.408361781	1.62E-21	postive
CCDC22	AC084809.1	0.312738836	8.32E-13	postive
CCDC22	AC087164.1	0.303130831	4.36E-12	postive
CCDC22	AC087289.1	0.407140755	2.19E-21	postive
CCDC22	AC087289.2	0.479706113	3.88E-30	postive
CCDC22	AC087289.5	0.369822038	1.19E-17	postive
CCDC22	AC087491.1	0.335209204	1.35E-14	postive
CCDC22	AC087500.1	0.391726388	8.73E-20	postive
CCDC22	AC087500.2	0.300007727	7.39E-12	postive
CCDC22	AC087501.4	0.348462327	1.01E-15	postive
CCDC22	AC087521.3	0.300218456	7.13E-12	postive
CCDC22	AC087645.2	0.382704934	6.91E-19	postive
CCDC22	AC087741.1	0.381989091	8.12E-19	postive

CCDC22	AC087741.2	0.312970604	7.99E-13	postive
CCDC22	AC090114.2	0.413088383	5.01E-22	postive
CCDC22	AC090116.1	0.322924581	1.34E-13	postive
CCDC22	AC090360.1	0.317669657	3.47E-13	postive
CCDC22	AC090425.2	0.304617992	3.39E-12	postive
CCDC22	AC090425.3	0.31075223	1.18E-12	postive
CCDC22	AC090515.3	0.331900773	2.54E-14	postive
CCDC22	AC090559.1	0.34307595	2.95E-15	postive
CCDC22	AC090587.1	0.480515192	3.01E-30	postive
CCDC22	AC090587.2	0.413591222	4.42E-22	postive
CCDC22	AC090589.3	0.315249898	5.34E-13	postive
CCDC22	AC090617.5	0.320871881	1.95E-13	postive
CCDC22	AC090825.1	0.423084581	3.93E-23	postive
CCDC22	AC090912.1	0.44123214	3.09E-25	postive
CCDC22	AC090970.2	0.386484505	2.93E-19	postive
CCDC22	AC091057.1	0.363940237	4.18E-17	postive
CCDC22	AC091153.3	0.35287031	4.16E-16	postive
CCDC22	AC091153.4	0.309705861	1.41E-12	postive
CCDC22	AC091271.1	0.438995152	5.71E-25	postive
CCDC22	AC091729.3	0.459418917	1.78E-27	postive
CCDC22	AC091965.1	0.338494043	7.20E-15	postive
CCDC22	AC091982.3	0.393263882	6.10E-20	postive
CCDC22	AC092070.2	0.315745428	4.89E-13	postive
CCDC22	AC092118.2	0.384779711	4.32E-19	postive
CCDC22	AC092119.3	0.305363881	2.99E-12	postive
CCDC22	AC092164.1	0.401787864	8.05E-21	postive
CCDC22	AC092295.2	0.454913058	6.58E-27	postive
CCDC22	AC092296.4	0.368358345	1.63E-17	postive
CCDC22	AC092301.1	0.341826749	3.77E-15	postive
CCDC22	AC092368.3	0.512744313	7.14E-35	postive
CCDC22	AC092431.1	0.362726012	5.41E-17	postive
CCDC22	AC092614.1	0.413408199	4.62E-22	postive
CCDC22	AC092634.3	0.313704901	7.02E-13	postive
CCDC22	AC092687.3	0.338921238	6.63E-15	postive
CCDC22	AC092718.2	0.3737662	5.05E-18	postive
CCDC22	AC092718.4	0.355320715	2.52E-16	postive
CCDC22	AC092720.1	0.322539287	1.44E-13	postive
CCDC22	AC092803.1	0.466143787	2.44E-28	postive
CCDC22	AC092910.3	0.376786356	2.60E-18	postive
CCDC22	AC092944.1	0.35760118	1.58E-16	postive
CCDC22	AC093157.1	0.334413807	1.58E-14	postive
CCDC22	AC093227.1	0.317450363	3.61E-13	postive
CCDC22	AC093249.6	0.449931276	2.73E-26	postive
CCDC22	AC093297.2	0.324008328	1.10E-13	postive
CCDC22	AC093520.1	0.34526683	1.91E-15	postive
CCDC22	AC093582.1	0.326849434	6.52E-14	postive
CCDC22	AC093627.5	0.367870954	1.81E-17	postive
CCDC22	AC093673.1	0.35530537	2.53E-16	postive
CCDC22	AC093726.1	0.356118282	2.14E-16	postive
CCDC22	AC093752.1	0.345624178	1.78E-15	postive

CCDC22	AC093752.3	0.431527589	4.28E-24	postive
CCDC22	AC096536.2	0.402224795	7.24E-21	postive
CCDC22	AC096540.1	0.314542205	6.05E-13	postive
CCDC22	AC096649.2	0.336621012	1.03E-14	postive
CCDC22	AC096677.1	0.336984232	9.63E-15	postive
CCDC22	AC096733.3	0.516009228	2.28E-35	postive
CCDC22	AC096772.6	0.344338127	2.30E-15	postive
CCDC22	AC096992.2	0.342096031	3.57E-15	postive
CCDC22	AC097358.2	0.369340303	1.32E-17	postive
CCDC22	AC097359.2	0.490723172	1.17E-31	postive
CCDC22	AC097468.3	0.355987518	2.20E-16	postive
CCDC22	AC097639.1	0.356310652	2.06E-16	postive
CCDC22	AC098484.1	0.397500676	2.24E-20	postive
CCDC22	AC098484.2	0.382984071	6.49E-19	postive
CCDC22	AC098487.1	0.313662564	7.07E-13	postive
CCDC22	AC098820.2	0.385495983	3.67E-19	postive
CCDC22	AC099343.4	0.481853909	1.98E-30	postive
CCDC22	AC099522.2	0.392258809	7.71E-20	postive
CCDC22	AC099568.2	0.400327589	1.14E-20	postive
CCDC22	AC099778.1	0.452081756	1.48E-26	postive
CCDC22	AC099811.3	0.384495507	4.61E-19	postive
CCDC22	AC099850.2	0.485129331	7.02E-31	postive
CCDC22	AC099850.4	0.384654589	4.44E-19	postive
CCDC22	AC100778.2	0.312795975	8.23E-13	postive
CCDC22	AC100786.1	0.347639539	1.19E-15	postive
CCDC22	AC100793.3	0.342692592	3.18E-15	postive
CCDC22	AC100803.2	0.367352562	2.02E-17	postive
CCDC22	AC100810.1	0.359755297	1.01E-16	postive
CCDC22	AC100812.1	0.328011836	5.26E-14	postive
CCDC22	AC100861.1	0.440276474	4.02E-25	postive
CCDC22	AC102953.1	0.30208079	5.21E-12	postive
CCDC22	AC103974.1	0.370018836	1.14E-17	postive
CCDC22	AC104109.2	0.436978045	9.88E-25	postive
CCDC22	AC104187.1	0.325817555	7.89E-14	postive
CCDC22	AC104521.1	0.386244005	3.09E-19	postive
CCDC22	AC104532.2	0.302649625	4.74E-12	postive
CCDC22	AC104561.1	0.342926105	3.04E-15	postive
CCDC22	AC104561.3	0.34773715	1.17E-15	postive
CCDC22	AC104564.3	0.414946693	3.14E-22	postive
CCDC22	AC104596.1	0.360291991	9.01E-17	postive
CCDC22	AC104653.1	0.322755664	1.38E-13	postive
CCDC22	AC104653.2	0.33549804	1.28E-14	postive
CCDC22	AC104667.1	0.300664664	6.62E-12	postive
CCDC22	AC104794.3	0.415520817	2.72E-22	postive
CCDC22	AC104819.3	0.307333532	2.13E-12	postive
CCDC22	AC104825.1	0.308725751	1.67E-12	postive
CCDC22	AC104971.1	0.32448964	1.01E-13	postive
CCDC22	AC105020.6	0.393676311	5.53E-20	postive
CCDC22	AC105036.3	0.356864096	1.84E-16	postive
CCDC22	AC105137.2	0.32881549	4.52E-14	postive

CCDC22	AC105206.2	0.340052434	5.33E-15	postive
CCDC22	AC105219.1	0.376145277	2.99E-18	postive
CCDC22	AC105345.1	0.341900736	3.71E-15	postive
CCDC22	AC105942.1	0.331828481	2.57E-14	postive
CCDC22	AC106739.1	0.375267656	3.63E-18	postive
CCDC22	AC106772.1	0.304523726	3.45E-12	postive
CCDC22	AC106779.1	0.324224711	1.06E-13	postive
CCDC22	AC106782.5	0.361968904	6.34E-17	postive
CCDC22	AC106786.2	0.332129545	2.43E-14	postive
CCDC22	AC106795.1	0.41803067	1.44E-22	postive
CCDC22	AC106820.5	0.320020274	2.27E-13	postive
CCDC22	AC106872.9	0.36611256	2.64E-17	postive
CCDC22	AC106886.2	0.309012693	1.59E-12	postive
CCDC22	AC106886.3	0.30236952	4.96E-12	postive
CCDC22	AC107027.3	0.361885747	6.45E-17	postive
CCDC22	AC107032.2	0.364263657	3.91E-17	postive
CCDC22	AC107068.1	0.332940627	2.08E-14	postive
CCDC22	AC107214.1	0.337670012	8.44E-15	postive
CCDC22	AC107294.2	0.304208862	3.63E-12	postive
CCDC22	AC107375.1	0.351417924	5.59E-16	postive
CCDC22	AC107884.1	0.351868104	5.10E-16	postive
CCDC22	AC107952.2	0.322466886	1.46E-13	postive
CCDC22	AC108010.1	0.308310867	1.80E-12	postive
CCDC22	AC108025.1	0.330664387	3.20E-14	postive
CCDC22	AC108047.1	0.305443961	2.94E-12	postive
CCDC22	AC108471.3	0.344841961	2.08E-15	postive
CCDC22	AC108488.1	0.528209592	2.84E-37	postive
CCDC22	AC108676.1	0.355378818	2.49E-16	postive
CCDC22	AC108860.2	0.33871698	6.90E-15	postive
CCDC22	AC109322.1	0.470622915	6.35E-29	postive
CCDC22	AC109347.1	0.501265073	3.61E-33	postive
CCDC22	AC109454.2	0.33361651	1.83E-14	postive
CCDC22	AC109460.1	0.502254335	2.59E-33	postive
CCDC22	AC109460.2	0.36697354	2.19E-17	postive
CCDC22	AC110079.1	0.413353216	4.69E-22	postive
CCDC22	AC112220.2	0.402284956	7.14E-21	postive
CCDC22	AC112236.2	0.300592314	6.70E-12	postive
CCDC22	AC112484.1	0.393598531	5.64E-20	postive
CCDC22	AC112907.3	0.312606467	8.51E-13	postive
CCDC22	AC113189.4	0.332347688	2.33E-14	postive
CCDC22	AC113608.2	0.309629921	1.43E-12	postive
CCDC22	AC114271.1	0.543328695	9.63E-40	postive
CCDC22	AC114490.1	0.336092003	1.14E-14	postive
CCDC22	AC114730.2	0.32370341	1.16E-13	postive
CCDC22	AC114956.2	0.379305856	1.48E-18	postive
CCDC22	AC115618.1	0.628719374	2.29E-56	postive
CCDC22	AC115618.2	0.575935366	1.62E-45	postive
CCDC22	AC116036.2	0.333915617	1.73E-14	postive
CCDC22	AC116913.1	0.417509396	1.64E-22	postive
CCDC22	AC116914.2	0.31388438	6.80E-13	postive

CCDC22	AC117394.2	0.313191411	7.68E-13	postive
CCDC22	AC118553.1	0.351342659	5.67E-16	postive
CCDC22	AC120114.1	0.359605818	1.04E-16	postive
CCDC22	AC121338.2	0.397472463	2.26E-20	postive
CCDC22	AC124016.2	0.411861857	6.80E-22	postive
CCDC22	AC124248.1	0.37611091	3.01E-18	postive
CCDC22	AC124283.2	0.353362129	3.76E-16	postive
CCDC22	AC124798.1	0.306240286	2.57E-12	postive
CCDC22	AC125257.1	0.441180471	3.14E-25	postive
CCDC22	AC125494.1	0.458622817	2.25E-27	postive
CCDC22	AC126283.1	0.325944288	7.71E-14	postive
CCDC22	AC126614.1	0.301839055	5.43E-12	postive
CCDC22	AC126755.1	0.349508661	8.21E-16	postive
CCDC22	AC127024.6	0.349001891	9.09E-16	postive
CCDC22	AC127537.1	0.30550209	2.92E-12	postive
CCDC22	AC129510.1	0.320955598	1.92E-13	postive
CCDC22	AC131009.3	0.302791567	4.62E-12	postive
CCDC22	AC131025.1	0.342641556	3.21E-15	postive
CCDC22	AC131097.3	0.357353994	1.66E-16	postive
CCDC22	AC131235.3	0.462630656	6.94E-28	postive
CCDC22	AC131235.4	0.348288609	1.05E-15	postive
CCDC22	AC132008.2	0.495214118	2.69E-32	postive
CCDC22	AC132192.2	0.333925146	1.73E-14	postive
CCDC22	AC132872.1	0.370442337	1.04E-17	postive
CCDC22	AC132872.3	0.410637608	9.23E-22	postive
CCDC22	AC132938.4	0.480712578	2.83E-30	postive
CCDC22	AC133528.2	0.461357907	1.01E-27	postive
CCDC22	AC133552.2	0.41984254	9.05E-23	postive
CCDC22	AC133552.5	0.352036333	4.93E-16	postive
CCDC22	AC134878.2	0.328335648	4.95E-14	postive
CCDC22	AC135048.1	0.335928796	1.18E-14	postive
CCDC22	AC135048.3	0.314144822	6.49E-13	postive
CCDC22	AC135050.1	0.502211487	2.63E-33	postive
CCDC22	AC135050.4	0.331611752	2.68E-14	postive
CCDC22	AC135050.5	0.378122062	1.93E-18	postive
CCDC22	AC135050.6	0.429371833	7.58E-24	postive
CCDC22	AC135178.4	0.357376921	1.65E-16	postive
CCDC22	AC135178.6	0.314444345	6.16E-13	postive
CCDC22	AC136431.1	0.339588119	5.83E-15	postive
CCDC22	AC136475.2	0.387205643	2.48E-19	postive
CCDC22	AC136604.3	0.300997018	6.26E-12	postive
CCDC22	AC137630.1	0.356012489	2.19E-16	postive
CCDC22	AC138028.4	0.317686522	3.46E-13	postive
CCDC22	AC138035.1	0.384733111	4.37E-19	postive
CCDC22	AC138123.1	0.311885084	9.66E-13	postive
CCDC22	AC138150.2	0.303231806	4.29E-12	postive
CCDC22	AC138207.2	0.309802623	1.39E-12	postive
CCDC22	AC138207.4	0.369103433	1.39E-17	postive
CCDC22	AC138207.8	0.400144927	1.19E-20	postive
CCDC22	AC138696.2	0.394888868	4.16E-20	postive

CCDC22	AC138761.1	0.335183547	1.36E-14	postive
CCDC22	AC138932.1	0.408784175	1.46E-21	postive
CCDC22	AC138956.2	0.333585414	1.84E-14	postive
CCDC22	AC138965.1	0.306356225	2.52E-12	postive
CCDC22	AC139149.1	0.319369822	2.56E-13	postive
CCDC22	AC139256.2	0.354761356	2.83E-16	postive
CCDC22	AC139256.3	0.427271655	1.32E-23	postive
CCDC22	AC139530.1	0.516142403	2.17E-35	postive
CCDC22	AC139768.1	0.431545051	4.26E-24	postive
CCDC22	AC139795.2	0.383618234	5.62E-19	postive
CCDC22	AC139887.2	0.305604633	2.86E-12	postive
CCDC22	AC141586.1	0.508949338	2.66E-34	postive
CCDC22	AC142472.1	0.424739655	2.56E-23	postive
CCDC22	AC144652.1	0.367706086	1.88E-17	postive
CCDC22	AC145098.1	0.408358299	1.62E-21	postive
CCDC22	AC145207.1	0.319746372	2.39E-13	postive
CCDC22	AC145207.5	0.330264534	3.45E-14	postive
CCDC22	AC145285.2	0.324064614	1.09E-13	postive
CCDC22	AC145285.6	0.406638563	2.48E-21	postive
CCDC22	AC145423.3	0.349935194	7.54E-16	postive
CCDC22	AC174065.1	0.335343755	1.32E-14	postive
CCDC22	AC209154.1	0.313098227	7.81E-13	postive
CCDC22	AC231533.2	0.409475406	1.23E-21	postive
CCDC22	AC231981.1	0.378465902	1.79E-18	postive
CCDC22	AC232271.1	0.488111181	2.71E-31	postive
CCDC22	AC233728.1	0.408264866	1.66E-21	postive
CCDC22	AC233992.3	0.42136499	6.12E-23	postive
CCDC22	AC241952.2	0.322824688	1.37E-13	postive
CCDC22	AC242376.2	0.331970306	2.50E-14	postive
CCDC22	AC243654.3	0.329193444	4.22E-14	postive
CCDC22	AC243773.1	0.326020686	7.60E-14	postive
CCDC22	AC244090.1	0.424638554	2.62E-23	postive
CCDC22	AC244669.1	0.367105396	2.13E-17	postive
CCDC22	AC245052.4	0.363557105	4.54E-17	postive
CCDC22	AC245060.2	0.322919866	1.34E-13	postive
CCDC22	AC245100.1	0.306245721	2.57E-12	postive
CCDC22	AC245140.2	0.477100905	8.73E-30	postive
CCDC22	AC245452.1	0.403266189	5.63E-21	postive
CCDC22	ACVR2B-AS1	0.453843974	8.95E-27	postive
CCDC22	AD000671.3	0.36573739	2.86E-17	postive
CCDC22	ADNP-AS1	0.355407137	2.48E-16	postive
CCDC22	AF001548.2	0.312557052	8.59E-13	postive
CCDC22	AF111167.2	0.333214232	1.98E-14	postive
CCDC22	AF129075.3	0.309085914	1.57E-12	postive
CCDC22	AF131216.4	0.377343821	2.29E-18	postive
CCDC22	AF196972.1	0.448674095	3.89E-26	postive
CCDC22	AF274858.1	0.336281404	1.10E-14	postive
CCDC22	AFDN-DT	0.436287282	1.19E-24	postive
CCDC22	AFG3L1P	0.452112681	1.47E-26	postive
CCDC22	AHSA2P	0.35896511	1.19E-16	postive

CCDC22	AL008718.2	0.357618917	1.57E-16	postive
CCDC22	AL008726.1	0.393264712	6.10E-20	postive
CCDC22	AL008729.1	0.371510467	8.25E-18	postive
CCDC22	AL009179.1	0.34674841	1.43E-15	postive
CCDC22	AL020996.1	0.490826416	1.13E-31	postive
CCDC22	AL021392.1	0.302980952	4.48E-12	postive
CCDC22	AL021707.3	0.384405282	4.70E-19	postive
CCDC22	AL021878.2	0.39037468	1.20E-19	postive
CCDC22	AL021937.1	0.308941639	1.61E-12	postive
CCDC22	AL022069.1	0.313489964	7.29E-13	postive
CCDC22	AL022157.1	0.48726563	3.55E-31	postive
CCDC22	AL022238.3	0.332668162	2.19E-14	postive
CCDC22	AL022311.1	0.330718962	3.17E-14	postive
CCDC22	AL022322.1	0.336845967	9.89E-15	postive
CCDC22	AL022326.1	0.39368718	5.52E-20	postive
CCDC22	AL022328.1	0.300823648	6.44E-12	postive
CCDC22	AL022328.2	0.357500844	1.61E-16	postive
CCDC22	AL022328.3	0.309345058	1.50E-12	postive
CCDC22	AL022328.4	0.336580146	1.04E-14	postive
CCDC22	AL023803.1	0.337018686	9.57E-15	postive
CCDC22	AL024498.1	0.423449066	3.57E-23	postive
CCDC22	AL024507.3	0.530391138	1.27E-37	postive
CCDC22	AL031055.1	0.40156216	8.50E-21	postive
CCDC22	AL031118.1	0.328331772	4.95E-14	postive
CCDC22	AL031123.1	0.371681959	7.95E-18	postive
CCDC22	AL031123.2	0.349684496	7.93E-16	postive
CCDC22	AL031186.1	0.423163136	3.85E-23	postive
CCDC22	AL031282.1	0.338318457	7.45E-15	postive
CCDC22	AL031282.2	0.329470387	4.00E-14	postive
CCDC22	AL031587.1	0.339139769	6.36E-15	postive
CCDC22	AL031600.1	0.444541189	1.24E-25	postive
CCDC22	AL031670.1	0.304024885	3.75E-12	postive
CCDC22	AL031709.1	0.337740423	8.33E-15	postive
CCDC22	AL031714.1	0.355551346	2.41E-16	postive
CCDC22	AL031775.1	0.330019449	3.61E-14	postive
CCDC22	AL031846.2	0.335460685	1.29E-14	postive
CCDC22	AL031963.2	0.303204274	4.31E-12	postive
CCDC22	AL031963.3	0.375363049	3.55E-18	postive
CCDC22	AL031985.3	0.348619025	9.82E-16	postive
CCDC22	AL032819.1	0.318799669	2.83E-13	postive
CCDC22	AL033527.3	0.318555864	2.96E-13	postive
CCDC22	AL034550.2	0.352408949	4.57E-16	postive
CCDC22	AL035458.2	0.378573526	1.75E-18	postive
CCDC22	AL035461.3	0.351999382	4.97E-16	postive
CCDC22	AL035587.1	0.46194071	8.50E-28	postive
CCDC22	AL049597.2	0.406714953	2.43E-21	postive
CCDC22	AL049794.1	0.368875854	1.46E-17	postive
CCDC22	AL049795.1	0.37221474	7.08E-18	postive
CCDC22	AL049796.1	0.376558419	2.73E-18	postive
CCDC22	AL049840.4	0.372923935	6.07E-18	postive

CCDC22	AL049840.5	0.37971333	1.35E-18	postive
CCDC22	AL049840.6	0.365894736	2.76E-17	postive
CCDC22	AL050309.1	0.501285575	3.59E-33	postive
CCDC22	AL050341.2	0.368409126	1.61E-17	postive
CCDC22	AL078581.2	0.365494861	3.01E-17	postive
CCDC22	AL078587.1	0.314135198	6.50E-13	postive
CCDC22	AL078590.2	0.319470169	2.51E-13	postive
CCDC22	AL078644.2	0.402308595	7.10E-21	postive
CCDC22	AL080276.2	0.330128367	3.54E-14	postive
CCDC22	AL096828.3	0.396874694	2.60E-20	postive
CCDC22	AL096870.1	0.312715486	8.35E-13	postive
CCDC22	AL109811.1	0.358525325	1.30E-16	postive
CCDC22	AL109917.1	0.436425961	1.15E-24	postive
CCDC22	AL109955.1	0.346396419	1.53E-15	postive
CCDC22	AL117327.1	0.349173497	8.79E-16	postive
CCDC22	AL117332.1	0.559786497	1.41E-42	postive
CCDC22	AL117336.2	0.332468397	2.28E-14	postive
CCDC22	AL118506.1	0.496999993	1.50E-32	postive
CCDC22	AL118511.1	0.407943315	1.80E-21	postive
CCDC22	AL118516.1	0.323452642	1.22E-13	postive
CCDC22	AL118558.1	0.302615532	4.76E-12	postive
CCDC22	AL118558.3	0.446035053	8.16E-26	postive
CCDC22	AL121574.1	0.358512847	1.30E-16	postive
CCDC22	AL121601.1	0.367727753	1.87E-17	postive
CCDC22	AL121603.2	0.41479706	3.26E-22	postive
CCDC22	AL121658.1	0.374107037	4.68E-18	postive
CCDC22	AL121820.2	0.316433612	4.33E-13	postive
CCDC22	AL121832.2	0.370384215	1.05E-17	postive
CCDC22	AL121890.4	0.385231034	3.90E-19	postive
CCDC22	AL121894.2	0.347297385	1.28E-15	postive
CCDC22	AL121906.1	0.322635769	1.41E-13	postive
CCDC22	AL121917.1	0.32007614	2.25E-13	postive
CCDC22	AL121944.2	0.560531746	1.04E-42	postive
CCDC22	AL121987.2	0.357946447	1.47E-16	postive
CCDC22	AL121992.1	0.329509289	3.97E-14	postive
CCDC22	AL122010.1	0.382402933	7.40E-19	postive
CCDC22	AL122035.1	0.325574182	8.25E-14	postive
CCDC22	AL122035.2	0.340070415	5.31E-15	postive
CCDC22	AL132639.3	0.380234053	1.21E-18	postive
CCDC22	AL132712.1	0.373581769	5.25E-18	postive
CCDC22	AL132780.1	0.362868326	5.25E-17	postive
CCDC22	AL132780.2	0.376705576	2.64E-18	postive
CCDC22	AL133215.2	0.38787591	2.13E-19	postive
CCDC22	AL133215.3	0.321264092	1.82E-13	postive
CCDC22	AL133216.2	0.414811431	3.25E-22	postive
CCDC22	AL133338.1	0.401645265	8.33E-21	postive
CCDC22	AL133367.1	0.34453012	2.21E-15	postive
CCDC22	AL133406.2	0.339220294	6.26E-15	postive
CCDC22	AL133520.1	0.382288441	7.59E-19	postive
CCDC22	AL135744.1	0.424758954	2.54E-23	postive

CCDC22	AL135791.1	0.413737236	4.26E-22	postive
CCDC22	AL135925.1	0.412468963	5.85E-22	postive
CCDC22	AL135999.1	0.348098081	1.09E-15	postive
CCDC22	AL135999.2	0.341701935	3.86E-15	postive
CCDC22	AL136040.1	0.361893504	6.44E-17	postive
CCDC22	AL136114.1	0.351741512	5.23E-16	postive
CCDC22	AL136162.1	0.359711982	1.02E-16	postive
CCDC22	AL136295.6	0.326087785	7.51E-14	postive
CCDC22	AL136295.7	0.382147988	7.84E-19	postive
CCDC22	AL136304.1	0.341290862	4.18E-15	postive
CCDC22	AL136307.1	0.331122734	2.94E-14	postive
CCDC22	AL136368.1	0.325061328	9.07E-14	postive
CCDC22	AL136531.1	0.329596646	3.91E-14	postive
CCDC22	AL137003.1	0.347774004	1.16E-15	postive
CCDC22	AL137058.2	0.356764561	1.87E-16	postive
CCDC22	AL137127.1	0.311493926	1.03E-12	postive
CCDC22	AL137186.2	0.395665535	3.47E-20	postive
CCDC22	AL137786.1	0.306486079	2.46E-12	postive
CCDC22	AL138724.2	0.330111577	3.55E-14	postive
CCDC22	AL138756.1	0.301251004	5.99E-12	postive
CCDC22	AL138762.2	0.354213036	3.16E-16	postive
CCDC22	AL138831.2	0.315342826	5.25E-13	postive
CCDC22	AL138921.1	0.342869756	3.07E-15	postive
CCDC22	AL138921.2	0.313409983	7.39E-13	postive
CCDC22	AL138995.1	0.302590655	4.78E-12	postive
CCDC22	AL139011.1	0.416238764	2.27E-22	postive
CCDC22	AL139035.1	0.465666949	2.82E-28	postive
CCDC22	AL139246.5	0.334541887	1.54E-14	postive
CCDC22	AL139260.1	0.438304355	6.89E-25	postive
CCDC22	AL139274.2	0.309737672	1.41E-12	postive
CCDC22	AL139287.1	0.430976071	4.95E-24	postive
CCDC22	AL139383.1	0.388109826	2.02E-19	postive
CCDC22	AL139384.1	0.362241991	5.99E-17	postive
CCDC22	AL139397.1	0.35327995	3.83E-16	postive
CCDC22	AL157832.1	0.334839497	1.45E-14	postive
CCDC22	AL158212.3	0.31572817	4.90E-13	postive
CCDC22	AL158835.2	0.321573508	1.72E-13	postive
CCDC22	AL160006.1	0.436173659	1.23E-24	postive
CCDC22	AL160313.2	0.378846828	1.64E-18	postive
CCDC22	AL161452.1	0.362899978	5.21E-17	postive
CCDC22	AL161668.3	0.32890831	4.45E-14	postive
CCDC22	AL161719.1	0.382224876	7.70E-19	postive
CCDC22	AL161729.1	0.327808592	5.46E-14	postive
CCDC22	AL161756.1	0.319264357	2.61E-13	postive
CCDC22	AL161772.1	0.32361347	1.18E-13	postive
CCDC22	AL161891.1	0.323124088	1.29E-13	postive
CCDC22	AL162231.2	0.354394023	3.05E-16	postive
CCDC22	AL162258.2	0.356034492	2.18E-16	postive
CCDC22	AL162274.2	0.316202469	4.51E-13	postive
CCDC22	AL162377.1	0.479673113	3.92E-30	postive

CCDC22	AL162595.1	0.362643342	5.50E-17	postive
CCDC22	AL162741.1	0.371695867	7.93E-18	postive
CCDC22	AL163051.1	0.314025308	6.63E-13	postive
CCDC22	AL353194.1	0.424254593	2.90E-23	postive
CCDC22	AL353593.1	0.346916685	1.38E-15	postive
CCDC22	AL353596.1	0.366106125	2.64E-17	postive
CCDC22	AL353625.1	0.330211906	3.48E-14	postive
CCDC22	AL353708.1	0.391151698	9.98E-20	postive
CCDC22	AL353708.3	0.315795415	4.85E-13	postive
CCDC22	AL353719.1	0.321671282	1.69E-13	postive
CCDC22	AL353743.1	0.358608896	1.28E-16	postive
CCDC22	AL353796.1	0.323797705	1.14E-13	postive
CCDC22	AL353807.2	0.311512721	1.03E-12	postive
CCDC22	AL354696.2	0.348385725	1.03E-15	postive
CCDC22	AL354740.1	0.457363848	3.24E-27	postive
CCDC22	AL354836.1	0.316949296	3.95E-13	postive
CCDC22	AL354892.2	0.503070029	1.97E-33	postive
CCDC22	AL354892.3	0.349138726	8.85E-16	postive
CCDC22	AL354920.1	0.417066677	1.84E-22	postive
CCDC22	AL354928.1	0.387058714	2.57E-19	postive
CCDC22	AL355001.2	0.466943752	1.92E-28	postive
CCDC22	AL355075.2	0.311019441	1.12E-12	postive
CCDC22	AL355353.2	0.380144596	1.23E-18	postive
CCDC22	AL355385.1	0.358938404	1.19E-16	postive
CCDC22	AL355574.1	0.401264991	9.13E-21	postive
CCDC22	AL355802.3	0.348499891	1.01E-15	postive
CCDC22	AL356019.2	0.320639528	2.03E-13	postive
CCDC22	AL356056.2	0.331323715	2.83E-14	postive
CCDC22	AL356235.1	0.3311206	2.94E-14	postive
CCDC22	AL356277.2	0.337063867	9.49E-15	postive
CCDC22	AL356299.3	0.364068573	4.07E-17	postive
CCDC22	AL356481.1	0.406136745	2.80E-21	postive
CCDC22	AL356481.3	0.340117978	5.26E-15	postive
CCDC22	AL356512.1	0.327060624	6.27E-14	postive
CCDC22	AL356599.1	0.399926322	1.26E-20	postive
CCDC22	AL357033.3	0.320602566	2.05E-13	postive
CCDC22	AL357033.4	0.308144375	1.85E-12	postive
CCDC22	AL357079.1	0.312722709	8.34E-13	postive
CCDC22	AL357140.2	0.383368402	5.95E-19	postive
CCDC22	AL357507.1	0.34233382	3.41E-15	postive
CCDC22	AL357673.1	0.391987017	8.22E-20	postive
CCDC22	AL357874.1	0.382082895	7.95E-19	postive
CCDC22	AL358334.2	0.314243666	6.38E-13	postive
CCDC22	AL358472.2	0.448602367	3.97E-26	postive
CCDC22	AL358472.3	0.367199818	2.09E-17	postive
CCDC22	AL358473.1	0.338150839	7.70E-15	postive
CCDC22	AL358613.2	0.306914232	2.29E-12	postive
CCDC22	AL358852.1	0.408977428	1.39E-21	postive
CCDC22	AL358933.1	0.325111336	8.99E-14	postive
CCDC22	AL359091.3	0.330405125	3.36E-14	postive

CCDC22	AL359198.1	0.358394393	1.34E-16	postive
CCDC22	AL359397.1	0.31191628	9.61E-13	postive
CCDC22	AL359513.1	0.351674947	5.30E-16	postive
CCDC22	AL359532.1	0.325686512	8.08E-14	postive
CCDC22	AL359541.2	0.313682673	7.04E-13	postive
CCDC22	AL359643.3	0.406188551	2.76E-21	postive
CCDC22	AL359711.2	0.413708708	4.29E-22	postive
CCDC22	AL359715.3	0.395281991	3.79E-20	postive
CCDC22	AL359921.1	0.307655036	2.01E-12	postive
CCDC22	AL359921.2	0.353474651	3.68E-16	postive
CCDC22	AL359962.1	0.319120029	2.67E-13	postive
CCDC22	AL360181.2	0.392039034	8.12E-20	postive
CCDC22	AL360270.3	0.313412774	7.39E-13	postive
CCDC22	AL365330.1	0.350541848	6.67E-16	postive
CCDC22	AL390719.1	0.318257362	3.12E-13	postive
CCDC22	AL390728.4	0.340095614	5.28E-15	postive
CCDC22	AL390879.1	0.34881175	9.45E-16	postive
CCDC22	AL390955.2	0.383024924	6.43E-19	postive
CCDC22	AL391069.2	0.385206362	3.92E-19	postive
CCDC22	AL391069.3	0.3294889	3.99E-14	postive
CCDC22	AL391121.1	0.325704825	8.06E-14	postive
CCDC22	AL391244.2	0.357657274	1.56E-16	postive
CCDC22	AL391422.4	0.359295235	1.11E-16	postive
CCDC22	AL391832.2	0.302234048	5.08E-12	postive
CCDC22	AL391832.3	0.384610264	4.49E-19	postive
CCDC22	AL391834.2	0.315505383	5.10E-13	postive
CCDC22	AL441992.2	0.441790056	2.65E-25	postive
CCDC22	AL442663.3	0.42048162	7.68E-23	postive
CCDC22	AL445228.3	0.309701445	1.41E-12	postive
CCDC22	AL445231.1	0.351105957	5.95E-16	postive
CCDC22	AL450326.1	0.436391012	1.16E-24	postive
CCDC22	AL450998.2	0.505318693	9.20E-34	postive
CCDC22	AL450998.3	0.317603447	3.51E-13	postive
CCDC22	AL451050.2	0.345799934	1.72E-15	postive
CCDC22	AL451074.2	0.328144984	5.13E-14	postive
CCDC22	AL451085.2	0.363662356	4.44E-17	postive
CCDC22	AL451085.3	0.450815237	2.12E-26	postive
CCDC22	AL451165.2	0.433875619	2.28E-24	postive
CCDC22	AL512408.1	0.367262002	2.06E-17	postive
CCDC22	AL512625.1	0.301494829	5.75E-12	postive
CCDC22	AL512625.2	0.369308124	1.33E-17	postive
CCDC22	AL512770.1	0.347601774	1.20E-15	postive
CCDC22	AL512791.2	0.310930298	1.14E-12	postive
CCDC22	AL513165.1	0.380219096	1.21E-18	postive
CCDC22	AL513320.1	0.31636578	4.38E-13	postive
CCDC22	AL513550.1	0.446828287	6.54E-26	postive
CCDC22	AL589743.1	0.317709961	3.44E-13	postive
CCDC22	AL589765.4	0.415090914	3.03E-22	postive
CCDC22	AL590133.1	0.381075278	9.98E-19	postive
CCDC22	AL590369.1	0.368886009	1.46E-17	postive

CCDC22	AL590617.2	0.373971585	4.82E-18	postive
CCDC22	AL591684.2	0.351662142	5.32E-16	postive
CCDC22	AL592071.1	0.303064353	4.41E-12	postive
CCDC22	AL592166.1	0.44412476	1.39E-25	postive
CCDC22	AL592435.1	0.363095553	5.00E-17	postive
CCDC22	AL592494.2	0.366207007	2.59E-17	postive
CCDC22	AL596094.1	0.379044007	1.57E-18	postive
CCDC22	AL596202.1	0.322897494	1.35E-13	postive
CCDC22	AL603832.1	0.343912645	2.50E-15	postive
CCDC22	AL603839.2	0.450166925	2.55E-26	postive
CCDC22	AL603839.3	0.33965276	5.75E-15	postive
CCDC22	AL604028.1	0.398576272	1.74E-20	postive
CCDC22	AL606489.1	0.307301861	2.14E-12	postive
CCDC22	AL606491.1	0.380243715	1.20E-18	postive
CCDC22	AL606760.1	0.375776797	3.24E-18	postive
CCDC22	AL606760.2	0.348092332	1.09E-15	postive
CCDC22	AL606834.1	0.376591502	2.71E-18	postive
CCDC22	AL645568.1	0.413300385	4.75E-22	postive
CCDC22	AL645728.1	0.381228489	9.64E-19	postive
CCDC22	AL645940.1	0.342132064	3.55E-15	postive
CCDC22	AL662791.1	0.371633293	8.04E-18	postive
CCDC22	AL662797.2	0.416209783	2.28E-22	postive
CCDC22	AL662844.4	0.366177215	2.60E-17	postive
CCDC22	AL669831.1	0.358020286	1.45E-16	postive
CCDC22	AL671710.1	0.425925893	1.88E-23	postive
CCDC22	AL683813.2	0.308085422	1.87E-12	postive
CCDC22	AL691432.1	0.331839494	2.57E-14	postive
CCDC22	AL691432.2	0.359963934	9.65E-17	postive
CCDC22	AL713998.1	0.364486842	3.73E-17	postive
CCDC22	AL731569.1	0.347912494	1.13E-15	postive
CCDC22	AL731571.1	0.386152628	3.16E-19	postive
CCDC22	AL731577.2	0.45473686	6.92E-27	postive
CCDC22	AL732314.8	0.525955636	6.47E-37	postive
CCDC22	AL732372.2	0.403212839	5.70E-21	postive
CCDC22	AL928654.1	0.416015349	2.40E-22	postive
CCDC22	AL928654.2	0.373730974	5.09E-18	postive
CCDC22	ALG1L13P	0.422762554	4.27E-23	postive
CCDC22	ALOX12-AS1	0.474551479	1.92E-29	postive
CCDC22	AMZ2P1	0.520971028	3.91E-36	postive
CCDC22	ANO7L1	0.455112098	6.21E-27	postive
CCDC22	AOC4P	0.32595262	7.70E-14	postive
CCDC22	AP000251.1	0.338729111	6.88E-15	postive
CCDC22	AP000253.1	0.356415602	2.01E-16	postive
CCDC22	AP000254.2	0.361006987	7.76E-17	postive
CCDC22	AP000295.10	0.37615208	2.99E-18	postive
CCDC22	AP000320.1	0.307092796	2.22E-12	postive
CCDC22	AP000347.1	0.317798946	3.39E-13	postive
CCDC22	AP000350.5	0.309199123	1.54E-12	postive
CCDC22	AP000442.1	0.503015524	2.01E-33	postive
CCDC22	AP000487.1	0.380621839	1.10E-18	postive

CCDC22	AP000525.1	0.366488497	2.43E-17	postive
CCDC22	AP000640.1	0.367273233	2.06E-17	postive
CCDC22	AP000692.1	0.332965298	2.07E-14	postive
CCDC22	AP000704.2	0.323915996	1.12E-13	postive
CCDC22	AP000759.1	0.439924573	4.43E-25	postive
CCDC22	AP000769.1	0.360942475	7.86E-17	postive
CCDC22	AP000781.1	0.422880236	4.14E-23	postive
CCDC22	AP000808.1	0.356830603	1.85E-16	postive
CCDC22	AP000880.1	0.311563068	1.02E-12	postive
CCDC22	AP000894.4	0.361107915	7.60E-17	postive
CCDC22	AP000944.1	0.378798601	1.66E-18	postive
CCDC22	AP001033.3	0.41824365	1.36E-22	postive
CCDC22	AP001062.1	0.32726891	6.03E-14	postive
CCDC22	AP001107.1	0.47007704	7.49E-29	postive
CCDC22	AP001107.4	0.323260297	1.26E-13	postive
CCDC22	AP001160.1	0.39547954	3.62E-20	postive
CCDC22	AP001160.2	0.374900061	3.94E-18	postive
CCDC22	AP001160.3	0.48828254	2.56E-31	postive
CCDC22	AP001198.2	0.323181464	1.28E-13	postive
CCDC22	AP001267.1	0.315431681	5.17E-13	postive
CCDC22	AP001267.3	0.371165161	8.90E-18	postive
CCDC22	AP001318.2	0.384803488	4.30E-19	postive
CCDC22	AP001351.1	0.300699023	6.58E-12	postive
CCDC22	AP001360.1	0.311271992	1.08E-12	postive
CCDC22	AP001372.2	0.43024401	6.02E-24	postive
CCDC22	AP001412.1	0.366869876	2.24E-17	postive
CCDC22	AP001453.2	0.417555943	1.62E-22	postive
CCDC22	AP001453.4	0.433219416	2.72E-24	postive
CCDC22	AP001462.1	0.453542695	9.75E-27	postive
CCDC22	AP001469.2	0.30697732	2.26E-12	postive
CCDC22	AP001469.3	0.34214106	3.54E-15	postive
CCDC22	AP001505.1	0.419353844	1.03E-22	postive
CCDC22	AP001527.2	0.363582377	4.51E-17	postive
CCDC22	AP001816.1	0.389739268	1.38E-19	postive
CCDC22	AP001830.1	0.333492116	1.88E-14	postive
CCDC22	AP001922.5	0.355264093	2.55E-16	postive
CCDC22	AP001922.6	0.30563454	2.85E-12	postive
CCDC22	AP002360.2	0.377236706	2.35E-18	postive
CCDC22	AP002387.2	0.474620956	1.87E-29	postive
CCDC22	AP002748.4	0.427701068	1.18E-23	postive
CCDC22	AP002754.2	0.361120781	7.58E-17	postive
CCDC22	AP002761.3	0.347279394	1.28E-15	postive
CCDC22	AP002807.1	0.356216908	2.10E-16	postive
CCDC22	AP002840.2	0.34299128	3.00E-15	postive
CCDC22	AP003068.1	0.380332986	1.18E-18	postive
CCDC22	AP003068.2	0.343639625	2.64E-15	postive
CCDC22	AP003096.1	0.341292942	4.18E-15	postive
CCDC22	AP003100.1	0.326438039	7.04E-14	postive
CCDC22	AP003392.4	0.31203838	9.41E-13	postive
CCDC22	AP003392.5	0.374211667	4.58E-18	postive

CCDC22	AP003469.2	0.327225685	6.08E-14	postive
CCDC22	AP003469.3	0.310012443	1.34E-12	postive
CCDC22	AP003486.1	0.398217938	1.89E-20	postive
CCDC22	AP003498.1	0.427375808	1.28E-23	postive
CCDC22	AP003680.1	0.37699395	2.48E-18	postive
CCDC22	AP003721.4	0.417950036	1.47E-22	postive
CCDC22	AP004609.5	0.408148281	1.71E-21	postive
CCDC22	AP005061.2	0.304252249	3.61E-12	postive
CCDC22	AP005262.2	0.304337814	3.56E-12	postive
CCDC22	AP005329.1	0.312246759	9.07E-13	postive
CCDC22	AP005329.2	0.327977271	5.29E-14	postive
CCDC22	AP005432.2	0.369737492	1.21E-17	postive
CCDC22	AP005482.4	0.34607008	1.63E-15	postive
CCDC22	AP005717.1	0.301281131	5.96E-12	postive
CCDC22	AP006216.1	0.318439358	3.02E-13	postive
CCDC22	AP006222.1	0.332565032	2.24E-14	postive
CCDC22	AP006621.2	0.34789408	1.14E-15	postive
CCDC22	AP006621.3	0.518353152	9.94E-36	postive
CCDC22	AP006623.1	0.318731239	2.87E-13	postive
CCDC22	APTR	0.304145768	3.67E-12	postive
CCDC22	ARHGAP31-AS1	0.325064017	9.07E-14	postive
CCDC22	ARHGAP5-AS1	0.340235982	5.14E-15	postive
CCDC22	ARHGEF35-AS1	0.300049708	7.33E-12	postive
CCDC22	ARMCX5-GPRASP2	0.483026385	1.37E-30	postive
CCDC22	ARRDC1-AS1	0.56057176	1.02E-42	postive
CCDC22	ASB16-AS1	0.580312569	2.42E-46	postive
CCDC22	ASH1L-AS1	0.376856579	2.56E-18	postive
CCDC22	ATF4P4	0.35538071	2.49E-16	postive
CCDC22	ATP13A5-AS1	0.320183537	2.21E-13	postive
CCDC22	ATP2A1-AS1	0.41720025	1.78E-22	postive
CCDC22	ATP2B1-AS1	0.315612522	5.01E-13	postive
CCDC22	ATP6V0E2-AS1	0.351128245	5.92E-16	postive
CCDC22	ATXN1-AS1	0.43629377	1.19E-24	postive
CCDC22	ATXN2-AS	0.397506104	2.24E-20	postive
CCDC22	BAALC-AS1	0.346205175	1.59E-15	postive
CCDC22	BACE1-AS	0.390070752	1.28E-19	postive
CCDC22	BAIAP2-DT	0.358605493	1.28E-16	postive
CCDC22	BCDIN3D-AS1	0.443544802	1.63E-25	postive
CCDC22	BDNF-AS	0.385337509	3.80E-19	postive
CCDC22	BHLHE40-AS1	0.33356913	1.85E-14	postive
CCDC22	BMS1P20	0.462763323	6.67E-28	postive
CCDC22	BOLA2P2	0.372383824	6.83E-18	postive
CCDC22	BOLA3-AS1	0.34310394	2.93E-15	postive
CCDC22	BRWD1-AS2	0.328377769	4.91E-14	postive
CCDC22	BTG3-AS1	0.396601201	2.78E-20	postive
CCDC22	BTN2A3P	0.49727794	1.36E-32	postive
CCDC22	BX322234.1	0.347583291	1.21E-15	postive
CCDC22	BX322557.10	0.390378961	1.19E-19	postive
CCDC22	BX537318.1	0.408516783	1.56E-21	postive
CCDC22	BX649601.1	0.312572902	8.56E-13	postive

CCDC22	C10orf25	0.419504358	9.87E-23	postive
CCDC22	C15orf56	0.350216621	7.12E-16	postive
CCDC22	C1orf147	0.328670437	4.65E-14	postive
CCDC22	C1orf229	0.420883468	6.93E-23	postive
CCDC22	C1RL-AS1	0.319140537	2.66E-13	postive
CCDC22	C22orf24	0.424384084	2.80E-23	postive
CCDC22	C22orf46	0.49041509	1.29E-31	postive
CCDC22	C5orf66	0.366403583	2.48E-17	postive
CCDC22	C6orf99	0.4213376	6.17E-23	postive
CCDC22	C9orf163	0.347593905	1.21E-15	postive
CCDC22	CA5BP1	0.581311321	1.56E-46	postive
CCDC22	CACNA1C-AS2	0.344432101	2.26E-15	postive
CCDC22	CACTIN-AS1	0.396506901	2.84E-20	postive
CCDC22	CAHM	0.365380603	3.08E-17	postive
CCDC22	CAMTA1-DT	0.37727783	2.33E-18	postive
CCDC22	CAPN10-DT	0.384444413	4.66E-19	postive
CCDC22	CASC11	0.364654877	3.60E-17	postive
CCDC22	CASK-AS1	0.328651988	4.66E-14	postive
CCDC22	CATSPER2P1	0.413924108	4.06E-22	postive
CCDC22	CBR3-AS1	0.329379477	4.07E-14	postive
CCDC22	CCDC183-AS1	0.346931331	1.38E-15	postive
CCDC22	CCDC84-DT	0.389274162	1.54E-19	postive
CCDC22	CCNT2-AS1	0.46698052	1.90E-28	postive
CCDC22	CCT6P1	0.4170831	1.83E-22	postive
CCDC22	CCT6P3	0.340063652	5.31E-15	postive
CCDC22	CD27-AS1	0.492186572	7.25E-32	postive
CCDC22	CD2BP2-DT	0.326024964	7.59E-14	postive
CCDC22	CD99P1	0.343078052	2.95E-15	postive
CCDC22	CDC37L1-DT	0.427279573	1.32E-23	postive
CCDC22	CEBPB-AS1	0.527072414	4.30E-37	postive
CCDC22	CENPBD1P1	0.513510741	5.47E-35	postive
CCDC22	CEP83-DT	0.443267708	1.76E-25	postive
CCDC22	CFL1P1	0.321783517	1.65E-13	postive
CCDC22	CHKB-CPT1B	0.313472749	7.31E-13	postive
CCDC22	CHKB-DT	0.363682614	4.42E-17	postive
CCDC22	CHROMR	0.351131361	5.92E-16	postive
CCDC22	CIDECP1	0.531521993	8.37E-38	postive
CCDC22	CIRBP-AS1	0.317105957	3.84E-13	postive
CCDC22	CKMT2-AS1	0.430655639	5.39E-24	postive
CCDC22	CLSTN2-AS1	0.306013603	2.67E-12	postive
CCDC22	CLUHP3	0.310017768	1.34E-12	postive
CCDC22	CNNM3-DT	0.406160264	2.78E-21	postive
CCDC22	COX10-AS1	0.423024289	3.99E-23	postive
CCDC22	CR381653.1	0.401342245	8.96E-21	postive
CCDC22	CROCCP2	0.415156298	2.98E-22	postive
CCDC22	CRTC3-AS1	0.445774498	8.78E-26	postive
CCDC22	CRYBB2P1	0.512060472	9.06E-35	postive
CCDC22	CRYGEP	0.367015998	2.17E-17	postive
CCDC22	CRYZL2P	0.406190549	2.76E-21	postive
CCDC22	CSTF3-DT	0.317144781	3.81E-13	postive

CCDC22	CTA-989H11.1	0.386321257	3.04E-19	postive
CCDC22	CTBP1-AS	0.302807662	4.61E-12	postive
CCDC22	CTBP1-DT	0.548338205	1.37E-40	postive
CCDC22	CTC-205M6.5	0.325492323	8.38E-14	postive
CCDC22	CTC-487M23.5	0.321174545	1.84E-13	postive
CCDC22	CTD-2132N18.4	0.35218508	4.78E-16	postive
CCDC22	CTD-2134A5.3	0.367553013	1.94E-17	postive
CCDC22	CTD-2245F17.3	0.318500912	2.99E-13	postive
CCDC22	CTD-2521M24.9	0.495614351	2.36E-32	postive
CCDC22	CTD-2540M10.1	0.359845914	9.89E-17	postive
CCDC22	CTD-2554C21.3	0.315010489	5.57E-13	postive
CCDC22	CTD-2574D22.4	0.395898057	3.28E-20	postive
CCDC22	CUTALP	0.376223578	2.94E-18	postive
CCDC22	CYP4F23P	0.309250084	1.53E-12	postive
CCDC22	CYP51A1-AS1	0.378584971	1.74E-18	postive
CCDC22	DANCR	0.367253814	2.07E-17	postive
CCDC22	DBIL5P	0.431159547	4.72E-24	postive
CCDC22	DCST1-AS1	0.413355612	4.68E-22	postive
CCDC22	DCXR-DT	0.400393698	1.12E-20	postive
CCDC22	DDX11-AS1	0.364112568	4.03E-17	postive
CCDC22	DDX39B-AS1	0.342575449	3.25E-15	postive
CCDC22	DEPDC1-AS1	0.336231704	1.11E-14	postive
CCDC22	DGCR11	0.388445278	1.87E-19	postive
CCDC22	DGUOK-AS1	0.312311334	8.97E-13	postive
CCDC22	DHRS4-AS1	0.475148213	1.59E-29	postive
CCDC22	DICER1-AS1	0.362216466	6.02E-17	postive
CCDC22	DLG5-AS1	0.333554353	1.86E-14	postive
CCDC22	DM1-AS	0.43648166	1.13E-24	postive
CCDC22	DNAJC27-AS1	0.398716675	1.68E-20	postive
CCDC22	DNAJC3-DT	0.420736114	7.20E-23	postive
CCDC22	DNM1P35	0.327344307	5.95E-14	postive
CCDC22	DNM1P46	0.306216337	2.58E-12	postive
CCDC22	DNMBP-AS1	0.307477467	2.08E-12	postive
CCDC22	DOCK9-DT	0.363149752	4.94E-17	postive
CCDC22	DPP9-AS1	0.393915048	5.23E-20	postive
CCDC22	DPY19L2P3	0.316099514	4.59E-13	postive
CCDC22	DSTNP2	0.366137288	2.62E-17	postive
CCDC22	DUXAP8	0.361719432	6.68E-17	postive
CCDC22	DUXAP9	0.39917843	1.50E-20	postive
CCDC22	DYRK3-AS1	0.419273799	1.05E-22	postive
CCDC22	EAF1-AS1	0.336671977	1.02E-14	postive
CCDC22	EBLN3P	0.444184173	1.37E-25	postive
CCDC22	EDRF1-DT	0.365169482	3.22E-17	postive
CCDC22	EIF1B-AS1	0.412516413	5.78E-22	postive
CCDC22	EIF2AK3-DT	0.518973598	7.97E-36	postive
CCDC22	EIF3J-DT	0.474239375	2.11E-29	postive
CCDC22	EIPR1-IT1	0.314124829	6.52E-13	postive
CCDC22	ELF3-AS1	0.429328524	7.67E-24	postive
CCDC22	ELOA-AS1	0.483585226	1.15E-30	postive
CCDC22	EMC1-AS1	0.468566976	1.18E-28	postive

CCDC22	EMC3-AS1	0.408673407	1.50E-21	postive
CCDC22	EMX2OS	0.315743223	4.89E-13	postive
CCDC22	ENTPD3-AS1	0.343391555	2.77E-15	postive
CCDC22	EP400P1	0.410731614	9.02E-22	postive
CCDC22	EPB41L4A-DT	0.465082893	3.35E-28	postive
CCDC22	EPCAM-DT	0.350487192	6.74E-16	postive
CCDC22	ERICD	0.349618395	8.03E-16	postive
CCDC22	ETV7-AS1	0.326887286	6.48E-14	postive
CCDC22	EXOC3-AS1	0.423774392	3.28E-23	postive
CCDC22	EXOSC10-AS1	0.376191453	2.96E-18	postive
CCDC22	FAHD2CP	0.407304928	2.10E-21	postive
CCDC22	FALEC	0.327820224	5.45E-14	postive
CCDC22	FAM111A-DT	0.551134712	4.56E-41	postive
CCDC22	FAM157C	0.313118349	7.78E-13	postive
CCDC22	FAM160A1-DT	0.347689529	1.18E-15	postive
CCDC22	FAM201A	0.315137304	5.45E-13	postive
CCDC22	FAM21EP	0.410521558	9.50E-22	postive
CCDC22	FAM21FP	0.479788876	3.78E-30	postive
CCDC22	FAM3D-AS1	0.34613239	1.61E-15	postive
CCDC22	FAM66B	0.318675706	2.90E-13	postive
CCDC22	FAM66C	0.333839781	1.76E-14	postive
CCDC22	FAM66D	0.369057429	1.40E-17	postive
CCDC22	FAM83C-AS1	0.311936423	9.57E-13	postive
CCDC22	FAM86B3P	0.440208251	4.10E-25	postive
CCDC22	FAM86C2P	0.58789139	8.40E-48	postive
CCDC22	FAM86DP	0.43911459	5.53E-25	postive
CCDC22	FAM86EP	0.485996028	5.33E-31	postive
CCDC22	FAM86FP	0.326922644	6.43E-14	postive
CCDC22	FAM86HP	0.420518408	7.61E-23	postive
CCDC22	FAM86JP	0.50870367	2.89E-34	postive
CCDC22	FGD5-AS1	0.505780003	7.86E-34	postive
CCDC22	FGF7P3	0.305272614	3.03E-12	postive
CCDC22	FLVCR1-DT	0.34365087	2.63E-15	postive
CCDC22	FMR1-AS1	0.303157673	4.34E-12	postive
CCDC22	FMR1-IT1	0.306295795	2.55E-12	postive
CCDC22	FO393418.1	0.397432249	2.28E-20	postive
CCDC22	FOXD2-AS1	0.369621211	1.24E-17	postive
CCDC22	FOXD3-AS1	0.34464357	2.17E-15	postive
CCDC22	FRG1-DT	0.334607488	1.52E-14	postive
CCDC22	FRG1HP	0.451578031	1.71E-26	postive
CCDC22	GAS5-AS1	0.319545476	2.48E-13	postive
CCDC22	GATA2-AS1	0.342372889	3.39E-15	postive
CCDC22	GBAP1	0.486096608	5.16E-31	postive
CCDC22	GCC2-AS1	0.532326557	6.21E-38	postive
CCDC22	GCSIR	0.360296593	9.00E-17	postive
CCDC22	GEMIN7-AS1	0.403797919	4.95E-21	postive
CCDC22	GHET1	0.357145405	1.73E-16	postive
CCDC22	GIHCG	0.38472833	4.37E-19	postive
CCDC22	GLIDR	0.415072868	3.04E-22	postive
CCDC22	GLUD1P3	0.322959446	1.33E-13	postive

CCDC22	GMDS-DT	0.327369879	5.92E-14	postive
CCDC22	GNRHR2	0.528802633	2.28E-37	postive
CCDC22	GPRC5D-AS1	0.30204374	5.25E-12	postive
CCDC22	GS1-124K5.3	0.370207159	1.09E-17	postive
CCDC22	GS1-358P8.4	0.449433405	3.14E-26	postive
CCDC22	GTF2IP1	0.490119679	1.42E-31	postive
CCDC22	GTF2IP13	0.306300362	2.54E-12	postive
CCDC22	GTF2IP20	0.321259863	1.82E-13	postive
CCDC22	GTF2IP4	0.454575891	7.25E-27	postive
CCDC22	GTF3C2-AS1	0.306015925	2.67E-12	postive
CCDC22	GTSE1-DT	0.431688434	4.10E-24	postive
CCDC22	GUSBP1	0.371638494	8.03E-18	postive
CCDC22	GUSBP11	0.365765	2.84E-17	postive
CCDC22	GUSBP2	0.30229728	5.03E-12	postive
CCDC22	H2AZ2P1	0.428641229	9.19E-24	postive
CCDC22	H2BC19P	0.301502387	5.75E-12	postive
CCDC22	HCG11	0.31164427	1.01E-12	postive
CCDC22	HCG15	0.377997161	1.98E-18	postive
CCDC22	HCG18	0.447957529	4.76E-26	postive
CCDC22	HCG25	0.487610982	3.18E-31	postive
CCDC22	HCG27	0.318644528	2.91E-13	postive
CCDC22	HCP5	0.350627871	6.56E-16	postive
CCDC22	HDAC11-AS1	0.380936188	1.03E-18	postive
CCDC22	HDHD5-AS1	0.331047176	2.98E-14	postive
CCDC22	HEIH	0.511909662	9.55E-35	postive
CCDC22	HERC2P2	0.300341151	6.98E-12	postive
CCDC22	HERC2P4	0.420258112	8.14E-23	postive
CCDC22	HERC2P9	0.331815392	2.58E-14	postive
CCDC22	HM13-IT1	0.478186164	6.23E-30	postive
CCDC22	HMGB1P5	0.414266947	3.73E-22	postive
CCDC22	HOXA-AS3	0.302251175	5.06E-12	postive
CCDC22	HOXB-AS1	0.318206068	3.15E-13	postive
CCDC22	HSD17B7P2	0.380226239	1.21E-18	postive
CCDC22	HYI-AS1	0.332938925	2.08E-14	postive
CCDC22	IDH1-AS1	0.405830431	3.02E-21	postive
CCDC22	IL10RB-DT	0.402574181	6.65E-21	postive
CCDC22	IL6R-AS1	0.318351472	3.07E-13	postive
CCDC22	ILF3-DT	0.54688832	2.42E-40	postive
CCDC22	INE1	0.47329835	2.81E-29	postive
CCDC22	INTS6-AS1	0.372534692	6.60E-18	postive
CCDC22	IPO9-AS1	0.480987637	2.60E-30	postive
CCDC22	IQCH-AS1	0.418533983	1.26E-22	postive
CCDC22	ITFG2-AS1	0.328841921	4.50E-14	postive
CCDC22	ITGA9-AS1	0.30877661	1.66E-12	postive
CCDC22	JPX	0.405024615	3.67E-21	postive
CCDC22	KB-1572G7.2	0.310784661	1.17E-12	postive
CCDC22	KCNIP2-AS1	0.351257954	5.77E-16	postive
CCDC22	KCNMB2-AS1	0.307782045	1.97E-12	postive
CCDC22	KCNQ1DN	0.300928524	6.33E-12	postive
CCDC22	KCTD21-AS1	0.319595163	2.45E-13	postive

CCDC22	KDM2B-DT	0.440087041	4.23E-25	postive
CCDC22	KDM4A-AS1	0.384734326	4.36E-19	postive
CCDC22	KDM7A-DT	0.384917701	4.19E-19	postive
CCDC22	KIF1C-AS1	0.339939511	5.44E-15	postive
CCDC22	KIF9-AS1	0.344756849	2.12E-15	postive
CCDC22	KMT2E-AS1	0.416658635	2.04E-22	postive
CCDC22	KRT8P12	0.527408979	3.81E-37	postive
CCDC22	KTN1-AS1	0.4821204	1.82E-30	postive
CCDC22	L3MBTL2-AS1	0.398498801	1.77E-20	postive
CCDC22	LAMTOR5-AS1	0.327384439	5.91E-14	postive
CCDC22	LBX2-AS1	0.475162174	1.59E-29	postive
CCDC22	LCMT1-AS1	0.315071805	5.51E-13	postive
CCDC22	LENG8-AS1	0.330583114	3.25E-14	postive
CCDC22	LGALS8-AS1	0.446441696	7.29E-26	postive
CCDC22	LHX1-DT	0.33508923	1.39E-14	postive
CCDC22	LIFR-AS1	0.356853955	1.84E-16	postive
CCDC22	LINC00106	0.324906247	9.33E-14	postive
CCDC22	LINC00115	0.391407833	9.40E-20	postive
CCDC22	LINC00173	0.356014293	2.19E-16	postive
CCDC22	LINC00174	0.47628873	1.12E-29	postive
CCDC22	LINC00205	0.373869251	4.93E-18	postive
CCDC22	LINC00235	0.35282	4.20E-16	postive
CCDC22	LINC00265	0.339238864	6.24E-15	postive
CCDC22	LINC00294	0.400759716	1.03E-20	postive
CCDC22	LINC00303	0.325130821	8.96E-14	postive
CCDC22	LINC00339	0.527636299	3.50E-37	postive
CCDC22	LINC00346	0.376461292	2.79E-18	postive
CCDC22	LINC00449	0.437458598	8.68E-25	postive
CCDC22	LINC00467	0.360352306	8.90E-17	postive
CCDC22	LINC00471	0.455506959	5.55E-27	postive
CCDC22	LINC00476	0.378822031	1.65E-18	postive
CCDC22	LINC00488	0.326089855	7.50E-14	postive
CCDC22	LINC00511	0.423040609	3.97E-23	postive
CCDC22	LINC00526	0.358890843	1.21E-16	postive
CCDC22	LINC00620	0.340499572	4.88E-15	postive
CCDC22	LINC00621	0.307863982	1.94E-12	postive
CCDC22	LINC00630	0.436452788	1.14E-24	postive
CCDC22	LINC00637	0.373711925	5.11E-18	postive
CCDC22	LINC00638	0.344877417	2.07E-15	postive
CCDC22	LINC00652	0.464589639	3.88E-28	postive
CCDC22	LINC00653	0.329980152	3.64E-14	postive
CCDC22	LINC00654	0.425673847	2.00E-23	postive
CCDC22	LINC00663	0.384901198	4.20E-19	postive
CCDC22	LINC00665	0.372277483	6.99E-18	postive
CCDC22	LINC00667	0.396860424	2.61E-20	postive
CCDC22	LINC00680	0.358427469	1.33E-16	postive
CCDC22	LINC00839	0.301132113	6.12E-12	postive
CCDC22	LINC00847	0.501261005	3.62E-33	postive
CCDC22	LINC00863	0.390530647	1.15E-19	postive
CCDC22	LINC00881	0.342156126	3.53E-15	postive

CCDC22	LINC00884	0.42059499	7.46E-23	postive
CCDC22	LINC00887	0.325553081	8.29E-14	postive
CCDC22	LINC00888	0.425803538	1.94E-23	postive
CCDC22	LINC00899	0.336919442	9.76E-15	postive
CCDC22	LINC00909	0.502294743	2.56E-33	postive
CCDC22	LINC00910	0.440986243	3.31E-25	postive
CCDC22	LINC00921	0.433057663	2.84E-24	postive
CCDC22	LINC00957	0.46636244	2.29E-28	postive
CCDC22	LINC00963	0.472951329	3.13E-29	postive
CCDC22	LINC01001	0.359568417	1.05E-16	postive
CCDC22	LINC01003	0.390019703	1.30E-19	postive
CCDC22	LINC01006	0.353587238	3.60E-16	postive
CCDC22	LINC01011	0.455019554	6.38E-27	postive
CCDC22	LINC01023	0.424056689	3.05E-23	postive
CCDC22	LINC01063	0.390578312	1.14E-19	postive
CCDC22	LINC01089	0.313506551	7.27E-13	postive
CCDC22	LINC01093	0.305492844	2.92E-12	postive
CCDC22	LINC01127	0.381652073	8.77E-19	postive
CCDC22	LINC01128	0.464124502	4.46E-28	postive
CCDC22	LINC01132	0.343338023	2.80E-15	postive
CCDC22	LINC01135	0.321196581	1.84E-13	postive
CCDC22	LINC01144	0.410105026	1.05E-21	postive
CCDC22	LINC01145	0.375858903	3.19E-18	postive
CCDC22	LINC01184	0.50808336	3.58E-34	postive
CCDC22	LINC01232	0.383650317	5.58E-19	postive
CCDC22	LINC01248	0.32796963	5.30E-14	postive
CCDC22	LINC01250	0.342724521	3.16E-15	postive
CCDC22	LINC01270	0.363246332	4.84E-17	postive
CCDC22	LINC01271	0.391940719	8.30E-20	postive
CCDC22	LINC01273	0.468113811	1.35E-28	postive
CCDC22	LINC01275	0.343935501	2.49E-15	postive
CCDC22	LINC01278	0.573245359	5.13E-45	postive
CCDC22	LINC01285	0.403373416	5.48E-21	postive
CCDC22	LINC01291	0.358934918	1.20E-16	postive
CCDC22	LINC01301	0.382939265	6.56E-19	postive
CCDC22	LINC01311	0.336172431	1.13E-14	postive
CCDC22	LINC01353	0.311258623	1.08E-12	postive
CCDC22	LINC01372	0.348111317	1.09E-15	postive
CCDC22	LINC01410	0.330419291	3.35E-14	postive
CCDC22	LINC01431	0.360394134	8.82E-17	postive
CCDC22	LINC01506	0.303851283	3.86E-12	postive
CCDC22	LINC01521	0.438355785	6.80E-25	postive
CCDC22	LINC01534	0.336896802	9.80E-15	postive
CCDC22	LINC01560	0.60900869	4.46E-52	postive
CCDC22	LINC01569	0.389859954	1.35E-19	postive
CCDC22	LINC01578	0.314081666	6.57E-13	postive
CCDC22	LINC01585	0.327374285	5.92E-14	postive
CCDC22	LINC01588	0.326932659	6.42E-14	postive
CCDC22	LINC01635	0.30463043	3.38E-12	postive
CCDC22	LINC01637	0.307701028	2.00E-12	postive

CCDC22	LINC01678	0.328151033	5.12E-14	postive
CCDC22	LINC01686	0.376058466	3.05E-18	postive
CCDC22	LINC01687	0.31455373	6.04E-13	postive
CCDC22	LINC01703	0.456724332	3.90E-27	postive
CCDC22	LINC01715	0.375301754	3.60E-18	postive
CCDC22	LINC01741	0.324209859	1.06E-13	postive
CCDC22	LINC01754	0.364846295	3.45E-17	postive
CCDC22	LINC01772	0.310802133	1.17E-12	postive
CCDC22	LINC01775	0.379008278	1.58E-18	postive
CCDC22	LINC01799	0.354046598	3.27E-16	postive
CCDC22	LINC01806	0.323944041	1.11E-13	postive
CCDC22	LINC01812	0.350726005	6.43E-16	postive
CCDC22	LINC01816	0.320693223	2.01E-13	postive
CCDC22	LINC01829	0.340910158	4.51E-15	postive
CCDC22	LINC01852	0.31264804	8.45E-13	postive
CCDC22	LINC01881	0.448366809	4.24E-26	postive
CCDC22	LINC01968	0.306296136	2.54E-12	postive
CCDC22	LINC01971	0.403040504	5.94E-21	postive
CCDC22	LINC01972	0.315585046	5.03E-13	postive
CCDC22	LINC01976	0.326675712	6.73E-14	postive
CCDC22	LINC01997	0.34356618	2.68E-15	postive
CCDC22	LINC02019	0.345768954	1.73E-15	postive
CCDC22	LINC02020	0.323188185	1.28E-13	postive
CCDC22	LINC02021	0.372490037	6.67E-18	postive
CCDC22	LINC02035	0.377918569	2.02E-18	postive
CCDC22	LINC02044	0.348854007	9.37E-16	postive
CCDC22	LINC02062	0.30798804	1.90E-12	postive
CCDC22	LINC02068	0.416356497	2.20E-22	postive
CCDC22	LINC02076	0.316853141	4.01E-13	postive
CCDC22	LINC02099	0.361352878	7.22E-17	postive
CCDC22	LINC02193	0.387120714	2.53E-19	postive
CCDC22	LINC02210	0.5087253	2.87E-34	postive
CCDC22	LINC02321	0.326572439	6.86E-14	postive
CCDC22	LINC02340	0.319192633	2.64E-13	postive
CCDC22	LINC02361	0.393003509	6.48E-20	postive
CCDC22	LINC02449	0.382365183	7.46E-19	postive
CCDC22	LINC02453	0.30363687	4.01E-12	postive
CCDC22	LINC02473	0.322622789	1.42E-13	postive
CCDC22	LINC02545	0.337506857	8.71E-15	postive
CCDC22	LINC02548	0.341338197	4.15E-15	postive
CCDC22	LINC02572	0.361046033	7.69E-17	postive
CCDC22	LINC02574	0.319811254	2.36E-13	postive
CCDC22	LINC02586	0.318978832	2.74E-13	postive
CCDC22	LINC02594	0.37139624	8.46E-18	postive
CCDC22	LINC02601	0.415287351	2.88E-22	postive
CCDC22	LINC02604	0.401008574	9.71E-21	postive
CCDC22	LINC02609	0.303128728	4.37E-12	postive
CCDC22	LINC02611	0.424138076	2.99E-23	postive
CCDC22	LINC02613	0.421605199	5.76E-23	postive
CCDC22	LINC02637	0.427374085	1.28E-23	postive

CCDC22	LINC02683	0.368500607	1.58E-17	postive
CCDC22	LINC02693	0.485019075	7.28E-31	postive
CCDC22	LINC02709	0.386179198	3.14E-19	postive
CCDC22	LINC02724	0.36036327	8.88E-17	postive
CCDC22	LINC02763	0.329553548	3.94E-14	postive
CCDC22	LINC02766	0.308804677	1.65E-12	postive
CCDC22	LINC02773	0.339237174	6.24E-15	postive
CCDC22	LIPE-AS1	0.339235152	6.24E-15	postive
CCDC22	LIX1L-AS1	0.332729576	2.17E-14	postive
CCDC22	LLPH-DT	0.339523273	5.90E-15	postive
CCDC22	LMCD1-AS1	0.301807504	5.46E-12	postive
CCDC22	LNCTAM34A	0.333159073	2.00E-14	postive
CCDC22	LOH12CR2	0.452609101	1.27E-26	postive
CCDC22	LOXL1-AS1	0.351488232	5.51E-16	postive
CCDC22	LPP-AS2	0.437759774	7.99E-25	postive
CCDC22	LRRC37A17P	0.415759863	2.56E-22	postive
CCDC22	LRRC37A6P	0.319258631	2.61E-13	postive
CCDC22	LRRC37BP1	0.510439906	1.59E-34	postive
CCDC22	LSP1P4	0.348215541	1.06E-15	postive
CCDC22	LY6E-DT	0.303859853	3.86E-12	postive
CCDC22	MAFG-DT	0.408759912	1.47E-21	postive
CCDC22	MAN1B1-DT	0.37283343	6.19E-18	postive
CCDC22	MAP2K4P1	0.371726404	7.88E-18	postive
CCDC22	MAP3K14-AS1	0.404337443	4.34E-21	postive
CCDC22	MAP3K2-DT	0.462117376	8.07E-28	postive
CCDC22	MAP4K3-DT	0.512538656	7.67E-35	postive
CCDC22	MAPK6-DT	0.383338798	5.99E-19	postive
CCDC22	MAPKAPK5-AS1	0.503772876	1.55E-33	postive
CCDC22	MATN1-AS1	0.406095999	2.83E-21	postive
CCDC22	MCM3AP-AS1	0.38781464	2.16E-19	postive
CCDC22	MCPH1-AS1	0.349834913	7.69E-16	postive
CCDC22	MDC1-AS1	0.312608651	8.51E-13	postive
CCDC22	MED14OS	0.478253412	6.10E-30	postive
CCDC22	MED8-AS1	0.33861777	7.03E-15	postive
CCDC22	MELTF-AS1	0.435418389	1.51E-24	postive
CCDC22	MHENCR	0.3296358	3.88E-14	postive
CCDC22	MID1IP1-AS1	0.415752499	2.56E-22	postive
CCDC22	MIF-AS1	0.396451278	2.88E-20	postive
CCDC22	MINCR	0.361504349	6.99E-17	postive
CCDC22	MIPEPP3	0.454573487	7.26E-27	postive
CCDC22	MIR210HG	0.311726118	9.93E-13	postive
CCDC22	MIR22HG	0.308082636	1.87E-12	postive
CCDC22	MIR34AHG	0.346724396	1.43E-15	postive
CCDC22	MIR4453HG	0.380012699	1.27E-18	postive
CCDC22	MIR762HG	0.508087975	3.57E-34	postive
CCDC22	MIR924HG	0.304527258	3.44E-12	postive
CCDC22	MKLN1-AS	0.333387583	1.91E-14	postive
CCDC22	MLIP-IT1	0.302273335	5.05E-12	postive
CCDC22	MMADHC-DT	0.32785953	5.41E-14	postive
CCDC22	MMP25-AS1	0.347075658	1.34E-15	postive

CCDC22	MORF4L2-AS1	0.33924671	6.23E-15	postive
CCDC22	MRPL20-AS1	0.568707971	3.50E-44	postive
CCDC22	MRPL45P2	0.346593592	1.47E-15	postive
CCDC22	MRPS31P4	0.43778771	7.93E-25	postive
CCDC22	MSH5-SAPCD1	0.391588535	9.01E-20	postive
CCDC22	MSL3P1	0.374369635	4.42E-18	postive
CCDC22	MTMR9LP	0.391012551	1.03E-19	postive
CCDC22	MUC20-OT1	0.411830329	6.86E-22	postive
CCDC22	MYLK-AS1	0.434438129	1.96E-24	postive
CCDC22	MZF1-AS1	0.307975454	1.91E-12	postive
CCDC22	NACA4P	0.41158479	7.29E-22	postive
CCDC22	NAPA-AS1	0.349699653	7.90E-16	postive
CCDC22	NBPF25P	0.422875929	4.15E-23	postive
CCDC22	NBPF8	0.42083899	7.01E-23	postive
CCDC22	NBR2	0.461785727	8.90E-28	postive
CCDC22	NCBP2-AS1	0.358692179	1.26E-16	postive
CCDC22	NCK1-DT	0.357624937	1.57E-16	postive
CCDC22	NDUFA6-DT	0.365141981	3.24E-17	postive
CCDC22	NDUFB2-AS1	0.447355458	5.64E-26	postive
CCDC22	NEBL-AS1	0.371642902	8.02E-18	postive
CCDC22	NIFK-AS1	0.601306336	1.76E-50	postive
CCDC22	NINJ2-AS1	0.391242269	9.77E-20	postive
CCDC22	NKAPP1	0.38157755	8.91E-19	postive
CCDC22	NNT-AS1	0.399049302	1.55E-20	postive
CCDC22	NOP14-AS1	0.479712446	3.87E-30	postive
CCDC22	NOP53-AS1	0.348433251	1.02E-15	postive
CCDC22	NORAD	0.4309508	4.99E-24	postive
CCDC22	NPEPPSP1	0.413677143	4.32E-22	postive
CCDC22	NPIPP1	0.355801878	2.28E-16	postive
CCDC22	NRAV	0.46833365	1.27E-28	postive
CCDC22	NRSN2-AS1	0.42205769	5.12E-23	postive
CCDC22	NSUN5P1	0.302099468	5.20E-12	postive
CCDC22	NSUN5P2	0.331408038	2.78E-14	postive
CCDC22	NUDT16P1	0.470819169	5.98E-29	postive
CCDC22	NUP50-DT	0.377256837	2.34E-18	postive
CCDC22	NUTM2A-AS1	0.423809329	3.26E-23	postive
CCDC22	NXT1-AS1	0.302612166	4.77E-12	postive
CCDC22	OGFR-AS1	0.430440261	5.71E-24	postive
CCDC22	OGFRP1	0.413998225	3.99E-22	postive
CCDC22	OIP5-AS1	0.3535655	3.61E-16	postive
CCDC22	OLMALINC	0.473315803	2.80E-29	postive
CCDC22	OR7E14P	0.402287817	7.13E-21	postive
CCDC22	OR7E161P	0.306494698	2.46E-12	postive
CCDC22	OSER1-DT	0.46741709	1.67E-28	postive
CCDC22	OSGEPL1-AS1	0.353400383	3.74E-16	postive
CCDC22	OTUD6B-AS1	0.39717047	2.43E-20	postive
CCDC22	OXCT1-AS1	0.319418586	2.53E-13	postive
CCDC22	PARD6G-AS1	0.373914886	4.88E-18	postive
CCDC22	PARGP1	0.306858345	2.31E-12	postive
CCDC22	PAXBP1-AS1	0.359638044	1.03E-16	postive

CCDC22	PAXIP1-AS1	0.411043415	8.34E-22	postive
CCDC22	PAXIP1-AS2	0.434368478	2.00E-24	postive
CCDC22	PCAT6	0.349939783	7.53E-16	postive
CCDC22	PCBP1-AS1	0.502471969	2.41E-33	postive
CCDC22	PCCA-DT	0.316264183	4.46E-13	postive
CCDC22	PDCD4-AS1	0.420811949	7.06E-23	postive
CCDC22	PDIA3P1	0.394279551	4.80E-20	postive
CCDC22	PDPK2P	0.347443755	1.24E-15	postive
CCDC22	PGAM1P8	0.318273075	3.11E-13	postive
CCDC22	PHKA2-AS1	0.309865365	1.37E-12	postive
CCDC22	PI4KAP2	0.415196517	2.95E-22	postive
CCDC22	PINK1-AS	0.473023692	3.06E-29	postive
CCDC22	PIPSL	0.37142103	8.42E-18	postive
CCDC22	PITPNA-AS1	0.462314724	7.62E-28	postive
CCDC22	PITRM1-AS1	0.3243764	1.03E-13	postive
CCDC22	PKD1P6	0.501626719	3.20E-33	postive
CCDC22	PLEKHA8P1	0.371687637	7.94E-18	postive
CCDC22	PLEKHM1P1	0.315985427	4.69E-13	postive
CCDC22	PMS2CL	0.503448309	1.73E-33	postive
CCDC22	PMS2P3	0.399162846	1.51E-20	postive
CCDC22	PMS2P4	0.499965953	5.58E-33	postive
CCDC22	POLH-AS1	0.334592133	1.52E-14	postive
CCDC22	POLR2J4	0.506099705	7.05E-34	postive
CCDC22	PPP1R26-AS1	0.350794885	6.34E-16	postive
CCDC22	PPP3CB-AS1	0.303508044	4.09E-12	postive
CCDC22	PPT2-EGFL8	0.521660527	3.05E-36	postive
CCDC22	PRC1-AS1	0.389524733	1.46E-19	postive
CCDC22	PRELID1P1	0.388294034	1.93E-19	postive
CCDC22	PRKAR2A-AS1	0.442283195	2.31E-25	postive
CCDC22	PRMT5-AS1	0.383434187	5.86E-19	postive
CCDC22	PRORSD1P	0.407535162	1.99E-21	postive
CCDC22	PRR34	0.301290509	5.95E-12	postive
CCDC22	PSLNR	0.319573377	2.46E-13	postive
CCDC22	PSMA3-AS1	0.349882009	7.62E-16	postive
CCDC22	PSMB8-AS1	0.388133043	2.01E-19	postive
CCDC22	PTENP1	0.30988673	1.37E-12	postive
CCDC22	PTMAP5	0.322434152	1.47E-13	postive
CCDC22	PTOV1-AS1	0.418044251	1.43E-22	postive
CCDC22	PTOV1-AS2	0.352593323	4.40E-16	postive
CCDC22	PXN-AS1	0.518957819	8.02E-36	postive
CCDC22	RAB11B-AS1	0.356516429	1.97E-16	postive
CCDC22	RAB30-DT	0.398487646	1.77E-20	postive
CCDC22	RAB4B-EGLN2	0.300652865	6.63E-12	postive
CCDC22	RABGAP1L-DT	0.439794304	4.59E-25	postive
CCDC22	RAET1K	0.335459879	1.29E-14	postive
CCDC22	RALY-AS1	0.436514695	1.12E-24	postive
CCDC22	RAP2C-AS1	0.392456335	7.36E-20	postive
CCDC22	RARA-AS1	0.44325114	1.77E-25	postive
CCDC22	RASA4CP	0.38560206	3.58E-19	postive
CCDC22	RASAL2-AS1	0.369312137	1.33E-17	postive

CCDC22	RASSF1-AS1	0.417257308	1.75E-22	postive
CCDC22	RASSF8-AS1	0.323951354	1.11E-13	postive
CCDC22	RB1-DT	0.303784347	3.91E-12	postive
CCDC22	RBM15-AS1	0.455697988	5.25E-27	postive
CCDC22	RBM26-AS1	0.398817166	1.64E-20	postive
CCDC22	RDM1P5	0.382129058	7.87E-19	postive
CCDC22	RHPN1-AS1	0.418601083	1.24E-22	postive
CCDC22	RN7SL832P	0.42796113	1.10E-23	postive
CCDC22	RNASEH1-AS1	0.509300178	2.35E-34	postive
CCDC22	RNF144A-AS1	0.315493794	5.11E-13	postive
CCDC22	RNF157-AS1	0.437236478	9.21E-25	postive
CCDC22	RNF216P1	0.498344184	9.58E-33	postive
CCDC22	RNFT1-DT	0.328742761	4.59E-14	postive
CCDC22	RP11-101E13.5	0.371247452	8.74E-18	postive
CCDC22	RP11-10C24.1	0.367376138	2.01E-17	postive
CCDC22	RP11-153F5.7	0.364276212	3.90E-17	postive
CCDC22	RP11-159G9.5	0.311230393	1.08E-12	postive
CCDC22	RP11-15N24.4	0.37927465	1.49E-18	postive
CCDC22	RP11-160O5.1	0.422611695	4.44E-23	postive
CCDC22	RP11-164P12.4	0.337448713	8.81E-15	postive
CCDC22	RP11-175O19.4	0.403454792	5.38E-21	postive
CCDC22	RP11-199F11.2	0.32438421	1.03E-13	postive
CCDC22	RP11-206L10.5	0.344862354	2.07E-15	postive
CCDC22	RP11-226L15.5	0.387962684	2.09E-19	postive
CCDC22	RP1-122P22.2	0.456086382	4.69E-27	postive
CCDC22	RP11-254F7.4	0.314386372	6.22E-13	postive
CCDC22	RP11-258C19.7	0.424536253	2.69E-23	postive
CCDC22	RP11-282O18.3	0.348644642	9.77E-16	postive
CCDC22	RP11-285F16.1	0.309036196	1.59E-12	postive
CCDC22	RP11-321A17.6	0.312533825	8.62E-13	postive
CCDC22	RP11-435O5.2	0.388523395	1.83E-19	postive
CCDC22	RP11-439L18.2	0.322034516	1.58E-13	postive
CCDC22	RP11-462G12.2	0.320683846	2.02E-13	postive
CCDC22	RP11-465N4.5	0.350323555	6.97E-16	postive
CCDC22	RP11-540B6.6	0.421620466	5.73E-23	postive
CCDC22	RP11-61L19.3	0.356082987	2.16E-16	postive
CCDC22	RP11-658F2.8	0.33923423	6.24E-15	postive
CCDC22	RP11-65I12.1	0.347373202	1.26E-15	postive
CCDC22	RP11-661A12.4	0.32737496	5.92E-14	postive
CCDC22	RP11-69E11.4	0.334406424	1.58E-14	postive
CCDC22	RP11-706O15.1	0.463057079	6.12E-28	postive
CCDC22	RP11-815I9.4	0.3401132	5.26E-15	postive
CCDC22	RP11-876N24.4	0.394686945	4.37E-20	postive
CCDC22	RP11-876N24.5	0.369049152	1.41E-17	postive
CCDC22	RP11-94P11.4	0.313112355	7.79E-13	postive
CCDC22	RP13-476E20.1	0.322460916	1.46E-13	postive
CCDC22	RP3-461P17.10	0.328432702	4.86E-14	postive
CCDC22	RP4-545L17.11	0.364317787	3.86E-17	postive
CCDC22	RP5-1039K5.19	0.352229431	4.74E-16	postive
CCDC22	RP5-1042I8.7	0.329214895	4.20E-14	postive

CCDC22	RP5-1112D6.4	0.364947709	3.38E-17	postive
CCDC22	RP5-1125A11.6	0.390554008	1.15E-19	postive
CCDC22	RP5-1125A11.7	0.389553283	1.45E-19	postive
CCDC22	RP5-1139B12.2	0.31721313	3.76E-13	postive
CCDC22	RP9P	0.30543676	2.95E-12	postive
CCDC22	RPARP-AS1	0.396247417	3.02E-20	postive
CCDC22	RPL13P5	0.408556756	1.54E-21	postive
CCDC22	RPL23AP53	0.353165172	3.92E-16	postive
CCDC22	RPL23AP7	0.526221634	5.87E-37	postive
CCDC22	RPL23AP82	0.555318322	8.59E-42	postive
CCDC22	RPL32P3	0.385252408	3.88E-19	postive
CCDC22	RPLP1P6	0.332303233	2.35E-14	postive
CCDC22	RPS10P7	0.305618634	2.86E-12	postive
CCDC22	RPS18P9	0.315755118	4.88E-13	postive
CCDC22	RPS4XP16	0.419060344	1.11E-22	postive
CCDC22	RPSAP69	0.328311903	4.97E-14	postive
CCDC22	RRN3P3	0.422857009	4.17E-23	postive
CCDC22	RRP7BP	0.463952817	4.69E-28	postive
CCDC22	RTCA-AS1	0.37791094	2.02E-18	postive
CCDC22	RUSC1-AS1	0.340748772	4.65E-15	postive
CCDC22	SAMMSON	0.322526415	1.44E-13	postive
CCDC22	SAP30-DT	0.358641593	1.27E-16	postive
CCDC22	SBDSP1	0.491153853	1.01E-31	postive
CCDC22	SBF2-AS1	0.405381902	3.37E-21	postive
CCDC22	SBNO1-AS1	0.312916717	8.06E-13	postive
CCDC22	SCAMP1-AS1	0.474804885	1.77E-29	postive
CCDC22	SCAND2P	0.413110845	4.98E-22	postive
CCDC22	SCGB1B2P	0.320776021	1.98E-13	postive
CCDC22	SCOC-AS1	0.366444241	2.46E-17	postive
CCDC22	SDAD1P1	0.355611493	2.38E-16	postive
CCDC22	SDCBP2-AS1	0.32005285	2.26E-13	postive
CCDC22	SDHAP1	0.49026865	1.35E-31	postive
CCDC22	SEC24B-AS1	0.458618931	2.25E-27	postive
CCDC22	SEPSECS-AS1	0.351219846	5.82E-16	postive
CCDC22	SEPTIN4-AS1	0.440701037	3.58E-25	postive
CCDC22	SEPTIN7-AS1	0.332918526	2.09E-14	postive
CCDC22	SEPTIN7P2	0.401363161	8.91E-21	postive
CCDC22	SERHL	0.400619964	1.07E-20	postive
CCDC22	SH3GL1P1	0.422131894	5.02E-23	postive
CCDC22	SHLD2P1	0.380182745	1.22E-18	postive
CCDC22	SLC25A30-AS1	0.318164166	3.17E-13	postive
CCDC22	SLC25A5-AS1	0.493413863	4.86E-32	postive
CCDC22	SLC25A5P1	0.330660953	3.20E-14	postive
CCDC22	SLX1A-SULT1A3	0.413897729	4.09E-22	postive
CCDC22	SMG7-AS1	0.428730569	8.98E-24	postive
CCDC22	SMIM25	0.334638789	1.51E-14	postive
CCDC22	SMPD5	0.340951289	4.47E-15	postive
CCDC22	SNAI3-AS1	0.433865703	2.29E-24	postive
CCDC22	SNHG1	0.368095823	1.73E-17	postive
CCDC22	SNHG10	0.431811346	3.97E-24	postive

CCDC22	SNHG11	0.581491762	1.44E-46	postive
CCDC22	SNHG12	0.402401108	6.94E-21	postive
CCDC22	SNHG16	0.438771878	6.07E-25	postive
CCDC22	SNHG17	0.434136553	2.13E-24	postive
CCDC22	SNHG20	0.458750953	2.17E-27	postive
CCDC22	SNHG21	0.421312846	6.21E-23	postive
CCDC22	SNHG28	0.390674141	1.11E-19	postive
CCDC22	SNHG30	0.45458909	7.22E-27	postive
CCDC22	SNHG32	0.305227074	3.06E-12	postive
CCDC22	SNHG6	0.324536308	9.99E-14	postive
CCDC22	SNHG7	0.336331392	1.09E-14	postive
CCDC22	SNHG8	0.326459528	7.01E-14	postive
CCDC22	SOX2-OT	0.409493657	1.23E-21	postive
CCDC22	SP2-AS1	0.371028187	9.17E-18	postive
CCDC22	SPAG5-AS1	0.448679664	3.89E-26	postive
CCDC22	SREBF2-AS1	0.491154689	1.01E-31	postive
CCDC22	SRRM2-AS1	0.301120036	6.13E-12	postive
CCDC22	SSR4P1	0.454907998	6.59E-27	postive
CCDC22	ST20-AS1	0.360324591	8.95E-17	postive
CCDC22	ST3GAL6-AS1	0.377195482	2.37E-18	postive
CCDC22	STAG3L1	0.377446012	2.24E-18	postive
CCDC22	STAG3L2	0.426442171	1.64E-23	postive
CCDC22	STAG3L3	0.44031948	3.97E-25	postive
CCDC22	STAG3L4	0.422105908	5.06E-23	postive
CCDC22	STAG3L5P	0.404909065	3.78E-21	postive
CCDC22	STAG3L5P-PVRIG2P-PILRB	0.42458876	2.66E-23	postive
CCDC22	STARD7-AS1	0.549094445	1.02E-40	postive
CCDC22	STK24-AS1	0.307272459	2.15E-12	postive
CCDC22	STK4-AS1	0.502038137	2.79E-33	postive
CCDC22	STX18-AS1	0.399646669	1.35E-20	postive
CCDC22	SUCLA2-AS1	0.367022292	2.17E-17	postive
CCDC22	SUCLG2-AS1	0.304847749	3.26E-12	postive
CCDC22	SUMO2P17	0.518215354	1.04E-35	postive
CCDC22	SUZ12P1	0.453818599	9.01E-27	postive
CCDC22	SVIL-AS1	0.478643361	5.41E-30	postive
CCDC22	SYS1-DBNDD2	0.415543829	2.70E-22	postive
CCDC22	TAF1A-AS1	0.392015474	8.16E-20	postive
CCDC22	TBC1D8-AS1	0.412995087	5.13E-22	postive
CCDC22	TCAM1P	0.341370636	4.12E-15	postive
CCDC22	TEKT4P2	0.320754314	1.99E-13	postive
CCDC22	TFAP2A-AS1	0.334603585	1.52E-14	postive
CCDC22	THAP7-AS1	0.333372782	1.92E-14	postive
CCDC22	THAP9-AS1	0.389733067	1.39E-19	postive
CCDC22	THORLNC	0.304116482	3.69E-12	postive
CCDC22	TIPARP-AS1	0.398174303	1.91E-20	postive
CCDC22	TM4SF19-AS1	0.318223527	3.14E-13	postive
CCDC22	TMC3-AS1	0.363190619	4.90E-17	postive
CCDC22	TMCC1-AS1	0.432127079	3.65E-24	postive
CCDC22	TMED2-DT	0.349148624	8.83E-16	postive
CCDC22	TMEM147-AS1	0.442183462	2.38E-25	postive

CCDC22	TMEM198B	0.416843613	1.94E-22	postive
CCDC22	TMEM202-AS1	0.406622889	2.48E-21	postive
CCDC22	TMEM231P1	0.35433607	3.09E-16	postive
CCDC22	TMEM26-AS1	0.341640928	3.91E-15	postive
CCDC22	TMEM44-AS1	0.499163472	7.29E-33	postive
CCDC22	TMEM99	0.363189014	4.90E-17	postive
CCDC22	TMEM9B-AS1	0.304401714	3.52E-12	postive
CCDC22	TMPO-AS1	0.413375953	4.66E-22	postive
CCDC22	TNK2-AS1	0.333848561	1.75E-14	postive
CCDC22	TNRC6C-AS1	0.327594463	5.68E-14	postive
CCDC22	TOB1-AS1	0.509350557	2.31E-34	postive
CCDC22	TOLLIP-AS1	0.470475028	6.64E-29	postive
CCDC22	TONSL-AS1	0.406867467	2.34E-21	postive
CCDC22	TP53TG1	0.300428198	6.88E-12	postive
CCDC22	TP73-AS1	0.511668114	1.04E-34	postive
CCDC22	TPI1P2	0.392455259	7.37E-20	postive
CCDC22	TPM3P9	0.349146433	8.83E-16	postive
CCDC22	TPT1-AS1	0.392923962	6.60E-20	postive
CCDC22	TPTEP2	0.378714899	1.69E-18	postive
CCDC22	TRAF3IP2-AS1	0.411201916	8.02E-22	postive
CCDC22	TRAM2-AS1	0.461583725	9.45E-28	postive
CCDC22	TRAPPC12-AS1	0.384189517	4.94E-19	postive
CCDC22	TRIM52-AS1	0.433675866	2.41E-24	postive
CCDC22	TRPM2-AS	0.327784957	5.48E-14	postive
CCDC22	TSPOAP1-AS1	0.356748159	1.88E-16	postive
CCDC22	TSPY26P	0.420009372	8.67E-23	postive
CCDC22	TSTD3	0.322152952	1.54E-13	postive
CCDC22	TTC28-AS1	0.542756286	1.20E-39	postive
CCDC22	TTC41P	0.362334647	5.87E-17	postive
CCDC22	TUBBP1	0.412326092	6.06E-22	postive
CCDC22	TUG1	0.517005801	1.60E-35	postive
CCDC22	TYMSOS	0.351273759	5.75E-16	postive
CCDC22	U47924.2	0.38777331	2.18E-19	postive
CCDC22	U52111.1	0.373892184	4.91E-18	postive
CCDC22	U62317.2	0.341868413	3.74E-15	postive
CCDC22	U91328.1	0.357542893	1.60E-16	postive
CCDC22	UBA6-AS1	0.508724919	2.87E-34	postive
CCDC22	UBAC2-AS1	0.508036762	3.64E-34	postive
CCDC22	UBE2D3-AS1	0.417823183	1.52E-22	postive
CCDC22	UBE2E1-AS1	0.364362458	3.83E-17	postive
CCDC22	UBE2Q1-AS1	0.308501011	1.74E-12	postive
CCDC22	UBL7-AS1	0.329264235	4.16E-14	postive
CCDC22	UBR5-AS1	0.313044911	7.88E-13	postive
CCDC22	UGDH-AS1	0.437777433	7.96E-25	postive
CCDC22	UPF3AP2	0.318484906	3.00E-13	postive
CCDC22	URAHP	0.336442857	1.07E-14	postive
CCDC22	URB1-AS1	0.486997068	3.87E-31	postive
CCDC22	USP27X-AS1	0.651349944	1.08E-61	postive
CCDC22	USP2-AS1	0.318537272	2.97E-13	postive
CCDC22	USP46-AS1	0.43450774	1.93E-24	postive

CCDC22	UVRAG-DT	0.383920865	5.25E-19	postive
CCDC22	UXT-AS1	0.631992332	4.13E-57	postive
CCDC22	VASH1-AS1	0.388516096	1.84E-19	postive
CCDC22	VDAC1P8	0.338138333	7.71E-15	postive
CCDC22	VPS13B-DT	0.352818067	4.21E-16	postive
CCDC22	VPS33B-DT	0.355944364	2.22E-16	postive
CCDC22	WAC-AS1	0.498294242	9.74E-33	postive
CCDC22	WARS2-AS1	0.394821235	4.23E-20	postive
CCDC22	WASH3P	0.542322397	1.42E-39	postive
CCDC22	WEE2-AS1	0.314639603	5.95E-13	postive
CCDC22	WHAMMP3	0.311663975	1.00E-12	postive
CCDC22	WWC2-AS2	0.314057314	6.59E-13	postive
CCDC22	WWTR1-AS1	0.428233756	1.02E-23	postive
CCDC22	YEATS2-AS1	0.396261645	3.01E-20	postive
CCDC22	YTHDF3-AS1	0.383111014	6.31E-19	postive
CCDC22	Z83844.2	0.423117603	3.89E-23	postive
CCDC22	Z83851.4	0.423392278	3.63E-23	postive
CCDC22	Z84485.1	0.325666913	8.11E-14	postive
CCDC22	Z92544.1	0.331520564	2.73E-14	postive
CCDC22	Z94721.1	0.385789885	3.43E-19	postive
CCDC22	Z95114.1	0.321843707	1.63E-13	postive
CCDC22	Z95115.1	0.421177454	6.43E-23	postive
CCDC22	Z97055.2	0.308358524	1.78E-12	postive
CCDC22	Z97634.1	0.428862062	8.67E-24	postive
CCDC22	Z98884.2	0.323615286	1.18E-13	postive
CCDC22	Z99572.1	0.415868017	2.49E-22	postive
CCDC22	ZBED3-AS1	0.310845388	1.16E-12	postive
CCDC22	ZBED5-AS1	0.40769599	1.91E-21	postive
CCDC22	ZBTB11-AS1	0.449616173	2.98E-26	postive
CCDC22	ZEB1-AS1	0.338229504	7.58E-15	postive
CCDC22	ZFX-AS1	0.34090126	4.51E-15	postive
CCDC22	ZNF137P	0.356677595	1.91E-16	postive
CCDC22	ZNF205-AS1	0.431499868	4.31E-24	postive
CCDC22	ZNF213-AS1	0.415000769	3.10E-22	postive
CCDC22	ZNF252P	0.426265468	1.72E-23	postive
CCDC22	ZNF271P	0.351853021	5.12E-16	postive
CCDC22	ZNF286B	0.319120021	2.67E-13	postive
CCDC22	ZNF30-AS1	0.321021302	1.90E-13	postive
CCDC22	ZNF32-AS2	0.399726686	1.32E-20	postive
CCDC22	ZNF337-AS1	0.308715058	1.68E-12	postive
CCDC22	ZNF37BP	0.337631533	8.51E-15	postive
CCDC22	ZNF426-DT	0.378315268	1.85E-18	postive
CCDC22	ZNF433-AS1	0.348230964	1.06E-15	postive
CCDC22	ZNF436-AS1	0.365733644	2.86E-17	postive
CCDC22	ZNF503-AS2	0.410518015	9.51E-22	postive
CCDC22	ZNF529-AS1	0.340131852	5.24E-15	postive
CCDC22	ZNF56	0.336892288	9.81E-15	postive
CCDC22	ZNF561-AS1	0.477860216	6.90E-30	postive
CCDC22	ZNF663P	0.334753482	1.48E-14	postive
CCDC22	ZNF674-AS1	0.568562244	3.72E-44	postive

CCDC22	ZNF687-AS1	0.465541426	2.93E-28	postive
CCDC22	ZNF767P	0.390496129	1.16E-19	postive
CCDC22	ZNF788P	0.443720365	1.56E-25	postive
CCDC22	ZNF793-AS1	0.347442752	1.24E-15	postive
CCDC22	ZNF815P	0.431352401	4.48E-24	postive
CCDC22	ZNF890P	0.33755879	8.63E-15	postive
CCDC22	ZNRD1ASP	0.330265208	3.45E-14	postive
CCDC22	ZNRD2-AS1	0.533023595	4.79E-38	postive
CCDC22	ZNRF2P1	0.325413254	8.50E-14	postive
CCDC22	ZSCAN12P1	0.347318496	1.27E-15	postive
CCDC22	ZSCAN16-AS1	0.364506465	3.71E-17	postive
CCS	A1BG-AS1	0.318142127	3.19E-13	postive
CCS	AATBC	0.393856325	5.31E-20	postive
CCS	ABCA11P	0.375203444	3.68E-18	postive
CCS	ABCC6P1	0.350087833	7.31E-16	postive
CCS	ABCC6P2	0.380246129	1.20E-18	postive
CCS	AC000068.1	0.37064388	9.96E-18	postive
CCS	AC000068.2	0.498472704	9.18E-33	postive
CCS	AC002059.1	0.345970614	1.67E-15	postive
CCS	AC002116.2	0.356320484	2.05E-16	postive
CCS	AC002128.1	0.352098097	4.87E-16	postive
CCS	AC002310.1	0.469418867	9.14E-29	postive
CCS	AC002310.2	0.340158203	5.22E-15	postive
CCS	AC002347.1	0.316158364	4.54E-13	postive
CCS	AC002398.1	0.50120486	3.69E-33	postive
CCS	AC002467.1	0.451202791	1.90E-26	postive
CCS	AC002470.1	0.423246468	3.77E-23	postive
CCS	AC002550.2	0.397461608	2.27E-20	postive
CCS	AC002553.1	0.37397942	4.82E-18	postive
CCS	AC003035.1	0.305320978	3.01E-12	postive
CCS	AC003070.1	0.410842961	8.77E-22	postive
CCS	AC003101.2	0.301781185	5.48E-12	postive
CCS	AC003102.1	0.510932884	1.34E-34	postive
CCS	AC003956.1	0.39547667	3.62E-20	postive
CCS	AC003965.1	0.306169232	2.60E-12	postive
CCS	AC004067.1	0.400762425	1.03E-20	postive
CCS	AC004069.1	0.395318655	3.76E-20	postive
CCS	AC004130.2	0.339912043	5.47E-15	postive
CCS	AC004148.1	0.454687239	7.02E-27	postive
CCS	AC004156.1	0.36810812	1.72E-17	postive
CCS	AC004221.1	0.351657703	5.32E-16	postive
CCS	AC004233.1	0.422998824	4.02E-23	postive
CCS	AC004241.1	0.441269037	3.06E-25	postive
CCS	AC004241.2	0.367316143	2.04E-17	postive
CCS	AC004241.3	0.3371165	9.39E-15	postive
CCS	AC004253.1	0.431375321	4.45E-24	postive
CCS	AC004263.1	0.447304215	5.72E-26	postive
CCS	AC004447.2	0.59127871	1.82E-48	postive
CCS	AC004466.1	0.401644288	8.33E-21	postive
CCS	AC004471.2	0.384607197	4.49E-19	postive

CCS	AC004477.1	0.428022672	1.08E-23	postive
CCS	AC004477.3	0.349699326	7.90E-16	postive
CCS	AC004584.1	0.342424986	3.35E-15	postive
CCS	AC004596.1	0.404632091	4.04E-21	postive
CCS	AC004687.1	0.312719927	8.35E-13	postive
CCS	AC004706.1	0.331990932	2.49E-14	postive
CCS	AC004771.2	0.503575364	1.66E-33	postive
CCS	AC004771.4	0.396150918	3.09E-20	postive
CCS	AC004801.2	0.38283335	6.72E-19	postive
CCS	AC004801.6	0.45895362	2.04E-27	postive
CCS	AC004803.1	0.337840768	8.17E-15	postive
CCS	AC004812.2	0.505453434	8.78E-34	postive
CCS	AC004825.2	0.426485165	1.62E-23	postive
CCS	AC004839.2	0.349551559	8.14E-16	postive
CCS	AC004846.2	0.399968246	1.25E-20	postive
CCS	AC004865.2	0.349609276	8.05E-16	postive
CCS	AC004890.2	0.484633212	8.22E-31	postive
CCS	AC004893.11	0.433435255	2.57E-24	postive
CCS	AC004895.1	0.345451475	1.85E-15	postive
CCS	AC004908.2	0.300271403	7.07E-12	postive
CCS	AC004918.1	0.483069548	1.35E-30	postive
CCS	AC004923.1	0.320923407	1.93E-13	postive
CCS	AC004923.4	0.395946739	3.24E-20	postive
CCS	AC004943.2	0.356570024	1.95E-16	postive
CCS	AC004943.3	0.36106635	7.66E-17	postive
CCS	AC004951.1	0.428034458	1.08E-23	postive
CCS	AC004951.4	0.399919929	1.26E-20	postive
CCS	AC004975.2	0.329306673	4.13E-14	postive
CCS	AC004980.1	0.470234685	7.14E-29	postive
CCS	AC004982.1	0.365604808	2.94E-17	postive
CCS	AC004982.2	0.445231092	1.02E-25	postive
CCS	AC005005.3	0.456348594	4.35E-27	postive
CCS	AC005005.4	0.353371891	3.76E-16	postive
CCS	AC005014.2	0.352997903	4.05E-16	postive
CCS	AC005034.4	0.395060387	4.00E-20	postive
CCS	AC005034.5	0.313303437	7.53E-13	postive
CCS	AC005034.6	0.335705822	1.23E-14	postive
CCS	AC005037.1	0.342966197	3.01E-15	postive
CCS	AC005041.3	0.490235893	1.37E-31	postive
CCS	AC005076.2	0.420807513	7.07E-23	postive
CCS	AC005083.1	0.349588934	8.08E-16	postive
CCS	AC005104.1	0.368543299	1.57E-17	postive
CCS	AC005225.3	0.34280256	3.11E-15	postive
CCS	AC005229.4	0.509846787	1.95E-34	postive
CCS	AC005253.1	0.431426106	4.39E-24	postive
CCS	AC005261.2	0.379192117	1.52E-18	postive
CCS	AC005261.3	0.34896621	9.16E-16	postive
CCS	AC005261.4	0.524320982	1.17E-36	postive
CCS	AC005288.1	0.362018257	6.27E-17	postive
CCS	AC005306.1	0.424209888	2.93E-23	postive

CCS	AC005324.5	0.435475268	1.48E-24	postive
CCS	AC005332.1	0.447625077	5.23E-26	postive
CCS	AC005332.4	0.377956514	2.00E-18	postive
CCS	AC005332.5	0.366578807	2.39E-17	postive
CCS	AC005363.2	0.330006255	3.62E-14	postive
CCS	AC005387.1	0.583934079	4.91E-47	postive
CCS	AC005387.2	0.477173733	8.53E-30	postive
CCS	AC005391.1	0.309614235	1.44E-12	postive
CCS	AC005393.1	0.392563246	7.18E-20	postive
CCS	AC005486.1	0.305898433	2.72E-12	postive
CCS	AC005498.2	0.329066133	4.32E-14	postive
CCS	AC005498.3	0.357970352	1.46E-16	postive
CCS	AC005519.1	0.378234688	1.88E-18	postive
CCS	AC005520.4	0.430303002	5.92E-24	postive
CCS	AC005534.1	0.337915523	8.05E-15	postive
CCS	AC005562.1	0.398574995	1.74E-20	postive
CCS	AC005586.1	0.370458627	1.04E-17	postive
CCS	AC005625.1	0.302095067	5.20E-12	postive
CCS	AC005670.1	0.340770541	4.63E-15	postive
CCS	AC005670.3	0.388563114	1.82E-19	postive
CCS	AC005695.3	0.359944517	9.69E-17	postive
CCS	AC005696.1	0.479052581	4.76E-30	postive
CCS	AC005759.1	0.321304679	1.80E-13	postive
CCS	AC005775.1	0.356221177	2.10E-16	postive
CCS	AC005776.2	0.342803813	3.11E-15	postive
CCS	AC005785.1	0.512911597	6.74E-35	postive
CCS	AC005790.1	0.341652485	3.90E-15	postive
CCS	AC005837.1	0.328306739	4.97E-14	postive
CCS	AC005840.2	0.336570335	1.04E-14	postive
CCS	AC005840.4	0.390908413	1.06E-19	postive
CCS	AC005899.7	0.333007603	2.06E-14	postive
CCS	AC005911.1	0.483977338	1.01E-30	postive
CCS	AC005920.1	0.311733427	9.92E-13	postive
CCS	AC005920.3	0.347082588	1.33E-15	postive
CCS	AC005954.1	0.325146419	8.93E-14	postive
CCS	AC006001.2	0.485934011	5.44E-31	postive
CCS	AC006001.3	0.490955531	1.08E-31	postive
CCS	AC006042.1	0.452264309	1.41E-26	postive
CCS	AC006064.3	0.379943751	1.29E-18	postive
CCS	AC006111.2	0.414302874	3.69E-22	postive
CCS	AC006213.1	0.348584117	9.89E-16	postive
CCS	AC006252.1	0.513428263	5.63E-35	postive
CCS	AC006254.1	0.415417142	2.79E-22	postive
CCS	AC006333.1	0.436430283	1.15E-24	postive
CCS	AC006435.2	0.472735224	3.34E-29	postive
CCS	AC006449.2	0.489156783	1.94E-31	postive
CCS	AC006449.5	0.352244469	4.73E-16	postive
CCS	AC006449.7	0.507923236	3.78E-34	postive
CCS	AC006483.2	0.330074399	3.58E-14	postive
CCS	AC006547.1	0.465317151	3.13E-28	postive

CCS	AC006942.1	0.494839707	3.05E-32	postive
CCS	AC007032.1	0.423180383	3.83E-23	postive
CCS	AC007038.1	0.36689851	2.23E-17	postive
CCS	AC007066.2	0.462640593	6.92E-28	postive
CCS	AC007098.1	0.335631555	1.25E-14	postive
CCS	AC007114.1	0.439636594	4.79E-25	postive
CCS	AC007220.1	0.329964929	3.65E-14	postive
CCS	AC007228.9	0.368940705	1.44E-17	postive
CCS	AC007285.1	0.30468309	3.35E-12	postive
CCS	AC007292.1	0.508790336	2.81E-34	postive
CCS	AC007292.2	0.471272946	5.21E-29	postive
CCS	AC007318.3	0.32227608	1.51E-13	postive
CCS	AC007342.4	0.350253655	7.07E-16	postive
CCS	AC007342.5	0.325027016	9.13E-14	postive
CCS	AC007383.2	0.546752411	2.55E-40	postive
CCS	AC007405.4	0.393467657	5.81E-20	postive
CCS	AC007406.5	0.315756927	4.88E-13	postive
CCS	AC007541.1	0.418068538	1.42E-22	postive
CCS	AC007566.1	0.39376626	5.42E-20	postive
CCS	AC007570.1	0.308952926	1.61E-12	postive
CCS	AC007613.1	0.370540137	1.02E-17	postive
CCS	AC007663.3	0.416763078	1.98E-22	postive
CCS	AC007663.4	0.412414318	5.93E-22	postive
CCS	AC007686.3	0.469594065	8.67E-29	postive
CCS	AC007773.1	0.3392865	6.18E-15	postive
CCS	AC007879.3	0.389319879	1.53E-19	postive
CCS	AC007879.4	0.324174818	1.07E-13	postive
CCS	AC007881.3	0.320878087	1.95E-13	postive
CCS	AC007938.3	0.309699184	1.41E-12	postive
CCS	AC008013.2	0.341050095	4.39E-15	postive
CCS	AC008014.1	0.358069893	1.43E-16	postive
CCS	AC008073.2	0.328641361	4.67E-14	postive
CCS	AC008105.1	0.400968069	9.80E-21	postive
CCS	AC008105.2	0.512926508	6.70E-35	postive
CCS	AC008124.1	0.402668794	6.50E-21	postive
CCS	AC008267.5	0.475730241	1.33E-29	postive
CCS	AC008280.3	0.415103102	3.02E-22	postive
CCS	AC008393.1	0.470969318	5.72E-29	postive
CCS	AC008438.1	0.532235571	6.42E-38	postive
CCS	AC008443.1	0.51331246	5.86E-35	postive
CCS	AC008443.5	0.352539849	4.45E-16	postive
CCS	AC008494.3	0.36273515	5.40E-17	postive
CCS	AC008608.2	0.429462937	7.40E-24	postive
CCS	AC008610.1	0.424389449	2.80E-23	postive
CCS	AC008622.2	0.505516829	8.60E-34	postive
CCS	AC008655.2	0.450076877	2.62E-26	postive
CCS	AC008734.1	0.444562358	1.23E-25	postive
CCS	AC008735.1	0.445905293	8.47E-26	postive
CCS	AC008735.2	0.459857031	1.57E-27	postive
CCS	AC008735.4	0.45468775	7.02E-27	postive

CCS	AC008750.5	0.353779648	3.46E-16	postive
CCS	AC008752.2	0.347360595	1.26E-15	postive
CCS	AC008758.4	0.307409763	2.10E-12	postive
CCS	AC008760.1	0.416213094	2.28E-22	postive
CCS	AC008763.1	0.426071394	1.81E-23	postive
CCS	AC008764.2	0.366330811	2.52E-17	postive
CCS	AC008764.6	0.453688883	9.35E-27	postive
CCS	AC008764.8	0.481790466	2.02E-30	postive
CCS	AC008771.1	0.449766007	2.86E-26	postive
CCS	AC008781.1	0.367762275	1.85E-17	postive
CCS	AC008870.3	0.511470772	1.11E-34	postive
CCS	AC008870.5	0.346403408	1.53E-15	postive
CCS	AC008915.3	0.602755524	8.89E-51	postive
CCS	AC008946.1	0.342611978	3.23E-15	postive
CCS	AC008966.1	0.427006173	1.41E-23	postive
CCS	AC008969.1	0.371248577	8.74E-18	postive
CCS	AC008982.2	0.350575117	6.63E-16	postive
CCS	AC009005.1	0.417169389	1.79E-22	postive
CCS	AC009019.1	0.33019087	3.50E-14	postive
CCS	AC009022.1	0.479656002	3.94E-30	postive
CCS	AC009034.1	0.306624949	2.41E-12	postive
CCS	AC009053.3	0.332538259	2.25E-14	postive
CCS	AC009061.2	0.511099468	1.26E-34	postive
CCS	AC009065.5	0.556314266	5.75E-42	postive
CCS	AC009065.7	0.316532528	4.25E-13	postive
CCS	AC009065.9	0.574697885	2.75E-45	postive
CCS	AC009087.1	0.318155854	3.18E-13	postive
CCS	AC009088.1	0.302585654	4.79E-12	postive
CCS	AC009097.2	0.334715984	1.49E-14	postive
CCS	AC009107.2	0.424056222	3.05E-23	postive
CCS	AC009113.1	0.522111779	2.60E-36	postive
CCS	AC009118.2	0.327333921	5.96E-14	postive
CCS	AC009118.3	0.510958761	1.33E-34	postive
CCS	AC009120.2	0.398827088	1.64E-20	postive
CCS	AC009121.3	0.332211797	2.39E-14	postive
CCS	AC009121.4	0.321055466	1.89E-13	postive
CCS	AC009126.1	0.549271528	9.50E-41	postive
CCS	AC009148.1	0.419616091	9.59E-23	postive
CCS	AC009159.3	0.319030517	2.72E-13	postive
CCS	AC009163.7	0.402139251	7.39E-21	postive
CCS	AC009171.2	0.417915095	1.48E-22	postive
CCS	AC009226.1	0.323551321	1.20E-13	postive
CCS	AC009237.14	0.419281047	1.05E-22	postive
CCS	AC009283.1	0.317212052	3.76E-13	postive
CCS	AC009309.2	0.391165145	9.95E-20	postive
CCS	AC009403.1	0.449881227	2.77E-26	postive
CCS	AC009404.1	0.441113089	3.20E-25	postive
CCS	AC009506.2	0.460745985	1.21E-27	postive
CCS	AC009570.1	0.359372341	1.09E-16	postive
CCS	AC009630.2	0.30238113	4.95E-12	postive

CCS	AC009690.2	0.524275498	1.19E-36	postive
CCS	AC009779.3	0.619320902	2.77E-54	postive
CCS	AC009812.1	0.39331476	6.02E-20	postive
CCS	AC009831.1	0.419943561	8.82E-23	postive
CCS	AC009961.1	0.308299045	1.80E-12	postive
CCS	AC010136.1	0.356273152	2.07E-16	postive
CCS	AC010201.1	0.335712684	1.23E-14	postive
CCS	AC010201.2	0.339125713	6.37E-15	postive
CCS	AC010226.1	0.393910249	5.24E-20	postive
CCS	AC010271.1	0.468062611	1.38E-28	postive
CCS	AC010273.2	0.373618343	5.21E-18	postive
CCS	AC010319.4	0.490154743	1.40E-31	postive
CCS	AC010320.2	0.311138878	1.10E-12	postive
CCS	AC010326.4	0.44235951	2.27E-25	postive
CCS	AC010326.5	0.463790634	4.92E-28	postive
CCS	AC010331.1	0.441313045	3.02E-25	postive
CCS	AC010336.2	0.354432597	3.03E-16	postive
CCS	AC010359.2	0.448385791	4.22E-26	postive
CCS	AC010422.1	0.340077346	5.30E-15	postive
CCS	AC010422.2	0.471139417	5.43E-29	postive
CCS	AC010442.1	0.432345089	3.44E-24	postive
CCS	AC010463.3	0.431393437	4.43E-24	postive
CCS	AC010491.1	0.465418218	3.03E-28	postive
CCS	AC010503.5	0.475476438	1.44E-29	postive
CCS	AC010504.1	0.402375641	6.98E-21	postive
CCS	AC010525.1	0.4364218	1.15E-24	postive
CCS	AC010530.1	0.311051227	1.12E-12	postive
CCS	AC010531.6	0.355211068	2.58E-16	postive
CCS	AC010536.1	0.351912756	5.05E-16	postive
CCS	AC010542.6	0.393352012	5.97E-20	postive
CCS	AC010547.1	0.33327721	1.96E-14	postive
CCS	AC010618.2	0.429012558	8.33E-24	postive
CCS	AC010618.3	0.44411422	1.39E-25	postive
CCS	AC010643.1	0.312835523	8.18E-13	postive
CCS	AC010654.1	0.329881497	3.71E-14	postive
CCS	AC010655.4	0.327551084	5.73E-14	postive
CCS	AC010680.2	0.300054347	7.33E-12	postive
CCS	AC010719.1	0.383380707	5.93E-19	postive
CCS	AC010761.1	0.470858931	5.91E-29	postive
CCS	AC010761.4	0.492214452	7.19E-32	postive
CCS	AC010761.6	0.44365253	1.58E-25	postive
CCS	AC010809.1	0.348885113	9.31E-16	postive
CCS	AC010809.2	0.347800307	1.16E-15	postive
CCS	AC010834.2	0.317536824	3.55E-13	postive
CCS	AC010864.1	0.494121873	3.86E-32	postive
CCS	AC010883.1	0.512070028	9.03E-35	postive
CCS	AC010894.2	0.417270953	1.74E-22	postive
CCS	AC010904.2	0.347687598	1.18E-15	postive
CCS	AC010913.1	0.404677112	4.00E-21	postive
CCS	AC010969.2	0.471680309	4.61E-29	postive

CCS	AC010973.1	0.342139994	3.54E-15	postive
CCS	AC010973.2	0.444472486	1.26E-25	postive
CCS	AC010997.4	0.49462668	3.27E-32	postive
CCS	AC010997.5	0.43590732	1.32E-24	postive
CCS	AC010999.1	0.307913287	1.93E-12	postive
CCS	AC011330.2	0.308389735	1.77E-12	postive
CCS	AC011374.2	0.343000942	2.99E-15	postive
CCS	AC011450.1	0.323622843	1.18E-13	postive
CCS	AC011461.1	0.402107297	7.45E-21	postive
CCS	AC011462.4	0.51603064	2.26E-35	postive
CCS	AC011466.3	0.36944122	1.29E-17	postive
CCS	AC011468.1	0.349361357	8.46E-16	postive
CCS	AC011468.5	0.345428293	1.85E-15	postive
CCS	AC011472.1	0.522958168	1.92E-36	postive
CCS	AC011472.2	0.4063179	2.68E-21	postive
CCS	AC011476.3	0.3035895	4.04E-12	postive
CCS	AC011479.3	0.346631097	1.46E-15	postive
CCS	AC011481.1	0.449559696	3.03E-26	postive
CCS	AC011481.2	0.313841865	6.85E-13	postive
CCS	AC011491.2	0.343105247	2.93E-15	postive
CCS	AC011498.3	0.309350867	1.50E-12	postive
CCS	AC011498.6	0.455454472	5.63E-27	postive
CCS	AC011511.1	0.451022644	2.00E-26	postive
CCS	AC011511.2	0.341225024	4.24E-15	postive
CCS	AC011731.1	0.443698704	1.56E-25	postive
CCS	AC011773.3	0.314520883	6.07E-13	postive
CCS	AC011921.1	0.445022536	1.08E-25	postive
CCS	AC011978.2	0.369626492	1.24E-17	postive
CCS	AC012063.1	0.447895828	4.85E-26	postive
CCS	AC012073.1	0.383989988	5.17E-19	postive
CCS	AC012146.1	0.317625215	3.50E-13	postive
CCS	AC012173.1	0.403753335	5.00E-21	postive
CCS	AC012184.3	0.470798769	6.02E-29	postive
CCS	AC012186.2	0.433096304	2.81E-24	postive
CCS	AC012236.1	0.312787565	8.25E-13	postive
CCS	AC012306.2	0.546807392	2.50E-40	postive
CCS	AC012313.1	0.469064408	1.02E-28	postive
CCS	AC012313.2	0.337705711	8.39E-15	postive
CCS	AC012313.3	0.321059718	1.88E-13	postive
CCS	AC012313.6	0.326831768	6.54E-14	postive
CCS	AC012313.8	0.308511936	1.74E-12	postive
CCS	AC012313.9	0.443818231	1.51E-25	postive
CCS	AC012360.2	0.393081088	6.36E-20	postive
CCS	AC012360.3	0.48874926	2.21E-31	postive
CCS	AC012435.2	0.330933211	3.04E-14	postive
CCS	AC012442.2	0.465958694	2.58E-28	postive
CCS	AC012442.3	0.325813928	7.90E-14	postive
CCS	AC012467.2	0.465535608	2.93E-28	postive
CCS	AC012510.1	0.528084644	2.97E-37	postive
CCS	AC012603.1	0.369813989	1.19E-17	postive

CCS	AC012615.1	0.498635602	8.70E-33	postive
CCS	AC012615.4	0.343649819	2.63E-15	postive
CCS	AC012615.6	0.476488633	1.05E-29	postive
CCS	AC012640.1	0.315267544	5.32E-13	postive
CCS	AC012640.4	0.331695086	2.64E-14	postive
CCS	AC012645.3	0.376050218	3.05E-18	postive
CCS	AC012645.4	0.336261032	1.11E-14	postive
CCS	AC012676.1	0.379901077	1.30E-18	postive
CCS	AC013356.2	0.361118527	7.58E-17	postive
CCS	AC013400.1	0.337987399	7.94E-15	postive
CCS	AC013468.1	0.327596851	5.68E-14	postive
CCS	AC013472.2	0.323971761	1.11E-13	postive
CCS	AC013726.2	0.348451983	1.02E-15	postive
CCS	AC013731.1	0.37369354	5.13E-18	postive
CCS	AC015660.3	0.30002071	7.37E-12	postive
CCS	AC015726.1	0.419525964	9.82E-23	postive
CCS	AC015726.2	0.317991849	3.27E-13	postive
CCS	AC015802.3	0.349983259	7.46E-16	postive
CCS	AC015802.4	0.467766562	1.50E-28	postive
CCS	AC015802.5	0.457393176	3.21E-27	postive
CCS	AC015818.3	0.33932054	6.14E-15	postive
CCS	AC015871.3	0.367647388	1.90E-17	postive
CCS	AC015912.3	0.387292733	2.43E-19	postive
CCS	AC015921.1	0.300431422	6.88E-12	postive
CCS	AC015922.3	0.39069211	1.11E-19	postive
CCS	AC015961.2	0.397532279	2.23E-20	postive
CCS	AC015982.2	0.433555653	2.49E-24	postive
CCS	AC016027.1	0.349663316	7.96E-16	postive
CCS	AC016065.1	0.510404537	1.61E-34	postive
CCS	AC016355.1	0.340714619	4.68E-15	postive
CCS	AC016394.2	0.482524705	1.60E-30	postive
CCS	AC016394.3	0.316003515	4.67E-13	postive
CCS	AC016526.2	0.351862452	5.11E-16	postive
CCS	AC016575.1	0.329425686	4.04E-14	postive
CCS	AC016722.2	0.385419391	3.73E-19	postive
CCS	AC016727.1	0.457256133	3.34E-27	postive
CCS	AC016737.1	0.489327559	1.83E-31	postive
CCS	AC016747.1	0.34066542	4.73E-15	postive
CCS	AC016773.2	0.4834934	1.18E-30	postive
CCS	AC016866.1	0.321920278	1.61E-13	postive
CCS	AC016876.2	0.515103078	3.13E-35	postive
CCS	AC016876.3	0.542092253	1.55E-39	postive
CCS	AC016876.4	0.456932412	3.67E-27	postive
CCS	AC017083.1	0.510831588	1.39E-34	postive
CCS	AC017104.1	0.331299836	2.84E-14	postive
CCS	AC018413.1	0.367211234	2.09E-17	postive
CCS	AC018521.5	0.420274518	8.10E-23	postive
CCS	AC018521.6	0.377059443	2.44E-18	postive
CCS	AC018521.7	0.313847221	6.84E-13	postive
CCS	AC018638.6	0.307817834	1.96E-12	postive

CCS	AC018647.2	0.429077394	8.19E-24	postive
CCS	AC018648.1	0.339230284	6.25E-15	postive
CCS	AC018653.3	0.391517535	9.17E-20	postive
CCS	AC018682.2	0.341973774	3.66E-15	postive
CCS	AC018690.1	0.447235489	5.83E-26	postive
CCS	AC018695.4	0.338216821	7.60E-15	postive
CCS	AC018755.3	0.397808907	2.09E-20	postive
CCS	AC018766.4	0.551498524	3.94E-41	postive
CCS	AC018809.1	0.4302408	6.02E-24	postive
CCS	AC018809.2	0.387424956	2.36E-19	postive
CCS	AC018845.3	0.427213493	1.34E-23	postive
CCS	AC019131.1	0.30642792	2.49E-12	postive
CCS	AC019131.2	0.335520654	1.28E-14	postive
CCS	AC019205.1	0.395602066	3.52E-20	postive
CCS	AC019294.2	0.484887477	7.59E-31	postive
CCS	AC020558.1	0.395315685	3.76E-20	postive
CCS	AC020558.2	0.476973074	9.08E-30	postive
CCS	AC020594.1	0.348282835	1.05E-15	postive
CCS	AC020658.5	0.308500476	1.74E-12	postive
CCS	AC020663.2	0.481907588	1.95E-30	postive
CCS	AC020765.2	0.352914523	4.12E-16	postive
CCS	AC020907.4	0.471074726	5.54E-29	postive
CCS	AC020910.5	0.389454541	1.48E-19	postive
CCS	AC020911.1	0.339429243	6.01E-15	postive
CCS	AC020915.2	0.307535454	2.06E-12	postive
CCS	AC020915.3	0.528183719	2.87E-37	postive
CCS	AC020978.4	0.336204104	1.12E-14	postive
CCS	AC020978.7	0.318511514	2.98E-13	postive
CCS	AC021016.3	0.410123371	1.05E-21	postive
CCS	AC021028.1	0.311590085	1.02E-12	postive
CCS	AC021148.1	0.402697618	6.46E-21	postive
CCS	AC021491.2	0.38043893	1.15E-18	postive
CCS	AC021755.2	0.439770582	4.62E-25	postive
CCS	AC021755.3	0.3133151	7.52E-13	postive
CCS	AC022007.1	0.497717981	1.18E-32	postive
CCS	AC022098.1	0.478196967	6.21E-30	postive
CCS	AC022098.4	0.319357121	2.56E-13	postive
CCS	AC022144.1	0.354750103	2.83E-16	postive
CCS	AC022150.2	0.489147074	1.94E-31	postive
CCS	AC022154.1	0.33057009	3.26E-14	postive
CCS	AC022167.2	0.522293502	2.43E-36	postive
CCS	AC022167.3	0.315258148	5.33E-13	postive
CCS	AC022167.4	0.367059029	2.15E-17	postive
CCS	AC022211.3	0.40587157	2.99E-21	postive
CCS	AC022296.4	0.355246353	2.56E-16	postive
CCS	AC022306.2	0.344691474	2.15E-15	postive
CCS	AC022364.1	0.312996877	7.95E-13	postive
CCS	AC022382.1	0.312921028	8.06E-13	postive
CCS	AC022400.1	0.437308214	9.04E-25	postive
CCS	AC022400.5	0.369245089	1.35E-17	postive

CCS	AC022400.6	0.311324702	1.07E-12	postive
CCS	AC022415.2	0.40767036	1.92E-21	postive
CCS	AC022558.3	0.335373136	1.31E-14	postive
CCS	AC022613.2	0.312666553	8.42E-13	postive
CCS	AC022762.2	0.36849622	1.58E-17	postive
CCS	AC022916.2	0.470001128	7.67E-29	postive
CCS	AC023302.1	0.33878103	6.81E-15	postive
CCS	AC023509.3	0.435152378	1.62E-24	postive
CCS	AC023632.2	0.320205077	2.20E-13	postive
CCS	AC023790.2	0.326858919	6.51E-14	postive
CCS	AC023794.2	0.323393161	1.23E-13	postive
CCS	AC023908.3	0.376016113	3.08E-18	postive
CCS	AC024060.2	0.471070867	5.54E-29	postive
CCS	AC024075.2	0.493101541	5.38E-32	postive
CCS	AC024243.1	0.433533046	2.50E-24	postive
CCS	AC024257.3	0.383551331	5.71E-19	postive
CCS	AC024257.4	0.309357795	1.50E-12	postive
CCS	AC024267.4	0.32674414	6.65E-14	postive
CCS	AC024361.1	0.420867814	6.96E-23	postive
CCS	AC024361.2	0.403257364	5.64E-21	postive
CCS	AC024361.3	0.359787259	1.00E-16	postive
CCS	AC024560.3	0.368572565	1.56E-17	postive
CCS	AC024575.2	0.490592818	1.22E-31	postive
CCS	AC024896.1	0.307168638	2.19E-12	postive
CCS	AC024941.2	0.350876084	6.23E-16	postive
CCS	AC025048.2	0.43743527	8.73E-25	postive
CCS	AC025048.4	0.313349854	7.47E-13	postive
CCS	AC025162.2	0.376826259	2.57E-18	postive
CCS	AC025165.5	0.304622193	3.39E-12	postive
CCS	AC025166.1	0.320273388	2.17E-13	postive
CCS	AC025171.1	0.384818143	4.28E-19	postive
CCS	AC025171.2	0.338576173	7.09E-15	postive
CCS	AC025171.5	0.372157392	7.17E-18	postive
CCS	AC025178.2	0.378184537	1.90E-18	postive
CCS	AC025181.2	0.45041684	2.38E-26	postive
CCS	AC025262.1	0.300217314	7.13E-12	postive
CCS	AC025265.1	0.400067528	1.22E-20	postive
CCS	AC025287.3	0.377357699	2.29E-18	postive
CCS	AC025682.2	0.425025124	2.37E-23	postive
CCS	AC025754.2	0.396388272	2.92E-20	postive
CCS	AC025857.2	0.323152754	1.29E-13	postive
CCS	AC026191.1	0.339403257	6.04E-15	postive
CCS	AC026304.1	0.394221992	4.87E-20	postive
CCS	AC026367.1	0.371514044	8.25E-18	postive
CCS	AC026367.2	0.3190533	2.71E-13	postive
CCS	AC026367.3	0.351185558	5.86E-16	postive
CCS	AC026401.3	0.334682316	1.50E-14	postive
CCS	AC026412.1	0.371951213	7.50E-18	postive
CCS	AC026412.3	0.304812489	3.28E-12	postive
CCS	AC026471.1	0.544877389	5.29E-40	postive

CCS	AC026471.2	0.406612501	2.49E-21	postive
CCS	AC026471.4	0.477059908	8.84E-30	postive
CCS	AC026691.1	0.422807437	4.22E-23	postive
CCS	AC026740.1	0.340884953	4.53E-15	postive
CCS	AC026801.2	0.362442776	5.74E-17	postive
CCS	AC026803.2	0.419868539	8.99E-23	postive
CCS	AC026979.2	0.449776475	2.85E-26	postive
CCS	AC026979.3	0.360676095	8.31E-17	postive
CCS	AC027020.2	0.320202834	2.20E-13	postive
CCS	AC027097.1	0.398724605	1.68E-20	postive
CCS	AC027097.2	0.317185939	3.78E-13	postive
CCS	AC027117.2	0.309879116	1.37E-12	postive
CCS	AC027237.5	0.348554572	9.95E-16	postive
CCS	AC027271.1	0.33699987	9.61E-15	postive
CCS	AC027307.1	0.382185216	7.77E-19	postive
CCS	AC027307.2	0.436730392	1.06E-24	postive
CCS	AC027575.3	0.340117968	5.26E-15	postive
CCS	AC027601.1	0.414050789	3.93E-22	postive
CCS	AC027601.2	0.548457419	1.31E-40	postive
CCS	AC027601.3	0.362504437	5.66E-17	postive
CCS	AC027644.3	0.559097029	1.86E-42	postive
CCS	AC027682.1	0.378206172	1.89E-18	postive
CCS	AC027682.4	0.368910909	1.45E-17	postive
CCS	AC027682.6	0.378106434	1.94E-18	postive
CCS	AC027763.2	0.364234054	3.93E-17	postive
CCS	AC027796.4	0.477985944	6.63E-30	postive
CCS	AC032044.1	0.355871107	2.25E-16	postive
CCS	AC034102.5	0.311668208	1.00E-12	postive
CCS	AC034102.6	0.385006549	4.10E-19	postive
CCS	AC034231.1	0.34745734	1.24E-15	postive
CCS	AC034236.2	0.498552881	8.94E-33	postive
CCS	AC036176.1	0.426829949	1.48E-23	postive
CCS	AC037459.2	0.426859757	1.47E-23	postive
CCS	AC040162.3	0.518212007	1.05E-35	postive
CCS	AC040169.1	0.404969682	3.72E-21	postive
CCS	AC040970.1	0.400562104	1.08E-20	postive
CCS	AC040977.1	0.449861938	2.78E-26	postive
CCS	AC044849.1	0.301709236	5.55E-12	postive
CCS	AC046134.2	0.432644184	3.18E-24	postive
CCS	AC046143.2	0.336136268	1.13E-14	postive
CCS	AC048341.2	0.334174373	1.65E-14	postive
CCS	AC048382.1	0.344649057	2.16E-15	postive
CCS	AC048382.2	0.369285353	1.34E-17	postive
CCS	AC048382.5	0.303986644	3.77E-12	postive
CCS	AC051619.6	0.374719719	4.09E-18	postive
CCS	AC053503.1	0.344672908	2.15E-15	postive
CCS	AC053503.4	0.457697619	2.94E-27	postive
CCS	AC055822.1	0.330341819	3.40E-14	postive
CCS	AC055855.2	0.429225168	7.88E-24	postive
CCS	AC060766.4	0.349049597	9.01E-16	postive

CCS	AC060766.7	0.402687936	6.47E-21	postive
CCS	AC060780.1	0.516011468	2.27E-35	postive
CCS	AC061992.2	0.367397842	2.00E-17	postive
CCS	AC064807.1	0.444777868	1.16E-25	postive
CCS	AC064807.4	0.386233528	3.10E-19	postive
CCS	AC064836.3	0.463233305	5.81E-28	postive
CCS	AC067750.1	0.495987591	2.09E-32	postive
CCS	AC067838.1	0.403534774	5.27E-21	postive
CCS	AC067852.2	0.600156998	3.02E-50	postive
CCS	AC067930.2	0.340333387	5.04E-15	postive
CCS	AC067930.4	0.368372419	1.63E-17	postive
CCS	AC068338.2	0.496219659	1.94E-32	postive
CCS	AC068338.3	0.429686125	6.97E-24	postive
CCS	AC068473.5	0.449346216	3.22E-26	postive
CCS	AC068580.1	0.346051788	1.64E-15	postive
CCS	AC068580.3	0.396153537	3.09E-20	postive
CCS	AC068620.1	0.373740841	5.07E-18	postive
CCS	AC068620.2	0.431202794	4.66E-24	postive
CCS	AC068724.2	0.342018116	3.63E-15	postive
CCS	AC068768.1	0.316869001	4.00E-13	postive
CCS	AC068790.8	0.300809717	6.46E-12	postive
CCS	AC068831.2	0.529471385	1.79E-37	postive
CCS	AC068831.3	0.404395029	4.28E-21	postive
CCS	AC068870.1	0.31369961	7.02E-13	postive
CCS	AC068870.2	0.420009134	8.67E-23	postive
CCS	AC068888.1	0.450688326	2.20E-26	postive
CCS	AC069185.1	0.321219409	1.83E-13	postive
CCS	AC069224.2	0.326505924	6.95E-14	postive
CCS	AC069307.1	0.422975508	4.04E-23	postive
CCS	AC069544.1	0.414502285	3.51E-22	postive
CCS	AC072061.1	0.512709209	7.23E-35	postive
CCS	AC073073.2	0.351430571	5.57E-16	postive
CCS	AC073111.1	0.350419602	6.84E-16	postive
CCS	AC073133.2	0.366290505	2.54E-17	postive
CCS	AC073195.2	0.554143124	1.38E-41	postive
CCS	AC073254.1	0.34876599	9.53E-16	postive
CCS	AC073263.1	0.306528172	2.45E-12	postive
CCS	AC073288.2	0.354864453	2.77E-16	postive
CCS	AC073314.1	0.365070637	3.29E-17	postive
CCS	AC073352.1	0.386474881	2.93E-19	postive
CCS	AC073389.1	0.344110948	2.41E-15	postive
CCS	AC073508.3	0.48177638	2.03E-30	postive
CCS	AC073517.1	0.408787486	1.46E-21	postive
CCS	AC073573.1	0.355464249	2.45E-16	postive
CCS	AC073575.4	0.520046488	5.44E-36	postive
CCS	AC073611.1	0.396192817	3.06E-20	postive
CCS	AC073655.2	0.426030858	1.82E-23	postive
CCS	AC073842.2	0.497052915	1.47E-32	postive
CCS	AC073869.1	0.574892215	2.53E-45	postive
CCS	AC073869.3	0.305422556	2.96E-12	postive

CCS	AC073896.2	0.472482356	3.61E-29	postive
CCS	AC073896.3	0.342229713	3.48E-15	postive
CCS	AC073896.4	0.574750603	2.69E-45	postive
CCS	AC073896.5	0.363244971	4.85E-17	postive
CCS	AC073957.3	0.345388446	1.87E-15	postive
CCS	AC074029.3	0.347569823	1.21E-15	postive
CCS	AC074032.1	0.312487067	8.69E-13	postive
CCS	AC074117.1	0.543264441	9.87E-40	postive
CCS	AC074212.1	0.498785048	8.27E-33	postive
CCS	AC078846.1	0.402125495	7.42E-21	postive
CCS	AC078909.2	0.333977482	1.71E-14	postive
CCS	AC079145.1	0.374289283	4.50E-18	postive
CCS	AC079148.1	0.367348186	2.03E-17	postive
CCS	AC079174.1	0.348330477	1.04E-15	postive
CCS	AC079174.2	0.505629903	8.27E-34	postive
CCS	AC079305.2	0.365731315	2.86E-17	postive
CCS	AC079322.1	0.385618347	3.57E-19	postive
CCS	AC079336.5	0.363701896	4.40E-17	postive
CCS	AC079414.3	0.514214055	4.28E-35	postive
CCS	AC079447.1	0.341531178	3.99E-15	postive
CCS	AC079807.1	0.523112063	1.81E-36	postive
CCS	AC079834.2	0.327132467	6.19E-14	postive
CCS	AC079848.1	0.334730072	1.48E-14	postive
CCS	AC079906.1	0.340956044	4.47E-15	postive
CCS	AC079907.1	0.383408427	5.90E-19	postive
CCS	AC079922.2	0.494108628	3.87E-32	postive
CCS	AC080013.5	0.324266928	1.05E-13	postive
CCS	AC080037.2	0.331453302	2.76E-14	postive
CCS	AC080038.3	0.352877545	4.16E-16	postive
CCS	AC080112.2	0.444251689	1.34E-25	postive
CCS	AC080129.2	0.471457357	4.93E-29	postive
CCS	AC083799.1	0.401603126	8.41E-21	postive
CCS	AC083862.2	0.322874933	1.35E-13	postive
CCS	AC083880.1	0.437105224	9.55E-25	postive
CCS	AC083964.1	0.461037385	1.11E-27	postive
CCS	AC084018.1	0.442027077	2.48E-25	postive
CCS	AC084018.2	0.378944422	1.61E-18	postive
CCS	AC084036.1	0.386831301	2.70E-19	postive
CCS	AC084125.2	0.497809527	1.14E-32	postive
CCS	AC084125.4	0.502217611	2.62E-33	postive
CCS	AC084809.1	0.362270579	5.95E-17	postive
CCS	AC084809.2	0.352689008	4.32E-16	postive
CCS	AC084824.6	0.411915597	6.71E-22	postive
CCS	AC084864.1	0.332181996	2.41E-14	postive
CCS	AC087164.1	0.373022721	5.94E-18	postive
CCS	AC087239.1	0.340130726	5.24E-15	postive
CCS	AC087269.1	0.318419193	3.03E-13	postive
CCS	AC087289.1	0.385706266	3.50E-19	postive
CCS	AC087289.2	0.602192386	1.16E-50	postive
CCS	AC087289.5	0.434514574	1.92E-24	postive

CCS	AC087301.1	0.319255537	2.61E-13	postive
CCS	AC087392.3	0.377859613	2.05E-18	postive
CCS	AC087392.4	0.328907884	4.45E-14	postive
CCS	AC087500.1	0.448589271	3.99E-26	postive
CCS	AC087500.2	0.342826317	3.10E-15	postive
CCS	AC087501.4	0.434346704	2.01E-24	postive
CCS	AC087521.3	0.362548159	5.61E-17	postive
CCS	AC087623.2	0.373504138	5.34E-18	postive
CCS	AC087645.2	0.370922777	9.38E-18	postive
CCS	AC087741.1	0.514331397	4.10E-35	postive
CCS	AC087741.2	0.488448566	2.43E-31	postive
CCS	AC087742.1	0.343541176	2.69E-15	postive
CCS	AC087752.3	0.403761421	4.99E-21	postive
CCS	AC087752.4	0.318299793	3.10E-13	postive
CCS	AC090114.2	0.514910798	3.35E-35	postive
CCS	AC090198.1	0.364827831	3.47E-17	postive
CCS	AC090246.1	0.316197609	4.51E-13	postive
CCS	AC090260.2	0.331457675	2.76E-14	postive
CCS	AC090425.2	0.368831881	1.47E-17	postive
CCS	AC090510.2	0.315009001	5.57E-13	postive
CCS	AC090515.3	0.400671703	1.05E-20	postive
CCS	AC090527.4	0.365862227	2.78E-17	postive
CCS	AC090587.1	0.538303873	6.58E-39	postive
CCS	AC090587.2	0.475506455	1.43E-29	postive
CCS	AC090589.3	0.476704649	9.86E-30	postive
CCS	AC090617.5	0.414593165	3.43E-22	postive
CCS	AC090844.2	0.301084655	6.16E-12	postive
CCS	AC090912.1	0.376154948	2.98E-18	postive
CCS	AC090970.2	0.371605713	8.08E-18	postive
CCS	AC091153.3	0.44338111	1.71E-25	postive
CCS	AC091153.4	0.417784589	1.53E-22	postive
CCS	AC091271.1	0.445304138	1.00E-25	postive
CCS	AC091390.5	0.411571284	7.32E-22	postive
CCS	AC091488.1	0.351644297	5.34E-16	postive
CCS	AC091729.3	0.531026889	1.01E-37	postive
CCS	AC091887.1	0.311586226	1.02E-12	postive
CCS	AC091965.1	0.312031085	9.42E-13	postive
CCS	AC091965.4	0.317708031	3.45E-13	postive
CCS	AC091982.3	0.427704142	1.18E-23	postive
CCS	AC092115.4	0.369987909	1.15E-17	postive
CCS	AC092117.1	0.323880847	1.13E-13	postive
CCS	AC092118.2	0.547485356	1.92E-40	postive
CCS	AC092119.2	0.478565973	5.54E-30	postive
CCS	AC092119.3	0.332680185	2.19E-14	postive
CCS	AC092123.1	0.458939233	2.05E-27	postive
CCS	AC092134.1	0.358561463	1.29E-16	postive
CCS	AC092164.1	0.334922286	1.43E-14	postive
CCS	AC092171.2	0.39132867	9.58E-20	postive
CCS	AC092171.3	0.361184295	7.47E-17	postive
CCS	AC092171.4	0.45736249	3.24E-27	postive

CCS	AC092171.5	0.446301509	7.58E-26	postive
CCS	AC092287.1	0.357225463	1.70E-16	postive
CCS	AC092295.2	0.371815673	7.72E-18	postive
CCS	AC092301.1	0.354187256	3.18E-16	postive
CCS	AC092368.3	0.488292555	2.56E-31	postive
CCS	AC092611.1	0.355119538	2.63E-16	postive
CCS	AC092614.1	0.384436376	4.67E-19	postive
CCS	AC092683.1	0.336769153	1.00E-14	postive
CCS	AC092687.3	0.335051265	1.40E-14	postive
CCS	AC092718.2	0.339160695	6.33E-15	postive
CCS	AC092718.4	0.379921034	1.29E-18	postive
CCS	AC092718.5	0.322569569	1.43E-13	postive
CCS	AC092720.1	0.424005411	3.09E-23	postive
CCS	AC092803.1	0.453259605	1.06E-26	postive
CCS	AC092807.3	0.327338376	5.96E-14	postive
CCS	AC092809.5	0.492801154	5.94E-32	postive
CCS	AC092903.2	0.342644908	3.21E-15	postive
CCS	AC092910.3	0.411672818	7.13E-22	postive
CCS	AC093157.1	0.499692801	6.11E-33	postive
CCS	AC093227.1	0.385862296	3.38E-19	postive
CCS	AC093249.2	0.442346989	2.27E-25	postive
CCS	AC093249.6	0.540417061	2.94E-39	postive
CCS	AC093297.2	0.338403603	7.33E-15	postive
CCS	AC093382.1	0.350966662	6.12E-16	postive
CCS	AC093458.1	0.31210938	9.29E-13	postive
CCS	AC093462.1	0.40303628	5.95E-21	postive
CCS	AC093495.1	0.335174543	1.36E-14	postive
CCS	AC093525.4	0.301211178	6.03E-12	postive
CCS	AC093582.1	0.395293232	3.78E-20	postive
CCS	AC093620.1	0.413413624	4.62E-22	postive
CCS	AC093627.4	0.366409366	2.48E-17	postive
CCS	AC093627.5	0.421799748	5.47E-23	postive
CCS	AC093673.1	0.359506656	1.06E-16	postive
CCS	AC093726.1	0.433255162	2.70E-24	postive
CCS	AC093726.2	0.45137988	1.81E-26	postive
CCS	AC093752.1	0.430947539	4.99E-24	postive
CCS	AC093752.3	0.467083585	1.85E-28	postive
CCS	AC095057.3	0.428956965	8.46E-24	postive
CCS	AC096536.2	0.384257275	4.86E-19	postive
CCS	AC096540.1	0.454923744	6.56E-27	postive
CCS	AC096642.1	0.389815156	1.36E-19	postive
CCS	AC096649.2	0.330734378	3.16E-14	postive
CCS	AC096677.1	0.568255333	4.24E-44	postive
CCS	AC096719.1	0.321109452	1.87E-13	postive
CCS	AC096733.3	0.405814243	3.03E-21	postive
CCS	AC096772.6	0.376053818	3.05E-18	postive
CCS	AC096992.2	0.382227152	7.70E-19	postive
CCS	AC097358.2	0.372082892	7.29E-18	postive
CCS	AC097359.2	0.44665143	6.87E-26	postive
CCS	AC097376.3	0.340327884	5.05E-15	postive

CCS	AC097381.1	0.320944744	1.92E-13	postive
CCS	AC097468.3	0.507563803	4.28E-34	postive
CCS	AC097532.2	0.315825431	4.82E-13	postive
CCS	AC097639.1	0.376567343	2.72E-18	postive
CCS	AC097641.2	0.380615258	1.11E-18	postive
CCS	AC098484.1	0.300052022	7.33E-12	postive
CCS	AC098484.2	0.412885593	5.27E-22	postive
CCS	AC098487.1	0.418628231	1.23E-22	postive
CCS	AC098591.2	0.386475871	2.93E-19	postive
CCS	AC098820.2	0.477501715	7.71E-30	postive
CCS	AC098820.4	0.372424585	6.77E-18	postive
CCS	AC098934.1	0.368798055	1.48E-17	postive
CCS	AC099343.4	0.509084155	2.54E-34	postive
CCS	AC099518.4	0.351733197	5.24E-16	postive
CCS	AC099518.6	0.343808244	2.55E-15	postive
CCS	AC099522.2	0.413261796	4.80E-22	postive
CCS	AC099568.2	0.450027723	2.66E-26	postive
CCS	AC099778.1	0.597708978	9.47E-50	postive
CCS	AC099791.2	0.409612432	1.19E-21	postive
CCS	AC099811.3	0.364802362	3.49E-17	postive
CCS	AC099850.2	0.382978743	6.50E-19	postive
CCS	AC100778.2	0.387470775	2.34E-19	postive
CCS	AC100778.3	0.323277105	1.26E-13	postive
CCS	AC100786.1	0.337153256	9.33E-15	postive
CCS	AC100791.2	0.309056294	1.58E-12	postive
CCS	AC100793.2	0.312765924	8.28E-13	postive
CCS	AC100793.3	0.376365167	2.85E-18	postive
CCS	AC100797.1	0.304730318	3.33E-12	postive
CCS	AC100810.1	0.55962442	1.50E-42	postive
CCS	AC100812.1	0.342340129	3.41E-15	postive
CCS	AC100814.2	0.36805492	1.74E-17	postive
CCS	AC100861.1	0.402758495	6.36E-21	postive
CCS	AC102953.1	0.32746652	5.82E-14	postive
CCS	AC103691.1	0.397511857	2.24E-20	postive
CCS	AC103706.1	0.338497564	7.20E-15	postive
CCS	AC103760.1	0.313813802	6.88E-13	postive
CCS	AC103957.2	0.348022155	1.11E-15	postive
CCS	AC103974.1	0.341302369	4.17E-15	postive
CCS	AC104109.2	0.493584347	4.60E-32	postive
CCS	AC104113.1	0.391695901	8.79E-20	postive
CCS	AC104118.1	0.324997602	9.18E-14	postive
CCS	AC104187.1	0.397467912	2.26E-20	postive
CCS	AC104463.2	0.442744269	2.04E-25	postive
CCS	AC104521.1	0.384885976	4.22E-19	postive
CCS	AC104532.2	0.385038158	4.07E-19	postive
CCS	AC104561.1	0.308231628	1.82E-12	postive
CCS	AC104561.3	0.362283732	5.93E-17	postive
CCS	AC104564.3	0.482040673	1.87E-30	postive
CCS	AC104667.1	0.322270776	1.51E-13	postive
CCS	AC104667.2	0.416662072	2.04E-22	postive

CCS	AC104794.3	0.45161176	1.69E-26	postive
CCS	AC104825.1	0.406353092	2.65E-21	postive
CCS	AC104938.1	0.332685157	2.19E-14	postive
CCS	AC105020.1	0.357903285	1.48E-16	postive
CCS	AC105020.5	0.315560298	5.05E-13	postive
CCS	AC105020.6	0.419880044	8.97E-23	postive
CCS	AC105036.3	0.38565008	3.54E-19	postive
CCS	AC105137.2	0.475887943	1.27E-29	postive
CCS	AC105219.1	0.471263244	5.23E-29	postive
CCS	AC105219.4	0.343442975	2.74E-15	postive
CCS	AC105285.1	0.359803772	9.98E-17	postive
CCS	AC105345.1	0.432173329	3.60E-24	postive
CCS	AC105935.1	0.314280241	6.34E-13	postive
CCS	AC106028.3	0.376561216	2.73E-18	postive
CCS	AC106052.1	0.308272386	1.81E-12	postive
CCS	AC106738.1	0.394880997	4.17E-20	postive
CCS	AC106739.1	0.325868991	7.82E-14	postive
CCS	AC106772.1	0.309594566	1.44E-12	postive
CCS	AC106779.1	0.371252861	8.73E-18	postive
CCS	AC106782.5	0.49717337	1.41E-32	postive
CCS	AC106795.1	0.388263196	1.95E-19	postive
CCS	AC106820.3	0.459062926	1.98E-27	postive
CCS	AC106820.5	0.402415971	6.91E-21	postive
CCS	AC106886.2	0.372599376	6.51E-18	postive
CCS	AC106886.3	0.457772271	2.88E-27	postive
CCS	AC107032.2	0.382166995	7.81E-19	postive
CCS	AC107068.1	0.351258534	5.77E-16	postive
CCS	AC107081.2	0.444294982	1.33E-25	postive
CCS	AC107214.1	0.397087757	2.48E-20	postive
CCS	AC107214.2	0.34678823	1.42E-15	postive
CCS	AC107294.1	0.329265805	4.16E-14	postive
CCS	AC107294.2	0.346564495	1.48E-15	postive
CCS	AC107294.3	0.341896174	3.72E-15	postive
CCS	AC107375.1	0.531206123	9.41E-38	postive
CCS	AC107464.3	0.352840646	4.19E-16	postive
CCS	AC107884.2	0.316177607	4.53E-13	postive
CCS	AC108047.1	0.387127569	2.53E-19	postive
CCS	AC108134.1	0.462887813	6.43E-28	postive
CCS	AC108134.2	0.446420018	7.33E-26	postive
CCS	AC108471.3	0.410304756	1.00E-21	postive
CCS	AC108488.1	0.552809926	2.34E-41	postive
CCS	AC108673.2	0.323506535	1.21E-13	postive
CCS	AC108673.3	0.411936409	6.68E-22	postive
CCS	AC108860.2	0.465235661	3.20E-28	postive
CCS	AC109322.1	0.540560735	2.79E-39	postive
CCS	AC109347.1	0.484675354	8.11E-31	postive
CCS	AC109347.2	0.32046416	2.10E-13	postive
CCS	AC109449.1	0.357522291	1.60E-16	postive
CCS	AC109454.2	0.300404024	6.91E-12	postive
CCS	AC109460.1	0.513453672	5.58E-35	postive

CCS	AC109460.2	0.44722698	5.85E-26	postive
CCS	AC110079.1	0.468549753	1.19E-28	postive
CCS	AC110285.1	0.392394117	7.47E-20	postive
CCS	AC110285.2	0.36355791	4.54E-17	postive
CCS	AC110609.1	0.358613772	1.28E-16	postive
CCS	AC112220.2	0.351096619	5.96E-16	postive
CCS	AC112236.2	0.337514235	8.70E-15	postive
CCS	AC112491.1	0.365982142	2.71E-17	postive
CCS	AC112694.1	0.363610788	4.49E-17	postive
CCS	AC112907.3	0.318770397	2.85E-13	postive
CCS	AC113139.1	0.304560211	3.42E-12	postive
CCS	AC113189.4	0.408829181	1.44E-21	postive
CCS	AC114271.1	0.482600675	1.56E-30	postive
CCS	AC114341.1	0.341823123	3.77E-15	postive
CCS	AC114490.1	0.445056127	1.07E-25	postive
CCS	AC114684.1	0.327481978	5.80E-14	postive
CCS	AC114730.1	0.309352419	1.50E-12	postive
CCS	AC114730.2	0.450484367	2.33E-26	postive
CCS	AC114730.3	0.415364736	2.83E-22	postive
CCS	AC114810.1	0.311946485	9.56E-13	postive
CCS	AC114956.2	0.334277938	1.62E-14	postive
CCS	AC115618.1	0.558869737	2.05E-42	postive
CCS	AC115618.2	0.395279667	3.80E-20	postive
CCS	AC116036.2	0.381604138	8.86E-19	postive
CCS	AC116407.2	0.315040339	5.54E-13	postive
CCS	AC116447.1	0.317317898	3.69E-13	postive
CCS	AC116667.1	0.340171146	5.20E-15	postive
CCS	AC116913.1	0.49447696	3.43E-32	postive
CCS	AC116914.2	0.443878713	1.49E-25	postive
CCS	AC117503.1	0.355161281	2.61E-16	postive
CCS	AC118553.1	0.423429586	3.59E-23	postive
CCS	AC118755.2	0.391968841	8.25E-20	postive
CCS	AC118758.3	0.337060007	9.50E-15	postive
CCS	AC120024.1	0.307170824	2.19E-12	postive
CCS	AC120036.4	0.30897127	1.60E-12	postive
CCS	AC120053.1	0.538181341	6.89E-39	postive
CCS	AC120114.1	0.3070942	2.22E-12	postive
CCS	AC120498.9	0.394629413	4.42E-20	postive
CCS	AC121338.2	0.479200722	4.54E-30	postive
CCS	AC121493.1	0.336616656	1.03E-14	postive
CCS	AC121757.1	0.303697544	3.96E-12	postive
CCS	AC121761.2	0.362857011	5.26E-17	postive
CCS	AC123768.2	0.343100053	2.94E-15	postive
CCS	AC124016.2	0.508036259	3.64E-34	postive
CCS	AC124248.1	0.306053775	2.65E-12	postive
CCS	AC124283.2	0.31887032	2.80E-13	postive
CCS	AC124944.2	0.364324786	3.86E-17	postive
CCS	AC124944.3	0.320020221	2.27E-13	postive
CCS	AC125257.1	0.487627456	3.16E-31	postive
CCS	AC125494.1	0.448756016	3.80E-26	postive

CCS	AC126118.1	0.383317224	6.02E-19	postive
CCS	AC126283.1	0.372075597	7.30E-18	postive
CCS	AC126755.1	0.393763739	5.42E-20	postive
CCS	AC126773.4	0.309635667	1.43E-12	postive
CCS	AC127024.5	0.365254975	3.17E-17	postive
CCS	AC127024.6	0.447608117	5.25E-26	postive
CCS	AC127070.1	0.366135438	2.62E-17	postive
CCS	AC127502.2	0.377053129	2.45E-18	postive
CCS	AC127537.1	0.430639592	5.42E-24	postive
CCS	AC128687.2	0.338033226	7.87E-15	postive
CCS	AC129492.4	0.300332251	6.99E-12	postive
CCS	AC129510.1	0.392223821	7.77E-20	postive
CCS	AC130343.2	0.342901309	3.05E-15	postive
CCS	AC130371.2	0.3005236	6.77E-12	postive
CCS	AC130456.3	0.312072479	9.35E-13	postive
CCS	AC130462.3	0.403334089	5.54E-21	postive
CCS	AC131009.3	0.40595946	2.92E-21	postive
CCS	AC131159.2	0.338193157	7.63E-15	postive
CCS	AC131235.3	0.380889573	1.04E-18	postive
CCS	AC132008.2	0.420242872	8.17E-23	postive
CCS	AC132192.2	0.485930731	5.44E-31	postive
CCS	AC132872.1	0.491315198	9.63E-32	postive
CCS	AC132872.2	0.506944245	5.28E-34	postive
CCS	AC132872.3	0.495939356	2.12E-32	postive
CCS	AC132938.3	0.445468277	9.56E-26	postive
CCS	AC132938.4	0.502407341	2.46E-33	postive
CCS	AC133528.2	0.639843445	6.28E-59	postive
CCS	AC133550.3	0.377382607	2.27E-18	postive
CCS	AC133552.2	0.49384877	4.22E-32	postive
CCS	AC133552.5	0.424187712	2.95E-23	postive
CCS	AC134682.1	0.349027836	9.05E-16	postive
CCS	AC134878.2	0.395161841	3.90E-20	postive
CCS	AC135050.1	0.587656952	9.34E-48	postive
CCS	AC135050.3	0.363287572	4.80E-17	postive
CCS	AC135050.4	0.370662037	9.92E-18	postive
CCS	AC135050.5	0.453214284	1.07E-26	postive
CCS	AC135050.6	0.607509134	9.20E-52	postive
CCS	AC135178.4	0.468211944	1.32E-28	postive
CCS	AC135178.6	0.476597697	1.02E-29	postive
CCS	AC135507.1	0.329162932	4.24E-14	postive
CCS	AC136475.2	0.530038178	1.45E-37	postive
CCS	AC136475.3	0.348745976	9.57E-16	postive
CCS	AC136604.2	0.363379178	4.71E-17	postive
CCS	AC136604.3	0.446766777	6.65E-26	postive
CCS	AC137630.1	0.423418588	3.60E-23	postive
CCS	AC137630.2	0.419132987	1.09E-22	postive
CCS	AC137894.1	0.351768815	5.20E-16	postive
CCS	AC137932.1	0.391256718	9.74E-20	postive
CCS	AC137932.3	0.444125151	1.39E-25	postive
CCS	AC138028.2	0.321718894	1.67E-13	postive

CCS	AC138028.4	0.415836171	2.51E-22	postive
CCS	AC138028.6	0.345018393	2.01E-15	postive
CCS	AC138035.1	0.411619028	7.23E-22	postive
CCS	AC138150.2	0.366837826	2.26E-17	postive
CCS	AC138207.4	0.378229049	1.88E-18	postive
CCS	AC138207.8	0.403902538	4.82E-21	postive
CCS	AC138230.1	0.478754528	5.22E-30	postive
CCS	AC138696.2	0.548229328	1.43E-40	postive
CCS	AC138932.1	0.412417146	5.92E-22	postive
CCS	AC138956.1	0.341165182	4.29E-15	postive
CCS	AC138956.2	0.366074466	2.66E-17	postive
CCS	AC139100.2	0.338468336	7.24E-15	postive
CCS	AC139149.1	0.393803434	5.37E-20	postive
CCS	AC139256.2	0.397208655	2.41E-20	postive
CCS	AC139256.3	0.501265403	3.61E-33	postive
CCS	AC139530.1	0.589007117	5.09E-48	postive
CCS	AC139768.1	0.529169639	2.00E-37	postive
CCS	AC139795.2	0.399172143	1.51E-20	postive
CCS	AC139795.3	0.316354914	4.39E-13	postive
CCS	AC139887.1	0.412598323	5.66E-22	postive
CCS	AC139887.2	0.479470869	4.18E-30	postive
CCS	AC141002.1	0.360814362	8.08E-17	postive
CCS	AC141586.1	0.375491404	3.45E-18	postive
CCS	AC142381.3	0.318256637	3.12E-13	postive
CCS	AC142472.1	0.542178993	1.50E-39	postive
CCS	AC144652.1	0.344125245	2.40E-15	postive
CCS	AC145098.1	0.319344613	2.57E-13	postive
CCS	AC145207.1	0.367237689	2.07E-17	postive
CCS	AC145207.5	0.328904958	4.45E-14	postive
CCS	AC145285.2	0.44924859	3.31E-26	postive
CCS	AC145285.6	0.506661844	5.82E-34	postive
CCS	AC145343.1	0.30856327	1.72E-12	postive
CCS	AC145423.2	0.475435249	1.46E-29	postive
CCS	AC145423.3	0.486093971	5.17E-31	postive
CCS	AC147067.1	0.362788493	5.34E-17	postive
CCS	AC147651.1	0.314281062	6.34E-13	postive
CCS	AC174065.1	0.35397858	3.32E-16	postive
CCS	AC211433.2	0.424035496	3.07E-23	postive
CCS	AC231533.2	0.373757172	5.06E-18	postive
CCS	AC231981.1	0.397363598	2.32E-20	postive
CCS	AC232271.1	0.54544198	4.25E-40	postive
CCS	AC233728.1	0.511909104	9.55E-35	postive
CCS	AC233992.3	0.397893117	2.04E-20	postive
CCS	AC234582.1	0.409965012	1.09E-21	postive
CCS	AC239803.1	0.316599327	4.20E-13	postive
CCS	AC242376.2	0.322298902	1.50E-13	postive
CCS	AC242426.2	0.341859243	3.74E-15	postive
CCS	AC242842.1	0.346973835	1.36E-15	postive
CCS	AC243919.2	0.371698039	7.92E-18	postive
CCS	AC244090.1	0.492212647	7.19E-32	postive

CCS	AC244090.3	0.302928954	4.52E-12	postive
CCS	AC244197.2	0.364993352	3.35E-17	postive
CCS	AC244669.1	0.351642589	5.34E-16	postive
CCS	AC245052.4	0.536339684	1.38E-38	postive
CCS	AC245060.2	0.376701475	2.65E-18	postive
CCS	AC245060.5	0.318479274	3.00E-13	postive
CCS	AC245060.6	0.430914821	5.03E-24	postive
CCS	AC245100.1	0.30826569	1.81E-12	postive
CCS	AC245140.1	0.384815786	4.28E-19	postive
CCS	AC245140.2	0.565897405	1.14E-43	postive
CCS	AC245452.1	0.437515711	8.54E-25	postive
CCS	AC245884.8	0.358007808	1.45E-16	postive
CCS	AC253536.6	0.437647482	8.24E-25	postive
CCS	AC254562.2	0.317881775	3.34E-13	postive
CCS	AC254562.3	0.407646209	1.93E-21	postive
CCS	ACTG1P17	0.460016949	1.50E-27	postive
CCS	ACTN1-AS1	0.320885545	1.94E-13	postive
CCS	ACVR2B-AS1	0.438504326	6.53E-25	postive
CCS	AD000671.3	0.404952553	3.74E-21	postive
CCS	AD001527.1	0.381586092	8.90E-19	postive
CCS	ADCY10P1	0.317911458	3.32E-13	postive
CCS	ADCY6-DT	0.341024933	4.41E-15	postive
CCS	ADNP-AS1	0.430812227	5.17E-24	postive
CCS	AF001548.2	0.331278583	2.85E-14	postive
CCS	AF064858.11	0.323602602	1.19E-13	postive
CCS	AF111167.2	0.312711398	8.36E-13	postive
CCS	AF111169.3	0.328864075	4.48E-14	postive
CCS	AF129075.3	0.360420566	8.77E-17	postive
CCS	AF131216.4	0.45146007	1.77E-26	postive
CCS	AF196972.1	0.371053472	9.11E-18	postive
CCS	AF213884.3	0.342638526	3.21E-15	postive
CCS	AFDN-DT	0.390878409	1.06E-19	postive
CCS	AFG3L1P	0.596902254	1.38E-49	postive
CCS	AGBL5-IT1	0.41443301	3.57E-22	postive
CCS	AHSA2P	0.541619022	1.86E-39	postive
CCS	AL008582.1	0.472462541	3.63E-29	postive
CCS	AL008635.1	0.346855065	1.40E-15	postive
CCS	AL008718.2	0.416045404	2.38E-22	postive
CCS	AL008729.1	0.424473429	2.74E-23	postive
CCS	AL009179.1	0.416074482	2.36E-22	postive
CCS	AL020996.1	0.550435229	6.01E-41	postive
CCS	AL021328.1	0.300392153	6.92E-12	postive
CCS	AL021392.1	0.434460405	1.95E-24	postive
CCS	AL021707.1	0.395285911	3.79E-20	postive
CCS	AL021707.2	0.330453672	3.33E-14	postive
CCS	AL021707.3	0.438070209	7.35E-25	postive
CCS	AL021707.4	0.317178833	3.79E-13	postive
CCS	AL021707.5	0.304691314	3.35E-12	postive
CCS	AL021707.6	0.471024763	5.62E-29	postive
CCS	AL021707.7	0.324390273	1.03E-13	postive

CCS	AL021707.8	0.34972294	7.87E-16	postive
CCS	AL021878.2	0.429387578	7.55E-24	postive
CCS	AL022069.1	0.419593823	9.65E-23	postive
CCS	AL022157.1	0.405575023	3.21E-21	postive
CCS	AL022238.3	0.393743099	5.45E-20	postive
CCS	AL022311.1	0.39458707	4.47E-20	postive
CCS	AL022322.1	0.358070337	1.43E-16	postive
CCS	AL022322.2	0.306335982	2.53E-12	postive
CCS	AL022324.3	0.314165519	6.47E-13	postive
CCS	AL022328.1	0.450617433	2.25E-26	postive
CCS	AL022328.2	0.528022104	3.04E-37	postive
CCS	AL022328.3	0.467274136	1.74E-28	postive
CCS	AL022328.4	0.439968546	4.37E-25	postive
CCS	AL022341.1	0.441523738	2.85E-25	postive
CCS	AL022341.2	0.401071954	9.56E-21	postive
CCS	AL022476.1	0.388277133	1.94E-19	postive
CCS	AL023284.4	0.432468734	3.33E-24	postive
CCS	AL023583.1	0.309925666	1.36E-12	postive
CCS	AL023653.1	0.332797821	2.14E-14	postive
CCS	AL023803.1	0.348487605	1.01E-15	postive
CCS	AL023803.2	0.343802866	2.56E-15	postive
CCS	AL023881.1	0.371603666	8.09E-18	postive
CCS	AL024498.1	0.416337302	2.21E-22	postive
CCS	AL024507.3	0.42685686	1.47E-23	postive
CCS	AL024508.1	0.31918096	2.64E-13	postive
CCS	AL031008.1	0.312836569	8.18E-13	postive
CCS	AL031055.1	0.365717079	2.87E-17	postive
CCS	AL031123.2	0.380526651	1.13E-18	postive
CCS	AL031186.1	0.495625066	2.35E-32	postive
CCS	AL031282.1	0.424088134	3.03E-23	postive
CCS	AL031282.2	0.427022024	1.41E-23	postive
CCS	AL031289.1	0.405414257	3.34E-21	postive
CCS	AL031432.3	0.34476153	2.12E-15	postive
CCS	AL031600.1	0.442080223	2.45E-25	postive
CCS	AL031673.1	0.386558243	2.88E-19	postive
CCS	AL031705.1	0.416001829	2.41E-22	postive
CCS	AL031709.1	0.461979143	8.41E-28	postive
CCS	AL031714.1	0.490491886	1.26E-31	postive
CCS	AL031717.1	0.374795368	4.03E-18	postive
CCS	AL031722.1	0.311649259	1.01E-12	postive
CCS	AL031775.1	0.473552559	2.60E-29	postive
CCS	AL031775.2	0.392992882	6.50E-20	postive
CCS	AL031846.2	0.360473398	8.67E-17	postive
CCS	AL031847.1	0.416070819	2.36E-22	postive
CCS	AL031963.2	0.379951455	1.28E-18	postive
CCS	AL031963.3	0.402228046	7.23E-21	postive
CCS	AL031985.3	0.320595806	2.05E-13	postive
CCS	AL032819.1	0.333183125	1.99E-14	postive
CCS	AL033384.2	0.438108794	7.27E-25	postive
CCS	AL033527.3	0.452835566	1.19E-26	postive

CCS	AL033528.3	0.309451143	1.48E-12	postive
CCS	AL034550.1	0.319238723	2.62E-13	postive
CCS	AL035071.1	0.46925694	9.60E-29	postive
CCS	AL035252.4	0.342826608	3.10E-15	postive
CCS	AL035461.3	0.38983439	1.35E-19	postive
CCS	AL035587.1	0.416010244	2.40E-22	postive
CCS	AL049597.2	0.411526259	7.40E-22	postive
CCS	AL049646.2	0.365348621	3.10E-17	postive
CCS	AL049780.1	0.380199947	1.21E-18	postive
CCS	AL049794.1	0.323625542	1.18E-13	postive
CCS	AL049795.1	0.489618045	1.67E-31	postive
CCS	AL049796.1	0.412922015	5.22E-22	postive
CCS	AL049840.4	0.365525224	2.99E-17	postive
CCS	AL049840.5	0.376472603	2.78E-18	postive
CCS	AL049840.6	0.510335728	1.65E-34	postive
CCS	AL050309.1	0.544349475	6.49E-40	postive
CCS	AL050341.2	0.405104981	3.60E-21	postive
CCS	AL078587.1	0.397706057	2.14E-20	postive
CCS	AL078644.2	0.348107994	1.09E-15	postive
CCS	AL080312.2	0.346409753	1.53E-15	postive
CCS	AL096701.3	0.399022734	1.56E-20	postive
CCS	AL096803.2	0.310671367	1.19E-12	postive
CCS	AL096828.3	0.471036875	5.60E-29	postive
CCS	AL096865.1	0.319140746	2.66E-13	postive
CCS	AL109659.2	0.35106782	6.00E-16	postive
CCS	AL109811.1	0.507569908	4.27E-34	postive
CCS	AL109811.2	0.430213119	6.07E-24	postive
CCS	AL109917.1	0.501883595	2.94E-33	postive
CCS	AL109936.3	0.356424927	2.01E-16	postive
CCS	AL109945.1	0.367975142	1.77E-17	postive
CCS	AL109955.1	0.360056985	9.46E-17	postive
CCS	AL110115.2	0.485062138	7.18E-31	postive
CCS	AL117209.1	0.46476241	3.69E-28	postive
CCS	AL117332.1	0.490244973	1.36E-31	postive
CCS	AL117336.2	0.483252798	1.27E-30	postive
CCS	AL117350.2	0.340110985	5.26E-15	postive
CCS	AL117379.1	0.439853085	4.52E-25	postive
CCS	AL118506.1	0.449970953	2.70E-26	postive
CCS	AL118511.1	0.456560375	4.09E-27	postive
CCS	AL118516.1	0.41776679	1.54E-22	postive
CCS	AL118558.1	0.303822996	3.88E-12	postive
CCS	AL118558.3	0.567570727	5.65E-44	postive
CCS	AL118558.4	0.504749211	1.12E-33	postive
CCS	AL121574.1	0.349671975	7.95E-16	postive
CCS	AL121583.1	0.441043458	3.26E-25	postive
CCS	AL121601.1	0.417269537	1.74E-22	postive
CCS	AL121603.2	0.385722973	3.48E-19	postive
CCS	AL121655.1	0.308951355	1.61E-12	postive
CCS	AL121658.1	0.362468054	5.71E-17	postive
CCS	AL121782.1	0.300304695	7.03E-12	postive

CCS	AL121820.2	0.301073939	6.18E-12	postive
CCS	AL121829.2	0.353333326	3.79E-16	postive
CCS	AL121832.2	0.436226236	1.21E-24	postive
CCS	AL121832.3	0.363351472	4.74E-17	postive
CCS	AL121845.4	0.31103726	1.12E-12	postive
CCS	AL121852.1	0.379979898	1.28E-18	postive
CCS	AL121890.5	0.300398845	6.92E-12	postive
CCS	AL121894.2	0.304042302	3.74E-12	postive
CCS	AL121906.1	0.308893405	1.63E-12	postive
CCS	AL121906.2	0.405658074	3.15E-21	postive
CCS	AL121917.1	0.393077141	6.37E-20	postive
CCS	AL121917.2	0.358944013	1.19E-16	postive
CCS	AL121944.2	0.500565425	4.57E-33	postive
CCS	AL121987.2	0.387967178	2.08E-19	postive
CCS	AL121992.1	0.429633436	7.07E-24	postive
CCS	AL122010.1	0.497750133	1.17E-32	postive
CCS	AL122023.3	0.435105376	1.64E-24	postive
CCS	AL122035.2	0.445825869	8.66E-26	postive
CCS	AL132639.2	0.375712143	3.29E-18	postive
CCS	AL132639.3	0.333561725	1.85E-14	postive
CCS	AL132657.1	0.347251253	1.29E-15	postive
CCS	AL132712.1	0.528530057	2.52E-37	postive
CCS	AL132765.2	0.392170769	7.87E-20	postive
CCS	AL132780.1	0.445860338	8.57E-26	postive
CCS	AL132780.2	0.450494637	2.33E-26	postive
CCS	AL132800.1	0.307710093	2.00E-12	postive
CCS	AL132857.1	0.320231502	2.19E-13	postive
CCS	AL132989.1	0.337753614	8.31E-15	postive
CCS	AL133153.2	0.347869348	1.14E-15	postive
CCS	AL133215.1	0.300235846	7.11E-12	postive
CCS	AL133215.2	0.427462251	1.25E-23	postive
CCS	AL133215.3	0.328260609	5.02E-14	postive
CCS	AL133216.2	0.532461902	5.90E-38	postive
CCS	AL133338.1	0.410812214	8.84E-22	postive
CCS	AL133353.1	0.353845917	3.41E-16	postive
CCS	AL133367.1	0.469846406	8.03E-29	postive
CCS	AL133406.2	0.373475086	5.38E-18	postive
CCS	AL133410.1	0.365444348	3.04E-17	postive
CCS	AL133520.1	0.403547465	5.26E-21	postive
CCS	AL133551.1	0.386324063	3.04E-19	postive
CCS	AL135744.1	0.312730339	8.33E-13	postive
CCS	AL135791.1	0.423335987	3.68E-23	postive
CCS	AL135910.1	0.359189007	1.13E-16	postive
CCS	AL135925.1	0.485487422	6.27E-31	postive
CCS	AL135999.1	0.50967442	2.07E-34	postive
CCS	AL135999.2	0.379757668	1.34E-18	postive
CCS	AL136038.7	0.341293277	4.18E-15	postive
CCS	AL136040.1	0.436191554	1.22E-24	postive
CCS	AL136162.1	0.324313141	1.04E-13	postive
CCS	AL136295.2	0.457520148	3.10E-27	postive

CCS	AL136295.6	0.4806094	2.93E-30	postive
CCS	AL136295.7	0.57001981	2.02E-44	postive
CCS	AL136304.1	0.489033212	2.01E-31	postive
CCS	AL136307.1	0.317920929	3.32E-13	postive
CCS	AL136368.1	0.35176012	5.21E-16	postive
CCS	AL136531.1	0.422518799	4.55E-23	postive
CCS	AL137003.1	0.336839304	9.91E-15	postive
CCS	AL137127.1	0.517793659	1.21E-35	postive
CCS	AL137186.2	0.428290978	1.01E-23	postive
CCS	AL137244.1	0.345414818	1.86E-15	postive
CCS	AL137802.2	0.364478621	3.73E-17	postive
CCS	AL138724.2	0.305230041	3.05E-12	postive
CCS	AL138756.1	0.334195642	1.64E-14	postive
CCS	AL138762.2	0.402688242	6.47E-21	postive
CCS	AL138831.1	0.351573856	5.41E-16	postive
CCS	AL138831.2	0.332483245	2.27E-14	postive
CCS	AL138921.1	0.467394306	1.68E-28	postive
CCS	AL138921.2	0.357473829	1.62E-16	postive
CCS	AL138966.2	0.325515601	8.34E-14	postive
CCS	AL139011.1	0.468884275	1.07E-28	postive
CCS	AL139021.2	0.335410989	1.30E-14	postive
CCS	AL139089.1	0.424589343	2.66E-23	postive
CCS	AL139099.3	0.379421791	1.45E-18	postive
CCS	AL139123.1	0.464041842	4.57E-28	postive
CCS	AL139246.5	0.366479147	2.44E-17	postive
CCS	AL139260.1	0.332158694	2.42E-14	postive
CCS	AL139286.2	0.333555008	1.85E-14	postive
CCS	AL139287.1	0.556402282	5.55E-42	postive
CCS	AL139289.1	0.331755155	2.61E-14	postive
CCS	AL139349.1	0.378376869	1.82E-18	postive
CCS	AL139383.1	0.312655149	8.44E-13	postive
CCS	AL139384.1	0.438063815	7.36E-25	postive
CCS	AL139397.1	0.362767224	5.36E-17	postive
CCS	AL139423.1	0.346935405	1.37E-15	postive
CCS	AL157373.2	0.311955721	9.54E-13	postive
CCS	AL157392.3	0.386589978	2.86E-19	postive
CCS	AL157392.4	0.310951945	1.14E-12	postive
CCS	AL157700.1	0.350539687	6.67E-16	postive
CCS	AL157756.1	0.4475008	5.41E-26	postive
CCS	AL157813.1	0.316470677	4.30E-13	postive
CCS	AL157832.1	0.358102516	1.42E-16	postive
CCS	AL157904.1	0.381183648	9.74E-19	postive
CCS	AL157935.3	0.384349977	4.76E-19	postive
CCS	AL158071.3	0.3094293	1.48E-12	postive
CCS	AL158151.4	0.363507223	4.59E-17	postive
CCS	AL158196.1	0.326244022	7.29E-14	postive
CCS	AL158212.2	0.4329629	2.92E-24	postive
CCS	AL158212.3	0.374867143	3.96E-18	postive
CCS	AL158834.2	0.30842958	1.76E-12	postive
CCS	AL158835.2	0.395210883	3.86E-20	postive

CCS	AL160006.1	0.5000927	5.35E-33	postive
CCS	AL160291.1	0.357105201	1.75E-16	postive
CCS	AL160313.2	0.43282269	3.03E-24	postive
CCS	AL161421.1	0.30017413	7.18E-12	postive
CCS	AL161452.1	0.532107374	6.74E-38	postive
CCS	AL161668.3	0.495571031	2.40E-32	postive
CCS	AL161725.2	0.316588861	4.21E-13	postive
CCS	AL161729.1	0.335102053	1.38E-14	postive
CCS	AL161729.3	0.427309321	1.31E-23	postive
CCS	AL161729.4	0.324959873	9.24E-14	postive
CCS	AL161756.1	0.332264697	2.37E-14	postive
CCS	AL162258.2	0.446381884	7.41E-26	postive
CCS	AL162274.2	0.476983332	9.05E-30	postive
CCS	AL162377.1	0.447615974	5.24E-26	postive
CCS	AL162431.1	0.44476245	1.16E-25	postive
CCS	AL162586.1	0.376928349	2.52E-18	postive
CCS	AL162595.1	0.389807659	1.36E-19	postive
CCS	AL162741.1	0.508642047	2.95E-34	postive
CCS	AL163051.1	0.365279099	3.15E-17	postive
CCS	AL353194.1	0.473304763	2.81E-29	postive
CCS	AL353593.1	0.339401399	6.04E-15	postive
CCS	AL353622.1	0.440815412	3.47E-25	postive
CCS	AL353622.2	0.390707303	1.11E-19	postive
CCS	AL353625.1	0.337152021	9.33E-15	postive
CCS	AL353708.1	0.437453455	8.69E-25	postive
CCS	AL353708.3	0.4859504	5.41E-31	postive
CCS	AL353719.1	0.330539595	3.28E-14	postive
CCS	AL353743.1	0.41109273	8.24E-22	postive
CCS	AL353795.2	0.300814247	6.45E-12	postive
CCS	AL353796.1	0.332944131	2.08E-14	postive
CCS	AL353801.3	0.366967175	2.20E-17	postive
CCS	AL353807.2	0.379120929	1.55E-18	postive
CCS	AL353997.2	0.39828418	1.86E-20	postive
CCS	AL354696.1	0.389004794	1.64E-19	postive
CCS	AL354696.2	0.330353556	3.39E-14	postive
CCS	AL354718.3	0.332502274	2.26E-14	postive
CCS	AL354733.3	0.353250298	3.85E-16	postive
CCS	AL354740.1	0.307807011	1.96E-12	postive
CCS	AL354760.1	0.458953004	2.04E-27	postive
CCS	AL354836.1	0.441687699	2.73E-25	postive
CCS	AL354892.2	0.523507565	1.57E-36	postive
CCS	AL354892.3	0.440757293	3.52E-25	postive
CCS	AL354920.1	0.441139907	3.17E-25	postive
CCS	AL354928.1	0.33321766	1.98E-14	postive
CCS	AL355001.2	0.482898928	1.42E-30	postive
CCS	AL355075.2	0.356707181	1.90E-16	postive
CCS	AL355297.3	0.34345773	2.74E-15	postive
CCS	AL355312.3	0.300741822	6.53E-12	postive
CCS	AL355353.2	0.51874179	8.66E-36	postive
CCS	AL355385.1	0.492004945	7.70E-32	postive

CCS	AL355388.1	0.452104844	1.47E-26	postive
CCS	AL355488.1	0.3506549	6.52E-16	postive
CCS	AL355490.2	0.33809546	7.78E-15	postive
CCS	AL355490.3	0.333260389	1.96E-14	postive
CCS	AL355574.1	0.450078416	2.62E-26	postive
CCS	AL355802.3	0.485619484	6.01E-31	postive
CCS	AL356019.2	0.32051739	2.08E-13	postive
CCS	AL356299.3	0.478123676	6.35E-30	postive
CCS	AL356481.1	0.440091208	4.23E-25	postive
CCS	AL356481.3	0.429692642	6.96E-24	postive
CCS	AL356488.3	0.441089944	3.22E-25	postive
CCS	AL356512.1	0.429976152	6.46E-24	postive
CCS	AL357033.4	0.379992869	1.27E-18	postive
CCS	AL357079.1	0.475152322	1.59E-29	postive
CCS	AL357140.2	0.394952613	4.10E-20	postive
CCS	AL357874.1	0.360322442	8.95E-17	postive
CCS	AL358072.1	0.320170648	2.21E-13	postive
CCS	AL358075.2	0.386595419	2.85E-19	postive
CCS	AL358472.2	0.507816726	3.92E-34	postive
CCS	AL358472.3	0.497899551	1.11E-32	postive
CCS	AL358781.2	0.392897938	6.64E-20	postive
CCS	AL358852.1	0.429288467	7.75E-24	postive
CCS	AL359091.3	0.445992668	8.26E-26	postive
CCS	AL359091.4	0.403952155	4.77E-21	postive
CCS	AL359198.1	0.379462644	1.43E-18	postive
CCS	AL359317.1	0.319935416	2.31E-13	postive
CCS	AL359397.1	0.306685007	2.38E-12	postive
CCS	AL359504.2	0.360696526	8.28E-17	postive
CCS	AL359513.1	0.337106194	9.41E-15	postive
CCS	AL359532.1	0.401801333	8.02E-21	postive
CCS	AL359643.3	0.391962029	8.26E-20	postive
CCS	AL359711.2	0.409146944	1.34E-21	postive
CCS	AL359715.1	0.431098344	4.79E-24	postive
CCS	AL359715.2	0.302024535	5.26E-12	postive
CCS	AL359715.3	0.334283191	1.62E-14	postive
CCS	AL359878.1	0.370477459	1.03E-17	postive
CCS	AL359878.2	0.336330519	1.09E-14	postive
CCS	AL359881.2	0.302226685	5.09E-12	postive
CCS	AL359921.1	0.448311848	4.31E-26	postive
CCS	AL359921.2	0.573966837	3.77E-45	postive
CCS	AL360181.2	0.455128867	6.18E-27	postive
CCS	AL360270.3	0.395326276	3.75E-20	postive
CCS	AL365330.1	0.575704352	1.79E-45	postive
CCS	AL390198.1	0.307870654	1.94E-12	postive
CCS	AL390719.1	0.41199058	6.59E-22	postive
CCS	AL390719.2	0.466325103	2.32E-28	postive
CCS	AL390728.4	0.431207138	4.66E-24	postive
CCS	AL390728.6	0.403843489	4.89E-21	postive
CCS	AL390879.1	0.446178556	7.84E-26	postive
CCS	AL391069.2	0.433581118	2.47E-24	postive

CCS	AL391069.3	0.355223056	2.57E-16	postive
CCS	AL391095.2	0.315365879	5.23E-13	postive
CCS	AL391121.1	0.304728351	3.33E-12	postive
CCS	AL391244.1	0.440655397	3.62E-25	postive
CCS	AL391244.2	0.50979099	1.99E-34	postive
CCS	AL391684.1	0.427765245	1.16E-23	postive
CCS	AL391825.1	0.306940833	2.28E-12	postive
CCS	AL391832.2	0.365277706	3.15E-17	postive
CCS	AL391834.2	0.382179105	7.78E-19	postive
CCS	AL391988.1	0.315064656	5.52E-13	postive
CCS	AL392172.1	0.45586546	5.00E-27	postive
CCS	AL441992.2	0.469065268	1.02E-28	postive
CCS	AL442128.2	0.335041016	1.40E-14	postive
CCS	AL442663.3	0.403807166	4.94E-21	postive
CCS	AL445222.2	0.459633404	1.67E-27	postive
CCS	AL445228.3	0.371843022	7.68E-18	postive
CCS	AL445231.1	0.376724225	2.63E-18	postive
CCS	AL445645.1	0.455139883	6.17E-27	postive
CCS	AL450326.1	0.341921977	3.70E-15	postive
CCS	AL450384.2	0.446946612	6.33E-26	postive
CCS	AL450998.2	0.526486333	5.33E-37	postive
CCS	AL450998.3	0.34537609	1.87E-15	postive
CCS	AL451042.2	0.310732048	1.18E-12	postive
CCS	AL451050.2	0.495081857	2.81E-32	postive
CCS	AL451085.2	0.445293478	1.00E-25	postive
CCS	AL451085.3	0.573102439	5.45E-45	postive
CCS	AL451165.2	0.486233464	4.94E-31	postive
CCS	AL512343.2	0.310234999	1.29E-12	postive
CCS	AL512408.1	0.492811174	5.92E-32	postive
CCS	AL512598.1	0.315392621	5.21E-13	postive
CCS	AL512625.1	0.329787506	3.77E-14	postive
CCS	AL512652.1	0.395561652	3.55E-20	postive
CCS	AL512770.1	0.494065161	3.93E-32	postive
CCS	AL512791.1	0.340433957	4.94E-15	postive
CCS	AL512791.2	0.318504297	2.99E-13	postive
CCS	AL513165.1	0.350730866	6.42E-16	postive
CCS	AL513190.1	0.349670068	7.95E-16	postive
CCS	AL513217.1	0.30897307	1.60E-12	postive
CCS	AL513218.1	0.465911256	2.62E-28	postive
CCS	AL513320.1	0.556437189	5.47E-42	postive
CCS	AL513477.1	0.3219681	1.60E-13	postive
CCS	AL513477.2	0.418787078	1.19E-22	postive
CCS	AL513550.1	0.362669125	5.47E-17	postive
CCS	AL583810.1	0.46321356	5.84E-28	postive
CCS	AL583810.2	0.332082209	2.45E-14	postive
CCS	AL589765.4	0.467629097	1.57E-28	postive
CCS	AL589765.5	0.307026348	2.25E-12	postive
CCS	AL589765.6	0.418098092	1.41E-22	postive
CCS	AL589843.1	0.321545952	1.72E-13	postive
CCS	AL589843.2	0.327884025	5.38E-14	postive

CCS	AL590096.1	0.460717973	1.22E-27	postive
CCS	AL590133.1	0.44929321	3.27E-26	postive
CCS	AL590369.1	0.426943088	1.44E-23	postive
CCS	AL590560.3	0.381679703	8.71E-19	postive
CCS	AL590617.2	0.501191821	3.70E-33	postive
CCS	AL590652.1	0.36243666	5.75E-17	postive
CCS	AL590708.1	0.359017006	1.18E-16	postive
CCS	AL590729.1	0.337632284	8.51E-15	postive
CCS	AL590822.1	0.330459284	3.33E-14	postive
CCS	AL591848.3	0.447438182	5.51E-26	postive
CCS	AL591895.1	0.471467405	4.91E-29	postive
CCS	AL592071.1	0.378602921	1.73E-18	postive
CCS	AL592148.3	0.328361439	4.92E-14	postive
CCS	AL592166.1	0.459765665	1.61E-27	postive
CCS	AL592211.1	0.489037066	2.01E-31	postive
CCS	AL592435.1	0.411038243	8.35E-22	postive
CCS	AL592494.2	0.310431246	1.25E-12	postive
CCS	AL596094.1	0.501022235	3.92E-33	postive
CCS	AL596202.1	0.376113797	3.01E-18	postive
CCS	AL596442.2	0.30648296	2.46E-12	postive
CCS	AL603832.1	0.403736971	5.02E-21	postive
CCS	AL603839.2	0.415881854	2.48E-22	postive
CCS	AL603839.3	0.414688281	3.35E-22	postive
CCS	AL604028.1	0.488291005	2.56E-31	postive
CCS	AL606491.1	0.434840513	1.76E-24	postive
CCS	AL606760.1	0.441437599	2.92E-25	postive
CCS	AL606760.2	0.502867891	2.11E-33	postive
CCS	AL606807.1	0.314109761	6.53E-13	postive
CCS	AL606834.1	0.307112928	2.21E-12	postive
CCS	AL606834.2	0.313142526	7.75E-13	postive
CCS	AL627171.3	0.346491275	1.50E-15	postive
CCS	AL627309.2	0.309404824	1.49E-12	postive
CCS	AL645568.1	0.477244881	8.34E-30	postive
CCS	AL645608.1	0.338471411	7.23E-15	postive
CCS	AL645728.1	0.377896255	2.03E-18	postive
CCS	AL645933.3	0.341689952	3.87E-15	postive
CCS	AL645940.1	0.385214021	3.91E-19	postive
CCS	AL645941.1	0.392257889	7.71E-20	postive
CCS	AL662791.1	0.303169572	4.34E-12	postive
CCS	AL662797.2	0.426380545	1.67E-23	postive
CCS	AL662844.4	0.421774813	5.51E-23	postive
CCS	AL662884.1	0.327182695	6.13E-14	postive
CCS	AL669831.1	0.354849904	2.78E-16	postive
CCS	AL671710.1	0.633830113	1.57E-57	postive
CCS	AL672291.1	0.310455417	1.24E-12	postive
CCS	AL683813.2	0.352597717	4.40E-16	postive
CCS	AL691403.2	0.313272653	7.57E-13	postive
CCS	AL691432.1	0.397885712	2.05E-20	postive
CCS	AL691432.2	0.445485232	9.52E-26	postive
CCS	AL691482.4	0.446385236	7.40E-26	postive

CCS	AL731533.2	0.401701238	8.22E-21	postive
CCS	AL731563.4	0.356094456	2.15E-16	postive
CCS	AL731566.2	0.339323697	6.13E-15	postive
CCS	AL731569.1	0.528127154	2.93E-37	postive
CCS	AL731571.1	0.472080811	4.08E-29	postive
CCS	AL731577.2	0.302638354	4.74E-12	postive
CCS	AL732314.6	0.343454151	2.74E-15	postive
CCS	AL732314.8	0.454317681	7.81E-27	postive
CCS	AL732372.2	0.468803728	1.10E-28	postive
CCS	AL844908.2	0.335283698	1.33E-14	postive
CCS	AL928654.1	0.375916263	3.15E-18	postive
CCS	AL928654.2	0.494639116	3.25E-32	postive
CCS	ALG1L13P	0.336832762	9.92E-15	postive
CCS	ALOX12-AS1	0.557305671	3.86E-42	postive
CCS	ALOX12P2	0.386540501	2.89E-19	postive
CCS	ALOX15P1	0.327045788	6.29E-14	postive
CCS	AMZ2P1	0.494124732	3.85E-32	postive
CCS	ANKRD19P	0.387147996	2.52E-19	postive
CCS	ANO7L1	0.549703449	8.02E-41	postive
CCS	AP000238.1	0.314216035	6.41E-13	postive
CCS	AP000253.1	0.307049111	2.24E-12	postive
CCS	AP000254.2	0.506423772	6.31E-34	postive
CCS	AP000255.1	0.368687445	1.52E-17	postive
CCS	AP000295.10	0.36950376	1.27E-17	postive
CCS	AP000320.1	0.323514326	1.20E-13	postive
CCS	AP000346.1	0.320808338	1.97E-13	postive
CCS	AP000346.2	0.461117116	1.08E-27	postive
CCS	AP000347.1	0.391837815	8.51E-20	postive
CCS	AP000350.5	0.324189979	1.06E-13	postive
CCS	AP000350.7	0.304854897	3.26E-12	postive
CCS	AP000439.1	0.377249963	2.34E-18	postive
CCS	AP000442.1	0.579432082	3.55E-46	postive
CCS	AP000487.1	0.514031954	4.56E-35	postive
CCS	AP000525.1	0.313116124	7.78E-13	postive
CCS	AP000553.2	0.371719555	7.89E-18	postive
CCS	AP000692.1	0.383220533	6.15E-19	postive
CCS	AP000692.2	0.34665724	1.45E-15	postive
CCS	AP000704.2	0.399310006	1.46E-20	postive
CCS	AP000759.1	0.413835643	4.15E-22	postive
CCS	AP000781.1	0.549375513	9.12E-41	postive
CCS	AP000787.1	0.317616867	3.50E-13	postive
CCS	AP000808.1	0.369145052	1.38E-17	postive
CCS	AP000845.1	0.366923829	2.22E-17	postive
CCS	AP000866.1	0.336321626	1.09E-14	postive
CCS	AP000866.5	0.303507806	4.09E-12	postive
CCS	AP000873.2	0.502371382	2.49E-33	postive
CCS	AP000873.4	0.393653018	5.57E-20	postive
CCS	AP000892.3	0.30819323	1.84E-12	postive
CCS	AP000894.4	0.383304926	6.04E-19	postive
CCS	AP001029.1	0.31417503	6.46E-13	postive

CCS	AP001033.3	0.360872031	7.98E-17	postive
CCS	AP001062.1	0.461919512	8.56E-28	postive
CCS	AP001107.1	0.569731949	2.28E-44	postive
CCS	AP001107.4	0.498613224	8.76E-33	postive
CCS	AP001107.6	0.408930803	1.41E-21	postive
CCS	AP001107.7	0.308405543	1.77E-12	postive
CCS	AP001107.8	0.356779638	1.87E-16	postive
CCS	AP001160.1	0.598665484	6.07E-50	postive
CCS	AP001160.2	0.42874953	8.93E-24	postive
CCS	AP001160.3	0.598574562	6.33E-50	postive
CCS	AP001160.4	0.343824066	2.55E-15	postive
CCS	AP001267.1	0.439218467	5.37E-25	postive
CCS	AP001267.3	0.545084484	4.88E-40	postive
CCS	AP001318.2	0.536508838	1.30E-38	postive
CCS	AP001351.1	0.397080817	2.48E-20	postive
CCS	AP001372.2	0.515594524	2.63E-35	postive
CCS	AP001412.1	0.418288368	1.35E-22	postive
CCS	AP001453.2	0.515975508	2.30E-35	postive
CCS	AP001453.4	0.604217417	4.44E-51	postive
CCS	AP001458.1	0.307158205	2.19E-12	postive
CCS	AP001462.1	0.533990626	3.34E-38	postive
CCS	AP001469.2	0.321157972	1.85E-13	postive
CCS	AP001469.3	0.436698206	1.07E-24	postive
CCS	AP001486.2	0.318183866	3.16E-13	postive
CCS	AP001505.1	0.478552739	5.56E-30	postive
CCS	AP001527.2	0.500077466	5.38E-33	postive
CCS	AP001542.3	0.340855865	4.55E-15	postive
CCS	AP001619.1	0.325388839	8.54E-14	postive
CCS	AP001626.1	0.321270604	1.81E-13	postive
CCS	AP001628.1	0.368568021	1.56E-17	postive
CCS	AP001630.1	0.300515228	6.78E-12	postive
CCS	AP001816.1	0.500797718	4.23E-33	postive
CCS	AP001830.1	0.49113266	1.02E-31	postive
CCS	AP001922.5	0.359089377	1.16E-16	postive
CCS	AP001922.6	0.401133502	9.42E-21	postive
CCS	AP001972.1	0.384857869	4.24E-19	postive
CCS	AP001992.1	0.365216455	3.19E-17	postive
CCS	AP002026.1	0.361935638	6.38E-17	postive
CCS	AP002360.2	0.533162834	4.55E-38	postive
CCS	AP002387.2	0.505249598	9.41E-34	postive
CCS	AP002490.1	0.420246039	8.16E-23	postive
CCS	AP002748.4	0.58694507	1.28E-47	postive
CCS	AP002761.1	0.463342538	5.62E-28	postive
CCS	AP002761.3	0.459309023	1.84E-27	postive
CCS	AP002761.4	0.378916801	1.62E-18	postive
CCS	AP002807.1	0.498070223	1.05E-32	postive
CCS	AP002812.2	0.301851983	5.42E-12	postive
CCS	AP002840.2	0.476393258	1.09E-29	postive
CCS	AP002992.1	0.370784571	9.66E-18	postive
CCS	AP003059.2	0.365361713	3.09E-17	postive

CCS	AP003068.1	0.522361682	2.37E-36	postive
CCS	AP003068.2	0.398519352	1.76E-20	postive
CCS	AP003096.1	0.484413853	8.82E-31	postive
CCS	AP003119.2	0.343178102	2.89E-15	postive
CCS	AP003352.1	0.44537053	9.83E-26	postive
CCS	AP003392.1	0.344362208	2.29E-15	postive
CCS	AP003392.3	0.35407256	3.26E-16	postive
CCS	AP003392.4	0.319263326	2.61E-13	postive
CCS	AP003392.5	0.464538702	3.94E-28	postive
CCS	AP003419.3	0.59962214	3.88E-50	postive
CCS	AP003486.1	0.415888255	2.48E-22	postive
CCS	AP003498.1	0.402180035	7.32E-21	postive
CCS	AP003559.1	0.328486294	4.81E-14	postive
CCS	AP003680.1	0.360550677	8.53E-17	postive
CCS	AP003721.4	0.378733993	1.68E-18	postive
CCS	AP004608.1	0.357443111	1.63E-16	postive
CCS	AP004609.5	0.464948114	3.49E-28	postive
CCS	AP005061.2	0.329672428	3.86E-14	postive
CCS	AP005329.1	0.442279835	2.32E-25	postive
CCS	AP005432.2	0.327289506	6.01E-14	postive
CCS	AP005482.4	0.350988384	6.09E-16	postive
CCS	AP005717.1	0.314185275	6.45E-13	postive
CCS	AP006222.1	0.355206789	2.58E-16	postive
CCS	AP006287.2	0.412019488	6.54E-22	postive
CCS	AP006621.2	0.415877588	2.48E-22	postive
CCS	AP006621.3	0.523091587	1.83E-36	postive
CCS	AP006621.4	0.394821238	4.23E-20	postive
CCS	AP006623.1	0.344585404	2.19E-15	postive
CCS	AP4B1-AS1	0.305320927	3.01E-12	postive
CCS	APTR	0.433212824	2.73E-24	postive
CCS	ARHGAP5-AS1	0.308988442	1.60E-12	postive
CCS	ARHGEF26-AS1	0.358065946	1.43E-16	postive
CCS	ARHGEF35-AS1	0.312642969	8.46E-13	postive
CCS	ARMCX5-GPRASP2	0.492412108	6.74E-32	postive
CCS	ARRDC1-AS1	0.606537879	1.47E-51	postive
CCS	ASB16-AS1	0.578744994	4.79E-46	postive
CCS	ASH1L-AS1	0.517999098	1.13E-35	postive
CCS	ASMTL-AS1	0.530447222	1.25E-37	postive
CCS	ATF4P4	0.361465213	7.05E-17	postive
CCS	ATP2A1-AS1	0.384236287	4.89E-19	postive
CCS	ATP2B1-AS1	0.328829093	4.51E-14	postive
CCS	ATP2C2-AS1	0.414381444	3.62E-22	postive
CCS	ATP6V0E2-AS1	0.378640712	1.72E-18	postive
CCS	ATXN1-AS1	0.425665477	2.01E-23	postive
CCS	ATXN2-AS	0.508146644	3.50E-34	postive
CCS	AZIN1-AS1	0.336033938	1.16E-14	postive
CCS	BAALC-AS1	0.308370097	1.78E-12	postive
CCS	BACE1-AS	0.567159568	6.71E-44	postive
CCS	BAIAP2-DT	0.435272897	1.57E-24	postive
CCS	BCDIN3D-AS1	0.509580631	2.14E-34	postive

CCS	BDNF-AS	0.352889452	4.14E-16	postive
CCS	BMS1P20	0.462190445	7.90E-28	postive
CCS	BMS1P4	0.331224686	2.88E-14	postive
CCS	BOLA2P2	0.331443067	2.77E-14	postive
CCS	BRWD1-AS2	0.427802315	1.15E-23	postive
CCS	BTG3-AS1	0.394511788	4.55E-20	postive
CCS	BTN2A3P	0.451409329	1.79E-26	postive
CCS	BTNL10	0.333785615	1.78E-14	postive
CCS	BX255925.1	0.340355993	5.02E-15	postive
CCS	BX284668.5	0.407230986	2.14E-21	postive
CCS	BX322557.10	0.358053221	1.44E-16	postive
CCS	BX537318.1	0.552568926	2.58E-41	postive
CCS	BX539320.1	0.395608422	3.51E-20	postive
CCS	BX649601.1	0.467435859	1.66E-28	postive
CCS	BX649632.1	0.442233033	2.35E-25	postive
CCS	C10orf25	0.309177011	1.55E-12	postive
CCS	C11orf72	0.323871653	1.13E-13	postive
CCS	C15orf56	0.441338122	3.00E-25	postive
CCS	C17orf82	0.302323124	5.00E-12	postive
CCS	C1orf147	0.341723918	3.84E-15	postive
CCS	C1orf229	0.444351428	1.31E-25	postive
CCS	C1RL-AS1	0.370290193	1.08E-17	postive
CCS	C22orf24	0.334776784	1.47E-14	postive
CCS	C22orf46	0.516916564	1.65E-35	postive
CCS	C2CD4D-AS1	0.302986563	4.47E-12	postive
CCS	C3orf35	0.341109569	4.33E-15	postive
CCS	C5orf66	0.406660085	2.46E-21	postive
CCS	C6orf99	0.369090736	1.39E-17	postive
CCS	C8orf37-AS1	0.302848031	4.58E-12	postive
CCS	C9orf139	0.313039863	7.89E-13	postive
CCS	C9orf147	0.371527416	8.22E-18	postive
CCS	C9orf163	0.48321532	1.29E-30	postive
CCS	CA5BP1	0.545013245	5.02E-40	postive
CCS	CACTIN-AS1	0.436406733	1.15E-24	postive
CCS	CAHM	0.492690285	6.16E-32	postive
CCS	CAMTA1-DT	0.380463257	1.14E-18	postive
CCS	CAPN10-DT	0.558512838	2.36E-42	postive
CCS	CASK-AS1	0.325519142	8.34E-14	postive
CCS	CASP16P	0.378709189	1.69E-18	postive
CCS	CATSPER2P1	0.41093335	8.57E-22	postive
CCS	CBR3-AS1	0.503494236	1.71E-33	postive
CCS	CCDC183-AS1	0.440628995	3.65E-25	postive
CCS	CCDC18-AS1	0.356605163	1.94E-16	postive
CCS	CCDC84-DT	0.340428058	4.95E-15	postive
CCS	CCNT2-AS1	0.437184272	9.35E-25	postive
CCS	CCT6P1	0.455077733	6.28E-27	postive
CCS	CCT6P3	0.432845673	3.01E-24	postive
CCS	CD27-AS1	0.543391874	9.39E-40	postive
CCS	CD2BP2-DT	0.433898877	2.27E-24	postive
CCS	CD99P1	0.434622067	1.87E-24	postive

CCS	CDC37L1-DT	0.47426012	2.09E-29	postive
CCS	CEBPA-DT	0.304855998	3.26E-12	postive
CCS	CEBPB-AS1	0.385942699	3.31E-19	postive
CCS	CENPBD1P1	0.476553551	1.03E-29	postive
CCS	CEP83-DT	0.329230105	4.19E-14	postive
CCS	CERNA1	0.338202625	7.62E-15	postive
CCS	CFL1P1	0.416525273	2.11E-22	postive
CCS	CHKB-CPT1B	0.377607729	2.16E-18	postive
CCS	CHKB-DT	0.435516795	1.47E-24	postive
CCS	CHROMR	0.310195864	1.30E-12	postive
CCS	CIDECP1	0.539301598	4.50E-39	postive
CCS	CIRBP-AS1	0.44486627	1.13E-25	postive
CCS	CKMT2-AS1	0.509465663	2.22E-34	postive
CCS	CLUHP3	0.422928531	4.09E-23	postive
CCS	CNNM3-DT	0.47928265	4.43E-30	postive
CCS	COA6-AS1	0.48454933	8.45E-31	postive
CCS	COL6A4P2	0.401712921	8.19E-21	postive
CCS	COX10-AS1	0.487552832	3.24E-31	postive
CCS	CR381653.1	0.368492391	1.58E-17	postive
CCS	CR559946.1	0.334973985	1.42E-14	postive
CCS	CR559946.2	0.402071724	7.51E-21	postive
CCS	CRNDE	0.453331676	1.04E-26	postive
CCS	CROCCP2	0.480813034	2.74E-30	postive
CCS	CROCCP3	0.463562181	5.27E-28	postive
CCS	CRTC3-AS1	0.366115439	2.64E-17	postive
CCS	CRYBB2P1	0.531464569	8.55E-38	postive
CCS	CRYZL2P	0.455255892	5.96E-27	postive
CCS	CSE1L-AS1	0.365835432	2.80E-17	postive
CCS	CSPG4P10	0.324689816	9.71E-14	postive
CCS	CSPG4P12	0.31319079	7.68E-13	postive
CCS	CT66	0.313815623	6.88E-13	postive
CCS	CTA-14H9.5	0.459829472	1.58E-27	postive
CCS	CTA-292E10.9	0.392276294	7.68E-20	postive
CCS	CTA-989H11.1	0.45246806	1.33E-26	postive
CCS	CTBP1-AS	0.482214231	1.77E-30	postive
CCS	CTBP1-DT	0.58776003	8.91E-48	postive
CCS	CTC-205M6.5	0.321638883	1.70E-13	postive
CCS	CTC-487M23.5	0.334568115	1.53E-14	postive
CCS	CTD-2132N18.4	0.524445309	1.12E-36	postive
CCS	CTD-2134A5.3	0.475332096	1.51E-29	postive
CCS	CTD-2521M24.9	0.400587777	1.07E-20	postive
CCS	CTD-2574D22.4	0.474976404	1.68E-29	postive
CCS	CTSLP3	0.319979117	2.29E-13	postive
CCS	CUTALP	0.44976977	2.86E-26	postive
CCS	CYP51A1-AS1	0.426678812	1.54E-23	postive
CCS	DACT3-AS1	0.341208826	4.25E-15	postive
CCS	DANCR	0.381179782	9.75E-19	postive
CCS	DARS-AS1	0.476335943	1.11E-29	postive
CCS	DBIL5P	0.38466819	4.43E-19	postive
CCS	DCST1-AS1	0.459968074	1.52E-27	postive

CCS	DCXR-DT	0.338109821	7.76E-15	postive
CCS	DDX11-AS1	0.382163625	7.81E-19	postive
CCS	DDX39B-AS1	0.440392749	3.89E-25	postive
CCS	DGCR11	0.394963948	4.09E-20	postive
CCS	DGUOK-AS1	0.457362598	3.24E-27	postive
CCS	DHCR24-DT	0.361771795	6.61E-17	postive
CCS	DHRS4-AS1	0.437109488	9.54E-25	postive
CCS	DHRS4L1	0.316070447	4.61E-13	postive
CCS	DICER1-AS1	0.561378606	7.34E-43	postive
CCS	DLG5-AS1	0.438447906	6.63E-25	postive
CCS	DLGAP1-AS1	0.32631749	7.20E-14	postive
CCS	DLGAP4-AS1	0.364075579	4.07E-17	postive
CCS	DM1-AS	0.578904257	4.47E-46	postive
CCS	DNAJC27-AS1	0.365321471	3.12E-17	postive
CCS	DNAJC3-DT	0.375715188	3.29E-18	postive
CCS	DNM1P35	0.389495721	1.46E-19	postive
CCS	DOC2GP	0.3855914	3.59E-19	postive
CCS	DOCK9-DT	0.376162257	2.98E-18	postive
CCS	DPP9-AS1	0.501129439	3.78E-33	postive
CCS	DPY19L2P3	0.338462941	7.25E-15	postive
CCS	DSCAS	0.347310226	1.28E-15	postive
CCS	DSCR9	0.35750956	1.61E-16	postive
CCS	DSTNP2	0.39906598	1.55E-20	postive
CCS	DTX2P1-UPK3BP1-PMS2P11	0.32136673	1.78E-13	postive
CCS	DYRK3-AS1	0.367243346	2.07E-17	postive
CCS	EBLN3P	0.398650147	1.71E-20	postive
CCS	EDRF1-DT	0.401418087	8.80E-21	postive
CCS	EHMT2-AS1	0.412691351	5.53E-22	postive
CCS	EIF1B-AS1	0.346862358	1.39E-15	postive
CCS	EIF2AK3-DT	0.472229289	3.90E-29	postive
CCS	EIF3J-DT	0.504297628	1.30E-33	postive
CCS	EIPR1-IT1	0.305280858	3.03E-12	postive
CCS	ELF3-AS1	0.528319753	2.73E-37	postive
CCS	ELOA-AS1	0.548095622	1.51E-40	postive
CCS	EMC1-AS1	0.542318847	1.42E-39	postive
CCS	EMC3-AS1	0.378469465	1.79E-18	postive
CCS	ENO1-AS1	0.443963989	1.45E-25	postive
CCS	ENTPD3-AS1	0.477524006	7.65E-30	postive
CCS	EP400P1	0.4577689	2.88E-27	postive
CCS	EPB41L4A-DT	0.47796527	6.67E-30	postive
CCS	EPCAM-DT	0.357667975	1.55E-16	postive
CCS	ERICD	0.44177156	2.67E-25	postive
CCS	ERVK13-1	0.363026233	5.08E-17	postive
CCS	ESPNP	0.344651074	2.16E-15	postive
CCS	ETV7-AS1	0.340887068	4.53E-15	postive
CCS	EXOC3-AS1	0.425392486	2.16E-23	postive
CCS	EXOSC10-AS1	0.388473693	1.85E-19	postive
CCS	EXTL3-AS1	0.431939966	3.83E-24	postive
CCS	FABP5P3	0.366580613	2.39E-17	postive
CCS	FAHD2CP	0.583951017	4.87E-47	postive

CCS	FALEC	0.430636472	5.42E-24	postive
CCS	FAM111A-DT	0.508399491	3.21E-34	postive
CCS	FAM135A-AS1	0.340905811	4.51E-15	postive
CCS	FAM157B	0.381021785	1.01E-18	postive
CCS	FAM157C	0.372551033	6.58E-18	postive
CCS	FAM160A1-DT	0.344773654	2.11E-15	postive
CCS	FAM201A	0.316586558	4.21E-13	postive
CCS	FAM21EP	0.375917393	3.15E-18	postive
CCS	FAM21FP	0.428133285	1.05E-23	postive
CCS	FAM25E	0.312850977	8.16E-13	postive
CCS	FAM66D	0.355473682	2.44E-16	postive
CCS	FAM83C-AS1	0.459902408	1.55E-27	postive
CCS	FAM86B3P	0.485929567	5.44E-31	postive
CCS	FAM86C2P	0.617112797	8.36E-54	postive
CCS	FAM86DP	0.389141301	1.59E-19	postive
CCS	FAM86EP	0.542326152	1.42E-39	postive
CCS	FAM86FP	0.361958651	6.35E-17	postive
CCS	FAM86HP	0.47947516	4.17E-30	postive
CCS	FAM86JP	0.503517201	1.69E-33	postive
CCS	FBXL19-AS1	0.355735748	2.32E-16	postive
CCS	FGD5-AS1	0.378665142	1.71E-18	postive
CCS	FGF7P3	0.310872809	1.15E-12	postive
CCS	FLNB-AS1	0.304142599	3.68E-12	postive
CCS	FLVCR1-DT	0.383227358	6.14E-19	postive
CCS	FMR1-IT1	0.376125432	3.00E-18	postive
CCS	FO393418.1	0.318046132	3.24E-13	postive
CCS	FO704657.1	0.387905063	2.11E-19	postive
CCS	FOXD2-AS1	0.423791129	3.27E-23	postive
CCS	FOXO6-AS1	0.368714416	1.51E-17	postive
CCS	FOXP4-AS1	0.319601708	2.45E-13	postive
CCS	FRG1-DT	0.344289238	2.32E-15	postive
CCS	FRG1HP	0.458228467	2.52E-27	postive
CCS	FZD4-DT	0.477147901	8.60E-30	postive
CCS	GABPB1-AS1	0.331407825	2.78E-14	postive
CCS	GARS-DT	0.358550879	1.29E-16	postive
CCS	GAS5-AS1	0.337728554	8.35E-15	postive
CCS	GAS8-AS1	0.368503173	1.58E-17	postive
CCS	GBAP1	0.508527329	3.07E-34	postive
CCS	GCC2-AS1	0.426185087	1.75E-23	postive
CCS	GEMIN7-AS1	0.50603717	7.20E-34	postive
CCS	GGNBP1	0.321574985	1.72E-13	postive
CCS	GHET1	0.333664516	1.82E-14	postive
CCS	GHRLOS	0.364555241	3.67E-17	postive
CCS	GIHCG	0.344031628	2.44E-15	postive
CCS	GLIDR	0.374875684	3.96E-18	postive
CCS	GLUD1P2	0.365091118	3.28E-17	postive
CCS	GLUD1P3	0.396276759	3.00E-20	postive
CCS	GMDS-DT	0.407891751	1.82E-21	postive
CCS	GNRHR2	0.526302173	5.70E-37	postive
CCS	GOLGA2P10	0.382609633	7.06E-19	postive

CCS	GOLGA2P5	0.415217147	2.93E-22	postive
CCS	GOLGA2P7	0.308301713	1.80E-12	postive
CCS	GOLGA6L5P	0.365299295	3.14E-17	postive
CCS	GPRC5D-AS1	0.389494275	1.47E-19	postive
CCS	GRHL3-AS1	0.336722408	1.01E-14	postive
CCS	GS1-124K5.3	0.395600477	3.52E-20	postive
CCS	GS1-358P8.4	0.364458767	3.75E-17	postive
CCS	GTF2IP1	0.470922753	5.80E-29	postive
CCS	GTF2IP13	0.48501808	7.28E-31	postive
CCS	GTF2IP20	0.549905077	7.41E-41	postive
CCS	GTF2IP4	0.31753988	3.55E-13	postive
CCS	GTF2IRD1P1	0.338011501	7.91E-15	postive
CCS	GTF2IRD2P1	0.31543186	5.17E-13	postive
CCS	GTF3C2-AS1	0.301876814	5.40E-12	postive
CCS	GTSE1-DT	0.394710309	4.34E-20	postive
CCS	GUCY1B2	0.366001286	2.70E-17	postive
CCS	GUSBP1	0.42232275	4.78E-23	postive
CCS	GUSBP11	0.50209731	2.73E-33	postive
CCS	GUSBP2	0.375046501	3.81E-18	postive
CCS	H1FX-AS1	0.434187801	2.10E-24	postive
CCS	H2AZ2P1	0.480771941	2.78E-30	postive
CCS	H2BC19P	0.351532229	5.46E-16	postive
CCS	H2BC20P	0.358789003	1.23E-16	postive
CCS	HCG11	0.310742883	1.18E-12	postive
CCS	HCG15	0.318214115	3.15E-13	postive
CCS	HCG17	0.34436176	2.29E-15	postive
CCS	HCG18	0.430451819	5.69E-24	postive
CCS	HCG25	0.568733464	3.47E-44	postive
CCS	HCG27	0.405814778	3.03E-21	postive
CCS	HDAC11-AS1	0.404781163	3.90E-21	postive
CCS	HDAC4-AS1	0.338394062	7.34E-15	postive
CCS	HDHD5-AS1	0.421842748	5.41E-23	postive
CCS	HEIH	0.516656964	1.81E-35	postive
CCS	HERC2P2	0.420860233	6.97E-23	postive
CCS	HERC2P9	0.360577025	8.49E-17	postive
CCS	HEXD-IT1	0.367735939	1.86E-17	postive
CCS	HIPK1-AS1	0.342756414	3.14E-15	postive
CCS	HM13-AS1	0.377533145	2.20E-18	postive
CCS	HM13-IT1	0.501287036	3.59E-33	postive
CCS	HMGA1P4	0.309467283	1.47E-12	postive
CCS	HMGB1P5	0.379031012	1.58E-18	postive
CCS	HMGN2P46	0.323024758	1.32E-13	postive
CCS	HMGN3-AS1	0.307603268	2.03E-12	postive
CCS	HOXB-AS1	0.381341611	9.40E-19	postive
CCS	HPN-AS1	0.450147333	2.57E-26	postive
CCS	HSD17B7P2	0.424479339	2.73E-23	postive
CCS	HYI-AS1	0.436578203	1.10E-24	postive
CCS	IBA57-DT	0.470306559	6.99E-29	postive
CCS	IDH1-AS1	0.470661722	6.28E-29	postive
CCS	IDI2-AS1	0.334460596	1.56E-14	postive

CCS	IER3-AS1	0.336375749	1.08E-14	postive
CCS	IGBP1-AS1	0.373017696	5.94E-18	postive
CCS	IL10RB-DT	0.410241035	1.02E-21	postive
CCS	IL6R-AS1	0.368747005	1.50E-17	postive
CCS	ILF3-DT	0.52920461	1.97E-37	postive
CCS	INE1	0.489118864	1.96E-31	postive
CCS	INTS4P1	0.309005422	1.60E-12	postive
CCS	INTS6-AS1	0.451684627	1.66E-26	postive
CCS	INTS6L-AS1	0.369219326	1.36E-17	postive
CCS	IPO9-AS1	0.402187175	7.31E-21	postive
CCS	IQCH-AS1	0.439925034	4.43E-25	postive
CCS	ITFG2-AS1	0.396823765	2.64E-20	postive
CCS	ITGA9-AS1	0.367195372	2.09E-17	postive
CCS	ITPR1-DT	0.364823857	3.47E-17	postive
CCS	JPX	0.43913841	5.49E-25	postive
CCS	KANSL1-AS1	0.32024502	2.18E-13	postive
CCS	KB-1572G7.2	0.477687919	7.27E-30	postive
CCS	KCNIP2-AS1	0.429132158	8.07E-24	postive
CCS	KCTD21-AS1	0.462327662	7.59E-28	postive
CCS	KDM4A-AS1	0.460023373	1.49E-27	postive
CCS	KDM7A-DT	0.388125013	2.01E-19	postive
CCS	KIAA1614-AS1	0.316946659	3.95E-13	postive
CCS	KIF1C-AS1	0.463649126	5.13E-28	postive
CCS	KIF9-AS1	0.401073045	9.56E-21	postive
CCS	KLF3-AS1	0.354711695	2.86E-16	postive
CCS	KMT2E-AS1	0.526642944	5.04E-37	postive
CCS	KRT18P55	0.310585852	1.21E-12	postive
CCS	KRT7-AS	0.313177351	7.70E-13	postive
CCS	KRT8P12	0.480820568	2.74E-30	postive
CCS	KTN1-AS1	0.320458642	2.10E-13	postive
CCS	L3MBTL2-AS1	0.429920743	6.55E-24	postive
CCS	LAMTOR5-AS1	0.385530294	3.64E-19	postive
CCS	LBX2-AS1	0.482558144	1.59E-30	postive
CCS	LENG8-AS1	0.509488281	2.21E-34	postive
CCS	LGALS8-AS1	0.417929165	1.48E-22	postive
CCS	LIFR-AS1	0.372720752	6.34E-18	postive
CCS	LINC00106	0.461068558	1.10E-27	postive
CCS	LINC00115	0.552268711	2.90E-41	postive
CCS	LINC00167	0.343097424	2.94E-15	postive
CCS	LINC00173	0.391952116	8.28E-20	postive
CCS	LINC00174	0.535368214	1.99E-38	postive
CCS	LINC00205	0.445105596	1.06E-25	postive
CCS	LINC00235	0.348089609	1.09E-15	postive
CCS	LINC00240	0.345661687	1.77E-15	postive
CCS	LINC002481	0.375993408	3.09E-18	postive
CCS	LINC00265	0.429455026	7.41E-24	postive
CCS	LINC00271	0.301822899	5.44E-12	postive
CCS	LINC00294	0.400426049	1.12E-20	postive
CCS	LINC00324	0.307389288	2.11E-12	postive
CCS	LINC00336	0.393146772	6.27E-20	postive

CCS	LINC00339	0.572471696	7.13E-45	postive
CCS	LINC00449	0.312305637	8.97E-13	postive
CCS	LINC00467	0.47771939	7.20E-30	postive
CCS	LINC00471	0.41829713	1.34E-22	postive
CCS	LINC00476	0.459055248	1.98E-27	postive
CCS	LINC00482	0.417511313	1.64E-22	postive
CCS	LINC00511	0.352021425	4.94E-16	postive
CCS	LINC00526	0.392117095	7.97E-20	postive
CCS	LINC00623	0.376537727	2.74E-18	postive
CCS	LINC00629	0.336322619	1.09E-14	postive
CCS	LINC00630	0.301362715	5.88E-12	postive
CCS	LINC00637	0.412643454	5.60E-22	postive
CCS	LINC00638	0.384424399	4.68E-19	postive
CCS	LINC00653	0.44404164	1.42E-25	postive
CCS	LINC00654	0.370910156	9.40E-18	postive
CCS	LINC00658	0.300195796	7.16E-12	postive
CCS	LINC00663	0.393715857	5.48E-20	postive
CCS	LINC00667	0.448576346	4.00E-26	postive
CCS	LINC00680	0.459892382	1.55E-27	postive
CCS	LINC00685	0.449816637	2.82E-26	postive
CCS	LINC00847	0.425574693	2.06E-23	postive
CCS	LINC00852	0.337124607	9.38E-15	postive
CCS	LINC00853	0.398198117	1.90E-20	postive
CCS	LINC00857	0.300913827	6.34E-12	postive
CCS	LINC00863	0.353092651	3.98E-16	postive
CCS	LINC00865	0.304388257	3.53E-12	postive
CCS	LINC00869	0.390964757	1.04E-19	postive
CCS	LINC00884	0.334726292	1.48E-14	postive
CCS	LINC00886	0.307743969	1.98E-12	postive
CCS	LINC00888	0.307829195	1.96E-12	postive
CCS	LINC00893	0.410945417	8.55E-22	postive
CCS	LINC00894	0.315123677	5.46E-13	postive
CCS	LINC00896	0.363611616	4.49E-17	postive
CCS	LINC00899	0.462343321	7.55E-28	postive
CCS	LINC00909	0.482349638	1.69E-30	postive
CCS	LINC00910	0.486245181	4.92E-31	postive
CCS	LINC00921	0.461431067	9.88E-28	postive
CCS	LINC00933	0.362663015	5.48E-17	postive
CCS	LINC00950	0.302902254	4.54E-12	postive
CCS	LINC00954	0.322971537	1.33E-13	postive
CCS	LINC00957	0.49982611	5.85E-33	postive
CCS	LINC00963	0.518405134	9.76E-36	postive
CCS	LINC00997	0.388123565	2.01E-19	postive
CCS	LINC01001	0.49022643	1.37E-31	postive
CCS	LINC01003	0.374343291	4.45E-18	postive
CCS	LINC01004	0.445538775	9.38E-26	postive
CCS	LINC01006	0.394599547	4.46E-20	postive
CCS	LINC01011	0.565095817	1.58E-43	postive
CCS	LINC01023	0.512777987	7.06E-35	postive
CCS	LINC01089	0.51384752	4.86E-35	postive

CCS	LINC01106	0.333061006	2.04E-14	postive
CCS	LINC01123	0.376679683	2.66E-18	postive
CCS	LINC01126	0.320712612	2.01E-13	postive
CCS	LINC01128	0.472001457	4.18E-29	postive
CCS	LINC01132	0.333644989	1.82E-14	postive
CCS	LINC01135	0.361555151	6.92E-17	postive
CCS	LINC01137	0.427620859	1.20E-23	postive
CCS	LINC01138	0.392052407	8.09E-20	postive
CCS	LINC01144	0.440635133	3.64E-25	postive
CCS	LINC01145	0.415647441	2.63E-22	postive
CCS	LINC01176	0.415980354	2.42E-22	postive
CCS	LINC01184	0.522867806	1.98E-36	postive
CCS	LINC01220	0.40758003	1.96E-21	postive
CCS	LINC01238	0.35276018	4.26E-16	postive
CCS	LINC01270	0.430062421	6.31E-24	postive
CCS	LINC01271	0.407103447	2.21E-21	postive
CCS	LINC01273	0.397934359	2.02E-20	postive
CCS	LINC01278	0.53715708	1.02E-38	postive
CCS	LINC01311	0.488304621	2.55E-31	postive
CCS	LINC01315	0.342357522	3.40E-15	postive
CCS	LINC01337	0.381965732	8.17E-19	postive
CCS	LINC01342	0.344329455	2.30E-15	postive
CCS	LINC01347	0.328339836	4.94E-14	postive
CCS	LINC01353	0.363246913	4.84E-17	postive
CCS	LINC01355	0.38883837	1.71E-19	postive
CCS	LINC01372	0.510151805	1.76E-34	postive
CCS	LINC01389	0.327477808	5.80E-14	postive
CCS	LINC01424	0.4046813	3.99E-21	postive
CCS	LINC01426	0.36613703	2.62E-17	postive
CCS	LINC01431	0.466700578	2.07E-28	postive
CCS	LINC01521	0.314965766	5.61E-13	postive
CCS	LINC01534	0.390937625	1.05E-19	postive
CCS	LINC01545	0.305280284	3.03E-12	postive
CCS	LINC01560	0.444091	1.40E-25	postive
CCS	LINC01562	0.346421104	1.52E-15	postive
CCS	LINC01569	0.518989914	7.93E-36	postive
CCS	LINC01578	0.353475659	3.68E-16	postive
CCS	LINC01588	0.389954652	1.32E-19	postive
CCS	LINC01607	0.363862017	4.25E-17	postive
CCS	LINC01637	0.320345006	2.14E-13	postive
CCS	LINC01703	0.457170381	3.43E-27	postive
CCS	LINC01715	0.384979461	4.13E-19	postive
CCS	LINC01770	0.57276973	6.28E-45	postive
CCS	LINC01772	0.430264238	5.98E-24	postive
CCS	LINC01786	0.41734534	1.71E-22	postive
CCS	LINC01816	0.3826422	7.01E-19	postive
CCS	LINC01881	0.305910415	2.72E-12	postive
CCS	LINC01909	0.311605578	1.01E-12	postive
CCS	LINC01952	0.3420285	3.62E-15	postive
CCS	LINC01977	0.417242909	1.76E-22	postive

CCS	LINC01993	0.333401342	1.91E-14	postive
CCS	LINC02014	0.369495301	1.28E-17	postive
CCS	LINC02019	0.355364629	2.50E-16	postive
CCS	LINC02021	0.438390623	6.73E-25	postive
CCS	LINC02026	0.303406831	4.17E-12	postive
CCS	LINC02035	0.343325158	2.81E-15	postive
CCS	LINC02062	0.458835799	2.11E-27	postive
CCS	LINC02193	0.420306443	8.04E-23	postive
CCS	LINC02210	0.436135416	1.24E-24	postive
CCS	LINC02298	0.352432247	4.55E-16	postive
CCS	LINC02332	0.365314778	3.13E-17	postive
CCS	LINC02352	0.319195129	2.64E-13	postive
CCS	LINC02361	0.375293789	3.61E-18	postive
CCS	LINC02363	0.313841153	6.85E-13	postive
CCS	LINC02447	0.316398332	4.35E-13	postive
CCS	LINC02449	0.346005886	1.65E-15	postive
CCS	LINC02482	0.487098522	3.75E-31	postive
CCS	LINC02585	0.499573542	6.36E-33	postive
CCS	LINC02601	0.376893128	2.54E-18	postive
CCS	LINC02603	0.34795352	1.12E-15	postive
CCS	LINC02604	0.570606905	1.57E-44	postive
CCS	LINC02609	0.302843015	4.58E-12	postive
CCS	LINC02611	0.312776245	8.26E-13	postive
CCS	LINC02615	0.362460806	5.72E-17	postive
CCS	LINC02631	0.324253771	1.05E-13	postive
CCS	LINC02637	0.324908759	9.33E-14	postive
CCS	LINC02693	0.358308639	1.36E-16	postive
CCS	LINC02709	0.319860012	2.34E-13	postive
CCS	LINC02739	0.358736356	1.25E-16	postive
CCS	LINC02749	0.310208231	1.29E-12	postive
CCS	LINC02777	0.360930373	7.88E-17	postive
CCS	LINC02803	0.38425273	4.87E-19	postive
CCS	LINC02818	0.331171836	2.91E-14	postive
CCS	LIPE-AS1	0.451853368	1.58E-26	postive
CCS	LIX1L-AS1	0.380170569	1.22E-18	postive
CCS	LLPH-DT	0.338389962	7.35E-15	postive
CCS	LMCD1-AS1	0.340299245	5.08E-15	postive
CCS	LMNTD2-AS1	0.335810282	1.21E-14	postive
CCS	LNCTAM34A	0.428040301	1.08E-23	postive
CCS	LOH12CR2	0.389690801	1.40E-19	postive
CCS	LOXL1-AS1	0.360345169	8.91E-17	postive
CCS	LPP-AS2	0.364823132	3.47E-17	postive
CCS	LRRC37A17P	0.347005511	1.36E-15	postive
CCS	LRRC37BP1	0.496334509	1.86E-32	postive
CCS	MAFG-DT	0.478807924	5.14E-30	postive
CCS	MAN1B1-DT	0.469452451	9.05E-29	postive
CCS	MAP3K14-AS1	0.387724197	2.20E-19	postive
CCS	MAP3K2-DT	0.311011012	1.13E-12	postive
CCS	MAP4K3-DT	0.473500693	2.64E-29	postive
CCS	MAPKAPK5-AS1	0.55839196	2.48E-42	postive

CCS	MATN1-AS1	0.499153805	7.32E-33	postive
CCS	MBL1P	0.306918806	2.29E-12	postive
CCS	MCCC1-AS1	0.30637446	2.51E-12	postive
CCS	MCM3AP-AS1	0.449458629	3.12E-26	postive
CCS	MCPH1-AS1	0.315404083	5.19E-13	postive
CCS	MDS2	0.300023844	7.37E-12	postive
CCS	MED14OS	0.33575748	1.22E-14	postive
CCS	MED8-AS1	0.498507361	9.07E-33	postive
CCS	MELTF-AS1	0.396059616	3.16E-20	postive
CCS	MHENCR	0.477269012	8.28E-30	postive
CCS	MIATNB	0.353783451	3.45E-16	postive
CCS	MIF-AS1	0.34245921	3.33E-15	postive
CCS	MINCR	0.435401083	1.51E-24	postive
CCS	MIPEPP3	0.443793314	1.52E-25	postive
CCS	MIR200CHG	0.404231988	4.45E-21	postive
CCS	MIR210HG	0.467078351	1.85E-28	postive
CCS	MIR22HG	0.393363924	5.96E-20	postive
CCS	MIR34AHG	0.404622052	4.05E-21	postive
CCS	MIR3936HG	0.45377189	9.13E-27	postive
CCS	MIR4435-2HG	0.303765218	3.92E-12	postive
CCS	MIR4453HG	0.405319711	3.42E-21	postive
CCS	MIR4458HG	0.321296135	1.80E-13	postive
CCS	MIR497HG	0.379927637	1.29E-18	postive
CCS	MIR503HG	0.388538745	1.83E-19	postive
CCS	MIR583HG	0.321851903	1.63E-13	postive
CCS	MIR600HG	0.387588915	2.27E-19	postive
CCS	MIR646HG	0.322942509	1.34E-13	postive
CCS	MIR762HG	0.574831653	2.60E-45	postive
CCS	MIS18A-AS1	0.363680984	4.42E-17	postive
CCS	MKLN1-AS	0.367028366	2.17E-17	postive
CCS	MMP25-AS1	0.455346804	5.81E-27	postive
CCS	MNX1-AS1	0.354566916	2.94E-16	postive
CCS	MNX1-AS2	0.375191765	3.69E-18	postive
CCS	MORF4L2-AS1	0.392094943	8.01E-20	postive
CCS	MRPL20-AS1	0.688144363	2.16E-71	postive
CCS	MRPL45P2	0.42482519	2.50E-23	postive
CCS	MRPS9-AS1	0.311181695	1.09E-12	postive
CCS	MSH5-SAPCD1	0.532515819	5.79E-38	postive
CCS	MSL3P1	0.340634418	4.76E-15	postive
CCS	MTMR9LP	0.496804042	1.60E-32	postive
CCS	MUC20-OT1	0.370910725	9.40E-18	postive
CCS	MYLK-AS1	0.428692208	9.07E-24	postive
CCS	MZF1-AS1	0.455737082	5.19E-27	postive
CCS	NACA4P	0.386832366	2.70E-19	postive
CCS	NALT1	0.45257203	1.29E-26	postive
CCS	NAPA-AS1	0.428878167	8.64E-24	postive
CCS	NARF-AS1	0.367025829	2.17E-17	postive
CCS	NARF-IT1	0.395448784	3.65E-20	postive
CCS	NBPF25P	0.468098653	1.36E-28	postive
CCS	NBPF8	0.426659206	1.55E-23	postive

CCS	NBR2	0.569578879	2.43E-44	postive
CCS	NCBP2-AS1	0.387128859	2.53E-19	postive
CCS	NCK1-DT	0.361044652	7.70E-17	postive
CCS	NDUFA6-DT	0.492516924	6.51E-32	postive
CCS	NDUFB2-AS1	0.574984871	2.43E-45	postive
CCS	NDUFV2-AS1	0.480516041	3.01E-30	postive
CCS	NEBL-AS1	0.372167574	7.15E-18	postive
CCS	NFYC-AS1	0.411975272	6.62E-22	postive
CCS	NIFK-AS1	0.613442747	5.14E-53	postive
CCS	NINJ2-AS1	0.384011998	5.14E-19	postive
CCS	NKAPP1	0.332707296	2.18E-14	postive
CCS	NKX2-1-AS1	0.397786031	2.10E-20	postive
CCS	NNT-AS1	0.346188645	1.59E-15	postive
CCS	NOP14-AS1	0.601959211	1.29E-50	postive
CCS	NOP53-AS1	0.326919797	6.44E-14	postive
CCS	NORAD	0.420408832	7.83E-23	postive
CCS	NPEPPSP1	0.335607077	1.25E-14	postive
CCS	NPIPB14P	0.429475988	7.37E-24	postive
CCS	NPIPB1P	0.384964519	4.14E-19	postive
CCS	NPIPP1	0.543672241	8.43E-40	postive
CCS	NPPA-AS1	0.418854859	1.17E-22	postive
CCS	NRADDP	0.308526899	1.73E-12	postive
CCS	NRAV	0.493626707	4.53E-32	postive
CCS	NRSN2-AS1	0.482120088	1.82E-30	postive
CCS	NSMCE1-DT	0.417127948	1.81E-22	postive
CCS	NSUN5P1	0.520358759	4.87E-36	postive
CCS	NSUN5P2	0.514123087	4.41E-35	postive
CCS	NUDT16P1	0.367377001	2.01E-17	postive
CCS	NUP50-DT	0.428517513	9.50E-24	postive
CCS	NUTM2A-AS1	0.405456719	3.30E-21	postive
CCS	NXT1-AS1	0.333127108	2.01E-14	postive
CCS	OCIAD1-AS1	0.310049493	1.33E-12	postive
CCS	ODC1-DT	0.32342347	1.22E-13	postive
CCS	OGFR-AS1	0.446537472	7.09E-26	postive
CCS	OGFRP1	0.409052577	1.37E-21	postive
CCS	OLMALINC	0.574000201	3.71E-45	postive
CCS	OR2A9P	0.379114115	1.55E-18	postive
CCS	OR7E14P	0.35230514	4.67E-16	postive
CCS	OSER1-DT	0.502618371	2.29E-33	postive
CCS	OSGEPL1-AS1	0.398640312	1.71E-20	postive
CCS	OTUD6B-AS1	0.379122631	1.54E-18	postive
CCS	PAN3-AS1	0.405724387	3.10E-21	postive
CCS	PARD3-AS1	0.377308877	2.31E-18	postive
CCS	PARD6G-AS1	0.324643499	9.80E-14	postive
CCS	PAXBP1-AS1	0.378468786	1.79E-18	postive
CCS	PAXIP1-AS1	0.557016269	4.33E-42	postive
CCS	PAXIP1-AS2	0.403626076	5.16E-21	postive
CCS	PCAT6	0.559428688	1.63E-42	postive
CCS	PCBP1-AS1	0.50813412	3.52E-34	postive
CCS	PCCA-DT	0.352504628	4.48E-16	postive

CCS	PCOTH	0.335382226	1.31E-14	postive
CCS	PDCD4-AS1	0.462138927	8.02E-28	postive
CCS	PDIA3P1	0.435702364	1.40E-24	postive
CCS	PDZK1P1	0.35215436	4.81E-16	postive
CCS	PGAM1P8	0.424140959	2.99E-23	postive
CCS	PHKA2-AS1	0.321554937	1.72E-13	postive
CCS	PI4KAP1	0.315109235	5.47E-13	postive
CCS	PI4KAP2	0.485061644	7.18E-31	postive
CCS	PIK3CD-AS2	0.34922383	8.70E-16	postive
CCS	PIK3IP1-AS1	0.38200318	8.10E-19	postive
CCS	PINK1-AS	0.413546023	4.47E-22	postive
CCS	PITPNA-AS1	0.529143729	2.01E-37	postive
CCS	PITRM1-AS1	0.378902552	1.62E-18	postive
CCS	PKD1P6	0.498849998	8.10E-33	postive
CCS	PLEKHA8P1	0.402749863	6.38E-21	postive
CCS	PLEKHM1P1	0.383503277	5.77E-19	postive
CCS	PMS2CL	0.516092203	2.21E-35	postive
CCS	PMS2P3	0.450641139	2.23E-26	postive
CCS	PMS2P4	0.458306396	2.46E-27	postive
CCS	POLH-AS1	0.323777603	1.15E-13	postive
CCS	POLR2J4	0.647101453	1.17E-60	postive
CCS	PPP1R14B-AS1	0.391278629	9.69E-20	postive
CCS	PPP1R26-AS1	0.441741943	2.69E-25	postive
CCS	PPP1R35-AS1	0.338976808	6.56E-15	postive
CCS	PPP3CB-AS1	0.392812692	6.78E-20	postive
CCS	PPP4R1-AS1	0.375803386	3.23E-18	postive
CCS	PPT2-EGFL8	0.563852298	2.65E-43	postive
CCS	PRC1-AS1	0.419800191	9.15E-23	postive
CCS	PRELID1P1	0.330549991	3.27E-14	postive
CCS	PRKAG2-AS1	0.379667673	1.37E-18	postive
CCS	PRKAR2A-AS1	0.487378883	3.43E-31	postive
CCS	PRKCZ-AS1	0.460781919	1.20E-27	postive
CCS	PRMT5-AS1	0.483771703	1.08E-30	postive
CCS	PRORSD1P	0.536585984	1.26E-38	postive
CCS	PRR34	0.310987838	1.13E-12	postive
CCS	PRR34-AS1	0.456668105	3.97E-27	postive
CCS	PRRT3-AS1	0.360189004	9.20E-17	postive
CCS	PRSS42P	0.328561817	4.74E-14	postive
CCS	PRSS46P	0.312378973	8.86E-13	postive
CCS	PSMA3-AS1	0.477638424	7.39E-30	postive
CCS	PSMB8-AS1	0.387274231	2.44E-19	postive
CCS	PSMG3-AS1	0.327609513	5.66E-14	postive
CCS	PTOV1-AS1	0.539113905	4.84E-39	postive
CCS	PTOV1-AS2	0.552201623	2.98E-41	postive
CCS	PXN-AS1	0.540880513	2.47E-39	postive
CCS	RAB11B-AS1	0.512388038	8.09E-35	postive
CCS	RAB30-DT	0.608275071	6.36E-52	postive
CCS	RABGAP1L-DT	0.345741963	1.74E-15	postive
CCS	RAD51-AS1	0.471568829	4.76E-29	postive
CCS	RALY-AS1	0.546160559	3.21E-40	postive

CCS	RAP2C-AS1	0.317856263	3.36E-13	postive
CCS	RARA-AS1	0.585323219	2.65E-47	postive
CCS	RASA4CP	0.435317455	1.55E-24	postive
CCS	RASSF10-DT	0.319234984	2.62E-13	postive
CCS	RASSF1-AS1	0.481087298	2.52E-30	postive
CCS	RB1-DT	0.311262875	1.08E-12	postive
CCS	RBM15-AS1	0.516129456	2.18E-35	postive
CCS	RBM26-AS1	0.329345991	4.10E-14	postive
CCS	RDM1P5	0.328475781	4.82E-14	postive
CCS	RFPL3S	0.368016153	1.76E-17	postive
CCS	RHPN1-AS1	0.406214387	2.75E-21	postive
CCS	RN7SL832P	0.326552716	6.89E-14	postive
CCS	RNASEH1-AS1	0.405226925	3.49E-21	postive
CCS	RNF139-AS1	0.403114859	5.84E-21	postive
CCS	RNF157-AS1	0.369296079	1.33E-17	postive
CCS	RNF216P1	0.535387962	1.98E-38	postive
CCS	RNFT1-DT	0.386515954	2.91E-19	postive
CCS	ROCK1P1	0.352099519	4.87E-16	postive
CCS	RP11-101E13.5	0.538500161	6.11E-39	postive
CCS	RP11-104N10.2	0.39186375	8.46E-20	postive
CCS	RP11-1055B8.9	0.332570857	2.23E-14	postive
CCS	RP11-10C24.2	0.336655262	1.03E-14	postive
CCS	RP11-111M22.4	0.467096144	1.84E-28	postive
CCS	RP11-111M22.5	0.374778468	4.04E-18	postive
CCS	RP11-153F5.7	0.418213521	1.37E-22	postive
CCS	RP11-175O19.4	0.464109636	4.48E-28	postive
CCS	RP11-188P20.3	0.311605806	1.01E-12	postive
CCS	RP11-192H23.8	0.377683258	2.13E-18	postive
CCS	RP11-199F11.2	0.513384115	5.71E-35	postive
CCS	RP11-206L10.5	0.337890902	8.09E-15	postive
CCS	RP11-226L15.5	0.473145406	2.95E-29	postive
CCS	RP1-122P22.2	0.366741534	2.31E-17	postive
CCS	RP11-258C19.7	0.520432078	4.74E-36	postive
CCS	RP11-282O18.3	0.316893961	3.98E-13	postive
CCS	RP11-285F16.1	0.368982612	1.43E-17	postive
CCS	RP11-2B6.3	0.435589378	1.44E-24	postive
CCS	RP11-314C16.1	0.369551507	1.26E-17	postive
CCS	RP11-321A17.6	0.345190454	1.94E-15	postive
CCS	RP11-346C20.4	0.35087274	6.24E-16	postive
CCS	RP11-376M2.2	0.302626772	4.75E-12	postive
CCS	RP11-380L11.4	0.341369278	4.12E-15	postive
CCS	RP11-429J17.8	0.410326284	9.97E-22	postive
CCS	RP11-434B12.1	0.313016089	7.92E-13	postive
CCS	RP11-435O5.2	0.422196205	4.94E-23	postive
CCS	RP11-455O6.9	0.421709524	5.60E-23	postive
CCS	RP11-465N4.5	0.574541221	2.94E-45	postive
CCS	RP11-492E3.51	0.383275892	6.08E-19	postive
CCS	RP11-508N22.13	0.346340249	1.55E-15	postive
CCS	RP11-529E10.6	0.444046346	1.42E-25	postive
CCS	RP11-539I5.1	0.316962898	3.94E-13	postive

CCS	RP11-540B6.6	0.504906068	1.06E-33	postive
CCS	RP11-658F2.8	0.642247938	1.70E-59	postive
CCS	RP11-661A12.4	0.301661782	5.59E-12	postive
CCS	RP11-69E11.4	0.419180427	1.07E-22	postive
CCS	RP11-706O15.1	0.388417456	1.88E-19	postive
CCS	RP11-86H7.7	0.305423113	2.96E-12	postive
CCS	RP11-876N24.4	0.422373527	4.72E-23	postive
CCS	RP11-876N24.5	0.412880165	5.28E-22	postive
CCS	RP11-91I20.2	0.317646159	3.48E-13	postive
CCS	RP11-97O12.6	0.35793855	1.47E-16	postive
CCS	RP11-98G7.1	0.342935432	3.03E-15	postive
CCS	RP1-199J3.7	0.351506808	5.49E-16	postive
CCS	RP13-476E20.1	0.369500336	1.28E-17	postive
CCS	RP3-355L5.5	0.314658987	5.93E-13	postive
CCS	RP3-461P17.10	0.364834616	3.46E-17	postive
CCS	RP4-545L17.11	0.482262586	1.74E-30	postive
CCS	RP5-1039K5.19	0.379187236	1.52E-18	postive
CCS	RP5-1042I8.7	0.343531023	2.70E-15	postive
CCS	RP5-1112D6.4	0.519999789	5.53E-36	postive
CCS	RP5-1125A11.6	0.398688665	1.69E-20	postive
CCS	RP5-1125A11.7	0.464411381	4.09E-28	postive
CCS	RP5-1139B12.2	0.365639389	2.92E-17	postive
CCS	RP9P	0.437230624	9.23E-25	postive
CCS	RPARP-AS1	0.499045008	7.59E-33	postive
CCS	RPL10P19	0.302516552	4.84E-12	postive
CCS	RPL12P25	0.424181631	2.95E-23	postive
CCS	RPL13P5	0.368012243	1.76E-17	postive
CCS	RPL22P19	0.302865009	4.57E-12	postive
CCS	RPL23AP53	0.358766205	1.24E-16	postive
CCS	RPL23AP7	0.428239333	1.02E-23	postive
CCS	RPL23AP82	0.443556465	1.63E-25	postive
CCS	RPL32P3	0.399570316	1.37E-20	postive
CCS	RPL37P6	0.304124189	3.69E-12	postive
CCS	RPL6P27	0.335990034	1.17E-14	postive
CCS	RPLP1P6	0.393472298	5.81E-20	postive
CCS	RPP38-DT	0.318609799	2.93E-13	postive
CCS	RPS10P7	0.491104224	1.03E-31	postive
CCS	RPS4XP16	0.390131263	1.26E-19	postive
CCS	RRN3P1	0.356822612	1.85E-16	postive
CCS	RRN3P3	0.533201735	4.48E-38	postive
CCS	RRP7BP	0.536391315	1.36E-38	postive
CCS	RTCA-AS1	0.397909312	2.04E-20	postive
CCS	RUSC1-AS1	0.515522963	2.70E-35	postive
CCS	SAP30-DT	0.314504015	6.09E-13	postive
CCS	SBDSP1	0.400257411	1.16E-20	postive
CCS	SBF2-AS1	0.396298237	2.98E-20	postive
CCS	SBNO1-AS1	0.482506981	1.61E-30	postive
CCS	SCAMP1-AS1	0.463705509	5.05E-28	postive
CCS	SCAND2P	0.507370121	4.57E-34	postive
CCS	SCAT2	0.336879384	9.83E-15	postive

CCS	SCGB1B2P	0.395787301	3.37E-20	postive
CCS	SCOC-AS1	0.315687283	4.94E-13	postive
CCS	SDAD1P1	0.388143606	2.00E-19	postive
CCS	SDCBP2-AS1	0.42786961	1.13E-23	postive
CCS	SDHAP1	0.479228394	4.50E-30	postive
CCS	SEC24B-AS1	0.501497617	3.34E-33	postive
CCS	SEMA3B-AS1	0.322057648	1.57E-13	postive
CCS	SEMA3F-AS1	0.487666104	3.12E-31	postive
CCS	SEPSECS-AS1	0.450941964	2.05E-26	postive
CCS	SEPTIN4-AS1	0.386093735	3.20E-19	postive
CCS	SEPTIN7-AS1	0.347989541	1.11E-15	postive
CCS	SEPTIN7P2	0.491334547	9.57E-32	postive
CCS	SERHL	0.426595808	1.57E-23	postive
CCS	SERTAD4-AS1	0.334310737	1.61E-14	postive
CCS	SGMS1-AS1	0.368164326	1.70E-17	postive
CCS	SH3BP5-AS1	0.417400057	1.69E-22	postive
CCS	SH3GL1P1	0.467495665	1.63E-28	postive
CCS	SLC12A9-AS1	0.413496348	4.52E-22	postive
CCS	SLC25A25-AS1	0.360680705	8.31E-17	postive
CCS	SLC25A30-AS1	0.356165608	2.12E-16	postive
CCS	SLC25A5-AS1	0.460170452	1.43E-27	postive
CCS	SLC25A5P1	0.432343991	3.44E-24	postive
CCS	SLC44A3-AS1	0.434134298	2.13E-24	postive
CCS	SLC9A3-AS1	0.381116547	9.89E-19	postive
CCS	SLX1A-SULT1A3	0.494615278	3.28E-32	postive
CCS	SMCR2	0.318461168	3.01E-13	postive
CCS	SMG1P7	0.377888966	2.03E-18	postive
CCS	SMG7-AS1	0.454141768	8.21E-27	postive
CCS	SMIM2-AS1	0.305580043	2.88E-12	postive
CCS	SNAI3-AS1	0.571107306	1.27E-44	postive
CCS	SNHG1	0.420756707	7.16E-23	postive
CCS	SNHG10	0.489352237	1.82E-31	postive
CCS	SNHG11	0.687840281	2.63E-71	postive
CCS	SNHG12	0.554262478	1.31E-41	postive
CCS	SNHG15	0.370062079	1.13E-17	postive
CCS	SNHG16	0.341809014	3.78E-15	postive
CCS	SNHG17	0.480127824	3.40E-30	postive
CCS	SNHG19	0.309898599	1.37E-12	postive
CCS	SNHG20	0.545759244	3.76E-40	postive
CCS	SNHG21	0.463382924	5.55E-28	postive
CCS	SNHG25	0.400750988	1.03E-20	postive
CCS	SNHG28	0.487513775	3.28E-31	postive
CCS	SNHG3	0.421316197	6.20E-23	postive
CCS	SNHG30	0.374983676	3.86E-18	postive
CCS	SNHG32	0.371867261	7.64E-18	postive
CCS	SNHG4	0.302837529	4.59E-12	postive
CCS	SNHG6	0.3253973	8.53E-14	postive
CCS	SNHG7	0.444757792	1.17E-25	postive
CCS	SNHG8	0.361599913	6.85E-17	postive
CCS	SNHG9	0.431444529	4.37E-24	postive

CCS	SP2-AS1	0.342294493	3.44E-15	postive
CCS	SPACA6P-AS	0.363066777	5.03E-17	postive
CCS	SPAG5-AS1	0.494405954	3.51E-32	postive
CCS	SPATA41	0.307832467	1.95E-12	postive
CCS	SPINT1-AS1	0.415749384	2.56E-22	postive
CCS	SPRY4-AS1	0.303187375	4.32E-12	postive
CCS	SREBF2-AS1	0.566304005	9.58E-44	postive
CCS	SRP14-AS1	0.448804036	3.75E-26	postive
CCS	SRP54-AS1	0.304332027	3.56E-12	postive
CCS	SRRM2-AS1	0.450841626	2.11E-26	postive
CCS	SSBP3-AS1	0.314405967	6.20E-13	postive
CCS	SSR4P1	0.489050051	2.00E-31	postive
CCS	ST20-AS1	0.383908878	5.26E-19	postive
CCS	STAG3L1	0.376623177	2.69E-18	postive
CCS	STAG3L2	0.473440065	2.69E-29	postive
CCS	STAG3L3	0.444545328	1.24E-25	postive
CCS	STAG3L4	0.431508088	4.30E-24	postive
CCS	STAG3L5P	0.516286506	2.06E-35	postive
CCS	STAG3L5P-PVRIG2P-PILRB	0.556449469	5.45E-42	postive
CCS	STAM-AS1	0.394084317	5.03E-20	postive
CCS	STARD7-AS1	0.563458343	3.12E-43	postive
CCS	STAU2-AS1	0.345921198	1.68E-15	postive
CCS	STIM2-AS1	0.379525043	1.41E-18	postive
CCS	STK4-AS1	0.357590349	1.58E-16	postive
CCS	STPG3-AS1	0.397370358	2.31E-20	postive
CCS	STX17-AS1	0.412853995	5.31E-22	postive
CCS	STX18-AS1	0.42192095	5.31E-23	postive
CCS	SUCLA2-AS1	0.469664907	8.49E-29	postive
CCS	SUGT1P4-STRA6LP	0.360288473	9.02E-17	postive
CCS	SUMO2P17	0.385212235	3.91E-19	postive
CCS	SUZ12P1	0.525938145	6.51E-37	postive
CCS	SVIL-AS1	0.368728085	1.51E-17	postive
CCS	SYNGAP1-AS1	0.365693863	2.88E-17	postive
CCS	SYS1-DBNDD2	0.433391493	2.60E-24	postive
CCS	SZT2-AS1	0.313722637	6.99E-13	postive
CCS	TAF1A-AS1	0.385591119	3.59E-19	postive
CCS	TAPT1-AS1	0.496157343	1.98E-32	postive
CCS	TBC1D8-AS1	0.390154576	1.26E-19	postive
CCS	TDRKH-AS1	0.418028007	1.44E-22	postive
CCS	TEKT4P2	0.392773647	6.84E-20	postive
CCS	TFAP2A-AS1	0.41900332	1.12E-22	postive
CCS	THAP7-AS1	0.396686861	2.72E-20	postive
CCS	THAP9-AS1	0.453465385	9.97E-27	postive
CCS	THORLNC	0.336104915	1.14E-14	postive
CCS	THUMPD3-AS1	0.475528228	1.42E-29	postive
CCS	TIPARP-AS1	0.440291827	4.00E-25	postive
CCS	TMC3-AS1	0.386081616	3.21E-19	postive
CCS	TMCC1-AS1	0.379053219	1.57E-18	postive
CCS	TMCO1-AS1	0.30954074	1.45E-12	postive
CCS	TMED2-DT	0.56048604	1.06E-42	postive

CCS	TMEM147-AS1	0.501474361	3.37E-33	postive
CCS	TMEM161B-AS1	0.392823752	6.76E-20	postive
CCS	TMEM191A	0.363298935	4.79E-17	postive
CCS	TMEM198B	0.496685327	1.66E-32	postive
CCS	TMEM202-AS1	0.440403739	3.88E-25	postive
CCS	TMEM254-AS1	0.368658914	1.53E-17	postive
CCS	TMEM44-AS1	0.462317839	7.61E-28	postive
CCS	TMEM99	0.403887695	4.84E-21	postive
CCS	TMEM9B-AS1	0.315459832	5.14E-13	postive
CCS	TNFRSF14-AS1	0.315118805	5.46E-13	postive
CCS	TNK2-AS1	0.311897771	9.64E-13	postive
CCS	TNKS2-AS1	0.317838472	3.37E-13	postive
CCS	TNRC6C-AS1	0.411976491	6.61E-22	postive
CCS	TOB1-AS1	0.550602791	5.62E-41	postive
CCS	TOLLIP-AS1	0.499824201	5.85E-33	postive
CCS	TONSL-AS1	0.476740566	9.75E-30	postive
CCS	TP53TG1	0.472963278	3.12E-29	postive
CCS	TP73-AS1	0.564483617	2.04E-43	postive
CCS	TPM3P9	0.336359576	1.09E-14	postive
CCS	TPT1-AS1	0.467388805	1.68E-28	postive
CCS	TPTEP2	0.414144342	3.84E-22	postive
CCS	TRAF3IP2-AS1	0.338335966	7.43E-15	postive
CCS	TRAM2-AS1	0.411760845	6.98E-22	postive
CCS	TRAPPC12-AS1	0.325022755	9.14E-14	postive
CCS	TRIM52-AS1	0.472100555	4.05E-29	postive
CCS	TRPM2-AS	0.361664594	6.76E-17	postive
CCS	TSC22D1-AS1	0.392766349	6.85E-20	postive
CCS	TSPY26P	0.373012066	5.95E-18	postive
CCS	TSTD3	0.412371728	5.99E-22	postive
CCS	TTC28-AS1	0.60097524	2.06E-50	postive
CCS	TTC39A-AS1	0.347133093	1.32E-15	postive
CCS	TTC41P	0.357599278	1.58E-16	postive
CCS	TUBBP5	0.304801488	3.29E-12	postive
CCS	TUG1	0.479846714	3.71E-30	postive
CCS	U47924.1	0.418286297	1.35E-22	postive
CCS	U47924.2	0.450859511	2.10E-26	postive
CCS	U47924.3	0.38395377	5.21E-19	postive
CCS	U52111.1	0.358773906	1.24E-16	postive
CCS	U62317.2	0.454005837	8.54E-27	postive
CCS	U62317.3	0.406010118	2.89E-21	postive
CCS	U73166.1	0.457378475	3.23E-27	postive
CCS	U82695.1	0.310219279	1.29E-12	postive
CCS	U91328.1	0.397015689	2.52E-20	postive
CCS	U91328.3	0.41582675	2.51E-22	postive
CCS	UBA6-AS1	0.530784149	1.10E-37	postive
CCS	UBAC2-AS1	0.392680016	6.99E-20	postive
CCS	UBE2D3-AS1	0.467782835	1.50E-28	postive
CCS	UBE2Q1-AS1	0.449662005	2.94E-26	postive
CCS	UBE2Q2P1	0.352718105	4.29E-16	postive
CCS	UBE2Q2P2	0.315876753	4.78E-13	postive

CCS	UBOX5-AS1	0.420598402	7.46E-23	postive
CCS	UBR5-AS1	0.421408736	6.05E-23	postive
CCS	UBXN10-AS1	0.360006471	9.56E-17	postive
CCS	UGDH-AS1	0.481428825	2.26E-30	postive
CCS	UNC93B6	0.363111493	4.98E-17	postive
CCS	UPF3AP2	0.336348907	1.09E-14	postive
CCS	URAHP	0.563027225	3.73E-43	postive
CCS	URB1-AS1	0.447690372	5.13E-26	postive
CCS	USP27X-AS1	0.503201096	1.88E-33	postive
CCS	USP46-AS1	0.456162832	4.59E-27	postive
CCS	UVRAG-DT	0.437478364	8.63E-25	postive
CCS	UXT-AS1	0.444398111	1.29E-25	postive
CCS	VASH1-AS1	0.509745027	2.02E-34	postive
CCS	VPS13B-DT	0.450242455	2.50E-26	postive
CCS	VPS33B-DT	0.323967598	1.11E-13	postive
CCS	VPS9D1-AS1	0.39668396	2.72E-20	postive
CCS	WAC-AS1	0.622632361	5.21E-55	postive
CCS	WARS2-AS1	0.415327277	2.85E-22	postive
CCS	WASH3P	0.62892993	2.05E-56	postive
CCS	WASIR2	0.3965994	2.78E-20	postive
CCS	WEE2-AS1	0.331881752	2.55E-14	postive
CCS	WHAMMP3	0.347446998	1.24E-15	postive
CCS	WWTR1-AS1	0.385365951	3.78E-19	postive
CCS	YEATS2-AS1	0.364714346	3.55E-17	postive
CCS	YTHDF3-AS1	0.444704007	1.18E-25	postive
CCS	Z69706.1	0.378600946	1.74E-18	postive
CCS	Z69720.1	0.342141232	3.54E-15	postive
CCS	Z83844.2	0.392002633	8.19E-20	postive
CCS	Z83851.4	0.452763469	1.22E-26	postive
CCS	Z84485.1	0.447809376	4.97E-26	postive
CCS	Z84492.1	0.357524737	1.60E-16	postive
CCS	Z92544.1	0.400336194	1.14E-20	postive
CCS	Z92544.2	0.424157191	2.97E-23	postive
CCS	Z94721.1	0.347184039	1.31E-15	postive
CCS	Z95115.1	0.430609835	5.46E-24	postive
CCS	Z97055.2	0.435075372	1.65E-24	postive
CCS	Z97634.1	0.501534711	3.30E-33	postive
CCS	Z97653.1	0.342766602	3.13E-15	postive
CCS	Z97832.2	0.367527798	1.95E-17	postive
CCS	Z98200.2	0.349601428	8.06E-16	postive
CCS	Z98884.2	0.339233439	6.24E-15	postive
CCS	Z98885.2	0.312786398	8.25E-13	postive
CCS	Z99572.1	0.330304655	3.42E-14	postive
CCS	ZBED3-AS1	0.316604638	4.20E-13	postive
CCS	ZBED5-AS1	0.419395973	1.01E-22	postive
CCS	ZBTB11-AS1	0.493113622	5.36E-32	postive
CCS	ZBTB40-IT1	0.310486268	1.23E-12	postive
CCS	ZBTB44-DT	0.493855195	4.21E-32	postive
CCS	ZFHX2-AS1	0.483323514	1.25E-30	postive
CCS	ZKSCAN2-DT	0.493253479	5.12E-32	postive

CCS	ZNF137P	0.35632135	2.05E-16	postive
CCS	ZNF205-AS1	0.533753036	3.65E-38	postive
CCS	ZNF213-AS1	0.580071967	2.69E-46	postive
CCS	ZNF236-DT	0.396837977	2.63E-20	postive
CCS	ZNF252P	0.405593963	3.20E-21	postive
CCS	ZNF252P-AS1	0.409007995	1.38E-21	postive
CCS	ZNF321P	0.356506589	1.98E-16	postive
CCS	ZNF32-AS1	0.421735264	5.57E-23	postive
CCS	ZNF32-AS2	0.440343193	3.95E-25	postive
CCS	ZNF337-AS1	0.451651111	1.67E-26	postive
CCS	ZNF341-AS1	0.407651223	1.93E-21	postive
CCS	ZNF37BP	0.409441961	1.24E-21	postive
CCS	ZNF426-DT	0.415643619	2.63E-22	postive
CCS	ZNF433-AS1	0.306045055	2.66E-12	postive
CCS	ZNF436-AS1	0.489754331	1.60E-31	postive
CCS	ZNF460-AS1	0.417217343	1.77E-22	postive
CCS	ZNF503-AS2	0.438790219	6.04E-25	postive
CCS	ZNF528-AS1	0.377816408	2.07E-18	postive
CCS	ZNF529-AS1	0.386372116	3.00E-19	postive
CCS	ZNF56	0.431357743	4.47E-24	postive
CCS	ZNF561-AS1	0.489727803	1.61E-31	postive
CCS	ZNF582-AS1	0.354985882	2.70E-16	postive
CCS	ZNF674-AS1	0.44855486	4.03E-26	postive
CCS	ZNF687-AS1	0.509770366	2.00E-34	postive
CCS	ZNF767P	0.523923945	1.35E-36	postive
CCS	ZNF815P	0.504279945	1.31E-33	postive
CCS	ZNF890P	0.363423159	4.67E-17	postive
CCS	ZNNT1	0.306286177	2.55E-12	postive
CCS	ZNRD1ASP	0.416815513	1.96E-22	postive
CCS	ZNRD2-AS1	0.65490537	1.44E-62	postive
CCS	ZSCAN16-AS1	0.414419352	3.59E-22	postive
CCS	ZSWIM8-AS1	0.340603355	4.78E-15	postive
CDKN2A	AC002116.2	0.400391392	1.13E-20	postive
CDKN2A	AC003070.2	0.390119056	1.27E-19	postive
CDKN2A	AC003101.2	0.396828326	2.63E-20	postive
CDKN2A	AC004477.1	0.362094336	6.18E-17	postive
CDKN2A	AC004839.2	0.374245944	4.54E-18	postive
CDKN2A	AC005005.3	0.304065261	3.72E-12	postive
CDKN2A	AC005837.1	0.338250464	7.55E-15	postive
CDKN2A	AC006449.5	0.312048647	9.39E-13	postive
CDKN2A	AC007032.1	0.326152949	7.42E-14	postive
CDKN2A	AC007036.1	0.372527509	6.62E-18	postive
CDKN2A	AC007250.1	0.340074411	5.30E-15	postive
CDKN2A	AC007365.1	0.30868251	1.69E-12	postive
CDKN2A	AC007786.1	0.319220372	2.63E-13	postive
CDKN2A	AC007823.1	0.320987022	1.91E-13	postive
CDKN2A	AC008063.1	0.344121658	2.40E-15	postive
CDKN2A	AC008267.5	0.311398083	1.05E-12	postive
CDKN2A	AC008443.5	0.373474901	5.38E-18	postive
CDKN2A	AC008619.1	0.367900392	1.80E-17	postive

CDKN2A	AC008764.6	0.33090442	3.06E-14	postive
CDKN2A	AC008991.1	0.364348644	3.84E-17	postive
CDKN2A	AC009055.2	0.360350697	8.90E-17	postive
CDKN2A	AC009097.2	0.325190641	8.86E-14	postive
CDKN2A	AC009118.2	0.310389395	1.25E-12	postive
CDKN2A	AC009121.3	0.359949992	9.68E-17	postive
CDKN2A	AC009121.4	0.390932686	1.05E-19	postive
CDKN2A	AC009309.2	0.372202717	7.10E-18	postive
CDKN2A	AC009630.2	0.359323196	1.10E-16	postive
CDKN2A	AC009988.1	0.386043459	3.24E-19	postive
CDKN2A	AC010173.1	0.30101173	6.24E-12	postive
CDKN2A	AC010205.1	0.389127249	1.60E-19	postive
CDKN2A	AC010319.5	0.379894264	1.30E-18	postive
CDKN2A	AC010422.2	0.359526902	1.06E-16	postive
CDKN2A	AC010531.6	0.429852059	6.67E-24	postive
CDKN2A	AC010655.2	0.324689091	9.71E-14	postive
CDKN2A	AC010834.2	0.328473897	4.82E-14	postive
CDKN2A	AC010894.2	0.382680707	6.95E-19	postive
CDKN2A	AC010913.1	0.376784091	2.60E-18	postive
CDKN2A	AC011005.4	0.356978588	1.79E-16	postive
CDKN2A	AC011445.2	0.378777404	1.67E-18	postive
CDKN2A	AC011474.1	0.357380785	1.65E-16	postive
CDKN2A	AC011474.2	0.366159942	2.61E-17	postive
CDKN2A	AC011524.1	0.316205591	4.51E-13	postive
CDKN2A	AC012360.3	0.301583176	5.67E-12	postive
CDKN2A	AC012456.2	0.310940582	1.14E-12	postive
CDKN2A	AC013724.1	0.381658897	8.75E-19	postive
CDKN2A	AC015813.4	0.358333156	1.35E-16	postive
CDKN2A	AC015909.2	0.373950918	4.85E-18	postive
CDKN2A	AC015912.3	0.425351366	2.18E-23	postive
CDKN2A	AC015936.1	0.318344994	3.07E-13	postive
CDKN2A	AC015982.2	0.311354693	1.06E-12	postive
CDKN2A	AC016134.1	0.345858615	1.70E-15	postive
CDKN2A	AC016394.2	0.314503857	6.09E-13	postive
CDKN2A	AC016590.3	0.341016657	4.41E-15	postive
CDKN2A	AC016773.2	0.345496579	1.83E-15	postive
CDKN2A	AC017083.1	0.373670328	5.15E-18	postive
CDKN2A	AC019069.1	0.351950523	5.02E-16	postive
CDKN2A	AC020765.2	0.352200174	4.77E-16	postive
CDKN2A	AC022167.4	0.328270383	5.01E-14	postive
CDKN2A	AC022784.6	0.300632443	6.65E-12	postive
CDKN2A	AC022915.1	0.316214274	4.50E-13	postive
CDKN2A	AC023813.3	0.32260104	1.42E-13	postive
CDKN2A	AC023906.5	0.394823032	4.23E-20	postive
CDKN2A	AC023983.2	0.458722015	2.18E-27	postive
CDKN2A	AC024361.2	0.306903127	2.29E-12	postive
CDKN2A	AC025048.2	0.329195117	4.22E-14	postive
CDKN2A	AC026304.1	0.4444527	1.27E-25	postive
CDKN2A	AC026316.2	0.310116241	1.32E-12	postive
CDKN2A	AC026333.4	0.313795816	6.91E-13	postive

CDKN2A	AC026401.3	0.321361189	1.78E-13	postive
CDKN2A	AC027559.1	0.319115977	2.68E-13	postive
CDKN2A	AC046143.2	0.360726506	8.23E-17	postive
CDKN2A	AC068831.2	0.344903047	2.06E-15	postive
CDKN2A	AC069213.1	0.30118628	6.06E-12	postive
CDKN2A	AC069544.1	0.348497929	1.01E-15	postive
CDKN2A	AC073195.2	0.31564431	4.98E-13	postive
CDKN2A	AC073352.1	0.364594529	3.64E-17	postive
CDKN2A	AC073529.1	0.34927645	8.61E-16	postive
CDKN2A	AC073842.2	0.315786708	4.85E-13	postive
CDKN2A	AC073863.1	0.37904862	1.57E-18	postive
CDKN2A	AC073869.1	0.324184143	1.07E-13	postive
CDKN2A	AC074044.1	0.307086316	2.22E-12	postive
CDKN2A	AC078880.3	0.38517573	3.95E-19	postive
CDKN2A	AC078880.4	0.346989799	1.36E-15	postive
CDKN2A	AC078880.5	0.378127744	1.93E-18	postive
CDKN2A	AC079209.1	0.333685791	1.81E-14	postive
CDKN2A	AC079313.2	0.372666246	6.42E-18	postive
CDKN2A	AC079922.2	0.47294342	3.13E-29	postive
CDKN2A	AC080013.5	0.433998903	2.21E-24	postive
CDKN2A	AC080129.2	0.347109285	1.33E-15	postive
CDKN2A	AC082651.1	0.375761519	3.26E-18	postive
CDKN2A	AC083799.1	0.310869919	1.15E-12	postive
CDKN2A	AC083880.1	0.395782444	3.37E-20	postive
CDKN2A	AC084757.3	0.39993578	1.26E-20	postive
CDKN2A	AC087379.2	0.336978958	9.64E-15	postive
CDKN2A	AC090155.2	0.30934865	1.50E-12	postive
CDKN2A	AC090193.1	0.310991859	1.13E-12	postive
CDKN2A	AC090692.1	0.384798695	4.30E-19	postive
CDKN2A	AC090970.2	0.429006773	8.35E-24	postive
CDKN2A	AC091114.1	0.367809648	1.83E-17	postive
CDKN2A	AC091271.1	0.311886097	9.66E-13	postive
CDKN2A	AC092295.2	0.327880441	5.39E-14	postive
CDKN2A	AC092431.1	0.328246751	5.03E-14	postive
CDKN2A	AC092718.4	0.354533841	2.96E-16	postive
CDKN2A	AC092803.1	0.37303479	5.92E-18	postive
CDKN2A	AC092910.3	0.308297129	1.80E-12	postive
CDKN2A	AC093916.1	0.324258445	1.05E-13	postive
CDKN2A	AC096543.2	0.396096424	3.13E-20	postive
CDKN2A	AC097358.2	0.375070103	3.79E-18	postive
CDKN2A	AC098484.2	0.302390974	4.95E-12	postive
CDKN2A	AC099343.4	0.327764031	5.50E-14	postive
CDKN2A	AC099522.2	0.326609105	6.82E-14	postive
CDKN2A	AC099786.1	0.33776466	8.29E-15	postive
CDKN2A	AC099786.2	0.342183394	3.51E-15	postive
CDKN2A	AC099791.2	0.327413359	5.87E-14	postive
CDKN2A	AC099850.2	0.305966473	2.69E-12	postive
CDKN2A	AC099850.4	0.337239369	9.17E-15	postive
CDKN2A	AC100803.2	0.367534862	1.95E-17	postive
CDKN2A	AC100812.1	0.355811368	2.28E-16	postive

CDKN2A	AC103770.1	0.36423508	3.93E-17	postive
CDKN2A	AC103855.2	0.328963994	4.40E-14	postive
CDKN2A	AC103925.1	0.302284068	5.04E-12	postive
CDKN2A	AC104435.2	0.365938588	2.74E-17	postive
CDKN2A	AC104777.1	0.355447595	2.46E-16	postive
CDKN2A	AC105137.2	0.337478	8.76E-15	postive
CDKN2A	AC106706.1	0.371044976	9.13E-18	postive
CDKN2A	AC106795.1	0.313020313	7.92E-13	postive
CDKN2A	AC106897.2	0.334365488	1.59E-14	postive
CDKN2A	AC107214.1	0.31449629	6.10E-13	postive
CDKN2A	AC108047.1	0.349189584	8.76E-16	postive
CDKN2A	AC108673.2	0.320247172	2.18E-13	postive
CDKN2A	AC108676.1	0.318995109	2.73E-13	postive
CDKN2A	AC114271.1	0.355189443	2.59E-16	postive
CDKN2A	AC120498.4	0.359250465	1.12E-16	postive
CDKN2A	AC124016.2	0.32924705	4.17E-14	postive
CDKN2A	AC128687.2	0.384707521	4.39E-19	postive
CDKN2A	AC128709.2	0.332678532	2.19E-14	postive
CDKN2A	AC131235.3	0.393204591	6.18E-20	postive
CDKN2A	AC131235.4	0.334002827	1.70E-14	postive
CDKN2A	AC132008.2	0.394740873	4.31E-20	postive
CDKN2A	AC137056.1	0.321062626	1.88E-13	postive
CDKN2A	AC137630.3	0.319715573	2.40E-13	postive
CDKN2A	AC138123.1	0.34749451	1.23E-15	postive
CDKN2A	AC144652.1	0.311049276	1.12E-12	postive
CDKN2A	AC233280.1	0.39149556	9.21E-20	postive
CDKN2A	AC243571.1	0.358985134	1.18E-16	postive
CDKN2A	AC243965.2	0.303738213	3.94E-12	postive
CDKN2A	AD000671.3	0.310085345	1.32E-12	postive
CDKN2A	AF274858.1	0.326644486	6.77E-14	postive
CDKN2A	AF279873.4	0.36905879	1.40E-17	postive
CDKN2A	AJ009632.2	0.362216263	6.02E-17	postive
CDKN2A	AL008729.2	0.344783079	2.11E-15	postive
CDKN2A	AL023803.1	0.497952864	1.09E-32	postive
CDKN2A	AL024498.1	0.31078381	1.17E-12	postive
CDKN2A	AL024507.3	0.432198101	3.58E-24	postive
CDKN2A	AL031055.1	0.391921101	8.34E-20	postive
CDKN2A	AL031429.1	0.367588384	1.92E-17	postive
CDKN2A	AL031432.3	0.309184843	1.55E-12	postive
CDKN2A	AL035461.3	0.346683338	1.45E-15	postive
CDKN2A	AL050341.2	0.303403294	4.17E-12	postive
CDKN2A	AL078590.2	0.334013832	1.70E-14	postive
CDKN2A	AL109918.1	0.312660931	8.43E-13	postive
CDKN2A	AL117332.1	0.422606842	4.44E-23	postive
CDKN2A	AL117335.1	0.344639852	2.17E-15	postive
CDKN2A	AL118523.1	0.303754772	3.93E-12	postive
CDKN2A	AL121574.1	0.316924824	3.96E-13	postive
CDKN2A	AL121601.1	0.390289262	1.22E-19	postive
CDKN2A	AL121820.2	0.401524724	8.57E-21	postive
CDKN2A	AL121832.2	0.353551539	3.62E-16	postive

CDKN2A	AL121906.1	0.335868277	1.19E-14	postive
CDKN2A	AL121970.1	0.336428825	1.07E-14	postive
CDKN2A	AL133342.1	0.300872249	6.39E-12	postive
CDKN2A	AL135744.1	0.474740702	1.81E-29	postive
CDKN2A	AL135785.1	0.315654958	4.97E-13	postive
CDKN2A	AL136172.1	0.308385547	1.78E-12	postive
CDKN2A	AL136366.1	0.404674013	4.00E-21	postive
CDKN2A	AL137060.3	0.335287882	1.33E-14	postive
CDKN2A	AL137224.1	0.353242951	3.86E-16	postive
CDKN2A	AL138724.2	0.367729244	1.87E-17	postive
CDKN2A	AL139260.1	0.496446358	1.80E-32	postive
CDKN2A	AL139274.2	0.30847408	1.75E-12	postive
CDKN2A	AL161640.2	0.307426427	2.10E-12	postive
CDKN2A	AL161719.1	0.31104429	1.12E-12	postive
CDKN2A	AL161937.1	0.377571612	2.18E-18	postive
CDKN2A	AL162377.1	0.301976287	5.31E-12	postive
CDKN2A	AL353751.1	0.305188591	3.08E-12	postive
CDKN2A	AL354754.1	0.390518952	1.16E-19	postive
CDKN2A	AL354766.2	0.31327963	7.56E-13	postive
CDKN2A	AL354864.1	0.437907663	7.68E-25	postive
CDKN2A	AL354928.1	0.302573946	4.80E-12	postive
CDKN2A	AL355300.1	0.370308995	1.07E-17	postive
CDKN2A	AL355574.1	0.317279873	3.72E-13	postive
CDKN2A	AL355607.2	0.322123367	1.55E-13	postive
CDKN2A	AL356966.1	0.309861607	1.38E-12	postive
CDKN2A	AL357033.2	0.371876807	7.62E-18	postive
CDKN2A	AL357140.2	0.30551551	2.91E-12	postive
CDKN2A	AL357992.1	0.337830886	8.19E-15	postive
CDKN2A	AL358852.1	0.305598338	2.87E-12	postive
CDKN2A	AL359504.2	0.385559447	3.62E-19	postive
CDKN2A	AL359513.1	0.368288484	1.66E-17	postive
CDKN2A	AL441992.2	0.361080168	7.64E-17	postive
CDKN2A	AL445228.3	0.373488367	5.36E-18	postive
CDKN2A	AL449423.1	0.551955056	3.29E-41	postive
CDKN2A	AL451050.2	0.386071844	3.22E-19	postive
CDKN2A	AL512604.4	0.416105232	2.34E-22	postive
CDKN2A	AL512625.2	0.352460876	4.52E-16	postive
CDKN2A	AL513165.1	0.324693095	9.71E-14	postive
CDKN2A	AL590428.1	0.367136886	2.12E-17	postive
CDKN2A	AL590617.2	0.320757543	1.99E-13	postive
CDKN2A	AL590705.1	0.326171943	7.39E-14	postive
CDKN2A	AL596094.1	0.382734359	6.87E-19	postive
CDKN2A	AL603839.2	0.304573751	3.42E-12	postive
CDKN2A	AP000251.1	0.448326375	4.29E-26	postive
CDKN2A	AP000640.2	0.406985339	2.27E-21	postive
CDKN2A	AP000880.1	0.359589131	1.04E-16	postive
CDKN2A	AP001160.2	0.319206378	2.63E-13	postive
CDKN2A	AP001189.5	0.357680741	1.55E-16	postive
CDKN2A	AP001636.3	0.359547999	1.05E-16	postive
CDKN2A	AP002008.3	0.392130067	7.95E-20	postive

CDKN2A	AP002360.2	0.329255447	4.17E-14	postive
CDKN2A	AP002387.2	0.302498334	4.86E-12	postive
CDKN2A	AP002802.1	0.393056478	6.40E-20	postive
CDKN2A	AP003119.2	0.397173772	2.43E-20	postive
CDKN2A	AP006216.1	0.35256991	4.42E-16	postive
CDKN2A	AP006621.3	0.397668274	2.16E-20	postive
CDKN2A	ARRDC1-AS1	0.358337423	1.35E-16	postive
CDKN2A	ASB16-AS1	0.32854978	4.75E-14	postive
CDKN2A	ATP2A1-AS1	0.35717901	1.72E-16	postive
CDKN2A	BVES-AS1	0.328105941	5.16E-14	postive
CDKN2A	BX470209.1	0.387155839	2.51E-19	postive
CDKN2A	BX470209.2	0.36971527	1.22E-17	postive
CDKN2A	BX649601.1	0.306152668	2.61E-12	postive
CDKN2A	C22orf24	0.338274753	7.51E-15	postive
CDKN2A	C6orf99	0.309425678	1.48E-12	postive
CDKN2A	CAMTA1-DT	0.308549602	1.73E-12	postive
CDKN2A	CDKN2A-DT	0.721805774	1.25E-81	postive
CDKN2A	CDKN2B-AS1	0.373961395	4.84E-18	postive
CDKN2A	CEACAMP10	0.357561316	1.59E-16	postive
CDKN2A	CEBPB-AS1	0.321691161	1.68E-13	postive
CDKN2A	CEP83-DT	0.390023426	1.30E-19	postive
CDKN2A	CT66	0.330723302	3.17E-14	postive
CDKN2A	CT75	0.413106008	4.99E-22	postive
CDKN2A	CTD-2540M10.1	0.476593826	1.02E-29	postive
CDKN2A	CYP4F23P	0.390294451	1.22E-19	postive
CDKN2A	DDX11-AS1	0.445314112	9.98E-26	postive
CDKN2A	DEPDC1-AS1	0.380123164	1.24E-18	postive
CDKN2A	DNAJC3-DT	0.315560468	5.05E-13	postive
CDKN2A	DUXAP9	0.323110832	1.30E-13	postive
CDKN2A	ELDR	0.30982868	1.38E-12	postive
CDKN2A	EMC3-AS1	0.390053265	1.29E-19	postive
CDKN2A	ERVMER61-1	0.306968877	2.27E-12	postive
CDKN2A	EXOC3-AS1	0.318916079	2.77E-13	postive
CDKN2A	EXOSC10-AS1	0.376420903	2.81E-18	postive
CDKN2A	FAM111A-DT	0.333692286	1.81E-14	postive
CDKN2A	FAM157A	0.302218316	5.09E-12	postive
CDKN2A	FAM197Y9	0.301743116	5.52E-12	postive
CDKN2A	FAM86JP	0.329338566	4.10E-14	postive
CDKN2A	FBP2P1	0.372404895	6.79E-18	postive
CDKN2A	FO393418.1	0.368718597	1.51E-17	postive
CDKN2A	FOXD2-AS1	0.361182228	7.48E-17	postive
CDKN2A	FOXD3-AS1	0.436475734	1.13E-24	postive
CDKN2A	GBP1P1	0.338961998	6.58E-15	postive
CDKN2A	GIHCG	0.315666596	4.96E-13	postive
CDKN2A	GOLGA8EP	0.347097691	1.33E-15	postive
CDKN2A	GPR176-DT	0.339337101	6.12E-15	postive
CDKN2A	HMMR-AS1	0.329498575	3.98E-14	postive
CDKN2A	HOMER3-AS1	0.32387437	1.13E-13	postive
CDKN2A	IGFL2-AS1	0.301172952	6.07E-12	postive
CDKN2A	IPO9-AS1	0.402606794	6.60E-21	postive

CDKN2A	KCNQ1DN	0.445413284	9.71E-26	postive
CDKN2A	KDM2B-DT	0.366236319	2.57E-17	postive
CDKN2A	KDM4A-AS1	0.321716706	1.67E-13	postive
CDKN2A	KMT2E-AS1	0.353823124	3.43E-16	postive
CDKN2A	KRT8P12	0.473480237	2.66E-29	postive
CDKN2A	LAMA5-AS1	0.405005003	3.69E-21	postive
CDKN2A	LAPTM4A-DT	0.328060475	5.21E-14	postive
CDKN2A	LGALS8-AS1	0.366159656	2.61E-17	postive
CDKN2A	LINC00323	0.333115215	2.02E-14	postive
CDKN2A	LINC00346	0.339572771	5.84E-15	postive
CDKN2A	LINC00449	0.30485194	3.26E-12	postive
CDKN2A	LINC00466	0.404074226	4.63E-21	postive
CDKN2A	LINC00471	0.337871359	8.12E-15	postive
CDKN2A	LINC00494	0.336263267	1.11E-14	postive
CDKN2A	LINC00652	0.314444142	6.16E-13	postive
CDKN2A	LINC00847	0.330451134	3.33E-14	postive
CDKN2A	LINC00884	0.441907936	2.57E-25	postive
CDKN2A	LINC00885	0.407698704	1.91E-21	postive
CDKN2A	LINC00887	0.306721289	2.37E-12	postive
CDKN2A	LINC01063	0.494019612	3.99E-32	postive
CDKN2A	LINC01122	0.361180259	7.48E-17	postive
CDKN2A	LINC01132	0.300223275	7.12E-12	postive
CDKN2A	LINC01151	0.386633403	2.83E-19	postive
CDKN2A	LINC01224	0.306288937	2.55E-12	postive
CDKN2A	LINC01232	0.347507598	1.23E-15	postive
CDKN2A	LINC01273	0.351595553	5.39E-16	postive
CDKN2A	LINC01275	0.427155106	1.36E-23	postive
CDKN2A	LINC01285	0.391698425	8.79E-20	postive
CDKN2A	LINC01293	0.322687722	1.40E-13	postive
CDKN2A	LINC01305	0.463292261	5.71E-28	postive
CDKN2A	LINC01311	0.316217444	4.50E-13	postive
CDKN2A	LINC01354	0.389023732	1.63E-19	postive
CDKN2A	LINC01366	0.361568887	6.90E-17	postive
CDKN2A	LINC01387	0.356959538	1.80E-16	postive
CDKN2A	LINC01497	0.374953442	3.89E-18	postive
CDKN2A	LINC01521	0.307192912	2.18E-12	postive
CDKN2A	LINC01567	0.357020721	1.78E-16	postive
CDKN2A	LINC01621	0.394345775	4.73E-20	postive
CDKN2A	LINC01686	0.380751142	1.07E-18	postive
CDKN2A	LINC01693	0.386819991	2.71E-19	postive
CDKN2A	LINC01771	0.319024873	2.72E-13	postive
CDKN2A	LINC01775	0.377605745	2.16E-18	postive
CDKN2A	LINC01842	0.36973224	1.21E-17	postive
CDKN2A	LINC01952	0.398553637	1.75E-20	postive
CDKN2A	LINC01968	0.307398253	2.11E-12	postive
CDKN2A	LINC01972	0.35741045	1.64E-16	postive
CDKN2A	LINC02068	0.33669976	1.02E-14	postive
CDKN2A	LINC02076	0.427012059	1.41E-23	postive
CDKN2A	LINC02130	0.325691336	8.08E-14	postive
CDKN2A	LINC02137	0.383126027	6.29E-19	postive

CDKN2A	LINC02321	0.331091675	2.95E-14	postive
CDKN2A	LINC02403	0.305496194	2.92E-12	postive
CDKN2A	LINC02412	0.36875362	1.50E-17	postive
CDKN2A	LINC02601	0.32080497	1.97E-13	postive
CDKN2A	LINC02707	0.355878712	2.25E-16	postive
CDKN2A	LINC02709	0.352016488	4.95E-16	postive
CDKN2A	LINC02740	0.34360094	2.66E-15	postive
CDKN2A	LINC02745	0.337831587	8.18E-15	postive
CDKN2A	LINC02757	0.333719607	1.80E-14	postive
CDKN2A	LINC02771	0.31481244	5.77E-13	postive
CDKN2A	LMNB1-DT	0.334443493	1.57E-14	postive
CDKN2A	LPP-AS2	0.455783639	5.12E-27	postive
CDKN2A	MAP3K2-DT	0.373081507	5.86E-18	postive
CDKN2A	MAPK6-DT	0.40707646	2.22E-21	postive
CDKN2A	MELTF-AS1	0.523251533	1.72E-36	postive
CDKN2A	MIR3150BHG	0.398472927	1.78E-20	postive
CDKN2A	MIR378D2HG	0.301774562	5.49E-12	postive
CDKN2A	MIR4435-2HG	0.303185432	4.32E-12	postive
CDKN2A	MIR924HG	0.393018805	6.46E-20	postive
CDKN2A	MT1DP	0.3469553	1.37E-15	postive
CDKN2A	MYLK-AS1	0.308231639	1.82E-12	postive
CDKN2A	NQO2-AS1	0.30635786	2.52E-12	postive
CDKN2A	NUP50-DT	0.317088105	3.85E-13	postive
CDKN2A	OR4G11P	0.369001498	1.42E-17	postive
CDKN2A	OR4G2P	0.341103681	4.34E-15	postive
CDKN2A	OR7E161P	0.354074068	3.26E-16	postive
CDKN2A	OXCT1-AS1	0.35398688	3.31E-16	postive
CDKN2A	PAUPAR	0.310339896	1.27E-12	postive
CDKN2A	PCCA-DT	0.303461368	4.13E-12	postive
CDKN2A	PPP1R35-AS1	0.316239487	4.48E-13	postive
CDKN2A	RABGAP1L-DT	0.322660908	1.41E-13	postive
CDKN2A	RAET1K	0.308191088	1.84E-12	postive
CDKN2A	RAMP2-AS1	0.365461295	3.03E-17	postive
CDKN2A	RAP2CP1	0.414772692	3.28E-22	postive
CDKN2A	RBMY2QP	0.374148368	4.64E-18	postive
CDKN2A	RN7SL832P	0.313032516	7.90E-13	postive
CDKN2A	RP11-115J16.1	0.367355524	2.02E-17	postive
CDKN2A	RP11-160O5.1	0.338316794	7.45E-15	postive
CDKN2A	RP11-254F7.4	0.343366364	2.79E-15	postive
CDKN2A	RP11-435O5.2	0.339873429	5.51E-15	postive
CDKN2A	RP11-98G7.1	0.356178825	2.11E-16	postive
CDKN2A	RP4-545L17.11	0.345718893	1.75E-15	postive
CDKN2A	RP4-784A16.3	0.352701544	4.31E-16	postive
CDKN2A	RPSAP69	0.309751789	1.40E-12	postive
CDKN2A	SAP30-DT	0.322415154	1.47E-13	postive
CDKN2A	SDHAP1	0.301017159	6.23E-12	postive
CDKN2A	SNHG10	0.343495123	2.72E-15	postive
CDKN2A	SNHG30	0.399329429	1.45E-20	postive
CDKN2A	SOX2-OT	0.430203754	6.08E-24	postive
CDKN2A	ST3GAL6-AS1	0.319244336	2.61E-13	postive

CDKN2A	STK24-AS1	0.387737049	2.20E-19	postive
CDKN2A	TFAP2A-AS1	0.401241861	9.18E-21	postive
CDKN2A	TM4SF19-AS1	0.356864329	1.84E-16	postive
CDKN2A	TMCC1-AS1	0.37667096	2.66E-18	postive
CDKN2A	TMEM44-AS1	0.446966967	6.29E-26	postive
CDKN2A	TMPO-AS1	0.469238623	9.65E-29	postive
CDKN2A	TPI1P2	0.319559448	2.47E-13	postive
CDKN2A	TPRG1-AS1	0.331582191	2.69E-14	postive
CDKN2A	TTTY20	0.356255553	2.08E-16	postive
CDKN2A	TUBBP9	0.36145452	7.06E-17	postive
CDKN2A	TYMSOS	0.407401199	2.05E-21	postive
CDKN2A	UBAC2-AS1	0.338780888	6.81E-15	postive
CDKN2A	URB1-AS1	0.324462445	1.01E-13	postive
CDKN2A	VPS33B-DT	0.316011588	4.66E-13	postive
CDKN2A	WWTR1-AS1	0.38457928	4.52E-19	postive
CDKN2A	Z82185.1	0.310703822	1.19E-12	postive
CDKN2A	Z82246.1	0.32151525	1.73E-13	postive
CDKN2A	Z92544.1	0.360849952	8.02E-17	postive
CDKN2A	Z97056.1	0.322432425	1.47E-13	postive
CDKN2A	ZEB1-AS1	0.320651949	2.03E-13	postive
CDKN2A	ZNF529-AS1	0.36976615	1.20E-17	postive
CDKN2A	ZNF687-AS1	0.321465755	1.75E-13	postive
CNNM1	AC003965.2	0.358172549	1.40E-16	postive
CNNM1	AC006262.2	0.336246143	1.11E-14	postive
CNNM1	AC009275.1	0.344730263	2.13E-15	postive
CNNM1	AC023158.1	0.408981444	1.39E-21	postive
CNNM1	AC023158.2	0.401146191	9.39E-21	postive
CNNM1	AC025366.1	0.357550231	1.59E-16	postive
CNNM1	AC120498.4	0.321197057	1.84E-13	postive
CNNM1	AL050403.1	0.301132454	6.12E-12	postive
CNNM1	AL450992.2	0.301595051	5.66E-12	postive
CNNM1	AL596223.1	0.301533295	5.72E-12	postive
CNNM1	AL606489.1	0.332256589	2.37E-14	postive
CNNM1	C20orf187	0.342441621	3.34E-15	postive
CNNM1	FOXC2-AS1	0.304673219	3.36E-12	postive
CNNM1	LINC00603	0.355520873	2.42E-16	postive
CNNM1	LINC00920	0.318829218	2.82E-13	postive
CNNM1	LINC02747	0.336607525	1.04E-14	postive
CNNM1	LNCAROD	0.333442014	1.89E-14	postive
CNNM1	MRPS30-DT	0.317265924	3.73E-13	postive
CNNM1	PSMD10P2	0.336707606	1.02E-14	postive
CNNM1	SLC9A7P1	0.322285483	1.51E-13	postive
COMMD1	A1BG-AS1	0.344902311	2.06E-15	postive
COMMD1	A2M-AS1	0.308888916	1.63E-12	postive
COMMD1	ABHD11-AS1	0.4037927	4.95E-21	postive
COMMD1	AC002116.2	0.377186851	2.38E-18	postive
COMMD1	AC002310.1	0.361195571	7.46E-17	postive
COMMD1	AC002467.1	0.551880981	3.39E-41	postive
COMMD1	AC002470.1	0.400282039	1.16E-20	postive
COMMD1	AC003965.1	0.325553023	8.29E-14	postive

COMMD1	AC004130.2	0.320030794	2.27E-13	postive
COMMD1	AC004241.1	0.440629028	3.65E-25	postive
COMMD1	AC004241.3	0.34189898	3.72E-15	postive
COMMD1	AC004477.1	0.305775581	2.78E-12	postive
COMMD1	AC004540.1	0.334918895	1.43E-14	postive
COMMD1	AC004771.2	0.307441254	2.09E-12	postive
COMMD1	AC004803.1	0.314001468	6.66E-13	postive
COMMD1	AC004812.2	0.366655526	2.35E-17	postive
COMMD1	AC004825.2	0.311917807	9.61E-13	postive
COMMD1	AC004846.1	0.391896215	8.39E-20	postive
COMMD1	AC004854.2	0.345768216	1.73E-15	postive
COMMD1	AC004921.1	0.328402729	4.89E-14	postive
COMMD1	AC004943.2	0.323008358	1.32E-13	postive
COMMD1	AC005034.4	0.351451629	5.55E-16	postive
COMMD1	AC005041.3	0.319278139	2.60E-13	postive
COMMD1	AC005076.2	0.389817791	1.36E-19	postive
COMMD1	AC005083.1	0.340619922	4.77E-15	postive
COMMD1	AC005229.4	0.427465405	1.25E-23	postive
COMMD1	AC005498.2	0.397631841	2.18E-20	postive
COMMD1	AC005520.4	0.459842989	1.57E-27	postive
COMMD1	AC005562.1	0.491220967	9.93E-32	postive
COMMD1	AC005670.3	0.383335736	5.99E-19	postive
COMMD1	AC005790.1	0.312246148	9.07E-13	postive
COMMD1	AC005837.1	0.363744317	4.36E-17	postive
COMMD1	AC006122.1	0.317486592	3.58E-13	postive
COMMD1	AC006213.1	0.382929247	6.57E-19	postive
COMMD1	AC006252.1	0.328512756	4.79E-14	postive
COMMD1	AC006333.1	0.524387434	1.14E-36	postive
COMMD1	AC006449.2	0.386437766	2.96E-19	postive
COMMD1	AC006449.5	0.331909412	2.53E-14	postive
COMMD1	AC006449.7	0.525135157	8.72E-37	postive
COMMD1	AC007032.1	0.387906678	2.11E-19	postive
COMMD1	AC007114.1	0.371347168	8.55E-18	postive
COMMD1	AC007342.4	0.3084027	1.77E-12	postive
COMMD1	AC007383.2	0.342762982	3.14E-15	postive
COMMD1	AC007389.1	0.313316943	7.51E-13	postive
COMMD1	AC007541.1	0.354909777	2.74E-16	postive
COMMD1	AC007773.1	0.306066499	2.65E-12	postive
COMMD1	AC007879.2	0.301347373	5.90E-12	postive
COMMD1	AC007881.3	0.361234008	7.40E-17	postive
COMMD1	AC007998.3	0.367012742	2.18E-17	postive
COMMD1	AC008014.1	0.492193587	7.24E-32	postive
COMMD1	AC008035.1	0.386337458	3.03E-19	postive
COMMD1	AC008105.2	0.408349351	1.63E-21	postive
COMMD1	AC008267.5	0.408288936	1.65E-21	postive
COMMD1	AC008393.1	0.374935471	3.90E-18	postive
COMMD1	AC008438.1	0.397792086	2.09E-20	postive
COMMD1	AC008443.1	0.375293592	3.61E-18	postive
COMMD1	AC008443.5	0.410271261	1.01E-21	postive
COMMD1	AC008494.3	0.41060244	9.31E-22	postive

COMMD1	AC008608.2	0.460521406	1.29E-27	postive
COMMD1	AC008747.1	0.389387824	1.50E-19	postive
COMMD1	AC008771.1	0.461104137	1.09E-27	postive
COMMD1	AC008915.3	0.558008728	2.90E-42	postive
COMMD1	AC008946.1	0.398393917	1.81E-20	postive
COMMD1	AC008966.1	0.464182501	4.38E-28	postive
COMMD1	AC009005.1	0.310786296	1.17E-12	postive
COMMD1	AC009061.2	0.459902661	1.55E-27	postive
COMMD1	AC009126.1	0.386951951	2.63E-19	postive
COMMD1	AC009171.2	0.375523403	3.43E-18	postive
COMMD1	AC009237.14	0.486686725	4.28E-31	postive
COMMD1	AC009309.2	0.459022462	2.00E-27	postive
COMMD1	AC009506.2	0.446699652	6.78E-26	postive
COMMD1	AC009630.1	0.305332882	3.00E-12	postive
COMMD1	AC009690.2	0.301585257	5.67E-12	postive
COMMD1	AC009779.3	0.469074547	1.01E-28	postive
COMMD1	AC009831.1	0.392200299	7.82E-20	postive
COMMD1	AC009948.3	0.342992544	3.00E-15	postive
COMMD1	AC009961.1	0.301803489	5.46E-12	postive
COMMD1	AC010173.1	0.419385054	1.02E-22	postive
COMMD1	AC010186.2	0.426258709	1.72E-23	postive
COMMD1	AC010226.1	0.377703917	2.12E-18	postive
COMMD1	AC010273.3	0.328576358	4.73E-14	postive
COMMD1	AC010280.2	0.375406172	3.52E-18	postive
COMMD1	AC010320.2	0.373364388	5.51E-18	postive
COMMD1	AC010442.1	0.363690328	4.41E-17	postive
COMMD1	AC010504.1	0.431471872	4.34E-24	postive
COMMD1	AC010655.4	0.381417492	9.24E-19	postive
COMMD1	AC010894.2	0.410954536	8.53E-22	postive
COMMD1	AC010913.1	0.308746347	1.67E-12	postive
COMMD1	AC010969.2	0.342840837	3.09E-15	postive
COMMD1	AC011290.1	0.314332648	6.28E-13	postive
COMMD1	AC011451.1	0.330056378	3.59E-14	postive
COMMD1	AC011731.1	0.311616146	1.01E-12	postive
COMMD1	AC012063.1	0.366161524	2.61E-17	postive
COMMD1	AC012184.3	0.336810878	9.96E-15	postive
COMMD1	AC012306.2	0.443715708	1.56E-25	postive
COMMD1	AC012360.2	0.328250506	5.03E-14	postive
COMMD1	AC012442.3	0.478388681	5.85E-30	postive
COMMD1	AC012555.1	0.312981461	7.97E-13	postive
COMMD1	AC012603.1	0.31815224	3.18E-13	postive
COMMD1	AC012615.1	0.364045931	4.09E-17	postive
COMMD1	AC012640.4	0.529661226	1.66E-37	postive
COMMD1	AC015922.2	0.33902555	6.50E-15	postive
COMMD1	AC015922.3	0.453037865	1.13E-26	postive
COMMD1	AC015982.2	0.513730248	5.06E-35	postive
COMMD1	AC016027.1	0.40025926	1.16E-20	postive
COMMD1	AC016065.1	0.423968438	3.12E-23	postive
COMMD1	AC016355.1	0.35657789	1.95E-16	postive
COMMD1	AC016394.2	0.436362696	1.17E-24	postive

COMMD1	AC016727.1	0.434468485	1.95E-24	postive
COMMD1	AC016747.1	0.538874042	5.30E-39	postive
COMMD1	AC016876.2	0.444899264	1.12E-25	postive
COMMD1	AC016876.3	0.38612099	3.18E-19	postive
COMMD1	AC016888.1	0.360650301	8.36E-17	postive
COMMD1	AC018521.5	0.605675964	2.22E-51	postive
COMMD1	AC018647.2	0.337815415	8.21E-15	postive
COMMD1	AC019131.2	0.305906464	2.72E-12	postive
COMMD1	AC020663.2	0.443762637	1.54E-25	postive
COMMD1	AC020765.2	0.332397126	2.31E-14	postive
COMMD1	AC020910.5	0.39202663	8.14E-20	postive
COMMD1	AC020915.3	0.374418992	4.37E-18	postive
COMMD1	AC021016.2	0.331749417	2.61E-14	postive
COMMD1	AC022092.1	0.341037161	4.40E-15	postive
COMMD1	AC022098.3	0.339417109	6.02E-15	postive
COMMD1	AC022167.4	0.344453402	2.25E-15	postive
COMMD1	AC022211.3	0.304893424	3.23E-12	postive
COMMD1	AC022613.2	0.331010259	3.00E-14	postive
COMMD1	AC022706.1	0.306359125	2.52E-12	postive
COMMD1	AC022916.2	0.360350783	8.90E-17	postive
COMMD1	AC023043.1	0.340168946	5.21E-15	postive
COMMD1	AC023509.3	0.380520453	1.13E-18	postive
COMMD1	AC023813.3	0.305676956	2.83E-12	postive
COMMD1	AC024243.1	0.377878476	2.04E-18	postive
COMMD1	AC024361.2	0.395561732	3.55E-20	postive
COMMD1	AC024575.2	0.387541884	2.30E-19	postive
COMMD1	AC024941.2	0.315147196	5.44E-13	postive
COMMD1	AC025048.4	0.500481269	4.70E-33	postive
COMMD1	AC025181.2	0.309496353	1.47E-12	postive
COMMD1	AC025287.2	0.305438786	2.95E-12	postive
COMMD1	AC025682.2	0.318106988	3.21E-13	postive
COMMD1	AC025754.2	0.303776653	3.91E-12	postive
COMMD1	AC026304.1	0.41553926	2.70E-22	postive
COMMD1	AC026412.1	0.373729774	5.09E-18	postive
COMMD1	AC026471.1	0.423252563	3.76E-23	postive
COMMD1	AC026471.2	0.301970767	5.31E-12	postive
COMMD1	AC026691.1	0.365601637	2.94E-17	postive
COMMD1	AC026801.2	0.409704512	1.16E-21	postive
COMMD1	AC026979.2	0.307060523	2.23E-12	postive
COMMD1	AC027097.1	0.362030843	6.26E-17	postive
COMMD1	AC027644.3	0.425656338	2.01E-23	postive
COMMD1	AC034199.1	0.336296398	1.10E-14	postive
COMMD1	AC036176.1	0.510452454	1.58E-34	postive
COMMD1	AC040970.1	0.350802625	6.33E-16	postive
COMMD1	AC046134.2	0.422816619	4.21E-23	postive
COMMD1	AC053503.1	0.340374757	5.00E-15	postive
COMMD1	AC053503.4	0.377734516	2.10E-18	postive
COMMD1	AC060766.7	0.334582094	1.53E-14	postive
COMMD1	AC061992.2	0.374165815	4.62E-18	postive
COMMD1	AC064807.1	0.46909758	1.01E-28	postive

COMMD1	AC064836.3	0.351472143	5.53E-16	postive
COMMD1	AC067852.2	0.532195088	6.52E-38	postive
COMMD1	AC068305.2	0.303093395	4.39E-12	postive
COMMD1	AC068338.2	0.480948115	2.63E-30	postive
COMMD1	AC068473.5	0.308927225	1.62E-12	postive
COMMD1	AC068620.1	0.394447526	4.62E-20	postive
COMMD1	AC068790.8	0.433972051	2.22E-24	postive
COMMD1	AC068870.2	0.409137124	1.34E-21	postive
COMMD1	AC069307.1	0.331744428	2.61E-14	postive
COMMD1	AC069544.1	0.307788956	1.97E-12	postive
COMMD1	AC073195.2	0.346353437	1.54E-15	postive
COMMD1	AC073508.3	0.451504867	1.75E-26	postive
COMMD1	AC073529.1	0.438988292	5.72E-25	postive
COMMD1	AC073611.1	0.431964741	3.81E-24	postive
COMMD1	AC073896.2	0.502287184	2.56E-33	postive
COMMD1	AC074044.1	0.319769162	2.38E-13	postive
COMMD1	AC079145.1	0.303841449	3.87E-12	postive
COMMD1	AC079209.1	0.302929456	4.52E-12	postive
COMMD1	AC079305.2	0.306824137	2.32E-12	postive
COMMD1	AC079447.1	0.303804416	3.89E-12	postive
COMMD1	AC079610.1	0.307031233	2.24E-12	postive
COMMD1	AC079807.1	0.362343903	5.86E-17	postive
COMMD1	AC079848.1	0.40742475	2.04E-21	postive
COMMD1	AC079922.2	0.394256671	4.83E-20	postive
COMMD1	AC080037.2	0.345133441	1.97E-15	postive
COMMD1	AC080112.2	0.433837807	2.31E-24	postive
COMMD1	AC083799.1	0.459964066	1.52E-27	postive
COMMD1	AC083880.1	0.323507852	1.21E-13	postive
COMMD1	AC084809.1	0.364606234	3.63E-17	postive
COMMD1	AC084809.2	0.322255261	1.52E-13	postive
COMMD1	AC087645.2	0.371233708	8.77E-18	postive
COMMD1	AC090114.2	0.375750809	3.26E-18	postive
COMMD1	AC090204.1	0.435692887	1.40E-24	postive
COMMD1	AC090409.1	0.306484033	2.46E-12	postive
COMMD1	AC090515.3	0.391493037	9.22E-20	postive
COMMD1	AC090587.1	0.436838544	1.03E-24	postive
COMMD1	AC090617.5	0.420861898	6.97E-23	postive
COMMD1	AC090912.1	0.415042143	3.07E-22	postive
COMMD1	AC090970.2	0.335556222	1.27E-14	postive
COMMD1	AC091271.1	0.406149152	2.79E-21	postive
COMMD1	AC091729.3	0.447577336	5.30E-26	postive
COMMD1	AC091982.3	0.484045511	9.91E-31	postive
COMMD1	AC092171.5	0.367736075	1.86E-17	postive
COMMD1	AC092368.3	0.350341418	6.95E-16	postive
COMMD1	AC092614.1	0.319882721	2.33E-13	postive
COMMD1	AC092687.3	0.312112167	9.28E-13	postive
COMMD1	AC092903.2	0.318600795	2.94E-13	postive
COMMD1	AC092944.1	0.324796185	9.52E-14	postive
COMMD1	AC093157.1	0.455947252	4.88E-27	postive
COMMD1	AC093503.1	0.303538224	4.07E-12	postive

COMMD1	AC093673.1	0.493221316	5.18E-32	postive
COMMD1	AC093726.1	0.305591323	2.87E-12	postive
COMMD1	AC096536.2	0.355016875	2.68E-16	postive
COMMD1	AC096677.1	0.312489093	8.69E-13	postive
COMMD1	AC096733.3	0.304362955	3.54E-12	postive
COMMD1	AC097358.2	0.300766578	6.50E-12	postive
COMMD1	AC097359.2	0.381581019	8.91E-19	postive
COMMD1	AC098484.1	0.355599484	2.38E-16	postive
COMMD1	AC098484.2	0.330800601	3.12E-14	postive
COMMD1	AC098487.1	0.394381715	4.69E-20	postive
COMMD1	AC098820.2	0.300852957	6.41E-12	postive
COMMD1	AC098934.1	0.330130064	3.54E-14	postive
COMMD1	AC099066.2	0.316811855	4.04E-13	postive
COMMD1	AC099343.4	0.423375407	3.64E-23	postive
COMMD1	AC099522.2	0.32687774	6.49E-14	postive
COMMD1	AC099548.2	0.334085092	1.68E-14	postive
COMMD1	AC099850.2	0.45681502	3.80E-27	postive
COMMD1	AC100810.1	0.457472702	3.14E-27	postive
COMMD1	AC100861.1	0.42239363	4.70E-23	postive
COMMD1	AC104109.2	0.39473045	4.32E-20	postive
COMMD1	AC104187.1	0.308793596	1.66E-12	postive
COMMD1	AC104653.1	0.506516967	6.11E-34	postive
COMMD1	AC104667.2	0.462007574	8.34E-28	postive
COMMD1	AC104794.3	0.326902112	6.46E-14	postive
COMMD1	AC105036.3	0.370251513	1.08E-17	postive
COMMD1	AC105285.1	0.304344971	3.55E-12	postive
COMMD1	AC105942.1	0.421934832	5.29E-23	postive
COMMD1	AC106739.1	0.302855769	4.57E-12	postive
COMMD1	AC106795.1	0.589283206	4.49E-48	postive
COMMD1	AC106897.2	0.305734462	2.80E-12	postive
COMMD1	AC107027.1	0.370744945	9.75E-18	postive
COMMD1	AC107032.2	0.428210056	1.03E-23	postive
COMMD1	AC107214.1	0.389867701	1.34E-19	postive
COMMD1	AC107294.2	0.352509893	4.48E-16	postive
COMMD1	AC108047.1	0.30768512	2.00E-12	postive
COMMD1	AC108471.3	0.332846507	2.12E-14	postive
COMMD1	AC108488.1	0.32417624	1.07E-13	postive
COMMD1	AC109322.1	0.317354678	3.67E-13	postive
COMMD1	AC109347.1	0.405565907	3.22E-21	postive
COMMD1	AC109454.2	0.313374756	7.44E-13	postive
COMMD1	AC110995.1	0.383128025	6.28E-19	postive
COMMD1	AC112220.2	0.38330978	6.03E-19	postive
COMMD1	AC112715.1	0.334660247	1.50E-14	postive
COMMD1	AC114271.1	0.424891453	2.46E-23	postive
COMMD1	AC115618.1	0.366401419	2.48E-17	postive
COMMD1	AC115618.2	0.326507334	6.95E-14	postive
COMMD1	AC116036.2	0.357920167	1.48E-16	postive
COMMD1	AC116407.2	0.315865599	4.79E-13	postive
COMMD1	AC116563.1	0.384496762	4.61E-19	postive
COMMD1	AC116913.1	0.30589645	2.73E-12	postive

COMMD1	AC118553.1	0.420491379	7.67E-23	postive
COMMD1	AC121338.2	0.406487187	2.57E-21	postive
COMMD1	AC124016.2	0.367078933	2.15E-17	postive
COMMD1	AC124242.1	0.33411299	1.67E-14	postive
COMMD1	AC124248.1	0.369682952	1.23E-17	postive
COMMD1	AC124276.1	0.30208443	5.21E-12	postive
COMMD1	AC124947.1	0.307755061	1.98E-12	postive
COMMD1	AC125257.1	0.472031597	4.14E-29	postive
COMMD1	AC125603.3	0.310163353	1.30E-12	postive
COMMD1	AC126283.1	0.347386815	1.26E-15	postive
COMMD1	AC130343.2	0.387598593	2.27E-19	postive
COMMD1	AC131235.3	0.321147742	1.85E-13	postive
COMMD1	AC132938.4	0.364659433	3.59E-17	postive
COMMD1	AC133552.2	0.391060729	1.02E-19	postive
COMMD1	AC133552.5	0.376385817	2.84E-18	postive
COMMD1	AC133785.1	0.316442229	4.32E-13	postive
COMMD1	AC135048.1	0.385275742	3.86E-19	postive
COMMD1	AC135048.3	0.387507616	2.32E-19	postive
COMMD1	AC135050.1	0.306492874	2.46E-12	postive
COMMD1	AC135586.2	0.316835959	4.03E-13	postive
COMMD1	AC136604.3	0.546012861	3.40E-40	postive
COMMD1	AC137630.1	0.315443692	5.16E-13	postive
COMMD1	AC138207.2	0.508741707	2.85E-34	postive
COMMD1	AC138207.8	0.41874549	1.20E-22	postive
COMMD1	AC138696.2	0.342178373	3.52E-15	postive
COMMD1	AC139768.1	0.384880287	4.22E-19	postive
COMMD1	AC139795.3	0.305199873	3.07E-12	postive
COMMD1	AC144652.1	0.393331835	6.00E-20	postive
COMMD1	AC147067.1	0.394254372	4.83E-20	postive
COMMD1	AC147651.4	0.423287309	3.73E-23	postive
COMMD1	AC231981.1	0.357841507	1.50E-16	postive
COMMD1	AC243919.1	0.496246959	1.92E-32	postive
COMMD1	AC244090.1	0.518261186	1.03E-35	postive
COMMD1	AC244669.1	0.5031786	1.90E-33	postive
COMMD1	AC245297.4	0.352024636	4.94E-16	postive
COMMD1	ACVR2B-AS1	0.34439143	2.28E-15	postive
COMMD1	AF111167.2	0.376218379	2.94E-18	postive
COMMD1	AF131216.4	0.387277089	2.44E-19	postive
COMMD1	AF274858.1	0.426840063	1.48E-23	postive
COMMD1	AL020996.1	0.308800215	1.65E-12	postive
COMMD1	AL021328.1	0.348116314	1.09E-15	postive
COMMD1	AL023581.2	0.355973702	2.21E-16	postive
COMMD1	AL023803.1	0.328584304	4.72E-14	postive
COMMD1	AL024498.1	0.465016095	3.42E-28	postive
COMMD1	AL024507.3	0.346026439	1.65E-15	postive
COMMD1	AL031055.1	0.35052268	6.70E-16	postive
COMMD1	AL031118.1	0.345221839	1.93E-15	postive
COMMD1	AL033527.3	0.385923383	3.33E-19	postive
COMMD1	AL035661.1	0.386522993	2.90E-19	postive
COMMD1	AL049597.2	0.56059203	1.01E-42	postive

COMMD1	AL050309.1	0.531489664	8.47E-38	postive
COMMD1	AL050341.2	0.355617251	2.37E-16	postive
COMMD1	AL078581.2	0.407038464	2.24E-21	postive
COMMD1	AL078590.2	0.335127511	1.37E-14	postive
COMMD1	AL078644.2	0.395907172	3.27E-20	postive
COMMD1	AL080250.1	0.370370786	1.06E-17	postive
COMMD1	AL096828.3	0.395036182	4.02E-20	postive
COMMD1	AL117332.1	0.454563695	7.28E-27	postive
COMMD1	AL118511.1	0.345930832	1.68E-15	postive
COMMD1	AL118516.1	0.354632044	2.90E-16	postive
COMMD1	AL118558.3	0.426891121	1.46E-23	postive
COMMD1	AL121601.1	0.350323997	6.97E-16	postive
COMMD1	AL121603.2	0.327337433	5.96E-14	postive
COMMD1	AL121944.2	0.524092338	1.27E-36	postive
COMMD1	AL122035.1	0.305817168	2.76E-12	postive
COMMD1	AL132639.2	0.302969669	4.49E-12	postive
COMMD1	AL132712.1	0.3049072	3.23E-12	postive
COMMD1	AL132857.1	0.32541153	8.50E-14	postive
COMMD1	AL133153.2	0.344469175	2.24E-15	postive
COMMD1	AL133215.2	0.329403018	4.05E-14	postive
COMMD1	AL133338.1	0.380067232	1.25E-18	postive
COMMD1	AL135744.1	0.345767299	1.73E-15	postive
COMMD1	AL135925.1	0.300724855	6.55E-12	postive
COMMD1	AL136040.1	0.473797366	2.41E-29	postive
COMMD1	AL136141.1	0.304981003	3.19E-12	postive
COMMD1	AL136307.1	0.322925086	1.34E-13	postive
COMMD1	AL137186.2	0.306130783	2.62E-12	postive
COMMD1	AL138724.2	0.364226996	3.94E-17	postive
COMMD1	AL138762.2	0.403469861	5.36E-21	postive
COMMD1	AL139246.5	0.308221336	1.83E-12	postive
COMMD1	AL139260.1	0.397233868	2.39E-20	postive
COMMD1	AL139260.2	0.317595453	3.52E-13	postive
COMMD1	AL139339.1	0.433459975	2.55E-24	postive
COMMD1	AL139384.1	0.344581291	2.19E-15	postive
COMMD1	AL157813.1	0.318204359	3.15E-13	postive
COMMD1	AL161421.1	0.352088801	4.88E-16	postive
COMMD1	AL161729.1	0.344567122	2.20E-15	postive
COMMD1	AL162231.2	0.467439456	1.66E-28	postive
COMMD1	AL162377.1	0.45402137	8.50E-27	postive
COMMD1	AL353194.1	0.303004206	4.46E-12	postive
COMMD1	AL353708.1	0.394945687	4.11E-20	postive
COMMD1	AL354696.1	0.318925367	2.77E-13	postive
COMMD1	AL354718.3	0.313236983	7.62E-13	postive
COMMD1	AL354740.1	0.373634913	5.19E-18	postive
COMMD1	AL354892.2	0.32745704	5.83E-14	postive
COMMD1	AL354920.1	0.575302254	2.12E-45	postive
COMMD1	AL355001.2	0.556789745	4.75E-42	postive
COMMD1	AL355353.2	0.336379378	1.08E-14	postive
COMMD1	AL356235.1	0.360762215	8.17E-17	postive
COMMD1	AL356481.1	0.407784456	1.87E-21	postive

COMMD1	AL356599.1	0.371641968	8.02E-18	postive
COMMD1	AL357140.2	0.400842867	1.01E-20	postive
COMMD1	AL357874.1	0.351230109	5.80E-16	postive
COMMD1	AL358472.2	0.428334268	9.97E-24	postive
COMMD1	AL358852.1	0.347522132	1.22E-15	postive
COMMD1	AL359182.1	0.315082886	5.50E-13	postive
COMMD1	AL359232.1	0.379690652	1.36E-18	postive
COMMD1	AL359513.1	0.396132224	3.10E-20	postive
COMMD1	AL359711.2	0.342814691	3.10E-15	postive
COMMD1	AL359715.3	0.472068153	4.09E-29	postive
COMMD1	AL359962.1	0.302134341	5.17E-12	postive
COMMD1	AL390198.1	0.356165755	2.12E-16	postive
COMMD1	AL390719.1	0.413410646	4.62E-22	postive
COMMD1	AL390955.2	0.458495262	2.33E-27	postive
COMMD1	AL391069.2	0.446017204	8.21E-26	postive
COMMD1	AL391832.3	0.340612892	4.78E-15	postive
COMMD1	AL392172.1	0.438951535	5.78E-25	postive
COMMD1	AL441992.2	0.568224022	4.29E-44	postive
COMMD1	AL442663.3	0.340436836	4.94E-15	postive
COMMD1	AL445231.1	0.310085673	1.32E-12	postive
COMMD1	AL450998.2	0.421816791	5.45E-23	postive
COMMD1	AL451085.3	0.418381328	1.32E-22	postive
COMMD1	AL451165.2	0.517746152	1.23E-35	postive
COMMD1	AL512408.1	0.316342827	4.40E-13	postive
COMMD1	AL512598.1	0.311392152	1.05E-12	postive
COMMD1	AL512625.1	0.341488605	4.03E-15	postive
COMMD1	AL513550.1	0.400212178	1.18E-20	postive
COMMD1	AL589765.4	0.311377324	1.06E-12	postive
COMMD1	AL589843.1	0.361367826	7.19E-17	postive
COMMD1	AL590617.2	0.34636876	1.54E-15	postive
COMMD1	AL590705.1	0.310107765	1.32E-12	postive
COMMD1	AL590764.1	0.363324433	4.77E-17	postive
COMMD1	AL592166.1	0.419320152	1.03E-22	postive
COMMD1	AL596094.1	0.328922381	4.44E-14	postive
COMMD1	AL604028.1	0.364216327	3.95E-17	postive
COMMD1	AL606491.1	0.313991446	6.67E-13	postive
COMMD1	AL606760.1	0.355296651	2.53E-16	postive
COMMD1	AL645728.1	0.359166003	1.14E-16	postive
COMMD1	AL732314.8	0.321234839	1.82E-13	postive
COMMD1	AL928654.1	0.359262602	1.12E-16	postive
COMMD1	ALOX12-AS1	0.324606784	9.86E-14	postive
COMMD1	AMZ2P1	0.539009722	5.03E-39	postive
COMMD1	ANO7L1	0.41920288	1.07E-22	postive
COMMD1	AP000251.1	0.31592033	4.74E-13	postive
COMMD1	AP000317.1	0.319224864	2.62E-13	postive
COMMD1	AP000317.2	0.319360649	2.56E-13	postive
COMMD1	AP000320.1	0.376215505	2.95E-18	postive
COMMD1	AP000704.2	0.324070904	1.09E-13	postive
COMMD1	AP000769.1	0.350827304	6.30E-16	postive
COMMD1	AP001160.2	0.301383474	5.86E-12	postive

COMMD1	AP001160.3	0.418652614	1.23E-22	postive
COMMD1	AP001267.3	0.342913505	3.05E-15	postive
COMMD1	AP001269.2	0.304807374	3.28E-12	postive
COMMD1	AP001347.1	0.37038137	1.05E-17	postive
COMMD1	AP001363.2	0.309171492	1.55E-12	postive
COMMD1	AP001372.2	0.449314308	3.25E-26	postive
COMMD1	AP001527.2	0.310956357	1.14E-12	postive
COMMD1	AP001816.1	0.559600052	1.52E-42	postive
COMMD1	AP001922.5	0.479700703	3.89E-30	postive
COMMD1	AP002360.2	0.347113902	1.33E-15	postive
COMMD1	AP002387.2	0.43912583	5.51E-25	postive
COMMD1	AP002748.4	0.471484502	4.89E-29	postive
COMMD1	AP002761.2	0.314197283	6.43E-13	postive
COMMD1	AP003392.5	0.32399599	1.10E-13	postive
COMMD1	AP003721.4	0.386600339	2.85E-19	postive
COMMD1	AP004609.5	0.412783904	5.41E-22	postive
COMMD1	AP005019.1	0.301618812	5.63E-12	postive
COMMD1	AP005205.2	0.367023818	2.17E-17	postive
COMMD1	AP005482.4	0.318950255	2.76E-13	postive
COMMD1	AP006621.3	0.339558491	5.86E-15	postive
COMMD1	APTR	0.334857623	1.45E-14	postive
COMMD1	ARF4-AS1	0.499938118	5.63E-33	postive
COMMD1	ARHGAP5-AS1	0.332844742	2.12E-14	postive
COMMD1	ARHGEF26-AS1	0.344644998	2.17E-15	postive
COMMD1	ARMCX5-GPRASP2	0.507752309	4.01E-34	postive
COMMD1	ARMCX7P	0.300027722	7.36E-12	postive
COMMD1	ARRDC1-AS1	0.416953005	1.89E-22	postive
COMMD1	ASB16-AS1	0.351877222	5.09E-16	postive
COMMD1	ATP2A1-AS1	0.370712747	9.81E-18	postive
COMMD1	ATXN1-AS1	0.337188951	9.26E-15	postive
COMMD1	ATXN2-AS	0.347043311	1.35E-15	postive
COMMD1	AZIN1-AS1	0.397245939	2.38E-20	postive
COMMD1	B4GALT1-AS1	0.458922172	2.06E-27	postive
COMMD1	BAALC-AS1	0.425842332	1.92E-23	postive
COMMD1	BDNF-AS	0.352831151	4.19E-16	postive
COMMD1	BHLHE40-AS1	0.441108399	3.20E-25	postive
COMMD1	BMS1P20	0.340502868	4.88E-15	postive
COMMD1	BOLA3-AS1	0.332306338	2.35E-14	postive
COMMD1	BTG3-AS1	0.356613224	1.93E-16	postive
COMMD1	BX284668.5	0.428676228	9.11E-24	postive
COMMD1	BX537318.1	0.37566035	3.33E-18	postive
COMMD1	C10orf25	0.366961542	2.20E-17	postive
COMMD1	C15orf56	0.314255026	6.37E-13	postive
COMMD1	C18orf65	0.331538691	2.72E-14	postive
COMMD1	C1orf229	0.307114209	2.21E-12	postive
COMMD1	C21orf62-AS1	0.338046345	7.85E-15	postive
COMMD1	C6orf99	0.4979348	1.10E-32	postive
COMMD1	C8orf37-AS1	0.357386316	1.65E-16	postive
COMMD1	CA5BP1	0.382201408	7.74E-19	postive
COMMD1	CARD8-AS1	0.516749529	1.75E-35	postive

COMMD1	CASP17P	0.380302335	1.19E-18	postive
COMMD1	CATSPER2P1	0.404453077	4.22E-21	postive
COMMD1	CCNT2-AS1	0.437217304	9.26E-25	postive
COMMD1	CCR5AS	0.301259252	5.99E-12	postive
COMMD1	CD27-AS1	0.434514448	1.92E-24	postive
COMMD1	CD2BP2-DT	0.340274459	5.10E-15	postive
COMMD1	CDC37L1-DT	0.393070144	6.38E-20	postive
COMMD1	CENPBD1P1	0.417733678	1.55E-22	postive
COMMD1	CEP83-DT	0.399192176	1.50E-20	postive
COMMD1	CERNA1	0.341817056	3.78E-15	postive
COMMD1	CETN4P	0.361490784	7.01E-17	postive
COMMD1	CFL1P1	0.331189792	2.90E-14	postive
COMMD1	CHKB-DT	0.451440639	1.78E-26	postive
COMMD1	CHROMR	0.386625894	2.83E-19	postive
COMMD1	CIDECP1	0.568131586	4.46E-44	postive
COMMD1	CKMT2-AS1	0.33655771	1.05E-14	postive
COMMD1	CNNM3-DT	0.321552743	1.72E-13	postive
COMMD1	CPNE8-AS1	0.38532062	3.82E-19	postive
COMMD1	CR381653.1	0.336248105	1.11E-14	postive
COMMD1	CRNDE	0.325650448	8.14E-14	postive
COMMD1	CRTC3-AS1	0.30850348	1.74E-12	postive
COMMD1	CTBP1-DT	0.403750437	5.01E-21	postive
COMMD1	CTD-2521M24.9	0.345894453	1.69E-15	postive
COMMD1	CYTOR	0.61937463	2.70E-54	postive
COMMD1	DANCR	0.381054857	1.00E-18	postive
COMMD1	DARS-AS1	0.448786153	3.77E-26	postive
COMMD1	DCST1-AS1	0.356032779	2.18E-16	postive
COMMD1	DEC1	0.326321233	7.19E-14	postive
COMMD1	DEPDC1-AS1	0.346916572	1.38E-15	postive
COMMD1	DGCR11	0.322182995	1.54E-13	postive
COMMD1	DHCR24-DT	0.3426922	3.18E-15	postive
COMMD1	DHRS4-AS1	0.501144599	3.76E-33	postive
COMMD1	DLG1-AS1	0.391202507	9.86E-20	postive
COMMD1	DLG5-AS1	0.332788324	2.14E-14	postive
COMMD1	DNAJC3-DT	0.329909754	3.69E-14	postive
COMMD1	DOCK9-DT	0.336617753	1.03E-14	postive
COMMD1	DPH6-DT	0.434377184	2.00E-24	postive
COMMD1	DSTNP2	0.300555008	6.74E-12	postive
COMMD1	DUBR	0.309933119	1.36E-12	postive
COMMD1	DYRK3-AS1	0.312942289	8.03E-13	postive
COMMD1	EBLN3P	0.379227681	1.51E-18	postive
COMMD1	EIF1B-AS1	0.477999633	6.60E-30	postive
COMMD1	EIF2AK3-DT	0.345602395	1.79E-15	postive
COMMD1	EIF3J-DT	0.434131138	2.13E-24	postive
COMMD1	ELF3-AS1	0.346622476	1.46E-15	postive
COMMD1	ELOA-AS1	0.43862637	6.31E-25	postive
COMMD1	EMC1-AS1	0.461822686	8.80E-28	postive
COMMD1	ENTPD3-AS1	0.56345412	3.13E-43	postive
COMMD1	EPB41L4A-DT	0.395902647	3.28E-20	postive
COMMD1	EPCAM-DT	0.431400061	4.42E-24	postive

COMMD1	ERICH6-AS1	0.310810864	1.17E-12	postive
COMMD1	ETV7-AS1	0.408733303	1.48E-21	postive
COMMD1	EXOC3-AS1	0.469242327	9.64E-29	postive
COMMD1	EXOSC10-AS1	0.500121277	5.30E-33	postive
COMMD1	FAHD2CP	0.389448666	1.48E-19	postive
COMMD1	FAM111A-DT	0.412712362	5.50E-22	postive
COMMD1	FAM21FP	0.36822294	1.68E-17	postive
COMMD1	FAM86C2P	0.427908369	1.12E-23	postive
COMMD1	FAM86DP	0.390940475	1.05E-19	postive
COMMD1	FAM86EP	0.468911811	1.07E-28	postive
COMMD1	FAM86JP	0.465766437	2.74E-28	postive
COMMD1	FGD5-AS1	0.525727577	7.03E-37	postive
COMMD1	FGF14-AS2	0.301439715	5.81E-12	postive
COMMD1	FGF7P3	0.330089114	3.57E-14	postive
COMMD1	FGF7P6	0.321648658	1.69E-13	postive
COMMD1	FLVCR1-DT	0.307009594	2.25E-12	postive
COMMD1	FO393418.1	0.391166122	9.95E-20	postive
COMMD1	FOXD3-AS1	0.361602053	6.85E-17	postive
COMMD1	FRG1HP	0.491790391	8.25E-32	postive
COMMD1	FZD4-DT	0.393192549	6.20E-20	postive
COMMD1	GAPLINC	0.330987882	3.01E-14	postive
COMMD1	GBP1P1	0.324939621	9.28E-14	postive
COMMD1	GGTA1P	0.394581055	4.48E-20	postive
COMMD1	GHET1	0.364994495	3.35E-17	postive
COMMD1	GIHCG	0.42671117	1.53E-23	postive
COMMD1	GLIDR	0.454175792	8.14E-27	postive
COMMD1	GNRHR2	0.432648249	3.17E-24	postive
COMMD1	GPRC5D-AS1	0.349182372	8.77E-16	postive
COMMD1	GTF2IRD1P1	0.309817654	1.39E-12	postive
COMMD1	GTSE1-DT	0.349245464	8.66E-16	postive
COMMD1	H2AZ2P1	0.369683593	1.23E-17	postive
COMMD1	HCG15	0.313291546	7.55E-13	postive
COMMD1	HCG18	0.341117254	4.33E-15	postive
COMMD1	HCG25	0.339508373	5.92E-15	postive
COMMD1	HCP5	0.442923837	1.94E-25	postive
COMMD1	HECW2-AS1	0.329684402	3.85E-14	postive
COMMD1	HEIH	0.52352048	1.56E-36	postive
COMMD1	HLA-DRB6	0.384921549	4.18E-19	postive
COMMD1	HLA-L	0.314244322	6.38E-13	postive
COMMD1	HMGB1P5	0.481098592	2.51E-30	postive
COMMD1	HOMER3-AS1	0.340532733	4.85E-15	postive
COMMD1	HORMAD2-AS1	0.303322612	4.23E-12	postive
COMMD1	IDH1-AS1	0.425897956	1.89E-23	postive
COMMD1	IL10RB-DT	0.350266156	7.05E-16	postive
COMMD1	IL6R-AS1	0.327215464	6.09E-14	postive
COMMD1	ILF3-DT	0.392956256	6.55E-20	postive
COMMD1	INTS6L-AS1	0.309996573	1.34E-12	postive
COMMD1	IPO9-AS1	0.329654802	3.87E-14	postive
COMMD1	IQCH-AS1	0.388611214	1.80E-19	postive
COMMD1	ITFG2-AS1	0.315706398	4.92E-13	postive

COMMD1	JPX	0.426062043	1.81E-23	postive
COMMD1	KANSL1-AS1	0.341389671	4.10E-15	postive
COMMD1	KCNMB2-AS1	0.322266235	1.51E-13	postive
COMMD1	KDM7A-DT	0.41539482	2.80E-22	postive
COMMD1	KIF9-AS1	0.304167281	3.66E-12	postive
COMMD1	KMT2E-AS1	0.433170204	2.76E-24	postive
COMMD1	KRT8P12	0.357363563	1.66E-16	postive
COMMD1	KTN1-AS1	0.408609684	1.52E-21	postive
COMMD1	LBX2-AS1	0.394452545	4.61E-20	postive
COMMD1	LCT-AS1	0.306182947	2.59E-12	postive
COMMD1	LINC00211	0.333489116	1.88E-14	postive
COMMD1	LINC00239	0.428331144	9.98E-24	postive
COMMD1	LINC00324	0.477809826	7.00E-30	postive
COMMD1	LINC00339	0.590829041	2.23E-48	postive
COMMD1	LINC00467	0.445857856	8.58E-26	postive
COMMD1	LINC00471	0.30711242	2.21E-12	postive
COMMD1	LINC00476	0.434148066	2.12E-24	postive
COMMD1	LINC00511	0.468482797	1.21E-28	postive
COMMD1	LINC00526	0.447159122	5.96E-26	postive
COMMD1	LINC00623	0.320470969	2.10E-13	postive
COMMD1	LINC00630	0.372310656	6.94E-18	postive
COMMD1	LINC00636	0.308999385	1.60E-12	postive
COMMD1	LINC00638	0.300203756	7.15E-12	postive
COMMD1	LINC00654	0.371102575	9.02E-18	postive
COMMD1	LINC00667	0.381657694	8.75E-19	postive
COMMD1	LINC00674	0.302330398	5.00E-12	postive
COMMD1	LINC00680	0.360610455	8.43E-17	postive
COMMD1	LINC00847	0.523134054	1.80E-36	postive
COMMD1	LINC00853	0.390282155	1.22E-19	postive
COMMD1	LINC00869	0.31806829	3.23E-13	postive
COMMD1	LINC00884	0.351605938	5.38E-16	postive
COMMD1	LINC00888	0.532902171	5.01E-38	postive
COMMD1	LINC00900	0.343953973	2.48E-15	postive
COMMD1	LINC00909	0.450268244	2.48E-26	postive
COMMD1	LINC00910	0.385721777	3.49E-19	postive
COMMD1	LINC00933	0.363255491	4.84E-17	postive
COMMD1	LINC00957	0.397966388	2.01E-20	postive
COMMD1	LINC00963	0.428247344	1.02E-23	postive
COMMD1	LINC01003	0.38903858	1.63E-19	postive
COMMD1	LINC01006	0.336344138	1.09E-14	postive
COMMD1	LINC01011	0.321709136	1.67E-13	postive
COMMD1	LINC01023	0.416136466	2.33E-22	postive
COMMD1	LINC01063	0.383730006	5.48E-19	postive
COMMD1	LINC01094	0.481136323	2.48E-30	postive
COMMD1	LINC01124	0.322758865	1.38E-13	postive
COMMD1	LINC01128	0.35048577	6.75E-16	postive
COMMD1	LINC01132	0.339725345	5.67E-15	postive
COMMD1	LINC01135	0.304551088	3.43E-12	postive
COMMD1	LINC01137	0.489135753	1.95E-31	postive
COMMD1	LINC01150	0.353137487	3.94E-16	postive

COMMD1	LINC01184	0.566512834	8.78E-44	postive
COMMD1	LINC01220	0.407667571	1.92E-21	postive
COMMD1	LINC01232	0.327355292	5.94E-14	postive
COMMD1	LINC01273	0.388057096	2.04E-19	postive
COMMD1	LINC01278	0.343101288	2.93E-15	postive
COMMD1	LINC01426	0.456859161	3.75E-27	postive
COMMD1	LINC01506	0.471570412	4.76E-29	postive
COMMD1	LINC01521	0.365668416	2.90E-17	postive
COMMD1	LINC01560	0.368430218	1.61E-17	postive
COMMD1	LINC01578	0.312013291	9.45E-13	postive
COMMD1	LINC01588	0.362102765	6.16E-17	postive
COMMD1	LINC01635	0.4277034	1.18E-23	postive
COMMD1	LINC01686	0.374625679	4.18E-18	postive
COMMD1	LINC01704	0.334767965	1.47E-14	postive
COMMD1	LINC01715	0.413220893	4.84E-22	postive
COMMD1	LINC01719	0.306666375	2.39E-12	postive
COMMD1	LINC01778	0.368186421	1.69E-17	postive
COMMD1	LINC01806	0.390782261	1.09E-19	postive
COMMD1	LINC01841	0.436236469	1.21E-24	postive
COMMD1	LINC01852	0.393483973	5.79E-20	postive
COMMD1	LINC01888	0.305957943	2.70E-12	postive
COMMD1	LINC01943	0.412896117	5.26E-22	postive
COMMD1	LINC02014	0.369603114	1.25E-17	postive
COMMD1	LINC02021	0.314279385	6.34E-13	postive
COMMD1	LINC02026	0.38305531	6.39E-19	postive
COMMD1	LINC02028	0.300877254	6.38E-12	postive
COMMD1	LINC02041	0.320026618	2.27E-13	postive
COMMD1	LINC02062	0.32648213	6.98E-14	postive
COMMD1	LINC02126	0.312348028	8.91E-13	postive
COMMD1	LINC02172	0.374166707	4.62E-18	postive
COMMD1	LINC02210	0.371134687	8.96E-18	postive
COMMD1	LINC02259	0.305991668	2.68E-12	postive
COMMD1	LINC02285	0.4461254	7.96E-26	postive
COMMD1	LINC02298	0.400898553	9.97E-21	postive
COMMD1	LINC02321	0.352927196	4.11E-16	postive
COMMD1	LINC02332	0.308545549	1.73E-12	postive
COMMD1	LINC02356	0.31961305	2.45E-13	postive
COMMD1	LINC02361	0.333237314	1.97E-14	postive
COMMD1	LINC02391	0.374700092	4.11E-18	postive
COMMD1	LINC02447	0.332107991	2.44E-14	postive
COMMD1	LINC02453	0.333685817	1.81E-14	postive
COMMD1	LINC02482	0.403725887	5.04E-21	postive
COMMD1	LINC02550	0.330639523	3.22E-14	postive
COMMD1	LINC02562	0.323180683	1.28E-13	postive
COMMD1	LINC02585	0.302913018	4.53E-12	postive
COMMD1	LINC02609	0.51540925	2.81E-35	postive
COMMD1	LINC02611	0.373453388	5.40E-18	postive
COMMD1	LINC02637	0.440145498	4.17E-25	postive
COMMD1	LINC02709	0.355944735	2.22E-16	postive
COMMD1	LINC02724	0.305360176	2.99E-12	postive

COMMD1	LINC02762	0.373373819	5.50E-18	postive
COMMD1	LINC02773	0.321024421	1.90E-13	postive
COMMD1	LINC02777	0.399804224	1.30E-20	postive
COMMD1	LINC02828	0.361652561	6.78E-17	postive
COMMD1	LINC02861	0.371651597	8.00E-18	postive
COMMD1	LIPE-AS1	0.551222105	4.40E-41	postive
COMMD1	LMCD1-AS1	0.384465104	4.64E-19	postive
COMMD1	LMO7-AS1	0.456201836	4.54E-27	postive
COMMD1	LNCTAM34A	0.352825004	4.20E-16	postive
COMMD1	LOH12CR2	0.456879749	3.73E-27	postive
COMMD1	LOXL1-AS1	0.364873775	3.43E-17	postive
COMMD1	LPP-AS2	0.399297888	1.46E-20	postive
COMMD1	LRRC37BP1	0.428279966	1.01E-23	postive
COMMD1	LSP1P4	0.418419293	1.30E-22	postive
COMMD1	LYPLAL1-DT	0.317755946	3.42E-13	postive
COMMD1	MACORIS	0.471700455	4.58E-29	postive
COMMD1	MAFG-DT	0.448163022	4.49E-26	postive
COMMD1	MAN1B1-DT	0.418025727	1.44E-22	postive
COMMD1	MAP2K4P1	0.381002574	1.01E-18	postive
COMMD1	MAP3K14-AS1	0.35065526	6.52E-16	postive
COMMD1	MAP3K2-DT	0.429737736	6.88E-24	postive
COMMD1	MAP4K3-DT	0.558759951	2.14E-42	postive
COMMD1	MAPK6-DT	0.320627172	2.04E-13	postive
COMMD1	MAPKAPK5-AS1	0.638123713	1.59E-58	postive
COMMD1	MED14OS	0.532946687	4.93E-38	postive
COMMD1	MINCR	0.300647924	6.63E-12	postive
COMMD1	MIPEPP3	0.359393344	1.09E-16	postive
COMMD1	MIR210HG	0.326181576	7.38E-14	postive
COMMD1	MIR22HG	0.364755047	3.52E-17	postive
COMMD1	MIR4435-2HG	0.553733185	1.62E-41	postive
COMMD1	MIR4458HG	0.386272914	3.07E-19	postive
COMMD1	MIR497HG	0.304395386	3.52E-12	postive
COMMD1	MIR583HG	0.326876336	6.49E-14	postive
COMMD1	MIR762HG	0.426103567	1.79E-23	postive
COMMD1	MKLN1-AS	0.391762249	8.66E-20	postive
COMMD1	MMADHC-DT	0.406094986	2.83E-21	postive
COMMD1	MRPL20-AS1	0.430973944	4.96E-24	postive
COMMD1	MRPL45P2	0.372266565	7.00E-18	postive
COMMD1	MRPS31P4	0.355314711	2.53E-16	postive
COMMD1	MRPS9-AS2	0.3158664	4.79E-13	postive
COMMD1	MSL3P1	0.359078973	1.16E-16	postive
COMMD1	MT1DP	0.322728527	1.39E-13	postive
COMMD1	MYLK-AS1	0.375926287	3.14E-18	postive
COMMD1	NACA4P	0.338461122	7.25E-15	postive
COMMD1	NAIPP1	0.423167953	3.84E-23	postive
COMMD1	NAIPP3	0.306686406	2.38E-12	postive
COMMD1	NANOGP1	0.315768636	4.87E-13	postive
COMMD1	NAPA-AS1	0.422150168	5.00E-23	postive
COMMD1	NBR2	0.342989693	3.00E-15	postive
COMMD1	NCK1-DT	0.517656086	1.27E-35	postive

COMMD1	NCMAP-DT	0.347302205	1.28E-15	postive
COMMD1	NDUFB2-AS1	0.316068768	4.62E-13	postive
COMMD1	NEBL-AS1	0.364700622	3.56E-17	postive
COMMD1	NIFK-AS1	0.532737422	5.33E-38	postive
COMMD1	NINJ2-AS1	0.345213244	1.94E-15	postive
COMMD1	NKAPP1	0.354236107	3.15E-16	postive
COMMD1	NNT-AS1	0.358447363	1.32E-16	postive
COMMD1	NOP14-AS1	0.472173269	3.96E-29	postive
COMMD1	NORAD	0.303245191	4.28E-12	postive
COMMD1	NR2F1-AS1	0.330106897	3.55E-14	postive
COMMD1	NRAV	0.568628114	3.62E-44	postive
COMMD1	NRSN2-AS1	0.483361679	1.23E-30	postive
COMMD1	NUDT16P1	0.551460245	4.00E-41	postive
COMMD1	NUP50-DT	0.416376881	2.19E-22	postive
COMMD1	NUTM2A-AS1	0.425165614	2.29E-23	postive
COMMD1	OGFRP1	0.315697604	4.93E-13	postive
COMMD1	OIP5-AS1	0.340953063	4.47E-15	postive
COMMD1	OLMALINC	0.338075588	7.81E-15	postive
COMMD1	OR7E14P	0.330339505	3.40E-14	postive
COMMD1	OSER1-DT	0.607777366	8.09E-52	postive
COMMD1	OSTCP1	0.350821944	6.30E-16	postive
COMMD1	OTUD6B-AS1	0.381752688	8.57E-19	postive
COMMD1	PAXIP1-AS1	0.326806229	6.57E-14	postive
COMMD1	PAXIP1-AS2	0.336751495	1.01E-14	postive
COMMD1	PCBP1-AS1	0.428723446	9.00E-24	postive
COMMD1	PCCA-DT	0.520867677	4.06E-36	postive
COMMD1	PCED1B-AS1	0.333785715	1.78E-14	postive
COMMD1	PDCD4-AS1	0.367068887	2.15E-17	postive
COMMD1	PIK3CD-AS2	0.462182158	7.92E-28	postive
COMMD1	PINK1-AS	0.335641688	1.25E-14	postive
COMMD1	PITPNA-AS1	0.536616612	1.25E-38	postive
COMMD1	PLBD1-AS1	0.365732121	2.86E-17	postive
COMMD1	PMS2P4	0.505811148	7.78E-34	postive
COMMD1	POLR2J4	0.388680589	1.77E-19	postive
COMMD1	PPP1R14B-AS1	0.311164898	1.10E-12	postive
COMMD1	PRELID1P1	0.50714337	4.94E-34	postive
COMMD1	PRKAG2-AS1	0.336685123	1.02E-14	postive
COMMD1	PRKAR2A-AS1	0.394157197	4.94E-20	postive
COMMD1	PRR34	0.348585128	9.89E-16	postive
COMMD1	PRR34-AS1	0.405945684	2.93E-21	postive
COMMD1	PSMB8-AS1	0.433099915	2.81E-24	postive
COMMD1	PXN-AS1	0.498312055	9.68E-33	postive
COMMD1	RAB11B-AS1	0.441861263	2.60E-25	postive
COMMD1	RAB30-DT	0.36324359	4.85E-17	postive
COMMD1	RABGAP1L-DT	0.307547354	2.05E-12	postive
COMMD1	RAET1K	0.306121593	2.62E-12	postive
COMMD1	RALY-AS1	0.413856915	4.13E-22	postive
COMMD1	RAP2C-AS1	0.342684169	3.19E-15	postive
COMMD1	RARA-AS1	0.505939467	7.44E-34	postive
COMMD1	RASSF1-AS1	0.372886764	6.12E-18	postive

COMMD1	RBM15-AS1	0.540928932	2.42E-39	postive
COMMD1	RDM1P5	0.349715522	7.88E-16	postive
COMMD1	RHPN1-AS1	0.302336665	4.99E-12	postive
COMMD1	RNASEH1-AS1	0.562774123	4.14E-43	postive
COMMD1	RNF216P1	0.488002272	2.81E-31	postive
COMMD1	RNFT1-DT	0.394467128	4.60E-20	postive
COMMD1	ROCK1P1	0.344439616	2.25E-15	postive
COMMD1	RP11-101E13.5	0.36813121	1.71E-17	postive
COMMD1	RP11-10C24.1	0.361018367	7.74E-17	postive
COMMD1	RP11-160O5.1	0.463668645	5.10E-28	postive
COMMD1	RP11-314C16.1	0.43000835	6.40E-24	postive
COMMD1	RP11-435O5.2	0.363567549	4.53E-17	postive
COMMD1	RP11-556E13.1	0.39641902	2.90E-20	postive
COMMD1	RP13-476E20.1	0.323010742	1.32E-13	postive
COMMD1	RP4-545L17.11	0.477070714	8.81E-30	postive
COMMD1	RP5-968J1.1	0.306801892	2.33E-12	postive
COMMD1	RP9P	0.311026868	1.12E-12	postive
COMMD1	RPARP-AS1	0.360418912	8.77E-17	postive
COMMD1	RPL12P38	0.354482418	2.99E-16	postive
COMMD1	RPL13AP3	0.329669272	3.86E-14	postive
COMMD1	RPL13AP7	0.420562835	7.53E-23	postive
COMMD1	RPL23AP53	0.324513797	1.00E-13	postive
COMMD1	RPL23AP7	0.616262839	1.28E-53	postive
COMMD1	RPL23AP82	0.641793983	2.18E-59	postive
COMMD1	RPL36AP21	0.306704608	2.37E-12	postive
COMMD1	RPL37P6	0.302347822	4.98E-12	postive
COMMD1	RPLP1P6	0.451424649	1.79E-26	postive
COMMD1	RPS18P9	0.435495631	1.48E-24	postive
COMMD1	RTCA-AS1	0.310061357	1.33E-12	postive
COMMD1	SAMD12-AS1	0.34343071	2.75E-15	postive
COMMD1	SAP30-DT	0.432782996	3.06E-24	postive
COMMD1	SBDSP1	0.42678261	1.50E-23	postive
COMMD1	SBF2-AS1	0.575645446	1.83E-45	postive
COMMD1	SCAMP1-AS1	0.558946531	1.98E-42	postive
COMMD1	SDAD1P1	0.339365364	6.08E-15	postive
COMMD1	SEC22B4P	0.308283333	1.81E-12	postive
COMMD1	SEC24B-AS1	0.376792639	2.59E-18	postive
COMMD1	SEPTIN4-AS1	0.505171979	9.67E-34	postive
COMMD1	SERTAD4-AS1	0.342841646	3.09E-15	postive
COMMD1	SLC25A34-AS1	0.327738295	5.53E-14	postive
COMMD1	SLC25A5-AS1	0.373890182	4.91E-18	postive
COMMD1	SLC44A3-AS1	0.311792204	9.82E-13	postive
COMMD1	SMIM25	0.324485701	1.01E-13	postive
COMMD1	SMIM2-AS1	0.304061852	3.73E-12	postive
COMMD1	SNAI3-AS1	0.3677946	1.84E-17	postive
COMMD1	SNHG10	0.355964019	2.21E-16	postive
COMMD1	SNHG11	0.459459878	1.76E-27	postive
COMMD1	SNHG15	0.432335314	3.45E-24	postive
COMMD1	SNHG16	0.398116722	1.94E-20	postive
COMMD1	SNHG17	0.339516259	5.91E-15	postive

COMMD1	SNHG28	0.301511549	5.74E-12	postive
COMMD1	SNHG29	0.429206043	7.92E-24	postive
COMMD1	SNHG30	0.499901305	5.70E-33	postive
COMMD1	SNHG32	0.39025583	1.23E-19	postive
COMMD1	SNHG5	0.382299538	7.58E-19	postive
COMMD1	SNHG6	0.336866723	9.85E-15	postive
COMMD1	SNHG8	0.346820231	1.41E-15	postive
COMMD1	SNHG9	0.321917021	1.61E-13	postive
COMMD1	SOCAR	0.34085744	4.55E-15	postive
COMMD1	SP2-AS1	0.395803474	3.36E-20	postive
COMMD1	SPINT1-AS1	0.401377099	8.88E-21	postive
COMMD1	SPRY4-AS1	0.341312221	4.17E-15	postive
COMMD1	SRP14-AS1	0.34722804	1.30E-15	postive
COMMD1	SSR4P1	0.334524532	1.54E-14	postive
COMMD1	ST20-AS1	0.344294233	2.32E-15	postive
COMMD1	STAG3L4	0.505697112	8.09E-34	postive
COMMD1	STARD7-AS1	0.408425038	1.60E-21	postive
COMMD1	STK4-AS1	0.553858476	1.54E-41	postive
COMMD1	STX17-AS1	0.449998755	2.68E-26	postive
COMMD1	SUCLA2-AS1	0.328125488	5.15E-14	postive
COMMD1	SUCLG2-AS1	0.331873376	2.55E-14	postive
COMMD1	SUMO2P17	0.385358255	3.79E-19	postive
COMMD1	SVIL-AS1	0.546818872	2.49E-40	postive
COMMD1	SYNPR-AS1	0.316225614	4.49E-13	postive
COMMD1	TAF1A-AS1	0.47440777	2.00E-29	postive
COMMD1	TDRKH-AS1	0.49515797	2.74E-32	postive
COMMD1	TEKT4P2	0.327754873	5.51E-14	postive
COMMD1	THAP7-AS1	0.377668671	2.13E-18	postive
COMMD1	TIPARP-AS1	0.38757675	2.28E-19	postive
COMMD1	TMC3-AS1	0.308480805	1.75E-12	postive
COMMD1	TMEM191A	0.41266463	5.57E-22	postive
COMMD1	TMEM202-AS1	0.302973898	4.48E-12	postive
COMMD1	TMEM26-AS1	0.355865927	2.25E-16	postive
COMMD1	TMEM44-AS1	0.450152525	2.56E-26	postive
COMMD1	TMEM99	0.523195909	1.76E-36	postive
COMMD1	TNKS2-AS1	0.346525073	1.49E-15	postive
COMMD1	TOB1-AS1	0.426883551	1.46E-23	postive
COMMD1	TOLLIP-AS1	0.331749254	2.61E-14	postive
COMMD1	TP53TG1	0.626829311	6.08E-56	postive
COMMD1	TP73-AS1	0.320952104	1.92E-13	postive
COMMD1	TPRG1-AS1	0.413676458	4.32E-22	postive
COMMD1	TRAM2-AS1	0.631695416	4.83E-57	postive
COMMD1	TRIM52-AS1	0.576131969	1.49E-45	postive
COMMD1	TSPY26P	0.321756991	1.66E-13	postive
COMMD1	TSTD3	0.530094762	1.42E-37	postive
COMMD1	TTC28-AS1	0.367744556	1.86E-17	postive
COMMD1	TUBBP1	0.309825841	1.38E-12	postive
COMMD1	TYMSOS	0.313101066	7.80E-13	postive
COMMD1	U47924.2	0.340811122	4.59E-15	postive
COMMD1	U62317.1	0.361584511	6.87E-17	postive

COMMD1	U91328.1	0.305645832	2.84E-12	postive
COMMD1	UBA6-AS1	0.584079367	4.60E-47	postive
COMMD1	UBAC2-AS1	0.447884239	4.86E-26	postive
COMMD1	UBE2D3-AS1	0.559048131	1.90E-42	postive
COMMD1	UBL7-AS1	0.523072838	1.84E-36	postive
COMMD1	UBR5-AS1	0.347243939	1.29E-15	postive
COMMD1	UGDH-AS1	0.389777666	1.37E-19	postive
COMMD1	URB1-AS1	0.536079654	1.52E-38	postive
COMMD1	USP27X-AS1	0.396781125	2.66E-20	postive
COMMD1	USP46-AS1	0.407242206	2.13E-21	postive
COMMD1	UXT-AS1	0.40733427	2.09E-21	postive
COMMD1	VPS33B-DT	0.353848385	3.41E-16	postive
COMMD1	WAC-AS1	0.331962024	2.51E-14	postive
COMMD1	WASH3P	0.332183562	2.40E-14	postive
COMMD1	WDFY3-AS2	0.346463184	1.51E-15	postive
COMMD1	WEE2-AS1	0.389930871	1.32E-19	postive
COMMD1	WWTR1-AS1	0.31728618	3.72E-13	postive
COMMD1	YTHDF3-AS1	0.372283857	6.98E-18	postive
COMMD1	Z83851.4	0.352999091	4.05E-16	postive
COMMD1	Z99572.1	0.301994578	5.29E-12	postive
COMMD1	ZBED5-AS1	0.457016824	3.58E-27	postive
COMMD1	ZBTB11-AS1	0.396043497	3.17E-20	postive
COMMD1	ZBTB44-DT	0.323328091	1.25E-13	postive
COMMD1	ZFAS1	0.491515173	9.02E-32	postive
COMMD1	ZFPM2-AS1	0.330063067	3.58E-14	postive
COMMD1	ZNF197-AS1	0.307374863	2.11E-12	postive
COMMD1	ZNF205-AS1	0.307490348	2.07E-12	postive
COMMD1	ZNF236-DT	0.447678393	5.15E-26	postive
COMMD1	ZNF252P	0.403571075	5.23E-21	postive
COMMD1	ZNF341-AS1	0.316433441	4.33E-13	postive
COMMD1	ZNF426-DT	0.329704402	3.83E-14	postive
COMMD1	ZNF503-AS2	0.382986596	6.49E-19	postive
COMMD1	ZNF529-AS1	0.364339583	3.84E-17	postive
COMMD1	ZNF542P	0.345438366	1.85E-15	postive
COMMD1	ZNF561-AS1	0.741755976	1.92E-88	postive
COMMD1	ZNF687-AS1	0.534637335	2.62E-38	postive
COMMD1	ZNF702P	0.356272428	2.07E-16	postive
COMMD1	ZNF788P	0.363327034	4.76E-17	postive
COMMD1	ZNF815P	0.439181163	5.43E-25	postive
COMMD1	ZNRD2-AS1	0.325279992	8.71E-14	postive
COMMD1	ZSCAN16-AS1	0.61733936	7.47E-54	postive
COX17	AC002116.2	0.479108736	4.68E-30	postive
COX17	AC002310.1	0.389455204	1.48E-19	postive
COX17	AC002310.2	0.32291747	1.34E-13	postive
COX17	AC002398.1	0.339962706	5.42E-15	postive
COX17	AC002451.1	0.330251123	3.46E-14	postive
COX17	AC002467.1	0.56570483	1.23E-43	postive
COX17	AC002470.1	0.479504181	4.13E-30	postive
COX17	AC003965.1	0.306717511	2.37E-12	postive
COX17	AC004241.1	0.513696724	5.12E-35	postive

COX17	AC004241.3	0.484488214	8.61E-31	postive
COX17	AC004447.2	0.30706423	2.23E-12	postive
COX17	AC004477.1	0.408282832	1.65E-21	postive
COX17	AC004596.1	0.323149237	1.29E-13	postive
COX17	AC004707.2	0.311174598	1.09E-12	postive
COX17	AC004801.6	0.405245948	3.48E-21	postive
COX17	AC004803.1	0.387055691	2.57E-19	postive
COX17	AC004812.2	0.437757534	8.00E-25	postive
COX17	AC004918.1	0.314557462	6.04E-13	postive
COX17	AC004943.2	0.30790675	1.93E-12	postive
COX17	AC004973.1	0.322602452	1.42E-13	postive
COX17	AC004980.1	0.340348083	5.03E-15	postive
COX17	AC005034.4	0.388936022	1.67E-19	postive
COX17	AC005037.1	0.375369575	3.55E-18	postive
COX17	AC005076.2	0.476897634	9.29E-30	postive
COX17	AC005083.1	0.321926573	1.61E-13	postive
COX17	AC005225.2	0.373216522	5.69E-18	postive
COX17	AC005229.4	0.515560645	2.67E-35	postive
COX17	AC005261.4	0.34368	2.62E-15	postive
COX17	AC005363.2	0.306775758	2.34E-12	postive
COX17	AC005498.2	0.302271999	5.05E-12	postive
COX17	AC005520.4	0.330477055	3.32E-14	postive
COX17	AC005562.1	0.38283662	6.71E-19	postive
COX17	AC005586.1	0.372302969	6.95E-18	postive
COX17	AC005670.1	0.308571714	1.72E-12	postive
COX17	AC005670.3	0.337505932	8.71E-15	postive
COX17	AC005695.1	0.301532856	5.72E-12	postive
COX17	AC005696.1	0.369497508	1.28E-17	postive
COX17	AC005837.1	0.356602265	1.94E-16	postive
COX17	AC005840.4	0.331438646	2.77E-14	postive
COX17	AC005911.1	0.32926808	4.16E-14	postive
COX17	AC005920.1	0.374209289	4.58E-18	postive
COX17	AC006001.2	0.317327745	3.69E-13	postive
COX17	AC006213.1	0.315124362	5.46E-13	postive
COX17	AC006252.1	0.40387555	4.86E-21	postive
COX17	AC006333.1	0.443626758	1.60E-25	postive
COX17	AC006449.2	0.365233133	3.18E-17	postive
COX17	AC006449.5	0.36612716	2.63E-17	postive
COX17	AC006449.7	0.582571854	8.96E-47	postive
COX17	AC006504.5	0.313702663	7.02E-13	postive
COX17	AC006547.1	0.340266728	5.11E-15	postive
COX17	AC006942.1	0.35751586	1.60E-16	postive
COX17	AC007029.2	0.30069218	6.58E-12	postive
COX17	AC007032.1	0.425335165	2.19E-23	postive
COX17	AC007036.1	0.350566053	6.64E-16	postive
COX17	AC007098.1	0.31059897	1.21E-12	postive
COX17	AC007114.1	0.43473337	1.81E-24	postive
COX17	AC007342.4	0.327209398	6.10E-14	postive
COX17	AC007365.1	0.335474499	1.29E-14	postive
COX17	AC007383.2	0.40038414	1.13E-20	postive

COX17	AC007541.1	0.472536842	3.55E-29	postive
COX17	AC007663.3	0.367558544	1.94E-17	postive
COX17	AC007773.1	0.479729532	3.85E-30	postive
COX17	AC007881.3	0.368816293	1.48E-17	postive
COX17	AC007952.2	0.354537853	2.96E-16	postive
COX17	AC008014.1	0.481329693	2.33E-30	postive
COX17	AC008035.1	0.409670132	1.17E-21	postive
COX17	AC008063.1	0.348551934	9.95E-16	postive
COX17	AC008105.2	0.346513347	1.49E-15	postive
COX17	AC008267.5	0.493317947	5.02E-32	postive
COX17	AC008393.1	0.34214396	3.54E-15	postive
COX17	AC008438.1	0.45363678	9.50E-27	postive
COX17	AC008443.1	0.494512609	3.39E-32	postive
COX17	AC008443.5	0.536151712	1.48E-38	postive
COX17	AC008494.3	0.313451142	7.34E-13	postive
COX17	AC008592.1	0.371860378	7.65E-18	postive
COX17	AC008608.2	0.518879846	8.24E-36	postive
COX17	AC008610.1	0.358871563	1.21E-16	postive
COX17	AC008619.1	0.335089742	1.38E-14	postive
COX17	AC008622.2	0.369948485	1.16E-17	postive
COX17	AC008747.1	0.386645006	2.82E-19	postive
COX17	AC008771.1	0.437608565	8.33E-25	postive
COX17	AC008915.3	0.533582839	3.89E-38	postive
COX17	AC008946.1	0.397269263	2.37E-20	postive
COX17	AC008966.1	0.347984466	1.11E-15	postive
COX17	AC008991.1	0.31955596	2.47E-13	postive
COX17	AC009005.1	0.320309544	2.16E-13	postive
COX17	AC009053.3	0.301546087	5.70E-12	postive
COX17	AC009061.2	0.519075383	7.69E-36	postive
COX17	AC009065.5	0.302725012	4.68E-12	postive
COX17	AC009065.9	0.365755056	2.85E-17	postive
COX17	AC009113.1	0.307026886	2.24E-12	postive
COX17	AC009118.2	0.482177259	1.79E-30	postive
COX17	AC009121.4	0.335245863	1.34E-14	postive
COX17	AC009126.1	0.380960439	1.02E-18	postive
COX17	AC009171.2	0.421701019	5.62E-23	postive
COX17	AC009237.14	0.371308414	8.62E-18	postive
COX17	AC009275.1	0.387552647	2.29E-19	postive
COX17	AC009309.2	0.417178831	1.79E-22	postive
COX17	AC009403.1	0.36293394	5.17E-17	postive
COX17	AC009506.2	0.376887691	2.54E-18	postive
COX17	AC009690.2	0.353879968	3.39E-16	postive
COX17	AC009779.3	0.582766864	8.22E-47	postive
COX17	AC009831.1	0.332480717	2.27E-14	postive
COX17	AC009948.3	0.317315058	3.70E-13	postive
COX17	AC009961.1	0.364505798	3.71E-17	postive
COX17	AC009988.1	0.328450376	4.84E-14	postive
COX17	AC010173.1	0.507830355	3.90E-34	postive
COX17	AC010203.2	0.309232273	1.53E-12	postive
COX17	AC010205.1	0.357370858	1.65E-16	postive

COX17	AC010226.1	0.345173269	1.95E-15	postive
COX17	AC010319.5	0.305308745	3.01E-12	postive
COX17	AC010320.2	0.326667936	6.74E-14	postive
COX17	AC010331.1	0.340068734	5.31E-15	postive
COX17	AC010359.2	0.348741093	9.58E-16	postive
COX17	AC010442.1	0.406088931	2.83E-21	postive
COX17	AC010503.5	0.349115072	8.89E-16	postive
COX17	AC010504.1	0.499269913	7.04E-33	postive
COX17	AC010531.6	0.483896017	1.04E-30	postive
COX17	AC010595.1	0.316301679	4.43E-13	postive
COX17	AC010655.4	0.389183268	1.57E-19	postive
COX17	AC010789.1	0.301190265	6.06E-12	postive
COX17	AC010834.2	0.410398508	9.79E-22	postive
COX17	AC010864.1	0.334805471	1.46E-14	postive
COX17	AC010894.2	0.471901304	4.31E-29	postive
COX17	AC010913.1	0.412622859	5.63E-22	postive
COX17	AC010969.2	0.301393284	5.85E-12	postive
COX17	AC010999.2	0.354181746	3.18E-16	postive
COX17	AC011445.2	0.326063464	7.54E-14	postive
COX17	AC011472.2	0.312170244	9.19E-13	postive
COX17	AC011474.1	0.311792012	9.82E-13	postive
COX17	AC011474.2	0.33411017	1.67E-14	postive
COX17	AC011524.1	0.318441154	3.02E-13	postive
COX17	AC011603.3	0.41221837	6.23E-22	postive
COX17	AC011731.1	0.305775822	2.78E-12	postive
COX17	AC012063.1	0.35434554	3.08E-16	postive
COX17	AC012073.1	0.312341788	8.92E-13	postive
COX17	AC012146.1	0.379150474	1.54E-18	postive
COX17	AC012184.3	0.514347003	4.08E-35	postive
COX17	AC012306.2	0.405935361	2.94E-21	postive
COX17	AC012313.1	0.306834669	2.32E-12	postive
COX17	AC012313.9	0.367508505	1.96E-17	postive
COX17	AC012442.2	0.365179944	3.22E-17	postive
COX17	AC012442.3	0.476221909	1.15E-29	postive
COX17	AC012456.2	0.323142241	1.29E-13	postive
COX17	AC012510.1	0.353285169	3.82E-16	postive
COX17	AC012555.1	0.434412636	1.98E-24	postive
COX17	AC012615.1	0.35621407	2.10E-16	postive
COX17	AC012640.4	0.442225695	2.35E-25	postive
COX17	AC013565.3	0.321332334	1.79E-13	postive
COX17	AC013724.1	0.386823296	2.71E-19	postive
COX17	AC015819.1	0.302168189	5.14E-12	postive
COX17	AC015912.3	0.387499065	2.32E-19	postive
COX17	AC015922.3	0.36250711	5.66E-17	postive
COX17	AC015982.2	0.467414759	1.67E-28	postive
COX17	AC016027.1	0.432610386	3.20E-24	postive
COX17	AC016065.1	0.500358651	4.90E-33	postive
COX17	AC016355.1	0.360208199	9.17E-17	postive
COX17	AC016394.2	0.511714154	1.02E-34	postive
COX17	AC016405.3	0.330831634	3.10E-14	postive

COX17	AC016727.1	0.409335479	1.27E-21	postive
COX17	AC016747.1	0.412169782	6.30E-22	postive
COX17	AC016773.2	0.336542399	1.05E-14	postive
COX17	AC016876.2	0.478968858	4.88E-30	postive
COX17	AC016876.3	0.399715456	1.32E-20	postive
COX17	AC016888.1	0.443814951	1.52E-25	postive
COX17	AC017002.1	0.307481667	2.08E-12	postive
COX17	AC017083.1	0.351100328	5.96E-16	postive
COX17	AC018413.1	0.333387117	1.91E-14	postive
COX17	AC018521.5	0.479070009	4.73E-30	postive
COX17	AC019069.1	0.323574578	1.19E-13	postive
COX17	AC019129.2	0.303594868	4.03E-12	postive
COX17	AC019131.2	0.325012816	9.15E-14	postive
COX17	AC020661.1	0.311623004	1.01E-12	postive
COX17	AC020663.2	0.489305096	1.84E-31	postive
COX17	AC020765.2	0.394042259	5.08E-20	postive
COX17	AC020904.2	0.361202526	7.45E-17	postive
COX17	AC020910.5	0.422213547	4.92E-23	postive
COX17	AC020915.3	0.365543646	2.98E-17	postive
COX17	AC021028.1	0.324235172	1.06E-13	postive
COX17	AC022007.1	0.30691908	2.29E-12	postive
COX17	AC022098.3	0.329781237	3.78E-14	postive
COX17	AC022167.4	0.39286475	6.69E-20	postive
COX17	AC022613.2	0.338731645	6.88E-15	postive
COX17	AC022784.6	0.36638406	2.49E-17	postive
COX17	AC022915.1	0.314181368	6.45E-13	postive
COX17	AC022916.2	0.351381813	5.63E-16	postive
COX17	AC023024.1	0.317607574	3.51E-13	postive
COX17	AC023043.1	0.354528456	2.97E-16	postive
COX17	AC023043.4	0.312419243	8.80E-13	postive
COX17	AC023509.3	0.493316138	5.02E-32	postive
COX17	AC023813.3	0.37947389	1.43E-18	postive
COX17	AC023983.2	0.315709337	4.92E-13	postive
COX17	AC024243.1	0.378651658	1.72E-18	postive
COX17	AC024267.7	0.4050264	3.67E-21	postive
COX17	AC024361.2	0.385045794	4.07E-19	postive
COX17	AC024559.2	0.308125407	1.86E-12	postive
COX17	AC024575.2	0.388827655	1.71E-19	postive
COX17	AC024884.2	0.318672937	2.90E-13	postive
COX17	AC025048.2	0.323435775	1.22E-13	postive
COX17	AC025048.4	0.342648493	3.21E-15	postive
COX17	AC025181.2	0.377894346	2.03E-18	postive
COX17	AC025682.2	0.39146318	9.28E-20	postive
COX17	AC026191.1	0.307609913	2.03E-12	postive
COX17	AC026254.2	0.347232946	1.30E-15	postive
COX17	AC026304.1	0.561439575	7.16E-43	postive
COX17	AC026401.3	0.362515106	5.65E-17	postive
COX17	AC026412.1	0.363566684	4.53E-17	postive
COX17	AC026471.1	0.47211613	4.03E-29	postive
COX17	AC026471.2	0.383711328	5.51E-19	postive

COX17	AC026691.1	0.353063253	4.00E-16	postive
COX17	AC026801.2	0.352918095	4.12E-16	postive
COX17	AC026979.2	0.396040615	3.17E-20	postive
COX17	AC027097.1	0.320248185	2.18E-13	postive
COX17	AC027307.2	0.348151241	1.08E-15	postive
COX17	AC027559.1	0.353777359	3.46E-16	postive
COX17	AC027644.3	0.494082122	3.91E-32	postive
COX17	AC027682.6	0.402945827	6.08E-21	postive
COX17	AC034102.3	0.302198098	5.11E-12	postive
COX17	AC034102.5	0.32198406	1.59E-13	postive
COX17	AC034236.2	0.301812901	5.45E-12	postive
COX17	AC034243.1	0.322510013	1.45E-13	postive
COX17	AC036176.1	0.462673929	6.85E-28	postive
COX17	AC040169.1	0.396137544	3.10E-20	postive
COX17	AC040970.1	0.375418739	3.51E-18	postive
COX17	AC046134.2	0.606779297	1.31E-51	postive
COX17	AC046143.1	0.302565548	4.80E-12	postive
COX17	AC046143.2	0.392321149	7.60E-20	postive
COX17	AC053503.1	0.305433479	2.95E-12	postive
COX17	AC053503.4	0.368533778	1.57E-17	postive
COX17	AC061992.2	0.385600634	3.58E-19	postive
COX17	AC062028.1	0.303573365	4.05E-12	postive
COX17	AC064807.1	0.53159143	8.16E-38	postive
COX17	AC064807.4	0.370523482	1.02E-17	postive
COX17	AC064836.3	0.373608711	5.22E-18	postive
COX17	AC067852.2	0.492338238	6.90E-32	postive
COX17	AC068189.1	0.341132551	4.32E-15	postive
COX17	AC068338.2	0.54636249	2.97E-40	postive
COX17	AC068473.5	0.38789489	2.12E-19	postive
COX17	AC068620.1	0.335699411	1.23E-14	postive
COX17	AC068790.8	0.347275134	1.28E-15	postive
COX17	AC068831.2	0.394792965	4.26E-20	postive
COX17	AC068870.2	0.374775599	4.04E-18	postive
COX17	AC068888.1	0.317272554	3.72E-13	postive
COX17	AC069213.1	0.329436168	4.03E-14	postive
COX17	AC069224.2	0.396033216	3.18E-20	postive
COX17	AC069544.1	0.393317	6.02E-20	postive
COX17	AC072061.1	0.321445408	1.76E-13	postive
COX17	AC073195.2	0.440823747	3.46E-25	postive
COX17	AC073352.1	0.376854791	2.56E-18	postive
COX17	AC073370.1	0.316937815	3.95E-13	postive
COX17	AC073508.3	0.405308936	3.43E-21	postive
COX17	AC073529.1	0.500343401	4.92E-33	postive
COX17	AC073569.2	0.349949425	7.52E-16	postive
COX17	AC073585.1	0.350525185	6.69E-16	postive
COX17	AC073611.1	0.433251911	2.70E-24	postive
COX17	AC073896.2	0.586304922	1.71E-47	postive
COX17	AC073896.4	0.382825397	6.73E-19	postive
COX17	AC074044.1	0.376172255	2.97E-18	postive
COX17	AC078802.1	0.305411347	2.96E-12	postive

COX17	AC078880.3	0.334978983	1.41E-14	postive
COX17	AC078880.5	0.345903739	1.69E-15	postive
COX17	AC079174.2	0.345508988	1.82E-15	postive
COX17	AC079209.1	0.39749976	2.24E-20	postive
COX17	AC079305.2	0.362561899	5.60E-17	postive
COX17	AC079313.2	0.331993564	2.49E-14	postive
COX17	AC079598.1	0.337194262	9.25E-15	postive
COX17	AC079807.1	0.397761005	2.11E-20	postive
COX17	AC079848.1	0.454348842	7.74E-27	postive
COX17	AC079922.2	0.480403429	3.12E-30	postive
COX17	AC080013.5	0.413483124	4.54E-22	postive
COX17	AC080037.2	0.321087663	1.87E-13	postive
COX17	AC080112.2	0.407487783	2.01E-21	postive
COX17	AC080129.2	0.398479014	1.78E-20	postive
COX17	AC082651.1	0.342556329	3.27E-15	postive
COX17	AC083798.2	0.385768491	3.45E-19	postive
COX17	AC083799.1	0.620309846	1.69E-54	postive
COX17	AC083880.1	0.50392054	1.48E-33	postive
COX17	AC084125.4	0.337495787	8.73E-15	postive
COX17	AC084357.2	0.310639971	1.20E-12	postive
COX17	AC087379.2	0.36347976	4.61E-17	postive
COX17	AC087482.1	0.314953882	5.63E-13	postive
COX17	AC087521.3	0.308072041	1.87E-12	postive
COX17	AC087623.2	0.360352842	8.90E-17	postive
COX17	AC087645.2	0.368185585	1.69E-17	postive
COX17	AC087741.2	0.398755356	1.66E-20	postive
COX17	AC090114.2	0.386272396	3.07E-19	postive
COX17	AC090204.1	0.35240273	4.58E-16	postive
COX17	AC090425.2	0.315115333	5.47E-13	postive
COX17	AC090505.2	0.301745069	5.52E-12	postive
COX17	AC090515.3	0.43679265	1.04E-24	postive
COX17	AC090587.1	0.568158495	4.41E-44	postive
COX17	AC090617.5	0.407951183	1.79E-21	postive
COX17	AC090912.1	0.426728421	1.52E-23	postive
COX17	AC090970.2	0.49860181	8.79E-33	postive
COX17	AC091114.1	0.367708699	1.88E-17	postive
COX17	AC091153.3	0.42980982	6.75E-24	postive
COX17	AC091153.4	0.324885771	9.37E-14	postive
COX17	AC091271.1	0.468657635	1.15E-28	postive
COX17	AC091390.5	0.395994461	3.21E-20	postive
COX17	AC091729.3	0.486340154	4.78E-31	postive
COX17	AC091982.3	0.493780072	4.31E-32	postive
COX17	AC092115.4	0.50542027	8.88E-34	postive
COX17	AC092118.1	0.357316444	1.67E-16	postive
COX17	AC092171.4	0.302953523	4.50E-12	postive
COX17	AC092171.5	0.331117234	2.94E-14	postive
COX17	AC092295.2	0.302970333	4.48E-12	postive
COX17	AC092368.3	0.393138122	6.28E-20	postive
COX17	AC092431.1	0.377765848	2.09E-18	postive
COX17	AC092687.3	0.311479521	1.04E-12	postive

COX17	AC092718.2	0.353788578	3.45E-16	postive
COX17	AC092718.4	0.476252208	1.13E-29	postive
COX17	AC092803.1	0.446121203	7.97E-26	postive
COX17	AC092903.2	0.33883674	6.74E-15	postive
COX17	AC092910.3	0.436583854	1.10E-24	postive
COX17	AC092944.1	0.372364912	6.85E-18	postive
COX17	AC093157.1	0.404138485	4.56E-21	postive
COX17	AC093249.2	0.351023445	6.05E-16	postive
COX17	AC093249.6	0.415908679	2.46E-22	postive
COX17	AC093503.1	0.30058115	6.71E-12	postive
COX17	AC093510.1	0.346338553	1.55E-15	postive
COX17	AC093520.1	0.364020956	4.11E-17	postive
COX17	AC093673.1	0.427815827	1.14E-23	postive
COX17	AC096536.2	0.3736735	5.15E-18	postive
COX17	AC096540.1	0.348141605	1.08E-15	postive
COX17	AC096543.2	0.331794002	2.59E-14	postive
COX17	AC096649.2	0.307945599	1.92E-12	postive
COX17	AC096677.1	0.421947407	5.27E-23	postive
COX17	AC096733.3	0.325908078	7.76E-14	postive
COX17	AC097359.2	0.378883994	1.63E-18	postive
COX17	AC097468.3	0.308904064	1.62E-12	postive
COX17	AC097532.2	0.318720777	2.87E-13	postive
COX17	AC098484.2	0.384639219	4.46E-19	postive
COX17	AC098487.1	0.420499946	7.65E-23	postive
COX17	AC098820.2	0.412990938	5.13E-22	postive
COX17	AC098934.1	0.36569042	2.89E-17	postive
COX17	AC099343.4	0.427217956	1.34E-23	postive
COX17	AC099518.4	0.356122073	2.14E-16	postive
COX17	AC099522.2	0.396349001	2.95E-20	postive
COX17	AC099568.2	0.312459664	8.74E-13	postive
COX17	AC099778.1	0.355566386	2.40E-16	postive
COX17	AC099791.2	0.326072103	7.53E-14	postive
COX17	AC099811.3	0.312551627	8.60E-13	postive
COX17	AC099850.2	0.456458783	4.21E-27	postive
COX17	AC099850.4	0.333643233	1.82E-14	postive
COX17	AC100803.2	0.390961341	1.04E-19	postive
COX17	AC100810.1	0.403259056	5.64E-21	postive
COX17	AC100847.1	0.31642408	4.33E-13	postive
COX17	AC100861.1	0.422562211	4.50E-23	postive
COX17	AC103760.1	0.347410611	1.25E-15	postive
COX17	AC103770.1	0.329203835	4.21E-14	postive
COX17	AC103855.2	0.330626515	3.22E-14	postive
COX17	AC103925.1	0.318512062	2.98E-13	postive
COX17	AC104109.2	0.392487831	7.31E-20	postive
COX17	AC104187.1	0.375819465	3.21E-18	postive
COX17	AC104435.2	0.32593018	7.73E-14	postive
COX17	AC104653.1	0.372973751	6.00E-18	postive
COX17	AC104667.2	0.555744181	7.24E-42	postive
COX17	AC104777.1	0.32344659	1.22E-13	postive
COX17	AC104794.3	0.327939259	5.33E-14	postive

COX17	AC105036.3	0.399994887	1.24E-20	postive
COX17	AC105137.2	0.348416696	1.02E-15	postive
COX17	AC105285.1	0.382599354	7.08E-19	postive
COX17	AC106706.1	0.338126419	7.73E-15	postive
COX17	AC106738.1	0.314002945	6.66E-13	postive
COX17	AC106791.1	0.304884356	3.24E-12	postive
COX17	AC106795.1	0.58600797	1.95E-47	postive
COX17	AC106897.2	0.32119388	1.84E-13	postive
COX17	AC107027.1	0.356861883	1.84E-16	postive
COX17	AC107027.3	0.333801487	1.77E-14	postive
COX17	AC107032.2	0.451156222	1.93E-26	postive
COX17	AC107214.1	0.437532606	8.50E-25	postive
COX17	AC107294.2	0.304595731	3.40E-12	postive
COX17	AC107959.3	0.381177167	9.75E-19	postive
COX17	AC108047.1	0.407220085	2.15E-21	postive
COX17	AC108673.2	0.31161426	1.01E-12	postive
COX17	AC108673.3	0.311804005	9.80E-13	postive
COX17	AC108860.2	0.397635225	2.17E-20	postive
COX17	AC109322.1	0.485015499	7.28E-31	postive
COX17	AC109347.1	0.398301941	1.85E-20	postive
COX17	AC110609.1	0.3313613	2.81E-14	postive
COX17	AC112220.2	0.37506208	3.80E-18	postive
COX17	AC112236.2	0.326164077	7.40E-14	postive
COX17	AC114271.1	0.390765935	1.09E-19	postive
COX17	AC114489.2	0.357904142	1.48E-16	postive
COX17	AC115618.1	0.399126793	1.52E-20	postive
COX17	AC115618.2	0.312953659	8.01E-13	postive
COX17	AC116025.2	0.310057408	1.33E-12	postive
COX17	AC116036.2	0.388256005	1.95E-19	postive
COX17	AC116351.1	0.40780564	1.86E-21	postive
COX17	AC116351.2	0.330870246	3.08E-14	postive
COX17	AC116913.1	0.421358416	6.13E-23	postive
COX17	AC118553.1	0.370934632	9.35E-18	postive
COX17	AC120498.4	0.329127941	4.27E-14	postive
COX17	AC121338.2	0.351955939	5.01E-16	postive
COX17	AC121761.2	0.324867988	9.40E-14	postive
COX17	AC123768.2	0.308524799	1.73E-12	postive
COX17	AC124016.2	0.489818704	1.56E-31	postive
COX17	AC124242.1	0.30017048	7.19E-12	postive
COX17	AC124248.1	0.424244258	2.91E-23	postive
COX17	AC124798.1	0.325790693	7.93E-14	postive
COX17	AC124947.1	0.372688461	6.39E-18	postive
COX17	AC124947.2	0.30842391	1.76E-12	postive
COX17	AC125257.1	0.447651511	5.19E-26	postive
COX17	AC125603.3	0.409221488	1.31E-21	postive
COX17	AC126283.1	0.364780646	3.50E-17	postive
COX17	AC127070.1	0.312280335	9.01E-13	postive
COX17	AC128687.2	0.480604418	2.93E-30	postive
COX17	AC130343.2	0.380502902	1.13E-18	postive
COX17	AC131009.3	0.376527165	2.75E-18	postive

COX17	AC131011.2	0.415533621	2.71E-22	postive
COX17	AC131235.3	0.492907236	5.74E-32	postive
COX17	AC131235.4	0.377972032	2.00E-18	postive
COX17	AC132008.2	0.493456779	4.79E-32	postive
COX17	AC132192.2	0.339962972	5.42E-15	postive
COX17	AC132938.4	0.362597608	5.55E-17	postive
COX17	AC133528.2	0.427087534	1.38E-23	postive
COX17	AC133552.2	0.343389603	2.77E-15	postive
COX17	AC133552.5	0.413027151	5.09E-22	postive
COX17	AC133785.1	0.429751698	6.85E-24	postive
COX17	AC135048.1	0.441949466	2.54E-25	postive
COX17	AC135048.3	0.399859196	1.28E-20	postive
COX17	AC135050.1	0.396046142	3.17E-20	postive
COX17	AC135586.2	0.399061298	1.55E-20	postive
COX17	AC136604.3	0.466540234	2.17E-28	postive
COX17	AC138123.1	0.349072486	8.97E-16	postive
COX17	AC138207.2	0.369721586	1.22E-17	postive
COX17	AC138696.2	0.458433049	2.38E-27	postive
COX17	AC139530.1	0.380224862	1.21E-18	postive
COX17	AC139768.1	0.448077329	4.60E-26	postive
COX17	AC142472.1	0.384311164	4.81E-19	postive
COX17	AC144652.1	0.413242881	4.82E-22	postive
COX17	AC145285.6	0.311117302	1.10E-12	postive
COX17	AC147067.1	0.313835682	6.86E-13	postive
COX17	AC211433.2	0.308082329	1.87E-12	postive
COX17	AC231981.1	0.36122782	7.41E-17	postive
COX17	AC233280.1	0.422024336	5.17E-23	postive
COX17	AC242842.1	0.343780579	2.57E-15	postive
COX17	AC243919.1	0.369872862	1.18E-17	postive
COX17	AC243964.3	0.335752449	1.22E-14	postive
COX17	AC244090.1	0.45636239	4.33E-27	postive
COX17	AC244669.1	0.505743606	7.96E-34	postive
COX17	AC245140.1	0.306951275	2.27E-12	postive
COX17	AC245140.2	0.384926654	4.18E-19	postive
COX17	AC245297.4	0.356060681	2.17E-16	postive
COX17	ACVR2B-AS1	0.401104862	9.48E-21	postive
COX17	AD000671.3	0.368596057	1.55E-17	postive
COX17	AF129075.3	0.301676456	5.58E-12	postive
COX17	AF131216.4	0.421485786	5.94E-23	postive
COX17	AF196972.1	0.342836355	3.09E-15	postive
COX17	AF274858.1	0.459383315	1.80E-27	postive
COX17	AF279873.4	0.319461734	2.51E-13	postive
COX17	AGAP1-IT1	0.305192682	3.07E-12	postive
COX17	AJ009632.2	0.342413188	3.36E-15	postive
COX17	AL008718.2	0.301782117	5.48E-12	postive
COX17	AL020996.1	0.413847691	4.14E-22	postive
COX17	AL023284.4	0.360134942	9.31E-17	postive
COX17	AL023803.1	0.434029931	2.19E-24	postive
COX17	AL023803.2	0.332507446	2.26E-14	postive
COX17	AL023803.3	0.326321123	7.19E-14	postive

COX17	AL024498.1	0.432460072	3.34E-24	postive
COX17	AL024507.3	0.408474888	1.58E-21	postive
COX17	AL024508.1	0.366296808	2.54E-17	postive
COX17	AL031055.1	0.464022923	4.60E-28	postive
COX17	AL031429.1	0.329977678	3.64E-14	postive
COX17	AL031432.3	0.352777893	4.24E-16	postive
COX17	AL031847.1	0.327243349	6.06E-14	postive
COX17	AL031963.3	0.369849022	1.18E-17	postive
COX17	AL033527.3	0.330781251	3.13E-14	postive
COX17	AL049597.2	0.427391177	1.28E-23	postive
COX17	AL049836.1	0.346380831	1.53E-15	postive
COX17	AL050309.1	0.462827496	6.55E-28	postive
COX17	AL050341.2	0.358687946	1.26E-16	postive
COX17	AL078590.2	0.393514359	5.75E-20	postive
COX17	AL078644.2	0.319137705	2.67E-13	postive
COX17	AL080250.1	0.418237548	1.36E-22	postive
COX17	AL080276.2	0.319437935	2.53E-13	postive
COX17	AL096828.3	0.534218404	3.07E-38	postive
COX17	AL109917.1	0.332189211	2.40E-14	postive
COX17	AL117332.1	0.54053742	2.81E-39	postive
COX17	AL117336.2	0.460835802	1.18E-27	postive
COX17	AL118506.1	0.392525446	7.25E-20	postive
COX17	AL118516.1	0.403473756	5.35E-21	postive
COX17	AL118523.1	0.302741277	4.66E-12	postive
COX17	AL118558.3	0.521801021	2.90E-36	postive
COX17	AL121574.1	0.402603094	6.61E-21	postive
COX17	AL121601.1	0.48189216	1.96E-30	postive
COX17	AL121603.2	0.329033955	4.34E-14	postive
COX17	AL121658.1	0.314960672	5.62E-13	postive
COX17	AL121832.2	0.364707646	3.56E-17	postive
COX17	AL121944.2	0.506695741	5.75E-34	postive
COX17	AL121970.1	0.376716733	2.64E-18	postive
COX17	AL122010.1	0.311778107	9.84E-13	postive
COX17	AL122023.3	0.30630185	2.54E-12	postive
COX17	AL132639.3	0.302190361	5.12E-12	postive
COX17	AL132712.1	0.372210852	7.09E-18	postive
COX17	AL133153.2	0.394272642	4.81E-20	postive
COX17	AL133215.2	0.504364127	1.27E-33	postive
COX17	AL133338.1	0.330954008	3.03E-14	postive
COX17	AL133353.1	0.380044501	1.26E-18	postive
COX17	AL133551.1	0.315773859	4.86E-13	postive
COX17	AL135744.1	0.466166433	2.43E-28	postive
COX17	AL136040.1	0.402848998	6.23E-21	postive
COX17	AL136162.1	0.36299059	5.11E-17	postive
COX17	AL136307.1	0.315267888	5.32E-13	postive
COX17	AL136366.1	0.37014315	1.11E-17	postive
COX17	AL137127.1	0.302781518	4.63E-12	postive
COX17	AL137186.2	0.394832578	4.22E-20	postive
COX17	AL137802.2	0.337768437	8.29E-15	postive
COX17	AL138724.2	0.427957336	1.10E-23	postive

COX17	AL138756.1	0.303963643	3.79E-12	postive
COX17	AL138762.2	0.337587717	8.58E-15	postive
COX17	AL139011.1	0.31355444	7.21E-13	postive
COX17	AL139035.1	0.419367479	1.02E-22	postive
COX17	AL139039.3	0.307386863	2.11E-12	postive
COX17	AL139246.5	0.313119482	7.78E-13	postive
COX17	AL139260.1	0.45947996	1.75E-27	postive
COX17	AL139289.1	0.479012248	4.82E-30	postive
COX17	AL139384.1	0.475222403	1.56E-29	postive
COX17	AL157400.2	0.345096539	1.98E-15	postive
COX17	AL157813.1	0.351071905	5.99E-16	postive
COX17	AL157904.1	0.314335636	6.28E-13	postive
COX17	AL158154.3	0.30771815	1.99E-12	postive
COX17	AL160313.2	0.309862518	1.37E-12	postive
COX17	AL161421.1	0.457330406	3.27E-27	postive
COX17	AL161729.1	0.332695478	2.18E-14	postive
COX17	AL161772.1	0.328947541	4.41E-14	postive
COX17	AL161937.1	0.401597618	8.42E-21	postive
COX17	AL162231.2	0.320845926	1.96E-13	postive
COX17	AL162258.2	0.300853789	6.41E-12	postive
COX17	AL162377.1	0.389704804	1.40E-19	postive
COX17	AL163051.1	0.316830612	4.03E-13	postive
COX17	AL353194.1	0.343154453	2.90E-15	postive
COX17	AL353708.1	0.399785266	1.30E-20	postive
COX17	AL353708.3	0.35637933	2.03E-16	postive
COX17	AL353743.1	0.304417243	3.51E-12	postive
COX17	AL353807.2	0.345484595	1.83E-15	postive
COX17	AL354696.2	0.318601934	2.94E-13	postive
COX17	AL354707.1	0.340870549	4.54E-15	postive
COX17	AL354718.3	0.457664834	2.97E-27	postive
COX17	AL354740.1	0.400030717	1.23E-20	postive
COX17	AL354824.1	0.367379881	2.01E-17	postive
COX17	AL354824.2	0.357175235	1.72E-16	postive
COX17	AL354892.2	0.352952834	4.09E-16	postive
COX17	AL354920.1	0.498617542	8.75E-33	postive
COX17	AL354928.1	0.336224204	1.11E-14	postive
COX17	AL355001.2	0.540929548	2.42E-39	postive
COX17	AL355297.3	0.326454503	7.02E-14	postive
COX17	AL355300.1	0.32891483	4.44E-14	postive
COX17	AL355312.3	0.362567227	5.59E-17	postive
COX17	AL355353.2	0.386580677	2.86E-19	postive
COX17	AL355574.1	0.397386657	2.31E-20	postive
COX17	AL355607.2	0.314942495	5.64E-13	postive
COX17	AL356481.1	0.405020044	3.68E-21	postive
COX17	AL356488.3	0.314379169	6.23E-13	postive
COX17	AL357033.2	0.352808156	4.21E-16	postive
COX17	AL357079.1	0.331861749	2.56E-14	postive
COX17	AL357140.2	0.377666245	2.14E-18	postive
COX17	AL357874.1	0.309119888	1.56E-12	postive
COX17	AL357992.1	0.320912149	1.93E-13	postive

COX17	AL358075.2	0.31507009	5.51E-13	postive
COX17	AL358472.2	0.44750456	5.41E-26	postive
COX17	AL358472.3	0.404768921	3.91E-21	postive
COX17	AL358852.1	0.432123438	3.65E-24	postive
COX17	AL359091.3	0.400339405	1.14E-20	postive
COX17	AL359182.1	0.3615952	6.86E-17	postive
COX17	AL359198.1	0.327436046	5.85E-14	postive
COX17	AL359513.1	0.412646877	5.59E-22	postive
COX17	AL359711.2	0.437663566	8.21E-25	postive
COX17	AL359715.3	0.379608848	1.39E-18	postive
COX17	AL359921.2	0.303346749	4.21E-12	postive
COX17	AL359962.1	0.44190578	2.57E-25	postive
COX17	AL359962.2	0.346537704	1.49E-15	postive
COX17	AL365181.3	0.314252169	6.37E-13	postive
COX17	AL390198.1	0.382083764	7.95E-19	postive
COX17	AL390955.2	0.394752898	4.30E-20	postive
COX17	AL391069.2	0.366637065	2.36E-17	postive
COX17	AL391832.2	0.414972946	3.12E-22	postive
COX17	AL391832.3	0.319757745	2.38E-13	postive
COX17	AL392172.1	0.459968521	1.52E-27	postive
COX17	AL441992.2	0.675808709	5.54E-68	postive
COX17	AL442663.3	0.311057221	1.12E-12	postive
COX17	AL445228.3	0.34683165	1.40E-15	postive
COX17	AL445231.1	0.378010854	1.98E-18	postive
COX17	AL445524.1	0.322342972	1.49E-13	postive
COX17	AL445645.1	0.302529847	4.83E-12	postive
COX17	AL449106.1	0.320302419	2.16E-13	postive
COX17	AL450468.1	0.319444931	2.52E-13	postive
COX17	AL450998.2	0.447288326	5.75E-26	postive
COX17	AL451050.2	0.437807017	7.89E-25	postive
COX17	AL451069.3	0.335486357	1.28E-14	postive
COX17	AL451085.2	0.360155187	9.27E-17	postive
COX17	AL451085.3	0.496070538	2.03E-32	postive
COX17	AL451165.2	0.543331402	9.62E-40	postive
COX17	AL512408.1	0.337156774	9.32E-15	postive
COX17	AL512598.1	0.334311575	1.61E-14	postive
COX17	AL512625.2	0.308849783	1.64E-12	postive
COX17	AL513550.1	0.371251519	8.73E-18	postive
COX17	AL583722.1	0.400210864	1.18E-20	postive
COX17	AL589765.4	0.305813258	2.76E-12	postive
COX17	AL590096.1	0.320986538	1.91E-13	postive
COX17	AL590428.1	0.305713132	2.81E-12	postive
COX17	AL590617.2	0.408035561	1.76E-21	postive
COX17	AL590666.2	0.311635941	1.01E-12	postive
COX17	AL590705.1	0.343088629	2.94E-15	postive
COX17	AL592166.1	0.404031488	4.68E-21	postive
COX17	AL592435.1	0.307960512	1.91E-12	postive
COX17	AL592494.2	0.375805409	3.22E-18	postive
COX17	AL596094.1	0.410717227	9.05E-22	postive
COX17	AL604028.1	0.366127006	2.63E-17	postive

COX17	AL606489.1	0.343924215	2.50E-15	postive
COX17	AL606807.1	0.342086373	3.58E-15	postive
COX17	AL606834.1	0.322927002	1.34E-13	postive
COX17	AL645568.1	0.353361887	3.76E-16	postive
COX17	AL645728.1	0.314681614	5.90E-13	postive
COX17	AL671710.1	0.302651341	4.73E-12	postive
COX17	AL691403.2	0.349444604	8.32E-16	postive
COX17	AL731533.2	0.387558485	2.29E-19	postive
COX17	AL731569.1	0.327558066	5.72E-14	postive
COX17	AL732314.8	0.363593115	4.50E-17	postive
COX17	AL807752.5	0.356428855	2.01E-16	postive
COX17	AL928654.1	0.348728362	9.61E-16	postive
COX17	ALDH1L1-AS2	0.322820232	1.37E-13	postive
COX17	ALG1L13P	0.345386376	1.87E-15	postive
COX17	ALOX12-AS1	0.373540216	5.30E-18	postive
COX17	AMZ2P1	0.426198564	1.75E-23	postive
COX17	ANO7L1	0.354356715	3.07E-16	postive
COX17	AP000251.1	0.404633437	4.04E-21	postive
COX17	AP000295.10	0.314216447	6.41E-13	postive
COX17	AP000350.5	0.306151646	2.61E-12	postive
COX17	AP000442.1	0.327613457	5.66E-14	postive
COX17	AP000640.2	0.312323496	8.95E-13	postive
COX17	AP000704.2	0.314029358	6.63E-13	postive
COX17	AP000759.1	0.330763348	3.14E-14	postive
COX17	AP000769.1	0.422148508	5.00E-23	postive
COX17	AP000781.1	0.3057709	2.78E-12	postive
COX17	AP000894.4	0.354846322	2.78E-16	postive
COX17	AP001160.2	0.416219364	2.28E-22	postive
COX17	AP001160.3	0.419037664	1.11E-22	postive
COX17	AP001189.5	0.332141481	2.42E-14	postive
COX17	AP001269.2	0.318540687	2.97E-13	postive
COX17	AP001372.2	0.432042875	3.73E-24	postive
COX17	AP001412.1	0.347401471	1.25E-15	postive
COX17	AP001453.4	0.348061562	1.10E-15	postive
COX17	AP001505.1	0.339574939	5.84E-15	postive
COX17	AP001527.2	0.357824427	1.51E-16	postive
COX17	AP001816.1	0.416856652	1.94E-22	postive
COX17	AP001922.5	0.356775857	1.87E-16	postive
COX17	AP002008.3	0.346000319	1.66E-15	postive
COX17	AP002360.2	0.525462235	7.74E-37	postive
COX17	AP002387.2	0.455559363	5.46E-27	postive
COX17	AP002748.4	0.523179321	1.77E-36	postive
COX17	AP003068.1	0.333275515	1.96E-14	postive
COX17	AP003068.2	0.313529537	7.24E-13	postive
COX17	AP003119.2	0.445397041	9.76E-26	postive
COX17	AP003352.1	0.313167644	7.71E-13	postive
COX17	AP003392.5	0.337977363	7.96E-15	postive
COX17	AP003465.2	0.300883134	6.38E-12	postive
COX17	AP003469.1	0.336570411	1.04E-14	postive
COX17	AP003469.2	0.396284951	2.99E-20	postive

COX17	AP003721.4	0.363267306	4.82E-17	postive
COX17	AP004609.5	0.442004162	2.50E-25	postive
COX17	AP005205.2	0.3029223	4.52E-12	postive
COX17	AP005482.4	0.335907138	1.18E-14	postive
COX17	AP006287.2	0.330659681	3.20E-14	postive
COX17	AP006621.3	0.48830582	2.54E-31	postive
COX17	APTR	0.318461704	3.01E-13	postive
COX17	ARF4-AS1	0.457527544	3.09E-27	postive
COX17	ARHGAP31-AS1	0.411803805	6.90E-22	postive
COX17	ARHGAP5-AS1	0.301873815	5.40E-12	postive
COX17	ARHGEF26-AS1	0.300708574	6.57E-12	postive
COX17	ARMCX5-GPRASP2	0.433296228	2.67E-24	postive
COX17	ARRDC1-AS1	0.630243246	1.03E-56	postive
COX17	ASB16-AS1	0.406769898	2.40E-21	postive
COX17	ASH1L-AS1	0.377934683	2.01E-18	postive
COX17	ATF4P4	0.321222562	1.83E-13	postive
COX17	ATP2A1-AS1	0.461466186	9.78E-28	postive
COX17	ATXN2-AS	0.495723634	2.28E-32	postive
COX17	AZIN1-AS1	0.363024561	5.08E-17	postive
COX17	B4GALT1-AS1	0.34266966	3.19E-15	postive
COX17	BAALC-AS1	0.343233798	2.86E-15	postive
COX17	BCDIN3D-AS1	0.41384513	4.14E-22	postive
COX17	BHLHE40-AS1	0.340605747	4.78E-15	postive
COX17	BMS1P20	0.333719628	1.80E-14	postive
COX17	BOLA3-AS1	0.320461453	2.10E-13	postive
COX17	BRWD1-AS2	0.380336726	1.18E-18	postive
COX17	BTG3-AS1	0.328212917	5.06E-14	postive
COX17	BVES-AS1	0.379943862	1.29E-18	postive
COX17	BX255925.1	0.317790509	3.39E-13	postive
COX17	BX284668.5	0.457356928	3.25E-27	postive
COX17	BX470209.1	0.382658405	6.99E-19	postive
COX17	BX470209.2	0.366293868	2.54E-17	postive
COX17	BX537318.1	0.390520565	1.16E-19	postive
COX17	BX539320.1	0.41800422	1.45E-22	postive
COX17	BX649601.1	0.332983974	2.07E-14	postive
COX17	BX649632.1	0.401722808	8.17E-21	postive
COX17	C10orf25	0.316070237	4.62E-13	postive
COX17	C18orf65	0.317754854	3.42E-13	postive
COX17	C1orf229	0.307671994	2.01E-12	postive
COX17	C1QTNF1-AS1	0.407885145	1.82E-21	postive
COX17	C22orf24	0.307810532	1.96E-12	postive
COX17	C2CD4D-AS1	0.308884015	1.63E-12	postive
COX17	C5orf66	0.3005303	6.77E-12	postive
COX17	C6orf99	0.471946929	4.25E-29	postive
COX17	C9orf147	0.350076378	7.33E-16	postive
COX17	C9orf163	0.431529649	4.27E-24	postive
COX17	CA5BP1	0.455746999	5.18E-27	postive
COX17	CAHM	0.378827532	1.65E-18	postive
COX17	CAMTA1-DT	0.347516412	1.22E-15	postive
COX17	CAPN10-DT	0.333602947	1.84E-14	postive

COX17	CASC9	0.3268349	6.54E-14	postive
COX17	CATIP-AS1	0.429868699	6.65E-24	postive
COX17	CATSPER2P1	0.488876691	2.12E-31	postive
COX17	CCNT2-AS1	0.358333737	1.35E-16	postive
COX17	CD27-AS1	0.491556895	8.90E-32	postive
COX17	CD99P1	0.383759156	5.45E-19	postive
COX17	CDC37L1-DT	0.389050445	1.62E-19	postive
COX17	CEACAMP10	0.300415681	6.90E-12	postive
COX17	CENPBD1P1	0.433712909	2.38E-24	postive
COX17	CEP83-DT	0.384926756	4.18E-19	postive
COX17	CHKB-DT	0.4876374	3.15E-31	postive
COX17	CIDECP1	0.658202662	2.15E-63	postive
COX17	CIRBP-AS1	0.305839357	2.75E-12	postive
COX17	CKMT2-AS1	0.318561253	2.96E-13	postive
COX17	CNNM3-DT	0.386335016	3.03E-19	postive
COX17	COA6-AS1	0.451176649	1.92E-26	postive
COX17	CPNE8-AS1	0.401988891	7.66E-21	postive
COX17	CR381653.1	0.361282635	7.32E-17	postive
COX17	CRNDE	0.307251911	2.16E-12	postive
COX17	CRTC3-AS1	0.343265578	2.84E-15	postive
COX17	CRYBB2P1	0.408496233	1.57E-21	postive
COX17	CRYZL2P	0.404413337	4.26E-21	postive
COX17	CT66	0.31083399	1.16E-12	postive
COX17	CTA-989H11.1	0.388298723	1.93E-19	postive
COX17	CTBP1-DT	0.396615292	2.77E-20	postive
COX17	CTD-2134A5.3	0.316579972	4.21E-13	postive
COX17	CTD-2521M24.9	0.36950045	1.28E-17	postive
COX17	CTD-2540M10.1	0.397779471	2.10E-20	postive
COX17	CYP4F23P	0.40572755	3.09E-21	postive
COX17	CYP51A1-AS1	0.335755962	1.22E-14	postive
COX17	CYTOR	0.472811602	3.26E-29	postive
COX17	DANCR	0.47504127	1.65E-29	postive
COX17	DARS-AS1	0.437345357	8.95E-25	postive
COX17	DBIL5P	0.437756284	8.00E-25	postive
COX17	DCST1-AS1	0.526650647	5.02E-37	postive
COX17	DCXR-DT	0.317873795	3.34E-13	postive
COX17	DDX11-AS1	0.446644215	6.89E-26	postive
COX17	DDX39B-AS1	0.377701845	2.12E-18	postive
COX17	DEPDC1-AS1	0.367288532	2.05E-17	postive
COX17	DGUOK-AS1	0.389205764	1.57E-19	postive
COX17	DHCR24-DT	0.389747069	1.38E-19	postive
COX17	DHRS4-AS1	0.393944694	5.20E-20	postive
COX17	DHRS4L1	0.326563615	6.88E-14	postive
COX17	DLG1-AS1	0.384085375	5.06E-19	postive
COX17	DLG3-AS1	0.308151497	1.85E-12	postive
COX17	DLG5-AS1	0.454391893	7.65E-27	postive
COX17	DLGAP1-AS1	0.399661643	1.34E-20	postive
COX17	DM1-AS	0.343403289	2.77E-15	postive
COX17	DNAJC27-AS1	0.302088049	5.21E-12	postive
COX17	DNAJC3-DT	0.40071396	1.04E-20	postive

COX17	DPH6-DT	0.303137655	4.36E-12	postive
COX17	DYRK3-AS1	0.339546449	5.87E-15	postive
COX17	EBLN3P	0.383966284	5.20E-19	postive
COX17	EIF1B-AS1	0.354294772	3.11E-16	postive
COX17	EIF2AK3-DT	0.400355687	1.14E-20	postive
COX17	EIF3J-DT	0.404097906	4.60E-21	postive
COX17	ELF3-AS1	0.47409491	2.20E-29	postive
COX17	ELOA-AS1	0.445617046	9.18E-26	postive
COX17	EMC1-AS1	0.48739736	3.41E-31	postive
COX17	EMC3-AS1	0.306929978	2.28E-12	postive
COX17	ENO1-AS1	0.394836924	4.21E-20	postive
COX17	ENTPD3-AS1	0.467039572	1.87E-28	postive
COX17	EPCAM-DT	0.309879923	1.37E-12	postive
COX17	ETV7-AS1	0.343250972	2.85E-15	postive
COX17	EXOC3-AS1	0.390904811	1.06E-19	postive
COX17	EXOSC10-AS1	0.557692908	3.30E-42	postive
COX17	F10-AS1	0.337905627	8.07E-15	postive
COX17	FALEC	0.310770952	1.17E-12	postive
COX17	FAM111A-DT	0.374550476	4.25E-18	postive
COX17	FAM21EP	0.316382309	4.37E-13	postive
COX17	FAM21FP	0.39905912	1.55E-20	postive
COX17	FAM86C2P	0.373961312	4.84E-18	postive
COX17	FAM86EP	0.368707275	1.51E-17	postive
COX17	FAM86HP	0.334863911	1.45E-14	postive
COX17	FAM86JP	0.608463426	5.81E-52	postive
COX17	FBP2P1	0.336450272	1.07E-14	postive
COX17	FBXL19-AS1	0.302025566	5.26E-12	postive
COX17	FGD5-AS1	0.488631322	2.29E-31	postive
COX17	FGF14-AS2	0.354743728	2.84E-16	postive
COX17	FLVCR1-DT	0.340570913	4.81E-15	postive
COX17	FO393418.1	0.407817956	1.85E-21	postive
COX17	FOXD2-AS1	0.348284959	1.05E-15	postive
COX17	FOXD3-AS1	0.395507599	3.60E-20	postive
COX17	FRG1HP	0.477390657	7.98E-30	postive
COX17	FRGCA	0.361575311	6.89E-17	postive
COX17	FZD4-DT	0.427901629	1.12E-23	postive
COX17	GAS5-AS1	0.375789116	3.24E-18	postive
COX17	GASAL1	0.37269733	6.37E-18	postive
COX17	GATA2-AS1	0.450852256	2.10E-26	postive
COX17	GBAP1	0.359937248	9.70E-17	postive
COX17	GCC2-AS1	0.41402707	3.96E-22	postive
COX17	GIHCG	0.466589619	2.14E-28	postive
COX17	GLIDR	0.345422525	1.86E-15	postive
COX17	GNRHR2	0.322910281	1.35E-13	postive
COX17	GOLGA8EP	0.35305704	4.01E-16	postive
COX17	GPR176-DT	0.320639401	2.03E-13	postive
COX17	GPRC5D-AS1	0.391181365	9.91E-20	postive
COX17	GSEC	0.42678654	1.50E-23	postive
COX17	GTF2IRD1P1	0.311916976	9.61E-13	postive
COX17	GTSE1-DT	0.325755815	7.98E-14	postive

COX17	GYG2P1	0.354736391	2.84E-16	postive
COX17	H1FX-AS1	0.312677976	8.41E-13	postive
COX17	H2AC10P	0.322971039	1.33E-13	postive
COX17	H2AZ2P1	0.329269449	4.16E-14	postive
COX17	HAR1A	0.345197803	1.94E-15	postive
COX17	HCG18	0.330837126	3.10E-14	postive
COX17	HCG25	0.378685322	1.70E-18	postive
COX17	HCP5	0.319504438	2.50E-13	postive
COX17	HDAC4-AS1	0.31939646	2.54E-13	postive
COX17	HDHD5-AS1	0.345547053	1.81E-15	postive
COX17	HEIH	0.48727912	3.54E-31	postive
COX17	HM13-AS1	0.329273079	4.15E-14	postive
COX17	HMGB1P5	0.463420633	5.49E-28	postive
COX17	HMMR-AS1	0.344784219	2.11E-15	postive
COX17	HPN-AS1	0.312593658	8.53E-13	postive
COX17	IDH1-AS1	0.484206496	9.42E-31	postive
COX17	IL6R-AS1	0.330992148	3.01E-14	postive
COX17	ILF3-DT	0.384804871	4.30E-19	postive
COX17	IPO9-AS1	0.374969803	3.88E-18	postive
COX17	IQCH-AS1	0.427259666	1.32E-23	postive
COX17	IRAIN	0.308019776	1.89E-12	postive
COX17	ITFG2-AS1	0.440643602	3.64E-25	postive
COX17	JPX	0.483888339	1.04E-30	postive
COX17	KCNMB2-AS1	0.447813255	4.96E-26	postive
COX17	KCNQ1DN	0.343384318	2.78E-15	postive
COX17	KDM2B-DT	0.321750023	1.66E-13	postive
COX17	KDM7A-DT	0.425374011	2.17E-23	postive
COX17	KMT2E-AS1	0.556861475	4.61E-42	postive
COX17	KRT8P12	0.523569302	1.54E-36	postive
COX17	KTN1-AS1	0.382804411	6.76E-19	postive
COX17	LAMA5-AS1	0.340942558	4.48E-15	postive
COX17	LAMTOR5-AS1	0.317796918	3.39E-13	postive
COX17	LAPTM4A-DT	0.334014073	1.70E-14	postive
COX17	LBX2-AS1	0.466529232	2.18E-28	postive
COX17	LGALS8-AS1	0.446310815	7.56E-26	postive
COX17	LINC00235	0.317947657	3.30E-13	postive
COX17	LINC00239	0.394993301	4.06E-20	postive
COX17	LINC00323	0.380961414	1.02E-18	postive
COX17	LINC00324	0.329408414	4.05E-14	postive
COX17	LINC00339	0.474488943	1.95E-29	postive
COX17	LINC00346	0.361721126	6.68E-17	postive
COX17	LINC00449	0.331256671	2.86E-14	postive
COX17	LINC00466	0.36933171	1.32E-17	postive
COX17	LINC00467	0.488513366	2.38E-31	postive
COX17	LINC00471	0.378999622	1.59E-18	postive
COX17	LINC00476	0.451025382	2.00E-26	postive
COX17	LINC00494	0.327427084	5.86E-14	postive
COX17	LINC00511	0.438145514	7.20E-25	postive
COX17	LINC00526	0.326960285	6.39E-14	postive
COX17	LINC00589	0.313193818	7.68E-13	postive

COX17	LINC00623	0.408972181	1.39E-21	postive
COX17	LINC00628	0.304724617	3.33E-12	postive
COX17	LINC00630	0.403478438	5.35E-21	postive
COX17	LINC00654	0.302683922	4.71E-12	postive
COX17	LINC00659	0.40637162	2.64E-21	postive
COX17	LINC00667	0.32377054	1.15E-13	postive
COX17	LINC00680	0.36707639	2.15E-17	postive
COX17	LINC00847	0.576251579	1.41E-45	postive
COX17	LINC00853	0.365758418	2.84E-17	postive
COX17	LINC00857	0.31577534	4.86E-13	postive
COX17	LINC00863	0.317637821	3.49E-13	postive
COX17	LINC00869	0.436985612	9.86E-25	postive
COX17	LINC00884	0.508374303	3.24E-34	postive
COX17	LINC00887	0.437216663	9.26E-25	postive
COX17	LINC00888	0.623486062	3.37E-55	postive
COX17	LINC00909	0.431709336	4.07E-24	postive
COX17	LINC00910	0.336980075	9.64E-15	postive
COX17	LINC00963	0.526091975	6.15E-37	postive
COX17	LINC01003	0.376741435	2.62E-18	postive
COX17	LINC01006	0.354428575	3.03E-16	postive
COX17	LINC01011	0.388883908	1.69E-19	postive
COX17	LINC01023	0.481314043	2.35E-30	postive
COX17	LINC01063	0.55789174	3.04E-42	postive
COX17	LINC01093	0.38452742	4.57E-19	postive
COX17	LINC01124	0.377409993	2.26E-18	postive
COX17	LINC01132	0.331716342	2.63E-14	postive
COX17	LINC01137	0.44352454	1.64E-25	postive
COX17	LINC01138	0.403219916	5.69E-21	postive
COX17	LINC01144	0.340334019	5.04E-15	postive
COX17	LINC01145	0.339288113	6.18E-15	postive
COX17	LINC01151	0.341663041	3.89E-15	postive
COX17	LINC01184	0.490178993	1.39E-31	postive
COX17	LINC01220	0.439465704	5.02E-25	postive
COX17	LINC01232	0.463670838	5.10E-28	postive
COX17	LINC01270	0.407051382	2.24E-21	postive
COX17	LINC01271	0.402701061	6.45E-21	postive
COX17	LINC01273	0.457154975	3.44E-27	postive
COX17	LINC01275	0.442924102	1.94E-25	postive
COX17	LINC01278	0.439360547	5.17E-25	postive
COX17	LINC01285	0.343533054	2.70E-15	postive
COX17	LINC01293	0.30081098	6.45E-12	postive
COX17	LINC01301	0.35149141	5.51E-16	postive
COX17	LINC01311	0.349801209	7.74E-16	postive
COX17	LINC01348	0.376177555	2.97E-18	postive
COX17	LINC01354	0.340800334	4.60E-15	postive
COX17	LINC01366	0.381371074	9.34E-19	postive
COX17	LINC01387	0.3378551	8.15E-15	postive
COX17	LINC01431	0.385744831	3.47E-19	postive
COX17	LINC01497	0.349612401	8.04E-16	postive
COX17	LINC01521	0.326595251	6.84E-14	postive

COX17	LINC01560	0.390231328	1.24E-19	postive
COX17	LINC01569	0.308139064	1.85E-12	postive
COX17	LINC01578	0.318497883	2.99E-13	postive
COX17	LINC01585	0.346829952	1.40E-15	postive
COX17	LINC01588	0.35277108	4.25E-16	postive
COX17	LINC01607	0.368419267	1.61E-17	postive
COX17	LINC01621	0.354136774	3.21E-16	postive
COX17	LINC01686	0.424715084	2.57E-23	postive
COX17	LINC01693	0.3502213	7.12E-16	postive
COX17	LINC01703	0.435680396	1.40E-24	postive
COX17	LINC01715	0.358725541	1.25E-16	postive
COX17	LINC01719	0.330354778	3.39E-14	postive
COX17	LINC01770	0.350942303	6.15E-16	postive
COX17	LINC01806	0.484694992	8.06E-31	postive
COX17	LINC01814	0.330871226	3.08E-14	postive
COX17	LINC01842	0.38029565	1.19E-18	postive
COX17	LINC01843	0.356638753	1.92E-16	postive
COX17	LINC01881	0.320513369	2.08E-13	postive
COX17	LINC01888	0.306057807	2.65E-12	postive
COX17	LINC01952	0.396634909	2.76E-20	postive
COX17	LINC01968	0.314739944	5.84E-13	postive
COX17	LINC01972	0.333609601	1.84E-14	postive
COX17	LINC01975	0.32829959	4.98E-14	postive
COX17	LINC02000	0.358474522	1.32E-16	postive
COX17	LINC02004	0.358182363	1.40E-16	postive
COX17	LINC02012	0.35964048	1.03E-16	postive
COX17	LINC02014	0.474528954	1.93E-29	postive
COX17	LINC02021	0.339349135	6.10E-15	postive
COX17	LINC02026	0.490352244	1.32E-31	postive
COX17	LINC02028	0.389880337	1.34E-19	postive
COX17	LINC02041	0.349592126	8.08E-16	postive
COX17	LINC02062	0.406759864	2.40E-21	postive
COX17	LINC02076	0.361195554	7.46E-17	postive
COX17	LINC02099	0.347385622	1.26E-15	postive
COX17	LINC02130	0.328351475	4.93E-14	postive
COX17	LINC02137	0.347490668	1.23E-15	postive
COX17	LINC02210	0.322909036	1.35E-13	postive
COX17	LINC02298	0.398074963	1.96E-20	postive
COX17	LINC02321	0.337354055	8.97E-15	postive
COX17	LINC02356	0.315457954	5.15E-13	postive
COX17	LINC02412	0.327545966	5.73E-14	postive
COX17	LINC02453	0.354008138	3.30E-16	postive
COX17	LINC02482	0.452972571	1.15E-26	postive
COX17	LINC02585	0.376834685	2.57E-18	postive
COX17	LINC02601	0.417307857	1.73E-22	postive
COX17	LINC02609	0.321156724	1.85E-13	postive
COX17	LINC02611	0.325005407	9.16E-14	postive
COX17	LINC02637	0.442269654	2.32E-25	postive
COX17	LINC02707	0.358877325	1.21E-16	postive
COX17	LINC02709	0.453925573	8.74E-27	postive

COX17	LINC02740	0.30140534	5.84E-12	postive
COX17	LINC02749	0.414792293	3.26E-22	postive
COX17	LINC02757	0.319861478	2.34E-13	postive
COX17	LINC02762	0.408982994	1.39E-21	postive
COX17	LINC02766	0.320746728	1.99E-13	postive
COX17	LINC02777	0.317066788	3.86E-13	postive
COX17	LINC02851	0.326908573	6.45E-14	postive
COX17	LIPE-AS1	0.460899123	1.16E-27	postive
COX17	LMCD1-AS1	0.301110497	6.14E-12	postive
COX17	LMO7-AS1	0.357875688	1.49E-16	postive
COX17	LNCTAM34A	0.351713846	5.26E-16	postive
COX17	LNX1-AS2	0.337378871	8.93E-15	postive
COX17	LOH12CR2	0.442177466	2.38E-25	postive
COX17	LOXL1-AS1	0.36429373	3.88E-17	postive
COX17	LPP-AS2	0.464628206	3.84E-28	postive
COX17	LRRC37BP1	0.317312968	3.70E-13	postive
COX17	LSP1P4	0.401829213	7.97E-21	postive
COX17	MAFG-DT	0.398779709	1.66E-20	postive
COX17	MAN1B1-DT	0.422918385	4.10E-23	postive
COX17	MAP3K14-AS1	0.341460736	4.05E-15	postive
COX17	MAP3K2-DT	0.40766498	1.92E-21	postive
COX17	MAP4K3-DT	0.43064811	5.40E-24	postive
COX17	MAPK6-DT	0.467073741	1.85E-28	postive
COX17	MAPKAPK5-AS1	0.661705788	2.78E-64	postive
COX17	MCF2L-AS1	0.309428522	1.48E-12	postive
COX17	MCPH1-AS1	0.327324394	5.97E-14	postive
COX17	MED14OS	0.42847755	9.60E-24	postive
COX17	MELTF-AS1	0.492524231	6.50E-32	postive
COX17	MHENCR	0.311227293	1.08E-12	postive
COX17	MIATNB	0.314643895	5.94E-13	postive
COX17	MIF-AS1	0.311236042	1.08E-12	postive
COX17	MINCR	0.425640783	2.02E-23	postive
COX17	MIPEPP3	0.368234278	1.68E-17	postive
COX17	MIR193BHG	0.409977972	1.09E-21	postive
COX17	MIR200CHG	0.474758268	1.80E-29	postive
COX17	MIR210HG	0.322749038	1.39E-13	postive
COX17	MIR22HG	0.302399981	4.94E-12	postive
COX17	MIR3150BHG	0.356733977	1.89E-16	postive
COX17	MIR4435-2HG	0.474452669	1.97E-29	postive
COX17	MIR4458HG	0.389037381	1.63E-19	postive
COX17	MIR583HG	0.311409443	1.05E-12	postive
COX17	MIR762HG	0.500689771	4.38E-33	postive
COX17	MIR924HG	0.373509309	5.34E-18	postive
COX17	MKLN1-AS	0.408767857	1.47E-21	postive
COX17	MMADHC-DT	0.301555706	5.69E-12	postive
COX17	MRPL20-AS1	0.444814024	1.15E-25	postive
COX17	MRPL45P2	0.344620844	2.18E-15	postive
COX17	MSH5-SAPCD1	0.303309205	4.23E-12	postive
COX17	MSL3P1	0.38455248	4.55E-19	postive
COX17	MT1DP	0.337233128	9.18E-15	postive

COX17	MYLK-AS1	0.42477674	2.53E-23	postive
COX17	NACA4P	0.406998483	2.27E-21	postive
COX17	NAPA-AS1	0.389270825	1.54E-19	postive
COX17	NBR2	0.449216268	3.34E-26	postive
COX17	NCK1-DT	0.566388386	9.25E-44	postive
COX17	NDUFB2-AS1	0.44582591	8.66E-26	postive
COX17	NEBL-AS1	0.451447897	1.77E-26	postive
COX17	NIFK-AS1	0.524961269	9.28E-37	postive
COX17	NINJ2-AS1	0.368149103	1.71E-17	postive
COX17	NKAPP1	0.331176694	2.91E-14	postive
COX17	NNT-AS1	0.381763565	8.55E-19	postive
COX17	NOP14-AS1	0.389236112	1.56E-19	postive
COX17	NRAV	0.583087586	7.14E-47	postive
COX17	NRSN2-AS1	0.62062697	1.44E-54	postive
COX17	NSUN5P2	0.314720957	5.86E-13	postive
COX17	NUDT16P1	0.622967294	4.39E-55	postive
COX17	NUP50-DT	0.479523362	4.11E-30	postive
COX17	NUTM2A-AS1	0.480949184	2.63E-30	postive
COX17	OGFRP1	0.308411262	1.77E-12	postive
COX17	OIP5-AS1	0.418148848	1.40E-22	postive
COX17	OLMALINC	0.464510199	3.98E-28	postive
COX17	OR2A9P	0.318696277	2.89E-13	postive
COX17	OR4G11P	0.340402285	4.97E-15	postive
COX17	OR7E14P	0.374842981	3.98E-18	postive
COX17	OSER1-DT	0.518753932	8.62E-36	postive
COX17	OTUD6B-AS1	0.446378898	7.42E-26	postive
COX17	PAXIP1-AS1	0.410958826	8.52E-22	postive
COX17	PCAT6	0.340104207	5.27E-15	postive
COX17	PCBP1-AS1	0.413245441	4.82E-22	postive
COX17	PCCA-DT	0.438043574	7.40E-25	postive
COX17	PDCD4-AS1	0.361960177	6.35E-17	postive
COX17	PDIA3P1	0.360408349	8.79E-17	postive
COX17	PINK1-AS	0.343295986	2.82E-15	postive
COX17	PITPNA-AS1	0.584644168	3.58E-47	postive
COX17	PLEKHA8P1	0.432200155	3.57E-24	postive
COX17	PMS2CL	0.356591535	1.94E-16	postive
COX17	PMS2P4	0.454862247	6.68E-27	postive
COX17	POLR2J4	0.440649275	3.63E-25	postive
COX17	PPP1R12A-AS1	0.304302916	3.58E-12	postive
COX17	PPP1R14B-AS1	0.323487572	1.21E-13	postive
COX17	PRELID1P1	0.479851475	3.71E-30	postive
COX17	PRKAG2-AS1	0.357456058	1.62E-16	postive
COX17	PRKAR2A-AS1	0.38738584	2.38E-19	postive
COX17	PRORSD1P	0.3623421	5.86E-17	postive
COX17	PRR34-AS1	0.443821951	1.51E-25	postive
COX17	PRRT3-AS1	0.318300432	3.10E-13	postive
COX17	PSMB8-AS1	0.405258001	3.47E-21	postive
COX17	PTOV1-AS1	0.376909912	2.53E-18	postive
COX17	PXN-AS1	0.582766773	8.22E-47	postive
COX17	PYCARD-AS1	0.342638426	3.21E-15	postive

COX17	RAB11B-AS1	0.46837945	1.25E-28	postive
COX17	RAB30-DT	0.456041659	4.75E-27	postive
COX17	RABGAP1L-DT	0.330698415	3.18E-14	postive
COX17	RAET1E-AS1	0.328394801	4.89E-14	postive
COX17	RALY-AS1	0.426662997	1.55E-23	postive
COX17	RARA-AS1	0.459298616	1.85E-27	postive
COX17	RASA4CP	0.370873424	9.48E-18	postive
COX17	RASAL2-AS1	0.316152654	4.55E-13	postive
COX17	RASSF1-AS1	0.359418907	1.08E-16	postive
COX17	RBM15-AS1	0.592000331	1.31E-48	postive
COX17	RBM26-AS1	0.321613365	1.70E-13	postive
COX17	RDM1P5	0.357492342	1.61E-16	postive
COX17	RHPN1-AS1	0.306948742	2.28E-12	postive
COX17	RNASEH1-AS1	0.547460078	1.93E-40	postive
COX17	RNF216P1	0.429555172	7.22E-24	postive
COX17	RP11-101E13.5	0.363865475	4.25E-17	postive
COX17	RP11-10A14.4	0.382413719	7.38E-19	postive
COX17	RP11-10C24.1	0.350131184	7.25E-16	postive
COX17	RP11-115J16.1	0.338186588	7.64E-15	postive
COX17	RP11-160O5.1	0.368742324	1.50E-17	postive
COX17	RP11-199F11.2	0.341743048	3.83E-15	postive
COX17	RP11-254F7.4	0.408173668	1.70E-21	postive
COX17	RP11-314C16.1	0.30725711	2.16E-12	postive
COX17	RP11-435O5.2	0.490903311	1.10E-31	postive
COX17	RP11-465N4.5	0.305185407	3.08E-12	postive
COX17	RP11-541P9.3	0.309497038	1.47E-12	postive
COX17	RP11-61L19.3	0.313148878	7.74E-13	postive
COX17	RP11-98G7.1	0.412220385	6.22E-22	postive
COX17	RP13-476E20.1	0.312154217	9.22E-13	postive
COX17	RP4-545L17.11	0.565021213	1.63E-43	postive
COX17	RP9P	0.441330496	3.01E-25	postive
COX17	RPARP-AS1	0.337895427	8.08E-15	postive
COX17	RPL12P25	0.306258093	2.56E-12	postive
COX17	RPL13P5	0.302284365	5.04E-12	postive
COX17	RPL23AP7	0.440770461	3.51E-25	postive
COX17	RPL23AP82	0.480760215	2.79E-30	postive
COX17	RPL37P6	0.343988209	2.46E-15	postive
COX17	RPLP1P6	0.461097395	1.09E-27	postive
COX17	RPS10P7	0.311775698	9.85E-13	postive
COX17	RPS18P9	0.372659605	6.43E-18	postive
COX17	RPS27P25	0.313838235	6.85E-13	postive
COX17	RPSAP69	0.389974117	1.31E-19	postive
COX17	RRN3P3	0.322569238	1.43E-13	postive
COX17	SAMD12-AS1	0.365790763	2.82E-17	postive
COX17	SAP30-DT	0.332999252	2.06E-14	postive
COX17	SBDSP1	0.408880609	1.43E-21	postive
COX17	SBF2-AS1	0.488859965	2.13E-31	postive
COX17	SBNO1-AS1	0.395269317	3.81E-20	postive
COX17	SCAMP1-AS1	0.522126319	2.58E-36	postive
COX17	SCAT1	0.332840906	2.12E-14	postive

COX17	SDHAP1	0.374680222	4.13E-18	postive
COX17	SEC24B-AS1	0.314713346	5.87E-13	postive
COX17	SEPSECS-AS1	0.365425738	3.05E-17	postive
COX17	SEPTIN4-AS1	0.424300983	2.86E-23	postive
COX17	SLC25A25-AS1	0.348958933	9.17E-16	postive
COX17	SLC25A5-AS1	0.362000664	6.30E-17	postive
COX17	SLC2A1-AS1	0.416146531	2.32E-22	postive
COX17	SNAI3-AS1	0.340702467	4.69E-15	postive
COX17	SNHG1	0.308318001	1.80E-12	postive
COX17	SNHG10	0.440843373	3.44E-25	postive
COX17	SNHG11	0.532971588	4.88E-38	postive
COX17	SNHG15	0.347894208	1.14E-15	postive
COX17	SNHG16	0.300297438	7.04E-12	postive
COX17	SNHG17	0.474653166	1.86E-29	postive
COX17	SNHG21	0.416349217	2.20E-22	postive
COX17	SNHG28	0.35724381	1.70E-16	postive
COX17	SNHG29	0.377691535	2.12E-18	postive
COX17	SNHG30	0.556198107	6.03E-42	postive
COX17	SNHG32	0.38633352	3.03E-19	postive
COX17	SNHG5	0.366752671	2.30E-17	postive
COX17	SNHG6	0.457259014	3.34E-27	postive
COX17	SNHG7	0.390505802	1.16E-19	postive
COX17	SNHG8	0.379690068	1.36E-18	postive
COX17	SNHG9	0.412285326	6.12E-22	postive
COX17	SP2-AS1	0.315443062	5.16E-13	postive
COX17	SPATA41	0.375924672	3.14E-18	postive
COX17	SPINT1-AS1	0.478641174	5.41E-30	postive
COX17	SREBF2-AS1	0.304235615	3.62E-12	postive
COX17	SRP14-AS1	0.383306611	6.03E-19	postive
COX17	ST20-AS1	0.371534568	8.21E-18	postive
COX17	ST3GAL6-AS1	0.331748284	2.61E-14	postive
COX17	STAG3L2	0.305157931	3.09E-12	postive
COX17	STAG3L4	0.480744129	2.80E-30	postive
COX17	STAG3L5P-PVRIG2P-PILRB	0.300248116	7.09E-12	postive
COX17	STARD7-AS1	0.492568482	6.41E-32	postive
COX17	STAU2-AS1	0.318310253	3.09E-13	postive
COX17	STK24-AS1	0.383006376	6.46E-19	postive
COX17	STK4-AS1	0.539746974	3.80E-39	postive
COX17	STX17-AS1	0.410549088	9.43E-22	postive
COX17	STX18-AS1	0.327494653	5.79E-14	postive
COX17	SUCLA2-AS1	0.37906725	1.56E-18	postive
COX17	SUMO2P17	0.372390624	6.82E-18	postive
COX17	SVIL-AS1	0.439460854	5.03E-25	postive
COX17	SYS1-DBNDD2	0.315953484	4.71E-13	postive
COX17	TAF1A-AS1	0.514626679	3.70E-35	postive
COX17	TDRKH-AS1	0.493870616	4.19E-32	postive
COX17	TEKT4P2	0.331183973	2.90E-14	postive
COX17	TFAP2A-AS1	0.325623725	8.18E-14	postive
COX17	THAP7-AS1	0.471354791	5.09E-29	postive
COX17	THAP9-AS1	0.345380998	1.87E-15	postive

COX17	TIPARP-AS1	0.46440361	4.10E-28	postive
COX17	TM4SF19-AS1	0.388939076	1.67E-19	postive
COX17	TMC3-AS1	0.418675007	1.22E-22	postive
COX17	TMCC1-AS1	0.405469869	3.29E-21	postive
COX17	TMCO1-AS1	0.301341399	5.90E-12	postive
COX17	TMED2-DT	0.342285311	3.44E-15	postive
COX17	TMEM161B-AS1	0.315021876	5.56E-13	postive
COX17	TMEM202-AS1	0.468097767	1.36E-28	postive
COX17	TMEM44-AS1	0.623960977	2.65E-55	postive
COX17	TMEM99	0.420778737	7.12E-23	postive
COX17	TMPO-AS1	0.526877184	4.62E-37	postive
COX17	TOB1-AS1	0.501472152	3.37E-33	postive
COX17	TP53TG1	0.470412257	6.77E-29	postive
COX17	TPI1P2	0.351048385	6.02E-16	postive
COX17	TPM3P9	0.311683759	1.00E-12	postive
COX17	TPRG1-AS1	0.42220133	4.94E-23	postive
COX17	TRAM2-AS1	0.543084758	1.06E-39	postive
COX17	TRIM52-AS1	0.526875061	4.63E-37	postive
COX17	TSTD3	0.432855932	3.00E-24	postive
COX17	TTC28-AS1	0.392538219	7.22E-20	postive
COX17	TTC39A-AS1	0.371565973	8.16E-18	postive
COX17	TTC41P	0.337128577	9.37E-15	postive
COX17	TTTY20	0.319858479	2.34E-13	postive
COX17	TUBBP9	0.353295653	3.82E-16	postive
COX17	TUG1	0.329440117	4.03E-14	postive
COX17	TULP3P1	0.305387835	2.97E-12	postive
COX17	TYMSOS	0.475886923	1.27E-29	postive
COX17	U47924.2	0.455888879	4.97E-27	postive
COX17	U62317.1	0.327143704	6.17E-14	postive
COX17	U91328.1	0.399506725	1.39E-20	postive
COX17	UBA6-AS1	0.508339182	3.28E-34	postive
COX17	UBAC2-AS1	0.432857276	3.00E-24	postive
COX17	UBE2D3-AS1	0.521888643	2.81E-36	postive
COX17	UBL7-AS1	0.455402597	5.72E-27	postive
COX17	UBR5-AS1	0.344468458	2.24E-15	postive
COX17	UBXN10-AS1	0.303914685	3.82E-12	postive
COX17	UPF3AP2	0.326654951	6.76E-14	postive
COX17	URB1-AS1	0.519584278	6.42E-36	postive
COX17	USP27X-AS1	0.446640754	6.89E-26	postive
COX17	USP46-AS1	0.426788732	1.50E-23	postive
COX17	UVRAG-DT	0.319620597	2.44E-13	postive
COX17	UXT-AS1	0.48989846	1.52E-31	postive
COX17	VPS13B-DT	0.445769004	8.79E-26	postive
COX17	VPS33B-DT	0.435435836	1.50E-24	postive
COX17	WAC-AS1	0.428746432	8.94E-24	postive
COX17	WASH3P	0.438956213	5.77E-25	postive
COX17	WEE2-AS1	0.346045842	1.64E-15	postive
COX17	WWTR1-AS1	0.531614537	8.09E-38	postive
COX17	YTHDF3-AS1	0.453025412	1.13E-26	postive
COX17	Z73429.1	0.351007858	6.07E-16	postive

COX17	Z82185.1	0.324372894	1.03E-13	postive
COX17	Z82246.1	0.306816394	2.33E-12	postive
COX17	Z83851.4	0.342576426	3.25E-15	postive
COX17	Z92544.1	0.390363046	1.20E-19	postive
COX17	Z95115.1	0.407508773	2.00E-21	postive
COX17	Z97634.1	0.416781111	1.98E-22	postive
COX17	Z99572.1	0.370159958	1.11E-17	postive
COX17	ZBED3-AS1	0.322088077	1.56E-13	postive
COX17	ZBED5-AS1	0.399889657	1.27E-20	postive
COX17	ZBTB11-AS1	0.436724567	1.06E-24	postive
COX17	ZFAS1	0.374019622	4.77E-18	postive
COX17	ZFPM2-AS1	0.366624529	2.36E-17	postive
COX17	ZNF213-AS1	0.306162641	2.60E-12	postive
COX17	ZNF236-DT	0.318385425	3.05E-13	postive
COX17	ZNF252P	0.386521457	2.90E-19	postive
COX17	ZNF337-AS1	0.3046521	3.37E-12	postive
COX17	ZNF341-AS1	0.300575762	6.71E-12	postive
COX17	ZNF426-DT	0.36134681	7.22E-17	postive
COX17	ZNF503-AS2	0.446004472	8.23E-26	postive
COX17	ZNF529-AS1	0.432415177	3.38E-24	postive
COX17	ZNF561-AS1	0.567604708	5.57E-44	postive
COX17	ZNF674-AS1	0.325858723	7.83E-14	postive
COX17	ZNF687-AS1	0.567599095	5.58E-44	postive
COX17	ZNRD2-AS1	0.382015262	8.08E-19	postive
COX17	ZSCAN16-AS1	0.600981491	2.05E-50	postive
CP	AC002310.2	0.312982287	7.97E-13	postive
CP	AC003965.2	0.340584665	4.80E-15	postive
CP	AC004241.3	0.315008778	5.57E-13	postive
CP	AC004862.1	0.545472731	4.20E-40	postive
CP	AC005229.4	0.483441214	1.20E-30	postive
CP	AC005280.1	0.438582861	6.39E-25	postive
CP	AC006372.1	0.328443804	4.85E-14	postive
CP	AC006372.3	0.307942662	1.92E-12	postive
CP	AC007100.1	0.527713578	3.40E-37	postive
CP	AC007298.2	0.477370661	8.03E-30	postive
CP	AC007342.4	0.319651294	2.43E-13	postive
CP	AC007365.1	0.387621461	2.26E-19	postive
CP	AC008267.5	0.454592863	7.22E-27	postive
CP	AC009171.2	0.407036175	2.25E-21	postive
CP	AC009403.1	0.393922574	5.22E-20	postive
CP	AC009407.1	0.301019225	6.23E-12	postive
CP	AC009779.3	0.417822412	1.52E-22	postive
CP	AC010168.1	0.310748336	1.18E-12	postive
CP	AC010203.2	0.34242241	3.35E-15	postive
CP	AC010643.1	0.404398518	4.28E-21	postive
CP	AC010737.1	0.315539233	5.07E-13	postive
CP	AC010894.2	0.339207226	6.27E-15	postive
CP	AC010907.2	0.306135859	2.62E-12	postive
CP	AC012146.1	0.433521358	2.51E-24	postive
CP	AC012467.2	0.322465465	1.46E-13	postive

CP	AC012676.1	0.434317968	2.03E-24	postive
CP	AC015917.2	0.559585641	1.53E-42	postive
CP	AC016044.1	0.538728771	5.60E-39	postive
CP	AC016550.2	0.32418014	1.07E-13	postive
CP	AC016747.1	0.317262257	3.73E-13	postive
CP	AC016888.1	0.380312505	1.18E-18	postive
CP	AC018653.3	0.356458107	2.00E-16	postive
CP	AC019185.1	0.503001046	2.02E-33	postive
CP	AC021074.3	0.551943164	3.31E-41	postive
CP	AC022098.3	0.302644675	4.74E-12	postive
CP	AC022167.4	0.348992981	9.11E-16	postive
CP	AC022217.4	0.320480186	2.09E-13	postive
CP	AC022816.1	0.559317186	1.70E-42	postive
CP	AC024361.2	0.311210488	1.09E-12	postive
CP	AC025254.1	0.495054037	2.84E-32	postive
CP	AC026462.3	0.504454295	1.23E-33	postive
CP	AC026471.1	0.306748425	2.35E-12	postive
CP	AC027237.4	0.339851499	5.54E-15	postive
CP	AC061961.2	0.486138299	5.09E-31	postive
CP	AC061975.6	0.321895856	1.62E-13	postive
CP	AC067930.3	0.51127116	1.19E-34	postive
CP	AC068506.1	0.437787786	7.93E-25	postive
CP	AC073107.1	0.347888679	1.14E-15	postive
CP	AC073365.1	0.404607428	4.06E-21	postive
CP	AC073842.1	0.458837903	2.11E-27	postive
CP	AC074237.1	0.334013456	1.70E-14	postive
CP	AC079807.1	0.307264084	2.16E-12	postive
CP	AC079921.2	0.534555852	2.70E-38	postive
CP	AC084036.1	0.445524195	9.42E-26	postive
CP	AC084125.4	0.408019233	1.76E-21	postive
CP	AC087241.2	0.390850918	1.07E-19	postive
CP	AC087482.1	0.404271645	4.41E-21	postive
CP	AC090587.1	0.397527125	2.23E-20	postive
CP	AC090912.1	0.331802166	2.58E-14	postive
CP	AC091057.1	0.362462361	5.72E-17	postive
CP	AC092368.3	0.311106444	1.11E-12	postive
CP	AC092490.1	0.303585951	4.04E-12	postive
CP	AC092925.1	0.565313174	1.45E-43	postive
CP	AC093484.2	0.346813325	1.41E-15	postive
CP	AC093582.1	0.408602166	1.53E-21	postive
CP	AC096536.2	0.403896353	4.83E-21	postive
CP	AC096649.2	0.341283417	4.19E-15	postive
CP	AC097501.1	0.355353258	2.51E-16	postive
CP	AC097504.2	0.358188266	1.40E-16	postive
CP	AC097512.1	0.353002148	4.05E-16	postive
CP	AC099552.1	0.567380518	6.11E-44	postive
CP	AC099552.3	0.576904208	1.06E-45	postive
CP	AC103746.1	0.396602521	2.78E-20	postive
CP	AC103760.1	0.323013466	1.32E-13	postive
CP	AC104051.2	0.560701476	9.69E-43	postive

CP	AC104561.3	0.325908622	7.76E-14	postive
CP	AC105105.2	0.543416815	9.30E-40	postive
CP	AC107021.2	0.38181352	8.45E-19	postive
CP	AC108517.1	0.621206684	1.07E-54	postive
CP	AC111149.2	0.37161503	8.07E-18	postive
CP	AC114485.1	0.563952154	2.54E-43	postive
CP	AC115618.1	0.546485269	2.83E-40	postive
CP	AC115618.2	0.330564072	3.26E-14	postive
CP	AC115619.1	0.569775383	2.23E-44	postive
CP	AC116036.2	0.337429894	8.84E-15	postive
CP	AC116348.5	0.345303898	1.90E-15	postive
CP	AC117386.2	0.34631913	1.55E-15	postive
CP	AC117394.2	0.314874582	5.71E-13	postive
CP	AC120114.1	0.30800837	1.90E-12	postive
CP	AC121761.1	0.323392122	1.23E-13	postive
CP	AC122108.3	0.389194427	1.57E-19	postive
CP	AC124947.2	0.310280984	1.28E-12	postive
CP	AC126323.1	0.351935435	5.03E-16	postive
CP	AC126323.3	0.509777539	2.00E-34	postive
CP	AC131009.1	0.410571186	9.38E-22	postive
CP	AC131009.2	0.385185051	3.94E-19	postive
CP	AC131956.2	0.317516825	3.57E-13	postive
CP	AC132938.4	0.390749335	1.10E-19	postive
CP	AC138646.1	0.36512206	3.26E-17	postive
CP	AC145207.1	0.354118666	3.23E-16	postive
CP	AC246785.3	0.397545812	2.22E-20	postive
CP	AC254633.1	0.355985074	2.20E-16	postive
CP	ADCY6-DT	0.432211363	3.56E-24	postive
CP	AF196972.1	0.349838213	7.69E-16	postive
CP	AL021154.1	0.32284823	1.36E-13	postive
CP	AL024507.3	0.433247639	2.70E-24	postive
CP	AL031727.2	0.306398614	2.50E-12	postive
CP	AL049543.1	0.309851454	1.38E-12	postive
CP	AL109917.1	0.347598371	1.20E-15	postive
CP	AL117329.1	0.305194763	3.07E-12	postive
CP	AL132639.2	0.338244808	7.56E-15	postive
CP	AL138826.1	0.576813024	1.11E-45	postive
CP	AL139035.1	0.300083572	7.29E-12	postive
CP	AL139039.3	0.343980375	2.47E-15	postive
CP	AL157896.1	0.612637274	7.63E-53	postive
CP	AL158071.4	0.304609707	3.40E-12	postive
CP	AL162411.1	0.425637006	2.02E-23	postive
CP	AL353708.1	0.332041513	2.47E-14	postive
CP	AL353764.1	0.379687048	1.36E-18	postive
CP	AL355096.1	0.330660065	3.20E-14	postive
CP	AL355102.4	0.513772632	4.99E-35	postive
CP	AL355482.1	0.318312202	3.09E-13	postive
CP	AL355574.1	0.308057814	1.88E-12	postive
CP	AL356215.1	0.306964243	2.27E-12	postive
CP	AL356652.1	0.419835906	9.07E-23	postive

CP	AL391095.3	0.568969477	3.14E-44	postive
CP	AL445231.1	0.352065254	4.90E-16	postive
CP	AL512408.1	0.354020661	3.29E-16	postive
CP	AL513327.2	0.315098416	5.48E-13	postive
CP	AL513550.1	0.365294204	3.14E-17	postive
CP	AL592494.1	0.556600214	5.13E-42	postive
CP	AL596211.1	0.373047514	5.91E-18	postive
CP	AL606760.1	0.361806529	6.56E-17	postive
CP	AL928654.1	0.32146269	1.75E-13	postive
CP	AP000781.1	0.300535912	6.76E-12	postive
CP	AP001318.2	0.301076141	6.17E-12	postive
CP	AP001372.2	0.334416509	1.57E-14	postive
CP	AP001830.1	0.312592451	8.53E-13	postive
CP	AP001830.2	0.367246457	2.07E-17	postive
CP	AP002360.2	0.31449672	6.10E-13	postive
CP	AP002387.2	0.335391944	1.31E-14	postive
CP	AP003680.1	0.328188781	5.09E-14	postive
CP	AP005262.2	0.309512839	1.46E-12	postive
CP	AP005264.4	0.510665705	1.47E-34	postive
CP	AP005264.5	0.433744897	2.36E-24	postive
CP	AP005329.2	0.518736634	8.68E-36	postive
CP	APOC1P1	0.467839906	1.47E-28	postive
CP	ARAP1-AS1	0.384842632	4.26E-19	postive
CP	BCAR3-AS1	0.429170021	7.99E-24	postive
CP	BX119904.2	0.306032806	2.66E-12	postive
CP	C22orf46	0.309342309	1.50E-12	postive
CP	C3P1	0.473155702	2.94E-29	postive
CP	C5orf34-AS1	0.492323932	6.94E-32	postive
CP	CATSPER2P1	0.309629647	1.43E-12	postive
CP	CERNA1	0.314633045	5.96E-13	postive
CP	CSE1L-AS1	0.474695892	1.83E-29	postive
CP	CTBP1-DT	0.337162459	9.31E-15	postive
CP	CTD-2521M24.9	0.322338915	1.49E-13	postive
CP	DHCR24-DT	0.337058389	9.50E-15	postive
CP	DHRSX-IT1	0.333526789	1.86E-14	postive
CP	ELF3-AS1	0.363780152	4.33E-17	postive
CP	ELOA-AS1	0.331445986	2.76E-14	postive
CP	ELOVL2-AS1	0.474254337	2.10E-29	postive
CP	ESRRAP2	0.322020981	1.58E-13	postive
CP	F11-AS1	0.305264354	3.04E-12	postive
CP	FAM111A-DT	0.324494991	1.01E-13	postive
CP	FAM86EP	0.322780188	1.38E-13	postive
CP	FMR1-AS1	0.396815962	2.64E-20	postive
CP	GCC2-AS1	0.334839191	1.45E-14	postive
CP	GOLGA2P11	0.321775795	1.65E-13	postive
CP	GS1-358P8.4	0.359074978	1.16E-16	postive
CP	HCG15	0.310610175	1.21E-12	postive
CP	HERC2P3	0.34118111	4.27E-15	postive
CP	HOXB-AS1	0.338305162	7.47E-15	postive
CP	IDH1-AS1	0.406690276	2.44E-21	postive

CP	KCNIP2-AS1	0.322865899	1.36E-13	postive
CP	KDM7A-DT	0.333840446	1.76E-14	postive
CP	LGR4-AS1	0.488202362	2.63E-31	postive
CP	LINC00167	0.301422312	5.82E-12	postive
CP	LINC00189	0.359635843	1.03E-16	postive
CP	LINC00471	0.301859674	5.41E-12	postive
CP	LINC00533	0.551311157	4.25E-41	postive
CP	LINC00605	0.411064043	8.30E-22	postive
CP	LINC00884	0.359541655	1.05E-16	postive
CP	LINC00898	0.331447737	2.76E-14	postive
CP	LINC00963	0.343396105	2.77E-15	postive
CP	LINC00992	0.487863379	2.93E-31	postive
CP	LINC01136	0.336505766	1.06E-14	postive
CP	LINC01234	0.380151632	1.23E-18	postive
CP	LINC01273	0.392534332	7.23E-20	postive
CP	LINC01278	0.324389501	1.03E-13	postive
CP	LINC01347	0.328320971	4.96E-14	postive
CP	LINC01399	0.355815204	2.28E-16	postive
CP	LINC01465	0.347899051	1.13E-15	postive
CP	LINC01545	0.352260005	4.71E-16	postive
CP	LINC01560	0.414798394	3.26E-22	postive
CP	LINC01588	0.32189887	1.62E-13	postive
CP	LINC01608	0.310380268	1.26E-12	postive
CP	LINC01632	0.550193799	6.61E-41	postive
CP	LINC01647	0.444373589	1.30E-25	postive
CP	LINC01687	0.355220144	2.57E-16	postive
CP	LINC01816	0.581990769	1.16E-46	postive
CP	LINC01827	0.366128522	2.63E-17	postive
CP	LINC01887	0.309853889	1.38E-12	postive
CP	LINC02032	0.363250929	4.84E-17	postive
CP	LINC02136	0.33866917	6.96E-15	postive
CP	LINC02197	0.424937387	2.43E-23	postive
CP	LINC02300	0.343641099	2.64E-15	postive
CP	LINC02370	0.40288103	6.18E-21	postive
CP	LINC02429	0.362676527	5.46E-17	postive
CP	LINC02432	0.407090014	2.22E-21	postive
CP	LINC02453	0.371110562	9.00E-18	postive
CP	LINC02525	0.333863399	1.75E-14	postive
CP	LINC02701	0.509298234	2.36E-34	postive
CP	LINC02799	0.356576686	1.95E-16	postive
CP	LNCSRLR	0.334079615	1.68E-14	postive
CP	MATN1-AS1	0.301012155	6.24E-12	postive
CP	MELTF-AS1	0.30522569	3.06E-12	postive
CP	MIR122HG	0.566496016	8.85E-44	postive
CP	MIR210HG	0.378959228	1.60E-18	postive
CP	MSH5-SAPCD1	0.331852878	2.56E-14	postive
CP	NRAV	0.329499216	3.98E-14	postive
CP	OGFRP1	0.416092777	2.35E-22	postive
CP	OLMALINC	0.338637465	7.01E-15	postive
CP	OR8G3P	0.446454122	7.26E-26	postive

CP	PAQR9-AS1	0.565049436	1.62E-43	postive
CP	PCAT5	0.322927712	1.34E-13	postive
CP	PDCD4-AS1	0.406673917	2.45E-21	postive
CP	PKP4-AS1	0.398387777	1.82E-20	postive
CP	PLEKHA8P1	0.304603544	3.40E-12	postive
CP	PLGLA	0.356586108	1.94E-16	postive
CP	PPY2P	0.313531946	7.23E-13	postive
CP	PRKAR2A-AS1	0.384208115	4.92E-19	postive
CP	PROSER2-AS1	0.377830654	2.06E-18	postive
CP	PXN-AS1	0.354646831	2.90E-16	postive
CP	RAB30-DT	0.396013692	3.19E-20	postive
CP	RHPN1-AS1	0.31168602	1.00E-12	postive
CP	RP11-392O1.4	0.325397298	8.53E-14	postive
CP	RP11-552M14.1	0.333704689	1.80E-14	postive
CP	RP3-323A16.1	0.349993387	7.45E-16	postive
CP	SAPCD1-AS1	0.493782859	4.31E-32	postive
CP	SELENOOLP	0.351009318	6.07E-16	postive
CP	SLC12A5-AS1	0.303648862	4.00E-12	postive
CP	SLC12A9-AS1	0.317228561	3.75E-13	postive
CP	SLC5A4-AS1	0.301221436	6.02E-12	postive
CP	SLC9A7P1	0.323927552	1.12E-13	postive
CP	SNHG21	0.322466896	1.46E-13	postive
CP	TAAR3P	0.32441663	1.02E-13	postive
CP	THRB-AS1	0.50600908	7.27E-34	postive
CP	TMC3-AS1	0.371879228	7.62E-18	postive
CP	TMPO-AS1	0.365191571	3.21E-17	postive
CP	TOB1-AS1	0.342385511	3.38E-15	postive
CP	TPM3P9	0.338540686	7.14E-15	postive
CP	TYMSOS	0.335553136	1.27E-14	postive
CP	U91324.1	0.339852932	5.54E-15	postive
CP	UBA6-AS1	0.345153254	1.96E-15	postive
CP	UPK1A-AS1	0.560785204	9.36E-43	postive
CP	USP27X-AS1	0.347953703	1.12E-15	postive
CP	UXT-AS1	0.490431742	1.28E-31	postive
CP	VPS9D1-AS1	0.306653691	2.39E-12	postive
CP	YWHAEP7	0.307488088	2.07E-12	postive
CP	Z83851.4	0.427304915	1.31E-23	postive
CP	ZNF687-AS1	0.418441113	1.30E-22	postive
CUTC	AC002116.2	0.321208834	1.83E-13	postive
CUTC	AC002310.1	0.362592931	5.56E-17	postive
CUTC	AC002467.1	0.455947966	4.88E-27	postive
CUTC	AC004241.1	0.492198677	7.23E-32	postive
CUTC	AC004241.2	0.374751109	4.07E-18	postive
CUTC	AC004241.3	0.361818646	6.54E-17	postive
CUTC	AC004477.3	0.317832026	3.37E-13	postive
CUTC	AC004596.1	0.363354988	4.73E-17	postive
CUTC	AC004771.2	0.371290598	8.66E-18	postive
CUTC	AC004801.6	0.388736013	1.75E-19	postive
CUTC	AC004803.1	0.434215502	2.08E-24	postive
CUTC	AC004812.2	0.393468811	5.81E-20	postive

CUTC	AC004893.11	0.303058449	4.42E-12	postive
CUTC	AC004918.1	0.308382919	1.78E-12	postive
CUTC	AC004943.2	0.344371734	2.29E-15	postive
CUTC	AC005034.4	0.408815527	1.45E-21	postive
CUTC	AC005034.6	0.328546459	4.76E-14	postive
CUTC	AC005037.1	0.304903661	3.23E-12	postive
CUTC	AC005076.2	0.350493804	6.73E-16	postive
CUTC	AC005229.4	0.325665625	8.12E-14	postive
CUTC	AC005288.1	0.358568685	1.29E-16	postive
CUTC	AC005324.5	0.352934224	4.11E-16	postive
CUTC	AC005520.4	0.361223652	7.41E-17	postive
CUTC	AC005562.1	0.414134776	3.85E-22	postive
CUTC	AC005586.1	0.371920032	7.55E-18	postive
CUTC	AC005670.3	0.411977862	6.61E-22	postive
CUTC	AC005696.1	0.348505031	1.00E-15	postive
CUTC	AC005837.1	0.308956746	1.61E-12	postive
CUTC	AC006001.2	0.331388061	2.79E-14	postive
CUTC	AC006064.3	0.323754339	1.15E-13	postive
CUTC	AC006206.2	0.314380356	6.23E-13	postive
CUTC	AC006213.1	0.350913571	6.19E-16	postive
CUTC	AC006252.1	0.386291532	3.06E-19	postive
CUTC	AC006333.1	0.382693902	6.93E-19	postive
CUTC	AC006449.2	0.478535056	5.59E-30	postive
CUTC	AC006449.7	0.477537984	7.62E-30	postive
CUTC	AC006504.5	0.327685071	5.58E-14	postive
CUTC	AC007114.1	0.323531476	1.20E-13	postive
CUTC	AC007228.9	0.304300741	3.58E-12	postive
CUTC	AC007342.4	0.382934879	6.56E-19	postive
CUTC	AC007383.2	0.473391458	2.73E-29	postive
CUTC	AC007406.5	0.351901928	5.07E-16	postive
CUTC	AC007541.1	0.306915097	2.29E-12	postive
CUTC	AC007773.1	0.398742628	1.67E-20	postive
CUTC	AC007952.2	0.318993375	2.74E-13	postive
CUTC	AC007998.3	0.343377975	2.78E-15	postive
CUTC	AC008014.1	0.483473964	1.19E-30	postive
CUTC	AC008035.1	0.394693269	4.36E-20	postive
CUTC	AC008105.2	0.307978765	1.91E-12	postive
CUTC	AC008124.1	0.363396538	4.69E-17	postive
CUTC	AC008267.5	0.302698217	4.70E-12	postive
CUTC	AC008280.3	0.34455228	2.21E-15	postive
CUTC	AC008393.1	0.300055195	7.33E-12	postive
CUTC	AC008438.1	0.440957309	3.34E-25	postive
CUTC	AC008443.1	0.493037746	5.50E-32	postive
CUTC	AC008610.1	0.352436544	4.54E-16	postive
CUTC	AC008758.4	0.332863845	2.11E-14	postive
CUTC	AC008771.1	0.401926376	7.78E-21	postive
CUTC	AC008915.3	0.456928668	3.68E-27	postive
CUTC	AC008946.1	0.324949993	9.26E-14	postive
CUTC	AC008966.1	0.422183975	4.96E-23	postive
CUTC	AC009061.2	0.414605475	3.42E-22	postive

CUTC	AC009065.9	0.359661252	1.03E-16	postive
CUTC	AC009113.1	0.413323808	4.72E-22	postive
CUTC	AC009118.3	0.334916898	1.43E-14	postive
CUTC	AC009126.1	0.357553739	1.59E-16	postive
CUTC	AC009226.1	0.370739574	9.76E-18	postive
CUTC	AC009237.14	0.314777824	5.80E-13	postive
CUTC	AC009309.2	0.342683233	3.19E-15	postive
CUTC	AC009403.1	0.43977746	4.61E-25	postive
CUTC	AC009506.2	0.386776486	2.74E-19	postive
CUTC	AC009690.2	0.345148299	1.96E-15	postive
CUTC	AC009779.3	0.467028903	1.88E-28	postive
CUTC	AC009831.1	0.329675886	3.85E-14	postive
CUTC	AC009948.3	0.305023262	3.16E-12	postive
CUTC	AC009961.1	0.302703309	4.69E-12	postive
CUTC	AC010226.1	0.306062738	2.65E-12	postive
CUTC	AC010273.3	0.356635113	1.92E-16	postive
CUTC	AC010504.1	0.351109332	5.95E-16	postive
CUTC	AC010595.1	0.31200621	9.46E-13	postive
CUTC	AC010761.1	0.327951272	5.32E-14	postive
CUTC	AC010789.1	0.305031566	3.16E-12	postive
CUTC	AC010809.1	0.348001337	1.11E-15	postive
CUTC	AC010864.1	0.356879091	1.83E-16	postive
CUTC	AC010969.2	0.364337431	3.85E-17	postive
CUTC	AC010997.4	0.347050586	1.34E-15	postive
CUTC	AC011731.1	0.422542142	4.52E-23	postive
CUTC	AC011978.2	0.366766305	2.29E-17	postive
CUTC	AC012063.1	0.418053476	1.43E-22	postive
CUTC	AC012073.1	0.305996626	2.68E-12	postive
CUTC	AC012146.1	0.405568897	3.22E-21	postive
CUTC	AC012184.3	0.313849758	6.84E-13	postive
CUTC	AC012306.2	0.364880476	3.43E-17	postive
CUTC	AC012313.1	0.332356157	2.33E-14	postive
CUTC	AC012360.3	0.32604638	7.56E-14	postive
CUTC	AC012442.2	0.330846462	3.09E-14	postive
CUTC	AC012442.3	0.363706427	4.40E-17	postive
CUTC	AC012467.2	0.375653027	3.33E-18	postive
CUTC	AC012510.1	0.340631871	4.76E-15	postive
CUTC	AC012555.1	0.303164432	4.34E-12	postive
CUTC	AC012603.1	0.333347024	1.93E-14	postive
CUTC	AC012640.4	0.316754144	4.09E-13	postive
CUTC	AC012676.1	0.369571164	1.26E-17	postive
CUTC	AC015802.4	0.323065204	1.31E-13	postive
CUTC	AC015922.2	0.370744676	9.75E-18	postive
CUTC	AC015922.3	0.39791476	2.03E-20	postive
CUTC	AC015982.2	0.390604423	1.13E-19	postive
CUTC	AC016065.1	0.401163286	9.35E-21	postive
CUTC	AC016355.1	0.306212647	2.58E-12	postive
CUTC	AC016394.2	0.521736669	2.97E-36	postive
CUTC	AC016394.3	0.326709648	6.69E-14	postive
CUTC	AC016727.1	0.416043194	2.38E-22	postive

CUTC	AC016747.1	0.387798381	2.17E-19	postive
CUTC	AC016866.1	0.315829106	4.82E-13	postive
CUTC	AC016876.2	0.426485453	1.62E-23	postive
CUTC	AC016876.3	0.461732876	9.04E-28	postive
CUTC	AC018413.1	0.332374124	2.32E-14	postive
CUTC	AC018521.5	0.618354442	4.50E-54	postive
CUTC	AC018521.6	0.353383964	3.75E-16	postive
CUTC	AC018647.2	0.347709905	1.18E-15	postive
CUTC	AC018690.1	0.334085938	1.68E-14	postive
CUTC	AC018809.2	0.363185414	4.91E-17	postive
CUTC	AC019131.2	0.366565444	2.39E-17	postive
CUTC	AC019294.2	0.31798843	3.28E-13	postive
CUTC	AC020558.2	0.328845786	4.50E-14	postive
CUTC	AC020661.1	0.335972767	1.17E-14	postive
CUTC	AC020663.2	0.317941271	3.30E-13	postive
CUTC	AC020915.3	0.39455718	4.50E-20	postive
CUTC	AC021016.2	0.321830204	1.64E-13	postive
CUTC	AC021028.1	0.422349624	4.75E-23	postive
CUTC	AC021148.1	0.34193975	3.69E-15	postive
CUTC	AC021224.1	0.353853698	3.41E-16	postive
CUTC	AC021752.1	0.316703625	4.12E-13	postive
CUTC	AC022098.1	0.333901298	1.74E-14	postive
CUTC	AC022211.3	0.335042309	1.40E-14	postive
CUTC	AC022217.4	0.310652031	1.20E-12	postive
CUTC	AC022306.2	0.357776241	1.52E-16	postive
CUTC	AC022400.1	0.348928917	9.23E-16	postive
CUTC	AC022400.5	0.348099503	1.09E-15	postive
CUTC	AC022400.6	0.317265605	3.73E-13	postive
CUTC	AC022613.2	0.358794337	1.23E-16	postive
CUTC	AC022916.2	0.316371174	4.37E-13	postive
CUTC	AC023509.3	0.423715911	3.33E-23	postive
CUTC	AC023632.2	0.301823446	5.44E-12	postive
CUTC	AC023790.2	0.320350846	2.14E-13	postive
CUTC	AC024575.2	0.388162934	1.99E-19	postive
CUTC	AC024896.1	0.366258992	2.56E-17	postive
CUTC	AC024941.2	0.302031745	5.26E-12	postive
CUTC	AC025048.4	0.333183751	1.99E-14	postive
CUTC	AC025262.1	0.313543563	7.22E-13	postive
CUTC	AC025265.1	0.337214379	9.22E-15	postive
CUTC	AC025682.2	0.36706606	2.15E-17	postive
CUTC	AC026367.2	0.309551343	1.45E-12	postive
CUTC	AC026367.3	0.376827873	2.57E-18	postive
CUTC	AC026412.1	0.300888535	6.37E-12	postive
CUTC	AC026471.1	0.32745792	5.83E-14	postive
CUTC	AC027097.1	0.421090721	6.57E-23	postive
CUTC	AC027097.2	0.317321547	3.69E-13	postive
CUTC	AC027601.1	0.367402616	2.00E-17	postive
CUTC	AC027644.3	0.304620058	3.39E-12	postive
CUTC	AC027763.2	0.332298412	2.35E-14	postive
CUTC	AC034236.2	0.314979421	5.60E-13	postive

CUTC	AC036176.1	0.372735426	6.32E-18	postive
CUTC	AC040970.1	0.35077761	6.36E-16	postive
CUTC	AC040977.1	0.396556365	2.81E-20	postive
CUTC	AC046134.2	0.469381544	9.24E-29	postive
CUTC	AC051619.4	0.363905307	4.21E-17	postive
CUTC	AC051619.6	0.332268961	2.37E-14	postive
CUTC	AC055720.2	0.354637366	2.90E-16	postive
CUTC	AC060780.1	0.346835024	1.40E-15	postive
CUTC	AC062028.1	0.325468882	8.42E-14	postive
CUTC	AC064807.1	0.443272992	1.76E-25	postive
CUTC	AC064807.4	0.350829973	6.29E-16	postive
CUTC	AC064836.3	0.318236087	3.13E-13	postive
CUTC	AC067750.1	0.388566134	1.82E-19	postive
CUTC	AC067838.1	0.339725792	5.67E-15	postive
CUTC	AC067852.2	0.523286099	1.70E-36	postive
CUTC	AC068338.2	0.42688262	1.46E-23	postive
CUTC	AC068473.5	0.376256366	2.92E-18	postive
CUTC	AC068790.8	0.309253189	1.53E-12	postive
CUTC	AC068831.2	0.352644804	4.36E-16	postive
CUTC	AC068831.3	0.318037888	3.25E-13	postive
CUTC	AC068870.2	0.33422509	1.63E-14	postive
CUTC	AC068888.1	0.412830442	5.34E-22	postive
CUTC	AC069185.1	0.344807383	2.10E-15	postive
CUTC	AC073254.1	0.371005797	9.21E-18	postive
CUTC	AC073508.3	0.391634028	8.92E-20	postive
CUTC	AC073569.2	0.336935506	9.73E-15	postive
CUTC	AC073575.4	0.343222836	2.87E-15	postive
CUTC	AC073585.1	0.334452111	1.56E-14	postive
CUTC	AC073611.1	0.409390988	1.26E-21	postive
CUTC	AC073896.2	0.433544434	2.50E-24	postive
CUTC	AC073896.3	0.304358855	3.54E-12	postive
CUTC	AC073896.4	0.307606519	2.03E-12	postive
CUTC	AC074117.1	0.392803447	6.79E-20	postive
CUTC	AC079145.1	0.315424167	5.18E-13	postive
CUTC	AC079414.3	0.309916204	1.36E-12	postive
CUTC	AC079807.1	0.334786096	1.47E-14	postive
CUTC	AC079848.1	0.319663622	2.42E-13	postive
CUTC	AC079907.1	0.304708992	3.34E-12	postive
CUTC	AC080112.2	0.412432885	5.90E-22	postive
CUTC	AC083798.2	0.349510919	8.21E-16	postive
CUTC	AC083799.1	0.367562564	1.93E-17	postive
CUTC	AC083843.1	0.335751355	1.22E-14	postive
CUTC	AC087741.2	0.313133055	7.76E-13	postive
CUTC	AC090198.1	0.34700125	1.36E-15	postive
CUTC	AC090204.1	0.469855663	8.01E-29	postive
CUTC	AC090587.1	0.3726467	6.45E-18	postive
CUTC	AC090617.5	0.385490974	3.67E-19	postive
CUTC	AC090912.1	0.331932119	2.52E-14	postive
CUTC	AC091057.1	0.320499165	2.08E-13	postive
CUTC	AC091153.3	0.354429796	3.03E-16	postive

CUTC	AC091153.4	0.300475329	6.83E-12	postive
CUTC	AC091271.1	0.340075375	5.30E-15	postive
CUTC	AC091390.5	0.460325218	1.37E-27	postive
CUTC	AC091729.3	0.370720508	9.80E-18	postive
CUTC	AC091887.1	0.329083452	4.30E-14	postive
CUTC	AC091965.1	0.339589242	5.83E-15	postive
CUTC	AC091982.3	0.309079691	1.58E-12	postive
CUTC	AC092115.4	0.472418505	3.68E-29	postive
CUTC	AC092118.1	0.331228032	2.88E-14	postive
CUTC	AC092118.2	0.304047134	3.74E-12	postive
CUTC	AC092123.1	0.409528342	1.21E-21	postive
CUTC	AC092134.1	0.306862985	2.31E-12	postive
CUTC	AC092164.1	0.330071491	3.58E-14	postive
CUTC	AC092295.2	0.315196768	5.39E-13	postive
CUTC	AC092368.3	0.325393144	8.53E-14	postive
CUTC	AC092614.1	0.342442396	3.34E-15	postive
CUTC	AC092969.1	0.302991283	4.47E-12	postive
CUTC	AC093157.1	0.386825701	2.71E-19	postive
CUTC	AC093484.4	0.358739827	1.24E-16	postive
CUTC	AC093503.1	0.311315443	1.07E-12	postive
CUTC	AC093523.2	0.365550536	2.97E-17	postive
CUTC	AC093627.5	0.305169598	3.09E-12	postive
CUTC	AC093752.1	0.377668593	2.13E-18	postive
CUTC	AC096536.2	0.324844939	9.44E-14	postive
CUTC	AC096677.1	0.378543092	1.76E-18	postive
CUTC	AC096772.6	0.354182842	3.18E-16	postive
CUTC	AC097468.3	0.412259182	6.16E-22	postive
CUTC	AC098484.1	0.379146807	1.54E-18	postive
CUTC	AC098484.2	0.338549707	7.13E-15	postive
CUTC	AC098487.1	0.424628208	2.63E-23	postive
CUTC	AC098820.2	0.542760531	1.20E-39	postive
CUTC	AC098820.4	0.40297215	6.04E-21	postive
CUTC	AC098934.1	0.311386415	1.05E-12	postive
CUTC	AC099343.4	0.311775398	9.85E-13	postive
CUTC	AC099778.1	0.301608603	5.64E-12	postive
CUTC	AC099850.2	0.454224707	8.02E-27	postive
CUTC	AC100810.1	0.405342814	3.40E-21	postive
CUTC	AC100847.1	0.351621021	5.36E-16	postive
CUTC	AC100861.1	0.408804855	1.45E-21	postive
CUTC	AC103760.1	0.311424797	1.05E-12	postive
CUTC	AC103764.1	0.305354334	2.99E-12	postive
CUTC	AC104109.2	0.336042619	1.15E-14	postive
CUTC	AC104113.1	0.362549611	5.61E-17	postive
CUTC	AC104596.1	0.335682414	1.24E-14	postive
CUTC	AC104667.2	0.361941048	6.38E-17	postive
CUTC	AC105036.3	0.432301804	3.48E-24	postive
CUTC	AC105285.1	0.362306348	5.91E-17	postive
CUTC	AC105942.1	0.340112344	5.26E-15	postive
CUTC	AC106791.1	0.3283808	4.91E-14	postive
CUTC	AC106795.1	0.424096407	3.02E-23	postive

CUTC	AC107027.3	0.341325169	4.16E-15	postive
CUTC	AC107032.2	0.394977286	4.08E-20	postive
CUTC	AC107068.1	0.38384615	5.34E-19	postive
CUTC	AC107214.1	0.360948476	7.85E-17	postive
CUTC	AC107375.1	0.366292437	2.54E-17	postive
CUTC	AC107959.3	0.338962344	6.58E-15	postive
CUTC	AC107982.3	0.339687596	5.72E-15	postive
CUTC	AC108471.3	0.302016469	5.27E-12	postive
CUTC	AC108488.1	0.375400077	3.53E-18	postive
CUTC	AC108860.2	0.313770355	6.94E-13	postive
CUTC	AC109322.1	0.312218171	9.11E-13	postive
CUTC	AC109347.1	0.386825721	2.71E-19	postive
CUTC	AC112220.2	0.365880244	2.77E-17	postive
CUTC	AC112694.1	0.310816949	1.16E-12	postive
CUTC	AC114271.1	0.325161182	8.91E-14	postive
CUTC	AC114489.2	0.322926027	1.34E-13	postive
CUTC	AC114810.1	0.410396153	9.80E-22	postive
CUTC	AC114956.2	0.333539949	1.86E-14	postive
CUTC	AC115618.1	0.346605017	1.47E-15	postive
CUTC	AC115618.2	0.437091365	9.58E-25	postive
CUTC	AC116913.1	0.415089377	3.03E-22	postive
CUTC	AC116914.2	0.368175784	1.70E-17	postive
CUTC	AC120053.1	0.328617627	4.69E-14	postive
CUTC	AC121761.1	0.350377167	6.90E-16	postive
CUTC	AC121761.2	0.319880645	2.33E-13	postive
CUTC	AC123768.2	0.365316118	3.13E-17	postive
CUTC	AC123768.3	0.374600687	4.20E-18	postive
CUTC	AC124016.2	0.367203774	2.09E-17	postive
CUTC	AC124242.1	0.35205681	4.91E-16	postive
CUTC	AC124283.2	0.313276175	7.57E-13	postive
CUTC	AC125257.1	0.42726095	1.32E-23	postive
CUTC	AC125494.1	0.382585987	7.10E-19	postive
CUTC	AC125807.2	0.31855049	2.96E-13	postive
CUTC	AC127070.1	0.318421953	3.03E-13	postive
CUTC	AC129507.3	0.303324893	4.22E-12	postive
CUTC	AC131009.3	0.411388303	7.66E-22	postive
CUTC	AC132192.2	0.323574129	1.19E-13	postive
CUTC	AC132938.4	0.435044464	1.67E-24	postive
CUTC	AC133528.2	0.357993133	1.45E-16	postive
CUTC	AC134878.2	0.326281264	7.24E-14	postive
CUTC	AC135050.1	0.352273977	4.70E-16	postive
CUTC	AC135050.6	0.301726758	5.53E-12	postive
CUTC	AC135178.4	0.398958418	1.59E-20	postive
CUTC	AC135178.6	0.347928511	1.13E-15	postive
CUTC	AC136475.2	0.36067763	8.31E-17	postive
CUTC	AC136604.3	0.419797025	9.16E-23	postive
CUTC	AC137932.1	0.311031399	1.12E-12	postive
CUTC	AC137932.2	0.338254639	7.54E-15	postive
CUTC	AC139530.1	0.322216119	1.53E-13	postive
CUTC	AC139768.1	0.409528409	1.21E-21	postive

CUTC	AC139887.2	0.384824017	4.28E-19	postive
CUTC	AC141586.1	0.339116381	6.39E-15	postive
CUTC	AC142472.1	0.345327577	1.89E-15	postive
CUTC	AC231981.1	0.464905232	3.54E-28	postive
CUTC	AC242426.2	0.319390758	2.55E-13	postive
CUTC	AC243919.1	0.382542821	7.17E-19	postive
CUTC	AC244669.1	0.401916199	7.80E-21	postive
CUTC	AC245140.2	0.341516221	4.00E-15	postive
CUTC	AC245297.4	0.379330515	1.47E-18	postive
CUTC	ADGRD1-AS1	0.434737328	1.81E-24	postive
CUTC	AF131216.4	0.315289353	5.30E-13	postive
CUTC	AFG3L1P	0.384253473	4.87E-19	postive
CUTC	AL009178.2	0.300838229	6.43E-12	postive
CUTC	AL009179.1	0.344891402	2.06E-15	postive
CUTC	AL020996.1	0.329173251	4.23E-14	postive
CUTC	AL021878.2	0.300708923	6.57E-12	postive
CUTC	AL022157.1	0.333155722	2.00E-14	postive
CUTC	AL022311.1	0.302533407	4.83E-12	postive
CUTC	AL023284.4	0.326759666	6.63E-14	postive
CUTC	AL024498.1	0.365411339	3.06E-17	postive
CUTC	AL024507.3	0.303521894	4.08E-12	postive
CUTC	AL031282.1	0.346543757	1.49E-15	postive
CUTC	AL031282.2	0.32917007	4.24E-14	postive
CUTC	AL031775.1	0.351702747	5.27E-16	postive
CUTC	AL033397.1	0.310680857	1.19E-12	postive
CUTC	AL035587.1	0.337660378	8.46E-15	postive
CUTC	AL035661.1	0.388824998	1.71E-19	postive
CUTC	AL049597.2	0.390372993	1.20E-19	postive
CUTC	AL049796.1	0.340295937	5.08E-15	postive
CUTC	AL049840.4	0.366103524	2.64E-17	postive
CUTC	AL049840.5	0.34233274	3.41E-15	postive
CUTC	AL049840.6	0.333944444	1.72E-14	postive
CUTC	AL050309.1	0.445388953	9.78E-26	postive
CUTC	AL080250.1	0.400279815	1.16E-20	postive
CUTC	AL080276.2	0.328898851	4.46E-14	postive
CUTC	AL117332.1	0.353398621	3.74E-16	postive
CUTC	AL117336.2	0.321138988	1.86E-13	postive
CUTC	AL118506.1	0.369360361	1.31E-17	postive
CUTC	AL118516.1	0.356430338	2.01E-16	postive
CUTC	AL118558.3	0.312500369	8.67E-13	postive
CUTC	AL121658.1	0.358992771	1.18E-16	postive
CUTC	AL121944.2	0.525513798	7.60E-37	postive
CUTC	AL122010.1	0.301282842	5.96E-12	postive
CUTC	AL122035.1	0.326649726	6.77E-14	postive
CUTC	AL132780.1	0.303645488	4.00E-12	postive
CUTC	AL133215.1	0.329554294	3.94E-14	postive
CUTC	AL133215.2	0.367855271	1.82E-17	postive
CUTC	AL133215.3	0.305344263	3.00E-12	postive
CUTC	AL133338.1	0.331695515	2.64E-14	postive
CUTC	AL133353.1	0.450933254	2.05E-26	postive

CUTC	AL133355.1	0.384735082	4.36E-19	postive
CUTC	AL135925.1	0.417815817	1.52E-22	postive
CUTC	AL136040.1	0.376910799	2.53E-18	postive
CUTC	AL137127.1	0.318327684	3.08E-13	postive
CUTC	AL138724.2	0.342068448	3.59E-15	postive
CUTC	AL138756.1	0.306368094	2.51E-12	postive
CUTC	AL138762.2	0.45870451	2.19E-27	postive
CUTC	AL138921.1	0.411463492	7.52E-22	postive
CUTC	AL138921.2	0.407409779	2.05E-21	postive
CUTC	AL139011.1	0.366781765	2.29E-17	postive
CUTC	AL139089.1	0.314451685	6.15E-13	postive
CUTC	AL139124.1	0.317532792	3.56E-13	postive
CUTC	AL139289.1	0.318262518	3.12E-13	postive
CUTC	AL139384.1	0.340861561	4.55E-15	postive
CUTC	AL139397.1	0.374294531	4.49E-18	postive
CUTC	AL157400.2	0.319934281	2.31E-13	postive
CUTC	AL157834.2	0.412042071	6.51E-22	postive
CUTC	AL158071.3	0.303059719	4.42E-12	postive
CUTC	AL158212.3	0.42164341	5.70E-23	postive
CUTC	AL158835.2	0.371013487	9.19E-18	postive
CUTC	AL160006.1	0.378224741	1.89E-18	postive
CUTC	AL160313.2	0.356472088	1.99E-16	postive
CUTC	AL161421.1	0.383776487	5.42E-19	postive
CUTC	AL162231.2	0.378429247	1.80E-18	postive
CUTC	AL162258.2	0.398040053	1.97E-20	postive
CUTC	AL162274.2	0.348668455	9.72E-16	postive
CUTC	AL162377.1	0.376486955	2.77E-18	postive
CUTC	AL162595.1	0.381921669	8.25E-19	postive
CUTC	AL162632.3	0.32251108	1.45E-13	postive
CUTC	AL353194.1	0.304732342	3.32E-12	postive
CUTC	AL353708.1	0.433155105	2.77E-24	postive
CUTC	AL353708.3	0.348913072	9.26E-16	postive
CUTC	AL353743.1	0.379632395	1.38E-18	postive
CUTC	AL354696.1	0.356916849	1.82E-16	postive
CUTC	AL354696.2	0.459129336	1.94E-27	postive
CUTC	AL354892.2	0.403244981	5.66E-21	postive
CUTC	AL354920.1	0.527345527	3.90E-37	postive
CUTC	AL355001.2	0.363618491	4.48E-17	postive
CUTC	AL355388.1	0.303937544	3.81E-12	postive
CUTC	AL355490.3	0.302671854	4.72E-12	postive
CUTC	AL355574.1	0.310097041	1.32E-12	postive
CUTC	AL355802.3	0.32591332	7.75E-14	postive
CUTC	AL356481.1	0.412054262	6.49E-22	postive
CUTC	AL356488.3	0.331530743	2.72E-14	postive
CUTC	AL358075.2	0.355819827	2.28E-16	postive
CUTC	AL358472.2	0.386965525	2.62E-19	postive
CUTC	AL359198.1	0.356147065	2.13E-16	postive
CUTC	AL359232.1	0.405486476	3.28E-21	postive
CUTC	AL359397.1	0.424253319	2.90E-23	postive
CUTC	AL359513.1	0.329522076	3.97E-14	postive

CUTC	AL359532.1	0.328193494	5.08E-14	postive
CUTC	AL359541.2	0.374770161	4.05E-18	postive
CUTC	AL359643.3	0.384511612	4.59E-19	postive
CUTC	AL359715.3	0.37123994	8.75E-18	postive
CUTC	AL359878.1	0.344852555	2.08E-15	postive
CUTC	AL359921.2	0.393359421	5.96E-20	postive
CUTC	AL360181.2	0.3228969	1.35E-13	postive
CUTC	AL365181.2	0.367266766	2.06E-17	postive
CUTC	AL365181.3	0.397227024	2.40E-20	postive
CUTC	AL390728.4	0.333931418	1.73E-14	postive
CUTC	AL390955.2	0.37196353	7.48E-18	postive
CUTC	AL391001.1	0.329068349	4.32E-14	postive
CUTC	AL391121.1	0.441428308	2.93E-25	postive
CUTC	AL391684.1	0.437553645	8.45E-25	postive
CUTC	AL391832.2	0.312471649	8.72E-13	postive
CUTC	AL391834.2	0.31538574	5.21E-13	postive
CUTC	AL392172.1	0.40496944	3.72E-21	postive
CUTC	AL441992.2	0.389664807	1.41E-19	postive
CUTC	AL445524.1	0.307545186	2.05E-12	postive
CUTC	AL450326.1	0.425651558	2.01E-23	postive
CUTC	AL450998.2	0.34622073	1.58E-15	postive
CUTC	AL451085.3	0.356905321	1.82E-16	postive
CUTC	AL451165.2	0.411913908	6.72E-22	postive
CUTC	AL512353.1	0.321705324	1.68E-13	postive
CUTC	AL512625.1	0.381460031	9.15E-19	postive
CUTC	AL512770.1	0.306115703	2.62E-12	postive
CUTC	AL513550.1	0.412397858	5.95E-22	postive
CUTC	AL590617.2	0.36610955	2.64E-17	postive
CUTC	AL590666.2	0.365115119	3.26E-17	postive
CUTC	AL591043.2	0.317872404	3.35E-13	postive
CUTC	AL591684.2	0.34689475	1.39E-15	postive
CUTC	AL592166.1	0.40934982	1.27E-21	postive
CUTC	AL592435.1	0.337068174	9.48E-15	postive
CUTC	AL596202.1	0.365175503	3.22E-17	postive
CUTC	AL645568.1	0.368795055	1.48E-17	postive
CUTC	AL645941.1	0.327610747	5.66E-14	postive
CUTC	AL662844.4	0.311946153	9.56E-13	postive
CUTC	AL671710.1	0.354457784	3.01E-16	postive
CUTC	AL731566.2	0.453452775	1.00E-26	postive
CUTC	AL731569.1	0.414214913	3.78E-22	postive
CUTC	AL731571.1	0.384719656	4.38E-19	postive
CUTC	AL731577.2	0.448922659	3.63E-26	postive
CUTC	AL732314.8	0.336269658	1.11E-14	postive
CUTC	ALOX12-AS1	0.500980917	3.97E-33	postive
CUTC	ALOX12P2	0.399249337	1.48E-20	postive
CUTC	AMZ2P1	0.437462206	8.67E-25	postive
CUTC	ANO7L1	0.3308259	3.11E-14	postive
CUTC	ANP32AP1	0.335095922	1.38E-14	postive
CUTC	AP000254.2	0.346709806	1.44E-15	postive
CUTC	AP000442.1	0.399624119	1.35E-20	postive

CUTC	AP000487.1	0.300593705	6.69E-12	postive
CUTC	AP000688.1	0.315117893	5.47E-13	postive
CUTC	AP000704.2	0.305565067	2.88E-12	postive
CUTC	AP000759.1	0.407294204	2.11E-21	postive
CUTC	AP000873.2	0.337836297	8.18E-15	postive
CUTC	AP001107.1	0.362540525	5.62E-17	postive
CUTC	AP001160.3	0.416694847	2.02E-22	postive
CUTC	AP001318.2	0.35319562	3.89E-16	postive
CUTC	AP001372.2	0.388108697	2.02E-19	postive
CUTC	AP001453.3	0.309521484	1.46E-12	postive
CUTC	AP001453.4	0.377599196	2.17E-18	postive
CUTC	AP001462.1	0.302827422	4.59E-12	postive
CUTC	AP001486.2	0.342533797	3.28E-15	postive
CUTC	AP001505.1	0.320604648	2.05E-13	postive
CUTC	AP001542.3	0.302778047	4.63E-12	postive
CUTC	AP001816.1	0.401854518	7.92E-21	postive
CUTC	AP002360.2	0.384609131	4.49E-19	postive
CUTC	AP002748.4	0.379922083	1.29E-18	postive
CUTC	AP002840.2	0.30987491	1.37E-12	postive
CUTC	AP003068.1	0.301466999	5.78E-12	postive
CUTC	AP003068.2	0.339316843	6.14E-15	postive
CUTC	AP003119.1	0.35345552	3.69E-16	postive
CUTC	AP003352.1	0.429161974	8.01E-24	postive
CUTC	AP003392.5	0.330994007	3.01E-14	postive
CUTC	AP003486.1	0.309934186	1.36E-12	postive
CUTC	AP004609.5	0.310084175	1.32E-12	postive
CUTC	AP005329.1	0.327105852	6.22E-14	postive
CUTC	AP005482.4	0.409990932	1.08E-21	postive
CUTC	AP006621.3	0.311258691	1.08E-12	postive
CUTC	APTR	0.325196922	8.85E-14	postive
CUTC	ARF4-AS1	0.406249579	2.72E-21	postive
CUTC	ARMCX5-GPRASP2	0.539738242	3.81E-39	postive
CUTC	ARRDC1-AS1	0.417723879	1.55E-22	postive
CUTC	ASB16-AS1	0.340007464	5.37E-15	postive
CUTC	ASH1L-AS1	0.408559253	1.54E-21	postive
CUTC	ATF4P4	0.403711058	5.05E-21	postive
CUTC	ATXN1-AS1	0.31692471	3.96E-13	postive
CUTC	ATXN2-AS	0.340308672	5.07E-15	postive
CUTC	AZIN1-AS1	0.417193162	1.78E-22	postive
CUTC	BAALC-AS1	0.448768986	3.79E-26	postive
CUTC	BAIAP2-DT	0.359098	1.16E-16	postive
CUTC	BCDIN3D-AS1	0.447406187	5.56E-26	postive
CUTC	BDNF-AS	0.316130757	4.57E-13	postive
CUTC	BEND3P1	0.315108612	5.47E-13	postive
CUTC	BHLHE40-AS1	0.364017956	4.12E-17	postive
CUTC	BMS1P20	0.414132115	3.85E-22	postive
CUTC	BNIP3P1	0.300431577	6.88E-12	postive
CUTC	BTNL10	0.339450769	5.98E-15	postive
CUTC	BVES-AS1	0.322408483	1.47E-13	postive
CUTC	BX284668.5	0.382242175	7.67E-19	postive

CUTC	BX537318.1	0.424051422	3.06E-23	postive
CUTC	BX649632.1	0.345777119	1.73E-15	postive
CUTC	C10orf25	0.325940798	7.71E-14	postive
CUTC	C18orf65	0.353844006	3.41E-16	postive
CUTC	C1RL-AS1	0.313192666	7.68E-13	postive
CUTC	C21orf62-AS1	0.40909811	1.35E-21	postive
CUTC	C22orf46	0.356376879	2.03E-16	postive
CUTC	C2CD4D-AS1	0.359376699	1.09E-16	postive
CUTC	C6orf99	0.340979411	4.45E-15	postive
CUTC	C8orf37-AS1	0.328172315	5.10E-14	postive
CUTC	C9orf147	0.315563491	5.05E-13	postive
CUTC	CA5BP1	0.484300021	9.14E-31	postive
CUTC	CAHM	0.324615836	9.84E-14	postive
CUTC	CARD8-AS1	0.304346107	3.55E-12	postive
CUTC	CASC8	0.328868737	4.48E-14	postive
CUTC	CASC9	0.326395628	7.09E-14	postive
CUTC	CATIP-AS1	0.372872762	6.14E-18	postive
CUTC	CATSPER2P1	0.396796917	2.65E-20	postive
CUTC	CCNT2-AS1	0.43303631	2.86E-24	postive
CUTC	CCT6P1	0.31440988	6.20E-13	postive
CUTC	CD27-AS1	0.416347155	2.20E-22	postive
CUTC	CD99P1	0.342008934	3.64E-15	postive
CUTC	CDC37L1-DT	0.355154155	2.61E-16	postive
CUTC	CENPBD1P1	0.484632765	8.22E-31	postive
CUTC	CERNA1	0.316818612	4.04E-13	postive
CUTC	CFL1P1	0.425283954	2.22E-23	postive
CUTC	CHROMR	0.34619773	1.59E-15	postive
CUTC	CIDECP1	0.486156425	5.06E-31	postive
CUTC	CKMT2-AS1	0.402476987	6.81E-21	postive
CUTC	CNNM3-DT	0.356483892	1.99E-16	postive
CUTC	COA6-AS1	0.383074867	6.36E-19	postive
CUTC	COX10-AS1	0.437495569	8.59E-25	postive
CUTC	CPNE8-AS1	0.335768675	1.22E-14	postive
CUTC	CR381653.1	0.37912634	1.54E-18	postive
CUTC	CRNDE	0.324648371	9.79E-14	postive
CUTC	CRTC3-AS1	0.367039669	2.16E-17	postive
CUTC	CRYBB2P1	0.310226535	1.29E-12	postive
CUTC	CRYZL2P	0.454508166	7.39E-27	postive
CUTC	CTA-989H11.1	0.364232179	3.93E-17	postive
CUTC	CTBP1-DT	0.472443807	3.65E-29	postive
CUTC	CUTALP	0.323862548	1.13E-13	postive
CUTC	CYP51A1-AS1	0.308020232	1.89E-12	postive
CUTC	CYTOR	0.341948457	3.68E-15	postive
CUTC	DANCR	0.368551957	1.56E-17	postive
CUTC	DARS-AS1	0.473872743	2.36E-29	postive
CUTC	DBIL5P	0.327962984	5.30E-14	postive
CUTC	DCXR-DT	0.346032788	1.64E-15	postive
CUTC	DDX39B-AS1	0.315811541	4.83E-13	postive
CUTC	DENND10P1	0.310890553	1.15E-12	postive
CUTC	DGCR11	0.393817234	5.35E-20	postive

CUTC	DHCR24-DT	0.319927907	2.31E-13	postive
CUTC	DHRS4-AS1	0.438694548	6.20E-25	postive
CUTC	DLEU1	0.456292147	4.42E-27	postive
CUTC	DLGAP1-AS1	0.525860115	6.70E-37	postive
CUTC	DLGAP1-AS2	0.369279792	1.34E-17	postive
CUTC	DNAJC27-AS1	0.33453824	1.54E-14	postive
CUTC	DNMBP-AS1	0.328264484	5.01E-14	postive
CUTC	DSCAS	0.324823477	9.48E-14	postive
CUTC	DSTNP2	0.457604735	3.02E-27	postive
CUTC	EBLN3P	0.426195125	1.75E-23	postive
CUTC	ECI2-DT	0.309912124	1.36E-12	postive
CUTC	EDRF1-DT	0.453061156	1.12E-26	postive
CUTC	EIF1B-AS1	0.4461705	7.86E-26	postive
CUTC	EIF2AK3-DT	0.364787399	3.50E-17	postive
CUTC	EIF3J-DT	0.502673633	2.25E-33	postive
CUTC	ELF3-AS1	0.326793156	6.59E-14	postive
CUTC	ELOA-AS1	0.43442607	1.97E-24	postive
CUTC	EMC1-AS1	0.413028972	5.08E-22	postive
CUTC	ENTPD3-AS1	0.572697224	6.48E-45	postive
CUTC	EPB41L4A-AS1	0.443808583	1.52E-25	postive
CUTC	EPCAM-DT	0.326678168	6.73E-14	postive
CUTC	FAHD2CP	0.331394994	2.79E-14	postive
CUTC	FALEC	0.34682701	1.40E-15	postive
CUTC	FAM111A-DT	0.318174466	3.17E-13	postive
CUTC	FAM201A	0.345665935	1.77E-15	postive
CUTC	FAM21EP	0.307492672	2.07E-12	postive
CUTC	FAM21FP	0.315092232	5.49E-13	postive
CUTC	FAM86B3P	0.336071483	1.15E-14	postive
CUTC	FAM86C2P	0.453537156	9.77E-27	postive
CUTC	FAM86DP	0.382531499	7.19E-19	postive
CUTC	FAM86EP	0.479744112	3.83E-30	postive
CUTC	FAM86FP	0.341685793	3.87E-15	postive
CUTC	FAM86HP	0.309650486	1.43E-12	postive
CUTC	FAM86JP	0.449355913	3.21E-26	postive
CUTC	FGD5-AS1	0.520490931	4.64E-36	postive
CUTC	FGF14-AS2	0.30278272	4.63E-12	postive
CUTC	FLVCR1-DT	0.389760649	1.38E-19	postive
CUTC	FOXP4-AS1	0.342671176	3.19E-15	postive
CUTC	FRG1HP	0.507883558	3.83E-34	postive
CUTC	FZD4-DT	0.426039263	1.82E-23	postive
CUTC	GAPLINC	0.356722767	1.89E-16	postive
CUTC	GAS5	0.415467055	2.75E-22	postive
CUTC	GASAL1	0.325466219	8.42E-14	postive
CUTC	GCC2-AS1	0.394254622	4.83E-20	postive
CUTC	GLIDR	0.408335728	1.63E-21	postive
CUTC	GLUD1P2	0.352581994	4.41E-16	postive
CUTC	GLUD1P3	0.333499324	1.87E-14	postive
CUTC	GMDS-DT	0.372022247	7.38E-18	postive
CUTC	GNRHR2	0.36092542	7.89E-17	postive
CUTC	GPRC5D-AS1	0.417122835	1.81E-22	postive

CUTC	GSEC	0.405004675	3.69E-21	postive
CUTC	GTF2H2B	0.303816625	3.89E-12	postive
CUTC	GTF2IP1	0.356343617	2.04E-16	postive
CUTC	GTF2IP4	0.320001875	2.28E-13	postive
CUTC	GTSE1-DT	0.305243192	3.05E-12	postive
CUTC	GUSBP1	0.301491566	5.76E-12	postive
CUTC	GUSBP2	0.326593703	6.84E-14	postive
CUTC	GYG2P1	0.308323805	1.80E-12	postive
CUTC	H2AZ2P1	0.331040458	2.98E-14	postive
CUTC	H2BC19P	0.301939911	5.34E-12	postive
CUTC	HCG11	0.305405885	2.96E-12	postive
CUTC	HCG17	0.303714318	3.95E-12	postive
CUTC	HCG18	0.398497732	1.77E-20	postive
CUTC	HCG25	0.44900431	3.55E-26	postive
CUTC	HEIH	0.442638739	2.10E-25	postive
CUTC	HMGB1P5	0.546665544	2.64E-40	postive
CUTC	HNRNPA1P6	0.3108727	1.15E-12	postive
CUTC	HPN-AS1	0.315479064	5.13E-13	postive
CUTC	HSP90AB2P	0.303803842	3.89E-12	postive
CUTC	ID2-AS1	0.312624122	8.49E-13	postive
CUTC	IL6R-AS1	0.347341074	1.27E-15	postive
CUTC	ILF3-DT	0.351311639	5.71E-16	postive
CUTC	INTS6-AS1	0.364644392	3.60E-17	postive
CUTC	IQCH-AS1	0.438645104	6.28E-25	postive
CUTC	IRAIN	0.322198697	1.53E-13	postive
CUTC	ITFG2-AS1	0.458753842	2.16E-27	postive
CUTC	ITGA9-AS1	0.331291592	2.85E-14	postive
CUTC	JPX	0.430781039	5.22E-24	postive
CUTC	KCNIP2-AS1	0.340415312	4.96E-15	postive
CUTC	KMT2E-AS1	0.319577856	2.46E-13	postive
CUTC	KRT18P55	0.347775098	1.16E-15	postive
CUTC	KTN1-AS1	0.468899825	1.07E-28	postive
CUTC	LAMTOR5-AS1	0.371567925	8.15E-18	postive
CUTC	LBX2-AS1	0.32949697	3.98E-14	postive
CUTC	LENG8-AS1	0.319684247	2.42E-13	postive
CUTC	LINC00239	0.41745473	1.66E-22	postive
CUTC	LINC002481	0.344778744	2.11E-15	postive
CUTC	LINC00324	0.417648404	1.58E-22	postive
CUTC	LINC00339	0.467715158	1.53E-28	postive
CUTC	LINC00467	0.550697633	5.42E-41	postive
CUTC	LINC00471	0.312422835	8.79E-13	postive
CUTC	LINC00476	0.472517133	3.57E-29	postive
CUTC	LINC00526	0.313999676	6.66E-13	postive
CUTC	LINC00623	0.315655539	4.97E-13	postive
CUTC	LINC00630	0.315018499	5.56E-13	postive
CUTC	LINC00654	0.317220874	3.76E-13	postive
CUTC	LINC00665	0.304140518	3.68E-12	postive
CUTC	LINC00667	0.447936123	4.79E-26	postive
CUTC	LINC00680	0.406345003	2.66E-21	postive
CUTC	LINC00847	0.332769657	2.15E-14	postive

CUTC	LINC00857	0.404115426	4.58E-21	postive
CUTC	LINC00863	0.546967966	2.34E-40	postive
CUTC	LINC00869	0.34344067	2.75E-15	postive
CUTC	LINC00888	0.371398769	8.46E-18	postive
CUTC	LINC00909	0.480622042	2.91E-30	postive
CUTC	LINC00910	0.398239639	1.88E-20	postive
CUTC	LINC00921	0.328950141	4.41E-14	postive
CUTC	LINC00933	0.347554882	1.21E-15	postive
CUTC	LINC00957	0.306658348	2.39E-12	postive
CUTC	LINC00963	0.479283882	4.43E-30	postive
CUTC	LINC00997	0.304363782	3.54E-12	postive
CUTC	LINC01011	0.428306613	1.00E-23	postive
CUTC	LINC01058	0.388243872	1.96E-19	postive
CUTC	LINC01128	0.390269186	1.22E-19	postive
CUTC	LINC01137	0.503818333	1.53E-33	postive
CUTC	LINC01138	0.316651082	4.16E-13	postive
CUTC	LINC01145	0.347734902	1.17E-15	postive
CUTC	LINC01184	0.476813625	9.54E-30	postive
CUTC	LINC01269	0.333572494	1.85E-14	postive
CUTC	LINC01270	0.328256638	5.02E-14	postive
CUTC	LINC01278	0.390586485	1.14E-19	postive
CUTC	LINC01301	0.317278826	3.72E-13	postive
CUTC	LINC01315	0.306066005	2.65E-12	postive
CUTC	LINC01353	0.336516508	1.05E-14	postive
CUTC	LINC01484	0.305272637	3.03E-12	postive
CUTC	LINC01504	0.332137773	2.43E-14	postive
CUTC	LINC01508	0.318104299	3.21E-13	postive
CUTC	LINC01515	0.332678005	2.19E-14	postive
CUTC	LINC01560	0.36670955	2.32E-17	postive
CUTC	LINC01569	0.303150086	4.35E-12	postive
CUTC	LINC01578	0.326826177	6.55E-14	postive
CUTC	LINC01635	0.332308483	2.35E-14	postive
CUTC	LINC01679	0.307666033	2.01E-12	postive
CUTC	LINC01719	0.439730247	4.67E-25	postive
CUTC	LINC01806	0.377080346	2.43E-18	postive
CUTC	LINC01881	0.442369118	2.26E-25	postive
CUTC	LINC01924	0.309688471	1.42E-12	postive
CUTC	LINC01942	0.368058356	1.74E-17	postive
CUTC	LINC02012	0.329278948	4.15E-14	postive
CUTC	LINC02014	0.364668415	3.59E-17	postive
CUTC	LINC02041	0.305709694	2.81E-12	postive
CUTC	LINC02109	0.324221163	1.06E-13	postive
CUTC	LINC02193	0.342459358	3.33E-15	postive
CUTC	LINC02210	0.374053042	4.74E-18	postive
CUTC	LINC02298	0.349831408	7.70E-16	postive
CUTC	LINC02495	0.317402021	3.64E-13	postive
CUTC	LINC02542	0.358910522	1.20E-16	postive
CUTC	LINC02569	0.317738539	3.43E-13	postive
CUTC	LINC02585	0.388114499	2.01E-19	postive
CUTC	LINC02637	0.329015295	4.36E-14	postive

CUTC	LINC02693	0.300362194	6.96E-12	postive
CUTC	LINC02709	0.31639105	4.36E-13	postive
CUTC	LINC02749	0.371087416	9.05E-18	postive
CUTC	LINC02761	0.308060816	1.88E-12	postive
CUTC	LINP1	0.321654662	1.69E-13	postive
CUTC	LIVAR	0.314673038	5.91E-13	postive
CUTC	LIX1L-AS1	0.357067722	1.76E-16	postive
CUTC	LMCD1-AS1	0.396720569	2.70E-20	postive
CUTC	LOH12CR2	0.386746244	2.76E-19	postive
CUTC	LRRC37A17P	0.344266228	2.33E-15	postive
CUTC	LRRC37BP1	0.428085132	1.06E-23	postive
CUTC	LSP1P4	0.329700462	3.84E-14	postive
CUTC	MAFG-DT	0.353474721	3.68E-16	postive
CUTC	MAN1B1-DT	0.34426479	2.33E-15	postive
CUTC	MAP2K4P1	0.441702159	2.72E-25	postive
CUTC	MAP3K14-AS1	0.438287871	6.92E-25	postive
CUTC	MAP4K3-DT	0.43576943	1.37E-24	postive
CUTC	MAPKAPK5-AS1	0.673872038	1.84E-67	postive
CUTC	MCM3AP-AS1	0.33334867	1.93E-14	postive
CUTC	MCPH1-AS1	0.342006761	3.64E-15	postive
CUTC	MFF-DT	0.356582356	1.95E-16	postive
CUTC	MIATNB	0.380375912	1.17E-18	postive
CUTC	MIF-AS1	0.340463815	4.92E-15	postive
CUTC	MINCR	0.435420362	1.51E-24	postive
CUTC	MIPEPP3	0.342138677	3.54E-15	postive
CUTC	MIR193BHG	0.334561634	1.53E-14	postive
CUTC	MIR22HG	0.423227262	3.79E-23	postive
CUTC	MIR3936HG	0.310462075	1.24E-12	postive
CUTC	MIR4435-2HG	0.404935801	3.75E-21	postive
CUTC	MIR4453HG	0.343351319	2.79E-15	postive
CUTC	MIR4713HG	0.320306264	2.16E-13	postive
CUTC	MIR762HG	0.356362753	2.04E-16	postive
CUTC	MKLN1-AS	0.469255303	9.60E-29	postive
CUTC	MMADHC-DT	0.379855352	1.31E-18	postive
CUTC	MRPL20-AS1	0.465443159	3.01E-28	postive
CUTC	MRPL45P2	0.45280836	1.20E-26	postive
CUTC	MRPS31P4	0.392019552	8.15E-20	postive
CUTC	MRPS31P5	0.333157936	2.00E-14	postive
CUTC	MSH5-SAPCD1	0.320343162	2.14E-13	postive
CUTC	MYLK-AS1	0.330514571	3.29E-14	postive
CUTC	NACA4P	0.396572256	2.80E-20	postive
CUTC	NAPA-AS1	0.452528742	1.30E-26	postive
CUTC	NBPF25P	0.324732345	9.64E-14	postive
CUTC	NBPF8	0.333736711	1.79E-14	postive
CUTC	NBR2	0.400425816	1.12E-20	postive
CUTC	NCK1-DT	0.44692079	6.37E-26	postive
CUTC	NDUFA6-DT	0.327435815	5.85E-14	postive
CUTC	NDUFB2-AS1	0.336556935	1.05E-14	postive
CUTC	NDUFV2-AS1	0.330137429	3.53E-14	postive
CUTC	NIFK-AS1	0.528636935	2.43E-37	postive

CUTC	NINJ2-AS1	0.34423438	2.35E-15	postive
CUTC	NKAPP1	0.33749481	8.73E-15	postive
CUTC	NNT-AS1	0.304097071	3.70E-12	postive
CUTC	NOP14-AS1	0.421103885	6.55E-23	postive
CUTC	NOP53-AS1	0.35022299	7.11E-16	postive
CUTC	NORAD	0.384449556	4.66E-19	postive
CUTC	NRAV	0.47629555	1.12E-29	postive
CUTC	NRSN2-AS1	0.57625244	1.41E-45	postive
CUTC	NUDT16P1	0.465479717	2.98E-28	postive
CUTC	NUTM2A-AS1	0.667780113	7.50E-66	postive
CUTC	NUTM2B-AS1	0.416979586	1.88E-22	postive
CUTC	OGFRP1	0.405426495	3.33E-21	postive
CUTC	OIP5-AS1	0.512244926	8.50E-35	postive
CUTC	OLMALINC	0.50593545	7.45E-34	postive
CUTC	OSER1-DT	0.450008003	2.67E-26	postive
CUTC	OSGEPL1-AS1	0.309775189	1.40E-12	postive
CUTC	OTUD6B-AS1	0.528971719	2.15E-37	postive
CUTC	PARGP1	0.322923578	1.34E-13	postive
CUTC	PAXBP1-AS1	0.326605901	6.82E-14	postive
CUTC	PAXIP1-AS1	0.332592282	2.23E-14	postive
CUTC	PAXIP1-AS2	0.362326643	5.88E-17	postive
CUTC	PCBP1-AS1	0.415750961	2.56E-22	postive
CUTC	PCOTH	0.335431993	1.30E-14	postive
CUTC	PDCD4-AS1	0.345914473	1.68E-15	postive
CUTC	PDIA3P1	0.405731587	3.09E-21	postive
CUTC	PINK1-AS	0.354898158	2.75E-16	postive
CUTC	PIPSL	0.371046153	9.13E-18	postive
CUTC	PITPNA-AS1	0.569985268	2.05E-44	postive
CUTC	PLEKHA8P1	0.42275413	4.28E-23	postive
CUTC	PMS2CL	0.34640119	1.53E-15	postive
CUTC	PMS2P4	0.402967982	6.05E-21	postive
CUTC	POLH-AS1	0.360353513	8.89E-17	postive
CUTC	POLR2J4	0.354529558	2.97E-16	postive
CUTC	PPP1R26-AS1	0.316373036	4.37E-13	postive
CUTC	PPP3CB-AS1	0.549791512	7.74E-41	postive
CUTC	PPT2-EGFL8	0.32248273	1.45E-13	postive
CUTC	PRELID1P1	0.424463476	2.75E-23	postive
CUTC	PRKCQ-AS1	0.410627881	9.25E-22	postive
CUTC	PRORSD1P	0.351531183	5.46E-16	postive
CUTC	PRR34	0.347839265	1.15E-15	postive
CUTC	PRR34-AS1	0.35039165	6.88E-16	postive
CUTC	PRRT3-AS1	0.368556478	1.56E-17	postive
CUTC	PSMA3-AS1	0.332508661	2.26E-14	postive
CUTC	PSMB8-AS1	0.341649061	3.90E-15	postive
CUTC	PTENP1	0.424213658	2.93E-23	postive
CUTC	PTMAP5	0.31178752	9.83E-13	postive
CUTC	PTOV1-AS1	0.39254585	7.21E-20	postive
CUTC	PXN-AS1	0.610677402	1.99E-52	postive
CUTC	RAB11B-AS1	0.40698721	2.27E-21	postive
CUTC	RAB30-DT	0.336186968	1.12E-14	postive

CUTC	RAD21-AS1	0.352156657	4.81E-16	postive
CUTC	RAD51-AS1	0.305368246	2.98E-12	postive
CUTC	RALY-AS1	0.322033035	1.58E-13	postive
CUTC	RAP2C-AS1	0.356800643	1.86E-16	postive
CUTC	RARA-AS1	0.366248578	2.56E-17	postive
CUTC	RASA4CP	0.31850032	2.99E-13	postive
CUTC	RASSF10-DT	0.307546707	2.05E-12	postive
CUTC	RBM15-AS1	0.532548062	5.72E-38	postive
CUTC	RBM26-AS1	0.345469761	1.84E-15	postive
CUTC	RNASEH1-AS1	0.447785454	5.00E-26	postive
CUTC	RNF139-AS1	0.300057951	7.32E-12	postive
CUTC	RNF216P1	0.389242316	1.55E-19	postive
CUTC	RP11-101E13.5	0.442982207	1.91E-25	postive
CUTC	RP11-104N10.2	0.310645352	1.20E-12	postive
CUTC	RP11-10C24.1	0.342520961	3.29E-15	postive
CUTC	RP11-175O19.4	0.408642206	1.51E-21	postive
CUTC	RP11-199F11.2	0.335986375	1.17E-14	postive
CUTC	RP11-226L15.5	0.375151303	3.72E-18	postive
CUTC	RP1-122P22.2	0.352254854	4.72E-16	postive
CUTC	RP11-282O18.3	0.323684839	1.17E-13	postive
CUTC	RP11-314C16.1	0.319905401	2.32E-13	postive
CUTC	RP11-337C18.9	0.31544874	5.15E-13	postive
CUTC	RP11-435O5.2	0.335472862	1.29E-14	postive
CUTC	RP11-529E10.6	0.300772016	6.50E-12	postive
CUTC	RP11-539I5.1	0.45272812	1.23E-26	postive
CUTC	RP11-542K23.9	0.337498776	8.73E-15	postive
CUTC	RP11-61L19.3	0.313551167	7.21E-13	postive
CUTC	RP11-706O15.1	0.323250931	1.26E-13	postive
CUTC	RP11-86H7.7	0.329344624	4.10E-14	postive
CUTC	RP11-89K10.1	0.378597653	1.74E-18	postive
CUTC	RP4-545L17.11	0.395541916	3.57E-20	postive
CUTC	RP9P	0.350264408	7.05E-16	postive
CUTC	RPARP-AS1	0.527561143	3.60E-37	postive
CUTC	RPL12P38	0.370851438	9.52E-18	postive
CUTC	RPL13AP3	0.364383496	3.81E-17	postive
CUTC	RPL13AP7	0.423848361	3.22E-23	postive
CUTC	RPL13P5	0.449489055	3.09E-26	postive
CUTC	RPL23AP53	0.399809395	1.29E-20	postive
CUTC	RPL23AP7	0.484910153	7.53E-31	postive
CUTC	RPL23AP82	0.559207586	1.78E-42	postive
CUTC	RPL29P30	0.330680078	3.19E-14	postive
CUTC	RPL36AP21	0.351648313	5.33E-16	postive
CUTC	RPL37P6	0.330351155	3.40E-14	postive
CUTC	RPL6P27	0.398734442	1.67E-20	postive
CUTC	RPS10P7	0.368180197	1.69E-17	postive
CUTC	RPS18P9	0.401338753	8.96E-21	postive
CUTC	RPS27AP16	0.334110044	1.67E-14	postive
CUTC	RPS4XP16	0.476288728	1.12E-29	postive
CUTC	RPS4XP2	0.329196042	4.21E-14	postive
CUTC	RPSAP31	0.326925611	6.43E-14	postive

CUTC	RRN3P3	0.342468233	3.32E-15	postive
CUTC	RRP7BP	0.395445839	3.65E-20	postive
CUTC	SAMD12-AS1	0.300286917	7.05E-12	postive
CUTC	SATB1-AS1	0.309886524	1.37E-12	postive
CUTC	SBDSP1	0.43556382	1.45E-24	postive
CUTC	SBF2-AS1	0.410655131	9.19E-22	postive
CUTC	SBNO1-AS1	0.351139081	5.91E-16	postive
CUTC	SCAMP1-AS1	0.460918362	1.15E-27	postive
CUTC	SCAND2P	0.371647932	8.01E-18	postive
CUTC	SDAD1P1	0.30851482	1.74E-12	postive
CUTC	SDCBP2-AS1	0.43455564	1.90E-24	postive
CUTC	SEC22B4P	0.334061345	1.68E-14	postive
CUTC	SEC24B-AS1	0.338877575	6.69E-15	postive
CUTC	SEPSECS-AS1	0.386835011	2.70E-19	postive
CUTC	SEPTIN4-AS1	0.345222058	1.93E-15	postive
CUTC	SGMS1-AS1	0.5102083	1.72E-34	postive
CUTC	SHLD2P1	0.302765452	4.64E-12	postive
CUTC	SLC12A9-AS1	0.303155079	4.35E-12	postive
CUTC	SLC25A5-AS1	0.303368492	4.19E-12	postive
CUTC	SLC2A1-AS1	0.331798887	2.59E-14	postive
CUTC	SLC44A3-AS1	0.399037572	1.56E-20	postive
CUTC	SMG7-AS1	0.433746726	2.36E-24	postive
CUTC	SNAI3-AS1	0.434048099	2.18E-24	postive
CUTC	SNHG1	0.401037613	9.64E-21	postive
CUTC	SNHG11	0.48452998	8.50E-31	postive
CUTC	SNHG12	0.320631949	2.04E-13	postive
CUTC	SNHG16	0.488596323	2.32E-31	postive
CUTC	SNHG17	0.374541305	4.26E-18	postive
CUTC	SNHG20	0.349295648	8.57E-16	postive
CUTC	SNHG21	0.425160096	2.29E-23	postive
CUTC	SNHG28	0.337207096	9.23E-15	postive
CUTC	SNHG29	0.479167985	4.59E-30	postive
CUTC	SNHG3	0.327470274	5.81E-14	postive
CUTC	SNHG32	0.480053196	3.48E-30	postive
CUTC	SNHG4	0.389353866	1.51E-19	postive
CUTC	SNHG5	0.49297259	5.62E-32	postive
CUTC	SNHG6	0.480739658	2.81E-30	postive
CUTC	SNHG7	0.465560188	2.91E-28	postive
CUTC	SNHG8	0.524686159	1.03E-36	postive
CUTC	SP2-AS1	0.307996493	1.90E-12	postive
CUTC	SPINT1-AS1	0.386842204	2.70E-19	postive
CUTC	SPRY4-AS1	0.326647102	6.77E-14	postive
CUTC	SREBF2-AS1	0.342293262	3.44E-15	postive
CUTC	SRP14-AS1	0.589757111	3.63E-48	postive
CUTC	ST20-AS1	0.349036466	9.03E-16	postive
CUTC	STAG3L1	0.360128736	9.32E-17	postive
CUTC	STAG3L2	0.37241728	6.78E-18	postive
CUTC	STAG3L3	0.319557116	2.47E-13	postive
CUTC	STAG3L4	0.365214959	3.19E-17	postive
CUTC	STAM-AS1	0.314097944	6.55E-13	postive

CUTC	STARD7-AS1	0.485808509	5.66E-31	postive
CUTC	STK4-AS1	0.40633133	2.67E-21	postive
CUTC	STX17-AS1	0.534544817	2.71E-38	postive
CUTC	STX18-AS1	0.455717017	5.22E-27	postive
CUTC	STXBP5-AS1	0.321507605	1.74E-13	postive
CUTC	SUCLA2-AS1	0.427490293	1.24E-23	postive
CUTC	SUCLG2-AS1	0.312495734	8.68E-13	postive
CUTC	SUMO2P17	0.418990169	1.13E-22	postive
CUTC	SUZ12P1	0.319225981	2.62E-13	postive
CUTC	SVIL-AS1	0.488815112	2.16E-31	postive
CUTC	SYNPR-AS1	0.317856313	3.36E-13	postive
CUTC	TAF1A-AS1	0.411925747	6.70E-22	postive
CUTC	TAPT1-AS1	0.398907392	1.61E-20	postive
CUTC	TBC1D8-AS1	0.340212647	5.16E-15	postive
CUTC	TDRKH-AS1	0.33134913	2.81E-14	postive
CUTC	THAP7-AS1	0.333241041	1.97E-14	postive
CUTC	THAP9-AS1	0.382324332	7.53E-19	postive
CUTC	THUMPD3-AS1	0.31613391	4.56E-13	postive
CUTC	TIPARP-AS1	0.360293869	9.01E-17	postive
CUTC	TMC3-AS1	0.345667054	1.77E-15	postive
CUTC	TMCC1-AS1	0.303448323	4.14E-12	postive
CUTC	TMED2-DT	0.319922658	2.31E-13	postive
CUTC	TMEM147-AS1	0.325188521	8.86E-14	postive
CUTC	TMEM161B-AS1	0.35434455	3.08E-16	postive
CUTC	TMEM198B	0.327918904	5.35E-14	postive
CUTC	TMEM202-AS1	0.347060432	1.34E-15	postive
CUTC	TMEM254-AS1	0.308729833	1.67E-12	postive
CUTC	TMEM44-AS1	0.391254247	9.74E-20	postive
CUTC	TMEM99	0.469874157	7.97E-29	postive
CUTC	TOB1-AS1	0.470833777	5.96E-29	postive
CUTC	TP53TG1	0.368201933	1.69E-17	postive
CUTC	TP73-AS1	0.355298933	2.53E-16	postive
CUTC	TPM3P9	0.348317426	1.04E-15	postive
CUTC	TPT1-AS1	0.355555205	2.40E-16	postive
CUTC	TPTEP2	0.314674173	5.91E-13	postive
CUTC	TRAF3IP2-AS1	0.346683157	1.45E-15	postive
CUTC	TRAM2-AS1	0.524294864	1.18E-36	postive
CUTC	TRAPPC12-AS1	0.307265983	2.15E-12	postive
CUTC	TRIM52-AS1	0.521671735	3.04E-36	postive
CUTC	TSTD3	0.606574515	1.44E-51	postive
CUTC	TTC28-AS1	0.46718051	1.79E-28	postive
CUTC	TTC41P	0.348676826	9.71E-16	postive
CUTC	TTN-AS1	0.301600818	5.65E-12	postive
CUTC	TUG1	0.393444906	5.84E-20	postive
CUTC	U47924.2	0.333243633	1.97E-14	postive
CUTC	U91328.1	0.416148931	2.32E-22	postive
CUTC	UBA6-AS1	0.462071679	8.18E-28	postive
CUTC	UBAC2-AS1	0.43748918	8.60E-25	postive
CUTC	UBE2D3-AS1	0.542770972	1.19E-39	postive
CUTC	UBL7-AS1	0.37781375	2.07E-18	postive

CUTC	UBR5-AS1	0.504043505	1.42E-33	postive
CUTC	UGDH-AS1	0.409052948	1.37E-21	postive
CUTC	UPF3AP2	0.386343173	3.02E-19	postive
CUTC	URAHP	0.327994732	5.27E-14	postive
CUTC	URB1-AS1	0.360876304	7.97E-17	postive
CUTC	USP27X-AS1	0.3259129	7.75E-14	postive
CUTC	USP46-AS1	0.403700378	5.07E-21	postive
CUTC	UXT-AS1	0.344298235	2.32E-15	postive
CUTC	VASH1-AS1	0.325693191	8.07E-14	postive
CUTC	VPS13B-DT	0.436431094	1.15E-24	postive
CUTC	VPS33B-DT	0.369687778	1.22E-17	postive
CUTC	WAC-AS1	0.477636911	7.39E-30	postive
CUTC	WARS2-AS1	0.379784019	1.33E-18	postive
CUTC	WASH3P	0.423914669	3.17E-23	postive
CUTC	WDFY3-AS2	0.325088525	9.03E-14	postive
CUTC	WEE2-AS1	0.341269389	4.20E-15	postive
CUTC	WWC2-AS2	0.363818036	4.29E-17	postive
CUTC	YTHDF3-AS1	0.340286935	5.09E-15	postive
CUTC	Z83851.4	0.377825018	2.06E-18	postive
CUTC	Z95115.1	0.359662521	1.03E-16	postive
CUTC	Z97055.2	0.312463675	8.73E-13	postive
CUTC	Z97634.1	0.330432522	3.34E-14	postive
CUTC	Z99572.1	0.31185688	9.71E-13	postive
CUTC	ZBED5-AS1	0.362428526	5.76E-17	postive
CUTC	ZBTB11-AS1	0.39301499	6.46E-20	postive
CUTC	ZBTB44-DT	0.415827504	2.51E-22	postive
CUTC	ZFAS1	0.404758153	3.92E-21	postive
CUTC	ZFPM2-AS1	0.301041682	6.21E-12	postive
CUTC	ZNF205-AS1	0.330219442	3.48E-14	postive
CUTC	ZNF213-AS1	0.372227374	7.06E-18	postive
CUTC	ZNF252P	0.500596097	4.52E-33	postive
CUTC	ZNF252P-AS1	0.301795109	5.47E-12	postive
CUTC	ZNF271P	0.310510159	1.23E-12	postive
CUTC	ZNF337-AS1	0.44249092	2.19E-25	postive
CUTC	ZNF503-AS1	0.337723966	8.36E-15	postive
CUTC	ZNF503-AS2	0.461162067	1.07E-27	postive
CUTC	ZNF529-AS1	0.359277557	1.11E-16	postive
CUTC	ZNF56	0.350902596	6.20E-16	postive
CUTC	ZNF561-AS1	0.49532965	2.59E-32	postive
CUTC	ZNF658B	0.303257392	4.27E-12	postive
CUTC	ZNF687-AS1	0.359154972	1.14E-16	postive
CUTC	ZNF767P	0.315775522	4.86E-13	postive
CUTC	ZNF815P	0.306101424	2.63E-12	postive
CUTC	ZNRD2-AS1	0.406397784	2.63E-21	postive
CUTC	ZPLD2P	0.302193721	5.11E-12	postive
CUTC	ZSCAN12P1	0.305997988	2.68E-12	postive
CUTC	ZSCAN16-AS1	0.454865906	6.67E-27	postive
DBH	AC000123.1	0.313816028	6.88E-13	postive
DBH	AC002128.1	0.402557771	6.68E-21	postive
DBH	AC002347.1	0.304729479	3.33E-12	postive

DBH	AC002553.1	0.313466906	7.32E-13	postive
DBH	AC003070.1	0.367414415	2.00E-17	postive
DBH	AC003102.1	0.372500199	6.65E-18	postive
DBH	AC004148.1	0.329757559	3.79E-14	postive
DBH	AC004253.1	0.414629511	3.40E-22	postive
DBH	AC004263.1	0.330249185	3.46E-14	postive
DBH	AC004447.2	0.370615728	1.00E-17	postive
DBH	AC004466.1	0.403538844	5.27E-21	postive
DBH	AC004477.1	0.345779541	1.73E-15	postive
DBH	AC004494.1	0.658553565	1.75E-63	postive
DBH	AC004585.1	0.381234568	9.63E-19	postive
DBH	AC004687.1	0.546451025	2.87E-40	postive
DBH	AC004771.2	0.366426057	2.47E-17	postive
DBH	AC004771.4	0.400252149	1.16E-20	postive
DBH	AC004840.1	0.309802394	1.39E-12	postive
DBH	AC004846.2	0.321517945	1.73E-13	postive
DBH	AC004865.2	0.436581783	1.10E-24	postive
DBH	AC004877.1	0.30468168	3.35E-12	postive
DBH	AC004890.2	0.393431566	5.86E-20	postive
DBH	AC004893.11	0.428874825	8.64E-24	postive
DBH	AC004908.1	0.310023248	1.34E-12	postive
DBH	AC004923.4	0.302949158	4.50E-12	postive
DBH	AC005104.1	0.361866595	6.48E-17	postive
DBH	AC005154.4	0.318283811	3.11E-13	postive
DBH	AC005253.1	0.438084491	7.32E-25	postive
DBH	AC005261.3	0.331548595	2.71E-14	postive
DBH	AC005306.1	0.467692249	1.54E-28	postive
DBH	AC005332.1	0.398563864	1.74E-20	postive
DBH	AC005332.4	0.419294291	1.04E-22	postive
DBH	AC005387.1	0.307442841	2.09E-12	postive
DBH	AC005387.2	0.379877929	1.30E-18	postive
DBH	AC005393.1	0.35405062	3.27E-16	postive
DBH	AC005519.1	0.423581452	3.45E-23	postive
DBH	AC005586.1	0.30274602	4.66E-12	postive
DBH	AC005670.1	0.312712721	8.36E-13	postive
DBH	AC005674.2	0.416625134	2.05E-22	postive
DBH	AC005746.1	0.300424024	6.89E-12	postive
DBH	AC005776.2	0.365843192	2.79E-17	postive
DBH	AC005785.1	0.331022674	2.99E-14	postive
DBH	AC005840.4	0.431856911	3.92E-24	postive
DBH	AC005899.2	0.304885319	3.24E-12	postive
DBH	AC005954.1	0.407392919	2.06E-21	postive
DBH	AC006001.3	0.40209041	7.48E-21	postive
DBH	AC006033.2	0.414095142	3.89E-22	postive
DBH	AC006042.1	0.316441928	4.32E-13	postive
DBH	AC006064.1	0.372953803	6.03E-18	postive
DBH	AC006116.11	0.304338667	3.56E-12	postive
DBH	AC006160.1	0.371659741	7.99E-18	postive
DBH	AC006254.1	0.475511061	1.43E-29	postive
DBH	AC006272.1	0.337466111	8.78E-15	postive

DBH	AC006369.1	0.371592081	8.11E-18	postive
DBH	AC006435.2	0.400445103	1.11E-20	postive
DBH	AC006480.3	0.301874449	5.40E-12	postive
DBH	AC006547.1	0.384110441	5.03E-19	postive
DBH	AC006557.1	0.305411815	2.96E-12	postive
DBH	AC007066.2	0.398407499	1.81E-20	postive
DBH	AC007278.1	0.522574954	2.20E-36	postive
DBH	AC007292.1	0.372628584	6.47E-18	postive
DBH	AC007292.2	0.423244531	3.77E-23	postive
DBH	AC007308.6	0.332032664	2.47E-14	postive
DBH	AC007376.2	0.303877118	3.85E-12	postive
DBH	AC007406.1	0.317967654	3.29E-13	postive
DBH	AC007566.1	0.369617194	1.24E-17	postive
DBH	AC007569.1	0.313905611	6.77E-13	postive
DBH	AC007663.4	0.360370291	8.86E-17	postive
DBH	AC007686.3	0.496780803	1.61E-32	postive
DBH	AC007728.2	0.618620543	3.94E-54	postive
DBH	AC007728.3	0.316150711	4.55E-13	postive
DBH	AC007938.1	0.325923552	7.74E-14	postive
DBH	AC007952.6	0.34423437	2.35E-15	postive
DBH	AC008033.3	0.305353867	2.99E-12	postive
DBH	AC008083.3	0.353771519	3.46E-16	postive
DBH	AC008105.1	0.380192487	1.22E-18	postive
DBH	AC008105.3	0.486043641	5.25E-31	postive
DBH	AC008115.3	0.45429237	7.87E-27	postive
DBH	AC008124.1	0.337443956	8.82E-15	postive
DBH	AC008434.1	0.406843946	2.35E-21	postive
DBH	AC008569.2	0.308678463	1.69E-12	postive
DBH	AC008635.2	0.326609307	6.82E-14	postive
DBH	AC008676.1	0.352002291	4.96E-16	postive
DBH	AC008735.1	0.530308509	1.31E-37	postive
DBH	AC008735.2	0.473148425	2.94E-29	postive
DBH	AC008735.4	0.502765315	2.18E-33	postive
DBH	AC008737.1	0.313502633	7.27E-13	postive
DBH	AC008750.1	0.423332207	3.68E-23	postive
DBH	AC008750.5	0.431300112	4.54E-24	postive
DBH	AC008764.2	0.397611709	2.19E-20	postive
DBH	AC008764.6	0.393037646	6.43E-20	postive
DBH	AC008764.8	0.476939803	9.17E-30	postive
DBH	AC008870.3	0.389360532	1.51E-19	postive
DBH	AC008870.5	0.320605818	2.05E-13	postive
DBH	AC008894.2	0.43011181	6.23E-24	postive
DBH	AC008906.1	0.306526211	2.45E-12	postive
DBH	AC008969.1	0.393253133	6.11E-20	postive
DBH	AC008982.2	0.395783627	3.37E-20	postive
DBH	AC009019.1	0.379599644	1.39E-18	postive
DBH	AC009065.9	0.312185949	9.16E-13	postive
DBH	AC009087.1	0.344890043	2.06E-15	postive
DBH	AC009088.1	0.313533174	7.23E-13	postive
DBH	AC009093.6	0.353464298	3.69E-16	postive

DBH	AC009107.2	0.392017196	8.16E-20	postive
DBH	AC009120.2	0.398487593	1.77E-20	postive
DBH	AC009120.3	0.371256982	8.72E-18	postive
DBH	AC009121.3	0.37328528	5.61E-18	postive
DBH	AC009126.1	0.311535885	1.03E-12	postive
DBH	AC009133.3	0.581087513	1.72E-46	postive
DBH	AC009404.1	0.313520206	7.25E-13	postive
DBH	AC009690.2	0.389805425	1.36E-19	postive
DBH	AC009950.1	0.318692102	2.89E-13	postive
DBH	AC010175.1	0.414480116	3.53E-22	postive
DBH	AC010201.1	0.308334136	1.79E-12	postive
DBH	AC010201.2	0.330188996	3.50E-14	postive
DBH	AC010203.1	0.329337157	4.10E-14	postive
DBH	AC010271.1	0.42275333	4.28E-23	postive
DBH	AC010319.4	0.388854954	1.70E-19	postive
DBH	AC010326.4	0.305556289	2.89E-12	postive
DBH	AC010525.1	0.38449648	4.61E-19	postive
DBH	AC010530.1	0.372610957	6.50E-18	postive
DBH	AC010531.5	0.323291741	1.25E-13	postive
DBH	AC010542.6	0.391916208	8.35E-20	postive
DBH	AC010618.2	0.319307002	2.59E-13	postive
DBH	AC010618.3	0.356429028	2.01E-16	postive
DBH	AC010619.3	0.447872936	4.88E-26	postive
DBH	AC010761.1	0.325845217	7.85E-14	postive
DBH	AC010761.4	0.340681872	4.71E-15	postive
DBH	AC010761.6	0.302547871	4.82E-12	postive
DBH	AC010809.1	0.304924837	3.22E-12	postive
DBH	AC010864.1	0.368037265	1.75E-17	postive
DBH	AC010883.1	0.337091838	9.44E-15	postive
DBH	AC010973.1	0.431608137	4.19E-24	postive
DBH	AC010998.2	0.33777429	8.28E-15	postive
DBH	AC010999.1	0.328455885	4.84E-14	postive
DBH	AC011337.1	0.42725361	1.32E-23	postive
DBH	AC011455.1	0.395626628	3.50E-20	postive
DBH	AC011461.1	0.339163405	6.33E-15	postive
DBH	AC011462.4	0.357999347	1.45E-16	postive
DBH	AC011466.1	0.469309732	9.45E-29	postive
DBH	AC011466.3	0.348055986	1.10E-15	postive
DBH	AC011472.1	0.367587203	1.92E-17	postive
DBH	AC011476.3	0.353777264	3.46E-16	postive
DBH	AC011479.3	0.330117439	3.55E-14	postive
DBH	AC011481.1	0.38785935	2.14E-19	postive
DBH	AC011491.2	0.370780989	9.67E-18	postive
DBH	AC011498.6	0.464150503	4.42E-28	postive
DBH	AC011511.1	0.342485696	3.31E-15	postive
DBH	AC011816.2	0.374626309	4.18E-18	postive
DBH	AC011921.1	0.314487506	6.11E-13	postive
DBH	AC012181.1	0.397075179	2.48E-20	postive
DBH	AC012181.2	0.436042618	1.27E-24	postive
DBH	AC012236.1	0.305391147	2.97E-12	postive

DBH	AC012313.1	0.387976476	2.08E-19	postive
DBH	AC012313.3	0.331753785	2.61E-14	postive
DBH	AC012313.5	0.321003838	1.90E-13	postive
DBH	AC012313.6	0.30992624	1.36E-12	postive
DBH	AC012360.3	0.384581573	4.52E-19	postive
DBH	AC012435.2	0.425603541	2.04E-23	postive
DBH	AC012467.2	0.31848955	2.99E-13	postive
DBH	AC012615.4	0.418492373	1.28E-22	postive
DBH	AC012615.6	0.504880661	1.07E-33	postive
DBH	AC012645.3	0.581280867	1.58E-46	postive
DBH	AC012676.1	0.35972238	1.01E-16	postive
DBH	AC013356.2	0.300054897	7.33E-12	postive
DBH	AC013403.2	0.352500382	4.49E-16	postive
DBH	AC013460.1	0.406224581	2.74E-21	postive
DBH	AC013472.2	0.303505089	4.10E-12	postive
DBH	AC015795.1	0.380316584	1.18E-18	postive
DBH	AC015802.3	0.377602104	2.17E-18	postive
DBH	AC015802.4	0.349742062	7.84E-16	postive
DBH	AC015813.1	0.302204463	5.10E-12	postive
DBH	AC015819.1	0.385377613	3.77E-19	postive
DBH	AC015849.3	0.426871836	1.46E-23	postive
DBH	AC015871.3	0.37845151	1.79E-18	postive
DBH	AC015911.3	0.597514705	1.04E-49	postive
DBH	AC015911.6	0.388800084	1.72E-19	postive
DBH	AC016586.1	0.404198707	4.49E-21	postive
DBH	AC016722.2	0.350074848	7.33E-16	postive
DBH	AC016737.1	0.32515935	8.91E-14	postive
DBH	AC016773.2	0.357006837	1.78E-16	postive
DBH	AC016957.2	0.35795848	1.46E-16	postive
DBH	AC017083.1	0.319791932	2.37E-13	postive
DBH	AC018521.6	0.333415079	1.90E-14	postive
DBH	AC018638.6	0.32078311	1.98E-13	postive
DBH	AC018653.3	0.530717648	1.13E-37	postive
DBH	AC018690.1	0.39488531	4.17E-20	postive
DBH	AC018695.4	0.377862452	2.04E-18	postive
DBH	AC018766.1	0.339288944	6.18E-15	postive
DBH	AC018766.4	0.478506262	5.64E-30	postive
DBH	AC019205.1	0.318763611	2.85E-13	postive
DBH	AC019294.2	0.403874169	4.86E-21	postive
DBH	AC020558.2	0.404175894	4.51E-21	postive
DBH	AC020659.1	0.32273108	1.39E-13	postive
DBH	AC020907.4	0.347926507	1.13E-15	postive
DBH	AC020911.1	0.440228251	4.07E-25	postive
DBH	AC020913.2	0.36194789	6.37E-17	postive
DBH	AC020915.2	0.350370788	6.90E-16	postive
DBH	AC020931.1	0.322478572	1.46E-13	postive
DBH	AC020978.2	0.326698635	6.71E-14	postive
DBH	AC021148.1	0.300958082	6.30E-12	postive
DBH	AC021188.1	0.519414145	6.82E-36	postive
DBH	AC021205.3	0.357030252	1.77E-16	postive

DBH	AC021739.2	0.313360945	7.46E-13	postive
DBH	AC021739.3	0.304316833	3.57E-12	postive
DBH	AC022098.1	0.485970297	5.37E-31	postive
DBH	AC022150.2	0.31795718	3.29E-13	postive
DBH	AC022154.1	0.413674249	4.32E-22	postive
DBH	AC022167.2	0.309658285	1.42E-12	postive
DBH	AC022182.2	0.381001859	1.01E-18	postive
DBH	AC022296.4	0.303015078	4.45E-12	postive
DBH	AC022392.1	0.326079218	7.52E-14	postive
DBH	AC022400.1	0.39385427	5.31E-20	postive
DBH	AC022400.5	0.406487995	2.57E-21	postive
DBH	AC022400.6	0.320134893	2.23E-13	postive
DBH	AC022558.1	0.302997185	4.46E-12	postive
DBH	AC022558.3	0.354247747	3.14E-16	postive
DBH	AC022762.2	0.303854432	3.86E-12	postive
DBH	AC022973.5	0.309402178	1.49E-12	postive
DBH	AC023355.2	0.474845381	1.75E-29	postive
DBH	AC023825.2	0.356693626	1.90E-16	postive
DBH	AC023908.3	0.477279638	8.26E-30	postive
DBH	AC024060.2	0.338569406	7.10E-15	postive
DBH	AC024075.1	0.339649591	5.76E-15	postive
DBH	AC024075.2	0.325202062	8.84E-14	postive
DBH	AC024257.3	0.340290581	5.08E-15	postive
DBH	AC024267.4	0.425273161	2.22E-23	postive
DBH	AC024361.3	0.36878383	1.49E-17	postive
DBH	AC025048.2	0.369525876	1.27E-17	postive
DBH	AC025164.2	0.438111173	7.26E-25	postive
DBH	AC025165.1	0.317929914	3.31E-13	postive
DBH	AC025176.1	0.31165023	1.01E-12	postive
DBH	AC025279.1	0.378399116	1.81E-18	postive
DBH	AC025287.3	0.45141098	1.79E-26	postive
DBH	AC026367.2	0.351975424	4.99E-16	postive
DBH	AC026471.4	0.345925662	1.68E-15	postive
DBH	AC027020.1	0.3445756	2.20E-15	postive
DBH	AC027020.2	0.378063334	1.96E-18	postive
DBH	AC027601.2	0.573059235	5.55E-45	postive
DBH	AC027601.3	0.467438254	1.66E-28	postive
DBH	AC027682.1	0.339754476	5.64E-15	postive
DBH	AC027682.4	0.317305032	3.70E-13	postive
DBH	AC027796.4	0.326353735	7.15E-14	postive
DBH	AC032044.1	0.314858357	5.72E-13	postive
DBH	AC034102.6	0.457399561	3.21E-27	postive
DBH	AC034236.2	0.390590939	1.14E-19	postive
DBH	AC034238.2	0.557745846	3.23E-42	postive
DBH	AC036108.3	0.317027135	3.89E-13	postive
DBH	AC037459.2	0.429983071	6.45E-24	postive
DBH	AC037487.4	0.355789373	2.29E-16	postive
DBH	AC040162.3	0.393705746	5.50E-20	postive
DBH	AC044849.1	0.328067414	5.20E-14	postive
DBH	AC048341.2	0.316596688	4.20E-13	postive

DBH	AC048382.2	0.305983993	2.68E-12	postive
DBH	AC055855.2	0.392997219	6.49E-20	postive
DBH	AC060234.2	0.384889936	4.21E-19	postive
DBH	AC060780.1	0.377072878	2.44E-18	postive
DBH	AC062037.3	0.339225765	6.25E-15	postive
DBH	AC066613.1	0.408751474	1.47E-21	postive
DBH	AC067750.1	0.368001966	1.76E-17	postive
DBH	AC067852.3	0.398583101	1.73E-20	postive
DBH	AC067930.3	0.34782372	1.15E-15	postive
DBH	AC067945.3	0.436504805	1.12E-24	postive
DBH	AC068580.1	0.331888154	2.54E-14	postive
DBH	AC068580.3	0.313170743	7.71E-13	postive
DBH	AC068870.1	0.321131865	1.86E-13	postive
DBH	AC069209.1	0.361016203	7.74E-17	postive
DBH	AC073111.1	0.413509067	4.51E-22	postive
DBH	AC073517.1	0.310944459	1.14E-12	postive
DBH	AC073575.4	0.405350164	3.39E-21	postive
DBH	AC073655.2	0.302619726	4.76E-12	postive
DBH	AC073842.2	0.36052786	8.58E-17	postive
DBH	AC073869.1	0.345378045	1.87E-15	postive
DBH	AC073957.3	0.330441394	3.34E-14	postive
DBH	AC074029.3	0.372425823	6.76E-18	postive
DBH	AC074117.1	0.316453782	4.31E-13	postive
DBH	AC078788.2	0.316170542	4.53E-13	postive
DBH	AC078846.1	0.342208109	3.50E-15	postive
DBH	AC079015.1	0.301531561	5.72E-12	postive
DBH	AC079174.2	0.36234133	5.86E-17	postive
DBH	AC079322.1	0.413761081	4.23E-22	postive
DBH	AC079336.5	0.307293409	2.14E-12	postive
DBH	AC079340.2	0.366962603	2.20E-17	postive
DBH	AC079584.2	0.360117925	9.34E-17	postive
DBH	AC079807.1	0.314131284	6.51E-13	postive
DBH	AC083862.2	0.459105024	1.95E-27	postive
DBH	AC084018.1	0.527104719	4.25E-37	postive
DBH	AC084018.2	0.493519215	4.70E-32	postive
DBH	AC084125.2	0.340166654	5.21E-15	postive
DBH	AC084781.1	0.318559199	2.96E-13	postive
DBH	AC084876.1	0.350524166	6.69E-16	postive
DBH	AC087164.1	0.345244759	1.92E-15	postive
DBH	AC087289.1	0.345629918	1.78E-15	postive
DBH	AC087289.2	0.488686788	2.25E-31	postive
DBH	AC087301.1	0.348856933	9.36E-16	postive
DBH	AC087392.4	0.330076767	3.57E-14	postive
DBH	AC087500.2	0.301893628	5.38E-12	postive
DBH	AC087741.1	0.412542748	5.74E-22	postive
DBH	AC090004.1	0.368339622	1.64E-17	postive
DBH	AC090260.2	0.354393115	3.05E-16	postive
DBH	AC090510.2	0.325464643	8.42E-14	postive
DBH	AC090559.1	0.317944347	3.30E-13	postive
DBH	AC090587.2	0.381080244	9.97E-19	postive

DBH	AC090589.3	0.399945913	1.25E-20	postive
DBH	AC090825.1	0.338163782	7.68E-15	postive
DBH	AC090948.2	0.382763003	6.82E-19	postive
DBH	AC090948.3	0.500832345	4.18E-33	postive
DBH	AC091057.1	0.394056413	5.06E-20	postive
DBH	AC091132.2	0.317651779	3.48E-13	postive
DBH	AC091180.3	0.397779445	2.10E-20	postive
DBH	AC091180.5	0.38837484	1.90E-19	postive
DBH	AC091180.6	0.475177588	1.58E-29	postive
DBH	AC091185.1	0.327063142	6.27E-14	postive
DBH	AC092118.2	0.391120062	1.01E-19	postive
DBH	AC092119.2	0.416374195	2.19E-22	postive
DBH	AC092123.1	0.342088784	3.58E-15	postive
DBH	AC092145.1	0.364426172	3.77E-17	postive
DBH	AC092295.1	0.302995872	4.47E-12	postive
DBH	AC092301.1	0.337138795	9.35E-15	postive
DBH	AC092375.2	0.38005092	1.26E-18	postive
DBH	AC092384.1	0.307094427	2.22E-12	postive
DBH	AC092384.2	0.330837193	3.10E-14	postive
DBH	AC092580.2	0.461055428	1.10E-27	postive
DBH	AC092611.1	0.348058418	1.10E-15	postive
DBH	AC092910.3	0.354262451	3.13E-16	postive
DBH	AC093010.2	0.318817146	2.82E-13	postive
DBH	AC093110.1	0.318466009	3.01E-13	postive
DBH	AC093278.2	0.347835838	1.15E-15	postive
DBH	AC093484.2	0.355309198	2.53E-16	postive
DBH	AC093495.1	0.368357528	1.63E-17	postive
DBH	AC093642.1	0.409039317	1.37E-21	postive
DBH	AC093726.2	0.350967729	6.12E-16	postive
DBH	AC093752.1	0.336793601	9.99E-15	postive
DBH	AC093752.3	0.407688983	1.91E-21	postive
DBH	AC096642.1	0.352833456	4.19E-16	postive
DBH	AC096734.1	0.447041909	6.16E-26	postive
DBH	AC096992.2	0.350212108	7.13E-16	postive
DBH	AC097382.2	0.377138799	2.40E-18	postive
DBH	AC097641.2	0.437181904	9.35E-25	postive
DBH	AC098479.1	0.317591869	3.52E-13	postive
DBH	AC099343.3	0.411287431	7.85E-22	postive
DBH	AC099524.1	0.409453895	1.24E-21	postive
DBH	AC099568.1	0.337121344	9.38E-15	postive
DBH	AC099778.1	0.37378775	5.02E-18	postive
DBH	AC100778.2	0.314146653	6.49E-13	postive
DBH	AC100778.3	0.302295547	5.03E-12	postive
DBH	AC103810.1	0.363799772	4.31E-17	postive
DBH	AC103858.1	0.450052413	2.64E-26	postive
DBH	AC104078.1	0.336418803	1.07E-14	postive
DBH	AC104109.4	0.307270617	2.15E-12	postive
DBH	AC104532.2	0.402662562	6.51E-21	postive
DBH	AC104564.3	0.447496411	5.42E-26	postive
DBH	AC104758.1	0.329427738	4.04E-14	postive

DBH	AC105020.5	0.363127241	4.97E-17	postive
DBH	AC105206.2	0.347507957	1.23E-15	postive
DBH	AC105219.1	0.358533816	1.30E-16	postive
DBH	AC105345.1	0.315401915	5.20E-13	postive
DBH	AC107081.2	0.30272575	4.67E-12	postive
DBH	AC107375.1	0.371614126	8.07E-18	postive
DBH	AC107959.1	0.365448389	3.04E-17	postive
DBH	AC108010.1	0.464771546	3.68E-28	postive
DBH	AC108134.3	0.47398626	2.28E-29	postive
DBH	AC108488.1	0.317597196	3.51E-13	postive
DBH	AC109460.1	0.445918802	8.43E-26	postive
DBH	AC109460.2	0.493366953	4.94E-32	postive
DBH	AC110079.1	0.439569448	4.88E-25	postive
DBH	AC112694.1	0.322914366	1.34E-13	postive
DBH	AC112907.3	0.370068127	1.13E-17	postive
DBH	AC114490.1	0.491481588	9.12E-32	postive
DBH	AC114730.2	0.379669633	1.37E-18	postive
DBH	AC114730.3	0.393926063	5.22E-20	postive
DBH	AC116348.5	0.30323005	4.29E-12	postive
DBH	AC116366.2	0.585704876	2.24E-47	postive
DBH	AC116535.2	0.323928979	1.12E-13	postive
DBH	AC116552.1	0.314706725	5.88E-13	postive
DBH	AC116667.1	0.369701688	1.22E-17	postive
DBH	AC116913.1	0.375919166	3.14E-18	postive
DBH	AC116914.1	0.565399578	1.40E-43	postive
DBH	AC116914.2	0.566491749	8.86E-44	postive
DBH	AC118344.1	0.340003852	5.38E-15	postive
DBH	AC119044.1	0.332292377	2.36E-14	postive
DBH	AC120498.6	0.31661758	4.19E-13	postive
DBH	AC120498.9	0.350610093	6.58E-16	postive
DBH	AC122108.3	0.31310966	7.79E-13	postive
DBH	AC122129.1	0.348845948	9.38E-16	postive
DBH	AC123768.3	0.307639051	2.02E-12	postive
DBH	AC124319.1	0.349920597	7.56E-16	postive
DBH	AC124944.2	0.324989917	9.19E-14	postive
DBH	AC125494.1	0.375690104	3.31E-18	postive
DBH	AC126118.1	0.36243581	5.75E-17	postive
DBH	AC126755.1	0.390253054	1.23E-19	postive
DBH	AC127024.4	0.32349777	1.21E-13	postive
DBH	AC127502.1	0.402018047	7.61E-21	postive
DBH	AC127502.2	0.44689823	6.41E-26	postive
DBH	AC129510.1	0.406123004	2.81E-21	postive
DBH	AC130462.3	0.41936007	1.02E-22	postive
DBH	AC131097.3	0.301898304	5.38E-12	postive
DBH	AC132008.2	0.331433121	2.77E-14	postive
DBH	AC132192.2	0.345086942	1.98E-15	postive
DBH	AC132872.1	0.354294834	3.11E-16	postive
DBH	AC132872.2	0.344032589	2.44E-15	postive
DBH	AC132872.3	0.41441221	3.59E-22	postive
DBH	AC132938.3	0.344458911	2.25E-15	postive

DBH	AC133550.3	0.379221008	1.51E-18	postive
DBH	AC133919.2	0.318219803	3.14E-13	postive
DBH	AC135050.3	0.447447398	5.50E-26	postive
DBH	AC135050.5	0.323970918	1.11E-13	postive
DBH	AC135782.3	0.382070526	7.98E-19	postive
DBH	AC135983.3	0.38854587	1.82E-19	postive
DBH	AC136475.2	0.334282282	1.62E-14	postive
DBH	AC136475.3	0.307525681	2.06E-12	postive
DBH	AC136604.2	0.321478511	1.75E-13	postive
DBH	AC137932.3	0.359959036	9.66E-17	postive
DBH	AC138028.2	0.368984806	1.43E-17	postive
DBH	AC138028.3	0.343538072	2.69E-15	postive
DBH	AC138028.4	0.501065168	3.86E-33	postive
DBH	AC138035.1	0.389096125	1.61E-19	postive
DBH	AC138207.1	0.431776516	4.00E-24	postive
DBH	AC138207.4	0.346725255	1.43E-15	postive
DBH	AC138207.5	0.303916186	3.82E-12	postive
DBH	AC138207.6	0.348160846	1.08E-15	postive
DBH	AC138932.1	0.416210851	2.28E-22	postive
DBH	AC138956.1	0.360239543	9.11E-17	postive
DBH	AC138956.2	0.377662548	2.14E-18	postive
DBH	AC139100.2	0.321913205	1.61E-13	postive
DBH	AC139256.2	0.307968421	1.91E-12	postive
DBH	AC139256.3	0.317083076	3.85E-13	postive
DBH	AC139795.1	0.335730243	1.23E-14	postive
DBH	AC139795.2	0.377588533	2.17E-18	postive
DBH	AC139887.2	0.304002176	3.76E-12	postive
DBH	AC141424.1	0.410248561	1.02E-21	postive
DBH	AC142472.1	0.319087053	2.69E-13	postive
DBH	AC145098.1	0.40817334	1.70E-21	postive
DBH	AC145285.3	0.386295872	3.06E-19	postive
DBH	AC145285.6	0.422642448	4.40E-23	postive
DBH	AC145423.3	0.315280262	5.31E-13	postive
DBH	AC232271.1	0.469303654	9.46E-29	postive
DBH	AC233728.1	0.390096566	1.27E-19	postive
DBH	AC239727.1	0.313481527	7.30E-13	postive
DBH	AC241952.2	0.379673338	1.37E-18	postive
DBH	AC242376.2	0.316542085	4.24E-13	postive
DBH	AC243829.1	0.447420046	5.54E-26	postive
DBH	AC243829.2	0.371427928	8.40E-18	postive
DBH	AC243960.1	0.45912364	1.94E-27	postive
DBH	AC244034.2	0.330673628	3.20E-14	postive
DBH	AC244100.1	0.310744368	1.18E-12	postive
DBH	AC244197.2	0.309150054	1.56E-12	postive
DBH	AC245052.4	0.437724472	8.07E-25	postive
DBH	AC245060.2	0.358178465	1.40E-16	postive
DBH	AC245060.6	0.4265928	1.58E-23	postive
DBH	AC245140.2	0.37086414	9.50E-18	postive
DBH	AC245452.1	0.338377195	7.37E-15	postive
DBH	AC245884.8	0.392495262	7.30E-20	postive

DBH	ACTG1P17	0.351204368	5.83E-16	postive
DBH	AD000671.3	0.339139985	6.36E-15	postive
DBH	ADGRE4P	0.333861064	1.75E-14	postive
DBH	ADIRF-AS1	0.359669816	1.03E-16	postive
DBH	ADPGK-AS1	0.366719123	2.32E-17	postive
DBH	AF111169.3	0.530465251	1.24E-37	postive
DBH	AF117829.1	0.306637479	2.40E-12	postive
DBH	AF127936.5	0.44623564	7.72E-26	postive
DBH	AFG3L1P	0.468954808	1.05E-28	postive
DBH	AGBL5-IT1	0.346274146	1.57E-15	postive
DBH	AHSA2P	0.434221886	2.08E-24	postive
DBH	AL008582.1	0.310426628	1.25E-12	postive
DBH	AL008727.1	0.35388404	3.38E-16	postive
DBH	AL021368.2	0.321819411	1.64E-13	postive
DBH	AL021707.3	0.43428756	2.04E-24	postive
DBH	AL021707.6	0.320653079	2.03E-13	postive
DBH	AL021707.7	0.366388522	2.49E-17	postive
DBH	AL021707.8	0.407407585	2.05E-21	postive
DBH	AL021937.1	0.378952441	1.60E-18	postive
DBH	AL021937.4	0.326104719	7.48E-14	postive
DBH	AL022238.3	0.360114084	9.35E-17	postive
DBH	AL022328.1	0.408015216	1.76E-21	postive
DBH	AL022328.2	0.428560452	9.39E-24	postive
DBH	AL022328.3	0.482007671	1.89E-30	postive
DBH	AL023653.1	0.461135727	1.08E-27	postive
DBH	AL031186.1	0.362700276	5.44E-17	postive
DBH	AL031282.2	0.334173341	1.65E-14	postive
DBH	AL031320.2	0.333812199	1.77E-14	postive
DBH	AL031595.2	0.338023753	7.89E-15	postive
DBH	AL031600.1	0.511708104	1.02E-34	postive
DBH	AL031670.1	0.394689419	4.36E-20	postive
DBH	AL031709.1	0.405741468	3.08E-21	postive
DBH	AL031714.1	0.403473506	5.35E-21	postive
DBH	AL031717.1	0.353562796	3.61E-16	postive
DBH	AL031846.1	0.50801722	3.66E-34	postive
DBH	AL031846.2	0.513590224	5.32E-35	postive
DBH	AL032821.1	0.396092521	3.13E-20	postive
DBH	AL034550.1	0.325419963	8.49E-14	postive
DBH	AL035587.1	0.379755414	1.34E-18	postive
DBH	AL049780.1	0.318772448	2.85E-13	postive
DBH	AL049795.1	0.351461601	5.54E-16	postive
DBH	AL049840.2	0.379374947	1.46E-18	postive
DBH	AL049840.4	0.4259909	1.84E-23	postive
DBH	AL049840.5	0.442936547	1.93E-25	postive
DBH	AL049840.6	0.412684563	5.54E-22	postive
DBH	AL109811.1	0.376525231	2.75E-18	postive
DBH	AL109811.2	0.373343222	5.54E-18	postive
DBH	AL109955.1	0.448840089	3.71E-26	postive
DBH	AL117209.1	0.364405212	3.79E-17	postive
DBH	AL117379.1	0.329183943	4.22E-14	postive

DBH	AL118558.1	0.348614265	9.83E-16	postive
DBH	AL121748.1	0.313716469	7.00E-13	postive
DBH	AL121845.4	0.390179225	1.25E-19	postive
DBH	AL121985.1	0.42359661	3.44E-23	postive
DBH	AL121987.2	0.397326585	2.34E-20	postive
DBH	AL121992.1	0.35514928	2.61E-16	postive
DBH	AL122035.2	0.39658002	2.79E-20	postive
DBH	AL132642.1	0.61265226	7.57E-53	postive
DBH	AL132989.1	0.486412199	4.67E-31	postive
DBH	AL133215.1	0.353714046	3.50E-16	postive
DBH	AL133367.1	0.361523478	6.96E-17	postive
DBH	AL133406.2	0.433644003	2.43E-24	postive
DBH	AL133410.1	0.30947036	1.47E-12	postive
DBH	AL133467.1	0.323336	1.24E-13	postive
DBH	AL135818.1	0.367552871	1.94E-17	postive
DBH	AL135999.1	0.410034713	1.07E-21	postive
DBH	AL136295.2	0.459949605	1.53E-27	postive
DBH	AL136295.7	0.349126305	8.87E-16	postive
DBH	AL136368.1	0.470753393	6.10E-29	postive
DBH	AL137003.1	0.337045659	9.52E-15	postive
DBH	AL137186.1	0.313383479	7.43E-13	postive
DBH	AL138921.1	0.345998959	1.66E-15	postive
DBH	AL139011.1	0.387390359	2.38E-19	postive
DBH	AL139021.3	0.32864064	4.67E-14	postive
DBH	AL139123.1	0.355396185	2.48E-16	postive
DBH	AL139287.1	0.370086591	1.12E-17	postive
DBH	AL139349.1	0.367683981	1.88E-17	postive
DBH	AL139352.1	0.389281542	1.54E-19	postive
DBH	AL157392.3	0.401169353	9.34E-21	postive
DBH	AL157756.1	0.311630938	1.01E-12	postive
DBH	AL157871.2	0.439103937	5.54E-25	postive
DBH	AL157871.5	0.318550676	2.96E-13	postive
DBH	AL157935.3	0.572120775	8.28E-45	postive
DBH	AL158071.4	0.363790696	4.32E-17	postive
DBH	AL158211.1	0.301030726	6.22E-12	postive
DBH	AL158212.3	0.398901377	1.61E-20	postive
DBH	AL158835.2	0.358643362	1.27E-16	postive
DBH	AL161452.1	0.351284014	5.74E-16	postive
DBH	AL161725.1	0.327343464	5.95E-14	postive
DBH	AL161756.1	0.340393499	4.98E-15	postive
DBH	AL162258.2	0.332573082	2.23E-14	postive
DBH	AL162414.1	0.308857749	1.64E-12	postive
DBH	AL162424.1	0.329541138	3.95E-14	postive
DBH	AL162586.1	0.394466566	4.60E-20	postive
DBH	AL162727.2	0.302111846	5.19E-12	postive
DBH	AL162741.1	0.417029344	1.85E-22	postive
DBH	AL353597.1	0.320574346	2.06E-13	postive
DBH	AL353622.1	0.346416866	1.52E-15	postive
DBH	AL353625.1	0.328629223	4.68E-14	postive
DBH	AL353796.1	0.333650225	1.82E-14	postive

DBH	AL354733.3	0.464537344	3.94E-28	postive
DBH	AL354809.1	0.300488951	6.81E-12	postive
DBH	AL354892.3	0.404587958	4.08E-21	postive
DBH	AL355075.2	0.36839542	1.62E-17	postive
DBH	AL355388.1	0.380846747	1.05E-18	postive
DBH	AL355574.1	0.371334759	8.58E-18	postive
DBH	AL355916.2	0.33165368	2.66E-14	postive
DBH	AL356481.1	0.311086087	1.11E-12	postive
DBH	AL356481.3	0.39170334	8.78E-20	postive
DBH	AL359091.3	0.329825127	3.75E-14	postive
DBH	AL359532.1	0.343682432	2.62E-15	postive
DBH	AL359878.1	0.357603674	1.58E-16	postive
DBH	AL359921.1	0.323036092	1.31E-13	postive
DBH	AL360181.1	0.365131416	3.25E-17	postive
DBH	AL360181.2	0.398315956	1.85E-20	postive
DBH	AL365330.1	0.338405551	7.33E-15	postive
DBH	AL390066.2	0.43917906	5.43E-25	postive
DBH	AL390728.4	0.438233263	7.03E-25	postive
DBH	AL391244.2	0.311494188	1.03E-12	postive
DBH	AL391684.1	0.40831805	1.64E-21	postive
DBH	AL442128.2	0.366041238	2.68E-17	postive
DBH	AL445931.1	0.329179724	4.23E-14	postive
DBH	AL450263.1	0.387638933	2.25E-19	postive
DBH	AL450384.2	0.321251549	1.82E-13	postive
DBH	AL450998.3	0.430877397	5.08E-24	postive
DBH	AL451050.2	0.319415943	2.54E-13	postive
DBH	AL451067.1	0.306015806	2.67E-12	postive
DBH	AL512303.1	0.30367642	3.98E-12	postive
DBH	AL512306.2	0.344100487	2.41E-15	postive
DBH	AL512306.3	0.383953309	5.21E-19	postive
DBH	AL512631.1	0.327092669	6.23E-14	postive
DBH	AL512652.1	0.311029323	1.12E-12	postive
DBH	AL512770.1	0.427932197	1.11E-23	postive
DBH	AL512791.1	0.448680578	3.89E-26	postive
DBH	AL513165.1	0.373123096	5.81E-18	postive
DBH	AL513190.1	0.303511278	4.09E-12	postive
DBH	AL513320.1	0.398820683	1.64E-20	postive
DBH	AL583810.2	0.307159975	2.19E-12	postive
DBH	AL590133.1	0.348112434	1.09E-15	postive
DBH	AL592164.1	0.403394998	5.46E-21	postive
DBH	AL592211.1	0.348942459	9.20E-16	postive
DBH	AL596202.1	0.322523554	1.44E-13	postive
DBH	AL603756.1	0.317731045	3.43E-13	postive
DBH	AL603832.1	0.406745435	2.41E-21	postive
DBH	AL606834.2	0.310488756	1.23E-12	postive
DBH	AL627309.5	0.335098163	1.38E-14	postive
DBH	AL645568.1	0.30327198	4.26E-12	postive
DBH	AL645941.1	0.379483872	1.43E-18	postive
DBH	AL662844.3	0.497273596	1.37E-32	postive
DBH	AL662844.4	0.48734248	3.47E-31	postive

DBH	AL662884.1	0.37649405	2.77E-18	postive
DBH	AL669831.1	0.389149322	1.59E-19	postive
DBH	AL671710.1	0.31237286	8.87E-13	postive
DBH	AL691432.1	0.384731504	4.37E-19	postive
DBH	AL731563.4	0.365569959	2.96E-17	postive
DBH	AL731566.2	0.321462921	1.75E-13	postive
DBH	AL731567.1	0.41025786	1.01E-21	postive
DBH	AL731569.1	0.408674112	1.50E-21	postive
DBH	AL731571.1	0.339961301	5.42E-15	postive
DBH	AL732372.2	0.313229046	7.63E-13	postive
DBH	AL928654.2	0.399269094	1.47E-20	postive
DBH	ALOX12-AS1	0.352457112	4.53E-16	postive
DBH	ANKRD36BP2	0.360440894	8.73E-17	postive
DBH	AP000346.2	0.301270479	5.97E-12	postive
DBH	AP000347.1	0.362521417	5.64E-17	postive
DBH	AP000442.1	0.355229339	2.57E-16	postive
DBH	AP000487.1	0.306044256	2.66E-12	postive
DBH	AP000692.1	0.3994473	1.41E-20	postive
DBH	AP000763.3	0.425660533	2.01E-23	postive
DBH	AP000845.1	0.313705691	7.02E-13	postive
DBH	AP000873.2	0.368981336	1.43E-17	postive
DBH	AP000873.4	0.418245725	1.36E-22	postive
DBH	AP001029.1	0.333945467	1.72E-14	postive
DBH	AP001029.2	0.31014168	1.31E-12	postive
DBH	AP001056.1	0.311607564	1.01E-12	postive
DBH	AP001062.1	0.392529309	7.24E-20	postive
DBH	AP001107.1	0.365134539	3.25E-17	postive
DBH	AP001107.4	0.378910231	1.62E-18	postive
DBH	AP001107.6	0.332887269	2.11E-14	postive
DBH	AP001160.1	0.329796094	3.77E-14	postive
DBH	AP001432.1	0.319205495	2.63E-13	postive
DBH	AP001458.1	0.378573206	1.75E-18	postive
DBH	AP001462.1	0.329495313	3.99E-14	postive
DBH	AP001469.2	0.312320106	8.95E-13	postive
DBH	AP001619.1	0.348329248	1.04E-15	postive
DBH	AP001625.2	0.322119076	1.55E-13	postive
DBH	AP001628.1	0.330476406	3.32E-14	postive
DBH	AP001767.3	0.350027025	7.40E-16	postive
DBH	AP001793.1	0.368117369	1.72E-17	postive
DBH	AP001893.1	0.333764031	1.78E-14	postive
DBH	AP002340.1	0.386706053	2.78E-19	postive
DBH	AP002490.1	0.371919286	7.55E-18	postive
DBH	AP002807.1	0.380380324	1.17E-18	postive
DBH	AP002812.2	0.313940385	6.73E-13	postive
DBH	AP003096.1	0.451707822	1.65E-26	postive
DBH	AP003392.1	0.323778711	1.15E-13	postive
DBH	AP003486.1	0.316192416	4.52E-13	postive
DBH	AP003557.1	0.420115525	8.44E-23	postive
DBH	AP006621.2	0.424932799	2.43E-23	postive
DBH	AP006621.4	0.345652379	1.77E-15	postive

DBH	AP006623.1	0.45084268	2.11E-26	postive
DBH	AP4B1-AS1	0.431471967	4.34E-24	postive
DBH	ARRDC1-AS1	0.317331681	3.69E-13	postive
DBH	ASB16-AS1	0.401728495	8.16E-21	postive
DBH	ASMTL-AS1	0.368047904	1.74E-17	postive
DBH	ATXN1-AS1	0.34342966	2.75E-15	postive
DBH	BCDIN3D-AS1	0.344275277	2.33E-15	postive
DBH	BHLHE40-AS1	0.315138528	5.45E-13	postive
DBH	BMS1P4	0.360842914	8.03E-17	postive
DBH	BOLA2P2	0.345575683	1.80E-15	postive
DBH	BTN2A3P	0.417903074	1.49E-22	postive
DBH	BX255923.2	0.328241171	5.04E-14	postive
DBH	C1orf147	0.446333595	7.51E-26	postive
DBH	C1orf220	0.459591175	1.69E-27	postive
DBH	C1RL-AS1	0.496534727	1.74E-32	postive
DBH	C22orf34	0.300606031	6.68E-12	postive
DBH	C2-AS1	0.374969207	3.88E-18	postive
DBH	C5orf34-AS1	0.31183723	9.74E-13	postive
DBH	C9orf139	0.641832177	2.13E-59	postive
DBH	CACNA1C-AS1	0.343177923	2.89E-15	postive
DBH	CACTIN-AS1	0.381686053	8.70E-19	postive
DBH	CAHM	0.314818037	5.76E-13	postive
DBH	CAPN10-DT	0.364769039	3.51E-17	postive
DBH	CARD11-AS1	0.373725795	5.09E-18	postive
DBH	CASP16P	0.318786212	2.84E-13	postive
DBH	CASP17P	0.340629383	4.76E-15	postive
DBH	CBX3P4	0.336230305	1.11E-14	postive
DBH	CCDC183-AS1	0.305769077	2.79E-12	postive
DBH	CCDC18-AS1	0.327630195	5.64E-14	postive
DBH	CCT6P1	0.312197563	9.15E-13	postive
DBH	CD27-AS1	0.300155456	7.20E-12	postive
DBH	CEP250-AS1	0.470666144	6.27E-29	postive
DBH	CHKB-CPT1B	0.344145929	2.39E-15	postive
DBH	CHRM3-AS2	0.668256559	5.63E-66	postive
DBH	CLUHP3	0.316566086	4.22E-13	postive
DBH	COL6A4P2	0.331208112	2.89E-14	postive
DBH	COPDA1	0.306214046	2.58E-12	postive
DBH	CR559946.1	0.333556726	1.85E-14	postive
DBH	CR559946.2	0.322977383	1.33E-13	postive
DBH	CR936218.1	0.311631177	1.01E-12	postive
DBH	CROCCP2	0.358227882	1.38E-16	postive
DBH	CROCCP3	0.367660112	1.89E-17	postive
DBH	CSNK1G2-AS1	0.403706828	5.06E-21	postive
DBH	CTBP1-AS	0.446467579	7.23E-26	postive
DBH	CTC-487M23.5	0.321937521	1.61E-13	postive
DBH	CTD-2574D22.4	0.371207564	8.82E-18	postive
DBH	CUTALP	0.343222028	2.87E-15	postive
DBH	CXCR2P1	0.467756253	1.51E-28	postive
DBH	CXXC5-AS1	0.331661696	2.65E-14	postive
DBH	DBH-AS1	0.894923463	1.19E-176	postive

DBH	DICER1-AS1	0.310234665	1.29E-12	postive
DBH	DLGAP4-AS1	0.304211262	3.63E-12	postive
DBH	DNM1P46	0.331146075	2.92E-14	postive
DBH	DNM1P47	0.346012912	1.65E-15	postive
DBH	DPP9-AS1	0.480846409	2.72E-30	postive
DBH	DSCR9	0.306561749	2.43E-12	postive
DBH	EML4-AS1	0.305287651	3.02E-12	postive
DBH	EP400P1	0.389645474	1.42E-19	postive
DBH	ERVK13-1	0.345547517	1.81E-15	postive
DBH	FAM157C	0.354582041	2.93E-16	postive
DBH	FAM21EP	0.305325477	3.00E-12	postive
DBH	FAM225A	0.321505491	1.74E-13	postive
DBH	FAM238A	0.500467552	4.72E-33	postive
DBH	FAM30A	0.476099691	1.19E-29	postive
DBH	FAM86HP	0.331220395	2.88E-14	postive
DBH	FBXL19-AS1	0.330907458	3.06E-14	postive
DBH	FER1L4	0.335556514	1.27E-14	postive
DBH	FMR1-IT1	0.356135669	2.13E-16	postive
DBH	GABPB1-AS1	0.331856503	2.56E-14	postive
DBH	GARS-DT	0.39138383	9.45E-20	postive
DBH	GEMIN7-AS1	0.315416682	5.18E-13	postive
DBH	GHRLOS	0.366070586	2.66E-17	postive
DBH	GLUD1P3	0.396579433	2.79E-20	postive
DBH	GOLGA2P5	0.397286711	2.36E-20	postive
DBH	GRPEL2-AS1	0.305924271	2.71E-12	postive
DBH	GS1-124K5.3	0.394330309	4.75E-20	postive
DBH	GTF2IP20	0.32140983	1.77E-13	postive
DBH	GUSBP11	0.326977158	6.37E-14	postive
DBH	GVINP1	0.516879483	1.67E-35	postive
DBH	H1FX-AS1	0.3035391	4.07E-12	postive
DBH	H2BP2	0.360274126	9.04E-17	postive
DBH	H3P37	0.327145043	6.17E-14	postive
DBH	HCG25	0.329967555	3.65E-14	postive
DBH	HCG27	0.505949842	7.42E-34	postive
DBH	HERC2P2	0.374476269	4.32E-18	postive
DBH	HERC2P9	0.358424839	1.33E-16	postive
DBH	HEXD-IT1	0.454067614	8.39E-27	postive
DBH	HLA-DQB1-AS1	0.365767948	2.84E-17	postive
DBH	HM13-IT1	0.339016622	6.51E-15	postive
DBH	HSD17B7P2	0.330901957	3.06E-14	postive
DBH	IATPR	0.309663923	1.42E-12	postive
DBH	IBA57-DT	0.335974183	1.17E-14	postive
DBH	IGBP1-AS1	0.413988909	4.00E-22	postive
DBH	IL21-AS1	0.491654655	8.62E-32	postive
DBH	IL21R-AS1	0.56784808	5.03E-44	postive
DBH	INE1	0.413375052	4.66E-22	postive
DBH	INHCAP	0.30057922	6.71E-12	postive
DBH	INTS6-AS1	0.319642666	2.43E-13	postive
DBH	IRF1-AS1	0.586958038	1.28E-47	postive
DBH	ITGB2-AS1	0.34911263	8.89E-16	postive

DBH	KB-1507C5.4	0.301501795	5.75E-12	postive
DBH	KCNIP2-AS1	0.325001811	9.17E-14	postive
DBH	KDM2B-DT	0.341664161	3.89E-15	postive
DBH	KDM4A-AS1	0.343042109	2.97E-15	postive
DBH	KIF1C-AS1	0.441225692	3.10E-25	postive
DBH	KLRA1P	0.362671944	5.47E-17	postive
DBH	LCT-AS1	0.302898306	4.54E-12	postive
DBH	LENG8-AS1	0.433273724	2.68E-24	postive
DBH	LINC00106	0.438835871	5.96E-25	postive
DBH	LINC00115	0.363408571	4.68E-17	postive
DBH	LINC00158	0.387814902	2.16E-19	postive
DBH	LINC00173	0.361947898	6.37E-17	postive
DBH	LINC00174	0.380623928	1.10E-18	postive
DBH	LINC00265	0.38631659	3.04E-19	postive
DBH	LINC00299	0.564224545	2.27E-43	postive
DBH	LINC00426	0.607095025	1.12E-51	postive
DBH	LINC00528	0.605835555	2.05E-51	postive
DBH	LINC00539	0.536874004	1.13E-38	postive
DBH	LINC00544	0.426882932	1.46E-23	postive
DBH	LINC00618	0.303276098	4.26E-12	postive
DBH	LINC00641	0.367467153	1.97E-17	postive
DBH	LINC00653	0.34734233	1.27E-15	postive
DBH	LINC00677	0.329440524	4.03E-14	postive
DBH	LINC00685	0.333896636	1.74E-14	postive
DBH	LINC00852	0.369171337	1.37E-17	postive
DBH	LINC00861	0.565534867	1.32E-43	postive
DBH	LINC00892	0.406173413	2.77E-21	postive
DBH	LINC00893	0.367558574	1.94E-17	postive
DBH	LINC00894	0.357162189	1.73E-16	postive
DBH	LINC00921	0.384253344	4.87E-19	postive
DBH	LINC00926	0.444501631	1.25E-25	postive
DBH	LINC00937	0.446546096	7.08E-26	postive
DBH	LINC00954	0.302297274	5.03E-12	postive
DBH	LINC00987	0.304032801	3.75E-12	postive
DBH	LINC00996	0.52030879	4.95E-36	postive
DBH	LINC01001	0.38931622	1.53E-19	postive
DBH	LINC01011	0.331206901	2.89E-14	postive
DBH	LINC01089	0.472673015	3.40E-29	postive
DBH	LINC01126	0.443431071	1.69E-25	postive
DBH	LINC01138	0.309195751	1.54E-12	postive
DBH	LINC01145	0.395935291	3.25E-20	postive
DBH	LINC01176	0.321010657	1.90E-13	postive
DBH	LINC01237	0.449021723	3.53E-26	postive
DBH	LINC01238	0.3769475	2.50E-18	postive
DBH	LINC01252	0.369911554	1.17E-17	postive
DBH	LINC01281	0.44282659	1.99E-25	postive
DBH	LINC01337	0.321716813	1.67E-13	postive
DBH	LINC01347	0.321642501	1.69E-13	postive
DBH	LINC01355	0.350337114	6.95E-16	postive
DBH	LINC01372	0.385973556	3.29E-19	postive

DBH	LINC01424	0.367861354	1.81E-17	postive
DBH	LINC01480	0.321372753	1.78E-13	postive
DBH	LINC01684	0.35149189	5.50E-16	postive
DBH	LINC01726	0.414503252	3.51E-22	postive
DBH	LINC01727	0.344319248	2.31E-15	postive
DBH	LINC01772	0.308565355	1.72E-12	postive
DBH	LINC01786	0.317089198	3.85E-13	postive
DBH	LINC01871	0.449477457	3.10E-26	postive
DBH	LINC01891	0.405832353	3.01E-21	postive
DBH	LINC01934	0.402208825	7.27E-21	postive
DBH	LINC02019	0.335840656	1.20E-14	postive
DBH	LINC02033	0.340257704	5.12E-15	postive
DBH	LINC02084	0.400661945	1.05E-20	postive
DBH	LINC02132	0.316791924	4.06E-13	postive
DBH	LINC02195	0.327100445	6.22E-14	postive
DBH	LINC02245	0.307816108	1.96E-12	postive
DBH	LINC02273	0.389157055	1.58E-19	postive
DBH	LINC02325	0.458888379	2.08E-27	postive
DBH	LINC02328	0.315060282	5.52E-13	postive
DBH	LINC02352	0.356289759	2.07E-16	postive
DBH	LINC02362	0.339613599	5.80E-15	postive
DBH	LINC02370	0.300341199	6.98E-12	postive
DBH	LINC02390	0.35674898	1.88E-16	postive
DBH	LINC02422	0.352159536	4.81E-16	postive
DBH	LINC02446	0.322360454	1.49E-13	postive
DBH	LINC02520	0.377026185	2.46E-18	postive
DBH	LINC02576	0.346303379	1.56E-15	postive
DBH	LINC02580	0.326361891	7.14E-14	postive
DBH	LINC02604	0.374963577	3.88E-18	postive
DBH	LINC02611	0.305303184	3.02E-12	postive
DBH	LINC02636	0.317188873	3.78E-13	postive
DBH	LINC02642	0.328092231	5.18E-14	postive
DBH	LINC02648	0.385005345	4.10E-19	postive
DBH	LINC02694	0.380992741	1.02E-18	postive
DBH	LINC02705	0.367107986	2.13E-17	postive
DBH	LINC02812	0.368438514	1.60E-17	postive
DBH	LIX1L-AS1	0.344284623	2.32E-15	postive
DBH	LRRC37A17P	0.336876213	9.84E-15	postive
DBH	LRRC8C-DT	0.347122996	1.32E-15	postive
DBH	MAGOH2P	0.412713064	5.50E-22	postive
DBH	MANEA-DT	0.350622337	6.56E-16	postive
DBH	MATN1-AS1	0.374292538	4.50E-18	postive
DBH	MCM3AP-AS1	0.36578422	2.83E-17	postive
DBH	MED8-AS1	0.319851372	2.34E-13	postive
DBH	MHENCR	0.353707725	3.51E-16	postive
DBH	MIAT	0.385213959	3.91E-19	postive
DBH	MIATNB	0.339640901	5.77E-15	postive
DBH	MIF-AS1	0.3078072	1.96E-12	postive
DBH	MIR155HG	0.473834549	2.39E-29	postive
DBH	MIR3142HG	0.334481839	1.56E-14	postive

DBH	MIR4453HG	0.390747273	1.10E-19	postive
DBH	MIR600HG	0.424528199	2.70E-23	postive
DBH	MKNK1-AS1	0.389207841	1.57E-19	postive
DBH	MMP25-AS1	0.576111556	1.50E-45	postive
DBH	MMP2-AS1	0.334330595	1.60E-14	postive
DBH	MORF4L2-AS1	0.312979747	7.97E-13	postive
DBH	MRPS31P5	0.363997525	4.13E-17	postive
DBH	MSH5-SAPCD1	0.360516923	8.60E-17	postive
DBH	MTMR9LP	0.402730237	6.41E-21	postive
DBH	MUC20-OT1	0.346910063	1.38E-15	postive
DBH	MZF1-AS1	0.321195405	1.84E-13	postive
DBH	NAPSB	0.316673937	4.14E-13	postive
DBH	NARF-AS1	0.334761305	1.47E-14	postive
DBH	NARF-IT1	0.355942587	2.22E-16	postive
DBH	NBPF8	0.420295055	8.06E-23	postive
DBH	NCBP2-AS1	0.422493466	4.58E-23	postive
DBH	NCF1B	0.455514769	5.53E-27	postive
DBH	NFYC-AS1	0.390215344	1.24E-19	postive
DBH	NPTN-IT1	0.301734742	5.53E-12	postive
DBH	NSMCE1-DT	0.3006745	6.60E-12	postive
DBH	NSUN5P1	0.308386069	1.78E-12	postive
DBH	NSUN5P2	0.302648084	4.74E-12	postive
DBH	NXT1-AS1	0.345083262	1.99E-15	postive
DBH	OGFR-AS1	0.454070164	8.39E-27	postive
DBH	OSGEPL1-AS1	0.335158359	1.37E-14	postive
DBH	PAN3-AS1	0.347717456	1.18E-15	postive
DBH	PAXBP1-AS1	0.385878713	3.36E-19	postive
DBH	PAXIP1-AS2	0.31696655	3.93E-13	postive
DBH	PCBP1-AS1	0.330534219	3.28E-14	postive
DBH	PCED1B-AS1	0.44330946	1.74E-25	postive
DBH	PDCD4-AS1	0.312102701	9.30E-13	postive
DBH	PEF1-AS1	0.309368112	1.50E-12	postive
DBH	PIK3IP1-AS1	0.304775385	3.30E-12	postive
DBH	PITRM1-AS1	0.386650411	2.82E-19	postive
DBH	PKD1P6	0.390061126	1.29E-19	postive
DBH	PLA2G4C-AS1	0.300794773	6.47E-12	postive
DBH	PLEKHM1P1	0.301916195	5.36E-12	postive
DBH	PMS2P3	0.379538924	1.41E-18	postive
DBH	PPP3CB-AS1	0.339216895	6.26E-15	postive
DBH	PPT2-EGFL8	0.398831796	1.63E-20	postive
DBH	PRKCZ-AS1	0.333928335	1.73E-14	postive
DBH	PRORSD1P	0.400077035	1.21E-20	postive
DBH	PRR7-AS1	0.344993533	2.02E-15	postive
DBH	PSMA3-AS1	0.414732575	3.31E-22	postive
DBH	PSMB8-AS1	0.434870643	1.75E-24	postive
DBH	PTOV1-AS2	0.479199807	4.54E-30	postive
DBH	RAD51-AS1	0.433413887	2.58E-24	postive
DBH	RBM5-AS1	0.372071598	7.31E-18	postive
DBH	RFPL3S	0.34849616	1.01E-15	postive
DBH	RNF139-AS1	0.340513623	4.87E-15	postive

DBH	RNF213-AS1	0.330447692	3.33E-14	postive
DBH	RP11-104N10.2	0.328374722	4.91E-14	postive
DBH	RP11-111M22.4	0.305733013	2.80E-12	postive
DBH	RP11-111M22.5	0.311226885	1.08E-12	postive
DBH	RP11-148O21.2	0.37703957	2.45E-18	postive
DBH	RP11-148O21.4	0.327642931	5.63E-14	postive
DBH	RP11-192H23.8	0.314202214	6.43E-13	postive
DBH	RP11-199F11.2	0.325538596	8.31E-14	postive
DBH	RP11-206L10.5	0.315182427	5.40E-13	postive
DBH	RP11-258C19.7	0.339520994	5.90E-15	postive
DBH	RP11-327F22.1	0.641135541	3.12E-59	postive
DBH	RP11-346C20.4	0.340767213	4.63E-15	postive
DBH	RP11-367G6.3	0.335048002	1.40E-14	postive
DBH	RP11-394O4.5	0.320386293	2.13E-13	postive
DBH	RP11-540B6.6	0.488763759	2.20E-31	postive
DBH	RP11-562A8.5	0.309719099	1.41E-12	postive
DBH	RP11-58E21.5	0.349905056	7.58E-16	postive
DBH	RP11-658F2.8	0.332455092	2.28E-14	postive
DBH	RP11-876N24.4	0.404320001	4.36E-21	postive
DBH	RP11-876N24.5	0.330591686	3.25E-14	postive
DBH	RP11-97O12.6	0.33938001	6.07E-15	postive
DBH	RP1-199J3.7	0.303802385	3.89E-12	postive
DBH	RP3-368A4.5	0.305730731	2.80E-12	postive
DBH	RP3-492J12.2	0.306962066	2.27E-12	postive
DBH	RPL32P3	0.483834837	1.06E-30	postive
DBH	RRN3P1	0.352037263	4.93E-16	postive
DBH	RRN3P2	0.364678517	3.58E-17	postive
DBH	RRP7BP	0.352970439	4.08E-16	postive
DBH	RUSC1-AS1	0.385729439	3.48E-19	postive
DBH	SCAND2P	0.380325672	1.18E-18	postive
DBH	SDHAP1	0.367111566	2.13E-17	postive
DBH	SEC1P	0.357989422	1.45E-16	postive
DBH	SEMA3F-AS1	0.436511855	1.12E-24	postive
DBH	SEMA6A-AS1	0.306072584	2.64E-12	postive
DBH	SEPTIN7P2	0.391394153	9.43E-20	postive
DBH	SGOL1-AS1	0.308505029	1.74E-12	postive
DBH	SH3BP5-AS1	0.434397771	1.98E-24	postive
DBH	SH3GL1P1	0.43522354	1.59E-24	postive
DBH	SIGLEC22P	0.30496459	3.20E-12	postive
DBH	SIRPG-AS1	0.406265455	2.71E-21	postive
DBH	SLC25A25-AS1	0.353097399	3.97E-16	postive
DBH	SLC25A5P1	0.3059181	2.72E-12	postive
DBH	SLX1A-SULT1A3	0.340958066	4.46E-15	postive
DBH	SMG1P7	0.333699296	1.80E-14	postive
DBH	SNHG12	0.366629486	2.36E-17	postive
DBH	SNHG20	0.447174909	5.93E-26	postive
DBH	SNHG21	0.307025308	2.25E-12	postive
DBH	SPAG5-AS1	0.471325564	5.13E-29	postive
DBH	SRRM2-AS1	0.346396778	1.53E-15	postive
DBH	STAG3L1	0.411439126	7.56E-22	postive

DBH	STAG3L2	0.390761728	1.09E-19	postive
DBH	STAG3L3	0.440620007	3.66E-25	postive
DBH	STAG3L5P	0.414698988	3.34E-22	postive
DBH	STAG3L5P-PVRIG2P-PILRB	0.377481942	2.23E-18	postive
DBH	STAM-AS1	0.400553464	1.08E-20	postive
DBH	STARD7-AS1	0.361566324	6.90E-17	postive
DBH	SUGT1P4-STRA6LP	0.341461027	4.05E-15	postive
DBH	SUZ12P1	0.338463268	7.25E-15	postive
DBH	SYNGAP1-AS1	0.326606605	6.82E-14	postive
DBH	SZT2-AS1	0.326505137	6.95E-14	postive
DBH	TBC1D27P	0.441223071	3.10E-25	postive
DBH	TBX2-AS1	0.352468303	4.52E-16	postive
DBH	TEX21P	0.341880826	3.73E-15	postive
DBH	THUMPD3-AS1	0.33763095	8.51E-15	postive
DBH	TMED2-DT	0.350667445	6.50E-16	postive
DBH	TMEM147-AS1	0.465307945	3.14E-28	postive
DBH	TMEM18-DT	0.305891251	2.73E-12	postive
DBH	TMEM198B	0.3971508	2.44E-20	postive
DBH	TMSB15B-AS1	0.323419488	1.23E-13	postive
DBH	TNFRSF14-AS1	0.30697158	2.27E-12	postive
DBH	TNK2-AS1	0.448131309	4.54E-26	postive
DBH	TNRC6C-AS1	0.360712913	8.25E-17	postive
DBH	TPT1-AS1	0.320469893	2.10E-13	postive
DBH	TPTEP2	0.321663784	1.69E-13	postive
DBH	TRBV11-2	0.305783401	2.78E-12	postive
DBH	TRG-AS1	0.582346699	9.90E-47	postive
DBH	TSC22D1-AS1	0.33901501	6.51E-15	postive
DBH	TSPOAP1-AS1	0.585999159	1.96E-47	postive
DBH	U62317.2	0.410903785	8.64E-22	postive
DBH	U62631.1	0.43174629	4.04E-24	postive
DBH	U73169.1	0.321877644	1.62E-13	postive
DBH	U91328.3	0.322155553	1.54E-13	postive
DBH	UBE2Q1-AS1	0.361477155	7.03E-17	postive
DBH	UBOX5-AS1	0.311081671	1.11E-12	postive
DBH	USP30-AS1	0.437596793	8.36E-25	postive
DBH	VASH1-AS1	0.348641358	9.77E-16	postive
DBH	VDAC1P8	0.38020817	1.21E-18	postive
DBH	WASH3P	0.321266732	1.81E-13	postive
DBH	WHAMMP3	0.327992655	5.27E-14	postive
DBH	YEATS2-AS1	0.328092939	5.18E-14	postive
DBH	Z82188.2	0.366961181	2.20E-17	postive
DBH	Z83844.2	0.413852081	4.14E-22	postive
DBH	Z84492.1	0.3066478	2.40E-12	postive
DBH	Z84723.1	0.318882605	2.79E-13	postive
DBH	Z94721.1	0.354745384	2.84E-16	postive
DBH	Z97832.2	0.37832369	1.85E-18	postive
DBH	Z97989.1	0.311522626	1.03E-12	postive
DBH	Z98884.2	0.34603372	1.64E-15	postive
DBH	Z99774.1	0.354062746	3.26E-16	postive
DBH	ZBTB40-IT1	0.366623686	2.37E-17	postive

DBH	ZDHHC20-IT1	0.33931753	6.14E-15	postive
DBH	ZFHX2-AS1	0.354578806	2.94E-16	postive
DBH	ZKSCAN2-DT	0.425795167	1.94E-23	postive
DBH	ZNF205-AS1	0.314523894	6.07E-13	postive
DBH	ZNF213-AS1	0.349582303	8.09E-16	postive
DBH	ZNF32-AS1	0.327054833	6.28E-14	postive
DBH	ZNF32-AS2	0.352853264	4.18E-16	postive
DBH	ZNF37BP	0.428018462	1.08E-23	postive
DBH	ZNF436-AS1	0.328704811	4.62E-14	postive
DBH	ZNF460-AS1	0.332777954	2.15E-14	postive
DBH	ZNF767P	0.469206636	9.75E-29	postive
DBH	ZNRD1ASP	0.414441204	3.57E-22	postive
DBH	ZNRD2-AS1	0.337657668	8.46E-15	postive
DBH	ZSWIM8-AS1	0.338796384	6.79E-15	postive
DLAT	ABALON	0.439366762	5.16E-25	postive
DLAT	AC000068.1	0.385529474	3.64E-19	postive
DLAT	AC002310.1	0.383596397	5.65E-19	postive
DLAT	AC002467.1	0.424195666	2.94E-23	postive
DLAT	AC002470.1	0.311288009	1.07E-12	postive
DLAT	AC004221.1	0.332443688	2.29E-14	postive
DLAT	AC004241.3	0.440737345	3.54E-25	postive
DLAT	AC004477.3	0.321421045	1.76E-13	postive
DLAT	AC004596.1	0.468447397	1.23E-28	postive
DLAT	AC004771.2	0.353734577	3.49E-16	postive
DLAT	AC004803.1	0.437299195	9.06E-25	postive
DLAT	AC004893.11	0.363689132	4.41E-17	postive
DLAT	AC004918.5	0.315789511	4.85E-13	postive
DLAT	AC004943.2	0.443168231	1.81E-25	postive
DLAT	AC005005.4	0.304602115	3.40E-12	postive
DLAT	AC005034.4	0.519165532	7.45E-36	postive
DLAT	AC005034.6	0.359283998	1.11E-16	postive
DLAT	AC005076.2	0.308062331	1.88E-12	postive
DLAT	AC005229.4	0.414717047	3.33E-22	postive
DLAT	AC005288.1	0.577357977	8.75E-46	postive
DLAT	AC005562.1	0.607796825	8.01E-52	postive
DLAT	AC005586.1	0.443477481	1.66E-25	postive
DLAT	AC005670.3	0.468368593	1.25E-28	postive
DLAT	AC005696.1	0.326687262	6.72E-14	postive
DLAT	AC005841.1	0.328011178	5.26E-14	postive
DLAT	AC005884.2	0.342045142	3.61E-15	postive
DLAT	AC006001.2	0.324572462	9.92E-14	postive
DLAT	AC006064.2	0.337636723	8.50E-15	postive
DLAT	AC006122.1	0.318546355	2.96E-13	postive
DLAT	AC006126.4	0.305249145	3.04E-12	postive
DLAT	AC006213.1	0.309554943	1.45E-12	postive
DLAT	AC006252.1	0.349928033	7.55E-16	postive
DLAT	AC006333.1	0.393225253	6.15E-20	postive
DLAT	AC006449.2	0.429025609	8.31E-24	postive
DLAT	AC006449.5	0.359111286	1.15E-16	postive
DLAT	AC006449.7	0.350774221	6.36E-16	postive

DLAT	AC006504.5	0.341880569	3.73E-15	postive
DLAT	AC006547.1	0.30556369	2.88E-12	postive
DLAT	AC007032.1	0.319325268	2.58E-13	postive
DLAT	AC007228.9	0.321664455	1.69E-13	postive
DLAT	AC007298.2	0.308933831	1.62E-12	postive
DLAT	AC007342.4	0.360827455	8.05E-17	postive
DLAT	AC007406.5	0.332864793	2.11E-14	postive
DLAT	AC007541.1	0.329710209	3.83E-14	postive
DLAT	AC007823.1	0.316465169	4.30E-13	postive
DLAT	AC008014.1	0.445537511	9.38E-26	postive
DLAT	AC008267.5	0.336267604	1.11E-14	postive
DLAT	AC008438.1	0.323549412	1.20E-13	postive
DLAT	AC008443.1	0.383603741	5.64E-19	postive
DLAT	AC008771.1	0.361160562	7.51E-17	postive
DLAT	AC008915.3	0.371190401	8.85E-18	postive
DLAT	AC008946.1	0.33435403	1.59E-14	postive
DLAT	AC008966.1	0.350001805	7.44E-16	postive
DLAT	AC009061.2	0.414070062	3.92E-22	postive
DLAT	AC009126.1	0.327697109	5.57E-14	postive
DLAT	AC009171.2	0.330526145	3.29E-14	postive
DLAT	AC009237.14	0.359243232	1.12E-16	postive
DLAT	AC009779.3	0.529786128	1.59E-37	postive
DLAT	AC009831.1	0.341422301	4.08E-15	postive
DLAT	AC009948.3	0.302313084	5.01E-12	postive
DLAT	AC009961.1	0.337936433	8.02E-15	postive
DLAT	AC010442.1	0.340929297	4.49E-15	postive
DLAT	AC010595.1	0.322595215	1.42E-13	postive
DLAT	AC010643.1	0.413256505	4.80E-22	postive
DLAT	AC010655.4	0.34678839	1.42E-15	postive
DLAT	AC010789.1	0.332687438	2.19E-14	postive
DLAT	AC010809.1	0.305776576	2.78E-12	postive
DLAT	AC010894.2	0.320797606	1.98E-13	postive
DLAT	AC010969.2	0.324216544	1.06E-13	postive
DLAT	AC011978.2	0.34110496	4.34E-15	postive
DLAT	AC012063.1	0.372343222	6.89E-18	postive
DLAT	AC012073.1	0.400376677	1.13E-20	postive
DLAT	AC012146.1	0.32686471	6.50E-14	postive
DLAT	AC012306.2	0.337032548	9.55E-15	postive
DLAT	AC012313.1	0.355102984	2.64E-16	postive
DLAT	AC012442.2	0.330274088	3.44E-14	postive
DLAT	AC012442.3	0.334371588	1.59E-14	postive
DLAT	AC012467.2	0.385104451	4.01E-19	postive
DLAT	AC012555.1	0.353874023	3.39E-16	postive
DLAT	AC012640.4	0.316094604	4.60E-13	postive
DLAT	AC015921.1	0.380627339	1.10E-18	postive
DLAT	AC015922.2	0.45854925	2.30E-27	postive
DLAT	AC015922.3	0.342074429	3.59E-15	postive
DLAT	AC016065.1	0.43236451	3.42E-24	postive
DLAT	AC016727.1	0.390166739	1.25E-19	postive
DLAT	AC016747.1	0.503064085	1.97E-33	postive

DLAT	AC016876.2	0.383686185	5.54E-19	postive
DLAT	AC016876.3	0.317398897	3.64E-13	postive
DLAT	AC016888.1	0.378884316	1.63E-18	postive
DLAT	AC018521.5	0.38183507	8.41E-19	postive
DLAT	AC018647.2	0.440989289	3.31E-25	postive
DLAT	AC019069.1	0.381431186	9.21E-19	postive
DLAT	AC020661.1	0.315503609	5.10E-13	postive
DLAT	AC020663.2	0.371672369	7.97E-18	postive
DLAT	AC020904.2	0.343146387	2.91E-15	postive
DLAT	AC020915.1	0.373619562	5.21E-18	postive
DLAT	AC020915.3	0.408309585	1.64E-21	postive
DLAT	AC021087.1	0.30460408	3.40E-12	postive
DLAT	AC021092.1	0.314893785	5.69E-13	postive
DLAT	AC021224.1	0.310255477	1.28E-12	postive
DLAT	AC022167.2	0.312834031	8.18E-13	postive
DLAT	AC022211.3	0.391495719	9.21E-20	postive
DLAT	AC022364.1	0.302912632	4.53E-12	postive
DLAT	AC022613.2	0.341744129	3.83E-15	postive
DLAT	AC022784.5	0.335251256	1.34E-14	postive
DLAT	AC023509.3	0.327291783	6.01E-14	postive
DLAT	AC024267.7	0.420232333	8.19E-23	postive
DLAT	AC024575.2	0.344239057	2.35E-15	postive
DLAT	AC025580.2	0.352831419	4.19E-16	postive
DLAT	AC025682.2	0.355377354	2.49E-16	postive
DLAT	AC026412.1	0.333956383	1.72E-14	postive
DLAT	AC026471.1	0.466059828	2.51E-28	postive
DLAT	AC027097.1	0.413398208	4.63E-22	postive
DLAT	AC027307.2	0.313992414	6.67E-13	postive
DLAT	AC027307.3	0.327921807	5.34E-14	postive
DLAT	AC040970.1	0.311316547	1.07E-12	postive
DLAT	AC046134.2	0.398690969	1.69E-20	postive
DLAT	AC060780.1	0.325180258	8.87E-14	postive
DLAT	AC061992.2	0.340737397	4.66E-15	postive
DLAT	AC064807.1	0.451758429	1.62E-26	postive
DLAT	AC067852.2	0.615209228	2.15E-53	postive
DLAT	AC068228.2	0.343874451	2.52E-15	postive
DLAT	AC068338.2	0.3750189	3.83E-18	postive
DLAT	AC068831.2	0.300668023	6.61E-12	postive
DLAT	AC068870.2	0.36913325	1.38E-17	postive
DLAT	AC068888.1	0.300392352	6.92E-12	postive
DLAT	AC069185.1	0.371586747	8.12E-18	postive
DLAT	AC073046.1	0.462417007	7.39E-28	postive
DLAT	AC073073.2	0.445681802	9.01E-26	postive
DLAT	AC073254.1	0.305412574	2.96E-12	postive
DLAT	AC073342.1	0.325460319	8.43E-14	postive
DLAT	AC073508.3	0.383996021	5.16E-19	postive
DLAT	AC073529.1	0.469256087	9.60E-29	postive
DLAT	AC073569.2	0.519313615	7.07E-36	postive
DLAT	AC073585.1	0.402150693	7.37E-21	postive
DLAT	AC073896.2	0.329516064	3.97E-14	postive

DLAT	AC073896.3	0.390664076	1.12E-19	postive
DLAT	AC074117.1	0.336990081	9.62E-15	postive
DLAT	AC079447.1	0.301471209	5.78E-12	postive
DLAT	AC079807.1	0.410614145	9.28E-22	postive
DLAT	AC080112.2	0.441063554	3.24E-25	postive
DLAT	AC083799.1	0.392062612	8.07E-20	postive
DLAT	AC087071.2	0.383750942	5.46E-19	postive
DLAT	AC087645.2	0.306577938	2.42E-12	postive
DLAT	AC090114.2	0.362063272	6.22E-17	postive
DLAT	AC090587.1	0.34638267	1.53E-15	postive
DLAT	AC090617.5	0.321223278	1.83E-13	postive
DLAT	AC091057.1	0.395316579	3.76E-20	postive
DLAT	AC091152.2	0.336010925	1.16E-14	postive
DLAT	AC091729.3	0.322573049	1.43E-13	postive
DLAT	AC091982.3	0.342081538	3.58E-15	postive
DLAT	AC092070.2	0.300781917	6.49E-12	postive
DLAT	AC092115.4	0.305553704	2.89E-12	postive
DLAT	AC092171.2	0.318508591	2.98E-13	postive
DLAT	AC092295.2	0.348687667	9.68E-16	postive
DLAT	AC092614.1	0.414707819	3.34E-22	postive
DLAT	AC092687.3	0.323664298	1.17E-13	postive
DLAT	AC092720.1	0.342677622	3.19E-15	postive
DLAT	AC093157.1	0.385533619	3.64E-19	postive
DLAT	AC093627.5	0.315915176	4.74E-13	postive
DLAT	AC093673.1	0.348104582	1.09E-15	postive
DLAT	AC096536.2	0.402932755	6.10E-21	postive
DLAT	AC096649.2	0.308552654	1.73E-12	postive
DLAT	AC096677.1	0.301823017	5.44E-12	postive
DLAT	AC096733.3	0.34883643	9.40E-16	postive
DLAT	AC096772.6	0.33288188	2.11E-14	postive
DLAT	AC097359.2	0.348763574	9.54E-16	postive
DLAT	AC098484.1	0.445546337	9.36E-26	postive
DLAT	AC098820.2	0.308747302	1.67E-12	postive
DLAT	AC098847.1	0.307078685	2.22E-12	postive
DLAT	AC098934.1	0.348241788	1.06E-15	postive
DLAT	AC098934.2	0.342216893	3.49E-15	postive
DLAT	AC099850.2	0.388889321	1.69E-19	postive
DLAT	AC099850.4	0.503041143	1.99E-33	postive
DLAT	AC100810.1	0.340451153	4.93E-15	postive
DLAT	AC100861.1	0.50388052	1.50E-33	postive
DLAT	AC104109.2	0.351354139	5.66E-16	postive
DLAT	AC104521.1	0.332038646	2.47E-14	postive
DLAT	AC104561.1	0.427876107	1.12E-23	postive
DLAT	AC104561.3	0.376084229	3.03E-18	postive
DLAT	AC105036.3	0.51998459	5.56E-36	postive
DLAT	AC105206.2	0.3100608	1.33E-12	postive
DLAT	AC105285.1	0.37523388	3.66E-18	postive
DLAT	AC105942.1	0.347801029	1.16E-15	postive
DLAT	AC106739.1	0.370477244	1.03E-17	postive
DLAT	AC106786.2	0.364224375	3.94E-17	postive

DLAT	AC106791.1	0.33343196	1.90E-14	postive
DLAT	AC106795.1	0.385889356	3.35E-19	postive
DLAT	AC107027.3	0.351368904	5.64E-16	postive
DLAT	AC107032.2	0.321648257	1.69E-13	postive
DLAT	AC107068.1	0.458829409	2.12E-27	postive
DLAT	AC107214.1	0.391133527	1.00E-19	postive
DLAT	AC107959.3	0.399959004	1.25E-20	postive
DLAT	AC107982.3	0.358723627	1.25E-16	postive
DLAT	AC108693.2	0.342843419	3.09E-15	postive
DLAT	AC109454.2	0.392988175	6.50E-20	postive
DLAT	AC112220.2	0.436424739	1.15E-24	postive
DLAT	AC112236.2	0.342473804	3.32E-15	postive
DLAT	AC114271.1	0.41620041	2.29E-22	postive
DLAT	AC114763.1	0.328777816	4.56E-14	postive
DLAT	AC114956.2	0.303908635	3.83E-12	postive
DLAT	AC115618.1	0.429006971	8.35E-24	postive
DLAT	AC115618.2	0.519962329	5.61E-36	postive
DLAT	AC116036.2	0.368439783	1.60E-17	postive
DLAT	AC116913.1	0.363023268	5.08E-17	postive
DLAT	AC120114.1	0.438457361	6.61E-25	postive
DLAT	AC121761.1	0.305018137	3.17E-12	postive
DLAT	AC124242.1	0.300450503	6.86E-12	postive
DLAT	AC124283.2	0.40291003	6.14E-21	postive
DLAT	AC125257.1	0.503312881	1.81E-33	postive
DLAT	AC125494.1	0.37587667	3.17E-18	postive
DLAT	AC125807.2	0.420294216	8.06E-23	postive
DLAT	AC126365.1	0.303182539	4.33E-12	postive
DLAT	AC131009.3	0.389730947	1.39E-19	postive
DLAT	AC131011.2	0.378254271	1.87E-18	postive
DLAT	AC131235.3	0.312284348	9.01E-13	postive
DLAT	AC132008.2	0.317646738	3.48E-13	postive
DLAT	AC132938.4	0.399759068	1.31E-20	postive
DLAT	AC133528.2	0.352236508	4.73E-16	postive
DLAT	AC135050.1	0.442853433	1.98E-25	postive
DLAT	AC135178.4	0.351862139	5.11E-16	postive
DLAT	AC139887.2	0.364132519	4.02E-17	postive
DLAT	AC141586.1	0.472354445	3.75E-29	postive
DLAT	AC144652.1	0.307185231	2.18E-12	postive
DLAT	AC231981.1	0.338284345	7.50E-15	postive
DLAT	AC245140.2	0.326260888	7.27E-14	postive
DLAT	ACVR2B-AS1	0.414960011	3.13E-22	postive
DLAT	AL008726.1	0.308843331	1.64E-12	postive
DLAT	AL021154.1	0.301829905	5.44E-12	postive
DLAT	AL024498.1	0.316885725	3.99E-13	postive
DLAT	AL024507.3	0.393929293	5.22E-20	postive
DLAT	AL031123.2	0.306902992	2.29E-12	postive
DLAT	AL031282.1	0.334018902	1.70E-14	postive
DLAT	AL031985.3	0.413977894	4.01E-22	postive
DLAT	AL035587.1	0.339931359	5.45E-15	postive
DLAT	AL049597.2	0.410094212	1.06E-21	postive

DLAT	AL049779.1	0.318309545	3.09E-13	postive
DLAT	AL049796.1	0.4014653	8.70E-21	postive
DLAT	AL049840.4	0.353258591	3.84E-16	postive
DLAT	AL049840.5	0.30649783	2.46E-12	postive
DLAT	AL050309.1	0.347215548	1.30E-15	postive
DLAT	AL078581.2	0.430768101	5.23E-24	postive
DLAT	AL078644.2	0.466648002	2.10E-28	postive
DLAT	AL079303.1	0.321146193	1.85E-13	postive
DLAT	AL080250.1	0.400359605	1.13E-20	postive
DLAT	AL109917.1	0.348738731	9.59E-16	postive
DLAT	AL117332.1	0.326207105	7.34E-14	postive
DLAT	AL117336.2	0.306143764	2.61E-12	postive
DLAT	AL118506.1	0.384374885	4.74E-19	postive
DLAT	AL121603.2	0.348430407	1.02E-15	postive
DLAT	AL121906.2	0.331341663	2.82E-14	postive
DLAT	AL121944.2	0.508117216	3.54E-34	postive
DLAT	AL122035.1	0.342035512	3.62E-15	postive
DLAT	AL132639.2	0.316628334	4.18E-13	postive
DLAT	AL133153.2	0.321497106	1.74E-13	postive
DLAT	AL133215.2	0.365343355	3.11E-17	postive
DLAT	AL136040.1	0.353127824	3.95E-16	postive
DLAT	AL138921.2	0.30638346	2.51E-12	postive
DLAT	AL139035.1	0.490771101	1.15E-31	postive
DLAT	AL139260.2	0.333668674	1.82E-14	postive
DLAT	AL139289.1	0.339388605	6.06E-15	postive
DLAT	AL139384.1	0.31095129	1.14E-12	postive
DLAT	AL157400.2	0.304321896	3.57E-12	postive
DLAT	AL158212.3	0.337799489	8.24E-15	postive
DLAT	AL160006.1	0.335831728	1.20E-14	postive
DLAT	AL160286.2	0.302915257	4.53E-12	postive
DLAT	AL161421.1	0.329761336	3.79E-14	postive
DLAT	AL161772.1	0.320974676	1.91E-13	postive
DLAT	AL161891.1	0.361034797	7.71E-17	postive
DLAT	AL162231.2	0.34130086	4.18E-15	postive
DLAT	AL162311.3	0.314337016	6.28E-13	postive
DLAT	AL162595.1	0.41176254	6.98E-22	postive
DLAT	AL353194.1	0.346805773	1.41E-15	postive
DLAT	AL353593.1	0.350380544	6.89E-16	postive
DLAT	AL353708.1	0.331110919	2.94E-14	postive
DLAT	AL353743.1	0.339734228	5.66E-15	postive
DLAT	AL353764.1	0.324433311	1.02E-13	postive
DLAT	AL353796.1	0.356161103	2.12E-16	postive
DLAT	AL354696.1	0.313331851	7.49E-13	postive
DLAT	AL354696.2	0.356269156	2.08E-16	postive
DLAT	AL354740.1	0.314862322	5.72E-13	postive
DLAT	AL354811.1	0.340097258	5.28E-15	postive
DLAT	AL354892.2	0.424468312	2.74E-23	postive
DLAT	AL354920.1	0.326668686	6.74E-14	postive
DLAT	AL355574.1	0.383715347	5.50E-19	postive
DLAT	AL356481.1	0.336452052	1.07E-14	postive

DLAT	AL356488.3	0.30287562	4.56E-12	postive
DLAT	AL357140.2	0.308690913	1.68E-12	postive
DLAT	AL359317.1	0.323571729	1.19E-13	postive
DLAT	AL359643.3	0.390072218	1.28E-19	postive
DLAT	AL359715.3	0.488531166	2.37E-31	postive
DLAT	AL360270.1	0.345747239	1.74E-15	postive
DLAT	AL365181.3	0.307479026	2.08E-12	postive
DLAT	AL391121.1	0.370949668	9.32E-18	postive
DLAT	AL391832.3	0.316045383	4.64E-13	postive
DLAT	AL441992.2	0.403871048	4.86E-21	postive
DLAT	AL445231.1	0.351487123	5.51E-16	postive
DLAT	AL450326.1	0.453252774	1.06E-26	postive
DLAT	AL450998.2	0.32197784	1.59E-13	postive
DLAT	AL512408.1	0.337127688	9.37E-15	postive
DLAT	AL512598.1	0.336977614	9.65E-15	postive
DLAT	AL513165.1	0.317682886	3.46E-13	postive
DLAT	AL513165.2	0.359231919	1.12E-16	postive
DLAT	AL513550.1	0.395648267	3.48E-20	postive
DLAT	AL589765.4	0.3398858	5.50E-15	postive
DLAT	AL590666.2	0.336115031	1.14E-14	postive
DLAT	AL591684.2	0.321522702	1.73E-13	postive
DLAT	AL592166.1	0.4332307	2.71E-24	postive
DLAT	AL604028.1	0.317401995	3.64E-13	postive
DLAT	AL606760.1	0.335423501	1.30E-14	postive
DLAT	AL606834.1	0.335737811	1.22E-14	postive
DLAT	AL645568.1	0.332515498	2.26E-14	postive
DLAT	AL669831.1	0.31196074	9.53E-13	postive
DLAT	AL731566.2	0.338196183	7.63E-15	postive
DLAT	AL731577.2	0.50723791	4.78E-34	postive
DLAT	AL732314.8	0.300035822	7.35E-12	postive
DLAT	AL928654.1	0.44707494	6.10E-26	postive
DLAT	ALG1L13P	0.339691091	5.71E-15	postive
DLAT	ALMS1-IT1	0.33320637	1.98E-14	postive
DLAT	AMZ2P1	0.36951552	1.27E-17	postive
DLAT	ANP32AP1	0.360882616	7.96E-17	postive
DLAT	AP000442.1	0.369601275	1.25E-17	postive
DLAT	AP000487.1	0.391212431	9.84E-20	postive
DLAT	AP000759.1	0.534156646	3.14E-38	postive
DLAT	AP000769.1	0.320175789	2.21E-13	postive
DLAT	AP000808.1	0.329486231	3.99E-14	postive
DLAT	AP001033.3	0.333182479	1.99E-14	postive
DLAT	AP001107.1	0.405014022	3.68E-21	postive
DLAT	AP001160.3	0.413818932	4.17E-22	postive
DLAT	AP001267.1	0.344668309	2.16E-15	postive
DLAT	AP001267.3	0.322560582	1.43E-13	postive
DLAT	AP001318.2	0.440371622	3.92E-25	postive
DLAT	AP001372.2	0.479831263	3.73E-30	postive
DLAT	AP001432.1	0.309729907	1.41E-12	postive
DLAT	AP001453.2	0.308290161	1.81E-12	postive
DLAT	AP001453.4	0.414366769	3.63E-22	postive

DLAT	AP001462.1	0.317014641	3.90E-13	postive
DLAT	AP001469.2	0.300230568	7.11E-12	postive
DLAT	AP001469.3	0.306925561	2.28E-12	postive
DLAT	AP001486.2	0.304924178	3.22E-12	postive
DLAT	AP001505.1	0.318702545	2.88E-13	postive
DLAT	AP001542.3	0.319429488	2.53E-13	postive
DLAT	AP001922.5	0.391553645	9.09E-20	postive
DLAT	AP002360.2	0.325448431	8.45E-14	postive
DLAT	AP002387.2	0.433997309	2.21E-24	postive
DLAT	AP002748.4	0.385558831	3.62E-19	postive
DLAT	AP002770.1	0.309567406	1.45E-12	postive
DLAT	AP002840.2	0.335980694	1.17E-14	postive
DLAT	AP003059.1	0.368083621	1.73E-17	postive
DLAT	AP003068.1	0.365993496	2.71E-17	postive
DLAT	AP003068.2	0.430395575	5.78E-24	postive
DLAT	AP003392.1	0.300708463	6.57E-12	postive
DLAT	AP003392.4	0.316818952	4.04E-13	postive
DLAT	AP003392.5	0.305806189	2.77E-12	postive
DLAT	AP003486.1	0.367447337	1.98E-17	postive
DLAT	AP003680.1	0.388603223	1.80E-19	postive
DLAT	AP004609.5	0.369294376	1.33E-17	postive
DLAT	AP005329.2	0.346684778	1.44E-15	postive
DLAT	AP005482.4	0.370353427	1.06E-17	postive
DLAT	ARHGAP5-AS1	0.318372845	3.06E-13	postive
DLAT	ARMCX5-GPRASP2	0.505188084	9.61E-34	postive
DLAT	ARRDC1-AS1	0.37571747	3.29E-18	postive
DLAT	ATF4P4	0.365187756	3.21E-17	postive
DLAT	ATP2B1-AS1	0.355064233	2.66E-16	postive
DLAT	BAALC-AS1	0.404274179	4.41E-21	postive
DLAT	BACE1-AS	0.421840273	5.42E-23	postive
DLAT	BAIAP2-DT	0.316261964	4.46E-13	postive
DLAT	BCDIN3D-AS1	0.343773319	2.57E-15	postive
DLAT	BEND3P1	0.384815689	4.28E-19	postive
DLAT	BHLHE40-AS1	0.305128929	3.11E-12	postive
DLAT	BMS1P20	0.489730199	1.61E-31	postive
DLAT	BOLA3-AS1	0.400058354	1.22E-20	postive
DLAT	BX322557.10	0.375825135	3.21E-18	postive
DLAT	C10orf25	0.364822845	3.47E-17	postive
DLAT	C15orf56	0.304888417	3.24E-12	postive
DLAT	C18orf65	0.34357685	2.67E-15	postive
DLAT	C22orf46	0.446380217	7.41E-26	postive
DLAT	C2CD4D-AS1	0.423400916	3.62E-23	postive
DLAT	C5orf66	0.307715052	1.99E-12	postive
DLAT	C6orf99	0.381633602	8.80E-19	postive
DLAT	CA5BP1	0.446248196	7.69E-26	postive
DLAT	CASC15	0.30429716	3.58E-12	postive
DLAT	CATSPER2P1	0.605393416	2.54E-51	postive
DLAT	CCDC84-DT	0.440969589	3.32E-25	postive
DLAT	CCNT2-AS1	0.402656574	6.52E-21	postive
DLAT	CD27-AS1	0.353991658	3.31E-16	postive

DLAT	CD99P1	0.402497222	6.78E-21	postive
DLAT	CENPBD1P1	0.509869141	1.94E-34	postive
DLAT	CERNA1	0.306109347	2.63E-12	postive
DLAT	CFL1P1	0.31503318	5.55E-13	postive
DLAT	CHKB-DT	0.319976785	2.29E-13	postive
DLAT	CIDECP1	0.489401327	1.79E-31	postive
DLAT	COX10-AS1	0.335536695	1.27E-14	postive
DLAT	CR381653.1	0.384187644	4.94E-19	postive
DLAT	CRTC3-AS1	0.318429825	3.03E-13	postive
DLAT	CRYBB2P1	0.323677344	1.17E-13	postive
DLAT	CRYZL2P	0.417459413	1.66E-22	postive
DLAT	CSAG4	0.355242235	2.56E-16	postive
DLAT	CSE1L-AS1	0.309286615	1.52E-12	postive
DLAT	CTA-989H11.1	0.364297848	3.88E-17	postive
DLAT	CTBP1-DT	0.571671903	1.00E-44	postive
DLAT	CTD-2245F17.3	0.336301072	1.10E-14	postive
DLAT	CTD-2574D22.4	0.334660432	1.50E-14	postive
DLAT	DANCR	0.440394419	3.89E-25	postive
DLAT	DARS-AS1	0.375870909	3.18E-18	postive
DLAT	DBIL5P	0.387245256	2.46E-19	postive
DLAT	DCST1-AS1	0.332385481	2.31E-14	postive
DLAT	DCXR-DT	0.315407643	5.19E-13	postive
DLAT	DDN-AS1	0.321239792	1.82E-13	postive
DLAT	DDX11-AS1	0.346592675	1.47E-15	postive
DLAT	DGCR11	0.452752486	1.22E-26	postive
DLAT	DHCR24-DT	0.312242915	9.07E-13	postive
DLAT	DHRS4-AS1	0.451035876	1.99E-26	postive
DLAT	DHRSX-IT1	0.31792123	3.32E-13	postive
DLAT	DLEU1	0.35507657	2.65E-16	postive
DLAT	DLG5-AS1	0.305602696	2.87E-12	postive
DLAT	DLGAP1-AS1	0.308876403	1.63E-12	postive
DLAT	DNAH10OS	0.308397887	1.77E-12	postive
DLAT	DNMBP-AS1	0.342300209	3.43E-15	postive
DLAT	DPY19L2P3	0.340561083	4.82E-15	postive
DLAT	DSTNP2	0.354545281	2.96E-16	postive
DLAT	DYRK3-AS1	0.310892947	1.15E-12	postive
DLAT	EBLN3P	0.515872474	2.39E-35	postive
DLAT	ECI2-DT	0.32832119	4.96E-14	postive
DLAT	EIF1B-AS1	0.348683871	9.69E-16	postive
DLAT	EIF2AK3-DT	0.325158196	8.91E-14	postive
DLAT	EIF3J-DT	0.436250657	1.20E-24	postive
DLAT	ELF3-AS1	0.489457823	1.76E-31	postive
DLAT	ELOA-AS1	0.335521631	1.28E-14	postive
DLAT	EMC1-AS1	0.301037816	6.21E-12	postive
DLAT	EMC3-AS1	0.329610779	3.90E-14	postive
DLAT	EMSLR	0.339441626	6.00E-15	postive
DLAT	ENTPD1-AS1	0.309701109	1.41E-12	postive
DLAT	ENTPD3-AS1	0.311980457	9.50E-13	postive
DLAT	EXOSC10-AS1	0.31191363	9.61E-13	postive
DLAT	FAM111A-DT	0.419185447	1.07E-22	postive

DLAT	FAM66B	0.31507514	5.51E-13	postive
DLAT	FAM66D	0.335551072	1.27E-14	postive
DLAT	FAM83A-AS1	0.30893244	1.62E-12	postive
DLAT	FAM86B3P	0.318645396	2.91E-13	postive
DLAT	FAM86C2P	0.494269253	3.67E-32	postive
DLAT	FAM86DP	0.413168956	4.91E-22	postive
DLAT	FAM86EP	0.530275927	1.33E-37	postive
DLAT	FAM86JP	0.436553943	1.11E-24	postive
DLAT	FBXL19-AS1	0.334530475	1.54E-14	postive
DLAT	FGD5-AS1	0.583746232	5.33E-47	postive
DLAT	FO393418.1	0.313886112	6.80E-13	postive
DLAT	FOXP4-AS1	0.300799538	6.47E-12	postive
DLAT	FRG1HP	0.476614908	1.01E-29	postive
DLAT	FZD4-DT	0.406382335	2.64E-21	postive
DLAT	GAPDHP72	0.334072673	1.68E-14	postive
DLAT	GASAL1	0.325998702	7.63E-14	postive
DLAT	GATA2-AS1	0.353958105	3.33E-16	postive
DLAT	GCC2-AS1	0.411448517	7.54E-22	postive
DLAT	GMDS-DT	0.371736219	7.86E-18	postive
DLAT	GNRHR2	0.410247739	1.02E-21	postive
DLAT	GPRC5D-AS1	0.33188092	2.55E-14	postive
DLAT	GS1-358P8.4	0.302301517	5.02E-12	postive
DLAT	GSEC	0.424632359	2.63E-23	postive
DLAT	GTF2H2B	0.30080812	6.46E-12	postive
DLAT	GTF2IP1	0.487849974	2.95E-31	postive
DLAT	GTF2IP4	0.453235992	1.07E-26	postive
DLAT	GTF2IP7	0.300852609	6.41E-12	postive
DLAT	GTSE1-DT	0.331646052	2.66E-14	postive
DLAT	GUSBP1	0.325517986	8.34E-14	postive
DLAT	GUSBP2	0.322147342	1.55E-13	postive
DLAT	H2AC10P	0.305635378	2.85E-12	postive
DLAT	H2BC19P	0.306805088	2.33E-12	postive
DLAT	HCG11	0.35113346	5.92E-16	postive
DLAT	HCG18	0.54061638	2.73E-39	postive
DLAT	HCG25	0.338377173	7.37E-15	postive
DLAT	HEIH	0.370434786	1.04E-17	postive
DLAT	HMGB1P5	0.493530138	4.68E-32	postive
DLAT	HSP90AA4P	0.345627872	1.78E-15	postive
DLAT	HSP90AB2P	0.394965615	4.09E-20	postive
DLAT	IDH1-AS1	0.368000028	1.76E-17	postive
DLAT	IQCH-AS1	0.417372045	1.70E-22	postive
DLAT	ITFG2-AS1	0.430050897	6.33E-24	postive
DLAT	JPX	0.354182978	3.18E-16	postive
DLAT	KCTD21-AS1	0.32719069	6.12E-14	postive
DLAT	KIF9-AS1	0.355651092	2.36E-16	postive
DLAT	KRT18P55	0.319991943	2.29E-13	postive
DLAT	KTN1-AS1	0.448946716	3.60E-26	postive
DLAT	LAMTOR5-AS1	0.323415843	1.23E-13	postive
DLAT	LINC002481	0.310692239	1.19E-12	postive
DLAT	LINC00294	0.345581642	1.80E-15	postive

DLAT	LINC00339	0.396180958	3.07E-20	postive
DLAT	LINC00449	0.406555836	2.53E-21	postive
DLAT	LINC00467	0.363440341	4.65E-17	postive
DLAT	LINC00471	0.38826694	1.94E-19	postive
DLAT	LINC00476	0.305835759	2.75E-12	postive
DLAT	LINC00511	0.378747245	1.68E-18	postive
DLAT	LINC00630	0.46112223	1.08E-27	postive
DLAT	LINC00638	0.310732601	1.18E-12	postive
DLAT	LINC00654	0.356695173	1.90E-16	postive
DLAT	LINC00665	0.339995188	5.38E-15	postive
DLAT	LINC00667	0.375354533	3.56E-18	postive
DLAT	LINC00674	0.339833443	5.56E-15	postive
DLAT	LINC00680	0.302824987	4.60E-12	postive
DLAT	LINC00847	0.331904223	2.53E-14	postive
DLAT	LINC00857	0.449357736	3.21E-26	postive
DLAT	LINC00863	0.475622786	1.38E-29	postive
DLAT	LINC00887	0.314320097	6.29E-13	postive
DLAT	LINC00888	0.437532507	8.50E-25	postive
DLAT	LINC00909	0.431316465	4.52E-24	postive
DLAT	LINC00910	0.373381434	5.49E-18	postive
DLAT	LINC00957	0.337498946	8.73E-15	postive
DLAT	LINC00963	0.494648337	3.25E-32	postive
DLAT	LINC01006	0.310988858	1.13E-12	postive
DLAT	LINC01128	0.507055363	5.09E-34	postive
DLAT	LINC01184	0.424503919	2.72E-23	postive
DLAT	LINC01220	0.333227439	1.97E-14	postive
DLAT	LINC01270	0.430035784	6.36E-24	postive
DLAT	LINC01271	0.457769713	2.88E-27	postive
DLAT	LINC01278	0.41496919	3.12E-22	postive
DLAT	LINC01348	0.308841828	1.64E-12	postive
DLAT	LINC01376	0.306026657	2.67E-12	postive
DLAT	LINC01395	0.304237068	3.62E-12	postive
DLAT	LINC01521	0.472399637	3.70E-29	postive
DLAT	LINC01560	0.411313644	7.80E-22	postive
DLAT	LINC01588	0.342566023	3.26E-15	postive
DLAT	LINC01679	0.30136649	5.88E-12	postive
DLAT	LINC01719	0.395889996	3.29E-20	postive
DLAT	LINC01775	0.377915694	2.02E-18	postive
DLAT	LINC01806	0.457612231	3.02E-27	postive
DLAT	LINC01814	0.351508876	5.49E-16	postive
DLAT	LINC01816	0.343691789	2.61E-15	postive
DLAT	LINC01881	0.496300736	1.88E-32	postive
DLAT	LINC01963	0.354620452	2.91E-16	postive
DLAT	LINC02014	0.390159718	1.26E-19	postive
DLAT	LINC02210	0.415345804	2.84E-22	postive
DLAT	LINC02236	0.320143273	2.22E-13	postive
DLAT	LINC02482	0.381254625	9.58E-19	postive
DLAT	LINC02637	0.311794673	9.82E-13	postive
DLAT	LINC02693	0.454071069	8.38E-27	postive
DLAT	LINC02709	0.408961291	1.40E-21	postive

DLAT	LINC02732	0.307120119	2.21E-12	postive
DLAT	LINC02749	0.332142861	2.42E-14	postive
DLAT	LINC02762	0.377390764	2.27E-18	postive
DLAT	LMCD1-AS1	0.337121181	9.38E-15	postive
DLAT	LOH12CR2	0.320940352	1.92E-13	postive
DLAT	LRRC37A16P	0.31285896	8.14E-13	postive
DLAT	LRRC37A17P	0.3846102	4.49E-19	postive
DLAT	LRRC37A6P	0.347322186	1.27E-15	postive
DLAT	LRRC37BP1	0.533622429	3.83E-38	postive
DLAT	MAFG-DT	0.376549962	2.74E-18	postive
DLAT	MAP2K4P1	0.480455595	3.07E-30	postive
DLAT	MAP3K14-AS1	0.415548749	2.70E-22	postive
DLAT	MAP3K2-DT	0.312053668	9.38E-13	postive
DLAT	MAP4K3-DT	0.479760035	3.82E-30	postive
DLAT	MAPK6-DT	0.357603204	1.58E-16	postive
DLAT	MAPKAPK5-AS1	0.484287845	9.18E-31	postive
DLAT	MCM3AP-AS1	0.354555664	2.95E-16	postive
DLAT	MCPH1-AS1	0.30432673	3.56E-12	postive
DLAT	MED14OS	0.332602699	2.22E-14	postive
DLAT	MFF-DT	0.372196665	7.11E-18	postive
DLAT	MID1IP1-AS1	0.371058716	9.10E-18	postive
DLAT	MIPEPP3	0.369535005	1.27E-17	postive
DLAT	MIR4435-2HG	0.330840247	3.10E-14	postive
DLAT	MIR4713HG	0.319364723	2.56E-13	postive
DLAT	MIR762HG	0.300327804	7.00E-12	postive
DLAT	MKLN1-AS	0.388255844	1.95E-19	postive
DLAT	MMADHC-DT	0.301915432	5.36E-12	postive
DLAT	MRPL20-AS1	0.497219556	1.39E-32	postive
DLAT	MRPL45P2	0.335116924	1.38E-14	postive
DLAT	MRPS31P4	0.468157868	1.34E-28	postive
DLAT	MSL3P1	0.36908597	1.39E-17	postive
DLAT	NACA4P	0.383453003	5.84E-19	postive
DLAT	NAIPP3	0.329592601	3.91E-14	postive
DLAT	NAPA-AS1	0.456596656	4.05E-27	postive
DLAT	NBPF25P	0.35903736	1.17E-16	postive
DLAT	NBPF8	0.303663453	3.99E-12	postive
DLAT	NBR2	0.417294061	1.73E-22	postive
DLAT	NCK1-DT	0.366065271	2.66E-17	postive
DLAT	NDUFB2-AS1	0.321359143	1.78E-13	postive
DLAT	NIFK-AS1	0.466855863	1.98E-28	postive
DLAT	NINJ2-AS1	0.35034367	6.94E-16	postive
DLAT	NNT-AS1	0.431111796	4.78E-24	postive
DLAT	NOP14-AS1	0.473930631	2.32E-29	postive
DLAT	NOP53-AS1	0.426164423	1.76E-23	postive
DLAT	NORAD	0.51799492	1.13E-35	postive
DLAT	NPEPPSP1	0.312751185	8.30E-13	postive
DLAT	NRAV	0.556500995	5.34E-42	postive
DLAT	NRSN2-AS1	0.466740968	2.04E-28	postive
DLAT	NUDT16P1	0.476484216	1.06E-29	postive
DLAT	NUP50-DT	0.304932531	3.21E-12	postive

DLAT	NUTM2A-AS1	0.61867572	3.83E-54	postive
DLAT	NUTM2B-AS1	0.351438806	5.56E-16	postive
DLAT	OGFRP1	0.437956804	7.58E-25	postive
DLAT	OIP5-AS1	0.567879453	4.96E-44	postive
DLAT	OLMALINC	0.416133468	2.33E-22	postive
DLAT	OR7E14P	0.309152894	1.56E-12	postive
DLAT	OSER1-DT	0.352385044	4.59E-16	postive
DLAT	OTUD6B-AS1	0.515822694	2.43E-35	postive
DLAT	PARGP1	0.320386509	2.13E-13	postive
DLAT	PAXIP1-AS2	0.307064241	2.23E-12	postive
DLAT	PCBP1-AS1	0.381717914	8.64E-19	postive
DLAT	PDCD4-AS1	0.349852511	7.66E-16	postive
DLAT	PDIA3P1	0.442206355	2.36E-25	postive
DLAT	PINK1-AS	0.46271936	6.76E-28	postive
DLAT	PIPSL	0.454482758	7.45E-27	postive
DLAT	PITPNA-AS1	0.434200978	2.09E-24	postive
DLAT	PLBD1-AS1	0.354201488	3.17E-16	postive
DLAT	PLEKHA8P1	0.482621015	1.55E-30	postive
DLAT	PMS2CL	0.476150287	1.17E-29	postive
DLAT	PMS2P3	0.320934345	1.93E-13	postive
DLAT	PMS2P4	0.442172133	2.39E-25	postive
DLAT	POLH-AS1	0.378853353	1.64E-18	postive
DLAT	POLR2J4	0.307152306	2.20E-12	postive
DLAT	PPP1R12A-AS1	0.312726039	8.34E-13	postive
DLAT	PPP1R14B-AS1	0.357073406	1.76E-16	postive
DLAT	PPT2-EGFL8	0.327642087	5.63E-14	postive
DLAT	PRELID1P1	0.374560802	4.24E-18	postive
DLAT	PRKAR2A-AS1	0.370412035	1.05E-17	postive
DLAT	PRR34	0.315076788	5.51E-13	postive
DLAT	PTENP1	0.494841957	3.05E-32	postive
DLAT	PTMAP5	0.327052391	6.28E-14	postive
DLAT	PTOV1-AS1	0.39508506	3.97E-20	postive
DLAT	PTPRG-AS1	0.318222514	3.14E-13	postive
DLAT	PXN-AS1	0.428665261	9.13E-24	postive
DLAT	RAB11B-AS1	0.307860299	1.94E-12	postive
DLAT	RAB30-DT	0.462063491	8.20E-28	postive
DLAT	RALY-AS1	0.36000207	9.57E-17	postive
DLAT	RAP2C-AS1	0.326019305	7.60E-14	postive
DLAT	RARA-AS1	0.421203309	6.38E-23	postive
DLAT	RASAL2-AS1	0.383529832	5.74E-19	postive
DLAT	RASSF1-AS1	0.312043508	9.40E-13	postive
DLAT	RBM15-AS1	0.47052024	6.55E-29	postive
DLAT	RHPN1-AS1	0.356704547	1.90E-16	postive
DLAT	RNASEH1-AS1	0.54750764	1.90E-40	postive
DLAT	RNF144A-AS1	0.328814172	4.53E-14	postive
DLAT	RNF216P1	0.446307056	7.57E-26	postive
DLAT	RNFT1-DT	0.310404009	1.25E-12	postive
DLAT	ROCK1P1	0.332167552	2.41E-14	postive
DLAT	RP11-101E13.5	0.361279554	7.33E-17	postive
DLAT	RP11-10A14.4	0.340405914	4.97E-15	postive

DLAT	RP11-10C24.1	0.450426053	2.37E-26	postive
DLAT	RP11-159G9.5	0.447980933	4.73E-26	postive
DLAT	RP11-15N24.4	0.360271712	9.05E-17	postive
DLAT	RP11-164P12.4	0.350556033	6.65E-16	postive
DLAT	RP11-175O19.4	0.410801496	8.86E-22	postive
DLAT	RP1-122P22.2	0.500205962	5.15E-33	postive
DLAT	RP11-264E20.1	0.305856753	2.74E-12	postive
DLAT	RP11-282O18.3	0.524037195	1.30E-36	postive
DLAT	RP11-337C18.9	0.32038175	2.13E-13	postive
DLAT	RP11-435O5.2	0.451225657	1.89E-26	postive
DLAT	RP11-439L18.2	0.383035943	6.41E-19	postive
DLAT	RP11-61L19.3	0.3316045	2.68E-14	postive
DLAT	RP11-706O15.1	0.379711271	1.35E-18	postive
DLAT	RP11-815I9.4	0.313084964	7.83E-13	postive
DLAT	RP11-86H7.7	0.305182126	3.08E-12	postive
DLAT	RP4-545L17.11	0.31785282	3.36E-13	postive
DLAT	RP5-1125A11.6	0.368493755	1.58E-17	postive
DLAT	RP9P	0.365464533	3.03E-17	postive
DLAT	RPL12P38	0.33311833	2.01E-14	postive
DLAT	RPL13AP3	0.305022263	3.16E-12	postive
DLAT	RPL13AP7	0.351868558	5.10E-16	postive
DLAT	RPL13P5	0.40506959	3.63E-21	postive
DLAT	RPL23AP53	0.469038833	1.03E-28	postive
DLAT	RPL23AP7	0.525780987	6.89E-37	postive
DLAT	RPL23AP82	0.483084685	1.34E-30	postive
DLAT	RPL36AP21	0.327086427	6.24E-14	postive
DLAT	RPS10P7	0.316212816	4.50E-13	postive
DLAT	RPS18P9	0.372805055	6.23E-18	postive
DLAT	RPS27AP16	0.301313551	5.93E-12	postive
DLAT	RPS4XP16	0.30771613	1.99E-12	postive
DLAT	RRN3P3	0.339069399	6.44E-15	postive
DLAT	RRP7BP	0.374382111	4.41E-18	postive
DLAT	SAMD12-AS1	0.40162407	8.37E-21	postive
DLAT	SBDSP1	0.542006449	1.60E-39	postive
DLAT	SBF2-AS1	0.511960415	9.38E-35	postive
DLAT	SCAMP1-AS1	0.354473242	3.00E-16	postive
DLAT	SCAND2P	0.334414378	1.58E-14	postive
DLAT	SCAT1	0.345451191	1.85E-15	postive
DLAT	SDAD1P1	0.425000181	2.39E-23	postive
DLAT	SDCBP2-AS1	0.309501169	1.46E-12	postive
DLAT	SEPSECS-AS1	0.351541717	5.45E-16	postive
DLAT	SEPTIN4-AS1	0.387300219	2.43E-19	postive
DLAT	SLC25A5-AS1	0.311966332	9.52E-13	postive
DLAT	SLC2A1-AS1	0.32975175	3.80E-14	postive
DLAT	SMG7-AS1	0.390157663	1.26E-19	postive
DLAT	SNAI3-AS1	0.315423008	5.18E-13	postive
DLAT	SNHG1	0.375209113	3.68E-18	postive
DLAT	SNHG11	0.426317196	1.69E-23	postive
DLAT	SNHG16	0.535995391	1.57E-38	postive
DLAT	SNHG17	0.437624421	8.29E-25	postive

DLAT	SNHG21	0.385098283	4.02E-19	postive
DLAT	SNHG29	0.358392145	1.34E-16	postive
DLAT	SNHG32	0.418331514	1.33E-22	postive
DLAT	SNHG4	0.311536306	1.03E-12	postive
DLAT	SNHG6	0.382486336	7.26E-19	postive
DLAT	SNHG7	0.357800998	1.51E-16	postive
DLAT	SNHG8	0.362490833	5.68E-17	postive
DLAT	SP2-AS1	0.418498464	1.28E-22	postive
DLAT	SPINT1-AS1	0.313913325	6.76E-13	postive
DLAT	SREBF2-AS1	0.35134877	5.67E-16	postive
DLAT	ST20-AS1	0.369945519	1.16E-17	postive
DLAT	STAG3L2	0.409839889	1.12E-21	postive
DLAT	STAG3L3	0.340182347	5.19E-15	postive
DLAT	STAG3L4	0.395061895	4.00E-20	postive
DLAT	STARD7-AS1	0.502646807	2.27E-33	postive
DLAT	STK4-AS1	0.451296976	1.85E-26	postive
DLAT	STT3A-AS1	0.309542133	1.45E-12	postive
DLAT	STX18-AS1	0.456586756	4.06E-27	postive
DLAT	SUCLG2-AS1	0.55240019	2.76E-41	postive
DLAT	SUMO2P17	0.430666972	5.38E-24	postive
DLAT	SVIL-AS1	0.566355977	9.38E-44	postive
DLAT	SYS1-DBNDD2	0.35454401	2.96E-16	postive
DLAT	TAF1A-AS1	0.407956438	1.79E-21	postive
DLAT	TAPT1-AS1	0.315527053	5.08E-13	postive
DLAT	TBC1D8-AS1	0.308023976	1.89E-12	postive
DLAT	THAP9-AS1	0.422778379	4.25E-23	postive
DLAT	TMC3-AS1	0.347140135	1.32E-15	postive
DLAT	TMEM202-AS1	0.375257698	3.64E-18	postive
DLAT	TMEM26-AS1	0.330425	3.35E-14	postive
DLAT	TMEM99	0.475677474	1.35E-29	postive
DLAT	TMPO-AS1	0.374247745	4.54E-18	postive
DLAT	TNFRSF10A-AS1	0.334680806	1.50E-14	postive
DLAT	TOB1-AS1	0.393543143	5.71E-20	postive
DLAT	TP73-AS1	0.39177072	8.64E-20	postive
DLAT	TPI1P2	0.316308302	4.42E-13	postive
DLAT	TPM3P9	0.383893612	5.28E-19	postive
DLAT	TRAF3IP2-AS1	0.316343243	4.40E-13	postive
DLAT	TRAM2-AS1	0.464531332	3.95E-28	postive
DLAT	TRAPPC12-AS1	0.397924051	2.03E-20	postive
DLAT	TRIM52-AS1	0.327481431	5.80E-14	postive
DLAT	TSTD3	0.443971501	1.45E-25	postive
DLAT	TTC28-AS1	0.355971259	2.21E-16	postive
DLAT	TTC41P	0.372754566	6.30E-18	postive
DLAT	TUBBP1	0.372031841	7.37E-18	postive
DLAT	TUG1	0.511631788	1.05E-34	postive
DLAT	TYMSOS	0.334269431	1.62E-14	postive
DLAT	U91328.1	0.389289666	1.54E-19	postive
DLAT	UBA6-AS1	0.583330099	6.41E-47	postive
DLAT	UBAC2-AS1	0.328553854	4.75E-14	postive
DLAT	UBE2D3-AS1	0.477894189	6.82E-30	postive

DLAT	UBL7-AS1	0.36506829	3.29E-17	postive
DLAT	UBR5-AS1	0.337849923	8.16E-15	postive
DLAT	UGDH-AS1	0.396897708	2.59E-20	postive
DLAT	UPF3AP2	0.372220264	7.07E-18	postive
DLAT	URB1-AS1	0.322582649	1.43E-13	postive
DLAT	USP27X-AS1	0.380375536	1.17E-18	postive
DLAT	USP46-AS1	0.52756077	3.60E-37	postive
DLAT	UXT-AS1	0.430657303	5.39E-24	postive
DLAT	VPS33B-DT	0.330799893	3.12E-14	postive
DLAT	WAC-AS1	0.414287045	3.71E-22	postive
DLAT	WARS2-AS1	0.40017003	1.19E-20	postive
DLAT	WASH3P	0.308381994	1.78E-12	postive
DLAT	WWC2-AS2	0.337141795	9.35E-15	postive
DLAT	WWTR1-AS1	0.301438034	5.81E-12	postive
DLAT	Z68871.1	0.368540906	1.57E-17	postive
DLAT	Z83851.4	0.413696359	4.30E-22	postive
DLAT	Z95115.1	0.313679962	7.05E-13	postive
DLAT	Z99572.1	0.462217545	7.84E-28	postive
DLAT	ZBED5-AS1	0.372696902	6.38E-18	postive
DLAT	ZBTB11-AS1	0.460693046	1.23E-27	postive
DLAT	ZBTB44-DT	0.318263523	3.12E-13	postive
DLAT	ZFAS1	0.362997012	5.11E-17	postive
DLAT	ZNF137P	0.321126016	1.86E-13	postive
DLAT	ZNF252P	0.562673245	4.31E-43	postive
DLAT	ZNF271P	0.391119019	1.01E-19	postive
DLAT	ZNF286B	0.316232031	4.48E-13	postive
DLAT	ZNF337-AS1	0.325953629	7.70E-14	postive
DLAT	ZNF341-AS1	0.360641124	8.38E-17	postive
DLAT	ZNF503-AS2	0.405134959	3.57E-21	postive
DLAT	ZNF542P	0.334412803	1.58E-14	postive
DLAT	ZNF561-AS1	0.408319045	1.64E-21	postive
DLAT	ZNF674-AS1	0.330015245	3.62E-14	postive
DLAT	ZNF687-AS1	0.436803267	1.04E-24	postive
DLAT	ZNF702P	0.330149916	3.53E-14	postive
DLAT	ZNF815P	0.313115684	7.78E-13	postive
DLAT	ZNRD2-AS1	0.345981938	1.66E-15	postive
DLAT	ZNRF2P1	0.34637014	1.54E-15	postive
DLAT	ZNRF2P2	0.337687243	8.42E-15	postive
DLAT	ZSCAN12P1	0.388976942	1.65E-19	postive
DLD	ABALON	0.305749801	2.79E-12	postive
DLD	AC000068.1	0.336408804	1.08E-14	postive
DLD	AC002116.2	0.378636391	1.72E-18	postive
DLD	AC002467.1	0.573949789	3.79E-45	postive
DLD	AC004241.3	0.332193971	2.40E-14	postive
DLD	AC004596.1	0.386612383	2.84E-19	postive
DLD	AC004771.2	0.336267912	1.11E-14	postive
DLD	AC004803.1	0.371919853	7.55E-18	postive
DLD	AC004812.2	0.350339154	6.95E-16	postive
DLD	AC004893.11	0.442757759	2.03E-25	postive
DLD	AC004917.1	0.309356365	1.50E-12	postive

DLD	AC004918.5	0.371964381	7.48E-18	postive
DLD	AC004943.2	0.370665046	9.92E-18	postive
DLD	AC004980.1	0.339126876	6.37E-15	postive
DLD	AC005034.4	0.484918846	7.51E-31	postive
DLD	AC005034.6	0.359125204	1.15E-16	postive
DLD	AC005050.1	0.314429289	6.17E-13	postive
DLD	AC005229.4	0.405727664	3.09E-21	postive
DLD	AC005288.1	0.50719783	4.85E-34	postive
DLD	AC005562.1	0.530303552	1.31E-37	postive
DLD	AC005586.1	0.493924422	4.11E-32	postive
DLD	AC005670.3	0.412362811	6.01E-22	postive
DLD	AC005696.1	0.314650066	5.94E-13	postive
DLD	AC005841.1	0.327809366	5.46E-14	postive
DLD	AC006001.2	0.450755804	2.16E-26	postive
DLD	AC006064.2	0.349752446	7.82E-16	postive
DLD	AC006064.3	0.308703804	1.68E-12	postive
DLD	AC006122.1	0.33146188	2.76E-14	postive
DLD	AC006252.1	0.306127045	2.62E-12	postive
DLD	AC006333.1	0.480406374	3.12E-30	postive
DLD	AC006449.2	0.323787534	1.15E-13	postive
DLD	AC006504.5	0.366335095	2.52E-17	postive
DLD	AC006547.1	0.313164629	7.72E-13	postive
DLD	AC007032.1	0.401623173	8.37E-21	postive
DLD	AC007342.4	0.330320474	3.41E-14	postive
DLD	AC008014.1	0.371276684	8.68E-18	postive
DLD	AC008267.5	0.330790978	3.13E-14	postive
DLD	AC008438.1	0.303111184	4.38E-12	postive
DLD	AC008443.1	0.397445246	2.27E-20	postive
DLD	AC008771.1	0.308908323	1.62E-12	postive
DLD	AC008915.3	0.316056765	4.63E-13	postive
DLD	AC008966.1	0.408389438	1.61E-21	postive
DLD	AC009061.2	0.331685111	2.64E-14	postive
DLD	AC009226.1	0.345768286	1.73E-15	postive
DLD	AC009237.14	0.315761526	4.88E-13	postive
DLD	AC009275.1	0.330537459	3.28E-14	postive
DLD	AC009403.1	0.341790694	3.79E-15	postive
DLD	AC009630.1	0.360209288	9.17E-17	postive
DLD	AC009690.2	0.30719708	2.18E-12	postive
DLD	AC009779.3	0.472295898	3.82E-29	postive
DLD	AC009831.1	0.325983922	7.65E-14	postive
DLD	AC009948.3	0.360180521	9.22E-17	postive
DLD	AC010186.2	0.320028479	2.27E-13	postive
DLD	AC010442.1	0.354222379	3.16E-16	postive
DLD	AC010595.1	0.355851671	2.26E-16	postive
DLD	AC010655.4	0.379881037	1.30E-18	postive
DLD	AC012063.1	0.331714417	2.63E-14	postive
DLD	AC012073.1	0.334961382	1.42E-14	postive
DLD	AC012146.1	0.317089981	3.85E-13	postive
DLD	AC012313.1	0.354753395	2.83E-16	postive
DLD	AC012555.1	0.353111355	3.96E-16	postive

DLD	AC015922.2	0.397895759	2.04E-20	postive
DLD	AC015922.3	0.321089361	1.87E-13	postive
DLD	AC016065.1	0.331966958	2.51E-14	postive
DLD	AC016394.2	0.329979202	3.64E-14	postive
DLD	AC016727.1	0.32164273	1.69E-13	postive
DLD	AC016747.1	0.378961329	1.60E-18	postive
DLD	AC016876.2	0.302581464	4.79E-12	postive
DLD	AC016876.3	0.396031211	3.18E-20	postive
DLD	AC016888.1	0.353229928	3.87E-16	postive
DLD	AC018521.5	0.385892074	3.35E-19	postive
DLD	AC018647.2	0.390869499	1.07E-19	postive
DLD	AC019069.1	0.328130872	5.14E-14	postive
DLD	AC020904.2	0.327467109	5.82E-14	postive
DLD	AC020915.1	0.351835008	5.13E-16	postive
DLD	AC020915.3	0.364840916	3.46E-17	postive
DLD	AC021028.1	0.352448708	4.53E-16	postive
DLD	AC021224.1	0.374603327	4.20E-18	postive
DLD	AC022101.1	0.307931365	1.92E-12	postive
DLD	AC022150.2	0.30090397	6.35E-12	postive
DLD	AC022211.3	0.323204485	1.27E-13	postive
DLD	AC023043.1	0.337884526	8.10E-15	postive
DLD	AC024267.7	0.337697202	8.40E-15	postive
DLD	AC025580.2	0.325663799	8.12E-14	postive
DLD	AC025822.2	0.310108574	1.32E-12	postive
DLD	AC026471.1	0.397475991	2.26E-20	postive
DLD	AC027097.1	0.366968497	2.20E-17	postive
DLD	AC027307.2	0.348307029	1.05E-15	postive
DLD	AC046134.2	0.377166911	2.39E-18	postive
DLD	AC064807.1	0.388961049	1.66E-19	postive
DLD	AC067852.2	0.527228653	4.07E-37	postive
DLD	AC068338.2	0.307349718	2.12E-12	postive
DLD	AC068870.2	0.338586205	7.08E-15	postive
DLD	AC073046.1	0.361597144	6.85E-17	postive
DLD	AC073073.2	0.534087259	3.22E-38	postive
DLD	AC073254.1	0.318029617	3.25E-13	postive
DLD	AC073508.3	0.310054049	1.33E-12	postive
DLD	AC073529.1	0.376776349	2.60E-18	postive
DLD	AC073569.2	0.467445482	1.66E-28	postive
DLD	AC073585.1	0.359808146	9.97E-17	postive
DLD	AC073611.1	0.31291141	8.07E-13	postive
DLD	AC078846.1	0.350513965	6.71E-16	postive
DLD	AC079174.1	0.304213576	3.63E-12	postive
DLD	AC079807.1	0.308647508	1.70E-12	postive
DLD	AC080023.1	0.357612885	1.57E-16	postive
DLD	AC080112.2	0.359065201	1.16E-16	postive
DLD	AC083799.1	0.443189549	1.80E-25	postive
DLD	AC083862.2	0.374329124	4.46E-18	postive
DLD	AC087071.2	0.417738024	1.55E-22	postive
DLD	AC087501.4	0.304060166	3.73E-12	postive
DLD	AC087645.2	0.310441846	1.24E-12	postive

DLD	AC090114.2	0.390814511	1.08E-19	postive
DLD	AC090204.1	0.305297617	3.02E-12	postive
DLD	AC090587.1	0.332334137	2.34E-14	postive
DLD	AC090617.5	0.339066425	6.45E-15	postive
DLD	AC090912.1	0.315382073	5.22E-13	postive
DLD	AC091057.1	0.36936641	1.31E-17	postive
DLD	AC091390.5	0.355706659	2.33E-16	postive
DLD	AC091729.3	0.397818266	2.08E-20	postive
DLD	AC092115.4	0.309554913	1.45E-12	postive
DLD	AC092171.2	0.339957239	5.42E-15	postive
DLD	AC092295.2	0.334054349	1.69E-14	postive
DLD	AC092368.3	0.35829207	1.37E-16	postive
DLD	AC092614.1	0.33373165	1.79E-14	postive
DLD	AC092718.4	0.338405191	7.33E-15	postive
DLD	AC092910.3	0.304838618	3.27E-12	postive
DLD	AC092944.1	0.311786741	9.83E-13	postive
DLD	AC093157.1	0.414801567	3.26E-22	postive
DLD	AC093484.4	0.311664494	1.00E-12	postive
DLD	AC093627.5	0.331931072	2.52E-14	postive
DLD	AC093673.1	0.425924991	1.88E-23	postive
DLD	AC096536.2	0.34867508	9.71E-16	postive
DLD	AC096677.1	0.314059381	6.59E-13	postive
DLD	AC096772.6	0.314164744	6.47E-13	postive
DLD	AC097359.2	0.369938616	1.16E-17	postive
DLD	AC098484.1	0.406870257	2.34E-21	postive
DLD	AC098820.2	0.315737645	4.90E-13	postive
DLD	AC099548.2	0.379132301	1.54E-18	postive
DLD	AC099850.2	0.399246231	1.48E-20	postive
DLD	AC099850.4	0.490938818	1.09E-31	postive
DLD	AC100861.1	0.465035514	3.40E-28	postive
DLD	AC105036.3	0.434254247	2.06E-24	postive
DLD	AC105285.1	0.433040178	2.86E-24	postive
DLD	AC106786.2	0.306685233	2.38E-12	postive
DLD	AC106791.1	0.331430416	2.77E-14	postive
DLD	AC106795.1	0.459513858	1.73E-27	postive
DLD	AC107027.3	0.324641412	9.80E-14	postive
DLD	AC107068.1	0.31882637	2.82E-13	postive
DLD	AC107214.1	0.386786994	2.73E-19	postive
DLD	AC107959.3	0.351008032	6.07E-16	postive
DLD	AC107982.3	0.315034453	5.55E-13	postive
DLD	AC108010.1	0.342094297	3.58E-15	postive
DLD	AC109347.1	0.335313716	1.33E-14	postive
DLD	AC109454.2	0.314063386	6.59E-13	postive
DLD	AC112220.2	0.450141227	2.57E-26	postive
DLD	AC114271.1	0.443869672	1.49E-25	postive
DLD	AC115618.1	0.30056524	6.73E-12	postive
DLD	AC115618.2	0.378920142	1.62E-18	postive
DLD	AC116913.1	0.362691766	5.45E-17	postive
DLD	AC124283.2	0.316987702	3.92E-13	postive
DLD	AC125257.1	0.410723438	9.03E-22	postive

DLD	AC125494.1	0.314412147	6.19E-13	postive
DLD	AC125807.2	0.365088085	3.28E-17	postive
DLD	AC126365.1	0.310683505	1.19E-12	postive
DLD	AC127521.1	0.305579841	2.88E-12	postive
DLD	AC131009.3	0.447042656	6.16E-26	postive
DLD	AC132008.2	0.353918329	3.36E-16	postive
DLD	AC133528.2	0.336970306	9.66E-15	postive
DLD	AC135050.1	0.366758564	2.30E-17	postive
DLD	AC135178.4	0.307891263	1.93E-12	postive
DLD	AC139887.2	0.305058107	3.15E-12	postive
DLD	AC141586.1	0.442325999	2.29E-25	postive
DLD	AC144652.1	0.355041002	2.67E-16	postive
DLD	AC145098.1	0.306584057	2.42E-12	postive
DLD	AC245140.2	0.309592235	1.44E-12	postive
DLD	ACVR2B-AS1	0.317616697	3.50E-13	postive
DLD	AL008726.1	0.384808113	4.29E-19	postive
DLD	AL009179.1	0.329305608	4.13E-14	postive
DLD	AL024498.1	0.322253669	1.52E-13	postive
DLD	AL024507.3	0.344609249	2.18E-15	postive
DLD	AL031775.1	0.302437159	4.91E-12	postive
DLD	AL031985.3	0.309302385	1.52E-12	postive
DLD	AL035587.1	0.34511129	1.97E-15	postive
DLD	AL049597.2	0.340425299	4.95E-15	postive
DLD	AL049779.1	0.322752042	1.38E-13	postive
DLD	AL049796.1	0.316007501	4.67E-13	postive
DLD	AL049840.4	0.401736858	8.14E-21	postive
DLD	AL049840.5	0.390858019	1.07E-19	postive
DLD	AL050309.1	0.300564869	6.73E-12	postive
DLD	AL078581.2	0.336695636	1.02E-14	postive
DLD	AL078644.2	0.421555373	5.83E-23	postive
DLD	AL118506.1	0.425023445	2.37E-23	postive
DLD	AL118558.3	0.326868037	6.50E-14	postive
DLD	AL121603.2	0.319578925	2.46E-13	postive
DLD	AL121944.2	0.496080484	2.03E-32	postive
DLD	AL133153.2	0.37420806	4.58E-18	postive
DLD	AL133215.2	0.313231471	7.63E-13	postive
DLD	AL136040.1	0.338470039	7.24E-15	postive
DLD	AL138724.2	0.395626417	3.50E-20	postive
DLD	AL139035.1	0.3736761	5.15E-18	postive
DLD	AL139039.3	0.320333201	2.15E-13	postive
DLD	AL139289.1	0.335549548	1.27E-14	postive
DLD	AL139384.1	0.305515964	2.91E-12	postive
DLD	AL158212.3	0.307396009	2.11E-12	postive
DLD	AL160006.1	0.320564917	2.06E-13	postive
DLD	AL162231.2	0.312796696	8.23E-13	postive
DLD	AL162595.1	0.416150859	2.32E-22	postive
DLD	AL353593.1	0.307256457	2.16E-12	postive
DLD	AL353708.1	0.304087712	3.71E-12	postive
DLD	AL353743.1	0.304865453	3.25E-12	postive
DLD	AL354696.1	0.318306864	3.09E-13	postive

DLD	AL354696.2	0.343082947	2.95E-15	postive
DLD	AL354707.1	0.309131022	1.56E-12	postive
DLD	AL354740.1	0.398165099	1.92E-20	postive
DLD	AL354920.1	0.386307963	3.05E-19	postive
DLD	AL355574.1	0.400306851	1.15E-20	postive
DLD	AL355802.3	0.320237887	2.19E-13	postive
DLD	AL356481.1	0.311422478	1.05E-12	postive
DLD	AL358115.1	0.302235529	5.08E-12	postive
DLD	AL359643.2	0.306593958	2.42E-12	postive
DLD	AL359643.3	0.369888224	1.17E-17	postive
DLD	AL359715.3	0.384370321	4.74E-19	postive
DLD	AL360270.1	0.337203734	9.24E-15	postive
DLD	AL365181.3	0.313605319	7.14E-13	postive
DLD	AL391121.1	0.326982399	6.36E-14	postive
DLD	AL391832.3	0.371977205	7.46E-18	postive
DLD	AL441992.2	0.436410592	1.15E-24	postive
DLD	AL445231.1	0.341135957	4.31E-15	postive
DLD	AL445524.1	0.304330219	3.56E-12	postive
DLD	AL450326.1	0.375929144	3.14E-18	postive
DLD	AL513165.1	0.326270421	7.26E-14	postive
DLD	AL513165.2	0.383487313	5.79E-19	postive
DLD	AL513550.1	0.378007643	1.98E-18	postive
DLD	AL583722.1	0.311536865	1.03E-12	postive
DLD	AL590666.2	0.328510039	4.79E-14	postive
DLD	AL591684.2	0.330104248	3.56E-14	postive
DLD	AL592166.1	0.334667206	1.50E-14	postive
DLD	AL606489.1	0.375548826	3.41E-18	postive
DLD	AL606834.1	0.329485073	3.99E-14	postive
DLD	AL645568.1	0.355927282	2.23E-16	postive
DLD	AL731577.2	0.465838449	2.68E-28	postive
DLD	AL928654.1	0.345416193	1.86E-15	postive
DLD	ALG1L13P	0.351130085	5.92E-16	postive
DLD	ALOX12-AS1	0.33292839	2.09E-14	postive
DLD	AMZ2P1	0.329073405	4.31E-14	postive
DLD	ANP32AP1	0.364692912	3.57E-17	postive
DLD	ANP32BP1	0.363551562	4.54E-17	postive
DLD	AP000442.1	0.328279061	5.00E-14	postive
DLD	AP000487.1	0.317522412	3.56E-13	postive
DLD	AP000759.1	0.499078286	7.50E-33	postive
DLD	AP001107.1	0.352311517	4.66E-16	postive
DLD	AP001160.3	0.358778011	1.23E-16	postive
DLD	AP001318.2	0.318380257	3.05E-13	postive
DLD	AP001372.2	0.376499876	2.77E-18	postive
DLD	AP001432.1	0.315429941	5.17E-13	postive
DLD	AP001453.4	0.305528984	2.90E-12	postive
DLD	AP001469.3	0.308960089	1.61E-12	postive
DLD	AP001505.1	0.305461826	2.94E-12	postive
DLD	AP002387.2	0.302658101	4.73E-12	postive
DLD	AP002748.4	0.38873976	1.74E-19	postive
DLD	AP002761.4	0.320564312	2.06E-13	postive

DLD	AP003059.1	0.310796748	1.17E-12	postive
DLD	AP003068.2	0.391991901	8.21E-20	postive
DLD	AP003119.2	0.31271867	8.35E-13	postive
DLD	AP003486.1	0.322337705	1.49E-13	postive
DLD	AP003680.1	0.303498265	4.10E-12	postive
DLD	AP005482.4	0.313840509	6.85E-13	postive
DLD	APTR	0.322223875	1.52E-13	postive
DLD	ARHGEF35-AS1	0.427623644	1.20E-23	postive
DLD	ARMCX5-GPRASP2	0.473532575	2.62E-29	postive
DLD	ARRDC1-AS1	0.412448218	5.88E-22	postive
DLD	ATF4P4	0.368806705	1.48E-17	postive
DLD	ATP5F1AP2	0.314047235	6.61E-13	postive
DLD	ATP6V0E2-AS1	0.312702642	8.37E-13	postive
DLD	BAALC-AS1	0.346821105	1.41E-15	postive
DLD	BCDIN3D-AS1	0.346226472	1.58E-15	postive
DLD	BEND3P1	0.310235487	1.29E-12	postive
DLD	BHLHE40-AS1	0.332658501	2.20E-14	postive
DLD	BMS1P20	0.412141216	6.35E-22	postive
DLD	BNIP3P1	0.325436687	8.47E-14	postive
DLD	BVES-AS1	0.32159581	1.71E-13	postive
DLD	BX322557.10	0.34857313	9.91E-16	postive
DLD	C18orf65	0.322477263	1.46E-13	postive
DLD	C22orf46	0.342325657	3.42E-15	postive
DLD	C2CD4D-AS1	0.392606294	7.11E-20	postive
DLD	C5orf66	0.300382878	6.94E-12	postive
DLD	CA5BP1	0.426385246	1.66E-23	postive
DLD	CATSPER2P1	0.463210192	5.85E-28	postive
DLD	CCNT2-AS1	0.402834351	6.25E-21	postive
DLD	CCT6P1	0.350729853	6.42E-16	postive
DLD	CD27-AS1	0.391952505	8.28E-20	postive
DLD	CD99P1	0.369618485	1.24E-17	postive
DLD	CENPBD1P1	0.470491645	6.61E-29	postive
DLD	CHKB-DT	0.31196387	9.53E-13	postive
DLD	CIDECP1	0.459579729	1.70E-27	postive
DLD	COX10-AS1	0.339148592	6.35E-15	postive
DLD	CR381653.1	0.315079464	5.50E-13	postive
DLD	CRYZL2P	0.500704848	4.36E-33	postive
DLD	CTA-989H11.1	0.372387243	6.82E-18	postive
DLD	CTBP1-DT	0.492636494	6.27E-32	postive
DLD	CTD-2245F17.3	0.308806159	1.65E-12	postive
DLD	CTD-2574D22.4	0.332804653	2.14E-14	postive
DLD	CU633906.2	0.304536101	3.44E-12	postive
DLD	CYTOR	0.316842128	4.02E-13	postive
DLD	DANCR	0.305918724	2.71E-12	postive
DLD	DARS-AS1	0.35034189	6.94E-16	postive
DLD	DBIL5P	0.317237856	3.75E-13	postive
DLD	DCST1-AS1	0.379071566	1.56E-18	postive
DLD	DDX11-AS1	0.324542157	9.98E-14	postive
DLD	DENND10P1	0.381821056	8.44E-19	postive
DLD	DGCR11	0.427833958	1.14E-23	postive

DLD	DHRS4-AS1	0.356649492	1.92E-16	postive
DLD	DLEU1	0.324344367	1.03E-13	postive
DLD	DPY19L2P3	0.35027119	7.04E-16	postive
DLD	DSTNP2	0.406693606	2.44E-21	postive
DLD	EBLN3P	0.487808233	2.99E-31	postive
DLD	ECI2-DT	0.377570671	2.18E-18	postive
DLD	EDRF1-DT	0.321830099	1.64E-13	postive
DLD	EIF1B-AS1	0.316823647	4.04E-13	postive
DLD	EIF3J-DT	0.366188101	2.60E-17	postive
DLD	ELF3-AS1	0.41880411	1.18E-22	postive
DLD	EMC3-AS1	0.312556657	8.59E-13	postive
DLD	EMSLR	0.488221825	2.61E-31	postive
DLD	ENTPD3-AS1	0.30582812	2.76E-12	postive
DLD	EP400P1	0.301847417	5.42E-12	postive
DLD	FAM111A-DT	0.419096255	1.10E-22	postive
DLD	FAM222A-AS1	0.372787054	6.25E-18	postive
DLD	FAM86C2P	0.39728462	2.36E-20	postive
DLD	FAM86DP	0.413915077	4.07E-22	postive
DLD	FAM86EP	0.451662197	1.67E-26	postive
DLD	FAM86JP	0.427109611	1.38E-23	postive
DLD	FGD5-AS1	0.528344579	2.70E-37	postive
DLD	FGF7P3	0.340729357	4.67E-15	postive
DLD	FGF7P6	0.307158979	2.19E-12	postive
DLD	FRG1HP	0.479745497	3.83E-30	postive
DLD	FTH1P10	0.343693673	2.61E-15	postive
DLD	FZD4-DT	0.388786438	1.73E-19	postive
DLD	GAPDHP72	0.370843554	9.54E-18	postive
DLD	GCC2-AS1	0.371035547	9.15E-18	postive
DLD	GMDS-DT	0.301611872	5.64E-12	postive
DLD	GNRHR2	0.362050606	6.23E-17	postive
DLD	GPRC5D-AS1	0.395488667	3.61E-20	postive
DLD	GSEC	0.388994393	1.64E-19	postive
DLD	GTF2IP1	0.549464602	8.81E-41	postive
DLD	GTF2IP4	0.435088723	1.65E-24	postive
DLD	GTF2IRD1P1	0.368277711	1.66E-17	postive
DLD	H2AZ1-DT	0.327916037	5.35E-14	postive
DLD	H2BC19P	0.315678142	4.95E-13	postive
DLD	HCG18	0.481998044	1.89E-30	postive
DLD	HCG25	0.351578433	5.41E-16	postive
DLD	HCP5	0.347485849	1.23E-15	postive
DLD	HEIH	0.374936952	3.90E-18	postive
DLD	HMGB1P5	0.467909202	1.44E-28	postive
DLD	HNRNPA1P6	0.321024766	1.90E-13	postive
DLD	HNRNPKP3	0.310591671	1.21E-12	postive
DLD	HSP90AA4P	0.407812634	1.86E-21	postive
DLD	HSP90AA6P	0.347303469	1.28E-15	postive
DLD	HSP90AB2P	0.405212036	3.51E-21	postive
DLD	IQCH-AS1	0.352770446	4.25E-16	postive
DLD	ITFG2-AS1	0.315397674	5.20E-13	postive
DLD	JPX	0.348851696	9.37E-16	postive

DLD	KATNBL1P6	0.306411052	2.50E-12	postive
DLD	KDM7A-DT	0.359615177	1.04E-16	postive
DLD	KMT2E-AS1	0.343904996	2.51E-15	postive
DLD	KRT18P55	0.333380712	1.92E-14	postive
DLD	KRT8P12	0.314458989	6.14E-13	postive
DLD	KTN1-AS1	0.438294446	6.91E-25	postive
DLD	LINC00205	0.316558518	4.23E-13	postive
DLD	LINC002481	0.308429206	1.76E-12	postive
DLD	LINC00339	0.338960129	6.58E-15	postive
DLD	LINC00467	0.427817461	1.14E-23	postive
DLD	LINC00471	0.339843419	5.55E-15	postive
DLD	LINC00476	0.319206571	2.63E-13	postive
DLD	LINC00511	0.373252356	5.65E-18	postive
DLD	LINC00615	0.312893831	8.09E-13	postive
DLD	LINC00628	0.311993763	9.48E-13	postive
DLD	LINC00630	0.470419033	6.75E-29	postive
DLD	LINC00654	0.330513178	3.29E-14	postive
DLD	LINC00667	0.340202572	5.17E-15	postive
DLD	LINC00847	0.372242383	7.04E-18	postive
DLD	LINC00857	0.400627858	1.06E-20	postive
DLD	LINC00863	0.453336797	1.03E-26	postive
DLD	LINC00888	0.446829437	6.54E-26	postive
DLD	LINC00909	0.398829705	1.64E-20	postive
DLD	LINC00910	0.33288262	2.11E-14	postive
DLD	LINC00933	0.329868409	3.72E-14	postive
DLD	LINC00963	0.462939097	6.33E-28	postive
DLD	LINC01003	0.353397291	3.74E-16	postive
DLD	LINC01006	0.301095692	6.15E-12	postive
DLD	LINC01011	0.394991926	4.06E-20	postive
DLD	LINC01094	0.348681391	9.70E-16	postive
DLD	LINC01128	0.434029441	2.19E-24	postive
DLD	LINC01184	0.416086895	2.35E-22	postive
DLD	LINC01270	0.357696514	1.55E-16	postive
DLD	LINC01271	0.363162512	4.93E-17	postive
DLD	LINC01278	0.423295816	3.72E-23	postive
DLD	LINC01521	0.315621718	5.00E-13	postive
DLD	LINC01560	0.345738725	1.74E-15	postive
DLD	LINC01588	0.305442493	2.95E-12	postive
DLD	LINC01679	0.30498819	3.18E-12	postive
DLD	LINC01719	0.326147183	7.43E-14	postive
DLD	LINC01775	0.416319575	2.22E-22	postive
DLD	LINC01806	0.369405314	1.30E-17	postive
DLD	LINC01881	0.471311516	5.15E-29	postive
DLD	LINC02014	0.41477192	3.28E-22	postive
DLD	LINC02210	0.348538939	9.98E-16	postive
DLD	LINC02236	0.317113458	3.83E-13	postive
DLD	LINC02280	0.332321088	2.34E-14	postive
DLD	LINC02298	0.31945699	2.52E-13	postive
DLD	LINC02637	0.39198556	8.22E-20	postive
DLD	LINC02693	0.445167897	1.04E-25	postive

DLD	LINC02709	0.406857588	2.35E-21	postive
DLD	LOXL1-AS1	0.302568065	4.80E-12	postive
DLD	LRRC37A17P	0.343074268	2.95E-15	postive
DLD	LRRC37BP1	0.450226668	2.51E-26	postive
DLD	MAP2K4P1	0.457782452	2.87E-27	postive
DLD	MAP3K14-AS1	0.444302831	1.32E-25	postive
DLD	MAP4K3-DT	0.475409139	1.47E-29	postive
DLD	MAPKAPK5-AS1	0.514783397	3.50E-35	postive
DLD	MCM3AP-AS1	0.349972746	7.48E-16	postive
DLD	MCPH1-AS1	0.323279396	1.26E-13	postive
DLD	MED14OS	0.337182262	9.27E-15	postive
DLD	MID1IP1-AS1	0.339614565	5.80E-15	postive
DLD	MIPEPP3	0.330470722	3.32E-14	postive
DLD	MIR22HG	0.304528675	3.44E-12	postive
DLD	MIR4435-2HG	0.420397857	7.85E-23	postive
DLD	MKLN1-AS	0.544498723	6.12E-40	postive
DLD	MRPL20-AS1	0.445691074	8.99E-26	postive
DLD	MRPL45P2	0.305824533	2.76E-12	postive
DLD	MRPS31P4	0.402350634	7.02E-21	postive
DLD	MSL3P1	0.363937729	4.19E-17	postive
DLD	NACA4P	0.419586764	9.67E-23	postive
DLD	NAIPP1	0.327651931	5.62E-14	postive
DLD	NAIPP3	0.327903822	5.36E-14	postive
DLD	NAPA-AS1	0.412973256	5.15E-22	postive
DLD	NBPF25P	0.370048525	1.13E-17	postive
DLD	NBPF8	0.325854178	7.84E-14	postive
DLD	NBR2	0.350233744	7.10E-16	postive
DLD	NCK1-DT	0.377903538	2.03E-18	postive
DLD	NDUFB2-AS1	0.480541388	2.99E-30	postive
DLD	NIFK-AS1	0.383579719	5.67E-19	postive
DLD	NNT-AS1	0.353517767	3.65E-16	postive
DLD	NOP14-AS1	0.439144661	5.48E-25	postive
DLD	NORAD	0.426252452	1.72E-23	postive
DLD	NRAV	0.538034415	7.29E-39	postive
DLD	NRSN2-AS1	0.490404792	1.29E-31	postive
DLD	NSUN5P1	0.334194753	1.64E-14	postive
DLD	NSUN5P2	0.344313316	2.31E-15	postive
DLD	NT5C3AP1	0.344970466	2.03E-15	postive
DLD	NUDT16P1	0.562308535	5.01E-43	postive
DLD	NUP50-DT	0.338362133	7.39E-15	postive
DLD	NUTM2A-AS1	0.575916124	1.63E-45	postive
DLD	NUTM2B-AS1	0.316498672	4.28E-13	postive
DLD	OGFRP1	0.438584979	6.38E-25	postive
DLD	OIP5-AS1	0.516484143	1.93E-35	postive
DLD	OLMALINC	0.336078489	1.15E-14	postive
DLD	OR2A1-AS1	0.355833039	2.27E-16	postive
DLD	OTUD6B-AS1	0.463222523	5.82E-28	postive
DLD	PAXIP1-AS1	0.341810018	3.78E-15	postive
DLD	PAXIP1-AS2	0.374980046	3.87E-18	postive
DLD	PDIA3P1	0.43379469	2.33E-24	postive

DLD	PGAM1P8	0.332716069	2.17E-14	postive
DLD	PINK1-AS	0.322252979	1.52E-13	postive
DLD	PIPSL	0.475337457	1.50E-29	postive
DLD	PITPNA-AS1	0.415947103	2.44E-22	postive
DLD	PLBD1-AS1	0.304048216	3.74E-12	postive
DLD	PLEKHA8P1	0.532152771	6.62E-38	postive
DLD	PMS2CL	0.463543889	5.30E-28	postive
DLD	PMS2P3	0.435166869	1.61E-24	postive
DLD	PMS2P4	0.512042639	9.12E-35	postive
DLD	POLH-AS1	0.3654197	3.06E-17	postive
DLD	POLR2J4	0.333268198	1.96E-14	postive
DLD	POT1-AS1	0.329711802	3.83E-14	postive
DLD	PPP1R12A-AS1	0.408952613	1.40E-21	postive
DLD	PPP1R26-AS1	0.31645355	4.31E-13	postive
DLD	PPP3CB-AS1	0.330108972	3.55E-14	postive
DLD	PPT2-EGFL8	0.311750251	9.89E-13	postive
DLD	PRELID1P1	0.420258604	8.14E-23	postive
DLD	PRKAR2A-AS1	0.305796059	2.77E-12	postive
DLD	PRRT3-AS1	0.300823598	6.44E-12	postive
DLD	PSMB8-AS1	0.305933562	2.71E-12	postive
DLD	PTENP1	0.493172779	5.26E-32	postive
DLD	PTMAP5	0.382576429	7.12E-19	postive
DLD	PTOV1-AS1	0.394072214	5.04E-20	postive
DLD	PXN-AS1	0.39468518	4.37E-20	postive
DLD	RAB30-DT	0.339015146	6.51E-15	postive
DLD	RAP2C-AS1	0.335874975	1.19E-14	postive
DLD	RASAL2-AS1	0.44754983	5.34E-26	postive
DLD	RBM15-AS1	0.509999315	1.85E-34	postive
DLD	RNASEH1-AS1	0.400148245	1.19E-20	postive
DLD	RNF216P1	0.517425426	1.38E-35	postive
DLD	RP11-101E13.5	0.328225378	5.05E-14	postive
DLD	RP11-10C24.1	0.444937603	1.11E-25	postive
DLD	RP11-159G9.5	0.376929726	2.51E-18	postive
DLD	RP11-164P12.4	0.342392465	3.37E-15	postive
DLD	RP11-175O19.4	0.377102911	2.42E-18	postive
DLD	RP11-226L15.5	0.316560888	4.23E-13	postive
DLD	RP1-122P22.2	0.434436543	1.96E-24	postive
DLD	RP11-282O18.3	0.449794237	2.84E-26	postive
DLD	RP11-435O5.2	0.467605612	1.58E-28	postive
DLD	RP11-439L18.2	0.344873504	2.07E-15	postive
DLD	RP11-539I5.1	0.337197628	9.25E-15	postive
DLD	RP11-58E21.3	0.343113118	2.93E-15	postive
DLD	RP11-86H7.7	0.319038878	2.71E-13	postive
DLD	RP5-1125A11.6	0.300107524	7.26E-12	postive
DLD	RP9P	0.320531229	2.07E-13	postive
DLD	RPL12P38	0.372478336	6.69E-18	postive
DLD	RPL13AP3	0.301344909	5.90E-12	postive
DLD	RPL13AP7	0.352804794	4.22E-16	postive
DLD	RPL13P5	0.430296365	5.93E-24	postive
DLD	RPL23AP53	0.449961703	2.71E-26	postive

DLD	RPL23AP7	0.501818359	3.00E-33	postive
DLD	RPL23AP82	0.513419143	5.65E-35	postive
DLD	RPL6P27	0.400464979	1.11E-20	postive
DLD	RPS10P7	0.304716849	3.33E-12	postive
DLD	RPS18P9	0.342088035	3.58E-15	postive
DLD	RPS27AP16	0.34558956	1.80E-15	postive
DLD	RRN3P3	0.342919352	3.04E-15	postive
DLD	RRP7BP	0.337440369	8.83E-15	postive
DLD	SAMD12-AS1	0.323085288	1.30E-13	postive
DLD	SBDSP1	0.662347112	1.90E-64	postive
DLD	SBF2-AS1	0.465587358	2.89E-28	postive
DLD	SCAMP1-AS1	0.403817796	4.92E-21	postive
DLD	SDAD1P1	0.323108208	1.30E-13	postive
DLD	SDHAP1	0.317368929	3.66E-13	postive
DLD	SHLD2P1	0.310469606	1.24E-12	postive
DLD	SLC12A9-AS1	0.438113583	7.26E-25	postive
DLD	SLC2A1-AS1	0.321760646	1.66E-13	postive
DLD	SLC44A3-AS1	0.312780682	8.26E-13	postive
DLD	SMG7-AS1	0.371049124	9.12E-18	postive
DLD	SNHG1	0.392000495	8.19E-20	postive
DLD	SNHG11	0.372823861	6.20E-18	postive
DLD	SNHG16	0.482826469	1.46E-30	postive
DLD	SNHG17	0.385244108	3.89E-19	postive
DLD	SNHG21	0.337890231	8.09E-15	postive
DLD	SNHG29	0.348681797	9.70E-16	postive
DLD	SNHG32	0.438085038	7.32E-25	postive
DLD	SNHG4	0.394261618	4.82E-20	postive
DLD	SNHG6	0.329890699	3.70E-14	postive
DLD	SNHG7	0.334508156	1.55E-14	postive
DLD	SNHG8	0.360246487	9.10E-17	postive
DLD	SP2-AS1	0.399638205	1.35E-20	postive
DLD	SREBF2-AS1	0.321449094	1.76E-13	postive
DLD	SRP14-AS1	0.330954994	3.03E-14	postive
DLD	ST20-AS1	0.308755861	1.67E-12	postive
DLD	STAG3L1	0.344798332	2.10E-15	postive
DLD	STAG3L2	0.513007941	6.52E-35	postive
DLD	STAG3L3	0.473157223	2.94E-29	postive
DLD	STAG3L4	0.572282307	7.73E-45	postive
DLD	STAG3L5P	0.337042192	9.53E-15	postive
DLD	STAG3L5P-PVRIG2P-PILRB	0.311302451	1.07E-12	postive
DLD	STARD7-AS1	0.495552449	2.41E-32	postive
DLD	STK4-AS1	0.419852174	9.03E-23	postive
DLD	STX18-AS1	0.372135826	7.20E-18	postive
DLD	STXBP5-AS1	0.33393202	1.73E-14	postive
DLD	SUCLG2-AS1	0.441922878	2.56E-25	postive
DLD	SUMO2P17	0.400136081	1.20E-20	postive
DLD	SVIL2P	0.312346224	8.91E-13	postive
DLD	SVIL-AS1	0.512742643	7.15E-35	postive
DLD	SYS1-DBNDD2	0.309392407	1.49E-12	postive
DLD	TAF1A-AS1	0.376854621	2.56E-18	postive

DLD	TAPT1-AS1	0.320507567	2.08E-13	postive
DLD	THAP9-AS1	0.420938852	6.83E-23	postive
DLD	TMEM202-AS1	0.334196212	1.64E-14	postive
DLD	TMEM99	0.397468721	2.26E-20	postive
DLD	TMPO-AS1	0.348525806	1.00E-15	postive
DLD	TOB1-AS1	0.344633177	2.17E-15	postive
DLD	TONSL-AS1	0.307335141	2.13E-12	postive
DLD	TP53TG1	0.389060976	1.62E-19	postive
DLD	TPI1P2	0.376452989	2.79E-18	postive
DLD	TPM3P9	0.372193595	7.11E-18	postive
DLD	TRAF3IP2-AS1	0.307232484	2.17E-12	postive
DLD	TRAM2-AS1	0.427050715	1.40E-23	postive
DLD	TRAPPC12-AS1	0.34149374	4.02E-15	postive
DLD	TRIM52-AS1	0.325018864	9.14E-14	postive
DLD	TSTD3	0.478486451	5.68E-30	postive
DLD	TUBBP1	0.412347622	6.03E-22	postive
DLD	TUG1	0.450764398	2.15E-26	postive
DLD	U91328.1	0.440306455	3.99E-25	postive
DLD	UBA6-AS1	0.508998834	2.61E-34	postive
DLD	UBAC2-AS1	0.339450008	5.99E-15	postive
DLD	UBE2D3-AS1	0.434840062	1.76E-24	postive
DLD	UBL7-AS1	0.339407505	6.04E-15	postive
DLD	UBR5-AS1	0.323879387	1.13E-13	postive
DLD	UPF3AP2	0.380832051	1.05E-18	postive
DLD	USP27X-AS1	0.32529101	8.70E-14	postive
DLD	USP46-AS1	0.420417097	7.81E-23	postive
DLD	UXT-AS1	0.401248276	9.16E-21	postive
DLD	WAC-AS1	0.427867561	1.13E-23	postive
DLD	WARS2-AS1	0.312737335	8.32E-13	postive
DLD	WASH3P	0.384965171	4.14E-19	postive
DLD	WASH6P	0.307632746	2.02E-12	postive
DLD	Z68871.1	0.307964317	1.91E-12	postive
DLD	Z83851.4	0.410857697	8.74E-22	postive
DLD	Z99572.1	0.368346258	1.64E-17	postive
DLD	ZBED5-AS1	0.342489449	3.31E-15	postive
DLD	ZBTB11-AS1	0.379406698	1.45E-18	postive
DLD	ZNF137P	0.339410542	6.03E-15	postive
DLD	ZNF252P	0.526155904	6.01E-37	postive
DLD	ZNF271P	0.337293399	9.08E-15	postive
DLD	ZNF337-AS1	0.304057221	3.73E-12	postive
DLD	ZNF503-AS2	0.373875253	4.93E-18	postive
DLD	ZNF529-AS1	0.3231945	1.28E-13	postive
DLD	ZNF542P	0.319212669	2.63E-13	postive
DLD	ZNF561-AS1	0.380414477	1.16E-18	postive
DLD	ZNF674-AS1	0.305835675	2.75E-12	postive
DLD	ZNF687-AS1	0.386641751	2.82E-19	postive
DLD	ZNF702P	0.320692401	2.01E-13	postive
DLD	ZNF788P	0.306457273	2.48E-12	postive
DLD	ZNF815P	0.301491839	5.76E-12	postive
DLD	ZNRD2-AS1	0.3089333	1.62E-12	postive

DLD	ZNRF2P1	0.330098158	3.56E-14	postive
DLD	ZNRF2P2	0.319221023	2.63E-13	postive
DLD	ZSCAN12P1	0.360739818	8.20E-17	postive
DLD	ZSCAN16-AS1	0.341092605	4.35E-15	postive
F5	AADACP1	0.348149973	1.08E-15	postive
F5	AC004009.1	0.3048203	3.28E-12	postive
F5	AC005280.1	0.321618132	1.70E-13	postive
F5	AC007637.1	0.308943333	1.61E-12	postive
F5	AC008154.1	0.395220151	3.85E-20	postive
F5	AC009041.1	0.476440667	1.07E-29	postive
F5	AC009407.1	0.337547614	8.65E-15	postive
F5	AC010745.1	0.360371138	8.86E-17	postive
F5	AC012485.2	0.354310862	3.10E-16	postive
F5	AC015908.3	0.331107445	2.95E-14	postive
F5	AC016705.2	0.353439397	3.71E-16	postive
F5	AC016735.1	0.490216993	1.37E-31	postive
F5	AC020656.2	0.349387403	8.42E-16	postive
F5	AC020779.2	0.435516132	1.47E-24	postive
F5	AC021218.1	0.300813763	6.45E-12	postive
F5	AC025265.3	0.323293565	1.25E-13	postive
F5	AC026774.1	0.42298437	4.03E-23	postive
F5	AC046168.2	0.327714798	5.55E-14	postive
F5	AC068580.2	0.314555871	6.04E-13	postive
F5	AC091588.3	0.320218257	2.19E-13	postive
F5	AC092375.1	0.360484367	8.65E-17	postive
F5	AC099518.2	0.359988628	9.60E-17	postive
F5	AC104035.1	0.415206573	2.94E-22	postive
F5	AC106876.1	0.436958934	9.94E-25	postive
F5	AC114488.1	0.699816948	8.84E-75	postive
F5	AC138305.1	0.372353704	6.87E-18	postive
F5	AC139720.1	0.457798717	2.86E-27	postive
F5	AC241644.2	0.331723352	2.62E-14	postive
F5	AC243960.3	0.397049239	2.50E-20	postive
F5	AF064860.2	0.365491118	3.01E-17	postive
F5	AF124730.2	0.325048236	9.09E-14	postive
F5	AL021408.1	0.464353822	4.17E-28	postive
F5	AL022318.1	0.303441526	4.14E-12	postive
F5	AL117382.2	0.300461668	6.84E-12	postive
F5	AL118505.1	0.357894151	1.48E-16	postive
F5	AL132655.3	0.314169504	6.46E-13	postive
F5	AL133383.3	0.317782497	3.40E-13	postive
F5	AL136531.2	0.36287213	5.24E-17	postive
F5	AL138930.1	0.449053384	3.50E-26	postive
F5	AL139246.3	0.361703425	6.70E-17	postive
F5	AL158206.1	0.393146348	6.27E-20	postive
F5	AL158837.1	0.529022998	2.11E-37	postive
F5	AL161669.1	0.335448666	1.29E-14	postive
F5	AL353803.1	0.312616087	8.50E-13	postive
F5	AL353803.2	0.417788823	1.53E-22	postive
F5	AL355075.5	0.474077904	2.21E-29	postive

F5	AL583722.1	0.362310749	5.90E-17 postive
F5	AL589745.1	0.353248523	3.85E-16 postive
F5	AL590627.1	0.303181282	4.33E-12 postive
F5	AL606489.1	0.386223525	3.11E-19 postive
F5	AP003555.2	0.373165187	5.76E-18 postive
F5	B3GALT5-AS1	0.357303719	1.68E-16 postive
F5	CYP4F26P	0.430411058	5.76E-24 postive
F5	DIO3OS	0.335736953	1.22E-14 postive
F5	GACAT2	0.319744036	2.39E-13 postive
F5	HNF1A-AS1	0.382158493	7.82E-19 postive
F5	KRT89P	0.323331887	1.25E-13 postive
F5	LINC00365	0.342657316	3.20E-15 postive
F5	LINC00920	0.564831665	1.77E-43 postive
F5	LINC01022	0.499518957	6.48E-33 postive
F5	LINC01258	0.305800692	2.77E-12 postive
F5	LINC01559	0.312208567	9.13E-13 postive
F5	LINC01593	0.309415189	1.49E-12 postive
F5	LINC01713	0.361705495	6.70E-17 postive
F5	LINC01843	0.352702299	4.31E-16 postive
F5	LINC01978	0.426884459	1.46E-23 postive
F5	LINC02086	0.34815663	1.08E-15 postive
F5	LINC02177	0.31977367	2.38E-13 postive
F5	LINC02313	0.357025772	1.78E-16 postive
F5	LINC02535	0.411224331	7.98E-22 postive
F5	LINC02747	0.546818346	2.49E-40 postive
F5	LINP1	0.30639434	2.50E-12 postive
F5	MGAT3-AS1	0.396248574	3.02E-20 postive
F5	MIR194-2HG	0.477376541	8.01E-30 postive
F5	MIR7515HG	0.47023144	7.15E-29 postive
F5	MMADHC-DT	0.361455573	7.06E-17 postive
F5	MUC12-AS1	0.317916726	3.32E-13 postive
F5	MYRF-AS1	0.386393962	2.99E-19 postive
F5	NPSR1-AS1	0.405847986	3.00E-21 postive
F5	PSMD10P2	0.378628122	1.72E-18 postive
F5	PTPRD-AS2	0.570487329	1.65E-44 postive
F5	RP5-968D22.3	0.330289844	3.43E-14 postive
F5	SH3PXD2A-AS1	0.30566721	2.83E-12 postive
F5	SLC25A25-AS1	0.304172082	3.66E-12 postive
F5	SSTR5-AS1	0.588385792	6.73E-48 postive
F5	TAAR3P	0.368853053	1.47E-17 postive
F5	TMEM220-AS1	0.328881608	4.47E-14 postive
F5	TMEM246-AS1	0.465809272	2.70E-28 postive
F5	TMEM51-AS1	0.301532216	5.72E-12 postive
F5	TRPC7-AS1	0.437917686	7.66E-25 postive
F5	WTAPP1	0.338198618	7.63E-15 postive
F5	XXYLT1-AS2	0.472445703	3.65E-29 postive
F8	A2M-AS1	0.44033064	3.96E-25 postive
F8	AC000403.1	0.304888594	3.24E-12 postive
F8	AC002091.1	0.312296761	8.99E-13 postive
F8	AC002091.2	0.355259521	2.55E-16 postive

F8	AC002368.1	0.311776652	9.85E-13	postive
F8	AC002401.2	0.385550261	3.62E-19	postive
F8	AC002470.1	0.300821236	6.44E-12	postive
F8	AC003070.2	0.363926927	4.20E-17	postive
F8	AC004112.1	0.365660359	2.90E-17	postive
F8	AC004241.1	0.342651769	3.21E-15	postive
F8	AC004540.1	0.351399947	5.61E-16	postive
F8	AC004771.2	0.356455417	2.00E-16	postive
F8	AC004825.2	0.325175471	8.88E-14	postive
F8	AC004854.2	0.307731681	1.99E-12	postive
F8	AC004943.2	0.347415081	1.25E-15	postive
F8	AC004947.1	0.337091086	9.44E-15	postive
F8	AC004973.1	0.410333487	9.95E-22	postive
F8	AC005076.2	0.31814687	3.18E-13	postive
F8	AC005180.1	0.349735756	7.85E-16	postive
F8	AC005180.2	0.401915863	7.80E-21	postive
F8	AC005332.4	0.353541236	3.63E-16	postive
F8	AC005520.4	0.309214695	1.54E-12	postive
F8	AC005670.3	0.426719785	1.52E-23	postive
F8	AC005695.1	0.381405113	9.27E-19	postive
F8	AC005759.1	0.302137278	5.16E-12	postive
F8	AC005790.1	0.315253811	5.34E-13	postive
F8	AC005884.1	0.413017248	5.10E-22	postive
F8	AC006033.2	0.406691698	2.44E-21	postive
F8	AC006126.4	0.312507608	8.66E-13	postive
F8	AC006213.1	0.308797488	1.65E-12	postive
F8	AC006333.1	0.381362415	9.36E-19	postive
F8	AC006547.1	0.312417791	8.80E-13	postive
F8	AC007036.1	0.42990523	6.58E-24	postive
F8	AC007114.1	0.366335874	2.51E-17	postive
F8	AC007383.2	0.308747599	1.67E-12	postive
F8	AC007406.3	0.355978223	2.20E-16	postive
F8	AC007613.1	0.348246532	1.06E-15	postive
F8	AC007638.2	0.364665441	3.59E-17	postive
F8	AC007671.1	0.36976974	1.20E-17	postive
F8	AC007728.2	0.305366553	2.98E-12	postive
F8	AC008063.1	0.369162381	1.37E-17	postive
F8	AC008124.1	0.339180812	6.31E-15	postive
F8	AC008267.5	0.305046031	3.15E-12	postive
F8	AC008393.1	0.479469818	4.18E-30	postive
F8	AC008438.1	0.326846444	6.52E-14	postive
F8	AC008443.1	0.3887488	1.74E-19	postive
F8	AC008443.5	0.501045491	3.89E-33	postive
F8	AC008443.6	0.308582594	1.72E-12	postive
F8	AC008494.3	0.524437416	1.12E-36	postive
F8	AC008592.1	0.309188085	1.55E-12	postive
F8	AC008619.1	0.407656678	1.93E-21	postive
F8	AC008629.1	0.383443474	5.85E-19	postive
F8	AC008669.1	0.384102574	5.04E-19	postive
F8	AC008747.1	0.328654786	4.66E-14	postive

F8	AC008758.4	0.341418857	4.08E-15	postive
F8	AC008764.2	0.37496171	3.88E-18	postive
F8	AC008771.1	0.446453191	7.26E-26	postive
F8	AC008946.1	0.308532682	1.73E-12	postive
F8	AC008966.1	0.400559454	1.08E-20	postive
F8	AC008991.1	0.397139358	2.45E-20	postive
F8	AC009041.3	0.394914943	4.14E-20	postive
F8	AC009053.3	0.496984899	1.50E-32	postive
F8	AC009055.2	0.324677378	9.73E-14	postive
F8	AC009061.2	0.456656151	3.98E-27	postive
F8	AC009090.2	0.311350365	1.06E-12	postive
F8	AC009113.1	0.400721868	1.04E-20	postive
F8	AC009126.1	0.480342694	3.18E-30	postive
F8	AC009159.3	0.356134765	2.13E-16	postive
F8	AC009163.7	0.422619127	4.43E-23	postive
F8	AC009171.2	0.387703102	2.21E-19	postive
F8	AC009237.14	0.376345142	2.86E-18	postive
F8	AC009506.2	0.468453459	1.22E-28	postive
F8	AC009549.1	0.304963742	3.20E-12	postive
F8	AC009690.2	0.32651283	6.94E-14	postive
F8	AC009779.3	0.360322828	8.95E-17	postive
F8	AC009831.1	0.385431078	3.72E-19	postive
F8	AC009962.1	0.355186076	2.59E-16	postive
F8	AC010205.1	0.38953616	1.45E-19	postive
F8	AC010226.1	0.545260488	4.56E-40	postive
F8	AC010240.4	0.32301941	1.32E-13	postive
F8	AC010273.2	0.323077836	1.30E-13	postive
F8	AC010319.5	0.363193901	4.90E-17	postive
F8	AC010320.2	0.331411171	2.78E-14	postive
F8	AC010422.1	0.306290892	2.55E-12	postive
F8	AC010504.1	0.307769623	1.98E-12	postive
F8	AC010969.2	0.313923787	6.75E-13	postive
F8	AC011346.1	0.307752549	1.98E-12	postive
F8	AC011374.2	0.309393255	1.49E-12	postive
F8	AC011472.4	0.308415174	1.77E-12	postive
F8	AC011474.1	0.359779337	1.00E-16	postive
F8	AC011474.2	0.384558409	4.54E-19	postive
F8	AC011477.3	0.378362367	1.83E-18	postive
F8	AC011524.1	0.447597862	5.27E-26	postive
F8	AC011899.2	0.399509178	1.39E-20	postive
F8	AC012184.3	0.409677442	1.17E-21	postive
F8	AC012306.2	0.303088751	4.40E-12	postive
F8	AC012313.5	0.389831633	1.36E-19	postive
F8	AC012555.1	0.317055595	3.87E-13	postive
F8	AC012603.1	0.309551887	1.45E-12	postive
F8	AC013553.3	0.305542397	2.90E-12	postive
F8	AC013724.1	0.355796145	2.29E-16	postive
F8	AC015819.1	0.425199101	2.27E-23	postive
F8	AC015908.3	0.311275345	1.07E-12	postive
F8	AC015922.2	0.364696649	3.56E-17	postive

F8	AC015922.3	0.310377247	1.26E-12	postive
F8	AC016727.1	0.308083001	1.87E-12	postive
F8	AC016747.1	0.377072397	2.44E-18	postive
F8	AC016888.1	0.348499297	1.01E-15	postive
F8	AC018521.5	0.320378381	2.13E-13	postive
F8	AC018521.6	0.353797505	3.44E-16	postive
F8	AC018647.1	0.464116493	4.47E-28	postive
F8	AC018755.4	0.359420459	1.08E-16	postive
F8	AC018809.2	0.367002681	2.18E-17	postive
F8	AC018845.3	0.32133986	1.79E-13	postive
F8	AC019131.2	0.368575972	1.56E-17	postive
F8	AC019193.2	0.352544043	4.45E-16	postive
F8	AC020978.4	0.303260034	4.27E-12	postive
F8	AC021016.2	0.433221844	2.72E-24	postive
F8	AC021483.2	0.382639722	7.02E-19	postive
F8	AC021752.1	0.324905858	9.33E-14	postive
F8	AC022098.1	0.383602607	5.64E-19	postive
F8	AC022098.3	0.343937373	2.49E-15	postive
F8	AC022217.4	0.320711095	2.01E-13	postive
F8	AC022509.3	0.362986141	5.12E-17	postive
F8	AC022613.2	0.475736338	1.33E-29	postive
F8	AC022915.1	0.359893233	9.79E-17	postive
F8	AC022916.2	0.380244434	1.20E-18	postive
F8	AC023813.3	0.372069661	7.31E-18	postive
F8	AC023906.5	0.329313218	4.12E-14	postive
F8	AC024075.1	0.372729113	6.33E-18	postive
F8	AC024075.3	0.340865248	4.55E-15	postive
F8	AC024132.4	0.304779185	3.30E-12	postive
F8	AC024337.2	0.365089171	3.28E-17	postive
F8	AC024575.2	0.318869343	2.80E-13	postive
F8	AC025048.2	0.302137076	5.16E-12	postive
F8	AC025171.1	0.371942288	7.51E-18	postive
F8	AC025171.2	0.330259625	3.45E-14	postive
F8	AC025181.2	0.301236522	6.01E-12	postive
F8	AC025279.1	0.321023335	1.90E-13	postive
F8	AC025470.2	0.367270753	2.06E-17	postive
F8	AC025822.2	0.396474477	2.86E-20	postive
F8	AC026304.1	0.360500137	8.63E-17	postive
F8	AC026369.2	0.32322559	1.27E-13	postive
F8	AC026471.1	0.384568241	4.53E-19	postive
F8	AC026691.1	0.533909924	3.44E-38	postive
F8	AC026780.2	0.313261583	7.59E-13	postive
F8	AC026801.2	0.325539002	8.31E-14	postive
F8	AC026904.2	0.417115989	1.81E-22	postive
F8	AC027559.1	0.398790337	1.65E-20	postive
F8	AC027682.6	0.401224843	9.21E-21	postive
F8	AC035140.1	0.403069348	5.90E-21	postive
F8	AC036176.1	0.426149608	1.77E-23	postive
F8	AC040168.1	0.372448066	6.73E-18	postive
F8	AC048382.5	0.319312103	2.58E-13	postive

F8	AC051619.6	0.308788775	1.66E-12	postive
F8	AC053503.4	0.321775272	1.65E-13	postive
F8	AC060766.6	0.372234403	7.05E-18	postive
F8	AC060766.7	0.415182158	2.96E-22	postive
F8	AC064805.1	0.361977041	6.33E-17	postive
F8	AC067852.2	0.366066811	2.66E-17	postive
F8	AC068338.2	0.394157054	4.94E-20	postive
F8	AC068580.3	0.337989549	7.94E-15	postive
F8	AC068870.2	0.460361112	1.35E-27	postive
F8	AC069209.1	0.372743306	6.31E-18	postive
F8	AC069213.1	0.363721529	4.38E-17	postive
F8	AC069224.2	0.444089227	1.40E-25	postive
F8	AC072061.1	0.364153763	4.00E-17	postive
F8	AC073254.1	0.315350758	5.24E-13	postive
F8	AC073332.1	0.323426024	1.22E-13	postive
F8	AC073508.3	0.300975201	6.28E-12	postive
F8	AC078785.1	0.317537332	3.55E-13	postive
F8	AC078880.3	0.382044226	8.02E-19	postive
F8	AC078880.4	0.379750511	1.34E-18	postive
F8	AC078880.5	0.365272768	3.15E-17	postive
F8	AC079209.1	0.387914829	2.11E-19	postive
F8	AC079313.2	0.350044733	7.37E-16	postive
F8	AC079807.1	0.331283952	2.85E-14	postive
F8	AC079848.1	0.489407628	1.78E-31	postive
F8	AC079922.2	0.374471743	4.32E-18	postive
F8	AC080013.5	0.368677325	1.52E-17	postive
F8	AC082651.1	0.360348445	8.90E-17	postive
F8	AC083843.1	0.31223127	9.09E-13	postive
F8	AC083843.2	0.301230312	6.02E-12	postive
F8	AC083973.1	0.31873456	2.87E-13	postive
F8	AC084757.3	0.377636528	2.15E-18	postive
F8	AC087071.1	0.351250011	5.78E-16	postive
F8	AC087379.2	0.345334927	1.89E-15	postive
F8	AC087500.1	0.340292847	5.08E-15	postive
F8	AC087521.3	0.37474761	4.07E-18	postive
F8	AC087645.2	0.320039944	2.27E-13	postive
F8	AC087854.1	0.341612328	3.93E-15	postive
F8	AC090115.1	0.322575343	1.43E-13	postive
F8	AC090136.3	0.321277425	1.81E-13	postive
F8	AC090155.2	0.320615591	2.04E-13	postive
F8	AC090193.1	0.349251825	8.65E-16	postive
F8	AC090505.2	0.31575929	4.88E-13	postive
F8	AC090515.3	0.318239621	3.13E-13	postive
F8	AC090559.1	0.461073896	1.10E-27	postive
F8	AC090587.1	0.438237171	7.02E-25	postive
F8	AC090617.5	0.326172662	7.39E-14	postive
F8	AC090825.1	0.457045814	3.55E-27	postive
F8	AC090912.1	0.341080062	4.36E-15	postive
F8	AC091057.3	0.307757033	1.98E-12	postive
F8	AC091114.1	0.348311255	1.04E-15	postive

F8	AC091982.1	0.335175493	1.36E-14	postive
F8	AC091982.3	0.396230964	3.03E-20	postive
F8	AC092070.2	0.31810943	3.21E-13	postive
F8	AC092134.1	0.352793174	4.23E-16	postive
F8	AC092354.2	0.32504199	9.10E-14	postive
F8	AC092368.3	0.363701301	4.40E-17	postive
F8	AC092431.1	0.438188482	7.11E-25	postive
F8	AC092803.1	0.329556499	3.94E-14	postive
F8	AC092944.1	0.347754976	1.17E-15	postive
F8	AC093010.3	0.320224055	2.19E-13	postive
F8	AC093110.1	0.304874207	3.25E-12	postive
F8	AC093278.2	0.543022006	1.08E-39	postive
F8	AC093726.1	0.447545013	5.35E-26	postive
F8	AC093752.1	0.324563306	9.94E-14	postive
F8	AC095050.1	0.326618333	6.81E-14	postive
F8	AC096536.2	0.305356217	2.99E-12	postive
F8	AC096543.2	0.333334791	1.93E-14	postive
F8	AC096677.1	0.335616633	1.25E-14	postive
F8	AC096733.3	0.482258589	1.74E-30	postive
F8	AC096772.6	0.374965294	3.88E-18	postive
F8	AC096921.2	0.312364431	8.88E-13	postive
F8	AC097359.2	0.419693846	9.40E-23	postive
F8	AC097376.3	0.372714418	6.35E-18	postive
F8	AC098484.1	0.391519033	9.16E-20	postive
F8	AC099343.4	0.524971385	9.25E-37	postive
F8	AC099518.4	0.363538391	4.55E-17	postive
F8	AC099522.2	0.361257296	7.36E-17	postive
F8	AC099811.3	0.312758213	8.29E-13	postive
F8	AC100786.1	0.405406796	3.34E-21	postive
F8	AC100803.2	0.398542461	1.75E-20	postive
F8	AC100803.3	0.307158443	2.19E-12	postive
F8	AC100849.1	0.306520852	2.45E-12	postive
F8	AC103770.1	0.381081972	9.96E-19	postive
F8	AC103855.2	0.319568943	2.47E-13	postive
F8	AC103925.1	0.369553982	1.26E-17	postive
F8	AC103974.1	0.334486931	1.55E-14	postive
F8	AC104076.1	0.392625132	7.08E-20	postive
F8	AC104083.1	0.419383294	1.02E-22	postive
F8	AC104109.2	0.483414131	1.21E-30	postive
F8	AC104123.1	0.308299945	1.80E-12	postive
F8	AC104211.2	0.427774564	1.15E-23	postive
F8	AC104232.1	0.4078468	1.84E-21	postive
F8	AC104237.3	0.336258959	1.11E-14	postive
F8	AC104435.2	0.347529464	1.22E-15	postive
F8	AC104590.1	0.337781432	8.26E-15	postive
F8	AC104596.1	0.378441027	1.80E-18	postive
F8	AC104777.1	0.363848285	4.27E-17	postive
F8	AC104794.3	0.409356401	1.27E-21	postive
F8	AC104819.3	0.330620898	3.23E-14	postive
F8	AC104825.1	0.324877591	9.38E-14	postive

F8	AC104938.1	0.329694811	3.84E-14	postive
F8	AC105020.6	0.455467799	5.61E-27	postive
F8	AC105942.1	0.374537986	4.26E-18	postive
F8	AC106706.1	0.39050749	1.16E-19	postive
F8	AC106739.1	0.33572874	1.23E-14	postive
F8	AC106786.2	0.471162211	5.39E-29	postive
F8	AC106820.5	0.360130205	9.32E-17	postive
F8	AC106881.1	0.40530141	3.43E-21	postive
F8	AC107027.3	0.302219815	5.09E-12	postive
F8	AC107884.1	0.353288388	3.82E-16	postive
F8	AC107959.1	0.445448446	9.62E-26	postive
F8	AC108134.2	0.349538909	8.16E-16	postive
F8	AC109347.1	0.487879449	2.92E-31	postive
F8	AC110995.1	0.376727064	2.63E-18	postive
F8	AC112206.2	0.342164324	3.53E-15	postive
F8	AC112220.2	0.332768957	2.15E-14	postive
F8	AC113382.1	0.372954319	6.03E-18	postive
F8	AC114271.1	0.351203859	5.83E-16	postive
F8	AC114488.2	0.322789481	1.38E-13	postive
F8	AC114956.2	0.330795475	3.12E-14	postive
F8	AC115618.2	0.321175114	1.84E-13	postive
F8	AC116312.1	0.379077265	1.56E-18	postive
F8	AC116351.1	0.384328049	4.79E-19	postive
F8	AC116407.1	0.369570227	1.26E-17	postive
F8	AC116913.1	0.309760795	1.40E-12	postive
F8	AC118553.1	0.406483484	2.57E-21	postive
F8	AC120049.1	0.310529694	1.22E-12	postive
F8	AC121338.2	0.312682776	8.40E-13	postive
F8	AC122129.1	0.365957922	2.73E-17	postive
F8	AC124861.1	0.334919058	1.43E-14	postive
F8	AC126177.3	0.343759563	2.58E-15	postive
F8	AC127521.1	0.374461945	4.33E-18	postive
F8	AC128709.3	0.469905206	7.89E-29	postive
F8	AC129492.4	0.33526367	1.34E-14	postive
F8	AC131097.3	0.433330321	2.64E-24	postive
F8	AC132008.2	0.310647157	1.20E-12	postive
F8	AC133552.2	0.334490884	1.55E-14	postive
F8	AC135012.3	0.467394643	1.68E-28	postive
F8	AC135050.6	0.315842823	4.81E-13	postive
F8	AC136604.3	0.300402907	6.91E-12	postive
F8	AC137056.1	0.417622998	1.60E-22	postive
F8	AC138123.1	0.454276849	7.90E-27	postive
F8	AC138207.8	0.406127465	2.80E-21	postive
F8	AC138356.1	0.382048482	8.02E-19	postive
F8	AC139768.1	0.300410005	6.90E-12	postive
F8	AC139795.1	0.352838587	4.19E-16	postive
F8	AC139795.2	0.396332991	2.96E-20	postive
F8	AC145098.1	0.354207586	3.17E-16	postive
F8	AC145124.1	0.365350214	3.10E-17	postive
F8	AC174065.1	0.316570447	4.22E-13	postive

F8	AC242426.2	0.328032313	5.24E-14	postive
F8	AC243571.1	0.37140382	8.45E-18	postive
F8	AC244090.1	0.499458178	6.61E-33	postive
F8	AC245140.2	0.321916029	1.61E-13	postive
F8	ADAMTS7P3	0.321739279	1.67E-13	postive
F8	ADAMTS9-AS2	0.333676936	1.81E-14	postive
F8	ADNP-AS1	0.345156443	1.96E-15	postive
F8	ADPGK-AS1	0.352759359	4.26E-16	postive
F8	AF111167.2	0.43866916	6.24E-25	postive
F8	AF131215.5	0.361582928	6.88E-17	postive
F8	AF131216.4	0.363913075	4.21E-17	postive
F8	AF274858.1	0.376199699	2.96E-18	postive
F8	AF279873.4	0.360693242	8.28E-17	postive
F8	AJ009632.2	0.365243695	3.17E-17	postive
F8	AL009178.2	0.402907347	6.14E-21	postive
F8	AL020996.1	0.446383262	7.41E-26	postive
F8	AL022476.1	0.314617895	5.97E-13	postive
F8	AL023806.1	0.40012765	1.20E-20	postive
F8	AL023881.1	0.338872597	6.69E-15	postive
F8	AL024498.1	0.307741674	1.98E-12	postive
F8	AL031055.1	0.371621853	8.06E-18	postive
F8	AL031429.1	0.383103065	6.32E-19	postive
F8	AL031432.3	0.3401051	5.27E-15	postive
F8	AL035530.2	0.306725072	2.36E-12	postive
F8	AL035587.1	0.360513336	8.60E-17	postive
F8	AL035661.1	0.387299183	2.43E-19	postive
F8	AL049597.2	0.368651858	1.53E-17	postive
F8	AL049796.1	0.340315614	5.06E-15	postive
F8	AL050309.1	0.390986774	1.04E-19	postive
F8	AL078581.2	0.492032435	7.63E-32	postive
F8	AL078590.2	0.516282025	2.07E-35	postive
F8	AL109741.1	0.487193174	3.64E-31	postive
F8	AL109804.1	0.312517067	8.65E-13	postive
F8	AL117327.1	0.307678337	2.01E-12	postive
F8	AL117332.1	0.310096838	1.32E-12	postive
F8	AL118506.1	0.390556921	1.15E-19	postive
F8	AL118523.1	0.362876295	5.24E-17	postive
F8	AL118558.3	0.324279017	1.05E-13	postive
F8	AL121574.1	0.340199575	5.17E-15	postive
F8	AL121601.1	0.366983964	2.19E-17	postive
F8	AL121894.2	0.311118759	1.10E-12	postive
F8	AL121944.2	0.309813821	1.39E-12	postive
F8	AL122035.1	0.446465911	7.24E-26	postive
F8	AL122035.2	0.303321879	4.23E-12	postive
F8	AL133342.1	0.345904242	1.69E-15	postive
F8	AL133355.1	0.388113939	2.01E-19	postive
F8	AL133371.2	0.372069288	7.31E-18	postive
F8	AL135744.1	0.325977522	7.66E-14	postive
F8	AL135791.1	0.343810176	2.55E-15	postive
F8	AL135960.1	0.329652499	3.87E-14	postive

F8	AL136366.1	0.329946078	3.66E-14	postive
F8	AL136368.1	0.325260388	8.74E-14	postive
F8	AL137003.1	0.304737678	3.32E-12	postive
F8	AL137026.1	0.303493023	4.10E-12	postive
F8	AL137186.2	0.355221735	2.57E-16	postive
F8	AL137779.2	0.352947902	4.10E-16	postive
F8	AL138995.1	0.317715818	3.44E-13	postive
F8	AL139011.1	0.339516429	5.91E-15	postive
F8	AL139035.1	0.331399968	2.79E-14	postive
F8	AL139260.1	0.409609096	1.19E-21	postive
F8	AL139339.1	0.396280508	3.00E-20	postive
F8	AL139384.1	0.378956837	1.60E-18	postive
F8	AL157394.1	0.383645521	5.59E-19	postive
F8	AL157895.2	0.359413406	1.08E-16	postive
F8	AL158071.1	0.3427896	3.12E-15	postive
F8	AL160313.2	0.38511883	4.00E-19	postive
F8	AL161640.2	0.363068488	5.03E-17	postive
F8	AL161785.1	0.338480429	7.22E-15	postive
F8	AL161937.1	0.366240388	2.57E-17	postive
F8	AL162231.2	0.486175126	5.03E-31	postive
F8	AL162377.1	0.399205904	1.50E-20	postive
F8	AL353593.1	0.32632025	7.19E-14	postive
F8	AL353708.1	0.318688015	2.89E-13	postive
F8	AL353743.1	0.363188778	4.90E-17	postive
F8	AL353743.2	0.315714378	4.92E-13	postive
F8	AL354740.1	0.427042304	1.40E-23	postive
F8	AL354824.1	0.434029933	2.19E-24	postive
F8	AL354824.2	0.30202377	5.26E-12	postive
F8	AL354861.3	0.334796308	1.46E-14	postive
F8	AL354920.1	0.450827577	2.12E-26	postive
F8	AL355001.2	0.305622396	2.86E-12	postive
F8	AL355297.3	0.307409267	2.10E-12	postive
F8	AL355300.1	0.386776246	2.74E-19	postive
F8	AL355312.2	0.328100843	5.17E-14	postive
F8	AL355607.2	0.306099114	2.63E-12	postive
F8	AL355803.1	0.380255208	1.20E-18	postive
F8	AL356056.2	0.439412598	5.09E-25	postive
F8	AL356235.1	0.390615156	1.13E-19	postive
F8	AL356481.1	0.383199533	6.18E-19	postive
F8	AL356512.1	0.373315794	5.57E-18	postive
F8	AL356599.1	0.568798396	3.37E-44	postive
F8	AL357033.2	0.382431967	7.35E-19	postive
F8	AL357033.3	0.410586179	9.35E-22	postive
F8	AL357033.4	0.364315149	3.86E-17	postive
F8	AL357054.4	0.404284771	4.40E-21	postive
F8	AL357146.1	0.514435445	3.96E-35	postive
F8	AL357992.1	0.360415229	8.78E-17	postive
F8	AL358472.2	0.36197939	6.33E-17	postive
F8	AL358852.1	0.452899139	1.17E-26	postive
F8	AL359076.1	0.311991264	9.48E-13	postive

F8	AL359091.3	0.305775474	2.78E-12	postive
F8	AL359182.1	0.30483834	3.27E-12	postive
F8	AL359532.1	0.300687594	6.59E-12	postive
F8	AL359541.2	0.322571244	1.43E-13	postive
F8	AL359962.1	0.41718712	1.78E-22	postive
F8	AL359962.2	0.316013828	4.66E-13	postive
F8	AL359979.2	0.304799977	3.29E-12	postive
F8	AL365181.1	0.328762189	4.57E-14	postive
F8	AL390755.1	0.407460361	2.02E-21	postive
F8	AL390760.1	0.311284484	1.07E-12	postive
F8	AL390879.1	0.326631898	6.79E-14	postive
F8	AL390955.2	0.423092528	3.92E-23	postive
F8	AL391069.2	0.30092548	6.33E-12	postive
F8	AL391832.3	0.430406807	5.76E-24	postive
F8	AL391834.2	0.32654171	6.90E-14	postive
F8	AL441992.2	0.322198306	1.53E-13	postive
F8	AL442663.3	0.446523251	7.12E-26	postive
F8	AL445228.3	0.333564305	1.85E-14	postive
F8	AL445423.1	0.417584377	1.61E-22	postive
F8	AL445426.1	0.34305664	2.96E-15	postive
F8	AL445645.1	0.308016486	1.89E-12	postive
F8	AL450326.1	0.492956623	5.64E-32	postive
F8	AL450998.2	0.341378894	4.11E-15	postive
F8	AL451042.2	0.346980519	1.36E-15	postive
F8	AL451165.2	0.342413256	3.36E-15	postive
F8	AL512625.1	0.305988389	2.68E-12	postive
F8	AL513550.1	0.342634091	3.22E-15	postive
F8	AL583722.1	0.313802663	6.90E-13	postive
F8	AL590226.1	0.437518675	8.53E-25	postive
F8	AL590428.1	0.414376926	3.62E-22	postive
F8	AL603839.2	0.419480353	9.93E-23	postive
F8	AL604028.1	0.322498277	1.45E-13	postive
F8	AL606469.1	0.401637775	8.34E-21	postive
F8	AL606491.1	0.352700096	4.31E-16	postive
F8	AL606760.1	0.347788693	1.16E-15	postive
F8	AL645728.1	0.432852278	3.00E-24	postive
F8	AL645933.3	0.361659595	6.77E-17	postive
F8	AL662844.4	0.325106148	9.00E-14	postive
F8	AL691403.2	0.320540979	2.07E-13	postive
F8	AL691432.2	0.335904836	1.19E-14	postive
F8	AL731577.2	0.343071227	2.95E-15	postive
F8	AL732314.8	0.371803646	7.74E-18	postive
F8	ALG1L13P	0.329601133	3.91E-14	postive
F8	ALOX12-AS1	0.373727154	5.09E-18	postive
F8	AMZ2P1	0.446260047	7.67E-26	postive
F8	ANO7L1	0.396946887	2.56E-20	postive
F8	AOC4P	0.336972522	9.66E-15	postive
F8	AP000229.1	0.339678461	5.73E-15	postive
F8	AP000253.1	0.322597319	1.42E-13	postive
F8	AP000254.2	0.311767133	9.86E-13	postive

F8	AP000295.10	0.335668083	1.24E-14	postive
F8	AP000317.1	0.323768614	1.15E-13	postive
F8	AP000317.2	0.336445214	1.07E-14	postive
F8	AP000438.1	0.377269526	2.33E-18	postive
F8	AP000439.1	0.324837942	9.45E-14	postive
F8	AP000442.1	0.336657801	1.03E-14	postive
F8	AP000640.1	0.323631383	1.18E-13	postive
F8	AP000781.1	0.313780076	6.92E-13	postive
F8	AP000866.1	0.381109453	9.90E-19	postive
F8	AP000911.1	0.384222385	4.90E-19	postive
F8	AP001160.3	0.310174325	1.30E-12	postive
F8	AP001189.1	0.412926757	5.22E-22	postive
F8	AP001189.3	0.435201018	1.60E-24	postive
F8	AP001189.5	0.459123562	1.94E-27	postive
F8	AP001363.2	0.32598046	7.66E-14	postive
F8	AP001372.2	0.366271812	2.55E-17	postive
F8	AP001437.1	0.32123359	1.83E-13	postive
F8	AP001816.1	0.371571479	8.15E-18	postive
F8	AP001922.5	0.403657654	5.12E-21	postive
F8	AP001922.6	0.332131379	2.43E-14	postive
F8	AP002008.3	0.360558358	8.52E-17	postive
F8	AP002761.3	0.339920948	5.46E-15	postive
F8	AP002840.2	0.409419838	1.25E-21	postive
F8	AP002954.1	0.360143517	9.29E-17	postive
F8	AP003469.2	0.302738731	4.66E-12	postive
F8	AP003486.1	0.399583946	1.37E-20	postive
F8	AP003721.4	0.463436079	5.47E-28	postive
F8	AP004609.5	0.329803429	3.76E-14	postive
F8	AP006621.3	0.371102643	9.02E-18	postive
F8	ARMCX5-GPRASP2	0.434453588	1.95E-24	postive
F8	ARRDC1-AS1	0.358248769	1.38E-16	postive
F8	ASB16-AS1	0.376693357	2.65E-18	postive
F8	ATP1A1-AS1	0.301929045	5.35E-12	postive
F8	ATXN1-AS1	0.32297054	1.33E-13	postive
F8	BAALC-AS1	0.374323405	4.47E-18	postive
F8	BAIAP2-DT	0.311298297	1.07E-12	postive
F8	BDNF-AS	0.486932921	3.95E-31	postive
F8	BRWD1-AS2	0.323254447	1.26E-13	postive
F8	BTG3-AS1	0.347567271	1.21E-15	postive
F8	BTN2A3P	0.352535833	4.45E-16	postive
F8	BX470209.1	0.397047812	2.50E-20	postive
F8	BX470209.2	0.42451478	2.71E-23	postive
F8	BX537318.1	0.304702682	3.34E-12	postive
F8	BX571846.1	0.384884827	4.22E-19	postive
F8	BX640514.2	0.431596959	4.20E-24	postive
F8	C15orf56	0.316149763	4.55E-13	postive
F8	C21orf62-AS1	0.468260849	1.30E-28	postive
F8	C22orf46	0.366568769	2.39E-17	postive
F8	C5orf64	0.330260682	3.45E-14	postive
F8	C6orf99	0.331761149	2.60E-14	postive

F8	CA5BP1	0.318654032	2.91E-13	postive
F8	CADM3-AS1	0.300285695	7.05E-12	postive
F8	CAMTA1-DT	0.349268061	8.62E-16	postive
F8	CARD8-AS1	0.398129951	1.93E-20	postive
F8	CASC2	0.407442465	2.03E-21	postive
F8	CASP16P	0.316273094	4.45E-13	postive
F8	CASP17P	0.323818933	1.14E-13	postive
F8	CCDC84-DT	0.357971136	1.46E-16	postive
F8	CCR5AS	0.314613024	5.98E-13	postive
F8	CD81-AS1	0.378829297	1.65E-18	postive
F8	CDC37L1-DT	0.384026939	5.13E-19	postive
F8	CEACAMP10	0.323563578	1.19E-13	postive
F8	CEBPB-AS1	0.30932659	1.51E-12	postive
F8	CENPBD1P1	0.349309039	8.55E-16	postive
F8	CERNA1	0.354687909	2.87E-16	postive
F8	CFL1P1	0.313927216	6.75E-13	postive
F8	CHROMR	0.378763884	1.67E-18	postive
F8	CKMT2-AS1	0.409827646	1.13E-21	postive
F8	CNTFR-AS1	0.303518469	4.09E-12	postive
F8	COX10-AS1	0.327774757	5.49E-14	postive
F8	CTBP1-DT	0.383358994	5.96E-19	postive
F8	CTC-487M23.5	0.352628794	4.37E-16	postive
F8	CTD-2245F17.3	0.350790493	6.34E-16	postive
F8	CTD-2521M24.9	0.353777131	3.46E-16	postive
F8	CTD-2540M10.1	0.316034739	4.64E-13	postive
F8	CYP4F23P	0.329866754	3.72E-14	postive
F8	DACT3-AS1	0.314433787	6.17E-13	postive
F8	DBIL5P	0.390908076	1.06E-19	postive
F8	DEC1	0.313559993	7.20E-13	postive
F8	DNAJC3-DT	0.434735074	1.81E-24	postive
F8	DNM1P46	0.377710351	2.12E-18	postive
F8	DNM1P47	0.386034042	3.25E-19	postive
F8	DNMBP-AS1	0.353166111	3.92E-16	postive
F8	DOCK9-DT	0.449602514	2.99E-26	postive
F8	DPH6-DT	0.417073572	1.83E-22	postive
F8	DUBR	0.317065525	3.86E-13	postive
F8	EBLN3P	0.317621916	3.50E-13	postive
F8	ECI2-DT	0.407756684	1.88E-21	postive
F8	EIF1B-AS1	0.493615799	4.55E-32	postive
F8	EIF2AK3-DT	0.333788249	1.77E-14	postive
F8	EIF3J-DT	0.464402511	4.11E-28	postive
F8	ELOA-AS1	0.317888043	3.34E-13	postive
F8	EMC1-AS1	0.423531045	3.50E-23	postive
F8	ENTPD3-AS1	0.35364911	3.55E-16	postive
F8	EP300-AS1	0.388263688	1.95E-19	postive
F8	EPB41L4A-DT	0.528551335	2.50E-37	postive
F8	EPHA1-AS1	0.340313906	5.06E-15	postive
F8	ERVE-1	0.381126871	9.86E-19	postive
F8	ETV7-AS1	0.324901487	9.34E-14	postive
F8	EXOSC10-AS1	0.346182594	1.60E-15	postive

F8	F10-AS1	0.314813849	5.77E-13	postive
F8	FAM111A-DT	0.480844357	2.72E-30	postive
F8	FAM160A1-DT	0.364333271	3.85E-17	postive
F8	FAM21EP	0.313348891	7.47E-13	postive
F8	FAM21FP	0.427975597	1.10E-23	postive
F8	FAM66A	0.31017581	1.30E-12	postive
F8	FAM66B	0.437330287	8.98E-25	postive
F8	FAM66C	0.350713586	6.44E-16	postive
F8	FAM66D	0.423526096	3.50E-23	postive
F8	FAM86DP	0.307228931	2.17E-12	postive
F8	FAM86JP	0.313946349	6.72E-13	postive
F8	FBP2P1	0.388607324	1.80E-19	postive
F8	FENDRR	0.400764868	1.03E-20	postive
F8	FGD5-AS1	0.440113254	4.20E-25	postive
F8	FGF13-AS1	0.309212003	1.54E-12	postive
F8	FGF14-AS2	0.396198233	3.06E-20	postive
F8	FGF7P3	0.359336963	1.10E-16	postive
F8	FHAD1-AS1	0.306612867	2.41E-12	postive
F8	FP671120.5	0.329560699	3.94E-14	postive
F8	FRG1HP	0.460065206	1.47E-27	postive
F8	GAS5-AS1	0.352379725	4.60E-16	postive
F8	GAS6-DT	0.341238699	4.23E-15	postive
F8	GGTA1P	0.465141652	3.30E-28	postive
F8	GIHCG	0.347155579	1.32E-15	postive
F8	GLIDR	0.397038709	2.50E-20	postive
F8	GNRHR2	0.336653072	1.03E-14	postive
F8	GOLGA2P7	0.312786805	8.25E-13	postive
F8	GOLGA8EP	0.343453959	2.74E-15	postive
F8	GS1-358P8.4	0.43717039	9.38E-25	postive
F8	GTF2IP4	0.339227156	6.25E-15	postive
F8	GUSBP1	0.31073222	1.18E-12	postive
F8	GUSBP5	0.313103301	7.80E-13	postive
F8	GVINP1	0.351641582	5.34E-16	postive
F8	H2AZ2P1	0.321950354	1.60E-13	postive
F8	HCG11	0.326247294	7.29E-14	postive
F8	HCG25	0.324479912	1.01E-13	postive
F8	HCP5	0.374719833	4.09E-18	postive
F8	HEIH	0.505540327	8.53E-34	postive
F8	HEXA-AS1	0.392292777	7.65E-20	postive
F8	HID1-AS1	0.477926872	6.75E-30	postive
F8	HLA-DQB1-AS1	0.305306552	3.01E-12	postive
F8	HLA-DRB6	0.410691813	9.11E-22	postive
F8	HLX-AS1	0.446039996	8.15E-26	postive
F8	IGFBP7-AS1	0.315349622	5.25E-13	postive
F8	IGFL2-AS1	0.307844212	1.95E-12	postive
F8	IL10RB-DT	0.329155297	4.25E-14	postive
F8	IL6R-AS1	0.466278417	2.35E-28	postive
F8	ILF3-DT	0.421455634	5.98E-23	postive
F8	INTS6L-AS1	0.348487171	1.01E-15	postive
F8	IPO9-AS1	0.324706657	9.68E-14	postive

F8	IQCH-AS1	0.387356862	2.40E-19	postive
F8	IRF1-AS1	0.335966786	1.17E-14	postive
F8	ITGA9-AS1	0.453348709	1.03E-26	postive
F8	JAZF1-AS1	0.322381713	1.48E-13	postive
F8	KANSL1-AS1	0.332902274	2.10E-14	postive
F8	KCNQ1DN	0.350876312	6.23E-16	postive
F8	KDM7A-DT	0.355798775	2.29E-16	postive
F8	KIAA1671-AS1	0.402688949	6.47E-21	postive
F8	KRT8P12	0.374860176	3.97E-18	postive
F8	L3MBTL2-AS1	0.360001974	9.57E-17	postive
F8	LAMA5-AS1	0.389023291	1.63E-19	postive
F8	LANCL1-AS1	0.328517513	4.78E-14	postive
F8	LGALS8-AS1	0.311917829	9.61E-13	postive
F8	LIFR-AS1	0.316767503	4.08E-13	postive
F8	LINC00032	0.320439419	2.11E-13	postive
F8	LINC00163	0.324390915	1.03E-13	postive
F8	LINC00235	0.399964481	1.25E-20	postive
F8	LINC00271	0.317685951	3.46E-13	postive
F8	LINC00278	0.353877207	3.39E-16	postive
F8	LINC00323	0.369294649	1.33E-17	postive
F8	LINC00324	0.341772508	3.81E-15	postive
F8	LINC00339	0.439102951	5.54E-25	postive
F8	LINC00346	0.319517067	2.49E-13	postive
F8	LINC00466	0.342127621	3.55E-15	postive
F8	LINC00467	0.323649564	1.18E-13	postive
F8	LINC00476	0.433600768	2.46E-24	postive
F8	LINC00494	0.39466973	4.38E-20	postive
F8	LINC00551	0.342387473	3.38E-15	postive
F8	LINC00630	0.387412239	2.37E-19	postive
F8	LINC00654	0.333404287	1.91E-14	postive
F8	LINC00663	0.398911747	1.60E-20	postive
F8	LINC00667	0.356545481	1.96E-16	postive
F8	LINC00674	0.324202454	1.06E-13	postive
F8	LINC00702	0.341618184	3.92E-15	postive
F8	LINC00847	0.55972665	1.44E-42	postive
F8	LINC00853	0.328044604	5.22E-14	postive
F8	LINC00863	0.367590643	1.92E-17	postive
F8	LINC00884	0.361992235	6.31E-17	postive
F8	LINC00886	0.339253931	6.22E-15	postive
F8	LINC00888	0.339611592	5.80E-15	postive
F8	LINC00891	0.357174749	1.72E-16	postive
F8	LINC00892	0.351586336	5.40E-16	postive
F8	LINC00899	0.359727156	1.01E-16	postive
F8	LINC00906	0.440493395	3.79E-25	postive
F8	LINC00909	0.411588372	7.28E-22	postive
F8	LINC00910	0.328355005	4.93E-14	postive
F8	LINC00920	0.325233969	8.79E-14	postive
F8	LINC00921	0.493134206	5.33E-32	postive
F8	LINC00924	0.344363362	2.29E-15	postive
F8	LINC00933	0.301342934	5.90E-12	postive

F8	LINC00950	0.310934879	1.14E-12	postive
F8	LINC00957	0.318581695	2.95E-13	postive
F8	LINC00963	0.36713625	2.12E-17	postive
F8	LINC00968	0.478933519	4.94E-30	postive
F8	LINC00987	0.500499945	4.67E-33	postive
F8	LINC01011	0.331814206	2.58E-14	postive
F8	LINC01016	0.311102504	1.11E-12	postive
F8	LINC01023	0.349245455	8.66E-16	postive
F8	LINC01031	0.419879708	8.97E-23	postive
F8	LINC01058	0.378511813	1.77E-18	postive
F8	LINC01063	0.386608833	2.85E-19	postive
F8	LINC01081	0.360160837	9.26E-17	postive
F8	LINC01082	0.322125714	1.55E-13	postive
F8	LINC01093	0.367085365	2.14E-17	postive
F8	LINC01128	0.318527468	2.97E-13	postive
F8	LINC01132	0.375626137	3.35E-18	postive
F8	LINC01135	0.412062391	6.47E-22	postive
F8	LINC01140	0.34090917	4.51E-15	postive
F8	LINC01150	0.347713386	1.18E-15	postive
F8	LINC01151	0.381865391	8.35E-19	postive
F8	LINC01154	0.332410339	2.30E-14	postive
F8	LINC01184	0.500957074	4.01E-33	postive
F8	LINC01197	0.324135048	1.08E-13	postive
F8	LINC01232	0.431937957	3.83E-24	postive
F8	LINC01273	0.409123867	1.34E-21	postive
F8	LINC01275	0.40813868	1.71E-21	postive
F8	LINC01278	0.511795357	9.94E-35	postive
F8	LINC01285	0.389421535	1.49E-19	postive
F8	LINC01293	0.384390213	4.72E-19	postive
F8	LINC01298	0.334695252	1.49E-14	postive
F8	LINC01352	0.507620777	4.19E-34	postive
F8	LINC01354	0.424444723	2.76E-23	postive
F8	LINC01366	0.450311075	2.45E-26	postive
F8	LINC01387	0.373259179	5.64E-18	postive
F8	LINC01412	0.362718982	5.41E-17	postive
F8	LINC01426	0.319115683	2.68E-13	postive
F8	LINC01443	0.312286973	9.00E-13	postive
F8	LINC01497	0.372235156	7.05E-18	postive
F8	LINC01506	0.343585462	2.67E-15	postive
F8	LINC01534	0.313415093	7.38E-13	postive
F8	LINC01550	0.312986045	7.96E-13	postive
F8	LINC01560	0.304476506	3.47E-12	postive
F8	LINC01569	0.306274403	2.55E-12	postive
F8	LINC01585	0.453294691	1.05E-26	postive
F8	LINC01621	0.365493762	3.01E-17	postive
F8	LINC01624	0.408362298	1.62E-21	postive
F8	LINC01686	0.377233531	2.35E-18	postive
F8	LINC01693	0.382670428	6.97E-19	postive
F8	LINC01778	0.354367175	3.07E-16	postive
F8	LINC01842	0.383505962	5.77E-19	postive

F8	LINC01852	0.466749441	2.04E-28	postive
F8	LINC01936	0.483965155	1.02E-30	postive
F8	LINC01968	0.328769276	4.56E-14	postive
F8	LINC02006	0.315871989	4.78E-13	postive
F8	LINC02016	0.328695912	4.63E-14	postive
F8	LINC02035	0.346563308	1.48E-15	postive
F8	LINC02073	0.335560779	1.27E-14	postive
F8	LINC02076	0.323451108	1.22E-13	postive
F8	LINC02099	0.372795624	6.24E-18	postive
F8	LINC02137	0.392190284	7.84E-20	postive
F8	LINC02172	0.320712016	2.01E-13	postive
F8	LINC02202	0.323640422	1.18E-13	postive
F8	LINC02234	0.361316039	7.27E-17	postive
F8	LINC02238	0.317148098	3.81E-13	postive
F8	LINC02243	0.349851516	7.67E-16	postive
F8	LINC02256	0.44081435	3.47E-25	postive
F8	LINC02273	0.31250103	8.67E-13	postive
F8	LINC02285	0.359747285	1.01E-16	postive
F8	LINC02361	0.391470969	9.26E-20	postive
F8	LINC02391	0.31827905	3.11E-13	postive
F8	LINC02412	0.395661666	3.47E-20	postive
F8	LINC02427	0.300909368	6.35E-12	postive
F8	LINC02447	0.40358059	5.22E-21	postive
F8	LINC02519	0.313980694	6.68E-13	postive
F8	LINC02569	0.417250219	1.75E-22	postive
F8	LINC02580	0.34078938	4.61E-15	postive
F8	LINC02586	0.340343532	5.03E-15	postive
F8	LINC02611	0.37478395	4.04E-18	postive
F8	LINC02637	0.396169658	3.08E-20	postive
F8	LINC02705	0.314776272	5.81E-13	postive
F8	LINC02707	0.441307798	3.03E-25	postive
F8	LINC02724	0.328841109	4.50E-14	postive
F8	LINC02731	0.302529853	4.83E-12	postive
F8	LINC02740	0.3391079	6.40E-15	postive
F8	LINC02757	0.46998612	7.70E-29	postive
F8	LINC02773	0.307734949	1.99E-12	postive
F8	LINC02777	0.325140028	8.94E-14	postive
F8	LINC02817	0.331965943	2.51E-14	postive
F8	LINC02861	0.467038789	1.87E-28	postive
F8	LNCTAM34A	0.346294485	1.56E-15	postive
F8	LOH12CR2	0.373003104	5.96E-18	postive
F8	LPAL2	0.357442043	1.63E-16	postive
F8	LPP-AS2	0.489132077	1.95E-31	postive
F8	LRRC37A16P	0.308393297	1.77E-12	postive
F8	LRRC37A17P	0.420740767	7.19E-23	postive
F8	LRRC8C-DT	0.423229626	3.78E-23	postive
F8	LY6E-DT	0.357596689	1.58E-16	postive
F8	LYPLAL1-DT	0.432544422	3.26E-24	postive
F8	MACORIS	0.307991959	1.90E-12	postive
F8	MAFTRR	0.528394019	2.65E-37	postive

F8	MAGI2-AS3	0.427866633	1.13E-23	postive
F8	MAP2K4P1	0.452180687	1.44E-26	postive
F8	MAP4K3-DT	0.330665646	3.20E-14	postive
F8	MAPK6-DT	0.323840893	1.13E-13	postive
F8	MBNL1-AS1	0.315958796	4.71E-13	postive
F8	MDS2	0.305456877	2.94E-12	postive
F8	MED14OS	0.481166248	2.46E-30	postive
F8	MIATNB	0.329821091	3.75E-14	postive
F8	MID1IP1-AS1	0.346979787	1.36E-15	postive
F8	MIR22HG	0.435726237	1.39E-24	postive
F8	MIR3150BHG	0.389724119	1.39E-19	postive
F8	MIR34AHG	0.348578394	9.90E-16	postive
F8	MIR497HG	0.411204845	8.01E-22	postive
F8	MIR99AHG	0.320857592	1.95E-13	postive
F8	MIRLET7BHG	0.309225139	1.54E-12	postive
F8	MKLN1-AS	0.307482299	2.08E-12	postive
F8	MMADHC-DT	0.373182729	5.73E-18	postive
F8	MRPS31P4	0.317085762	3.85E-13	postive
F8	MT1DP	0.386686351	2.80E-19	postive
F8	NAIPP3	0.322263363	1.51E-13	postive
F8	NAPSB	0.332864299	2.11E-14	postive
F8	NBPF25P	0.310676056	1.19E-12	postive
F8	NBR2	0.30638972	2.50E-12	postive
F8	NEBL-AS1	0.420124944	8.42E-23	postive
F8	NIFK-AS1	0.442207979	2.36E-25	postive
F8	NINJ2-AS1	0.313972036	6.69E-13	postive
F8	NKAPP1	0.559034912	1.91E-42	postive
F8	NOP14-AS1	0.339164736	6.33E-15	postive
F8	NORAD	0.372336387	6.90E-18	postive
F8	NR2F1-AS1	0.324037296	1.09E-13	postive
F8	NRSN2-AS1	0.330777837	3.13E-14	postive
F8	NUDT16P1	0.441152894	3.16E-25	postive
F8	OIP5-AS1	0.408131836	1.72E-21	postive
F8	OR4G11P	0.391188347	9.89E-20	postive
F8	OR4G2P	0.365499068	3.01E-17	postive
F8	OR7E47P	0.361059154	7.67E-17	postive
F8	OSER1-DT	0.44375952	1.54E-25	postive
F8	OTUD6B-AS1	0.306232061	2.57E-12	postive
F8	OXCT1-AS1	0.451920896	1.55E-26	postive
F8	PARAL1	0.314206037	6.42E-13	postive
F8	PAXBP1-AS1	0.311178208	1.09E-12	postive
F8	PAXIP1-AS2	0.541392664	2.03E-39	postive
F8	PCBP1-AS1	0.327870192	5.40E-14	postive
F8	PCCA-DT	0.331460252	2.76E-14	postive
F8	PDCD4-AS1	0.335528911	1.27E-14	postive
F8	PINK1-AS	0.418735698	1.20E-22	postive
F8	PLAC9P1	0.30061267	6.67E-12	postive
F8	PMS2P4	0.423759024	3.30E-23	postive
F8	PPP3CB-AS1	0.349180886	8.77E-16	postive
F8	PRELID1P1	0.326322119	7.19E-14	postive

F8	PRKAR2A-AS1	0.398938417	1.59E-20	postive
F8	PRR34	0.39727007	2.37E-20	postive
F8	PSMB8-AS1	0.453486029	9.91E-27	postive
F8	PTENP1	0.362205114	6.03E-17	postive
F8	PTPRD-AS1	0.316995878	3.91E-13	postive
F8	RAET1E-AS1	0.344032399	2.44E-15	postive
F8	RAP2C-AS1	0.506259599	6.68E-34	postive
F8	RARA-AS1	0.318883237	2.79E-13	postive
F8	RASSF1-AS1	0.32840941	4.88E-14	postive
F8	RASSF8-AS1	0.309357645	1.50E-12	postive
F8	RB1-DT	0.301307108	5.94E-12	postive
F8	RBM26-AS1	0.471821728	4.41E-29	postive
F8	RNFT1-DT	0.334160034	1.65E-14	postive
F8	RP11-10C24.1	0.369522096	1.27E-17	postive
F8	RP11-115J16.1	0.403228195	5.68E-21	postive
F8	RP11-136H19.1	0.354620316	2.91E-16	postive
F8	RP11-159G9.5	0.34755599	1.21E-15	postive
F8	RP11-164P12.4	0.337543	8.65E-15	postive
F8	RP11-182J1.1	0.393978225	5.16E-20	postive
F8	RP1-122P22.2	0.390324337	1.21E-19	postive
F8	RP11-285F16.1	0.379888225	1.30E-18	postive
F8	RP11-314C16.1	0.348508104	1.00E-15	postive
F8	RP11-337C18.9	0.310113468	1.32E-12	postive
F8	RP11-462G12.2	0.477562853	7.56E-30	postive
F8	RP11-467J12.4	0.363692644	4.41E-17	postive
F8	RP11-539I5.1	0.407325416	2.09E-21	postive
F8	RP11-58E21.3	0.311151441	1.10E-12	postive
F8	RP11-876N24.4	0.491359635	9.49E-32	postive
F8	RP11-876N24.5	0.447705495	5.11E-26	postive
F8	RP3-337H4.10	0.337177102	9.28E-15	postive
F8	RP4-535B20.1	0.332442934	2.29E-14	postive
F8	RP4-575N6.5	0.391896998	8.39E-20	postive
F8	RP5-1042I8.7	0.322689389	1.40E-13	postive
F8	RP5-1139B12.2	0.313593308	7.16E-13	postive
F8	RPARP-AS1	0.304551068	3.43E-12	postive
F8	RPL13P5	0.31310009	7.81E-13	postive
F8	RPL23AP53	0.362687881	5.45E-17	postive
F8	RPL23AP7	0.3037176	3.95E-12	postive
F8	RPL23AP82	0.394062629	5.06E-20	postive
F8	RPLP1P6	0.336517733	1.05E-14	postive
F8	RPS10P7	0.307744614	1.98E-12	postive
F8	RTCA-AS1	0.364181242	3.98E-17	postive
F8	SAP30-DT	0.379745475	1.34E-18	postive
F8	SBDSP1	0.340010185	5.37E-15	postive
F8	SCAMP1-AS1	0.498210755	1.00E-32	postive
F8	SCAND2P	0.386260298	3.08E-19	postive
F8	SDAD1P1	0.431442935	4.37E-24	postive
F8	SEC22B4P	0.425201659	2.27E-23	postive
F8	SEC24B-AS1	0.423546607	3.48E-23	postive
F8	SEPSECS-AS1	0.338861115	6.71E-15	postive

F8	SEPTIN4-AS1	0.396103886	3.13E-20	postive
F8	SEPTIN7-AS1	0.438203972	7.08E-25	postive
F8	SERTAD4-AS1	0.329907798	3.69E-14	postive
F8	SGMS1-AS1	0.33350648	1.87E-14	postive
F8	SHLD2P1	0.423996587	3.10E-23	postive
F8	SIGLEC17P	0.352371114	4.61E-16	postive
F8	SIGLEC22P	0.396809853	2.64E-20	postive
F8	SLC25A5-AS1	0.501693335	3.13E-33	postive
F8	SMC5-AS1	0.324618033	9.84E-14	postive
F8	SMG7-AS1	0.307002815	2.25E-12	postive
F8	SMIM25	0.427942535	1.11E-23	postive
F8	SNAI3-AS1	0.328097125	5.17E-14	postive
F8	SNHG28	0.332490614	2.27E-14	postive
F8	SNHG31	0.341367691	4.12E-15	postive
F8	SPART-AS1	0.357733935	1.53E-16	postive
F8	SPATA41	0.37623855	2.93E-18	postive
F8	SRP14-AS1	0.306229586	2.57E-12	postive
F8	SSR4P1	0.337926098	8.04E-15	postive
F8	ST20-AS1	0.386126693	3.18E-19	postive
F8	ST3GAL6-AS1	0.391555797	9.08E-20	postive
F8	STARD7-AS1	0.320129898	2.23E-13	postive
F8	STK24-AS1	0.457053757	3.55E-27	postive
F8	STK4-AS1	0.406593023	2.50E-21	postive
F8	STRA6LP	0.315419597	5.18E-13	postive
F8	SVIL-AS1	0.408836601	1.44E-21	postive
F8	SYS1-DBNDD2	0.312417781	8.80E-13	postive
F8	TAF1A-AS1	0.356338509	2.05E-16	postive
F8	TBX5-AS1	0.48802425	2.79E-31	postive
F8	TCAF2P1	0.31252248	8.64E-13	postive
F8	TM4SF19-AS1	0.32174659	1.66E-13	postive
F8	TMC3-AS1	0.322584776	1.43E-13	postive
F8	TMCC1-AS1	0.326887495	6.48E-14	postive
F8	TMEM161B-AS1	0.313235063	7.62E-13	postive
F8	TMEM202-AS1	0.409529676	1.21E-21	postive
F8	TMEM231P1	0.363578511	4.52E-17	postive
F8	TMEM44-AS1	0.308213501	1.83E-12	postive
F8	TMEM99	0.327311652	5.99E-14	postive
F8	TMLHE-AS1	0.369730107	1.21E-17	postive
F8	TOB1-AS1	0.37842221	1.81E-18	postive
F8	TOLLIP-AS1	0.359051937	1.17E-16	postive
F8	TP53TG1	0.349332807	8.51E-16	postive
F8	TP73-AS1	0.347894032	1.14E-15	postive
F8	TPRG1-AS1	0.488373236	2.49E-31	postive
F8	TRAF3IP2-AS1	0.346123556	1.62E-15	postive
F8	TRAM2-AS1	0.491233438	9.89E-32	postive
F8	TRIM52-AS1	0.407291979	2.11E-21	postive
F8	TSPOAP1-AS1	0.403429046	5.41E-21	postive
F8	TSTD3	0.375610707	3.37E-18	postive
F8	TTC28-AS1	0.402907732	6.14E-21	postive
F8	TTTY18	0.322437302	1.47E-13	postive

F8	TTTY20	0.369751446	1.21E-17	postive
F8	TUBBP9	0.400954991	9.83E-21	postive
F8	TULP3P1	0.337513286	8.70E-15	postive
F8	UBA6-AS1	0.352919266	4.12E-16	postive
F8	UBE2D3-AS1	0.342731707	3.16E-15	postive
F8	UGDH-AS1	0.36051393	8.60E-17	postive
F8	URB1-AS1	0.378856589	1.64E-18	postive
F8	USP27X-AS1	0.582962023	7.55E-47	postive
F8	USP46-AS1	0.409301675	1.29E-21	postive
F8	UXT-AS1	0.438811729	6.00E-25	postive
F8	WAC-AS1	0.357077944	1.76E-16	postive
F8	WDFY3-AS2	0.48983558	1.55E-31	postive
F8	WEE2-AS1	0.343657909	2.63E-15	postive
F8	WWC2-AS2	0.341357868	4.13E-15	postive
F8	WWTR1-AS1	0.420032628	8.62E-23	postive
F8	Z68871.1	0.308848642	1.64E-12	postive
F8	Z82185.1	0.396337039	2.96E-20	postive
F8	Z82246.1	0.406782816	2.39E-21	postive
F8	Z92544.1	0.325783293	7.94E-14	postive
F8	Z97634.1	0.312806081	8.22E-13	postive
F8	Z97989.1	0.383040451	6.41E-19	postive
F8	Z99572.1	0.326010666	7.61E-14	postive
F8	ZBED3-AS1	0.33083009	3.10E-14	postive
F8	ZBED5-AS1	0.305937365	2.71E-12	postive
F8	ZBTB11-AS1	0.304267	3.60E-12	postive
F8	ZBTB44-DT	0.329657262	3.87E-14	postive
F8	ZFX-AS1	0.331356065	2.81E-14	postive
F8	ZFY-AS1	0.338277074	7.51E-15	postive
F8	ZNF205-AS1	0.402932215	6.10E-21	postive
F8	ZNF213-AS1	0.30778851	1.97E-12	postive
F8	ZNF426-DT	0.370423728	1.04E-17	postive
F8	ZNF561-AS1	0.363492799	4.60E-17	postive
F8	ZNF658B	0.505618624	8.30E-34	postive
F8	ZNF788P	0.395411111	3.68E-20	postive
F8	ZSCAN16-AS1	0.396906346	2.58E-20	postive
FDX1	AC002467.1	0.306835854	2.32E-12	postive
FDX1	AC004241.1	0.336852793	9.88E-15	postive
FDX1	AC005034.4	0.319253128	2.61E-13	postive
FDX1	AC005176.1	0.316023448	4.65E-13	postive
FDX1	AC005392.1	0.403885556	4.84E-21	postive
FDX1	AC005532.1	0.519537489	6.52E-36	postive
FDX1	AC008440.10	0.422777632	4.25E-23	postive
FDX1	AC008750.7	0.371041739	9.14E-18	postive
FDX1	AC008753.2	0.623714236	3.00E-55	postive
FDX1	AC008753.3	0.324149274	1.07E-13	postive
FDX1	AC008771.1	0.324053423	1.09E-13	postive
FDX1	AC008915.3	0.342663558	3.20E-15	postive
FDX1	AC009061.2	0.312893144	8.09E-13	postive
FDX1	AC009779.3	0.394018016	5.11E-20	postive
FDX1	AC010328.1	0.531868455	7.36E-38	postive

FDX1	AC010969.2	0.308596	1.71E-12	postive
FDX1	AC011453.1	0.489538319	1.71E-31	postive
FDX1	AC011487.2	0.661449411	3.23E-64	postive
FDX1	AC015849.5	0.445052192	1.07E-25	postive
FDX1	AC015921.1	0.312253416	9.06E-13	postive
FDX1	AC015922.2	0.308935631	1.61E-12	postive
FDX1	AC015922.3	0.327108032	6.22E-14	postive
FDX1	AC016747.1	0.301300519	5.94E-12	postive
FDX1	AC018781.1	0.577887726	6.95E-46	postive
FDX1	AC018904.2	0.362829524	5.29E-17	postive
FDX1	AC019209.2	0.330731312	3.16E-14	postive
FDX1	AC020914.1	0.43307161	2.83E-24	postive
FDX1	AC022217.4	0.328998013	4.37E-14	postive
FDX1	AC022613.2	0.37200586	7.41E-18	postive
FDX1	AC022872.1	0.478365691	5.89E-30	postive
FDX1	AC025946.1	0.632870938	2.60E-57	postive
FDX1	AC026765.3	0.324640972	9.80E-14	postive
FDX1	AC026785.3	0.410123733	1.05E-21	postive
FDX1	AC046134.2	0.333281564	1.95E-14	postive
FDX1	AC064834.1	0.636820259	3.19E-58	postive
FDX1	AC067852.2	0.364297569	3.88E-17	postive
FDX1	AC068338.2	0.305400927	2.97E-12	postive
FDX1	AC068870.2	0.318099205	3.21E-13	postive
FDX1	AC069029.1	0.311835459	9.75E-13	postive
FDX1	AC073508.3	0.310104126	1.32E-12	postive
FDX1	AC083799.1	0.307678233	2.01E-12	postive
FDX1	AC090541.1	0.492393452	6.78E-32	postive
FDX1	AC090826.1	0.528677671	2.39E-37	postive
FDX1	AC091045.2	0.393386504	5.92E-20	postive
FDX1	AC092078.1	0.346975398	1.36E-15	postive
FDX1	AC092118.1	0.321736719	1.67E-13	postive
FDX1	AC092687.3	0.359038323	1.17E-16	postive
FDX1	AC092718.4	0.313830717	6.86E-13	postive
FDX1	AC092809.3	0.689679349	7.90E-72	postive
FDX1	AC096536.2	0.313528142	7.24E-13	postive
FDX1	AC099329.2	0.333579036	1.85E-14	postive
FDX1	AC099567.1	0.635652045	5.96E-58	postive
FDX1	AC103843.1	0.370264349	1.08E-17	postive
FDX1	AC105036.3	0.362447765	5.73E-17	postive
FDX1	AC106795.1	0.311599017	1.02E-12	postive
FDX1	AC108112.1	0.646713226	1.45E-60	postive
FDX1	AC110288.1	0.324743485	9.62E-14	postive
FDX1	AC110619.1	0.383644958	5.59E-19	postive
FDX1	AC112220.2	0.327045593	6.29E-14	postive
FDX1	AC114812.2	0.33956425	5.85E-15	postive
FDX1	AC116913.1	0.359718786	1.02E-16	postive
FDX1	AC119677.1	0.313958008	6.71E-13	postive
FDX1	AC121764.3	0.416203887	2.29E-22	postive
FDX1	AC123788.1	0.337229073	9.19E-15	postive
FDX1	AC125807.2	0.306635653	2.40E-12	postive

FDX1	AC142086.1	0.447091211	6.07E-26	postive
FDX1	AC239809.3	0.435724835	1.39E-24	postive
FDX1	AC241377.3	0.377115856	2.41E-18	postive
FDX1	AC243967.3	0.625672493	1.10E-55	postive
FDX1	AC245041.2	0.50279467	2.16E-33	postive
FDX1	AF067845.2	0.451414089	1.79E-26	postive
FDX1	AJ003147.2	0.339810595	5.58E-15	postive
FDX1	AL049555.1	0.341792918	3.79E-15	postive
FDX1	AL049597.2	0.301448318	5.80E-12	postive
FDX1	AL049796.1	0.432973942	2.91E-24	postive
FDX1	AL078581.2	0.304621753	3.39E-12	postive
FDX1	AL078612.3	0.481440835	2.25E-30	postive
FDX1	AL080317.2	0.345119443	1.97E-15	postive
FDX1	AL121944.2	0.406745581	2.41E-21	postive
FDX1	AL138760.1	0.309541359	1.45E-12	postive
FDX1	AL139002.1	0.592466414	1.06E-48	postive
FDX1	AL139384.1	0.300695597	6.58E-12	postive
FDX1	AL158066.1	0.460852581	1.17E-27	postive
FDX1	AL161421.1	0.426343683	1.68E-23	postive
FDX1	AL161431.1	0.475729879	1.33E-29	postive
FDX1	AL354674.1	0.665534829	2.88E-65	postive
FDX1	AL354920.1	0.306195189	2.59E-12	postive
FDX1	AL355001.2	0.324322082	1.04E-13	postive
FDX1	AL390294.1	0.684061605	3.03E-70	postive
FDX1	AL390835.1	0.491199281	1.00E-31	postive
FDX1	AL391704.1	0.428393915	9.81E-24	postive
FDX1	AL441992.2	0.316339332	4.40E-13	postive
FDX1	AL450322.1	0.386467502	2.94E-19	postive
FDX1	AL450326.1	0.309648585	1.43E-12	postive
FDX1	AL512347.1	0.586990578	1.26E-47	postive
FDX1	AL512363.1	0.452309544	1.39E-26	postive
FDX1	AL513318.2	0.352679651	4.33E-16	postive
FDX1	AL513534.3	0.303328278	4.22E-12	postive
FDX1	AL591806.1	0.301911988	5.36E-12	postive
FDX1	AL596223.1	0.457941846	2.74E-27	postive
FDX1	AL627443.1	0.300098944	7.27E-12	postive
FDX1	AL645608.1	0.339787622	5.61E-15	postive
FDX1	AL691403.1	0.622542132	5.45E-55	postive
FDX1	AP000442.1	0.31658325	4.21E-13	postive
FDX1	AP000679.1	0.313187557	7.69E-13	postive
FDX1	AP000755.1	0.477987851	6.63E-30	postive
FDX1	AP001318.2	0.4050003	3.69E-21	postive
FDX1	AP001372.2	0.362895496	5.22E-17	postive
FDX1	AP001631.1	0.491364717	9.48E-32	postive
FDX1	AP001816.1	0.316104372	4.59E-13	postive
FDX1	AP002387.2	0.307033599	2.24E-12	postive
FDX1	AP002478.1	0.300059089	7.32E-12	postive
FDX1	AP005019.1	0.418437344	1.30E-22	postive
FDX1	AP005137.2	0.382532008	7.19E-19	postive
FDX1	AP005202.1	0.630479996	9.13E-57	postive

FDX1	ARMCX5-GPRASP2	0.329759071	3.79E-14	postive
FDX1	ATP2B1-AS1	0.337393672	8.91E-15	postive
FDX1	BASP1-AS1	0.474660723	1.85E-29	postive
FDX1	C22orf46	0.307474133	2.08E-12	postive
FDX1	C6orf99	0.332441265	2.29E-14	postive
FDX1	C9orf170	0.413483267	4.54E-22	postive
FDX1	CA5BP1	0.305926448	2.71E-12	postive
FDX1	CATSPER2P1	0.329118019	4.28E-14	postive
FDX1	CFL1P1	0.321791288	1.65E-13	postive
FDX1	CIDECP1	0.363362294	4.73E-17	postive
FDX1	CRIM1-DT	0.666323195	1.80E-65	postive
FDX1	CTBP1-DT	0.335625723	1.25E-14	postive
FDX1	DLGAP1-AS1	0.319731782	2.39E-13	postive
FDX1	DOCK8-AS1	0.342405635	3.36E-15	postive
FDX1	EIF3J-DT	0.34102123	4.41E-15	postive
FDX1	ELF3-AS1	0.303846935	3.87E-12	postive
FDX1	EMC1-AS1	0.304633822	3.38E-12	postive
FDX1	ENTPD3-AS1	0.301226376	6.02E-12	postive
FDX1	ERVH48-1	0.541220794	2.16E-39	postive
FDX1	FAM25BP	0.608876768	4.76E-52	postive
FDX1	FAM86C2P	0.302833611	4.59E-12	postive
FDX1	FAM86EP	0.369274313	1.34E-17	postive
FDX1	FGD5-AS1	0.359509076	1.06E-16	postive
FDX1	FP236240.1	0.478347974	5.93E-30	postive
FDX1	FRG1HP	0.322774447	1.38E-13	postive
FDX1	FZD4-DT	0.309244448	1.53E-12	postive
FDX1	GASAL1	0.361891326	6.44E-17	postive
FDX1	GATA3-AS1	0.70485105	2.72E-76	postive
FDX1	GSEC	0.405823778	3.02E-21	postive
FDX1	HEIH	0.315176649	5.41E-13	postive
FDX1	HERC2P5	0.308571982	1.72E-12	postive
FDX1	HMGB1P5	0.326045886	7.57E-14	postive
FDX1	IL6-AS1	0.311446769	1.04E-12	postive
FDX1	IQCH-AS1	0.391563342	9.07E-20	postive
FDX1	KRT18P55	0.324792379	9.53E-14	postive
FDX1	KRT223P	0.594213035	4.76E-49	postive
FDX1	LAPTM4A-DT	0.315026465	5.55E-13	postive
FDX1	LINC00189	0.43572977	1.39E-24	postive
FDX1	LINC00243	0.500269312	5.04E-33	postive
FDX1	LINC00337	0.310610897	1.21E-12	postive
FDX1	LINC00339	0.329410639	4.05E-14	postive
FDX1	LINC00370	0.681545184	1.51E-69	postive
FDX1	LINC00476	0.304330198	3.56E-12	postive
FDX1	LINC00592	0.406236143	2.73E-21	postive
FDX1	LINC00857	0.362544635	5.62E-17	postive
FDX1	LINC00963	0.388113999	2.01E-19	postive
FDX1	LINC01059	0.300320499	7.01E-12	postive
FDX1	LINC01137	0.39986518	1.28E-20	postive
FDX1	LINC01184	0.337889582	8.09E-15	postive
FDX1	LINC01230	0.452428606	1.34E-26	postive

FDX1	LINC01278	0.371212943	8.80E-18	postive
FDX1	LINC01300	0.309728458	1.41E-12	postive
FDX1	LINC01391	0.466683019	2.08E-28	postive
FDX1	LINC01447	0.369304737	1.33E-17	postive
FDX1	LINC01588	0.310967489	1.13E-12	postive
FDX1	LINC01623	0.362148228	6.11E-17	postive
FDX1	LINC01714	0.335601194	1.26E-14	postive
FDX1	LINC01716	0.635710708	5.78E-58	postive
FDX1	LINC01731	0.33260688	2.22E-14	postive
FDX1	LINC01825	0.659049715	1.31E-63	postive
FDX1	LINC01960	0.691229003	2.85E-72	postive
FDX1	LINC02111	0.317808222	3.38E-13	postive
FDX1	LINC02136	0.319116785	2.68E-13	postive
FDX1	LINC02164	0.670565402	1.39E-66	postive
FDX1	LINC02178	0.316942663	3.95E-13	postive
FDX1	LINC02255	0.42307956	3.93E-23	postive
FDX1	LINC02518	0.63750161	2.22E-58	postive
FDX1	LINC02570	0.433561409	2.48E-24	postive
FDX1	LINC02617	0.308711745	1.68E-12	postive
FDX1	LINC02637	0.426587566	1.58E-23	postive
FDX1	LINC02639	0.406528921	2.54E-21	postive
FDX1	LINC02669	0.471119826	5.46E-29	postive
FDX1	LINC02732	0.396622841	2.76E-20	postive
FDX1	LINC02805	0.394039803	5.08E-20	postive
FDX1	LINC02814	0.320565271	2.06E-13	postive
FDX1	LIPC-AS1	0.437545226	8.47E-25	postive
FDX1	LNX1-AS2	0.344845818	2.08E-15	postive
FDX1	MAGEA10-MAGEA5	0.349642826	7.99E-16	postive
FDX1	MAP4K3-DT	0.328479614	4.82E-14	postive
FDX1	MAPKAPK5-AS1	0.368419182	1.61E-17	postive
FDX1	MRPL20-AS1	0.321056253	1.88E-13	postive
FDX1	MTUS2-AS1	0.369732839	1.21E-17	postive
FDX1	NAPA-AS1	0.342253502	3.47E-15	postive
FDX1	NIFK-AS1	0.333427448	1.90E-14	postive
FDX1	NOP14-AS1	0.343303125	2.82E-15	postive
FDX1	NRAV	0.339065066	6.45E-15	postive
FDX1	NRSN2-AS1	0.3262684	7.26E-14	postive
FDX1	NUDT16P1	0.30535498	2.99E-12	postive
FDX1	NUTM2A-AS1	0.382163465	7.81E-19	postive
FDX1	OIP5-AS1	0.37436779	4.42E-18	postive
FDX1	OLMALINC	0.310266471	1.28E-12	postive
FDX1	OSER1-DT	0.300735562	6.54E-12	postive
FDX1	PLAC9P1	0.359485073	1.07E-16	postive
FDX1	PLEKHA8P1	0.340046745	5.33E-15	postive
FDX1	PLUT	0.448104836	4.57E-26	postive
FDX1	PPIAP46	0.679942695	4.17E-69	postive
FDX1	PSG10P	0.668480968	4.92E-66	postive
FDX1	RAB6C-AS1	0.508329598	3.29E-34	postive
FDX1	RARA-AS1	0.303182002	4.33E-12	postive
FDX1	RBM15-AS1	0.308887911	1.63E-12	postive

FDX1	RNF216P1	0.313411144	7.39E-13	postive
FDX1	RP11-10C24.1	0.31173342	9.92E-13	postive
FDX1	RP11-379F12.3	0.69116619	2.97E-72	postive
FDX1	RP5-1112D6.4	0.35476493	2.83E-16	postive
FDX1	RPL23AP7	0.307214583	2.17E-12	postive
FDX1	RPL23AP82	0.330548341	3.27E-14	postive
FDX1	SLC2A1-AS1	0.325234525	8.79E-14	postive
FDX1	SLC9A7P1	0.313573482	7.18E-13	postive
FDX1	SLFNL1-AS1	0.532724912	5.35E-38	postive
FDX1	SPINT1-AS1	0.36626818	2.55E-17	postive
FDX1	STARD7-AS1	0.338879121	6.69E-15	postive
FDX1	STX17-AS1	0.338426796	7.30E-15	postive
FDX1	STX18-AS1	0.30064831	6.63E-12	postive
FDX1	SVIL-AS1	0.314401747	6.20E-13	postive
FDX1	TBC1D8-AS1	0.336682929	1.02E-14	postive
FDX1	TMEM202-AS1	0.320839739	1.96E-13	postive
FDX1	TMEM99	0.396947768	2.56E-20	postive
FDX1	TOB1-AS1	0.309157138	1.55E-12	postive
FDX1	TPM3P9	0.362054196	6.23E-17	postive
FDX1	TPTE2P2	0.423777115	3.28E-23	postive
FDX1	TRAM2-AS1	0.344439682	2.25E-15	postive
FDX1	TSTD3	0.370812546	9.60E-18	postive
FDX1	UBA6-AS1	0.350080355	7.32E-16	postive
FDX1	UBE2D3-AS1	0.387745565	2.19E-19	postive
FDX1	USP46-AS1	0.365117318	3.26E-17	postive
FDX1	WWC2-AS2	0.301952166	5.33E-12	postive
FDX1	Z99943.1	0.301702415	5.56E-12	postive
FDX1	ZNF561-AS1	0.310078009	1.32E-12	postive
FDX1	ZNF687-AS1	0.310570227	1.22E-12	postive
GCSH	AC000068.1	0.307471133	2.08E-12	postive
GCSH	AC002116.2	0.318337293	3.08E-13	postive
GCSH	AC002310.1	0.372983404	5.99E-18	postive
GCSH	AC002467.1	0.379861392	1.31E-18	postive
GCSH	AC002470.1	0.419097242	1.10E-22	postive
GCSH	AC004241.1	0.348118248	1.09E-15	postive
GCSH	AC004241.2	0.321609784	1.70E-13	postive
GCSH	AC004241.3	0.318929588	2.77E-13	postive
GCSH	AC004477.1	0.316475678	4.29E-13	postive
GCSH	AC004596.1	0.346351634	1.54E-15	postive
GCSH	AC004771.2	0.313131974	7.76E-13	postive
GCSH	AC004803.1	0.38569245	3.51E-19	postive
GCSH	AC004812.2	0.387720231	2.21E-19	postive
GCSH	AC004943.2	0.42993892	6.52E-24	postive
GCSH	AC005034.4	0.440388303	3.90E-25	postive
GCSH	AC005034.6	0.304415284	3.51E-12	postive
GCSH	AC005076.2	0.304125483	3.69E-12	postive
GCSH	AC005229.4	0.346833341	1.40E-15	postive
GCSH	AC005288.1	0.398778894	1.66E-20	postive
GCSH	AC005562.1	0.381825145	8.43E-19	postive
GCSH	AC005670.3	0.355992931	2.20E-16	postive

GCSH	AC005837.1	0.334688694	1.50E-14	postive
GCSH	AC005920.4	0.334125916	1.66E-14	postive
GCSH	AC006001.2	0.375227723	3.66E-18	postive
GCSH	AC006213.1	0.30085144	6.41E-12	postive
GCSH	AC006252.1	0.387160132	2.51E-19	postive
GCSH	AC006333.1	0.362068995	6.21E-17	postive
GCSH	AC006449.2	0.371838279	7.69E-18	postive
GCSH	AC006449.5	0.325776678	7.95E-14	postive
GCSH	AC006449.7	0.376303475	2.89E-18	postive
GCSH	AC007032.1	0.391120674	1.01E-19	postive
GCSH	AC007342.4	0.353339552	3.78E-16	postive
GCSH	AC007383.2	0.338341084	7.42E-15	postive
GCSH	AC007541.1	0.336676428	1.02E-14	postive
GCSH	AC008014.1	0.312309487	8.97E-13	postive
GCSH	AC008267.5	0.384064844	5.08E-19	postive
GCSH	AC008438.1	0.317686983	3.46E-13	postive
GCSH	AC008443.1	0.369314292	1.33E-17	postive
GCSH	AC008608.2	0.339944057	5.44E-15	postive
GCSH	AC008915.3	0.386682225	2.80E-19	postive
GCSH	AC008966.1	0.338018863	7.89E-15	postive
GCSH	AC009061.2	0.413680498	4.32E-22	postive
GCSH	AC009065.9	0.301778059	5.49E-12	postive
GCSH	AC009113.1	0.381062937	1.00E-18	postive
GCSH	AC009118.2	0.326544091	6.90E-14	postive
GCSH	AC009171.2	0.375451778	3.49E-18	postive
GCSH	AC009275.1	0.326385379	7.11E-14	postive
GCSH	AC009309.2	0.391048005	1.02E-19	postive
GCSH	AC009779.3	0.422838417	4.19E-23	postive
GCSH	AC010442.1	0.326370089	7.13E-14	postive
GCSH	AC010504.1	0.317835198	3.37E-13	postive
GCSH	AC010531.6	0.318737984	2.86E-13	postive
GCSH	AC010536.1	0.302726869	4.67E-12	postive
GCSH	AC010643.1	0.315579942	5.04E-13	postive
GCSH	AC010655.4	0.300616445	6.67E-12	postive
GCSH	AC010789.1	0.322271778	1.51E-13	postive
GCSH	AC010809.1	0.343758822	2.58E-15	postive
GCSH	AC010864.1	0.303048459	4.43E-12	postive
GCSH	AC010894.2	0.419451804	1.00E-22	postive
GCSH	AC010913.1	0.354032742	3.28E-16	postive
GCSH	AC012063.1	0.368427417	1.61E-17	postive
GCSH	AC012073.1	0.370941765	9.34E-18	postive
GCSH	AC012146.1	0.300087478	7.29E-12	postive
GCSH	AC012184.3	0.435348456	1.54E-24	postive
GCSH	AC012306.2	0.406995123	2.27E-21	postive
GCSH	AC012313.1	0.300684974	6.59E-12	postive
GCSH	AC012360.3	0.339035306	6.49E-15	postive
GCSH	AC012467.2	0.382377582	7.44E-19	postive
GCSH	AC012510.1	0.323543846	1.20E-13	postive
GCSH	AC012555.1	0.318206106	3.15E-13	postive
GCSH	AC012640.4	0.332521783	2.26E-14	postive

GCSH	AC012676.1	0.359267254	1.12E-16	postive
GCSH	AC015912.3	0.301773446	5.49E-12	postive
GCSH	AC015982.2	0.386538694	2.89E-19	postive
GCSH	AC016065.1	0.503228791	1.87E-33	postive
GCSH	AC016394.2	0.385420434	3.73E-19	postive
GCSH	AC016405.3	0.314452404	6.15E-13	postive
GCSH	AC016727.1	0.307413331	2.10E-12	postive
GCSH	AC016747.1	0.362218673	6.02E-17	postive
GCSH	AC016876.2	0.402850156	6.23E-21	postive
GCSH	AC016876.3	0.353120313	3.95E-16	postive
GCSH	AC016888.1	0.333316481	1.94E-14	postive
GCSH	AC017033.1	0.30408097	3.71E-12	postive
GCSH	AC018521.5	0.360502193	8.62E-17	postive
GCSH	AC018647.2	0.309616949	1.43E-12	postive
GCSH	AC018690.1	0.300081419	7.29E-12	postive
GCSH	AC019069.1	0.375440445	3.49E-18	postive
GCSH	AC020663.2	0.386214833	3.11E-19	postive
GCSH	AC020904.2	0.311246525	1.08E-12	postive
GCSH	AC020915.3	0.305335524	3.00E-12	postive
GCSH	AC021087.1	0.329447817	4.02E-14	postive
GCSH	AC022007.1	0.33374309	1.79E-14	postive
GCSH	AC022167.2	0.30269729	4.70E-12	postive
GCSH	AC022167.4	0.322578732	1.43E-13	postive
GCSH	AC022211.3	0.402559215	6.68E-21	postive
GCSH	AC022613.2	0.302818669	4.60E-12	postive
GCSH	AC023043.1	0.410213852	1.03E-21	postive
GCSH	AC023043.4	0.307176948	2.19E-12	postive
GCSH	AC023090.1	0.318845929	2.81E-13	postive
GCSH	AC023509.3	0.331698481	2.64E-14	postive
GCSH	AC024267.7	0.301448028	5.80E-12	postive
GCSH	AC024361.2	0.384250054	4.87E-19	postive
GCSH	AC024575.2	0.333627995	1.83E-14	postive
GCSH	AC025265.1	0.329679913	3.85E-14	postive
GCSH	AC025682.2	0.322890137	1.35E-13	postive
GCSH	AC026304.1	0.321814058	1.64E-13	postive
GCSH	AC026401.3	0.343315623	2.81E-15	postive
GCSH	AC026471.1	0.369950386	1.16E-17	postive
GCSH	AC026691.1	0.304063946	3.73E-12	postive
GCSH	AC027097.1	0.324936538	9.28E-14	postive
GCSH	AC027601.1	0.304845187	3.26E-12	postive
GCSH	AC027644.3	0.359272797	1.11E-16	postive
GCSH	AC027682.6	0.341433466	4.07E-15	postive
GCSH	AC036176.1	0.324387917	1.03E-13	postive
GCSH	AC040169.1	0.320445522	2.11E-13	postive
GCSH	AC040970.1	0.341392518	4.10E-15	postive
GCSH	AC046134.2	0.446694207	6.79E-26	postive
GCSH	AC053503.1	0.331912171	2.53E-14	postive
GCSH	AC061992.2	0.302235353	5.08E-12	postive
GCSH	AC064807.1	0.429147895	8.04E-24	postive
GCSH	AC064836.3	0.314798829	5.78E-13	postive

GCSH	AC067852.2	0.526817256	4.72E-37	postive
GCSH	AC068025.1	0.33924143	6.23E-15	postive
GCSH	AC068228.2	0.308049706	1.88E-12	postive
GCSH	AC068338.2	0.363566773	4.53E-17	postive
GCSH	AC068473.5	0.351588682	5.40E-16	postive
GCSH	AC068831.2	0.369732051	1.21E-17	postive
GCSH	AC069185.1	0.330514826	3.29E-14	postive
GCSH	AC069544.1	0.379287472	1.49E-18	postive
GCSH	AC073195.2	0.360029216	9.52E-17	postive
GCSH	AC073342.1	0.305450961	2.94E-12	postive
GCSH	AC073508.3	0.361061619	7.67E-17	postive
GCSH	AC073529.1	0.342169791	3.52E-15	postive
GCSH	AC073569.2	0.358345617	1.35E-16	postive
GCSH	AC073585.1	0.322613945	1.42E-13	postive
GCSH	AC073611.1	0.35081377	6.31E-16	postive
GCSH	AC073842.1	0.311450998	1.04E-12	postive
GCSH	AC073896.2	0.378761513	1.67E-18	postive
GCSH	AC074117.1	0.404645824	4.03E-21	postive
GCSH	AC079145.1	0.31433302	6.28E-13	postive
GCSH	AC079305.2	0.366924607	2.22E-17	postive
GCSH	AC079807.1	0.402006586	7.63E-21	postive
GCSH	AC079922.2	0.336710591	1.02E-14	postive
GCSH	AC080112.2	0.465005276	3.43E-28	postive
GCSH	AC083799.1	0.338642263	7.00E-15	postive
GCSH	AC083880.1	0.317260444	3.73E-13	postive
GCSH	AC087741.2	0.366403099	2.48E-17	postive
GCSH	AC090204.1	0.351619195	5.36E-16	postive
GCSH	AC090587.1	0.348766657	9.53E-16	postive
GCSH	AC090970.2	0.32784769	5.42E-14	postive
GCSH	AC091057.1	0.343198926	2.88E-15	postive
GCSH	AC091153.3	0.413827429	4.16E-22	postive
GCSH	AC091271.1	0.355361372	2.50E-16	postive
GCSH	AC091729.3	0.332596651	2.22E-14	postive
GCSH	AC092115.4	0.456995659	3.61E-27	postive
GCSH	AC092118.1	0.355544868	2.41E-16	postive
GCSH	AC092614.1	0.322310401	1.50E-13	postive
GCSH	AC092687.3	0.304667933	3.36E-12	postive
GCSH	AC092718.2	0.460817992	1.18E-27	postive
GCSH	AC092718.4	0.508077124	3.59E-34	postive
GCSH	AC092718.5	0.31336001	7.46E-13	postive
GCSH	AC092720.1	0.343769387	2.57E-15	postive
GCSH	AC092803.1	0.358922368	1.20E-16	postive
GCSH	AC093249.6	0.362415389	5.77E-17	postive
GCSH	AC093673.1	0.348466854	1.01E-15	postive
GCSH	AC096536.2	0.402099978	7.46E-21	postive
GCSH	AC097358.2	0.369673103	1.23E-17	postive
GCSH	AC098487.1	0.353298294	3.81E-16	postive
GCSH	AC098820.2	0.430317787	5.90E-24	postive
GCSH	AC099343.4	0.304940516	3.21E-12	postive
GCSH	AC099518.1	0.314513485	6.08E-13	postive

GCSH	AC099778.1	0.320067933	2.25E-13	postive
GCSH	AC099850.2	0.402194143	7.29E-21	postive
GCSH	AC099850.4	0.359595394	1.04E-16	postive
GCSH	AC100810.1	0.367194913	2.09E-17	postive
GCSH	AC100847.1	0.354426014	3.03E-16	postive
GCSH	AC100861.1	0.365172829	3.22E-17	postive
GCSH	AC104561.1	0.356379277	2.03E-16	postive
GCSH	AC104561.3	0.350518657	6.70E-16	postive
GCSH	AC104667.2	0.332577787	2.23E-14	postive
GCSH	AC105036.3	0.402750252	6.38E-21	postive
GCSH	AC105285.1	0.323853862	1.13E-13	postive
GCSH	AC106795.1	0.402055426	7.54E-21	postive
GCSH	AC107032.2	0.373745671	5.07E-18	postive
GCSH	AC107068.1	0.387095425	2.55E-19	postive
GCSH	AC107214.1	0.366409787	2.48E-17	postive
GCSH	AC108860.2	0.346482597	1.50E-15	postive
GCSH	AC109322.1	0.388419879	1.88E-19	postive
GCSH	AC109347.1	0.318495804	2.99E-13	postive
GCSH	AC112220.2	0.334183942	1.65E-14	postive
GCSH	AC114271.1	0.33892992	6.62E-15	postive
GCSH	AC114341.1	0.308148913	1.85E-12	postive
GCSH	AC115618.1	0.410953536	8.53E-22	postive
GCSH	AC116036.2	0.387486259	2.33E-19	postive
GCSH	AC124016.2	0.314963037	5.62E-13	postive
GCSH	AC124283.2	0.305717195	2.81E-12	postive
GCSH	AC125257.1	0.415527231	2.71E-22	postive
GCSH	AC125437.1	0.332155866	2.42E-14	postive
GCSH	AC125494.1	0.322944633	1.34E-13	postive
GCSH	AC131009.3	0.380480722	1.14E-18	postive
GCSH	AC131011.2	0.310429195	1.25E-12	postive
GCSH	AC131235.3	0.362587122	5.57E-17	postive
GCSH	AC132008.2	0.323019695	1.32E-13	postive
GCSH	AC132938.4	0.4180396	1.43E-22	postive
GCSH	AC133528.2	0.404172068	4.52E-21	postive
GCSH	AC133552.2	0.340773831	4.63E-15	postive
GCSH	AC133552.5	0.317673259	3.47E-13	postive
GCSH	AC135048.3	0.309757376	1.40E-12	postive
GCSH	AC135050.1	0.393304765	6.04E-20	postive
GCSH	AC138696.2	0.350639574	6.54E-16	postive
GCSH	AC139530.1	0.392072214	8.05E-20	postive
GCSH	AC139887.2	0.315879156	4.77E-13	postive
GCSH	AC141586.1	0.339202552	6.28E-15	postive
GCSH	AC231981.1	0.354130539	3.22E-16	postive
GCSH	AC243919.1	0.309398492	1.49E-12	postive
GCSH	AC245140.2	0.303490921	4.11E-12	postive
GCSH	ACVR2B-AS1	0.390444108	1.18E-19	postive
GCSH	AFG3L1P	0.368668222	1.53E-17	postive
GCSH	AL008721.1	0.304131182	3.68E-12	postive
GCSH	AL020996.1	0.317410795	3.63E-13	postive
GCSH	AL023803.1	0.305665988	2.83E-12	postive

GCSH	AL024498.1	0.343420303	2.76E-15	postive
GCSH	AL024507.3	0.446365453	7.44E-26	postive
GCSH	AL031055.1	0.309003769	1.60E-12	postive
GCSH	AL031123.2	0.371432525	8.39E-18	postive
GCSH	AL031847.1	0.32091707	1.93E-13	postive
GCSH	AL031963.3	0.310247981	1.29E-12	postive
GCSH	AL031985.3	0.355247344	2.56E-16	postive
GCSH	AL049597.2	0.328854572	4.49E-14	postive
GCSH	AL049794.1	0.391237663	9.78E-20	postive
GCSH	AL049796.1	0.345161361	1.95E-15	postive
GCSH	AL078644.2	0.342666824	3.20E-15	postive
GCSH	AL096828.3	0.353056562	4.01E-16	postive
GCSH	AL109917.1	0.369191833	1.36E-17	postive
GCSH	AL117332.1	0.41447151	3.54E-22	postive
GCSH	AL117336.2	0.349126921	8.87E-16	postive
GCSH	AL118506.1	0.375811642	3.22E-18	postive
GCSH	AL118516.1	0.365434882	3.05E-17	postive
GCSH	AL118558.3	0.327700361	5.57E-14	postive
GCSH	AL121658.1	0.361631635	6.81E-17	postive
GCSH	AL121832.2	0.340164658	5.21E-15	postive
GCSH	AL121944.2	0.494562258	3.34E-32	postive
GCSH	AL132639.2	0.331245486	2.87E-14	postive
GCSH	AL133215.2	0.39664062	2.75E-20	postive
GCSH	AL133353.1	0.328662406	4.66E-14	postive
GCSH	AL135925.1	0.350409321	6.85E-16	postive
GCSH	AL136040.1	0.331390347	2.79E-14	postive
GCSH	AL136162.1	0.342297997	3.44E-15	postive
GCSH	AL137186.2	0.355021327	2.68E-16	postive
GCSH	AL138724.2	0.42695842	1.43E-23	postive
GCSH	AL138762.2	0.410130203	1.05E-21	postive
GCSH	AL138921.1	0.305408576	2.96E-12	postive
GCSH	AL139035.1	0.343028687	2.98E-15	postive
GCSH	AL139288.1	0.313580166	7.17E-13	postive
GCSH	AL139289.1	0.314154752	6.48E-13	postive
GCSH	AL157400.2	0.349591827	8.08E-16	postive
GCSH	AL161421.1	0.359473143	1.07E-16	postive
GCSH	AL162231.2	0.305409823	2.96E-12	postive
GCSH	AL162258.2	0.304009161	3.76E-12	postive
GCSH	AL162377.1	0.307751199	1.98E-12	postive
GCSH	AL162595.1	0.365236567	3.18E-17	postive
GCSH	AL353194.1	0.341864525	3.74E-15	postive
GCSH	AL353708.1	0.374262981	4.53E-18	postive
GCSH	AL353708.3	0.322885291	1.35E-13	postive
GCSH	AL354696.2	0.321363046	1.78E-13	postive
GCSH	AL354892.2	0.425420519	2.14E-23	postive
GCSH	AL354920.1	0.349917869	7.56E-16	postive
GCSH	AL355001.2	0.377466425	2.23E-18	postive
GCSH	AL355574.1	0.330846482	3.09E-14	postive
GCSH	AL355802.3	0.358711794	1.25E-16	postive
GCSH	AL356299.3	0.300599337	6.69E-12	postive

GCSH	AL356488.3	0.309444935	1.48E-12	postive
GCSH	AL358115.1	0.307248467	2.16E-12	postive
GCSH	AL358472.2	0.300735954	6.54E-12	postive
GCSH	AL358472.3	0.317541407	3.55E-13	postive
GCSH	AL359198.1	0.352793068	4.23E-16	postive
GCSH	AL359643.3	0.389757166	1.38E-19	postive
GCSH	AL359715.3	0.404364936	4.31E-21	postive
GCSH	AL359921.2	0.317555542	3.54E-13	postive
GCSH	AL391121.1	0.309060578	1.58E-12	postive
GCSH	AL441992.2	0.387877312	2.13E-19	postive
GCSH	AL445524.1	0.317029853	3.89E-13	postive
GCSH	AL450326.1	0.304108223	3.70E-12	postive
GCSH	AL450998.2	0.330803752	3.12E-14	postive
GCSH	AL451085.3	0.391016286	1.03E-19	postive
GCSH	AL451165.2	0.366052964	2.67E-17	postive
GCSH	AL512598.1	0.344693062	2.14E-15	postive
GCSH	AL513550.1	0.303013252	4.45E-12	postive
GCSH	AL590617.2	0.377099206	2.42E-18	postive
GCSH	AL592166.1	0.403484341	5.34E-21	postive
GCSH	AL596094.1	0.365603316	2.94E-17	postive
GCSH	AL606760.1	0.301484846	5.76E-12	postive
GCSH	AL645568.1	0.34154507	3.98E-15	postive
GCSH	AL662797.2	0.319894714	2.33E-13	postive
GCSH	AL731569.1	0.307278608	2.15E-12	postive
GCSH	AL731577.2	0.328488101	4.81E-14	postive
GCSH	AL732314.8	0.312607031	8.51E-13	postive
GCSH	ALG1L13P	0.31372902	6.99E-13	postive
GCSH	ALMS1-IT1	0.317598932	3.51E-13	postive
GCSH	AMZ2P1	0.357641712	1.56E-16	postive
GCSH	AP000253.1	0.30391699	3.82E-12	postive
GCSH	AP000320.1	0.302536448	4.83E-12	postive
GCSH	AP000442.1	0.304290739	3.58E-12	postive
GCSH	AP000487.1	0.321871099	1.63E-13	postive
GCSH	AP000759.1	0.341511295	4.01E-15	postive
GCSH	AP000769.1	0.388659395	1.78E-19	postive
GCSH	AP000894.4	0.328392773	4.90E-14	postive
GCSH	AP001160.1	0.316461576	4.30E-13	postive
GCSH	AP001160.3	0.415894572	2.47E-22	postive
GCSH	AP001267.1	0.303006699	4.46E-12	postive
GCSH	AP001372.2	0.34854451	9.97E-16	postive
GCSH	AP001453.4	0.345600694	1.79E-15	postive
GCSH	AP001462.1	0.3193266	2.58E-13	postive
GCSH	AP001469.3	0.351400575	5.61E-16	postive
GCSH	AP001505.1	0.33779247	8.25E-15	postive
GCSH	AP001527.2	0.392735156	6.90E-20	postive
GCSH	AP001816.1	0.366791466	2.28E-17	postive
GCSH	AP002360.2	0.415939231	2.44E-22	postive
GCSH	AP002387.2	0.41205107	6.49E-22	postive
GCSH	AP002748.4	0.425541257	2.07E-23	postive
GCSH	AP003068.1	0.338218625	7.60E-15	postive

GCSH	AP003119.2	0.31026316	1.28E-12	postive
GCSH	AP003352.1	0.318582521	2.95E-13	postive
GCSH	AP004609.5	0.335542528	1.27E-14	postive
GCSH	AP005329.2	0.336376061	1.08E-14	postive
GCSH	AP005482.4	0.338377087	7.37E-15	postive
GCSH	ARF4-AS1	0.318009727	3.26E-13	postive
GCSH	ARHGAP5-AS1	0.344264904	2.33E-15	postive
GCSH	ARMCX5-GPRASP2	0.364822633	3.47E-17	postive
GCSH	ARRDC1-AS1	0.403384759	5.47E-21	postive
GCSH	ASB16-AS1	0.30017957	7.18E-12	postive
GCSH	ASH1L-AS1	0.361632129	6.80E-17	postive
GCSH	ATF4P4	0.336878025	9.83E-15	postive
GCSH	ATP2A1-AS1	0.329048429	4.33E-14	postive
GCSH	ATXN2-AS	0.389958732	1.32E-19	postive
GCSH	BAALC-AS1	0.317254655	3.74E-13	postive
GCSH	BCDIN3D-AS1	0.324818322	9.49E-14	postive
GCSH	BMS1P20	0.326643571	6.77E-14	postive
GCSH	BOLA3-AS1	0.309643444	1.43E-12	postive
GCSH	BVES-AS1	0.353122048	3.95E-16	postive
GCSH	BX537318.1	0.366161666	2.61E-17	postive
GCSH	C10orf25	0.314531279	6.06E-13	postive
GCSH	C18orf65	0.313736239	6.98E-13	postive
GCSH	C22orf46	0.344724181	2.13E-15	postive
GCSH	C2CD4D-AS1	0.309021493	1.59E-12	postive
GCSH	C6orf99	0.362970403	5.14E-17	postive
GCSH	CA5BP1	0.437495389	8.59E-25	postive
GCSH	CASC8	0.31791884	3.32E-13	postive
GCSH	CATSPER2P1	0.440044999	4.28E-25	postive
GCSH	CCNT2-AS1	0.345699203	1.76E-15	postive
GCSH	CD27-AS1	0.400493489	1.10E-20	postive
GCSH	CDC37L1-DT	0.318950169	2.76E-13	postive
GCSH	CENPBD1P1	0.375760902	3.26E-18	postive
GCSH	CERNA3	0.307583464	2.04E-12	postive
GCSH	CFL1P1	0.308631813	1.70E-12	postive
GCSH	CHKB-DT	0.341172516	4.28E-15	postive
GCSH	CIDECP1	0.483652179	1.12E-30	postive
GCSH	CNNM3-DT	0.32317301	1.28E-13	postive
GCSH	COA6-AS1	0.357636865	1.56E-16	postive
GCSH	CR381653.1	0.319380327	2.55E-13	postive
GCSH	CRTC3-AS1	0.374371386	4.42E-18	postive
GCSH	CRYBB2P1	0.302765282	4.64E-12	postive
GCSH	CSE1L-AS1	0.315680085	4.95E-13	postive
GCSH	CTA-989H11.1	0.368149753	1.71E-17	postive
GCSH	CTBP1-DT	0.444059248	1.42E-25	postive
GCSH	CYTOR	0.380203538	1.21E-18	postive
GCSH	DANCR	0.411701366	7.08E-22	postive
GCSH	DARS-AS1	0.305387371	2.97E-12	postive
GCSH	DBIL5P	0.395649845	3.48E-20	postive
GCSH	DCST1-AS1	0.301887022	5.39E-12	postive
GCSH	DCXR-DT	0.348553198	9.95E-16	postive

GCSH	DDX39B-AS1	0.326098033	7.49E-14	postive
GCSH	DEPDC1-AS1	0.300231381	7.11E-12	postive
GCSH	DGCR11	0.325170305	8.89E-14	postive
GCSH	DHCR24-DT	0.378011243	1.98E-18	postive
GCSH	DHRS4-AS1	0.387486111	2.33E-19	postive
GCSH	DLEU1	0.318082019	3.22E-13	postive
GCSH	DLGAP1-AS1	0.317560343	3.54E-13	postive
GCSH	DM1-AS	0.362978737	5.13E-17	postive
GCSH	DSTNP2	0.358320143	1.36E-16	postive
GCSH	DUXAP9	0.351046952	6.02E-16	postive
GCSH	DYRK3-AS1	0.418055039	1.43E-22	postive
GCSH	EBLN3P	0.326963792	6.38E-14	postive
GCSH	EIF1B-AS1	0.332215532	2.39E-14	postive
GCSH	EIF2AK3-DT	0.369824211	1.19E-17	postive
GCSH	EIF3J-DT	0.381855512	8.37E-19	postive
GCSH	ELF3-AS1	0.323880735	1.13E-13	postive
GCSH	ELOA-AS1	0.319491751	2.50E-13	postive
GCSH	EMC1-AS1	0.338098706	7.77E-15	postive
GCSH	EMC3-AS1	0.319729509	2.40E-13	postive
GCSH	EMSLR	0.329672574	3.86E-14	postive
GCSH	ENTPD3-AS1	0.347835449	1.15E-15	postive
GCSH	EPB41L4A-AS1	0.312021699	9.43E-13	postive
GCSH	EXOSC10-AS1	0.334888863	1.44E-14	postive
GCSH	FAM21FP	0.311738695	9.91E-13	postive
GCSH	FAM83C-AS1	0.341908861	3.71E-15	postive
GCSH	FAM86B3P	0.336473238	1.06E-14	postive
GCSH	FAM86C2P	0.468828464	1.09E-28	postive
GCSH	FAM86DP	0.338051235	7.85E-15	postive
GCSH	FAM86EP	0.523006834	1.88E-36	postive
GCSH	FAM86JP	0.411427881	7.58E-22	postive
GCSH	FGD5-AS1	0.410565333	9.40E-22	postive
GCSH	FIRRE	0.325365213	8.58E-14	postive
GCSH	FLVCR1-DT	0.335927005	1.18E-14	postive
GCSH	FO393418.1	0.311466548	1.04E-12	postive
GCSH	FRG1HP	0.30523098	3.05E-12	postive
GCSH	FZD4-DT	0.352928429	4.11E-16	postive
GCSH	GCC2-AS1	0.43947171	5.01E-25	postive
GCSH	GNRHR2	0.350874552	6.24E-16	postive
GCSH	GPR176-DT	0.306178549	2.60E-12	postive
GCSH	GPRC5D-AS1	0.308234927	1.82E-12	postive
GCSH	GSEC	0.412354186	6.02E-22	postive
GCSH	GTF2IP1	0.328454242	4.84E-14	postive
GCSH	GTSE1-DT	0.388907731	1.68E-19	postive
GCSH	H2BC19P	0.339495851	5.93E-15	postive
GCSH	HCG18	0.424975494	2.40E-23	postive
GCSH	HCG25	0.352809905	4.21E-16	postive
GCSH	HDAC11-AS1	0.303096166	4.39E-12	postive
GCSH	HEIH	0.338123424	7.74E-15	postive
GCSH	HM13-IT1	0.318192111	3.16E-13	postive
GCSH	HMGB1P5	0.397084983	2.48E-20	postive

GCSH	IDH1-AS1	0.413454316	4.57E-22	postive
GCSH	ILF3-DT	0.346751145	1.43E-15	postive
GCSH	INGX	0.300282516	7.05E-12	postive
GCSH	IQCH-AS1	0.370784281	9.66E-18	postive
GCSH	IRAIN	0.3227255	1.39E-13	postive
GCSH	ITFG2-AS1	0.381859308	8.37E-19	postive
GCSH	JPX	0.310202764	1.30E-12	postive
GCSH	KCNIP2-AS1	0.312047093	9.39E-13	postive
GCSH	KCTD21-AS1	0.347696127	1.18E-15	postive
GCSH	KMT2E-AS1	0.373394151	5.47E-18	postive
GCSH	KRT8P12	0.33523408	1.35E-14	postive
GCSH	KTN1-AS1	0.480715054	2.83E-30	postive
GCSH	LGALS8-AS1	0.306537292	2.44E-12	postive
GCSH	LINC00205	0.306166711	2.60E-12	postive
GCSH	LINC00235	0.319783884	2.37E-13	postive
GCSH	LINC002481	0.317831033	3.37E-13	postive
GCSH	LINC00339	0.420346908	7.95E-23	postive
GCSH	LINC00449	0.346431658	1.52E-15	postive
GCSH	LINC00467	0.349555979	8.14E-16	postive
GCSH	LINC00471	0.365727342	2.86E-17	postive
GCSH	LINC00630	0.304154018	3.67E-12	postive
GCSH	LINC00665	0.407972743	1.78E-21	postive
GCSH	LINC00680	0.317770893	3.41E-13	postive
GCSH	LINC00847	0.308402834	1.77E-12	postive
GCSH	LINC00857	0.313044738	7.88E-13	postive
GCSH	LINC00863	0.346039362	1.64E-15	postive
GCSH	LINC00887	0.332624908	2.21E-14	postive
GCSH	LINC00888	0.304704258	3.34E-12	postive
GCSH	LINC00909	0.396092705	3.13E-20	postive
GCSH	LINC00910	0.375815896	3.22E-18	postive
GCSH	LINC00963	0.352587575	4.41E-16	postive
GCSH	LINC01003	0.321425053	1.76E-13	postive
GCSH	LINC01011	0.329223896	4.19E-14	postive
GCSH	LINC01023	0.319479971	2.51E-13	postive
GCSH	LINC01128	0.302020088	5.27E-12	postive
GCSH	LINC01137	0.315522123	5.09E-13	postive
GCSH	LINC01184	0.332090114	2.45E-14	postive
GCSH	LINC01220	0.32266228	1.41E-13	postive
GCSH	LINC01270	0.332478784	2.27E-14	postive
GCSH	LINC01271	0.333386688	1.91E-14	postive
GCSH	LINC01273	0.337160035	9.31E-15	postive
GCSH	LINC01278	0.329590405	3.92E-14	postive
GCSH	LINC01301	0.330618129	3.23E-14	postive
GCSH	LINC01311	0.306805547	2.33E-12	postive
GCSH	LINC01315	0.329673105	3.85E-14	postive
GCSH	LINC01521	0.346670292	1.45E-15	postive
GCSH	LINC01560	0.416538796	2.10E-22	postive
GCSH	LINC01572	0.338426259	7.30E-15	postive
GCSH	LINC01703	0.348969986	9.15E-16	postive
GCSH	LINC01719	0.397034026	2.51E-20	postive

GCSH	LINC01806	0.388603706	1.80E-19	postive
GCSH	LINC01816	0.328949277	4.41E-14	postive
GCSH	LINC01881	0.349421563	8.36E-16	postive
GCSH	LINC02298	0.301464299	5.78E-12	postive
GCSH	LINC02482	0.366648825	2.35E-17	postive
GCSH	LINC02485	0.315933669	4.73E-13	postive
GCSH	LINC02585	0.327429387	5.86E-14	postive
GCSH	LINC02637	0.312586086	8.54E-13	postive
GCSH	LINC02693	0.315001908	5.58E-13	postive
GCSH	LINC02709	0.375458439	3.48E-18	postive
GCSH	LINC02732	0.341068767	4.37E-15	postive
GCSH	LMCD1-AS1	0.32969029	3.84E-14	postive
GCSH	LOH12CR2	0.389444834	1.48E-19	postive
GCSH	LRRC37BP1	0.337019299	9.57E-15	postive
GCSH	LSP1P4	0.312637704	8.47E-13	postive
GCSH	MAFG-DT	0.400698268	1.05E-20	postive
GCSH	MAN1B1-DT	0.359956704	9.66E-17	postive
GCSH	MAP3K14-AS1	0.328519369	4.78E-14	postive
GCSH	MAP3K2-DT	0.400435665	1.11E-20	postive
GCSH	MAP4K3-DT	0.384163126	4.97E-19	postive
GCSH	MAPKAPK5-AS1	0.54709401	2.23E-40	postive
GCSH	MCM3AP-AS1	0.314313884	6.30E-13	postive
GCSH	MDC1-AS1	0.321762148	1.66E-13	postive
GCSH	MFF-DT	0.313956285	6.71E-13	postive
GCSH	MINCR	0.375458356	3.48E-18	postive
GCSH	MIR193BHG	0.386236789	3.10E-19	postive
GCSH	MIR4435-2HG	0.438663968	6.25E-25	postive
GCSH	MIR762HG	0.421944482	5.27E-23	postive
GCSH	MRPL20-AS1	0.407779908	1.87E-21	postive
GCSH	MRPL45P2	0.371166714	8.89E-18	postive
GCSH	MSL3P1	0.37522629	3.66E-18	postive
GCSH	MYLK-AS1	0.371387259	8.48E-18	postive
GCSH	NACA4P	0.310768344	1.17E-12	postive
GCSH	NAPA-AS1	0.391341415	9.55E-20	postive
GCSH	NBR2	0.385396961	3.75E-19	postive
GCSH	NCK1-DT	0.429472179	7.38E-24	postive
GCSH	NDUFB2-AS1	0.309930252	1.36E-12	postive
GCSH	NIFK-AS1	0.495384418	2.55E-32	postive
GCSH	NINJ2-AS1	0.30498407	3.19E-12	postive
GCSH	NOP14-AS1	0.43745496	8.68E-25	postive
GCSH	NORAD	0.354297266	3.11E-16	postive
GCSH	NRAV	0.454928096	6.55E-27	postive
GCSH	NRSN2-AS1	0.458330328	2.45E-27	postive
GCSH	NUDT16P1	0.441796595	2.65E-25	postive
GCSH	NUP50-DT	0.343744091	2.59E-15	postive
GCSH	NUTM2A-AS1	0.470599101	6.40E-29	postive
GCSH	OGFRP1	0.396225723	3.04E-20	postive
GCSH	OIP5-AS1	0.402501222	6.77E-21	postive
GCSH	OLMALINC	0.336825091	9.93E-15	postive
GCSH	OSER1-DT	0.437486952	8.61E-25	postive

GCSH	OTUD6B-AS1	0.41510517	3.02E-22	postive
GCSH	PARD6G-AS1	0.33466499	1.50E-14	postive
GCSH	PAXIP1-AS1	0.343116973	2.93E-15	postive
GCSH	PCAT6	0.404449871	4.22E-21	postive
GCSH	PCBP1-AS1	0.34716185	1.31E-15	postive
GCSH	PDCD4-AS1	0.318510856	2.98E-13	postive
GCSH	PDIA3P1	0.337286516	9.09E-15	postive
GCSH	PDPK2P	0.305067052	3.14E-12	postive
GCSH	PINK1-AS	0.317722853	3.44E-13	postive
GCSH	PIPSL	0.306745812	2.36E-12	postive
GCSH	PITPNA-AS1	0.461637024	9.30E-28	postive
GCSH	PLEKHA8P1	0.358497938	1.31E-16	postive
GCSH	PMS2CL	0.306875157	2.30E-12	postive
GCSH	PMS2P4	0.338255261	7.54E-15	postive
GCSH	POLH-AS1	0.317085052	3.85E-13	postive
GCSH	PPP1R14B-AS1	0.340295863	5.08E-15	postive
GCSH	PPT2-EGFL8	0.351133793	5.92E-16	postive
GCSH	PRELID1P1	0.343062548	2.96E-15	postive
GCSH	PRKAR2A-AS1	0.336655107	1.03E-14	postive
GCSH	PRMT5-AS1	0.304211829	3.63E-12	postive
GCSH	PRORSD1P	0.336156294	1.13E-14	postive
GCSH	PRRT3-AS1	0.429794956	6.78E-24	postive
GCSH	PVT1	0.301560801	5.69E-12	postive
GCSH	PXN-AS1	0.490964472	1.08E-31	postive
GCSH	RAB30-DT	0.40069881	1.05E-20	postive
GCSH	RAD21-AS1	0.359480957	1.07E-16	postive
GCSH	RALY-AS1	0.379147561	1.54E-18	postive
GCSH	RARA-AS1	0.358908584	1.20E-16	postive
GCSH	RASAL2-AS1	0.445542325	9.37E-26	postive
GCSH	RASSF1-AS1	0.328307044	4.97E-14	postive
GCSH	RBM15-AS1	0.383921558	5.25E-19	postive
GCSH	RHPN1-AS1	0.366075039	2.66E-17	postive
GCSH	RN7SL832P	0.309516634	1.46E-12	postive
GCSH	RNASEH1-AS1	0.517962944	1.14E-35	postive
GCSH	RNF139-AS1	0.321993381	1.59E-13	postive
GCSH	RP11-101E13.5	0.350244269	7.08E-16	postive
GCSH	RP11-10C24.1	0.317140399	3.81E-13	postive
GCSH	RP11-199F11.2	0.317086062	3.85E-13	postive
GCSH	RP11-435O5.2	0.369624548	1.24E-17	postive
GCSH	RP11-61L19.3	0.421597536	5.77E-23	postive
GCSH	RP11-706O15.1	0.354785709	2.81E-16	postive
GCSH	RP11-89K10.1	0.305397528	2.97E-12	postive
GCSH	RP4-545L17.11	0.372134664	7.21E-18	postive
GCSH	RPARP-AS1	0.324683137	9.72E-14	postive
GCSH	RPL13P5	0.352166737	4.80E-16	postive
GCSH	RPL23AP53	0.304839927	3.26E-12	postive
GCSH	RPL23AP7	0.49567593	2.31E-32	postive
GCSH	RPL23AP82	0.463051683	6.13E-28	postive
GCSH	RRN3P3	0.337399561	8.90E-15	postive
GCSH	RRP7BP	0.38711386	2.53E-19	postive

GCSH	SBDSP1	0.419311172	1.04E-22	postive
GCSH	SBF2-AS1	0.362592114	5.56E-17	postive
GCSH	SBNO1-AS1	0.337028928	9.55E-15	postive
GCSH	SCAMP1-AS1	0.372278627	6.98E-18	postive
GCSH	SDCBP2-AS1	0.300899251	6.36E-12	postive
GCSH	SEC24B-AS1	0.320074292	2.25E-13	postive
GCSH	SEPSECS-AS1	0.328180699	5.09E-14	postive
GCSH	SEPTIN4-AS1	0.390196033	1.25E-19	postive
GCSH	SLC12A9-AS1	0.301198649	6.05E-12	postive
GCSH	SLC2A1-AS1	0.366986178	2.19E-17	postive
GCSH	SMG7-AS1	0.305196994	3.07E-12	postive
GCSH	SMPD5	0.304974189	3.19E-12	postive
GCSH	SNAI3-AS1	0.335587735	1.26E-14	postive
GCSH	SNHG1	0.402676026	6.49E-21	postive
GCSH	SNHG10	0.320540075	2.07E-13	postive
GCSH	SNHG11	0.435669255	1.41E-24	postive
GCSH	SNHG16	0.441540136	2.84E-25	postive
GCSH	SNHG17	0.443928643	1.47E-25	postive
GCSH	SNHG19	0.304319743	3.57E-12	postive
GCSH	SNHG20	0.349492805	8.24E-16	postive
GCSH	SNHG21	0.415454	2.76E-22	postive
GCSH	SNHG29	0.306554061	2.43E-12	postive
GCSH	SNHG3	0.300196832	7.15E-12	postive
GCSH	SNHG30	0.378856116	1.64E-18	postive
GCSH	SNHG32	0.353043112	4.02E-16	postive
GCSH	SNHG4	0.36743949	1.99E-17	postive
GCSH	SNHG5	0.304514443	3.45E-12	postive
GCSH	SNHG6	0.406002376	2.89E-21	postive
GCSH	SNHG8	0.401318836	9.01E-21	postive
GCSH	SORD2P	0.349100763	8.91E-16	postive
GCSH	SP2-AS1	0.392948556	6.56E-20	postive
GCSH	SREBF2-AS1	0.343123135	2.92E-15	postive
GCSH	SRP14-AS1	0.302249106	5.07E-12	postive
GCSH	ST20-AS1	0.320446355	2.10E-13	postive
GCSH	STAG3L2	0.337104902	9.41E-15	postive
GCSH	STAG3L4	0.328003917	5.26E-14	postive
GCSH	STARD7-AS1	0.41914497	1.08E-22	postive
GCSH	STK4-AS1	0.480153861	3.37E-30	postive
GCSH	STX18-AS1	0.369461125	1.29E-17	postive
GCSH	SUCLA2-AS1	0.336289255	1.10E-14	postive
GCSH	SUCLG2-AS1	0.325019637	9.14E-14	postive
GCSH	SUMO2P17	0.362333037	5.87E-17	postive
GCSH	SVIL-AS1	0.364319327	3.86E-17	postive
GCSH	TAF1A-AS1	0.365991019	2.71E-17	postive
GCSH	TDRKH-AS1	0.323208667	1.27E-13	postive
GCSH	THAP7-AS1	0.306759314	2.35E-12	postive
GCSH	THAP9-AS1	0.331440732	2.77E-14	postive
GCSH	TIPARP-AS1	0.384727675	4.37E-19	postive
GCSH	TMC3-AS1	0.35799795	1.45E-16	postive
GCSH	TMCC1-AS1	0.359679589	1.02E-16	postive

GCSH	TMED2-DT	0.336547056	1.05E-14	postive
GCSH	TMEM202-AS1	0.379383778	1.46E-18	postive
GCSH	TMEM44-AS1	0.396629209	2.76E-20	postive
GCSH	TMEM99	0.436355418	1.17E-24	postive
GCSH	TMPO-AS1	0.31054712	1.22E-12	postive
GCSH	TOB1-AS1	0.496732141	1.63E-32	postive
GCSH	TOLLIP-AS1	0.300444973	6.86E-12	postive
GCSH	TRAM2-AS1	0.455863952	5.00E-27	postive
GCSH	TRAPPC12-AS1	0.356164555	2.12E-16	postive
GCSH	TRIM52-AS1	0.377431886	2.25E-18	postive
GCSH	TSTD3	0.31154619	1.03E-12	postive
GCSH	TTC28-AS1	0.314225542	6.40E-13	postive
GCSH	TUG1	0.359978379	9.62E-17	postive
GCSH	TYMSOS	0.344586449	2.19E-15	postive
GCSH	U47924.2	0.353579623	3.60E-16	postive
GCSH	U91328.1	0.334550742	1.53E-14	postive
GCSH	UBA6-AS1	0.447979951	4.73E-26	postive
GCSH	UBAC2-AS1	0.372994883	5.97E-18	postive
GCSH	UBE2D3-AS1	0.469757427	8.25E-29	postive
GCSH	UBL7-AS1	0.318218024	3.14E-13	postive
GCSH	UBR5-AS1	0.353162458	3.92E-16	postive
GCSH	UGDH-AS1	0.385436238	3.72E-19	postive
GCSH	URAHP	0.337879717	8.11E-15	postive
GCSH	URB1-AS1	0.449699178	2.91E-26	postive
GCSH	USP27X-AS1	0.338774979	6.82E-15	postive
GCSH	USP46-AS1	0.356815399	1.85E-16	postive
GCSH	UXT-AS1	0.418274137	1.35E-22	postive
GCSH	VPS13B-DT	0.346632345	1.46E-15	postive
GCSH	VPS33B-DT	0.409783946	1.14E-21	postive
GCSH	VPS9D1-AS1	0.448871747	3.68E-26	postive
GCSH	WAC-AS1	0.403047549	5.93E-21	postive
GCSH	WARS2-AS1	0.306404918	2.50E-12	postive
GCSH	WASH3P	0.327371226	5.92E-14	postive
GCSH	WWTR1-AS1	0.339132724	6.37E-15	postive
GCSH	YTHDF3-AS1	0.402381266	6.97E-21	postive
GCSH	Z83851.4	0.393033741	6.43E-20	postive
GCSH	Z84485.1	0.301515255	5.73E-12	postive
GCSH	Z95114.1	0.321935887	1.61E-13	postive
GCSH	Z95115.1	0.347516231	1.22E-15	postive
GCSH	Z97634.1	0.338800373	6.79E-15	postive
GCSH	ZBTB11-AS1	0.408534329	1.55E-21	postive
GCSH	ZBTB44-DT	0.324109159	1.08E-13	postive
GCSH	ZFAS1	0.354291121	3.11E-16	postive
GCSH	ZNF213-AS1	0.307778964	1.97E-12	postive
GCSH	ZNF252P	0.380370433	1.17E-18	postive
GCSH	ZNF271P	0.325438682	8.46E-14	postive
GCSH	ZNF426-DT	0.301125385	6.12E-12	postive
GCSH	ZNF503-AS1	0.327068669	6.26E-14	postive
GCSH	ZNF503-AS2	0.440976931	3.32E-25	postive
GCSH	ZNF561-AS1	0.344522936	2.22E-15	postive

GCSH	ZNF674-AS1	0.301726533	5.53E-12	postive
GCSH	ZNF687-AS1	0.473501305	2.64E-29	postive
GCSH	ZNRD2-AS1	0.378464141	1.79E-18	postive
GCSH	ZSCAN12P1	0.32072464	2.00E-13	postive
GCSH	ZSCAN16-AS1	0.34915982	8.81E-16	postive
GLS	AC000123.1	0.365988863	2.71E-17	postive
GLS	AC002519.1	0.363906504	4.21E-17	postive
GLS	AC004066.2	0.312528768	8.63E-13	postive
GLS	AC004156.1	0.339401776	6.04E-15	postive
GLS	AC004448.2	0.361552054	6.92E-17	postive
GLS	AC004951.4	0.31197151	9.52E-13	postive
GLS	AC005162.2	0.320535338	2.07E-13	postive
GLS	AC005332.3	0.309123275	1.56E-12	postive
GLS	AC005332.6	0.312479154	8.71E-13	postive
GLS	AC005540.1	0.54881891	1.14E-40	postive
GLS	AC005837.4	0.386362032	3.01E-19	postive
GLS	AC005899.2	0.350193757	7.15E-16	postive
GLS	AC005899.8	0.370906632	9.41E-18	postive
GLS	AC006017.1	0.314771088	5.81E-13	postive
GLS	AC006042.3	0.404222471	4.46E-21	postive
GLS	AC006160.1	0.319444186	2.52E-13	postive
GLS	AC006460.1	0.368553725	1.56E-17	postive
GLS	AC007216.2	0.340322762	5.05E-15	postive
GLS	AC007292.2	0.323727838	1.16E-13	postive
GLS	AC007390.2	0.302072565	5.22E-12	postive
GLS	AC007552.2	0.302694902	4.70E-12	postive
GLS	AC007879.3	0.318546627	2.96E-13	postive
GLS	AC007879.4	0.32836309	4.92E-14	postive
GLS	AC008115.3	0.324362097	1.03E-13	postive
GLS	AC008467.1	0.303779345	3.91E-12	postive
GLS	AC008743.1	0.361700099	6.71E-17	postive
GLS	AC008770.3	0.310542827	1.22E-12	postive
GLS	AC008875.1	0.330585916	3.25E-14	postive
GLS	AC008906.2	0.301078575	6.17E-12	postive
GLS	AC009226.1	0.386949268	2.63E-19	postive
GLS	AC009229.2	0.320699653	2.01E-13	postive
GLS	AC009404.1	0.331371433	2.80E-14	postive
GLS	AC010401.1	0.312971348	7.98E-13	postive
GLS	AC010615.2	0.334626007	1.51E-14	postive
GLS	AC011726.3	0.311657131	1.01E-12	postive
GLS	AC012020.1	0.44822685	4.41E-26	postive
GLS	AC012254.1	0.306250735	2.56E-12	postive
GLS	AC012254.3	0.314806191	5.78E-13	postive
GLS	AC012368.1	0.325964528	7.68E-14	postive
GLS	AC012462.1	0.306398078	2.50E-12	postive
GLS	AC012593.2	0.3251631	8.90E-14	postive
GLS	AC012668.3	0.300402536	6.91E-12	postive
GLS	AC015813.1	0.339363718	6.09E-15	postive
GLS	AC015849.3	0.303463794	4.13E-12	postive
GLS	AC017060.1	0.300815062	6.45E-12	postive

GLS	AC019080.5	0.365613151	2.93E-17	postive
GLS	AC019117.2	0.303543151	4.07E-12	postive
GLS	AC019133.1	0.312472982	8.71E-13	postive
GLS	AC020594.1	0.318909743	2.78E-13	postive
GLS	AC022211.2	0.354677267	2.88E-16	postive
GLS	AC022211.4	0.327097629	6.23E-14	postive
GLS	AC022400.1	0.353097315	3.97E-16	postive
GLS	AC022400.6	0.320170814	2.21E-13	postive
GLS	AC023855.2	0.325578603	8.25E-14	postive
GLS	AC024361.3	0.303861882	3.86E-12	postive
GLS	AC025171.3	0.308231176	1.82E-12	postive
GLS	AC027796.1	0.310700918	1.19E-12	postive
GLS	AC034229.1	0.301062749	6.19E-12	postive
GLS	AC037487.4	0.329371281	4.08E-14	postive
GLS	AC055811.1	0.315315013	5.28E-13	postive
GLS	AC063976.1	0.367449584	1.98E-17	postive
GLS	AC067852.3	0.397278261	2.37E-20	postive
GLS	AC067945.3	0.301746444	5.51E-12	postive
GLS	AC068418.2	0.309249713	1.53E-12	postive
GLS	AC068594.1	0.318004627	3.27E-13	postive
GLS	AC069503.1	0.340973793	4.45E-15	postive
GLS	AC073389.3	0.317636493	3.49E-13	postive
GLS	AC073517.1	0.378726168	1.69E-18	postive
GLS	AC073592.1	0.334096945	1.67E-14	postive
GLS	AC079921.1	0.320988453	1.91E-13	postive
GLS	AC087289.1	0.452388195	1.36E-26	postive
GLS	AC087289.2	0.301139041	6.11E-12	postive
GLS	AC087318.1	0.314045162	6.61E-13	postive
GLS	AC091173.1	0.305863254	2.74E-12	postive
GLS	AC091212.1	0.303275784	4.26E-12	postive
GLS	AC092656.1	0.339112176	6.39E-15	postive
GLS	AC104109.4	0.344613817	2.18E-15	postive
GLS	AC107294.2	0.31699279	3.92E-13	postive
GLS	AC108010.1	0.347698736	1.18E-15	postive
GLS	AC114490.1	0.303765238	3.92E-12	postive
GLS	AC114763.1	0.304417458	3.51E-12	postive
GLS	AC119044.1	0.323842265	1.13E-13	postive
GLS	AC124319.1	0.306484397	2.46E-12	postive
GLS	AC130650.2	0.327327181	5.97E-14	postive
GLS	AC132872.3	0.325735862	8.01E-14	postive
GLS	AC138207.1	0.300899221	6.36E-12	postive
GLS	AC138207.4	0.310486938	1.23E-12	postive
GLS	AC141586.1	0.309862236	1.38E-12	postive
GLS	AC145285.3	0.327591039	5.68E-14	postive
GLS	AC244093.5	0.398480746	1.78E-20	postive
GLS	AL008726.1	0.334962358	1.42E-14	postive
GLS	AL031429.2	0.322198378	1.53E-13	postive
GLS	AL035252.3	0.397931331	2.03E-20	postive
GLS	AL035530.2	0.318669661	2.90E-13	postive
GLS	AL109946.1	0.303670174	3.98E-12	postive

GLS	AL121845.4	0.304590568	3.41E-12	postive
GLS	AL121987.2	0.358958646	1.19E-16	postive
GLS	AL132640.3	0.318489024	3.00E-13	postive
GLS	AL133230.2	0.332870293	2.11E-14	postive
GLS	AL133304.1	0.307293656	2.14E-12	postive
GLS	AL133330.1	0.34782092	1.15E-15	postive
GLS	AL133387.2	0.319257864	2.61E-13	postive
GLS	AL137003.1	0.358773532	1.24E-16	postive
GLS	AL139010.1	0.311933428	9.58E-13	postive
GLS	AL139407.1	0.325174774	8.88E-14	postive
GLS	AL157394.1	0.313767549	6.94E-13	postive
GLS	AL157838.1	0.365048371	3.31E-17	postive
GLS	AL157932.1	0.3110318	1.12E-12	postive
GLS	AL158166.1	0.395160946	3.90E-20	postive
GLS	AL158166.2	0.398973591	1.58E-20	postive
GLS	AL162595.1	0.312422021	8.79E-13	postive
GLS	AL162727.2	0.31196641	9.52E-13	postive
GLS	AL353801.2	0.333032551	2.05E-14	postive
GLS	AL354710.2	0.356977206	1.79E-16	postive
GLS	AL354989.1	0.482979409	1.39E-30	postive
GLS	AL357874.2	0.334875271	1.44E-14	postive
GLS	AL358334.3	0.307597182	2.03E-12	postive
GLS	AL390195.3	0.301637092	5.62E-12	postive
GLS	AL589843.2	0.332605968	2.22E-14	postive
GLS	AL590385.2	0.332781688	2.15E-14	postive
GLS	AL592435.1	0.30229847	5.02E-12	postive
GLS	AL645608.8	0.355849882	2.26E-16	postive
GLS	AP001029.2	0.311934729	9.58E-13	postive
GLS	AP001432.1	0.390909058	1.06E-19	postive
GLS	AP001469.2	0.359203476	1.13E-16	postive
GLS	AP001574.1	0.303739932	3.94E-12	postive
GLS	AP001767.3	0.354959751	2.72E-16	postive
GLS	AP003419.2	0.323897311	1.12E-13	postive
GLS	AP003472.2	0.352898863	4.14E-16	postive
GLS	AP003498.1	0.328702316	4.62E-14	postive
GLS	ATP1B3-AS1	0.30362215	4.02E-12	postive
GLS	BET1-AS1	0.424076573	3.04E-23	postive
GLS	BMS1P4	0.322122836	1.55E-13	postive
GLS	CACNA1G-AS1	0.343283758	2.83E-15	postive
GLS	CACTIN-AS1	0.384053904	5.09E-19	postive
GLS	CARD11-AS1	0.369531905	1.27E-17	postive
GLS	CAVIN2-AS1	0.308491449	1.74E-12	postive
GLS	CCDC18-AS1	0.321815226	1.64E-13	postive
GLS	CD44-AS1	0.350162901	7.20E-16	postive
GLS	CR936218.1	0.347943469	1.12E-15	postive
GLS	CTD-2574D22.4	0.308205004	1.83E-12	postive
GLS	CYP4A22-AS1	0.326176766	7.38E-14	postive
GLS	DLEU2	0.365453042	3.04E-17	postive
GLS	DPP4-DT	0.57143545	1.11E-44	postive
GLS	ECE1-AS1	0.340284658	5.09E-15	postive

GLS	ENO1-IT1	0.336582109	1.04E-14	postive
GLS	ERVK13-1	0.301569265	5.68E-12	postive
GLS	FAM13A-AS1	0.307315513	2.14E-12	postive
GLS	FAM185BP	0.311274481	1.08E-12	postive
GLS	FER1L4	0.336557378	1.05E-14	postive
GLS	FP325335.1	0.395370517	3.72E-20	postive
GLS	GARS-DT	0.346444719	1.52E-15	postive
GLS	GLCCI1-DT	0.413726177	4.27E-22	postive
GLS	GLUD1P3	0.36045051	8.72E-17	postive
GLS	GNG12-AS1	0.32187863	1.62E-13	postive
GLS	GPRACR	0.398240838	1.88E-20	postive
GLS	GTF3C2-AS1	0.359722271	1.01E-16	postive
GLS	HMGA2-AS1	0.31373336	6.98E-13	postive
GLS	HNRNPA1P30	0.314511233	6.09E-13	postive
GLS	LAMTOR5-AS1	0.303731025	3.94E-12	postive
GLS	LINC00398	0.355586159	2.39E-16	postive
GLS	LINC00486	0.303473224	4.12E-12	postive
GLS	LINC00630	0.302402164	4.94E-12	postive
GLS	LINC00674	0.332549901	2.24E-14	postive
GLS	LINC00880	0.318655091	2.91E-13	postive
GLS	LINC00910	0.320842898	1.96E-13	postive
GLS	LINC01097	0.355696462	2.33E-16	postive
GLS	LINC01185	0.421525166	5.88E-23	postive
GLS	LINC01303	0.339444554	5.99E-15	postive
GLS	LINC01460	0.350509942	6.71E-16	postive
GLS	LINC01563	0.327085778	6.24E-14	postive
GLS	LINC01852	0.330541665	3.28E-14	postive
GLS	LINC01946	0.33717771	9.28E-15	postive
GLS	LINC01948	0.307845328	1.95E-12	postive
GLS	LINC02003	0.398997969	1.57E-20	postive
GLS	LINC02029	0.394718899	4.33E-20	postive
GLS	LINC02080	0.314563062	6.03E-13	postive
GLS	LINC02094	0.320915091	1.93E-13	postive
GLS	LINC02361	0.301676661	5.58E-12	postive
GLS	LINC02656	0.346659836	1.45E-15	postive
GLS	LINC02768	0.331305201	2.84E-14	postive
GLS	LRRC37A16P	0.336294265	1.10E-14	postive
GLS	LUCAT1	0.315697627	4.93E-13	postive
GLS	MAP2K4P1	0.304153341	3.67E-12	postive
GLS	MCM3AP-AS1	0.333465594	1.89E-14	postive
GLS	OGFR-AS1	0.313672177	7.06E-13	postive
GLS	P4HA2-AS1	0.327028422	6.31E-14	postive
GLS	PAXBP1-AS1	0.317738614	3.43E-13	postive
GLS	PDZK1P1	0.35303537	4.02E-16	postive
GLS	PHKA2-AS1	0.348987478	9.12E-16	postive
GLS	PICART1	0.33106792	2.97E-14	postive
GLS	PMS2P4	0.341918328	3.70E-15	postive
GLS	PPP1R12A-AS1	0.35739293	1.65E-16	postive
GLS	PRR7-AS1	0.370639245	9.97E-18	postive
GLS	PRSS42P	0.302634321	4.75E-12	postive

GLS	RBFADN	0.31882426	2.82E-13	postive
GLS	RBM5-AS1	0.308306635	1.80E-12	postive
GLS	RDM1P5	0.345610996	1.79E-15	postive
GLS	RFPL3S	0.312126189	9.26E-13	postive
GLS	RMDN2-AS1	0.320384547	2.13E-13	postive
GLS	RNF216P1	0.320003028	2.28E-13	postive
GLS	RP11-159G9.5	0.338320798	7.45E-15	postive
GLS	RP1-116K23.1	0.300613487	6.67E-12	postive
GLS	RP11-217L21.1	0.366862591	2.25E-17	postive
GLS	RP11-345J18.2	0.401922917	7.79E-21	postive
GLS	RP11-465K1.2	0.347526591	1.22E-15	postive
GLS	RP4-765H13.1	0.307917775	1.93E-12	postive
GLS	RP4-809F18.2	0.336503607	1.06E-14	postive
GLS	RUNDC3A-AS1	0.391963227	8.26E-20	postive
GLS	SAP30L-AS1	0.30092182	6.34E-12	postive
GLS	SEPTIN9-DT	0.323919198	1.12E-13	postive
GLS	SH3GL1P1	0.304286435	3.59E-12	postive
GLS	SNHG15	0.313245738	7.61E-13	postive
GLS	SP2-AS1	0.398613437	1.72E-20	postive
GLS	SVIL2P	0.307430514	2.09E-12	postive
GLS	TBC1D29P	0.361714251	6.69E-17	postive
GLS	TIMM23B-AGAP6	0.303901325	3.83E-12	postive
GLS	TPI1P2	0.341128107	4.32E-15	postive
GLS	TRIM64DP	0.30470473	3.34E-12	postive
GLS	WAKMAR2	0.303238816	4.29E-12	postive
GLS	Z69666.1	0.350432136	6.82E-16	postive
GLS	Z94160.1	0.384649677	4.45E-19	postive
GLS	Z94721.1	0.375740508	3.27E-18	postive
GLS	Z98885.2	0.387799071	2.17E-19	postive
GLS	Z98885.3	0.31855307	2.96E-13	postive
GLS	ZNF876P	0.300294287	7.04E-12	postive
GPC1	AC002467.1	0.302871362	4.56E-12	postive
GPC1	AC005034.4	0.315922606	4.74E-13	postive
GPC1	AC005261.4	0.323773357	1.15E-13	postive
GPC1	AC005288.1	0.313679287	7.05E-13	postive
GPC1	AC007383.2	0.319906562	2.32E-13	postive
GPC1	AC007785.1	0.321420021	1.76E-13	postive
GPC1	AC009126.1	0.318378672	3.06E-13	postive
GPC1	AC009237.14	0.33423772	1.63E-14	postive
GPC1	AC009509.5	0.311367594	1.06E-12	postive
GPC1	AC010913.1	0.302263383	5.05E-12	postive
GPC1	AC011451.1	0.315963294	4.70E-13	postive
GPC1	AC012063.1	0.345180804	1.95E-15	postive
GPC1	AC012306.2	0.355867844	2.25E-16	postive
GPC1	AC012603.1	0.301997534	5.29E-12	postive
GPC1	AC023301.1	0.369765883	1.20E-17	postive
GPC1	AC024267.7	0.313669468	7.06E-13	postive
GPC1	AC026401.3	0.309054518	1.58E-12	postive
GPC1	AC027237.5	0.383087695	6.34E-19	postive
GPC1	AC027702.2	0.303221704	4.30E-12	postive

GPC1	AC046143.1	0.32098116	1.91E-13	postive
GPC1	AC046143.2	0.35353029	3.64E-16	postive
GPC1	AC053503.4	0.330812733	3.11E-14	postive
GPC1	AC067852.2	0.39768783	2.15E-20	postive
GPC1	AC068580.3	0.344888944	2.06E-15	postive
GPC1	AC068870.2	0.319906192	2.32E-13	postive
GPC1	AC073569.2	0.306413439	2.49E-12	postive
GPC1	AC079922.2	0.337400235	8.89E-15	postive
GPC1	AC080112.2	0.320456642	2.10E-13	postive
GPC1	AC084757.3	0.312079781	9.34E-13	postive
GPC1	AC087645.2	0.317722453	3.44E-13	postive
GPC1	AC090515.3	0.305520081	2.91E-12	postive
GPC1	AC090617.5	0.311099628	1.11E-12	postive
GPC1	AC092171.2	0.310050737	1.33E-12	postive
GPC1	AC092376.2	0.33118292	2.90E-14	postive
GPC1	AC092431.1	0.312619955	8.49E-13	postive
GPC1	AC093673.1	0.419152329	1.08E-22	postive
GPC1	AC097468.3	0.317998123	3.27E-13	postive
GPC1	AC099066.2	0.344715913	2.14E-15	postive
GPC1	AC100847.1	0.306828141	2.32E-12	postive
GPC1	AC104411.2	0.32128328	1.81E-13	postive
GPC1	AC108463.2	0.330325364	3.41E-14	postive
GPC1	AC109347.1	0.3137451	6.97E-13	postive
GPC1	AC114271.1	0.362713008	5.42E-17	postive
GPC1	AC128709.1	0.318341296	3.08E-13	postive
GPC1	AC128709.2	0.321406516	1.77E-13	postive
GPC1	AC130371.2	0.350860481	6.25E-16	postive
GPC1	AC131235.3	0.302830483	4.59E-12	postive
GPC1	AC132008.2	0.334322758	1.60E-14	postive
GPC1	AC134043.2	0.304900174	3.23E-12	postive
GPC1	AC134312.5	0.33294702	2.08E-14	postive
GPC1	AC135050.1	0.301782268	5.48E-12	postive
GPC1	AC142381.3	0.321906927	1.62E-13	postive
GPC1	AC147067.1	0.346444641	1.52E-15	postive
GPC1	AF015262.1	0.314830139	5.75E-13	postive
GPC1	AGAP2-AS1	0.424614407	2.64E-23	postive
GPC1	AL008718.2	0.322138592	1.55E-13	postive
GPC1	AL021392.1	0.303107607	4.38E-12	postive
GPC1	AL022344.1	0.300182319	7.17E-12	postive
GPC1	AL024507.3	0.303290076	4.25E-12	postive
GPC1	AL031055.1	0.307573235	2.04E-12	postive
GPC1	AL033384.1	0.335697002	1.23E-14	postive
GPC1	AL049597.2	0.319539418	2.48E-13	postive
GPC1	AL117332.1	0.307540053	2.06E-12	postive
GPC1	AL118506.1	0.328390916	4.90E-14	postive
GPC1	AL137186.2	0.304096321	3.71E-12	postive
GPC1	AL139035.1	0.307542322	2.05E-12	postive
GPC1	AL139339.1	0.30121738	6.03E-12	postive
GPC1	AL139393.3	0.305376214	2.98E-12	postive
GPC1	AL162231.2	0.332979646	2.07E-14	postive

GPC1	AL354740.1	0.327130388	6.19E-14	postive
GPC1	AL356515.1	0.325732337	8.02E-14	postive
GPC1	AL359643.3	0.304709616	3.34E-12	postive
GPC1	AL390955.2	0.317212736	3.76E-13	postive
GPC1	AL441992.2	0.330968628	3.02E-14	postive
GPC1	AL450326.1	0.319665058	2.42E-13	postive
GPC1	AL590428.1	0.30821689	1.83E-12	postive
GPC1	AL604028.1	0.320523382	2.08E-13	postive
GPC1	AP000695.1	0.422314056	4.79E-23	postive
GPC1	AP000695.2	0.3760875	3.03E-18	postive
GPC1	AP001107.5	0.378548566	1.76E-18	postive
GPC1	AP001160.2	0.303439911	4.14E-12	postive
GPC1	AP001330.4	0.371102836	9.02E-18	postive
GPC1	AP001816.1	0.332540003	2.25E-14	postive
GPC1	AP006621.3	0.316539267	4.25E-13	postive
GPC1	ARRDC1-AS1	0.355489409	2.44E-16	postive
GPC1	ASB16-AS1	0.333200054	1.98E-14	postive
GPC1	BTG3-AS1	0.320604718	2.05E-13	postive
GPC1	BX322562.1	0.345187586	1.94E-15	postive
GPC1	BX640514.2	0.331539905	2.72E-14	postive
GPC1	C22orf46	0.329293262	4.14E-14	postive
GPC1	C2orf27A	0.330401244	3.36E-14	postive
GPC1	CENPBD1P1	0.373425342	5.44E-18	postive
GPC1	CIDECP1	0.331585619	2.69E-14	postive
GPC1	CT75	0.322264968	1.51E-13	postive
GPC1	CTBP1-DT	0.323901139	1.12E-13	postive
GPC1	CYTOR	0.311828584	9.76E-13	postive
GPC1	DBIL5P	0.308369713	1.78E-12	postive
GPC1	EIF2AK3-DT	0.302545449	4.82E-12	postive
GPC1	EMC1-AS1	0.322345568	1.49E-13	postive
GPC1	FAHD2CP	0.34405725	2.43E-15	postive
GPC1	FAM225B	0.323844431	1.13E-13	postive
GPC1	FAM86JP	0.372549755	6.58E-18	postive
GPC1	FGD5-AS1	0.311132204	1.10E-12	postive
GPC1	FO393418.1	0.305654114	2.84E-12	postive
GPC1	FOXD2-AS1	0.326938282	6.41E-14	postive
GPC1	GCC2-AS1	0.31179378	9.82E-13	postive
GPC1	GPRC5D-AS1	0.313089637	7.82E-13	postive
GPC1	ILF3-DT	0.332372811	2.32E-14	postive
GPC1	KRT8P12	0.460155	1.44E-27	postive
GPC1	LINC00205	0.316894398	3.98E-13	postive
GPC1	LINC00346	0.363732252	4.37E-17	postive
GPC1	LINC00471	0.313255059	7.60E-13	postive
GPC1	LINC00885	0.304663471	3.36E-12	postive
GPC1	LINC01096	0.410552405	9.43E-22	postive
GPC1	LINC01128	0.314478509	6.12E-13	postive
GPC1	LINC01137	0.318819836	2.82E-13	postive
GPC1	LINC01561	0.339513818	5.91E-15	postive
GPC1	LINC01583	0.303118345	4.37E-12	postive
GPC1	LINC01615	0.301180837	6.07E-12	postive

GPC1	LINC01704	0.310130552	1.31E-12	postive
GPC1	LINC01711	0.308440077	1.76E-12	postive
GPC1	LINC01932	0.314631583	5.96E-13	postive
GPC1	LINC01968	0.3614396	7.09E-17	postive
GPC1	LINC02298	0.309728201	1.41E-12	postive
GPC1	LINC02544	0.386890087	2.67E-19	postive
GPC1	LINC02593	0.308238006	1.82E-12	postive
GPC1	LINC02693	0.398458267	1.79E-20	postive
GPC1	LIVAR	0.308290535	1.81E-12	postive
GPC1	LNCTAM34A	0.309724296	1.41E-12	postive
GPC1	LPP-AS2	0.403138767	5.81E-21	postive
GPC1	LSP1P4	0.336077876	1.15E-14	postive
GPC1	LUARIS	0.341796558	3.79E-15	postive
GPC1	MAP3K2-DT	0.318083148	3.22E-13	postive
GPC1	MELTF-AS1	0.323168873	1.28E-13	postive
GPC1	MIR193BHG	0.323912064	1.12E-13	postive
GPC1	MIR4435-2HG	0.412106995	6.40E-22	postive
GPC1	MRPL20-AS1	0.305037536	3.16E-12	postive
GPC1	MSC-AS1	0.332154054	2.42E-14	postive
GPC1	MSL3P1	0.332405421	2.31E-14	postive
GPC1	NAPA-AS1	0.320487992	2.09E-13	postive
GPC1	NIFK-AS1	0.317692041	3.46E-13	postive
GPC1	NOP14-AS1	0.371697656	7.93E-18	postive
GPC1	NORAD	0.361769858	6.61E-17	postive
GPC1	NRAV	0.304577371	3.41E-12	postive
GPC1	NUDT16P1	0.320032656	2.27E-13	postive
GPC1	OSER1-DT	0.314028685	6.63E-13	postive
GPC1	PGAM1P8	0.321385686	1.78E-13	postive
GPC1	PINK1-AS	0.408647479	1.51E-21	postive
GPC1	PLEKHA8P1	0.306483756	2.46E-12	postive
GPC1	PTENP1	0.308740772	1.67E-12	postive
GPC1	RASAL2-AS1	0.337526453	8.68E-15	postive
GPC1	RNF216P1	0.32635469	7.15E-14	postive
GPC1	RP1-93H18.1	0.308506867	1.74E-12	postive
GPC1	RPL23AP7	0.319590703	2.46E-13	postive
GPC1	RPL23AP82	0.347118366	1.33E-15	postive
GPC1	RPS18P9	0.328196536	5.08E-14	postive
GPC1	SAP30-DT	0.313895446	6.78E-13	postive
GPC1	SEPTIN4-AS1	0.31424028	6.38E-13	postive
GPC1	SH3RF3-AS1	0.351595178	5.39E-16	postive
GPC1	SNAI3-AS1	0.304414546	3.51E-12	postive
GPC1	SPATA41	0.33742397	8.85E-15	postive
GPC1	STARD7-AS1	0.386324613	3.04E-19	postive
GPC1	TM4SF19-AS1	0.302242782	5.07E-12	postive
GPC1	TMEM44-AS1	0.320867054	1.95E-13	postive
GPC1	TMEM92-AS1	0.408513892	1.56E-21	postive
GPC1	TPRG1-AS1	0.312363471	8.88E-13	postive
GPC1	TTC28-AS1	0.300640842	6.64E-12	postive
GPC1	TUG1	0.310380723	1.26E-12	postive
GPC1	U47924.2	0.344713296	2.14E-15	postive

GPC1	UBA6-AS1	0.328282624	5.00E-14	postive
GPC1	WAC-AS1	0.303503563	4.10E-12	postive
GPC1	WNT5A-AS1	0.303701857	3.96E-12	postive
GPC1	ZBTB11-AS1	0.301211587	6.03E-12	postive
GPC1	ZEB1-AS1	0.318891991	2.79E-13	postive
HEPH	ABHD11-AS1	0.311709565	9.96E-13	postive
HEPH	AC005381.1	0.592268073	1.16E-48	postive
HEPH	AC005550.2	0.3158569	4.79E-13	postive
HEPH	AC007663.2	0.361501558	6.99E-17	postive
HEPH	AC007998.3	0.313779269	6.93E-13	postive
HEPH	AC008691.1	0.419967283	8.77E-23	postive
HEPH	AC008897.3	0.32898149	4.39E-14	postive
HEPH	AC009078.2	0.382318289	7.54E-19	postive
HEPH	AC009093.1	0.304151546	3.67E-12	postive
HEPH	AC009093.2	0.301758563	5.50E-12	postive
HEPH	AC009509.5	0.349063525	8.98E-16	postive
HEPH	AC010503.5	0.325897514	7.78E-14	postive
HEPH	AC010547.2	0.382432181	7.35E-19	postive
HEPH	AC012494.1	0.352037061	4.93E-16	postive
HEPH	AC015908.3	0.319647132	2.43E-13	postive
HEPH	AC016027.1	0.301848921	5.42E-12	postive
HEPH	AC018904.1	0.513690626	5.13E-35	postive
HEPH	AC020779.2	0.349499406	8.23E-16	postive
HEPH	AC021218.1	0.643464307	8.74E-60	postive
HEPH	AC021483.2	0.312710111	8.36E-13	postive
HEPH	AC022150.1	0.451106275	1.96E-26	postive
HEPH	AC024651.2	0.44747025	5.46E-26	postive
HEPH	AC025265.3	0.584272255	4.23E-47	postive
HEPH	AC068631.1	0.344995333	2.02E-15	postive
HEPH	AC068870.2	0.304758667	3.31E-12	postive
HEPH	AC079466.1	0.324020273	1.10E-13	postive
HEPH	AC087857.1	0.487428814	3.37E-31	postive
HEPH	AC090376.1	0.335129015	1.37E-14	postive
HEPH	AC090409.1	0.312161027	9.21E-13	postive
HEPH	AC092376.2	0.363349656	4.74E-17	postive
HEPH	AC092818.1	0.540241904	3.15E-39	postive
HEPH	AC100807.2	0.307061055	2.23E-12	postive
HEPH	AC103563.7	0.373559997	5.28E-18	postive
HEPH	AC105399.1	0.315739432	4.89E-13	postive
HEPH	AC106876.1	0.340542762	4.84E-15	postive
HEPH	AC106881.1	0.309829454	1.38E-12	postive
HEPH	AC116337.3	0.424631342	2.63E-23	postive
HEPH	AC120049.1	0.346111024	1.62E-15	postive
HEPH	AC134312.5	0.301754025	5.51E-12	postive
HEPH	AF124730.2	0.328840935	4.50E-14	postive
HEPH	AGAP2-AS1	0.317322833	3.69E-13	postive
HEPH	AL009178.2	0.308314366	1.80E-12	postive
HEPH	AL118505.1	0.519046443	7.77E-36	postive
HEPH	AL121761.1	0.314334554	6.28E-13	postive
HEPH	AL121894.2	0.318906755	2.78E-13	postive

HEPH	AL121895.2	0.38773012	2.20E-19	postive
HEPH	AL133355.1	0.335403915	1.30E-14	postive
HEPH	AL136084.3	0.331353304	2.81E-14	postive
HEPH	AL138930.1	0.492236182	7.14E-32	postive
HEPH	AL139246.3	0.561687727	6.47E-43	postive
HEPH	AL158206.1	0.503559875	1.67E-33	postive
HEPH	AL160286.3	0.377562765	2.19E-18	postive
HEPH	AL162727.1	0.388490708	1.85E-19	postive
HEPH	AL358134.1	0.320389903	2.13E-13	postive
HEPH	AL365203.2	0.322185188	1.54E-13	postive
HEPH	AL583722.1	0.375043259	3.81E-18	postive
HEPH	AL590627.1	0.444382482	1.29E-25	postive
HEPH	AP000679.1	0.328201954	5.07E-14	postive
HEPH	AP000695.1	0.372187095	7.12E-18	postive
HEPH	AP000695.2	0.41487798	3.20E-22	postive
HEPH	AP001189.3	0.319699398	2.41E-13	postive
HEPH	AP001330.4	0.314637619	5.95E-13	postive
HEPH	AP003123.1	0.41099046	8.45E-22	postive
HEPH	BMPR1B-DT	0.366816358	2.27E-17	postive
HEPH	BX324167.1	0.394481609	4.58E-20	postive
HEPH	BX324167.2	0.344770251	2.11E-15	postive
HEPH	C2orf27A	0.319079514	2.69E-13	postive
HEPH	FGD5-AS1	0.323815853	1.14E-13	postive
HEPH	GPC5-IT1	0.307760171	1.98E-12	postive
HEPH	HCG21	0.417818587	1.52E-22	postive
HEPH	HOTTIP	0.33449871	1.55E-14	postive
HEPH	LEF1-AS1	0.322421689	1.47E-13	postive
HEPH	LINC00365	0.457208562	3.39E-27	postive
HEPH	LINC00443	0.493317375	5.02E-32	postive
HEPH	LINC00940	0.349911891	7.57E-16	postive
HEPH	LINC01033	0.353631567	3.56E-16	postive
HEPH	LINC01133	0.499738716	6.02E-33	postive
HEPH	LINC01559	0.627549462	4.19E-56	postive
HEPH	LINC01561	0.375651861	3.33E-18	postive
HEPH	LINC01594	0.314886466	5.69E-13	postive
HEPH	LINC01614	0.332820138	2.13E-14	postive
HEPH	LINC01713	0.454874996	6.65E-27	postive
HEPH	LINC01978	0.31833855	3.08E-13	postive
HEPH	LINC02011	0.359282337	1.11E-16	postive
HEPH	LINC02244	0.310418786	1.25E-12	postive
HEPH	LINC02324	0.329307106	4.13E-14	postive
HEPH	LINC02544	0.314226115	6.40E-13	postive
HEPH	LINC02593	0.399082024	1.54E-20	postive
HEPH	LINC02747	0.489812232	1.57E-31	postive
HEPH	LOXL1-AS1	0.344565246	2.20E-15	postive
HEPH	MGAT3-AS1	0.32109904	1.87E-13	postive
HEPH	MIR194-2HG	0.322562705	1.43E-13	postive
HEPH	MRPS30-DT	0.3406758	4.72E-15	postive
HEPH	MUC12-AS1	0.481753617	2.04E-30	postive
HEPH	MYRF-AS1	0.325998562	7.63E-14	postive

HEPH	OR7E22P	0.412016544	6.55E-22	postive
HEPH	PSMD10P2	0.553834531	1.56E-41	postive
HEPH	RASA4CP	0.355806455	2.28E-16	postive
HEPH	RP11-10C24.2	0.306017206	2.67E-12	postive
HEPH	RP11-514D23.3	0.319216634	2.63E-13	postive
HEPH	RP11-86H7.7	0.314137328	6.50E-13	postive
HEPH	RP3-355L5.5	0.368587123	1.55E-17	postive
HEPH	SH3RF3-AS1	0.321214471	1.83E-13	postive
HEPH	SSTR5-AS1	0.480794352	2.76E-30	postive
HEPH	TBX5-AS1	0.350780126	6.36E-16	postive
HEPH	TLX1NB	0.335180348	1.36E-14	postive
HEPH	TNFRSF10A-AS1	0.318473619	3.00E-13	postive
LIAS	ABCA11P	0.367186088	2.10E-17	postive
LIAS	AC002116.2	0.322888019	1.35E-13	postive
LIAS	AC002310.1	0.377236159	2.35E-18	postive
LIAS	AC002467.1	0.523956896	1.34E-36	postive
LIAS	AC004241.1	0.467175845	1.79E-28	postive
LIAS	AC004241.2	0.444851749	1.14E-25	postive
LIAS	AC004490.1	0.336109747	1.14E-14	postive
LIAS	AC004596.1	0.369272421	1.34E-17	postive
LIAS	AC004771.2	0.367291727	2.05E-17	postive
LIAS	AC004803.1	0.336916237	9.76E-15	postive
LIAS	AC004812.2	0.313471563	7.31E-13	postive
LIAS	AC004862.1	0.425004481	2.39E-23	postive
LIAS	AC005034.4	0.352124659	4.84E-16	postive
LIAS	AC005037.1	0.32163766	1.70E-13	postive
LIAS	AC005076.2	0.34263343	3.22E-15	postive
LIAS	AC005229.4	0.338997812	6.53E-15	postive
LIAS	AC005280.1	0.430281781	5.96E-24	postive
LIAS	AC005324.5	0.369834475	1.19E-17	postive
LIAS	AC005520.4	0.424096696	3.02E-23	postive
LIAS	AC005562.1	0.32096623	1.92E-13	postive
LIAS	AC005670.1	0.312363538	8.88E-13	postive
LIAS	AC005670.3	0.37250567	6.65E-18	postive
LIAS	AC006001.2	0.471750544	4.51E-29	postive
LIAS	AC006213.1	0.360176608	9.23E-17	postive
LIAS	AC006252.1	0.37437531	4.42E-18	postive
LIAS	AC006333.1	0.333131983	2.01E-14	postive
LIAS	AC006449.2	0.414183201	3.81E-22	postive
LIAS	AC006449.7	0.372099619	7.26E-18	postive
LIAS	AC007100.1	0.435488129	1.48E-24	postive
LIAS	AC007114.1	0.318514957	2.98E-13	postive
LIAS	AC007228.9	0.325805583	7.91E-14	postive
LIAS	AC007298.2	0.385551869	3.62E-19	postive
LIAS	AC007318.3	0.348587499	9.88E-16	postive
LIAS	AC007342.4	0.310507563	1.23E-12	postive
LIAS	AC007383.2	0.489778573	1.58E-31	postive
LIAS	AC007566.1	0.309332963	1.51E-12	postive
LIAS	AC007773.1	0.316091158	4.60E-13	postive
LIAS	AC008014.1	0.37400216	4.79E-18	postive

LIAS	AC008124.1	0.300223409	7.12E-12	postive
LIAS	AC008267.5	0.470345015	6.91E-29	postive
LIAS	AC008280.3	0.358748204	1.24E-16	postive
LIAS	AC008393.1	0.348245825	1.06E-15	postive
LIAS	AC008438.1	0.394921527	4.13E-20	postive
LIAS	AC008443.1	0.474358985	2.03E-29	postive
LIAS	AC008610.1	0.369665937	1.23E-17	postive
LIAS	AC008771.1	0.362354791	5.85E-17	postive
LIAS	AC008915.3	0.393395344	5.91E-20	postive
LIAS	AC008946.1	0.347766457	1.16E-15	postive
LIAS	AC008966.1	0.546229387	3.13E-40	postive
LIAS	AC009065.9	0.331164007	2.91E-14	postive
LIAS	AC009113.1	0.359353299	1.10E-16	postive
LIAS	AC009118.3	0.324339675	1.04E-13	postive
LIAS	AC009126.1	0.389624878	1.42E-19	postive
LIAS	AC009171.2	0.309784684	1.39E-12	postive
LIAS	AC009226.1	0.320226542	2.19E-13	postive
LIAS	AC009403.1	0.57023272	1.84E-44	postive
LIAS	AC009506.2	0.354293407	3.11E-16	postive
LIAS	AC009690.2	0.402066186	7.52E-21	postive
LIAS	AC009779.3	0.416154024	2.32E-22	postive
LIAS	AC009831.1	0.318349702	3.07E-13	postive
LIAS	AC010273.2	0.348398204	1.03E-15	postive
LIAS	AC010273.3	0.313813744	6.88E-13	postive
LIAS	AC010319.4	0.303562434	4.06E-12	postive
LIAS	AC010737.1	0.324092597	1.08E-13	postive
LIAS	AC010761.1	0.383369503	5.95E-19	postive
LIAS	AC010761.6	0.396613749	2.77E-20	postive
LIAS	AC010809.1	0.330836239	3.10E-14	postive
LIAS	AC010894.2	0.33088506	3.07E-14	postive
LIAS	AC010969.2	0.335724398	1.23E-14	postive
LIAS	AC010973.2	0.310397217	1.25E-12	postive
LIAS	AC011450.1	0.313403539	7.40E-13	postive
LIAS	AC011461.1	0.320963949	1.92E-13	postive
LIAS	AC011921.1	0.365041654	3.31E-17	postive
LIAS	AC012063.1	0.429578005	7.18E-24	postive
LIAS	AC012146.1	0.54028207	3.10E-39	postive
LIAS	AC012360.3	0.32799764	5.27E-14	postive
LIAS	AC012467.2	0.471905716	4.30E-29	postive
LIAS	AC012555.1	0.31802381	3.26E-13	postive
LIAS	AC012603.1	0.318346541	3.07E-13	postive
LIAS	AC012676.1	0.480212621	3.31E-30	postive
LIAS	AC013468.1	0.322642677	1.41E-13	postive
LIAS	AC015802.4	0.300703593	6.57E-12	postive
LIAS	AC015917.2	0.492766358	6.01E-32	postive
LIAS	AC015982.2	0.353544592	3.63E-16	postive
LIAS	AC016044.1	0.303373609	4.19E-12	postive
LIAS	AC016394.2	0.446414816	7.34E-26	postive
LIAS	AC016405.3	0.314720815	5.86E-13	postive
LIAS	AC016727.1	0.409411684	1.25E-21	postive

LIAS	AC016747.1	0.365699827	2.88E-17	postive
LIAS	AC016876.3	0.48463285	8.22E-31	postive
LIAS	AC018413.1	0.358400406	1.34E-16	postive
LIAS	AC018521.5	0.48583821	5.61E-31	postive
LIAS	AC018521.6	0.313185827	7.69E-13	postive
LIAS	AC018521.7	0.301379145	5.87E-12	postive
LIAS	AC018647.2	0.356092707	2.15E-16	postive
LIAS	AC018653.3	0.338071898	7.81E-15	postive
LIAS	AC018690.1	0.385911522	3.34E-19	postive
LIAS	AC018809.2	0.394252323	4.83E-20	postive
LIAS	AC019131.2	0.315046546	5.54E-13	postive
LIAS	AC019185.1	0.321226181	1.83E-13	postive
LIAS	AC019294.2	0.359866763	9.85E-17	postive
LIAS	AC020743.3	0.302805545	4.61E-12	postive
LIAS	AC020915.3	0.355234315	2.57E-16	postive
LIAS	AC021074.3	0.428221303	1.03E-23	postive
LIAS	AC021148.1	0.549360986	9.18E-41	postive
LIAS	AC021224.1	0.336221205	1.12E-14	postive
LIAS	AC021752.1	0.331843321	2.56E-14	postive
LIAS	AC021755.2	0.331293841	2.84E-14	postive
LIAS	AC021755.3	0.334759289	1.48E-14	postive
LIAS	AC021851.1	0.305680618	2.83E-12	postive
LIAS	AC022098.1	0.318004124	3.27E-13	postive
LIAS	AC022101.1	0.301990139	5.29E-12	postive
LIAS	AC022167.2	0.316258524	4.46E-13	postive
LIAS	AC022306.2	0.408245232	1.67E-21	postive
LIAS	AC022400.5	0.330148981	3.53E-14	postive
LIAS	AC022613.2	0.345955566	1.67E-15	postive
LIAS	AC022816.1	0.413321772	4.72E-22	postive
LIAS	AC023043.1	0.363992578	4.14E-17	postive
LIAS	AC023090.1	0.36854611	1.57E-17	postive
LIAS	AC023509.3	0.405772951	3.06E-21	postive
LIAS	AC023790.2	0.303283842	4.25E-12	postive
LIAS	AC024575.2	0.398075279	1.96E-20	postive
LIAS	AC024896.1	0.338981597	6.55E-15	postive
LIAS	AC024941.2	0.323096177	1.30E-13	postive
LIAS	AC025048.2	0.32850609	4.79E-14	postive
LIAS	AC025254.1	0.402816114	6.28E-21	postive
LIAS	AC025265.1	0.329358232	4.09E-14	postive
LIAS	AC025682.2	0.339190904	6.29E-15	postive
LIAS	AC026191.1	0.385299861	3.84E-19	postive
LIAS	AC026202.2	0.33825155	7.55E-15	postive
LIAS	AC026367.3	0.337161308	9.31E-15	postive
LIAS	AC026462.3	0.407034584	2.25E-21	postive
LIAS	AC026471.1	0.32430755	1.04E-13	postive
LIAS	AC026691.1	0.300140028	7.22E-12	postive
LIAS	AC026785.3	0.31576566	4.87E-13	postive
LIAS	AC027097.1	0.364908415	3.41E-17	postive
LIAS	AC027237.4	0.377487091	2.22E-18	postive
LIAS	AC027644.3	0.321286742	1.81E-13	postive

LIAS	AC032044.1	0.313664572	7.07E-13	postive
LIAS	AC034102.8	0.318910394	2.78E-13	postive
LIAS	AC034236.2	0.332225035	2.39E-14	postive
LIAS	AC036176.1	0.346616584	1.46E-15	postive
LIAS	AC040970.1	0.35455448	2.95E-16	postive
LIAS	AC040977.1	0.366408426	2.48E-17	postive
LIAS	AC046134.2	0.398123895	1.94E-20	postive
LIAS	AC051619.4	0.387560643	2.29E-19	postive
LIAS	AC055720.2	0.317098223	3.84E-13	postive
LIAS	AC055855.2	0.327541584	5.74E-14	postive
LIAS	AC060780.1	0.366524004	2.42E-17	postive
LIAS	AC061961.2	0.385897553	3.35E-19	postive
LIAS	AC064807.1	0.322301939	1.50E-13	postive
LIAS	AC067838.1	0.327772891	5.49E-14	postive
LIAS	AC067852.2	0.445915823	8.44E-26	postive
LIAS	AC067930.3	0.443978953	1.45E-25	postive
LIAS	AC068338.2	0.464067171	4.54E-28	postive
LIAS	AC068506.1	0.388348173	1.91E-19	postive
LIAS	AC068831.2	0.367040027	2.16E-17	postive
LIAS	AC068831.3	0.301799946	5.47E-12	postive
LIAS	AC068870.2	0.318365696	3.06E-13	postive
LIAS	AC068888.1	0.353136521	3.94E-16	postive
LIAS	AC069185.1	0.327079145	6.25E-14	postive
LIAS	AC073111.1	0.337022439	9.56E-15	postive
LIAS	AC073254.1	0.318329005	3.08E-13	postive
LIAS	AC073508.3	0.334653079	1.51E-14	postive
LIAS	AC073611.1	0.349975887	7.48E-16	postive
LIAS	AC073842.1	0.361612282	6.83E-17	postive
LIAS	AC073896.2	0.39185208	8.48E-20	postive
LIAS	AC073896.3	0.300121531	7.25E-12	postive
LIAS	AC074117.1	0.347247544	1.29E-15	postive
LIAS	AC078881.1	0.314115794	6.53E-13	postive
LIAS	AC079145.1	0.309768341	1.40E-12	postive
LIAS	AC079360.1	0.314125727	6.51E-13	postive
LIAS	AC079921.2	0.439925135	4.43E-25	postive
LIAS	AC080112.2	0.311032547	1.12E-12	postive
LIAS	AC083798.2	0.336341991	1.09E-14	postive
LIAS	AC083799.1	0.302090358	5.20E-12	postive
LIAS	AC083900.1	0.308147059	1.85E-12	postive
LIAS	AC084036.1	0.39453066	4.53E-20	postive
LIAS	AC084125.4	0.415365596	2.83E-22	postive
LIAS	AC087289.2	0.300098431	7.27E-12	postive
LIAS	AC087741.2	0.369741525	1.21E-17	postive
LIAS	AC090204.1	0.390931167	1.05E-19	postive
LIAS	AC090587.1	0.364013626	4.12E-17	postive
LIAS	AC090587.2	0.375180066	3.70E-18	postive
LIAS	AC090617.5	0.307298745	2.14E-12	postive
LIAS	AC090825.1	0.31870465	2.88E-13	postive
LIAS	AC090912.1	0.382142138	7.85E-19	postive
LIAS	AC091057.1	0.41462732	3.40E-22	postive

LIAS	AC091133.3	0.359384625	1.09E-16	postive
LIAS	AC091153.3	0.395868629	3.30E-20	postive
LIAS	AC091390.5	0.325114008	8.98E-14	postive
LIAS	AC091729.3	0.392692072	6.97E-20	postive
LIAS	AC091887.1	0.311194203	1.09E-12	postive
LIAS	AC091965.1	0.390116386	1.27E-19	postive
LIAS	AC091965.4	0.318739047	2.86E-13	postive
LIAS	AC092115.4	0.429051855	8.25E-24	postive
LIAS	AC092123.1	0.342516775	3.29E-15	postive
LIAS	AC092134.1	0.361528665	6.95E-17	postive
LIAS	AC092164.1	0.322991366	1.33E-13	postive
LIAS	AC092295.1	0.329809949	3.76E-14	postive
LIAS	AC092295.2	0.316337384	4.40E-13	postive
LIAS	AC092634.3	0.40226038	7.18E-21	postive
LIAS	AC092925.1	0.434309885	2.03E-24	postive
LIAS	AC092944.1	0.344935144	2.04E-15	postive
LIAS	AC093157.1	0.425484706	2.10E-23	postive
LIAS	AC093227.1	0.309392773	1.49E-12	postive
LIAS	AC093297.2	0.341520434	4.00E-15	postive
LIAS	AC093484.2	0.342127347	3.55E-15	postive
LIAS	AC093484.4	0.366311416	2.53E-17	postive
LIAS	AC093510.1	0.301146609	6.10E-12	postive
LIAS	AC093582.1	0.346725885	1.43E-15	postive
LIAS	AC093627.5	0.419872753	8.98E-23	postive
LIAS	AC093726.1	0.381355926	9.37E-19	postive
LIAS	AC093752.1	0.372199205	7.11E-18	postive
LIAS	AC093752.3	0.315182254	5.40E-13	postive
LIAS	AC095057.3	0.345687695	1.76E-15	postive
LIAS	AC096536.2	0.423626305	3.41E-23	postive
LIAS	AC096586.2	0.300165575	7.19E-12	postive
LIAS	AC096772.6	0.307718482	1.99E-12	postive
LIAS	AC097359.2	0.325238687	8.78E-14	postive
LIAS	AC097468.1	0.322225892	1.52E-13	postive
LIAS	AC097468.3	0.363320737	4.77E-17	postive
LIAS	AC097505.1	0.326876102	6.49E-14	postive
LIAS	AC098484.1	0.387620253	2.26E-19	postive
LIAS	AC098484.2	0.316771766	4.07E-13	postive
LIAS	AC098487.1	0.391408998	9.40E-20	postive
LIAS	AC098820.2	0.514250463	4.22E-35	postive
LIAS	AC098820.4	0.426036029	1.82E-23	postive
LIAS	AC099552.1	0.434177034	2.11E-24	postive
LIAS	AC099552.3	0.44079123	3.49E-25	postive
LIAS	AC099778.1	0.320731941	2.00E-13	postive
LIAS	AC099850.2	0.369252797	1.35E-17	postive
LIAS	AC100786.1	0.374806489	4.02E-18	postive
LIAS	AC100810.1	0.309388645	1.49E-12	postive
LIAS	AC100847.1	0.40375563	5.00E-21	postive
LIAS	AC100861.1	0.351985944	4.98E-16	postive
LIAS	AC103746.1	0.419700641	9.39E-23	postive
LIAS	AC103760.1	0.365311154	3.13E-17	postive

LIAS	AC104051.2	0.431726488	4.06E-24	postive
LIAS	AC104109.2	0.307090267	2.22E-12	postive
LIAS	AC104113.1	0.388680507	1.77E-19	postive
LIAS	AC104667.2	0.35431667	3.10E-16	postive
LIAS	AC105036.3	0.436558424	1.11E-24	postive
LIAS	AC105105.2	0.368536489	1.57E-17	postive
LIAS	AC105285.1	0.371138157	8.95E-18	postive
LIAS	AC105942.1	0.315480011	5.13E-13	postive
LIAS	AC106028.3	0.314395886	6.21E-13	postive
LIAS	AC106795.1	0.365014017	3.33E-17	postive
LIAS	AC107032.2	0.452332209	1.38E-26	postive
LIAS	AC107068.1	0.445148294	1.05E-25	postive
LIAS	AC107214.1	0.317441062	3.61E-13	postive
LIAS	AC107375.1	0.316552538	4.24E-13	postive
LIAS	AC108471.3	0.408740291	1.48E-21	postive
LIAS	AC108517.1	0.372715126	6.35E-18	postive
LIAS	AC109322.1	0.327354743	5.94E-14	postive
LIAS	AC109347.1	0.414041328	3.94E-22	postive
LIAS	AC110079.1	0.335163119	1.37E-14	postive
LIAS	AC112220.2	0.351196596	5.84E-16	postive
LIAS	AC112491.1	0.34648691	1.50E-15	postive
LIAS	AC112694.1	0.320335233	2.15E-13	postive
LIAS	AC114271.1	0.360446693	8.72E-17	postive
LIAS	AC114485.1	0.393931498	5.21E-20	postive
LIAS	AC114763.1	0.308182849	1.84E-12	postive
LIAS	AC114810.1	0.340320124	5.06E-15	postive
LIAS	AC114956.2	0.318806886	2.83E-13	postive
LIAS	AC115618.1	0.488813808	2.16E-31	postive
LIAS	AC115618.2	0.424016278	3.08E-23	postive
LIAS	AC115619.1	0.432307467	3.47E-24	postive
LIAS	AC116913.1	0.442792027	2.01E-25	postive
LIAS	AC116914.2	0.348380187	1.03E-15	postive
LIAS	AC117394.2	0.32932547	4.11E-14	postive
LIAS	AC120053.1	0.305820997	2.76E-12	postive
LIAS	AC121338.2	0.33426645	1.62E-14	postive
LIAS	AC121761.1	0.382710018	6.91E-19	postive
LIAS	AC122108.3	0.332255357	2.37E-14	postive
LIAS	AC123768.2	0.400839324	1.01E-20	postive
LIAS	AC123768.3	0.339355685	6.10E-15	postive
LIAS	AC124016.2	0.335802764	1.21E-14	postive
LIAS	AC125257.1	0.306774235	2.34E-12	postive
LIAS	AC125616.1	0.342670811	3.19E-15	postive
LIAS	AC126323.3	0.398350288	1.83E-20	postive
LIAS	AC129507.2	0.307585817	2.04E-12	postive
LIAS	AC129507.3	0.340320038	5.06E-15	postive
LIAS	AC131009.3	0.455656485	5.31E-27	postive
LIAS	AC132938.3	0.307348326	2.12E-12	postive
LIAS	AC132938.4	0.413111966	4.98E-22	postive
LIAS	AC133528.2	0.396906391	2.58E-20	postive
LIAS	AC134878.2	0.315697263	4.93E-13	postive

LIAS	AC135050.1	0.338631472	7.01E-15	postive
LIAS	AC135050.6	0.341410759	4.09E-15	postive
LIAS	AC135178.4	0.365249213	3.17E-17	postive
LIAS	AC135178.6	0.339422043	6.02E-15	postive
LIAS	AC135803.1	0.302822498	4.60E-12	postive
LIAS	AC136475.2	0.300892725	6.37E-12	postive
LIAS	AC138035.1	0.363161411	4.93E-17	postive
LIAS	AC139887.1	0.313115016	7.79E-13	postive
LIAS	AC139887.2	0.394862822	4.19E-20	postive
LIAS	AC141586.1	0.374630465	4.18E-18	postive
LIAS	AC142472.1	0.338945107	6.60E-15	postive
LIAS	AC145207.1	0.302885154	4.55E-12	postive
LIAS	AC145423.3	0.300605564	6.68E-12	postive
LIAS	AC231533.2	0.308742376	1.67E-12	postive
LIAS	AC231981.1	0.383997666	5.16E-19	postive
LIAS	AC232271.1	0.348647223	9.76E-16	postive
LIAS	AC243919.1	0.541647406	1.84E-39	postive
LIAS	AC244669.1	0.330408972	3.36E-14	postive
LIAS	AC245140.2	0.342886622	3.06E-15	postive
LIAS	AC246785.3	0.363117442	4.98E-17	postive
LIAS	AC254562.3	0.301068466	6.18E-12	postive
LIAS	ACTG1P17	0.314266413	6.35E-13	postive
LIAS	ADCY6-DT	0.359018032	1.17E-16	postive
LIAS	AF196972.1	0.354469966	3.00E-16	postive
LIAS	AFG3L1P	0.33866663	6.97E-15	postive
LIAS	AHSA2P	0.31904911	2.71E-13	postive
LIAS	AL008635.1	0.301062358	6.19E-12	postive
LIAS	AL008721.1	0.374289893	4.50E-18	postive
LIAS	AL009179.1	0.461991422	8.38E-28	postive
LIAS	AL020996.1	0.320003142	2.28E-13	postive
LIAS	AL022157.1	0.379392344	1.45E-18	postive
LIAS	AL022311.1	0.376580261	2.72E-18	postive
LIAS	AL022324.3	0.341217342	4.24E-15	postive
LIAS	AL024498.1	0.343668317	2.63E-15	postive
LIAS	AL024507.3	0.389708193	1.39E-19	postive
LIAS	AL031282.1	0.31232724	8.94E-13	postive
LIAS	AL031282.2	0.349458549	8.30E-16	postive
LIAS	AL031673.1	0.31924827	2.61E-13	postive
LIAS	AL031775.1	0.371717889	7.89E-18	postive
LIAS	AL031775.2	0.379427518	1.44E-18	postive
LIAS	AL031847.1	0.304167274	3.66E-12	postive
LIAS	AL035530.2	0.325973396	7.67E-14	postive
LIAS	AL035661.1	0.419261321	1.05E-22	postive
LIAS	AL049543.1	0.304809761	3.28E-12	postive
LIAS	AL049597.2	0.315251804	5.34E-13	postive
LIAS	AL049796.1	0.314793723	5.79E-13	postive
LIAS	AL049840.4	0.353973452	3.32E-16	postive
LIAS	AL049840.5	0.340978402	4.45E-15	postive
LIAS	AL049840.6	0.341112428	4.33E-15	postive
LIAS	AL109917.1	0.324478804	1.01E-13	postive

LIAS	AL118506.1	0.308699388	1.68E-12	postive
LIAS	AL121658.1	0.364401557	3.79E-17	postive
LIAS	AL121944.2	0.55057964	5.67E-41	postive
LIAS	AL122023.3	0.32997853	3.64E-14	postive
LIAS	AL132712.1	0.336103986	1.14E-14	postive
LIAS	AL132780.1	0.350990942	6.09E-16	postive
LIAS	AL133215.1	0.312441943	8.76E-13	postive
LIAS	AL133215.3	0.302607306	4.77E-12	postive
LIAS	AL133353.1	0.33136589	2.81E-14	postive
LIAS	AL133355.1	0.337601602	8.56E-15	postive
LIAS	AL136040.1	0.397395699	2.30E-20	postive
LIAS	AL136084.2	0.312414611	8.80E-13	postive
LIAS	AL136295.2	0.307823033	1.96E-12	postive
LIAS	AL136295.7	0.302818189	4.60E-12	postive
LIAS	AL136304.1	0.350854847	6.26E-16	postive
LIAS	AL137003.1	0.315791024	4.85E-13	postive
LIAS	AL138762.2	0.348052057	1.10E-15	postive
LIAS	AL138826.1	0.447665101	5.17E-26	postive
LIAS	AL138921.1	0.394474638	4.59E-20	postive
LIAS	AL139011.1	0.369903238	1.17E-17	postive
LIAS	AL139021.2	0.311994123	9.48E-13	postive
LIAS	AL139039.3	0.403500421	5.32E-21	postive
LIAS	AL139089.1	0.309205412	1.54E-12	postive
LIAS	AL139289.1	0.367444384	1.98E-17	postive
LIAS	AL157400.2	0.349204459	8.73E-16	postive
LIAS	AL157832.1	0.311508524	1.03E-12	postive
LIAS	AL157896.1	0.426093932	1.79E-23	postive
LIAS	AL158071.3	0.30873264	1.67E-12	postive
LIAS	AL158071.4	0.342380662	3.38E-15	postive
LIAS	AL158212.3	0.303997696	3.77E-12	postive
LIAS	AL160006.1	0.341981463	3.66E-15	postive
LIAS	AL160291.1	0.314101744	6.54E-13	postive
LIAS	AL160313.2	0.319112441	2.68E-13	postive
LIAS	AL161421.1	0.378253917	1.87E-18	postive
LIAS	AL162231.2	0.344984301	2.02E-15	postive
LIAS	AL162258.2	0.403494666	5.33E-21	postive
LIAS	AL162411.1	0.319273002	2.60E-13	postive
LIAS	AL162595.1	0.364170797	3.98E-17	postive
LIAS	AL162632.3	0.323195755	1.28E-13	postive
LIAS	AL353622.1	0.322973559	1.33E-13	postive
LIAS	AL353622.2	0.398873036	1.62E-20	postive
LIAS	AL353708.1	0.49312743	5.34E-32	postive
LIAS	AL353708.3	0.377906528	2.03E-18	postive
LIAS	AL353743.1	0.318504412	2.99E-13	postive
LIAS	AL354696.1	0.465940428	2.60E-28	postive
LIAS	AL354696.2	0.459036925	1.99E-27	postive
LIAS	AL354892.2	0.327319161	5.98E-14	postive
LIAS	AL354920.1	0.54535779	4.39E-40	postive
LIAS	AL355102.4	0.440627777	3.65E-25	postive
LIAS	AL355388.1	0.335080889	1.39E-14	postive

LIAS	AL355574.1	0.335784768	1.21E-14	postive
LIAS	AL355802.3	0.394889788	4.16E-20	postive
LIAS	AL356488.3	0.324827554	9.47E-14	postive
LIAS	AL356599.1	0.31009398	1.32E-12	postive
LIAS	AL356652.1	0.40952658	1.22E-21	postive
LIAS	AL358472.2	0.349781604	7.77E-16	postive
LIAS	AL359232.1	0.47061121	6.37E-29	postive
LIAS	AL359397.1	0.352495966	4.49E-16	postive
LIAS	AL359541.2	0.360339868	8.92E-17	postive
LIAS	AL359643.3	0.457152182	3.45E-27	postive
LIAS	AL359715.3	0.34060699	4.78E-15	postive
LIAS	AL359878.1	0.320679558	2.02E-13	postive
LIAS	AL359921.1	0.311634297	1.01E-12	postive
LIAS	AL359921.2	0.346907801	1.38E-15	postive
LIAS	AL360270.1	0.321128941	1.86E-13	postive
LIAS	AL360270.3	0.321163899	1.85E-13	postive
LIAS	AL365330.1	0.332159656	2.42E-14	postive
LIAS	AL390195.2	0.329328743	4.11E-14	postive
LIAS	AL390728.4	0.363573721	4.52E-17	postive
LIAS	AL391001.1	0.311454042	1.04E-12	postive
LIAS	AL391095.3	0.403482098	5.34E-21	postive
LIAS	AL391244.2	0.349176929	8.78E-16	postive
LIAS	AL391684.1	0.325363236	8.58E-14	postive
LIAS	AL392172.1	0.307534579	2.06E-12	postive
LIAS	AL442663.3	0.325155505	8.92E-14	postive
LIAS	AL445231.1	0.390417266	1.18E-19	postive
LIAS	AL450326.1	0.312002352	9.46E-13	postive
LIAS	AL450384.2	0.31041219	1.25E-12	postive
LIAS	AL451165.2	0.394190603	4.91E-20	postive
LIAS	AL512353.1	0.308080337	1.87E-12	postive
LIAS	AL512625.1	0.319059903	2.70E-13	postive
LIAS	AL512791.2	0.309045823	1.58E-12	postive
LIAS	AL513165.1	0.318022557	3.26E-13	postive
LIAS	AL513550.1	0.462801792	6.60E-28	postive
LIAS	AL590652.1	0.348267352	1.05E-15	postive
LIAS	AL591043.2	0.335975004	1.17E-14	postive
LIAS	AL592166.1	0.447526801	5.38E-26	postive
LIAS	AL592435.1	0.335504624	1.28E-14	postive
LIAS	AL592494.1	0.448229021	4.41E-26	postive
LIAS	AL604028.1	0.397314586	2.35E-20	postive
LIAS	AL606760.1	0.323101929	1.30E-13	postive
LIAS	AL645568.1	0.379702531	1.36E-18	postive
LIAS	AL645608.2	0.319826599	2.35E-13	postive
LIAS	AL645940.1	0.351341521	5.67E-16	postive
LIAS	AL645941.1	0.339362387	6.09E-15	postive
LIAS	AL662791.1	0.324495146	1.01E-13	postive
LIAS	AL662797.2	0.309186377	1.55E-12	postive
LIAS	AL669831.1	0.334089579	1.68E-14	postive
LIAS	AL671710.1	0.340725099	4.67E-15	postive
LIAS	AL691432.1	0.357492488	1.61E-16	postive

LIAS	AL731566.2	0.332713045	2.18E-14	postive
LIAS	AL731569.1	0.305323928	3.01E-12	postive
LIAS	AL731571.1	0.310865509	1.15E-12	postive
LIAS	AL731577.2	0.313717245	7.00E-13	postive
LIAS	ALOX12-AS1	0.469027473	1.03E-28	postive
LIAS	AMZ2P1	0.394776346	4.27E-20	postive
LIAS	ANO7L1	0.321515897	1.73E-13	postive
LIAS	ANP32AP1	0.360007544	9.56E-17	postive
LIAS	AP000254.2	0.349803415	7.74E-16	postive
LIAS	AP000442.1	0.381377556	9.32E-19	postive
LIAS	AP000446.1	0.336995123	9.61E-15	postive
LIAS	AP000487.1	0.345878879	1.70E-15	postive
LIAS	AP000704.2	0.301429322	5.82E-12	postive
LIAS	AP000873.2	0.347861863	1.14E-15	postive
LIAS	AP001107.1	0.340336634	5.04E-15	postive
LIAS	AP001160.1	0.309249657	1.53E-12	postive
LIAS	AP001160.3	0.351093866	5.97E-16	postive
LIAS	AP001318.2	0.31823729	3.13E-13	postive
LIAS	AP001372.2	0.324951728	9.26E-14	postive
LIAS	AP001432.1	0.3228036	1.37E-13	postive
LIAS	AP001453.4	0.398506914	1.77E-20	postive
LIAS	AP001496.2	0.300751053	6.52E-12	postive
LIAS	AP001505.1	0.35348999	3.67E-16	postive
LIAS	AP001628.1	0.30934746	1.50E-12	postive
LIAS	AP001816.1	0.40905963	1.36E-21	postive
LIAS	AP002026.1	0.386966557	2.62E-19	postive
LIAS	AP002360.2	0.323776364	1.15E-13	postive
LIAS	AP002748.4	0.325358369	8.59E-14	postive
LIAS	AP002807.1	0.319033177	2.72E-13	postive
LIAS	AP003352.1	0.37860958	1.73E-18	postive
LIAS	AP003392.5	0.336530117	1.05E-14	postive
LIAS	AP005264.4	0.390201683	1.24E-19	postive
LIAS	AP005264.5	0.365194585	3.21E-17	postive
LIAS	AP005329.1	0.306318546	2.54E-12	postive
LIAS	AP005329.2	0.443791627	1.52E-25	postive
LIAS	AP005482.4	0.330718029	3.17E-14	postive
LIAS	AP006222.1	0.313487427	7.29E-13	postive
LIAS	AP006621.3	0.317602245	3.51E-13	postive
LIAS	APOC1P1	0.415326514	2.85E-22	postive
LIAS	APTR	0.421898412	5.34E-23	postive
LIAS	ARAP1-AS1	0.367063692	2.15E-17	postive
LIAS	ARF4-AS1	0.41746124	1.66E-22	postive
LIAS	ARMCX5-GPRASP2	0.418063728	1.43E-22	postive
LIAS	ARRDC1-AS1	0.374306607	4.48E-18	postive
LIAS	ASB16-AS1	0.339895269	5.49E-15	postive
LIAS	ASH1L-AS1	0.348307722	1.05E-15	postive
LIAS	ASMTL-AS1	0.317921134	3.32E-13	postive
LIAS	ATF4P4	0.381254666	9.58E-19	postive
LIAS	ATXN2-AS	0.340646287	4.74E-15	postive
LIAS	BAALC-AS1	0.304400224	3.52E-12	postive

LIAS	BCAR3-AS1	0.440032449	4.30E-25	postive
LIAS	BCDIN3D-AS1	0.433869215	2.29E-24	postive
LIAS	BDNF-AS	0.413376198	4.66E-22	postive
LIAS	BMS1P20	0.342792362	3.12E-15	postive
LIAS	BNIP3P1	0.302303041	5.02E-12	postive
LIAS	BTN2A3P	0.327919824	5.35E-14	postive
LIAS	BTNL10	0.352407756	4.57E-16	postive
LIAS	BX284668.5	0.304895478	3.23E-12	postive
LIAS	BX322557.10	0.328929533	4.43E-14	postive
LIAS	BX537318.1	0.436371608	1.16E-24	postive
LIAS	BX649632.1	0.389110596	1.60E-19	postive
LIAS	C15orf56	0.316876511	4.00E-13	postive
LIAS	C18orf65	0.370547445	1.02E-17	postive
LIAS	C1RL-AS1	0.313062157	7.86E-13	postive
LIAS	C21orf62-AS1	0.349097386	8.92E-16	postive
LIAS	C22orf46	0.39127264	9.70E-20	postive
LIAS	C3P1	0.393323478	6.01E-20	postive
LIAS	C5orf34-AS1	0.447534324	5.36E-26	postive
LIAS	C6orf99	0.303316507	4.23E-12	postive
LIAS	C9orf147	0.347811033	1.15E-15	postive
LIAS	CA3-AS1	0.337061973	9.49E-15	postive
LIAS	CA5BP1	0.357878916	1.49E-16	postive
LIAS	CATIP-AS1	0.340865487	4.55E-15	postive
LIAS	CATSPER2P1	0.340522462	4.86E-15	postive
LIAS	CCDC183-AS1	0.325440465	8.46E-14	postive
LIAS	CCDC18-AS1	0.300995364	6.26E-12	postive
LIAS	CCNT2-AS1	0.387680325	2.23E-19	postive
LIAS	CCT6P1	0.371621162	8.06E-18	postive
LIAS	CCT6P3	0.310575885	1.21E-12	postive
LIAS	CD27-AS1	0.353123791	3.95E-16	postive
LIAS	CDC37L1-DT	0.37470258	4.11E-18	postive
LIAS	CERNA1	0.391727021	8.73E-20	postive
LIAS	CFL1P1	0.410546616	9.44E-22	postive
LIAS	CHROMR	0.302393239	4.94E-12	postive
LIAS	CIDECP1	0.395954766	3.24E-20	postive
LIAS	CKMT2-AS1	0.437835113	7.83E-25	postive
LIAS	CNNM3-DT	0.339062593	6.45E-15	postive
LIAS	COA6-AS1	0.34547816	1.84E-15	postive
LIAS	COX10-AS1	0.425087818	2.33E-23	postive
LIAS	CR381653.1	0.316155465	4.55E-13	postive
LIAS	CRTC3-AS1	0.40416429	4.53E-21	postive
LIAS	CRYZL2P	0.446565734	7.04E-26	postive
LIAS	CSE1L-AS1	0.338915161	6.64E-15	postive
LIAS	CTA-989H11.1	0.38403887	5.11E-19	postive
LIAS	CTBP1-DT	0.53093929	1.04E-37	postive
LIAS	CTD-2134A5.3	0.367131545	2.12E-17	postive
LIAS	CTD-2521M24.9	0.33010964	3.55E-14	postive
LIAS	CTD-2574D22.4	0.314946704	5.63E-13	postive
LIAS	CU634019.6	0.324434118	1.02E-13	postive
LIAS	CYP51A1-AS1	0.338722859	6.89E-15	postive

LIAS	CYTOR	0.321277349	1.81E-13	postive
LIAS	DANCR	0.352426248	4.55E-16	postive
LIAS	DARS-AS1	0.485443411	6.36E-31	postive
LIAS	DDX39B-AS1	0.352073673	4.89E-16	postive
LIAS	DENND10P1	0.345770681	1.73E-15	postive
LIAS	DGCR11	0.32179975	1.65E-13	postive
LIAS	DHCR24-DT	0.388244854	1.95E-19	postive
LIAS	DICER1-AS1	0.330790974	3.13E-14	postive
LIAS	DLEU1	0.436653128	1.08E-24	postive
LIAS	DLGAP1-AS1	0.367403723	2.00E-17	postive
LIAS	DM1-AS	0.344692135	2.15E-15	postive
LIAS	DNAJC27-AS1	0.303433154	4.15E-12	postive
LIAS	DSTNP2	0.382253143	7.66E-19	postive
LIAS	DYRK3-AS1	0.313513361	7.26E-13	postive
LIAS	EBLN3P	0.32324299	1.27E-13	postive
LIAS	ECI2-DT	0.3144266	6.18E-13	postive
LIAS	EDRF1-DT	0.348134917	1.08E-15	postive
LIAS	EHMT2-AS1	0.327069669	6.26E-14	postive
LIAS	EIF1B-AS1	0.486689809	4.27E-31	postive
LIAS	EIF2AK3-DT	0.337112872	9.40E-15	postive
LIAS	EIF3J-DT	0.525144275	8.69E-37	postive
LIAS	ELF3-AS1	0.32786096	5.41E-14	postive
LIAS	ELOA-AS1	0.45976735	1.61E-27	postive
LIAS	ELOVL2-AS1	0.380819194	1.06E-18	postive
LIAS	EMC1-AS1	0.414423869	3.58E-22	postive
LIAS	EMSLR	0.464609479	3.86E-28	postive
LIAS	ENTPD3-AS1	0.540977799	2.38E-39	postive
LIAS	EPB41L4A-AS1	0.499477553	6.57E-33	postive
LIAS	F11-AS1	0.344694662	2.14E-15	postive
LIAS	FAHD2CP	0.357820735	1.51E-16	postive
LIAS	FALEC	0.307982482	1.90E-12	postive
LIAS	FAM111A-DT	0.311857464	9.71E-13	postive
LIAS	FAM160A1-DT	0.309296385	1.52E-12	postive
LIAS	FAM86B3P	0.324052853	1.09E-13	postive
LIAS	FAM86C2P	0.512439885	7.94E-35	postive
LIAS	FAM86DP	0.40067875	1.05E-20	postive
LIAS	FAM86EP	0.558249379	2.63E-42	postive
LIAS	FAM86FP	0.344132656	2.40E-15	postive
LIAS	FAM86HP	0.414232999	3.76E-22	postive
LIAS	FAM86JP	0.409879814	1.11E-21	postive
LIAS	FBXL19-AS1	0.322642544	1.41E-13	postive
LIAS	FGD5-AS1	0.424019232	3.08E-23	postive
LIAS	FLVCR1-DT	0.435814268	1.35E-24	postive
LIAS	FRG1HP	0.442760104	2.03E-25	postive
LIAS	GABPB1-AS1	0.327340372	5.95E-14	postive
LIAS	GAS5	0.536594761	1.26E-38	postive
LIAS	GCC2-AS1	0.42068323	7.30E-23	postive
LIAS	GLIDR	0.406562307	2.52E-21	postive
LIAS	GLIS2-AS1	0.398905465	1.61E-20	postive
LIAS	GMDS-DT	0.358987947	1.18E-16	postive

LIAS	GNRHR2	0.343493141	2.72E-15	postive
LIAS	GOLGA2P7	0.311690391	1.00E-12	postive
LIAS	GPRC5D-AS1	0.392608012	7.11E-20	postive
LIAS	GS1-124K5.3	0.428315591	1.00E-23	postive
LIAS	GSEC	0.330426854	3.35E-14	postive
LIAS	GTF2IP1	0.412442901	5.89E-22	postive
LIAS	GTF2IP20	0.311403136	1.05E-12	postive
LIAS	GTF2IRD2P1	0.387631822	2.25E-19	postive
LIAS	GUCY1B2	0.304933128	3.21E-12	postive
LIAS	GUSBP2	0.311988949	9.49E-13	postive
LIAS	H2AZ1-DT	0.323941171	1.11E-13	postive
LIAS	H2AZ2P1	0.347573782	1.21E-15	postive
LIAS	HCG15	0.329577907	3.92E-14	postive
LIAS	HCG18	0.407731086	1.89E-21	postive
LIAS	HCG25	0.470598791	6.40E-29	postive
LIAS	HEIH	0.400684605	1.05E-20	postive
LIAS	HERC2P3	0.368954628	1.43E-17	postive
LIAS	HERC2P9	0.32118638	1.84E-13	postive
LIAS	HIPK1-AS1	0.323841233	1.13E-13	postive
LIAS	HMGB1P5	0.410008005	1.08E-21	postive
LIAS	HSP90AB2P	0.324656429	9.77E-14	postive
LIAS	IDH1-AS1	0.358648136	1.27E-16	postive
LIAS	IL6R-AS1	0.382640119	7.02E-19	postive
LIAS	ILF3-DT	0.310196177	1.30E-12	postive
LIAS	INTS6-AS1	0.386993769	2.61E-19	postive
LIAS	IQCH-AS1	0.447417481	5.54E-26	postive
LIAS	ITFG2-AS1	0.473450357	2.68E-29	postive
LIAS	JPX	0.354920246	2.74E-16	postive
LIAS	KCNIP2-AS1	0.45092127	2.06E-26	postive
LIAS	KDM4A-AS1	0.411808409	6.90E-22	postive
LIAS	KMT2E-AS1	0.321460641	1.75E-13	postive
LIAS	KRT18P55	0.300568898	6.72E-12	postive
LIAS	KTN1-AS1	0.507596576	4.23E-34	postive
LIAS	LAMTOR5-AS1	0.369321044	1.33E-17	postive
LIAS	LGR4-AS1	0.340898258	4.52E-15	postive
LIAS	LIMD1-AS1	0.339885413	5.50E-15	postive
LIAS	LINC00106	0.361634899	6.80E-17	postive
LIAS	LINC00174	0.402668654	6.50E-21	postive
LIAS	LINC00205	0.385595535	3.59E-19	postive
LIAS	LINC00239	0.322895741	1.35E-13	postive
LIAS	LINC002481	0.450794715	2.14E-26	postive
LIAS	LINC00271	0.33696318	9.67E-15	postive
LIAS	LINC00324	0.354612466	2.92E-16	postive
LIAS	LINC00339	0.476411966	1.08E-29	postive
LIAS	LINC00467	0.584568234	3.71E-47	postive
LIAS	LINC00471	0.314409753	6.20E-13	postive
LIAS	LINC00476	0.384546705	4.55E-19	postive
LIAS	LINC00533	0.437847764	7.80E-25	postive
LIAS	LINC00638	0.311194027	1.09E-12	postive
LIAS	LINC00663	0.340134647	5.24E-15	postive

LIAS	LINC00665	0.341257002	4.21E-15	postive
LIAS	LINC00667	0.350903175	6.20E-16	postive
LIAS	LINC00680	0.338070782	7.82E-15	postive
LIAS	LINC00863	0.412310726	6.08E-22	postive
LIAS	LINC00886	0.345055064	2.00E-15	postive
LIAS	LINC00909	0.439756371	4.64E-25	postive
LIAS	LINC00910	0.390608101	1.13E-19	postive
LIAS	LINC00921	0.322148442	1.55E-13	postive
LIAS	LINC00933	0.397468704	2.26E-20	postive
LIAS	LINC00957	0.315492092	5.11E-13	postive
LIAS	LINC00963	0.437853544	7.79E-25	postive
LIAS	LINC00992	0.423557501	3.47E-23	postive
LIAS	LINC01001	0.320367646	2.14E-13	postive
LIAS	LINC01011	0.450933406	2.05E-26	postive
LIAS	LINC01058	0.319031067	2.72E-13	postive
LIAS	LINC01136	0.338715419	6.90E-15	postive
LIAS	LINC01137	0.428936449	8.50E-24	postive
LIAS	LINC01145	0.347420861	1.25E-15	postive
LIAS	LINC01184	0.378411482	1.81E-18	postive
LIAS	LINC01270	0.308396558	1.77E-12	postive
LIAS	LINC01273	0.355687923	2.34E-16	postive
LIAS	LINC01278	0.341524133	4.00E-15	postive
LIAS	LINC01301	0.300189606	7.16E-12	postive
LIAS	LINC01315	0.313200932	7.67E-13	postive
LIAS	LINC01347	0.320397413	2.12E-13	postive
LIAS	LINC01372	0.35790647	1.48E-16	postive
LIAS	LINC01431	0.304845992	3.26E-12	postive
LIAS	LINC01465	0.30108433	6.16E-12	postive
LIAS	LINC01508	0.39728854	2.36E-20	postive
LIAS	LINC01545	0.322329376	1.50E-13	postive
LIAS	LINC01556	0.309010765	1.59E-12	postive
LIAS	LINC01560	0.457012269	3.59E-27	postive
LIAS	LINC01569	0.336381766	1.08E-14	postive
LIAS	LINC01578	0.337137695	9.35E-15	postive
LIAS	LINC01589	0.31287554	8.12E-13	postive
LIAS	LINC01632	0.440301801	3.99E-25	postive
LIAS	LINC01635	0.385025739	4.08E-19	postive
LIAS	LINC01647	0.347315118	1.27E-15	postive
LIAS	LINC01687	0.374941471	3.90E-18	postive
LIAS	LINC01703	0.347846337	1.15E-15	postive
LIAS	LINC01715	0.351942798	5.02E-16	postive
LIAS	LINC01719	0.369185514	1.37E-17	postive
LIAS	LINC01770	0.321283817	1.81E-13	postive
LIAS	LINC01806	0.315032023	5.55E-13	postive
LIAS	LINC01816	0.417860806	1.50E-22	postive
LIAS	LINC01827	0.348744341	9.58E-16	postive
LIAS	LINC01881	0.362372914	5.82E-17	postive
LIAS	LINC01942	0.365759522	2.84E-17	postive
LIAS	LINC02147	0.358246373	1.38E-16	postive
LIAS	LINC02193	0.348152353	1.08E-15	postive

LIAS	LINC02298	0.405921845	2.95E-21	postive
LIAS	LINC02370	0.362034252	6.25E-17	postive
LIAS	LINC02405	0.305301021	3.02E-12	postive
LIAS	LINC02447	0.364181965	3.98E-17	postive
LIAS	LINC02453	0.379088359	1.56E-18	postive
LIAS	LINC02569	0.453014503	1.13E-26	postive
LIAS	LINC02585	0.345130279	1.97E-15	postive
LIAS	LINC02604	0.391730561	8.72E-20	postive
LIAS	LINC02637	0.304860257	3.25E-12	postive
LIAS	LINC02701	0.378267697	1.87E-18	postive
LIAS	LIX1L-AS1	0.308759983	1.66E-12	postive
LIAS	LOH12CR2	0.330129766	3.54E-14	postive
LIAS	LRRC37A17P	0.306083252	2.64E-12	postive
LIAS	LRRC37A5P	0.322019993	1.58E-13	postive
LIAS	LRRC37BP1	0.369004126	1.42E-17	postive
LIAS	MAGOH2P	0.307974753	1.91E-12	postive
LIAS	MAN1B1-DT	0.364323866	3.86E-17	postive
LIAS	MAP2K4P1	0.390843204	1.07E-19	postive
LIAS	MAP3K14-AS1	0.388691722	1.76E-19	postive
LIAS	MAP3K2-DT	0.31156627	1.02E-12	postive
LIAS	MAP4K3-DT	0.386814781	2.71E-19	postive
LIAS	MAPKAPK5-AS1	0.583758841	5.31E-47	postive
LIAS	MATN1-AS1	0.368550642	1.56E-17	postive
LIAS	MCM3AP-AS1	0.394902455	4.15E-20	postive
LIAS	MFF-DT	0.322470178	1.46E-13	postive
LIAS	MIATNB	0.353404645	3.73E-16	postive
LIAS	MIF-AS1	0.309090052	1.57E-12	postive
LIAS	MINCR	0.494485224	3.42E-32	postive
LIAS	MIR122HG	0.438429907	6.66E-25	postive
LIAS	MIR200CHG	0.327197629	6.11E-14	postive
LIAS	MIR22HG	0.370374536	1.06E-17	postive
LIAS	MIR3936HG	0.391525459	9.15E-20	postive
LIAS	MIR4435-2HG	0.327191686	6.12E-14	postive
LIAS	MIR762HG	0.353221409	3.87E-16	postive
LIAS	MKLN1-AS	0.447454697	5.49E-26	postive
LIAS	MMADHC-DT	0.307957201	1.91E-12	postive
LIAS	MRPL20-AS1	0.463103634	6.03E-28	postive
LIAS	MRPL45P2	0.509705889	2.05E-34	postive
LIAS	MRPS31P4	0.313685362	7.04E-13	postive
LIAS	MSH5-SAPCD1	0.462714583	6.77E-28	postive
LIAS	MYLK-AS1	0.33944049	6.00E-15	postive
LIAS	NACA4P	0.32903977	4.34E-14	postive
LIAS	NALT1	0.354774178	2.82E-16	postive
LIAS	NAPA-AS1	0.332043689	2.47E-14	postive
LIAS	NBPF8	0.37538955	3.53E-18	postive
LIAS	NBR2	0.399123928	1.52E-20	postive
LIAS	NCK1-DT	0.346964803	1.37E-15	postive
LIAS	NDUFA6-DT	0.331700403	2.63E-14	postive
LIAS	NDUFB2-AS1	0.354007179	3.30E-16	postive
LIAS	NIFK-AS1	0.464005644	4.62E-28	postive

LIAS	NOP14-AS1	0.455894892	4.96E-27	postive
LIAS	NORAD	0.34280573	3.11E-15	postive
LIAS	NRAV	0.413123335	4.96E-22	postive
LIAS	NRSN2-AS1	0.433461602	2.55E-24	postive
LIAS	NSMCE1-DT	0.312309378	8.97E-13	postive
LIAS	NSUN5P1	0.381721701	8.63E-19	postive
LIAS	NSUN5P2	0.399889542	1.27E-20	postive
LIAS	NUDT16P1	0.405058803	3.64E-21	postive
LIAS	NUTM2A-AS1	0.460246687	1.40E-27	postive
LIAS	OGFRP1	0.509222714	2.42E-34	postive
LIAS	OIP5-AS1	0.490231329	1.37E-31	postive
LIAS	OLMALINC	0.387645528	2.24E-19	postive
LIAS	OSER1-DT	0.411916514	6.71E-22	postive
LIAS	OSGEPL1-AS1	0.37867697	1.71E-18	postive
LIAS	OTUD6B-AS1	0.439522753	4.94E-25	postive
LIAS	PAQR9-AS1	0.435976982	1.30E-24	postive
LIAS	PARD6G-AS1	0.32423781	1.06E-13	postive
LIAS	PAXIP1-AS1	0.342252245	3.47E-15	postive
LIAS	PAXIP1-AS2	0.414043862	3.94E-22	postive
LIAS	PCBP1-AS1	0.371655327	8.00E-18	postive
LIAS	PDCD4-AS1	0.353541348	3.63E-16	postive
LIAS	PDIA3P1	0.38698579	2.61E-19	postive
LIAS	PIPSL	0.354803069	2.80E-16	postive
LIAS	PITPNA-AS1	0.489374928	1.80E-31	postive
LIAS	PLEKHA8P1	0.382233137	7.69E-19	postive
LIAS	PMS2CL	0.326732377	6.66E-14	postive
LIAS	PMS2P3	0.355882089	2.25E-16	postive
LIAS	PMS2P4	0.380866336	1.05E-18	postive
LIAS	POLH-AS1	0.318901272	2.78E-13	postive
LIAS	POLR2J4	0.335561592	1.27E-14	postive
LIAS	PPP1R12A-AS1	0.341976889	3.66E-15	postive
LIAS	PPP3CB-AS1	0.451227216	1.89E-26	postive
LIAS	PPT2-EGFL8	0.353823176	3.43E-16	postive
LIAS	PRELID1P1	0.426005084	1.84E-23	postive
LIAS	PRKAR2A-AS1	0.326222645	7.32E-14	postive
LIAS	PRKCQ-AS1	0.35293363	4.11E-16	postive
LIAS	PRMT5-AS1	0.378993563	1.59E-18	postive
LIAS	PRORSD1P	0.382403617	7.40E-19	postive
LIAS	PROSER2-AS1	0.332443426	2.29E-14	postive
LIAS	PRRT3-AS1	0.394508667	4.55E-20	postive
LIAS	PSMA3-AS1	0.3251247	8.97E-14	postive
LIAS	PTENP1	0.32744113	5.84E-14	postive
LIAS	PTOV1-AS1	0.323389489	1.23E-13	postive
LIAS	PXN-AS1	0.6635793	9.20E-65	postive
LIAS	RAB11B-AS1	0.389836267	1.35E-19	postive
LIAS	RAB30-DT	0.34998948	7.46E-16	postive
LIAS	RAD21-AS1	0.432955777	2.92E-24	postive
LIAS	RAD51-AS1	0.346299336	1.56E-15	postive
LIAS	RALY-AS1	0.347296049	1.28E-15	postive
LIAS	RAP2C-AS1	0.31982691	2.35E-13	postive

LIAS	RBM15-AS1	0.47520382	1.57E-29	postive
LIAS	RBM26-AS1	0.357152308	1.73E-16	postive
LIAS	RDM1P5	0.313660547	7.07E-13	postive
LIAS	RHPN1-AS1	0.304482428	3.47E-12	postive
LIAS	RNASEH1-AS1	0.33000615	3.62E-14	postive
LIAS	RNF216P1	0.340433428	4.94E-15	postive
LIAS	RNU6ATAC35P	0.311521918	1.03E-12	postive
LIAS	RP11-101E13.5	0.422971569	4.04E-23	postive
LIAS	RP11-10C24.1	0.328538746	4.76E-14	postive
LIAS	RP11-114N19.3	0.355343972	2.51E-16	postive
LIAS	RP11-153F5.7	0.350873548	6.24E-16	postive
LIAS	RP11-199F11.2	0.30116556	6.08E-12	postive
LIAS	RP11-226L15.5	0.342535158	3.28E-15	postive
LIAS	RP11-314C16.1	0.308359207	1.78E-12	postive
LIAS	RP11-392O1.4	0.455054563	6.32E-27	postive
LIAS	RP11-435O5.2	0.304116527	3.69E-12	postive
LIAS	RP11-539I5.1	0.357065067	1.76E-16	postive
LIAS	RP11-540B6.6	0.393703491	5.50E-20	postive
LIAS	RP11-658F2.8	0.300451892	6.86E-12	postive
LIAS	RP13-476E20.1	0.403532494	5.28E-21	postive
LIAS	RP1-90G24.6	0.321416724	1.77E-13	postive
LIAS	RP4-545L17.11	0.3454143	1.86E-15	postive
LIAS	RP5-1039K5.19	0.31713553	3.82E-13	postive
LIAS	RP5-1112D6.4	0.316923806	3.96E-13	postive
LIAS	RPARP-AS1	0.518791191	8.51E-36	postive
LIAS	RPL12P38	0.378819949	1.65E-18	postive
LIAS	RPL13AP3	0.395252549	3.82E-20	postive
LIAS	RPL13AP7	0.464036984	4.58E-28	postive
LIAS	RPL13P5	0.345488366	1.83E-15	postive
LIAS	RPL23AP53	0.396450625	2.88E-20	postive
LIAS	RPL23AP7	0.435643451	1.42E-24	postive
LIAS	RPL23AP82	0.533010818	4.81E-38	postive
LIAS	RPL29P2	0.313434726	7.36E-13	postive
LIAS	RPL29P30	0.376669682	2.66E-18	postive
LIAS	RPL32P3	0.308258877	1.82E-12	postive
LIAS	RPL36AP21	0.311756873	9.88E-13	postive
LIAS	RPL37P6	0.335707318	1.23E-14	postive
LIAS	RPL6P27	0.506431402	6.30E-34	postive
LIAS	RPLP1P6	0.331454425	2.76E-14	postive
LIAS	RPS18P9	0.386982093	2.61E-19	postive
LIAS	RPS27AP16	0.428599332	9.29E-24	postive
LIAS	RPS4XP16	0.429935013	6.53E-24	postive
LIAS	RPS4XP2	0.326808805	6.57E-14	postive
LIAS	RPSAP31	0.337244383	9.16E-15	postive
LIAS	RRN3P3	0.320004407	2.28E-13	postive
LIAS	RRP7BP	0.40583355	3.01E-21	postive
LIAS	RUSC1-AS1	0.310510387	1.23E-12	postive
LIAS	SAPCD1-AS1	0.501394098	3.46E-33	postive
LIAS	SBDSP1	0.481166578	2.46E-30	postive
LIAS	SBF2-AS1	0.304972361	3.19E-12	postive

LIAS	SBNO1-AS1	0.384278284	4.84E-19	postive
LIAS	SCAMP1-AS1	0.470549086	6.49E-29	postive
LIAS	SCAND2P	0.401223144	9.22E-21	postive
LIAS	SCOC-AS1	0.301733468	5.53E-12	postive
LIAS	SDCBP2-AS1	0.395101246	3.96E-20	postive
LIAS	SEC24B-AS1	0.396267307	3.01E-20	postive
LIAS	SEMA3F-AS1	0.307943374	1.92E-12	postive
LIAS	SEPSECS-AS1	0.417049969	1.84E-22	postive
LIAS	SEPTIN4-AS1	0.31642421	4.33E-13	postive
LIAS	SEPTIN7-AS1	0.380054141	1.25E-18	postive
LIAS	SEPTIN7P2	0.367315726	2.04E-17	postive
LIAS	SERHL	0.357862174	1.49E-16	postive
LIAS	SGMS1-AS1	0.405875202	2.98E-21	postive
LIAS	SLC12A9-AS1	0.575643821	1.83E-45	postive
LIAS	SLC44A3-AS1	0.364580441	3.65E-17	postive
LIAS	SLC5A4-AS1	0.33059245	3.24E-14	postive
LIAS	SMG7-AS1	0.440212624	4.09E-25	postive
LIAS	SMIM2-AS1	0.307484248	2.07E-12	postive
LIAS	SMPD5	0.333466757	1.89E-14	postive
LIAS	SNAI3-AS1	0.361021977	7.73E-17	postive
LIAS	SNHG1	0.4356702	1.41E-24	postive
LIAS	SNHG11	0.457142152	3.46E-27	postive
LIAS	SNHG12	0.370843655	9.54E-18	postive
LIAS	SNHG14	0.373627815	5.20E-18	postive
LIAS	SNHG15	0.338160267	7.68E-15	postive
LIAS	SNHG16	0.506184915	6.85E-34	postive
LIAS	SNHG17	0.36421323	3.95E-17	postive
LIAS	SNHG20	0.448406205	4.20E-26	postive
LIAS	SNHG21	0.517801739	1.21E-35	postive
LIAS	SNHG25	0.323828961	1.14E-13	postive
LIAS	SNHG28	0.35635714	2.04E-16	postive
LIAS	SNHG29	0.462813699	6.57E-28	postive
LIAS	SNHG3	0.40813788	1.71E-21	postive
LIAS	SNHG32	0.51361446	5.27E-35	postive
LIAS	SNHG4	0.517769185	1.22E-35	postive
LIAS	SNHG5	0.398507542	1.77E-20	postive
LIAS	SNHG6	0.389204987	1.57E-19	postive
LIAS	SNHG7	0.36471548	3.55E-17	postive
LIAS	SNHG8	0.596627185	1.56E-49	postive
LIAS	SP2-AS1	0.327192896	6.12E-14	postive
LIAS	SPAG5-AS1	0.393195351	6.19E-20	postive
LIAS	SPINT1-AS1	0.355524227	2.42E-16	postive
LIAS	SREBF2-AS1	0.351123259	5.93E-16	postive
LIAS	SRP14-AS1	0.618373815	4.46E-54	postive
LIAS	SSR4P1	0.378524049	1.77E-18	postive
LIAS	ST20-AS1	0.316032511	4.65E-13	postive
LIAS	STAG3L1	0.354108963	3.23E-16	postive
LIAS	STAG3L2	0.40591626	2.95E-21	postive
LIAS	STAG3L3	0.390300266	1.22E-19	postive
LIAS	STAG3L4	0.402731601	6.41E-21	postive

LIAS	STAG3L5P	0.350435813	6.81E-16	postive
LIAS	STAG3L5P-PVRIG2P-PILRB	0.319129995	2.67E-13	postive
LIAS	STAM-AS1	0.328320368	4.96E-14	postive
LIAS	STARD7-AS1	0.458228466	2.52E-27	postive
LIAS	STK4-AS1	0.430986922	4.94E-24	postive
LIAS	STX17-AS1	0.501932402	2.89E-33	postive
LIAS	STX18-AS1	0.47189313	4.32E-29	postive
LIAS	SUCLA2-AS1	0.415978544	2.42E-22	postive
LIAS	SUCLG2-AS1	0.306825204	2.32E-12	postive
LIAS	SUMO2P17	0.419098946	1.09E-22	postive
LIAS	SVIL-AS1	0.341677126	3.88E-15	postive
LIAS	TAF1A-AS1	0.418776108	1.19E-22	postive
LIAS	TAPT1-AS1	0.491204097	9.98E-32	postive
LIAS	TBC1D8-AS1	0.38187509	8.34E-19	postive
LIAS	TESC-AS1	0.316726955	4.11E-13	postive
LIAS	THAP7-AS1	0.300457275	6.85E-12	postive
LIAS	THAP9-AS1	0.393980238	5.15E-20	postive
LIAS	THRB-AS1	0.481692616	2.08E-30	postive
LIAS	TIPARP-AS1	0.465465592	2.99E-28	postive
LIAS	TMC3-AS1	0.374161446	4.63E-18	postive
LIAS	TMEM147-AS1	0.369529158	1.27E-17	postive
LIAS	TMEM161B-AS1	0.447296776	5.73E-26	postive
LIAS	TMEM198B	0.362491437	5.68E-17	postive
LIAS	TMEM202-AS1	0.341341398	4.14E-15	postive
LIAS	TMEM254-AS1	0.322029623	1.58E-13	postive
LIAS	TMEM44-AS1	0.312920876	8.06E-13	postive
LIAS	TMEM99	0.411743207	7.01E-22	postive
LIAS	TMEM9B-AS1	0.363299329	4.79E-17	postive
LIAS	TOB1-AS1	0.461331843	1.02E-27	postive
LIAS	TOLLIP-AS1	0.308306973	1.80E-12	postive
LIAS	TP53TG1	0.354967757	2.71E-16	postive
LIAS	TP73-AS1	0.340101519	5.27E-15	postive
LIAS	TPM3P9	0.378447703	1.80E-18	postive
LIAS	TPT1-AS1	0.343024982	2.98E-15	postive
LIAS	TRAM2-AS1	0.501160768	3.74E-33	postive
LIAS	TRIM31-AS1	0.323599519	1.19E-13	postive
LIAS	TRIM52-AS1	0.438814338	6.00E-25	postive
LIAS	TSTD3	0.527836447	3.25E-37	postive
LIAS	TTC28-AS1	0.390343461	1.20E-19	postive
LIAS	TTC41P	0.340165111	5.21E-15	postive
LIAS	TUBBP1	0.30166553	5.59E-12	postive
LIAS	TUG1	0.32967354	3.85E-14	postive
LIAS	U47924.2	0.339184702	6.30E-15	postive
LIAS	U91328.1	0.382387973	7.43E-19	postive
LIAS	UBA6-AS1	0.445020317	1.08E-25	postive
LIAS	UBAC2-AS1	0.376778838	2.60E-18	postive
LIAS	UBE2D3-AS1	0.544204881	6.86E-40	postive
LIAS	UBE2Q2P2	0.301455305	5.79E-12	postive
LIAS	UBL7-AS1	0.375019198	3.83E-18	postive
LIAS	UBR5-AS1	0.437734426	8.05E-25	postive

LIAS	UGDH-AS1	0.554869172	1.03E-41	postive
LIAS	UPF3AP2	0.328989928	4.38E-14	postive
LIAS	UPK1A-AS1	0.443979407	1.45E-25	postive
LIAS	URAHP	0.303033276	4.44E-12	postive
LIAS	URB1-AS1	0.306509661	2.45E-12	postive
LIAS	USP27X-AS1	0.333839822	1.76E-14	postive
LIAS	USP46-AS1	0.328701974	4.62E-14	postive
LIAS	UXT-AS1	0.478824735	5.11E-30	postive
LIAS	VASH1-AS1	0.361084706	7.63E-17	postive
LIAS	VDAC1P8	0.33535086	1.32E-14	postive
LIAS	VPS13B-DT	0.376625059	2.69E-18	postive
LIAS	VPS33B-DT	0.407837138	1.84E-21	postive
LIAS	VPS9D1-AS1	0.377900538	2.03E-18	postive
LIAS	WAC-AS1	0.415391293	2.81E-22	postive
LIAS	WARS2-AS1	0.437945268	7.60E-25	postive
LIAS	WASH3P	0.450186236	2.54E-26	postive
LIAS	WASH6P	0.304900623	3.23E-12	postive
LIAS	WDFY3-AS2	0.390854591	1.07E-19	postive
LIAS	WEE2-AS1	0.4001003	1.21E-20	postive
LIAS	WHAMMP3	0.319417908	2.53E-13	postive
LIAS	WWC2-AS2	0.317011953	3.90E-13	postive
LIAS	Z83851.4	0.528708448	2.36E-37	postive
LIAS	Z84485.1	0.387668459	2.23E-19	postive
LIAS	Z97055.2	0.338467472	7.24E-15	postive
LIAS	Z97634.1	0.321412247	1.77E-13	postive
LIAS	ZBED5-AS1	0.381921814	8.25E-19	postive
LIAS	ZBTB11-AS1	0.389920302	1.33E-19	postive
LIAS	ZBTB44-DT	0.470610341	6.37E-29	postive
LIAS	ZFAS1	0.504265965	1.31E-33	postive
LIAS	ZNF205-AS1	0.369833564	1.19E-17	postive
LIAS	ZNF213-AS1	0.381763811	8.55E-19	postive
LIAS	ZNF252P	0.380669531	1.09E-18	postive
LIAS	ZNF252P-AS1	0.33846292	7.25E-15	postive
LIAS	ZNF337-AS1	0.492720763	6.10E-32	postive
LIAS	ZNF436-AS1	0.317317216	3.69E-13	postive
LIAS	ZNF460-AS1	0.309993377	1.34E-12	postive
LIAS	ZNF503-AS2	0.309218937	1.54E-12	postive
LIAS	ZNF56	0.360567429	8.51E-17	postive
LIAS	ZNF561-AS1	0.386533463	2.90E-19	postive
LIAS	ZNF658B	0.367176067	2.10E-17	postive
LIAS	ZNF687-AS1	0.389510889	1.46E-19	postive
LIAS	ZNF767P	0.331761034	2.60E-14	postive
LIAS	ZNRD1ASP	0.390032874	1.29E-19	postive
LIAS	ZNRD2-AS1	0.323845898	1.13E-13	postive
LIAS	ZSCAN12P1	0.306368004	2.51E-12	postive
LIAS	ZSCAN16-AS1	0.448152082	4.51E-26	postive
LIPT1	A1BG-AS1	0.327305232	5.99E-14	postive
LIPT1	A2M-AS1	0.406251545	2.72E-21	postive
LIPT1	ABCA11P	0.392066695	8.06E-20	postive
LIPT1	ABCC6P2	0.349937246	7.53E-16	postive

LIPT1	AC000068.1	0.369910606	1.17E-17	postive
LIPT1	AC000068.2	0.320476688	2.09E-13	postive
LIPT1	AC002091.2	0.307307284	2.14E-12	postive
LIPT1	AC002116.2	0.395049638	4.01E-20	postive
LIPT1	AC002310.1	0.450895357	2.08E-26	postive
LIPT1	AC002467.1	0.496637576	1.69E-32	postive
LIPT1	AC002470.1	0.320655184	2.03E-13	postive
LIPT1	AC002550.2	0.314024216	6.63E-13	postive
LIPT1	AC003102.1	0.329045193	4.34E-14	postive
LIPT1	AC003956.1	0.317898046	3.33E-13	postive
LIPT1	AC003965.1	0.319949206	2.30E-13	postive
LIPT1	AC004148.1	0.306470147	2.47E-12	postive
LIPT1	AC004241.1	0.548344009	1.37E-40	postive
LIPT1	AC004241.2	0.406291041	2.69E-21	postive
LIPT1	AC004241.3	0.336688743	1.02E-14	postive
LIPT1	AC004253.1	0.316547431	4.24E-13	postive
LIPT1	AC004447.2	0.317696531	3.45E-13	postive
LIPT1	AC004471.2	0.317956692	3.30E-13	postive
LIPT1	AC004477.1	0.41434008	3.66E-22	postive
LIPT1	AC004477.3	0.306605301	2.41E-12	postive
LIPT1	AC004596.1	0.443722919	1.55E-25	postive
LIPT1	AC004771.2	0.474626889	1.87E-29	postive
LIPT1	AC004771.4	0.333138953	2.01E-14	postive
LIPT1	AC004801.6	0.371002689	9.22E-18	postive
LIPT1	AC004803.1	0.377550563	2.19E-18	postive
LIPT1	AC004812.2	0.444196951	1.36E-25	postive
LIPT1	AC004825.2	0.361369288	7.19E-17	postive
LIPT1	AC004846.1	0.33006696	3.58E-14	postive
LIPT1	AC004865.2	0.34640164	1.53E-15	postive
LIPT1	AC004893.11	0.371636095	8.03E-18	postive
LIPT1	AC004921.1	0.318360733	3.06E-13	postive
LIPT1	AC004943.2	0.399889931	1.27E-20	postive
LIPT1	AC004951.1	0.326886599	6.48E-14	postive
LIPT1	AC004980.1	0.316834645	4.03E-13	postive
LIPT1	AC004982.2	0.30025674	7.08E-12	postive
LIPT1	AC005005.3	0.377937027	2.01E-18	postive
LIPT1	AC005005.4	0.304932395	3.21E-12	postive
LIPT1	AC005034.4	0.407499722	2.00E-21	postive
LIPT1	AC005034.6	0.411433313	7.57E-22	postive
LIPT1	AC005037.1	0.329576115	3.93E-14	postive
LIPT1	AC005041.3	0.351819667	5.15E-16	postive
LIPT1	AC005076.2	0.429626794	7.08E-24	postive
LIPT1	AC005083.1	0.340765851	4.63E-15	postive
LIPT1	AC005091.1	0.31018292	1.30E-12	postive
LIPT1	AC005104.1	0.306292303	2.55E-12	postive
LIPT1	AC005225.3	0.303129751	4.37E-12	postive
LIPT1	AC005229.4	0.385162966	3.96E-19	postive
LIPT1	AC005253.1	0.376649202	2.68E-18	postive
LIPT1	AC005280.1	0.366586699	2.38E-17	postive
LIPT1	AC005288.1	0.422532496	4.53E-23	postive

LIPT1	AC005324.5	0.350344424	6.94E-16	postive
LIPT1	AC005332.1	0.349146061	8.83E-16	postive
LIPT1	AC005332.4	0.386545281	2.89E-19	postive
LIPT1	AC005332.6	0.323096517	1.30E-13	postive
LIPT1	AC005387.1	0.321651931	1.69E-13	postive
LIPT1	AC005387.2	0.350785025	6.35E-16	postive
LIPT1	AC005476.2	0.300512497	6.79E-12	postive
LIPT1	AC005498.2	0.334977989	1.41E-14	postive
LIPT1	AC005498.3	0.370035717	1.14E-17	postive
LIPT1	AC005519.1	0.325721028	8.03E-14	postive
LIPT1	AC005520.4	0.477470051	7.78E-30	postive
LIPT1	AC005562.1	0.468356498	1.26E-28	postive
LIPT1	AC005586.1	0.3313119	2.83E-14	postive
LIPT1	AC005670.3	0.467854283	1.46E-28	postive
LIPT1	AC005674.1	0.305653863	2.84E-12	postive
LIPT1	AC005696.1	0.34990943	7.58E-16	postive
LIPT1	AC005837.1	0.463786638	4.93E-28	postive
LIPT1	AC005840.4	0.31522158	5.37E-13	postive
LIPT1	AC006001.2	0.406815612	2.37E-21	postive
LIPT1	AC006001.3	0.315761419	4.88E-13	postive
LIPT1	AC006026.3	0.307143361	2.20E-12	postive
LIPT1	AC006064.2	0.303660961	3.99E-12	postive
LIPT1	AC006111.2	0.302295028	5.03E-12	postive
LIPT1	AC006122.1	0.302163524	5.14E-12	postive
LIPT1	AC006213.1	0.460568598	1.27E-27	postive
LIPT1	AC006252.1	0.435950526	1.31E-24	postive
LIPT1	AC006333.1	0.445944669	8.37E-26	postive
LIPT1	AC006449.2	0.456385996	4.30E-27	postive
LIPT1	AC006449.5	0.317035037	3.89E-13	postive
LIPT1	AC006449.7	0.497353255	1.33E-32	postive
LIPT1	AC006504.5	0.391327312	9.58E-20	postive
LIPT1	AC006547.1	0.359567693	1.05E-16	postive
LIPT1	AC007032.1	0.303191609	4.32E-12	postive
LIPT1	AC007038.1	0.319610051	2.45E-13	postive
LIPT1	AC007066.2	0.405602547	3.19E-21	postive
LIPT1	AC007114.1	0.395268867	3.81E-20	postive
LIPT1	AC007228.9	0.318280294	3.11E-13	postive
LIPT1	AC007292.1	0.331144055	2.93E-14	postive
LIPT1	AC007292.2	0.333639459	1.83E-14	postive
LIPT1	AC007318.3	0.364704252	3.56E-17	postive
LIPT1	AC007342.4	0.368169211	1.70E-17	postive
LIPT1	AC007383.2	0.477289474	8.23E-30	postive
LIPT1	AC007406.5	0.344179406	2.37E-15	postive
LIPT1	AC007541.1	0.339856684	5.53E-15	postive
LIPT1	AC007566.1	0.344024517	2.45E-15	postive
LIPT1	AC007663.4	0.37574954	3.26E-18	postive
LIPT1	AC007686.3	0.337859391	8.14E-15	postive
LIPT1	AC007881.3	0.321665322	1.69E-13	postive
LIPT1	AC008014.1	0.428248622	1.02E-23	postive
LIPT1	AC008035.1	0.314536529	6.06E-13	postive

LIPT1	AC008105.2	0.399269421	1.47E-20	postive
LIPT1	AC008124.1	0.45406537	8.40E-27	postive
LIPT1	AC008267.5	0.435271454	1.57E-24	postive
LIPT1	AC008280.3	0.456615894	4.03E-27	postive
LIPT1	AC008393.1	0.431044163	4.86E-24	postive
LIPT1	AC008438.1	0.509966028	1.87E-34	postive
LIPT1	AC008443.1	0.49257114	6.40E-32	postive
LIPT1	AC008443.5	0.317458712	3.60E-13	postive
LIPT1	AC008494.3	0.430293753	5.94E-24	postive
LIPT1	AC008608.2	0.34812338	1.08E-15	postive
LIPT1	AC008610.1	0.323171058	1.28E-13	postive
LIPT1	AC008622.2	0.30789304	1.93E-12	postive
LIPT1	AC008629.1	0.337133718	9.36E-15	postive
LIPT1	AC008655.2	0.323593525	1.19E-13	postive
LIPT1	AC008669.1	0.323230157	1.27E-13	postive
LIPT1	AC008735.1	0.315635467	4.99E-13	postive
LIPT1	AC008735.4	0.320309731	2.16E-13	postive
LIPT1	AC008758.4	0.337150341	9.33E-15	postive
LIPT1	AC008760.1	0.322997031	1.32E-13	postive
LIPT1	AC008764.2	0.388127443	2.01E-19	postive
LIPT1	AC008764.6	0.357086476	1.75E-16	postive
LIPT1	AC008764.8	0.315345992	5.25E-13	postive
LIPT1	AC008771.1	0.472334311	3.77E-29	postive
LIPT1	AC008781.1	0.311122046	1.10E-12	postive
LIPT1	AC008870.3	0.305115505	3.11E-12	postive
LIPT1	AC008870.5	0.302955618	4.50E-12	postive
LIPT1	AC008915.3	0.512933475	6.69E-35	postive
LIPT1	AC008946.1	0.395891076	3.29E-20	postive
LIPT1	AC008966.1	0.533091709	4.67E-38	postive
LIPT1	AC008969.1	0.339892784	5.49E-15	postive
LIPT1	AC009019.1	0.310140388	1.31E-12	postive
LIPT1	AC009041.3	0.330821184	3.11E-14	postive
LIPT1	AC009053.3	0.302954921	4.50E-12	postive
LIPT1	AC009061.2	0.456229231	4.50E-27	postive
LIPT1	AC009065.5	0.355570071	2.40E-16	postive
LIPT1	AC009065.9	0.398229796	1.89E-20	postive
LIPT1	AC009087.1	0.308029178	1.89E-12	postive
LIPT1	AC009107.2	0.367707941	1.88E-17	postive
LIPT1	AC009113.1	0.453318115	1.04E-26	postive
LIPT1	AC009118.3	0.322339041	1.49E-13	postive
LIPT1	AC009121.4	0.306530508	2.44E-12	postive
LIPT1	AC009126.1	0.480848766	2.71E-30	postive
LIPT1	AC009163.7	0.377906372	2.03E-18	postive
LIPT1	AC009171.2	0.353004458	4.05E-16	postive
LIPT1	AC009226.1	0.325780047	7.95E-14	postive
LIPT1	AC009237.14	0.5283208	2.73E-37	postive
LIPT1	AC009309.2	0.464279188	4.26E-28	postive
LIPT1	AC009403.1	0.462828509	6.54E-28	postive
LIPT1	AC009404.1	0.342305539	3.43E-15	postive
LIPT1	AC009495.3	0.35903117	1.17E-16	postive

LIPT1	AC009506.2	0.583108924	7.07E-47	postive
LIPT1	AC009690.2	0.414870916	3.20E-22	postive
LIPT1	AC009779.3	0.526250853	5.81E-37	postive
LIPT1	AC009831.1	0.392025423	8.14E-20	postive
LIPT1	AC009948.3	0.318075042	3.23E-13	postive
LIPT1	AC009961.1	0.432796396	3.05E-24	postive
LIPT1	AC010186.2	0.354463681	3.01E-16	postive
LIPT1	AC010226.1	0.394370347	4.70E-20	postive
LIPT1	AC010273.2	0.324736609	9.63E-14	postive
LIPT1	AC010319.4	0.399715856	1.32E-20	postive
LIPT1	AC010320.2	0.338617854	7.03E-15	postive
LIPT1	AC010359.2	0.343799652	2.56E-15	postive
LIPT1	AC010422.1	0.34752099	1.22E-15	postive
LIPT1	AC010422.2	0.302384244	4.95E-12	postive
LIPT1	AC010442.1	0.374763441	4.06E-18	postive
LIPT1	AC010504.1	0.411684178	7.11E-22	postive
LIPT1	AC010618.3	0.388277705	1.94E-19	postive
LIPT1	AC010643.1	0.382139291	7.85E-19	postive
LIPT1	AC010761.1	0.343597851	2.66E-15	postive
LIPT1	AC010761.4	0.331756339	2.61E-14	postive
LIPT1	AC010761.6	0.339171601	6.32E-15	postive
LIPT1	AC010864.1	0.313866907	6.82E-13	postive
LIPT1	AC010883.1	0.335713807	1.23E-14	postive
LIPT1	AC010894.2	0.421817467	5.45E-23	postive
LIPT1	AC010969.2	0.445301876	1.00E-25	postive
LIPT1	AC010997.4	0.308704238	1.68E-12	postive
LIPT1	AC011374.2	0.324842336	9.44E-14	postive
LIPT1	AC011450.1	0.362114983	6.15E-17	postive
LIPT1	AC011451.1	0.354728064	2.85E-16	postive
LIPT1	AC011466.3	0.300931531	6.33E-12	postive
LIPT1	AC011468.5	0.364161909	3.99E-17	postive
LIPT1	AC011477.2	0.407229547	2.14E-21	postive
LIPT1	AC011481.1	0.343562135	2.68E-15	postive
LIPT1	AC011511.1	0.32226226	1.51E-13	postive
LIPT1	AC011511.2	0.301530304	5.72E-12	postive
LIPT1	AC011603.3	0.345869229	1.70E-15	postive
LIPT1	AC011731.1	0.349335832	8.50E-16	postive
LIPT1	AC011921.1	0.398531981	1.76E-20	postive
LIPT1	AC011978.2	0.300035638	7.35E-12	postive
LIPT1	AC012063.1	0.483930364	1.03E-30	postive
LIPT1	AC012073.1	0.34474291	2.12E-15	postive
LIPT1	AC012146.1	0.435721475	1.39E-24	postive
LIPT1	AC012184.3	0.34839382	1.03E-15	postive
LIPT1	AC012306.2	0.43714486	9.45E-25	postive
LIPT1	AC012313.1	0.361763603	6.62E-17	postive
LIPT1	AC012313.5	0.364155197	4.00E-17	postive
LIPT1	AC012313.9	0.309755967	1.40E-12	postive
LIPT1	AC012360.3	0.454226103	8.02E-27	postive
LIPT1	AC012435.3	0.319777422	2.38E-13	postive
LIPT1	AC012442.2	0.396746292	2.68E-20	postive

LIPT1	AC012442.3	0.398212123	1.89E-20	postive
LIPT1	AC012467.2	0.492646254	6.25E-32	postive
LIPT1	AC012510.1	0.313300394	7.54E-13	postive
LIPT1	AC012555.1	0.325845087	7.85E-14	postive
LIPT1	AC012603.1	0.325443363	8.45E-14	postive
LIPT1	AC012615.1	0.406528999	2.54E-21	postive
LIPT1	AC012640.4	0.413251232	4.81E-22	postive
LIPT1	AC012676.1	0.39862074	1.72E-20	postive
LIPT1	AC013468.1	0.434579416	1.89E-24	postive
LIPT1	AC015660.3	0.303193371	4.32E-12	postive
LIPT1	AC015726.1	0.300039806	7.35E-12	postive
LIPT1	AC015802.4	0.428423539	9.74E-24	postive
LIPT1	AC015802.5	0.362130547	6.13E-17	postive
LIPT1	AC015871.3	0.314608876	5.98E-13	postive
LIPT1	AC015917.2	0.416784309	1.97E-22	postive
LIPT1	AC015922.2	0.382159618	7.82E-19	postive
LIPT1	AC015922.3	0.41018778	1.03E-21	postive
LIPT1	AC015982.2	0.500843156	4.16E-33	postive
LIPT1	AC016027.1	0.33053394	3.28E-14	postive
LIPT1	AC016065.1	0.380503042	1.13E-18	postive
LIPT1	AC016355.1	0.414626136	3.40E-22	postive
LIPT1	AC016394.2	0.517409637	1.39E-35	postive
LIPT1	AC016396.1	0.30294923	4.50E-12	postive
LIPT1	AC016405.3	0.327459519	5.82E-14	postive
LIPT1	AC016526.2	0.324738617	9.63E-14	postive
LIPT1	AC016590.3	0.304393121	3.52E-12	postive
LIPT1	AC016722.2	0.314705198	5.88E-13	postive
LIPT1	AC016727.1	0.527008667	4.41E-37	postive
LIPT1	AC016737.1	0.3134639	7.32E-13	postive
LIPT1	AC016747.1	0.444543611	1.24E-25	postive
LIPT1	AC016773.2	0.306168557	2.60E-12	postive
LIPT1	AC016876.2	0.388091318	2.03E-19	postive
LIPT1	AC016876.3	0.405043588	3.65E-21	postive
LIPT1	AC016888.1	0.329989138	3.63E-14	postive
LIPT1	AC017083.1	0.360757583	8.17E-17	postive
LIPT1	AC018413.1	0.301676449	5.58E-12	postive
LIPT1	AC018521.5	0.533494362	4.02E-38	postive
LIPT1	AC018521.6	0.399649937	1.34E-20	postive
LIPT1	AC018647.2	0.402408663	6.93E-21	postive
LIPT1	AC018653.3	0.348444648	1.02E-15	postive
LIPT1	AC018690.1	0.439333694	5.20E-25	postive
LIPT1	AC018809.2	0.444109288	1.40E-25	postive
LIPT1	AC018845.3	0.344722084	2.13E-15	postive
LIPT1	AC019131.1	0.328311612	4.97E-14	postive
LIPT1	AC019131.2	0.369890778	1.17E-17	postive
LIPT1	AC019205.1	0.394806801	4.24E-20	postive
LIPT1	AC019294.2	0.427063752	1.39E-23	postive
LIPT1	AC020558.2	0.317194503	3.78E-13	postive
LIPT1	AC020663.2	0.375945431	3.13E-18	postive
LIPT1	AC020910.5	0.322517996	1.45E-13	postive

LIPT1	AC020915.3	0.502374385	2.49E-33	postive
LIPT1	AC021016.2	0.345935336	1.68E-15	postive
LIPT1	AC021087.1	0.319461676	2.51E-13	postive
LIPT1	AC021148.1	0.438496303	6.54E-25	postive
LIPT1	AC021483.2	0.317150778	3.81E-13	postive
LIPT1	AC021752.1	0.355722939	2.32E-16	postive
LIPT1	AC021755.2	0.352299167	4.67E-16	postive
LIPT1	AC021851.1	0.365359907	3.10E-17	postive
LIPT1	AC022007.1	0.33855284	7.12E-15	postive
LIPT1	AC022098.1	0.401491575	8.64E-21	postive
LIPT1	AC022098.3	0.302893991	4.54E-12	postive
LIPT1	AC022101.1	0.367960162	1.78E-17	postive
LIPT1	AC022150.2	0.420273394	8.11E-23	postive
LIPT1	AC022167.2	0.404577699	4.09E-21	postive
LIPT1	AC022167.3	0.332511422	2.26E-14	postive
LIPT1	AC022167.4	0.382915153	6.59E-19	postive
LIPT1	AC022211.3	0.426285514	1.71E-23	postive
LIPT1	AC022217.4	0.319531345	2.48E-13	postive
LIPT1	AC022306.2	0.306375993	2.51E-12	postive
LIPT1	AC022364.1	0.357572021	1.59E-16	postive
LIPT1	AC022400.1	0.319787873	2.37E-13	postive
LIPT1	AC022400.5	0.308436423	1.76E-12	postive
LIPT1	AC022400.6	0.304251964	3.61E-12	postive
LIPT1	AC022415.2	0.321454702	1.75E-13	postive
LIPT1	AC022613.2	0.377277675	2.33E-18	postive
LIPT1	AC022762.2	0.303644476	4.00E-12	postive
LIPT1	AC022916.2	0.435181217	1.61E-24	postive
LIPT1	AC023043.1	0.347380767	1.26E-15	postive
LIPT1	AC023509.3	0.387434873	2.35E-19	postive
LIPT1	AC023632.2	0.387948907	2.09E-19	postive
LIPT1	AC023790.2	0.318370937	3.06E-13	postive
LIPT1	AC023908.3	0.348019807	1.11E-15	postive
LIPT1	AC024060.2	0.370911724	9.40E-18	postive
LIPT1	AC024075.1	0.351918567	5.05E-16	postive
LIPT1	AC024075.2	0.373133819	5.80E-18	postive
LIPT1	AC024075.3	0.342726328	3.16E-15	postive
LIPT1	AC024243.1	0.342834596	3.09E-15	postive
LIPT1	AC024361.2	0.386549697	2.88E-19	postive
LIPT1	AC024575.2	0.488395872	2.47E-31	postive
LIPT1	AC024896.1	0.340558162	4.83E-15	postive
LIPT1	AC024941.2	0.376438924	2.80E-18	postive
LIPT1	AC025048.2	0.414161616	3.83E-22	postive
LIPT1	AC025048.4	0.399206835	1.49E-20	postive
LIPT1	AC025171.1	0.411747231	7.00E-22	postive
LIPT1	AC025171.2	0.319922504	2.31E-13	postive
LIPT1	AC025181.2	0.338783434	6.81E-15	postive
LIPT1	AC025262.1	0.358850803	1.22E-16	postive
LIPT1	AC025265.1	0.304910475	3.23E-12	postive
LIPT1	AC025682.2	0.448984799	3.57E-26	postive
LIPT1	AC026191.1	0.328907049	4.45E-14	postive

LIPT1	AC026202.2	0.356474897	1.99E-16	postive
LIPT1	AC026304.1	0.373240129	5.66E-18	postive
LIPT1	AC026367.2	0.30472264	3.33E-12	postive
LIPT1	AC026367.3	0.360159124	9.26E-17	postive
LIPT1	AC026412.1	0.303039062	4.43E-12	postive
LIPT1	AC026458.2	0.332235795	2.38E-14	postive
LIPT1	AC026471.1	0.442918912	1.94E-25	postive
LIPT1	AC026471.2	0.332804164	2.14E-14	postive
LIPT1	AC026691.1	0.425401009	2.15E-23	postive
LIPT1	AC026801.2	0.395401879	3.69E-20	postive
LIPT1	AC026803.2	0.314990029	5.59E-13	postive
LIPT1	AC027097.1	0.413888568	4.10E-22	postive
LIPT1	AC027097.2	0.358813358	1.23E-16	postive
LIPT1	AC027237.4	0.373734977	5.08E-18	postive
LIPT1	AC027559.1	0.344338475	2.30E-15	postive
LIPT1	AC027601.2	0.342126146	3.55E-15	postive
LIPT1	AC027644.3	0.351224983	5.81E-16	postive
LIPT1	AC034102.5	0.328614927	4.70E-14	postive
LIPT1	AC034231.1	0.325658481	8.13E-14	postive
LIPT1	AC034236.2	0.428872784	8.65E-24	postive
LIPT1	AC036176.1	0.436531463	1.12E-24	postive
LIPT1	AC037459.2	0.316927147	3.96E-13	postive
LIPT1	AC040970.1	0.366596541	2.38E-17	postive
LIPT1	AC040977.1	0.399736571	1.32E-20	postive
LIPT1	AC046134.2	0.49149725	9.08E-32	postive
LIPT1	AC048351.2	0.329893283	3.70E-14	postive
LIPT1	AC048382.5	0.337527493	8.68E-15	postive
LIPT1	AC051619.4	0.345786649	1.73E-15	postive
LIPT1	AC051619.6	0.367231772	2.08E-17	postive
LIPT1	AC053503.1	0.381466162	9.14E-19	postive
LIPT1	AC053503.4	0.309852997	1.38E-12	postive
LIPT1	AC055720.2	0.326547462	6.90E-14	postive
LIPT1	AC055855.2	0.309089513	1.57E-12	postive
LIPT1	AC060766.4	0.304199479	3.64E-12	postive
LIPT1	AC060766.7	0.403426114	5.41E-21	postive
LIPT1	AC060780.1	0.501454958	3.39E-33	postive
LIPT1	AC063943.2	0.310637253	1.20E-12	postive
LIPT1	AC064807.1	0.456092643	4.68E-27	postive
LIPT1	AC064836.3	0.395397281	3.69E-20	postive
LIPT1	AC067750.1	0.349300318	8.56E-16	postive
LIPT1	AC067852.2	0.555174128	9.10E-42	postive
LIPT1	AC067930.3	0.313747601	6.96E-13	postive
LIPT1	AC068338.2	0.518002432	1.13E-35	postive
LIPT1	AC068338.3	0.337289018	9.09E-15	postive
LIPT1	AC068473.5	0.432652994	3.17E-24	postive
LIPT1	AC068580.3	0.3361363	1.13E-14	postive
LIPT1	AC068620.1	0.421317766	6.20E-23	postive
LIPT1	AC068620.2	0.332273771	2.36E-14	postive
LIPT1	AC068790.8	0.306151284	2.61E-12	postive
LIPT1	AC068831.2	0.42493348	2.43E-23	postive

LIPT1	AC068831.3	0.304290009	3.58E-12	postive
LIPT1	AC068870.2	0.410479441	9.60E-22	postive
LIPT1	AC068888.1	0.366319414	2.52E-17	postive
LIPT1	AC069185.1	0.348177807	1.07E-15	postive
LIPT1	AC069224.2	0.3684565	1.60E-17	postive
LIPT1	AC069307.1	0.359999259	9.58E-17	postive
LIPT1	AC072061.1	0.340107819	5.27E-15	postive
LIPT1	AC073046.1	0.317289795	3.71E-13	postive
LIPT1	AC073073.2	0.356800273	1.86E-16	postive
LIPT1	AC073195.2	0.407691524	1.91E-21	postive
LIPT1	AC073254.1	0.448612109	3.96E-26	postive
LIPT1	AC073332.1	0.319235397	2.62E-13	postive
LIPT1	AC073508.3	0.408681624	1.50E-21	postive
LIPT1	AC073529.1	0.371625746	8.05E-18	postive
LIPT1	AC073569.2	0.361294903	7.30E-17	postive
LIPT1	AC073575.4	0.365953779	2.73E-17	postive
LIPT1	AC073611.1	0.389613277	1.43E-19	postive
LIPT1	AC073655.2	0.348838452	9.40E-16	postive
LIPT1	AC073842.1	0.304888632	3.24E-12	postive
LIPT1	AC073842.2	0.328549347	4.76E-14	postive
LIPT1	AC073869.1	0.397336618	2.33E-20	postive
LIPT1	AC073896.2	0.509836535	1.96E-34	postive
LIPT1	AC073896.3	0.447709415	5.11E-26	postive
LIPT1	AC074029.3	0.346779079	1.42E-15	postive
LIPT1	AC074044.1	0.347623955	1.20E-15	postive
LIPT1	AC074117.1	0.429944691	6.51E-24	postive
LIPT1	AC078785.1	0.385393578	3.76E-19	postive
LIPT1	AC078802.1	0.327432531	5.85E-14	postive
LIPT1	AC078846.1	0.306807794	2.33E-12	postive
LIPT1	AC079145.1	0.399592852	1.36E-20	postive
LIPT1	AC079174.2	0.314764528	5.82E-13	postive
LIPT1	AC079322.1	0.303308325	4.24E-12	postive
LIPT1	AC079414.3	0.320639937	2.03E-13	postive
LIPT1	AC079447.1	0.450727586	2.18E-26	postive
LIPT1	AC079610.1	0.335636945	1.25E-14	postive
LIPT1	AC079807.1	0.491392948	9.39E-32	postive
LIPT1	AC079848.1	0.432299522	3.48E-24	postive
LIPT1	AC079921.2	0.360744282	8.20E-17	postive
LIPT1	AC079922.2	0.386104313	3.19E-19	postive
LIPT1	AC080038.3	0.307715292	1.99E-12	postive
LIPT1	AC080112.2	0.359065624	1.16E-16	postive
LIPT1	AC083798.2	0.417431243	1.67E-22	postive
LIPT1	AC083799.1	0.444868765	1.13E-25	postive
LIPT1	AC083964.1	0.313252105	7.60E-13	postive
LIPT1	AC084036.1	0.331864768	2.55E-14	postive
LIPT1	AC084125.4	0.321142122	1.86E-13	postive
LIPT1	AC084809.1	0.360609351	8.43E-17	postive
LIPT1	AC087289.2	0.398820626	1.64E-20	postive
LIPT1	AC087301.1	0.331994641	2.49E-14	postive
LIPT1	AC087500.1	0.412420175	5.92E-22	postive

LIPT1	AC087501.4	0.369064687	1.40E-17	postive
LIPT1	AC087521.3	0.378388114	1.82E-18	postive
LIPT1	AC087741.1	0.354796171	2.81E-16	postive
LIPT1	AC087741.2	0.37251041	6.64E-18	postive
LIPT1	AC090114.2	0.356040109	2.18E-16	postive
LIPT1	AC090198.1	0.313650797	7.08E-13	postive
LIPT1	AC090587.1	0.4462701	7.65E-26	postive
LIPT1	AC090587.2	0.420067526	8.55E-23	postive
LIPT1	AC090589.3	0.316609994	4.19E-13	postive
LIPT1	AC090617.5	0.462067044	8.19E-28	postive
LIPT1	AC090825.1	0.460300808	1.38E-27	postive
LIPT1	AC090912.1	0.471360576	5.08E-29	postive
LIPT1	AC091057.1	0.331771033	2.60E-14	postive
LIPT1	AC091057.3	0.319317559	2.58E-13	postive
LIPT1	AC091153.3	0.387101068	2.54E-19	postive
LIPT1	AC091153.4	0.312254029	9.06E-13	postive
LIPT1	AC091271.1	0.405518465	3.25E-21	postive
LIPT1	AC091390.5	0.303210645	4.31E-12	postive
LIPT1	AC091488.1	0.313092714	7.82E-13	postive
LIPT1	AC091563.1	0.305604744	2.86E-12	postive
LIPT1	AC091729.3	0.434682721	1.84E-24	postive
LIPT1	AC091887.1	0.320447649	2.10E-13	postive
LIPT1	AC091965.1	0.420561807	7.53E-23	postive
LIPT1	AC091965.4	0.358372401	1.34E-16	postive
LIPT1	AC091982.1	0.320680742	2.02E-13	postive
LIPT1	AC091982.3	0.405101406	3.60E-21	postive
LIPT1	AC092070.2	0.414036279	3.95E-22	postive
LIPT1	AC092115.4	0.352964873	4.08E-16	postive
LIPT1	AC092117.1	0.340826297	4.58E-15	postive
LIPT1	AC092118.2	0.326003909	7.62E-14	postive
LIPT1	AC092119.2	0.351577063	5.41E-16	postive
LIPT1	AC092123.1	0.367489557	1.97E-17	postive
LIPT1	AC092164.1	0.431780913	4.00E-24	postive
LIPT1	AC092171.5	0.319137718	2.67E-13	postive
LIPT1	AC092295.1	0.32641639	7.06E-14	postive
LIPT1	AC092295.2	0.411140876	8.14E-22	postive
LIPT1	AC092368.3	0.445505632	9.47E-26	postive
LIPT1	AC092614.1	0.396209575	3.05E-20	postive
LIPT1	AC092634.3	0.346045111	1.64E-15	postive
LIPT1	AC092650.1	0.344278655	2.33E-15	postive
LIPT1	AC092720.1	0.309093114	1.57E-12	postive
LIPT1	AC092803.1	0.313074565	7.84E-13	postive
LIPT1	AC092903.2	0.362331321	5.88E-17	postive
LIPT1	AC092910.3	0.401130851	9.42E-21	postive
LIPT1	AC092944.1	0.385023565	4.09E-19	postive
LIPT1	AC092953.2	0.321883141	1.62E-13	postive
LIPT1	AC093157.1	0.460074511	1.47E-27	postive
LIPT1	AC093157.2	0.300673343	6.61E-12	postive
LIPT1	AC093227.1	0.392063463	8.07E-20	postive
LIPT1	AC093249.6	0.328987566	4.38E-14	postive

LIPT1	AC093297.2	0.307534775	2.06E-12	postive
LIPT1	AC093503.1	0.300242629	7.10E-12	postive
LIPT1	AC093510.1	0.364513803	3.71E-17	postive
LIPT1	AC093582.1	0.311505359	1.03E-12	postive
LIPT1	AC093627.5	0.458774471	2.15E-27	postive
LIPT1	AC093673.1	0.300622822	6.66E-12	postive
LIPT1	AC093726.1	0.460470786	1.31E-27	postive
LIPT1	AC093726.2	0.380021429	1.26E-18	postive
LIPT1	AC093752.1	0.456102399	4.67E-27	postive
LIPT1	AC093752.3	0.357956416	1.46E-16	postive
LIPT1	AC096536.2	0.439414632	5.09E-25	postive
LIPT1	AC096540.1	0.328811158	4.53E-14	postive
LIPT1	AC096649.2	0.329883738	3.71E-14	postive
LIPT1	AC096677.1	0.388062862	2.04E-19	postive
LIPT1	AC096733.3	0.347416625	1.25E-15	postive
LIPT1	AC096772.6	0.411425641	7.59E-22	postive
LIPT1	AC096992.2	0.412003023	6.57E-22	postive
LIPT1	AC097359.2	0.377919455	2.02E-18	postive
LIPT1	AC097376.3	0.385192138	3.93E-19	postive
LIPT1	AC097468.3	0.376625388	2.69E-18	postive
LIPT1	AC097505.1	0.33161415	2.68E-14	postive
LIPT1	AC097639.1	0.333717631	1.80E-14	postive
LIPT1	AC098484.1	0.471522434	4.83E-29	postive
LIPT1	AC098484.2	0.421623529	5.73E-23	postive
LIPT1	AC098487.1	0.300439838	6.87E-12	postive
LIPT1	AC098591.2	0.305189688	3.08E-12	postive
LIPT1	AC098820.2	0.534341241	2.93E-38	postive
LIPT1	AC098820.4	0.349448629	8.31E-16	postive
LIPT1	AC098828.2	0.306902936	2.29E-12	postive
LIPT1	AC099343.4	0.503395496	1.76E-33	postive
LIPT1	AC099518.4	0.319901223	2.32E-13	postive
LIPT1	AC099522.2	0.327128505	6.19E-14	postive
LIPT1	AC099568.2	0.354239272	3.15E-16	postive
LIPT1	AC099778.1	0.428774253	8.88E-24	postive
LIPT1	AC099850.2	0.481362454	2.31E-30	postive
LIPT1	AC100778.2	0.31823666	3.13E-13	postive
LIPT1	AC100786.1	0.39788483	2.05E-20	postive
LIPT1	AC100810.1	0.347939812	1.12E-15	postive
LIPT1	AC100847.1	0.383617389	5.62E-19	postive
LIPT1	AC100861.1	0.354141743	3.21E-16	postive
LIPT1	AC103746.1	0.347700234	1.18E-15	postive
LIPT1	AC103760.1	0.356936442	1.81E-16	postive
LIPT1	AC103974.1	0.324491259	1.01E-13	postive
LIPT1	AC104083.1	0.312794004	8.24E-13	postive
LIPT1	AC104109.2	0.428600688	9.29E-24	postive
LIPT1	AC104113.1	0.359761795	1.01E-16	postive
LIPT1	AC104187.1	0.330038581	3.60E-14	postive
LIPT1	AC104532.2	0.337850857	8.15E-15	postive
LIPT1	AC104564.3	0.346604189	1.47E-15	postive
LIPT1	AC104596.1	0.411113878	8.20E-22	postive

LIPT1	AC104653.1	0.317727329	3.43E-13	postive
LIPT1	AC104653.2	0.312981452	7.97E-13	postive
LIPT1	AC104667.2	0.346418459	1.52E-15	postive
LIPT1	AC104794.3	0.431533792	4.27E-24	postive
LIPT1	AC104819.3	0.354915087	2.74E-16	postive
LIPT1	AC104825.1	0.324792955	9.53E-14	postive
LIPT1	AC105036.3	0.48295592	1.40E-30	postive
LIPT1	AC105137.2	0.349686093	7.93E-16	postive
LIPT1	AC105206.2	0.352927946	4.11E-16	postive
LIPT1	AC105219.1	0.341749379	3.83E-15	postive
LIPT1	AC105285.1	0.439688305	4.72E-25	postive
LIPT1	AC105345.1	0.303130051	4.37E-12	postive
LIPT1	AC105935.1	0.323812737	1.14E-13	postive
LIPT1	AC105942.1	0.351918308	5.05E-16	postive
LIPT1	AC106028.3	0.308123008	1.86E-12	postive
LIPT1	AC106779.1	0.32850436	4.80E-14	postive
LIPT1	AC106782.5	0.323410367	1.23E-13	postive
LIPT1	AC106786.2	0.38932308	1.52E-19	postive
LIPT1	AC106791.1	0.336324639	1.09E-14	postive
LIPT1	AC106795.1	0.469193539	9.78E-29	postive
LIPT1	AC106820.5	0.377422077	2.25E-18	postive
LIPT1	AC107027.3	0.377932514	2.01E-18	postive
LIPT1	AC107032.2	0.463578275	5.24E-28	postive
LIPT1	AC107068.1	0.364851876	3.45E-17	postive
LIPT1	AC107214.1	0.320601846	2.05E-13	postive
LIPT1	AC107294.2	0.304630132	3.38E-12	postive
LIPT1	AC107375.1	0.33112023	2.94E-14	postive
LIPT1	AC107884.1	0.327922318	5.34E-14	postive
LIPT1	AC107982.3	0.325780363	7.95E-14	postive
LIPT1	AC108134.2	0.37032302	1.07E-17	postive
LIPT1	AC108471.3	0.425453624	2.12E-23	postive
LIPT1	AC108488.1	0.44017024	4.14E-25	postive
LIPT1	AC108673.3	0.309158171	1.55E-12	postive
LIPT1	AC108704.1	0.3162695	4.45E-13	postive
LIPT1	AC108860.2	0.326651784	6.76E-14	postive
LIPT1	AC109322.1	0.383445808	5.85E-19	postive
LIPT1	AC109347.1	0.486638237	4.34E-31	postive
LIPT1	AC109449.1	0.324012418	1.10E-13	postive
LIPT1	AC109454.2	0.360705415	8.26E-17	postive
LIPT1	AC109460.1	0.355603909	2.38E-16	postive
LIPT1	AC109460.2	0.36699524	2.18E-17	postive
LIPT1	AC110079.1	0.358146478	1.41E-16	postive
LIPT1	AC110995.1	0.333698382	1.80E-14	postive
LIPT1	AC112220.2	0.426409319	1.65E-23	postive
LIPT1	AC112694.1	0.323768532	1.15E-13	postive
LIPT1	AC112907.3	0.345180464	1.95E-15	postive
LIPT1	AC113189.4	0.300471567	6.83E-12	postive
LIPT1	AC114271.1	0.451639198	1.68E-26	postive
LIPT1	AC114490.1	0.384911199	4.19E-19	postive
LIPT1	AC114763.1	0.402024608	7.60E-21	postive

LIPT1	AC114810.1	0.305139373	3.10E-12	postive
LIPT1	AC114956.2	0.441149789	3.16E-25	postive
LIPT1	AC115618.1	0.489314571	1.84E-31	postive
LIPT1	AC115618.2	0.4679999	1.40E-28	postive
LIPT1	AC116407.2	0.345801696	1.72E-15	postive
LIPT1	AC116535.2	0.352055871	4.91E-16	postive
LIPT1	AC116913.1	0.443956229	1.46E-25	postive
LIPT1	AC116914.2	0.379862125	1.31E-18	postive
LIPT1	AC117394.2	0.324187818	1.06E-13	postive
LIPT1	AC118553.1	0.419001464	1.12E-22	postive
LIPT1	AC119150.1	0.301043859	6.21E-12	postive
LIPT1	AC120053.1	0.360504009	8.62E-17	postive
LIPT1	AC120114.1	0.331531551	2.72E-14	postive
LIPT1	AC121338.2	0.462533426	7.14E-28	postive
LIPT1	AC121761.1	0.374566754	4.23E-18	postive
LIPT1	AC123768.2	0.335292276	1.33E-14	postive
LIPT1	AC123768.3	0.337935863	8.02E-15	postive
LIPT1	AC124016.2	0.38788503	2.12E-19	postive
LIPT1	AC124248.1	0.383901533	5.27E-19	postive
LIPT1	AC125257.1	0.479414154	4.25E-30	postive
LIPT1	AC125494.1	0.371217319	8.80E-18	postive
LIPT1	AC126283.1	0.340300601	5.07E-15	postive
LIPT1	AC127024.5	0.323859665	1.13E-13	postive
LIPT1	AC127024.6	0.344837591	2.08E-15	postive
LIPT1	AC127502.2	0.306335873	2.53E-12	postive
LIPT1	AC127521.1	0.343438338	2.75E-15	postive
LIPT1	AC127537.1	0.338059796	7.83E-15	postive
LIPT1	AC130324.1	0.382978123	6.50E-19	postive
LIPT1	AC130371.2	0.300570263	6.72E-12	postive
LIPT1	AC131009.3	0.350806905	6.32E-16	postive
LIPT1	AC131235.3	0.367358322	2.02E-17	postive
LIPT1	AC132008.2	0.402791783	6.31E-21	postive
LIPT1	AC132192.2	0.398854822	1.63E-20	postive
LIPT1	AC132938.3	0.317706656	3.45E-13	postive
LIPT1	AC132938.4	0.458933718	2.05E-27	postive
LIPT1	AC133528.2	0.392584039	7.15E-20	postive
LIPT1	AC133552.2	0.442163985	2.39E-25	postive
LIPT1	AC133552.5	0.312176672	9.18E-13	postive
LIPT1	AC134878.2	0.310925486	1.14E-12	postive
LIPT1	AC135048.3	0.35739328	1.65E-16	postive
LIPT1	AC135050.1	0.372120994	7.23E-18	postive
LIPT1	AC135050.5	0.365927441	2.74E-17	postive
LIPT1	AC135050.6	0.426677594	1.54E-23	postive
LIPT1	AC135178.4	0.393158463	6.25E-20	postive
LIPT1	AC135178.6	0.315050631	5.53E-13	postive
LIPT1	AC136475.2	0.304615576	3.39E-12	postive
LIPT1	AC136604.3	0.397722719	2.13E-20	postive
LIPT1	AC137630.1	0.349029265	9.04E-16	postive
LIPT1	AC138028.4	0.313928921	6.74E-13	postive
LIPT1	AC138035.1	0.398932512	1.60E-20	postive

LIPT1	AC138207.2	0.339630931	5.78E-15	postive
LIPT1	AC138207.4	0.315437609	5.16E-13	postive
LIPT1	AC138207.5	0.305963513	2.69E-12	postive
LIPT1	AC138207.8	0.442335552	2.28E-25	postive
LIPT1	AC138230.1	0.343703624	2.61E-15	postive
LIPT1	AC138932.1	0.316319721	4.41E-13	postive
LIPT1	AC138956.2	0.320605466	2.05E-13	postive
LIPT1	AC139256.2	0.360224742	9.14E-17	postive
LIPT1	AC139256.3	0.348516487	1.00E-15	postive
LIPT1	AC139530.1	0.418972046	1.13E-22	postive
LIPT1	AC139768.1	0.436704958	1.06E-24	postive
LIPT1	AC139795.2	0.380649252	1.10E-18	postive
LIPT1	AC139795.3	0.330798991	3.12E-14	postive
LIPT1	AC139887.2	0.378512943	1.77E-18	postive
LIPT1	AC141586.1	0.427390313	1.28E-23	postive
LIPT1	AC142472.1	0.402645832	6.54E-21	postive
LIPT1	AC145098.1	0.324409553	1.02E-13	postive
LIPT1	AC145124.1	0.308037585	1.89E-12	postive
LIPT1	AC145207.5	0.312437378	8.77E-13	postive
LIPT1	AC145285.2	0.392472088	7.34E-20	postive
LIPT1	AC145285.6	0.331360981	2.81E-14	postive
LIPT1	AC145423.3	0.336976999	9.65E-15	postive
LIPT1	AC147067.1	0.301312755	5.93E-12	postive
LIPT1	AC147651.4	0.314231102	6.39E-13	postive
LIPT1	AC231533.2	0.301290742	5.95E-12	postive
LIPT1	AC231981.1	0.462326937	7.59E-28	postive
LIPT1	AC232271.1	0.397235737	2.39E-20	postive
LIPT1	AC233728.1	0.325297719	8.68E-14	postive
LIPT1	AC242426.2	0.411558927	7.34E-22	postive
LIPT1	AC243919.1	0.460877188	1.16E-27	postive
LIPT1	AC244090.1	0.368085882	1.73E-17	postive
LIPT1	AC244669.1	0.434290257	2.04E-24	postive
LIPT1	AC245052.4	0.329614814	3.90E-14	postive
LIPT1	AC245060.6	0.356514425	1.97E-16	postive
LIPT1	AC245140.2	0.392457338	7.36E-20	postive
LIPT1	AC245297.4	0.33825657	7.54E-15	postive
LIPT1	AC245452.1	0.324594543	9.88E-14	postive
LIPT1	ACTG1P17	0.308980566	1.60E-12	postive
LIPT1	ACVR2B-AS1	0.340311278	5.06E-15	postive
LIPT1	AD000671.3	0.358544869	1.30E-16	postive
LIPT1	ADCY6-DT	0.329952227	3.66E-14	postive
LIPT1	ADNP-AS1	0.441135396	3.18E-25	postive
LIPT1	AF111167.2	0.387830122	2.15E-19	postive
LIPT1	AF117829.1	0.320006909	2.28E-13	postive
LIPT1	AF131216.4	0.331847676	2.56E-14	postive
LIPT1	AF196972.1	0.379229081	1.51E-18	postive
LIPT1	AFDN-DT	0.375809658	3.22E-18	postive
LIPT1	AFG3L1P	0.363264651	4.83E-17	postive
LIPT1	AHSA2P	0.349711644	7.88E-16	postive
LIPT1	AL008721.1	0.318552059	2.96E-13	postive

LIPT1	AL008729.1	0.363851364	4.26E-17	postive
LIPT1	AL009179.1	0.367338443	2.03E-17	postive
LIPT1	AL020996.1	0.428066448	1.07E-23	postive
LIPT1	AL021368.2	0.307536481	2.06E-12	postive
LIPT1	AL021707.3	0.322814806	1.37E-13	postive
LIPT1	AL021878.2	0.340454369	4.92E-15	postive
LIPT1	AL022069.1	0.35385635	3.40E-16	postive
LIPT1	AL022157.1	0.407492491	2.01E-21	postive
LIPT1	AL022311.1	0.365549361	2.97E-17	postive
LIPT1	AL022476.1	0.339682825	5.72E-15	postive
LIPT1	AL023581.2	0.315991688	4.68E-13	postive
LIPT1	AL024498.1	0.523493591	1.58E-36	postive
LIPT1	AL024507.3	0.449812829	2.82E-26	postive
LIPT1	AL031055.1	0.311194109	1.09E-12	postive
LIPT1	AL031123.2	0.361578931	6.88E-17	postive
LIPT1	AL031186.1	0.333676966	1.81E-14	postive
LIPT1	AL031282.1	0.437697478	8.13E-25	postive
LIPT1	AL031282.2	0.416703325	2.01E-22	postive
LIPT1	AL031289.1	0.349472743	8.27E-16	postive
LIPT1	AL031673.1	0.319564725	2.47E-13	postive
LIPT1	AL031709.1	0.313517663	7.25E-13	postive
LIPT1	AL031714.1	0.319346741	2.57E-13	postive
LIPT1	AL031775.1	0.405186226	3.53E-21	postive
LIPT1	AL031775.2	0.313472867	7.31E-13	postive
LIPT1	AL031846.2	0.301910195	5.36E-12	postive
LIPT1	AL031985.3	0.33092689	3.05E-14	postive
LIPT1	AL033527.3	0.409196136	1.32E-21	postive
LIPT1	AL035252.4	0.306073948	2.64E-12	postive
LIPT1	AL035530.2	0.335362073	1.31E-14	postive
LIPT1	AL035587.1	0.435286588	1.56E-24	postive
LIPT1	AL035661.1	0.385144663	3.98E-19	postive
LIPT1	AL049597.2	0.493781778	4.31E-32	postive
LIPT1	AL049780.1	0.311439899	1.04E-12	postive
LIPT1	AL049794.1	0.328236323	5.04E-14	postive
LIPT1	AL049796.1	0.428201339	1.03E-23	postive
LIPT1	AL049840.4	0.405622955	3.17E-21	postive
LIPT1	AL049840.5	0.400696448	1.05E-20	postive
LIPT1	AL049840.6	0.374728652	4.09E-18	postive
LIPT1	AL050309.1	0.44332383	1.74E-25	postive
LIPT1	AL050341.2	0.470560938	6.47E-29	postive
LIPT1	AL078581.2	0.377141996	2.40E-18	postive
LIPT1	AL078644.2	0.321145736	1.85E-13	postive
LIPT1	AL080250.1	0.31603788	4.64E-13	postive
LIPT1	AL080276.2	0.317107737	3.84E-13	postive
LIPT1	AL096678.1	0.332162867	2.41E-14	postive
LIPT1	AL096803.2	0.309304897	1.51E-12	postive
LIPT1	AL109811.1	0.357864781	1.49E-16	postive
LIPT1	AL109811.2	0.357812309	1.51E-16	postive
LIPT1	AL109917.1	0.334143554	1.66E-14	postive
LIPT1	AL109955.1	0.389299279	1.53E-19	postive

LIPT1	AL117327.1	0.30748069	2.08E-12	postive
LIPT1	AL117329.1	0.305883434	2.73E-12	postive
LIPT1	AL117332.1	0.452392641	1.36E-26	postive
LIPT1	AL117336.2	0.375186193	3.70E-18	postive
LIPT1	AL118506.1	0.443511505	1.65E-25	postive
LIPT1	AL118511.1	0.37933148	1.47E-18	postive
LIPT1	AL118516.1	0.337910774	8.06E-15	postive
LIPT1	AL118558.3	0.36588001	2.77E-17	postive
LIPT1	AL121574.1	0.303081396	4.40E-12	postive
LIPT1	AL121583.1	0.303068939	4.41E-12	postive
LIPT1	AL121603.2	0.363115186	4.98E-17	postive
LIPT1	AL121658.1	0.408291683	1.65E-21	postive
LIPT1	AL121890.4	0.348378948	1.03E-15	postive
LIPT1	AL121894.2	0.30340678	4.17E-12	postive
LIPT1	AL121917.1	0.345618613	1.79E-15	postive
LIPT1	AL121944.2	0.549202916	9.76E-41	postive
LIPT1	AL121987.2	0.325956696	7.69E-14	postive
LIPT1	AL121992.1	0.309573653	1.45E-12	postive
LIPT1	AL122010.1	0.42560789	2.04E-23	postive
LIPT1	AL122023.3	0.329760504	3.79E-14	postive
LIPT1	AL122035.1	0.397703909	2.14E-20	postive
LIPT1	AL122035.2	0.400697716	1.05E-20	postive
LIPT1	AL132657.1	0.320801012	1.97E-13	postive
LIPT1	AL132712.1	0.335572878	1.26E-14	postive
LIPT1	AL132780.1	0.405646725	3.15E-21	postive
LIPT1	AL132780.2	0.365043625	3.31E-17	postive
LIPT1	AL132800.1	0.375620372	3.36E-18	postive
LIPT1	AL132989.1	0.345237713	1.93E-15	postive
LIPT1	AL133215.2	0.31949239	2.50E-13	postive
LIPT1	AL133215.3	0.300284974	7.05E-12	postive
LIPT1	AL133338.1	0.469658645	8.50E-29	postive
LIPT1	AL133353.1	0.307656658	2.01E-12	postive
LIPT1	AL133367.1	0.343878004	2.52E-15	postive
LIPT1	AL133406.2	0.336865621	9.86E-15	postive
LIPT1	AL133410.1	0.302560268	4.81E-12	postive
LIPT1	AL133520.1	0.323990828	1.10E-13	postive
LIPT1	AL135791.1	0.386444971	2.95E-19	postive
LIPT1	AL135925.1	0.366808849	2.27E-17	postive
LIPT1	AL135999.1	0.328104644	5.17E-14	postive
LIPT1	AL136038.7	0.305370814	2.98E-12	postive
LIPT1	AL136040.1	0.511212445	1.22E-34	postive
LIPT1	AL136141.1	0.312510171	8.66E-13	postive
LIPT1	AL136295.2	0.373387632	5.48E-18	postive
LIPT1	AL136295.6	0.316348304	4.39E-13	postive
LIPT1	AL136295.7	0.344843416	2.08E-15	postive
LIPT1	AL136304.1	0.327406356	5.88E-14	postive
LIPT1	AL136368.1	0.343074316	2.95E-15	postive
LIPT1	AL136531.1	0.370103914	1.12E-17	postive
LIPT1	AL137003.1	0.420483381	7.68E-23	postive
LIPT1	AL137127.1	0.340977233	4.45E-15	postive

LIPT1	AL137186.2	0.336601618	1.04E-14	postive
LIPT1	AL137779.2	0.316816669	4.04E-13	postive
LIPT1	AL138724.2	0.33979026	5.60E-15	postive
LIPT1	AL138762.2	0.404282179	4.40E-21	postive
LIPT1	AL138921.1	0.409876598	1.11E-21	postive
LIPT1	AL138921.2	0.330821664	3.11E-14	postive
LIPT1	AL139011.1	0.462172395	7.94E-28	postive
LIPT1	AL139021.2	0.343848026	2.53E-15	postive
LIPT1	AL139035.1	0.372744629	6.31E-18	postive
LIPT1	AL139039.3	0.337343029	8.99E-15	postive
LIPT1	AL139089.1	0.345338135	1.89E-15	postive
LIPT1	AL139260.1	0.302935755	4.51E-12	postive
LIPT1	AL139260.2	0.308780673	1.66E-12	postive
LIPT1	AL139274.2	0.311362267	1.06E-12	postive
LIPT1	AL139286.2	0.35549224	2.43E-16	postive
LIPT1	AL139287.1	0.405890707	2.97E-21	postive
LIPT1	AL139289.1	0.30693671	2.28E-12	postive
LIPT1	AL139339.1	0.345160707	1.96E-15	postive
LIPT1	AL139352.1	0.33546858	1.29E-14	postive
LIPT1	AL139353.2	0.315621539	5.00E-13	postive
LIPT1	AL139383.1	0.353091868	3.98E-16	postive
LIPT1	AL139384.1	0.34170471	3.86E-15	postive
LIPT1	AL139397.1	0.370274691	1.08E-17	postive
LIPT1	AL157400.2	0.315408201	5.19E-13	postive
LIPT1	AL157400.3	0.337807648	8.22E-15	postive
LIPT1	AL157813.1	0.317036458	3.88E-13	postive
LIPT1	AL157832.1	0.324442906	1.02E-13	postive
LIPT1	AL158071.4	0.339733562	5.67E-15	postive
LIPT1	AL158212.3	0.338152819	7.69E-15	postive
LIPT1	AL158835.2	0.309795013	1.39E-12	postive
LIPT1	AL160006.1	0.447216085	5.87E-26	postive
LIPT1	AL160313.2	0.38053107	1.13E-18	postive
LIPT1	AL161421.1	0.346150683	1.61E-15	postive
LIPT1	AL161668.3	0.304251909	3.61E-12	postive
LIPT1	AL161719.1	0.308468378	1.75E-12	postive
LIPT1	AL161725.2	0.323755472	1.15E-13	postive
LIPT1	AL161729.1	0.389428962	1.49E-19	postive
LIPT1	AL162171.1	0.394320832	4.76E-20	postive
LIPT1	AL162231.2	0.47080931	6.00E-29	postive
LIPT1	AL162258.2	0.430005204	6.41E-24	postive
LIPT1	AL162377.1	0.492091333	7.48E-32	postive
LIPT1	AL162431.1	0.39575357	3.39E-20	postive
LIPT1	AL162586.1	0.336644333	1.03E-14	postive
LIPT1	AL162595.1	0.463803142	4.90E-28	postive
LIPT1	AL162632.3	0.307214142	2.17E-12	postive
LIPT1	AL162741.1	0.329028357	4.35E-14	postive
LIPT1	AL353194.1	0.437606296	8.33E-25	postive
LIPT1	AL353593.1	0.343961196	2.48E-15	postive
LIPT1	AL353596.1	0.434272193	2.05E-24	postive
LIPT1	AL353622.1	0.310678266	1.19E-12	postive

LIPT1	AL353622.2	0.337182163	9.28E-15	postive
LIPT1	AL353625.1	0.318786107	2.84E-13	postive
LIPT1	AL353708.1	0.527068758	4.31E-37	postive
LIPT1	AL353708.3	0.389324354	1.52E-19	postive
LIPT1	AL353743.1	0.376682071	2.66E-18	postive
LIPT1	AL353997.2	0.306764802	2.35E-12	postive
LIPT1	AL354696.1	0.436391027	1.16E-24	postive
LIPT1	AL354696.2	0.47788021	6.85E-30	postive
LIPT1	AL354740.1	0.324271385	1.05E-13	postive
LIPT1	AL354760.1	0.341890818	3.72E-15	postive
LIPT1	AL354892.2	0.410386607	9.82E-22	postive
LIPT1	AL354892.3	0.335169564	1.36E-14	postive
LIPT1	AL354920.1	0.532388721	6.07E-38	postive
LIPT1	AL354928.1	0.394634343	4.42E-20	postive
LIPT1	AL355001.2	0.435284106	1.56E-24	postive
LIPT1	AL355102.4	0.332445683	2.29E-14	postive
LIPT1	AL355297.3	0.303564169	4.06E-12	postive
LIPT1	AL355353.2	0.370313118	1.07E-17	postive
LIPT1	AL355385.1	0.303663543	3.99E-12	postive
LIPT1	AL355388.1	0.362938466	5.17E-17	postive
LIPT1	AL355574.1	0.414880148	3.19E-22	postive
LIPT1	AL355802.3	0.34095934	4.46E-15	postive
LIPT1	AL356019.2	0.336649707	1.03E-14	postive
LIPT1	AL356235.1	0.38233492	7.52E-19	postive
LIPT1	AL356299.3	0.333704726	1.80E-14	postive
LIPT1	AL356481.1	0.526467324	5.37E-37	postive
LIPT1	AL356488.3	0.376661623	2.67E-18	postive
LIPT1	AL356512.1	0.33363012	1.83E-14	postive
LIPT1	AL356599.1	0.439180779	5.43E-25	postive
LIPT1	AL357033.3	0.339520502	5.90E-15	postive
LIPT1	AL357033.4	0.323077766	1.30E-13	postive
LIPT1	AL357079.1	0.4548481	6.71E-27	postive
LIPT1	AL357140.2	0.414766229	3.29E-22	postive
LIPT1	AL357673.1	0.34727855	1.28E-15	postive
LIPT1	AL357874.1	0.332796176	2.14E-14	postive
LIPT1	AL358075.2	0.311375429	1.06E-12	postive
LIPT1	AL358472.2	0.500333558	4.94E-33	postive
LIPT1	AL358472.3	0.329347649	4.10E-14	postive
LIPT1	AL358852.1	0.307413778	2.10E-12	postive
LIPT1	AL358933.1	0.31650343	4.27E-13	postive
LIPT1	AL359091.3	0.37436875	4.42E-18	postive
LIPT1	AL359198.1	0.353461032	3.69E-16	postive
LIPT1	AL359232.1	0.42245485	4.62E-23	postive
LIPT1	AL359317.1	0.304369869	3.54E-12	postive
LIPT1	AL359317.2	0.307423147	2.10E-12	postive
LIPT1	AL359397.1	0.324334128	1.04E-13	postive
LIPT1	AL359513.1	0.408217276	1.68E-21	postive
LIPT1	AL359541.2	0.365855511	2.79E-17	postive
LIPT1	AL359643.3	0.459645978	1.67E-27	postive
LIPT1	AL359710.1	0.320011719	2.28E-13	postive

LIPT1	AL359711.2	0.335353992	1.32E-14	postive
LIPT1	AL359715.1	0.315221975	5.37E-13	postive
LIPT1	AL359715.3	0.484860194	7.65E-31	postive
LIPT1	AL359878.1	0.315427717	5.17E-13	postive
LIPT1	AL359921.1	0.340839289	4.57E-15	postive
LIPT1	AL359921.2	0.4362984	1.19E-24	postive
LIPT1	AL360270.3	0.35132779	5.69E-16	postive
LIPT1	AL365295.1	0.329050855	4.33E-14	postive
LIPT1	AL365330.1	0.415305208	2.87E-22	postive
LIPT1	AL390195.2	0.318144446	3.19E-13	postive
LIPT1	AL390198.1	0.371255669	8.72E-18	postive
LIPT1	AL390719.1	0.314103107	6.54E-13	postive
LIPT1	AL390728.4	0.452800552	1.21E-26	postive
LIPT1	AL390728.6	0.359464389	1.07E-16	postive
LIPT1	AL390879.1	0.309057684	1.58E-12	postive
LIPT1	AL390955.2	0.410367028	9.87E-22	postive
LIPT1	AL391069.2	0.418250936	1.36E-22	postive
LIPT1	AL391244.2	0.447176823	5.93E-26	postive
LIPT1	AL391684.1	0.322780118	1.38E-13	postive
LIPT1	AL391832.2	0.315764176	4.87E-13	postive
LIPT1	AL391834.2	0.308171436	1.84E-12	postive
LIPT1	AL392172.1	0.40509034	3.61E-21	postive
LIPT1	AL441992.2	0.425260796	2.23E-23	postive
LIPT1	AL442663.3	0.405819985	3.02E-21	postive
LIPT1	AL445222.2	0.303214636	4.30E-12	postive
LIPT1	AL445231.1	0.450366312	2.41E-26	postive
LIPT1	AL445645.1	0.336955616	9.69E-15	postive
LIPT1	AL450326.1	0.396129064	3.11E-20	postive
LIPT1	AL450384.2	0.347011007	1.35E-15	postive
LIPT1	AL450998.2	0.467403185	1.68E-28	postive
LIPT1	AL451050.2	0.32570306	8.06E-14	postive
LIPT1	AL451074.2	0.326509802	6.94E-14	postive
LIPT1	AL451074.6	0.308748073	1.67E-12	postive
LIPT1	AL451085.2	0.380054279	1.25E-18	postive
LIPT1	AL451085.3	0.462054894	8.22E-28	postive
LIPT1	AL451165.2	0.444977826	1.10E-25	postive
LIPT1	AL512343.2	0.303567263	4.05E-12	postive
LIPT1	AL512353.1	0.300994184	6.26E-12	postive
LIPT1	AL512408.1	0.382618802	7.05E-19	postive
LIPT1	AL512625.1	0.380344671	1.18E-18	postive
LIPT1	AL512625.2	0.309636202	1.43E-12	postive
LIPT1	AL512770.1	0.325876496	7.81E-14	postive
LIPT1	AL512791.1	0.310564268	1.22E-12	postive
LIPT1	AL512791.2	0.399072635	1.54E-20	postive
LIPT1	AL513165.1	0.392195912	7.82E-20	postive
LIPT1	AL513327.2	0.304054179	3.73E-12	postive
LIPT1	AL513477.1	0.340283104	5.09E-15	postive
LIPT1	AL513550.1	0.528451858	2.60E-37	postive
LIPT1	AL589765.4	0.309804722	1.39E-12	postive
LIPT1	AL589843.1	0.345984737	1.66E-15	postive

LIPT1	AL590096.1	0.33679123	1.00E-14	postive
LIPT1	AL590133.1	0.325452188	8.44E-14	postive
LIPT1	AL590617.2	0.340502509	4.88E-15	postive
LIPT1	AL590652.1	0.358179108	1.40E-16	postive
LIPT1	AL590729.1	0.306109213	2.63E-12	postive
LIPT1	AL590764.1	0.337256791	9.14E-15	postive
LIPT1	AL591043.2	0.382818845	6.74E-19	postive
LIPT1	AL591684.2	0.314134207	6.50E-13	postive
LIPT1	AL591895.1	0.315531789	5.08E-13	postive
LIPT1	AL592166.1	0.51957412	6.44E-36	postive
LIPT1	AL592435.1	0.439062557	5.60E-25	postive
LIPT1	AL596094.1	0.3289924	4.38E-14	postive
LIPT1	AL596202.1	0.37327333	5.62E-18	postive
LIPT1	AL603832.1	0.329907503	3.69E-14	postive
LIPT1	AL603839.2	0.396618115	2.77E-20	postive
LIPT1	AL603839.3	0.367949828	1.78E-17	postive
LIPT1	AL604028.1	0.467649293	1.56E-28	postive
LIPT1	AL606491.1	0.377939616	2.01E-18	postive
LIPT1	AL606760.1	0.393205529	6.18E-20	postive
LIPT1	AL606760.2	0.375303376	3.60E-18	postive
LIPT1	AL627171.3	0.363530629	4.56E-17	postive
LIPT1	AL645568.1	0.464950654	3.49E-28	postive
LIPT1	AL645728.1	0.42323648	3.78E-23	postive
LIPT1	AL645933.3	0.329092139	4.30E-14	postive
LIPT1	AL645940.1	0.394620406	4.43E-20	postive
LIPT1	AL662791.1	0.348306429	1.05E-15	postive
LIPT1	AL662844.3	0.342282504	3.45E-15	postive
LIPT1	AL662844.4	0.406455942	2.59E-21	postive
LIPT1	AL669831.1	0.369615475	1.24E-17	postive
LIPT1	AL671710.1	0.361846154	6.51E-17	postive
LIPT1	AL691432.1	0.375890928	3.16E-18	postive
LIPT1	AL691432.2	0.389990431	1.31E-19	postive
LIPT1	AL731563.4	0.364547747	3.68E-17	postive
LIPT1	AL731566.2	0.371662313	7.99E-18	postive
LIPT1	AL731569.1	0.357349969	1.66E-16	postive
LIPT1	AL731571.1	0.324396104	1.03E-13	postive
LIPT1	AL731577.2	0.407868956	1.83E-21	postive
LIPT1	AL732314.8	0.335303052	1.33E-14	postive
LIPT1	AL732372.2	0.339916077	5.47E-15	postive
LIPT1	AL844908.2	0.306699633	2.37E-12	postive
LIPT1	AL928654.1	0.321218923	1.83E-13	postive
LIPT1	AL928654.2	0.318269414	3.12E-13	postive
LIPT1	ALG1L13P	0.32400254	1.10E-13	postive
LIPT1	ALOX12-AS1	0.536658648	1.23E-38	postive
LIPT1	AMZ2P1	0.571243732	1.20E-44	postive
LIPT1	ANO7L1	0.465648901	2.83E-28	postive
LIPT1	AP000253.1	0.326336553	7.17E-14	postive
LIPT1	AP000254.2	0.408798848	1.45E-21	postive
LIPT1	AP000255.1	0.305523133	2.91E-12	postive
LIPT1	AP000317.1	0.325975991	7.66E-14	postive

LIPT1	AP000320.1	0.313180278	7.70E-13	postive
LIPT1	AP000442.1	0.452747072	1.22E-26	postive
LIPT1	AP000487.1	0.441691628	2.72E-25	postive
LIPT1	AP000692.1	0.320485284	2.09E-13	postive
LIPT1	AP000704.2	0.426356949	1.68E-23	postive
LIPT1	AP000759.1	0.307803834	1.96E-12	postive
LIPT1	AP000781.1	0.35538702	2.49E-16	postive
LIPT1	AP000787.1	0.310695242	1.19E-12	postive
LIPT1	AP000808.1	0.325764066	7.97E-14	postive
LIPT1	AP000845.1	0.395440982	3.65E-20	postive
LIPT1	AP000866.1	0.372139588	7.20E-18	postive
LIPT1	AP000873.2	0.421352407	6.14E-23	postive
LIPT1	AP000894.4	0.365007304	3.34E-17	postive
LIPT1	AP001107.1	0.410547888	9.44E-22	postive
LIPT1	AP001160.1	0.37079708	9.64E-18	postive
LIPT1	AP001160.3	0.502962987	2.04E-33	postive
LIPT1	AP001178.3	0.323884798	1.13E-13	postive
LIPT1	AP001267.1	0.331416385	2.78E-14	postive
LIPT1	AP001267.3	0.369856249	1.18E-17	postive
LIPT1	AP001318.2	0.423061554	3.95E-23	postive
LIPT1	AP001330.4	0.311231434	1.08E-12	postive
LIPT1	AP001351.1	0.329232253	4.19E-14	postive
LIPT1	AP001372.2	0.492309343	6.97E-32	postive
LIPT1	AP001412.1	0.336154948	1.13E-14	postive
LIPT1	AP001432.1	0.41673945	2.00E-22	postive
LIPT1	AP001453.2	0.365306963	3.13E-17	postive
LIPT1	AP001453.4	0.396573744	2.80E-20	postive
LIPT1	AP001462.1	0.425979179	1.85E-23	postive
LIPT1	AP001469.2	0.347021546	1.35E-15	postive
LIPT1	AP001469.3	0.377818549	2.06E-18	postive
LIPT1	AP001486.2	0.351986166	4.98E-16	postive
LIPT1	AP001505.1	0.370088225	1.12E-17	postive
LIPT1	AP001527.2	0.331903647	2.54E-14	postive
LIPT1	AP001628.1	0.311883014	9.66E-13	postive
LIPT1	AP001816.1	0.446629748	6.91E-26	postive
LIPT1	AP001830.1	0.370467486	1.03E-17	postive
LIPT1	AP001922.5	0.368067876	1.74E-17	postive
LIPT1	AP002026.1	0.331239522	2.87E-14	postive
LIPT1	AP002360.2	0.400630765	1.06E-20	postive
LIPT1	AP002387.2	0.39528569	3.79E-20	postive
LIPT1	AP002748.4	0.430354122	5.84E-24	postive
LIPT1	AP002812.2	0.315633254	4.99E-13	postive
LIPT1	AP002840.2	0.417256706	1.75E-22	postive
LIPT1	AP003068.2	0.339248997	6.22E-15	postive
LIPT1	AP003096.1	0.372090748	7.28E-18	postive
LIPT1	AP003352.1	0.322665591	1.41E-13	postive
LIPT1	AP003392.4	0.392714896	6.93E-20	postive
LIPT1	AP003392.5	0.460087235	1.47E-27	postive
LIPT1	AP003419.3	0.300793953	6.47E-12	postive
LIPT1	AP003486.1	0.395012897	4.04E-20	postive

LIPT1	AP003498.1	0.350583599	6.61E-16	postive
LIPT1	AP003721.4	0.41625065	2.26E-22	postive
LIPT1	AP003973.2	0.304363666	3.54E-12	postive
LIPT1	AP004609.5	0.438581688	6.39E-25	postive
LIPT1	AP005061.2	0.36942751	1.30E-17	postive
LIPT1	AP005205.2	0.303190126	4.32E-12	postive
LIPT1	AP005329.1	0.376832034	2.57E-18	postive
LIPT1	AP005329.2	0.439147563	5.48E-25	postive
LIPT1	AP005432.2	0.365236815	3.18E-17	postive
LIPT1	AP005482.4	0.368670385	1.53E-17	postive
LIPT1	AP005717.1	0.302882799	4.55E-12	postive
LIPT1	AP006222.1	0.341268816	4.20E-15	postive
LIPT1	AP006621.3	0.408732302	1.48E-21	postive
LIPT1	APOC1P1	0.364256467	3.91E-17	postive
LIPT1	APTR	0.450858993	2.10E-26	postive
LIPT1	ARF4-AS1	0.427102614	1.38E-23	postive
LIPT1	ARHGEF26-AS1	0.369234083	1.35E-17	postive
LIPT1	ARMCX5-GPRASP2	0.565033462	1.63E-43	postive
LIPT1	ARNILA	0.306028615	2.66E-12	postive
LIPT1	ARRDC1-AS1	0.466067307	2.50E-28	postive
LIPT1	ASB16-AS1	0.470031114	7.60E-29	postive
LIPT1	ASH1L-AS1	0.462589539	7.02E-28	postive
LIPT1	ATF4P4	0.313687556	7.04E-13	postive
LIPT1	ATP1A1-AS1	0.327392389	5.90E-14	postive
LIPT1	ATXN1-AS1	0.435326553	1.54E-24	postive
LIPT1	ATXN2-AS	0.325687196	8.08E-14	postive
LIPT1	AZIN1-AS1	0.335212821	1.35E-14	postive
LIPT1	B4GALT1-AS1	0.352209665	4.76E-16	postive
LIPT1	BAALC-AS1	0.387623357	2.26E-19	postive
LIPT1	BACE1-AS	0.393265897	6.09E-20	postive
LIPT1	BAIAP2-DT	0.353799752	3.44E-16	postive
LIPT1	BCAR3-AS1	0.353048156	4.01E-16	postive
LIPT1	BCDIN3D-AS1	0.525369393	8.00E-37	postive
LIPT1	BDNF-AS	0.48334664	1.24E-30	postive
LIPT1	BEND3P1	0.324821994	9.48E-14	postive
LIPT1	BHLHE40-AS1	0.444092616	1.40E-25	postive
LIPT1	BMS1P20	0.471222331	5.29E-29	postive
LIPT1	BOLA2P2	0.335966277	1.17E-14	postive
LIPT1	BRWD1-AS2	0.306080284	2.64E-12	postive
LIPT1	BTG3-AS1	0.358890341	1.21E-16	postive
LIPT1	BTN2A3P	0.437684349	8.16E-25	postive
LIPT1	BVES-AS1	0.310572232	1.22E-12	postive
LIPT1	BX284668.5	0.320614317	2.04E-13	postive
LIPT1	BX322557.10	0.433044714	2.85E-24	postive
LIPT1	BX537318.1	0.409309138	1.28E-21	postive
LIPT1	BX649601.1	0.341577431	3.96E-15	postive
LIPT1	BX649632.1	0.347803818	1.16E-15	postive
LIPT1	C10orf25	0.358440705	1.32E-16	postive
LIPT1	C15orf56	0.336812937	9.96E-15	postive
LIPT1	C1orf147	0.300968081	6.29E-12	postive

LIPT1	C1RL-AS1	0.325590339	8.23E-14	postive
LIPT1	C21orf62-AS1	0.496343959	1.86E-32	postive
LIPT1	C22orf46	0.423069198	3.94E-23	postive
LIPT1	C5orf34-AS1	0.339117601	6.38E-15	postive
LIPT1	C6orf99	0.425949125	1.86E-23	postive
LIPT1	C9orf147	0.412908833	5.24E-22	postive
LIPT1	CA5BP1	0.47931251	4.39E-30	postive
LIPT1	CAHM	0.314838181	5.74E-13	postive
LIPT1	CAMTA1-DT	0.328787715	4.55E-14	postive
LIPT1	CAPN10-DT	0.395405802	3.69E-20	postive
LIPT1	CARD8-AS1	0.450239491	2.50E-26	postive
LIPT1	CASC2	0.305464605	2.93E-12	postive
LIPT1	CASP17P	0.354406325	3.04E-16	postive
LIPT1	CATSPER2P1	0.398444434	1.79E-20	postive
LIPT1	CBR3-AS1	0.337814309	8.21E-15	postive
LIPT1	CCDC183-AS1	0.314869702	5.71E-13	postive
LIPT1	CCDC18-AS1	0.34035793	5.02E-15	postive
LIPT1	CCDC84-DT	0.382820186	6.74E-19	postive
LIPT1	CCNT2-AS1	0.531404385	8.74E-38	postive
LIPT1	CCT6P1	0.389806372	1.36E-19	postive
LIPT1	CCT6P3	0.361395904	7.15E-17	postive
LIPT1	CD27-AS1	0.446305244	7.57E-26	postive
LIPT1	CD2BP2-DT	0.362647971	5.50E-17	postive
LIPT1	CDC37L1-DT	0.430751014	5.26E-24	postive
LIPT1	CEBPB-AS1	0.407749149	1.88E-21	postive
LIPT1	CENPBD1P1	0.485250086	6.76E-31	postive
LIPT1	CEP250-AS1	0.344146918	2.39E-15	postive
LIPT1	CEP83-DT	0.414071829	3.91E-22	postive
LIPT1	CERNA1	0.476847597	9.44E-30	postive
LIPT1	CFL1P1	0.474196058	2.14E-29	postive
LIPT1	CHKB-DT	0.31501859	5.56E-13	postive
LIPT1	CHROMR	0.394095108	5.02E-20	postive
LIPT1	CIDECP1	0.499842804	5.82E-33	postive
LIPT1	CKMT2-AS1	0.514094002	4.46E-35	postive
LIPT1	CLUHP3	0.334637694	1.51E-14	postive
LIPT1	CNNM3-DT	0.500662213	4.42E-33	postive
LIPT1	COA6-AS1	0.388382104	1.89E-19	postive
LIPT1	COX10-AS1	0.525170129	8.61E-37	postive
LIPT1	CR381653.1	0.402444848	6.87E-21	postive
LIPT1	CRNDE	0.311296411	1.07E-12	postive
LIPT1	CROCCP2	0.347768648	1.16E-15	postive
LIPT1	CROCCP3	0.311644359	1.01E-12	postive
LIPT1	CRTC3-AS1	0.466341645	2.30E-28	postive
LIPT1	CRYBB2P1	0.356398877	2.02E-16	postive
LIPT1	CRYZL2P	0.413190594	4.88E-22	postive
LIPT1	CSE1L-AS1	0.339167196	6.32E-15	postive
LIPT1	CTA-14H9.5	0.326238462	7.30E-14	postive
LIPT1	CTA-989H11.1	0.345475652	1.84E-15	postive
LIPT1	CTBP1-DT	0.522457985	2.29E-36	postive
LIPT1	CTC-487M23.5	0.34493615	2.04E-15	postive

LIPT1	CTD-2132N18.4	0.330134593	3.54E-14	postive
LIPT1	CTD-2134A5.3	0.364675793	3.58E-17	postive
LIPT1	CTD-2245F17.3	0.388611787	1.80E-19	postive
LIPT1	CTD-2521M24.9	0.467553654	1.60E-28	postive
LIPT1	CTD-2574D22.4	0.437607668	8.33E-25	postive
LIPT1	CUTALP	0.37438709	4.40E-18	postive
LIPT1	CYP51A1-AS1	0.345263386	1.92E-15	postive
LIPT1	CYTOR	0.420977466	6.77E-23	postive
LIPT1	DANCR	0.341201923	4.26E-15	postive
LIPT1	DARS-AS1	0.494375724	3.55E-32	postive
LIPT1	DBIL5P	0.37536656	3.55E-18	postive
LIPT1	DCST1-AS1	0.31389809	6.78E-13	postive
LIPT1	DCXR-DT	0.400934823	9.88E-21	postive
LIPT1	DDX11-AS1	0.326587268	6.85E-14	postive
LIPT1	DDX39B-AS1	0.318714535	2.88E-13	postive
LIPT1	DGUOK-AS1	0.317117524	3.83E-13	postive
LIPT1	DHCR24-DT	0.410463359	9.64E-22	postive
LIPT1	DHRS4-AS1	0.4591745	1.91E-27	postive
LIPT1	DICER1-AS1	0.349898514	7.59E-16	postive
LIPT1	DLEU1	0.400432911	1.11E-20	postive
LIPT1	DLEU7-AS1	0.306221077	2.58E-12	postive
LIPT1	DLG1-AS1	0.307383857	2.11E-12	postive
LIPT1	DLGAP1-AS1	0.342881812	3.06E-15	postive
LIPT1	DLGAP4-AS1	0.322992355	1.33E-13	postive
LIPT1	DM1-AS	0.36712548	2.12E-17	postive
LIPT1	DNAJC27-AS1	0.399225412	1.49E-20	postive
LIPT1	DNAJC3-DT	0.388003175	2.07E-19	postive
LIPT1	DNM1P35	0.313127205	7.77E-13	postive
LIPT1	DOCK8-AS1	0.311235478	1.08E-12	postive
LIPT1	DOCK9-DT	0.3038908	3.84E-12	postive
LIPT1	DPH6-DT	0.317797201	3.39E-13	postive
LIPT1	DPY19L2P3	0.331176108	2.91E-14	postive
LIPT1	DSCAS	0.356306902	2.06E-16	postive
LIPT1	DSCR9	0.358692337	1.26E-16	postive
LIPT1	DSTNP2	0.403335056	5.54E-21	postive
LIPT1	DUBR	0.327109078	6.21E-14	postive
LIPT1	DYRK3-AS1	0.346366901	1.54E-15	postive
LIPT1	EBLN3P	0.510829738	1.39E-34	postive
LIPT1	ECI2-DT	0.388181245	1.98E-19	postive
LIPT1	EDRF1-DT	0.375921684	3.14E-18	postive
LIPT1	EIF1B-AS1	0.551032622	4.74E-41	postive
LIPT1	EIF2AK3-DT	0.476544963	1.04E-29	postive
LIPT1	EIF3J-DT	0.579513172	3.43E-46	postive
LIPT1	ELF3-AS1	0.416356881	2.20E-22	postive
LIPT1	ELOA-AS1	0.53984014	3.67E-39	postive
LIPT1	EMC1-AS1	0.559473303	1.60E-42	postive
LIPT1	EMC3-AS1	0.343297292	2.82E-15	postive
LIPT1	ENO1-AS1	0.324748195	9.61E-14	postive
LIPT1	ENTPD1-AS1	0.310221867	1.29E-12	postive
LIPT1	ENTPD3-AS1	0.543210308	1.01E-39	postive

LIPT1	EP400P1	0.302656713	4.73E-12	postive
LIPT1	EPB41L4A-AS1	0.424181926	2.95E-23	postive
LIPT1	EPB41L4A-DT	0.418045653	1.43E-22	postive
LIPT1	EPCAM-DT	0.401556437	8.51E-21	postive
LIPT1	ETV7-AS1	0.342134079	3.55E-15	postive
LIPT1	EXOC3-AS1	0.376262025	2.91E-18	postive
LIPT1	EXOSC10-AS1	0.379273385	1.49E-18	postive
LIPT1	FAAHP1	0.321174843	1.84E-13	postive
LIPT1	FABP5P3	0.320501438	2.08E-13	postive
LIPT1	FAHD2CP	0.488369909	2.49E-31	postive
LIPT1	FALEC	0.359227569	1.12E-16	postive
LIPT1	FAM111A-DT	0.555254827	8.81E-42	postive
LIPT1	FAM157B	0.365811506	2.81E-17	postive
LIPT1	FAM160A1-DT	0.409074817	1.36E-21	postive
LIPT1	FAM21EP	0.356307526	2.06E-16	postive
LIPT1	FAM21FP	0.412869625	5.29E-22	postive
LIPT1	FAM66C	0.310524999	1.23E-12	postive
LIPT1	FAM66D	0.306357232	2.52E-12	postive
LIPT1	FAM86B3P	0.366980348	2.19E-17	postive
LIPT1	FAM86C2P	0.584198173	4.37E-47	postive
LIPT1	FAM86DP	0.407512846	2.00E-21	postive
LIPT1	FAM86EP	0.532121796	6.70E-38	postive
LIPT1	FAM86HP	0.424912199	2.44E-23	postive
LIPT1	FAM86JP	0.553906409	1.51E-41	postive
LIPT1	FAM87B	0.326906874	6.45E-14	postive
LIPT1	FBXL19-AS1	0.309155905	1.55E-12	postive
LIPT1	FGD5-AS1	0.561337777	7.47E-43	postive
LIPT1	FGF7P3	0.315306842	5.29E-13	postive
LIPT1	FLVCR1-DT	0.425234433	2.25E-23	postive
LIPT1	FMR1-AS1	0.334001993	1.70E-14	postive
LIPT1	FO393418.1	0.352975903	4.07E-16	postive
LIPT1	FO704657.1	0.315481724	5.12E-13	postive
LIPT1	FOXD2-AS1	0.312915527	8.06E-13	postive
LIPT1	FRG1-DT	0.338781812	6.81E-15	postive
LIPT1	FRG1HP	0.571937257	8.95E-45	postive
LIPT1	FZD4-DT	0.344849656	2.08E-15	postive
LIPT1	GABPB1-AS1	0.309296656	1.52E-12	postive
LIPT1	GAS5	0.379839657	1.32E-18	postive
LIPT1	GAS5-AS1	0.385090986	4.02E-19	postive
LIPT1	GBAP1	0.328614051	4.70E-14	postive
LIPT1	GCC2-AS1	0.486235054	4.94E-31	postive
LIPT1	GEMIN7-AS1	0.370944386	9.33E-18	postive
LIPT1	GGTA1P	0.336008077	1.16E-14	postive
LIPT1	GHET1	0.372135252	7.21E-18	postive
LIPT1	GIHCG	0.355439268	2.46E-16	postive
LIPT1	GLIDR	0.477049071	8.87E-30	postive
LIPT1	GLUD1P3	0.351256837	5.77E-16	postive
LIPT1	GMDS-DT	0.419419777	1.01E-22	postive
LIPT1	GNRHR2	0.507463263	4.42E-34	postive
LIPT1	GOLGA2P5	0.306421934	2.49E-12	postive

LIPT1	GPRC5D-AS1	0.402859366	6.21E-21	postive
LIPT1	GS1-124K5.3	0.386045305	3.24E-19	postive
LIPT1	GS1-358P8.4	0.335984619	1.17E-14	postive
LIPT1	GSEC	0.344524737	2.22E-15	postive
LIPT1	GTF2IP1	0.455112119	6.21E-27	postive
LIPT1	GTF2IP20	0.321399373	1.77E-13	postive
LIPT1	GTF2IP4	0.44093149	3.36E-25	postive
LIPT1	GTF2IRD2P1	0.369564599	1.26E-17	postive
LIPT1	GTSE1-DT	0.379388551	1.46E-18	postive
LIPT1	GUSBP1	0.405212974	3.51E-21	postive
LIPT1	GUSBP2	0.385813097	3.41E-19	postive
LIPT1	H2AZ2P1	0.391094331	1.01E-19	postive
LIPT1	HCG11	0.325408687	8.51E-14	postive
LIPT1	HCG15	0.371283407	8.67E-18	postive
LIPT1	HCG18	0.495665385	2.32E-32	postive
LIPT1	HCG25	0.538294673	6.60E-39	postive
LIPT1	HCG27	0.362641814	5.50E-17	postive
LIPT1	HCP5	0.44997824	2.69E-26	postive
LIPT1	HDAC11-AS1	0.317089836	3.85E-13	postive
LIPT1	HDHD5-AS1	0.340211777	5.16E-15	postive
LIPT1	HEIH	0.507138181	4.95E-34	postive
LIPT1	HERC2P3	0.344794906	2.10E-15	postive
LIPT1	HERC2P4	0.315903957	4.75E-13	postive
LIPT1	HIPK1-AS1	0.378634567	1.72E-18	postive
LIPT1	HLA-DPB2	0.332217139	2.39E-14	postive
LIPT1	HLA-DRB6	0.385234454	3.89E-19	postive
LIPT1	HLA-L	0.341136793	4.31E-15	postive
LIPT1	HM13-IT1	0.407693241	1.91E-21	postive
LIPT1	HMGB1P5	0.449956716	2.71E-26	postive
LIPT1	HSD17B7P2	0.364763107	3.51E-17	postive
LIPT1	IBA57-DT	0.314678269	5.91E-13	postive
LIPT1	IDH1-AS1	0.388499387	1.84E-19	postive
LIPT1	IL10RB-DT	0.379978814	1.28E-18	postive
LIPT1	IL6R-AS1	0.391046703	1.02E-19	postive
LIPT1	ILF3-DT	0.472163939	3.98E-29	postive
LIPT1	INE1	0.328184876	5.09E-14	postive
LIPT1	INTS6-AS1	0.454602416	7.20E-27	postive
LIPT1	IPO5P1	0.391046361	1.02E-19	postive
LIPT1	IPO9-AS1	0.35002679	7.40E-16	postive
LIPT1	IQCH-AS1	0.489659098	1.65E-31	postive
LIPT1	IRF1-AS1	0.361042459	7.70E-17	postive
LIPT1	ITFG2-AS1	0.449604288	2.99E-26	postive
LIPT1	ITGA9-AS1	0.438795615	6.03E-25	postive
LIPT1	ITPR1-DT	0.309245821	1.53E-12	postive
LIPT1	JPX	0.42567408	2.00E-23	postive
LIPT1	KANSL1-AS1	0.311876806	9.68E-13	postive
LIPT1	KCNIP2-AS1	0.366657362	2.35E-17	postive
LIPT1	KCTD21-AS1	0.368311368	1.65E-17	postive
LIPT1	KDM2B-DT	0.333147079	2.00E-14	postive
LIPT1	KDM4A-AS1	0.465612435	2.86E-28	postive

LIPT1	KDM7A-DT	0.339702193	5.70E-15	postive
LIPT1	KIAA1671-AS1	0.309516981	1.46E-12	postive
LIPT1	KIF1C-AS1	0.340234557	5.14E-15	postive
LIPT1	KIF9-AS1	0.308621085	1.71E-12	postive
LIPT1	KLHL7-DT	0.307315487	2.14E-12	postive
LIPT1	KMT2E-AS1	0.399089878	1.54E-20	postive
LIPT1	KRT8P12	0.41303744	5.07E-22	postive
LIPT1	KTN1-AS1	0.540947499	2.40E-39	postive
LIPT1	L3MBTL2-AS1	0.37994872	1.28E-18	postive
LIPT1	LAMTOR5-AS1	0.442087451	2.44E-25	postive
LIPT1	LBX2-AS1	0.350986061	6.10E-16	postive
LIPT1	LENG8-AS1	0.376634728	2.68E-18	postive
LIPT1	LGR4-AS1	0.324668774	9.75E-14	postive
LIPT1	LIMD1-AS1	0.406371588	2.64E-21	postive
LIPT1	LINC00115	0.335990409	1.17E-14	postive
LIPT1	LINC00173	0.300006703	7.39E-12	postive
LIPT1	LINC00174	0.366265544	2.55E-17	postive
LIPT1	LINC00205	0.435292782	1.56E-24	postive
LIPT1	LINC00235	0.327790257	5.48E-14	postive
LIPT1	LINC002481	0.354317493	3.10E-16	postive
LIPT1	LINC00294	0.318296381	3.10E-13	postive
LIPT1	LINC00310	0.304345336	3.55E-12	postive
LIPT1	LINC00324	0.478887449	5.01E-30	postive
LIPT1	LINC00339	0.667261732	1.02E-65	postive
LIPT1	LINC00449	0.389554166	1.45E-19	postive
LIPT1	LINC00467	0.485681451	5.89E-31	postive
LIPT1	LINC00471	0.41561378	2.65E-22	postive
LIPT1	LINC00476	0.536909229	1.12E-38	postive
LIPT1	LINC00526	0.463332768	5.64E-28	postive
LIPT1	LINC00539	0.384021296	5.13E-19	postive
LIPT1	LINC00630	0.39057337	1.14E-19	postive
LIPT1	LINC00638	0.327963498	5.30E-14	postive
LIPT1	LINC00652	0.310685838	1.19E-12	postive
LIPT1	LINC00653	0.38485111	4.25E-19	postive
LIPT1	LINC00654	0.385511787	3.66E-19	postive
LIPT1	LINC00663	0.420343707	7.96E-23	postive
LIPT1	LINC00665	0.35872231	1.25E-16	postive
LIPT1	LINC00667	0.511253775	1.20E-34	postive
LIPT1	LINC00674	0.322726769	1.39E-13	postive
LIPT1	LINC00680	0.512644102	7.40E-35	postive
LIPT1	LINC00847	0.447088292	6.08E-26	postive
LIPT1	LINC00853	0.360081174	9.41E-17	postive
LIPT1	LINC00863	0.386184047	3.14E-19	postive
LIPT1	LINC00886	0.331621061	2.67E-14	postive
LIPT1	LINC00888	0.340735772	4.66E-15	postive
LIPT1	LINC00892	0.316168816	4.53E-13	postive
LIPT1	LINC00898	0.312605401	8.51E-13	postive
LIPT1	LINC00899	0.354564879	2.94E-16	postive
LIPT1	LINC00900	0.313009206	7.93E-13	postive
LIPT1	LINC00909	0.570671318	1.53E-44	postive

LIPT1	LINC00910	0.470753053	6.10E-29	postive
LIPT1	LINC00921	0.458076464	2.64E-27	postive
LIPT1	LINC00933	0.408754011	1.47E-21	postive
LIPT1	LINC00957	0.384933153	4.17E-19	postive
LIPT1	LINC00963	0.437478463	8.63E-25	postive
LIPT1	LINC00987	0.310037302	1.33E-12	postive
LIPT1	LINC00992	0.315536949	5.07E-13	postive
LIPT1	LINC01001	0.329707621	3.83E-14	postive
LIPT1	LINC01003	0.3372203	9.21E-15	postive
LIPT1	LINC01006	0.307209294	2.18E-12	postive
LIPT1	LINC01011	0.510497567	1.56E-34	postive
LIPT1	LINC01023	0.328167306	5.11E-14	postive
LIPT1	LINC01058	0.335519702	1.28E-14	postive
LIPT1	LINC01089	0.300792164	6.48E-12	postive
LIPT1	LINC01094	0.339030789	6.49E-15	postive
LIPT1	LINC01128	0.485687105	5.88E-31	postive
LIPT1	LINC01135	0.365369318	3.09E-17	postive
LIPT1	LINC01137	0.473743705	2.45E-29	postive
LIPT1	LINC01138	0.359548364	1.05E-16	postive
LIPT1	LINC01140	0.326055892	7.55E-14	postive
LIPT1	LINC01144	0.390678632	1.11E-19	postive
LIPT1	LINC01145	0.390985388	1.04E-19	postive
LIPT1	LINC01184	0.520387167	4.82E-36	postive
LIPT1	LINC01220	0.342970463	3.01E-15	postive
LIPT1	LINC01232	0.305599759	2.87E-12	postive
LIPT1	LINC01270	0.335322077	1.32E-14	postive
LIPT1	LINC01271	0.3080262	1.89E-12	postive
LIPT1	LINC01273	0.40277153	6.34E-21	postive
LIPT1	LINC01278	0.4830552	1.36E-30	postive
LIPT1	LINC01291	0.339218357	6.26E-15	postive
LIPT1	LINC01315	0.35233058	4.64E-16	postive
LIPT1	LINC01337	0.306930394	2.28E-12	postive
LIPT1	LINC01347	0.325611399	8.20E-14	postive
LIPT1	LINC01355	0.317991337	3.27E-13	postive
LIPT1	LINC01372	0.396048823	3.17E-20	postive
LIPT1	LINC01424	0.382796001	6.77E-19	postive
LIPT1	LINC01426	0.329372	4.08E-14	postive
LIPT1	LINC01431	0.329404115	4.05E-14	postive
LIPT1	LINC01465	0.30737494	2.11E-12	postive
LIPT1	LINC01506	0.375433904	3.50E-18	postive
LIPT1	LINC01521	0.345456216	1.84E-15	postive
LIPT1	LINC01534	0.386230641	3.10E-19	postive
LIPT1	LINC01545	0.326707721	6.69E-14	postive
LIPT1	LINC01560	0.549119205	1.01E-40	postive
LIPT1	LINC01569	0.411115674	8.19E-22	postive
LIPT1	LINC01578	0.394408262	4.66E-20	postive
LIPT1	LINC01588	0.321239099	1.82E-13	postive
LIPT1	LINC01635	0.433593354	2.46E-24	postive
LIPT1	LINC01703	0.352710771	4.30E-16	postive
LIPT1	LINC01715	0.423203779	3.81E-23	postive

LIPT1	LINC01719	0.389906514	1.33E-19	postive
LIPT1	LINC01725	0.32050284	2.08E-13	postive
LIPT1	LINC01772	0.379711591	1.35E-18	postive
LIPT1	LINC01806	0.363712822	4.39E-17	postive
LIPT1	LINC01816	0.337083148	9.45E-15	postive
LIPT1	LINC01836	0.319666339	2.42E-13	postive
LIPT1	LINC01841	0.351344053	5.67E-16	postive
LIPT1	LINC01852	0.356402602	2.02E-16	postive
LIPT1	LINC01881	0.428183358	1.04E-23	postive
LIPT1	LINC01942	0.313168995	7.71E-13	postive
LIPT1	LINC02014	0.309355775	1.50E-12	postive
LIPT1	LINC02019	0.327749944	5.52E-14	postive
LIPT1	LINC02021	0.353253281	3.85E-16	postive
LIPT1	LINC02035	0.321380806	1.78E-13	postive
LIPT1	LINC02062	0.341811271	3.78E-15	postive
LIPT1	LINC02073	0.300167815	7.19E-12	postive
LIPT1	LINC02147	0.327047257	6.29E-14	postive
LIPT1	LINC02193	0.365091298	3.28E-17	postive
LIPT1	LINC02210	0.434074508	2.16E-24	postive
LIPT1	LINC02256	0.332802367	2.14E-14	postive
LIPT1	LINC02285	0.365890472	2.77E-17	postive
LIPT1	LINC02298	0.31851867	2.98E-13	postive
LIPT1	LINC02340	0.304170687	3.66E-12	postive
LIPT1	LINC02361	0.323066232	1.31E-13	postive
LIPT1	LINC02391	0.33592677	1.18E-14	postive
LIPT1	LINC02427	0.302742414	4.66E-12	postive
LIPT1	LINC02447	0.381075297	9.98E-19	postive
LIPT1	LINC02453	0.356231599	2.09E-16	postive
LIPT1	LINC02519	0.347075844	1.34E-15	postive
LIPT1	LINC02550	0.348660648	9.74E-16	postive
LIPT1	LINC02569	0.336163907	1.13E-14	postive
LIPT1	LINC02585	0.397110941	2.46E-20	postive
LIPT1	LINC02604	0.324271108	1.05E-13	postive
LIPT1	LINC02609	0.39718412	2.42E-20	postive
LIPT1	LINC02611	0.366452996	2.45E-17	postive
LIPT1	LINC02613	0.308595996	1.71E-12	postive
LIPT1	LINC02637	0.414135934	3.85E-22	postive
LIPT1	LINC02693	0.369375691	1.31E-17	postive
LIPT1	LINC02724	0.357751262	1.53E-16	postive
LIPT1	LINC02762	0.300835889	6.43E-12	postive
LIPT1	LINC02763	0.322213512	1.53E-13	postive
LIPT1	LINC02777	0.355879976	2.25E-16	postive
LIPT1	LINC02861	0.357218785	1.71E-16	postive
LIPT1	LIPE-AS1	0.404135592	4.56E-21	postive
LIPT1	LIX1L-AS1	0.328033361	5.23E-14	postive
LIPT1	LMCD1-AS1	0.382134584	7.86E-19	postive
LIPT1	LNCTAM34A	0.370633452	9.98E-18	postive
LIPT1	LOH12CR2	0.499098323	7.45E-33	postive
LIPT1	LOXL1-AS1	0.356070721	2.16E-16	postive
LIPT1	LPP-AS2	0.384806588	4.29E-19	postive

LIPT1	LRRC37A17P	0.395606414	3.51E-20	postive
LIPT1	LRRC37BP1	0.487453573	3.35E-31	postive
LIPT1	LRRC8C-DT	0.35472917	2.85E-16	postive
LIPT1	LSP1P4	0.325976677	7.66E-14	postive
LIPT1	LYPLAL1-DT	0.34726245	1.29E-15	postive
LIPT1	MACORIS	0.332500692	2.26E-14	postive
LIPT1	MAFG-DT	0.378199574	1.90E-18	postive
LIPT1	MAN1B1-DT	0.386593053	2.86E-19	postive
LIPT1	MAP2K4P1	0.425690532	1.99E-23	postive
LIPT1	MAP3K14-AS1	0.408434053	1.59E-21	postive
LIPT1	MAP3K2-DT	0.500207801	5.15E-33	postive
LIPT1	MAP4K3-DT	0.412298709	6.10E-22	postive
LIPT1	MAPKAPK5-AS1	0.633583483	1.79E-57	postive
LIPT1	MATN1-AS1	0.412341119	6.04E-22	postive
LIPT1	MCM3AP-AS1	0.474388461	2.01E-29	postive
LIPT1	MCPH1-AS1	0.306273876	2.55E-12	postive
LIPT1	MED14OS	0.418936923	1.14E-22	postive
LIPT1	MED8-AS1	0.322380036	1.48E-13	postive
LIPT1	MFF-DT	0.396953254	2.56E-20	postive
LIPT1	MIATNB	0.41016777	1.04E-21	postive
LIPT1	MIF-AS1	0.34390483	2.51E-15	postive
LIPT1	MINCR	0.390745263	1.10E-19	postive
LIPT1	MIPEPP3	0.40648032	2.57E-21	postive
LIPT1	MIR155HG	0.31337518	7.44E-13	postive
LIPT1	MIR22HG	0.405123729	3.58E-21	postive
LIPT1	MIR34AHG	0.327635074	5.64E-14	postive
LIPT1	MIR3936HG	0.424915527	2.44E-23	postive
LIPT1	MIR4435-2HG	0.4007311	1.04E-20	postive
LIPT1	MIR4453HG	0.428220944	1.03E-23	postive
LIPT1	MIR4458HG	0.360206253	9.17E-17	postive
LIPT1	MIR497HG	0.311831034	9.75E-13	postive
LIPT1	MIR762HG	0.475773011	1.32E-29	postive
LIPT1	MKLN1-AS	0.445990317	8.27E-26	postive
LIPT1	MKNK1-AS1	0.324093462	1.08E-13	postive
LIPT1	MMADHC-DT	0.41905461	1.11E-22	postive
LIPT1	MORF4L2-AS1	0.320416969	2.12E-13	postive
LIPT1	MRPL20-AS1	0.617318422	7.55E-54	postive
LIPT1	MRPL45P2	0.523651467	1.49E-36	postive
LIPT1	MRPS31P4	0.398087088	1.95E-20	postive
LIPT1	MRPS31P5	0.351226271	5.81E-16	postive
LIPT1	MRPS9-AS1	0.329111272	4.28E-14	postive
LIPT1	MRPS9-AS2	0.311320391	1.07E-12	postive
LIPT1	MSH5-SAPCD1	0.400365474	1.13E-20	postive
LIPT1	MSL3P1	0.388555521	1.82E-19	postive
LIPT1	MUC20-OT1	0.345219757	1.93E-15	postive
LIPT1	MYLK-AS1	0.434400654	1.98E-24	postive
LIPT1	MZF1-AS1	0.333546325	1.86E-14	postive
LIPT1	NACA4P	0.342199332	3.50E-15	postive
LIPT1	NAIPP1	0.337944747	8.01E-15	postive
LIPT1	NAPA-AS1	0.426999436	1.42E-23	postive

LIPT1	NBPF25P	0.434868633	1.75E-24	postive
LIPT1	NBPF8	0.380074504	1.25E-18	postive
LIPT1	NBR2	0.497216759	1.39E-32	postive
LIPT1	NCBP2-AS1	0.309857223	1.38E-12	postive
LIPT1	NCK1-DT	0.531700376	7.83E-38	postive
LIPT1	NCMAP-DT	0.384165268	4.97E-19	postive
LIPT1	NDUFA6-DT	0.402271131	7.16E-21	postive
LIPT1	NDUFB2-AS1	0.366387505	2.49E-17	postive
LIPT1	NDUFV2-AS1	0.370175515	1.10E-17	postive
LIPT1	NEBL-AS1	0.308038015	1.89E-12	postive
LIPT1	NFYC-AS1	0.353387619	3.75E-16	postive
LIPT1	NIFK-AS1	0.658524436	1.78E-63	postive
LIPT1	NINJ2-AS1	0.366810115	2.27E-17	postive
LIPT1	NKAPP1	0.472406216	3.69E-29	postive
LIPT1	NNT-AS1	0.369890521	1.17E-17	postive
LIPT1	NOP14-AS1	0.502931076	2.06E-33	postive
LIPT1	NOP53-AS1	0.31040025	1.25E-12	postive
LIPT1	NORAD	0.431698566	4.09E-24	postive
LIPT1	NPIPP1	0.313814258	6.88E-13	postive
LIPT1	NRAV	0.519490254	6.63E-36	postive
LIPT1	NRSN2-AS1	0.518544796	9.29E-36	postive
LIPT1	NSMCE1-DT	0.340799371	4.60E-15	postive
LIPT1	NSUN5P2	0.309972206	1.35E-12	postive
LIPT1	NUDT16P1	0.522073395	2.63E-36	postive
LIPT1	NUP50-DT	0.339258103	6.21E-15	postive
LIPT1	NUTM2A-AS1	0.52526625	8.31E-37	postive
LIPT1	NUTM2B-AS1	0.324947855	9.26E-14	postive
LIPT1	OCIAD1-AS1	0.318467871	3.01E-13	postive
LIPT1	OGFR-AS1	0.317857123	3.35E-13	postive
LIPT1	OGFRP1	0.462355858	7.52E-28	postive
LIPT1	OIP5-AS1	0.519479141	6.66E-36	postive
LIPT1	OLMALINC	0.39784055	2.07E-20	postive
LIPT1	OSER1-DT	0.593703555	6.02E-49	postive
LIPT1	OSGEPL1-AS1	0.492769861	6.00E-32	postive
LIPT1	OSTCP1	0.318004587	3.27E-13	postive
LIPT1	OTUD6B-AS1	0.483330946	1.24E-30	postive
LIPT1	PAN3-AS1	0.384362829	4.75E-19	postive
LIPT1	PAXBP1-AS1	0.395814454	3.35E-20	postive
LIPT1	PAXIP1-AS1	0.398949711	1.59E-20	postive
LIPT1	PAXIP1-AS2	0.515073485	3.16E-35	postive
LIPT1	PCBP1-AS1	0.553771734	1.60E-41	postive
LIPT1	PCCA-DT	0.37656728	2.72E-18	postive
LIPT1	PCED1B-AS1	0.349604184	8.06E-16	postive
LIPT1	PDCD4-AS1	0.458107385	2.61E-27	postive
LIPT1	PDIA3P1	0.397979963	2.00E-20	postive
LIPT1	PIK3CD-AS2	0.41526982	2.89E-22	postive
LIPT1	PINK1-AS	0.44194935	2.54E-25	postive
LIPT1	PIPSL	0.364889905	3.42E-17	postive
LIPT1	PITPNA-AS1	0.53518143	2.14E-38	postive
LIPT1	PKD1P6	0.382746133	6.85E-19	postive

LIPT1	PLBD1-AS1	0.328420262	4.87E-14	postive
LIPT1	PLEKHA8P1	0.444537537	1.24E-25	postive
LIPT1	PMS2CL	0.398960435	1.59E-20	postive
LIPT1	PMS2P3	0.37753918	2.20E-18	postive
LIPT1	PMS2P4	0.496410088	1.82E-32	postive
LIPT1	POLH-AS1	0.328947088	4.42E-14	postive
LIPT1	POLR2J4	0.463140924	5.97E-28	postive
LIPT1	PPP1R12A-AS1	0.300297949	7.03E-12	postive
LIPT1	PPP1R26-AS1	0.371974731	7.46E-18	postive
LIPT1	PPP3CB-AS1	0.454623395	7.15E-27	postive
LIPT1	PPP4R1-AS1	0.309843087	1.38E-12	postive
LIPT1	PPT2-EGFL8	0.412244886	6.18E-22	postive
LIPT1	PRC1-AS1	0.339805612	5.59E-15	postive
LIPT1	PRELID1P1	0.41592174	2.46E-22	postive
LIPT1	PRKAR2A-AS1	0.446005453	8.23E-26	postive
LIPT1	PRKCZ-AS1	0.31011759	1.32E-12	postive
LIPT1	PRMT5-AS1	0.342440852	3.34E-15	postive
LIPT1	PRORSD1P	0.470998449	5.67E-29	postive
LIPT1	PROSER2-AS1	0.305112635	3.12E-12	postive
LIPT1	PRR34	0.385915106	3.33E-19	postive
LIPT1	PRRT3-AS1	0.303303468	4.24E-12	postive
LIPT1	PSMA3-AS1	0.445353813	9.87E-26	postive
LIPT1	PSMB8-AS1	0.48233208	1.70E-30	postive
LIPT1	PTENP1	0.358885553	1.21E-16	postive
LIPT1	PTOV1-AS1	0.451102365	1.96E-26	postive
LIPT1	PXN-AS1	0.586939137	1.29E-47	postive
LIPT1	RAB11B-AS1	0.369341853	1.32E-17	postive
LIPT1	RAB30-DT	0.488079609	2.74E-31	postive
LIPT1	RABGAP1L-DT	0.40211837	7.43E-21	postive
LIPT1	RAD21-AS1	0.373951696	4.85E-18	postive
LIPT1	RAD51-AS1	0.370068288	1.13E-17	postive
LIPT1	RAET1K	0.34661837	1.46E-15	postive
LIPT1	RALY-AS1	0.466907207	1.95E-28	postive
LIPT1	RAP2C-AS1	0.381933192	8.23E-19	postive
LIPT1	RARA-AS1	0.38947101	1.47E-19	postive
LIPT1	RASAL2-AS1	0.346733956	1.43E-15	postive
LIPT1	RASSF1-AS1	0.333043208	2.04E-14	postive
LIPT1	RB1-DT	0.340834057	4.57E-15	postive
LIPT1	RBM15-AS1	0.495062318	2.83E-32	postive
LIPT1	RBM26-AS1	0.435248635	1.58E-24	postive
LIPT1	RDM1P5	0.397341692	2.33E-20	postive
LIPT1	RHPN1-AS1	0.333350208	1.93E-14	postive
LIPT1	RN7SL832P	0.371596542	8.10E-18	postive
LIPT1	RNASEH1-AS1	0.468461904	1.22E-28	postive
LIPT1	RNF157-AS1	0.360585938	8.47E-17	postive
LIPT1	RNF216P1	0.430789401	5.21E-24	postive
LIPT1	RNFT1-DT	0.329042701	4.34E-14	postive
LIPT1	ROCK1P1	0.363663032	4.44E-17	postive
LIPT1	RP11-101E13.5	0.465785485	2.72E-28	postive
LIPT1	RP11-10C24.1	0.400599761	1.07E-20	postive

LIPT1	RP11-111M22.4	0.301543456	5.71E-12	postive
LIPT1	RP11-153F5.7	0.345417849	1.86E-15	postive
LIPT1	RP11-159G9.5	0.356084084	2.16E-16	postive
LIPT1	RP11-15H20.6	0.301878888	5.39E-12	postive
LIPT1	RP11-15N24.4	0.353642672	3.56E-16	postive
LIPT1	RP11-160O5.1	0.327291293	6.01E-14	postive
LIPT1	RP11-164P12.4	0.319072861	2.70E-13	postive
LIPT1	RP11-175O19.4	0.325058113	9.08E-14	postive
LIPT1	RP11-199F11.2	0.331042412	2.98E-14	postive
LIPT1	RP11-206L10.5	0.318577721	2.95E-13	postive
LIPT1	RP11-226L15.5	0.434048633	2.18E-24	postive
LIPT1	RP1-122P22.2	0.402827761	6.26E-21	postive
LIPT1	RP11-258C19.7	0.362979637	5.13E-17	postive
LIPT1	RP11-282O18.3	0.355026483	2.68E-16	postive
LIPT1	RP11-314C16.1	0.361522593	6.96E-17	postive
LIPT1	RP11-337C18.9	0.379376977	1.46E-18	postive
LIPT1	RP11-392O1.4	0.304141899	3.68E-12	postive
LIPT1	RP11-435O5.2	0.410859749	8.73E-22	postive
LIPT1	RP11-462G12.2	0.324430393	1.02E-13	postive
LIPT1	RP11-465N4.5	0.313432743	7.36E-13	postive
LIPT1	RP11-539I5.1	0.419473857	9.95E-23	postive
LIPT1	RP11-540B6.6	0.38645006	2.95E-19	postive
LIPT1	RP11-658F2.8	0.401942958	7.75E-21	postive
LIPT1	RP11-661A12.4	0.311399836	1.05E-12	postive
LIPT1	RP11-706O15.1	0.355278686	2.54E-16	postive
LIPT1	RP11-86H7.7	0.325129787	8.96E-14	postive
LIPT1	RP11-876N24.4	0.385584626	3.60E-19	postive
LIPT1	RP11-876N24.5	0.401136623	9.41E-21	postive
LIPT1	RP11-89K10.1	0.363703105	4.40E-17	postive
LIPT1	RP13-36G14.4	0.301100943	6.15E-12	postive
LIPT1	RP13-476E20.1	0.414677518	3.36E-22	postive
LIPT1	RP1-90G24.6	0.30126983	5.98E-12	postive
LIPT1	RP3-461P17.10	0.308357476	1.78E-12	postive
LIPT1	RP4-545L17.11	0.461100298	1.09E-27	postive
LIPT1	RP5-1039K5.19	0.354211737	3.17E-16	postive
LIPT1	RP5-1042I8.7	0.301643293	5.61E-12	postive
LIPT1	RP5-1112D6.4	0.386476073	2.93E-19	postive
LIPT1	RP5-1125A11.6	0.425632692	2.02E-23	postive
LIPT1	RP5-1125A11.7	0.383192504	6.19E-19	postive
LIPT1	RP5-1139B12.2	0.324820692	9.48E-14	postive
LIPT1	RPARP-AS1	0.549506666	8.66E-41	postive
LIPT1	RPL12P25	0.364184373	3.97E-17	postive
LIPT1	RPL12P38	0.336591781	1.04E-14	postive
LIPT1	RPL13AP3	0.346092531	1.63E-15	postive
LIPT1	RPL13AP7	0.432903662	2.96E-24	postive
LIPT1	RPL13P5	0.403330922	5.54E-21	postive
LIPT1	RPL23AP53	0.387096589	2.54E-19	postive
LIPT1	RPL23AP7	0.555716598	7.32E-42	postive
LIPT1	RPL23AP82	0.567275894	6.39E-44	postive
LIPT1	RPL32P3	0.396346539	2.95E-20	postive

LIPT1	RPL34-AS1	0.340767065	4.63E-15	postive
LIPT1	RPL39P5	0.303421272	4.16E-12	postive
LIPT1	RPL6P27	0.318901633	2.78E-13	postive
LIPT1	RPLP1P6	0.413146348	4.94E-22	postive
LIPT1	RPS10P7	0.397528694	2.23E-20	postive
LIPT1	RPS18P9	0.472597554	3.48E-29	postive
LIPT1	RPS27AP16	0.323295713	1.25E-13	postive
LIPT1	RPS4XP16	0.513124122	6.26E-35	postive
LIPT1	RPSAP31	0.334493705	1.55E-14	postive
LIPT1	RRN3P3	0.438032724	7.42E-25	postive
LIPT1	RRP7BP	0.425158747	2.29E-23	postive
LIPT1	RTCA-AS1	0.420177376	8.31E-23	postive
LIPT1	RUSC1-AS1	0.325852804	7.84E-14	postive
LIPT1	SAMD12-AS1	0.313395972	7.41E-13	postive
LIPT1	SAP30-DT	0.322126694	1.55E-13	postive
LIPT1	SAPCD1-AS1	0.367210227	2.09E-17	postive
LIPT1	SBDSP1	0.476448752	1.07E-29	postive
LIPT1	SBF2-AS1	0.436858923	1.02E-24	postive
LIPT1	SCAMP1-AS1	0.588910975	5.31E-48	postive
LIPT1	SCAND2P	0.491821862	8.17E-32	postive
LIPT1	SCOC-AS1	0.365226543	3.19E-17	postive
LIPT1	SDAD1P1	0.375608886	3.37E-18	postive
LIPT1	SDCBP2-AS1	0.49632921	1.87E-32	postive
LIPT1	SDHAP1	0.366641698	2.36E-17	postive
LIPT1	SEC22B4P	0.393001131	6.48E-20	postive
LIPT1	SEC24B-AS1	0.509270041	2.38E-34	postive
LIPT1	SEMA3F-AS1	0.350099815	7.29E-16	postive
LIPT1	SEPSECS-AS1	0.472255539	3.87E-29	postive
LIPT1	SEPTIN4-AS1	0.468086311	1.37E-28	postive
LIPT1	SEPTIN7-AS1	0.415614617	2.65E-22	postive
LIPT1	SEPTIN7P2	0.426880577	1.46E-23	postive
LIPT1	SERHL	0.383311471	6.03E-19	postive
LIPT1	SERTAD4-AS1	0.362805149	5.32E-17	postive
LIPT1	SGMS1-AS1	0.394033359	5.09E-20	postive
LIPT1	SGOL1-AS1	0.307231592	2.17E-12	postive
LIPT1	SH3BP5-AS1	0.316872503	4.00E-13	postive
LIPT1	SH3GL1P1	0.320152794	2.22E-13	postive
LIPT1	SHLD2P1	0.356543856	1.96E-16	postive
LIPT1	SLC12A9-AS1	0.387068443	2.56E-19	postive
LIPT1	SLC25A5-AS1	0.421273615	6.27E-23	postive
LIPT1	SLC25A5P1	0.320834765	1.96E-13	postive
LIPT1	SLC44A3-AS1	0.411950759	6.66E-22	postive
LIPT1	SLC5A4-AS1	0.301539141	5.71E-12	postive
LIPT1	SMC5-AS1	0.314851237	5.73E-13	postive
LIPT1	SMG7-AS1	0.465107339	3.33E-28	postive
LIPT1	SMPD5	0.317764104	3.41E-13	postive
LIPT1	SNAI3-AS1	0.416573057	2.08E-22	postive
LIPT1	SNHG1	0.396054826	3.16E-20	postive
LIPT1	SNHG10	0.388768014	1.73E-19	postive
LIPT1	SNHG11	0.536347757	1.38E-38	postive

LIPT1	SNHG12	0.389968263	1.31E-19	postive
LIPT1	SNHG15	0.306795029	2.34E-12	postive
LIPT1	SNHG16	0.483582454	1.15E-30	postive
LIPT1	SNHG17	0.374348644	4.44E-18	postive
LIPT1	SNHG20	0.494502981	3.40E-32	postive
LIPT1	SNHG21	0.466596852	2.13E-28	postive
LIPT1	SNHG28	0.452001165	1.52E-26	postive
LIPT1	SNHG29	0.372708209	6.36E-18	postive
LIPT1	SNHG3	0.363917619	4.20E-17	postive
LIPT1	SNHG30	0.409999251	1.08E-21	postive
LIPT1	SNHG31	0.32442203	1.02E-13	postive
LIPT1	SNHG32	0.440712089	3.57E-25	postive
LIPT1	SNHG4	0.398454453	1.79E-20	postive
LIPT1	SNHG5	0.405435737	3.32E-21	postive
LIPT1	SNHG6	0.353039833	4.02E-16	postive
LIPT1	SNHG7	0.319465321	2.51E-13	postive
LIPT1	SNHG8	0.428090413	1.06E-23	postive
LIPT1	SP2-AS1	0.448643861	3.93E-26	postive
LIPT1	SPAG5-AS1	0.41521589	2.93E-22	postive
LIPT1	SPART-AS1	0.325816294	7.89E-14	postive
LIPT1	SPINT1-AS1	0.401024901	9.67E-21	postive
LIPT1	SREBF2-AS1	0.441190097	3.13E-25	postive
LIPT1	SRP14-AS1	0.48172753	2.06E-30	postive
LIPT1	SRP54-AS1	0.378313474	1.85E-18	postive
LIPT1	SRRM2-AS1	0.31673017	4.10E-13	postive
LIPT1	SSR4P1	0.466638169	2.11E-28	postive
LIPT1	ST20-AS1	0.387773048	2.18E-19	postive
LIPT1	STAG3L1	0.428002529	1.09E-23	postive
LIPT1	STAG3L2	0.441888334	2.58E-25	postive
LIPT1	STAG3L3	0.428585771	9.33E-24	postive
LIPT1	STAG3L4	0.438946061	5.79E-25	postive
LIPT1	STAG3L5P	0.350504359	6.72E-16	postive
LIPT1	STAG3L5P-PVRIG2P-PILRB	0.340633347	4.76E-15	postive
LIPT1	STAM-AS1	0.349168368	8.79E-16	postive
LIPT1	STARD7-AS1	0.60841664	5.94E-52	postive
LIPT1	STK4-AS1	0.507665238	4.13E-34	postive
LIPT1	STX17-AS1	0.455871758	4.99E-27	postive
LIPT1	STX18-AS1	0.492377696	6.82E-32	postive
LIPT1	SUCLA2-AS1	0.419298786	1.04E-22	postive
LIPT1	SUCLG2-AS1	0.315774016	4.86E-13	postive
LIPT1	SUGT1P4-STRA6LP	0.317949064	3.30E-13	postive
LIPT1	SUMO2P17	0.466022233	2.53E-28	postive
LIPT1	SUZ12P1	0.394928457	4.12E-20	postive
LIPT1	SVIL-AS1	0.499690956	6.12E-33	postive
LIPT1	SYNPR-AS1	0.343381334	2.78E-15	postive
LIPT1	SYS1-DBNDD2	0.348845514	9.38E-16	postive
LIPT1	TAF1A-AS1	0.44359927	1.61E-25	postive
LIPT1	TAPT1-AS1	0.40748745	2.01E-21	postive
LIPT1	TBC1D8-AS1	0.474396373	2.01E-29	postive
LIPT1	TDRKH-AS1	0.389088474	1.61E-19	postive

LIPT1	THAP7-AS1	0.300389432	6.93E-12	postive
LIPT1	THAP9-AS1	0.480388813	3.13E-30	postive
LIPT1	THRB-AS1	0.379940049	1.29E-18	postive
LIPT1	THUMPD3-AS1	0.373457872	5.40E-18	postive
LIPT1	TIPARP-AS1	0.440473475	3.81E-25	postive
LIPT1	TMC3-AS1	0.452268277	1.40E-26	postive
LIPT1	TMCC1-AS1	0.406028136	2.87E-21	postive
LIPT1	TMCO1-AS1	0.345126966	1.97E-15	postive
LIPT1	TMED2-DT	0.334128782	1.66E-14	postive
LIPT1	TMEM147-AS1	0.397993547	2.00E-20	postive
LIPT1	TMEM161B-AS1	0.471842496	4.38E-29	postive
LIPT1	TMEM198B	0.351126106	5.93E-16	postive
LIPT1	TMEM202-AS1	0.414949757	3.14E-22	postive
LIPT1	TMEM231P1	0.30188287	5.39E-12	postive
LIPT1	TMEM44-AS1	0.486239966	4.93E-31	postive
LIPT1	TMEM99	0.4493065	3.26E-26	postive
LIPT1	TMEM9B-AS1	0.387893543	2.12E-19	postive
LIPT1	TNKS2-AS1	0.351418133	5.59E-16	postive
LIPT1	TNRC6C-AS1	0.319689937	2.41E-13	postive
LIPT1	TOB1-AS1	0.512647195	7.39E-35	postive
LIPT1	TOLLIP-AS1	0.422991275	4.02E-23	postive
LIPT1	TP53TG1	0.417287686	1.74E-22	postive
LIPT1	TP73-AS1	0.48924786	1.88E-31	postive
LIPT1	TPM3P9	0.39118985	9.89E-20	postive
LIPT1	TPT1-AS1	0.412618126	5.63E-22	postive
LIPT1	TPTE2P1	0.300614896	6.67E-12	postive
LIPT1	TPTEP2	0.374627035	4.18E-18	postive
LIPT1	TRAF3IP2-AS1	0.458931603	2.05E-27	postive
LIPT1	TRAM2-AS1	0.596799955	1.44E-49	postive
LIPT1	TRBV11-2	0.314068338	6.58E-13	postive
LIPT1	TRIM52-AS1	0.516787621	1.73E-35	postive
LIPT1	TSC22D1-AS1	0.305942478	2.70E-12	postive
LIPT1	TSPOAP1-AS1	0.359082998	1.16E-16	postive
LIPT1	TSPY26P	0.336507889	1.06E-14	postive
LIPT1	TSTD3	0.514442932	3.95E-35	postive
LIPT1	TTC28-AS1	0.536091646	1.52E-38	postive
LIPT1	TTC41P	0.418549969	1.26E-22	postive
LIPT1	TTN-AS1	0.324307687	1.04E-13	postive
LIPT1	TUBBP5	0.314789061	5.79E-13	postive
LIPT1	TUG1	0.456643278	3.99E-27	postive
LIPT1	U47924.2	0.388640987	1.78E-19	postive
LIPT1	U91328.1	0.415486719	2.74E-22	postive
LIPT1	UBA6-AS1	0.577397949	8.60E-46	postive
LIPT1	UBAC2-AS1	0.457759842	2.89E-27	postive
LIPT1	UBE2D3-AS1	0.580926143	1.85E-46	postive
LIPT1	UBE2Q1-AS1	0.317645351	3.48E-13	postive
LIPT1	UBL7-AS1	0.409278236	1.29E-21	postive
LIPT1	UBR5-AS1	0.445398027	9.75E-26	postive
LIPT1	UGDH-AS1	0.541391837	2.03E-39	postive
LIPT1	UPK1A-AS1	0.316516884	4.26E-13	postive

LIPT1	URB1-AS1	0.480445803	3.08E-30	postive
LIPT1	USP27X-AS1	0.446902116	6.41E-26	postive
LIPT1	USP2-AS1	0.308338787	1.79E-12	postive
LIPT1	USP30-AS1	0.300146908	7.21E-12	postive
LIPT1	USP46-AS1	0.454953949	6.50E-27	postive
LIPT1	UVRAG-DT	0.406587493	2.51E-21	postive
LIPT1	UXT-AS1	0.541006955	2.35E-39	postive
LIPT1	VASH1-AS1	0.412084464	6.44E-22	postive
LIPT1	VDAC1P8	0.366120692	2.63E-17	postive
LIPT1	VIM-AS1	0.326361069	7.14E-14	postive
LIPT1	VPS13B-DT	0.335159958	1.37E-14	postive
LIPT1	VPS33B-DT	0.433163301	2.76E-24	postive
LIPT1	WAC-AS1	0.496905922	1.54E-32	postive
LIPT1	WARS2-AS1	0.511633384	1.05E-34	postive
LIPT1	WASH3P	0.491249859	9.84E-32	postive
LIPT1	WASIR2	0.321570406	1.72E-13	postive
LIPT1	WDFY3-AS2	0.381065346	1.00E-18	postive
LIPT1	WEE2-AS1	0.430732207	5.28E-24	postive
LIPT1	WWC2-AS2	0.319524392	2.49E-13	postive
LIPT1	YTHDF3-AS1	0.383496618	5.78E-19	postive
LIPT1	Z83844.2	0.32621853	7.33E-14	postive
LIPT1	Z83851.4	0.493649525	4.50E-32	postive
LIPT1	Z84485.1	0.36654276	2.41E-17	postive
LIPT1	Z94721.1	0.319745125	2.39E-13	postive
LIPT1	Z95115.1	0.409640622	1.18E-21	postive
LIPT1	Z97055.2	0.414556662	3.46E-22	postive
LIPT1	Z97634.1	0.445590542	9.24E-26	postive
LIPT1	Z97989.1	0.393697245	5.51E-20	postive
LIPT1	Z98884.2	0.344457197	2.25E-15	postive
LIPT1	Z99572.1	0.381516979	9.04E-19	postive
LIPT1	ZBED3-AS1	0.315170452	5.41E-13	postive
LIPT1	ZBED5-AS1	0.406732955	2.42E-21	postive
LIPT1	ZBTB11-AS1	0.482536833	1.60E-30	postive
LIPT1	ZBTB44-DT	0.50411596	1.38E-33	postive
LIPT1	ZFAS1	0.454454744	7.51E-27	postive
LIPT1	ZFHX2-AS1	0.303077565	4.40E-12	postive
LIPT1	ZKSCAN2-DT	0.340063062	5.31E-15	postive
LIPT1	ZKSCAN7-AS1	0.316054075	4.63E-13	postive
LIPT1	ZNF137P	0.406258444	2.72E-21	postive
LIPT1	ZNF197-AS1	0.39035397	1.20E-19	postive
LIPT1	ZNF205-AS1	0.471148301	5.41E-29	postive
LIPT1	ZNF213-AS1	0.470756934	6.10E-29	postive
LIPT1	ZNF252P	0.467346055	1.71E-28	postive
LIPT1	ZNF271P	0.308977628	1.60E-12	postive
LIPT1	ZNF321P	0.339556792	5.86E-15	postive
LIPT1	ZNF32-AS1	0.304553095	3.43E-12	postive
LIPT1	ZNF32-AS2	0.322991275	1.33E-13	postive
LIPT1	ZNF337-AS1	0.489928866	1.51E-31	postive
LIPT1	ZNF341-AS1	0.327106993	6.22E-14	postive
LIPT1	ZNF426-DT	0.33744894	8.81E-15	postive

LIPT1	ZNF433-AS1	0.341756308	3.82E-15	postive
LIPT1	ZNF436-AS1	0.40058553	1.07E-20	postive
LIPT1	ZNF460-AS1	0.32649886	6.96E-14	postive
LIPT1	ZNF503-AS2	0.413599889	4.41E-22	postive
LIPT1	ZNF529-AS1	0.405102414	3.60E-21	postive
LIPT1	ZNF542P	0.421194405	6.40E-23	postive
LIPT1	ZNF56	0.477336254	8.11E-30	postive
LIPT1	ZNF561-AS1	0.562464779	4.70E-43	postive
LIPT1	ZNF582-AS1	0.342835934	3.09E-15	postive
LIPT1	ZNF658B	0.430648251	5.40E-24	postive
LIPT1	ZNF667-AS1	0.324124298	1.08E-13	postive
LIPT1	ZNF674-AS1	0.411540118	7.37E-22	postive
LIPT1	ZNF687-AS1	0.517806558	1.21E-35	postive
LIPT1	ZNF702P	0.38416427	4.97E-19	postive
LIPT1	ZNF767P	0.349050683	9.00E-16	postive
LIPT1	ZNF788P	0.373242038	5.66E-18	postive
LIPT1	ZNF793-AS1	0.330351124	3.40E-14	postive
LIPT1	ZNF815P	0.410221951	1.02E-21	postive
LIPT1	ZNRD1ASP	0.408425511	1.60E-21	postive
LIPT1	ZNRD2-AS1	0.526016938	6.33E-37	postive
LIPT1	ZSCAN12P1	0.324866453	9.40E-14	postive
LIPT1	ZSCAN16-AS1	0.504570804	1.19E-33	postive
LIPT2	A2M-AS1	0.321957183	1.60E-13	postive
LIPT2	AC000068.1	0.358906163	1.20E-16	postive
LIPT2	AC002116.2	0.32128533	1.81E-13	postive
LIPT2	AC002310.1	0.502840919	2.13E-33	postive
LIPT2	AC002310.2	0.324724343	9.65E-14	postive
LIPT2	AC002467.1	0.443845624	1.50E-25	postive
LIPT2	AC002470.1	0.430432853	5.72E-24	postive
LIPT2	AC003965.1	0.309432739	1.48E-12	postive
LIPT2	AC004130.2	0.31387684	6.81E-13	postive
LIPT2	AC004221.1	0.366086218	2.65E-17	postive
LIPT2	AC004241.1	0.427580189	1.22E-23	postive
LIPT2	AC004241.2	0.318528719	2.97E-13	postive
LIPT2	AC004241.3	0.456849273	3.76E-27	postive
LIPT2	AC004477.1	0.368734594	1.50E-17	postive
LIPT2	AC004596.1	0.392268661	7.69E-20	postive
LIPT2	AC004771.2	0.314420997	6.18E-13	postive
LIPT2	AC004801.6	0.312523951	8.64E-13	postive
LIPT2	AC004803.1	0.439253949	5.32E-25	postive
LIPT2	AC004812.2	0.317661431	3.47E-13	postive
LIPT2	AC004825.2	0.348304523	1.05E-15	postive
LIPT2	AC004918.1	0.330445739	3.34E-14	postive
LIPT2	AC004923.1	0.34083991	4.57E-15	postive
LIPT2	AC004943.2	0.303941622	3.80E-12	postive
LIPT2	AC005005.3	0.343332389	2.80E-15	postive
LIPT2	AC005014.2	0.327587972	5.69E-14	postive
LIPT2	AC005037.1	0.419441286	1.00E-22	postive
LIPT2	AC005076.2	0.394444354	4.62E-20	postive
LIPT2	AC005083.1	0.325509546	8.35E-14	postive

LIPT2	AC005229.4	0.534939579	2.34E-38	postive
LIPT2	AC005280.1	0.353650028	3.55E-16	postive
LIPT2	AC005324.5	0.303016864	4.45E-12	postive
LIPT2	AC005562.1	0.313885861	6.80E-13	postive
LIPT2	AC005586.1	0.366191428	2.59E-17	postive
LIPT2	AC005670.1	0.310380968	1.26E-12	postive
LIPT2	AC005696.1	0.360925346	7.89E-17	postive
LIPT2	AC005837.1	0.374434839	4.36E-18	postive
LIPT2	AC005920.1	0.300659033	6.62E-12	postive
LIPT2	AC006026.3	0.314849034	5.73E-13	postive
LIPT2	AC006252.1	0.454675191	7.05E-27	postive
LIPT2	AC006333.1	0.347945783	1.12E-15	postive
LIPT2	AC006449.2	0.314986899	5.59E-13	postive
LIPT2	AC006449.5	0.415779544	2.55E-22	postive
LIPT2	AC006449.7	0.505811497	7.78E-34	postive
LIPT2	AC006483.2	0.30314957	4.35E-12	postive
LIPT2	AC007032.1	0.346401827	1.53E-15	postive
LIPT2	AC007098.1	0.310138655	1.31E-12	postive
LIPT2	AC007114.1	0.365181758	3.22E-17	postive
LIPT2	AC007255.1	0.31867455	2.90E-13	postive
LIPT2	AC007298.2	0.307324096	2.13E-12	postive
LIPT2	AC007342.4	0.369373234	1.31E-17	postive
LIPT2	AC007365.1	0.380789035	1.06E-18	postive
LIPT2	AC007383.2	0.360568852	8.50E-17	postive
LIPT2	AC007541.1	0.444058543	1.42E-25	postive
LIPT2	AC007773.1	0.320290732	2.17E-13	postive
LIPT2	AC007881.3	0.31462877	5.96E-13	postive
LIPT2	AC008014.1	0.356569021	1.95E-16	postive
LIPT2	AC008267.5	0.445941176	8.38E-26	postive
LIPT2	AC008438.1	0.345376532	1.87E-15	postive
LIPT2	AC008443.1	0.359232878	1.12E-16	postive
LIPT2	AC008443.5	0.341182239	4.27E-15	postive
LIPT2	AC008608.2	0.365093976	3.28E-17	postive
LIPT2	AC008771.1	0.334970167	1.42E-14	postive
LIPT2	AC008915.3	0.516128594	2.18E-35	postive
LIPT2	AC009061.2	0.376394873	2.83E-18	postive
LIPT2	AC009065.9	0.312351912	8.90E-13	postive
LIPT2	AC009113.1	0.342085398	3.58E-15	postive
LIPT2	AC009118.2	0.342384545	3.38E-15	postive
LIPT2	AC009126.1	0.414453751	3.56E-22	postive
LIPT2	AC009171.2	0.399288238	1.47E-20	postive
LIPT2	AC009237.14	0.312443498	8.76E-13	postive
LIPT2	AC009309.2	0.385684659	3.52E-19	postive
LIPT2	AC009403.1	0.368162604	1.70E-17	postive
LIPT2	AC009407.1	0.350380358	6.89E-16	postive
LIPT2	AC009506.2	0.400424634	1.12E-20	postive
LIPT2	AC009690.2	0.316696374	4.13E-13	postive
LIPT2	AC009779.3	0.557129575	4.14E-42	postive
LIPT2	AC009831.1	0.304101475	3.70E-12	postive
LIPT2	AC010168.1	0.33633256	1.09E-14	postive

LIPT2	AC010173.1	0.325907825	7.76E-14	postive
LIPT2	AC010203.2	0.341580228	3.95E-15	postive
LIPT2	AC010442.1	0.319041213	2.71E-13	postive
LIPT2	AC010504.1	0.32190317	1.62E-13	postive
LIPT2	AC010531.6	0.344255787	2.34E-15	postive
LIPT2	AC010536.1	0.331879878	2.55E-14	postive
LIPT2	AC010643.1	0.434918662	1.72E-24	postive
LIPT2	AC010655.4	0.365251037	3.17E-17	postive
LIPT2	AC010761.6	0.332799201	2.14E-14	postive
LIPT2	AC010789.1	0.332887936	2.10E-14	postive
LIPT2	AC010834.2	0.368956514	1.43E-17	postive
LIPT2	AC010864.1	0.310934272	1.14E-12	postive
LIPT2	AC010883.1	0.354759266	2.83E-16	postive
LIPT2	AC010894.2	0.486848484	4.06E-31	postive
LIPT2	AC010913.1	0.346927954	1.38E-15	postive
LIPT2	AC010999.2	0.337054927	9.50E-15	postive
LIPT2	AC011603.3	0.354828231	2.79E-16	postive
LIPT2	AC012063.1	0.33780568	8.23E-15	postive
LIPT2	AC012073.1	0.32016513	2.21E-13	postive
LIPT2	AC012146.1	0.44452205	1.24E-25	postive
LIPT2	AC012184.3	0.426720294	1.52E-23	postive
LIPT2	AC012306.2	0.40943608	1.24E-21	postive
LIPT2	AC012313.9	0.368225204	1.68E-17	postive
LIPT2	AC012360.3	0.315788498	4.85E-13	postive
LIPT2	AC012442.3	0.409190399	1.32E-21	postive
LIPT2	AC012456.2	0.340123372	5.25E-15	postive
LIPT2	AC012467.2	0.352623263	4.38E-16	postive
LIPT2	AC012555.1	0.347095921	1.33E-15	postive
LIPT2	AC012640.4	0.431959346	3.81E-24	postive
LIPT2	AC013553.4	0.357388867	1.65E-16	postive
LIPT2	AC015908.3	0.329603239	3.91E-14	postive
LIPT2	AC015912.3	0.304471088	3.48E-12	postive
LIPT2	AC015917.2	0.369419245	1.30E-17	postive
LIPT2	AC015982.2	0.352861394	4.17E-16	postive
LIPT2	AC016027.1	0.35613728	2.13E-16	postive
LIPT2	AC016044.1	0.321866244	1.63E-13	postive
LIPT2	AC016065.1	0.408413526	1.60E-21	postive
LIPT2	AC016355.1	0.456699507	3.93E-27	postive
LIPT2	AC016394.2	0.435741982	1.38E-24	postive
LIPT2	AC016727.1	0.37161227	8.07E-18	postive
LIPT2	AC016747.1	0.388561754	1.82E-19	postive
LIPT2	AC016876.2	0.536660703	1.22E-38	postive
LIPT2	AC016888.1	0.377198228	2.37E-18	postive
LIPT2	AC018413.1	0.313864715	6.82E-13	postive
LIPT2	AC018521.5	0.393076413	6.37E-20	postive
LIPT2	AC018618.1	0.318654828	2.91E-13	postive
LIPT2	AC018690.1	0.326355187	7.15E-14	postive
LIPT2	AC018845.3	0.331118994	2.94E-14	postive
LIPT2	AC019069.1	0.310629455	1.20E-12	postive
LIPT2	AC019294.2	0.326141328	7.43E-14	postive

LIPT2	AC020663.2	0.413955532	4.03E-22	postive
LIPT2	AC021028.1	0.328626615	4.69E-14	postive
LIPT2	AC021087.1	0.312883493	8.11E-13	postive
LIPT2	AC021146.12	0.330239392	3.47E-14	postive
LIPT2	AC022098.3	0.306140941	2.61E-12	postive
LIPT2	AC022167.4	0.324053652	1.09E-13	postive
LIPT2	AC022364.1	0.319403397	2.54E-13	postive
LIPT2	AC022784.6	0.335249831	1.34E-14	postive
LIPT2	AC022916.2	0.327048348	6.28E-14	postive
LIPT2	AC023509.3	0.507657594	4.14E-34	postive
LIPT2	AC024243.1	0.310592128	1.21E-12	postive
LIPT2	AC024267.7	0.356760821	1.88E-16	postive
LIPT2	AC024361.2	0.401687887	8.24E-21	postive
LIPT2	AC024575.2	0.441918829	2.56E-25	postive
LIPT2	AC024884.2	0.311848248	9.72E-13	postive
LIPT2	AC025181.2	0.375906138	3.15E-18	postive
LIPT2	AC025287.2	0.345960496	1.67E-15	postive
LIPT2	AC025682.2	0.302025021	5.26E-12	postive
LIPT2	AC026304.1	0.414838126	3.23E-22	postive
LIPT2	AC026367.3	0.316675228	4.14E-13	postive
LIPT2	AC026412.1	0.345607531	1.79E-15	postive
LIPT2	AC026471.1	0.47805454	6.49E-30	postive
LIPT2	AC026471.2	0.378596084	1.74E-18	postive
LIPT2	AC026689.1	0.337416182	8.87E-15	postive
LIPT2	AC026801.2	0.366641091	2.36E-17	postive
LIPT2	AC027097.1	0.312951334	8.01E-13	postive
LIPT2	AC027237.4	0.386294996	3.06E-19	postive
LIPT2	AC027559.1	0.338143718	7.71E-15	postive
LIPT2	AC027644.3	0.368162821	1.70E-17	postive
LIPT2	AC027682.6	0.389442672	1.48E-19	postive
LIPT2	AC036176.1	0.388333559	1.92E-19	postive
LIPT2	AC040169.1	0.381375398	9.33E-19	postive
LIPT2	AC040970.1	0.310753412	1.18E-12	postive
LIPT2	AC046134.2	0.462916876	6.38E-28	postive
LIPT2	AC051619.4	0.319185762	2.64E-13	postive
LIPT2	AC053503.1	0.320378405	2.13E-13	postive
LIPT2	AC053503.4	0.330207579	3.49E-14	postive
LIPT2	AC060780.1	0.32113875	1.86E-13	postive
LIPT2	AC061992.2	0.424287387	2.87E-23	postive
LIPT2	AC062028.1	0.310797527	1.17E-12	postive
LIPT2	AC064807.1	0.510176148	1.74E-34	postive
LIPT2	AC064807.2	0.306719222	2.37E-12	postive
LIPT2	AC064807.4	0.373787635	5.02E-18	postive
LIPT2	AC064836.3	0.318500885	2.99E-13	postive
LIPT2	AC067750.1	0.318192199	3.16E-13	postive
LIPT2	AC067852.2	0.443116814	1.84E-25	postive
LIPT2	AC068338.2	0.587724469	9.06E-48	postive
LIPT2	AC068473.5	0.31226061	9.05E-13	postive
LIPT2	AC068620.1	0.358895408	1.21E-16	postive
LIPT2	AC068831.2	0.371549061	8.19E-18	postive

LIPT2	AC068831.3	0.320006835	2.28E-13	postive
LIPT2	AC068888.1	0.313490178	7.29E-13	postive
LIPT2	AC069185.1	0.300178576	7.18E-12	postive
LIPT2	AC069224.2	0.372694584	6.38E-18	postive
LIPT2	AC069281.1	0.300419728	6.89E-12	postive
LIPT2	AC069544.1	0.305362328	2.99E-12	postive
LIPT2	AC072061.1	0.331152688	2.92E-14	postive
LIPT2	AC073195.2	0.454663592	7.07E-27	postive
LIPT2	AC073508.3	0.417704713	1.56E-22	postive
LIPT2	AC073529.1	0.511312466	1.17E-34	postive
LIPT2	AC073585.1	0.328394854	4.89E-14	postive
LIPT2	AC073842.1	0.379615341	1.38E-18	postive
LIPT2	AC073896.2	0.437052329	9.69E-25	postive
LIPT2	AC073896.3	0.322302716	1.50E-13	postive
LIPT2	AC074117.1	0.337473401	8.77E-15	postive
LIPT2	AC078881.1	0.312446171	8.76E-13	postive
LIPT2	AC079174.2	0.392109097	7.98E-20	postive
LIPT2	AC079305.2	0.363092106	5.00E-17	postive
LIPT2	AC079447.1	0.332609468	2.22E-14	postive
LIPT2	AC079807.1	0.391274372	9.70E-20	postive
LIPT2	AC079848.1	0.406457869	2.59E-21	postive
LIPT2	AC079922.2	0.365103017	3.27E-17	postive
LIPT2	AC080013.5	0.32566052	8.12E-14	postive
LIPT2	AC080037.2	0.315239018	5.35E-13	postive
LIPT2	AC080129.2	0.310546555	1.22E-12	postive
LIPT2	AC083799.1	0.405911433	2.96E-21	postive
LIPT2	AC083867.2	0.303207701	4.31E-12	postive
LIPT2	AC083880.1	0.45948087	1.75E-27	postive
LIPT2	AC084036.1	0.332164766	2.41E-14	postive
LIPT2	AC084125.4	0.345951473	1.67E-15	postive
LIPT2	AC087379.2	0.314859882	5.72E-13	postive
LIPT2	AC087482.1	0.395323692	3.76E-20	postive
LIPT2	AC087623.2	0.393169757	6.23E-20	postive
LIPT2	AC087741.2	0.351449591	5.55E-16	postive
LIPT2	AC087855.2	0.302063473	5.23E-12	postive
LIPT2	AC090114.2	0.306076372	2.64E-12	postive
LIPT2	AC090164.3	0.362472878	5.70E-17	postive
LIPT2	AC090515.3	0.356113995	2.14E-16	postive
LIPT2	AC090587.1	0.579422801	3.57E-46	postive
LIPT2	AC090617.5	0.348535398	9.98E-16	postive
LIPT2	AC090912.1	0.416344528	2.21E-22	postive
LIPT2	AC090970.2	0.459636418	1.67E-27	postive
LIPT2	AC091057.1	0.324170206	1.07E-13	postive
LIPT2	AC091153.3	0.512898676	6.77E-35	postive
LIPT2	AC091153.4	0.354551289	2.95E-16	postive
LIPT2	AC091271.1	0.437046876	9.70E-25	postive
LIPT2	AC091729.3	0.44779327	4.99E-26	postive
LIPT2	AC091982.3	0.455390694	5.74E-27	postive
LIPT2	AC092115.4	0.421803303	5.47E-23	postive
LIPT2	AC092171.3	0.327539871	5.74E-14	postive

LIPT2	AC092171.5	0.428535623	9.45E-24	postive
LIPT2	AC092198.1	0.349238552	8.67E-16	postive
LIPT2	AC092287.1	0.303954276	3.80E-12	postive
LIPT2	AC092431.1	0.306421678	2.49E-12	postive
LIPT2	AC092687.3	0.347383809	1.26E-15	postive
LIPT2	AC092718.2	0.36527687	3.15E-17	postive
LIPT2	AC092718.4	0.317357114	3.67E-13	postive
LIPT2	AC092720.1	0.338315364	7.46E-15	postive
LIPT2	AC092803.1	0.465690878	2.80E-28	postive
LIPT2	AC092910.3	0.357995488	1.45E-16	postive
LIPT2	AC092941.2	0.3028923	4.54E-12	postive
LIPT2	AC092969.1	0.31061202	1.21E-12	postive
LIPT2	AC093157.1	0.38650091	2.92E-19	postive
LIPT2	AC093249.2	0.329652775	3.87E-14	postive
LIPT2	AC093249.6	0.47275101	3.32E-29	postive
LIPT2	AC093510.1	0.30005586	7.33E-12	postive
LIPT2	AC096536.2	0.360291419	9.01E-17	postive
LIPT2	AC096540.1	0.357676135	1.55E-16	postive
LIPT2	AC096649.2	0.321493485	1.74E-13	postive
LIPT2	AC096677.1	0.330326844	3.41E-14	postive
LIPT2	AC097359.2	0.304053395	3.73E-12	postive
LIPT2	AC097532.2	0.312851336	8.15E-13	postive
LIPT2	AC098484.2	0.34938417	8.42E-16	postive
LIPT2	AC098820.2	0.427026482	1.41E-23	postive
LIPT2	AC098934.1	0.413151205	4.93E-22	postive
LIPT2	AC099343.4	0.375780542	3.24E-18	postive
LIPT2	AC099518.1	0.314671608	5.92E-13	postive
LIPT2	AC099518.4	0.308294102	1.80E-12	postive
LIPT2	AC099522.2	0.370818205	9.59E-18	postive
LIPT2	AC099778.1	0.316369246	4.38E-13	postive
LIPT2	AC099791.2	0.316741586	4.09E-13	postive
LIPT2	AC099850.2	0.379630621	1.38E-18	postive
LIPT2	AC100791.2	0.309802007	1.39E-12	postive
LIPT2	AC100803.2	0.314180576	6.45E-13	postive
LIPT2	AC100810.1	0.36573428	2.86E-17	postive
LIPT2	AC100847.1	0.300678482	6.60E-12	postive
LIPT2	AC100861.1	0.318841868	2.81E-13	postive
LIPT2	AC103746.1	0.303062146	4.42E-12	postive
LIPT2	AC103760.1	0.332066565	2.46E-14	postive
LIPT2	AC103957.2	0.354601752	2.92E-16	postive
LIPT2	AC104041.1	0.336384254	1.08E-14	postive
LIPT2	AC104109.2	0.418031328	1.44E-22	postive
LIPT2	AC104135.1	0.329700336	3.84E-14	postive
LIPT2	AC104187.1	0.354630846	2.91E-16	postive
LIPT2	AC104561.1	0.311674086	1.00E-12	postive
LIPT2	AC104667.1	0.353184682	3.90E-16	postive
LIPT2	AC104667.2	0.408808034	1.45E-21	postive
LIPT2	AC105036.3	0.362763071	5.36E-17	postive
LIPT2	AC105137.2	0.353408972	3.73E-16	postive
LIPT2	AC105285.1	0.310539123	1.22E-12	postive

LIPT2	AC105942.1	0.306768582	2.35E-12	postive
LIPT2	AC106795.1	0.348566311	9.92E-16	postive
LIPT2	AC106886.3	0.32996983	3.65E-14	postive
LIPT2	AC107032.2	0.462915814	6.38E-28	postive
LIPT2	AC107068.1	0.310465079	1.24E-12	postive
LIPT2	AC107214.1	0.318056855	3.24E-13	postive
LIPT2	AC108047.1	0.331617142	2.68E-14	postive
LIPT2	AC108471.3	0.402872574	6.19E-21	postive
LIPT2	AC108488.1	0.332800404	2.14E-14	postive
LIPT2	AC108860.2	0.392906624	6.63E-20	postive
LIPT2	AC109322.1	0.460320907	1.37E-27	postive
LIPT2	AC109347.1	0.340388424	4.99E-15	postive
LIPT2	AC110609.1	0.368121539	1.72E-17	postive
LIPT2	AC114271.1	0.355221133	2.57E-16	postive
LIPT2	AC114489.2	0.315223054	5.36E-13	postive
LIPT2	AC114956.2	0.33712837	9.37E-15	postive
LIPT2	AC115618.1	0.479228089	4.50E-30	postive
LIPT2	AC115618.2	0.305084368	3.13E-12	postive
LIPT2	AC116025.2	0.355169053	2.60E-16	postive
LIPT2	AC116036.2	0.380810719	1.06E-18	postive
LIPT2	AC116913.1	0.319327086	2.58E-13	postive
LIPT2	AC117386.2	0.382425662	7.36E-19	postive
LIPT2	AC118553.1	0.390544514	1.15E-19	postive
LIPT2	AC120114.1	0.39646535	2.87E-20	postive
LIPT2	AC121338.2	0.415518668	2.72E-22	postive
LIPT2	AC121761.1	0.352339165	4.64E-16	postive
LIPT2	AC121761.2	0.322067097	1.57E-13	postive
LIPT2	AC123768.2	0.363416008	4.67E-17	postive
LIPT2	AC124016.2	0.365358774	3.10E-17	postive
LIPT2	AC124067.2	0.326692034	6.71E-14	postive
LIPT2	AC124248.1	0.399791111	1.30E-20	postive
LIPT2	AC124798.1	0.326325955	7.18E-14	postive
LIPT2	AC124947.1	0.375024327	3.83E-18	postive
LIPT2	AC124947.2	0.446859127	6.48E-26	postive
LIPT2	AC125257.1	0.395938336	3.25E-20	postive
LIPT2	AC125603.3	0.382537377	7.18E-19	postive
LIPT2	AC126283.1	0.308312685	1.80E-12	postive
LIPT2	AC126696.3	0.319379639	2.55E-13	postive
LIPT2	AC128687.2	0.331384038	2.80E-14	postive
LIPT2	AC129492.4	0.34136349	4.12E-15	postive
LIPT2	AC130343.2	0.388307629	1.93E-19	postive
LIPT2	AC131009.3	0.327026252	6.31E-14	postive
LIPT2	AC131011.2	0.381542137	8.98E-19	postive
LIPT2	AC131235.3	0.458509577	2.32E-27	postive
LIPT2	AC131235.4	0.315536072	5.07E-13	postive
LIPT2	AC132008.2	0.326189824	7.37E-14	postive
LIPT2	AC132192.2	0.33689233	9.81E-15	postive
LIPT2	AC132872.2	0.30342958	4.15E-12	postive
LIPT2	AC132938.4	0.447577777	5.30E-26	postive
LIPT2	AC133528.2	0.398592191	1.73E-20	postive

LIPT2	AC133552.2	0.314774011	5.81E-13	postive
LIPT2	AC133552.5	0.375202325	3.68E-18	postive
LIPT2	AC133785.1	0.353041925	4.02E-16	postive
LIPT2	AC135048.1	0.312456377	8.74E-13	postive
LIPT2	AC135048.3	0.440499195	3.78E-25	postive
LIPT2	AC135050.1	0.364074634	4.07E-17	postive
LIPT2	AC135050.6	0.323370647	1.24E-13	postive
LIPT2	AC136475.1	0.309763807	1.40E-12	postive
LIPT2	AC136475.2	0.30467993	3.35E-12	postive
LIPT2	AC136604.3	0.326551861	6.89E-14	postive
LIPT2	AC137630.1	0.305570615	2.88E-12	postive
LIPT2	AC138696.2	0.426512568	1.61E-23	postive
LIPT2	AC139530.1	0.374582163	4.22E-18	postive
LIPT2	AC139768.1	0.364444889	3.76E-17	postive
LIPT2	AC142472.1	0.348884795	9.31E-16	postive
LIPT2	AC144652.1	0.371469288	8.33E-18	postive
LIPT2	AC145207.1	0.339211706	6.27E-15	postive
LIPT2	AC145207.7	0.315425172	5.18E-13	postive
LIPT2	AC231981.1	0.466816003	2.00E-28	postive
LIPT2	AC244090.1	0.314927787	5.65E-13	postive
LIPT2	AC244669.1	0.334272136	1.62E-14	postive
LIPT2	AC245140.2	0.334922302	1.43E-14	postive
LIPT2	AC246793.1	0.341995474	3.65E-15	postive
LIPT2	ACVR2B-AS1	0.423999566	3.10E-23	postive
LIPT2	ADCY6-DT	0.453172572	1.08E-26	postive
LIPT2	AF131216.4	0.364598997	3.64E-17	postive
LIPT2	AF196972.1	0.359639515	1.03E-16	postive
LIPT2	AF233439.1	0.321894766	1.62E-13	postive
LIPT2	AL020996.1	0.407187631	2.16E-21	postive
LIPT2	AL021154.1	0.318016388	3.26E-13	postive
LIPT2	AL022069.1	0.351237808	5.79E-16	postive
LIPT2	AL023803.1	0.324762971	9.58E-14	postive
LIPT2	AL023803.2	0.348384348	1.03E-15	postive
LIPT2	AL024498.1	0.370754333	9.73E-18	postive
LIPT2	AL024507.3	0.493648314	4.50E-32	postive
LIPT2	AL024508.1	0.409769943	1.14E-21	postive
LIPT2	AL031055.1	0.36283989	5.28E-17	postive
LIPT2	AL031432.3	0.305892692	2.73E-12	postive
LIPT2	AL031963.3	0.336525937	1.05E-14	postive
LIPT2	AL033527.3	0.336469077	1.06E-14	postive
LIPT2	AL035587.1	0.302063823	5.23E-12	postive
LIPT2	AL049597.2	0.355458186	2.45E-16	postive
LIPT2	AL049796.1	0.349079154	8.95E-16	postive
LIPT2	AL050309.1	0.397785913	2.10E-20	postive
LIPT2	AL080250.1	0.343491583	2.72E-15	postive
LIPT2	AL096828.3	0.530148353	1.39E-37	postive
LIPT2	AL109917.1	0.437948863	7.59E-25	postive
LIPT2	AL117329.1	0.328235366	5.04E-14	postive
LIPT2	AL117332.1	0.471587493	4.74E-29	postive
LIPT2	AL117336.2	0.374381573	4.41E-18	postive

LIPT2	AL118506.1	0.378911648	1.62E-18	postive
LIPT2	AL118511.1	0.395756	3.39E-20	postive
LIPT2	AL118516.1	0.319882588	2.33E-13	postive
LIPT2	AL118558.3	0.419963656	8.78E-23	postive
LIPT2	AL121574.1	0.374513825	4.28E-18	postive
LIPT2	AL121601.1	0.350769162	6.37E-16	postive
LIPT2	AL121603.2	0.366653489	2.35E-17	postive
LIPT2	AL121832.2	0.349918018	7.56E-16	postive
LIPT2	AL121944.2	0.418070665	1.42E-22	postive
LIPT2	AL121992.1	0.318374471	3.06E-13	postive
LIPT2	AL122023.3	0.329503208	3.98E-14	postive
LIPT2	AL132639.2	0.300270328	7.07E-12	postive
LIPT2	AL132712.1	0.438686636	6.21E-25	postive
LIPT2	AL133153.2	0.360242204	9.10E-17	postive
LIPT2	AL133215.2	0.472072158	4.09E-29	postive
LIPT2	AL133216.2	0.316487085	4.28E-13	postive
LIPT2	AL133338.1	0.34822624	1.06E-15	postive
LIPT2	AL133353.1	0.392873382	6.68E-20	postive
LIPT2	AL133551.1	0.361539947	6.94E-17	postive
LIPT2	AL135744.1	0.361404918	7.14E-17	postive
LIPT2	AL135905.1	0.336012747	1.16E-14	postive
LIPT2	AL135925.1	0.311800249	9.81E-13	postive
LIPT2	AL136040.1	0.40432378	4.35E-21	postive
LIPT2	AL136162.1	0.409212244	1.31E-21	postive
LIPT2	AL136307.1	0.410672928	9.15E-22	postive
LIPT2	AL137186.2	0.30190913	5.37E-12	postive
LIPT2	AL137802.2	0.348433832	1.02E-15	postive
LIPT2	AL138762.2	0.315876645	4.78E-13	postive
LIPT2	AL139035.1	0.429362064	7.60E-24	postive
LIPT2	AL139039.3	0.404905971	3.78E-21	postive
LIPT2	AL139260.1	0.310779397	1.17E-12	postive
LIPT2	AL139289.1	0.347763788	1.17E-15	postive
LIPT2	AL139383.1	0.339535441	5.89E-15	postive
LIPT2	AL139384.1	0.390846438	1.07E-19	postive
LIPT2	AL157400.2	0.351581795	5.41E-16	postive
LIPT2	AL157904.1	0.359769874	1.00E-16	postive
LIPT2	AL158151.1	0.310066869	1.33E-12	postive
LIPT2	AL160291.1	0.303952763	3.80E-12	postive
LIPT2	AL160313.2	0.334539163	1.54E-14	postive
LIPT2	AL161421.1	0.461450629	9.82E-28	postive
LIPT2	AL161719.1	0.376361453	2.85E-18	postive
LIPT2	AL161729.1	0.452574394	1.29E-26	postive
LIPT2	AL161772.1	0.381781351	8.51E-19	postive
LIPT2	AL161937.1	0.329261191	4.16E-14	postive
LIPT2	AL162258.2	0.309339176	1.51E-12	postive
LIPT2	AL162377.1	0.349733201	7.85E-16	postive
LIPT2	AL162411.1	0.322294053	1.51E-13	postive
LIPT2	AL163051.1	0.361508544	6.98E-17	postive
LIPT2	AL353194.1	0.363399653	4.69E-17	postive
LIPT2	AL353622.1	0.303516149	4.09E-12	postive

LIPT2	AL353622.2	0.305710169	2.81E-12	postive
LIPT2	AL353708.1	0.403257331	5.64E-21	postive
LIPT2	AL353708.3	0.300236201	7.11E-12	postive
LIPT2	AL353764.1	0.339148951	6.35E-15	postive
LIPT2	AL354707.1	0.398815728	1.64E-20	postive
LIPT2	AL354824.1	0.327259666	6.04E-14	postive
LIPT2	AL354892.2	0.455031627	6.36E-27	postive
LIPT2	AL354920.1	0.427299183	1.31E-23	postive
LIPT2	AL354928.1	0.35700274	1.78E-16	postive
LIPT2	AL354993.2	0.302518826	4.84E-12	postive
LIPT2	AL355001.2	0.529550313	1.73E-37	postive
LIPT2	AL355353.2	0.316453601	4.31E-13	postive
LIPT2	AL355574.1	0.320678246	2.02E-13	postive
LIPT2	AL356488.3	0.367997957	1.76E-17	postive
LIPT2	AL358075.2	0.359319885	1.10E-16	postive
LIPT2	AL358472.2	0.340338522	5.04E-15	postive
LIPT2	AL358852.1	0.329848117	3.73E-14	postive
LIPT2	AL359091.3	0.300408424	6.91E-12	postive
LIPT2	AL359198.1	0.431803254	3.97E-24	postive
LIPT2	AL359397.1	0.322446188	1.46E-13	postive
LIPT2	AL359513.1	0.400302346	1.15E-20	postive
LIPT2	AL359643.3	0.315966654	4.70E-13	postive
LIPT2	AL359711.2	0.371564715	8.16E-18	postive
LIPT2	AL359715.3	0.367496328	1.96E-17	postive
LIPT2	AL359878.2	0.36734635	2.03E-17	postive
LIPT2	AL359962.1	0.380642449	1.10E-18	postive
LIPT2	AL359962.2	0.31186554	9.69E-13	postive
LIPT2	AL390955.2	0.368915579	1.45E-17	postive
LIPT2	AL391069.2	0.393068288	6.38E-20	postive
LIPT2	AL391069.3	0.304027075	3.75E-12	postive
LIPT2	AL391152.1	0.340876417	4.54E-15	postive
LIPT2	AL391244.1	0.358441376	1.32E-16	postive
LIPT2	AL391832.2	0.328754782	4.58E-14	postive
LIPT2	AL441992.2	0.460196069	1.42E-27	postive
LIPT2	AL442663.3	0.354495278	2.99E-16	postive
LIPT2	AL445231.1	0.466626207	2.12E-28	postive
LIPT2	AL445645.1	0.300191877	7.16E-12	postive
LIPT2	AL450326.1	0.371134373	8.96E-18	postive
LIPT2	AL450998.2	0.485654199	5.94E-31	postive
LIPT2	AL451050.2	0.334386356	1.58E-14	postive
LIPT2	AL451085.3	0.374224508	4.56E-18	postive
LIPT2	AL451165.2	0.430263005	5.99E-24	postive
LIPT2	AL512408.1	0.495626537	2.35E-32	postive
LIPT2	AL512598.1	0.406528776	2.54E-21	postive
LIPT2	AL512625.1	0.317611863	3.51E-13	postive
LIPT2	AL512625.2	0.323466899	1.22E-13	postive
LIPT2	AL512791.2	0.37384756	4.96E-18	postive
LIPT2	AL513550.1	0.424168081	2.97E-23	postive
LIPT2	AL590096.1	0.300736205	6.54E-12	postive
LIPT2	AL590617.2	0.357271001	1.69E-16	postive

LIPT2	AL592166.1	0.418241863	1.36E-22	postive
LIPT2	AL592494.2	0.346970122	1.37E-15	postive
LIPT2	AL596094.1	0.412374235	5.99E-22	postive
LIPT2	AL604028.1	0.326528844	6.92E-14	postive
LIPT2	AL606491.1	0.31156859	1.02E-12	postive
LIPT2	AL606760.1	0.376378768	2.84E-18	postive
LIPT2	AL645568.1	0.303679298	3.98E-12	postive
LIPT2	AL662791.1	0.301520503	5.73E-12	postive
LIPT2	AL691403.2	0.305798841	2.77E-12	postive
LIPT2	AL731566.2	0.335757453	1.22E-14	postive
LIPT2	AL731569.1	0.336123571	1.14E-14	postive
LIPT2	AL732314.8	0.320674953	2.02E-13	postive
LIPT2	AL928654.1	0.451498976	1.75E-26	postive
LIPT2	AMZ2P1	0.409763212	1.15E-21	postive
LIPT2	AP000251.1	0.322222587	1.53E-13	postive
LIPT2	AP000320.1	0.338137168	7.72E-15	postive
LIPT2	AP000350.5	0.305883623	2.73E-12	postive
LIPT2	AP000442.1	0.321206249	1.83E-13	postive
LIPT2	AP000487.1	0.325523683	8.33E-14	postive
LIPT2	AP000704.2	0.300315163	7.01E-12	postive
LIPT2	AP000759.1	0.309341889	1.51E-12	postive
LIPT2	AP000769.1	0.346334452	1.55E-15	postive
LIPT2	AP000781.1	0.352456599	4.53E-16	postive
LIPT2	AP000873.2	0.360313567	8.97E-17	postive
LIPT2	AP000894.4	0.374229203	4.56E-18	postive
LIPT2	AP001033.3	0.304943929	3.21E-12	postive
LIPT2	AP001160.3	0.425517751	2.09E-23	postive
LIPT2	AP001267.3	0.342525377	3.29E-15	postive
LIPT2	AP001372.2	0.751452204	5.49E-92	postive
LIPT2	AP001412.1	0.330992254	3.01E-14	postive
LIPT2	AP001453.4	0.350238525	7.09E-16	postive
LIPT2	AP001462.1	0.334865392	1.45E-14	postive
LIPT2	AP001527.2	0.348166213	1.08E-15	postive
LIPT2	AP001922.5	0.365851354	2.79E-17	postive
LIPT2	AP001922.6	0.320879815	1.95E-13	postive
LIPT2	AP001972.1	0.325865281	7.82E-14	postive
LIPT2	AP001972.4	0.311045325	1.12E-12	postive
LIPT2	AP002360.2	0.549850672	7.57E-41	postive
LIPT2	AP002387.2	0.497491905	1.27E-32	postive
LIPT2	AP002748.4	0.470331842	6.94E-29	postive
LIPT2	AP002761.1	0.306331542	2.53E-12	postive
LIPT2	AP003068.1	0.345987131	1.66E-15	postive
LIPT2	AP003119.2	0.407240605	2.14E-21	postive
LIPT2	AP003419.3	0.313287273	7.55E-13	postive
LIPT2	AP003680.1	0.3830906	6.34E-19	postive
LIPT2	AP003721.4	0.380921141	1.03E-18	postive
LIPT2	AP004609.5	0.416301917	2.23E-22	postive
LIPT2	AP005061.2	0.349274655	8.61E-16	postive
LIPT2	AP005329.2	0.417858548	1.50E-22	postive
LIPT2	AP006621.3	0.423523785	3.51E-23	postive

LIPT2	APOC1P1	0.318529054	2.97E-13	postive
LIPT2	ARHGAP31-AS1	0.352019104	4.95E-16	postive
LIPT2	ARMCX5-GPRASP2	0.371732819	7.86E-18	postive
LIPT2	ARRDC1-AS1	0.446753671	6.68E-26	postive
LIPT2	ASB16-AS1	0.328224951	5.05E-14	postive
LIPT2	ASMTL-AS1	0.324712362	9.67E-14	postive
LIPT2	ATP2A1-AS1	0.355337763	2.51E-16	postive
LIPT2	ATP6V0E2-AS1	0.321282433	1.81E-13	postive
LIPT2	ATXN2-AS	0.446816708	6.56E-26	postive
LIPT2	AZIN1-AS1	0.33192603	2.52E-14	postive
LIPT2	BAALC-AS1	0.372223484	7.07E-18	postive
LIPT2	BCAR3-AS1	0.417743583	1.55E-22	postive
LIPT2	BCDIN3D-AS1	0.413485567	4.53E-22	postive
LIPT2	BMS1P20	0.340404006	4.97E-15	postive
LIPT2	BOLA3-AS1	0.355582974	2.39E-16	postive
LIPT2	BRWD1-AS2	0.362876368	5.24E-17	postive
LIPT2	BVES-AS1	0.318154893	3.18E-13	postive
LIPT2	BX284668.2	0.40178335	8.05E-21	postive
LIPT2	BX284668.5	0.403856008	4.88E-21	postive
LIPT2	BX537318.1	0.388157081	1.99E-19	postive
LIPT2	BX539320.1	0.363067662	5.03E-17	postive
LIPT2	BX649632.1	0.372765757	6.28E-18	postive
LIPT2	C10orf25	0.400336519	1.14E-20	postive
LIPT2	C11orf72	0.31257016	8.57E-13	postive
LIPT2	C15orf56	0.347863371	1.14E-15	postive
LIPT2	C18orf65	0.306743186	2.36E-12	postive
LIPT2	C1orf229	0.33728929	9.09E-15	postive
LIPT2	C22orf46	0.327515226	5.76E-14	postive
LIPT2	C2orf48	0.382952382	6.54E-19	postive
LIPT2	C6orf99	0.507945433	3.75E-34	postive
LIPT2	C9orf163	0.32452069	1.00E-13	postive
LIPT2	CA5BP1	0.400536787	1.09E-20	postive
LIPT2	CAHM	0.33931962	6.14E-15	postive
LIPT2	CAMTA1-DT	0.363254804	4.84E-17	postive
LIPT2	CASC9	0.306162465	2.60E-12	postive
LIPT2	CATIP-AS1	0.348017626	1.11E-15	postive
LIPT2	CATSPER2P1	0.570017555	2.02E-44	postive
LIPT2	CCDC84-DT	0.362631551	5.52E-17	postive
LIPT2	CD27-AS1	0.429086762	8.17E-24	postive
LIPT2	CD99P1	0.304002761	3.76E-12	postive
LIPT2	CDC37L1-DT	0.314681671	5.90E-13	postive
LIPT2	CDIPTOSP	0.325446297	8.45E-14	postive
LIPT2	CEBPB-AS1	0.315575338	5.04E-13	postive
LIPT2	CENPBD1P1	0.339888138	5.50E-15	postive
LIPT2	CEP83-DT	0.381071519	9.99E-19	postive
LIPT2	CERNA1	0.372471995	6.70E-18	postive
LIPT2	CERNA3	0.306084865	2.64E-12	postive
LIPT2	CFL1P1	0.307426835	2.10E-12	postive
LIPT2	CHKB-DT	0.36907412	1.40E-17	postive
LIPT2	CIDECP1	0.425604395	2.04E-23	postive

LIPT2	CIRBP-AS1	0.318074298	3.23E-13	postive
LIPT2	CNNM3-DT	0.416601093	2.07E-22	postive
LIPT2	COA6-AS1	0.351124195	5.93E-16	postive
LIPT2	CPNE8-AS1	0.305692652	2.82E-12	postive
LIPT2	CR381653.1	0.349839813	7.68E-16	postive
LIPT2	CRTC3-AS1	0.379083726	1.56E-18	postive
LIPT2	CRYBB2P1	0.35540697	2.48E-16	postive
LIPT2	CSE1L-AS1	0.379532157	1.41E-18	postive
LIPT2	CTA-989H11.1	0.403587597	5.21E-21	postive
LIPT2	CTBP1-DT	0.455344755	5.81E-27	postive
LIPT2	CTD-2134A5.3	0.394399921	4.67E-20	postive
LIPT2	CTD-2521M24.9	0.336943296	9.71E-15	postive
LIPT2	CYP51A1-AS1	0.373821804	4.99E-18	postive
LIPT2	DANCR	0.412567916	5.71E-22	postive
LIPT2	DARS-AS1	0.377752235	2.10E-18	postive
LIPT2	DBIL5P	0.429090553	8.16E-24	postive
LIPT2	DCST1-AS1	0.356927516	1.81E-16	postive
LIPT2	DCXR-DT	0.442398496	2.24E-25	postive
LIPT2	DDX11-AS1	0.415576948	2.68E-22	postive
LIPT2	DDX39B-AS1	0.327960219	5.31E-14	postive
LIPT2	DGUOK-AS1	0.302199332	5.11E-12	postive
LIPT2	DHCR24-DT	0.321759741	1.66E-13	postive
LIPT2	DHRS4-AS1	0.38747412	2.33E-19	postive
LIPT2	DHRSX-IT1	0.327224535	6.08E-14	postive
LIPT2	DICER1-AS1	0.318042518	3.24E-13	postive
LIPT2	DLG1-AS1	0.395038258	4.02E-20	postive
LIPT2	DLG5-AS1	0.463899564	4.77E-28	postive
LIPT2	DLGAP1-AS1	0.343371068	2.78E-15	postive
LIPT2	DNAJC3-DT	0.311637973	1.01E-12	postive
LIPT2	DPY19L2P3	0.313321794	7.51E-13	postive
LIPT2	DRAIC	0.380239292	1.20E-18	postive
LIPT2	DYRK3-AS1	0.445629043	9.14E-26	postive
LIPT2	EIF1B-AS1	0.317774129	3.40E-13	postive
LIPT2	EIF2AK3-DT	0.424828676	2.50E-23	postive
LIPT2	EIF3J-DT	0.42311811	3.89E-23	postive
LIPT2	ELF3-AS1	0.502583	2.32E-33	postive
LIPT2	ELOA-AS1	0.444534887	1.24E-25	postive
LIPT2	EMC1-AS1	0.417971752	1.46E-22	postive
LIPT2	ENO1-AS1	0.47838954	5.85E-30	postive
LIPT2	ENTPD3-AS1	0.377586501	2.17E-18	postive
LIPT2	EXOC3-AS1	0.315842089	4.81E-13	postive
LIPT2	EXOSC10-AS1	0.452961464	1.15E-26	postive
LIPT2	FAM111A-DT	0.355438847	2.46E-16	postive
LIPT2	FAM215A	0.319058945	2.70E-13	postive
LIPT2	FAM21FP	0.306741195	2.36E-12	postive
LIPT2	FAM86C2P	0.458610685	2.26E-27	postive
LIPT2	FAM86EP	0.393209117	6.18E-20	postive
LIPT2	FAM86JP	0.506540875	6.06E-34	postive
LIPT2	FBXL19-AS1	0.335543367	1.27E-14	postive
LIPT2	FGD5-AS1	0.37317152	5.75E-18	postive

LIPT2	FLVCR1-DT	0.316462721	4.30E-13	postive
LIPT2	FMR1-AS1	0.34534125	1.89E-15	postive
LIPT2	FO393418.1	0.307383335	2.11E-12	postive
LIPT2	FOXD3-AS1	0.322818738	1.37E-13	postive
LIPT2	FOXP4-AS1	0.342973347	3.01E-15	postive
LIPT2	FRG1HP	0.358514767	1.30E-16	postive
LIPT2	FZD4-DT	0.405916616	2.95E-21	postive
LIPT2	GASAL1	0.385899306	3.35E-19	postive
LIPT2	GCC2-AS1	0.406197704	2.76E-21	postive
LIPT2	GIHCG	0.377373288	2.28E-18	postive
LIPT2	GLIDR	0.375901772	3.16E-18	postive
LIPT2	GMDS-DT	0.311608508	1.01E-12	postive
LIPT2	GPR176-DT	0.393174288	6.23E-20	postive
LIPT2	GTSE1-DT	0.350603909	6.59E-16	postive
LIPT2	GYG2P1	0.364050565	4.09E-17	postive
LIPT2	H2AC10P	0.305491528	2.92E-12	postive
LIPT2	H2AZ2P1	0.324313864	1.04E-13	postive
LIPT2	HCG15	0.343995965	2.46E-15	postive
LIPT2	HCG25	0.402180387	7.32E-21	postive
LIPT2	HDAC4-AS1	0.339650964	5.76E-15	postive
LIPT2	HEIH	0.481229486	2.41E-30	postive
LIPT2	HM13-AS1	0.344648494	2.16E-15	postive
LIPT2	HMGB1P5	0.326115767	7.47E-14	postive
LIPT2	ID2-AS1	0.315616012	5.00E-13	postive
LIPT2	IDH1-AS1	0.527923327	3.15E-37	postive
LIPT2	IL6R-AS1	0.321437693	1.76E-13	postive
LIPT2	IQCH-AS1	0.459961099	1.52E-27	postive
LIPT2	ITFG2-AS1	0.510518895	1.55E-34	postive
LIPT2	JPX	0.334226904	1.63E-14	postive
LIPT2	KCNMB2-AS1	0.360994253	7.78E-17	postive
LIPT2	KCTD21-AS1	0.486359683	4.75E-31	postive
LIPT2	KDM2B-DT	0.307823889	1.96E-12	postive
LIPT2	KDM4A-AS1	0.333047032	2.04E-14	postive
LIPT2	KDM7A-DT	0.301356718	5.89E-12	postive
LIPT2	KMT2E-AS1	0.40504133	3.66E-21	postive
LIPT2	KRT18P59	0.361028763	7.72E-17	postive
LIPT2	KRT8P12	0.341416478	4.08E-15	postive
LIPT2	KTN1-AS1	0.394146995	4.96E-20	postive
LIPT2	LAMTOR5-AS1	0.38883763	1.71E-19	postive
LIPT2	LBX2-AS1	0.397294057	2.36E-20	postive
LIPT2	LGALS8-AS1	0.471470099	4.91E-29	postive
LIPT2	LIFR-AS1	0.352114897	4.85E-16	postive
LIPT2	LINC00106	0.300299604	7.03E-12	postive
LIPT2	LINC00167	0.368715764	1.51E-17	postive
LIPT2	LINC00173	0.300477341	6.83E-12	postive
LIPT2	LINC00235	0.338659446	6.98E-15	postive
LIPT2	LINC00239	0.316384701	4.36E-13	postive
LIPT2	LINC00337	0.331209438	2.89E-14	postive
LIPT2	LINC00339	0.484484805	8.62E-31	postive
LIPT2	LINC00449	0.407792373	1.86E-21	postive

LIPT2	LINC00467	0.388114948	2.01E-19	postive
LIPT2	LINC00471	0.430667587	5.38E-24	postive
LIPT2	LINC00476	0.346418566	1.52E-15	postive
LIPT2	LINC00511	0.306968362	2.27E-12	postive
LIPT2	LINC00602	0.337096762	9.43E-15	postive
LIPT2	LINC00605	0.314974548	5.61E-13	postive
LIPT2	LINC00623	0.333943869	1.72E-14	postive
LIPT2	LINC00654	0.400166401	1.19E-20	postive
LIPT2	LINC00665	0.32624519	7.29E-14	postive
LIPT2	LINC00680	0.315437682	5.16E-13	postive
LIPT2	LINC00847	0.419850849	9.03E-23	postive
LIPT2	LINC00853	0.390399501	1.19E-19	postive
LIPT2	LINC00863	0.313919361	6.76E-13	postive
LIPT2	LINC00869	0.366724751	2.31E-17	postive
LIPT2	LINC00884	0.462300345	7.65E-28	postive
LIPT2	LINC00888	0.464020941	4.60E-28	postive
LIPT2	LINC00898	0.308994554	1.60E-12	postive
LIPT2	LINC00900	0.332376635	2.32E-14	postive
LIPT2	LINC00909	0.426960913	1.43E-23	postive
LIPT2	LINC00910	0.324290548	1.05E-13	postive
LIPT2	LINC00963	0.476027459	1.22E-29	postive
LIPT2	LINC01006	0.346015296	1.65E-15	postive
LIPT2	LINC01023	0.339750273	5.65E-15	postive
LIPT2	LINC01063	0.414141375	3.85E-22	postive
LIPT2	LINC01124	0.379786557	1.33E-18	postive
LIPT2	LINC01132	0.301379508	5.87E-12	postive
LIPT2	LINC01136	0.373360253	5.52E-18	postive
LIPT2	LINC01138	0.306346892	2.52E-12	postive
LIPT2	LINC01144	0.337902263	8.07E-15	postive
LIPT2	LINC01184	0.389761351	1.38E-19	postive
LIPT2	LINC01220	0.366719448	2.32E-17	postive
LIPT2	LINC01232	0.347353199	1.26E-15	postive
LIPT2	LINC01270	0.458199061	2.54E-27	postive
LIPT2	LINC01271	0.450104394	2.60E-26	postive
LIPT2	LINC01273	0.452222689	1.42E-26	postive
LIPT2	LINC01275	0.336817391	9.95E-15	postive
LIPT2	LINC01278	0.345177329	1.95E-15	postive
LIPT2	LINC01285	0.310127761	1.31E-12	postive
LIPT2	LINC01348	0.425317103	2.20E-23	postive
LIPT2	LINC01410	0.357153462	1.73E-16	postive
LIPT2	LINC01431	0.416722142	2.00E-22	postive
LIPT2	LINC01465	0.375121009	3.75E-18	postive
LIPT2	LINC01509	0.38312092	6.29E-19	postive
LIPT2	LINC01521	0.396506152	2.84E-20	postive
LIPT2	LINC01560	0.45165143	1.67E-26	postive
LIPT2	LINC01569	0.317900026	3.33E-13	postive
LIPT2	LINC01607	0.32128812	1.81E-13	postive
LIPT2	LINC01635	0.310601265	1.21E-12	postive
LIPT2	LINC01639	0.301253062	5.99E-12	postive
LIPT2	LINC01647	0.342651986	3.21E-15	postive

LIPT2	LINC01686	0.32792051	5.35E-14	postive
LIPT2	LINC01703	0.362550148	5.61E-17	postive
LIPT2	LINC01715	0.348484205	1.01E-15	postive
LIPT2	LINC01719	0.431034614	4.88E-24	postive
LIPT2	LINC01770	0.426136412	1.77E-23	postive
LIPT2	LINC01806	0.35740374	1.64E-16	postive
LIPT2	LINC01814	0.332693425	2.18E-14	postive
LIPT2	LINC01816	0.49676192	1.62E-32	postive
LIPT2	LINC01843	0.323142312	1.29E-13	postive
LIPT2	LINC01887	0.300477595	6.83E-12	postive
LIPT2	LINC01952	0.372004128	7.41E-18	postive
LIPT2	LINC01977	0.323036145	1.31E-13	postive
LIPT2	LINC02014	0.44297712	1.91E-25	postive
LIPT2	LINC02026	0.366298066	2.54E-17	postive
LIPT2	LINC02028	0.426182032	1.75E-23	postive
LIPT2	LINC02197	0.305361339	2.99E-12	postive
LIPT2	LINC02308	0.302946197	4.50E-12	postive
LIPT2	LINC02370	0.321003446	1.90E-13	postive
LIPT2	LINC02387	0.324985027	9.20E-14	postive
LIPT2	LINC02453	0.417614158	1.60E-22	postive
LIPT2	LINC02482	0.459651698	1.66E-27	postive
LIPT2	LINC02579	0.39320043	6.19E-20	postive
LIPT2	LINC02585	0.322056114	1.57E-13	postive
LIPT2	LINC02601	0.455383127	5.75E-27	postive
LIPT2	LINC02637	0.338929564	6.62E-15	postive
LIPT2	LINC02671	0.316678928	4.14E-13	postive
LIPT2	LINC02701	0.42152221	5.88E-23	postive
LIPT2	LINC02709	0.426941496	1.44E-23	postive
LIPT2	LINC02749	0.433422434	2.58E-24	postive
LIPT2	LINC02762	0.373589356	5.25E-18	postive
LIPT2	LINC02763	0.301767047	5.50E-12	postive
LIPT2	LINC02766	0.318964401	2.75E-13	postive
LIPT2	LINC02777	0.325809749	7.90E-14	postive
LIPT2	LINC02846	0.314926695	5.65E-13	postive
LIPT2	LMCD1-AS1	0.374992935	3.86E-18	postive
LIPT2	LNCTAM34A	0.324108164	1.08E-13	postive
LIPT2	LNX1-AS2	0.329565444	3.93E-14	postive
LIPT2	LOH12CR2	0.457465785	3.15E-27	postive
LIPT2	LOXL1-AS1	0.336771088	1.00E-14	postive
LIPT2	LPP-AS2	0.381835628	8.41E-19	postive
LIPT2	MAFG-DT	0.40403781	4.67E-21	postive
LIPT2	MAFTRR	0.307273207	2.15E-12	postive
LIPT2	MAN1B1-DT	0.351157747	5.89E-16	postive
LIPT2	MAP3K2-DT	0.400030645	1.23E-20	postive
LIPT2	MAP4K3-DT	0.319390021	2.55E-13	postive
LIPT2	MAPK6-DT	0.455333109	5.83E-27	postive
LIPT2	MAPKAPK5-AS1	0.50318727	1.89E-33	postive
LIPT2	MELTF-AS1	0.405424381	3.33E-21	postive
LIPT2	MHENCR	0.353298206	3.81E-16	postive
LIPT2	MID1IP1-AS1	0.341788566	3.80E-15	postive

LIPT2	MINCR	0.366666204	2.34E-17	postive
LIPT2	MIPEPP3	0.357172405	1.72E-16	postive
LIPT2	MIR200CHG	0.344515787	2.22E-15	postive
LIPT2	MIR548XHG	0.305313647	3.01E-12	postive
LIPT2	MIR762HG	0.476616128	1.01E-29	postive
LIPT2	MKLN1-AS	0.314487892	6.11E-13	postive
LIPT2	MRPL20-AS1	0.472841846	3.23E-29	postive
LIPT2	MSH5-SAPCD1	0.416318869	2.22E-22	postive
LIPT2	MYLK-AS1	0.35483464	2.79E-16	postive
LIPT2	NACA4P	0.368900798	1.45E-17	postive
LIPT2	NBR2	0.402693319	6.47E-21	postive
LIPT2	NCK1-DT	0.368282627	1.66E-17	postive
LIPT2	NDUFA6-DT	0.313574328	7.18E-13	postive
LIPT2	NDUFB2-AS1	0.415090942	3.03E-22	postive
LIPT2	NEBL-AS1	0.361597079	6.86E-17	postive
LIPT2	NIFK-AS1	0.463359133	5.59E-28	postive
LIPT2	NINJ2-AS1	0.383396129	5.91E-19	postive
LIPT2	NNT-AS1	0.418670019	1.22E-22	postive
LIPT2	NOP14-AS1	0.388049884	2.04E-19	postive
LIPT2	NPIPP1	0.324523535	1.00E-13	postive
LIPT2	NRAV	0.492137915	7.37E-32	postive
LIPT2	NRSN2-AS1	0.485332747	6.58E-31	postive
LIPT2	NUDT16P1	0.43527078	1.57E-24	postive
LIPT2	NUP50-DT	0.377124896	2.41E-18	postive
LIPT2	NUTM2A-AS1	0.366459673	2.45E-17	postive
LIPT2	ODC1-DT	0.346072706	1.63E-15	postive
LIPT2	OGFRP1	0.355615387	2.37E-16	postive
LIPT2	OLMALINC	0.527694531	3.43E-37	postive
LIPT2	OR7E14P	0.308315074	1.80E-12	postive
LIPT2	OSER1-DT	0.448333466	4.28E-26	postive
LIPT2	OSGEPL1-AS1	0.331527191	2.72E-14	postive
LIPT2	OTUD6B-AS1	0.337950562	8.00E-15	postive
LIPT2	PARD3-AS1	0.312414275	8.81E-13	postive
LIPT2	PAXIP1-AS1	0.362473346	5.70E-17	postive
LIPT2	PCAT6	0.345613292	1.79E-15	postive
LIPT2	PCBP1-AS1	0.459239278	1.88E-27	postive
LIPT2	PDCD4-AS1	0.461317008	1.02E-27	postive
LIPT2	PDIA3P1	0.367609406	1.92E-17	postive
LIPT2	PINK1-AS	0.353962902	3.33E-16	postive
LIPT2	PITPNA-AS1	0.524564134	1.07E-36	postive
LIPT2	PLBD1-AS1	0.340617762	4.77E-15	postive
LIPT2	PLEKHA8P1	0.320698314	2.01E-13	postive
LIPT2	PMS2CL	0.321189537	1.84E-13	postive
LIPT2	PMS2P4	0.352935161	4.11E-16	postive
LIPT2	POLR2J4	0.393370716	5.95E-20	postive
LIPT2	PPP1R35-AS1	0.302172927	5.13E-12	postive
LIPT2	PPY2P	0.306123301	2.62E-12	postive
LIPT2	PRC1-AS1	0.383785931	5.41E-19	postive
LIPT2	PRKAG2-AS1	0.371123636	8.98E-18	postive
LIPT2	PRKAR2A-AS1	0.417167284	1.79E-22	postive

LIPT2	PRORSD1P	0.322425417	1.47E-13	postive
LIPT2	PXN-AS1	0.531286295	9.13E-38	postive
LIPT2	RAB11B-AS1	0.356150227	2.13E-16	postive
LIPT2	RAB30-DT	0.559089908	1.87E-42	postive
LIPT2	RABGAP1L-DT	0.316837209	4.03E-13	postive
LIPT2	RALY-AS1	0.439109352	5.53E-25	postive
LIPT2	RARA-AS1	0.353108921	3.96E-16	postive
LIPT2	RASSF1-AS1	0.3811057	9.91E-19	postive
LIPT2	RBM15-AS1	0.450075903	2.62E-26	postive
LIPT2	RBM26-AS1	0.323341001	1.24E-13	postive
LIPT2	RDM1P5	0.326893066	6.47E-14	postive
LIPT2	RHPN1-AS1	0.398694895	1.69E-20	postive
LIPT2	RN7SL832P	0.317315297	3.70E-13	postive
LIPT2	RNASEH1-AS1	0.507269114	4.73E-34	postive
LIPT2	RNFT1-DT	0.316605715	4.20E-13	postive
LIPT2	ROCK1P1	0.33977005	5.63E-15	postive
LIPT2	RP11-10A14.4	0.395667563	3.46E-20	postive
LIPT2	RP11-111M22.4	0.324462407	1.01E-13	postive
LIPT2	RP11-199F11.2	0.319171159	2.65E-13	postive
LIPT2	RP11-254F7.4	0.369084497	1.40E-17	postive
LIPT2	RP11-282O18.3	0.312642047	8.46E-13	postive
LIPT2	RP11-314C16.1	0.372929773	6.06E-18	postive
LIPT2	RP11-435O5.2	0.36520811	3.20E-17	postive
LIPT2	RP11-61E11.2	0.305787401	2.78E-12	postive
LIPT2	RP11-658F2.8	0.30145263	5.79E-12	postive
LIPT2	RP11-98G7.1	0.403807905	4.94E-21	postive
LIPT2	RP13-476E20.1	0.379415965	1.45E-18	postive
LIPT2	RP4-545L17.11	0.357672362	1.55E-16	postive
LIPT2	RP9P	0.420326169	8.00E-23	postive
LIPT2	RPARP-AS1	0.380037271	1.26E-18	postive
LIPT2	RPL23AP7	0.343419547	2.76E-15	postive
LIPT2	RPL23AP82	0.36770976	1.87E-17	postive
LIPT2	RPLP1P6	0.414427314	3.58E-22	postive
LIPT2	RPS27P25	0.468462211	1.22E-28	postive
LIPT2	SAMD12-AS1	0.312085691	9.33E-13	postive
LIPT2	SAPCD1-AS1	0.39311731	6.31E-20	postive
LIPT2	SBDSP1	0.305866703	2.74E-12	postive
LIPT2	SBF2-AS1	0.498414813	9.36E-33	postive
LIPT2	SBNO1-AS1	0.460294552	1.38E-27	postive
LIPT2	SCAMP1-AS1	0.39853773	1.75E-20	postive
LIPT2	SCAND2P	0.300580166	6.71E-12	postive
LIPT2	SCAT8	0.30394575	3.80E-12	postive
LIPT2	SDAD1P1	0.302945949	4.50E-12	postive
LIPT2	SDCBP2-AS1	0.3141097	6.53E-13	postive
LIPT2	SEC24B-AS1	0.312973934	7.98E-13	postive
LIPT2	SEPSECS-AS1	0.446945986	6.33E-26	postive
LIPT2	SEPTIN4-AS1	0.402051491	7.55E-21	postive
LIPT2	SERHL	0.310303363	1.27E-12	postive
LIPT2	SGMS1-AS1	0.329602268	3.91E-14	postive
LIPT2	SLC25A25-AS1	0.301791741	5.47E-12	postive

LIPT2	SLC25A5-AS1	0.321544994	1.72E-13	postive
LIPT2	SMG7-AS1	0.33226082	2.37E-14	postive
LIPT2	SNAI3-AS1	0.310340021	1.27E-12	postive
LIPT2	SNHG1	0.319179865	2.65E-13	postive
LIPT2	SNHG10	0.426413204	1.65E-23	postive
LIPT2	SNHG11	0.553466613	1.80E-41	postive
LIPT2	SNHG17	0.390236635	1.23E-19	postive
LIPT2	SNHG21	0.434031378	2.19E-24	postive
LIPT2	SNHG25	0.301521731	5.73E-12	postive
LIPT2	SNHG28	0.341153665	4.30E-15	postive
LIPT2	SNHG30	0.348242787	1.06E-15	postive
LIPT2	SNHG6	0.336342161	1.09E-14	postive
LIPT2	SOX2-OT	0.344594087	2.19E-15	postive
LIPT2	SPATA41	0.310588729	1.21E-12	postive
LIPT2	SREBF2-AS1	0.360812817	8.08E-17	postive
LIPT2	SRP14-AS1	0.388016329	2.06E-19	postive
LIPT2	SSR4P1	0.332087335	2.45E-14	postive
LIPT2	STAG3L4	0.368753873	1.50E-17	postive
LIPT2	STARD7-AS1	0.465366601	3.08E-28	postive
LIPT2	STAU2-AS1	0.391135742	1.00E-19	postive
LIPT2	STIM2-AS1	0.302413703	4.93E-12	postive
LIPT2	STK4-AS1	0.483486609	1.18E-30	postive
LIPT2	STX17-AS1	0.318063911	3.23E-13	postive
LIPT2	STX18-AS1	0.341926861	3.69E-15	postive
LIPT2	SUCLA2-AS1	0.407903479	1.81E-21	postive
LIPT2	SYS1-DBNDD2	0.358790736	1.23E-16	postive
LIPT2	TAF1A-AS1	0.461083683	1.09E-27	postive
LIPT2	TBC1D8-AS1	0.393573935	5.67E-20	postive
LIPT2	THAP7-AS1	0.429218186	7.89E-24	postive
LIPT2	THRB-AS1	0.346097357	1.62E-15	postive
LIPT2	TIPARP-AS1	0.417674386	1.57E-22	postive
LIPT2	TMC3-AS1	0.475669637	1.36E-29	postive
LIPT2	TMCC1-AS1	0.369868575	1.18E-17	postive
LIPT2	TMED2-DT	0.316571077	4.22E-13	postive
LIPT2	TMEM202-AS1	0.463210698	5.84E-28	postive
LIPT2	TMEM220-AS1	0.343430665	2.75E-15	postive
LIPT2	TMEM44-AS1	0.500102642	5.33E-33	postive
LIPT2	TMEM99	0.371878519	7.62E-18	postive
LIPT2	TMPO-AS1	0.538079122	7.16E-39	postive
LIPT2	TOB1-AS1	0.509594436	2.13E-34	postive
LIPT2	TOLLIP-AS1	0.333997944	1.71E-14	postive
LIPT2	TP53TG1	0.302307048	5.02E-12	postive
LIPT2	TPM3P9	0.31678951	4.06E-13	postive
LIPT2	TRAF3IP2-AS1	0.314785637	5.80E-13	postive
LIPT2	TRAM2-AS1	0.482020209	1.88E-30	postive
LIPT2	TRIM52-AS1	0.375008086	3.84E-18	postive
LIPT2	TSPY26P	0.339193216	6.29E-15	postive
LIPT2	TSTD3	0.321954369	1.60E-13	postive
LIPT2	TTC28-AS1	0.357985443	1.46E-16	postive
LIPT2	TTC39A-AS1	0.383009888	6.45E-19	postive

LIPT2	TTC41P	0.443222687	1.79E-25	postive
LIPT2	TUG1	0.305145995	3.10E-12	postive
LIPT2	TUSC8	0.308581704	1.72E-12	postive
LIPT2	TYMSOS	0.461494999	9.70E-28	postive
LIPT2	U47924.2	0.403099002	5.86E-21	postive
LIPT2	U91328.1	0.337189906	9.26E-15	postive
LIPT2	UBA6-AS1	0.440699442	3.58E-25	postive
LIPT2	UBAC2-AS1	0.418599379	1.24E-22	postive
LIPT2	UBBP4	0.355636936	2.36E-16	postive
LIPT2	UBE2D3-AS1	0.381062655	1.00E-18	postive
LIPT2	UBL7-AS1	0.427007495	1.41E-23	postive
LIPT2	UGDH-AS1	0.398771988	1.66E-20	postive
LIPT2	URB1-AS1	0.431430908	4.39E-24	postive
LIPT2	USP27X-AS1	0.42686035	1.47E-23	postive
LIPT2	USP46-AS1	0.457311102	3.29E-27	postive
LIPT2	UVRAG-DT	0.452442038	1.34E-26	postive
LIPT2	UXT-AS1	0.484844731	7.69E-31	postive
LIPT2	VLDLR-AS1	0.326425836	7.05E-14	postive
LIPT2	VPS13B-DT	0.382444091	7.33E-19	postive
LIPT2	VPS33B-DT	0.394764168	4.29E-20	postive
LIPT2	WAC-AS1	0.39366346	5.55E-20	postive
LIPT2	WARS2-AS1	0.304987293	3.18E-12	postive
LIPT2	WASH3P	0.38158506	8.90E-19	postive
LIPT2	WEE2-AS1	0.330621496	3.23E-14	postive
LIPT2	WWTR1-AS1	0.439568176	4.88E-25	postive
LIPT2	YTHDF3-AS1	0.397325879	2.34E-20	postive
LIPT2	Z73429.1	0.335634202	1.25E-14	postive
LIPT2	Z83851.4	0.432358046	3.43E-24	postive
LIPT2	Z84485.1	0.354726884	2.85E-16	postive
LIPT2	Z92544.1	0.352045547	4.92E-16	postive
LIPT2	Z95115.1	0.384326924	4.79E-19	postive
LIPT2	Z97634.1	0.354696336	2.87E-16	postive
LIPT2	Z99572.1	0.421521802	5.88E-23	postive
LIPT2	Z99943.1	0.314733558	5.85E-13	postive
LIPT2	ZBED3-AS1	0.365068402	3.29E-17	postive
LIPT2	ZBED5-AS1	0.360796196	8.11E-17	postive
LIPT2	ZBTB11-AS1	0.364134078	4.02E-17	postive
LIPT2	ZNF213-AS1	0.304827416	3.27E-12	postive
LIPT2	ZNF252P	0.383096338	6.33E-19	postive
LIPT2	ZNF337-AS1	0.319042948	2.71E-13	postive
LIPT2	ZNF426-DT	0.343962509	2.48E-15	postive
LIPT2	ZNF503-AS2	0.428521312	9.49E-24	postive
LIPT2	ZNF561-AS1	0.436994891	9.84E-25	postive
LIPT2	ZNF674-AS1	0.353282686	3.83E-16	postive
LIPT2	ZNF687-AS1	0.475728619	1.33E-29	postive
LIPT2	ZNRD2-AS1	0.410053495	1.07E-21	postive
LIPT2	ZPLD2P	0.37622863	2.94E-18	postive
LIPT2	ZSCAN16-AS1	0.344187839	2.37E-15	postive
LOX	A2M-AS1	0.32429953	1.04E-13	postive
LOX	ABALON	0.30782326	1.96E-12	postive

LOX	AC000403.1	0.393869473	5.29E-20	postive
LOX	AC004160.1	0.384043912	5.11E-19	postive
LOX	AC006126.4	0.313248577	7.60E-13	postive
LOX	AC007998.3	0.46034931	1.36E-27	postive
LOX	AC008742.1	0.353241892	3.86E-16	postive
LOX	AC009093.1	0.49907742	7.51E-33	postive
LOX	AC009093.2	0.339646031	5.76E-15	postive
LOX	AC009509.5	0.37620498	2.95E-18	postive
LOX	AC009549.1	0.336851312	9.88E-15	postive
LOX	AC009831.1	0.315991545	4.68E-13	postive
LOX	AC010343.3	0.309121047	1.56E-12	postive
LOX	AC010980.2	0.407998665	1.77E-21	postive
LOX	AC011352.1	0.431795534	3.98E-24	postive
LOX	AC011352.3	0.44884208	3.71E-26	postive
LOX	AC011595.2	0.340726447	4.67E-15	postive
LOX	AC016396.2	0.327536528	5.74E-14	postive
LOX	AC023301.1	0.394928909	4.12E-20	postive
LOX	AC026310.2	0.350450823	6.79E-16	postive
LOX	AC026356.1	0.308981159	1.60E-12	postive
LOX	AC026904.2	0.312540722	8.61E-13	postive
LOX	AC027237.5	0.327437095	5.85E-14	postive
LOX	AC034199.1	0.476494635	1.05E-29	postive
LOX	AC039056.2	0.328814677	4.53E-14	postive
LOX	AC068473.4	0.34270409	3.17E-15	postive
LOX	AC073569.2	0.368166558	1.70E-17	postive
LOX	AC073611.1	0.309559262	1.45E-12	postive
LOX	AC079070.1	0.424107979	3.01E-23	postive
LOX	AC079298.3	0.476195063	1.15E-29	postive
LOX	AC090125.1	0.420566631	7.52E-23	postive
LOX	AC090559.1	0.320428668	2.11E-13	postive
LOX	AC091212.1	0.334830075	1.46E-14	postive
LOX	AC092376.2	0.517658196	1.27E-35	postive
LOX	AC093567.1	0.304300757	3.58E-12	postive
LOX	AC093627.3	0.311738108	9.91E-13	postive
LOX	AC093627.6	0.36941413	1.30E-17	postive
LOX	AC093673.1	0.368830998	1.47E-17	postive
LOX	AC093895.2	0.330090077	3.57E-14	postive
LOX	AC099066.2	0.481700254	2.08E-30	postive
LOX	AC099548.2	0.361364883	7.20E-17	postive
LOX	AC099850.4	0.338391783	7.35E-15	postive
LOX	AC100849.1	0.422443089	4.64E-23	postive
LOX	AC106881.1	0.377276666	2.33E-18	postive
LOX	AC107021.1	0.356200119	2.10E-16	postive
LOX	AC107021.2	0.476485729	1.06E-29	postive
LOX	AC108463.2	0.525716956	7.05E-37	postive
LOX	AC109479.1	0.398881879	1.62E-20	postive
LOX	AC112715.1	0.453329421	1.04E-26	postive
LOX	AC112721.1	0.482268272	1.74E-30	postive
LOX	AC112721.2	0.434349171	2.01E-24	postive
LOX	AC113346.1	0.487627242	3.16E-31	postive

LOX	AC125807.2	0.313205631	7.66E-13	postive
LOX	AC134312.5	0.550507266	5.84E-41	postive
LOX	AC147651.4	0.312771637	8.27E-13	postive
LOX	ACTA2-AS1	0.364345174	3.84E-17	postive
LOX	AF015262.1	0.385569556	3.61E-19	postive
LOX	AF127577.4	0.32402847	1.10E-13	postive
LOX	AJ011932.1	0.300281454	7.05E-12	postive
LOX	AL009178.2	0.340555122	4.83E-15	postive
LOX	AL035409.1	0.328624459	4.69E-14	postive
LOX	AL049833.1	0.365987739	2.71E-17	postive
LOX	AL078581.2	0.318571997	2.95E-13	postive
LOX	AL078644.2	0.393087971	6.35E-20	postive
LOX	AL109809.1	0.356425202	2.01E-16	postive
LOX	AL109924.1	0.462117888	8.07E-28	postive
LOX	AL122035.1	0.373026439	5.93E-18	postive
LOX	AL133163.2	0.365408244	3.06E-17	postive
LOX	AL133371.2	0.300924362	6.33E-12	postive
LOX	AL136084.3	0.520119479	5.30E-36	postive
LOX	AL136987.1	0.434151657	2.12E-24	postive
LOX	AL139339.1	0.365030313	3.32E-17	postive
LOX	AL139351.1	0.392585144	7.15E-20	postive
LOX	AL139393.3	0.39722401	2.40E-20	postive
LOX	AL162457.2	0.428529759	9.47E-24	postive
LOX	AL354811.1	0.496287514	1.89E-32	postive
LOX	AL354919.2	0.392783886	6.82E-20	postive
LOX	AL356414.1	0.431049551	4.86E-24	postive
LOX	AL391832.3	0.403203864	5.71E-21	postive
LOX	AL596087.2	0.339119721	6.38E-15	postive
LOX	AL683807.2	0.300228343	7.12E-12	postive
LOX	AL691447.2	0.421520841	5.88E-23	postive
LOX	AP000695.1	0.627901312	3.49E-56	postive
LOX	AP000695.2	0.509390111	2.28E-34	postive
LOX	AP000759.1	0.328790314	4.55E-14	postive
LOX	AP001107.5	0.383697648	5.52E-19	postive
LOX	AP001189.3	0.352077676	4.89E-16	postive
LOX	AP001330.4	0.401892778	7.84E-21	postive
LOX	AP001434.1	0.510827287	1.39E-34	postive
LOX	AP001471.1	0.373719855	5.10E-18	postive
LOX	AP002414.2	0.326158894	7.41E-14	postive
LOX	AP002989.1	0.312533198	8.62E-13	postive
LOX	AP003071.3	0.331740246	2.61E-14	postive
LOX	AP003071.4	0.451716941	1.64E-26	postive
LOX	BNC2-AS1	0.309713731	1.41E-12	postive
LOX	BX284613.2	0.313770728	6.94E-13	postive
LOX	BX322234.1	0.420197708	8.27E-23	postive
LOX	C2orf27A	0.514732399	3.57E-35	postive
LOX	CARD8-AS1	0.327752085	5.52E-14	postive
LOX	CT75	0.35628169	2.07E-16	postive
LOX	CTC-786C10.1	0.445654111	9.08E-26	postive
LOX	CXXC1P1	0.405696936	3.12E-21	postive

LOX	CYTOR	0.349851404	7.67E-16	postive
LOX	DNM3OS	0.307477831	2.08E-12	postive
LOX	FAM225B	0.33267434	2.19E-14	postive
LOX	FGD5-AS1	0.302706787	4.69E-12	postive
LOX	FNDC1-IT1	0.33392621	1.73E-14	postive
LOX	GORAB-AS1	0.407026008	2.25E-21	postive
LOX	HAS2-AS1	0.392646295	7.04E-20	postive
LOX	HECW2-AS1	0.470349727	6.90E-29	postive
LOX	HIF1A-AS1	0.402658165	6.52E-21	postive
LOX	HMGA2-AS1	0.309391478	1.49E-12	postive
LOX	IL6-AS1	0.310637606	1.20E-12	postive
LOX	INHBA-AS1	0.39512303	3.94E-20	postive
LOX	LCT-AS1	0.457460917	3.15E-27	postive
LOX	LEF1-AS1	0.474527835	1.93E-29	postive
LOX	LINC00211	0.407406456	2.05E-21	postive
LOX	LINC00460	0.324617462	9.84E-14	postive
LOX	LINC00702	0.425012861	2.38E-23	postive
LOX	LINC00880	0.32433342	1.04E-13	postive
LOX	LINC00941	0.308399509	1.77E-12	postive
LOX	LINC00973	0.35081614	6.31E-16	postive
LOX	LINC01048	0.435367358	1.53E-24	postive
LOX	LINC01050	0.354699201	2.86E-16	postive
LOX	LINC01093	0.357748041	1.53E-16	postive
LOX	LINC01094	0.487266966	3.55E-31	postive
LOX	LINC01116	0.30848983	1.74E-12	postive
LOX	LINC01117	0.302939657	4.51E-12	postive
LOX	LINC01141	0.309641915	1.43E-12	postive
LOX	LINC01142	0.34643638	1.52E-15	postive
LOX	LINC01235	0.415700342	2.60E-22	postive
LOX	LINC01283	0.308856366	1.64E-12	postive
LOX	LINC01303	0.447965951	4.75E-26	postive
LOX	LINC01429	0.485470002	6.30E-31	postive
LOX	LINC01500	0.328579414	4.73E-14	postive
LOX	LINC01505	0.387253494	2.45E-19	postive
LOX	LINC01561	0.511038971	1.29E-34	postive
LOX	LINC01583	0.379022214	1.58E-18	postive
LOX	LINC01614	0.696230602	1.01E-73	postive
LOX	LINC01615	0.375253067	3.64E-18	postive
LOX	LINC01638	0.415892593	2.47E-22	postive
LOX	LINC01705	0.452066461	1.49E-26	postive
LOX	LINC01711	0.379962601	1.28E-18	postive
LOX	LINC01907	0.345078953	1.99E-15	postive
LOX	LINC01915	0.347385809	1.26E-15	postive
LOX	LINC01929	0.42332346	3.69E-23	postive
LOX	LINC01943	0.391987294	8.22E-20	postive
LOX	LINC02015	0.367397681	2.00E-17	postive
LOX	LINC02126	0.337947224	8.00E-15	postive
LOX	LINC02345	0.308063751	1.88E-12	postive
LOX	LINC02391	0.305348994	2.99E-12	postive
LOX	LINC02408	0.410736624	9.00E-22	postive

LOX	LINC02454	0.321952866	1.60E-13	postive
LOX	LINC02521	0.300506121	6.79E-12	postive
LOX	LINC02544	0.49887198	8.04E-33	postive
LOX	LINC02550	0.300591762	6.70E-12	postive
LOX	LINC02551	0.343785952	2.57E-15	postive
LOX	LINC02611	0.301171264	6.08E-12	postive
LOX	LINC02693	0.396864583	2.61E-20	postive
LOX	LINC02798	0.318573774	2.95E-13	postive
LOX	LINC02828	0.342697142	3.18E-15	postive
LOX	LNCOG	0.317797207	3.39E-13	postive
LOX	LNCSRLR	0.363399602	4.69E-17	postive
LOX	MAGI2-AS3	0.374554264	4.25E-18	postive
LOX	MAP2K4P1	0.301913008	5.36E-12	postive
LOX	MED14OS	0.306527078	2.45E-12	postive
LOX	MIR100HG	0.362889735	5.22E-17	postive
LOX	MIR3945HG	0.330420762	3.35E-14	postive
LOX	MIR4435-2HG	0.471881345	4.33E-29	postive
LOX	MRPS31P4	0.325218927	8.81E-14	postive
LOX	MSC-AS1	0.573327643	4.95E-45	postive
LOX	MT1L	0.444150711	1.38E-25	postive
LOX	MYHAS	0.307047018	2.24E-12	postive
LOX	NAIPP1	0.316339141	4.40E-13	postive
LOX	NKILA	0.306168372	2.60E-12	postive
LOX	PLEKHA8P1	0.30064766	6.63E-12	postive
LOX	PRICKLE2-DT	0.584178425	4.41E-47	postive
LOX	PTENP1	0.404054077	4.65E-21	postive
LOX	PTPRD-AS1	0.400625791	1.06E-20	postive
LOX	RASAL2-AS1	0.395611112	3.51E-20	postive
LOX	RP11-264E20.1	0.447350631	5.65E-26	postive
LOX	RP11-264E20.2	0.320258454	2.18E-13	postive
LOX	RP11-439L18.2	0.375884802	3.17E-18	postive
LOX	RP11-514D23.3	0.331609248	2.68E-14	postive
LOX	RP11-524D16__A.3	0.394302769	4.78E-20	postive
LOX	RP11-53M11.3	0.310299034	1.27E-12	postive
LOX	RP11-556E13.1	0.421450391	5.99E-23	postive
LOX	RP11-86H7.7	0.319722342	2.40E-13	postive
LOX	RP1-93H18.1	0.48114456	2.47E-30	postive
LOX	RP5-968J1.1	0.405447049	3.31E-21	postive
LOX	RPL23AP7	0.366559633	2.40E-17	postive
LOX	RPL23AP82	0.342914719	3.04E-15	postive
LOX	RPSAP52	0.321839486	1.64E-13	postive
LOX	RXYLT1-AS1	0.349371253	8.44E-16	postive
LOX	SAP30-DT	0.314527137	6.07E-13	postive
LOX	SH3RF3-AS1	0.411463298	7.52E-22	postive
LOX	SOCAR	0.351485616	5.51E-16	postive
LOX	STXBP5-AS1	0.430387022	5.79E-24	postive
LOX	SVIL-AS1	0.35635684	2.04E-16	postive
LOX	TBX5-AS1	0.334968916	1.42E-14	postive
LOX	THEM7P	0.303344368	4.21E-12	postive
LOX	TMEM26-AS1	0.375044754	3.81E-18	postive

LOX	WNT5A-AS1	0.373833504	4.97E-18	postive
LOX	WWC2-AS2	0.304464256	3.48E-12	postive
LOXL1	A2M-AS1	0.353475632	3.68E-16	postive
LOXL1	AC000067.1	0.341304445	4.17E-15	postive
LOXL1	AC000403.1	0.492684518	6.17E-32	postive
LOXL1	AC004130.2	0.33067211	3.20E-14	postive
LOXL1	AC004160.1	0.318614527	2.93E-13	postive
LOXL1	AC004854.2	0.365313478	3.13E-17	postive
LOXL1	AC005034.4	0.303371966	4.19E-12	postive
LOXL1	AC005083.1	0.313276315	7.57E-13	postive
LOXL1	AC005261.4	0.35680072	1.86E-16	postive
LOXL1	AC006126.4	0.358279609	1.37E-16	postive
LOXL1	AC007750.1	0.368782834	1.49E-17	postive
LOXL1	AC007998.3	0.380489619	1.14E-18	postive
LOXL1	AC008742.1	0.327770021	5.50E-14	postive
LOXL1	AC009093.1	0.510135782	1.77E-34	postive
LOXL1	AC009093.2	0.439948624	4.40E-25	postive
LOXL1	AC009126.1	0.325750367	7.99E-14	postive
LOXL1	AC009509.5	0.455321532	5.85E-27	postive
LOXL1	AC009831.1	0.450056515	2.63E-26	postive
LOXL1	AC010931.1	0.499317002	6.93E-33	postive
LOXL1	AC011352.1	0.316138544	4.56E-13	postive
LOXL1	AC011451.1	0.303512342	4.09E-12	postive
LOXL1	AC011595.2	0.349951946	7.51E-16	postive
LOXL1	AC012306.2	0.360732383	8.22E-17	postive
LOXL1	AC012360.2	0.331413047	2.78E-14	postive
LOXL1	AC013553.3	0.339755598	5.64E-15	postive
LOXL1	AC015712.2	0.362347444	5.86E-17	postive
LOXL1	AC016027.1	0.40261085	6.60E-21	postive
LOXL1	AC018904.1	0.314983485	5.60E-13	postive
LOXL1	AC022098.1	0.326259058	7.27E-14	postive
LOXL1	AC023301.1	0.546466	2.85E-40	postive
LOXL1	AC026904.2	0.316705293	4.12E-13	postive
LOXL1	AC027237.5	0.50015678	5.24E-33	postive
LOXL1	AC027319.1	0.331073592	2.96E-14	postive
LOXL1	AC034199.1	0.332225512	2.39E-14	postive
LOXL1	AC060766.7	0.338290506	7.49E-15	postive
LOXL1	AC067852.2	0.356337136	2.05E-16	postive
LOXL1	AC068234.2	0.301098832	6.15E-12	postive
LOXL1	AC068305.2	0.363711955	4.39E-17	postive
LOXL1	AC068870.2	0.383756186	5.45E-19	postive
LOXL1	AC073172.1	0.379116228	1.55E-18	postive
LOXL1	AC074011.1	0.307388546	2.11E-12	postive
LOXL1	AC079070.1	0.311510707	1.03E-12	postive
LOXL1	AC079298.3	0.391985194	8.22E-20	postive
LOXL1	AC080112.2	0.345434286	1.85E-15	postive
LOXL1	AC087289.5	0.369862223	1.18E-17	postive
LOXL1	AC087477.5	0.308237713	1.82E-12	postive
LOXL1	AC087627.1	0.332874988	2.11E-14	postive
LOXL1	AC090136.3	0.301539916	5.71E-12	postive

LOXL1	AC090229.1	0.331143776	2.93E-14	postive
LOXL1	AC090409.1	0.341471785	4.04E-15	postive
LOXL1	AC090515.3	0.30229643	5.03E-12	postive
LOXL1	AC090559.1	0.311210314	1.09E-12	postive
LOXL1	AC092368.3	0.320912385	1.93E-13	postive
LOXL1	AC092376.2	0.569397223	2.62E-44	postive
LOXL1	AC093627.6	0.311458727	1.04E-12	postive
LOXL1	AC093673.1	0.346034994	1.64E-15	postive
LOXL1	AC093895.2	0.313292421	7.55E-13	postive
LOXL1	AC099066.2	0.536279539	1.41E-38	postive
LOXL1	AC099518.6	0.32407984	1.09E-13	postive
LOXL1	AC104083.1	0.399955444	1.25E-20	postive
LOXL1	AC105020.6	0.30958448	1.44E-12	postive
LOXL1	AC106739.1	0.440035585	4.29E-25	postive
LOXL1	AC106881.1	0.46407824	4.52E-28	postive
LOXL1	AC107294.3	0.315891072	4.76E-13	postive
LOXL1	AC108463.2	0.44928951	3.27E-26	postive
LOXL1	AC108488.1	0.365458597	3.03E-17	postive
LOXL1	AC110995.1	0.300916875	6.34E-12	postive
LOXL1	AC112715.1	0.488232301	2.61E-31	postive
LOXL1	AC113346.1	0.359671825	1.03E-16	postive
LOXL1	AC114271.1	0.386622174	2.84E-19	postive
LOXL1	AC115618.2	0.320158966	2.22E-13	postive
LOXL1	AC121247.2	0.309685748	1.42E-12	postive
LOXL1	AC126773.4	0.321242209	1.82E-13	postive
LOXL1	AC133552.2	0.305711081	2.81E-12	postive
LOXL1	AC134312.5	0.593490074	6.63E-49	postive
LOXL1	AC135050.1	0.312366478	8.88E-13	postive
LOXL1	AC136475.1	0.301510534	5.74E-12	postive
LOXL1	AC139768.1	0.322104628	1.56E-13	postive
LOXL1	AC142381.3	0.334712823	1.49E-14	postive
LOXL1	AC147067.1	0.359460989	1.07E-16	postive
LOXL1	AC244669.1	0.331757015	2.61E-14	postive
LOXL1	ACTA2-AS1	0.4436101	1.60E-25	postive
LOXL1	ACTN1-AS1	0.321368698	1.78E-13	postive
LOXL1	ADAMTS7P1	0.324354423	1.03E-13	postive
LOXL1	AF015262.1	0.518161771	1.06E-35	postive
LOXL1	AGAP2-AS1	0.344851929	2.08E-15	postive
LOXL1	AL008638.6	0.305182961	3.08E-12	postive
LOXL1	AL009178.2	0.303541097	4.07E-12	postive
LOXL1	AL023803.2	0.379921471	1.29E-18	postive
LOXL1	AL031651.2	0.341596837	3.94E-15	postive
LOXL1	AL034417.3	0.311918903	9.60E-13	postive
LOXL1	AL035587.1	0.300715526	6.56E-12	postive
LOXL1	AL049569.1	0.354593249	2.93E-16	postive
LOXL1	AL049597.2	0.370748421	9.74E-18	postive
LOXL1	AL050309.1	0.376936889	2.51E-18	postive
LOXL1	AL078581.2	0.328995052	4.38E-14	postive
LOXL1	AL109924.1	0.497222614	1.39E-32	postive
LOXL1	AL122035.1	0.324513112	1.00E-13	postive

LOXL1	AL133415.1	0.329040604	4.34E-14	postive
LOXL1	AL136084.3	0.443848173	1.50E-25	postive
LOXL1	AL136987.1	0.384832935	4.27E-19	postive
LOXL1	AL137003.1	0.307249731	2.16E-12	postive
LOXL1	AL137186.2	0.360334238	8.93E-17	postive
LOXL1	AL139339.1	0.436900628	1.01E-24	postive
LOXL1	AL139351.1	0.43419209	2.10E-24	postive
LOXL1	AL139393.3	0.367991427	1.76E-17	postive
LOXL1	AL157394.1	0.303108151	4.38E-12	postive
LOXL1	AL161785.1	0.318056818	3.24E-13	postive
LOXL1	AL162457.2	0.428485232	9.58E-24	postive
LOXL1	AL162727.1	0.346081672	1.63E-15	postive
LOXL1	AL356417.2	0.333733117	1.79E-14	postive
LOXL1	AL356599.1	0.3091965	1.54E-12	postive
LOXL1	AL365203.2	0.305170275	3.09E-12	postive
LOXL1	AL390955.2	0.316288068	4.44E-13	postive
LOXL1	AL445423.1	0.33266065	2.20E-14	postive
LOXL1	AL596087.2	0.443665925	1.58E-25	postive
LOXL1	AMZ2P1	0.313634124	7.10E-13	postive
LOXL1	AOC4P	0.301309066	5.94E-12	postive
LOXL1	AP000695.1	0.425975613	1.85E-23	postive
LOXL1	AP000695.2	0.382152454	7.83E-19	postive
LOXL1	AP001107.3	0.30708598	2.22E-12	postive
LOXL1	AP001107.5	0.4330993	2.81E-24	postive
LOXL1	AP001189.3	0.335637951	1.25E-14	postive
LOXL1	AP001330.4	0.548750339	1.17E-40	postive
LOXL1	AP001434.1	0.440917723	3.37E-25	postive
LOXL1	AP001471.1	0.30222683	5.09E-12	postive
LOXL1	AP001816.1	0.329582057	3.92E-14	postive
LOXL1	AP002414.2	0.381766819	8.54E-19	postive
LOXL1	AP003071.3	0.311920753	9.60E-13	postive
LOXL1	AP003071.4	0.426369961	1.67E-23	postive
LOXL1	ARMCX5-GPRASP2	0.337891836	8.09E-15	postive
LOXL1	ATE1-AS1	0.313658207	7.07E-13	postive
LOXL1	ATXN1-AS1	0.340892499	4.52E-15	postive
LOXL1	BHLHE40-AS1	0.327644842	5.63E-14	postive
LOXL1	BTN2A3P	0.312301151	8.98E-13	postive
LOXL1	BX284613.2	0.382834713	6.71E-19	postive
LOXL1	BX322234.1	0.419470981	9.96E-23	postive
LOXL1	C10orf25	0.323618218	1.18E-13	postive
LOXL1	C2orf27A	0.526585014	5.14E-37	postive
LOXL1	CACTIN-AS1	0.326755624	6.64E-14	postive
LOXL1	CARD8-AS1	0.303456137	4.13E-12	postive
LOXL1	CD99P1	0.311846669	9.73E-13	postive
LOXL1	CENPBD1P1	0.374400867	4.39E-18	postive
LOXL1	CIDECP1	0.334619666	1.51E-14	postive
LOXL1	COL6A4P2	0.328225287	5.05E-14	postive
LOXL1	CTA-14H9.5	0.321535572	1.73E-13	postive
LOXL1	CXXC1P1	0.317258466	3.73E-13	postive
LOXL1	DACT3-AS1	0.323311744	1.25E-13	postive

LOXL1	DNM3OS	0.398724412	1.68E-20	postive
LOXL1	EMC1-AS1	0.343784923	2.57E-15	postive
LOXL1	EPB41L4A-DT	0.388685249	1.77E-19	postive
LOXL1	FAM225B	0.439631537	4.80E-25	postive
LOXL1	FAM66C	0.321503354	1.74E-13	postive
LOXL1	FGD5-AS1	0.396579584	2.79E-20	postive
LOXL1	FNDC1-IT1	0.32929083	4.14E-14	postive
LOXL1	GGTA1P	0.425269328	2.23E-23	postive
LOXL1	GORAB-AS1	0.326548664	6.89E-14	postive
LOXL1	GTF2IP4	0.313074913	7.84E-13	postive
LOXL1	HCG11	0.301060211	6.19E-12	postive
LOXL1	HCG20	0.31346379	7.32E-13	postive
LOXL1	HECW2-AS1	0.476834479	9.48E-30	postive
LOXL1	HLX-AS1	0.308852198	1.64E-12	postive
LOXL1	HMGA2-AS1	0.371428655	8.40E-18	postive
LOXL1	IGFBP7-AS1	0.353819709	3.43E-16	postive
LOXL1	IL10RB-DT	0.387391307	2.38E-19	postive
LOXL1	ILF3-DT	0.302262836	5.05E-12	postive
LOXL1	INHBA-AS1	0.387342809	2.41E-19	postive
LOXL1	L3MBTL2-AS1	0.339808202	5.58E-15	postive
LOXL1	LCT-AS1	0.411069566	8.29E-22	postive
LOXL1	LEF1-AS1	0.480392978	3.13E-30	postive
LOXL1	LINC00310	0.413580524	4.43E-22	postive
LOXL1	LINC00398	0.345030642	2.01E-15	postive
LOXL1	LINC00511	0.311330147	1.06E-12	postive
LOXL1	LINC00526	0.309064155	1.58E-12	postive
LOXL1	LINC00623	0.31440464	6.20E-13	postive
LOXL1	LINC00656	0.311102336	1.11E-12	postive
LOXL1	LINC00667	0.33727082	9.12E-15	postive
LOXL1	LINC00702	0.392108234	7.99E-20	postive
LOXL1	LINC00933	0.323051232	1.31E-13	postive
LOXL1	LINC00957	0.336797091	9.99E-15	postive
LOXL1	LINC01048	0.332995709	2.06E-14	postive
LOXL1	LINC01094	0.327870434	5.40E-14	postive
LOXL1	LINC01128	0.342462237	3.33E-15	postive
LOXL1	LINC01142	0.33171338	2.63E-14	postive
LOXL1	LINC01150	0.335222898	1.35E-14	postive
LOXL1	LINC01184	0.329007623	4.37E-14	postive
LOXL1	LINC01235	0.379379289	1.46E-18	postive
LOXL1	LINC01352	0.332683294	2.19E-14	postive
LOXL1	LINC01429	0.470970824	5.71E-29	postive
LOXL1	LINC01449	0.455618915	5.37E-27	postive
LOXL1	LINC01505	0.487314235	3.50E-31	postive
LOXL1	LINC01521	0.306632306	2.40E-12	postive
LOXL1	LINC01561	0.569209842	2.84E-44	postive
LOXL1	LINC01583	0.331654523	2.66E-14	postive
LOXL1	LINC01614	0.543913704	7.68E-40	postive
LOXL1	LINC01615	0.306509599	2.45E-12	postive
LOXL1	LINC01638	0.372016461	7.39E-18	postive
LOXL1	LINC01704	0.33298795	2.07E-14	postive

LOXL1	LINC01705	0.414351704	3.65E-22	postive
LOXL1	LINC01711	0.457809821	2.85E-27	postive
LOXL1	LINC01915	0.4187222	1.21E-22	postive
LOXL1	LINC01929	0.367400992	2.00E-17	postive
LOXL1	LINC01936	0.313519304	7.25E-13	postive
LOXL1	LINC01943	0.333659198	1.82E-14	postive
LOXL1	LINC02126	0.461024295	1.11E-27	postive
LOXL1	LINC02202	0.327097934	6.23E-14	postive
LOXL1	LINC02345	0.332710654	2.18E-14	postive
LOXL1	LINC02408	0.41088484	8.68E-22	postive
LOXL1	LINC02519	0.453589061	9.63E-27	postive
LOXL1	LINC02544	0.590425751	2.68E-48	postive
LOXL1	LINC02693	0.346962529	1.37E-15	postive
LOXL1	LINC02828	0.316391195	4.36E-13	postive
LOXL1	LMO7-AS1	0.334395393	1.58E-14	postive
LOXL1	LNCOG	0.321702155	1.68E-13	postive
LOXL1	LOXL1-AS1	0.537932388	7.57E-39	postive
LOXL1	LUARIS	0.312645216	8.46E-13	postive
LOXL1	MAGI2-AS3	0.35105374	6.01E-16	postive
LOXL1	MAP4K3-DT	0.302716758	4.68E-12	postive
LOXL1	MIR4435-2HG	0.3773032	2.32E-18	postive
LOXL1	MIR497HG	0.405658162	3.15E-21	postive
LOXL1	MIR762HG	0.31243925	8.77E-13	postive
LOXL1	MSC-AS1	0.401271191	9.11E-21	postive
LOXL1	MTMR9LP	0.300195874	7.16E-12	postive
LOXL1	NANOGP1	0.309675564	1.42E-12	postive
LOXL1	NOP14-AS1	0.31588916	4.77E-13	postive
LOXL1	NORAD	0.31584648	4.80E-13	postive
LOXL1	NR2F1-AS1	0.358285784	1.37E-16	postive
LOXL1	OR7E47P	0.400254973	1.16E-20	postive
LOXL1	PCED1B-AS1	0.341925838	3.70E-15	postive
LOXL1	PINK1-AS	0.352593489	4.40E-16	postive
LOXL1	PLBD1-AS1	0.315441889	5.16E-13	postive
LOXL1	POLR2J4	0.304298458	3.58E-12	postive
LOXL1	PRICKLE2-DT	0.418921531	1.15E-22	postive
LOXL1	PRR34	0.341420915	4.08E-15	postive
LOXL1	PTPRD-AS1	0.411143484	8.14E-22	postive
LOXL1	RAP2C-AS1	0.314298151	6.32E-13	postive
LOXL1	RARA-AS1	0.300899123	6.36E-12	postive
LOXL1	RASGRF2-AS1	0.455866254	5.00E-27	postive
LOXL1	RNF216P1	0.418770591	1.19E-22	postive
LOXL1	RP11-180N14.1	0.300250408	7.09E-12	postive
LOXL1	RP1-122P22.2	0.337566329	8.61E-15	postive
LOXL1	RP11-264E20.1	0.343242732	2.85E-15	postive
LOXL1	RP11-394O4.5	0.31226862	9.03E-13	postive
LOXL1	RP11-514D23.3	0.517306231	1.44E-35	postive
LOXL1	RP11-524D16__A.3	0.416896808	1.92E-22	postive
LOXL1	RP11-556E13.1	0.340429913	4.95E-15	postive
LOXL1	RP11-86H7.7	0.403076007	5.89E-21	postive
LOXL1	RP1-93H18.1	0.429902743	6.59E-24	postive

LOXL1	RP3-355L5.5	0.333304151	1.95E-14	postive
LOXL1	RP5-1042I8.7	0.312180781	9.17E-13	postive
LOXL1	RPL23AP7	0.377282824	2.33E-18	postive
LOXL1	RPL23AP82	0.420191897	8.28E-23	postive
LOXL1	RXYLT1-AS1	0.323512789	1.21E-13	postive
LOXL1	SEPTIN4-AS1	0.323927957	1.12E-13	postive
LOXL1	SERTAD4-AS1	0.396148105	3.09E-20	postive
LOXL1	SH3RF3-AS1	0.480323342	3.20E-30	postive
LOXL1	SLC25A34-AS1	0.354504781	2.98E-16	postive
LOXL1	SOCAR	0.353315106	3.80E-16	postive
LOXL1	SSR4P1	0.322221862	1.53E-13	postive
LOXL1	STXBP5-AS1	0.309137703	1.56E-12	postive
LOXL1	SVIL-AS1	0.323024483	1.32E-13	postive
LOXL1	TBX5-AS1	0.481116594	2.50E-30	postive
LOXL1	THEM7P	0.307023839	2.25E-12	postive
LOXL1	TMEM231P1	0.302073925	5.22E-12	postive
LOXL1	TUG1	0.346077518	1.63E-15	postive
LOXL1	U82695.1	0.346665907	1.45E-15	postive
LOXL1	UBA6-AS1	0.306699474	2.37E-12	postive
LOXL1	WNT5A-AS1	0.363027055	5.07E-17	postive
LOXL1	Z97353.2	0.316886486	3.99E-13	postive
LOXL1	ZNF561-AS1	0.378984929	1.59E-18	postive
LOXL1	ZNF815P	0.313433976	7.36E-13	postive
LOXL2	AC000403.1	0.324768328	9.57E-14	postive
LOXL2	AC004080.2	0.323406974	1.23E-13	postive
LOXL2	AC004704.1	0.472075194	4.08E-29	postive
LOXL2	AC004930.1	0.404552419	4.12E-21	postive
LOXL2	AC009093.1	0.318755061	2.86E-13	postive
LOXL2	AC009486.1	0.309803218	1.39E-12	postive
LOXL2	AC012513.1	0.588033118	7.89E-48	postive
LOXL2	AC016168.1	0.338075479	7.81E-15	postive
LOXL2	AC017048.3	0.379434059	1.44E-18	postive
LOXL2	AC019069.1	0.316004156	4.67E-13	postive
LOXL2	AC034199.1	0.313249188	7.60E-13	postive
LOXL2	AC044784.1	0.492762816	6.01E-32	postive
LOXL2	AC069155.1	0.614713371	2.75E-53	postive
LOXL2	AC073569.2	0.310014488	1.34E-12	postive
LOXL2	AC074286.1	0.589251404	4.56E-48	postive
LOXL2	AC090197.1	0.318473884	3.00E-13	postive
LOXL2	AC091979.1	0.345981281	1.66E-15	postive
LOXL2	AC092171.2	0.310723677	1.18E-12	postive
LOXL2	AC093673.1	0.306945391	2.28E-12	postive
LOXL2	AC099520.1	0.469279079	9.53E-29	postive
LOXL2	AC099850.4	0.326550223	6.89E-14	postive
LOXL2	AC103808.1	0.323003741	1.32E-13	postive
LOXL2	AC103982.1	0.316164983	4.54E-13	postive
LOXL2	AC104119.1	0.319794118	2.37E-13	postive
LOXL2	AC107021.2	0.308522002	1.73E-12	postive
LOXL2	AC108463.2	0.338500088	7.19E-15	postive
LOXL2	AC112721.1	0.302033423	5.25E-12	postive

LOXL2	AC113346.1	0.356046588	2.17E-16	postive
LOXL2	AC114803.1	0.303925566	3.81E-12	postive
LOXL2	AC125807.2	0.378459614	1.79E-18	postive
LOXL2	AC134312.5	0.367725059	1.87E-17	postive
LOXL2	AC139718.1	0.494906556	2.98E-32	postive
LOXL2	AC139718.2	0.457105506	3.49E-27	postive
LOXL2	AC211486.9	0.523658969	1.49E-36	postive
LOXL2	AJ239318.1	0.313432033	7.36E-13	postive
LOXL2	AL049775.1	0.332368172	2.32E-14	postive
LOXL2	AL049775.3	0.315048158	5.53E-13	postive
LOXL2	AL078644.2	0.316776878	4.07E-13	postive
LOXL2	AL136084.3	0.319437159	2.53E-13	postive
LOXL2	AL136322.1	0.414080404	3.91E-22	postive
LOXL2	AL138828.1	0.303319411	4.23E-12	postive
LOXL2	AL161668.1	0.610259893	2.43E-52	postive
LOXL2	AL354811.1	0.371174214	8.88E-18	postive
LOXL2	AL359918.3	0.432371584	3.41E-24	postive
LOXL2	AL691515.1	0.331492025	2.74E-14	postive
LOXL2	AP000695.1	0.397065775	2.49E-20	postive
LOXL2	AP000695.2	0.395689092	3.45E-20	postive
LOXL2	AP001107.3	0.37511079	3.76E-18	postive
LOXL2	AP003071.4	0.30361384	4.02E-12	postive
LOXL2	C2orf27A	0.304968871	3.19E-12	postive
LOXL2	CYTOR	0.304016158	3.76E-12	postive
LOXL2	DENND5B-AS1	0.426181356	1.75E-23	postive
LOXL2	FLG-AS1	0.408864007	1.43E-21	postive
LOXL2	HAS2-AS1	0.450114348	2.59E-26	postive
LOXL2	IL12A-AS1	0.307898346	1.93E-12	postive
LOXL2	LINC00239	0.411311427	7.81E-22	postive
LOXL2	LINC00592	0.349293766	8.58E-16	postive
LOXL2	LINC00598	0.327907498	5.36E-14	postive
LOXL2	LINC00626	0.309276791	1.52E-12	postive
LOXL2	LINC00645	0.315381753	5.22E-13	postive
LOXL2	LINC00707	0.543321241	9.65E-40	postive
LOXL2	LINC01059	0.386867027	2.68E-19	postive
LOXL2	LINC01116	0.47602512	1.22E-29	postive
LOXL2	LINC01117	0.407034816	2.25E-21	postive
LOXL2	LINC01262	0.33357756	1.85E-14	postive
LOXL2	LINC01312	0.308031184	1.89E-12	postive
LOXL2	LINC01351	0.405659986	3.14E-21	postive
LOXL2	LINC01429	0.301088142	6.16E-12	postive
LOXL2	LINC01446	0.363170626	4.92E-17	postive
LOXL2	LINC01546	0.322241137	1.52E-13	postive
LOXL2	LINC01561	0.376230334	2.94E-18	postive
LOXL2	LINC01614	0.403228864	5.68E-21	postive
LOXL2	LINC01705	0.327320368	5.98E-14	postive
LOXL2	LINC02320	0.351829695	5.14E-16	postive
LOXL2	LINC02323	0.465596458	2.88E-28	postive
LOXL2	LINC02587	0.338461381	7.25E-15	postive
LOXL2	LINC02599	0.327302795	6.00E-14	postive

LOXL2	LINC02693	0.312842292	8.17E-13	postive
LOXL2	MIR100HG	0.353669641	3.54E-16	postive
LOXL2	MIR1915HG	0.503209093	1.88E-33	postive
LOXL2	MIR4435-2HG	0.393474362	5.80E-20	postive
LOXL2	MSC-AS1	0.320938344	1.93E-13	postive
LOXL2	MT1DP	0.342343392	3.41E-15	postive
LOXL2	MT1L	0.445556736	9.33E-26	postive
LOXL2	NKILA	0.306879238	2.30E-12	postive
LOXL2	NLGN4Y-AS1	0.577264445	9.11E-46	postive
LOXL2	PLEKHA8P1	0.311871314	9.68E-13	postive
LOXL2	PPIAP48	0.334579052	1.53E-14	postive
LOXL2	PRICKLE2-DT	0.314617369	5.97E-13	postive
LOXL2	RASAL2-AS1	0.317325654	3.69E-13	postive
LOXL2	RNF216P1	0.353071498	3.99E-16	postive
LOXL2	RP11-264E20.1	0.330968052	3.02E-14	postive
LOXL2	RP11-439I14.2	0.33727114	9.12E-15	postive
LOXL2	RP11-543B16.3	0.577383123	8.65E-46	postive
LOXL2	RP1-93H18.1	0.313154262	7.73E-13	postive
LOXL3	A1BG-AS1	0.390013276	1.30E-19	postive
LOXL3	A2M-AS1	0.341042072	4.39E-15	postive
LOXL3	ABALON	0.409269596	1.30E-21	postive
LOXL3	AC000068.1	0.410536099	9.46E-22	postive
LOXL3	AC000403.1	0.49353009	4.68E-32	postive
LOXL3	AC002091.1	0.349399042	8.40E-16	postive
LOXL3	AC002091.2	0.383566298	5.69E-19	postive
LOXL3	AC002116.2	0.327329017	5.97E-14	postive
LOXL3	AC002398.1	0.346730091	1.43E-15	postive
LOXL3	AC002428.2	0.334529877	1.54E-14	postive
LOXL3	AC002456.1	0.351788899	5.18E-16	postive
LOXL3	AC003973.1	0.360405533	8.80E-17	postive
LOXL3	AC004067.1	0.307288991	2.15E-12	postive
LOXL3	AC004241.3	0.306696819	2.38E-12	postive
LOXL3	AC004447.2	0.341608668	3.93E-15	postive
LOXL3	AC004466.1	0.348289046	1.05E-15	postive
LOXL3	AC004477.3	0.305505187	2.91E-12	postive
LOXL3	AC004771.2	0.418680272	1.22E-22	postive
LOXL3	AC004771.4	0.356043953	2.17E-16	postive
LOXL3	AC004812.2	0.357112567	1.74E-16	postive
LOXL3	AC004893.11	0.345518682	1.82E-15	postive
LOXL3	AC004921.1	0.315743706	4.89E-13	postive
LOXL3	AC004943.2	0.398505322	1.77E-20	postive
LOXL3	AC005005.3	0.328118762	5.15E-14	postive
LOXL3	AC005034.6	0.322092652	1.56E-13	postive
LOXL3	AC005041.3	0.334883653	1.44E-14	postive
LOXL3	AC005046.2	0.309511212	1.46E-12	postive
LOXL3	AC005253.1	0.434423506	1.97E-24	postive
LOXL3	AC005288.1	0.398258118	1.87E-20	postive
LOXL3	AC005332.4	0.427647083	1.19E-23	postive
LOXL3	AC005387.2	0.361541083	6.94E-17	postive
LOXL3	AC005519.1	0.412029956	6.53E-22	postive

LOXL3	AC005562.1	0.451515046	1.74E-26	postive
LOXL3	AC005586.1	0.339824798	5.57E-15	postive
LOXL3	AC005625.1	0.306459505	2.47E-12	postive
LOXL3	AC005670.3	0.404006504	4.70E-21	postive
LOXL3	AC005845.1	0.307722891	1.99E-12	postive
LOXL3	AC006033.2	0.369211199	1.36E-17	postive
LOXL3	AC006449.5	0.311574158	1.02E-12	postive
LOXL3	AC006547.1	0.333752329	1.79E-14	postive
LOXL3	AC007292.2	0.359881092	9.82E-17	postive
LOXL3	AC007663.4	0.369443132	1.29E-17	postive
LOXL3	AC007728.2	0.351189346	5.85E-16	postive
LOXL3	AC007823.1	0.315741062	4.89E-13	postive
LOXL3	AC007998.3	0.31271872	8.35E-13	postive
LOXL3	AC008124.1	0.331705039	2.63E-14	postive
LOXL3	AC008434.1	0.31130685	1.07E-12	postive
LOXL3	AC008494.3	0.33348304	1.88E-14	postive
LOXL3	AC008543.1	0.383108356	6.31E-19	postive
LOXL3	AC008735.2	0.369895017	1.17E-17	postive
LOXL3	AC008742.1	0.342221636	3.49E-15	postive
LOXL3	AC008750.1	0.368860466	1.46E-17	postive
LOXL3	AC008764.2	0.309608359	1.44E-12	postive
LOXL3	AC008938.1	0.343413632	2.76E-15	postive
LOXL3	AC009093.1	0.415929186	2.45E-22	postive
LOXL3	AC009093.2	0.437013503	9.79E-25	postive
LOXL3	AC009121.3	0.342842284	3.09E-15	postive
LOXL3	AC009126.1	0.336974721	9.65E-15	postive
LOXL3	AC009309.2	0.33396858	1.71E-14	postive
LOXL3	AC009509.5	0.314982254	5.60E-13	postive
LOXL3	AC009630.2	0.348722023	9.62E-16	postive
LOXL3	AC009690.2	0.313162812	7.72E-13	postive
LOXL3	AC009831.1	0.429053544	8.24E-24	postive
LOXL3	AC010319.4	0.400790421	1.02E-20	postive
LOXL3	AC010504.1	0.303699505	3.96E-12	postive
LOXL3	AC010615.2	0.357158058	1.73E-16	postive
LOXL3	AC010618.3	0.338012854	7.90E-15	postive
LOXL3	AC010655.4	0.305331997	3.00E-12	postive
LOXL3	AC010729.2	0.321778382	1.65E-13	postive
LOXL3	AC011005.4	0.423015722	4.00E-23	postive
LOXL3	AC011337.1	0.30750741	2.07E-12	postive
LOXL3	AC011447.3	0.357552982	1.59E-16	postive
LOXL3	AC011450.1	0.321826718	1.64E-13	postive
LOXL3	AC011451.1	0.369859155	1.18E-17	postive
LOXL3	AC011466.1	0.31743889	3.62E-13	postive
LOXL3	AC011466.3	0.35238832	4.59E-16	postive
LOXL3	AC011595.2	0.312127813	9.26E-13	postive
LOXL3	AC011899.2	0.336960719	9.68E-15	postive
LOXL3	AC012063.1	0.37987921	1.30E-18	postive
LOXL3	AC012073.1	0.303309697	4.23E-12	postive
LOXL3	AC012146.3	0.338813661	6.77E-15	postive
LOXL3	AC012306.2	0.349572768	8.11E-16	postive

LOXL3	AC012313.1	0.302192604	5.12E-12	postive
LOXL3	AC012360.3	0.31349133	7.29E-13	postive
LOXL3	AC012467.2	0.312969993	7.99E-13	postive
LOXL3	AC012645.3	0.302503486	4.85E-12	postive
LOXL3	AC015712.1	0.338499369	7.20E-15	postive
LOXL3	AC015712.2	0.395935007	3.25E-20	postive
LOXL3	AC015909.2	0.431547194	4.25E-24	postive
LOXL3	AC015909.3	0.361328242	7.25E-17	postive
LOXL3	AC016027.1	0.325499711	8.37E-14	postive
LOXL3	AC016582.3	0.312303979	8.98E-13	postive
LOXL3	AC016590.3	0.305366496	2.98E-12	postive
LOXL3	AC016722.2	0.313155443	7.73E-13	postive
LOXL3	AC016876.3	0.308472466	1.75E-12	postive
LOXL3	AC018445.1	0.311749003	9.89E-13	postive
LOXL3	AC018653.3	0.307539967	2.06E-12	postive
LOXL3	AC019069.1	0.332223904	2.39E-14	postive
LOXL3	AC019205.1	0.445126107	1.05E-25	postive
LOXL3	AC020913.2	0.321946379	1.60E-13	postive
LOXL3	AC020915.1	0.399577111	1.37E-20	postive
LOXL3	AC020915.3	0.380869501	1.05E-18	postive
LOXL3	AC021188.1	0.384941673	4.16E-19	postive
LOXL3	AC022098.1	0.407437197	2.03E-21	postive
LOXL3	AC022150.2	0.352743687	4.27E-16	postive
LOXL3	AC022211.3	0.352694267	4.31E-16	postive
LOXL3	AC022364.1	0.309480384	1.47E-12	postive
LOXL3	AC022415.2	0.366854357	2.25E-17	postive
LOXL3	AC022706.1	0.333835793	1.76E-14	postive
LOXL3	AC022916.2	0.310615497	1.21E-12	postive
LOXL3	AC023301.1	0.357738866	1.53E-16	postive
LOXL3	AC023983.1	0.312352128	8.90E-13	postive
LOXL3	AC023983.2	0.304489942	3.47E-12	postive
LOXL3	AC024075.1	0.300193496	7.16E-12	postive
LOXL3	AC025048.2	0.31249899	8.68E-13	postive
LOXL3	AC025165.1	0.334125609	1.66E-14	postive
LOXL3	AC025176.1	0.32359578	1.19E-13	postive
LOXL3	AC025287.3	0.316347453	4.39E-13	postive
LOXL3	AC025682.2	0.307372998	2.12E-12	postive
LOXL3	AC025811.1	0.303562292	4.06E-12	postive
LOXL3	AC025857.2	0.334127387	1.66E-14	postive
LOXL3	AC026310.2	0.360909455	7.92E-17	postive
LOXL3	AC026356.1	0.381457022	9.16E-19	postive
LOXL3	AC026356.2	0.322993999	1.32E-13	postive
LOXL3	AC026401.3	0.394139589	4.96E-20	postive
LOXL3	AC026904.1	0.314151836	6.48E-13	postive
LOXL3	AC027097.2	0.413135651	4.95E-22	postive
LOXL3	AC027237.5	0.321068192	1.88E-13	postive
LOXL3	AC027601.2	0.347316463	1.27E-15	postive
LOXL3	AC034102.6	0.418243855	1.36E-22	postive
LOXL3	AC034199.1	0.360446081	8.72E-17	postive
LOXL3	AC037459.2	0.3373312	9.01E-15	postive

LOXL3	AC046143.1	0.39547061	3.63E-20	postive
LOXL3	AC051649.1	0.344770634	2.11E-15	postive
LOXL3	AC060780.1	0.311204034	1.09E-12	postive
LOXL3	AC064805.1	0.32907028	4.31E-14	postive
LOXL3	AC064807.1	0.36158696	6.87E-17	postive
LOXL3	AC067773.1	0.409850957	1.12E-21	postive
LOXL3	AC067852.2	0.37580266	3.23E-18	postive
LOXL3	AC068473.3	0.382773504	6.81E-19	postive
LOXL3	AC068580.1	0.348719394	9.62E-16	postive
LOXL3	AC068580.3	0.38054755	1.12E-18	postive
LOXL3	AC068790.9	0.345766682	1.73E-15	postive
LOXL3	AC068831.2	0.304724168	3.33E-12	postive
LOXL3	AC069209.1	0.313232176	7.63E-13	postive
LOXL3	AC073046.1	0.389461149	1.48E-19	postive
LOXL3	AC073343.2	0.303948391	3.80E-12	postive
LOXL3	AC073569.2	0.421948591	5.27E-23	postive
LOXL3	AC073611.1	0.367293659	2.05E-17	postive
LOXL3	AC073869.1	0.357010138	1.78E-16	postive
LOXL3	AC074011.1	0.418468367	1.29E-22	postive
LOXL3	AC074117.1	0.322510768	1.45E-13	postive
LOXL3	AC074135.1	0.466622872	2.12E-28	postive
LOXL3	AC074135.2	0.307458483	2.08E-12	postive
LOXL3	AC078785.1	0.382082815	7.95E-19	postive
LOXL3	AC078883.1	0.307782802	1.97E-12	postive
LOXL3	AC079070.1	0.361053862	7.68E-17	postive
LOXL3	AC079298.3	0.321497749	1.74E-13	postive
LOXL3	AC079807.1	0.354715041	2.86E-16	postive
LOXL3	AC079922.2	0.362910456	5.20E-17	postive
LOXL3	AC080112.2	0.316636768	4.17E-13	postive
LOXL3	AC083862.2	0.338239378	7.57E-15	postive
LOXL3	AC084018.2	0.307757767	1.98E-12	postive
LOXL3	AC087500.1	0.301305726	5.94E-12	postive
LOXL3	AC087627.1	0.323788492	1.15E-13	postive
LOXL3	AC087672.2	0.366972074	2.20E-17	postive
LOXL3	AC090229.1	0.327568451	5.71E-14	postive
LOXL3	AC090425.3	0.354856679	2.77E-16	postive
LOXL3	AC090559.1	0.429054478	8.24E-24	postive
LOXL3	AC090825.1	0.32328649	1.26E-13	postive
LOXL3	AC091729.2	0.322447184	1.46E-13	postive
LOXL3	AC092295.2	0.334460564	1.56E-14	postive
LOXL3	AC092376.2	0.427418957	1.27E-23	postive
LOXL3	AC092614.1	0.371041441	9.14E-18	postive
LOXL3	AC092910.3	0.317074783	3.86E-13	postive
LOXL3	AC092953.2	0.325974886	7.67E-14	postive
LOXL3	AC093627.5	0.308685475	1.69E-12	postive
LOXL3	AC096733.3	0.34577084	1.73E-15	postive
LOXL3	AC097534.1	0.345984055	1.66E-15	postive
LOXL3	AC098484.1	0.324018212	1.10E-13	postive
LOXL3	AC098934.2	0.333346656	1.93E-14	postive
LOXL3	AC099066.2	0.420448485	7.75E-23	postive

LOXL3	AC099548.2	0.330964349	3.03E-14	postive
LOXL3	AC099681.1	0.312231873	9.09E-13	postive
LOXL3	AC099681.3	0.332210363	2.39E-14	postive
LOXL3	AC099778.1	0.301132828	6.11E-12	postive
LOXL3	AC099850.2	0.321553532	1.72E-13	postive
LOXL3	AC099850.4	0.42312354	3.89E-23	postive
LOXL3	AC100793.3	0.35289353	4.14E-16	postive
LOXL3	AC100849.1	0.328634655	4.68E-14	postive
LOXL3	AC104564.3	0.352618476	4.38E-16	postive
LOXL3	AC105206.2	0.338613856	7.04E-15	postive
LOXL3	AC105942.1	0.311808362	9.79E-13	postive
LOXL3	AC106739.1	0.328172763	5.10E-14	postive
LOXL3	AC106881.1	0.34788785	1.14E-15	postive
LOXL3	AC107021.2	0.361372505	7.19E-17	postive
LOXL3	AC107398.3	0.310779392	1.17E-12	postive
LOXL3	AC108025.1	0.30744948	2.09E-12	postive
LOXL3	AC108134.3	0.321883346	1.62E-13	postive
LOXL3	AC108463.2	0.418200685	1.38E-22	postive
LOXL3	AC108488.1	0.449095923	3.46E-26	postive
LOXL3	AC109454.2	0.320402071	2.12E-13	postive
LOXL3	AC109460.1	0.404720642	3.95E-21	postive
LOXL3	AC109460.2	0.369142724	1.38E-17	postive
LOXL3	AC109479.1	0.345317815	1.90E-15	postive
LOXL3	AC110995.1	0.32171258	1.67E-13	postive
LOXL3	AC112220.2	0.300868946	6.39E-12	postive
LOXL3	AC112715.1	0.359584987	1.04E-16	postive
LOXL3	AC113346.1	0.352987557	4.06E-16	postive
LOXL3	AC114271.1	0.378012283	1.98E-18	postive
LOXL3	AC114956.2	0.356211563	2.10E-16	postive
LOXL3	AC115618.2	0.344509075	2.22E-15	postive
LOXL3	AC116914.2	0.33144636	2.76E-14	postive
LOXL3	AC120114.1	0.310914761	1.14E-12	postive
LOXL3	AC123912.4	0.331339621	2.82E-14	postive
LOXL3	AC124045.1	0.340615334	4.77E-15	postive
LOXL3	AC124283.2	0.312681883	8.40E-13	postive
LOXL3	AC125494.1	0.411848279	6.83E-22	postive
LOXL3	AC125807.2	0.30478326	3.30E-12	postive
LOXL3	AC131025.1	0.310523683	1.23E-12	postive
LOXL3	AC131097.3	0.307520248	2.06E-12	postive
LOXL3	AC132008.2	0.428088416	1.06E-23	postive
LOXL3	AC132872.1	0.355335489	2.51E-16	postive
LOXL3	AC134312.5	0.517935438	1.15E-35	postive
LOXL3	AC135050.4	0.308273729	1.81E-12	postive
LOXL3	AC137834.2	0.444579243	1.23E-25	postive
LOXL3	AC138207.1	0.303019114	4.45E-12	postive
LOXL3	AC138207.4	0.387207612	2.48E-19	postive
LOXL3	AC138207.5	0.322773752	1.38E-13	postive
LOXL3	AC138904.1	0.306301653	2.54E-12	postive
LOXL3	AC138932.1	0.311355181	1.06E-12	postive
LOXL3	AC141586.1	0.326214893	7.33E-14	postive

LOXL3	AC142381.3	0.307718818	1.99E-12	postive
LOXL3	AC145098.1	0.447242769	5.82E-26	postive
LOXL3	AC147651.4	0.407609965	1.95E-21	postive
LOXL3	AC244453.2	0.303049389	4.43E-12	postive
LOXL3	AC245060.2	0.356881069	1.83E-16	postive
LOXL3	AC245452.1	0.356518478	1.97E-16	postive
LOXL3	ACTA2-AS1	0.332810019	2.14E-14	postive
LOXL3	ACTN1-AS1	0.343370293	2.78E-15	postive
LOXL3	ACVR2B-AS1	0.33186691	2.55E-14	postive
LOXL3	AD000671.3	0.321121413	1.86E-13	postive
LOXL3	ADAMTS7P1	0.347874091	1.14E-15	postive
LOXL3	ADCY10P1	0.304485317	3.47E-12	postive
LOXL3	ADGRE4P	0.322588254	1.43E-13	postive
LOXL3	ADNP-AS1	0.326430253	7.05E-14	postive
LOXL3	AFG3L1P	0.302330585	5.00E-12	postive
LOXL3	AL008638.6	0.301234766	6.01E-12	postive
LOXL3	AL008726.1	0.375359819	3.56E-18	postive
LOXL3	AL009178.2	0.310637449	1.20E-12	postive
LOXL3	AL021707.3	0.332522632	2.26E-14	postive
LOXL3	AL023803.2	0.302662092	4.73E-12	postive
LOXL3	AL024507.3	0.341376437	4.11E-15	postive
LOXL3	AL031118.1	0.464859608	3.58E-28	postive
LOXL3	AL031600.1	0.331946294	2.51E-14	postive
LOXL3	AL031670.1	0.470854321	5.92E-29	postive
LOXL3	AL031714.1	0.304815594	3.28E-12	postive
LOXL3	AL031846.1	0.364196562	3.96E-17	postive
LOXL3	AL034550.1	0.312890241	8.10E-13	postive
LOXL3	AL034550.2	0.32148192	1.74E-13	postive
LOXL3	AL035071.1	0.320819178	1.97E-13	postive
LOXL3	AL035587.1	0.384400696	4.71E-19	postive
LOXL3	AL049569.1	0.383081154	6.35E-19	postive
LOXL3	AL049597.2	0.318941164	2.76E-13	postive
LOXL3	AL049779.1	0.345443154	1.85E-15	postive
LOXL3	AL049830.3	0.305181104	3.08E-12	postive
LOXL3	AL049838.1	0.393542356	5.71E-20	postive
LOXL3	AL049840.4	0.405839749	3.01E-21	postive
LOXL3	AL049840.5	0.433093405	2.82E-24	postive
LOXL3	AL049840.6	0.343361686	2.79E-15	postive
LOXL3	AL078581.2	0.361427866	7.10E-17	postive
LOXL3	AL078644.2	0.364784743	3.50E-17	postive
LOXL3	AL096865.1	0.306916866	2.29E-12	postive
LOXL3	AL109809.1	0.358446056	1.32E-16	postive
LOXL3	AL109811.1	0.350410956	6.85E-16	postive
LOXL3	AL109955.1	0.304803971	3.28E-12	postive
LOXL3	AL117332.1	0.335213154	1.35E-14	postive
LOXL3	AL118506.1	0.31254776	8.60E-13	postive
LOXL3	AL118558.1	0.338767085	6.83E-15	postive
LOXL3	AL121890.4	0.368229384	1.68E-17	postive
LOXL3	AL122035.1	0.423706852	3.34E-23	postive
LOXL3	AL122035.2	0.409585055	1.20E-21	postive

LOXL3	AL132780.1	0.340162746	5.21E-15	postive
LOXL3	AL133338.1	0.341237536	4.23E-15	postive
LOXL3	AL133371.2	0.310104446	1.32E-12	postive
LOXL3	AL133406.2	0.36842086	1.61E-17	postive
LOXL3	AL136084.3	0.345595449	1.79E-15	postive
LOXL3	AL136172.1	0.320062923	2.26E-13	postive
LOXL3	AL136295.2	0.305194923	3.07E-12	postive
LOXL3	AL136368.1	0.310900301	1.15E-12	postive
LOXL3	AL137003.1	0.354735926	2.84E-16	postive
LOXL3	AL137058.2	0.444002286	1.44E-25	postive
LOXL3	AL137186.2	0.340757424	4.64E-15	postive
LOXL3	AL138995.1	0.319413438	2.54E-13	postive
LOXL3	AL139287.1	0.308316553	1.80E-12	postive
LOXL3	AL139339.1	0.348109168	1.09E-15	postive
LOXL3	AL139351.1	0.326767379	6.62E-14	postive
LOXL3	AL139393.3	0.349875005	7.63E-16	postive
LOXL3	AL139423.1	0.367694049	1.88E-17	postive
LOXL3	AL157932.1	0.367808125	1.84E-17	postive
LOXL3	AL158212.3	0.335678455	1.24E-14	postive
LOXL3	AL160006.1	0.349080311	8.95E-16	postive
LOXL3	AL161785.2	0.300798607	6.47E-12	postive
LOXL3	AL161935.1	0.31790531	3.33E-13	postive
LOXL3	AL162377.1	0.337391675	8.91E-15	postive
LOXL3	AL162424.1	0.331453182	2.76E-14	postive
LOXL3	AL162727.1	0.329620424	3.89E-14	postive
LOXL3	AL163195.2	0.396611582	2.77E-20	postive
LOXL3	AL353194.1	0.31952344	2.49E-13	postive
LOXL3	AL353625.1	0.376689948	2.65E-18	postive
LOXL3	AL353751.1	0.338138291	7.71E-15	postive
LOXL3	AL353796.1	0.309205069	1.54E-12	postive
LOXL3	AL354809.1	0.316992819	3.92E-13	postive
LOXL3	AL354811.1	0.397841145	2.07E-20	postive
LOXL3	AL354836.1	0.303738257	3.94E-12	postive
LOXL3	AL354892.2	0.321408894	1.77E-13	postive
LOXL3	AL355075.2	0.343147027	2.91E-15	postive
LOXL3	AL355385.1	0.310374293	1.26E-12	postive
LOXL3	AL356019.2	0.311335515	1.06E-12	postive
LOXL3	AL356299.3	0.325388748	8.54E-14	postive
LOXL3	AL356481.1	0.371650317	8.01E-18	postive
LOXL3	AL356481.3	0.302913077	4.53E-12	postive
LOXL3	AL357874.1	0.328118167	5.15E-14	postive
LOXL3	AL358472.2	0.322159063	1.54E-13	postive
LOXL3	AL358473.1	0.351012379	6.07E-16	postive
LOXL3	AL359317.1	0.324749658	9.61E-14	postive
LOXL3	AL359532.1	0.320532041	2.07E-13	postive
LOXL3	AL359538.1	0.340259627	5.11E-15	postive
LOXL3	AL359643.3	0.321452482	1.75E-13	postive
LOXL3	AL359715.2	0.311377551	1.06E-12	postive
LOXL3	AL360181.2	0.357707899	1.54E-16	postive
LOXL3	AL390955.2	0.3374057	8.88E-15	postive

LOXL3	AL391832.3	0.336945748	9.71E-15	postive
LOXL3	AL450998.2	0.381361742	9.36E-19	postive
LOXL3	AL450998.3	0.359992022	9.59E-17	postive
LOXL3	AL512791.1	0.357369449	1.65E-16	postive
LOXL3	AL513550.1	0.3284256	4.87E-14	postive
LOXL3	AL589743.5	0.34849194	1.01E-15	postive
LOXL3	AL590385.2	0.329349894	4.10E-14	postive
LOXL3	AL590617.2	0.345633783	1.78E-15	postive
LOXL3	AL592166.1	0.32380437	1.14E-13	postive
LOXL3	AL603839.2	0.314177092	6.46E-13	postive
LOXL3	AL606834.1	0.346043251	1.64E-15	postive
LOXL3	AL606834.2	0.333673292	1.81E-14	postive
LOXL3	AL627309.5	0.309486073	1.47E-12	postive
LOXL3	AL645568.1	0.300156193	7.20E-12	postive
LOXL3	AL662844.4	0.328021506	5.25E-14	postive
LOXL3	AL691432.1	0.313097001	7.81E-13	postive
LOXL3	AL731567.1	0.342728581	3.16E-15	postive
LOXL3	AL731577.2	0.328008766	5.26E-14	postive
LOXL3	AP000442.1	0.403493616	5.33E-21	postive
LOXL3	AP000525.1	0.384314837	4.80E-19	postive
LOXL3	AP000640.1	0.351341418	5.67E-16	postive
LOXL3	AP000662.1	0.399460866	1.41E-20	postive
LOXL3	AP000692.1	0.324063378	1.09E-13	postive
LOXL3	AP000695.1	0.346473326	1.51E-15	postive
LOXL3	AP000759.1	0.311720549	9.94E-13	postive
LOXL3	AP001057.1	0.367873377	1.81E-17	postive
LOXL3	AP001107.1	0.374352576	4.44E-18	postive
LOXL3	AP001107.5	0.363565697	4.53E-17	postive
LOXL3	AP001160.1	0.322688266	1.40E-13	postive
LOXL3	AP001189.3	0.329252438	4.17E-14	postive
LOXL3	AP001330.4	0.397842313	2.07E-20	postive
LOXL3	AP001434.1	0.319324498	2.58E-13	postive
LOXL3	AP001462.1	0.321808917	1.64E-13	postive
LOXL3	AP002449.1	0.307519347	2.06E-12	postive
LOXL3	AP002989.1	0.333368977	1.92E-14	postive
LOXL3	AP003071.4	0.378996911	1.59E-18	postive
LOXL3	AP003392.1	0.302522907	4.84E-12	postive
LOXL3	AP003392.4	0.346113994	1.62E-15	postive
LOXL3	AP003486.1	0.335783418	1.21E-14	postive
LOXL3	AP003721.1	0.335335967	1.32E-14	postive
LOXL3	AP005019.1	0.301096513	6.15E-12	postive
LOXL3	AP005432.2	0.306590078	2.42E-12	postive
LOXL3	ARRDC1-AS1	0.327463387	5.82E-14	postive
LOXL3	ASB16-AS1	0.339319609	6.14E-15	postive
LOXL3	ATP6V0E2-AS1	0.379895968	1.30E-18	postive
LOXL3	ATXN1-AS1	0.432674006	3.15E-24	postive
LOXL3	BHLHE40-AS1	0.333213085	1.98E-14	postive
LOXL3	BMS1P20	0.406503695	2.56E-21	postive
LOXL3	BOLA2P2	0.382402865	7.40E-19	postive
LOXL3	BOLA3-AS1	0.353643925	3.55E-16	postive

LOXL3	BTN2A3P	0.574458595	3.05E-45	postive
LOXL3	BX284613.2	0.363843131	4.27E-17	postive
LOXL3	BX322234.1	0.474210535	2.13E-29	postive
LOXL3	BX322557.10	0.300479388	6.82E-12	postive
LOXL3	C10orf25	0.464403188	4.10E-28	postive
LOXL3	C22orf24	0.378277623	1.86E-18	postive
LOXL3	C2orf27A	0.548699327	1.19E-40	postive
LOXL3	C9orf139	0.348850507	9.37E-16	postive
LOXL3	CACNA1C-AS1	0.304575496	3.41E-12	postive
LOXL3	CACTIN-AS1	0.358204805	1.39E-16	postive
LOXL3	CARD8-AS1	0.358345203	1.35E-16	postive
LOXL3	CCDC84-DT	0.361091541	7.62E-17	postive
LOXL3	CCNT2-AS1	0.350996412	6.08E-16	postive
LOXL3	CD27-AS1	0.340274219	5.10E-15	postive
LOXL3	CEBPB-AS1	0.300458215	6.85E-12	postive
LOXL3	CENPBD1P1	0.428491619	9.56E-24	postive
LOXL3	CEP250-AS1	0.356509019	1.98E-16	postive
LOXL3	CEP83-DT	0.409387041	1.26E-21	postive
LOXL3	CETN4P	0.306295469	2.55E-12	postive
LOXL3	CHROMR	0.43877838	6.06E-25	postive
LOXL3	CIDECP1	0.350940616	6.15E-16	postive
LOXL3	COL4A2-AS1	0.393495526	5.77E-20	postive
LOXL3	COL6A4P2	0.320411596	2.12E-13	postive
LOXL3	CRYBB2P1	0.332992437	2.06E-14	postive
LOXL3	CSTF3-DT	0.330935684	3.04E-14	postive
LOXL3	CTC-205M6.5	0.343582778	2.67E-15	postive
LOXL3	CTC-786C10.1	0.398563004	1.74E-20	postive
LOXL3	CTD-2231H16.1	0.311207331	1.09E-12	postive
LOXL3	CTD-2521M24.9	0.350892658	6.21E-16	postive
LOXL3	CTD-2574D22.4	0.330136011	3.53E-14	postive
LOXL3	DACT3-AS1	0.320545814	2.07E-13	postive
LOXL3	DHRS4-AS1	0.351961686	5.00E-16	postive
LOXL3	DNM3OS	0.301651456	5.60E-12	postive
LOXL3	DUBR	0.368911978	1.45E-17	postive
LOXL3	DUXAP8	0.388638603	1.79E-19	postive
LOXL3	DUXAP9	0.358105292	1.42E-16	postive
LOXL3	EAF1-AS1	0.35845998	1.32E-16	postive
LOXL3	EBLN3P	0.373171245	5.75E-18	postive
LOXL3	EIF2AK3-DT	0.314939978	5.64E-13	postive
LOXL3	EPB41L4A-DT	0.312601449	8.52E-13	postive
LOXL3	FAM111A-DT	0.414625808	3.40E-22	postive
LOXL3	FAM66C	0.304360461	3.54E-12	postive
LOXL3	FGD5-AS1	0.4360756	1.26E-24	postive
LOXL3	FGD5P1	0.336301124	1.10E-14	postive
LOXL3	FKBP9P1	0.426821812	1.48E-23	postive
LOXL3	FO393418.1	0.371891575	7.60E-18	postive
LOXL3	FOXCUT	0.326762809	6.63E-14	postive
LOXL3	FOXD2-AS1	0.303416968	4.16E-12	postive
LOXL3	FRG1-DT	0.345356131	1.88E-15	postive
LOXL3	FRG1HP	0.363159016	4.93E-17	postive

LOXL3	FRMD6-AS1	0.354713274	2.86E-16	postive
LOXL3	GCC2-AS1	0.33989889	5.49E-15	postive
LOXL3	GGTA1P	0.362290244	5.93E-17	postive
LOXL3	GNRHR2	0.324960395	9.24E-14	postive
LOXL3	GORAB-AS1	0.318340774	3.08E-13	postive
LOXL3	GS1-358P8.4	0.356934599	1.81E-16	postive
LOXL3	GTF2IP1	0.350647539	6.53E-16	postive
LOXL3	GTF2IP4	0.373843515	4.96E-18	postive
LOXL3	GUSBP5	0.323054978	1.31E-13	postive
LOXL3	H1-9	0.375660544	3.33E-18	postive
LOXL3	HAS2-AS1	0.316697921	4.13E-13	postive
LOXL3	HCG11	0.326912976	6.44E-14	postive
LOXL3	HCG18	0.405136979	3.57E-21	postive
LOXL3	HCG25	0.300655416	6.63E-12	postive
LOXL3	HECW2-AS1	0.33372157	1.80E-14	postive
LOXL3	HM13-IT1	0.390366529	1.20E-19	postive
LOXL3	HMGB1P5	0.354492629	2.99E-16	postive
LOXL3	HOMER3-AS1	0.301072224	6.18E-12	postive
LOXL3	HSPD1P6	0.300420063	6.89E-12	postive
LOXL3	IL10RB-DT	0.309561962	1.45E-12	postive
LOXL3	ILF3-DT	0.352681222	4.32E-16	postive
LOXL3	INKA2-AS1	0.402615149	6.59E-21	postive
LOXL3	IPO5P1	0.416422321	2.16E-22	postive
LOXL3	IPO9-AS1	0.359308735	1.11E-16	postive
LOXL3	KDM2B-DT	0.366522905	2.42E-17	postive
LOXL3	KRT18P59	0.304429497	3.50E-12	postive
LOXL3	KRT8P12	0.316997926	3.91E-13	postive
LOXL3	L3MBTL2-AS1	0.365476327	3.02E-17	postive
LOXL3	L3MBTL4-AS1	0.360998041	7.77E-17	postive
LOXL3	LCT-AS1	0.331900464	2.54E-14	postive
LOXL3	LEF1-AS1	0.318172076	3.17E-13	postive
LOXL3	LINC00115	0.305102444	3.12E-12	postive
LOXL3	LINC00294	0.314038867	6.62E-13	postive
LOXL3	LINC00449	0.355922023	2.23E-16	postive
LOXL3	LINC00471	0.32586488	7.82E-14	postive
LOXL3	LINC00511	0.406193404	2.76E-21	postive
LOXL3	LINC00539	0.300927523	6.33E-12	postive
LOXL3	LINC00545	0.306626973	2.40E-12	postive
LOXL3	LINC00618	0.350087367	7.31E-16	postive
LOXL3	LINC00622	0.310420997	1.25E-12	postive
LOXL3	LINC00630	0.335664651	1.24E-14	postive
LOXL3	LINC00652	0.39199327	8.20E-20	postive
LOXL3	LINC00654	0.33232633	2.34E-14	postive
LOXL3	LINC00664	0.352872047	4.16E-16	postive
LOXL3	LINC00665	0.347714389	1.18E-15	postive
LOXL3	LINC00667	0.305631105	2.85E-12	postive
LOXL3	LINC00888	0.350778112	6.36E-16	postive
LOXL3	LINC00900	0.357111481	1.74E-16	postive
LOXL3	LINC00909	0.30658255	2.42E-12	postive
LOXL3	LINC00937	0.356284114	2.07E-16	postive

LOXL3	LINC00987	0.321613488	1.70E-13	postive
LOXL3	LINC01006	0.317397381	3.64E-13	postive
LOXL3	LINC01050	0.324328405	1.04E-13	postive
LOXL3	LINC01094	0.395999228	3.20E-20	postive
LOXL3	LINC01096	0.349429295	8.35E-16	postive
LOXL3	LINC01122	0.357284291	1.68E-16	postive
LOXL3	LINC01127	0.321773525	1.65E-13	postive
LOXL3	LINC01128	0.438596421	6.36E-25	postive
LOXL3	LINC01140	0.321113257	1.87E-13	postive
LOXL3	LINC01142	0.30917529	1.55E-12	postive
LOXL3	LINC01150	0.342909415	3.05E-15	postive
LOXL3	LINC01224	0.352369083	4.61E-16	postive
LOXL3	LINC01233	0.387779086	2.18E-19	postive
LOXL3	LINC01283	0.389185985	1.57E-19	postive
LOXL3	LINC01410	0.357720089	1.54E-16	postive
LOXL3	LINC01415	0.364284983	3.89E-17	postive
LOXL3	LINC01429	0.352382924	4.59E-16	postive
LOXL3	LINC01521	0.399005042	1.57E-20	postive
LOXL3	LINC01534	0.31409757	6.55E-13	postive
LOXL3	LINC01560	0.409440466	1.24E-21	postive
LOXL3	LINC01561	0.371290422	8.66E-18	postive
LOXL3	LINC01614	0.40572053	3.10E-21	postive
LOXL3	LINC01638	0.311611024	1.01E-12	postive
LOXL3	LINC01678	0.334693209	1.49E-14	postive
LOXL3	LINC01679	0.326458843	7.01E-14	postive
LOXL3	LINC01684	0.313143827	7.75E-13	postive
LOXL3	LINC01775	0.34327037	2.84E-15	postive
LOXL3	LINC01858	0.34454177	2.21E-15	postive
LOXL3	LINC01881	0.320925913	1.93E-13	postive
LOXL3	LINC01943	0.318032201	3.25E-13	postive
LOXL3	LINC01963	0.334782144	1.47E-14	postive
LOXL3	LINC01973	0.419136674	1.08E-22	postive
LOXL3	LINC02019	0.351160517	5.89E-16	postive
LOXL3	LINC02035	0.322508565	1.45E-13	postive
LOXL3	LINC02068	0.303313462	4.23E-12	postive
LOXL3	LINC02210	0.440701655	3.58E-25	postive
LOXL3	LINC02285	0.328950395	4.41E-14	postive
LOXL3	LINC02311	0.346630948	1.46E-15	postive
LOXL3	LINC02391	0.303042486	4.43E-12	postive
LOXL3	LINC02392	0.317378762	3.65E-13	postive
LOXL3	LINC02408	0.37137013	8.51E-18	postive
LOXL3	LINC02611	0.447036235	6.17E-26	postive
LOXL3	LINC02630	0.352765451	4.25E-16	postive
LOXL3	LINC02668	0.310892811	1.15E-12	postive
LOXL3	LINC02693	0.468912162	1.07E-28	postive
LOXL3	LINC02724	0.369859223	1.18E-17	postive
LOXL3	LINC02773	0.301480281	5.77E-12	postive
LOXL3	LINC02803	0.334098305	1.67E-14	postive
LOXL3	LRP1-AS	0.330096302	3.56E-14	postive
LOXL3	LRP4-AS1	0.409708045	1.16E-21	postive

LOXL3	LRRC37A17P	0.379596269	1.39E-18	postive
LOXL3	LRRC37BP1	0.391879745	8.42E-20	postive
LOXL3	LRRC8C-DT	0.356881505	1.83E-16	postive
LOXL3	MAPKAPK5-AS1	0.31936485	2.56E-13	postive
LOXL3	MED14OS	0.348589183	9.88E-16	postive
LOXL3	MIPEPP3	0.377582786	2.18E-18	postive
LOXL3	MIR155HG	0.315848066	4.80E-13	postive
LOXL3	MIR4435-2HG	0.34363484	2.64E-15	postive
LOXL3	MIR4453HG	0.34340847	2.76E-15	postive
LOXL3	MKNK1-AS1	0.34380538	2.56E-15	postive
LOXL3	MRPL20-AS1	0.350157814	7.21E-16	postive
LOXL3	MRPS31P4	0.545622168	3.96E-40	postive
LOXL3	MRPS31P5	0.359685182	1.02E-16	postive
LOXL3	MRPS9-AS1	0.443894102	1.48E-25	postive
LOXL3	MSC-AS1	0.35819698	1.39E-16	postive
LOXL3	MTMR9LP	0.446165633	7.87E-26	postive
LOXL3	MUC20-OT1	0.362303194	5.91E-17	postive
LOXL3	NAIPP1	0.341096974	4.35E-15	postive
LOXL3	NAIPP3	0.337454457	8.80E-15	postive
LOXL3	NARF-IT1	0.307655702	2.01E-12	postive
LOXL3	NBPF25P	0.317922268	3.32E-13	postive
LOXL3	NBPF8	0.347654335	1.19E-15	postive
LOXL3	NCBP2-AS1	0.336198353	1.12E-14	postive
LOXL3	NCF1B	0.355939695	2.22E-16	postive
LOXL3	NFIA-AS2	0.301824601	5.44E-12	postive
LOXL3	NIFK-AS1	0.346638145	1.46E-15	postive
LOXL3	NORAD	0.335715614	1.23E-14	postive
LOXL3	NUTM2A-AS1	0.309558218	1.45E-12	postive
LOXL3	OGFR-AS1	0.321227569	1.83E-13	postive
LOXL3	PAN3-AS1	0.305712961	2.81E-12	postive
LOXL3	PAUPAR	0.345393552	1.87E-15	postive
LOXL3	PCBP1-AS1	0.404367922	4.31E-21	postive
LOXL3	PCED1B-AS1	0.323911733	1.12E-13	postive
LOXL3	PEF1-AS1	0.320036301	2.27E-13	postive
LOXL3	PIK3IP1-AS1	0.32813759	5.13E-14	postive
LOXL3	PINK1-AS	0.426955508	1.43E-23	postive
LOXL3	PKD1P6	0.354957182	2.72E-16	postive
LOXL3	PMS2CL	0.404092695	4.61E-21	postive
LOXL3	PMS2P3	0.302961049	4.49E-12	postive
LOXL3	PMS2P4	0.319700173	2.41E-13	postive
LOXL3	POLH-AS1	0.37750774	2.21E-18	postive
LOXL3	POLR2J4	0.315983148	4.69E-13	postive
LOXL3	PPT2-EGFL8	0.49020839	1.38E-31	postive
LOXL3	PRICKLE2-DT	0.355338647	2.51E-16	postive
LOXL3	PRMT5-AS1	0.313317879	7.51E-13	postive
LOXL3	PSMB8-AS1	0.309936181	1.36E-12	postive
LOXL3	PTENP1	0.328876897	4.47E-14	postive
LOXL3	PTOV1-AS1	0.321616066	1.70E-13	postive
LOXL3	PTPRD-AS1	0.339597096	5.82E-15	postive
LOXL3	PTPRG-AS1	0.316514885	4.26E-13	postive

LOXL3	RAET1K	0.385308599	3.83E-19	postive
LOXL3	RASAL2-AS1	0.439810125	4.57E-25	postive
LOXL3	RASGRF2-AS1	0.329512128	3.97E-14	postive
LOXL3	RNF144A-AS1	0.308141127	1.85E-12	postive
LOXL3	RNF216P1	0.357323906	1.67E-16	postive
LOXL3	RP11-159G9.5	0.36558008	2.95E-17	postive
LOXL3	RP11-15H20.6	0.373206806	5.70E-18	postive
LOXL3	RP11-160O5.1	0.371194425	8.84E-18	postive
LOXL3	RP1-122P22.2	0.39379315	5.39E-20	postive
LOXL3	RP11-22P4.1	0.334399681	1.58E-14	postive
LOXL3	RP11-264E20.1	0.354314321	3.10E-16	postive
LOXL3	RP11-282O18.3	0.404854938	3.83E-21	postive
LOXL3	RP11-346C20.4	0.314250616	6.37E-13	postive
LOXL3	RP11-439L18.2	0.344845158	2.08E-15	postive
LOXL3	RP11-540B6.6	0.323583412	1.19E-13	postive
LOXL3	RP11-556E13.1	0.330203154	3.49E-14	postive
LOXL3	RP11-65I12.1	0.314266983	6.35E-13	postive
LOXL3	RP11-815I9.4	0.514055128	4.52E-35	postive
LOXL3	RP1-93H18.1	0.43714298	9.45E-25	postive
LOXL3	RP5-1039K5.19	0.317693574	3.45E-13	postive
LOXL3	RP5-1125A11.6	0.309019297	1.59E-12	postive
LOXL3	RP5-968J1.1	0.315415234	5.18E-13	postive
LOXL3	RPL23AP7	0.446102038	8.01E-26	postive
LOXL3	RPL23AP82	0.406252073	2.72E-21	postive
LOXL3	RPL32P3	0.358348776	1.35E-16	postive
LOXL3	RRN3P3	0.315295969	5.30E-13	postive
LOXL3	RRP7BP	0.364124638	4.02E-17	postive
LOXL3	RXYLT1-AS1	0.406952969	2.29E-21	postive
LOXL3	SAMMSON	0.300950497	6.31E-12	postive
LOXL3	SAP30-DT	0.447690972	5.13E-26	postive
LOXL3	SBDSP1	0.32335935	1.24E-13	postive
LOXL3	SDHAP1	0.417721755	1.56E-22	postive
LOXL3	SEPTIN4-AS1	0.322788207	1.38E-13	postive
LOXL3	SEPTIN7P2	0.32237214	1.48E-13	postive
LOXL3	SH3GL1P1	0.307184263	2.18E-12	postive
LOXL3	SH3RF3-AS1	0.37212612	7.22E-18	postive
LOXL3	SIGLEC22P	0.399671083	1.34E-20	postive
LOXL3	SMIM25	0.363818289	4.29E-17	postive
LOXL3	SMPD5	0.336400466	1.08E-14	postive
LOXL3	SNHG1	0.316764718	4.08E-13	postive
LOXL3	SNHG20	0.30750706	2.07E-12	postive
LOXL3	SPAG5-AS1	0.326781505	6.60E-14	postive
LOXL3	SREBF2-AS1	0.341386315	4.11E-15	postive
LOXL3	STAG3L1	0.314775849	5.81E-13	postive
LOXL3	STAG3L2	0.357672913	1.55E-16	postive
LOXL3	STAG3L3	0.348711196	9.64E-16	postive
LOXL3	STARD7-AS1	0.385319439	3.82E-19	postive
LOXL3	STX18-AS1	0.349691752	7.92E-16	postive
LOXL3	SUMO2P17	0.321266056	1.81E-13	postive
LOXL3	SUZ12P1	0.354087647	3.25E-16	postive

LOXL3	SVIL2P	0.326971466	6.38E-14	postive
LOXL3	SVIL-AS1	0.407268094	2.12E-21	postive
LOXL3	THAP9-AS1	0.353764635	3.47E-16	postive
LOXL3	TMEM26-AS1	0.332406979	2.31E-14	postive
LOXL3	TMSB15B-AS1	0.317990845	3.28E-13	postive
LOXL3	TNK2-AS1	0.343731439	2.59E-15	postive
LOXL3	TP73-AS1	0.396219529	3.04E-20	postive
LOXL3	TPT1-AS1	0.36912309	1.38E-17	postive
LOXL3	TPTEP2	0.3208336	1.96E-13	postive
LOXL3	TRAF3IP2-AS1	0.456021049	4.78E-27	postive
LOXL3	TSPY26P	0.457283053	3.32E-27	postive
LOXL3	TTC28-AS1	0.359686811	1.02E-16	postive
LOXL3	TUBBP1	0.322488507	1.45E-13	postive
LOXL3	TUG1	0.482122234	1.82E-30	postive
LOXL3	UBA6-AS1	0.325923981	7.74E-14	postive
LOXL3	UBE2E1-AS1	0.318143718	3.19E-13	postive
LOXL3	VDAC1P8	0.344589656	2.19E-15	postive
LOXL3	WAKMAR2	0.300329815	7.00E-12	postive
LOXL3	WARS2-AS1	0.310846108	1.16E-12	postive
LOXL3	WNT5A-AS1	0.366212288	2.58E-17	postive
LOXL3	YEATS2-AS1	0.439134504	5.50E-25	postive
LOXL3	Z83844.2	0.334753874	1.48E-14	postive
LOXL3	Z95115.1	0.35019812	7.15E-16	postive
LOXL3	Z97989.1	0.368338008	1.64E-17	postive
LOXL3	Z98884.2	0.401223514	9.22E-21	postive
LOXL3	ZEB1-AS1	0.36081646	8.07E-17	postive
LOXL3	ZNF137P	0.372372151	6.84E-18	postive
LOXL3	ZNF197-AS1	0.334328713	1.60E-14	postive
LOXL3	ZNF286B	0.514378098	4.04E-35	postive
LOXL3	ZNF37BP	0.315584596	5.03E-13	postive
LOXL3	ZNF433-AS1	0.301623468	5.63E-12	postive
LOXL3	ZNF436-AS1	0.327292539	6.01E-14	postive
LOXL3	ZNF528-AS1	0.369836538	1.19E-17	postive
LOXL3	ZNF529-AS1	0.361515004	6.97E-17	postive
LOXL3	ZNF542P	0.342548573	3.27E-15	postive
LOXL3	ZNF561-AS1	0.30391458	3.82E-12	postive
LOXL3	ZNF571-AS1	0.304103564	3.70E-12	postive
LOXL3	ZNF667-AS1	0.326634784	6.79E-14	postive
LOXL3	ZNF767P	0.31469811	5.89E-13	postive
LOXL3	ZNF788P	0.399710701	1.33E-20	postive
LOXL3	ZNF815P	0.307354993	2.12E-12	postive
LOXL3	ZNRD2-AS1	0.306453071	2.48E-12	postive
LOXL4	AC002429.2	0.424123366	3.00E-23	postive
LOXL4	AC006504.1	0.402981381	6.03E-21	postive
LOXL4	AC006504.5	0.326835832	6.54E-14	postive
LOXL4	AC008517.1	0.452567502	1.29E-26	postive
LOXL4	AC008771.1	0.424746163	2.55E-23	postive
LOXL4	AC009237.14	0.30544167	2.95E-12	postive
LOXL4	AC009495.1	0.301286653	5.96E-12	postive
LOXL4	AC009495.3	0.317682523	3.46E-13	postive

LOXL4	AC011467.4	0.366164968	2.61E-17	postive
LOXL4	AC012050.1	0.32228049	1.51E-13	postive
LOXL4	AC012636.1	0.394728601	4.32E-20	postive
LOXL4	AC013391.3	0.436200792	1.22E-24	postive
LOXL4	AC015853.2	0.330137207	3.53E-14	postive
LOXL4	AC026780.1	0.41358098	4.43E-22	postive
LOXL4	AC060814.2	0.309377533	1.50E-12	postive
LOXL4	AC067747.1	0.445547782	9.35E-26	postive
LOXL4	AC079248.1	0.419286776	1.04E-22	postive
LOXL4	AC087399.1	0.411930946	6.69E-22	postive
LOXL4	AC097359.2	0.305939542	2.71E-12	postive
LOXL4	AC099542.2	0.312345353	8.91E-13	postive
LOXL4	AC103855.3	0.317317278	3.69E-13	postive
LOXL4	AC104819.3	0.333503496	1.87E-14	postive
LOXL4	AC110285.5	0.307677216	2.01E-12	postive
LOXL4	AC114316.1	0.42922729	7.87E-24	postive
LOXL4	AC116345.1	0.312756358	8.29E-13	postive
LOXL4	AL096711.2	0.350627518	6.56E-16	postive
LOXL4	AL096865.1	0.356184764	2.11E-16	postive
LOXL4	AL121781.1	0.349577805	8.10E-16	postive
LOXL4	AL139241.1	0.737796782	4.84E-87	postive
LOXL4	AL139350.1	0.426865044	1.47E-23	postive
LOXL4	AL355592.1	0.311979548	9.50E-13	postive
LOXL4	AL355596.1	0.456324466	4.38E-27	postive
LOXL4	AL356133.1	0.306818355	2.33E-12	postive
LOXL4	AL451137.2	0.318947782	2.76E-13	postive
LOXL4	AL713965.1	0.327523482	5.75E-14	postive
LOXL4	AP000439.1	0.379780987	1.33E-18	postive
LOXL4	AP001207.3	0.343176648	2.89E-15	postive
LOXL4	AP002856.1	0.470078211	7.49E-29	postive
LOXL4	AP002856.3	0.455820365	5.07E-27	postive
LOXL4	AP002856.4	0.337351867	8.98E-15	postive
LOXL4	C15orf56	0.331060435	2.97E-14	postive
LOXL4	C9orf135-DT	0.302371687	4.96E-12	postive
LOXL4	CTC-487M23.5	0.312928047	8.05E-13	postive
LOXL4	GS1-358P8.4	0.303590892	4.04E-12	postive
LOXL4	HEXA-AS1	0.400792916	1.02E-20	postive
LOXL4	KIAA1671-AS1	0.301666475	5.59E-12	postive
LOXL4	LINC00578	0.362371253	5.83E-17	postive
LOXL4	LINC00662	0.409497845	1.22E-21	postive
LOXL4	LINC00928	0.40301423	5.98E-21	postive
LOXL4	LINC01336	0.339175704	6.31E-15	postive
LOXL4	LINC01440	0.339820167	5.57E-15	postive
LOXL4	LINC01483	0.322949405	1.34E-13	postive
LOXL4	LINC01579	0.377935234	2.01E-18	postive
LOXL4	LINC01950	0.52762644	3.52E-37	postive
LOXL4	LINC02234	0.315610029	5.01E-13	postive
LOXL4	LINC02430	0.42084072	7.01E-23	postive
LOXL4	LINC02712	0.301519006	5.73E-12	postive
LOXL4	MIR9-3HG	0.412319112	6.07E-22	postive

LOXL4	NR2F1-AS1	0.381946332	8.20E-19	postive
LOXL4	RP11-1082L8.3	0.469912475	7.87E-29	postive
LOXL4	RP11-164P12.4	0.324671503	9.74E-14	postive
LOXL4	RP11-876N24.5	0.329181585	4.23E-14	postive
LOXL4	SEC24B-AS1	0.335345428	1.32E-14	postive
LOXL4	SNHG19	0.35208175	4.88E-16	postive
LOXL4	TCERG1L-AS1	0.362108753	6.16E-17	postive
LOXL4	VIM-AS1	0.326938441	6.41E-14	postive
LOXL4	Z82186.1	0.304981281	3.19E-12	postive
METTL17	A1BG-AS1	0.32099652	1.91E-13	postive
METTL17	A2M-AS1	0.307314665	2.14E-12	postive
METTL17	AATBC	0.37097384	9.27E-18	postive
METTL17	ABCA11P	0.480245911	3.28E-30	postive
METTL17	AC000068.1	0.45367919	9.38E-27	postive
METTL17	AC000068.2	0.481236201	2.40E-30	postive
METTL17	AC000123.1	0.315636291	4.99E-13	postive
METTL17	AC001226.1	0.354263816	3.13E-16	postive
METTL17	AC002059.1	0.366314871	2.53E-17	postive
METTL17	AC002116.2	0.45176723	1.62E-26	postive
METTL17	AC002128.1	0.514085179	4.47E-35	postive
METTL17	AC002128.2	0.343864184	2.53E-15	postive
METTL17	AC002310.1	0.566656429	8.27E-44	postive
METTL17	AC002310.2	0.360356459	8.89E-17	postive
METTL17	AC002347.1	0.323305901	1.25E-13	postive
METTL17	AC002398.1	0.465465294	2.99E-28	postive
METTL17	AC002467.1	0.37741764	2.26E-18	postive
METTL17	AC002470.1	0.403633697	5.15E-21	postive
METTL17	AC002550.2	0.440274115	4.02E-25	postive
METTL17	AC002553.1	0.494673859	3.22E-32	postive
METTL17	AC003070.1	0.508486201	3.12E-34	postive
METTL17	AC003101.2	0.400630039	1.06E-20	postive
METTL17	AC003102.1	0.571708541	9.86E-45	postive
METTL17	AC003956.1	0.50169773	3.13E-33	postive
METTL17	AC004012.1	0.34147723	4.03E-15	postive
METTL17	AC004067.1	0.402698789	6.46E-21	postive
METTL17	AC004069.1	0.422894576	4.13E-23	postive
METTL17	AC004076.2	0.304310146	3.57E-12	postive
METTL17	AC004148.1	0.557650012	3.35E-42	postive
METTL17	AC004156.1	0.314949288	5.63E-13	postive
METTL17	AC004233.1	0.435886946	1.33E-24	postive
METTL17	AC004241.1	0.441014069	3.28E-25	postive
METTL17	AC004241.3	0.338652951	6.98E-15	postive
METTL17	AC004241.4	0.324422963	1.02E-13	postive
METTL17	AC004253.1	0.568451732	3.90E-44	postive
METTL17	AC004263.1	0.489359812	1.81E-31	postive
METTL17	AC004447.2	0.626553389	7.01E-56	postive
METTL17	AC004466.1	0.506060506	7.14E-34	postive
METTL17	AC004466.3	0.301700423	5.56E-12	postive
METTL17	AC004471.2	0.351323825	5.69E-16	postive
METTL17	AC004477.1	0.517187847	1.50E-35	postive

METTL17	AC004584.1	0.445706419	8.95E-26	postive
METTL17	AC004596.1	0.430860403	5.11E-24	postive
METTL17	AC004687.1	0.350025608	7.40E-16	postive
METTL17	AC004706.1	0.331971574	2.50E-14	postive
METTL17	AC004771.1	0.315741819	4.89E-13	postive
METTL17	AC004771.2	0.544461333	6.21E-40	postive
METTL17	AC004771.4	0.497172804	1.41E-32	postive
METTL17	AC004801.2	0.429033908	8.29E-24	postive
METTL17	AC004801.6	0.389954945	1.32E-19	postive
METTL17	AC004803.1	0.385160892	3.96E-19	postive
METTL17	AC004812.2	0.537300807	9.62E-39	postive
METTL17	AC004825.2	0.432712291	3.12E-24	postive
METTL17	AC004832.6	0.348887399	9.30E-16	postive
METTL17	AC004839.2	0.434526709	1.92E-24	postive
METTL17	AC004840.1	0.33934133	6.11E-15	postive
METTL17	AC004846.2	0.498186323	1.01E-32	postive
METTL17	AC004854.2	0.358391519	1.34E-16	postive
METTL17	AC004865.2	0.409247263	1.30E-21	postive
METTL17	AC004877.1	0.319131319	2.67E-13	postive
METTL17	AC004890.2	0.529089718	2.05E-37	postive
METTL17	AC004893.11	0.566360665	9.36E-44	postive
METTL17	AC004895.1	0.383646735	5.59E-19	postive
METTL17	AC004908.1	0.391037993	1.02E-19	postive
METTL17	AC004908.2	0.434542173	1.91E-24	postive
METTL17	AC004918.1	0.482902354	1.42E-30	postive
METTL17	AC004918.5	0.35004042	7.38E-16	postive
METTL17	AC004923.4	0.369586998	1.25E-17	postive
METTL17	AC004943.2	0.379066356	1.56E-18	postive
METTL17	AC004943.3	0.346161194	1.60E-15	postive
METTL17	AC004951.1	0.446981879	6.26E-26	postive
METTL17	AC004951.4	0.448526732	4.06E-26	postive
METTL17	AC004975.2	0.309919425	1.36E-12	postive
METTL17	AC004980.1	0.551307759	4.25E-41	postive
METTL17	AC004982.2	0.308338267	1.79E-12	postive
METTL17	AC005005.3	0.537094573	1.04E-38	postive
METTL17	AC005005.4	0.39772009	2.13E-20	postive
METTL17	AC005014.2	0.32821035	5.06E-14	postive
METTL17	AC005034.5	0.333780431	1.78E-14	postive
METTL17	AC005034.6	0.309823855	1.38E-12	postive
METTL17	AC005041.3	0.573733743	4.16E-45	postive
METTL17	AC005076.2	0.413015352	5.10E-22	postive
METTL17	AC005083.1	0.34545433	1.84E-15	postive
METTL17	AC005089.1	0.38458164	4.52E-19	postive
METTL17	AC005091.1	0.336301376	1.10E-14	postive
METTL17	AC005104.1	0.536321096	1.39E-38	postive
METTL17	AC005225.1	0.319750186	2.39E-13	postive
METTL17	AC005225.3	0.400235221	1.17E-20	postive
METTL17	AC005229.4	0.40868867	1.50E-21	postive
METTL17	AC005253.1	0.584036309	4.69E-47	postive
METTL17	AC005261.2	0.371479745	8.31E-18	postive

METTL17	AC005261.3	0.412715176	5.50E-22	postive
METTL17	AC005261.4	0.388324808	1.92E-19	postive
METTL17	AC005288.1	0.364770397	3.51E-17	postive
METTL17	AC005306.1	0.517287393	1.45E-35	postive
METTL17	AC005324.1	0.364280704	3.89E-17	postive
METTL17	AC005324.4	0.326359278	7.14E-14	postive
METTL17	AC005324.5	0.401326298	8.99E-21	postive
METTL17	AC005332.1	0.458119593	2.60E-27	postive
METTL17	AC005332.4	0.453819593	9.01E-27	postive
METTL17	AC005363.2	0.372818571	6.21E-18	postive
METTL17	AC005387.1	0.613383219	5.29E-53	postive
METTL17	AC005387.2	0.596993388	1.32E-49	postive
METTL17	AC005391.1	0.402658933	6.52E-21	postive
METTL17	AC005393.1	0.426262692	1.72E-23	postive
METTL17	AC005486.1	0.321632057	1.70E-13	postive
METTL17	AC005498.2	0.338413152	7.32E-15	postive
METTL17	AC005498.3	0.424005532	3.09E-23	postive
METTL17	AC005519.1	0.633029071	2.39E-57	postive
METTL17	AC005520.4	0.40644004	2.60E-21	postive
METTL17	AC005562.1	0.402661842	6.52E-21	postive
METTL17	AC005586.1	0.411538933	7.38E-22	postive
METTL17	AC005670.1	0.356559992	1.95E-16	postive
METTL17	AC005670.3	0.409633431	1.18E-21	postive
METTL17	AC005674.1	0.334207295	1.64E-14	postive
METTL17	AC005674.2	0.388018941	2.06E-19	postive
METTL17	AC005696.1	0.452850953	1.19E-26	postive
METTL17	AC005696.3	0.318338152	3.08E-13	postive
METTL17	AC005759.1	0.318212807	3.15E-13	postive
METTL17	AC005775.1	0.31439238	6.21E-13	postive
METTL17	AC005776.2	0.429915785	6.56E-24	postive
METTL17	AC005785.1	0.588956973	5.20E-48	postive
METTL17	AC005829.1	0.30251022	4.85E-12	postive
METTL17	AC005832.1	0.350585729	6.61E-16	postive
METTL17	AC005837.1	0.37878834	1.66E-18	postive
METTL17	AC005840.2	0.32704687	6.29E-14	postive
METTL17	AC005840.4	0.450012768	2.67E-26	postive
METTL17	AC005841.1	0.376140511	2.99E-18	postive
METTL17	AC005899.7	0.357155625	1.73E-16	postive
METTL17	AC005911.1	0.545562041	4.05E-40	postive
METTL17	AC005920.1	0.300073246	7.30E-12	postive
METTL17	AC005920.3	0.315531751	5.08E-13	postive
METTL17	AC005954.1	0.435244858	1.58E-24	postive
METTL17	AC006001.2	0.511757166	1.01E-34	postive
METTL17	AC006001.3	0.551614964	3.77E-41	postive
METTL17	AC006017.1	0.339666875	5.74E-15	postive
METTL17	AC006042.1	0.495112946	2.79E-32	postive
METTL17	AC006064.3	0.429901657	6.59E-24	postive
METTL17	AC006111.2	0.439512697	4.96E-25	postive
METTL17	AC006111.3	0.306209126	2.58E-12	postive
METTL17	AC006116.11	0.365937366	2.74E-17	postive

METTL17	AC006160.1	0.312189775	9.16E-13	postive
METTL17	AC006213.1	0.341752237	3.82E-15	postive
METTL17	AC006252.1	0.537148461	1.02E-38	postive
METTL17	AC006254.1	0.458828399	2.12E-27	postive
METTL17	AC006333.1	0.39340344	5.90E-20	postive
METTL17	AC006435.2	0.589159987	4.75E-48	postive
METTL17	AC006449.2	0.526537134	5.23E-37	postive
METTL17	AC006449.3	0.333002578	2.06E-14	postive
METTL17	AC006449.5	0.411048645	8.33E-22	postive
METTL17	AC006449.7	0.52221713	2.50E-36	postive
METTL17	AC006480.3	0.339039811	6.48E-15	postive
METTL17	AC006483.2	0.317943034	3.30E-13	postive
METTL17	AC006504.5	0.343884439	2.52E-15	postive
METTL17	AC006547.1	0.498929538	7.89E-33	postive
METTL17	AC006557.1	0.365145523	3.24E-17	postive
METTL17	AC006942.1	0.451624487	1.69E-26	postive
METTL17	AC006946.2	0.306563652	2.43E-12	postive
METTL17	AC006946.3	0.327415533	5.87E-14	postive
METTL17	AC006994.2	0.341365584	4.12E-15	postive
METTL17	AC007032.1	0.358684729	1.26E-16	postive
METTL17	AC007038.1	0.475811579	1.30E-29	postive
METTL17	AC007066.2	0.603028002	7.81E-51	postive
METTL17	AC007098.1	0.405903402	2.96E-21	postive
METTL17	AC007114.1	0.398600845	1.73E-20	postive
METTL17	AC007216.4	0.380978336	1.02E-18	postive
METTL17	AC007220.1	0.329595321	3.91E-14	postive
METTL17	AC007228.9	0.305385801	2.97E-12	postive
METTL17	AC007292.1	0.497237166	1.38E-32	postive
METTL17	AC007292.2	0.499094188	7.46E-33	postive
METTL17	AC007308.6	0.395841148	3.33E-20	postive
METTL17	AC007383.2	0.51725915	1.46E-35	postive
METTL17	AC007405.4	0.364794232	3.49E-17	postive
METTL17	AC007406.5	0.347302298	1.28E-15	postive
METTL17	AC007541.1	0.391537129	9.12E-20	postive
METTL17	AC007566.1	0.477850071	6.92E-30	postive
METTL17	AC007613.1	0.339479878	5.95E-15	postive
METTL17	AC007663.3	0.370714513	9.81E-18	postive
METTL17	AC007663.4	0.506222981	6.76E-34	postive
METTL17	AC007686.3	0.583416428	6.17E-47	postive
METTL17	AC007848.1	0.332523286	2.26E-14	postive
METTL17	AC007879.3	0.366609102	2.37E-17	postive
METTL17	AC007879.4	0.347118654	1.33E-15	postive
METTL17	AC007881.3	0.346726541	1.43E-15	postive
METTL17	AC007938.1	0.332191004	2.40E-14	postive
METTL17	AC007938.3	0.32914437	4.26E-14	postive
METTL17	AC007950.2	0.322637358	1.41E-13	postive
METTL17	AC007993.2	0.329757391	3.79E-14	postive
METTL17	AC008013.2	0.350377654	6.89E-16	postive
METTL17	AC008073.2	0.31532443	5.27E-13	postive
METTL17	AC008079.1	0.34668371	1.45E-15	postive

METTL17	AC008105.1	0.4913002	9.68E-32	postive
METTL17	AC008105.2	0.350731415	6.42E-16	postive
METTL17	AC008115.3	0.341667952	3.89E-15	postive
METTL17	AC008124.1	0.458789432	2.14E-27	postive
METTL17	AC008267.5	0.446416761	7.34E-26	postive
METTL17	AC008280.3	0.31596033	4.71E-13	postive
METTL17	AC008393.1	0.441361942	2.98E-25	postive
METTL17	AC008434.1	0.398373138	1.82E-20	postive
METTL17	AC008438.1	0.518625259	9.03E-36	postive
METTL17	AC008443.1	0.587069326	1.21E-47	postive
METTL17	AC008443.5	0.351624295	5.36E-16	postive
METTL17	AC008443.6	0.327496162	5.78E-14	postive
METTL17	AC008494.3	0.355576346	2.39E-16	postive
METTL17	AC008522.1	0.328274438	5.00E-14	postive
METTL17	AC008543.1	0.417565655	1.62E-22	postive
METTL17	AC008569.2	0.329577757	3.92E-14	postive
METTL17	AC008592.1	0.303560858	4.06E-12	postive
METTL17	AC008592.4	0.334793209	1.47E-14	postive
METTL17	AC008608.2	0.390570266	1.14E-19	postive
METTL17	AC008610.1	0.425564647	2.06E-23	postive
METTL17	AC008622.2	0.458360206	2.43E-27	postive
METTL17	AC008635.2	0.374794415	4.03E-18	postive
METTL17	AC008655.2	0.490705547	1.17E-31	postive
METTL17	AC008687.3	0.345357826	1.88E-15	postive
METTL17	AC008731.1	0.305429872	2.95E-12	postive
METTL17	AC008734.1	0.454694819	7.01E-27	postive
METTL17	AC008735.1	0.571877247	9.18E-45	postive
METTL17	AC008735.2	0.583539949	5.84E-47	postive
METTL17	AC008735.4	0.524075215	1.28E-36	postive
METTL17	AC008750.5	0.428075058	1.07E-23	postive
METTL17	AC008752.2	0.373213687	5.69E-18	postive
METTL17	AC008758.4	0.386104084	3.19E-19	postive
METTL17	AC008760.1	0.466981236	1.90E-28	postive
METTL17	AC008763.1	0.45117865	1.92E-26	postive
METTL17	AC008764.2	0.45322579	1.07E-26	postive
METTL17	AC008764.6	0.599842833	3.50E-50	postive
METTL17	AC008764.8	0.538673598	5.72E-39	postive
METTL17	AC008771.1	0.316794658	4.06E-13	postive
METTL17	AC008781.1	0.444686295	1.19E-25	postive
METTL17	AC008870.3	0.562007952	5.67E-43	postive
METTL17	AC008870.5	0.473277921	2.83E-29	postive
METTL17	AC008894.2	0.408406595	1.60E-21	postive
METTL17	AC008915.3	0.509945261	1.89E-34	postive
METTL17	AC008946.1	0.347521136	1.22E-15	postive
METTL17	AC008966.1	0.328643986	4.67E-14	postive
METTL17	AC008969.1	0.496595541	1.71E-32	postive
METTL17	AC008982.2	0.433459885	2.55E-24	postive
METTL17	AC009005.1	0.40443389	4.24E-21	postive
METTL17	AC009019.1	0.400723498	1.04E-20	postive
METTL17	AC009022.1	0.520069336	5.40E-36	postive

METTL17	AC009041.3	0.310457648	1.24E-12	postive
METTL17	AC009053.3	0.338853903	6.72E-15	postive
METTL17	AC009061.2	0.487372497	3.43E-31	postive
METTL17	AC009065.5	0.542330375	1.41E-39	postive
METTL17	AC009065.7	0.390927568	1.05E-19	postive
METTL17	AC009065.9	0.65449606	1.81E-62	postive
METTL17	AC009087.1	0.437900109	7.69E-25	postive
METTL17	AC009088.1	0.401088799	9.52E-21	postive
METTL17	AC009090.1	0.364737278	3.53E-17	postive
METTL17	AC009097.2	0.355564262	2.40E-16	postive
METTL17	AC009107.2	0.486323744	4.80E-31	postive
METTL17	AC009113.1	0.509105574	2.52E-34	postive
METTL17	AC009118.2	0.322299374	1.50E-13	postive
METTL17	AC009118.3	0.523024616	1.87E-36	postive
METTL17	AC009120.2	0.495026328	2.87E-32	postive
METTL17	AC009120.3	0.390225334	1.24E-19	postive
METTL17	AC009120.5	0.336280757	1.10E-14	postive
METTL17	AC009121.3	0.486315343	4.81E-31	postive
METTL17	AC009121.4	0.410481727	9.59E-22	postive
METTL17	AC009126.1	0.52089207	4.02E-36	postive
METTL17	AC009148.1	0.466403009	2.26E-28	postive
METTL17	AC009159.3	0.374814759	4.01E-18	postive
METTL17	AC009163.7	0.477744191	7.15E-30	postive
METTL17	AC009171.2	0.384288355	4.83E-19	postive
METTL17	AC009226.1	0.308830444	1.64E-12	postive
METTL17	AC009237.14	0.302230379	5.08E-12	postive
METTL17	AC009283.1	0.355017532	2.68E-16	postive
METTL17	AC009309.2	0.398002518	1.99E-20	postive
METTL17	AC009403.1	0.454025339	8.49E-27	postive
METTL17	AC009404.1	0.455615899	5.37E-27	postive
METTL17	AC009506.2	0.444886045	1.12E-25	postive
METTL17	AC009570.1	0.345664044	1.77E-15	postive
METTL17	AC009630.2	0.351085874	5.98E-16	postive
METTL17	AC009690.2	0.669433173	2.76E-66	postive
METTL17	AC009779.3	0.522014164	2.69E-36	postive
METTL17	AC009812.1	0.30320021	4.31E-12	postive
METTL17	AC009831.1	0.383863478	5.32E-19	postive
METTL17	AC009950.1	0.308066679	1.88E-12	postive
METTL17	AC009955.3	0.328327519	4.96E-14	postive
METTL17	AC009955.4	0.315209108	5.38E-13	postive
METTL17	AC009970.1	0.332634029	2.21E-14	postive
METTL17	AC010136.1	0.33949206	5.94E-15	postive
METTL17	AC010201.1	0.437843903	7.81E-25	postive
METTL17	AC010201.2	0.445323914	9.96E-26	postive
METTL17	AC010203.1	0.361413114	7.12E-17	postive
METTL17	AC010203.2	0.349770023	7.79E-16	postive
METTL17	AC010245.2	0.337677608	8.43E-15	postive
METTL17	AC010271.1	0.456047131	4.75E-27	postive
METTL17	AC010319.4	0.604229365	4.42E-51	postive
METTL17	AC010320.2	0.33211639	2.44E-14	postive

METTL17	AC010326.4	0.443970014	1.45E-25	postive
METTL17	AC010326.5	0.422612533	4.44E-23	postive
METTL17	AC010331.1	0.361955621	6.36E-17	postive
METTL17	AC010336.2	0.346373274	1.54E-15	postive
METTL17	AC010359.2	0.417787085	1.53E-22	postive
METTL17	AC010422.1	0.419830281	9.08E-23	postive
METTL17	AC010422.2	0.498361645	9.52E-33	postive
METTL17	AC010442.1	0.370117463	1.12E-17	postive
METTL17	AC010463.3	0.598653815	6.10E-50	postive
METTL17	AC010491.1	0.425796762	1.94E-23	postive
METTL17	AC010504.1	0.447430097	5.52E-26	postive
METTL17	AC010525.1	0.493918334	4.12E-32	postive
METTL17	AC010530.1	0.417961315	1.46E-22	postive
METTL17	AC010531.5	0.32661278	6.81E-14	postive
METTL17	AC010531.6	0.365327771	3.12E-17	postive
METTL17	AC010536.1	0.312229915	9.09E-13	postive
METTL17	AC010542.6	0.471195366	5.34E-29	postive
METTL17	AC010547.1	0.370620333	1.00E-17	postive
METTL17	AC010618.2	0.492780657	5.98E-32	postive
METTL17	AC010618.3	0.551955248	3.29E-41	postive
METTL17	AC010655.2	0.30382312	3.88E-12	postive
METTL17	AC010655.4	0.339470358	5.96E-15	postive
METTL17	AC010680.4	0.302059997	5.23E-12	postive
METTL17	AC010719.1	0.481797302	2.01E-30	postive
METTL17	AC010761.1	0.560447933	1.07E-42	postive
METTL17	AC010761.4	0.532904749	5.01E-38	postive
METTL17	AC010761.6	0.412815591	5.36E-22	postive
METTL17	AC010809.1	0.425097081	2.33E-23	postive
METTL17	AC010809.2	0.4356532	1.41E-24	postive
METTL17	AC010834.2	0.34677866	1.42E-15	postive
METTL17	AC010864.1	0.488637067	2.29E-31	postive
METTL17	AC010883.1	0.515898432	2.37E-35	postive
METTL17	AC010894.2	0.358970561	1.19E-16	postive
METTL17	AC010904.2	0.308122171	1.86E-12	postive
METTL17	AC010913.1	0.398569468	1.74E-20	postive
METTL17	AC010969.2	0.410049805	1.07E-21	postive
METTL17	AC010973.1	0.478398654	5.83E-30	postive
METTL17	AC010973.2	0.568620383	3.63E-44	postive
METTL17	AC010976.1	0.314980085	5.60E-13	postive
METTL17	AC010997.2	0.386432587	2.96E-19	postive
METTL17	AC010997.4	0.566046245	1.07E-43	postive
METTL17	AC010997.5	0.478582376	5.51E-30	postive
METTL17	AC010998.2	0.356472506	1.99E-16	postive
METTL17	AC010999.2	0.364365892	3.82E-17	postive
METTL17	AC011005.4	0.411164846	8.10E-22	postive
METTL17	AC011290.1	0.301954227	5.33E-12	postive
METTL17	AC011330.2	0.327071328	6.26E-14	postive
METTL17	AC011337.1	0.344974652	2.03E-15	postive
METTL17	AC011444.1	0.308491831	1.74E-12	postive
METTL17	AC011450.1	0.40975698	1.15E-21	postive

METTL17	AC011451.1	0.323647164	1.18E-13	postive
METTL17	AC011461.1	0.434410311	1.98E-24	postive
METTL17	AC011462.4	0.541723736	1.78E-39	postive
METTL17	AC011466.3	0.436351161	1.17E-24	postive
METTL17	AC011468.1	0.363086839	5.01E-17	postive
METTL17	AC011468.2	0.303704341	3.96E-12	postive
METTL17	AC011468.5	0.349373768	8.44E-16	postive
METTL17	AC011472.1	0.578701665	4.88E-46	postive
METTL17	AC011472.2	0.308554272	1.72E-12	postive
METTL17	AC011476.3	0.393554135	5.70E-20	postive
METTL17	AC011477.1	0.363063671	5.04E-17	postive
METTL17	AC011477.2	0.374496789	4.30E-18	postive
METTL17	AC011479.3	0.382808518	6.75E-19	postive
METTL17	AC011481.1	0.537796664	7.97E-39	postive
METTL17	AC011487.1	0.351432561	5.57E-16	postive
METTL17	AC011491.2	0.433919127	2.26E-24	postive
METTL17	AC011498.3	0.415482191	2.74E-22	postive
METTL17	AC011498.6	0.477411514	7.92E-30	postive
METTL17	AC011511.1	0.496302946	1.88E-32	postive
METTL17	AC011511.3	0.319912909	2.32E-13	postive
METTL17	AC011603.3	0.34147448	4.04E-15	postive
METTL17	AC011731.1	0.443095232	1.85E-25	postive
METTL17	AC011773.3	0.312128903	9.26E-13	postive
METTL17	AC011825.4	0.345033674	2.01E-15	postive
METTL17	AC011921.1	0.417422861	1.68E-22	postive
METTL17	AC012063.1	0.472672904	3.40E-29	postive
METTL17	AC012073.1	0.356347331	2.04E-16	postive
METTL17	AC012146.3	0.332575154	2.23E-14	postive
METTL17	AC012173.1	0.414806942	3.25E-22	postive
METTL17	AC012181.1	0.328766473	4.57E-14	postive
METTL17	AC012181.2	0.354154761	3.20E-16	postive
METTL17	AC012184.3	0.430199262	6.09E-24	postive
METTL17	AC012186.2	0.525420627	7.86E-37	postive
METTL17	AC012236.1	0.325259313	8.75E-14	postive
METTL17	AC012306.2	0.454722323	6.95E-27	postive
METTL17	AC012313.1	0.474143883	2.17E-29	postive
METTL17	AC012313.2	0.354633398	2.90E-16	postive
METTL17	AC012313.3	0.34419207	2.37E-15	postive
METTL17	AC012313.6	0.415319449	2.86E-22	postive
METTL17	AC012313.8	0.376693054	2.65E-18	postive
METTL17	AC012313.9	0.354129825	3.22E-16	postive
METTL17	AC012360.3	0.510655051	1.48E-34	postive
METTL17	AC012435.2	0.486099788	5.16E-31	postive
METTL17	AC012435.3	0.358758728	1.24E-16	postive
METTL17	AC012442.2	0.367114076	2.13E-17	postive
METTL17	AC012467.2	0.46931236	9.44E-29	postive
METTL17	AC012485.1	0.333523813	1.87E-14	postive
METTL17	AC012510.1	0.388696364	1.76E-19	postive
METTL17	AC012615.1	0.569763741	2.25E-44	postive
METTL17	AC012615.4	0.354301315	3.11E-16	postive

METTL17	AC012615.6	0.598837854	5.60E-50	postive
METTL17	AC012640.1	0.391059644	1.02E-19	postive
METTL17	AC012640.4	0.339841942	5.55E-15	postive
METTL17	AC012645.3	0.403260617	5.64E-21	postive
METTL17	AC012645.4	0.415553527	2.69E-22	postive
METTL17	AC012676.1	0.341315709	4.16E-15	postive
METTL17	AC012676.3	0.396195757	3.06E-20	postive
METTL17	AC012676.4	0.416256224	2.26E-22	postive
METTL17	AC013356.2	0.430144255	6.18E-24	postive
METTL17	AC013400.1	0.355321164	2.52E-16	postive
METTL17	AC013468.1	0.419428037	1.01E-22	postive
METTL17	AC013472.2	0.331232665	2.88E-14	postive
METTL17	AC013553.4	0.324596012	9.88E-14	postive
METTL17	AC013652.1	0.333001114	2.06E-14	postive
METTL17	AC013726.2	0.315022949	5.56E-13	postive
METTL17	AC013731.1	0.406757764	2.40E-21	postive
METTL17	AC015660.3	0.326624905	6.80E-14	postive
METTL17	AC015712.1	0.328973139	4.39E-14	postive
METTL17	AC015726.1	0.405893113	2.97E-21	postive
METTL17	AC015795.1	0.369754927	1.21E-17	postive
METTL17	AC015802.3	0.412186426	6.28E-22	postive
METTL17	AC015802.4	0.490242744	1.36E-31	postive
METTL17	AC015802.5	0.45536292	5.78E-27	postive
METTL17	AC015813.1	0.385362592	3.78E-19	postive
METTL17	AC015818.2	0.338990958	6.54E-15	postive
METTL17	AC015818.3	0.30120175	6.04E-12	postive
METTL17	AC015819.1	0.348812643	9.44E-16	postive
METTL17	AC015849.3	0.45536281	5.78E-27	postive
METTL17	AC015853.1	0.307238077	2.16E-12	postive
METTL17	AC015871.3	0.456990569	3.61E-27	postive
METTL17	AC015909.2	0.427627914	1.20E-23	postive
METTL17	AC015912.3	0.355943234	2.22E-16	postive
METTL17	AC015961.2	0.427993699	1.09E-23	postive
METTL17	AC015982.2	0.453357763	1.03E-26	postive
METTL17	AC016027.1	0.406122235	2.81E-21	postive
METTL17	AC016065.1	0.47169103	4.59E-29	postive
METTL17	AC016355.1	0.398365176	1.83E-20	postive
METTL17	AC016394.2	0.566944329	7.34E-44	postive
METTL17	AC016394.3	0.354450541	3.01E-16	postive
METTL17	AC016405.3	0.365035816	3.32E-17	postive
METTL17	AC016526.2	0.43706301	9.66E-25	postive
METTL17	AC016586.1	0.363138198	4.96E-17	postive
METTL17	AC016590.3	0.310519227	1.23E-12	postive
METTL17	AC016722.2	0.477992311	6.62E-30	postive
METTL17	AC016727.1	0.418324452	1.33E-22	postive
METTL17	AC016737.1	0.551653673	3.71E-41	postive
METTL17	AC016738.1	0.335318724	1.33E-14	postive
METTL17	AC016747.4	0.340264019	5.11E-15	postive
METTL17	AC016773.2	0.524319769	1.17E-36	postive
METTL17	AC016876.2	0.42791664	1.11E-23	postive

METTL17	AC016876.3	0.607918829	7.55E-52	postive
METTL17	AC016876.4	0.429564882	7.20E-24	postive
METTL17	AC017083.1	0.535210301	2.11E-38	postive
METTL17	AC018413.1	0.338433991	7.29E-15	postive
METTL17	AC018521.5	0.361990193	6.31E-17	postive
METTL17	AC018521.6	0.407312667	2.10E-21	postive
METTL17	AC018521.7	0.437287948	9.09E-25	postive
METTL17	AC018638.6	0.349962259	7.50E-16	postive
METTL17	AC018638.7	0.376356552	2.85E-18	postive
METTL17	AC018647.2	0.387334817	2.41E-19	postive
METTL17	AC018648.1	0.338379987	7.36E-15	postive
METTL17	AC018653.3	0.505595786	8.37E-34	postive
METTL17	AC018690.1	0.462911822	6.38E-28	postive
METTL17	AC018695.4	0.47154329	4.80E-29	postive
METTL17	AC018755.1	0.331650579	2.66E-14	postive
METTL17	AC018755.3	0.345102065	1.98E-15	postive
METTL17	AC018766.1	0.408150959	1.71E-21	postive
METTL17	AC018766.4	0.621589586	8.84E-55	postive
METTL17	AC018809.1	0.525865733	6.68E-37	postive
METTL17	AC018809.2	0.389546297	1.45E-19	postive
METTL17	AC018845.3	0.456612032	4.03E-27	postive
METTL17	AC019069.1	0.303707516	3.96E-12	postive
METTL17	AC019131.2	0.342293461	3.44E-15	postive
METTL17	AC019205.1	0.531469632	8.53E-38	postive
METTL17	AC019294.2	0.558972418	1.96E-42	postive
METTL17	AC020558.1	0.343017933	2.98E-15	postive
METTL17	AC020558.2	0.583797841	5.21E-47	postive
METTL17	AC020594.1	0.324167553	1.07E-13	postive
METTL17	AC020658.5	0.310164324	1.30E-12	postive
METTL17	AC020663.2	0.360434941	8.74E-17	postive
METTL17	AC020765.2	0.391047653	1.02E-19	postive
METTL17	AC020904.2	0.323768861	1.15E-13	postive
METTL17	AC020907.4	0.562892368	3.94E-43	postive
METTL17	AC020908.1	0.329858146	3.72E-14	postive
METTL17	AC020910.5	0.34377623	2.57E-15	postive
METTL17	AC020911.1	0.480545022	2.98E-30	postive
METTL17	AC020913.2	0.381992123	8.12E-19	postive
METTL17	AC020915.1	0.309648808	1.43E-12	postive
METTL17	AC020915.2	0.370536921	1.02E-17	postive
METTL17	AC020915.3	0.508979829	2.63E-34	postive
METTL17	AC020916.1	0.322671711	1.41E-13	postive
METTL17	AC020931.1	0.340319567	5.06E-15	postive
METTL17	AC020978.1	0.326328015	7.18E-14	postive
METTL17	AC020978.2	0.343297775	2.82E-15	postive
METTL17	AC020978.4	0.320187398	2.21E-13	postive
METTL17	AC020978.7	0.342116133	3.56E-15	postive
METTL17	AC021016.3	0.409503522	1.22E-21	postive
METTL17	AC021148.1	0.375884885	3.17E-18	postive
METTL17	AC021205.3	0.35771635	1.54E-16	postive
METTL17	AC021242.3	0.354256106	3.14E-16	postive

METTL17	AC021321.1	0.354933901	2.73E-16	postive
METTL17	AC021491.2	0.410269914	1.01E-21	postive
METTL17	AC021739.3	0.304806358	3.28E-12	postive
METTL17	AC021752.1	0.321236187	1.82E-13	postive
METTL17	AC021755.2	0.498143061	1.02E-32	postive
METTL17	AC021755.3	0.343639355	2.64E-15	postive
METTL17	AC021851.1	0.337437915	8.83E-15	postive
METTL17	AC022007.1	0.466636864	2.11E-28	postive
METTL17	AC022098.1	0.529105944	2.04E-37	postive
METTL17	AC022101.1	0.349008383	9.08E-16	postive
METTL17	AC022150.2	0.506217194	6.77E-34	postive
METTL17	AC022154.1	0.469543241	8.80E-29	postive
METTL17	AC022167.2	0.519361925	6.95E-36	postive
METTL17	AC022167.4	0.317782469	3.40E-13	postive
METTL17	AC022211.3	0.400742406	1.03E-20	postive
METTL17	AC022211.4	0.3570921	1.75E-16	postive
METTL17	AC022296.4	0.3964436	2.88E-20	postive
METTL17	AC022306.2	0.302803367	4.61E-12	postive
METTL17	AC022364.1	0.315740968	4.89E-13	postive
METTL17	AC022382.1	0.307861263	1.94E-12	postive
METTL17	AC022400.1	0.43182018	3.96E-24	postive
METTL17	AC022400.5	0.417904812	1.48E-22	postive
METTL17	AC022400.6	0.403528628	5.28E-21	postive
METTL17	AC022415.2	0.46705707	1.86E-28	postive
METTL17	AC022558.3	0.374292929	4.50E-18	postive
METTL17	AC022748.2	0.305825558	2.76E-12	postive
METTL17	AC022762.2	0.425088322	2.33E-23	postive
METTL17	AC022784.2	0.318214101	3.15E-13	postive
METTL17	AC022784.6	0.3881204	2.01E-19	postive
METTL17	AC022819.1	0.416374268	2.19E-22	postive
METTL17	AC022916.2	0.422585875	4.47E-23	postive
METTL17	AC022973.5	0.303103617	4.38E-12	postive
METTL17	AC023302.1	0.393179209	6.22E-20	postive
METTL17	AC023355.2	0.469600129	8.65E-29	postive
METTL17	AC023509.1	0.321828632	1.64E-13	postive
METTL17	AC023509.3	0.422369383	4.73E-23	postive
METTL17	AC023632.2	0.364309447	3.87E-17	postive
METTL17	AC023790.2	0.351354031	5.66E-16	postive
METTL17	AC023794.2	0.316965563	3.93E-13	postive
METTL17	AC023908.3	0.52560683	7.34E-37	postive
METTL17	AC023983.2	0.411754945	6.99E-22	postive
METTL17	AC024060.2	0.554979502	9.84E-42	postive
METTL17	AC024075.1	0.341478075	4.03E-15	postive
METTL17	AC024075.2	0.505221977	9.50E-34	postive
METTL17	AC024075.3	0.301000244	6.25E-12	postive
METTL17	AC024243.1	0.481147995	2.47E-30	postive
METTL17	AC024257.3	0.469335057	9.37E-29	postive
METTL17	AC024257.4	0.31090628	1.15E-12	postive
METTL17	AC024267.4	0.429365585	7.59E-24	postive
METTL17	AC024337.2	0.300833431	6.43E-12	postive

METTL17	AC024361.1	0.389017326	1.64E-19	postive
METTL17	AC024361.2	0.400263918	1.16E-20	postive
METTL17	AC024361.3	0.452570985	1.29E-26	postive
METTL17	AC024560.1	0.301307479	5.94E-12	postive
METTL17	AC024560.3	0.352761069	4.25E-16	postive
METTL17	AC024575.2	0.545531154	4.10E-40	postive
METTL17	AC024884.2	0.318423444	3.03E-13	postive
METTL17	AC024896.1	0.337676437	8.43E-15	postive
METTL17	AC024941.2	0.337703874	8.39E-15	postive
METTL17	AC025048.2	0.498287365	9.76E-33	postive
METTL17	AC025048.4	0.309792142	1.39E-12	postive
METTL17	AC025162.2	0.403017467	5.98E-21	postive
METTL17	AC025165.1	0.387555853	2.29E-19	postive
METTL17	AC025171.1	0.402750535	6.38E-21	postive
METTL17	AC025171.2	0.39224834	7.73E-20	postive
METTL17	AC025171.5	0.390230131	1.24E-19	postive
METTL17	AC025176.1	0.332326326	2.34E-14	postive
METTL17	AC025178.2	0.404526093	4.15E-21	postive
METTL17	AC025181.2	0.337553516	8.64E-15	postive
METTL17	AC025265.1	0.443849713	1.50E-25	postive
METTL17	AC025287.2	0.303498634	4.10E-12	postive
METTL17	AC025287.3	0.501124026	3.79E-33	postive
METTL17	AC025580.2	0.344549115	2.21E-15	postive
METTL17	AC025682.2	0.40032857	1.14E-20	postive
METTL17	AC025754.2	0.301629002	5.63E-12	postive
METTL17	AC025857.2	0.418283938	1.35E-22	postive
METTL17	AC026150.2	0.308343741	1.79E-12	postive
METTL17	AC026254.2	0.34970683	7.89E-16	postive
METTL17	AC026304.1	0.396901928	2.59E-20	postive
METTL17	AC026333.4	0.387078659	2.56E-19	postive
METTL17	AC026367.1	0.359559571	1.05E-16	postive
METTL17	AC026367.2	0.306723425	2.36E-12	postive
METTL17	AC026367.3	0.309713104	1.41E-12	postive
METTL17	AC026401.3	0.390539697	1.15E-19	postive
METTL17	AC026471.1	0.525096142	8.84E-37	postive
METTL17	AC026471.2	0.405343158	3.40E-21	postive
METTL17	AC026471.4	0.552968785	2.20E-41	postive
METTL17	AC026495.1	0.353292597	3.82E-16	postive
METTL17	AC026691.1	0.471098995	5.50E-29	postive
METTL17	AC026740.1	0.329625633	3.89E-14	postive
METTL17	AC026771.1	0.324371282	1.03E-13	postive
METTL17	AC026803.2	0.394947572	4.11E-20	postive
METTL17	AC026979.1	0.310170332	1.30E-12	postive
METTL17	AC026979.2	0.366738729	2.31E-17	postive
METTL17	AC026979.3	0.338073174	7.81E-15	postive
METTL17	AC027020.2	0.438438498	6.64E-25	postive
METTL17	AC027097.1	0.450489076	2.33E-26	postive
METTL17	AC027097.2	0.367661186	1.89E-17	postive
METTL17	AC027237.4	0.340853806	4.56E-15	postive
METTL17	AC027237.5	0.31426648	6.35E-13	postive

METTL17	AC027271.1	0.393934615	5.21E-20	postive
METTL17	AC027307.1	0.388240374	1.96E-19	postive
METTL17	AC027307.2	0.317705387	3.45E-13	postive
METTL17	AC027559.1	0.331911643	2.53E-14	postive
METTL17	AC027601.1	0.328878335	4.47E-14	postive
METTL17	AC027601.2	0.627226696	4.95E-56	postive
METTL17	AC027601.3	0.449948792	2.72E-26	postive
METTL17	AC027607.1	0.361365468	7.20E-17	postive
METTL17	AC027644.3	0.429048781	8.25E-24	postive
METTL17	AC027682.1	0.430088432	6.27E-24	postive
METTL17	AC027682.4	0.419903572	8.91E-23	postive
METTL17	AC027682.5	0.387778261	2.18E-19	postive
METTL17	AC027682.6	0.360190545	9.20E-17	postive
METTL17	AC027763.2	0.308097379	1.87E-12	postive
METTL17	AC027796.4	0.492163395	7.31E-32	postive
METTL17	AC032044.1	0.486679777	4.29E-31	postive
METTL17	AC034102.5	0.306492536	2.46E-12	postive
METTL17	AC034102.6	0.507566805	4.27E-34	postive
METTL17	AC034231.1	0.307400626	2.10E-12	postive
METTL17	AC034236.2	0.540659669	2.68E-39	postive
METTL17	AC034236.3	0.354305536	3.10E-16	postive
METTL17	AC036108.3	0.375682069	3.31E-18	postive
METTL17	AC036176.1	0.355819644	2.28E-16	postive
METTL17	AC037459.2	0.536847048	1.14E-38	postive
METTL17	AC040162.3	0.610899717	1.78E-52	postive
METTL17	AC040169.1	0.339308016	6.15E-15	postive
METTL17	AC040970.1	0.300587709	6.70E-12	postive
METTL17	AC040977.1	0.375447188	3.49E-18	postive
METTL17	AC044802.1	0.313343612	7.48E-13	postive
METTL17	AC044849.1	0.325568612	8.26E-14	postive
METTL17	AC046134.2	0.401857856	7.91E-21	postive
METTL17	AC046143.2	0.335037471	1.40E-14	postive
METTL17	AC048337.1	0.354368642	3.07E-16	postive
METTL17	AC048341.1	0.347340895	1.27E-15	postive
METTL17	AC048341.2	0.408172144	1.70E-21	postive
METTL17	AC048382.1	0.358458699	1.32E-16	postive
METTL17	AC048382.2	0.39160325	8.98E-20	postive
METTL17	AC051619.6	0.302419299	4.92E-12	postive
METTL17	AC053503.1	0.338418884	7.31E-15	postive
METTL17	AC053503.4	0.373125067	5.81E-18	postive
METTL17	AC055822.1	0.393470728	5.81E-20	postive
METTL17	AC055855.2	0.565439518	1.37E-43	postive
METTL17	AC055855.3	0.341335787	4.15E-15	postive
METTL17	AC060766.7	0.344924446	2.05E-15	postive
METTL17	AC060780.1	0.548983998	1.06E-40	postive
METTL17	AC062037.3	0.37156704	8.15E-18	postive
METTL17	AC063943.2	0.34662625	1.46E-15	postive
METTL17	AC064807.1	0.460683018	1.23E-27	postive
METTL17	AC064807.4	0.30140258	5.84E-12	postive
METTL17	AC064836.3	0.388583497	1.81E-19	postive

METTL17	AC066613.1	0.424326697	2.85E-23	postive
METTL17	AC067750.1	0.536098294	1.51E-38	postive
METTL17	AC067838.1	0.317685524	3.46E-13	postive
METTL17	AC067852.2	0.497541254	1.25E-32	postive
METTL17	AC067852.3	0.390963298	1.04E-19	postive
METTL17	AC067930.2	0.408151034	1.71E-21	postive
METTL17	AC067930.4	0.431146576	4.73E-24	postive
METTL17	AC067942.1	0.3486001	9.86E-16	postive
METTL17	AC068025.1	0.300041407	7.34E-12	postive
METTL17	AC068039.4	0.311110386	1.11E-12	postive
METTL17	AC068196.1	0.315150315	5.43E-13	postive
METTL17	AC068338.2	0.450796463	2.14E-26	postive
METTL17	AC068338.3	0.465851742	2.67E-28	postive
METTL17	AC068473.5	0.476637281	1.01E-29	postive
METTL17	AC068580.1	0.362160312	6.09E-17	postive
METTL17	AC068580.3	0.399946343	1.25E-20	postive
METTL17	AC068620.1	0.469423278	9.13E-29	postive
METTL17	AC068620.2	0.541497695	1.95E-39	postive
METTL17	AC068724.2	0.43037867	5.80E-24	postive
METTL17	AC068768.1	0.386314105	3.04E-19	postive
METTL17	AC068831.2	0.613833666	4.24E-53	postive
METTL17	AC068831.3	0.392360525	7.53E-20	postive
METTL17	AC068870.1	0.323980392	1.11E-13	postive
METTL17	AC068870.2	0.363825557	4.29E-17	postive
METTL17	AC068888.1	0.42306356	3.95E-23	postive
METTL17	AC069148.1	0.342575843	3.25E-15	postive
METTL17	AC069185.1	0.349153055	8.82E-16	postive
METTL17	AC069213.1	0.316301276	4.43E-13	postive
METTL17	AC069222.1	0.365049743	3.31E-17	postive
METTL17	AC069224.2	0.41157523	7.31E-22	postive
METTL17	AC069277.1	0.301560964	5.69E-12	postive
METTL17	AC069307.1	0.336216249	1.12E-14	postive
METTL17	AC069544.1	0.400036662	1.23E-20	postive
METTL17	AC072061.1	0.504205247	1.34E-33	postive
METTL17	AC073073.2	0.349841698	7.68E-16	postive
METTL17	AC073111.1	0.408214193	1.68E-21	postive
METTL17	AC073195.2	0.532080215	6.80E-38	postive
METTL17	AC073288.2	0.432124664	3.65E-24	postive
METTL17	AC073321.1	0.329584456	3.92E-14	postive
METTL17	AC073349.1	0.321230462	1.83E-13	postive
METTL17	AC073352.1	0.440786976	3.49E-25	postive
METTL17	AC073487.1	0.385375352	3.77E-19	postive
METTL17	AC073508.3	0.372527416	6.62E-18	postive
METTL17	AC073517.1	0.37711655	2.41E-18	postive
METTL17	AC073529.1	0.351832417	5.14E-16	postive
METTL17	AC073569.2	0.31245835	8.74E-13	postive
METTL17	AC073573.1	0.436834991	1.03E-24	postive
METTL17	AC073575.4	0.593812024	5.72E-49	postive
METTL17	AC073611.1	0.525311818	8.17E-37	postive
METTL17	AC073655.2	0.435016993	1.68E-24	postive

METTL17	AC073842.2	0.607104381	1.12E-51	postive
METTL17	AC073869.1	0.619696287	2.30E-54	postive
METTL17	AC073896.2	0.523091959	1.83E-36	postive
METTL17	AC073896.3	0.391211364	9.84E-20	postive
METTL17	AC073896.4	0.467343549	1.71E-28	postive
METTL17	AC073896.5	0.351475685	5.52E-16	postive
METTL17	AC073957.3	0.409700322	1.16E-21	postive
METTL17	AC074011.1	0.306380628	2.51E-12	postive
METTL17	AC074029.3	0.468510763	1.20E-28	postive
METTL17	AC074044.1	0.370923173	9.38E-18	postive
METTL17	AC074117.1	0.504356889	1.27E-33	postive
METTL17	AC074212.1	0.471831723	4.40E-29	postive
METTL17	AC078785.1	0.309148937	1.56E-12	postive
METTL17	AC078846.1	0.473831109	2.39E-29	postive
METTL17	AC078909.2	0.365384705	3.08E-17	postive
METTL17	AC079145.1	0.310576996	1.21E-12	postive
METTL17	AC079148.1	0.315765343	4.87E-13	postive
METTL17	AC079174.1	0.358426352	1.33E-16	postive
METTL17	AC079174.2	0.526026601	6.30E-37	postive
METTL17	AC079305.2	0.334570013	1.53E-14	postive
METTL17	AC079322.1	0.465817262	2.69E-28	postive
METTL17	AC079336.5	0.41121571	7.99E-22	postive
METTL17	AC079414.3	0.508197234	3.44E-34	postive
METTL17	AC079447.1	0.378228007	1.89E-18	postive
METTL17	AC079684.2	0.321948453	1.60E-13	postive
METTL17	AC079807.1	0.523706154	1.46E-36	postive
METTL17	AC079848.1	0.335683234	1.24E-14	postive
METTL17	AC079906.1	0.389228016	1.56E-19	postive
METTL17	AC079907.1	0.47423692	2.11E-29	postive
METTL17	AC079922.2	0.559209526	1.78E-42	postive
METTL17	AC080013.5	0.399451051	1.41E-20	postive
METTL17	AC080038.3	0.332592726	2.23E-14	postive
METTL17	AC080112.2	0.311440678	1.04E-12	postive
METTL17	AC080129.2	0.538356584	6.45E-39	postive
METTL17	AC083798.2	0.352988016	4.06E-16	postive
METTL17	AC083799.1	0.400435705	1.11E-20	postive
METTL17	AC083862.2	0.367111656	2.13E-17	postive
METTL17	AC083880.1	0.462049604	8.23E-28	postive
METTL17	AC083964.1	0.313370875	7.44E-13	postive
METTL17	AC084018.1	0.578539301	5.24E-46	postive
METTL17	AC084018.2	0.511047687	1.29E-34	postive
METTL17	AC084117.1	0.311903432	9.63E-13	postive
METTL17	AC084125.2	0.561590771	6.73E-43	postive
METTL17	AC084125.4	0.500225879	5.12E-33	postive
METTL17	AC084346.1	0.318420511	3.03E-13	postive
METTL17	AC084768.1	0.325012846	9.15E-14	postive
METTL17	AC084809.1	0.330874041	3.08E-14	postive
METTL17	AC084824.4	0.352516685	4.47E-16	postive
METTL17	AC084824.5	0.332204982	2.39E-14	postive
METTL17	AC084824.6	0.491081695	1.04E-31	postive

METTL17	AC084864.1	0.327818123	5.45E-14	postive
METTL17	AC084876.1	0.314189895	6.44E-13	postive
METTL17	AC087164.1	0.393708626	5.49E-20	postive
METTL17	AC087289.1	0.406205197	2.75E-21	postive
METTL17	AC087289.2	0.637082241	2.78E-58	postive
METTL17	AC087289.5	0.394731254	4.32E-20	postive
METTL17	AC087301.1	0.47018779	7.24E-29	postive
METTL17	AC087341.1	0.317394262	3.64E-13	postive
METTL17	AC087392.3	0.455772201	5.14E-27	postive
METTL17	AC087392.4	0.427533126	1.23E-23	postive
METTL17	AC087392.5	0.351287381	5.74E-16	postive
METTL17	AC087500.1	0.506415154	6.33E-34	postive
METTL17	AC087500.2	0.375628815	3.35E-18	postive
METTL17	AC087501.4	0.434597798	1.88E-24	postive
METTL17	AC087521.3	0.345428206	1.85E-15	postive
METTL17	AC087741.1	0.537893345	7.69E-39	postive
METTL17	AC087741.2	0.48228898	1.73E-30	postive
METTL17	AC087752.3	0.397269259	2.37E-20	postive
METTL17	AC087752.4	0.356300918	2.06E-16	postive
METTL17	AC090004.1	0.411974901	6.62E-22	postive
METTL17	AC090114.2	0.466062685	2.50E-28	postive
METTL17	AC090198.1	0.397877855	2.05E-20	postive
METTL17	AC090246.1	0.333193958	1.99E-14	postive
METTL17	AC090260.2	0.343578318	2.67E-15	postive
METTL17	AC090360.1	0.322279859	1.51E-13	postive
METTL17	AC090425.2	0.385686104	3.51E-19	postive
METTL17	AC090425.3	0.361793462	6.58E-17	postive
METTL17	AC090510.2	0.454108188	8.29E-27	postive
METTL17	AC090515.3	0.349811643	7.73E-16	postive
METTL17	AC090527.4	0.443346357	1.73E-25	postive
METTL17	AC090587.1	0.380315948	1.18E-18	postive
METTL17	AC090587.2	0.641280804	2.88E-59	postive
METTL17	AC090589.3	0.590661843	2.41E-48	postive
METTL17	AC090617.5	0.450068855	2.62E-26	postive
METTL17	AC090825.1	0.306187248	2.59E-12	postive
METTL17	AC090912.1	0.341012965	4.42E-15	postive
METTL17	AC090948.3	0.317841873	3.36E-13	postive
METTL17	AC090970.2	0.383578422	5.67E-19	postive
METTL17	AC091057.1	0.418559988	1.26E-22	postive
METTL17	AC091057.3	0.353269733	3.84E-16	postive
METTL17	AC091153.3	0.358339253	1.35E-16	postive
METTL17	AC091153.4	0.527814787	3.28E-37	postive
METTL17	AC091180.5	0.339156434	6.34E-15	postive
METTL17	AC091180.6	0.340905197	4.51E-15	postive
METTL17	AC091185.1	0.381957849	8.18E-19	postive
METTL17	AC091271.1	0.401000725	9.72E-21	postive
METTL17	AC091390.5	0.381576269	8.92E-19	postive
METTL17	AC091544.5	0.332356069	2.33E-14	postive
METTL17	AC091729.3	0.504919409	1.05E-33	postive
METTL17	AC091887.1	0.347816696	1.15E-15	postive

METTL17	AC092070.2	0.345332717	1.89E-15	postive
METTL17	AC092111.1	0.30149899	5.75E-12	postive
METTL17	AC092115.4	0.352953163	4.09E-16	postive
METTL17	AC092117.1	0.376583376	2.72E-18	postive
METTL17	AC092118.2	0.538152355	6.97E-39	postive
METTL17	AC092119.2	0.56982741	2.19E-44	postive
METTL17	AC092123.1	0.461507474	9.66E-28	postive
METTL17	AC092134.1	0.335006772	1.41E-14	postive
METTL17	AC092164.1	0.382706587	6.91E-19	postive
METTL17	AC092171.2	0.321792957	1.65E-13	postive
METTL17	AC092171.4	0.486344189	4.77E-31	postive
METTL17	AC092171.5	0.43131526	4.53E-24	postive
METTL17	AC092295.1	0.302475311	4.88E-12	postive
METTL17	AC092295.2	0.373322801	5.56E-18	postive
METTL17	AC092301.1	0.446710988	6.76E-26	postive
METTL17	AC092368.3	0.433194095	2.74E-24	postive
METTL17	AC092375.2	0.359916641	9.74E-17	postive
METTL17	AC092535.5	0.304792323	3.29E-12	postive
METTL17	AC092574.1	0.322247127	1.52E-13	postive
METTL17	AC092611.1	0.501021948	3.92E-33	postive
METTL17	AC092614.1	0.346161553	1.60E-15	postive
METTL17	AC092634.3	0.350922673	6.18E-16	postive
METTL17	AC092683.1	0.31228806	9.00E-13	postive
METTL17	AC092718.2	0.356105183	2.15E-16	postive
METTL17	AC092718.4	0.324570337	9.93E-14	postive
METTL17	AC092718.5	0.312374184	8.87E-13	postive
METTL17	AC092720.1	0.397210181	2.40E-20	postive
METTL17	AC092802.3	0.301376995	5.87E-12	postive
METTL17	AC092803.1	0.431445661	4.37E-24	postive
METTL17	AC092809.5	0.526398511	5.50E-37	postive
METTL17	AC092903.2	0.347419307	1.25E-15	postive
METTL17	AC092910.3	0.546767363	2.54E-40	postive
METTL17	AC092944.1	0.369318928	1.33E-17	postive
METTL17	AC092953.2	0.325045496	9.10E-14	postive
METTL17	AC092969.1	0.373142582	5.78E-18	postive
METTL17	AC093157.1	0.529917701	1.51E-37	postive
METTL17	AC093227.1	0.388236499	1.96E-19	postive
METTL17	AC093249.2	0.523909263	1.36E-36	postive
METTL17	AC093249.6	0.482051432	1.86E-30	postive
METTL17	AC093382.1	0.327505636	5.77E-14	postive
METTL17	AC093458.1	0.301124502	6.12E-12	postive
METTL17	AC093462.1	0.488195562	2.64E-31	postive
METTL17	AC093484.4	0.473150969	2.94E-29	postive
METTL17	AC093495.1	0.419821342	9.10E-23	postive
METTL17	AC093525.4	0.322110706	1.56E-13	postive
METTL17	AC093582.1	0.393978202	5.16E-20	postive
METTL17	AC093620.1	0.471773143	4.48E-29	postive
METTL17	AC093627.5	0.446739572	6.70E-26	postive
METTL17	AC093627.7	0.308206058	1.83E-12	postive
METTL17	AC093673.1	0.303663075	3.99E-12	postive

METTL17	AC093726.1	0.422104292	5.06E-23	postive
METTL17	AC093726.2	0.457480261	3.13E-27	postive
METTL17	AC093734.1	0.370091937	1.12E-17	postive
METTL17	AC093752.1	0.539483777	4.20E-39	postive
METTL17	AC093752.3	0.550664371	5.49E-41	postive
METTL17	AC093772.1	0.321633829	1.70E-13	postive
METTL17	AC093788.1	0.405504354	3.27E-21	postive
METTL17	AC093797.1	0.31360615	7.14E-13	postive
METTL17	AC093835.1	0.341910536	3.71E-15	postive
METTL17	AC095057.3	0.411920649	6.71E-22	postive
METTL17	AC096536.2	0.307221873	2.17E-12	postive
METTL17	AC096540.1	0.428573768	9.36E-24	postive
METTL17	AC096586.2	0.354314966	3.10E-16	postive
METTL17	AC096642.1	0.471080232	5.53E-29	postive
METTL17	AC096649.2	0.318137131	3.19E-13	postive
METTL17	AC096677.1	0.438365015	6.78E-25	postive
METTL17	AC096733.3	0.354850648	2.78E-16	postive
METTL17	AC096772.6	0.324152123	1.07E-13	postive
METTL17	AC096992.2	0.38439914	4.71E-19	postive
METTL17	AC097358.2	0.358394105	1.34E-16	postive
METTL17	AC097359.2	0.488599329	2.32E-31	postive
METTL17	AC097376.3	0.375235328	3.66E-18	postive
METTL17	AC097382.2	0.388367627	1.90E-19	postive
METTL17	AC097468.3	0.398673484	1.70E-20	postive
METTL17	AC097639.1	0.338459003	7.25E-15	postive
METTL17	AC097641.2	0.452896163	1.17E-26	postive
METTL17	AC098484.1	0.3400588	5.32E-15	postive
METTL17	AC098484.2	0.544743984	5.57E-40	postive
METTL17	AC098487.1	0.370950051	9.32E-18	postive
METTL17	AC098591.2	0.31408026	6.57E-13	postive
METTL17	AC098614.3	0.303028915	4.44E-12	postive
METTL17	AC098820.2	0.410040671	1.07E-21	postive
METTL17	AC098820.4	0.338711196	6.91E-15	postive
METTL17	AC098869.2	0.327551719	5.72E-14	postive
METTL17	AC098934.1	0.307883457	1.94E-12	postive
METTL17	AC099343.4	0.538837053	5.37E-39	postive
METTL17	AC099522.2	0.414476784	3.53E-22	postive
METTL17	AC099568.2	0.403829376	4.91E-21	postive
METTL17	AC099681.2	0.31568822	4.94E-13	postive
METTL17	AC099778.1	0.606692251	1.36E-51	postive
METTL17	AC099791.2	0.505624962	8.29E-34	postive
METTL17	AC099811.3	0.322302296	1.50E-13	postive
METTL17	AC099850.2	0.379579787	1.39E-18	postive
METTL17	AC100778.2	0.527026837	4.38E-37	postive
METTL17	AC100778.3	0.482836764	1.45E-30	postive
METTL17	AC100786.1	0.440205522	4.10E-25	postive
METTL17	AC100793.2	0.323782319	1.15E-13	postive
METTL17	AC100793.3	0.368515323	1.58E-17	postive
METTL17	AC100797.1	0.304067237	3.72E-12	postive
METTL17	AC100803.4	0.328654743	4.66E-14	postive

METTL17	AC100810.1	0.407617327	1.95E-21	postive
METTL17	AC100812.1	0.32123771	1.82E-13	postive
METTL17	AC100861.1	0.38386564	5.32E-19	postive
METTL17	AC102953.1	0.416964665	1.89E-22	postive
METTL17	AC103691.1	0.383958079	5.21E-19	postive
METTL17	AC103706.1	0.419530772	9.80E-23	postive
METTL17	AC103760.1	0.34310894	2.93E-15	postive
METTL17	AC103974.1	0.388531053	1.83E-19	postive
METTL17	AC104109.2	0.346426048	1.52E-15	postive
METTL17	AC104109.4	0.329950011	3.66E-14	postive
METTL17	AC104117.5	0.333569678	1.85E-14	postive
METTL17	AC104118.1	0.313312811	7.52E-13	postive
METTL17	AC104135.1	0.328068986	5.20E-14	postive
METTL17	AC104187.1	0.391954592	8.28E-20	postive
METTL17	AC104257.1	0.322044952	1.58E-13	postive
METTL17	AC104463.2	0.412288481	6.12E-22	postive
METTL17	AC104521.1	0.351306072	5.72E-16	postive
METTL17	AC104532.2	0.477911097	6.79E-30	postive
METTL17	AC104561.1	0.313485647	7.29E-13	postive
METTL17	AC104561.3	0.305875382	2.74E-12	postive
METTL17	AC104564.3	0.584230195	4.31E-47	postive
METTL17	AC104596.1	0.305791008	2.77E-12	postive
METTL17	AC104653.2	0.317833742	3.37E-13	postive
METTL17	AC104667.2	0.321723416	1.67E-13	postive
METTL17	AC104758.1	0.330796757	3.12E-14	postive
METTL17	AC104785.1	0.308028988	1.89E-12	postive
METTL17	AC104794.3	0.386974374	2.62E-19	postive
METTL17	AC104825.1	0.376769704	2.61E-18	postive
METTL17	AC104971.1	0.304560787	3.42E-12	postive
METTL17	AC105020.1	0.344514158	2.22E-15	postive
METTL17	AC105020.5	0.372149558	7.18E-18	postive
METTL17	AC105020.6	0.423835236	3.23E-23	postive
METTL17	AC105036.3	0.417963138	1.46E-22	postive
METTL17	AC105137.2	0.543117911	1.04E-39	postive
METTL17	AC105206.2	0.375562132	3.40E-18	postive
METTL17	AC105219.1	0.496029781	2.06E-32	postive
METTL17	AC105285.1	0.416979037	1.88E-22	postive
METTL17	AC105345.1	0.496487096	1.77E-32	postive
METTL17	AC105935.1	0.428607664	9.27E-24	postive
METTL17	AC105942.1	0.306524241	2.45E-12	postive
METTL17	AC106028.3	0.350154835	7.21E-16	postive
METTL17	AC106037.2	0.319595954	2.45E-13	postive
METTL17	AC106052.1	0.407809446	1.86E-21	postive
METTL17	AC106738.1	0.301116144	6.13E-12	postive
METTL17	AC106772.1	0.36675406	2.30E-17	postive
METTL17	AC106779.1	0.523091843	1.83E-36	postive
METTL17	AC106782.5	0.463321502	5.66E-28	postive
METTL17	AC106795.1	0.492146006	7.35E-32	postive
METTL17	AC106795.2	0.320320987	2.15E-13	postive
METTL17	AC106820.2	0.312663428	8.43E-13	postive

METTL17	AC106820.3	0.39668908	2.72E-20	postive
METTL17	AC106820.4	0.317666184	3.47E-13	postive
METTL17	AC106820.5	0.408820006	1.45E-21	postive
METTL17	AC106886.2	0.384331581	4.78E-19	postive
METTL17	AC106886.3	0.389347276	1.52E-19	postive
METTL17	AC107068.1	0.346645253	1.46E-15	postive
METTL17	AC107081.2	0.533874697	3.49E-38	postive
METTL17	AC107214.1	0.455041811	6.34E-27	postive
METTL17	AC107214.2	0.334703373	1.49E-14	postive
METTL17	AC107294.1	0.307412274	2.10E-12	postive
METTL17	AC107294.2	0.304951477	3.20E-12	postive
METTL17	AC107375.1	0.606209007	1.72E-51	postive
METTL17	AC107464.3	0.454700512	7.00E-27	postive
METTL17	AC107884.1	0.346516051	1.49E-15	postive
METTL17	AC107884.2	0.372601431	6.51E-18	postive
METTL17	AC108010.1	0.424031663	3.07E-23	postive
METTL17	AC108047.1	0.320114634	2.24E-13	postive
METTL17	AC108134.1	0.46288367	6.44E-28	postive
METTL17	AC108134.2	0.338755752	6.85E-15	postive
METTL17	AC108134.3	0.331830952	2.57E-14	postive
METTL17	AC108471.3	0.340810236	4.59E-15	postive
METTL17	AC108488.1	0.54891852	1.09E-40	postive
METTL17	AC108673.2	0.374242527	4.55E-18	postive
METTL17	AC108673.3	0.41217085	6.30E-22	postive
METTL17	AC108860.2	0.427675767	1.19E-23	postive
METTL17	AC109322.1	0.486323765	4.80E-31	postive
METTL17	AC109347.1	0.541590967	1.88E-39	postive
METTL17	AC109361.1	0.35037233	6.90E-16	postive
METTL17	AC109449.1	0.378606971	1.73E-18	postive
METTL17	AC109460.1	0.602242322	1.13E-50	postive
METTL17	AC109460.2	0.610037355	2.71E-52	postive
METTL17	AC110079.1	0.585368787	2.60E-47	postive
METTL17	AC110285.1	0.404355701	4.32E-21	postive
METTL17	AC110285.2	0.43295788	2.92E-24	postive
METTL17	AC110285.5	0.317069451	3.86E-13	postive
METTL17	AC110769.2	0.32473196	9.64E-14	postive
METTL17	AC112220.2	0.361993339	6.31E-17	postive
METTL17	AC112484.1	0.357798516	1.51E-16	postive
METTL17	AC112491.1	0.377563576	2.19E-18	postive
METTL17	AC112694.1	0.477263839	8.30E-30	postive
METTL17	AC112907.3	0.415885151	2.48E-22	postive
METTL17	AC113139.1	0.373582889	5.25E-18	postive
METTL17	AC113189.4	0.339848656	5.54E-15	postive
METTL17	AC113382.1	0.305162584	3.09E-12	postive
METTL17	AC114271.1	0.479623204	3.98E-30	postive
METTL17	AC114490.1	0.531430681	8.66E-38	postive
METTL17	AC114684.1	0.336267514	1.11E-14	postive
METTL17	AC114730.2	0.499774333	5.95E-33	postive
METTL17	AC114730.3	0.54419675	6.88E-40	postive
METTL17	AC114956.2	0.422430327	4.65E-23	postive

METTL17	AC115618.1	0.445769652	8.79E-26	postive
METTL17	AC115618.2	0.350072164	7.33E-16	postive
METTL17	AC116366.2	0.331363613	2.81E-14	postive
METTL17	AC116407.2	0.447862825	4.89E-26	postive
METTL17	AC116447.1	0.331896275	2.54E-14	postive
METTL17	AC116535.2	0.393024085	6.45E-20	postive
METTL17	AC116552.1	0.308720295	1.68E-12	postive
METTL17	AC116667.1	0.432307571	3.47E-24	postive
METTL17	AC116913.1	0.566266005	9.74E-44	postive
METTL17	AC116914.2	0.5742075	3.40E-45	postive
METTL17	AC117490.2	0.324179125	1.07E-13	postive
METTL17	AC117503.1	0.38503607	4.07E-19	postive
METTL17	AC118344.1	0.30236662	4.97E-12	postive
METTL17	AC118553.1	0.374114963	4.68E-18	postive
METTL17	AC118755.2	0.398535765	1.75E-20	postive
METTL17	AC118758.3	0.382602616	7.07E-19	postive
METTL17	AC120053.1	0.509207546	2.43E-34	postive
METTL17	AC120114.1	0.374425753	4.37E-18	postive
METTL17	AC120498.9	0.448601757	3.97E-26	postive
METTL17	AC121338.2	0.419289014	1.04E-22	postive
METTL17	AC121493.1	0.343396374	2.77E-15	postive
METTL17	AC121761.2	0.342120501	3.56E-15	postive
METTL17	AC122129.1	0.366842695	2.26E-17	postive
METTL17	AC123768.2	0.343177982	2.89E-15	postive
METTL17	AC123768.3	0.353130596	3.95E-16	postive
METTL17	AC124016.2	0.538278825	6.64E-39	postive
METTL17	AC124045.1	0.33557174	1.26E-14	postive
METTL17	AC124248.1	0.321129608	1.86E-13	postive
METTL17	AC124944.2	0.462682836	6.83E-28	postive
METTL17	AC124944.3	0.353955394	3.34E-16	postive
METTL17	AC125257.1	0.422789617	4.24E-23	postive
METTL17	AC125494.1	0.539488431	4.19E-39	postive
METTL17	AC125494.2	0.318096644	3.21E-13	postive
METTL17	AC126118.1	0.495532039	2.43E-32	postive
METTL17	AC126283.1	0.393863851	5.30E-20	postive
METTL17	AC126755.1	0.442843588	1.98E-25	postive
METTL17	AC126773.6	0.324366197	1.03E-13	postive
METTL17	AC127002.2	0.303470399	4.12E-12	postive
METTL17	AC127024.2	0.342120666	3.56E-15	postive
METTL17	AC127024.4	0.413570315	4.44E-22	postive
METTL17	AC127024.5	0.402498213	6.78E-21	postive
METTL17	AC127024.6	0.430539191	5.56E-24	postive
METTL17	AC127496.2	0.387760595	2.19E-19	postive
METTL17	AC127502.1	0.34514876	1.96E-15	postive
METTL17	AC127502.2	0.447969952	4.75E-26	postive
METTL17	AC127537.1	0.354870839	2.77E-16	postive
METTL17	AC128687.2	0.343890247	2.51E-15	postive
METTL17	AC129510.1	0.490041294	1.45E-31	postive
METTL17	AC130324.1	0.360011239	9.55E-17	postive
METTL17	AC130456.3	0.304464531	3.48E-12	postive

METTL17	AC130462.3	0.490522676	1.24E-31	postive
METTL17	AC131009.3	0.466603077	2.13E-28	postive
METTL17	AC131025.1	0.414624895	3.41E-22	postive
METTL17	AC131235.3	0.441667583	2.74E-25	postive
METTL17	AC131235.4	0.378253588	1.87E-18	postive
METTL17	AC132008.2	0.583186953	6.83E-47	postive
METTL17	AC132192.2	0.57381553	4.02E-45	postive
METTL17	AC132872.1	0.465213621	3.23E-28	postive
METTL17	AC132872.2	0.524207034	1.22E-36	postive
METTL17	AC132872.3	0.54173431	1.78E-39	postive
METTL17	AC132938.2	0.319063058	2.70E-13	postive
METTL17	AC132938.3	0.489055618	2.00E-31	postive
METTL17	AC132938.4	0.406344203	2.66E-21	postive
METTL17	AC133528.2	0.60603471	1.87E-51	postive
METTL17	AC133550.3	0.493229621	5.16E-32	postive
METTL17	AC133552.2	0.448094356	4.58E-26	postive
METTL17	AC133552.5	0.361941132	6.38E-17	postive
METTL17	AC133919.2	0.400753904	1.03E-20	postive
METTL17	AC134682.1	0.310513554	1.23E-12	postive
METTL17	AC134775.1	0.323281754	1.26E-13	postive
METTL17	AC134878.2	0.421344554	6.16E-23	postive
METTL17	AC135048.1	0.321152315	1.85E-13	postive
METTL17	AC135050.1	0.514633732	3.69E-35	postive
METTL17	AC135050.3	0.452823183	1.20E-26	postive
METTL17	AC135050.4	0.454760058	6.88E-27	postive
METTL17	AC135050.5	0.556058855	6.38E-42	postive
METTL17	AC135050.6	0.603628078	5.88E-51	postive
METTL17	AC135178.4	0.516773462	1.74E-35	postive
METTL17	AC135178.6	0.437482929	8.62E-25	postive
METTL17	AC135507.1	0.351546684	5.44E-16	postive
METTL17	AC135983.3	0.383609668	5.63E-19	postive
METTL17	AC136469.1	0.301429101	5.82E-12	postive
METTL17	AC136475.2	0.488330551	2.52E-31	postive
METTL17	AC136475.3	0.382256694	7.65E-19	postive
METTL17	AC136604.2	0.476640481	1.01E-29	postive
METTL17	AC136604.3	0.41010316	1.05E-21	postive
METTL17	AC136777.1	0.3559909	2.20E-16	postive
METTL17	AC137630.1	0.467406364	1.67E-28	postive
METTL17	AC137630.2	0.392497112	7.29E-20	postive
METTL17	AC137834.2	0.317006523	3.91E-13	postive
METTL17	AC137894.1	0.333454085	1.89E-14	postive
METTL17	AC137932.3	0.419235771	1.06E-22	postive
METTL17	AC138028.2	0.396087348	3.14E-20	postive
METTL17	AC138028.4	0.531977246	7.07E-38	postive
METTL17	AC138028.6	0.402532314	6.72E-21	postive
METTL17	AC138035.1	0.51909643	7.63E-36	postive
METTL17	AC138150.2	0.429948561	6.51E-24	postive
METTL17	AC138207.4	0.406640168	2.47E-21	postive
METTL17	AC138207.8	0.366541158	2.41E-17	postive
METTL17	AC138230.1	0.576586479	1.22E-45	postive

METTL17	AC138393.1	0.363401045	4.69E-17	postive
METTL17	AC138696.2	0.385545025	3.63E-19	postive
METTL17	AC138811.1	0.304471915	3.48E-12	postive
METTL17	AC138904.1	0.331103367	2.95E-14	postive
METTL17	AC138932.1	0.498519834	9.04E-33	postive
METTL17	AC138956.1	0.483902208	1.04E-30	postive
METTL17	AC138956.2	0.51646816	1.94E-35	postive
METTL17	AC139100.2	0.391158225	9.96E-20	postive
METTL17	AC139149.1	0.363820817	4.29E-17	postive
METTL17	AC139256.2	0.460741598	1.21E-27	postive
METTL17	AC139256.3	0.519470458	6.68E-36	postive
METTL17	AC139530.1	0.578203831	6.06E-46	postive
METTL17	AC139768.1	0.528457262	2.59E-37	postive
METTL17	AC139795.2	0.440584561	3.69E-25	postive
METTL17	AC139887.1	0.476228818	1.14E-29	postive
METTL17	AC139887.2	0.506953514	5.27E-34	postive
METTL17	AC139887.4	0.362030367	6.26E-17	postive
METTL17	AC141002.1	0.374700641	4.11E-18	postive
METTL17	AC141586.1	0.43069352	5.34E-24	postive
METTL17	AC142472.1	0.510887196	1.36E-34	postive
METTL17	AC144652.1	0.365133969	3.25E-17	postive
METTL17	AC144836.1	0.30138156	5.86E-12	postive
METTL17	AC145098.1	0.318161503	3.18E-13	postive
METTL17	AC145207.5	0.306543257	2.44E-12	postive
METTL17	AC145285.2	0.406452311	2.59E-21	postive
METTL17	AC145285.3	0.360156699	9.27E-17	postive
METTL17	AC145285.6	0.536479821	1.31E-38	postive
METTL17	AC145423.2	0.396511746	2.84E-20	postive
METTL17	AC145423.3	0.541795844	1.74E-39	postive
METTL17	AC147651.1	0.311313927	1.07E-12	postive
METTL17	AC211433.2	0.330274554	3.44E-14	postive
METTL17	AC211476.4	0.34186273	3.74E-15	postive
METTL17	AC231533.2	0.355277592	2.54E-16	postive
METTL17	AC231981.1	0.383570898	5.68E-19	postive
METTL17	AC232271.1	0.597098905	1.26E-49	postive
METTL17	AC233280.1	0.410697957	9.09E-22	postive
METTL17	AC233728.1	0.625407765	1.26E-55	postive
METTL17	AC233992.3	0.329352823	4.09E-14	postive
METTL17	AC234582.1	0.419397934	1.01E-22	postive
METTL17	AC241952.2	0.36957268	1.26E-17	postive
METTL17	AC242376.2	0.38963944	1.42E-19	postive
METTL17	AC242426.2	0.369416978	1.30E-17	postive
METTL17	AC242842.1	0.354912793	2.74E-16	postive
METTL17	AC243654.1	0.315135619	5.45E-13	postive
METTL17	AC243654.3	0.341818016	3.77E-15	postive
METTL17	AC243919.2	0.49513282	2.77E-32	postive
METTL17	AC243965.2	0.407335205	2.09E-21	postive
METTL17	AC244034.2	0.313905905	6.77E-13	postive
METTL17	AC244090.1	0.325884711	7.79E-14	postive
METTL17	AC244090.3	0.322033004	1.58E-13	postive

METTL17	AC244197.2	0.449617144	2.98E-26	postive
METTL17	AC244669.1	0.397031715	2.51E-20	postive
METTL17	AC245014.1	0.352461789	4.52E-16	postive
METTL17	AC245052.4	0.594106354	5.00E-49	postive
METTL17	AC245060.2	0.495886266	2.16E-32	postive
METTL17	AC245060.5	0.451733713	1.64E-26	postive
METTL17	AC245060.6	0.520483951	4.65E-36	postive
METTL17	AC245100.1	0.346146973	1.61E-15	postive
METTL17	AC245140.1	0.379311805	1.48E-18	postive
METTL17	AC245140.2	0.646144983	1.99E-60	postive
METTL17	AC245187.1	0.301839379	5.43E-12	postive
METTL17	AC245452.1	0.519432092	6.77E-36	postive
METTL17	AC245519.1	0.317595895	3.52E-13	postive
METTL17	AC245884.8	0.427131611	1.37E-23	postive
METTL17	AC253536.6	0.559242031	1.76E-42	postive
METTL17	AC254562.2	0.362328021	5.88E-17	postive
METTL17	AC254562.3	0.450439993	2.36E-26	postive
METTL17	ACTG1P17	0.390238852	1.23E-19	postive
METTL17	ACTN1-AS1	0.38339693	5.91E-19	postive
METTL17	ACVR2B-AS1	0.497104101	1.44E-32	postive
METTL17	AD000671.3	0.402596928	6.62E-21	postive
METTL17	AD001527.1	0.458166833	2.57E-27	postive
METTL17	ADCY10P1	0.389296587	1.53E-19	postive
METTL17	ADIRF-AS1	0.373508934	5.34E-18	postive
METTL17	ADNP-AS1	0.496455148	1.79E-32	postive
METTL17	AF111169.3	0.429397789	7.53E-24	postive
METTL17	AF117829.1	0.352985118	4.07E-16	postive
METTL17	AF123462.1	0.304673851	3.36E-12	postive
METTL17	AF129075.2	0.327096915	6.23E-14	postive
METTL17	AF129075.3	0.382868954	6.66E-19	postive
METTL17	AF131216.4	0.321295896	1.80E-13	postive
METTL17	AF186192.1	0.316780808	4.07E-13	postive
METTL17	AF213884.3	0.346586205	1.47E-15	postive
METTL17	AFDN-DT	0.454483637	7.45E-27	postive
METTL17	AFG3L1P	0.669904557	2.08E-66	postive
METTL17	AGAP12P	0.341339092	4.14E-15	postive
METTL17	AGBL5-IT1	0.386730912	2.77E-19	postive
METTL17	AHSA2P	0.63021683	1.05E-56	postive
METTL17	AJ239328.1	0.303966408	3.79E-12	postive
METTL17	AL008582.1	0.502744051	2.20E-33	postive
METTL17	AL008635.1	0.331213083	2.89E-14	postive
METTL17	AL008718.2	0.411499569	7.45E-22	postive
METTL17	AL008721.1	0.368191243	1.69E-17	postive
METTL17	AL008721.2	0.313901874	6.78E-13	postive
METTL17	AL008726.1	0.325483993	8.39E-14	postive
METTL17	AL008727.1	0.323650914	1.18E-13	postive
METTL17	AL008729.1	0.444128026	1.39E-25	postive
METTL17	AL008729.2	0.328667278	4.65E-14	postive
METTL17	AL009179.1	0.533537638	3.95E-38	postive
METTL17	AL020996.1	0.546848219	2.46E-40	postive

METTL17	AL021368.2	0.417367595	1.70E-22	postive
METTL17	AL021392.1	0.42741209	1.27E-23	postive
METTL17	AL021707.1	0.411519201	7.41E-22	postive
METTL17	AL021707.2	0.371996961	7.43E-18	postive
METTL17	AL021707.3	0.504362851	1.27E-33	postive
METTL17	AL021707.4	0.407275663	2.12E-21	postive
METTL17	AL021707.5	0.363164034	4.93E-17	postive
METTL17	AL021707.6	0.496780807	1.61E-32	postive
METTL17	AL021707.7	0.410160287	1.04E-21	postive
METTL17	AL021707.8	0.464036529	4.58E-28	postive
METTL17	AL021878.2	0.561295549	7.60E-43	postive
METTL17	AL021937.1	0.356668822	1.91E-16	postive
METTL17	AL022069.1	0.461313611	1.02E-27	postive
METTL17	AL022157.1	0.47295665	3.12E-29	postive
METTL17	AL022238.3	0.460801215	1.19E-27	postive
METTL17	AL022311.1	0.504382549	1.26E-33	postive
METTL17	AL022322.1	0.431247723	4.61E-24	postive
METTL17	AL022322.2	0.402241412	7.21E-21	postive
METTL17	AL022324.3	0.384861427	4.24E-19	postive
METTL17	AL022328.1	0.551853952	3.42E-41	postive
METTL17	AL022328.2	0.625108401	1.47E-55	postive
METTL17	AL022328.3	0.563000961	3.77E-43	postive
METTL17	AL022328.4	0.413733358	4.26E-22	postive
METTL17	AL022341.1	0.416713055	2.01E-22	postive
METTL17	AL022341.2	0.466392279	2.27E-28	postive
METTL17	AL022476.1	0.403448759	5.39E-21	postive
METTL17	AL023284.4	0.361298319	7.30E-17	postive
METTL17	AL023653.1	0.307528093	2.06E-12	postive
METTL17	AL023803.1	0.303968728	3.79E-12	postive
METTL17	AL024498.1	0.477339787	8.10E-30	postive
METTL17	AL024507.3	0.433480876	2.54E-24	postive
METTL17	AL031055.1	0.355172104	2.60E-16	postive
METTL17	AL031118.1	0.347644363	1.19E-15	postive
METTL17	AL031123.2	0.344618938	2.18E-15	postive
METTL17	AL031186.1	0.565346336	1.43E-43	postive
METTL17	AL031282.1	0.452146414	1.45E-26	postive
METTL17	AL031282.2	0.51482137	3.46E-35	postive
METTL17	AL031289.1	0.449295641	3.27E-26	postive
METTL17	AL031320.2	0.310772936	1.17E-12	postive
METTL17	AL031432.3	0.354826264	2.79E-16	postive
METTL17	AL031600.1	0.567175625	6.66E-44	postive
METTL17	AL031670.1	0.476541606	1.04E-29	postive
METTL17	AL031705.1	0.497284353	1.36E-32	postive
METTL17	AL031709.1	0.585450615	2.50E-47	postive
METTL17	AL031714.1	0.593660599	6.14E-49	postive
METTL17	AL031717.1	0.439369268	5.15E-25	postive
METTL17	AL031722.1	0.336693773	1.02E-14	postive
METTL17	AL031775.1	0.362502123	5.67E-17	postive
METTL17	AL031775.2	0.512905674	6.75E-35	postive
METTL17	AL031846.2	0.465575295	2.90E-28	postive

METTL17	AL031847.1	0.327161018	6.16E-14	postive
METTL17	AL031963.2	0.439815547	4.56E-25	postive
METTL17	AL031963.3	0.361524828	6.96E-17	postive
METTL17	AL031985.3	0.301111026	6.14E-12	postive
METTL17	AL033384.2	0.469651672	8.52E-29	postive
METTL17	AL033527.3	0.562323161	4.98E-43	postive
METTL17	AL033528.3	0.388385439	1.89E-19	postive
METTL17	AL034550.1	0.430970024	4.96E-24	postive
METTL17	AL035071.1	0.442175093	2.38E-25	postive
METTL17	AL035252.4	0.389150519	1.59E-19	postive
METTL17	AL035461.3	0.454674004	7.05E-27	postive
METTL17	AL035530.2	0.313541188	7.22E-13	postive
METTL17	AL035587.1	0.473542777	2.61E-29	postive
METTL17	AL049552.1	0.358361784	1.35E-16	postive
METTL17	AL049597.2	0.360017756	9.54E-17	postive
METTL17	AL049646.2	0.417845002	1.51E-22	postive
METTL17	AL049779.1	0.332983392	2.07E-14	postive
METTL17	AL049780.1	0.504261827	1.32E-33	postive
METTL17	AL049780.2	0.375001138	3.85E-18	postive
METTL17	AL049794.1	0.320210423	2.20E-13	postive
METTL17	AL049795.1	0.561570921	6.79E-43	postive
METTL17	AL049796.1	0.385061624	4.05E-19	postive
METTL17	AL049840.2	0.459726714	1.63E-27	postive
METTL17	AL049840.4	0.497548524	1.25E-32	postive
METTL17	AL049840.5	0.526371759	5.56E-37	postive
METTL17	AL049840.6	0.596222374	1.89E-49	postive
METTL17	AL050309.1	0.454003837	8.55E-27	postive
METTL17	AL050341.2	0.497340057	1.34E-32	postive
METTL17	AL078587.1	0.414978378	3.12E-22	postive
METTL17	AL078590.2	0.304345898	3.55E-12	postive
METTL17	AL078644.2	0.419256872	1.05E-22	postive
METTL17	AL080250.1	0.368094717	1.73E-17	postive
METTL17	AL080276.2	0.362717597	5.42E-17	postive
METTL17	AL080312.2	0.315388648	5.21E-13	postive
METTL17	AL096701.3	0.495348696	2.58E-32	postive
METTL17	AL096803.2	0.354610434	2.92E-16	postive
METTL17	AL096828.3	0.336580998	1.04E-14	postive
METTL17	AL096865.1	0.353285249	3.82E-16	postive
METTL17	AL096870.1	0.303382884	4.18E-12	postive
METTL17	AL109659.2	0.384216531	4.91E-19	postive
METTL17	AL109804.1	0.393004665	6.48E-20	postive
METTL17	AL109811.1	0.631378059	5.71E-57	postive
METTL17	AL109811.2	0.540124484	3.29E-39	postive
METTL17	AL109917.1	0.449329628	3.23E-26	postive
METTL17	AL109945.1	0.432328818	3.45E-24	postive
METTL17	AL109955.1	0.412845186	5.32E-22	postive
METTL17	AL110115.2	0.413157408	4.92E-22	postive
METTL17	AL110505.1	0.317354663	3.67E-13	postive
METTL17	AL117209.1	0.628742758	2.26E-56	postive
METTL17	AL117332.1	0.443629237	1.60E-25	postive

METTL17	AL117336.2	0.491953565	7.83E-32	postive
METTL17	AL117350.2	0.369515979	1.27E-17	postive
METTL17	AL117379.1	0.495354792	2.57E-32	postive
METTL17	AL118506.1	0.456708931	3.92E-27	postive
METTL17	AL118511.1	0.414352017	3.65E-22	postive
METTL17	AL118516.1	0.451655591	1.67E-26	postive
METTL17	AL118558.1	0.450031666	2.65E-26	postive
METTL17	AL118558.3	0.622757296	4.89E-55	postive
METTL17	AL118558.4	0.544532283	6.05E-40	postive
METTL17	AL121574.1	0.375518254	3.43E-18	postive
METTL17	AL121583.1	0.471796728	4.45E-29	postive
METTL17	AL121601.1	0.422584868	4.47E-23	postive
METTL17	AL121603.2	0.414423237	3.58E-22	postive
METTL17	AL121655.1	0.320283338	2.17E-13	postive
METTL17	AL121658.1	0.407258445	2.13E-21	postive
METTL17	AL121772.3	0.362997459	5.11E-17	postive
METTL17	AL121829.2	0.302884293	4.55E-12	postive
METTL17	AL121832.1	0.328835478	4.51E-14	postive
METTL17	AL121832.2	0.452947088	1.16E-26	postive
METTL17	AL121832.3	0.462259596	7.74E-28	postive
METTL17	AL121845.4	0.416261423	2.25E-22	postive
METTL17	AL121852.1	0.465554783	2.91E-28	postive
METTL17	AL121890.4	0.416676694	2.03E-22	postive
METTL17	AL121890.5	0.346732055	1.43E-15	postive
METTL17	AL121906.1	0.366069327	2.66E-17	postive
METTL17	AL121906.2	0.369924348	1.16E-17	postive
METTL17	AL121917.1	0.40832828	1.63E-21	postive
METTL17	AL121917.2	0.359985322	9.60E-17	postive
METTL17	AL121944.2	0.497508787	1.26E-32	postive
METTL17	AL121987.2	0.482101255	1.83E-30	postive
METTL17	AL121992.1	0.472519725	3.57E-29	postive
METTL17	AL121992.3	0.338559148	7.11E-15	postive
METTL17	AL122010.1	0.544080838	7.20E-40	postive
METTL17	AL122023.3	0.435139342	1.62E-24	postive
METTL17	AL122035.1	0.355487131	2.44E-16	postive
METTL17	AL122035.2	0.55405051	1.43E-41	postive
METTL17	AL122125.1	0.413574395	4.43E-22	postive
METTL17	AL132639.2	0.411513749	7.42E-22	postive
METTL17	AL132639.3	0.426089769	1.80E-23	postive
METTL17	AL132655.2	0.323591579	1.19E-13	postive
METTL17	AL132712.1	0.55652892	5.28E-42	postive
METTL17	AL132765.2	0.436569519	1.10E-24	postive
METTL17	AL132780.1	0.659876089	8.11E-64	postive
METTL17	AL132780.2	0.476528077	1.04E-29	postive
METTL17	AL132800.1	0.373984644	4.81E-18	postive
METTL17	AL132989.1	0.561497164	6.99E-43	postive
METTL17	AL133153.2	0.354982559	2.70E-16	postive
METTL17	AL133215.1	0.37807917	1.95E-18	postive
METTL17	AL133215.2	0.480137794	3.39E-30	postive
METTL17	AL133215.3	0.36447522	3.74E-17	postive

METTL17	AL133216.2	0.421934471	5.29E-23	postive
METTL17	AL133338.1	0.412914854	5.23E-22	postive
METTL17	AL133367.1	0.540365278	3.00E-39	postive
METTL17	AL133406.2	0.513470332	5.55E-35	postive
METTL17	AL133410.1	0.468944965	1.05E-28	postive
METTL17	AL133520.1	0.383969346	5.19E-19	postive
METTL17	AL133523.1	0.305631417	2.85E-12	postive
METTL17	AL133551.1	0.355130842	2.62E-16	postive
METTL17	AL135744.1	0.443879356	1.49E-25	postive
METTL17	AL135791.1	0.427588785	1.21E-23	postive
METTL17	AL135910.1	0.367152704	2.11E-17	postive
METTL17	AL135925.1	0.429861101	6.66E-24	postive
METTL17	AL135999.1	0.668299519	5.48E-66	postive
METTL17	AL135999.2	0.400879284	1.00E-20	postive
METTL17	AL136038.5	0.315553056	5.06E-13	postive
METTL17	AL136038.7	0.376447019	2.80E-18	postive
METTL17	AL136040.1	0.429758969	6.84E-24	postive
METTL17	AL136084.2	0.477719942	7.20E-30	postive
METTL17	AL136141.1	0.317464567	3.60E-13	postive
METTL17	AL136172.1	0.307058525	2.23E-12	postive
METTL17	AL136295.2	0.696250771	9.97E-74	postive
METTL17	AL136295.6	0.6011004	1.94E-50	postive
METTL17	AL136295.7	0.681092596	2.01E-69	postive
METTL17	AL136304.1	0.500779521	4.25E-33	postive
METTL17	AL136368.1	0.386464641	2.94E-19	postive
METTL17	AL136531.1	0.455979119	4.84E-27	postive
METTL17	AL136985.2	0.311001617	1.13E-12	postive
METTL17	AL137003.1	0.387292044	2.43E-19	postive
METTL17	AL137058.2	0.373022998	5.94E-18	postive
METTL17	AL137060.3	0.341332092	4.15E-15	postive
METTL17	AL137127.1	0.560841004	9.15E-43	postive
METTL17	AL137186.2	0.428458411	9.65E-24	postive
METTL17	AL137244.1	0.368002298	1.76E-17	postive
METTL17	AL137779.2	0.327031701	6.30E-14	postive
METTL17	AL137802.2	0.457997823	2.70E-27	postive
METTL17	AL138724.2	0.356731754	1.89E-16	postive
METTL17	AL138762.2	0.330951473	3.03E-14	postive
METTL17	AL138831.1	0.367647166	1.90E-17	postive
METTL17	AL138831.2	0.391561299	9.07E-20	postive
METTL17	AL138921.1	0.557770255	3.19E-42	postive
METTL17	AL138921.2	0.405549443	3.23E-21	postive
METTL17	AL138960.1	0.347189388	1.31E-15	postive
METTL17	AL138976.2	0.449152934	3.40E-26	postive
METTL17	AL138995.1	0.344052058	2.43E-15	postive
METTL17	AL139011.1	0.510561215	1.52E-34	postive
METTL17	AL139021.2	0.359031662	1.17E-16	postive
METTL17	AL139021.3	0.422043566	5.14E-23	postive
METTL17	AL139039.3	0.368662802	1.53E-17	postive
METTL17	AL139089.1	0.596200136	1.91E-49	postive
METTL17	AL139099.3	0.544562248	5.98E-40	postive

METTL17	AL139123.1	0.514064817	4.50E-35	postive
METTL17	AL139246.5	0.321105262	1.87E-13	postive
METTL17	AL139260.1	0.396834009	2.63E-20	postive
METTL17	AL139286.2	0.401597914	8.42E-21	postive
METTL17	AL139287.1	0.64320533	1.01E-59	postive
METTL17	AL139289.1	0.404380009	4.30E-21	postive
METTL17	AL139349.1	0.546398131	2.93E-40	postive
METTL17	AL139353.2	0.407186309	2.16E-21	postive
METTL17	AL139384.1	0.352176	4.79E-16	postive
METTL17	AL139397.1	0.413497502	4.52E-22	postive
METTL17	AL139423.1	0.335011168	1.41E-14	postive
METTL17	AL157373.2	0.432008157	3.76E-24	postive
METTL17	AL157392.3	0.442508847	2.17E-25	postive
METTL17	AL157392.4	0.336539857	1.05E-14	postive
METTL17	AL157400.4	0.300651473	6.63E-12	postive
METTL17	AL157756.1	0.44233719	2.28E-25	postive
METTL17	AL157813.1	0.319471477	2.51E-13	postive
METTL17	AL157832.1	0.433758145	2.36E-24	postive
METTL17	AL157871.5	0.455524393	5.52E-27	postive
METTL17	AL157871.6	0.344633209	2.17E-15	postive
METTL17	AL157904.1	0.445053961	1.07E-25	postive
METTL17	AL157935.3	0.428481267	9.59E-24	postive
METTL17	AL158063.1	0.414057828	3.93E-22	postive
METTL17	AL158151.2	0.310276289	1.28E-12	postive
METTL17	AL158151.4	0.351313033	5.71E-16	postive
METTL17	AL158196.1	0.372186113	7.13E-18	postive
METTL17	AL158212.2	0.506012282	7.26E-34	postive
METTL17	AL158212.3	0.391679428	8.83E-20	postive
METTL17	AL158834.2	0.331560112	2.70E-14	postive
METTL17	AL158835.2	0.388448841	1.87E-19	postive
METTL17	AL159169.2	0.305467872	2.93E-12	postive
METTL17	AL160006.1	0.475386516	1.48E-29	postive
METTL17	AL160291.1	0.359761214	1.01E-16	postive
METTL17	AL160313.2	0.388755757	1.74E-19	postive
METTL17	AL160314.2	0.363015265	5.09E-17	postive
METTL17	AL161452.1	0.64435851	5.35E-60	postive
METTL17	AL161668.3	0.780778429	8.75E-104	postive
METTL17	AL161669.3	0.304245018	3.61E-12	postive
METTL17	AL161725.2	0.405977846	2.91E-21	postive
METTL17	AL161729.1	0.437469472	8.65E-25	postive
METTL17	AL161729.3	0.386228524	3.10E-19	postive
METTL17	AL161729.4	0.465419363	3.03E-28	postive
METTL17	AL161756.1	0.431849233	3.93E-24	postive
METTL17	AL162171.1	0.322680204	1.40E-13	postive
METTL17	AL162231.2	0.345200961	1.94E-15	postive
METTL17	AL162258.2	0.475551782	1.41E-29	postive
METTL17	AL162274.1	0.31031875	1.27E-12	postive
METTL17	AL162274.2	0.496462733	1.79E-32	postive
METTL17	AL162377.1	0.51844469	9.62E-36	postive
METTL17	AL162426.1	0.332170272	2.41E-14	postive

METTL17	AL162431.1	0.403879194	4.85E-21	postive
METTL17	AL162586.1	0.48598416	5.35E-31	postive
METTL17	AL162632.1	0.368585442	1.55E-17	postive
METTL17	AL162741.1	0.498111932	1.03E-32	postive
METTL17	AL163051.1	0.383561681	5.70E-19	postive
METTL17	AL353194.1	0.513034337	6.46E-35	postive
METTL17	AL353593.1	0.303407575	4.16E-12	postive
METTL17	AL353596.1	0.354597861	2.92E-16	postive
METTL17	AL353622.1	0.51280782	6.99E-35	postive
METTL17	AL353622.2	0.460971064	1.13E-27	postive
METTL17	AL353625.1	0.519137109	7.52E-36	postive
METTL17	AL353708.1	0.336342469	1.09E-14	postive
METTL17	AL353708.3	0.440841165	3.44E-25	postive
METTL17	AL353743.1	0.504828446	1.09E-33	postive
METTL17	AL353795.2	0.357316864	1.67E-16	postive
METTL17	AL353796.1	0.370044115	1.13E-17	postive
METTL17	AL353801.3	0.449821639	2.81E-26	postive
METTL17	AL353807.2	0.342146934	3.54E-15	postive
METTL17	AL353997.2	0.392379968	7.50E-20	postive
METTL17	AL354696.1	0.482122818	1.82E-30	postive
METTL17	AL354696.2	0.375433978	3.50E-18	postive
METTL17	AL354707.1	0.360277024	9.04E-17	postive
METTL17	AL354718.3	0.331565808	2.70E-14	postive
METTL17	AL354733.3	0.54720282	2.14E-40	postive
METTL17	AL354740.1	0.401857513	7.91E-21	postive
METTL17	AL354760.1	0.429933525	6.53E-24	postive
METTL17	AL354809.1	0.415722527	2.58E-22	postive
METTL17	AL354836.1	0.537490229	8.95E-39	postive
METTL17	AL354892.2	0.464352902	4.17E-28	postive
METTL17	AL354892.3	0.573367982	4.86E-45	postive
METTL17	AL354920.1	0.34783931	1.15E-15	postive
METTL17	AL355001.2	0.435651142	1.42E-24	postive
METTL17	AL355075.2	0.610335952	2.35E-52	postive
METTL17	AL355297.2	0.344369802	2.29E-15	postive
METTL17	AL355297.3	0.394799629	4.25E-20	postive
METTL17	AL355353.2	0.446444377	7.28E-26	postive
METTL17	AL355385.1	0.549043746	1.04E-40	postive
METTL17	AL355388.1	0.598454608	6.70E-50	postive
METTL17	AL355472.3	0.300543705	6.75E-12	postive
METTL17	AL355488.1	0.485987012	5.35E-31	postive
METTL17	AL355490.3	0.368023566	1.75E-17	postive
METTL17	AL355574.1	0.462670112	6.86E-28	postive
METTL17	AL355802.3	0.501095458	3.83E-33	postive
METTL17	AL356019.2	0.463210199	5.85E-28	postive
METTL17	AL356299.3	0.578651601	4.99E-46	postive
METTL17	AL356481.1	0.474625691	1.87E-29	postive
METTL17	AL356481.3	0.521879456	2.82E-36	postive
METTL17	AL356488.3	0.369222211	1.35E-17	postive
METTL17	AL356512.1	0.473333237	2.78E-29	postive
METTL17	AL356599.1	0.340425876	4.95E-15	postive

METTL17	AL357079.1	0.508721461	2.87E-34	postive
METTL17	AL357140.2	0.447421201	5.54E-26	postive
METTL17	AL357874.1	0.392046773	8.10E-20	postive
METTL17	AL357874.2	0.338397423	7.34E-15	postive
METTL17	AL358072.1	0.395361882	3.72E-20	postive
METTL17	AL358075.2	0.318761471	2.85E-13	postive
METTL17	AL358115.1	0.373523431	5.32E-18	postive
METTL17	AL358334.3	0.384863416	4.24E-19	postive
METTL17	AL358472.2	0.605654291	2.24E-51	postive
METTL17	AL358472.3	0.442422428	2.23E-25	postive
METTL17	AL358781.2	0.371181186	8.87E-18	postive
METTL17	AL358852.1	0.42314879	3.86E-23	postive
METTL17	AL358933.1	0.336773651	1.00E-14	postive
METTL17	AL359091.3	0.455318586	5.86E-27	postive
METTL17	AL359091.4	0.314255943	6.37E-13	postive
METTL17	AL359198.1	0.324471205	1.01E-13	postive
METTL17	AL359317.1	0.465514985	2.95E-28	postive
METTL17	AL359317.2	0.325900079	7.77E-14	postive
METTL17	AL359397.1	0.307672514	2.01E-12	postive
METTL17	AL359504.2	0.450828446	2.12E-26	postive
METTL17	AL359513.1	0.414693657	3.35E-22	postive
METTL17	AL359532.1	0.364257225	3.91E-17	postive
METTL17	AL359636.2	0.340038756	5.34E-15	postive
METTL17	AL359643.3	0.309467422	1.47E-12	postive
METTL17	AL359711.2	0.331983932	2.50E-14	postive
METTL17	AL359715.1	0.559681808	1.47E-42	postive
METTL17	AL359715.2	0.471308236	5.16E-29	postive
METTL17	AL359715.3	0.354020283	3.29E-16	postive
METTL17	AL359878.1	0.378851991	1.64E-18	postive
METTL17	AL359921.1	0.533072653	4.70E-38	postive
METTL17	AL359921.2	0.570049367	1.99E-44	postive
METTL17	AL360181.2	0.542169321	1.50E-39	postive
METTL17	AL360270.3	0.301334145	5.91E-12	postive
METTL17	AL365330.1	0.570149574	1.91E-44	postive
METTL17	AL390037.1	0.311275079	1.07E-12	postive
METTL17	AL390066.2	0.362427702	5.76E-17	postive
METTL17	AL390195.2	0.304756173	3.31E-12	postive
METTL17	AL390719.1	0.357683654	1.55E-16	postive
METTL17	AL390719.2	0.533408908	4.15E-38	postive
METTL17	AL390728.4	0.594776545	3.68E-49	postive
METTL17	AL390728.6	0.513899766	4.77E-35	postive
METTL17	AL390760.1	0.313162153	7.72E-13	postive
METTL17	AL390879.1	0.469762102	8.24E-29	postive
METTL17	AL390955.2	0.334371076	1.59E-14	postive
METTL17	AL391069.2	0.39508295	3.98E-20	postive
METTL17	AL391069.3	0.333759684	1.78E-14	postive
METTL17	AL391152.1	0.357740772	1.53E-16	postive
METTL17	AL391244.1	0.432429045	3.36E-24	postive
METTL17	AL391244.2	0.564528011	2.01E-43	postive
METTL17	AL391684.1	0.508207005	3.43E-34	postive

METTL17	AL391832.2	0.334007265	1.70E-14	postive
METTL17	AL391834.2	0.313775356	6.93E-13	postive
METTL17	AL392172.1	0.344471777	2.24E-15	postive
METTL17	AL441992.2	0.439568338	4.88E-25	postive
METTL17	AL442128.2	0.465241741	3.20E-28	postive
METTL17	AL442663.3	0.444756787	1.17E-25	postive
METTL17	AL445222.2	0.450726348	2.18E-26	postive
METTL17	AL445228.3	0.399229168	1.49E-20	postive
METTL17	AL445231.1	0.369803639	1.19E-17	postive
METTL17	AL445490.1	0.409341144	1.27E-21	postive
METTL17	AL445645.1	0.443307686	1.74E-25	postive
METTL17	AL445931.1	0.337762217	8.30E-15	postive
METTL17	AL450263.1	0.379177784	1.53E-18	postive
METTL17	AL450306.1	0.403948739	4.77E-21	postive
METTL17	AL450384.2	0.466964225	1.91E-28	postive
METTL17	AL450998.2	0.538456266	6.21E-39	postive
METTL17	AL450998.3	0.521634657	3.08E-36	postive
METTL17	AL451050.2	0.583909513	4.96E-47	postive
METTL17	AL451074.2	0.304219865	3.63E-12	postive
METTL17	AL451085.2	0.440740884	3.54E-25	postive
METTL17	AL451085.3	0.496994083	1.50E-32	postive
METTL17	AL451165.2	0.423356924	3.66E-23	postive
METTL17	AL512303.1	0.365144507	3.24E-17	postive
METTL17	AL512343.2	0.370697344	9.85E-18	postive
METTL17	AL512408.1	0.468033475	1.39E-28	postive
METTL17	AL512625.1	0.337027765	9.55E-15	postive
METTL17	AL512625.2	0.350777028	6.36E-16	postive
METTL17	AL512652.1	0.44105699	3.24E-25	postive
METTL17	AL512770.1	0.589066114	4.95E-48	postive
METTL17	AL512791.1	0.552883198	2.27E-41	postive
METTL17	AL512791.2	0.437229077	9.23E-25	postive
METTL17	AL513165.1	0.353549014	3.62E-16	postive
METTL17	AL513190.1	0.381247927	9.60E-19	postive
METTL17	AL513217.1	0.311763712	9.87E-13	postive
METTL17	AL513218.1	0.553400562	1.85E-41	postive
METTL17	AL513320.1	0.587604763	9.56E-48	postive
METTL17	AL513327.1	0.362809241	5.31E-17	postive
METTL17	AL513327.3	0.302059106	5.23E-12	postive
METTL17	AL513477.1	0.369493373	1.28E-17	postive
METTL17	AL513477.2	0.410496461	9.56E-22	postive
METTL17	AL513534.3	0.316344602	4.40E-13	postive
METTL17	AL513550.1	0.515599406	2.63E-35	postive
METTL17	AL583722.3	0.347337766	1.27E-15	postive
METTL17	AL583810.1	0.599299477	4.51E-50	postive
METTL17	AL583810.2	0.411706553	7.07E-22	postive
METTL17	AL583856.2	0.370822044	9.58E-18	postive
METTL17	AL589765.6	0.384687094	4.41E-19	postive
METTL17	AL589843.1	0.323524255	1.20E-13	postive
METTL17	AL589843.2	0.313289751	7.55E-13	postive
METTL17	AL590096.1	0.464027141	4.59E-28	postive

METTL17	AL590133.1	0.449590662	3.00E-26	postive
METTL17	AL590226.2	0.303011333	4.45E-12	postive
METTL17	AL590369.1	0.500551747	4.59E-33	postive
METTL17	AL590560.3	0.365731054	2.86E-17	postive
METTL17	AL590617.2	0.531126937	9.69E-38	postive
METTL17	AL590652.1	0.522083094	2.62E-36	postive
METTL17	AL590666.1	0.302530146	4.83E-12	postive
METTL17	AL590708.1	0.406868817	2.34E-21	postive
METTL17	AL590729.1	0.45157669	1.71E-26	postive
METTL17	AL590822.1	0.389647243	1.41E-19	postive
METTL17	AL591684.2	0.379616155	1.38E-18	postive
METTL17	AL591848.3	0.445324672	9.96E-26	postive
METTL17	AL591895.1	0.453909327	8.78E-27	postive
METTL17	AL592071.1	0.38764886	2.24E-19	postive
METTL17	AL592148.3	0.326789934	6.59E-14	postive
METTL17	AL592166.1	0.552095625	3.11E-41	postive
METTL17	AL592211.1	0.552043344	3.18E-41	postive
METTL17	AL592435.1	0.469132915	9.96E-29	postive
METTL17	AL592494.2	0.346580438	1.48E-15	postive
METTL17	AL596094.1	0.438377042	6.76E-25	postive
METTL17	AL596202.1	0.502399615	2.47E-33	postive
METTL17	AL596325.2	0.324338731	1.04E-13	postive
METTL17	AL603832.1	0.469283104	9.52E-29	postive
METTL17	AL603839.2	0.446978432	6.27E-26	postive
METTL17	AL603839.3	0.405302652	3.43E-21	postive
METTL17	AL604028.1	0.43643657	1.14E-24	postive
METTL17	AL606489.1	0.315457758	5.15E-13	postive
METTL17	AL606491.1	0.346932752	1.38E-15	postive
METTL17	AL606534.1	0.311006182	1.13E-12	postive
METTL17	AL606760.1	0.341121849	4.32E-15	postive
METTL17	AL606760.2	0.583954686	4.86E-47	postive
METTL17	AL606760.3	0.314904352	5.68E-13	postive
METTL17	AL606834.1	0.344454474	2.25E-15	postive
METTL17	AL606834.2	0.381043808	1.00E-18	postive
METTL17	AL627171.3	0.41805774	1.43E-22	postive
METTL17	AL627309.2	0.398784818	1.65E-20	postive
METTL17	AL627309.5	0.404055969	4.65E-21	postive
METTL17	AL645568.1	0.543780118	8.09E-40	postive
METTL17	AL645608.1	0.34817099	1.07E-15	postive
METTL17	AL645608.2	0.432032314	3.74E-24	postive
METTL17	AL645608.6	0.372659733	6.43E-18	postive
METTL17	AL645608.7	0.306245736	2.57E-12	postive
METTL17	AL645728.1	0.412235978	6.20E-22	postive
METTL17	AL645933.3	0.399005299	1.57E-20	postive
METTL17	AL645940.1	0.476934517	9.19E-30	postive
METTL17	AL645941.1	0.507301916	4.68E-34	postive
METTL17	AL662791.1	0.332384409	2.32E-14	postive
METTL17	AL662797.2	0.477283882	8.24E-30	postive
METTL17	AL662844.3	0.431871714	3.90E-24	postive
METTL17	AL662844.4	0.534788469	2.48E-38	postive

METTL17	AL662884.1	0.431973505	3.80E-24	postive
METTL17	AL669831.1	0.475264506	1.54E-29	postive
METTL17	AL671710.1	0.590430344	2.67E-48	postive
METTL17	AL683813.2	0.424048564	3.06E-23	postive
METTL17	AL691403.2	0.345561065	1.81E-15	postive
METTL17	AL691432.1	0.517158152	1.52E-35	postive
METTL17	AL691432.2	0.443875742	1.49E-25	postive
METTL17	AL691482.4	0.448926986	3.62E-26	postive
METTL17	AL731533.2	0.360453791	8.71E-17	postive
METTL17	AL731563.4	0.395941906	3.25E-20	postive
METTL17	AL731566.2	0.45059049	2.26E-26	postive
METTL17	AL731567.1	0.338636174	7.01E-15	postive
METTL17	AL731569.1	0.591825529	1.42E-48	postive
METTL17	AL731571.1	0.561852845	6.04E-43	postive
METTL17	AL731577.2	0.34024969	5.12E-15	postive
METTL17	AL732314.6	0.429635522	7.07E-24	postive
METTL17	AL732314.8	0.467877942	1.45E-28	postive
METTL17	AL732372.2	0.505864269	7.64E-34	postive
METTL17	AL845552.2	0.377967674	2.00E-18	postive
METTL17	AL928654.1	0.436887902	1.01E-24	postive
METTL17	AL928654.2	0.703960008	5.06E-76	postive
METTL17	ALG1L13P	0.492625092	6.29E-32	postive
METTL17	ALOX12-AS1	0.556730624	4.86E-42	postive
METTL17	ALOX12P2	0.350473007	6.76E-16	postive
METTL17	ALOX15P1	0.356351287	2.04E-16	postive
METTL17	AMZ2P1	0.4722463	3.88E-29	postive
METTL17	ANKRD10-IT1	0.395719476	3.42E-20	postive
METTL17	ANKRD19P	0.301675089	5.58E-12	postive
METTL17	ANO7L1	0.529572622	1.72E-37	postive
METTL17	ANP32AP1	0.305772249	2.78E-12	postive
METTL17	AP000238.1	0.33579685	1.21E-14	postive
METTL17	AP000251.1	0.333160406	2.00E-14	postive
METTL17	AP000253.1	0.354498959	2.98E-16	postive
METTL17	AP000254.2	0.485504606	6.23E-31	postive
METTL17	AP000255.1	0.370547097	1.02E-17	postive
METTL17	AP000346.1	0.312763511	8.28E-13	postive
METTL17	AP000346.2	0.512986709	6.56E-35	postive
METTL17	AP000347.1	0.487154342	3.68E-31	postive
METTL17	AP000350.5	0.377033127	2.46E-18	postive
METTL17	AP000350.7	0.401151872	9.38E-21	postive
METTL17	AP000442.1	0.611425824	1.38E-52	postive
METTL17	AP000446.1	0.337631845	8.51E-15	postive
METTL17	AP000473.5	0.33529791	1.33E-14	postive
METTL17	AP000487.1	0.514818471	3.46E-35	postive
METTL17	AP000525.1	0.37169041	7.94E-18	postive
METTL17	AP000553.2	0.358058095	1.43E-16	postive
METTL17	AP000640.1	0.32165643	1.69E-13	postive
METTL17	AP000692.1	0.482341154	1.70E-30	postive
METTL17	AP000704.2	0.445251282	1.02E-25	postive
METTL17	AP000781.1	0.477834439	6.95E-30	postive

METTL17	AP000787.1	0.321439514	1.76E-13	postive
METTL17	AP000845.1	0.458883136	2.08E-27	postive
METTL17	AP000866.1	0.41396004	4.03E-22	postive
METTL17	AP000866.2	0.321964148	1.60E-13	postive
METTL17	AP000866.5	0.319741756	2.39E-13	postive
METTL17	AP000873.2	0.443866826	1.49E-25	postive
METTL17	AP000873.4	0.488036198	2.77E-31	postive
METTL17	AP000880.1	0.311551782	1.02E-12	postive
METTL17	AP000892.3	0.337820649	8.20E-15	postive
METTL17	AP000894.4	0.343513002	2.71E-15	postive
METTL17	AP001021.2	0.352514749	4.47E-16	postive
METTL17	AP001029.1	0.372480993	6.68E-18	postive
METTL17	AP001029.2	0.377537422	2.20E-18	postive
METTL17	AP001062.1	0.430043927	6.34E-24	postive
METTL17	AP001107.1	0.582036621	1.13E-46	postive
METTL17	AP001107.4	0.554852963	1.04E-41	postive
METTL17	AP001107.6	0.419307959	1.04E-22	postive
METTL17	AP001107.7	0.303086444	4.40E-12	postive
METTL17	AP001107.8	0.400552012	1.08E-20	postive
METTL17	AP001107.9	0.398140987	1.93E-20	postive
METTL17	AP001160.1	0.668841046	3.95E-66	postive
METTL17	AP001160.2	0.377556089	2.19E-18	postive
METTL17	AP001160.3	0.517271917	1.46E-35	postive
METTL17	AP001160.4	0.356395166	2.02E-16	postive
METTL17	AP001178.3	0.393790411	5.39E-20	postive
METTL17	AP001267.1	0.420868621	6.96E-23	postive
METTL17	AP001267.3	0.393851369	5.31E-20	postive
METTL17	AP001318.2	0.380765783	1.07E-18	postive
METTL17	AP001347.1	0.359696345	1.02E-16	postive
METTL17	AP001351.1	0.339771764	5.62E-15	postive
METTL17	AP001372.2	0.382608894	7.06E-19	postive
METTL17	AP001412.1	0.479974766	3.57E-30	postive
METTL17	AP001432.1	0.330076886	3.57E-14	postive
METTL17	AP001453.2	0.507644465	4.16E-34	postive
METTL17	AP001453.4	0.540822061	2.52E-39	postive
METTL17	AP001458.1	0.407633498	1.94E-21	postive
METTL17	AP001462.1	0.507954766	3.74E-34	postive
METTL17	AP001469.2	0.417151326	1.80E-22	postive
METTL17	AP001469.3	0.449102911	3.45E-26	postive
METTL17	AP001486.2	0.307717964	1.99E-12	postive
METTL17	AP001505.1	0.306610901	2.41E-12	postive
METTL17	AP001527.2	0.456203721	4.54E-27	postive
METTL17	AP001619.1	0.376631096	2.69E-18	postive
METTL17	AP001625.2	0.339131459	6.37E-15	postive
METTL17	AP001628.1	0.404573514	4.10E-21	postive
METTL17	AP001793.1	0.387810879	2.16E-19	postive
METTL17	AP001816.1	0.400231731	1.17E-20	postive
METTL17	AP001830.1	0.436819429	1.03E-24	postive
METTL17	AP001893.1	0.434466328	1.95E-24	postive
METTL17	AP001992.1	0.30983839	1.38E-12	postive

METTL17	AP002026.1	0.555202673	9.00E-42	postive
METTL17	AP002360.2	0.408972764	1.39E-21	postive
METTL17	AP002387.2	0.383313418	6.02E-19	postive
METTL17	AP002490.1	0.491744777	8.38E-32	postive
METTL17	AP002748.4	0.432493924	3.31E-24	postive
METTL17	AP002761.1	0.316488244	4.28E-13	postive
METTL17	AP002761.3	0.324075345	1.09E-13	postive
METTL17	AP002807.1	0.501618715	3.21E-33	postive
METTL17	AP002812.2	0.355259202	2.55E-16	postive
METTL17	AP002840.2	0.387438159	2.35E-19	postive
METTL17	AP003059.2	0.358461031	1.32E-16	postive
METTL17	AP003068.1	0.380742253	1.08E-18	postive
METTL17	AP003068.2	0.331753236	2.61E-14	postive
METTL17	AP003096.1	0.57275653	6.31E-45	postive
METTL17	AP003119.2	0.31869401	2.89E-13	postive
METTL17	AP003170.3	0.390001744	1.30E-19	postive
METTL17	AP003352.1	0.52207749	2.63E-36	postive
METTL17	AP003392.1	0.424088853	3.03E-23	postive
METTL17	AP003392.4	0.391875659	8.43E-20	postive
METTL17	AP003392.5	0.458267409	2.49E-27	postive
METTL17	AP003419.3	0.493153548	5.29E-32	postive
METTL17	AP003486.1	0.412705368	5.51E-22	postive
METTL17	AP003498.1	0.437196221	9.32E-25	postive
METTL17	AP003721.4	0.308084874	1.87E-12	postive
METTL17	AP004608.1	0.365963405	2.72E-17	postive
METTL17	AP004609.5	0.387676139	2.23E-19	postive
METTL17	AP005061.2	0.369665108	1.23E-17	postive
METTL17	AP005329.1	0.496844227	1.57E-32	postive
METTL17	AP005432.2	0.33436343	1.59E-14	postive
METTL17	AP005482.4	0.333712166	1.80E-14	postive
METTL17	AP005899.1	0.306903866	2.29E-12	postive
METTL17	AP006216.1	0.39038342	1.19E-19	postive
METTL17	AP006222.1	0.382628669	7.03E-19	postive
METTL17	AP006287.2	0.334251832	1.62E-14	postive
METTL17	AP006621.2	0.574550655	2.93E-45	postive
METTL17	AP006621.3	0.584061697	4.64E-47	postive
METTL17	AP006621.4	0.505554203	8.49E-34	postive
METTL17	AP006623.1	0.474225023	2.12E-29	postive
METTL17	AP4B1-AS1	0.438960635	5.76E-25	postive
METTL17	APTR	0.437347369	8.94E-25	postive
METTL17	ARHGAP27P1-BPTFP1-KPNA2P3	0.375771357	3.25E-18	postive
METTL17	ARHGAP5-AS1	0.337665102	8.45E-15	postive
METTL17	ARHGEF2-AS1	0.325446243	8.45E-14	postive
METTL17	ARHGEF35-AS1	0.413129086	4.96E-22	postive
METTL17	ARMCX5-GPRASP2	0.418610033	1.24E-22	postive
METTL17	ARRDC1-AS1	0.645102166	3.55E-60	postive
METTL17	ASB16-AS1	0.605386927	2.54E-51	postive
METTL17	ASH1L-AS1	0.56503649	1.62E-43	postive
METTL17	ASMTL-AS1	0.537239795	9.84E-39	postive

METTL17	ATF4P4	0.387948039	2.09E-19	postive
METTL17	ATP1A1-AS1	0.346078977	1.63E-15	postive
METTL17	ATP2A1-AS1	0.388005347	2.07E-19	postive
METTL17	ATP2B1-AS1	0.357868635	1.49E-16	postive
METTL17	ATP2C2-AS1	0.445498209	9.48E-26	postive
METTL17	ATP6V0E2-AS1	0.423112458	3.90E-23	postive
METTL17	ATXN1-AS1	0.461523708	9.61E-28	postive
METTL17	ATXN2-AS	0.483751042	1.09E-30	postive
METTL17	BACE1-AS	0.492652147	6.23E-32	postive
METTL17	BAIAP2-DT	0.309190135	1.55E-12	postive
METTL17	BCDIN3D-AS1	0.563443799	3.14E-43	postive
METTL17	BDNF-AS	0.306321016	2.53E-12	postive
METTL17	BHLHE40-AS1	0.360529363	8.57E-17	postive
METTL17	BMS1P1	0.373700567	5.12E-18	postive
METTL17	BMS1P2	0.351027354	6.05E-16	postive
METTL17	BMS1P20	0.492356172	6.86E-32	postive
METTL17	BMS1P4	0.407948481	1.79E-21	postive
METTL17	BOLA2P2	0.463518174	5.34E-28	postive
METTL17	BRWD1-AS2	0.314172107	6.46E-13	postive
METTL17	BTG3-AS1	0.401681535	8.25E-21	postive
METTL17	BTN2A3P	0.591292949	1.81E-48	postive
METTL17	BTNL10	0.315531726	5.08E-13	postive
METTL17	BVES-AS1	0.312925846	8.05E-13	postive
METTL17	BX284668.5	0.321048772	1.89E-13	postive
METTL17	BX293535.1	0.394733643	4.32E-20	postive
METTL17	BX322557.10	0.384442682	4.66E-19	postive
METTL17	BX537318.1	0.495074157	2.82E-32	postive
METTL17	BX539320.1	0.339056586	6.46E-15	postive
METTL17	BX649601.1	0.426120991	1.78E-23	postive
METTL17	BX649632.1	0.492482341	6.59E-32	postive
METTL17	C10orf25	0.321716108	1.67E-13	postive
METTL17	C15orf56	0.333602963	1.84E-14	postive
METTL17	C18orf65	0.304332279	3.56E-12	postive
METTL17	C1orf147	0.474848862	1.75E-29	postive
METTL17	C1orf220	0.450652832	2.22E-26	postive
METTL17	C1orf229	0.448078436	4.60E-26	postive
METTL17	C1RL-AS1	0.496558206	1.73E-32	postive
METTL17	C22orf24	0.428308168	1.00E-23	postive
METTL17	C22orf46	0.451546876	1.72E-26	postive
METTL17	C2-AS1	0.482945063	1.40E-30	postive
METTL17	C3orf35	0.411244151	7.94E-22	postive
METTL17	C5orf66	0.45664365	3.99E-27	postive
METTL17	C6orf99	0.305574117	2.88E-12	postive
METTL17	C9orf139	0.393661656	5.55E-20	postive
METTL17	C9orf147	0.433983329	2.22E-24	postive
METTL17	C9orf163	0.517475251	1.36E-35	postive
METTL17	CA5BP1	0.458657198	2.23E-27	postive
METTL17	CACTIN-AS1	0.485110901	7.07E-31	postive
METTL17	CAHM	0.503739484	1.57E-33	postive
METTL17	CAMTA1-DT	0.398309096	1.85E-20	postive

METTL17	CAPN10-DT	0.651841166	8.21E-62	postive
METTL17	CASK-AS1	0.407222878	2.14E-21	postive
METTL17	CASP16P	0.39798338	2.00E-20	postive
METTL17	CATSPER2P1	0.354241517	3.15E-16	postive
METTL17	CBR3-AS1	0.425717199	1.98E-23	postive
METTL17	CBX3P4	0.388259168	1.95E-19	postive
METTL17	CCDC183-AS1	0.531783221	7.60E-38	postive
METTL17	CCDC18-AS1	0.406426487	2.61E-21	postive
METTL17	CCDC84-DT	0.37942099	1.45E-18	postive
METTL17	CCNT2-AS1	0.498191603	1.01E-32	postive
METTL17	CCT6P1	0.585088061	2.94E-47	postive
METTL17	CCT6P3	0.47211526	4.03E-29	postive
METTL17	CD27-AS1	0.597915359	8.61E-50	postive
METTL17	CD2BP2-DT	0.326916548	6.44E-14	postive
METTL17	CD99P1	0.39814373	1.93E-20	postive
METTL17	CDC37L1-DT	0.43111113	4.78E-24	postive
METTL17	CEBPB-AS1	0.44048799	3.79E-25	postive
METTL17	CENPBD1P1	0.408500351	1.57E-21	postive
METTL17	CEP250-AS1	0.343599151	2.66E-15	postive
METTL17	CEP83-DT	0.410857109	8.74E-22	postive
METTL17	CERNA1	0.311890855	9.65E-13	postive
METTL17	CFL1P1	0.356769043	1.87E-16	postive
METTL17	CHKB-CPT1B	0.464971226	3.47E-28	postive
METTL17	CHKB-DT	0.34539492	1.87E-15	postive
METTL17	CHROMR	0.443078826	1.86E-25	postive
METTL17	CIDECP1	0.531162742	9.56E-38	postive
METTL17	CIRBP-AS1	0.416054436	2.37E-22	postive
METTL17	CKMT2-AS1	0.559964498	1.31E-42	postive
METTL17	CLUHP3	0.520133736	5.27E-36	postive
METTL17	CNNM3-DT	0.483059909	1.35E-30	postive
METTL17	COA6-AS1	0.426296414	1.70E-23	postive
METTL17	COL4A2-AS1	0.3276157	5.66E-14	postive
METTL17	COL6A4P2	0.411023843	8.38E-22	postive
METTL17	COLCA1	0.36026306	9.06E-17	postive
METTL17	COX10-AS1	0.519700159	6.16E-36	postive
METTL17	CR381653.1	0.39467076	4.38E-20	postive
METTL17	CR559946.1	0.373149978	5.77E-18	postive
METTL17	CR559946.2	0.46530858	3.14E-28	postive
METTL17	CR936218.1	0.303334546	4.22E-12	postive
METTL17	CRNDE	0.426525288	1.60E-23	postive
METTL17	CROCCP2	0.528349655	2.70E-37	postive
METTL17	CROCCP3	0.558145055	2.74E-42	postive
METTL17	CRTC3-AS1	0.377530047	2.20E-18	postive
METTL17	CRYBB2P1	0.593651764	6.16E-49	postive
METTL17	CRYZL2P	0.448859185	3.69E-26	postive
METTL17	CRYZL2P-SEC16B	0.36057824	8.49E-17	postive
METTL17	CSE1L-AS1	0.386231491	3.10E-19	postive
METTL17	CSPG4P10	0.32227368	1.51E-13	postive
METTL17	CT66	0.469144064	9.93E-29	postive
METTL17	CTA-14H9.5	0.309172131	1.55E-12	postive

METTL17	CTA-292E10.9	0.357322686	1.67E-16	postive
METTL17	CTA-989H11.1	0.425887655	1.89E-23	postive
METTL17	CTB-119C2.1	0.31471499	5.87E-13	postive
METTL17	CTBP1-AS	0.587423371	1.04E-47	postive
METTL17	CTBP1-DT	0.516461058	1.94E-35	postive
METTL17	CTC-487M23.5	0.302987105	4.47E-12	postive
METTL17	CTD-2132N18.4	0.540676595	2.67E-39	postive
METTL17	CTD-2134A5.3	0.577237723	9.21E-46	postive
METTL17	CTD-2240J17.2	0.322404848	1.48E-13	postive
METTL17	CTD-2521M24.9	0.443782214	1.53E-25	postive
METTL17	CTD-2527I21.9	0.30355928	4.06E-12	postive
METTL17	CTD-2574D22.4	0.522548693	2.22E-36	postive
METTL17	CTSLP3	0.334689222	1.49E-14	postive
METTL17	CU633906.2	0.315600359	5.02E-13	postive
METTL17	CU633967.1	0.415644825	2.63E-22	postive
METTL17	CU638689.4	0.302240016	5.07E-12	postive
METTL17	CUTALP	0.481163381	2.46E-30	postive
METTL17	CXXC5-AS1	0.334768577	1.47E-14	postive
METTL17	CYP4A22-AS1	0.313683338	7.04E-13	postive
METTL17	CYP4F23P	0.336775263	1.00E-14	postive
METTL17	CYP51A1-AS1	0.372176276	7.14E-18	postive
METTL17	DAAM2-AS1	0.376754475	2.61E-18	postive
METTL17	DACT3-AS1	0.349662357	7.96E-16	postive
METTL17	DANCR	0.337885345	8.10E-15	postive
METTL17	DARS-AS1	0.468419527	1.24E-28	postive
METTL17	DBH-AS1	0.329628619	3.89E-14	postive
METTL17	DBIL5P	0.401621987	8.37E-21	postive
METTL17	DCST1-AS1	0.441299139	3.04E-25	postive
METTL17	DCXR-DT	0.312722222	8.34E-13	postive
METTL17	DDX11-AS1	0.466686977	2.08E-28	postive
METTL17	DDX39B-AS1	0.398910108	1.60E-20	postive
METTL17	DGCR11	0.351259295	5.77E-16	postive
METTL17	DGUOK-AS1	0.482479482	1.63E-30	postive
METTL17	DHRS4-AS1	0.543227064	1.00E-39	postive
METTL17	DHRS4L1	0.41853956	1.26E-22	postive
METTL17	DICER1-AS1	0.583451609	6.08E-47	postive
METTL17	DLG5-AS1	0.340106415	5.27E-15	postive
METTL17	DLGAP4-AS1	0.416159548	2.31E-22	postive
METTL17	DM1-AS	0.601152056	1.89E-50	postive
METTL17	DNAJC27-AS1	0.302404285	4.94E-12	postive
METTL17	DNAJC3-DT	0.424306946	2.86E-23	postive
METTL17	DNM1P35	0.369975303	1.15E-17	postive
METTL17	DOC2GP	0.400093651	1.21E-20	postive
METTL17	DOCK9-DT	0.400645788	1.06E-20	postive
METTL17	DPP9-AS1	0.548557733	1.26E-40	postive
METTL17	DPY19L2P3	0.406166006	2.78E-21	postive
METTL17	DSCAS	0.345132042	1.97E-15	postive
METTL17	DSCR9	0.333964666	1.72E-14	postive
METTL17	DTX2P1-UPK3BP1-PMS2P11	0.391116423	1.01E-19	postive
METTL17	DUXAP8	0.326787195	6.60E-14	postive

METTL17	DUXAP9	0.345567323	1.80E-15	postive
METTL17	DYRK3-AS1	0.325974645	7.67E-14	postive
METTL17	EAF1-AS1	0.356632449	1.93E-16	postive
METTL17	EBLN3P	0.435964429	1.30E-24	postive
METTL17	ECI2-DT	0.329564103	3.93E-14	postive
METTL17	EDRF1-AS1	0.315019257	5.56E-13	postive
METTL17	EDRF1-DT	0.340160738	5.21E-15	postive
METTL17	EHMT2-AS1	0.573395673	4.81E-45	postive
METTL17	EIF1B-AS1	0.387545362	2.30E-19	postive
METTL17	EIF2AK3-DT	0.437173126	9.37E-25	postive
METTL17	EIF3J-DT	0.539623507	3.98E-39	postive
METTL17	ELF3-AS1	0.526486603	5.33E-37	postive
METTL17	ELOA-AS1	0.515506793	2.72E-35	postive
METTL17	EMC1-AS1	0.47522286	1.56E-29	postive
METTL17	EMC3-AS1	0.469891814	7.92E-29	postive
METTL17	ENO1-AS1	0.372755193	6.29E-18	postive
METTL17	ENTPD3-AS1	0.378272899	1.87E-18	postive
METTL17	EP300-AS1	0.337104294	9.42E-15	postive
METTL17	EP400P1	0.521902122	2.80E-36	postive
METTL17	EPB41L4A-AS1	0.320119435	2.23E-13	postive
METTL17	EPB41L4A-DT	0.342559533	3.26E-15	postive
METTL17	EPCAM-DT	0.32524539	8.77E-14	postive
METTL17	ERICD	0.330201456	3.49E-14	postive
METTL17	ERVK13-1	0.411083047	8.26E-22	postive
METTL17	ESPNP	0.408955691	1.40E-21	postive
METTL17	ETV5-AS1	0.339084353	6.43E-15	postive
METTL17	ETV7-AS1	0.438107841	7.27E-25	postive
METTL17	EXOC3-AS1	0.371098509	9.03E-18	postive
METTL17	EXOSC10-AS1	0.357736304	1.53E-16	postive
METTL17	EXTL3-AS1	0.416150001	2.32E-22	postive
METTL17	FAAHP1	0.314029328	6.63E-13	postive
METTL17	FABP5P3	0.343445318	2.74E-15	postive
METTL17	FAHD2CP	0.490781155	1.14E-31	postive
METTL17	FALEC	0.481733955	2.06E-30	postive
METTL17	FAM111A-DT	0.497612238	1.22E-32	postive
METTL17	FAM135A-AS1	0.355036846	2.67E-16	postive
METTL17	FAM13A-AS1	0.33252496	2.25E-14	postive
METTL17	FAM157A	0.330286974	3.44E-14	postive
METTL17	FAM157B	0.378383385	1.82E-18	postive
METTL17	FAM157C	0.490154878	1.40E-31	postive
METTL17	FAM160A1-DT	0.408539223	1.55E-21	postive
METTL17	FAM201A	0.310272499	1.28E-12	postive
METTL17	FAM21EP	0.391249901	9.75E-20	postive
METTL17	FAM21FP	0.432265459	3.51E-24	postive
METTL17	FAM41C	0.346872738	1.39E-15	postive
METTL17	FAM66C	0.31233883	8.92E-13	postive
METTL17	FAM66D	0.394736912	4.31E-20	postive
METTL17	FAM83C-AS1	0.389166487	1.58E-19	postive
METTL17	FAM86B3P	0.572976116	5.75E-45	postive
METTL17	FAM86C2P	0.504287006	1.30E-33	postive

METTL17	FAM86DP	0.332930318	2.09E-14	postive
METTL17	FAM86EP	0.408723697	1.48E-21	postive
METTL17	FAM86FP	0.382922478	6.58E-19	postive
METTL17	FAM86HP	0.577050449	9.99E-46	postive
METTL17	FAM86JP	0.467180168	1.79E-28	postive
METTL17	FBXL19-AS1	0.320278577	2.17E-13	postive
METTL17	FGD5-AS1	0.43898982	5.72E-25	postive
METTL17	FGF7P3	0.388369252	1.90E-19	postive
METTL17	FGF7P6	0.312855796	8.15E-13	postive
METTL17	FLNB-AS1	0.396500059	2.85E-20	postive
METTL17	FLRT1	0.346927803	1.38E-15	postive
METTL17	FLVCR1-DT	0.371251136	8.73E-18	postive
METTL17	FMR1-IT1	0.462615117	6.97E-28	postive
METTL17	FO393418.1	0.422420192	4.66E-23	postive
METTL17	FOXD2-AS1	0.423551872	3.48E-23	postive
METTL17	FOXD3-AS1	0.305211663	3.06E-12	postive
METTL17	FOXO6-AS1	0.429225503	7.88E-24	postive
METTL17	FOXP4-AS1	0.3354072	1.30E-14	postive
METTL17	FRG1-DT	0.443351812	1.72E-25	postive
METTL17	FRG1HP	0.554481058	1.20E-41	postive
METTL17	FRMD6-AS1	0.34174825	3.83E-15	postive
METTL17	FUT8-AS1	0.300530541	6.77E-12	postive
METTL17	FZD4-DT	0.332226858	2.39E-14	postive
METTL17	GABPB1-AS1	0.384744433	4.35E-19	postive
METTL17	GARS-DT	0.413484548	4.54E-22	postive
METTL17	GAS5	0.329021871	4.35E-14	postive
METTL17	GAS5-AS1	0.378183946	1.90E-18	postive
METTL17	GAS8-AS1	0.474673187	1.84E-29	postive
METTL17	GATA2-AS1	0.32079222	1.98E-13	postive
METTL17	GATA6-AS1	0.338683115	6.94E-15	postive
METTL17	GBAP1	0.547339047	2.03E-40	postive
METTL17	GCC2-AS1	0.441732858	2.69E-25	postive
METTL17	GEMIN7-AS1	0.531094421	9.81E-38	postive
METTL17	GGNBP1	0.304329054	3.56E-12	postive
METTL17	GHET1	0.352856498	4.17E-16	postive
METTL17	GHRLOS	0.419488527	9.91E-23	postive
METTL17	GLIDR	0.450695617	2.20E-26	postive
METTL17	GLUD1P2	0.318422778	3.03E-13	postive
METTL17	GLUD1P3	0.43449964	1.93E-24	postive
METTL17	GMDS-DT	0.417661565	1.58E-22	postive
METTL17	GNRHR2	0.577824443	7.14E-46	postive
METTL17	GOLGA2P10	0.359803724	9.98E-17	postive
METTL17	GOLGA2P5	0.480282241	3.24E-30	postive
METTL17	GOLGA6L5P	0.385332249	3.81E-19	postive
METTL17	GPRC5D-AS1	0.405617459	3.18E-21	postive
METTL17	GRHL3-AS1	0.333187952	1.99E-14	postive
METTL17	GRPEL2-AS1	0.321184896	1.84E-13	postive
METTL17	GRTP1-AS1	0.313198573	7.67E-13	postive
METTL17	GS1-124K5.3	0.479686516	3.90E-30	postive
METTL17	GS1-358P8.4	0.35164101	5.34E-16	postive

METTL17	GTF2IP1	0.563166217	3.52E-43	postive
METTL17	GTF2IP13	0.444059117	1.42E-25	postive
METTL17	GTF2IP20	0.542985751	1.10E-39	postive
METTL17	GTF2IP4	0.393068223	6.38E-20	postive
METTL17	GTF3C2-AS1	0.3907569	1.09E-19	postive
METTL17	GTSE1-DT	0.35872166	1.25E-16	postive
METTL17	GUCY1B2	0.440312243	3.98E-25	postive
METTL17	GUSBP1	0.458565878	2.29E-27	postive
METTL17	GUSBP11	0.54043904	2.92E-39	postive
METTL17	GUSBP2	0.454519344	7.37E-27	postive
METTL17	H1FX-AS1	0.493945724	4.09E-32	postive
METTL17	H2AZ2P1	0.495843421	2.19E-32	postive
METTL17	H2BC19P	0.373802336	5.01E-18	postive
METTL17	H2BC20P	0.378000902	1.98E-18	postive
METTL17	H3P37	0.312394026	8.84E-13	postive
METTL17	HCG15	0.338083885	7.80E-15	postive
METTL17	HCG17	0.36393838	4.19E-17	postive
METTL17	HCG18	0.462646946	6.90E-28	postive
METTL17	HCG25	0.619391414	2.68E-54	postive
METTL17	HCG27	0.55594318	6.68E-42	postive
METTL17	HCP5	0.346488287	1.50E-15	postive
METTL17	HDAC11-AS1	0.408010481	1.77E-21	postive
METTL17	HDAC4-AS1	0.300872832	6.39E-12	postive
METTL17	HDHD5-AS1	0.385913389	3.34E-19	postive
METTL17	HEIH	0.481377512	2.30E-30	postive
METTL17	HERC2P2	0.51122526	1.21E-34	postive
METTL17	HERC2P4	0.318984978	2.74E-13	postive
METTL17	HERC2P9	0.461935883	8.52E-28	postive
METTL17	HEXD-IT1	0.458271064	2.49E-27	postive
METTL17	HIPK1-AS1	0.340074144	5.30E-15	postive
METTL17	HM13-IT1	0.570524966	1.63E-44	postive
METTL17	HMGB1P5	0.36684292	2.26E-17	postive
METTL17	HMGN2P46	0.390322057	1.21E-19	postive
METTL17	HMMR-AS1	0.324831664	9.46E-14	postive
METTL17	HNRNPU-AS1	0.393648277	5.57E-20	postive
METTL17	HOXB-AS1	0.372000226	7.42E-18	postive
METTL17	HPN-AS1	0.385045219	4.07E-19	postive
METTL17	HSD17B7P2	0.495921198	2.14E-32	postive
METTL17	HYI-AS1	0.465912769	2.62E-28	postive
METTL17	IBA57-DT	0.52244584	2.30E-36	postive
METTL17	IDH1-AS1	0.398804493	1.65E-20	postive
METTL17	IDI2-AS1	0.364828433	3.47E-17	postive
METTL17	IGBP1-AS1	0.447522077	5.38E-26	postive
METTL17	IL10RB-DT	0.303366008	4.19E-12	postive
METTL17	IL6R-AS1	0.355968321	2.21E-16	postive
METTL17	ILF3-DT	0.53288008	5.05E-38	postive
METTL17	INE1	0.540535127	2.81E-39	postive
METTL17	INHCAP	0.315213107	5.37E-13	postive
METTL17	INTS4P1	0.385864633	3.37E-19	postive
METTL17	INTS4P2	0.339207475	6.27E-15	postive

METTL17	INTS6-AS1	0.486666937	4.30E-31	postive
METTL17	INTS6L-AS1	0.344357302	2.29E-15	postive
METTL17	IPO5P1	0.433745087	2.36E-24	postive
METTL17	IPO9-AS1	0.509694261	2.06E-34	postive
METTL17	IQCH-AS1	0.443286535	1.75E-25	postive
METTL17	IRF1-AS1	0.35742769	1.63E-16	postive
METTL17	ITFG1-AS1	0.330153302	3.52E-14	postive
METTL17	ITFG2-AS1	0.403564934	5.24E-21	postive
METTL17	ITGA9-AS1	0.357922148	1.47E-16	postive
METTL17	ITPR1-DT	0.42689746	1.45E-23	postive
METTL17	JPX	0.435418772	1.51E-24	postive
METTL17	KB-1572G7.2	0.552487404	2.66E-41	postive
METTL17	KCNIP2-AS1	0.453076188	1.11E-26	postive
METTL17	KCNMB2-AS1	0.307417533	2.10E-12	postive
METTL17	KCTD21-AS1	0.440138966	4.17E-25	postive
METTL17	KDM2B-DT	0.411562283	7.33E-22	postive
METTL17	KDM4A-AS1	0.617354535	7.41E-54	postive
METTL17	KDM7A-DT	0.363244144	4.85E-17	postive
METTL17	KIAA1614-AS1	0.316356774	4.39E-13	postive
METTL17	KIF1C-AS1	0.567275601	6.39E-44	postive
METTL17	KIF9-AS1	0.39755318	2.22E-20	postive
METTL17	KLF3-AS1	0.448824129	3.73E-26	postive
METTL17	KLRA1P	0.402292001	7.12E-21	postive
METTL17	KMT2E-AS1	0.568234974	4.27E-44	postive
METTL17	KRT7-AS	0.368762381	1.50E-17	postive
METTL17	KRT8P12	0.470896542	5.84E-29	postive
METTL17	KTN1-AS1	0.442396903	2.24E-25	postive
METTL17	L3MBTL2-AS1	0.466422484	2.25E-28	postive
METTL17	LAMC1-AS1	0.473440107	2.69E-29	postive
METTL17	LAMTOR5-AS1	0.425552922	2.07E-23	postive
METTL17	LBX2-AS1	0.410621963	9.26E-22	postive
METTL17	LCMT1-AS1	0.320496227	2.09E-13	postive
METTL17	LENG8-AS1	0.561106367	8.21E-43	postive
METTL17	LGALS8-AS1	0.425164163	2.29E-23	postive
METTL17	LGALSL-DT	0.410459103	9.65E-22	postive
METTL17	LIFR-AS1	0.360155222	9.27E-17	postive
METTL17	LINC00106	0.531120987	9.71E-38	postive
METTL17	LINC00112	0.31008012	1.32E-12	postive
METTL17	LINC00115	0.612403308	8.55E-53	postive
METTL17	LINC00173	0.440356906	3.93E-25	postive
METTL17	LINC00174	0.566053577	1.06E-43	postive
METTL17	LINC00205	0.46545861	3.00E-28	postive
METTL17	LINC00235	0.370117319	1.12E-17	postive
METTL17	LINC00240	0.329038631	4.34E-14	postive
METTL17	LINC002481	0.316725792	4.11E-13	postive
METTL17	LINC00265	0.507722215	4.05E-34	postive
METTL17	LINC00294	0.362964477	5.14E-17	postive
METTL17	LINC00298	0.305478281	2.93E-12	postive
METTL17	LINC00299	0.300585612	6.70E-12	postive
METTL17	LINC00324	0.310974126	1.13E-12	postive

METTL17	LINC00336	0.339667497	5.74E-15	postive
METTL17	LINC00339	0.49659325	1.71E-32	postive
METTL17	LINC00449	0.350327777	6.96E-16	postive
METTL17	LINC00461	0.390549812	1.15E-19	postive
METTL17	LINC00467	0.419293757	1.04E-22	postive
METTL17	LINC00471	0.458881032	2.08E-27	postive
METTL17	LINC00476	0.497710238	1.18E-32	postive
METTL17	LINC00482	0.407603871	1.95E-21	postive
METTL17	LINC00511	0.317240478	3.75E-13	postive
METTL17	LINC00526	0.442675867	2.08E-25	postive
METTL17	LINC00528	0.345347898	1.88E-15	postive
METTL17	LINC00536	0.304677335	3.36E-12	postive
METTL17	LINC00539	0.36158451	6.87E-17	postive
METTL17	LINC00563	0.345800476	1.72E-15	postive
METTL17	LINC00618	0.404104314	4.59E-21	postive
METTL17	LINC00623	0.367642266	1.90E-17	postive
METTL17	LINC00630	0.395380664	3.71E-20	postive
METTL17	LINC00637	0.386791609	2.73E-19	postive
METTL17	LINC00638	0.383084736	6.34E-19	postive
METTL17	LINC00641	0.424029919	3.07E-23	postive
METTL17	LINC00652	0.341203799	4.26E-15	postive
METTL17	LINC00653	0.554217879	1.34E-41	postive
METTL17	LINC00654	0.38710703	2.54E-19	postive
METTL17	LINC00663	0.462765272	6.67E-28	postive
METTL17	LINC00665	0.334032162	1.69E-14	postive
METTL17	LINC00667	0.479780518	3.79E-30	postive
METTL17	LINC00677	0.381297107	9.49E-19	postive
METTL17	LINC00680	0.504565559	1.19E-33	postive
METTL17	LINC00685	0.496722826	1.64E-32	postive
METTL17	LINC00847	0.469879985	7.95E-29	postive
METTL17	LINC00852	0.349371188	8.44E-16	postive
METTL17	LINC00853	0.336056919	1.15E-14	postive
METTL17	LINC00858	0.326195646	7.36E-14	postive
METTL17	LINC00863	0.328583993	4.72E-14	postive
METTL17	LINC00865	0.304342512	3.55E-12	postive
METTL17	LINC00869	0.397916956	2.03E-20	postive
METTL17	LINC00884	0.385936976	3.32E-19	postive
METTL17	LINC00888	0.378630401	1.72E-18	postive
METTL17	LINC00893	0.492058938	7.56E-32	postive
METTL17	LINC00894	0.403341348	5.53E-21	postive
METTL17	LINC00896	0.39845595	1.79E-20	postive
METTL17	LINC00899	0.554607518	1.14E-41	postive
METTL17	LINC00909	0.590841983	2.22E-48	postive
METTL17	LINC00910	0.45176469	1.62E-26	postive
METTL17	LINC00921	0.548529403	1.27E-40	postive
METTL17	LINC00937	0.339619768	5.79E-15	postive
METTL17	LINC00950	0.343065933	2.96E-15	postive
METTL17	LINC00954	0.337236109	9.18E-15	postive
METTL17	LINC00957	0.431262745	4.59E-24	postive
METTL17	LINC00963	0.479277941	4.44E-30	postive

METTL17	LINC01001	0.571649361	1.01E-44	postive
METTL17	LINC01003	0.350897373	6.21E-16	postive
METTL17	LINC01004	0.50486098	1.07E-33	postive
METTL17	LINC01011	0.580945937	1.83E-46	postive
METTL17	LINC01023	0.424677593	2.60E-23	postive
METTL17	LINC01089	0.621312437	1.02E-54	postive
METTL17	LINC01106	0.330439053	3.34E-14	postive
METTL17	LINC01122	0.329694709	3.84E-14	postive
METTL17	LINC01123	0.340752253	4.65E-15	postive
METTL17	LINC01126	0.399533832	1.38E-20	postive
METTL17	LINC01128	0.424918458	2.44E-23	postive
METTL17	LINC01132	0.398030018	1.98E-20	postive
METTL17	LINC01135	0.352062359	4.90E-16	postive
METTL17	LINC01137	0.308666175	1.69E-12	postive
METTL17	LINC01138	0.437261298	9.15E-25	postive
METTL17	LINC01144	0.438710038	6.17E-25	postive
METTL17	LINC01145	0.511985	9.30E-35	postive
METTL17	LINC01176	0.50216445	2.67E-33	postive
METTL17	LINC01184	0.410615345	9.28E-22	postive
METTL17	LINC01220	0.377320296	2.31E-18	postive
METTL17	LINC01237	0.319458889	2.52E-13	postive
METTL17	LINC01238	0.366785689	2.28E-17	postive
METTL17	LINC01270	0.403307436	5.57E-21	postive
METTL17	LINC01271	0.452228792	1.42E-26	postive
METTL17	LINC01273	0.388427944	1.87E-19	postive
METTL17	LINC01276	0.308833963	1.64E-12	postive
METTL17	LINC01278	0.479813751	3.75E-30	postive
METTL17	LINC01285	0.317024774	3.89E-13	postive
METTL17	LINC01311	0.570183214	1.88E-44	postive
METTL17	LINC01337	0.394346297	4.73E-20	postive
METTL17	LINC01342	0.375566544	3.40E-18	postive
METTL17	LINC01347	0.321951238	1.60E-13	postive
METTL17	LINC01353	0.323030005	1.32E-13	postive
METTL17	LINC01355	0.510083545	1.80E-34	postive
METTL17	LINC01372	0.603288986	6.90E-51	postive
METTL17	LINC01409	0.347856719	1.14E-15	postive
METTL17	LINC01410	0.377623892	2.16E-18	postive
METTL17	LINC01424	0.470456102	6.68E-29	postive
METTL17	LINC01431	0.374198638	4.59E-18	postive
METTL17	LINC01507	0.337751741	8.31E-15	postive
METTL17	LINC01521	0.394286734	4.80E-20	postive
METTL17	LINC01534	0.449118045	3.43E-26	postive
METTL17	LINC01560	0.489370779	1.81E-31	postive
METTL17	LINC01562	0.372431652	6.75E-18	postive
METTL17	LINC01569	0.492510643	6.53E-32	postive
METTL17	LINC01572	0.306290659	2.55E-12	postive
METTL17	LINC01578	0.314000027	6.66E-13	postive
METTL17	LINC01588	0.470094484	7.45E-29	postive
METTL17	LINC01644	0.334583742	1.53E-14	postive
METTL17	LINC01686	0.372057745	7.33E-18	postive

METTL17	LINC01703	0.401469273	8.69E-21	postive
METTL17	LINC01715	0.411147054	8.13E-22	postive
METTL17	LINC01770	0.513261075	5.97E-35	postive
METTL17	LINC01772	0.504302728	1.30E-33	postive
METTL17	LINC01786	0.510626394	1.49E-34	postive
METTL17	LINC01800	0.302885681	4.55E-12	postive
METTL17	LINC01816	0.313482271	7.30E-13	postive
METTL17	LINC01881	0.337592496	8.57E-15	postive
METTL17	LINC01909	0.449006311	3.54E-26	postive
METTL17	LINC01952	0.365809084	2.81E-17	postive
METTL17	LINC01972	0.333457405	1.89E-14	postive
METTL17	LINC01977	0.337933013	8.03E-15	postive
METTL17	LINC02014	0.374210793	4.58E-18	postive
METTL17	LINC02019	0.360495011	8.63E-17	postive
METTL17	LINC02021	0.553670264	1.66E-41	postive
METTL17	LINC02026	0.356214893	2.10E-16	postive
METTL17	LINC02035	0.31951545	2.49E-13	postive
METTL17	LINC02062	0.461189316	1.06E-27	postive
METTL17	LINC02076	0.426151924	1.77E-23	postive
METTL17	LINC02099	0.310918989	1.14E-12	postive
METTL17	LINC02193	0.384413884	4.69E-19	postive
METTL17	LINC02210	0.479166737	4.59E-30	postive
METTL17	LINC02235	0.313336687	7.49E-13	postive
METTL17	LINC02280	0.358894037	1.21E-16	postive
METTL17	LINC02298	0.325919407	7.74E-14	postive
METTL17	LINC02315	0.30040695	6.91E-12	postive
METTL17	LINC02352	0.390909399	1.06E-19	postive
METTL17	LINC02361	0.321579785	1.71E-13	postive
METTL17	LINC02363	0.346274718	1.57E-15	postive
METTL17	LINC02427	0.301942816	5.34E-12	postive
METTL17	LINC02435	0.307125098	2.21E-12	postive
METTL17	LINC02449	0.337948058	8.00E-15	postive
METTL17	LINC02482	0.34061474	4.77E-15	postive
METTL17	LINC02585	0.403946326	4.77E-21	postive
METTL17	LINC02601	0.351883579	5.08E-16	postive
METTL17	LINC02604	0.587885044	8.43E-48	postive
METTL17	LINC02611	0.30779067	1.97E-12	postive
METTL17	LINC02614	0.325275336	8.72E-14	postive
METTL17	LINC02615	0.328889226	4.46E-14	postive
METTL17	LINC02637	0.395730052	3.41E-20	postive
METTL17	LINC02693	0.348198327	1.07E-15	postive
METTL17	LINC02709	0.311849807	9.72E-13	postive
METTL17	LINC02739	0.308757338	1.67E-12	postive
METTL17	LINC02777	0.309870922	1.37E-12	postive
METTL17	LINC02803	0.41107151	8.29E-22	postive
METTL17	LINC02818	0.313636716	7.10E-13	postive
METTL17	LINC02851	0.389009685	1.64E-19	postive
METTL17	LIX1L-AS1	0.389097352	1.61E-19	postive
METTL17	LMCD1-AS1	0.318491183	2.99E-13	postive
METTL17	LMNTD2-AS1	0.380379598	1.17E-18	postive

METTL17	LNCTAM34A	0.435033373	1.67E-24	postive
METTL17	LOH12CR2	0.377870922	2.04E-18	postive
METTL17	LOXL1-AS1	0.363602721	4.49E-17	postive
METTL17	LPP-AS2	0.401155076	9.37E-21	postive
METTL17	LRRC37A17P	0.421399232	6.07E-23	postive
METTL17	LRRC37BP1	0.522669223	2.13E-36	postive
METTL17	MAGOH2P	0.422722505	4.31E-23	postive
METTL17	MAN1B1-DT	0.423858012	3.21E-23	postive
METTL17	MANEA-DT	0.35936754	1.09E-16	postive
METTL17	MAP3K14-AS1	0.440810483	3.47E-25	postive
METTL17	MAP3K2-DT	0.311763639	9.87E-13	postive
METTL17	MAP4K3-DT	0.402144751	7.38E-21	postive
METTL17	MAPK6-DT	0.358410817	1.33E-16	postive
METTL17	MAPKAPK5-AS1	0.589055735	4.98E-48	postive
METTL17	MATN1-AS1	0.536970684	1.09E-38	postive
METTL17	MBL1P	0.333991815	1.71E-14	postive
METTL17	MCCC1-AS1	0.432082532	3.69E-24	postive
METTL17	MCM3AP-AS1	0.529778377	1.59E-37	postive
METTL17	MCPH1-AS1	0.33153298	2.72E-14	postive
METTL17	MDS2	0.325351698	8.60E-14	postive
METTL17	MED8-AS1	0.557914434	3.01E-42	postive
METTL17	MELTF-AS1	0.486306031	4.83E-31	postive
METTL17	METTL14-DT	0.346384312	1.53E-15	postive
METTL17	MHENCR	0.514717761	3.58E-35	postive
METTL17	MIATNB	0.412555105	5.72E-22	postive
METTL17	MIF-AS1	0.399483912	1.40E-20	postive
METTL17	MINCR	0.377392652	2.27E-18	postive
METTL17	MIPEPP3	0.488340072	2.52E-31	postive
METTL17	MIR200CHG	0.359293855	1.11E-16	postive
METTL17	MIR210HG	0.477529842	7.64E-30	postive
METTL17	MIR22HG	0.36441718	3.78E-17	postive
METTL17	MIR34AHG	0.452655361	1.26E-26	postive
METTL17	MIR3936HG	0.500230581	5.11E-33	postive
METTL17	MIR4453HG	0.479825966	3.74E-30	postive
METTL17	MIR497HG	0.324989995	9.19E-14	postive
METTL17	MIR503HG	0.340439709	4.94E-15	postive
METTL17	MIR600HG	0.468320987	1.27E-28	postive
METTL17	MIR762HG	0.580971949	1.81E-46	postive
METTL17	MIS18A-AS1	0.45961261	1.68E-27	postive
METTL17	MKLN1-AS	0.353731559	3.49E-16	postive
METTL17	MKNK1-AS1	0.395698235	3.44E-20	postive
METTL17	MMADHC-DT	0.310473803	1.24E-12	postive
METTL17	MMP25-AS1	0.469434523	9.10E-29	postive
METTL17	MNX1-AS2	0.33867534	6.95E-15	postive
METTL17	MORF4L2-AS1	0.532540458	5.73E-38	postive
METTL17	MROCKI	0.392042845	8.11E-20	postive
METTL17	MRPL20-AS1	0.574400436	3.13E-45	postive
METTL17	MRPL45P2	0.516608699	1.84E-35	postive
METTL17	MRPS31P4	0.419374679	1.02E-22	postive
METTL17	MRPS31P5	0.433998629	2.21E-24	postive

METTL17	MRPS9-AS1	0.405260791	3.47E-21	postive
METTL17	MSH5-SAPCD1	0.492802498	5.94E-32	postive
METTL17	MTMR9LP	0.576630682	1.20E-45	postive
METTL17	MUC20-OT1	0.549954434	7.26E-41	postive
METTL17	MYLK-AS1	0.42525749	2.23E-23	postive
METTL17	MZF1-AS1	0.476994506	9.02E-30	postive
METTL17	NACA4P	0.364067436	4.07E-17	postive
METTL17	NALT1	0.527416511	3.80E-37	postive
METTL17	NAPA-AS1	0.350624292	6.56E-16	postive
METTL17	NARF-AS1	0.459263395	1.86E-27	postive
METTL17	NARF-IT1	0.459013179	2.01E-27	postive
METTL17	NBPF25P	0.47420227	2.13E-29	postive
METTL17	NBPF8	0.589599685	3.89E-48	postive
METTL17	NBR2	0.541801628	1.73E-39	postive
METTL17	NCBP2-AS1	0.52495589	9.30E-37	postive
METTL17	NCK1-DT	0.348006417	1.11E-15	postive
METTL17	NDUFA6-DT	0.549837989	7.60E-41	postive
METTL17	NDUFB2-AS1	0.541148916	2.22E-39	postive
METTL17	NDUFV2-AS1	0.493011338	5.54E-32	postive
METTL17	NEBL-AS1	0.318478434	3.00E-13	postive
METTL17	NFYC-AS1	0.550167903	6.68E-41	postive
METTL17	NIFK-AS1	0.573235631	5.15E-45	postive
METTL17	NINJ2-AS1	0.358880564	1.21E-16	postive
METTL17	NKAPP1	0.395899018	3.28E-20	postive
METTL17	NKX2-1-AS1	0.317579118	3.53E-13	postive
METTL17	NNT-AS1	0.379852909	1.31E-18	postive
METTL17	NOP14-AS1	0.495920832	2.14E-32	postive
METTL17	NORAD	0.316075231	4.61E-13	postive
METTL17	NPEPPSP1	0.367276653	2.06E-17	postive
METTL17	NPIPB14P	0.491819165	8.18E-32	postive
METTL17	NPIPB1P	0.439114169	5.53E-25	postive
METTL17	NPIPP1	0.546422064	2.90E-40	postive
METTL17	NPPA-AS1	0.412429363	5.91E-22	postive
METTL17	NRADDP	0.381662549	8.74E-19	postive
METTL17	NRAV	0.40379792	4.95E-21	postive
METTL17	NRSN2-AS1	0.543976934	7.49E-40	postive
METTL17	NSMCE1-DT	0.516487814	1.92E-35	postive
METTL17	NSUN5P1	0.68288452	6.43E-70	postive
METTL17	NSUN5P2	0.670804648	1.20E-66	postive
METTL17	NUCB1-AS1	0.311068799	1.11E-12	postive
METTL17	NUDT16P1	0.368820495	1.48E-17	postive
METTL17	NUP50-DT	0.460494666	1.30E-27	postive
METTL17	NUTM2A-AS1	0.390468539	1.17E-19	postive
METTL17	NUTM2B-AS1	0.305518192	2.91E-12	postive
METTL17	NXT1-AS1	0.472182024	3.95E-29	postive
METTL17	OBI1-AS1	0.409408989	1.25E-21	postive
METTL17	OCIAD1-AS1	0.404885884	3.80E-21	postive
METTL17	ODF2-AS1	0.36827562	1.66E-17	postive
METTL17	OGFR-AS1	0.541590588	1.88E-39	postive
METTL17	OGFRP1	0.390804946	1.08E-19	postive

METTL17	OIP5-AS1	0.344387283	2.28E-15	postive
METTL17	OLMALINC	0.492442658	6.67E-32	postive
METTL17	OR2A1-AS1	0.386454571	2.95E-19	postive
METTL17	OR2A9P	0.404446933	4.23E-21	postive
METTL17	OSER1-DT	0.430715334	5.31E-24	postive
METTL17	OSGEPL1-AS1	0.465596233	2.88E-28	postive
METTL17	OTUD6B-AS1	0.399104565	1.53E-20	postive
METTL17	OXCT1-AS1	0.322144914	1.55E-13	postive
METTL17	PAN3-AS1	0.527557587	3.60E-37	postive
METTL17	PARD3-AS1	0.344125746	2.40E-15	postive
METTL17	PARD6G-AS1	0.385895528	3.35E-19	postive
METTL17	PARGP1	0.390885306	1.06E-19	postive
METTL17	PAXBP1-AS1	0.399262157	1.48E-20	postive
METTL17	PAXIP1-AS1	0.547986627	1.57E-40	postive
METTL17	PAXIP1-AS2	0.461426121	9.89E-28	postive
METTL17	PCAT6	0.396673143	2.73E-20	postive
METTL17	PCBP1-AS1	0.571603396	1.03E-44	postive
METTL17	PCCA-DT	0.336864612	9.86E-15	postive
METTL17	PCOLCE-AS1	0.319531128	2.48E-13	postive
METTL17	PCOTH	0.328882195	4.47E-14	postive
METTL17	PDCD4-AS1	0.362807847	5.31E-17	postive
METTL17	PDIA3P1	0.413060367	5.04E-22	postive
METTL17	PDPK2P	0.300182423	7.17E-12	postive
METTL17	PDZK1P1	0.334758254	1.48E-14	postive
METTL17	PEF1-AS1	0.421498657	5.92E-23	postive
METTL17	PGAM1P8	0.416977186	1.88E-22	postive
METTL17	PI4KAP1	0.348907112	9.27E-16	postive
METTL17	PI4KAP2	0.505226931	9.49E-34	postive
METTL17	PIK3IP1-AS1	0.489710398	1.62E-31	postive
METTL17	PINK1-AS	0.383680651	5.54E-19	postive
METTL17	PIPSL	0.32829577	4.99E-14	postive
METTL17	PITPNA-AS1	0.458792033	2.14E-27	postive
METTL17	PITRM1-AS1	0.447940573	4.79E-26	postive
METTL17	PKD1P6	0.557032006	4.31E-42	postive
METTL17	PLA2G4C-AS1	0.330886509	3.07E-14	postive
METTL17	PLEKHA8P1	0.357313665	1.67E-16	postive
METTL17	PLEKHM1P1	0.448055488	4.63E-26	postive
METTL17	PMS2CL	0.561400418	7.28E-43	postive
METTL17	PMS2P3	0.571471676	1.09E-44	postive
METTL17	PMS2P4	0.418529492	1.27E-22	postive
METTL17	POLH-AS1	0.408067373	1.74E-21	postive
METTL17	POLR2J4	0.587231378	1.13E-47	postive
METTL17	PPIEL	0.324001131	1.10E-13	postive
METTL17	PPP1R26-AS1	0.499483762	6.56E-33	postive
METTL17	PPP1R35-AS1	0.37736508	2.28E-18	postive
METTL17	PPP3CB-AS1	0.464213237	4.34E-28	postive
METTL17	PPP4R1L	0.361563493	6.90E-17	postive
METTL17	PPT2-EGFL8	0.670037778	1.92E-66	postive
METTL17	PRC1-AS1	0.432577372	3.23E-24	postive
METTL17	PRELID1P1	0.331515753	2.73E-14	postive

METTL17	PRKAG2-AS1	0.378572022	1.75E-18	postive
METTL17	PRKAR2A-AS1	0.442896975	1.95E-25	postive
METTL17	PRKCZ-AS1	0.474591344	1.89E-29	postive
METTL17	PRMT5-AS1	0.763668416	1.08E-96	postive
METTL17	PRORSD1P	0.621730726	8.23E-55	postive
METTL17	PRR34	0.300159604	7.20E-12	postive
METTL17	PRR7-AS1	0.333624744	1.83E-14	postive
METTL17	PRSS42P	0.396289328	2.99E-20	postive
METTL17	PRSS46P	0.316966397	3.93E-13	postive
METTL17	PSMA3-AS1	0.645478101	2.88E-60	postive
METTL17	PSMB8-AS1	0.422392819	4.70E-23	postive
METTL17	PSPC1-AS2	0.410057463	1.07E-21	postive
METTL17	PTMAP5	0.306473736	2.47E-12	postive
METTL17	PTOV1-AS1	0.542653715	1.25E-39	postive
METTL17	PTOV1-AS2	0.625631332	1.13E-55	postive
METTL17	PTPRVP	0.483077599	1.35E-30	postive
METTL17	PXN-AS1	0.539117393	4.83E-39	postive
METTL17	RAB11B-AS1	0.332493345	2.27E-14	postive
METTL17	RAB30-DT	0.455163575	6.12E-27	postive
METTL17	RABGAP1L-DT	0.305359425	2.99E-12	postive
METTL17	RAD51-AS1	0.613029821	6.29E-53	postive
METTL17	RAET1K	0.364564923	3.67E-17	postive
METTL17	RALY-AS1	0.359332538	1.10E-16	postive
METTL17	RAP2C-AS1	0.354315808	3.10E-16	postive
METTL17	RARA-AS1	0.402858337	6.21E-21	postive
METTL17	RASA4CP	0.42196439	5.25E-23	postive
METTL17	RASGRF2-AS1	0.310231035	1.29E-12	postive
METTL17	RASSF10-DT	0.327202271	6.11E-14	postive
METTL17	RASSF1-AS1	0.49102384	1.06E-31	postive
METTL17	RASSF8-AS1	0.30123294	6.01E-12	postive
METTL17	RBFADN	0.333725161	1.80E-14	postive
METTL17	RBM15-AS1	0.45522798	6.01E-27	postive
METTL17	RBM26-AS1	0.414619692	3.41E-22	postive
METTL17	RBM5-AS1	0.397541242	2.22E-20	postive
METTL17	RDM1P5	0.401177426	9.32E-21	postive
METTL17	RFPL3S	0.380586819	1.11E-18	postive
METTL17	RGMB-AS1	0.307009222	2.25E-12	postive
METTL17	RHOA-IT1	0.320420821	2.11E-13	postive
METTL17	RHPN1-AS1	0.398644497	1.71E-20	postive
METTL17	RN7SL832P	0.375237034	3.65E-18	postive
METTL17	RNASEH1-AS1	0.420143017	8.38E-23	postive
METTL17	RNF139-AS1	0.493621088	4.54E-32	postive
METTL17	RNF157-AS1	0.344905014	2.06E-15	postive
METTL17	RNF216P1	0.481973088	1.91E-30	postive
METTL17	ROCK1P1	0.405022518	3.67E-21	postive
METTL17	RP11-101E13.5	0.419323104	1.03E-22	postive
METTL17	RP11-104N10.2	0.433962361	2.23E-24	postive
METTL17	RP11-1055B8.9	0.327681997	5.59E-14	postive
METTL17	RP11-10A14.4	0.324762521	9.58E-14	postive
METTL17	RP11-10C24.1	0.320864854	1.95E-13	postive

METTL17	RP11-10C24.2	0.328330852	4.95E-14	postive
METTL17	RP11-111M22.4	0.459767639	1.61E-27	postive
METTL17	RP11-111M22.5	0.396362202	2.94E-20	postive
METTL17	RP11-141C7.4	0.43336862	2.62E-24	postive
METTL17	RP11-153F5.7	0.394131327	4.97E-20	postive
METTL17	RP11-15H20.6	0.355924269	2.23E-16	postive
METTL17	RP11-165J3.6	0.312120152	9.27E-13	postive
METTL17	RP11-175O19.4	0.360653416	8.35E-17	postive
METTL17	RP11-188P20.3	0.343100735	2.94E-15	postive
METTL17	RP11-192H23.8	0.41355993	4.45E-22	postive
METTL17	RP11-199F11.2	0.542895257	1.14E-39	postive
METTL17	RP11-206L10.3	0.335689463	1.23E-14	postive
METTL17	RP11-206L10.5	0.407274351	2.12E-21	postive
METTL17	RP11-226L15.5	0.438202574	7.09E-25	postive
METTL17	RP1-122P22.2	0.312434464	8.77E-13	postive
METTL17	RP11-22P4.1	0.379557476	1.40E-18	postive
METTL17	RP11-254F7.4	0.376614299	2.70E-18	postive
METTL17	RP11-258C19.7	0.552727479	2.42E-41	postive
METTL17	RP11-268J15.5	0.372035177	7.36E-18	postive
METTL17	RP11-282O18.3	0.377044179	2.45E-18	postive
METTL17	RP11-2B6.3	0.374057901	4.73E-18	postive
METTL17	RP11-314C16.1	0.378784877	1.67E-18	postive
METTL17	RP11-346C20.4	0.417906944	1.48E-22	postive
METTL17	RP11-376M2.2	0.309748799	1.40E-12	postive
METTL17	RP11-394O4.5	0.355432571	2.46E-16	postive
METTL17	RP11-429J17.8	0.308181697	1.84E-12	postive
METTL17	RP11-434B12.1	0.393560569	5.69E-20	postive
METTL17	RP11-435O5.2	0.462064782	8.20E-28	postive
METTL17	RP11-455O6.9	0.444390988	1.29E-25	postive
METTL17	RP11-465N4.5	0.613931731	4.04E-53	postive
METTL17	RP11-492E3.51	0.460618274	1.25E-27	postive
METTL17	RP11-508N22.13	0.303324089	4.22E-12	postive
METTL17	RP11-529E10.6	0.398756269	1.66E-20	postive
METTL17	RP11-539I5.1	0.318678124	2.90E-13	postive
METTL17	RP11-540B6.6	0.660058712	7.29E-64	postive
METTL17	RP11-562A8.4	0.399855824	1.28E-20	postive
METTL17	RP11-562A8.5	0.368678305	1.52E-17	postive
METTL17	RP11-58E21.4	0.340783739	4.62E-15	postive
METTL17	RP11-658F2.8	0.544345404	6.50E-40	postive
METTL17	RP11-69E11.4	0.42593401	1.87E-23	postive
METTL17	RP11-706O15.1	0.449994421	2.68E-26	postive
METTL17	RP11-876N24.4	0.472099416	4.05E-29	postive
METTL17	RP11-876N24.5	0.367224119	2.08E-17	postive
METTL17	RP11-932O9.10	0.341401681	4.09E-15	postive
METTL17	RP11-97O12.6	0.470794815	6.03E-29	postive
METTL17	RP11-98G7.1	0.308507857	1.74E-12	postive
METTL17	RP1-199J3.7	0.389176114	1.58E-19	postive
METTL17	RP13-36G14.4	0.342853218	3.08E-15	postive
METTL17	RP13-476E20.1	0.472011759	4.16E-29	postive
METTL17	RP3-323A16.1	0.305317777	3.01E-12	postive

METTL17	RP3-368A4.5	0.328674974	4.65E-14	postive
METTL17	RP3-461P17.10	0.430612065	5.46E-24	postive
METTL17	RP3-486I3.7	0.312372473	8.87E-13	postive
METTL17	RP4-545L17.11	0.398456137	1.79E-20	postive
METTL17	RP4-614O4.12	0.300270436	7.07E-12	postive
METTL17	RP5-1039K5.19	0.456724255	3.90E-27	postive
METTL17	RP5-1112D6.4	0.510085236	1.80E-34	postive
METTL17	RP5-1125A11.6	0.342152711	3.54E-15	postive
METTL17	RP5-1125A11.7	0.393211962	6.17E-20	postive
METTL17	RP9P	0.36735435	2.02E-17	postive
METTL17	RPARP-AS1	0.536992768	1.08E-38	postive
METTL17	RPL12P25	0.45469981	7.00E-27	postive
METTL17	RPL22P19	0.352229069	4.74E-16	postive
METTL17	RPL23AP53	0.460515434	1.29E-27	postive
METTL17	RPL23AP7	0.374221162	4.57E-18	postive
METTL17	RPL23AP82	0.416552483	2.09E-22	postive
METTL17	RPL32P3	0.561913707	5.90E-43	postive
METTL17	RPL6P27	0.38284494	6.70E-19	postive
METTL17	RPLP1P6	0.333756116	1.79E-14	postive
METTL17	RPP38-DT	0.381064485	1.00E-18	postive
METTL17	RPS10P7	0.458110455	2.61E-27	postive
METTL17	RPS18P9	0.316254057	4.47E-13	postive
METTL17	RPS4XP16	0.453432347	1.01E-26	postive
METTL17	RPSAP69	0.373209778	5.70E-18	postive
METTL17	RRN3P1	0.520809713	4.14E-36	postive
METTL17	RRN3P3	0.497608006	1.22E-32	postive
METTL17	RRP7BP	0.613593093	4.77E-53	postive
METTL17	RTCA-AS1	0.381372383	9.33E-19	postive
METTL17	RUSC1-AS1	0.656271513	6.55E-63	postive
METTL17	SAP30-DT	0.446519624	7.13E-26	postive
METTL17	SBDSP1	0.445650721	9.09E-26	postive
METTL17	SBF2-AS1	0.364361159	3.83E-17	postive
METTL17	SBNO1-AS1	0.441159551	3.15E-25	postive
METTL17	SCAMP1-AS1	0.494273247	3.67E-32	postive
METTL17	SCAND2P	0.566405485	9.19E-44	postive
METTL17	SCAT2	0.458287967	2.48E-27	postive
METTL17	SCGB1B2P	0.341429425	4.07E-15	postive
METTL17	SDAD1P1	0.391128345	1.00E-19	postive
METTL17	SDCBP2-AS1	0.462058778	8.21E-28	postive
METTL17	SDHAP1	0.643136607	1.05E-59	postive
METTL17	SEC22B4P	0.316585144	4.21E-13	postive
METTL17	SEC24B-AS1	0.486110369	5.14E-31	postive
METTL17	SEMA3F-AS1	0.623778133	2.91E-55	postive
METTL17	SEPSECS-AS1	0.398240525	1.88E-20	postive
METTL17	SEPTIN4-AS1	0.44502145	1.08E-25	postive
METTL17	SEPTIN7-AS1	0.383069733	6.37E-19	postive
METTL17	SEPTIN7P2	0.58880921	5.56E-48	postive
METTL17	SEPTIN7P9	0.351092154	5.97E-16	postive
METTL17	SERHL	0.459475767	1.75E-27	postive
METTL17	SERTAD4-AS1	0.32152784	1.73E-13	postive

METTL17	SGMS1-AS1	0.323418761	1.23E-13	postive
METTL17	SH3BP5-AS1	0.513861998	4.84E-35	postive
METTL17	SH3GL1P1	0.558607991	2.27E-42	postive
METTL17	SIAH2-AS1	0.347839881	1.15E-15	postive
METTL17	SLC12A9-AS1	0.44223701	2.34E-25	postive
METTL17	SLC25A25-AS1	0.375942506	3.13E-18	postive
METTL17	SLC25A30-AS1	0.423970834	3.12E-23	postive
METTL17	SLC25A5-AS1	0.476260704	1.13E-29	postive
METTL17	SLC25A5P1	0.51290408	6.76E-35	postive
METTL17	SLC2A1-AS1	0.309316291	1.51E-12	postive
METTL17	SLC44A3-AS1	0.371418726	8.42E-18	postive
METTL17	SLC9A3-AS1	0.405700382	3.11E-21	postive
METTL17	SLFNL1-AS1	0.319761505	2.38E-13	postive
METTL17	SLX1A-SULT1A3	0.514004307	4.60E-35	postive
METTL17	SMC5-AS1	0.315320433	5.27E-13	postive
METTL17	SMG1P7	0.473099169	2.99E-29	postive
METTL17	SMG7-AS1	0.491642253	8.66E-32	postive
METTL17	SNAI3-AS1	0.492230383	7.15E-32	postive
METTL17	SNHG1	0.576872588	1.08E-45	postive
METTL17	SNHG10	0.634396776	1.16E-57	postive
METTL17	SNHG11	0.668243236	5.67E-66	postive
METTL17	SNHG12	0.642887297	1.20E-59	postive
METTL17	SNHG15	0.423499791	3.53E-23	postive
METTL17	SNHG16	0.316545671	4.24E-13	postive
METTL17	SNHG17	0.488126333	2.70E-31	postive
METTL17	SNHG20	0.621038056	1.17E-54	postive
METTL17	SNHG21	0.525099194	8.83E-37	postive
METTL17	SNHG25	0.353147533	3.93E-16	postive
METTL17	SNHG28	0.4843373	9.03E-31	postive
METTL17	SNHG3	0.486024239	5.28E-31	postive
METTL17	SNHG30	0.447252233	5.81E-26	postive
METTL17	SNHG32	0.452582472	1.28E-26	postive
METTL17	SNHG4	0.446918279	6.38E-26	postive
METTL17	SNHG6	0.31239928	8.83E-13	postive
METTL17	SNHG7	0.444030628	1.43E-25	postive
METTL17	SNHG8	0.37158464	8.12E-18	postive
METTL17	SNHG9	0.304951984	3.20E-12	postive
METTL17	SNRK-AS1	0.321063128	1.88E-13	postive
METTL17	SOX2-OT	0.323989831	1.10E-13	postive
METTL17	SPACA6P-AS	0.415307253	2.87E-22	postive
METTL17	SPAG5-AS1	0.592894444	8.71E-49	postive
METTL17	SPART-AS1	0.323631842	1.18E-13	postive
METTL17	SPATA41	0.346562638	1.48E-15	postive
METTL17	SPINT1-AS1	0.320205083	2.20E-13	postive
METTL17	SREBF2-AS1	0.559845581	1.37E-42	postive
METTL17	SRP14-AS1	0.481864408	1.97E-30	postive
METTL17	SRP54-AS1	0.430510937	5.60E-24	postive
METTL17	SRRM2-AS1	0.46649338	2.20E-28	postive
METTL17	SSBP3-AS1	0.495247374	2.67E-32	postive
METTL17	SSR4P1	0.411832114	6.86E-22	postive

METTL17	ST20-AS1	0.368372763	1.63E-17	postive
METTL17	ST3GAL6-AS1	0.329547111	3.95E-14	postive
METTL17	STAG3L1	0.456838276	3.77E-27	postive
METTL17	STAG3L2	0.603952079	5.04E-51	postive
METTL17	STAG3L3	0.602687483	9.18E-51	postive
METTL17	STAG3L4	0.453939077	8.71E-27	postive
METTL17	STAG3L5P	0.6251766	1.42E-55	postive
METTL17	STAG3L5P-PVRIG2P-PILRB	0.646466209	1.67E-60	postive
METTL17	STAM-AS1	0.533722042	3.69E-38	postive
METTL17	STARD7-AS1	0.58006558	2.69E-46	postive
METTL17	STK4-AS1	0.344888504	2.06E-15	postive
METTL17	STPG3-AS1	0.47583644	1.29E-29	postive
METTL17	STRA6LP	0.326814701	6.56E-14	postive
METTL17	STT3A-AS1	0.323360798	1.24E-13	postive
METTL17	STX17-AS1	0.333442951	1.89E-14	postive
METTL17	STX18-AS1	0.504641474	1.16E-33	postive
METTL17	SUCLA2-AS1	0.437716657	8.09E-25	postive
METTL17	SUGT1P4-STRA6LP	0.440659766	3.62E-25	postive
METTL17	SUMO2P17	0.423425955	3.60E-23	postive
METTL17	SUZ12P1	0.599759161	3.64E-50	postive
METTL17	SVIL-AS1	0.338566592	7.10E-15	postive
METTL17	SYNGAP1-AS1	0.379980924	1.28E-18	postive
METTL17	SYS1-DBNDD2	0.341424553	4.08E-15	postive
METTL17	SZT2-AS1	0.431338945	4.50E-24	postive
METTL17	TAF1A-AS1	0.374889633	3.94E-18	postive
METTL17	TAPT1-AS1	0.507195482	4.85E-34	postive
METTL17	TBC1D8-AS1	0.399319495	1.46E-20	postive
METTL17	TBILA	0.305076775	3.14E-12	postive
METTL17	TDRKH-AS1	0.367931035	1.79E-17	postive
METTL17	TEKT4P2	0.370663836	9.92E-18	postive
METTL17	TEX21P	0.302853283	4.57E-12	postive
METTL17	TFAP2A-AS1	0.436754415	1.05E-24	postive
METTL17	THAP7-AS1	0.357493242	1.61E-16	postive
METTL17	THAP9-AS1	0.538169868	6.92E-39	postive
METTL17	THRB-AS1	0.312407678	8.82E-13	postive
METTL17	THUMPD3-AS1	0.53038239	1.28E-37	postive
METTL17	TIPARP-AS1	0.417440812	1.67E-22	postive
METTL17	TMC3-AS1	0.394026416	5.10E-20	postive
METTL17	TMCC1-AS1	0.432427629	3.36E-24	postive
METTL17	TMCO1-AS1	0.313225476	7.64E-13	postive
METTL17	TMED2-DT	0.612422354	8.48E-53	postive
METTL17	TMEM147-AS1	0.57981268	3.01E-46	postive
METTL17	TMEM161B-AS1	0.453583413	9.64E-27	postive
METTL17	TMEM198B	0.600208546	2.95E-50	postive
METTL17	TMEM202-AS1	0.399565339	1.37E-20	postive
METTL17	TMEM220-AS1	0.30056983	6.72E-12	postive
METTL17	TMEM231P1	0.354081473	3.25E-16	postive
METTL17	TMEM44-AS1	0.525034287	9.04E-37	postive
METTL17	TMEM51-AS1	0.339571457	5.85E-15	postive
METTL17	TMEM9B-AS1	0.379540773	1.41E-18	postive

METTL17	TMPO-AS1	0.387981593	2.08E-19	postive
METTL17	TNFRSF14-AS1	0.408602573	1.53E-21	postive
METTL17	TNK2-AS1	0.394491837	4.57E-20	postive
METTL17	TNKS2-AS1	0.365632672	2.92E-17	postive
METTL17	TNRC6C-AS1	0.406932198	2.30E-21	postive
METTL17	TOB1-AS1	0.485319906	6.61E-31	postive
METTL17	TOLLIP-AS1	0.429166871	8.00E-24	postive
METTL17	TONSL-AS1	0.501900409	2.92E-33	postive
METTL17	TP53TG1	0.319497325	2.50E-13	postive
METTL17	TP73-AS1	0.589769809	3.61E-48	postive
METTL17	TPI1P2	0.323935334	1.12E-13	postive
METTL17	TPM3P9	0.335321722	1.32E-14	postive
METTL17	TPT1-AS1	0.503546679	1.68E-33	postive
METTL17	TPTEP2	0.535922213	1.62E-38	postive
METTL17	TRAF3IP2-AS1	0.465989229	2.56E-28	postive
METTL17	TRAM2-AS1	0.404892065	3.79E-21	postive
METTL17	TRAPPC12-AS1	0.337029992	9.55E-15	postive
METTL17	TRIM52-AS1	0.465973066	2.57E-28	postive
METTL17	TRMT2B-AS1	0.386963126	2.62E-19	postive
METTL17	TRPC2	0.356854767	1.84E-16	postive
METTL17	TSC22D1-AS1	0.439786198	4.60E-25	postive
METTL17	TSPEAR-AS1	0.338561936	7.11E-15	postive
METTL17	TSPOAP1-AS1	0.33979224	5.60E-15	postive
METTL17	TSPY26P	0.393053909	6.40E-20	postive
METTL17	TSTD3	0.468440087	1.23E-28	postive
METTL17	TTC28-AS1	0.62041149	1.60E-54	postive
METTL17	TTC41P	0.380068021	1.25E-18	postive
METTL17	TTN-AS1	0.32657639	6.86E-14	postive
METTL17	TUBBP1	0.3396627	5.74E-15	postive
METTL17	TUBBP5	0.406963042	2.29E-21	postive
METTL17	TUG1	0.541986912	1.61E-39	postive
METTL17	TYMSOS	0.349298287	8.57E-16	postive
METTL17	U47924.1	0.503365849	1.78E-33	postive
METTL17	U47924.2	0.449007986	3.54E-26	postive
METTL17	U47924.3	0.357738316	1.53E-16	postive
METTL17	U52111.1	0.305666907	2.83E-12	postive
METTL17	U62317.2	0.491789321	8.25E-32	postive
METTL17	U62317.3	0.358081215	1.43E-16	postive
METTL17	U73166.1	0.476406464	1.08E-29	postive
METTL17	U82695.1	0.331145756	2.92E-14	postive
METTL17	U91328.1	0.499191785	7.23E-33	postive
METTL17	U91328.3	0.461532974	9.59E-28	postive
METTL17	UBA6-AS1	0.498451124	9.24E-33	postive
METTL17	UBAC2-AS1	0.37272013	6.34E-18	postive
METTL17	UBE2D3-AS1	0.441354363	2.99E-25	postive
METTL17	UBE2E1-AS1	0.334455335	1.56E-14	postive
METTL17	UBE2Q1-AS1	0.584937237	3.15E-47	postive
METTL17	UBE2Q2P1	0.365663431	2.90E-17	postive
METTL17	UBE2Q2P2	0.463017947	6.19E-28	postive
METTL17	UBL7-AS1	0.351977218	4.99E-16	postive

METTL17	UBOX5-AS1	0.533773908	3.62E-38	postive
METTL17	UBR5-AS1	0.441735028	2.69E-25	postive
METTL17	UCKL1-AS1	0.307148302	2.20E-12	postive
METTL17	UGDH-AS1	0.453170465	1.09E-26	postive
METTL17	UPF3AP2	0.305921704	2.71E-12	postive
METTL17	URAHP	0.572476518	7.11E-45	postive
METTL17	URB1-AS1	0.418715271	1.21E-22	postive
METTL17	USP27X-AS1	0.416070798	2.36E-22	postive
METTL17	USP32P1	0.310099184	1.32E-12	postive
METTL17	USP3-AS1	0.375803973	3.22E-18	postive
METTL17	USP46-AS1	0.392852745	6.71E-20	postive
METTL17	UXT-AS1	0.443152247	1.82E-25	postive
METTL17	VASH1-AS1	0.570999226	1.33E-44	postive
METTL17	VDAC1P8	0.440399996	3.89E-25	postive
METTL17	VLDLR-AS1	0.310255394	1.28E-12	postive
METTL17	VPS13B-DT	0.426463748	1.63E-23	postive
METTL17	VPS33B-DT	0.347081597	1.34E-15	postive
METTL17	VPS9D1-AS1	0.383163325	6.23E-19	postive
METTL17	WAC-AS1	0.493945178	4.09E-32	postive
METTL17	WARS2-AS1	0.452701998	1.24E-26	postive
METTL17	WASH3P	0.633328336	2.04E-57	postive
METTL17	WASH6P	0.441441012	2.92E-25	postive
METTL17	WASIR2	0.363384687	4.71E-17	postive
METTL17	WEE2-AS1	0.395947898	3.24E-20	postive
METTL17	WHAMMP3	0.375038661	3.82E-18	postive
METTL17	WWTR1-AS1	0.432548174	3.26E-24	postive
METTL17	YEATS2-AS1	0.490860364	1.12E-31	postive
METTL17	YTHDF3-AS1	0.332640554	2.21E-14	postive
METTL17	Z69706.1	0.350818258	6.31E-16	postive
METTL17	Z69720.1	0.416176512	2.30E-22	postive
METTL17	Z69733.1	0.310816433	1.16E-12	postive
METTL17	Z73429.1	0.317384706	3.65E-13	postive
METTL17	Z82214.1	0.334470982	1.56E-14	postive
METTL17	Z82243.1	0.349743234	7.83E-16	postive
METTL17	Z83844.2	0.460372461	1.35E-27	postive
METTL17	Z83851.4	0.401953188	7.73E-21	postive
METTL17	Z84485.1	0.51530591	2.92E-35	postive
METTL17	Z84492.1	0.423895875	3.18E-23	postive
METTL17	Z92544.1	0.460874679	1.16E-27	postive
METTL17	Z92544.2	0.316813613	4.04E-13	postive
METTL17	Z94721.1	0.358852011	1.22E-16	postive
METTL17	Z95115.1	0.443936334	1.46E-25	postive
METTL17	Z97055.2	0.519565644	6.46E-36	postive
METTL17	Z97634.1	0.462619616	6.96E-28	postive
METTL17	Z97832.2	0.469690853	8.42E-29	postive
METTL17	Z97989.1	0.36506111	3.30E-17	postive
METTL17	Z98200.2	0.441152798	3.16E-25	postive
METTL17	Z98884.2	0.468804074	1.10E-28	postive
METTL17	Z98885.2	0.328208687	5.07E-14	postive
METTL17	Z99572.1	0.38773743	2.20E-19	postive

METTL17	ZBED3-AS1	0.395425126	3.67E-20	postive
METTL17	ZBED5-AS1	0.375578898	3.39E-18	postive
METTL17	ZBTB11-AS1	0.477076397	8.79E-30	postive
METTL17	ZBTB40-IT1	0.403334277	5.54E-21	postive
METTL17	ZBTB44-DT	0.411121785	8.18E-22	postive
METTL17	ZDHHC20-IT1	0.335896547	1.19E-14	postive
METTL17	ZEB1-AS1	0.308545069	1.73E-12	postive
METTL17	ZFHX2-AS1	0.662409304	1.84E-64	postive
METTL17	ZKSCAN2-DT	0.54836532	1.36E-40	postive
METTL17	ZMIZ1-AS1	0.331606333	2.68E-14	postive
METTL17	ZNF137P	0.436301562	1.19E-24	postive
METTL17	ZNF192P1	0.345087063	1.98E-15	postive
METTL17	ZNF205-AS1	0.523335855	1.67E-36	postive
METTL17	ZNF213-AS1	0.614926734	2.47E-53	postive
METTL17	ZNF252P	0.41695476	1.89E-22	postive
METTL17	ZNF252P-AS1	0.599177728	4.78E-50	postive
METTL17	ZNF286B	0.334952227	1.42E-14	postive
METTL17	ZNF300P1	0.356629423	1.93E-16	postive
METTL17	ZNF30-AS1	0.369526296	1.27E-17	postive
METTL17	ZNF321P	0.410457231	9.65E-22	postive
METTL17	ZNF32-AS1	0.521873349	2.83E-36	postive
METTL17	ZNF32-AS2	0.522370081	2.37E-36	postive
METTL17	ZNF337-AS1	0.418240105	1.36E-22	postive
METTL17	ZNF341-AS1	0.325786828	7.94E-14	postive
METTL17	ZNF346-IT1	0.369943805	1.16E-17	postive
METTL17	ZNF37BP	0.489989979	1.48E-31	postive
METTL17	ZNF426-DT	0.414788929	3.27E-22	postive
METTL17	ZNF433-AS1	0.415129008	3.00E-22	postive
METTL17	ZNF436-AS1	0.598287918	7.24E-50	postive
METTL17	ZNF460-AS1	0.42547297	2.11E-23	postive
METTL17	ZNF503-AS2	0.42404999	3.06E-23	postive
METTL17	ZNF528-AS1	0.408268767	1.66E-21	postive
METTL17	ZNF529-AS1	0.42914735	8.04E-24	postive
METTL17	ZNF56	0.49827548	9.80E-33	postive
METTL17	ZNF561-AS1	0.418842474	1.17E-22	postive
METTL17	ZNF582-AS1	0.321098769	1.87E-13	postive
METTL17	ZNF674-AS1	0.435889731	1.33E-24	postive
METTL17	ZNF687-AS1	0.436055006	1.27E-24	postive
METTL17	ZNF767P	0.642008949	1.94E-59	postive
METTL17	ZNF788P	0.331892018	2.54E-14	postive
METTL17	ZNF815P	0.48062905	2.91E-30	postive
METTL17	ZNF876P	0.32437276	1.03E-13	postive
METTL17	ZNF890P	0.336353545	1.09E-14	postive
METTL17	ZNNT1	0.302299163	5.02E-12	postive
METTL17	ZNRD1ASP	0.572462379	7.16E-45	postive
METTL17	ZNRD2-AS1	0.624929382	1.61E-55	postive
METTL17	ZNRF2P2	0.330566023	3.26E-14	postive
METTL17	ZSCAN16-AS1	0.466949833	1.92E-28	postive
METTL17	ZSWIM8-AS1	0.399455534	1.41E-20	postive
MOXD1	A2M-AS1	0.389125232	1.60E-19	postive

MOXD1	AC000403.1	0.557184935	4.05E-42	postive
MOXD1	AC002091.2	0.333552404	1.86E-14	postive
MOXD1	AC004080.2	0.308302936	1.80E-12	postive
MOXD1	AC004540.1	0.320358203	2.14E-13	postive
MOXD1	AC004865.2	0.301286801	5.96E-12	postive
MOXD1	AC005342.2	0.311566226	1.02E-12	postive
MOXD1	AC006033.2	0.340478194	4.90E-15	postive
MOXD1	AC006126.4	0.350796942	6.34E-16	postive
MOXD1	AC007671.1	0.330072765	3.58E-14	postive
MOXD1	AC007998.3	0.472161337	3.98E-29	postive
MOXD1	AC008494.3	0.30268711	4.71E-12	postive
MOXD1	AC008957.1	0.305680291	2.83E-12	postive
MOXD1	AC009486.1	0.337954752	7.99E-15	postive
MOXD1	AC009831.1	0.339309217	6.15E-15	postive
MOXD1	AC010247.1	0.391863736	8.46E-20	postive
MOXD1	AC010931.1	0.467200357	1.78E-28	postive
MOXD1	AC011899.2	0.300743351	6.53E-12	postive
MOXD1	AC012085.2	0.330910163	3.06E-14	postive
MOXD1	AC013553.3	0.357077399	1.76E-16	postive
MOXD1	AC015712.2	0.312975212	7.98E-13	postive
MOXD1	AC018697.1	0.490378507	1.30E-31	postive
MOXD1	AC018755.4	0.311484326	1.04E-12	postive
MOXD1	AC021016.2	0.32887813	4.47E-14	postive
MOXD1	AC022164.1	0.318035881	3.25E-13	postive
MOXD1	AC026904.2	0.434437574	1.96E-24	postive
MOXD1	AC034199.1	0.323925609	1.12E-13	postive
MOXD1	AC034245.1	0.33585474	1.20E-14	postive
MOXD1	AC060766.7	0.338216026	7.60E-15	postive
MOXD1	AC064875.1	0.398065653	1.96E-20	postive
MOXD1	AC079298.3	0.368506478	1.58E-17	postive
MOXD1	AC087477.5	0.386695823	2.79E-19	postive
MOXD1	AC090136.3	0.509293749	2.36E-34	postive
MOXD1	AC090559.1	0.429033982	8.29E-24	postive
MOXD1	AC090825.1	0.32318523	1.28E-13	postive
MOXD1	AC091979.1	0.372716859	6.35E-18	postive
MOXD1	AC092376.2	0.482450681	1.64E-30	postive
MOXD1	AC093278.2	0.394206056	4.89E-20	postive
MOXD1	AC096733.3	0.365460229	3.03E-17	postive
MOXD1	AC099524.1	0.313017976	7.92E-13	postive
MOXD1	AC100849.1	0.337838403	8.17E-15	postive
MOXD1	AC103808.1	0.330821297	3.11E-14	postive
MOXD1	AC103808.2	0.338413397	7.32E-15	postive
MOXD1	AC103982.1	0.376249425	2.92E-18	postive
MOXD1	AC104083.1	0.455909518	4.94E-27	postive
MOXD1	AC104119.1	0.336019027	1.16E-14	postive
MOXD1	AC105942.1	0.317949006	3.30E-13	postive
MOXD1	AC106739.1	0.317132797	3.82E-13	postive
MOXD1	AC106881.1	0.505052081	1.01E-33	postive
MOXD1	AC107294.3	0.305797726	2.77E-12	postive
MOXD1	AC107959.1	0.363606746	4.49E-17	postive

MOXD1	AC108463.2	0.315969281	4.70E-13	postive
MOXD1	AC110995.1	0.400188791	1.18E-20	postive
MOXD1	AC112715.1	0.305563322	2.89E-12	postive
MOXD1	AC119044.1	0.317639557	3.49E-13	postive
MOXD1	AC120049.1	0.39265096	7.04E-20	postive
MOXD1	AC145098.1	0.39865067	1.71E-20	postive
MOXD1	AC147651.4	0.303359719	4.20E-12	postive
MOXD1	AC243960.1	0.304028523	3.75E-12	postive
MOXD1	ACTA2-AS1	0.351393249	5.62E-16	postive
MOXD1	ADAMTS7P4	0.328164281	5.11E-14	postive
MOXD1	AF111167.2	0.314974155	5.61E-13	postive
MOXD1	AF127936.5	0.340504303	4.88E-15	postive
MOXD1	AJ239318.1	0.326798674	6.58E-14	postive
MOXD1	AL009178.2	0.333299448	1.95E-14	postive
MOXD1	AL031651.2	0.309460814	1.47E-12	postive
MOXD1	AL049569.1	0.328376029	4.91E-14	postive
MOXD1	AL049775.1	0.348098098	1.09E-15	postive
MOXD1	AL049775.3	0.321226323	1.83E-13	postive
MOXD1	AL049838.1	0.357410405	1.64E-16	postive
MOXD1	AL078581.2	0.40759809	1.96E-21	postive
MOXD1	AL109741.1	0.316829141	4.03E-13	postive
MOXD1	AL109924.1	0.307635336	2.02E-12	postive
MOXD1	AL121749.1	0.313731189	6.98E-13	postive
MOXD1	AL122035.1	0.355616794	2.37E-16	postive
MOXD1	AL133371.2	0.419170297	1.08E-22	postive
MOXD1	AL136084.3	0.390149176	1.26E-19	postive
MOXD1	AL137003.1	0.304539597	3.44E-12	postive
MOXD1	AL137058.2	0.348249463	1.06E-15	postive
MOXD1	AL139339.1	0.433582828	2.47E-24	postive
MOXD1	AL139393.3	0.339949564	5.43E-15	postive
MOXD1	AL157394.1	0.341878872	3.73E-15	postive
MOXD1	AL157895.2	0.404134729	4.56E-21	postive
MOXD1	AL160153.1	0.310374401	1.26E-12	postive
MOXD1	AL353751.1	0.349638271	8.00E-16	postive
MOXD1	AL353753.1	0.383749291	5.46E-19	postive
MOXD1	AL356599.1	0.348926907	9.23E-16	postive
MOXD1	AL357054.4	0.353378397	3.75E-16	postive
MOXD1	AL390955.2	0.413222408	4.84E-22	postive
MOXD1	AL445423.1	0.424657968	2.61E-23	postive
MOXD1	AL445426.1	0.447178337	5.93E-26	postive
MOXD1	AL590764.1	0.341594126	3.94E-15	postive
MOXD1	AL596087.2	0.425859234	1.91E-23	postive
MOXD1	AP000317.1	0.310665152	1.20E-12	postive
MOXD1	AP001107.5	0.3232951	1.25E-13	postive
MOXD1	AP001189.3	0.423323745	3.69E-23	postive
MOXD1	AP001330.4	0.329522238	3.97E-14	postive
MOXD1	AP003071.4	0.302124771	5.17E-12	postive
MOXD1	AP003355.2	0.461671519	9.20E-28	postive
MOXD1	ARMCX5-GPRASP2	0.304988537	3.18E-12	postive
MOXD1	ATXN1-AS1	0.31852627	2.98E-13	postive

MOXD1	BAALC-AS1	0.307154158	2.20E-12	postive
MOXD1	BTN2A3P	0.32289033	1.35E-13	postive
MOXD1	BX284613.2	0.352672094	4.33E-16	postive
MOXD1	BX322234.1	0.331041983	2.98E-14	postive
MOXD1	C2orf27A	0.345449016	1.85E-15	postive
MOXD1	CARD8-AS1	0.442812911	2.00E-25	postive
MOXD1	CASP17P	0.353762725	3.47E-16	postive
MOXD1	CEP250-AS1	0.332323522	2.34E-14	postive
MOXD1	CTC-786C10.1	0.342613668	3.23E-15	postive
MOXD1	DACT3-AS1	0.426671038	1.54E-23	postive
MOXD1	DNM3OS	0.574101125	3.55E-45	postive
MOXD1	EML4-AS1	0.317974409	3.28E-13	postive
MOXD1	EPB41L4A-DT	0.361870185	6.47E-17	postive
MOXD1	FAM66C	0.359859414	9.86E-17	postive
MOXD1	FGD5-AS1	0.364059493	4.08E-17	postive
MOXD1	FNDC1-IT1	0.349818044	7.72E-16	postive
MOXD1	GGTA1P	0.470673608	6.25E-29	postive
MOXD1	GTF2IP4	0.348428182	1.02E-15	postive
MOXD1	GVINP1	0.329415279	4.05E-14	postive
MOXD1	HCG11	0.348959807	9.17E-16	postive
MOXD1	HECW2-AS1	0.470233868	7.14E-29	postive
MOXD1	HLX-AS1	0.411793718	6.92E-22	postive
MOXD1	IGFBP7-AS1	0.403820025	4.92E-21	postive
MOXD1	IL10RB-DT	0.352541797	4.45E-16	postive
MOXD1	IL12A-AS1	0.338193522	7.63E-15	postive
MOXD1	INHBA-AS1	0.389384744	1.50E-19	postive
MOXD1	ITGA9-AS1	0.34192702	3.69E-15	postive
MOXD1	L3MBTL2-AS1	0.331962639	2.51E-14	postive
MOXD1	L3MBTL4-AS1	0.392177231	7.86E-20	postive
MOXD1	LCT-AS1	0.32741396	5.87E-14	postive
MOXD1	LEF1-AS1	0.344863959	2.07E-15	postive
MOXD1	LINC00163	0.348419986	1.02E-15	postive
MOXD1	LINC00310	0.41984079	9.06E-23	postive
MOXD1	LINC00398	0.32743712	5.85E-14	postive
MOXD1	LINC00402	0.305650244	2.84E-12	postive
MOXD1	LINC00426	0.319673554	2.42E-13	postive
MOXD1	LINC00469	0.3589342	1.20E-16	postive
MOXD1	LINC00582	0.310044773	1.33E-12	postive
MOXD1	LINC00598	0.39356751	5.68E-20	postive
MOXD1	LINC00626	0.408908315	1.42E-21	postive
MOXD1	LINC00667	0.327731304	5.54E-14	postive
MOXD1	LINC00702	0.357642777	1.56E-16	postive
MOXD1	LINC00892	0.318163862	3.18E-13	postive
MOXD1	LINC00900	0.364033781	4.10E-17	postive
MOXD1	LINC00924	0.455251124	5.97E-27	postive
MOXD1	LINC00968	0.338590767	7.07E-15	postive
MOXD1	LINC00987	0.405844836	3.01E-21	postive
MOXD1	LINC01013	0.374941327	3.90E-18	postive
MOXD1	LINC01048	0.318777313	2.84E-13	postive
MOXD1	LINC01081	0.357061769	1.76E-16	postive

MOXD1	LINC01094	0.397216531	2.40E-20	postive
MOXD1	LINC01128	0.336902034	9.79E-15	postive
MOXD1	LINC01140	0.354250449	3.14E-16	postive
MOXD1	LINC01150	0.435217225	1.59E-24	postive
MOXD1	LINC01197	0.395862051	3.31E-20	postive
MOXD1	LINC01262	0.342540975	3.28E-15	postive
MOXD1	LINC01352	0.480089043	3.44E-30	postive
MOXD1	LINC01412	0.389736184	1.39E-19	postive
MOXD1	LINC01415	0.367917141	1.79E-17	postive
MOXD1	LINC01449	0.366443337	2.46E-17	postive
MOXD1	LINC01506	0.326233607	7.31E-14	postive
MOXD1	LINC01546	0.428929733	8.52E-24	postive
MOXD1	LINC01614	0.333095801	2.02E-14	postive
MOXD1	LINC01638	0.344888185	2.06E-15	postive
MOXD1	LINC01679	0.343072233	2.95E-15	postive
MOXD1	LINC01684	0.351101163	5.96E-16	postive
MOXD1	LINC01781	0.312615256	8.50E-13	postive
MOXD1	LINC01915	0.44605658	8.12E-26	postive
MOXD1	LINC01936	0.495751715	2.26E-32	postive
MOXD1	LINC02073	0.404417642	4.26E-21	postive
MOXD1	LINC02126	0.600818282	2.22E-50	postive
MOXD1	LINC02202	0.418934085	1.14E-22	postive
MOXD1	LINC02273	0.346842716	1.40E-15	postive
MOXD1	LINC02285	0.392419554	7.43E-20	postive
MOXD1	LINC02325	0.308089848	1.87E-12	postive
MOXD1	LINC02391	0.454813064	6.77E-27	postive
MOXD1	LINC02408	0.423617137	3.42E-23	postive
MOXD1	LINC02519	0.591500249	1.65E-48	postive
MOXD1	LINC02520	0.333988533	1.71E-14	postive
MOXD1	LINC02529	0.308560679	1.72E-12	postive
MOXD1	LINC02544	0.446489385	7.19E-26	postive
MOXD1	LINC02587	0.367656826	1.90E-17	postive
MOXD1	LINC02599	0.32868428	4.64E-14	postive
MOXD1	LINC02611	0.336728767	1.01E-14	postive
MOXD1	LINC02693	0.318602405	2.93E-13	postive
MOXD1	LINC02798	0.33892676	6.62E-15	postive
MOXD1	LINC02828	0.332846908	2.12E-14	postive
MOXD1	LINC02829	0.358935166	1.20E-16	postive
MOXD1	LRRC37A17P	0.326759447	6.63E-14	postive
MOXD1	LRRC8C-DT	0.356925411	1.81E-16	postive
MOXD1	MACORIS	0.396411069	2.91E-20	postive
MOXD1	MAGI2-AS3	0.388080513	2.03E-19	postive
MOXD1	MAP2K4P1	0.324697625	9.70E-14	postive
MOXD1	MED14OS	0.30004976	7.33E-12	postive
MOXD1	MIR497HG	0.416393459	2.18E-22	postive
MOXD1	MMADHC-DT	0.37028977	1.08E-17	postive
MOXD1	MRPS31P4	0.340043183	5.33E-15	postive
MOXD1	MSC-AS1	0.368122991	1.72E-17	postive
MOXD1	NAIPP1	0.308164952	1.85E-12	postive
MOXD1	NAIPP3	0.320101935	2.24E-13	postive

MOXD1	NAPSB	0.407370617	2.07E-21	postive
MOXD1	NBPF25P	0.315903197	4.75E-13	postive
MOXD1	NCF1B	0.307895619	1.93E-12	postive
MOXD1	NORAD	0.304964914	3.20E-12	postive
MOXD1	OR14L1P	0.300173042	7.18E-12	postive
MOXD1	OR7E47P	0.381212819	9.67E-19	postive
MOXD1	OR7E94P	0.331757464	2.61E-14	postive
MOXD1	PAXIP1-AS2	0.376135271	3.00E-18	postive
MOXD1	PCED1B-AS1	0.452362468	1.37E-26	postive
MOXD1	PPIAP48	0.351781579	5.19E-16	postive
MOXD1	PRICKLE2-DT	0.356303196	2.06E-16	postive
MOXD1	PRR34	0.386431332	2.96E-19	postive
MOXD1	PTENP1	0.33780163	8.23E-15	postive
MOXD1	PTPRD-AS1	0.455999113	4.81E-27	postive
MOXD1	RAP2C-AS1	0.368812876	1.48E-17	postive
MOXD1	RASGRF2-AS1	0.319905894	2.32E-13	postive
MOXD1	RORB-AS1	0.321130692	1.86E-13	postive
MOXD1	RP11-180N14.1	0.308627282	1.70E-12	postive
MOXD1	RP1-122P22.2	0.371982831	7.45E-18	postive
MOXD1	RP11-439I14.2	0.346717403	1.44E-15	postive
MOXD1	RP11-514D23.3	0.451091139	1.96E-26	postive
MOXD1	RP11-524D16__A.3	0.30543732	2.95E-12	postive
MOXD1	RP1-93H18.1	0.348651738	9.75E-16	postive
MOXD1	RPL23AP7	0.30359445	4.03E-12	postive
MOXD1	RPL23AP82	0.360210063	9.16E-17	postive
MOXD1	RXYLT1-AS1	0.398504792	1.77E-20	postive
MOXD1	SEC22B4P	0.321884503	1.62E-13	postive
MOXD1	SH3RF3-AS1	0.414600716	3.43E-22	postive
MOXD1	SIGLEC17P	0.309985538	1.35E-12	postive
MOXD1	SIGLEC22P	0.342712167	3.17E-15	postive
MOXD1	SOCAR	0.310063991	1.33E-12	postive
MOXD1	SVIL2P	0.356281463	2.07E-16	postive
MOXD1	SVIL-AS1	0.331014011	3.00E-14	postive
MOXD1	TBX5-AS1	0.606242571	1.69E-51	postive
MOXD1	TMSB15B-AS1	0.317707112	3.45E-13	postive
MOXD1	TRAF3IP2-AS1	0.3251791	8.88E-14	postive
MOXD1	TRBV11-2	0.338254509	7.54E-15	postive
MOXD1	TSPOAP1-AS1	0.361556525	6.91E-17	postive
MOXD1	Z97353.2	0.344816915	2.09E-15	postive
MOXD1	Z97989.1	0.412124105	6.37E-22	postive
MOXD2P	AC027343.1	0.335410468	1.30E-14	postive
MOXD2P	AC110716.1	0.313558857	7.20E-13	postive
MOXD2P	AC244517.5	0.321267888	1.81E-13	postive
MOXD2P	LINC00207	0.305629891	2.85E-12	postive
MOXD2P	LINC02142	0.315785962	4.85E-13	postive
MOXD2P	LINC02464	0.315826608	4.82E-13	postive
MOXD2P	MOXD2P	1	0	postive
MOXD2P	SMARCA5-AS1	0.305957357	2.70E-12	postive
MPC1	ABCC6P2	0.418123299	1.40E-22	postive
MPC1	ABHD11-AS1	0.309289784	1.52E-12	postive

MPC1	AC002467.1	0.367151526	2.11E-17	postive
MPC1	AC004241.1	0.41243044	5.90E-22	postive
MPC1	AC004540.1	0.316920912	3.97E-13	postive
MPC1	AC004812.2	0.324415907	1.02E-13	postive
MPC1	AC004862.1	0.322027536	1.58E-13	postive
MPC1	AC004921.1	0.343095896	2.94E-15	postive
MPC1	AC004982.1	0.359074782	1.16E-16	postive
MPC1	AC004982.2	0.381648842	8.77E-19	postive
MPC1	AC005083.1	0.306726578	2.36E-12	postive
MPC1	AC005229.4	0.321032887	1.89E-13	postive
MPC1	AC005520.4	0.33708443	9.45E-15	postive
MPC1	AC005562.1	0.349673298	7.95E-16	postive
MPC1	AC006001.2	0.301498857	5.75E-12	postive
MPC1	AC006213.1	0.358603866	1.28E-16	postive
MPC1	AC006333.1	0.401556951	8.51E-21	postive
MPC1	AC006449.2	0.383172092	6.22E-19	postive
MPC1	AC006449.7	0.37862812	1.72E-18	postive
MPC1	AC006942.1	0.331415176	2.78E-14	postive
MPC1	AC007100.1	0.318824499	2.82E-13	postive
MPC1	AC007114.1	0.400188695	1.18E-20	postive
MPC1	AC007228.9	0.342635122	3.22E-15	postive
MPC1	AC007342.4	0.303564485	4.06E-12	postive
MPC1	AC007383.2	0.316349831	4.39E-13	postive
MPC1	AC007405.4	0.349728844	7.86E-16	postive
MPC1	AC008014.1	0.357438344	1.63E-16	postive
MPC1	AC008105.2	0.417423671	1.68E-22	postive
MPC1	AC008267.5	0.443712564	1.56E-25	postive
MPC1	AC008393.1	0.362627398	5.52E-17	postive
MPC1	AC008438.1	0.43484413	1.76E-24	postive
MPC1	AC008443.1	0.375287351	3.61E-18	postive
MPC1	AC008443.5	0.393446317	5.84E-20	postive
MPC1	AC008494.3	0.384192549	4.94E-19	postive
MPC1	AC008608.2	0.355134517	2.62E-16	postive
MPC1	AC008669.1	0.353422949	3.72E-16	postive
MPC1	AC008758.4	0.302344081	4.99E-12	postive
MPC1	AC008771.1	0.428459836	9.64E-24	postive
MPC1	AC008915.3	0.498032653	1.06E-32	postive
MPC1	AC008946.1	0.392463162	7.35E-20	postive
MPC1	AC008966.1	0.393474083	5.80E-20	postive
MPC1	AC009061.2	0.348113904	1.09E-15	postive
MPC1	AC009065.5	0.308819209	1.65E-12	postive
MPC1	AC009113.1	0.354835228	2.79E-16	postive
MPC1	AC009126.1	0.392323046	7.60E-20	postive
MPC1	AC009171.2	0.308922603	1.62E-12	postive
MPC1	AC009403.1	0.412080289	6.44E-22	postive
MPC1	AC009506.2	0.389928398	1.33E-19	postive
MPC1	AC009690.2	0.330846953	3.09E-14	postive
MPC1	AC009779.3	0.358971978	1.19E-16	postive
MPC1	AC009831.1	0.314220385	6.41E-13	postive
MPC1	AC009961.1	0.327880877	5.39E-14	postive

MPC1	AC010226.1	0.45425916	7.94E-27	postive
MPC1	AC010273.2	0.311450858	1.04E-12	postive
MPC1	AC010320.2	0.341696122	3.87E-15	postive
MPC1	AC010442.1	0.339392048	6.05E-15	postive
MPC1	AC010907.2	0.375472106	3.47E-18	postive
MPC1	AC010969.2	0.346020249	1.65E-15	postive
MPC1	AC012146.1	0.41154031	7.37E-22	postive
MPC1	AC012306.2	0.303057282	4.42E-12	postive
MPC1	AC012467.2	0.347876565	1.14E-15	postive
MPC1	AC012615.1	0.352808044	4.21E-16	postive
MPC1	AC012640.4	0.336794168	9.99E-15	postive
MPC1	AC015802.4	0.306242751	2.57E-12	postive
MPC1	AC015917.2	0.458000391	2.69E-27	postive
MPC1	AC015922.2	0.306687733	2.38E-12	postive
MPC1	AC015922.3	0.393598151	5.64E-20	postive
MPC1	AC015982.2	0.386990926	2.61E-19	postive
MPC1	AC016394.2	0.376000203	3.09E-18	postive
MPC1	AC016727.1	0.327290264	6.01E-14	postive
MPC1	AC016747.1	0.372063955	7.32E-18	postive
MPC1	AC016876.2	0.303082173	4.40E-12	postive
MPC1	AC016876.3	0.323557845	1.20E-13	postive
MPC1	AC016888.1	0.321680294	1.68E-13	postive
MPC1	AC018521.5	0.447695289	5.13E-26	postive
MPC1	AC019131.2	0.354870986	2.77E-16	postive
MPC1	AC020663.2	0.382156131	7.82E-19	postive
MPC1	AC020910.5	0.376031141	3.07E-18	postive
MPC1	AC020915.3	0.32384984	1.13E-13	postive
MPC1	AC021016.2	0.370914294	9.39E-18	postive
MPC1	AC021074.3	0.319897127	2.32E-13	postive
MPC1	AC021146.12	0.322093577	1.56E-13	postive
MPC1	AC022167.3	0.301784401	5.48E-12	postive
MPC1	AC022167.4	0.317773399	3.41E-13	postive
MPC1	AC022816.1	0.30993159	1.36E-12	postive
MPC1	AC022916.2	0.320368776	2.13E-13	postive
MPC1	AC023509.3	0.332203857	2.40E-14	postive
MPC1	AC024361.2	0.320049604	2.26E-13	postive
MPC1	AC024575.2	0.333987902	1.71E-14	postive
MPC1	AC025048.2	0.307707167	2.00E-12	postive
MPC1	AC025048.4	0.434689174	1.83E-24	postive
MPC1	AC025181.2	0.30746658	2.08E-12	postive
MPC1	AC025262.1	0.306038679	2.66E-12	postive
MPC1	AC026304.1	0.325379277	8.56E-14	postive
MPC1	AC026412.1	0.350406931	6.85E-16	postive
MPC1	AC026471.2	0.301946301	5.33E-12	postive
MPC1	AC026691.1	0.344270819	2.33E-15	postive
MPC1	AC026780.2	0.326949883	6.40E-14	postive
MPC1	AC027097.1	0.301180177	6.07E-12	postive
MPC1	AC027237.4	0.335397817	1.31E-14	postive
MPC1	AC027559.1	0.313219165	7.64E-13	postive
MPC1	AC027644.3	0.384486783	4.62E-19	postive

MPC1	AC027682.6	0.340275003	5.10E-15	postive
MPC1	AC036176.1	0.412371529	5.99E-22	postive
MPC1	AC060766.4	0.341161309	4.29E-15	postive
MPC1	AC060766.7	0.371415513	8.43E-18	postive
MPC1	AC064807.1	0.374303999	4.49E-18	postive
MPC1	AC067852.2	0.391257717	9.74E-20	postive
MPC1	AC067930.3	0.355650129	2.36E-16	postive
MPC1	AC068338.2	0.417384036	1.69E-22	postive
MPC1	AC068473.5	0.331998744	2.49E-14	postive
MPC1	AC068790.8	0.313032813	7.90E-13	postive
MPC1	AC068870.2	0.344480042	2.24E-15	postive
MPC1	AC073254.1	0.337343543	8.99E-15	postive
MPC1	AC073611.1	0.320430332	2.11E-13	postive
MPC1	AC073896.2	0.47333288	2.78E-29	postive
MPC1	AC073896.3	0.311217033	1.09E-12	postive
MPC1	AC079848.1	0.391947659	8.29E-20	postive
MPC1	AC083798.2	0.310112287	1.32E-12	postive
MPC1	AC083799.1	0.307258632	2.16E-12	postive
MPC1	AC084125.4	0.33878431	6.81E-15	postive
MPC1	AC084809.1	0.329098729	4.29E-14	postive
MPC1	AC087482.1	0.319428196	2.53E-13	postive
MPC1	AC087521.3	0.369005535	1.42E-17	postive
MPC1	AC090061.1	0.365285635	3.15E-17	postive
MPC1	AC090587.1	0.462133948	8.03E-28	postive
MPC1	AC090617.5	0.337915953	8.05E-15	postive
MPC1	AC090825.1	0.379495921	1.42E-18	postive
MPC1	AC090912.1	0.422219047	4.91E-23	postive
MPC1	AC091153.3	0.376531438	2.75E-18	postive
MPC1	AC091729.3	0.352177845	4.79E-16	postive
MPC1	AC091849.2	0.329813161	3.76E-14	postive
MPC1	AC091965.1	0.333237936	1.97E-14	postive
MPC1	AC091965.4	0.310910508	1.15E-12	postive
MPC1	AC091982.3	0.340292679	5.08E-15	postive
MPC1	AC092925.1	0.328836278	4.51E-14	postive
MPC1	AC092944.1	0.322521624	1.44E-13	postive
MPC1	AC093157.1	0.332267187	2.37E-14	postive
MPC1	AC093510.1	0.31611722	4.58E-13	postive
MPC1	AC093726.1	0.375754306	3.26E-18	postive
MPC1	AC096536.2	0.377379921	2.28E-18	postive
MPC1	AC096677.1	0.319897856	2.32E-13	postive
MPC1	AC097359.2	0.396806337	2.65E-20	postive
MPC1	AC098484.1	0.322440381	1.47E-13	postive
MPC1	AC098484.2	0.309505866	1.46E-12	postive
MPC1	AC098820.2	0.325033991	9.12E-14	postive
MPC1	AC099343.4	0.350138742	7.23E-16	postive
MPC1	AC099552.1	0.332269837	2.37E-14	postive
MPC1	AC099552.3	0.325276809	8.72E-14	postive
MPC1	AC099850.2	0.397983715	2.00E-20	postive
MPC1	AC100786.1	0.300930712	6.33E-12	postive
MPC1	AC100810.1	0.307620286	2.03E-12	postive

MPC1	AC104051.2	0.342352937	3.40E-15	postive
MPC1	AC104109.2	0.349242568	8.66E-16	postive
MPC1	AC104653.1	0.314651725	5.94E-13	postive
MPC1	AC104667.2	0.391369783	9.49E-20	postive
MPC1	AC104794.4	0.346946565	1.37E-15	postive
MPC1	AC106791.1	0.356582671	1.95E-16	postive
MPC1	AC106795.1	0.410860944	8.73E-22	postive
MPC1	AC107032.2	0.398937289	1.59E-20	postive
MPC1	AC108488.1	0.329727985	3.82E-14	postive
MPC1	AC109322.1	0.333211328	1.98E-14	postive
MPC1	AC109347.1	0.324503885	1.00E-13	postive
MPC1	AC109454.2	0.310764417	1.18E-12	postive
MPC1	AC110995.1	0.319113201	2.68E-13	postive
MPC1	AC112220.2	0.304349886	3.55E-12	postive
MPC1	AC115618.1	0.418158113	1.39E-22	postive
MPC1	AC115618.2	0.450110299	2.59E-26	postive
MPC1	AC115619.1	0.327154998	6.16E-14	postive
MPC1	AC116913.1	0.36178259	6.59E-17	postive
MPC1	AC118553.1	0.314602121	5.99E-13	postive
MPC1	AC125257.1	0.392669773	7.01E-20	postive
MPC1	AC126323.3	0.306513473	2.45E-12	postive
MPC1	AC130343.2	0.312559825	8.58E-13	postive
MPC1	AC132938.4	0.362021139	6.27E-17	postive
MPC1	AC133552.2	0.326468402	7.00E-14	postive
MPC1	AC135050.6	0.320266284	2.17E-13	postive
MPC1	AC136604.3	0.427619715	1.20E-23	postive
MPC1	AC138207.2	0.368037733	1.75E-17	postive
MPC1	AC138207.8	0.404179364	4.51E-21	postive
MPC1	AC139530.1	0.351745731	5.23E-16	postive
MPC1	AC139768.1	0.423132535	3.88E-23	postive
MPC1	AC226101.1	0.354337189	3.08E-16	postive
MPC1	AC231981.1	0.323443916	1.22E-13	postive
MPC1	AC232271.1	0.312864952	8.14E-13	postive
MPC1	AC243919.1	0.468806879	1.10E-28	postive
MPC1	AC244090.1	0.447209465	5.88E-26	postive
MPC1	AC244669.1	0.338119586	7.74E-15	postive
MPC1	AC245297.4	0.321572939	1.72E-13	postive
MPC1	AC246785.3	0.310223497	1.29E-12	postive
MPC1	ADCY6-DT	0.375586117	3.38E-18	postive
MPC1	AF111167.2	0.359386254	1.09E-16	postive
MPC1	AF196972.1	0.351697362	5.28E-16	postive
MPC1	AF274858.1	0.336281917	1.10E-14	postive
MPC1	AFDN-DT	0.326570205	6.87E-14	postive
MPC1	AL020996.1	0.343580701	2.67E-15	postive
MPC1	AL022069.1	0.316099052	4.59E-13	postive
MPC1	AL024498.1	0.330163269	3.52E-14	postive
MPC1	AL024507.3	0.320464042	2.10E-13	postive
MPC1	AL031775.1	0.355131776	2.62E-16	postive
MPC1	AL033519.4	0.377260711	2.34E-18	postive
MPC1	AL035446.1	0.313945967	6.72E-13	postive

MPC1	AL035587.1	0.360218206	9.15E-17	postive
MPC1	AL035661.1	0.313968149	6.70E-13	postive
MPC1	AL049597.2	0.317396774	3.64E-13	postive
MPC1	AL050309.1	0.404989498	3.70E-21	postive
MPC1	AL050341.2	0.305260834	3.04E-12	postive
MPC1	AL078581.2	0.418713634	1.21E-22	postive
MPC1	AL078590.2	0.338219549	7.59E-15	postive
MPC1	AL080276.2	0.305575584	2.88E-12	postive
MPC1	AL096828.3	0.315679727	4.95E-13	postive
MPC1	AL109917.1	0.331981009	2.50E-14	postive
MPC1	AL118506.1	0.304857205	3.25E-12	postive
MPC1	AL118558.3	0.315163717	5.42E-13	postive
MPC1	AL121601.1	0.330984548	3.01E-14	postive
MPC1	AL121944.2	0.456753485	3.87E-27	postive
MPC1	AL132857.1	0.356987217	1.79E-16	postive
MPC1	AL133338.1	0.372516552	6.63E-18	postive
MPC1	AL137003.1	0.313824361	6.87E-13	postive
MPC1	AL138826.1	0.317834558	3.37E-13	postive
MPC1	AL139011.1	0.32996581	3.65E-14	postive
MPC1	AL139260.1	0.351226378	5.81E-16	postive
MPC1	AL139384.1	0.316378457	4.37E-13	postive
MPC1	AL157896.1	0.313977528	6.69E-13	postive
MPC1	AL158071.1	0.365630504	2.92E-17	postive
MPC1	AL158071.4	0.338872623	6.69E-15	postive
MPC1	AL160313.2	0.311229	1.08E-12	postive
MPC1	AL162231.2	0.36528178	3.15E-17	postive
MPC1	AL162258.2	0.306900976	2.29E-12	postive
MPC1	AL162377.1	0.32179807	1.65E-13	postive
MPC1	AL353708.1	0.41817161	1.39E-22	postive
MPC1	AL353743.1	0.339036401	6.49E-15	postive
MPC1	AL354740.1	0.309226843	1.54E-12	postive
MPC1	AL354892.2	0.337238748	9.17E-15	postive
MPC1	AL354920.1	0.50262441	2.29E-33	postive
MPC1	AL354928.1	0.30746904	2.08E-12	postive
MPC1	AL355001.2	0.334779724	1.47E-14	postive
MPC1	AL355353.2	0.336501098	1.06E-14	postive
MPC1	AL355490.2	0.39805422	1.97E-20	postive
MPC1	AL356056.2	0.30446075	3.48E-12	postive
MPC1	AL356481.1	0.392391013	7.48E-20	postive
MPC1	AL356599.1	0.377466222	2.23E-18	postive
MPC1	AL358472.2	0.4026502	6.53E-21	postive
MPC1	AL358852.1	0.33545564	1.29E-14	postive
MPC1	AL359232.1	0.314707982	5.88E-13	postive
MPC1	AL359513.1	0.336237139	1.11E-14	postive
MPC1	AL359711.2	0.360456529	8.70E-17	postive
MPC1	AL359715.3	0.374953927	3.89E-18	postive
MPC1	AL390067.1	0.361429933	7.10E-17	postive
MPC1	AL390198.1	0.300490859	6.81E-12	postive
MPC1	AL390760.1	0.302154783	5.15E-12	postive
MPC1	AL390955.2	0.394235364	4.85E-20	postive

MPC1	AL391069.2	0.46680015	2.01E-28	postive
MPC1	AL391095.3	0.37295591	6.02E-18	postive
MPC1	AL391840.1	0.324843855	9.44E-14	postive
MPC1	AL392172.1	0.419741629	9.29E-23	postive
MPC1	AL441992.2	0.369797007	1.20E-17	postive
MPC1	AL442663.3	0.327836662	5.43E-14	postive
MPC1	AL450998.2	0.311880203	9.67E-13	postive
MPC1	AL451085.3	0.336830109	9.92E-15	postive
MPC1	AL451165.2	0.469100203	1.01E-28	postive
MPC1	AL512329.2	0.333157534	2.00E-14	postive
MPC1	AL512357.1	0.332947682	2.08E-14	postive
MPC1	AL513550.1	0.410514044	9.52E-22	postive
MPC1	AL589843.1	0.324205973	1.06E-13	postive
MPC1	AL590133.1	0.318217843	3.14E-13	postive
MPC1	AL590764.1	0.309113331	1.57E-12	postive
MPC1	AL592494.1	0.334675639	1.50E-14	postive
MPC1	AL606491.1	0.337106645	9.41E-15	postive
MPC1	AL645568.1	0.30269947	4.70E-12	postive
MPC1	AL645728.1	0.315591565	5.02E-13	postive
MPC1	AL732314.8	0.355521014	2.42E-16	postive
MPC1	ALOX12-AS1	0.38705805	2.57E-19	postive
MPC1	AMZ2P1	0.489258848	1.87E-31	postive
MPC1	ANO7L1	0.346333804	1.55E-15	postive
MPC1	AOC4P	0.324204131	1.06E-13	postive
MPC1	AP000229.1	0.347211955	1.30E-15	postive
MPC1	AP000254.2	0.376880639	2.54E-18	postive
MPC1	AP000317.1	0.363308003	4.78E-17	postive
MPC1	AP000704.2	0.317702021	3.45E-13	postive
MPC1	AP001160.3	0.374611854	4.19E-18	postive
MPC1	AP001267.3	0.320796204	1.98E-13	postive
MPC1	AP001372.2	0.358037838	1.44E-16	postive
MPC1	AP001505.1	0.305029439	3.16E-12	postive
MPC1	AP001816.1	0.423738177	3.32E-23	postive
MPC1	AP001922.5	0.336201672	1.12E-14	postive
MPC1	AP002387.2	0.345596443	1.79E-15	postive
MPC1	AP002748.4	0.460130562	1.45E-27	postive
MPC1	AP003392.4	0.315992086	4.68E-13	postive
MPC1	AP003392.5	0.344121772	2.40E-15	postive
MPC1	AP004609.5	0.359266984	1.12E-16	postive
MPC1	AP005329.2	0.338818273	6.76E-15	postive
MPC1	AP006621.3	0.347593552	1.21E-15	postive
MPC1	APOC1P1	0.421134989	6.50E-23	postive
MPC1	APTR	0.352482074	4.50E-16	postive
MPC1	ARF4-AS1	0.464998408	3.44E-28	postive
MPC1	ARHGEF26-AS1	0.380250008	1.20E-18	postive
MPC1	ARMCX5-GPRASP2	0.420755888	7.16E-23	postive
MPC1	ARRDC1-AS1	0.39753994	2.22E-20	postive
MPC1	ASH1L-AS1	0.351733697	5.24E-16	postive
MPC1	ATXN2-AS	0.325390003	8.54E-14	postive
MPC1	AZIN1-AS1	0.312341321	8.92E-13	postive

MPC1	B4GALT1-AS1	0.315478329	5.13E-13	postive
MPC1	BAALC-AS1	0.319871291	2.34E-13	postive
MPC1	BAIAP2-DT	0.329799784	3.76E-14	postive
MPC1	BCAR3-AS1	0.34376028	2.58E-15	postive
MPC1	BCRP3	0.394094164	5.02E-20	postive
MPC1	BDNF-AS	0.312619265	8.49E-13	postive
MPC1	BMS1P20	0.323632902	1.18E-13	postive
MPC1	BX284668.5	0.396597335	2.78E-20	postive
MPC1	BX649632.1	0.314164902	6.47E-13	postive
MPC1	C21orf62-AS1	0.363385377	4.70E-17	postive
MPC1	C6orf99	0.429282971	7.76E-24	postive
MPC1	C9orf147	0.327249172	6.06E-14	postive
MPC1	CA5BP1	0.392614605	7.10E-20	postive
MPC1	CAHM	0.335490719	1.28E-14	postive
MPC1	CARD8-AS1	0.419008915	1.12E-22	postive
MPC1	CBR3-AS1	0.307586096	2.04E-12	postive
MPC1	CCNT2-AS1	0.37319555	5.72E-18	postive
MPC1	CCR5AS	0.375719071	3.29E-18	postive
MPC1	CCT6P1	0.303004384	4.46E-12	postive
MPC1	CDC37L1-DT	0.355558662	2.40E-16	postive
MPC1	CEBPA-DT	0.308044111	1.88E-12	postive
MPC1	CENPBD1P1	0.32368533	1.17E-13	postive
MPC1	CERNA1	0.354578352	2.94E-16	postive
MPC1	CHKB-DT	0.342507502	3.30E-15	postive
MPC1	CIDECP1	0.406919038	2.31E-21	postive
MPC1	CKMT2-AS1	0.393557796	5.69E-20	postive
MPC1	CNNM3-DT	0.350486098	6.75E-16	postive
MPC1	COX10-AS1	0.335061023	1.39E-14	postive
MPC1	CRTC3-AS1	0.315797719	4.84E-13	postive
MPC1	CTBP1-DT	0.342138141	3.55E-15	postive
MPC1	CTD-2521M24.9	0.331876646	2.55E-14	postive
MPC1	CYTOR	0.316815109	4.04E-13	postive
MPC1	DANCR	0.344619663	2.18E-15	postive
MPC1	DCST1-AS1	0.310992049	1.13E-12	postive
MPC1	DCXR-DT	0.322863163	1.36E-13	postive
MPC1	DHRS4-AS1	0.316701918	4.12E-13	postive
MPC1	DNAJC27-AS1	0.327804356	5.46E-14	postive
MPC1	EBLN3P	0.337186619	9.27E-15	postive
MPC1	EDRF1-DT	0.312454894	8.74E-13	postive
MPC1	EIF1B-AS1	0.343790484	2.56E-15	postive
MPC1	EIF3J-DT	0.38900861	1.64E-19	postive
MPC1	ELOA-AS1	0.371292713	8.65E-18	postive
MPC1	ELOVL2-AS1	0.306670666	2.39E-12	postive
MPC1	EMC1-AS1	0.435967476	1.30E-24	postive
MPC1	ENTPD3-AS1	0.48569529	5.87E-31	postive
MPC1	EPB41L4A-AS1	0.323372099	1.24E-13	postive
MPC1	EPB41L4A-DT	0.438146811	7.19E-25	postive
MPC1	ETV7-AS1	0.375690231	3.31E-18	postive
MPC1	EXOC3-AS1	0.358805902	1.23E-16	postive
MPC1	EXOSC10-AS1	0.361246506	7.38E-17	postive

MPC1	FABP5P3	0.303149191	4.35E-12	postive
MPC1	FAHD2CP	0.300488103	6.81E-12	postive
MPC1	FAM111A-DT	0.375694077	3.30E-18	postive
MPC1	FAM21FP	0.318229154	3.14E-13	postive
MPC1	FAM86C2P	0.407519735	1.99E-21	postive
MPC1	FAM86EP	0.364901006	3.41E-17	postive
MPC1	FAM86JP	0.351064706	6.00E-16	postive
MPC1	FGD5-AS1	0.421940955	5.28E-23	postive
MPC1	FLVCR1-DT	0.358660245	1.27E-16	postive
MPC1	FRG1HP	0.422059898	5.12E-23	postive
MPC1	GGTA1P	0.347896188	1.13E-15	postive
MPC1	GIHCG	0.372187542	7.12E-18	postive
MPC1	GLIDR	0.32343993	1.22E-13	postive
MPC1	GNRHR2	0.364650941	3.60E-17	postive
MPC1	GTF2IP1	0.311370334	1.06E-12	postive
MPC1	GTF2IP4	0.342416559	3.36E-15	postive
MPC1	HCG18	0.318553365	2.96E-13	postive
MPC1	HCG25	0.38453015	4.57E-19	postive
MPC1	HCP5	0.357078601	1.76E-16	postive
MPC1	HEIH	0.496775544	1.61E-32	postive
MPC1	HERC2P3	0.335324387	1.32E-14	postive
MPC1	HLA-DRB6	0.380520907	1.13E-18	postive
MPC1	HMGB1P5	0.310009925	1.34E-12	postive
MPC1	HMGN3-AS1	0.309622295	1.43E-12	postive
MPC1	HORMAD2-AS1	0.375287538	3.61E-18	postive
MPC1	IDH1-AS1	0.326574055	6.86E-14	postive
MPC1	IDI2-AS1	0.33176254	2.60E-14	postive
MPC1	IL10RB-DT	0.345972894	1.66E-15	postive
MPC1	IL6R-AS1	0.425619647	2.03E-23	postive
MPC1	ILF3-DT	0.360351615	8.90E-17	postive
MPC1	IQCH-AS1	0.415479297	2.75E-22	postive
MPC1	ITFG2-AS1	0.328331642	4.95E-14	postive
MPC1	ITGA9-AS1	0.372768164	6.28E-18	postive
MPC1	JPX	0.339142388	6.35E-15	postive
MPC1	KANSL1-AS1	0.366859703	2.25E-17	postive
MPC1	KMT2E-AS1	0.333767674	1.78E-14	postive
MPC1	KTN1-AS1	0.330308426	3.42E-14	postive
MPC1	LINC00324	0.389030802	1.63E-19	postive
MPC1	LINC00339	0.511586987	1.07E-34	postive
MPC1	LINC00467	0.379742027	1.35E-18	postive
MPC1	LINC00476	0.430824623	5.16E-24	postive
MPC1	LINC00533	0.300025625	7.36E-12	postive
MPC1	LINC00667	0.35425026	3.14E-16	postive
MPC1	LINC00671	0.306731066	2.36E-12	postive
MPC1	LINC00680	0.382192842	7.76E-19	postive
MPC1	LINC00847	0.468662589	1.15E-28	postive
MPC1	LINC00888	0.304827049	3.27E-12	postive
MPC1	LINC00891	0.497721656	1.18E-32	postive
MPC1	LINC00909	0.401299086	9.05E-21	postive
MPC1	LINC00910	0.321890787	1.62E-13	postive

MPC1	LINC00921	0.337270153	9.12E-15	postive
MPC1	LINC00957	0.343732034	2.59E-15	postive
MPC1	LINC00963	0.351237894	5.79E-16	postive
MPC1	LINC00992	0.305655139	2.84E-12	postive
MPC1	LINC01003	0.361363162	7.20E-17	postive
MPC1	LINC01011	0.39343552	5.86E-20	postive
MPC1	LINC01023	0.393356198	5.97E-20	postive
MPC1	LINC01094	0.316409191	4.34E-13	postive
MPC1	LINC01128	0.412632088	5.61E-22	postive
MPC1	LINC01137	0.340128471	5.25E-15	postive
MPC1	LINC01150	0.314968109	5.61E-13	postive
MPC1	LINC01184	0.518182326	1.06E-35	postive
MPC1	LINC01273	0.47608535	1.19E-29	postive
MPC1	LINC01278	0.408971558	1.39E-21	postive
MPC1	LINC01412	0.325819584	7.89E-14	postive
MPC1	LINC01426	0.339465977	5.97E-15	postive
MPC1	LINC01506	0.373639155	5.19E-18	postive
MPC1	LINC01508	0.328468693	4.83E-14	postive
MPC1	LINC01558	0.323405237	1.23E-13	postive
MPC1	LINC01560	0.406228934	2.74E-21	postive
MPC1	LINC01578	0.31305551	7.87E-13	postive
MPC1	LINC01585	0.307847303	1.95E-12	postive
MPC1	LINC01632	0.310081166	1.32E-12	postive
MPC1	LINC01635	0.404414613	4.26E-21	postive
MPC1	LINC01703	0.365117898	3.26E-17	postive
MPC1	LINC01715	0.343457499	2.74E-15	postive
MPC1	LINC01806	0.349772954	7.79E-16	postive
MPC1	LINC01816	0.31601531	4.66E-13	postive
MPC1	LINC01827	0.394539195	4.52E-20	postive
MPC1	LINC01923	0.309099861	1.57E-12	postive
MPC1	LINC01942	0.310434252	1.24E-12	postive
MPC1	LINC02062	0.319467148	2.51E-13	postive
MPC1	LINC02147	0.311391311	1.05E-12	postive
MPC1	LINC02259	0.300673311	6.61E-12	postive
MPC1	LINC02285	0.361511063	6.98E-17	postive
MPC1	LINC02391	0.308656533	1.69E-12	postive
MPC1	LINC02447	0.315993162	4.68E-13	postive
MPC1	LINC02482	0.32320039	1.28E-13	postive
MPC1	LINC02519	0.338730998	6.88E-15	postive
MPC1	LINC02532	0.321566275	1.72E-13	postive
MPC1	LINC02609	0.426561658	1.59E-23	postive
MPC1	LINC02611	0.306674156	2.39E-12	postive
MPC1	LINC02637	0.333473334	1.88E-14	postive
MPC1	LINC02777	0.309514553	1.46E-12	postive
MPC1	LINC02861	0.473921787	2.32E-29	postive
MPC1	LIPE-AS1	0.357666519	1.56E-16	postive
MPC1	LMO7-AS1	0.331552982	2.71E-14	postive
MPC1	LRRC37BP1	0.337906936	8.07E-15	postive
MPC1	MACORIS	0.348461258	1.01E-15	postive
MPC1	MAN1B1-DT	0.310941468	1.14E-12	postive

MPC1	MAP4K3-DT	0.416654911	2.04E-22	postive
MPC1	MAPKAPK5-AS1	0.489963674	1.49E-31	postive
MPC1	MED14OS	0.438746461	6.11E-25	postive
MPC1	MHENCR	0.306874098	2.30E-12	postive
MPC1	MINCR	0.383760905	5.44E-19	postive
MPC1	MIPEPP3	0.318079714	3.22E-13	postive
MPC1	MIR122HG	0.32953002	3.96E-14	postive
MPC1	MIR22HG	0.383540888	5.72E-19	postive
MPC1	MIR4458HG	0.375906348	3.15E-18	postive
MPC1	MIR497HG	0.315220624	5.37E-13	postive
MPC1	MIR583HG	0.313925089	6.75E-13	postive
MPC1	MIR762HG	0.333702314	1.80E-14	postive
MPC1	MKLN1-AS	0.406342987	2.66E-21	postive
MPC1	MMADHC-DT	0.312059391	9.37E-13	postive
MPC1	MRPL20-AS1	0.431614567	4.18E-24	postive
MPC1	MRPL45P2	0.321166114	1.85E-13	postive
MPC1	MSH5-SAPCD1	0.345646718	1.78E-15	postive
MPC1	NACA4P	0.361211515	7.43E-17	postive
MPC1	NAPSB	0.316923212	3.96E-13	postive
MPC1	NBR2	0.31927487	2.60E-13	postive
MPC1	NCK1-DT	0.313891072	6.79E-13	postive
MPC1	NCMAP-DT	0.370289038	1.08E-17	postive
MPC1	NDUFB2-AS1	0.327568139	5.71E-14	postive
MPC1	NEBL-AS1	0.330910743	3.06E-14	postive
MPC1	NIFK-AS1	0.425649168	2.02E-23	postive
MPC1	NKAPP1	0.304738224	3.32E-12	postive
MPC1	NNT-AS1	0.360815378	8.08E-17	postive
MPC1	NOP14-AS1	0.345167755	1.95E-15	postive
MPC1	NRAV	0.323434605	1.22E-13	postive
MPC1	NRSN2-AS1	0.40304088	5.94E-21	postive
MPC1	NUDT16P1	0.406484223	2.57E-21	postive
MPC1	NUP50-DT	0.309041772	1.59E-12	postive
MPC1	NUTM2A-AS1	0.334336691	1.60E-14	postive
MPC1	OIP5-AS1	0.363519046	4.57E-17	postive
MPC1	OLMALINC	0.315379118	5.22E-13	postive
MPC1	OSER1-DT	0.461437035	9.86E-28	postive
MPC1	OTUD6B-AS1	0.364560089	3.67E-17	postive
MPC1	PAQR9-AS1	0.327698551	5.57E-14	postive
MPC1	PARAL1	0.320215739	2.19E-13	postive
MPC1	PAXIP1-AS1	0.320139482	2.23E-13	postive
MPC1	PAXIP1-AS2	0.380889967	1.04E-18	postive
MPC1	PCBP1-AS1	0.344866447	2.07E-15	postive
MPC1	PCCA-DT	0.360189664	9.20E-17	postive
MPC1	PDCD4-AS1	0.347475818	1.23E-15	postive
MPC1	PHKA2-AS1	0.315693829	4.93E-13	postive
MPC1	PIK3CD-AS2	0.44304762	1.87E-25	postive
MPC1	PITPNA-AS1	0.477938616	6.73E-30	postive
MPC1	PLBD1-AS1	0.311665352	1.00E-12	postive
MPC1	PMS2P4	0.436635245	1.08E-24	postive
MPC1	POLR2J4	0.386921517	2.65E-19	postive

MPC1	PRELID1P1	0.417966495	1.46E-22	postive
MPC1	PRR34	0.318177275	3.17E-13	postive
MPC1	PSMB8-AS1	0.423332621	3.68E-23	postive
MPC1	PXN-AS1	0.494244204	3.70E-32	postive
MPC1	RAB11B-AS1	0.349867635	7.64E-16	postive
MPC1	RAB30-DT	0.445450341	9.61E-26	postive
MPC1	RALY-AS1	0.336286593	1.10E-14	postive
MPC1	RAP2C-AS1	0.303032483	4.44E-12	postive
MPC1	RARA-AS1	0.34131865	4.16E-15	postive
MPC1	RASA4CP	0.306260147	2.56E-12	postive
MPC1	RBM15-AS1	0.47304914	3.03E-29	postive
MPC1	RNF216P1	0.370736248	9.76E-18	postive
MPC1	RNFT1-DT	0.353975343	3.32E-16	postive
MPC1	RP11-101E13.5	0.341848983	3.75E-15	postive
MPC1	RP11-226L15.5	0.306393695	2.50E-12	postive
MPC1	RP11-258C19.7	0.302221981	5.09E-12	postive
MPC1	RP11-462G12.2	0.332767479	2.15E-14	postive
MPC1	RP11-89K10.1	0.38047757	1.14E-18	postive
MPC1	RP4-545L17.11	0.336131079	1.13E-14	postive
MPC1	RPARP-AS1	0.410824184	8.81E-22	postive
MPC1	RPL12P38	0.304139276	3.68E-12	postive
MPC1	RPL13AP7	0.340785815	4.62E-15	postive
MPC1	RPL23AP53	0.301499575	5.75E-12	postive
MPC1	RPL23AP7	0.400942724	9.86E-21	postive
MPC1	RPL23AP82	0.484958658	7.42E-31	postive
MPC1	RPS18P9	0.37883587	1.65E-18	postive
MPC1	SAPCD1-AS1	0.333316543	1.94E-14	postive
MPC1	SBDSP1	0.433048618	2.85E-24	postive
MPC1	SBF2-AS1	0.315312698	5.28E-13	postive
MPC1	SCAMP1-AS1	0.53741543	9.21E-39	postive
MPC1	SEC24B-AS1	0.313310059	7.52E-13	postive
MPC1	SEPSECS-AS1	0.341831067	3.76E-15	postive
MPC1	SEPTIN4-AS1	0.34253349	3.28E-15	postive
MPC1	SEPTIN7P2	0.322220149	1.53E-13	postive
MPC1	SLC12A9-AS1	0.332703656	2.18E-14	postive
MPC1	SLC25A5-AS1	0.326345046	7.16E-14	postive
MPC1	SMIM25	0.314696842	5.89E-13	postive
MPC1	SNAI3-AS1	0.318400815	3.04E-13	postive
MPC1	SNHG11	0.389025763	1.63E-19	postive
MPC1	SNHG16	0.431784678	3.99E-24	postive
MPC1	SNHG20	0.319109467	2.68E-13	postive
MPC1	SNHG21	0.379126304	1.54E-18	postive
MPC1	SNHG28	0.30810932	1.86E-12	postive
MPC1	SNHG29	0.356036222	2.18E-16	postive
MPC1	SNHG30	0.414091426	3.89E-22	postive
MPC1	SNHG32	0.375360449	3.56E-18	postive
MPC1	SNHG5	0.335035919	1.40E-14	postive
MPC1	SNHG6	0.373635417	5.19E-18	postive
MPC1	SNHG7	0.326261825	7.27E-14	postive
MPC1	SNHG8	0.385122278	4.00E-19	postive

MPC1	SNHG9	0.317828969	3.37E-13	postive
MPC1	SP2-AS1	0.309211206	1.54E-12	postive
MPC1	SPINT1-AS1	0.346874401	1.39E-15	postive
MPC1	SRP14-AS1	0.373036716	5.92E-18	postive
MPC1	SSR4P1	0.326311159	7.20E-14	postive
MPC1	STAG3L4	0.476549316	1.03E-29	postive
MPC1	STARD7-AS1	0.318300285	3.10E-13	postive
MPC1	STK4-AS1	0.40781407	1.85E-21	postive
MPC1	STX17-AS1	0.386301549	3.05E-19	postive
MPC1	SUMO2P17	0.348074983	1.09E-15	postive
MPC1	SVIL-AS1	0.353232547	3.87E-16	postive
MPC1	SYNPR-AS1	0.348192424	1.07E-15	postive
MPC1	SYS1-DBNDD2	0.303699214	3.96E-12	postive
MPC1	TAF1A-AS1	0.370480035	1.03E-17	postive
MPC1	TDRKH-AS1	0.368652961	1.53E-17	postive
MPC1	TIPARP-AS1	0.338016596	7.90E-15	postive
MPC1	TMC3-AS1	0.389663529	1.41E-19	postive
MPC1	TMEM161B-AS1	0.342518942	3.29E-15	postive
MPC1	TMEM202-AS1	0.345180382	1.95E-15	postive
MPC1	TMEM44-AS1	0.348958031	9.17E-16	postive
MPC1	TMEM99	0.368255125	1.67E-17	postive
MPC1	TOB1-AS1	0.430619525	5.45E-24	postive
MPC1	TOLLIP-AS1	0.321353804	1.79E-13	postive
MPC1	TP53TG1	0.39708889	2.47E-20	postive
MPC1	TP73-AS1	0.321439658	1.76E-13	postive
MPC1	TPM3P9	0.300976386	6.28E-12	postive
MPC1	TRAF3IP2-AS1	0.339784346	5.61E-15	postive
MPC1	TRAM2-AS1	0.524154579	1.24E-36	postive
MPC1	TRIM52-AS1	0.56092474	8.84E-43	postive
MPC1	TSTD3	0.52005956	5.42E-36	postive
MPC1	TTC28-AS1	0.386031275	3.25E-19	postive
MPC1	U91328.1	0.361627399	6.81E-17	postive
MPC1	UBA6-AS1	0.369255126	1.34E-17	postive
MPC1	UBAC2-AS1	0.308053527	1.88E-12	postive
MPC1	UBE2D3-AS1	0.40852	1.56E-21	postive
MPC1	UBL7-AS1	0.326925878	6.43E-14	postive
MPC1	UBR5-AS1	0.38992454	1.33E-19	postive
MPC1	UBXN10-AS1	0.319613182	2.45E-13	postive
MPC1	UGDH-AS1	0.360475589	8.67E-17	postive
MPC1	UPK1A-AS1	0.30115072	6.10E-12	postive
MPC1	URB1-AS1	0.381841948	8.40E-19	postive
MPC1	USP27X-AS1	0.403734642	5.02E-21	postive
MPC1	UXT-AS1	0.513844996	4.86E-35	postive
MPC1	VPS13B-DT	0.32557092	8.26E-14	postive
MPC1	WAC-AS1	0.331830122	2.57E-14	postive
MPC1	WASH3P	0.353082927	3.98E-16	postive
MPC1	WDFY3-AS2	0.319984161	2.29E-13	postive
MPC1	YTHDF3-AS1	0.351975719	4.99E-16	postive
MPC1	YWHAEP7	0.301902717	5.37E-12	postive
MPC1	Z97634.1	0.37552074	3.43E-18	postive

MPC1	Z97989.1	0.313823601	6.87E-13	postive
MPC1	ZBED3-AS1	0.363157888	4.94E-17	postive
MPC1	ZBED5-AS1	0.349059496	8.99E-16	postive
MPC1	ZBTB44-DT	0.313403339	7.40E-13	postive
MPC1	ZFAS1	0.397987885	2.00E-20	postive
MPC1	ZNF205-AS1	0.334314819	1.61E-14	postive
MPC1	ZNF213-AS1	0.328136813	5.13E-14	postive
MPC1	ZNF236-DT	0.339467329	5.97E-15	postive
MPC1	ZNF252P	0.323076242	1.31E-13	postive
MPC1	ZNF337-AS1	0.306193153	2.59E-12	postive
MPC1	ZNF426-DT	0.346741266	1.43E-15	postive
MPC1	ZNF433-AS1	0.346410348	1.53E-15	postive
MPC1	ZNF542P	0.316256592	4.46E-13	postive
MPC1	ZNF561-AS1	0.466219238	2.39E-28	postive
MPC1	ZNF658B	0.301619781	5.63E-12	postive
MPC1	ZNF674-AS1	0.380198167	1.21E-18	postive
MPC1	ZNF687-AS1	0.445059929	1.07E-25	postive
MPC1	ZNF702P	0.400537785	1.09E-20	postive
MPC1	ZNF788P	0.340931749	4.49E-15	postive
MPC1	ZNF815P	0.303067549	4.41E-12	postive
MPC1	ZNRD2-AS1	0.326866783	6.50E-14	postive
MPC1	ZSCAN16-AS1	0.555523248	7.91E-42	postive
MT1DP	AC003070.2	0.376045575	3.06E-18	postive
MT1DP	AC004477.1	0.311538961	1.03E-12	postive
MT1DP	AC004930.1	0.387052256	2.57E-19	postive
MT1DP	AC004973.1	0.508155384	3.49E-34	postive
MT1DP	AC005050.1	0.309539641	1.45E-12	postive
MT1DP	AC005695.1	0.48570391	5.85E-31	postive
MT1DP	AC005871.2	0.468122817	1.35E-28	postive
MT1DP	AC006449.5	0.31507265	5.51E-13	postive
MT1DP	AC007036.1	0.690661131	4.14E-72	postive
MT1DP	AC007389.5	0.300206857	7.14E-12	postive
MT1DP	AC007541.1	0.328513165	4.79E-14	postive
MT1DP	AC007638.2	0.334754098	1.48E-14	postive
MT1DP	AC007785.1	0.372513939	6.63E-18	postive
MT1DP	AC008063.1	0.666707487	1.43E-65	postive
MT1DP	AC008267.5	0.308109023	1.86E-12	postive
MT1DP	AC008443.5	0.541619117	1.86E-39	postive
MT1DP	AC008592.1	0.446494831	7.18E-26	postive
MT1DP	AC008619.1	0.68884981	1.36E-71	postive
MT1DP	AC008991.1	0.672063437	5.58E-67	postive
MT1DP	AC009053.3	0.366089506	2.65E-17	postive
MT1DP	AC009055.2	0.615367697	1.99E-53	postive
MT1DP	AC009061.2	0.357059367	1.76E-16	postive
MT1DP	AC009118.2	0.362011165	6.28E-17	postive
MT1DP	AC009121.4	0.305037129	3.16E-12	postive
MT1DP	AC009159.3	0.331595369	2.69E-14	postive
MT1DP	AC009171.2	0.366695558	2.33E-17	postive
MT1DP	AC009275.1	0.318413504	3.04E-13	postive
MT1DP	AC009549.1	0.449910754	2.74E-26	postive

MT1DP	AC009988.1	0.543725033	8.26E-40	postive
MT1DP	AC010173.1	0.440567662	3.71E-25	postive
MT1DP	AC010203.2	0.409880015	1.11E-21	postive
MT1DP	AC010205.1	0.623559584	3.25E-55	postive
MT1DP	AC010319.5	0.574554315	2.93E-45	postive
MT1DP	AC010320.2	0.306009699	2.67E-12	postive
MT1DP	AC010504.1	0.308308259	1.80E-12	postive
MT1DP	AC010531.6	0.325971836	7.67E-14	postive
MT1DP	AC010834.2	0.410732357	9.01E-22	postive
MT1DP	AC010913.1	0.39944222	1.41E-20	postive
MT1DP	AC010931.2	0.481593032	2.15E-30	postive
MT1DP	AC011474.1	0.657291637	3.64E-63	postive
MT1DP	AC011474.2	0.689788232	7.36E-72	postive
MT1DP	AC011524.1	0.656979334	4.36E-63	postive
MT1DP	AC011603.3	0.388656825	1.78E-19	postive
MT1DP	AC012184.3	0.406153233	2.79E-21	postive
MT1DP	AC012456.2	0.369532825	1.27E-17	postive
MT1DP	AC012513.1	0.488444857	2.43E-31	postive
MT1DP	AC012555.1	0.429875289	6.63E-24	postive
MT1DP	AC013724.1	0.658252876	2.09E-63	postive
MT1DP	AC015818.2	0.301266101	5.98E-12	postive
MT1DP	AC016134.1	0.397179608	2.42E-20	postive
MT1DP	AC016737.2	0.326236115	7.30E-14	postive
MT1DP	AC016888.1	0.385479836	3.68E-19	postive
MT1DP	AC017002.1	0.394253796	4.83E-20	postive
MT1DP	AC017033.1	0.309217303	1.54E-12	postive
MT1DP	AC019069.1	0.332571332	2.23E-14	postive
MT1DP	AC020904.2	0.321818666	1.64E-13	postive
MT1DP	AC021660.2	0.304199545	3.64E-12	postive
MT1DP	AC022098.3	0.312851152	8.15E-13	postive
MT1DP	AC022167.4	0.321495348	1.74E-13	postive
MT1DP	AC022748.2	0.455385997	5.74E-27	postive
MT1DP	AC022784.3	0.417189146	1.78E-22	postive
MT1DP	AC022784.6	0.359196488	1.13E-16	postive
MT1DP	AC022915.1	0.589671611	3.77E-48	postive
MT1DP	AC023813.3	0.579828003	2.99E-46	postive
MT1DP	AC023906.5	0.537788969	8.00E-39	postive
MT1DP	AC023983.2	0.49772546	1.18E-32	postive
MT1DP	AC024361.2	0.373232334	5.67E-18	postive
MT1DP	AC024559.2	0.39760739	2.19E-20	postive
MT1DP	AC025048.2	0.320488257	2.09E-13	postive
MT1DP	AC025174.1	0.332486188	2.27E-14	postive
MT1DP	AC025287.2	0.30388872	3.84E-12	postive
MT1DP	AC026254.2	0.394076337	5.04E-20	postive
MT1DP	AC026304.1	0.426277738	1.71E-23	postive
MT1DP	AC026316.2	0.382251489	7.66E-19	postive
MT1DP	AC026691.1	0.324039199	1.09E-13	postive
MT1DP	AC027279.2	0.479287156	4.42E-30	postive
MT1DP	AC027559.1	0.577004514	1.02E-45	postive
MT1DP	AC027682.6	0.464963139	3.48E-28	postive

MT1DP	AC034114.2	0.349815132	7.72E-16	postive
MT1DP	AC036176.1	0.345130843	1.97E-15	postive
MT1DP	AC040168.1	0.316747598	4.09E-13	postive
MT1DP	AC044784.1	0.421874673	5.37E-23	postive
MT1DP	AC053503.4	0.370335453	1.06E-17	postive
MT1DP	AC064807.1	0.312120349	9.27E-13	postive
MT1DP	AC068189.1	0.492981216	5.60E-32	postive
MT1DP	AC068473.4	0.366250443	2.56E-17	postive
MT1DP	AC069155.1	0.47899007	4.85E-30	postive
MT1DP	AC069213.1	0.375511578	3.44E-18	postive
MT1DP	AC069224.2	0.392479905	7.32E-20	postive
MT1DP	AC073370.1	0.68882023	1.39E-71	postive
MT1DP	AC073529.1	0.468418066	1.24E-28	postive
MT1DP	AC073863.1	0.478375696	5.87E-30	postive
MT1DP	AC074286.1	0.50565282	8.21E-34	postive
MT1DP	AC078880.3	0.686674087	5.61E-71	postive
MT1DP	AC078880.4	0.651099251	1.25E-61	postive
MT1DP	AC078880.5	0.671427639	8.22E-67	postive
MT1DP	AC079209.1	0.621399851	9.73E-55	postive
MT1DP	AC079313.2	0.653338686	3.51E-62	postive
MT1DP	AC079598.1	0.496856936	1.57E-32	postive
MT1DP	AC079922.2	0.548921663	1.09E-40	postive
MT1DP	AC080013.5	0.466089709	2.48E-28	postive
MT1DP	AC082651.1	0.645898103	2.29E-60	postive
MT1DP	AC083867.2	0.367833073	1.83E-17	postive
MT1DP	AC084346.1	0.386326136	3.04E-19	postive
MT1DP	AC084757.3	0.683401265	4.62E-70	postive
MT1DP	AC087379.2	0.585911252	2.04E-47	postive
MT1DP	AC087482.1	0.49587901	2.17E-32	postive
MT1DP	AC090155.2	0.597143061	1.23E-49	postive
MT1DP	AC090193.1	0.582279378	1.02E-46	postive
MT1DP	AC090505.2	0.569802778	2.21E-44	postive
MT1DP	AC090587.1	0.33995936	5.42E-15	postive
MT1DP	AC090692.1	0.486269018	4.89E-31	postive
MT1DP	AC090825.1	0.314664073	5.92E-13	postive
MT1DP	AC090912.1	0.300540017	6.75E-12	postive
MT1DP	AC090970.2	0.519786075	5.97E-36	postive
MT1DP	AC091114.1	0.639791062	6.46E-59	postive
MT1DP	AC091729.3	0.351742945	5.23E-16	postive
MT1DP	AC091982.3	0.303027973	4.44E-12	postive
MT1DP	AC092115.4	0.317092455	3.85E-13	postive
MT1DP	AC092431.1	0.510499654	1.56E-34	postive
MT1DP	AC092718.2	0.330700423	3.18E-14	postive
MT1DP	AC092803.1	0.379072991	1.56E-18	postive
MT1DP	AC093520.1	0.438903351	5.85E-25	postive
MT1DP	AC093673.1	0.318323741	3.09E-13	postive
MT1DP	AC096543.2	0.646976846	1.26E-60	postive
MT1DP	AC097359.2	0.302301205	5.02E-12	postive
MT1DP	AC097372.1	0.40646601	2.58E-21	postive
MT1DP	AC097494.1	0.375240513	3.65E-18	postive

MT1DP	AC099343.4	0.390383743	1.19E-19	postive
MT1DP	AC099520.1	0.381025369	1.01E-18	postive
MT1DP	AC099522.2	0.372730388	6.33E-18	postive
MT1DP	AC099786.1	0.520527681	4.58E-36	postive
MT1DP	AC099786.2	0.465725141	2.77E-28	postive
MT1DP	AC100802.1	0.386903027	2.66E-19	postive
MT1DP	AC100803.2	0.582918278	7.69E-47	postive
MT1DP	AC100849.2	0.301091466	6.16E-12	postive
MT1DP	AC102953.1	0.351008844	6.07E-16	postive
MT1DP	AC103770.1	0.685199872	1.46E-70	postive
MT1DP	AC103855.2	0.595401771	2.76E-49	postive
MT1DP	AC103925.1	0.599311997	4.49E-50	postive
MT1DP	AC104435.2	0.535723101	1.74E-38	postive
MT1DP	AC104777.1	0.668234428	5.70E-66	postive
MT1DP	AC104794.3	0.337097051	9.43E-15	postive
MT1DP	AC106706.1	0.693858009	4.97E-73	postive
MT1DP	AC107373.1	0.474145126	2.17E-29	postive
MT1DP	AC108047.1	0.333568331	1.85E-14	postive
MT1DP	AC109347.1	0.33458837	1.52E-14	postive
MT1DP	AC113382.1	0.375065576	3.79E-18	postive
MT1DP	AC116351.1	0.541169398	2.21E-39	postive
MT1DP	AC116407.2	0.300334529	6.99E-12	postive
MT1DP	AC116609.1	0.461806119	8.85E-28	postive
MT1DP	AC116609.3	0.524250059	1.20E-36	postive
MT1DP	AC118553.1	0.372534505	6.61E-18	postive
MT1DP	AC119677.1	0.379058083	1.57E-18	postive
MT1DP	AC120498.4	0.526427308	5.45E-37	postive
MT1DP	AC124798.1	0.314409013	6.20E-13	postive
MT1DP	AC124861.1	0.382838619	6.71E-19	postive
MT1DP	AC124947.1	0.311223731	1.08E-12	postive
MT1DP	AC125603.3	0.3427007	3.18E-15	postive
MT1DP	AC128709.2	0.466895452	1.95E-28	postive
MT1DP	AC128709.3	0.468737915	1.12E-28	postive
MT1DP	AC131097.3	0.353205378	3.89E-16	postive
MT1DP	AC131235.3	0.34282519	3.10E-15	postive
MT1DP	AC131235.4	0.331899758	2.54E-14	postive
MT1DP	AC132008.2	0.409481918	1.23E-21	postive
MT1DP	AC133785.1	0.659445437	1.04E-63	postive
MT1DP	AC135048.1	0.333532408	1.86E-14	postive
MT1DP	AC135166.1	0.379295039	1.49E-18	postive
MT1DP	AC137056.1	0.620851934	1.28E-54	postive
MT1DP	AC138123.1	0.647729341	8.27E-61	postive
MT1DP	AC138207.2	0.442031494	2.48E-25	postive
MT1DP	AC144836.1	0.313984737	6.68E-13	postive
MT1DP	AC174065.1	0.345328603	1.89E-15	postive
MT1DP	AC211486.9	0.459686575	1.65E-27	postive
MT1DP	AC233280.1	0.34761255	1.20E-15	postive
MT1DP	AC243571.1	0.601447285	1.65E-50	postive
MT1DP	AC243830.1	0.378785541	1.67E-18	postive
MT1DP	AC244090.1	0.319219666	2.63E-13	postive

MT1DP	ACTN1-AS1	0.303214375	4.30E-12	postive
MT1DP	ADAMTS7P4	0.354627894	2.91E-16	postive
MT1DP	AF196972.1	0.309104576	1.57E-12	postive
MT1DP	AF274858.1	0.328303306	4.98E-14	postive
MT1DP	AF279873.4	0.660903475	4.45E-64	postive
MT1DP	AJ009632.2	0.691323451	2.68E-72	postive
MT1DP	AL008718.2	0.318722831	2.87E-13	postive
MT1DP	AL023803.1	0.353424705	3.72E-16	postive
MT1DP	AL024498.1	0.311976337	9.51E-13	postive
MT1DP	AL024507.3	0.351852819	5.12E-16	postive
MT1DP	AL031055.1	0.362884199	5.23E-17	postive
MT1DP	AL031429.1	0.686041457	8.46E-71	postive
MT1DP	AL031432.3	0.322933732	1.34E-13	postive
MT1DP	AL078590.2	0.603762633	5.51E-51	postive
MT1DP	AL096828.3	0.376330553	2.87E-18	postive
MT1DP	AL118506.1	0.314896848	5.68E-13	postive
MT1DP	AL118511.2	0.348687365	9.69E-16	postive
MT1DP	AL118523.1	0.602627222	9.44E-51	postive
MT1DP	AL121574.1	0.457645228	2.99E-27	postive
MT1DP	AL121601.1	0.403893246	4.83E-21	postive
MT1DP	AL121823.1	0.309534245	1.46E-12	postive
MT1DP	AL121827.1	0.487197169	3.63E-31	postive
MT1DP	AL121970.1	0.446043663	8.14E-26	postive
MT1DP	AL133342.1	0.479745854	3.83E-30	postive
MT1DP	AL135744.1	0.564771618	1.81E-43	postive
MT1DP	AL135785.1	0.486732471	4.21E-31	postive
MT1DP	AL136141.1	0.310556137	1.22E-12	postive
MT1DP	AL136322.1	0.331521042	2.72E-14	postive
MT1DP	AL136366.1	0.470612804	6.37E-29	postive
MT1DP	AL136501.1	0.337856282	8.15E-15	postive
MT1DP	AL136526.1	0.401714654	8.19E-21	postive
MT1DP	AL137186.2	0.35837409	1.34E-16	postive
MT1DP	AL138686.1	0.371437512	8.39E-18	postive
MT1DP	AL139035.1	0.344636855	2.17E-15	postive
MT1DP	AL139039.3	0.333064918	2.04E-14	postive
MT1DP	AL139260.1	0.628876628	2.11E-56	postive
MT1DP	AL139384.1	0.437114235	9.53E-25	postive
MT1DP	AL157937.1	0.341083597	4.36E-15	postive
MT1DP	AL158829.1	0.312251289	9.06E-13	postive
MT1DP	AL158850.1	0.344673504	2.15E-15	postive
MT1DP	AL161640.2	0.448564938	4.01E-26	postive
MT1DP	AL161668.1	0.511934257	9.47E-35	postive
MT1DP	AL161937.1	0.668714289	4.27E-66	postive
MT1DP	AL353052.1	0.32642264	7.06E-14	postive
MT1DP	AL353653.1	0.308539192	1.73E-12	postive
MT1DP	AL353743.2	0.413518298	4.50E-22	postive
MT1DP	AL354740.1	0.447026334	6.19E-26	postive
MT1DP	AL354766.2	0.418699408	1.21E-22	postive
MT1DP	AL354824.1	0.50125309	3.63E-33	postive
MT1DP	AL354824.2	0.407641666	1.93E-21	postive

MT1DP	AL354928.1	0.333628904	1.83E-14	postive
MT1DP	AL355300.1	0.69179358	1.96E-72	postive
MT1DP	AL355312.3	0.308359975	1.78E-12	postive
MT1DP	AL355512.1	0.43541061	1.51E-24	postive
MT1DP	AL355607.2	0.56489787	1.72E-43	postive
MT1DP	AL356515.1	0.323140429	1.29E-13	postive
MT1DP	AL356599.1	0.322344667	1.49E-13	postive
MT1DP	AL356966.1	0.434779979	1.79E-24	postive
MT1DP	AL357033.2	0.692493093	1.23E-72	postive
MT1DP	AL357146.1	0.395831579	3.33E-20	postive
MT1DP	AL357992.1	0.503122056	1.93E-33	postive
MT1DP	AL358472.2	0.341222339	4.24E-15	postive
MT1DP	AL358852.1	0.304159148	3.67E-12	postive
MT1DP	AL359182.1	0.373796018	5.01E-18	postive
MT1DP	AL359962.1	0.55366728	1.66E-41	postive
MT1DP	AL359962.2	0.417111692	1.82E-22	postive
MT1DP	AL365181.1	0.449924375	2.73E-26	postive
MT1DP	AL390955.2	0.398986027	1.58E-20	postive
MT1DP	AL391832.3	0.351914961	5.05E-16	postive
MT1DP	AL441992.2	0.415056009	3.06E-22	postive
MT1DP	AL442128.2	0.318671422	2.90E-13	postive
MT1DP	AL442663.3	0.308184646	1.84E-12	postive
MT1DP	AL445228.3	0.391292495	9.66E-20	postive
MT1DP	AL451069.3	0.530956499	1.03E-37	postive
MT1DP	AL451074.2	0.376810779	2.58E-18	postive
MT1DP	AL512625.2	0.300993702	6.26E-12	postive
MT1DP	AL583722.1	0.314338954	6.27E-13	postive
MT1DP	AL590004.3	0.340456503	4.92E-15	postive
MT1DP	AL590428.1	0.677688598	1.72E-68	postive
MT1DP	AL590705.1	0.326815957	6.56E-14	postive
MT1DP	AL603839.2	0.302967571	4.49E-12	postive
MT1DP	AL606489.1	0.346773402	1.42E-15	postive
MT1DP	AL732323.1	0.304947542	3.21E-12	postive
MT1DP	AL772202.1	0.470332619	6.93E-29	postive
MT1DP	ALDH1L1-AS2	0.312622127	8.49E-13	postive
MT1DP	ALG1L13P	0.330369764	3.38E-14	postive
MT1DP	AMZ2P1	0.330139588	3.53E-14	postive
MT1DP	ANO7L1	0.344278138	2.33E-15	postive
MT1DP	AP000229.1	0.401102204	9.49E-21	postive
MT1DP	AP000251.1	0.367972847	1.77E-17	postive
MT1DP	AP000253.1	0.461829135	8.79E-28	postive
MT1DP	AP000640.2	0.449571865	3.02E-26	postive
MT1DP	AP000880.1	0.309924725	1.36E-12	postive
MT1DP	AP000911.1	0.364639143	3.61E-17	postive
MT1DP	AP001063.1	0.449258243	3.30E-26	postive
MT1DP	AP001189.5	0.658381466	1.94E-63	postive
MT1DP	AP001453.3	0.301343424	5.90E-12	postive
MT1DP	AP001636.3	0.346892031	1.39E-15	postive
MT1DP	AP002008.3	0.672127929	5.36E-67	postive
MT1DP	AP003465.2	0.31015249	1.31E-12	postive

MT1DP	AP003469.1	0.332133861	2.43E-14	postive
MT1DP	AP003469.2	0.432501568	3.30E-24	postive
MT1DP	AP005229.2	0.312120764	9.27E-13	postive
MT1DP	AP005242.1	0.40949566	1.22E-21	postive
MT1DP	AP006621.3	0.491774873	8.29E-32	postive
MT1DP	ARRDC1-AS1	0.318520517	2.98E-13	postive
MT1DP	BTG3-AS1	0.332217866	2.39E-14	postive
MT1DP	BX470209.1	0.660529102	5.54E-64	postive
MT1DP	BX470209.2	0.675169515	8.24E-68	postive
MT1DP	BX640514.2	0.478922469	4.96E-30	postive
MT1DP	C1QTNF1-AS1	0.535820705	1.68E-38	postive
MT1DP	C5orf60	0.349775143	7.78E-16	postive
MT1DP	C6orf99	0.445178839	1.04E-25	postive
MT1DP	C9orf170	0.41448757	3.53E-22	postive
MT1DP	CAMTA1-DT	0.388293216	1.93E-19	postive
MT1DP	CCDC84-DT	0.336253556	1.11E-14	postive
MT1DP	CEACAMP10	0.598720785	5.91E-50	postive
MT1DP	CEBPB-AS1	0.307777182	1.97E-12	postive
MT1DP	CEP83-DT	0.323170732	1.28E-13	postive
MT1DP	CERS3-AS1	0.448578536	4.00E-26	postive
MT1DP	CES5AP1	0.302460724	4.89E-12	postive
MT1DP	CSTF3-DT	0.320944372	1.92E-13	postive
MT1DP	CT66	0.311127192	1.10E-12	postive
MT1DP	CTD-2521M24.9	0.324177033	1.07E-13	postive
MT1DP	CTD-2540M10.1	0.608499373	5.71E-52	postive
MT1DP	CYP4F23P	0.574040192	3.65E-45	postive
MT1DP	DBIL5P	0.383327274	6.01E-19	postive
MT1DP	DCST1-AS1	0.349213801	8.71E-16	postive
MT1DP	DEC1	0.308727537	1.67E-12	postive
MT1DP	DEPDC1-AS1	0.343636443	2.64E-15	postive
MT1DP	DNAJC3-DT	0.410966252	8.50E-22	postive
MT1DP	DNM1P47	0.390110416	1.27E-19	postive
MT1DP	DOCK9-DT	0.35989018	9.80E-17	postive
MT1DP	ELDR	0.435164137	1.61E-24	postive
MT1DP	EPHA1-AS1	0.318663645	2.90E-13	postive
MT1DP	ERVMER61-1	0.373398574	5.47E-18	postive
MT1DP	EXOC3-AS1	0.342600381	3.24E-15	postive
MT1DP	EXOSC10-AS1	0.437744991	8.03E-25	postive
MT1DP	F10-AS1	0.563013256	3.75E-43	postive
MT1DP	FALEC	0.324676578	9.74E-14	postive
MT1DP	FAM197Y9	0.515639508	2.59E-35	postive
MT1DP	FAM21FP	0.30679229	2.34E-12	postive
MT1DP	FAM230C	0.349139019	8.85E-16	postive
MT1DP	FAM86JP	0.338430739	7.29E-15	postive
MT1DP	FBP2P1	0.689381336	9.61E-72	postive
MT1DP	FO393418.1	0.379973309	1.28E-18	postive
MT1DP	FOXD3-AS1	0.501827836	2.99E-33	postive
MT1DP	FZD4-DT	0.319776877	2.38E-13	postive
MT1DP	GAS6-DT	0.402002499	7.64E-21	postive
MT1DP	GBAP1	0.379142703	1.54E-18	postive

MT1DP	GIHCG	0.410422686	9.73E-22	postive
MT1DP	GOLGA8EP	0.614157299	3.61E-53	postive
MT1DP	GYG2P1	0.302781206	4.63E-12	postive
MT1DP	H2AC10P	0.397399832	2.30E-20	postive
MT1DP	HMMR-AS1	0.371562393	8.16E-18	postive
MT1DP	HUNK-AS1	0.318956386	2.75E-13	postive
MT1DP	IDH1-AS1	0.355941009	2.22E-16	postive
MT1DP	IGFL2-AS1	0.596407443	1.73E-49	postive
MT1DP	IPO9-AS1	0.390466053	1.17E-19	postive
MT1DP	KCNMB2-AS1	0.384491757	4.61E-19	postive
MT1DP	KCNQ1DN	0.558595739	2.29E-42	postive
MT1DP	KDM2B-DT	0.391248154	9.76E-20	postive
MT1DP	KDM7A-DT	0.357733411	1.53E-16	postive
MT1DP	KRT8P12	0.475784485	1.31E-29	postive
MT1DP	KTN1-AS1	0.356415365	2.01E-16	postive
MT1DP	L29074.1	0.346046135	1.64E-15	postive
MT1DP	LAMA5-AS1	0.680094636	3.78E-69	postive
MT1DP	LBX2-AS1	0.306428617	2.49E-12	postive
MT1DP	LGALS8-AS1	0.442905191	1.95E-25	postive
MT1DP	LINC00239	0.419671195	9.46E-23	postive
MT1DP	LINC00293	0.338937153	6.61E-15	postive
MT1DP	LINC00323	0.488906165	2.10E-31	postive
MT1DP	LINC00346	0.493542864	4.66E-32	postive
MT1DP	LINC00466	0.639948899	5.93E-59	postive
MT1DP	LINC00471	0.329503665	3.98E-14	postive
MT1DP	LINC00494	0.650287458	1.97E-61	postive
MT1DP	LINC00592	0.305072292	3.14E-12	postive
MT1DP	LINC00599	0.427292361	1.31E-23	postive
MT1DP	LINC00630	0.314034166	6.62E-13	postive
MT1DP	LINC00659	0.515245049	2.98E-35	postive
MT1DP	LINC00668	0.352380104	4.60E-16	postive
MT1DP	LINC00707	0.42262226	4.43E-23	postive
MT1DP	LINC00847	0.499048197	7.58E-33	postive
MT1DP	LINC00853	0.347688629	1.18E-15	postive
MT1DP	LINC00884	0.627599051	4.08E-56	postive
MT1DP	LINC00885	0.477757662	7.12E-30	postive
MT1DP	LINC00887	0.481141278	2.48E-30	postive
MT1DP	LINC00888	0.43404708	2.18E-24	postive
MT1DP	LINC00906	0.585016263	3.04E-47	postive
MT1DP	LINC01028	0.368500869	1.58E-17	postive
MT1DP	LINC01031	0.372142466	7.19E-18	postive
MT1DP	LINC01059	0.351032387	6.04E-16	postive
MT1DP	LINC01063	0.660497446	5.64E-64	postive
MT1DP	LINC01093	0.472293877	3.82E-29	postive
MT1DP	LINC01132	0.37176556	7.81E-18	postive
MT1DP	LINC01137	0.362576292	5.58E-17	postive
MT1DP	LINC01151	0.681806796	1.28E-69	postive
MT1DP	LINC01154	0.427079958	1.39E-23	postive
MT1DP	LINC01232	0.60810949	6.89E-52	postive
MT1DP	LINC01273	0.590865289	2.20E-48	postive

MT1DP	LINC01275	0.695611994	1.53E-73	postive
MT1DP	LINC01285	0.55870364	2.19E-42	postive
MT1DP	LINC01293	0.609839802	2.98E-52	postive
MT1DP	LINC01298	0.52684332	4.68E-37	postive
MT1DP	LINC01323	0.356234078	2.09E-16	postive
MT1DP	LINC01351	0.319805984	2.36E-13	postive
MT1DP	LINC01354	0.683965264	3.22E-70	postive
MT1DP	LINC01366	0.681113295	1.99E-69	postive
MT1DP	LINC01387	0.659046625	1.31E-63	postive
MT1DP	LINC01443	0.497202274	1.40E-32	postive
MT1DP	LINC01446	0.325164451	8.90E-14	postive
MT1DP	LINC01497	0.65237812	6.06E-62	postive
MT1DP	LINC01509	0.316993407	3.91E-13	postive
MT1DP	LINC01523	0.42209495	5.07E-23	postive
MT1DP	LINC01567	0.317345534	3.68E-13	postive
MT1DP	LINC01585	0.506533317	6.08E-34	postive
MT1DP	LINC01609	0.340639641	4.75E-15	postive
MT1DP	LINC01621	0.655791093	8.64E-63	postive
MT1DP	LINC01686	0.541429848	2.00E-39	postive
MT1DP	LINC01693	0.712570352	1.13E-78	postive
MT1DP	LINC01703	0.433180244	2.75E-24	postive
MT1DP	LINC01771	0.319711103	2.40E-13	postive
MT1DP	LINC01842	0.691395071	2.55E-72	postive
MT1DP	LINC01855	0.445866369	8.56E-26	postive
MT1DP	LINC01914	0.301812379	5.45E-12	postive
MT1DP	LINC01952	0.416249655	2.26E-22	postive
MT1DP	LINC01968	0.487611575	3.18E-31	postive
MT1DP	LINC01972	0.392341941	7.56E-20	postive
MT1DP	LINC01975	0.323167008	1.28E-13	postive
MT1DP	LINC02026	0.437796227	7.91E-25	postive
MT1DP	LINC02028	0.415953519	2.44E-22	postive
MT1DP	LINC02076	0.541615795	1.86E-39	postive
MT1DP	LINC02099	0.456483959	4.18E-27	postive
MT1DP	LINC02130	0.543106621	1.05E-39	postive
MT1DP	LINC02137	0.698294886	2.50E-74	postive
MT1DP	LINC02238	0.547847701	1.66E-40	postive
MT1DP	LINC02243	0.537449717	9.09E-39	postive
MT1DP	LINC02269	0.361620226	6.82E-17	postive
MT1DP	LINC02321	0.300205592	7.14E-12	postive
MT1DP	LINC02369	0.404559246	4.11E-21	postive
MT1DP	LINC02412	0.69391699	4.78E-73	postive
MT1DP	LINC02541	0.344401856	2.27E-15	postive
MT1DP	LINC02570	0.395227036	3.84E-20	postive
MT1DP	LINC02574	0.310019276	1.34E-12	postive
MT1DP	LINC02601	0.448958303	3.59E-26	postive
MT1DP	LINC02609	0.303464873	4.12E-12	postive
MT1DP	LINC02637	0.374472815	4.32E-18	postive
MT1DP	LINC02707	0.688394103	1.83E-71	postive
MT1DP	LINC02709	0.425849141	1.91E-23	postive
MT1DP	LINC02731	0.369096477	1.39E-17	postive

MT1DP	LINC02740	0.639616761	7.10E-59	postive
MT1DP	LINC02745	0.567528987	5.74E-44	postive
MT1DP	LINC02757	0.666164149	1.98E-65	postive
MT1DP	LINC02765	0.318518842	2.98E-13	postive
MT1DP	LINC02766	0.31477585	5.81E-13	postive
MT1DP	LINC02817	0.336008955	1.16E-14	postive
MT1DP	LINC02851	0.312770027	8.27E-13	postive
MT1DP	LINC02861	0.345547354	1.81E-15	postive
MT1DP	LMNB1-DT	0.412275698	6.14E-22	postive
MT1DP	LPP-AS2	0.580454532	2.27E-46	postive
MT1DP	LUNAR1	0.419908818	8.90E-23	postive
MT1DP	LY6E-DT	0.36259438	5.56E-17	postive
MT1DP	MAFTRR	0.357220126	1.71E-16	postive
MT1DP	MAP3K2-DT	0.367445671	1.98E-17	postive
MT1DP	MAPK6-DT	0.492247278	7.11E-32	postive
MT1DP	MED14OS	0.302547034	4.82E-12	postive
MT1DP	MELTF-AS1	0.448854458	3.70E-26	postive
MT1DP	MIR1915HG	0.34380685	2.55E-15	postive
MT1DP	MIR3150BHG	0.653635857	2.96E-62	postive
MT1DP	MIR3681HG	0.51787093	1.18E-35	postive
MT1DP	MIR924HG	0.467269728	1.75E-28	postive
MT1DP	MLIP-IT1	0.349113916	8.89E-16	postive
MT1DP	MT1DP	1	0	postive
MT1DP	MT1L	0.485634389	5.98E-31	postive
MT1DP	NEBL-AS1	0.373839609	4.97E-18	postive
MT1DP	NKAPP1	0.332548162	2.24E-14	postive
MT1DP	NLGN4Y-AS1	0.51774361	1.23E-35	postive
MT1DP	NUDT16P1	0.324029604	1.10E-13	postive
MT1DP	NUP50-DT	0.426797592	1.49E-23	postive
MT1DP	OR4G11P	0.692557871	1.18E-72	postive
MT1DP	OR4G2P	0.653687979	2.88E-62	postive
MT1DP	OSER1-DT	0.338927059	6.62E-15	postive
MT1DP	OXCT1-AS1	0.510132459	1.77E-34	postive
MT1DP	PAUPAR	0.390454574	1.17E-19	postive
MT1DP	PCCA-DT	0.416646349	2.04E-22	postive
MT1DP	PRKAR2A-AS1	0.310833263	1.16E-12	postive
MT1DP	RAET1E-AS1	0.379441508	1.44E-18	postive
MT1DP	RAMP2-AS1	0.434209825	2.09E-24	postive
MT1DP	RAP2CP1	0.321974368	1.60E-13	postive
MT1DP	RASSF8-AS1	0.36741284	2.00E-17	postive
MT1DP	RBM26-AS1	0.311894552	9.65E-13	postive
MT1DP	RBMY2QP	0.344457743	2.25E-15	postive
MT1DP	REG1CP	0.420284901	8.08E-23	postive
MT1DP	RP11-115J16.1	0.691631787	2.18E-72	postive
MT1DP	RP11-14C10.5	0.389836441	1.35E-19	postive
MT1DP	RP11-254F7.4	0.335489215	1.28E-14	postive
MT1DP	RP11-335E6.4	0.368557713	1.56E-17	postive
MT1DP	RP11-462G12.2	0.325702085	8.06E-14	postive
MT1DP	RP11-467J12.4	0.300419386	6.89E-12	postive
MT1DP	RP11-543B16.3	0.494661235	3.23E-32	postive

MT1DP	RP3-418C23.2	0.321329696	1.79E-13	postive
MT1DP	SAP30-DT	0.347327239	1.27E-15	postive
MT1DP	SCAMP1-AS1	0.32951232	3.97E-14	postive
MT1DP	SEPTIN4-AS1	0.435760004	1.37E-24	postive
MT1DP	SLC25A5-AS1	0.396958815	2.55E-20	postive
MT1DP	SPATA41	0.342648585	3.21E-15	postive
MT1DP	ST3GAL6-AS1	0.412102191	6.41E-22	postive
MT1DP	STK24-AS1	0.611498952	1.33E-52	postive
MT1DP	STK4-AS1	0.414988459	3.11E-22	postive
MT1DP	TAF1A-AS1	0.31813887	3.19E-13	postive
MT1DP	TCAM1P	0.341554932	3.97E-15	postive
MT1DP	TEX36-AS1	0.461181307	1.06E-27	postive
MT1DP	THAP7-AS1	0.312764547	8.28E-13	postive
MT1DP	TM4SF19-AS1	0.587386373	1.05E-47	postive
MT1DP	TMC3-AS1	0.393708968	5.49E-20	postive
MT1DP	TMCC1-AS1	0.538752905	5.55E-39	postive
MT1DP	TMEM44-AS1	0.413247352	4.81E-22	postive
MT1DP	TMLHE-AS1	0.374971826	3.87E-18	postive
MT1DP	TMPO-AS1	0.492385571	6.80E-32	postive
MT1DP	TPRG1-AS1	0.612654058	7.57E-53	postive
MT1DP	TTTY15	0.311238711	1.08E-12	postive
MT1DP	TTTY18	0.529278581	1.92E-37	postive
MT1DP	TTTY20	0.673724898	2.01E-67	postive
MT1DP	TUBBP9	0.67781731	1.58E-68	postive
MT1DP	TULP3P1	0.509519407	2.18E-34	postive
MT1DP	TYMSOS	0.43294022	2.93E-24	postive
MT1DP	ULK4P3	0.344788078	2.10E-15	postive
MT1DP	URB1-AS1	0.346610292	1.47E-15	postive
MT1DP	USP12-AS2	0.304235657	3.62E-12	postive
MT1DP	USP27X-AS1	0.364284783	3.89E-17	postive
MT1DP	UXT-AS1	0.41039335	9.81E-22	postive
MT1DP	WWTR1-AS1	0.509592402	2.13E-34	postive
MT1DP	Z82185.1	0.59231411	1.14E-48	postive
MT1DP	Z82246.1	0.535971479	1.59E-38	postive
MT1DP	Z92544.1	0.480216454	3.31E-30	postive
MT1DP	ZNF788P	0.325549935	8.29E-14	postive
MT1X	AC002401.4	0.42414134	2.99E-23	postive
MT1X	AC004917.1	0.325016093	9.15E-14	postive
MT1X	AC008103.1	0.385273047	3.86E-19	postive
MT1X	AC009549.1	0.413077646	5.02E-22	postive
MT1X	AC010173.1	0.397222963	2.40E-20	postive
MT1X	AC011451.1	0.395643634	3.48E-20	postive
MT1X	AC017033.1	0.311299636	1.07E-12	postive
MT1X	AC027319.1	0.332950234	2.08E-14	postive
MT1X	AC046143.1	0.331040422	2.98E-14	postive
MT1X	AC068228.2	0.336734954	1.01E-14	postive
MT1X	AC073611.1	0.342496034	3.31E-15	postive
MT1X	AC079598.1	0.360828031	8.05E-17	postive
MT1X	AC082651.3	0.304384879	3.53E-12	postive
MT1X	AC090673.1	0.325024365	9.13E-14	postive

MT1X	AC091138.1	0.330919086	3.05E-14	postive
MT1X	AC092718.4	0.365187117	3.21E-17	postive
MT1X	AC093673.1	0.474237853	2.11E-29	postive
MT1X	AC097518.1	0.524886612	9.54E-37	postive
MT1X	AC104653.1	0.309463249	1.47E-12	postive
MT1X	AC112220.2	0.304645398	3.37E-12	postive
MT1X	AC117462.1	0.363095484	5.00E-17	postive
MT1X	AC139722.1	0.327867032	5.40E-14	postive
MT1X	AGAP2-AS1	0.327047069	6.29E-14	postive
MT1X	AL049541.2	0.305937976	2.71E-12	postive
MT1X	AL137186.2	0.317112528	3.83E-13	postive
MT1X	AL138724.2	0.376144101	2.99E-18	postive
MT1X	AL139220.2	0.422837228	4.19E-23	postive
MT1X	AL356515.1	0.325204513	8.83E-14	postive
MT1X	AL365203.2	0.369403578	1.30E-17	postive
MT1X	AL390719.1	0.366483612	2.44E-17	postive
MT1X	AL441992.2	0.409892969	1.11E-21	postive
MT1X	AL450322.2	0.339567023	5.85E-15	postive
MT1X	AP000695.1	0.309139998	1.56E-12	postive
MT1X	AP000695.2	0.335721954	1.23E-14	postive
MT1X	AP001816.1	0.381375988	9.33E-19	postive
MT1X	AP001922.5	0.301447188	5.80E-12	postive
MT1X	AP003555.1	0.304909501	3.23E-12	postive
MT1X	AP005121.2	0.328552806	4.75E-14	postive
MT1X	AP005137.2	0.307550451	2.05E-12	postive
MT1X	AP006565.1	0.332404135	2.31E-14	postive
MT1X	APCDD1L-DT	0.336641719	1.03E-14	postive
MT1X	ASIC4-AS1	0.32369623	1.17E-13	postive
MT1X	C1QTNF1-AS1	0.399465791	1.41E-20	postive
MT1X	CIDECP1	0.374799123	4.02E-18	postive
MT1X	CNIH3-AS2	0.369118261	1.38E-17	postive
MT1X	CYTOR	0.486574587	4.43E-31	postive
MT1X	DEPDC1-AS1	0.371880257	7.62E-18	postive
MT1X	GSEC	0.369931698	1.16E-17	postive
MT1X	JPX	0.319461728	2.51E-13	postive
MT1X	LINC00313	0.308920671	1.62E-12	postive
MT1X	LINC00973	0.32389433	1.12E-13	postive
MT1X	LINC01137	0.334549646	1.54E-14	postive
MT1X	LINC01218	0.397437671	2.28E-20	postive
MT1X	LINC01303	0.344133735	2.40E-15	postive
MT1X	LINC01443	0.406892767	2.33E-21	postive
MT1X	LINC01444	0.408676339	1.50E-21	postive
MT1X	LINC01705	0.34133986	4.14E-15	postive
MT1X	LINC01792	0.306474093	2.47E-12	postive
MT1X	LINC02584	0.304900793	3.23E-12	postive
MT1X	LINC02742	0.331622714	2.67E-14	postive
MT1X	LINC02802	0.304204793	3.64E-12	postive
MT1X	LIPE-AS1	0.308711911	1.68E-12	postive
MT1X	LSP1P4	0.360553424	8.53E-17	postive
MT1X	MIR193BHG	0.425294274	2.21E-23	postive

MT1X	MIR4435-2HG	0.484406422	8.84E-31	postive
MT1X	MT1JP	0.426140204	1.77E-23	postive
MT1X	MT1L	0.426135574	1.78E-23	postive
MT1X	NRAV	0.379610843	1.39E-18	postive
MT1X	NUDT16P1	0.302882029	4.55E-12	postive
MT1X	PPP1R14B-AS1	0.308130272	1.86E-12	postive
MT1X	PPP1R2P1	0.312837595	8.17E-13	postive
MT1X	PYCARD-AS1	0.407000994	2.26E-21	postive
MT1X	RASAL2-AS1	0.361468777	7.04E-17	postive
MT1X	RNF216P1	0.323826038	1.14E-13	postive
MT1X	RP11-102K13.5	0.350922252	6.18E-16	postive
MT1X	RP11-10C24.1	0.326737516	6.66E-14	postive
MT1X	RP11-556E13.1	0.339745294	5.65E-15	postive
MT1X	RPL23AP82	0.300079	7.30E-12	postive
MT1X	RPSAP52	0.418090605	1.42E-22	postive
MT1X	SAP30-DT	0.325520021	8.34E-14	postive
MT1X	SERPINB9P1	0.302584256	4.79E-12	postive
MT1X	SIRLNT	0.393263501	6.10E-20	postive
MT1X	SLCO4A1-AS1	0.425047058	2.36E-23	postive
MT1X	STXBP5-AS1	0.350850851	6.27E-16	postive
MT1X	TP53TG1	0.349236716	8.67E-16	postive
MT1X	UBA6-AS1	0.321803598	1.65E-13	postive
MT1X	ZNF561-AS1	0.342837296	3.09E-15	postive
MT3	AC003001.1	0.314267688	6.35E-13	postive
MT3	AC004080.4	0.319629906	2.44E-13	postive
MT3	AC004522.4	0.326853294	6.52E-14	postive
MT3	AC004882.2	0.313498462	7.28E-13	postive
MT3	AC005393.1	0.304347041	3.55E-12	postive
MT3	AC006960.3	0.346000197	1.66E-15	postive
MT3	AC006960.4	0.44235875	2.27E-25	postive
MT3	AC007423.1	0.30346632	4.12E-12	postive
MT3	AC008825.1	0.365314139	3.13E-17	postive
MT3	AC008938.1	0.328500925	4.80E-14	postive
MT3	AC010275.1	0.327516175	5.76E-14	postive
MT3	AC012640.4	0.308581007	1.72E-12	postive
MT3	AC013287.1	0.349634918	8.01E-16	postive
MT3	AC013401.1	0.307891799	1.93E-12	postive
MT3	AC016394.2	0.336690587	1.02E-14	postive
MT3	AC016822.1	0.300598922	6.69E-12	postive
MT3	AC018766.1	0.327821103	5.45E-14	postive
MT3	AC019064.1	0.545224729	4.62E-40	postive
MT3	AC021242.2	0.465973383	2.57E-28	postive
MT3	AC021504.1	0.387184255	2.49E-19	postive
MT3	AC022762.1	0.310227969	1.29E-12	postive
MT3	AC023510.2	0.322417108	1.47E-13	postive
MT3	AC024681.2	0.395411871	3.68E-20	postive
MT3	AC026803.2	0.337532177	8.67E-15	postive
MT3	AC034226.1	0.327415253	5.87E-14	postive
MT3	AC046168.2	0.310347066	1.26E-12	postive
MT3	AC063948.1	0.524665386	1.03E-36	postive

MT3	AC066612.1	0.331508085	2.73E-14	postive
MT3	AC068620.1	0.300960514	6.29E-12	postive
MT3	AC068875.1	0.421397296	6.07E-23	postive
MT3	AC069277.1	0.364779989	3.50E-17	postive
MT3	AC078882.1	0.559231673	1.77E-42	postive
MT3	AC079310.1	0.324188326	1.06E-13	postive
MT3	AC079942.1	0.438759777	6.09E-25	postive
MT3	AC083864.1	0.342908753	3.05E-15	postive
MT3	AC087379.1	0.351899467	5.07E-16	postive
MT3	AC090386.1	0.318060577	3.23E-13	postive
MT3	AC090398.1	0.388467258	1.86E-19	postive
MT3	AC090709.1	0.38298516	6.49E-19	postive
MT3	AC090735.1	0.449613182	2.99E-26	postive
MT3	AC092506.1	0.317914647	3.32E-13	postive
MT3	AC092755.1	0.357586578	1.58E-16	postive
MT3	AC092979.1	0.57270456	6.45E-45	postive
MT3	AC093763.1	0.306295298	2.55E-12	postive
MT3	AC096540.1	0.320703205	2.01E-13	postive
MT3	AC098484.2	0.330499774	3.30E-14	postive
MT3	AC099343.4	0.302856767	4.57E-12	postive
MT3	AC100793.4	0.388030486	2.05E-19	postive
MT3	AC100802.1	0.310504592	1.23E-12	postive
MT3	AC104078.2	0.39231516	7.61E-20	postive
MT3	AC106729.1	0.323580081	1.19E-13	postive
MT3	AC108704.1	0.391482343	9.24E-20	postive
MT3	AC112219.1	0.564383517	2.13E-43	postive
MT3	AC113208.4	0.341116111	4.33E-15	postive
MT3	AC119674.1	0.354497459	2.99E-16	postive
MT3	AC124254.2	0.329712746	3.83E-14	postive
MT3	AC126178.1	0.468059817	1.38E-28	postive
MT3	AC130371.2	0.30045124	6.86E-12	postive
MT3	AC131159.2	0.371148221	8.93E-18	postive
MT3	AC134508.1	0.496604839	1.70E-32	postive
MT3	AC136604.3	0.30196258	5.32E-12	postive
MT3	AC138024.1	0.338467175	7.24E-15	postive
MT3	AC144652.1	0.314031131	6.62E-13	postive
MT3	AC144831.1	0.391099257	1.01E-19	postive
MT3	AL008638.4	0.319227839	2.62E-13	postive
MT3	AL008638.5	0.302721173	4.68E-12	postive
MT3	AL020994.1	0.352872069	4.16E-16	postive
MT3	AL022316.1	0.41854107	1.26E-22	postive
MT3	AL022334.2	0.544359822	6.46E-40	postive
MT3	AL031658.1	0.46407976	4.52E-28	postive
MT3	AL035425.2	0.425149644	2.30E-23	postive
MT3	AL035425.4	0.367247467	2.07E-17	postive
MT3	AL050341.2	0.441240711	3.09E-25	postive
MT3	AL078602.1	0.355152764	2.61E-16	postive
MT3	AL109976.1	0.363076611	5.02E-17	postive
MT3	AL117335.1	0.465489834	2.97E-28	postive
MT3	AL117350.2	0.314060581	6.59E-13	postive

MT3	AL122010.1	0.332200378	2.40E-14	postive
MT3	AL133396.2	0.337224124	9.20E-15	postive
MT3	AL135785.1	0.391088673	1.01E-19	postive
MT3	AL136018.1	0.384029701	5.12E-19	postive
MT3	AL136537.1	0.336911914	9.77E-15	postive
MT3	AL138889.1	0.321567679	1.72E-13	postive
MT3	AL139093.1	0.327871729	5.39E-14	postive
MT3	AL139286.2	0.376257226	2.92E-18	postive
MT3	AL160411.2	0.362120837	6.14E-17	postive
MT3	AL353681.1	0.332333746	2.34E-14	postive
MT3	AL353732.1	0.362647136	5.50E-17	postive
MT3	AL355482.1	0.387366787	2.39E-19	postive
MT3	AL356488.3	0.345310588	1.90E-15	postive
MT3	AL357079.1	0.395406766	3.68E-20	postive
MT3	AL358075.2	0.363875194	4.24E-17	postive
MT3	AL358394.3	0.330315067	3.42E-14	postive
MT3	AL359915.1	0.314384062	6.22E-13	postive
MT3	AL360175.1	0.384140224	5.00E-19	postive
MT3	AL390955.2	0.317209216	3.77E-13	postive
MT3	AL450306.1	0.312876756	8.12E-13	postive
MT3	AL512274.1	0.412101962	6.41E-22	postive
MT3	AL591167.1	0.555036281	9.62E-42	postive
MT3	AL604028.1	0.322990857	1.33E-13	postive
MT3	AL606760.3	0.370856751	9.51E-18	postive
MT3	AL713866.1	0.378215279	1.89E-18	postive
MT3	AP000802.1	0.323241899	1.27E-13	postive
MT3	AP000845.1	0.385698986	3.50E-19	postive
MT3	AP000851.2	0.418506535	1.27E-22	postive
MT3	AP001150.1	0.402577913	6.65E-21	postive
MT3	AP001636.3	0.436790004	1.04E-24	postive
MT3	AP002840.1	0.556458026	5.43E-42	postive
MT3	AP005061.2	0.361748535	6.64E-17	postive
MT3	AP006216.1	0.385326153	3.81E-19	postive
MT3	AP006545.1	0.314492681	6.11E-13	postive
MT3	ARNILA	0.399869287	1.28E-20	postive
MT3	AZGP1P1	0.39231537	7.61E-20	postive
MT3	BVES-AS1	0.35700977	1.78E-16	postive
MT3	CACNA2D3-AS1	0.304534739	3.44E-12	postive
MT3	CASC17	0.460104556	1.46E-27	postive
MT3	CDKN2B-AS1	0.450957717	2.04E-26	postive
MT3	CLCN3P1	0.338581632	7.08E-15	postive
MT3	DLG3-AS1	0.374016251	4.78E-18	postive
MT3	DNAJB3	0.421297494	6.23E-23	postive
MT3	FAM182A	0.519007656	7.88E-36	postive
MT3	FOXD2-AS1	0.323985923	1.11E-13	postive
MT3	FP236315.1	0.353506497	3.66E-16	postive
MT3	FP325330.3	0.36535935	3.10E-17	postive
MT3	FRMD6-AS1	0.309847009	1.38E-12	postive
MT3	HOTAIRM1	0.372563958	6.56E-18	postive
MT3	HUNK-AS1	0.437559172	8.44E-25	postive

MT3	IPO5P1	0.31561217	5.01E-13	postive
MT3	KCNJ2-AS1	0.380920853	1.03E-18	postive
MT3	LIFR-AS1	0.308584069	1.72E-12	postive
MT3	LINC00221	0.320688506	2.01E-13	postive
MT3	LINC00393	0.362314214	5.90E-17	postive
MT3	LINC00566	0.405051591	3.65E-21	postive
MT3	LINC00877	0.3966082	2.77E-20	postive
MT3	LINC00879	0.340787374	4.62E-15	postive
MT3	LINC00885	0.375987507	3.10E-18	postive
MT3	LINC00898	0.309057038	1.58E-12	postive
MT3	LINC01036	0.354700663	2.86E-16	postive
MT3	LINC01037	0.404042946	4.66E-21	postive
MT3	LINC01144	0.383903236	5.27E-19	postive
MT3	LINC01189	0.310818616	1.16E-12	postive
MT3	LINC01198	0.421967187	5.24E-23	postive
MT3	LINC01255	0.420546821	7.56E-23	postive
MT3	LINC01305	0.434299478	2.04E-24	postive
MT3	LINC01326	0.558378516	2.50E-42	postive
MT3	LINC01344	0.384104623	5.04E-19	postive
MT3	LINC01360	0.554394873	1.24E-41	postive
MT3	LINC01470	0.305898721	2.72E-12	postive
MT3	LINC01474	0.421004362	6.72E-23	postive
MT3	LINC01503	0.437319351	9.01E-25	postive
MT3	LINC01672	0.520015737	5.50E-36	postive
MT3	LINC01698	0.502520431	2.37E-33	postive
MT3	LINC01771	0.496949321	1.52E-32	postive
MT3	LINC01780	0.475749303	1.32E-29	postive
MT3	LINC01885	0.343877183	2.52E-15	postive
MT3	LINC01928	0.567907486	4.90E-44	postive
MT3	LINC02012	0.392087184	8.03E-20	postive
MT3	LINC02046	0.388530534	1.83E-19	postive
MT3	LINC02103	0.381654536	8.76E-19	postive
MT3	LINC02554	0.504322754	1.29E-33	postive
MT3	LINC02598	0.404792951	3.88E-21	postive
MT3	LINC02650	0.360325801	8.95E-17	postive
MT3	LINC02728	0.341234776	4.23E-15	postive
MT3	LINC02760	0.387512239	2.31E-19	postive
MT3	LINC02771	0.380534099	1.13E-18	postive
MT3	LINC02820	0.308930333	1.62E-12	postive
MT3	LINC02840	0.307302651	2.14E-12	postive
MT3	LPP-AS2	0.311375671	1.06E-12	postive
MT3	MIR205HG	0.327862815	5.40E-14	postive
MT3	MRGPRG-AS1	0.330431179	3.34E-14	postive
MT3	MYB-AS1	0.310229433	1.29E-12	postive
MT3	MYCL-AS1	0.316654715	4.16E-13	postive
MT3	NCAM1-AS1	0.33671192	1.02E-14	postive
MT3	NMRAL2P	0.320046736	2.26E-13	postive
MT3	NPHP3-AS1	0.30168195	5.58E-12	postive
MT3	OR2S1P	0.313241899	7.61E-13	postive
MT3	OR5G5P	0.373198246	5.71E-18	postive

MT3	PART1	0.370133565	1.11E-17	postive
MT3	PCAT1	0.403577231	5.22E-21	postive
MT3	PCAT18	0.314020028	6.64E-13	postive
MT3	PPP1R35-AS1	0.319325681	2.58E-13	postive
MT3	PTENP1-AS	0.518718372	8.73E-36	postive
MT3	RAMP2-AS1	0.36018023	9.22E-17	postive
MT3	RP11-141C7.4	0.303879526	3.84E-12	postive
MT3	RP11-298O21.6	0.421810695	5.46E-23	postive
MT3	RP11-298O21.7	0.536248282	1.43E-38	postive
MT3	RP11-541P9.3	0.360957697	7.84E-17	postive
MT3	RP11-659O3.1	0.343293523	2.83E-15	postive
MT3	RP11-680F20.6	0.520753101	4.23E-36	postive
MT3	RP4-784A16.3	0.40216905	7.34E-21	postive
MT3	RXYLT1-AS1	0.350179117	7.18E-16	postive
MT3	SAA3P	0.33253	2.25E-14	postive
MT3	SSBP3-AS1	0.327848297	5.42E-14	postive
MT3	STARD4-AS1	0.415185182	2.96E-22	postive
MT3	TCL6	0.395559354	3.55E-20	postive
MT3	TMCO1-AS1	0.391921184	8.34E-20	postive
MT3	TMEM44-AS1	0.329281503	4.15E-14	postive
MT3	TSBP1-AS1	0.327076162	6.25E-14	postive
MT3	Z97056.1	0.430172004	6.13E-24	postive
MT3	Z97353.2	0.327271686	6.03E-14	postive
MT4	AC003986.2	0.35367235	3.53E-16	postive
MT4	AC003986.3	0.409206446	1.32E-21	postive
MT4	AC005776.2	0.434799315	1.78E-24	postive
MT4	AC007493.1	0.384603601	4.50E-19	postive
MT4	AC007493.2	0.446172187	7.86E-26	postive
MT4	AC007608.1	0.436935838	1.00E-24	postive
MT4	AC009852.1	0.326110582	7.48E-14	postive
MT4	AC009902.3	0.306671449	2.39E-12	postive
MT4	AC011467.3	0.303038935	4.43E-12	postive
MT4	AC016705.1	0.332202373	2.40E-14	postive
MT4	AC093673.2	0.37393545	4.86E-18	postive
MT4	AC104971.1	0.384361286	4.75E-19	postive
MT4	AC114811.2	0.312607112	8.51E-13	postive
MT4	AC114877.3	0.34497155	2.03E-15	postive
MT4	AC120042.1	0.504185336	1.35E-33	postive
MT4	AC120042.2	0.468410528	1.24E-28	postive
MT4	AL022337.1	0.524028282	1.30E-36	postive
MT4	AL031058.1	0.498809376	8.21E-33	postive
MT4	AL080273.1	0.323330177	1.25E-13	postive
MT4	AL121832.1	0.439718622	4.68E-25	postive
MT4	AL135902.1	0.500395107	4.84E-33	postive
MT4	AL136526.1	0.321414125	1.77E-13	postive
MT4	AP001025.1	0.388575605	1.81E-19	postive
MT4	LINC00622	0.334803039	1.46E-14	postive
MT4	LINC00867	0.583004132	7.41E-47	postive
MT4	LINC00868	0.517146528	1.52E-35	postive
MT4	LINC01549	0.545494148	4.16E-40	postive

MT4	LINC01618	0.38054663	1.12E-18	postive
MT4	LINC01854	0.346984913	1.36E-15	postive
MT4	LINC02119	0.574277739	3.30E-45	postive
MT4	LINC02559	0.37453963	4.26E-18	postive
MT4	MTHFD2P1	0.492392888	6.78E-32	postive
MT4	NAGPA-AS1	0.467516809	1.62E-28	postive
MT4	NEUROG2-AS1	0.448901781	3.65E-26	postive
MT4	RP11-401P9.4	0.308099233	1.87E-12	postive
MT-CO3	AC000068.2	0.33295595	2.08E-14	postive
MT-CO3	AC002044.1	0.309404977	1.49E-12	postive
MT-CO3	AC002059.1	0.442646416	2.09E-25	postive
MT-CO3	AC002398.1	0.493674446	4.46E-32	postive
MT-CO3	AC002470.1	0.307293691	2.14E-12	postive
MT-CO3	AC002550.2	0.339339919	6.11E-15	postive
MT-CO3	AC002553.1	0.300996412	6.26E-12	postive
MT-CO3	AC003070.1	0.30957483	1.45E-12	postive
MT-CO3	AC003102.1	0.481868577	1.97E-30	postive
MT-CO3	AC004000.1	0.475476478	1.44E-29	postive
MT-CO3	AC004067.1	0.316570445	4.22E-13	postive
MT-CO3	AC004069.1	0.439162667	5.45E-25	postive
MT-CO3	AC004148.1	0.355500685	2.43E-16	postive
MT-CO3	AC004233.1	0.312062277	9.37E-13	postive
MT-CO3	AC004253.1	0.384715448	4.38E-19	postive
MT-CO3	AC004263.1	0.34831528	1.04E-15	postive
MT-CO3	AC004447.2	0.504317407	1.29E-33	postive
MT-CO3	AC004466.1	0.328069167	5.20E-14	postive
MT-CO3	AC004801.2	0.366997332	2.18E-17	postive
MT-CO3	AC004801.6	0.363799814	4.31E-17	postive
MT-CO3	AC004812.2	0.351115395	5.94E-16	postive
MT-CO3	AC004839.2	0.343253513	2.85E-15	postive
MT-CO3	AC004846.2	0.450819473	2.12E-26	postive
MT-CO3	AC004890.2	0.326181698	7.38E-14	postive
MT-CO3	AC004895.1	0.307767563	1.98E-12	postive
MT-CO3	AC004918.1	0.430213488	6.06E-24	postive
MT-CO3	AC004923.4	0.312282553	9.01E-13	postive
MT-CO3	AC004943.3	0.355514114	2.42E-16	postive
MT-CO3	AC004951.1	0.405060658	3.64E-21	postive
MT-CO3	AC004951.4	0.485963457	5.39E-31	postive
MT-CO3	AC004980.1	0.359190135	1.13E-16	postive
MT-CO3	AC004982.1	0.30250715	4.85E-12	postive
MT-CO3	AC004982.2	0.310032678	1.33E-12	postive
MT-CO3	AC005005.3	0.310871906	1.15E-12	postive
MT-CO3	AC005014.2	0.39919872	1.50E-20	postive
MT-CO3	AC005041.3	0.345241749	1.92E-15	postive
MT-CO3	AC005229.4	0.329844059	3.73E-14	postive
MT-CO3	AC005261.2	0.334116946	1.67E-14	postive
MT-CO3	AC005261.4	0.467529228	1.61E-28	postive
MT-CO3	AC005306.1	0.408830112	1.44E-21	postive
MT-CO3	AC005324.5	0.459912926	1.54E-27	postive
MT-CO3	AC005332.1	0.462134005	8.03E-28	postive

MT-CO3	AC005387.1	0.459429929	1.78E-27	postive
MT-CO3	AC005387.2	0.367375252	2.01E-17	postive
MT-CO3	AC005393.1	0.365548952	2.97E-17	postive
MT-CO3	AC005695.3	0.314053119	6.60E-13	postive
MT-CO3	AC005696.1	0.327004242	6.34E-14	postive
MT-CO3	AC005753.1	0.335196244	1.36E-14	postive
MT-CO3	AC005776.2	0.338900918	6.66E-15	postive
MT-CO3	AC005785.1	0.501546131	3.29E-33	postive
MT-CO3	AC005840.2	0.317809938	3.38E-13	postive
MT-CO3	AC005840.4	0.306723235	2.37E-12	postive
MT-CO3	AC005911.1	0.428957486	8.46E-24	postive
MT-CO3	AC005920.3	0.306994257	2.26E-12	postive
MT-CO3	AC006001.3	0.350183533	7.17E-16	postive
MT-CO3	AC006042.1	0.480720886	2.82E-30	postive
MT-CO3	AC006111.2	0.474433364	1.99E-29	postive
MT-CO3	AC006254.1	0.377619263	2.16E-18	postive
MT-CO3	AC006435.2	0.345003038	2.02E-15	postive
MT-CO3	AC006449.7	0.335233009	1.35E-14	postive
MT-CO3	AC006557.1	0.340860325	4.55E-15	postive
MT-CO3	AC006942.1	0.360197377	9.19E-17	postive
MT-CO3	AC007038.1	0.30039746	6.92E-12	postive
MT-CO3	AC007066.2	0.312729435	8.33E-13	postive
MT-CO3	AC007114.1	0.316713656	4.12E-13	postive
MT-CO3	AC007292.1	0.350614929	6.57E-16	postive
MT-CO3	AC007879.3	0.309650079	1.43E-12	postive
MT-CO3	AC008073.2	0.302993228	4.47E-12	postive
MT-CO3	AC008105.1	0.318474516	3.00E-13	postive
MT-CO3	AC008608.2	0.300492938	6.81E-12	postive
MT-CO3	AC008610.1	0.366441781	2.46E-17	postive
MT-CO3	AC008735.2	0.316837171	4.03E-13	postive
MT-CO3	AC008735.4	0.344995608	2.02E-15	postive
MT-CO3	AC008764.6	0.374548811	4.25E-18	postive
MT-CO3	AC008764.8	0.340451285	4.93E-15	postive
MT-CO3	AC008870.3	0.343569581	2.68E-15	postive
MT-CO3	AC009019.1	0.303819345	3.88E-12	postive
MT-CO3	AC009022.1	0.375224767	3.66E-18	postive
MT-CO3	AC009061.2	0.318338652	3.08E-13	postive
MT-CO3	AC009065.5	0.480879864	2.69E-30	postive
MT-CO3	AC009065.9	0.457519473	3.10E-27	postive
MT-CO3	AC009097.2	0.408981484	1.39E-21	postive
MT-CO3	AC009107.2	0.346492483	1.50E-15	postive
MT-CO3	AC009118.3	0.317878283	3.34E-13	postive
MT-CO3	AC009120.2	0.32107852	1.88E-13	postive
MT-CO3	AC009148.1	0.352468017	4.52E-16	postive
MT-CO3	AC009171.2	0.340941618	4.48E-15	postive
MT-CO3	AC009404.1	0.329843093	3.73E-14	postive
MT-CO3	AC009690.2	0.344952177	2.04E-15	postive
MT-CO3	AC009779.3	0.313835213	6.86E-13	postive
MT-CO3	AC010271.1	0.300628588	6.66E-12	postive
MT-CO3	AC010319.4	0.334098973	1.67E-14	postive

MT-CO3	AC010326.4	0.342038524	3.61E-15	postive
MT-CO3	AC010326.5	0.414896033	3.18E-22	postive
MT-CO3	AC010331.1	0.319585951	2.46E-13	postive
MT-CO3	AC010359.2	0.356248867	2.08E-16	postive
MT-CO3	AC010422.2	0.478071124	6.46E-30	postive
MT-CO3	AC010463.3	0.37414109	4.65E-18	postive
MT-CO3	AC010491.1	0.35943393	1.08E-16	postive
MT-CO3	AC010503.5	0.37969349	1.36E-18	postive
MT-CO3	AC010525.1	0.368596767	1.55E-17	postive
MT-CO3	AC010531.6	0.315249185	5.34E-13	postive
MT-CO3	AC010542.6	0.44119575	3.12E-25	postive
MT-CO3	AC010618.2	0.463364174	5.59E-28	postive
MT-CO3	AC010618.3	0.367167569	2.11E-17	postive
MT-CO3	AC010719.1	0.369857988	1.18E-17	postive
MT-CO3	AC010761.1	0.358891225	1.21E-16	postive
MT-CO3	AC010809.2	0.342923132	3.04E-15	postive
MT-CO3	AC010864.1	0.392700606	6.96E-20	postive
MT-CO3	AC010883.1	0.483939403	1.02E-30	postive
MT-CO3	AC010973.2	0.354253454	3.14E-16	postive
MT-CO3	AC010997.4	0.39872079	1.68E-20	postive
MT-CO3	AC010997.5	0.388777637	1.73E-19	postive
MT-CO3	AC011446.1	0.335853139	1.20E-14	postive
MT-CO3	AC011461.1	0.316549518	4.24E-13	postive
MT-CO3	AC011462.4	0.388404364	1.88E-19	postive
MT-CO3	AC011472.1	0.340934708	4.48E-15	postive
MT-CO3	AC011472.2	0.455106598	6.22E-27	postive
MT-CO3	AC011481.1	0.339524031	5.90E-15	postive
MT-CO3	AC011498.6	0.359441871	1.08E-16	postive
MT-CO3	AC011511.1	0.341825817	3.77E-15	postive
MT-CO3	AC012173.1	0.37865183	1.72E-18	postive
MT-CO3	AC012186.2	0.376024124	3.07E-18	postive
MT-CO3	AC012306.2	0.30892058	1.62E-12	postive
MT-CO3	AC012313.1	0.300651684	6.63E-12	postive
MT-CO3	AC012313.3	0.31086343	1.16E-12	postive
MT-CO3	AC012313.9	0.312850167	8.16E-13	postive
MT-CO3	AC012360.3	0.352664316	4.34E-16	postive
MT-CO3	AC012368.1	0.305195155	3.07E-12	postive
MT-CO3	AC012510.1	0.55905497	1.90E-42	postive
MT-CO3	AC012615.1	0.344038786	2.44E-15	postive
MT-CO3	AC012615.6	0.379607093	1.39E-18	postive
MT-CO3	AC012645.4	0.325790118	7.93E-14	postive
MT-CO3	AC012676.4	0.30088624	6.37E-12	postive
MT-CO3	AC013472.2	0.303639307	4.00E-12	postive
MT-CO3	AC013731.1	0.306473734	2.47E-12	postive
MT-CO3	AC015726.1	0.380678235	1.09E-18	postive
MT-CO3	AC015802.4	0.341927767	3.69E-15	postive
MT-CO3	AC015802.5	0.393488971	5.78E-20	postive
MT-CO3	AC015912.3	0.309746986	1.40E-12	postive
MT-CO3	AC015961.2	0.362884304	5.23E-17	postive
MT-CO3	AC016027.1	0.308382856	1.78E-12	postive

MT-CO3	AC016065.1	0.340458047	4.92E-15	postive
MT-CO3	AC016737.1	0.374004097	4.79E-18	postive
MT-CO3	AC016773.2	0.419224733	1.06E-22	postive
MT-CO3	AC016876.3	0.361299975	7.30E-17	postive
MT-CO3	AC016876.4	0.4417536	2.68E-25	postive
MT-CO3	AC016957.2	0.318314996	3.09E-13	postive
MT-CO3	AC017083.1	0.406170393	2.78E-21	postive
MT-CO3	AC018638.6	0.406895907	2.32E-21	postive
MT-CO3	AC018647.2	0.305161412	3.09E-12	postive
MT-CO3	AC018648.1	0.415025676	3.08E-22	postive
MT-CO3	AC018766.4	0.449674708	2.93E-26	postive
MT-CO3	AC018809.1	0.372296829	6.96E-18	postive
MT-CO3	AC018904.1	0.325629918	8.17E-14	postive
MT-CO3	AC020594.1	0.356951839	1.80E-16	postive
MT-CO3	AC020663.2	0.348160023	1.08E-15	postive
MT-CO3	AC020907.4	0.352494928	4.49E-16	postive
MT-CO3	AC020978.7	0.305717142	2.81E-12	postive
MT-CO3	AC022007.1	0.425975204	1.85E-23	postive
MT-CO3	AC022098.2	0.306460087	2.47E-12	postive
MT-CO3	AC022150.2	0.301609418	5.64E-12	postive
MT-CO3	AC022167.2	0.436761497	1.05E-24	postive
MT-CO3	AC022296.4	0.399066952	1.55E-20	postive
MT-CO3	AC022400.1	0.319945883	2.30E-13	postive
MT-CO3	AC022762.2	0.335397771	1.31E-14	postive
MT-CO3	AC023302.1	0.358703082	1.25E-16	postive
MT-CO3	AC024060.2	0.394212181	4.88E-20	postive
MT-CO3	AC024075.2	0.451784848	1.61E-26	postive
MT-CO3	AC024361.3	0.327076398	6.25E-14	postive
MT-CO3	AC025162.2	0.342159215	3.53E-15	postive
MT-CO3	AC025171.5	0.331638643	2.67E-14	postive
MT-CO3	AC025175.1	0.309159894	1.55E-12	postive
MT-CO3	AC025178.2	0.385619485	3.57E-19	postive
MT-CO3	AC025181.2	0.326543177	6.90E-14	postive
MT-CO3	AC026333.4	0.341998196	3.64E-15	postive
MT-CO3	AC026367.2	0.358354604	1.35E-16	postive
MT-CO3	AC026471.4	0.473904407	2.34E-29	postive
MT-CO3	AC026740.1	0.450897466	2.07E-26	postive
MT-CO3	AC026803.2	0.36473692	3.53E-17	postive
MT-CO3	AC026979.2	0.402767794	6.35E-21	postive
MT-CO3	AC027271.1	0.340097925	5.28E-15	postive
MT-CO3	AC027307.1	0.319378067	2.55E-13	postive
MT-CO3	AC027307.2	0.303077233	4.40E-12	postive
MT-CO3	AC027575.3	0.351875899	5.09E-16	postive
MT-CO3	AC027601.2	0.386341945	3.02E-19	postive
MT-CO3	AC027644.3	0.466924765	1.94E-28	postive
MT-CO3	AC027682.4	0.308040668	1.89E-12	postive
MT-CO3	AC027796.4	0.326952769	6.40E-14	postive
MT-CO3	AC034102.5	0.318886583	2.79E-13	postive
MT-CO3	AC034236.2	0.30693012	2.28E-12	postive
MT-CO3	AC040162.3	0.413220828	4.85E-22	postive

MT-CO3	AC040169.1	0.360723217	8.23E-17	postive
MT-CO3	AC040977.1	0.368264284	1.66E-17	postive
MT-CO3	AC046143.2	0.313498373	7.28E-13	postive
MT-CO3	AC055764.2	0.322840248	1.36E-13	postive
MT-CO3	AC055855.2	0.344087733	2.42E-15	postive
MT-CO3	AC067750.1	0.308308419	1.80E-12	postive
MT-CO3	AC067838.1	0.353588562	3.59E-16	postive
MT-CO3	AC067930.4	0.339284711	6.18E-15	postive
MT-CO3	AC068039.4	0.35095322	6.14E-16	postive
MT-CO3	AC068620.2	0.371922127	7.55E-18	postive
MT-CO3	AC068724.2	0.360934032	7.88E-17	postive
MT-CO3	AC068831.2	0.348010761	1.11E-15	postive
MT-CO3	AC069307.1	0.395687035	3.45E-20	postive
MT-CO3	AC072061.1	0.4408176	3.47E-25	postive
MT-CO3	AC073195.2	0.361660786	6.76E-17	postive
MT-CO3	AC073352.1	0.354351834	3.08E-16	postive
MT-CO3	AC073575.4	0.40170869	8.20E-21	postive
MT-CO3	AC073611.1	0.338367432	7.38E-15	postive
MT-CO3	AC073655.2	0.413503519	4.51E-22	postive
MT-CO3	AC073842.2	0.366002923	2.70E-17	postive
MT-CO3	AC073869.1	0.366218024	2.58E-17	postive
MT-CO3	AC073896.4	0.495457973	2.49E-32	postive
MT-CO3	AC073957.3	0.351732448	5.24E-16	postive
MT-CO3	AC074117.1	0.370664326	9.92E-18	postive
MT-CO3	AC074212.1	0.366516696	2.42E-17	postive
MT-CO3	AC078909.2	0.341003844	4.42E-15	postive
MT-CO3	AC079148.1	0.301865616	5.41E-12	postive
MT-CO3	AC079174.2	0.413591633	4.41E-22	postive
MT-CO3	AC079322.1	0.356630327	1.93E-16	postive
MT-CO3	AC079336.5	0.317594599	3.52E-13	postive
MT-CO3	AC079414.3	0.399324279	1.45E-20	postive
MT-CO3	AC079906.1	0.331645821	2.66E-14	postive
MT-CO3	AC079907.1	0.314274361	6.35E-13	postive
MT-CO3	AC079922.2	0.346709649	1.44E-15	postive
MT-CO3	AC080129.2	0.443950027	1.46E-25	postive
MT-CO3	AC083880.1	0.387186973	2.49E-19	postive
MT-CO3	AC084036.1	0.33331993	1.94E-14	postive
MT-CO3	AC084125.2	0.370730057	9.78E-18	postive
MT-CO3	AC084125.4	0.419479505	9.93E-23	postive
MT-CO3	AC084824.6	0.346190584	1.59E-15	postive
MT-CO3	AC084876.1	0.302433451	4.91E-12	postive
MT-CO3	AC087289.2	0.47156673	4.77E-29	postive
MT-CO3	AC087289.5	0.32734987	5.94E-14	postive
MT-CO3	AC087392.3	0.32933569	4.11E-14	postive
MT-CO3	AC087500.2	0.304416638	3.51E-12	postive
MT-CO3	AC087741.1	0.356255456	2.08E-16	postive
MT-CO3	AC087741.2	0.381045191	1.00E-18	postive
MT-CO3	AC087742.1	0.348343858	1.04E-15	postive
MT-CO3	AC090114.2	0.382020253	8.07E-19	postive
MT-CO3	AC090425.2	0.428137479	1.05E-23	postive

MT-CO3	AC090515.3	0.32591445	7.75E-14	postive
MT-CO3	AC090589.3	0.344948407	2.04E-15	postive
MT-CO3	AC090970.2	0.36897013	1.43E-17	postive
MT-CO3	AC091390.5	0.303228354	4.29E-12	postive
MT-CO3	AC091488.1	0.31167462	1.00E-12	postive
MT-CO3	AC091729.3	0.409595078	1.20E-21	postive
MT-CO3	AC092118.2	0.414906477	3.17E-22	postive
MT-CO3	AC092119.2	0.32910847	4.28E-14	postive
MT-CO3	AC092171.2	0.4250362	2.37E-23	postive
MT-CO3	AC092171.4	0.413778062	4.21E-22	postive
MT-CO3	AC092718.4	0.415211426	2.94E-22	postive
MT-CO3	AC092807.3	0.309687642	1.42E-12	postive
MT-CO3	AC092809.5	0.461366469	1.01E-27	postive
MT-CO3	AC093151.2	0.30040734	6.91E-12	postive
MT-CO3	AC093157.1	0.384350472	4.76E-19	postive
MT-CO3	AC093249.6	0.386103894	3.19E-19	postive
MT-CO3	AC093462.1	0.347915528	1.13E-15	postive
MT-CO3	AC093620.1	0.373144597	5.78E-18	postive
MT-CO3	AC093734.1	0.311238838	1.08E-12	postive
MT-CO3	AC096540.1	0.367198012	2.09E-17	postive
MT-CO3	AC096642.1	0.317660668	3.47E-13	postive
MT-CO3	AC096677.1	0.411608752	7.25E-22	postive
MT-CO3	AC097358.2	0.311581972	1.02E-12	postive
MT-CO3	AC097468.3	0.394086692	5.03E-20	postive
MT-CO3	AC097641.2	0.328257125	5.02E-14	postive
MT-CO3	AC098591.2	0.342997127	3.00E-15	postive
MT-CO3	AC099343.4	0.319589662	2.46E-13	postive
MT-CO3	AC099518.6	0.360005748	9.56E-17	postive
MT-CO3	AC099568.2	0.330119476	3.55E-14	postive
MT-CO3	AC099778.1	0.443384519	1.71E-25	postive
MT-CO3	AC099791.2	0.316012683	4.66E-13	postive
MT-CO3	AC100810.1	0.31242687	8.79E-13	postive
MT-CO3	AC100814.2	0.32391172	1.12E-13	postive
MT-CO3	AC102953.2	0.376983969	2.48E-18	postive
MT-CO3	AC103691.1	0.457353645	3.25E-27	postive
MT-CO3	AC104463.2	0.387615145	2.26E-19	postive
MT-CO3	AC104564.3	0.30052272	6.77E-12	postive
MT-CO3	AC104695.3	0.399815587	1.29E-20	postive
MT-CO3	AC105020.1	0.354285997	3.12E-16	postive
MT-CO3	AC105219.1	0.327528859	5.75E-14	postive
MT-CO3	AC105345.1	0.313906499	6.77E-13	postive
MT-CO3	AC105935.2	0.339463403	5.97E-15	postive
MT-CO3	AC106782.5	0.406832815	2.36E-21	postive
MT-CO3	AC106820.3	0.411571094	7.32E-22	postive
MT-CO3	AC106886.2	0.307246731	2.16E-12	postive
MT-CO3	AC106886.3	0.431888112	3.89E-24	postive
MT-CO3	AC107081.2	0.377111007	2.42E-18	postive
MT-CO3	AC107294.1	0.318989343	2.74E-13	postive
MT-CO3	AC107375.1	0.384935021	4.17E-19	postive
MT-CO3	AC108134.2	0.315521536	5.09E-13	postive

MT-CO3	AC108673.2	0.394302614	4.78E-20	postive
MT-CO3	AC108673.3	0.492706985	6.12E-32	postive
MT-CO3	AC108860.2	0.305273494	3.03E-12	postive
MT-CO3	AC109322.1	0.383666434	5.56E-19	postive
MT-CO3	AC109449.1	0.351737105	5.24E-16	postive
MT-CO3	AC109460.1	0.358372187	1.34E-16	postive
MT-CO3	AC110079.1	0.322365457	1.49E-13	postive
MT-CO3	AC110285.1	0.303616461	4.02E-12	postive
MT-CO3	AC110285.2	0.314362304	6.25E-13	postive
MT-CO3	AC114271.1	0.306038314	2.66E-12	postive
MT-CO3	AC114490.1	0.349639994	8.00E-16	postive
MT-CO3	AC114730.3	0.300051848	7.33E-12	postive
MT-CO3	AC116667.1	0.304777357	3.30E-12	postive
MT-CO3	AC116913.1	0.371064101	9.09E-18	postive
MT-CO3	AC116914.2	0.326126183	7.45E-14	postive
MT-CO3	AC118553.1	0.343831645	2.54E-15	postive
MT-CO3	AC118755.2	0.332057875	2.46E-14	postive
MT-CO3	AC120053.1	0.430860121	5.11E-24	postive
MT-CO3	AC120349.1	0.303136115	4.36E-12	postive
MT-CO3	AC120498.9	0.35879103	1.23E-16	postive
MT-CO3	AC124016.2	0.335110026	1.38E-14	postive
MT-CO3	AC124944.2	0.311215608	1.09E-12	postive
MT-CO3	AC125494.2	0.308109243	1.86E-12	postive
MT-CO3	AC126118.1	0.336217386	1.12E-14	postive
MT-CO3	AC127024.6	0.365464487	3.03E-17	postive
MT-CO3	AC128687.2	0.351809255	5.16E-16	postive
MT-CO3	AC130371.2	0.318799582	2.83E-13	postive
MT-CO3	AC130462.3	0.435610682	1.43E-24	postive
MT-CO3	AC131009.3	0.325122662	8.97E-14	postive
MT-CO3	AC131159.2	0.360040953	9.49E-17	postive
MT-CO3	AC132872.1	0.46183138	8.78E-28	postive
MT-CO3	AC132872.2	0.485982054	5.35E-31	postive
MT-CO3	AC132872.3	0.39530839	3.77E-20	postive
MT-CO3	AC132938.2	0.329545128	3.95E-14	postive
MT-CO3	AC132938.3	0.371015889	9.19E-18	postive
MT-CO3	AC133528.2	0.537591306	8.62E-39	postive
MT-CO3	AC133552.2	0.312051813	9.38E-13	postive
MT-CO3	AC133552.5	0.328382115	4.91E-14	postive
MT-CO3	AC135048.1	0.30399	3.77E-12	postive
MT-CO3	AC135050.1	0.379594659	1.39E-18	postive
MT-CO3	AC135050.3	0.412722097	5.49E-22	postive
MT-CO3	AC135050.4	0.323212384	1.27E-13	postive
MT-CO3	AC135050.6	0.422747801	4.29E-23	postive
MT-CO3	AC135178.6	0.381408727	9.26E-19	postive
MT-CO3	AC135507.1	0.308563882	1.72E-12	postive
MT-CO3	AC136469.1	0.301007249	6.25E-12	postive
MT-CO3	AC136475.2	0.30971457	1.41E-12	postive
MT-CO3	AC137630.2	0.338196405	7.63E-15	postive
MT-CO3	AC137630.3	0.342537948	3.28E-15	postive
MT-CO3	AC137932.3	0.376614121	2.70E-18	postive

MT-CO3	AC138230.1	0.357537907	1.60E-16	postive
MT-CO3	AC138696.2	0.443564993	1.62E-25	postive
MT-CO3	AC139100.2	0.335611057	1.25E-14	postive
MT-CO3	AC139256.3	0.302355029	4.98E-12	postive
MT-CO3	AC139530.1	0.442090784	2.44E-25	postive
MT-CO3	AC142472.1	0.396803461	2.65E-20	postive
MT-CO3	AC144831.1	0.318781202	2.84E-13	postive
MT-CO3	AC145207.5	0.329122736	4.27E-14	postive
MT-CO3	AC145285.2	0.359004825	1.18E-16	postive
MT-CO3	AC145285.6	0.352187571	4.78E-16	postive
MT-CO3	AC145423.2	0.381362221	9.36E-19	postive
MT-CO3	AC145423.3	0.326573957	6.86E-14	postive
MT-CO3	AC211433.2	0.370872557	9.48E-18	postive
MT-CO3	AC231533.2	0.310780684	1.17E-12	postive
MT-CO3	AC232271.1	0.361317596	7.27E-17	postive
MT-CO3	AC233728.1	0.410242916	1.02E-21	postive
MT-CO3	AC239803.1	0.349908848	7.58E-16	postive
MT-CO3	AC244090.1	0.342963548	3.02E-15	postive
MT-CO3	AC244090.3	0.303536812	4.07E-12	postive
MT-CO3	AC244197.2	0.306273185	2.55E-12	postive
MT-CO3	AC245052.4	0.36102231	7.73E-17	postive
MT-CO3	AC245060.6	0.331008919	3.00E-14	postive
MT-CO3	AC245140.1	0.307000394	2.26E-12	postive
MT-CO3	AC245140.2	0.3809441	1.03E-18	postive
MT-CO3	AC245884.8	0.308763505	1.66E-12	postive
MT-CO3	AC253536.6	0.406957509	2.29E-21	postive
MT-CO3	ACTG1P17	0.455950638	4.88E-27	postive
MT-CO3	AD000671.3	0.36029318	9.01E-17	postive
MT-CO3	AD001527.1	0.327316788	5.98E-14	postive
MT-CO3	AF038458.2	0.305126211	3.11E-12	postive
MT-CO3	AF111169.3	0.303001841	4.46E-12	postive
MT-CO3	AFG3L1P	0.40172734	8.16E-21	postive
MT-CO3	AGBL5-IT1	0.446557436	7.05E-26	postive
MT-CO3	AHSA2P	0.359345377	1.10E-16	postive
MT-CO3	AL008582.1	0.354149855	3.21E-16	postive
MT-CO3	AL008635.1	0.374706833	4.11E-18	postive
MT-CO3	AL008727.1	0.30258136	4.79E-12	postive
MT-CO3	AL008729.2	0.303835714	3.87E-12	postive
MT-CO3	AL020996.1	0.333082706	2.03E-14	postive
MT-CO3	AL021392.1	0.313199507	7.67E-13	postive
MT-CO3	AL021707.1	0.411083027	8.26E-22	postive
MT-CO3	AL021707.6	0.478459155	5.72E-30	postive
MT-CO3	AL021707.8	0.311363621	1.06E-12	postive
MT-CO3	AL021878.2	0.307681237	2.01E-12	postive
MT-CO3	AL022328.1	0.351393251	5.62E-16	postive
MT-CO3	AL022328.2	0.438766316	6.08E-25	postive
MT-CO3	AL022328.3	0.342425882	3.35E-15	postive
MT-CO3	AL022476.1	0.325862911	7.83E-14	postive
MT-CO3	AL023284.4	0.414531609	3.49E-22	postive
MT-CO3	AL031186.1	0.358113691	1.42E-16	postive

MT-CO3	AL031432.3	0.346767435	1.42E-15	postive
MT-CO3	AL031673.1	0.350197252	7.15E-16	postive
MT-CO3	AL031705.1	0.348892512	9.29E-16	postive
MT-CO3	AL031709.1	0.355565436	2.40E-16	postive
MT-CO3	AL031714.1	0.370024693	1.14E-17	postive
MT-CO3	AL031722.1	0.316653545	4.16E-13	postive
MT-CO3	AL031733.2	0.353997724	3.31E-16	postive
MT-CO3	AL031775.1	0.381394792	9.29E-19	postive
MT-CO3	AL031775.2	0.309353038	1.50E-12	postive
MT-CO3	AL031847.1	0.307596512	2.04E-12	postive
MT-CO3	AL033384.2	0.421936275	5.29E-23	postive
MT-CO3	AL035071.1	0.54202101	1.59E-39	postive
MT-CO3	AL035461.3	0.357812557	1.51E-16	postive
MT-CO3	AL049646.2	0.321875953	1.62E-13	postive
MT-CO3	AL049795.1	0.347765557	1.16E-15	postive
MT-CO3	AL049840.6	0.407257983	2.13E-21	postive
MT-CO3	AL078604.2	0.301991611	5.29E-12	postive
MT-CO3	AL080312.2	0.304559841	3.42E-12	postive
MT-CO3	AL096701.3	0.3948092	4.24E-20	postive
MT-CO3	AL096828.3	0.376646126	2.68E-18	postive
MT-CO3	AL109659.2	0.319030009	2.72E-13	postive
MT-CO3	AL109811.1	0.380849121	1.05E-18	postive
MT-CO3	AL109811.2	0.369646338	1.24E-17	postive
MT-CO3	AL110115.2	0.328215761	5.06E-14	postive
MT-CO3	AL117209.1	0.391403368	9.41E-20	postive
MT-CO3	AL117336.2	0.435369944	1.53E-24	postive
MT-CO3	AL117379.1	0.444817977	1.15E-25	postive
MT-CO3	AL118558.3	0.420972637	6.77E-23	postive
MT-CO3	AL118558.4	0.4913727	9.45E-32	postive
MT-CO3	AL121583.1	0.349724096	7.86E-16	postive
MT-CO3	AL121601.1	0.339885789	5.50E-15	postive
MT-CO3	AL121829.2	0.30479274	3.29E-12	postive
MT-CO3	AL121832.2	0.381342781	9.40E-19	postive
MT-CO3	AL121832.3	0.31476178	5.82E-13	postive
MT-CO3	AL121852.1	0.334047547	1.69E-14	postive
MT-CO3	AL121906.2	0.346128398	1.61E-15	postive
MT-CO3	AL121917.2	0.31742257	3.63E-13	postive
MT-CO3	AL121987.2	0.313802924	6.90E-13	postive
MT-CO3	AL122010.1	0.436956157	9.94E-25	postive
MT-CO3	AL122023.3	0.32056497	2.06E-13	postive
MT-CO3	AL132639.2	0.30107014	6.18E-12	postive
MT-CO3	AL132712.1	0.331298728	2.84E-14	postive
MT-CO3	AL133216.2	0.446977362	6.27E-26	postive
MT-CO3	AL133367.1	0.334001605	1.70E-14	postive
MT-CO3	AL133410.1	0.303999898	3.77E-12	postive
MT-CO3	AL133520.1	0.323466628	1.22E-13	postive
MT-CO3	AL133551.1	0.345454238	1.84E-15	postive
MT-CO3	AL135999.1	0.36394922	4.18E-17	postive
MT-CO3	AL136295.2	0.388619441	1.79E-19	postive
MT-CO3	AL136295.6	0.4469834	6.26E-26	postive

MT-CO3	AL136295.7	0.542089668	1.55E-39	postive
MT-CO3	AL136304.1	0.327250762	6.05E-14	postive
MT-CO3	AL137127.1	0.470907368	5.83E-29	postive
MT-CO3	AL137244.1	0.341081557	4.36E-15	postive
MT-CO3	AL137802.2	0.332691188	2.18E-14	postive
MT-CO3	AL139089.1	0.318513247	2.98E-13	postive
MT-CO3	AL139123.1	0.380766241	1.07E-18	postive
MT-CO3	AL139286.2	0.326473554	6.99E-14	postive
MT-CO3	AL139287.1	0.39868795	1.69E-20	postive
MT-CO3	AL139384.1	0.334002092	1.70E-14	postive
MT-CO3	AL157392.3	0.343531747	2.70E-15	postive
MT-CO3	AL157392.4	0.355122006	2.63E-16	postive
MT-CO3	AL157756.1	0.379904862	1.30E-18	postive
MT-CO3	AL157904.1	0.40878763	1.46E-21	postive
MT-CO3	AL158151.4	0.388854008	1.70E-19	postive
MT-CO3	AL158196.1	0.338856161	6.72E-15	postive
MT-CO3	AL158212.2	0.383660056	5.57E-19	postive
MT-CO3	AL158834.2	0.335511787	1.28E-14	postive
MT-CO3	AL160006.1	0.412250555	6.18E-22	postive
MT-CO3	AL161452.1	0.437886156	7.72E-25	postive
MT-CO3	AL161668.3	0.400128069	1.20E-20	postive
MT-CO3	AL161729.3	0.450747049	2.17E-26	postive
MT-CO3	AL162274.2	0.445649319	9.09E-26	postive
MT-CO3	AL162431.1	0.346621376	1.46E-15	postive
MT-CO3	AL162586.1	0.311364062	1.06E-12	postive
MT-CO3	AL162741.1	0.408154223	1.71E-21	postive
MT-CO3	AL353622.1	0.335546453	1.27E-14	postive
MT-CO3	AL353708.3	0.342086135	3.58E-15	postive
MT-CO3	AL353796.1	0.320519518	2.08E-13	postive
MT-CO3	AL353807.2	0.372222238	7.07E-18	postive
MT-CO3	AL354760.1	0.393484186	5.79E-20	postive
MT-CO3	AL354836.1	0.374360275	4.43E-18	postive
MT-CO3	AL354892.3	0.415563637	2.69E-22	postive
MT-CO3	AL355297.3	0.315628622	4.99E-13	postive
MT-CO3	AL355353.2	0.370262198	1.08E-17	postive
MT-CO3	AL355385.1	0.306924212	2.28E-12	postive
MT-CO3	AL355472.3	0.318932062	2.77E-13	postive
MT-CO3	AL355488.1	0.334522406	1.54E-14	postive
MT-CO3	AL355574.1	0.300034058	7.35E-12	postive
MT-CO3	AL355802.3	0.373992194	4.80E-18	postive
MT-CO3	AL356299.3	0.340551543	4.83E-15	postive
MT-CO3	AL356481.3	0.356158079	2.12E-16	postive
MT-CO3	AL356488.3	0.383720455	5.49E-19	postive
MT-CO3	AL356512.1	0.302782441	4.63E-12	postive
MT-CO3	AL357079.1	0.356495445	1.98E-16	postive
MT-CO3	AL358072.1	0.307257172	2.16E-12	postive
MT-CO3	AL358075.2	0.325285336	8.70E-14	postive
MT-CO3	AL358472.2	0.318194599	3.16E-13	postive
MT-CO3	AL358472.3	0.424939874	2.43E-23	postive
MT-CO3	AL358781.2	0.440251497	4.05E-25	postive

MT-CO3	AL358852.1	0.354713655	2.86E-16	postive
MT-CO3	AL359091.3	0.331166322	2.91E-14	postive
MT-CO3	AL359532.1	0.390102283	1.27E-19	postive
MT-CO3	AL359715.1	0.333227976	1.97E-14	postive
MT-CO3	AL359921.1	0.304214934	3.63E-12	postive
MT-CO3	AL359921.2	0.401409551	8.81E-21	postive
MT-CO3	AL365330.1	0.393518481	5.74E-20	postive
MT-CO3	AL390066.2	0.319424611	2.53E-13	postive
MT-CO3	AL390719.1	0.318328353	3.08E-13	postive
MT-CO3	AL390719.2	0.417337771	1.71E-22	postive
MT-CO3	AL390726.4	0.313839859	6.85E-13	postive
MT-CO3	AL390728.6	0.3222423	1.52E-13	postive
MT-CO3	AL390879.1	0.372544826	6.59E-18	postive
MT-CO3	AL391244.2	0.345803835	1.72E-15	postive
MT-CO3	AL391684.1	0.32730536	5.99E-14	postive
MT-CO3	AL445222.2	0.381132531	9.85E-19	postive
MT-CO3	AL445228.3	0.328785959	4.55E-14	postive
MT-CO3	AL445645.1	0.358134448	1.41E-16	postive
MT-CO3	AL450384.2	0.384863677	4.24E-19	postive
MT-CO3	AL450998.2	0.301098368	6.15E-12	postive
MT-CO3	AL451050.2	0.439460958	5.03E-25	postive
MT-CO3	AL451085.3	0.335972882	1.17E-14	postive
MT-CO3	AL512408.1	0.357048868	1.77E-16	postive
MT-CO3	AL512652.1	0.329247521	4.17E-14	postive
MT-CO3	AL512770.1	0.478143917	6.31E-30	postive
MT-CO3	AL513190.1	0.31216217	9.20E-13	postive
MT-CO3	AL513218.1	0.45543133	5.67E-27	postive
MT-CO3	AL513320.1	0.480169374	3.36E-30	postive
MT-CO3	AL513477.2	0.388733611	1.75E-19	postive
MT-CO3	AL583810.1	0.421110052	6.54E-23	postive
MT-CO3	AL589765.6	0.326303176	7.21E-14	postive
MT-CO3	AL589843.2	0.305723197	2.81E-12	postive
MT-CO3	AL590093.1	0.323872623	1.13E-13	postive
MT-CO3	AL590096.1	0.412938171	5.20E-22	postive
MT-CO3	AL590560.3	0.322939138	1.34E-13	postive
MT-CO3	AL590617.2	0.472140916	4.00E-29	postive
MT-CO3	AL590708.1	0.321283236	1.81E-13	postive
MT-CO3	AL591848.3	0.431219582	4.64E-24	postive
MT-CO3	AL591895.1	0.40574537	3.08E-21	postive
MT-CO3	AL592071.1	0.420914741	6.88E-23	postive
MT-CO3	AL592211.1	0.460607233	1.26E-27	postive
MT-CO3	AL596094.1	0.395078472	3.98E-20	postive
MT-CO3	AL603839.3	0.337417284	8.86E-15	postive
MT-CO3	AL604028.1	0.394577817	4.48E-20	postive
MT-CO3	AL606760.2	0.456119876	4.65E-27	postive
MT-CO3	AL627309.2	0.311935141	9.58E-13	postive
MT-CO3	AL671710.1	0.437831799	7.84E-25	postive
MT-CO3	AL672291.1	0.339141382	6.35E-15	postive
MT-CO3	AL691482.4	0.413429875	4.60E-22	postive
MT-CO3	AL731533.2	0.360504713	8.62E-17	postive

MT-CO3	AL731569.1	0.358458591	1.32E-16	postive
MT-CO3	AL732372.2	0.357186367	1.72E-16	postive
MT-CO3	AL844908.2	0.359143494	1.14E-16	postive
MT-CO3	AL928654.2	0.349097348	8.92E-16	postive
MT-CO3	ALOX12-AS1	0.355644074	2.36E-16	postive
MT-CO3	ANKRD19P	0.415760334	2.56E-22	postive
MT-CO3	ANO7L1	0.320173527	2.21E-13	postive
MT-CO3	AP000240.1	0.442164906	2.39E-25	postive
MT-CO3	AP000254.2	0.39015326	1.26E-19	postive
MT-CO3	AP000255.1	0.351003473	6.08E-16	postive
MT-CO3	AP000345.2	0.344757577	2.12E-15	postive
MT-CO3	AP000346.1	0.327870319	5.40E-14	postive
MT-CO3	AP000346.2	0.46109914	1.09E-27	postive
MT-CO3	AP000442.1	0.339801478	5.59E-15	postive
MT-CO3	AP000553.2	0.339347404	6.11E-15	postive
MT-CO3	AP000692.2	0.300441139	6.87E-12	postive
MT-CO3	AP000781.1	0.362434358	5.75E-17	postive
MT-CO3	AP000873.4	0.327196171	6.12E-14	postive
MT-CO3	AP001062.1	0.431575812	4.22E-24	postive
MT-CO3	AP001160.1	0.486832243	4.08E-31	postive
MT-CO3	AP001160.2	0.359144659	1.14E-16	postive
MT-CO3	AP001160.3	0.364484566	3.73E-17	postive
MT-CO3	AP001160.4	0.394967443	4.09E-20	postive
MT-CO3	AP001318.2	0.3566627	1.91E-16	postive
MT-CO3	AP001453.2	0.340692096	4.70E-15	postive
MT-CO3	AP001453.4	0.405814107	3.03E-21	postive
MT-CO3	AP001505.1	0.389217254	1.56E-19	postive
MT-CO3	AP001625.2	0.316734353	4.10E-13	postive
MT-CO3	AP001830.1	0.350970036	6.12E-16	postive
MT-CO3	AP001972.1	0.348357446	1.03E-15	postive
MT-CO3	AP001992.1	0.314342886	6.27E-13	postive
MT-CO3	AP002360.2	0.35377607	3.46E-16	postive
MT-CO3	AP002387.2	0.344428423	2.26E-15	postive
MT-CO3	AP002748.4	0.310578419	1.21E-12	postive
MT-CO3	AP002761.1	0.31477822	5.80E-13	postive
MT-CO3	AP002807.1	0.373415675	5.45E-18	postive
MT-CO3	AP002812.5	0.322215466	1.53E-13	postive
MT-CO3	AP002992.1	0.404747763	3.93E-21	postive
MT-CO3	AP003068.1	0.370123927	1.11E-17	postive
MT-CO3	AP003096.1	0.328434834	4.86E-14	postive
MT-CO3	AP003419.3	0.419335504	1.03E-22	postive
MT-CO3	AP005329.1	0.371821766	7.71E-18	postive
MT-CO3	AP006621.2	0.313795733	6.91E-13	postive
MT-CO3	AP006621.3	0.379608982	1.39E-18	postive
MT-CO3	ARRDC1-AS1	0.451316021	1.84E-26	postive
MT-CO3	ASB16-AS1	0.355486192	2.44E-16	postive
MT-CO3	ASH1L-AS1	0.320181391	2.21E-13	postive
MT-CO3	ASMTL-AS1	0.390447256	1.18E-19	postive
MT-CO3	ATP2C2-AS1	0.354599886	2.92E-16	postive
MT-CO3	ATXN2-AS	0.324914881	9.32E-14	postive

MT-CO3	BACE1-AS	0.464538659	3.94E-28	postive
MT-CO3	BRWD1-AS2	0.347623188	1.20E-15	postive
MT-CO3	BX255925.1	0.359337016	1.10E-16	postive
MT-CO3	BX537318.1	0.351873984	5.09E-16	postive
MT-CO3	BX649601.1	0.423586806	3.45E-23	postive
MT-CO3	BX649632.1	0.398257039	1.87E-20	postive
MT-CO3	C3orf35	0.342218493	3.49E-15	postive
MT-CO3	C9orf163	0.383624008	5.62E-19	postive
MT-CO3	CAHM	0.411364072	7.70E-22	postive
MT-CO3	CAPN10-DT	0.4183854	1.31E-22	postive
MT-CO3	CBR3-AS1	0.383805037	5.39E-19	postive
MT-CO3	CIDECP1	0.330202349	3.49E-14	postive
MT-CO3	CIRBP-AS1	0.336451455	1.07E-14	postive
MT-CO3	CNNM3-DT	0.334774195	1.47E-14	postive
MT-CO3	COA6-AS1	0.419556201	9.74E-23	postive
MT-CO3	COL6A4P2	0.355403885	2.48E-16	postive
MT-CO3	CRYBB2P1	0.392981683	6.51E-20	postive
MT-CO3	CTA-14H9.5	0.310744874	1.18E-12	postive
MT-CO3	CTA-989H11.1	0.335541924	1.27E-14	postive
MT-CO3	CTBP1-AS	0.343883242	2.52E-15	postive
MT-CO3	CTD-2132N18.4	0.376611808	2.70E-18	postive
MT-CO3	CUTALP	0.305987491	2.68E-12	postive
MT-CO3	DCST1-AS1	0.402660855	6.52E-21	postive
MT-CO3	DDX11-AS1	0.30831715	1.80E-12	postive
MT-CO3	DDX39B-AS1	0.369829086	1.19E-17	postive
MT-CO3	DGUOK-AS1	0.375026651	3.83E-18	postive
MT-CO3	DICER1-AS1	0.406307984	2.68E-21	postive
MT-CO3	DLG5-AS1	0.386511522	2.91E-19	postive
MT-CO3	DM1-AS	0.34277436	3.13E-15	postive
MT-CO3	DNAJB5-DT	0.338978791	6.56E-15	postive
MT-CO3	DOC2GP	0.31211202	9.28E-13	postive
MT-CO3	DPP9-AS1	0.357148517	1.73E-16	postive
MT-CO3	ELF3-AS1	0.34661055	1.47E-15	postive
MT-CO3	ENO1-AS1	0.374931638	3.91E-18	postive
MT-CO3	EP400P1	0.315523073	5.09E-13	postive
MT-CO3	EXOSC10-AS1	0.331978871	2.50E-14	postive
MT-CO3	EXTL3-AS1	0.348019215	1.11E-15	postive
MT-CO3	FAHD2CP	0.397231072	2.39E-20	postive
MT-CO3	FAM83C-AS1	0.359194543	1.13E-16	postive
MT-CO3	FAM86HP	0.318305242	3.10E-13	postive
MT-CO3	FARP1-AS1	0.355225942	2.57E-16	postive
MT-CO3	FMR1-IT1	0.310541739	1.22E-12	postive
MT-CO3	FOXD2-AS1	0.396901095	2.59E-20	postive
MT-CO3	FOXO6-AS1	0.386142535	3.17E-19	postive
MT-CO3	GARS-DT	0.341075307	4.36E-15	postive
MT-CO3	GBAP1	0.329598062	3.91E-14	postive
MT-CO3	GEMIN7-AS1	0.3343763	1.59E-14	postive
MT-CO3	GHRLOS	0.331135359	2.93E-14	postive
MT-CO3	GK-IT1	0.360543799	8.55E-17	postive
MT-CO3	GOLGA2P5	0.333997485	1.71E-14	postive

MT-CO3	GTF2IP13	0.558745928	2.15E-42	postive
MT-CO3	GTF2IP20	0.466665909	2.09E-28	postive
MT-CO3	GUSBP11	0.437704239	8.12E-25	postive
MT-CO3	H1FX-AS1	0.30917131	1.55E-12	postive
MT-CO3	HDHD5-AS1	0.337034197	9.54E-15	postive
MT-CO3	HIPK1-AS1	0.347530174	1.22E-15	postive
MT-CO3	HM13-IT1	0.316887172	3.99E-13	postive
MT-CO3	HMGA1P4	0.341004502	4.42E-15	postive
MT-CO3	HPN-AS1	0.31943726	2.53E-13	postive
MT-CO3	HYI-AS1	0.426984001	1.42E-23	postive
MT-CO3	IBA57-DT	0.378448495	1.80E-18	postive
MT-CO3	IGBP1-AS1	0.416623024	2.06E-22	postive
MT-CO3	IL10RB-DT	0.301633084	5.62E-12	postive
MT-CO3	ILF3-DT	0.344315818	2.31E-15	postive
MT-CO3	INE1	0.34244673	3.34E-15	postive
MT-CO3	INTS6-AS1	0.34515502	1.96E-15	postive
MT-CO3	ITGB2-AS1	0.309266118	1.52E-12	postive
MT-CO3	KB-1572G7.2	0.432792186	3.05E-24	postive
MT-CO3	KDM4A-AS1	0.389353413	1.51E-19	postive
MT-CO3	KDM7A-DT	0.328986066	4.38E-14	postive
MT-CO3	KIF1C-AS1	0.314189693	6.44E-13	postive
MT-CO3	KMT2E-AS1	0.387183951	2.49E-19	postive
MT-CO3	KRT8P12	0.370199325	1.10E-17	postive
MT-CO3	LBX2-AS1	0.300639246	6.64E-12	postive
MT-CO3	LENG8-AS1	0.333521508	1.87E-14	postive
MT-CO3	LINC00115	0.423331157	3.68E-23	postive
MT-CO3	LINC00173	0.311997848	9.47E-13	postive
MT-CO3	LINC00174	0.450411983	2.38E-26	postive
MT-CO3	LINC00265	0.427822139	1.14E-23	postive
MT-CO3	LINC00482	0.303562856	4.06E-12	postive
MT-CO3	LINC00685	0.477212768	8.43E-30	postive
MT-CO3	LINC00853	0.36608437	2.65E-17	postive
MT-CO3	LINC00957	0.36138425	7.17E-17	postive
MT-CO3	LINC00963	0.389259902	1.55E-19	postive
MT-CO3	LINC00997	0.351470472	5.53E-16	postive
MT-CO3	LINC01001	0.39258954	7.14E-20	postive
MT-CO3	LINC01004	0.326232124	7.31E-14	postive
MT-CO3	LINC01011	0.365902314	2.76E-17	postive
MT-CO3	LINC01023	0.343514601	2.71E-15	postive
MT-CO3	LINC01089	0.398289653	1.86E-20	postive
MT-CO3	LINC01138	0.323359485	1.24E-13	postive
MT-CO3	LINC01144	0.366451283	2.45E-17	postive
MT-CO3	LINC01176	0.388171695	1.99E-19	postive
MT-CO3	LINC01311	0.386975695	2.62E-19	postive
MT-CO3	LINC01355	0.328552505	4.75E-14	postive
MT-CO3	LINC01372	0.422142008	5.01E-23	postive
MT-CO3	LINC01424	0.376229723	2.94E-18	postive
MT-CO3	LINC01431	0.329343382	4.10E-14	postive
MT-CO3	LINC01569	0.411190568	8.04E-22	postive
MT-CO3	LINC01770	0.37176591	7.81E-18	postive

MT-CO3	LINC01978	0.301234686	6.01E-12	postive
MT-CO3	LINC02012	0.314489578	6.11E-13	postive
MT-CO3	LINC02021	0.328935445	4.42E-14	postive
MT-CO3	LINC02062	0.303286786	4.25E-12	postive
MT-CO3	LINC02482	0.419855744	9.02E-23	postive
MT-CO3	LINC02585	0.365900801	2.76E-17	postive
MT-CO3	LINC02604	0.523209298	1.75E-36	postive
MT-CO3	LINC02615	0.301649884	5.61E-12	postive
MT-CO3	LINC02631	0.369228096	1.35E-17	postive
MT-CO3	LINC02803	0.415275468	2.89E-22	postive
MT-CO3	LIX1L-AS1	0.327242518	6.06E-14	postive
MT-CO3	MAN1B1-DT	0.352943768	4.10E-16	postive
MT-CO3	MATN1-AS1	0.321933959	1.61E-13	postive
MT-CO3	MED8-AS1	0.371258207	8.72E-18	postive
MT-CO3	MELTF-AS1	0.329865881	3.72E-14	postive
MT-CO3	MHENCR	0.464977576	3.46E-28	postive
MT-CO3	MIR200CHG	0.412575074	5.69E-22	postive
MT-CO3	MIR210HG	0.329228795	4.19E-14	postive
MT-CO3	MIR600HG	0.333935197	1.73E-14	postive
MT-CO3	MIR762HG	0.379187457	1.52E-18	postive
MT-CO3	MIS18A-AS1	0.346381903	1.53E-15	postive
MT-CO3	MMP25-AS1	0.315847494	4.80E-13	postive
MT-CO3	MRPL20-AS1	0.358453671	1.32E-16	postive
MT-CO3	MSH5-SAPCD1	0.417591521	1.61E-22	postive
MT-CO3	MTMR9LP	0.335848564	1.20E-14	postive
MT-CO3	MUC20-OT1	0.304152192	3.67E-12	postive
MT-CO3	MZF1-AS1	0.301601997	5.65E-12	postive
MT-CO3	NALT1	0.358203818	1.39E-16	postive
MT-CO3	NBR2	0.349947473	7.52E-16	postive
MT-CO3	NDUFB2-AS1	0.444337437	1.31E-25	postive
MT-CO3	NDUFV2-AS1	0.338317936	7.45E-15	postive
MT-CO3	NPIPB14P	0.333209084	1.98E-14	postive
MT-CO3	NPIPP1	0.426623967	1.56E-23	postive
MT-CO3	NPPA-AS1	0.380832858	1.05E-18	postive
MT-CO3	NSMCE1-DT	0.371202177	8.83E-18	postive
MT-CO3	NSUN5P1	0.389779357	1.37E-19	postive
MT-CO3	NSUN5P2	0.38699487	2.60E-19	postive
MT-CO3	NUP50-DT	0.301891604	5.38E-12	postive
MT-CO3	OLMALINC	0.381896262	8.30E-19	postive
MT-CO3	OR2A9P	0.31320005	7.67E-13	postive
MT-CO3	PARD3-AS1	0.379123711	1.54E-18	postive
MT-CO3	PAXIP1-AS1	0.375628179	3.35E-18	postive
MT-CO3	PCAT6	0.427195805	1.34E-23	postive
MT-CO3	PDZK1P1	0.31912038	2.67E-13	postive
MT-CO3	PGAM1P8	0.350580911	6.62E-16	postive
MT-CO3	PIK3IP1-AS1	0.378125049	1.93E-18	postive
MT-CO3	PMS2CL	0.306167688	2.60E-12	postive
MT-CO3	POLR2J4	0.485142109	7.00E-31	postive
MT-CO3	PPP1R14B-AS1	0.400196355	1.18E-20	postive
MT-CO3	PPP1R35-AS1	0.407153464	2.18E-21	postive

MT-CO3	PPT2-EGFL8	0.334599738	1.52E-14	postive
MT-CO3	PRKCZ-AS1	0.334059082	1.69E-14	postive
MT-CO3	PRMT5-AS1	0.360529499	8.57E-17	postive
MT-CO3	PRORSD1P	0.34286015	3.08E-15	postive
MT-CO3	PRR34-AS1	0.302667657	4.72E-12	postive
MT-CO3	PSMA3-AS1	0.315158703	5.43E-13	postive
MT-CO3	PTOV1-AS1	0.343143254	2.91E-15	postive
MT-CO3	PTOV1-AS2	0.443644556	1.59E-25	postive
MT-CO3	RAD51-AS1	0.327651406	5.62E-14	postive
MT-CO3	RALY-AS1	0.35961733	1.04E-16	postive
MT-CO3	RARA-AS1	0.351608079	5.38E-16	postive
MT-CO3	RASA4CP	0.3234826	1.21E-13	postive
MT-CO3	RASSF1-AS1	0.387281871	2.44E-19	postive
MT-CO3	RBM15-AS1	0.355248531	2.56E-16	postive
MT-CO3	RFPL3S	0.323542656	1.20E-13	postive
MT-CO3	RHPN1-AS1	0.308057763	1.88E-12	postive
MT-CO3	RNF216P1	0.368363656	1.63E-17	postive
MT-CO3	RP11-101E13.5	0.344043565	2.44E-15	postive
MT-CO3	RP11-111M22.4	0.435651971	1.42E-24	postive
MT-CO3	RP11-141C7.4	0.316334342	4.40E-13	postive
MT-CO3	RP11-175O19.4	0.341318179	4.16E-15	postive
MT-CO3	RP11-188P20.3	0.402206531	7.27E-21	postive
MT-CO3	RP11-192H23.8	0.39681027	2.64E-20	postive
MT-CO3	RP11-199F11.2	0.412567009	5.71E-22	postive
MT-CO3	RP11-226L15.5	0.30873349	1.67E-12	postive
MT-CO3	RP11-258C19.7	0.408722964	1.48E-21	postive
MT-CO3	RP11-2B6.3	0.351490745	5.51E-16	postive
MT-CO3	RP11-352G9.1	0.323887388	1.13E-13	postive
MT-CO3	RP11-455O6.9	0.400893913	9.98E-21	postive
MT-CO3	RP11-465N4.5	0.501114599	3.80E-33	postive
MT-CO3	RP11-492E3.51	0.324729448	9.64E-14	postive
MT-CO3	RP11-508N22.13	0.304450425	3.49E-12	postive
MT-CO3	RP11-529E10.6	0.429925162	6.55E-24	postive
MT-CO3	RP11-540B6.6	0.381437947	9.20E-19	postive
MT-CO3	RP11-658F2.8	0.376420791	2.81E-18	postive
MT-CO3	RP11-69E11.4	0.3305855	3.25E-14	postive
MT-CO3	RP3-461P17.10	0.339267967	6.20E-15	postive
MT-CO3	RP4-545L17.11	0.356248651	2.08E-16	postive
MT-CO3	RP5-1112D6.4	0.393443277	5.85E-20	postive
MT-CO3	RP5-1125A11.7	0.346130114	1.61E-15	postive
MT-CO3	RP5-937E21.8	0.311193445	1.09E-12	postive
MT-CO3	RP9P	0.36250884	5.66E-17	postive
MT-CO3	RPL12P25	0.429061277	8.23E-24	postive
MT-CO3	RPL37P6	0.314673093	5.91E-13	postive
MT-CO3	RPS10P7	0.326626986	6.80E-14	postive
MT-CO3	RRN3P3	0.421328414	6.18E-23	postive
MT-CO3	RRP7BP	0.324658247	9.77E-14	postive
MT-CO3	RUSC1-AS1	0.412499625	5.80E-22	postive
MT-CO3	SBNO1-AS1	0.368404005	1.62E-17	postive
MT-CO3	SDHAP1	0.324803343	9.51E-14	postive

MT-CO3	SEMA3F-AS1	0.403019071	5.98E-21	postive
MT-CO3	SEPTIN7P2	0.368196529	1.69E-17	postive
MT-CO3	SH3BP5-AS1	0.347573302	1.21E-15	postive
MT-CO3	SH3GL1P1	0.327711387	5.56E-14	postive
MT-CO3	SLC25A25-AS1	0.335373296	1.31E-14	postive
MT-CO3	SLC25A5-AS1	0.339799465	5.59E-15	postive
MT-CO3	SLC25A5P1	0.359278316	1.11E-16	postive
MT-CO3	SLC2A1-AS1	0.327952932	5.31E-14	postive
MT-CO3	SLC9A3-AS1	0.374632043	4.17E-18	postive
MT-CO3	SLX1A-SULT1A3	0.328605713	4.71E-14	postive
MT-CO3	SMG1P7	0.433509419	2.52E-24	postive
MT-CO3	SNAI3-AS1	0.311220979	1.09E-12	postive
MT-CO3	SNHG10	0.321399544	1.77E-13	postive
MT-CO3	SNHG11	0.381011563	1.01E-18	postive
MT-CO3	SNHG12	0.389720612	1.39E-19	postive
MT-CO3	SNHG15	0.320725838	2.00E-13	postive
MT-CO3	SNHG20	0.371633652	8.04E-18	postive
MT-CO3	SNHG25	0.322006425	1.59E-13	postive
MT-CO3	SNHG9	0.425812501	1.93E-23	postive
MT-CO3	SNRK-AS1	0.328079743	5.19E-14	postive
MT-CO3	SPAG5-AS1	0.327675344	5.59E-14	postive
MT-CO3	SREBF2-AS1	0.431111615	4.78E-24	postive
MT-CO3	SRRM2-AS1	0.331658712	2.66E-14	postive
MT-CO3	STAG3L5P	0.325366645	8.58E-14	postive
MT-CO3	STAG3L5P-PVRIG2P-PILRB	0.412790314	5.40E-22	postive
MT-CO3	STPG3-AS1	0.361832145	6.53E-17	postive
MT-CO3	SUCLA2-AS1	0.340525716	4.86E-15	postive
MT-CO3	SUZ12P1	0.322711359	1.40E-13	postive
MT-CO3	SYNGAP1-AS1	0.325357604	8.59E-14	postive
MT-CO3	TAPT1-AS1	0.317158208	3.80E-13	postive
MT-CO3	TFAP2A-AS1	0.317719479	3.44E-13	postive
MT-CO3	THAP9-AS1	0.300276484	7.06E-12	postive
MT-CO3	TMED2-DT	0.413894917	4.09E-22	postive
MT-CO3	TMEM147-AS1	0.336871948	9.85E-15	postive
MT-CO3	TMEM198B	0.353742073	3.48E-16	postive
MT-CO3	TMEM44-AS1	0.322664357	1.41E-13	postive
MT-CO3	TMEM51-AS1	0.302281697	5.04E-12	postive
MT-CO3	TNRC6C-AS1	0.322580565	1.43E-13	postive
MT-CO3	TOB1-AS1	0.314429545	6.17E-13	postive
MT-CO3	TONSL-AS1	0.312329277	8.94E-13	postive
MT-CO3	TTC28-AS1	0.302000739	5.28E-12	postive
MT-CO3	U47924.1	0.360149468	9.28E-17	postive
MT-CO3	U47924.3	0.325378504	8.56E-14	postive
MT-CO3	U62317.2	0.391305441	9.63E-20	postive
MT-CO3	U73166.1	0.339312585	6.15E-15	postive
MT-CO3	U91328.3	0.386019571	3.26E-19	postive
MT-CO3	UBOX5-AS1	0.351841473	5.13E-16	postive
MT-CO3	URAHP	0.412581848	5.69E-22	postive
MT-CO3	VASH1-AS1	0.411963453	6.63E-22	postive
MT-CO3	VPS13B-DT	0.353999077	3.31E-16	postive

MT-CO3	WAC-AS1	0.435646295	1.42E-24	postive
MT-CO3	WASH3P	0.385570555	3.61E-19	postive
MT-CO3	WWTR1-AS1	0.302325292	5.00E-12	postive
MT-CO3	Z69706.1	0.329732412	3.81E-14	postive
MT-CO3	Z69720.1	0.309001154	1.60E-12	postive
MT-CO3	Z92544.1	0.324091942	1.08E-13	postive
MT-CO3	Z92544.2	0.302567723	4.80E-12	postive
MT-CO3	Z94721.1	0.305649712	2.84E-12	postive
MT-CO3	Z95115.1	0.300138326	7.23E-12	postive
MT-CO3	Z97634.1	0.340599376	4.79E-15	postive
MT-CO3	Z97653.1	0.357452583	1.63E-16	postive
MT-CO3	ZFHX2-AS1	0.387880354	2.13E-19	postive
MT-CO3	ZKSCAN2-DT	0.343601893	2.66E-15	postive
MT-CO3	ZNF205-AS1	0.33202113	2.48E-14	postive
MT-CO3	ZNF213-AS1	0.428278866	1.01E-23	postive
MT-CO3	ZNF32-AS1	0.347034664	1.35E-15	postive
MT-CO3	ZNF32-AS2	0.316269088	4.45E-13	postive
MT-CO3	ZNF37BP	0.306496673	2.46E-12	postive
MT-CO3	ZNF426-DT	0.330839181	3.10E-14	postive
MT-CO3	ZNF436-AS1	0.334524807	1.54E-14	postive
MT-CO3	ZNF687-AS1	0.301884401	5.39E-12	postive
MT-CO3	ZNRD2-AS1	0.392174016	7.86E-20	postive
MT-CO3	ZSWIM8-AS1	0.31041548	1.25E-12	postive
MTF1	A1BG-AS1	0.332175277	2.41E-14	postive
MTF1	A2M-AS1	0.367320181	2.04E-17	postive
MTF1	AATBC	0.307271446	2.15E-12	postive
MTF1	ABALON	0.360003412	9.57E-17	postive
MTF1	AC000068.1	0.451395071	1.80E-26	postive
MTF1	AC000068.2	0.355078671	2.65E-16	postive
MTF1	AC000123.1	0.365190695	3.21E-17	postive
MTF1	AC000403.1	0.447712197	5.10E-26	postive
MTF1	AC002091.1	0.354907251	2.75E-16	postive
MTF1	AC002091.2	0.40456041	4.11E-21	postive
MTF1	AC002128.2	0.325267837	8.73E-14	postive
MTF1	AC002398.1	0.300685688	6.59E-12	postive
MTF1	AC002467.1	0.302577646	4.79E-12	postive
MTF1	AC002550.2	0.343510677	2.71E-15	postive
MTF1	AC004067.1	0.360814368	8.08E-17	postive
MTF1	AC004076.2	0.321767274	1.66E-13	postive
MTF1	AC004112.1	0.401804098	8.01E-21	postive
MTF1	AC004241.1	0.306117618	2.62E-12	postive
MTF1	AC004447.2	0.315812635	4.83E-13	postive
MTF1	AC004466.1	0.38729222	2.43E-19	postive
MTF1	AC004471.2	0.333948079	1.72E-14	postive
MTF1	AC004477.3	0.45604295	4.75E-27	postive
MTF1	AC004492.1	0.318917068	2.77E-13	postive
MTF1	AC004540.1	0.301463635	5.78E-12	postive
MTF1	AC004596.1	0.51147247	1.11E-34	postive
MTF1	AC004771.2	0.464300451	4.23E-28	postive
MTF1	AC004771.4	0.37656713	2.72E-18	postive

MTF1	AC004801.6	0.4379291	7.63E-25	postive
MTF1	AC004803.1	0.346597641	1.47E-15	postive
MTF1	AC004812.2	0.34760117	1.20E-15	postive
MTF1	AC004830.2	0.312494949	8.68E-13	postive
MTF1	AC004846.1	0.32600343	7.63E-14	postive
MTF1	AC004865.2	0.336835356	9.91E-15	postive
MTF1	AC004877.1	0.342067473	3.59E-15	postive
MTF1	AC004890.2	0.33205292	2.46E-14	postive
MTF1	AC004893.11	0.604180429	4.52E-51	postive
MTF1	AC004918.5	0.51378425	4.97E-35	postive
MTF1	AC004943.2	0.515627151	2.60E-35	postive
MTF1	AC004951.1	0.518279658	1.02E-35	postive
MTF1	AC004980.1	0.425778208	1.95E-23	postive
MTF1	AC005005.3	0.386260039	3.08E-19	postive
MTF1	AC005034.4	0.488039654	2.77E-31	postive
MTF1	AC005034.6	0.603444199	6.41E-51	postive
MTF1	AC005041.3	0.351799521	5.17E-16	postive
MTF1	AC005104.1	0.323922897	1.12E-13	postive
MTF1	AC005229.4	0.378660139	1.71E-18	postive
MTF1	AC005253.1	0.453877722	8.86E-27	postive
MTF1	AC005261.2	0.395915759	3.27E-20	postive
MTF1	AC005261.3	0.413409385	4.62E-22	postive
MTF1	AC005261.4	0.38134964	9.38E-19	postive
MTF1	AC005264.1	0.30308414	4.40E-12	postive
MTF1	AC005288.1	0.614338947	3.31E-53	postive
MTF1	AC005332.4	0.398714629	1.68E-20	postive
MTF1	AC005332.6	0.436755929	1.05E-24	postive
MTF1	AC005387.2	0.331034499	2.99E-14	postive
MTF1	AC005498.3	0.394335208	4.74E-20	postive
MTF1	AC005562.1	0.551365752	4.16E-41	postive
MTF1	AC005586.1	0.460868939	1.17E-27	postive
MTF1	AC005670.3	0.541995279	1.61E-39	postive
MTF1	AC005674.2	0.363005375	5.10E-17	postive
MTF1	AC005746.1	0.333019175	2.05E-14	postive
MTF1	AC005776.2	0.300214155	7.13E-12	postive
MTF1	AC005828.4	0.317692921	3.45E-13	postive
MTF1	AC005840.2	0.315830242	4.82E-13	postive
MTF1	AC005840.4	0.364818892	3.47E-17	postive
MTF1	AC005845.1	0.332984256	2.07E-14	postive
MTF1	AC006001.2	0.400462076	1.11E-20	postive
MTF1	AC006001.3	0.389115409	1.60E-19	postive
MTF1	AC006033.2	0.414577638	3.45E-22	postive
MTF1	AC006064.2	0.385534065	3.64E-19	postive
MTF1	AC006111.2	0.332422703	2.30E-14	postive
MTF1	AC006116.11	0.327351596	5.94E-14	postive
MTF1	AC006126.4	0.385468809	3.69E-19	postive
MTF1	AC006160.1	0.301462928	5.78E-12	postive
MTF1	AC006213.1	0.378505514	1.77E-18	postive
MTF1	AC006213.2	0.429272411	7.78E-24	postive
MTF1	AC006213.4	0.347591091	1.21E-15	postive

MTF1	AC006213.5	0.367943659	1.78E-17	postive
MTF1	AC006333.1	0.327665733	5.60E-14	postive
MTF1	AC006449.2	0.398219858	1.89E-20	postive
MTF1	AC006449.7	0.304238544	3.62E-12	postive
MTF1	AC006504.5	0.351891656	5.08E-16	postive
MTF1	AC006547.1	0.485364642	6.52E-31	postive
MTF1	AC007040.1	0.321340954	1.79E-13	postive
MTF1	AC007066.2	0.37971553	1.35E-18	postive
MTF1	AC007228.9	0.321978718	1.59E-13	postive
MTF1	AC007285.1	0.337884856	8.10E-15	postive
MTF1	AC007292.1	0.331537216	2.72E-14	postive
MTF1	AC007292.2	0.443958835	1.46E-25	postive
MTF1	AC007406.5	0.524945529	9.34E-37	postive
MTF1	AC007566.1	0.346106689	1.62E-15	postive
MTF1	AC007622.2	0.329165524	4.24E-14	postive
MTF1	AC007637.1	0.319082263	2.69E-13	postive
MTF1	AC007663.4	0.448034566	4.66E-26	postive
MTF1	AC007686.3	0.301271299	5.97E-12	postive
MTF1	AC007728.2	0.34004882	5.33E-15	postive
MTF1	AC007728.3	0.341275861	4.20E-15	postive
MTF1	AC007780.1	0.346638829	1.46E-15	postive
MTF1	AC007823.1	0.425333046	2.19E-23	postive
MTF1	AC007938.1	0.310946927	1.14E-12	postive
MTF1	AC007996.1	0.310207511	1.29E-12	postive
MTF1	AC008074.2	0.367214509	2.08E-17	postive
MTF1	AC008115.3	0.328076617	5.19E-14	postive
MTF1	AC008124.1	0.460751876	1.21E-27	postive
MTF1	AC008438.1	0.33119585	2.90E-14	postive
MTF1	AC008443.1	0.339386118	6.06E-15	postive
MTF1	AC008494.2	0.332422298	2.30E-14	postive
MTF1	AC008494.3	0.406628462	2.48E-21	postive
MTF1	AC008537.2	0.34307444	2.95E-15	postive
MTF1	AC008543.1	0.34716509	1.31E-15	postive
MTF1	AC008569.2	0.318275241	3.11E-13	postive
MTF1	AC008622.2	0.370455854	1.04E-17	postive
MTF1	AC008635.2	0.344738516	2.13E-15	postive
MTF1	AC008655.2	0.338550297	7.12E-15	postive
MTF1	AC008734.1	0.319281149	2.60E-13	postive
MTF1	AC008735.1	0.377312463	2.31E-18	postive
MTF1	AC008735.2	0.382528927	7.19E-19	postive
MTF1	AC008735.4	0.445153649	1.04E-25	postive
MTF1	AC008750.1	0.327341118	5.95E-14	postive
MTF1	AC008764.2	0.530206244	1.36E-37	postive
MTF1	AC008764.8	0.326893057	6.47E-14	postive
MTF1	AC008770.3	0.32727927	6.02E-14	postive
MTF1	AC008771.1	0.310838463	1.16E-12	postive
MTF1	AC008870.5	0.327819294	5.45E-14	postive
MTF1	AC008894.2	0.348465988	1.01E-15	postive
MTF1	AC008897.3	0.303097369	4.39E-12	postive
MTF1	AC008906.1	0.343377806	2.78E-15	postive

MTF1	AC008969.1	0.39348359	5.79E-20	postive
MTF1	AC008982.2	0.369668714	1.23E-17	postive
MTF1	AC009019.1	0.341467604	4.04E-15	postive
MTF1	AC009041.3	0.434467964	1.95E-24	postive
MTF1	AC009065.9	0.310683586	1.19E-12	postive
MTF1	AC009107.2	0.310654846	1.20E-12	postive
MTF1	AC009113.1	0.325302268	8.68E-14	postive
MTF1	AC009118.3	0.305664371	2.84E-12	postive
MTF1	AC009120.3	0.372419266	6.77E-18	postive
MTF1	AC009121.3	0.320828317	1.96E-13	postive
MTF1	AC009126.1	0.414813707	3.25E-22	postive
MTF1	AC009163.7	0.309173668	1.55E-12	postive
MTF1	AC009237.14	0.363191719	4.90E-17	postive
MTF1	AC009318.2	0.407725279	1.90E-21	postive
MTF1	AC009318.3	0.402216329	7.26E-21	postive
MTF1	AC009404.1	0.329339854	4.10E-14	postive
MTF1	AC009506.2	0.414210063	3.78E-22	postive
MTF1	AC009690.2	0.355358007	2.50E-16	postive
MTF1	AC009779.3	0.458794837	2.14E-27	postive
MTF1	AC009812.3	0.32194013	1.61E-13	postive
MTF1	AC009831.1	0.569029603	3.06E-44	postive
MTF1	AC009948.3	0.310499775	1.23E-12	postive
MTF1	AC009950.1	0.333643082	1.82E-14	postive
MTF1	AC009961.1	0.301536374	5.71E-12	postive
MTF1	AC010203.1	0.451038094	1.99E-26	postive
MTF1	AC010207.1	0.304519913	3.45E-12	postive
MTF1	AC010271.1	0.324022249	1.10E-13	postive
MTF1	AC010273.2	0.41144828	7.54E-22	postive
MTF1	AC010319.4	0.374181292	4.61E-18	postive
MTF1	AC010320.4	0.312393304	8.84E-13	postive
MTF1	AC010326.4	0.357224275	1.70E-16	postive
MTF1	AC010326.5	0.304618697	3.39E-12	postive
MTF1	AC010335.1	0.314267626	6.35E-13	postive
MTF1	AC010442.1	0.337175559	9.29E-15	postive
MTF1	AC010504.1	0.336798932	9.98E-15	postive
MTF1	AC010530.1	0.359057359	1.17E-16	postive
MTF1	AC010618.3	0.336469561	1.06E-14	postive
MTF1	AC010643.1	0.319717972	2.40E-13	postive
MTF1	AC010655.4	0.3257152	8.04E-14	postive
MTF1	AC010680.2	0.304956404	3.20E-12	postive
MTF1	AC010761.4	0.345931613	1.68E-15	postive
MTF1	AC010809.1	0.383578637	5.67E-19	postive
MTF1	AC010834.3	0.312201593	9.14E-13	postive
MTF1	AC010969.2	0.397683836	2.15E-20	postive
MTF1	AC010973.1	0.38805171	2.04E-19	postive
MTF1	AC011451.1	0.359467366	1.07E-16	postive
MTF1	AC011466.1	0.372830312	6.19E-18	postive
MTF1	AC011466.3	0.351859022	5.11E-16	postive
MTF1	AC011468.1	0.304125594	3.69E-12	postive
MTF1	AC011476.3	0.458872973	2.09E-27	postive

MTF1	AC011477.1	0.339846091	5.54E-15	postive
MTF1	AC011477.2	0.375039628	3.82E-18	postive
MTF1	AC011477.3	0.369000813	1.42E-17	postive
MTF1	AC011479.4	0.327827716	5.44E-14	postive
MTF1	AC011511.1	0.335537365	1.27E-14	postive
MTF1	AC011731.1	0.306685575	2.38E-12	postive
MTF1	AC011815.1	0.389934221	1.32E-19	postive
MTF1	AC011899.2	0.388729401	1.75E-19	postive
MTF1	AC011912.1	0.385244742	3.89E-19	postive
MTF1	AC012063.1	0.433358971	2.62E-24	postive
MTF1	AC012073.1	0.402653654	6.53E-21	postive
MTF1	AC012173.1	0.308290172	1.81E-12	postive
MTF1	AC012306.2	0.4038514	4.88E-21	postive
MTF1	AC012313.1	0.508020382	3.66E-34	postive
MTF1	AC012313.2	0.350188618	7.16E-16	postive
MTF1	AC012313.5	0.525807566	6.83E-37	postive
MTF1	AC012313.6	0.310049107	1.33E-12	postive
MTF1	AC012313.8	0.362485336	5.69E-17	postive
MTF1	AC012360.2	0.307196014	2.18E-12	postive
MTF1	AC012360.3	0.322327519	1.50E-13	postive
MTF1	AC012442.2	0.359602211	1.04E-16	postive
MTF1	AC012467.2	0.497186295	1.41E-32	postive
MTF1	AC012510.1	0.332180778	2.41E-14	postive
MTF1	AC012615.4	0.357204507	1.71E-16	postive
MTF1	AC012615.6	0.321603928	1.71E-13	postive
MTF1	AC012618.3	0.340228987	5.15E-15	postive
MTF1	AC012676.1	0.306534442	2.44E-12	postive
MTF1	AC013356.2	0.327120686	6.20E-14	postive
MTF1	AC013403.2	0.337802495	8.23E-15	postive
MTF1	AC013468.1	0.309722595	1.41E-12	postive
MTF1	AC015712.2	0.351306967	5.71E-16	postive
MTF1	AC015726.1	0.318384884	3.05E-13	postive
MTF1	AC015795.1	0.337275601	9.11E-15	postive
MTF1	AC015849.3	0.387201265	2.48E-19	postive
MTF1	AC015871.3	0.359827339	9.93E-17	postive
MTF1	AC015922.2	0.485089591	7.11E-31	postive
MTF1	AC015922.3	0.344246622	2.34E-15	postive
MTF1	AC016027.1	0.39602226	3.19E-20	postive
MTF1	AC016586.1	0.334549162	1.54E-14	postive
MTF1	AC016722.2	0.428248457	1.02E-23	postive
MTF1	AC016727.1	0.365879803	2.77E-17	postive
MTF1	AC016747.1	0.338919452	6.63E-15	postive
MTF1	AC016888.1	0.388220774	1.97E-19	postive
MTF1	AC017100.1	0.305109665	3.12E-12	postive
MTF1	AC018521.6	0.504756404	1.11E-33	postive
MTF1	AC018645.3	0.308309342	1.80E-12	postive
MTF1	AC018647.2	0.521859138	2.84E-36	postive
MTF1	AC018653.3	0.391337607	9.56E-20	postive
MTF1	AC018690.1	0.432354718	3.43E-24	postive
MTF1	AC018695.4	0.331266311	2.86E-14	postive

MTF1	AC018766.1	0.333956715	1.72E-14	postive
MTF1	AC018766.4	0.3388236	6.76E-15	postive
MTF1	AC018809.2	0.485447816	6.35E-31	postive
MTF1	AC019069.1	0.322342043	1.49E-13	postive
MTF1	AC019205.1	0.467789192	1.49E-28	postive
MTF1	AC020911.1	0.348989681	9.12E-16	postive
MTF1	AC020915.1	0.449321095	3.24E-26	postive
MTF1	AC020915.2	0.392121387	7.96E-20	postive
MTF1	AC020915.3	0.476750899	9.72E-30	postive
MTF1	AC020978.1	0.307048386	2.24E-12	postive
MTF1	AC020978.2	0.306274111	2.55E-12	postive
MTF1	AC021016.2	0.373558408	5.28E-18	postive
MTF1	AC021148.1	0.38385587	5.33E-19	postive
MTF1	AC021224.1	0.357263831	1.69E-16	postive
MTF1	AC021483.2	0.340157544	5.22E-15	postive
MTF1	AC021752.1	0.300737998	6.53E-12	postive
MTF1	AC022098.1	0.46639453	2.27E-28	postive
MTF1	AC022150.2	0.526963427	4.48E-37	postive
MTF1	AC022150.4	0.363049652	5.05E-17	postive
MTF1	AC022154.1	0.4055579	3.22E-21	postive
MTF1	AC022167.2	0.442982941	1.91E-25	postive
MTF1	AC022211.3	0.402063885	7.53E-21	postive
MTF1	AC022364.1	0.409814833	1.13E-21	postive
MTF1	AC022400.1	0.365779968	2.83E-17	postive
MTF1	AC022400.3	0.342210999	3.49E-15	postive
MTF1	AC022400.5	0.336706949	1.02E-14	postive
MTF1	AC022400.6	0.376894287	2.53E-18	postive
MTF1	AC022509.3	0.369407864	1.30E-17	postive
MTF1	AC022613.2	0.344624485	2.17E-15	postive
MTF1	AC022762.2	0.354871572	2.77E-16	postive
MTF1	AC022916.2	0.357781397	1.52E-16	postive
MTF1	AC023043.1	0.309517806	1.46E-12	postive
MTF1	AC023043.4	0.417393991	1.69E-22	postive
MTF1	AC023355.2	0.313625006	7.12E-13	postive
MTF1	AC023790.2	0.306770841	2.35E-12	postive
MTF1	AC024075.1	0.54828715	1.40E-40	postive
MTF1	AC024075.2	0.364686694	3.57E-17	postive
MTF1	AC024075.3	0.519313653	7.07E-36	postive
MTF1	AC024267.4	0.446052098	8.13E-26	postive
MTF1	AC024267.7	0.413402655	4.63E-22	postive
MTF1	AC024560.3	0.303454097	4.13E-12	postive
MTF1	AC024575.2	0.318957488	2.75E-13	postive
MTF1	AC024896.1	0.331936526	2.52E-14	postive
MTF1	AC025048.2	0.379961477	1.28E-18	postive
MTF1	AC025171.1	0.37201914	7.39E-18	postive
MTF1	AC025287.3	0.390299736	1.22E-19	postive
MTF1	AC025580.2	0.383979671	5.18E-19	postive
MTF1	AC025682.2	0.337038621	9.53E-15	postive
MTF1	AC025822.2	0.301060838	6.19E-12	postive
MTF1	AC026150.2	0.316494837	4.28E-13	postive

MTF1	AC026401.3	0.38820322	1.97E-19	postive
MTF1	AC026471.1	0.507342714	4.61E-34	postive
MTF1	AC026471.4	0.305161774	3.09E-12	postive
MTF1	AC026691.1	0.375459979	3.48E-18	postive
MTF1	AC026740.1	0.302175517	5.13E-12	postive
MTF1	AC026803.1	0.304141108	3.68E-12	postive
MTF1	AC026803.2	0.34917361	8.78E-16	postive
MTF1	AC026904.2	0.313710333	7.01E-13	postive
MTF1	AC026979.3	0.305479486	2.93E-12	postive
MTF1	AC027020.1	0.300113983	7.25E-12	postive
MTF1	AC027237.5	0.385791171	3.43E-19	postive
MTF1	AC027307.2	0.3521198	4.85E-16	postive
MTF1	AC027307.3	0.311970747	9.52E-13	postive
MTF1	AC027601.2	0.316491897	4.28E-13	postive
MTF1	AC032044.1	0.303175829	4.33E-12	postive
MTF1	AC034102.6	0.425074922	2.34E-23	postive
MTF1	AC037459.2	0.488003617	2.80E-31	postive
MTF1	AC044860.1	0.304091453	3.71E-12	postive
MTF1	AC046143.2	0.304345989	3.55E-12	postive
MTF1	AC060766.6	0.342762264	3.14E-15	postive
MTF1	AC060766.7	0.42705365	1.40E-23	postive
MTF1	AC060780.1	0.488293687	2.55E-31	postive
MTF1	AC064807.1	0.317590739	3.52E-13	postive
MTF1	AC066613.1	0.379110432	1.55E-18	postive
MTF1	AC067750.1	0.373990709	4.80E-18	postive
MTF1	AC067852.2	0.53897579	5.10E-39	postive
MTF1	AC067852.3	0.33500804	1.41E-14	postive
MTF1	AC067930.4	0.324619841	9.84E-14	postive
MTF1	AC068473.5	0.307686445	2.00E-12	postive
MTF1	AC068580.1	0.399143747	1.52E-20	postive
MTF1	AC068580.3	0.465542853	2.92E-28	postive
MTF1	AC068790.9	0.355403985	2.48E-16	postive
MTF1	AC068870.2	0.418149679	1.39E-22	postive
MTF1	AC069213.1	0.327430102	5.86E-14	postive
MTF1	AC072061.1	0.378845908	1.64E-18	postive
MTF1	AC073046.1	0.565277769	1.47E-43	postive
MTF1	AC073073.2	0.57056359	1.60E-44	postive
MTF1	AC073254.1	0.48257111	1.58E-30	postive
MTF1	AC073343.2	0.357943572	1.47E-16	postive
MTF1	AC073476.3	0.344234868	2.35E-15	postive
MTF1	AC073508.3	0.344160627	2.38E-15	postive
MTF1	AC073517.1	0.350160185	7.20E-16	postive
MTF1	AC073569.2	0.56719225	6.61E-44	postive
MTF1	AC073575.4	0.325436388	8.47E-14	postive
MTF1	AC073842.2	0.337332478	9.01E-15	postive
MTF1	AC073869.1	0.376537389	2.74E-18	postive
MTF1	AC073896.3	0.489249987	1.88E-31	postive
MTF1	AC074011.1	0.388835449	1.71E-19	postive
MTF1	AC074029.3	0.328125275	5.15E-14	postive
MTF1	AC074117.1	0.325232357	8.79E-14	postive

MTF1	AC074194.1	0.337642847	8.49E-15	postive
MTF1	AC078785.1	0.352076142	4.89E-16	postive
MTF1	AC078846.1	0.308838487	1.64E-12	postive
MTF1	AC079610.1	0.352879893	4.15E-16	postive
MTF1	AC079807.1	0.48826541	2.58E-31	postive
MTF1	AC079848.1	0.335869067	1.19E-14	postive
MTF1	AC079921.2	0.326783344	6.60E-14	postive
MTF1	AC080112.2	0.393655538	5.56E-20	postive
MTF1	AC083799.1	0.349636037	8.01E-16	postive
MTF1	AC083862.2	0.434472102	1.94E-24	postive
MTF1	AC083964.1	0.37127925	8.68E-18	postive
MTF1	AC084018.2	0.339887484	5.50E-15	postive
MTF1	AC087071.2	0.358306273	1.36E-16	postive
MTF1	AC087289.1	0.354045023	3.27E-16	postive
MTF1	AC087289.2	0.327069703	6.26E-14	postive
MTF1	AC087289.5	0.397244504	2.39E-20	postive
MTF1	AC087301.1	0.345346476	1.88E-15	postive
MTF1	AC087392.4	0.339919656	5.46E-15	postive
MTF1	AC087392.5	0.310378834	1.26E-12	postive
MTF1	AC087500.1	0.352734411	4.28E-16	postive
MTF1	AC087501.4	0.384643116	4.46E-19	postive
MTF1	AC087521.3	0.347510585	1.23E-15	postive
MTF1	AC087627.1	0.305445202	2.94E-12	postive
MTF1	AC087645.2	0.346390177	1.53E-15	postive
MTF1	AC090114.2	0.460807361	1.19E-27	postive
MTF1	AC090115.1	0.301767215	5.50E-12	postive
MTF1	AC090136.3	0.319691799	2.41E-13	postive
MTF1	AC090198.1	0.308407683	1.77E-12	postive
MTF1	AC090229.1	0.46847633	1.21E-28	postive
MTF1	AC090425.2	0.318958838	2.75E-13	postive
MTF1	AC090425.3	0.401375076	8.89E-21	postive
MTF1	AC090559.1	0.46454514	3.94E-28	postive
MTF1	AC090587.2	0.438755841	6.09E-25	postive
MTF1	AC090589.3	0.313771628	6.93E-13	postive
MTF1	AC090607.1	0.300057732	7.32E-12	postive
MTF1	AC090617.5	0.403636762	5.15E-21	postive
MTF1	AC091057.1	0.355583304	2.39E-16	postive
MTF1	AC091132.5	0.371992662	7.43E-18	postive
MTF1	AC091152.2	0.352848762	4.18E-16	postive
MTF1	AC091180.3	0.333698389	1.80E-14	postive
MTF1	AC091180.6	0.321372453	1.78E-13	postive
MTF1	AC091729.3	0.314995303	5.59E-13	postive
MTF1	AC091982.1	0.361498765	7.00E-17	postive
MTF1	AC092070.2	0.566619268	8.40E-44	postive
MTF1	AC092070.3	0.30000641	7.39E-12	postive
MTF1	AC092171.2	0.483005894	1.38E-30	postive
MTF1	AC092295.2	0.476708442	9.85E-30	postive
MTF1	AC092368.3	0.490511451	1.25E-31	postive
MTF1	AC092376.2	0.350778724	6.36E-16	postive
MTF1	AC092611.1	0.3163913	4.36E-13	postive

MTF1	AC092614.1	0.46908002	1.01E-28	postive
MTF1	AC092634.3	0.39786891	2.06E-20	postive
MTF1	AC092718.5	0.458938151	2.05E-27	postive
MTF1	AC092747.4	0.403134972	5.81E-21	postive
MTF1	AC092802.3	0.317889691	3.34E-13	postive
MTF1	AC092910.3	0.425600994	2.04E-23	postive
MTF1	AC092953.2	0.434227727	2.08E-24	postive
MTF1	AC093010.3	0.318047817	3.24E-13	postive
MTF1	AC093227.1	0.335691785	1.23E-14	postive
MTF1	AC093278.2	0.393013101	6.46E-20	postive
MTF1	AC093297.2	0.388486803	1.85E-19	postive
MTF1	AC093495.1	0.355628439	2.37E-16	postive
MTF1	AC093525.4	0.305634117	2.85E-12	postive
MTF1	AC093525.7	0.341717954	3.85E-15	postive
MTF1	AC093582.1	0.322262243	1.51E-13	postive
MTF1	AC093627.5	0.43509553	1.64E-24	postive
MTF1	AC093726.1	0.382938018	6.56E-19	postive
MTF1	AC093726.2	0.311037681	1.12E-12	postive
MTF1	AC093752.1	0.411710754	7.07E-22	postive
MTF1	AC093752.3	0.378827018	1.65E-18	postive
MTF1	AC096536.2	0.409134762	1.34E-21	postive
MTF1	AC096540.1	0.336247146	1.11E-14	postive
MTF1	AC096677.1	0.343422799	2.76E-15	postive
MTF1	AC096733.3	0.502992232	2.02E-33	postive
MTF1	AC096772.6	0.498256311	9.86E-33	postive
MTF1	AC096992.2	0.480127598	3.40E-30	postive
MTF1	AC097359.2	0.364427467	3.77E-17	postive
MTF1	AC097376.3	0.336524814	1.05E-14	postive
MTF1	AC097382.2	0.378898168	1.62E-18	postive
MTF1	AC097468.3	0.368577025	1.56E-17	postive
MTF1	AC097639.1	0.351019028	6.06E-16	postive
MTF1	AC097641.2	0.309056075	1.58E-12	postive
MTF1	AC098484.1	0.640047542	5.62E-59	postive
MTF1	AC098484.2	0.338292155	7.49E-15	postive
MTF1	AC098934.2	0.324855953	9.42E-14	postive
MTF1	AC099343.4	0.405305533	3.43E-21	postive
MTF1	AC099518.4	0.301240672	6.01E-12	postive
MTF1	AC099548.2	0.37078926	9.65E-18	postive
MTF1	AC099778.1	0.415206164	2.94E-22	postive
MTF1	AC099850.4	0.371491903	8.29E-18	postive
MTF1	AC100778.2	0.320012465	2.28E-13	postive
MTF1	AC100793.3	0.318210775	3.15E-13	postive
MTF1	AC100830.2	0.324068458	1.09E-13	postive
MTF1	AC100849.1	0.368347536	1.63E-17	postive
MTF1	AC100861.1	0.34403555	2.44E-15	postive
MTF1	AC103974.1	0.30459469	3.40E-12	postive
MTF1	AC104083.1	0.365170355	3.22E-17	postive
MTF1	AC104109.2	0.415023444	3.08E-22	postive
MTF1	AC104109.4	0.412220348	6.22E-22	postive
MTF1	AC104113.1	0.310063354	1.33E-12	postive

MTF1	AC104118.1	0.313698901	7.02E-13	postive
MTF1	AC104411.2	0.405861996	2.99E-21	postive
MTF1	AC104532.2	0.382625943	7.04E-19	postive
MTF1	AC104564.3	0.348686447	9.69E-16	postive
MTF1	AC104596.1	0.325172665	8.89E-14	postive
MTF1	AC104794.3	0.378119698	1.93E-18	postive
MTF1	AC104819.3	0.34771854	1.18E-15	postive
MTF1	AC104825.1	0.437619195	8.30E-25	postive
MTF1	AC104938.1	0.339808327	5.58E-15	postive
MTF1	AC105020.6	0.408099515	1.73E-21	postive
MTF1	AC105036.3	0.374509144	4.29E-18	postive
MTF1	AC105206.2	0.516156845	2.16E-35	postive
MTF1	AC105219.1	0.347867195	1.14E-15	postive
MTF1	AC105285.1	0.356493786	1.98E-16	postive
MTF1	AC105345.1	0.339635927	5.77E-15	postive
MTF1	AC105935.1	0.376856334	2.56E-18	postive
MTF1	AC105942.1	0.340521495	4.86E-15	postive
MTF1	AC106739.1	0.490027368	1.46E-31	postive
MTF1	AC106782.5	0.324161518	1.07E-13	postive
MTF1	AC106786.2	0.420625479	7.41E-23	postive
MTF1	AC106820.5	0.421785957	5.49E-23	postive
MTF1	AC107021.2	0.3023828	4.95E-12	postive
MTF1	AC107027.3	0.48647297	4.58E-31	postive
MTF1	AC107068.1	0.423249641	3.76E-23	postive
MTF1	AC107214.1	0.304232736	3.62E-12	postive
MTF1	AC107214.2	0.348274908	1.05E-15	postive
MTF1	AC107959.1	0.360632527	8.39E-17	postive
MTF1	AC107982.3	0.336320767	1.09E-14	postive
MTF1	AC108010.1	0.490790404	1.14E-31	postive
MTF1	AC108134.1	0.312644787	8.46E-13	postive
MTF1	AC108449.2	0.40213219	7.40E-21	postive
MTF1	AC108463.2	0.365860086	2.78E-17	postive
MTF1	AC108488.1	0.383980665	5.18E-19	postive
MTF1	AC108693.2	0.318612535	2.93E-13	postive
MTF1	AC109347.1	0.409081126	1.36E-21	postive
MTF1	AC109449.1	0.371282275	8.67E-18	postive
MTF1	AC109454.2	0.439064986	5.60E-25	postive
MTF1	AC109460.1	0.37811431	1.93E-18	postive
MTF1	AC109460.2	0.439604792	4.83E-25	postive
MTF1	AC110079.1	0.446214197	7.77E-26	postive
MTF1	AC110995.1	0.327257954	6.05E-14	postive
MTF1	AC112220.2	0.485656506	5.94E-31	postive
MTF1	AC112236.2	0.301625357	5.63E-12	postive
MTF1	AC112694.1	0.391455863	9.30E-20	postive
MTF1	AC112907.3	0.347455718	1.24E-15	postive
MTF1	AC114271.1	0.534625138	2.63E-38	postive
MTF1	AC114341.1	0.350082831	7.32E-16	postive
MTF1	AC114488.2	0.346467396	1.51E-15	postive
MTF1	AC114490.1	0.387385897	2.38E-19	postive
MTF1	AC114730.2	0.303797117	3.90E-12	postive

MTF1	AC114956.2	0.423920694	3.16E-23	postive
MTF1	AC115618.1	0.305191861	3.07E-12	postive
MTF1	AC115618.2	0.489884143	1.53E-31	postive
MTF1	AC116366.2	0.354703413	2.86E-16	postive
MTF1	AC116447.1	0.35076317	6.38E-16	postive
MTF1	AC116667.1	0.305708259	2.81E-12	postive
MTF1	AC116913.1	0.396063436	3.16E-20	postive
MTF1	AC117394.2	0.32111393	1.87E-13	postive
MTF1	AC118344.1	0.359366289	1.09E-16	postive
MTF1	AC119674.1	0.388278753	1.94E-19	postive
MTF1	AC120114.1	0.459759077	1.61E-27	postive
MTF1	AC121338.2	0.421153662	6.47E-23	postive
MTF1	AC121761.1	0.32672284	6.68E-14	postive
MTF1	AC122129.1	0.414324342	3.67E-22	postive
MTF1	AC123912.1	0.333211971	1.98E-14	postive
MTF1	AC124045.1	0.361158863	7.51E-17	postive
MTF1	AC124283.2	0.403897803	4.83E-21	postive
MTF1	AC125257.1	0.472972702	3.11E-29	postive
MTF1	AC125494.1	0.438271634	6.95E-25	postive
MTF1	AC125494.2	0.308247782	1.82E-12	postive
MTF1	AC125807.2	0.521329446	3.44E-36	postive
MTF1	AC126755.1	0.441267071	3.06E-25	postive
MTF1	AC127502.1	0.381081805	9.96E-19	postive
MTF1	AC127521.1	0.351399372	5.61E-16	postive
MTF1	AC129510.1	0.371656371	8.00E-18	postive
MTF1	AC130371.2	0.422626116	4.42E-23	postive
MTF1	AC131009.3	0.315639203	4.98E-13	postive
MTF1	AC131097.3	0.308694374	1.68E-12	postive
MTF1	AC131159.2	0.438588062	6.38E-25	postive
MTF1	AC131235.3	0.306538927	2.44E-12	postive
MTF1	AC132008.2	0.428894081	8.60E-24	postive
MTF1	AC132872.1	0.306539242	2.44E-12	postive
MTF1	AC132938.4	0.401526966	8.57E-21	postive
MTF1	AC133528.2	0.307181128	2.19E-12	postive
MTF1	AC133552.2	0.343322623	2.81E-15	postive
MTF1	AC135050.1	0.393515106	5.75E-20	postive
MTF1	AC135050.4	0.310987158	1.13E-12	postive
MTF1	AC135050.5	0.447740981	5.06E-26	postive
MTF1	AC135050.6	0.32433229	1.04E-13	postive
MTF1	AC135178.4	0.400205664	1.18E-20	postive
MTF1	AC135983.3	0.403935172	4.79E-21	postive
MTF1	AC136604.2	0.329670339	3.86E-14	postive
MTF1	AC137630.1	0.41477726	3.28E-22	postive
MTF1	AC138028.2	0.453129367	1.10E-26	postive
MTF1	AC138028.4	0.360009235	9.56E-17	postive
MTF1	AC138028.6	0.348191111	1.07E-15	postive
MTF1	AC138035.1	0.45085781	2.10E-26	postive
MTF1	AC138207.1	0.300683116	6.59E-12	postive
MTF1	AC138207.4	0.398148394	1.92E-20	postive
MTF1	AC138207.5	0.352308372	4.66E-16	postive

MTF1	AC138207.6	0.409224689	1.31E-21	postive
MTF1	AC138207.8	0.323715901	1.16E-13	postive
MTF1	AC138356.1	0.311460564	1.04E-12	postive
MTF1	AC138932.1	0.495162252	2.74E-32	postive
MTF1	AC138956.1	0.322701652	1.40E-13	postive
MTF1	AC138956.2	0.392191283	7.83E-20	postive
MTF1	AC139149.1	0.330924256	3.05E-14	postive
MTF1	AC139256.2	0.30402585	3.75E-12	postive
MTF1	AC139256.3	0.353351234	3.77E-16	postive
MTF1	AC139768.1	0.355332034	2.52E-16	postive
MTF1	AC139795.1	0.358100008	1.42E-16	postive
MTF1	AC139795.2	0.423015384	4.00E-23	postive
MTF1	AC139887.2	0.372028928	7.37E-18	postive
MTF1	AC141586.1	0.531511245	8.40E-38	postive
MTF1	AC142381.3	0.449329384	3.23E-26	postive
MTF1	AC144831.1	0.47748595	7.74E-30	postive
MTF1	AC145098.1	0.498269422	9.82E-33	postive
MTF1	AC145207.5	0.450791502	2.14E-26	postive
MTF1	AC145285.3	0.353361134	3.77E-16	postive
MTF1	AC145285.6	0.322341716	1.49E-13	postive
MTF1	AC226118.1	0.310332232	1.27E-12	postive
MTF1	AC234775.3	0.373877284	4.93E-18	postive
MTF1	AC241952.2	0.485044747	7.22E-31	postive
MTF1	AC242376.2	0.339448421	5.99E-15	postive
MTF1	AC242426.2	0.320293028	2.16E-13	postive
MTF1	AC243919.2	0.337457813	8.80E-15	postive
MTF1	AC244453.3	0.320254049	2.18E-13	postive
MTF1	AC245052.4	0.384593999	4.51E-19	postive
MTF1	AC245060.2	0.382567064	7.13E-19	postive
MTF1	AC245060.5	0.318140846	3.19E-13	postive
MTF1	AC245060.6	0.384017754	5.14E-19	postive
MTF1	AC245140.2	0.402260071	7.18E-21	postive
MTF1	AC245452.1	0.461364186	1.01E-27	postive
MTF1	ACTA2-AS1	0.315336762	5.26E-13	postive
MTF1	ACVR2B-AS1	0.398783067	1.65E-20	postive
MTF1	AD000671.3	0.384269777	4.85E-19	postive
MTF1	ADGRE4P	0.333746218	1.79E-14	postive
MTF1	ADGRF5P1	0.316636002	4.17E-13	postive
MTF1	ADNP-AS1	0.407474481	2.02E-21	postive
MTF1	ADPGK-AS1	0.345543347	1.81E-15	postive
MTF1	AF001548.2	0.370901631	9.42E-18	postive
MTF1	AF111167.2	0.414963783	3.13E-22	postive
MTF1	AF111169.3	0.35651405	1.97E-16	postive
MTF1	AF127936.5	0.328392972	4.90E-14	postive
MTF1	AFG3L1P	0.375717718	3.29E-18	postive
MTF1	AGBL5-IT1	0.362647885	5.50E-17	postive
MTF1	AL008721.2	0.375379935	3.54E-18	postive
MTF1	AL008726.1	0.454785993	6.83E-27	postive
MTF1	AL008729.1	0.302899847	4.54E-12	postive
MTF1	AL009178.2	0.330262029	3.45E-14	postive

MTF1	AL020996.1	0.448909736	3.64E-26	postive
MTF1	AL021391.1	0.321055429	1.89E-13	postive
MTF1	AL021707.3	0.449870624	2.78E-26	postive
MTF1	AL021707.5	0.329883819	3.71E-14	postive
MTF1	AL021707.7	0.30299787	4.46E-12	postive
MTF1	AL021878.2	0.333244539	1.97E-14	postive
MTF1	AL021937.1	0.452613226	1.27E-26	postive
MTF1	AL021937.4	0.427836527	1.14E-23	postive
MTF1	AL022157.1	0.301942856	5.34E-12	postive
MTF1	AL022238.3	0.384114824	5.02E-19	postive
MTF1	AL022328.1	0.310514987	1.23E-12	postive
MTF1	AL022328.2	0.31152918	1.03E-12	postive
MTF1	AL022476.1	0.315016522	5.56E-13	postive
MTF1	AL024498.1	0.303208167	4.31E-12	postive
MTF1	AL024507.3	0.39359908	5.64E-20	postive
MTF1	AL031055.1	0.309649459	1.43E-12	postive
MTF1	AL031123.2	0.354935062	2.73E-16	postive
MTF1	AL031282.1	0.45184265	1.59E-26	postive
MTF1	AL031282.2	0.440067423	4.26E-25	postive
MTF1	AL031320.2	0.384863067	4.24E-19	postive
MTF1	AL031600.1	0.322159635	1.54E-13	postive
MTF1	AL031670.1	0.393250219	6.12E-20	postive
MTF1	AL031709.1	0.309839228	1.38E-12	postive
MTF1	AL031714.1	0.361326384	7.26E-17	postive
MTF1	AL031717.1	0.344207668	2.36E-15	postive
MTF1	AL031775.1	0.407957555	1.79E-21	postive
MTF1	AL031846.2	0.323317735	1.25E-13	postive
MTF1	AL031985.3	0.552334306	2.83E-41	postive
MTF1	AL034550.1	0.361738618	6.65E-17	postive
MTF1	AL035252.4	0.345897617	1.69E-15	postive
MTF1	AL035530.2	0.33376092	1.78E-14	postive
MTF1	AL035587.1	0.607416121	9.62E-52	postive
MTF1	AL049569.1	0.314087955	6.56E-13	postive
MTF1	AL049597.2	0.431885148	3.89E-24	postive
MTF1	AL049779.1	0.419079575	1.10E-22	postive
MTF1	AL049795.1	0.337496292	8.73E-15	postive
MTF1	AL049796.1	0.471349493	5.09E-29	postive
MTF1	AL049840.2	0.370522449	1.02E-17	postive
MTF1	AL049840.4	0.598840366	5.59E-50	postive
MTF1	AL049840.5	0.590609884	2.46E-48	postive
MTF1	AL049840.6	0.436683003	1.07E-24	postive
MTF1	AL049871.1	0.324760943	9.59E-14	postive
MTF1	AL050309.1	0.355765435	2.30E-16	postive
MTF1	AL050341.2	0.418254995	1.36E-22	postive
MTF1	AL050343.2	0.424986711	2.40E-23	postive
MTF1	AL078581.2	0.502833066	2.13E-33	postive
MTF1	AL078644.2	0.458269951	2.49E-27	postive
MTF1	AL096678.1	0.314715159	5.87E-13	postive
MTF1	AL109809.1	0.320432854	2.11E-13	postive
MTF1	AL109811.1	0.338181699	7.65E-15	postive

MTF1	AL109955.1	0.379304467	1.48E-18	postive
MTF1	AL117327.1	0.355774016	2.30E-16	postive
MTF1	AL117335.1	0.32027865	2.17E-13	postive
MTF1	AL117336.2	0.306946991	2.28E-12	postive
MTF1	AL117350.2	0.30226658	5.05E-12	postive
MTF1	AL118506.1	0.466524273	2.18E-28	postive
MTF1	AL118558.1	0.4572461	3.35E-27	postive
MTF1	AL121603.2	0.336552429	1.05E-14	postive
MTF1	AL121772.3	0.318012674	3.26E-13	postive
MTF1	AL121894.2	0.345811264	1.72E-15	postive
MTF1	AL121944.2	0.435020241	1.68E-24	postive
MTF1	AL121987.2	0.351307383	5.71E-16	postive
MTF1	AL121992.1	0.325338512	8.62E-14	postive
MTF1	AL122010.1	0.593954967	5.36E-49	postive
MTF1	AL122023.3	0.330909026	3.06E-14	postive
MTF1	AL122035.1	0.524011182	1.31E-36	postive
MTF1	AL122035.2	0.432650568	3.17E-24	postive
MTF1	AL132780.1	0.33256133	2.24E-14	postive
MTF1	AL132780.2	0.319362802	2.56E-13	postive
MTF1	AL132989.1	0.304700826	3.34E-12	postive
MTF1	AL133297.2	0.317428006	3.62E-13	postive
MTF1	AL133355.1	0.348222251	1.06E-15	postive
MTF1	AL133367.1	0.333635718	1.83E-14	postive
MTF1	AL133371.2	0.368273182	1.66E-17	postive
MTF1	AL133406.2	0.427559638	1.22E-23	postive
MTF1	AL133520.1	0.353306306	3.81E-16	postive
MTF1	AL135791.1	0.347386097	1.26E-15	postive
MTF1	AL135999.2	0.366430712	2.46E-17	postive
MTF1	AL136040.1	0.338746367	6.86E-15	postive
MTF1	AL136084.3	0.351007062	6.07E-16	postive
MTF1	AL136368.1	0.335330878	1.32E-14	postive
MTF1	AL137003.1	0.375887164	3.17E-18	postive
MTF1	AL137058.2	0.419765079	9.23E-23	postive
MTF1	AL137127.1	0.343378814	2.78E-15	postive
MTF1	AL137186.2	0.322799512	1.37E-13	postive
MTF1	AL137244.1	0.335052366	1.39E-14	postive
MTF1	AL138756.1	0.393491542	5.78E-20	postive
MTF1	AL138921.2	0.346062461	1.64E-15	postive
MTF1	AL138995.1	0.358628634	1.27E-16	postive
MTF1	AL139011.1	0.414638426	3.39E-22	postive
MTF1	AL139035.1	0.372089717	7.28E-18	postive
MTF1	AL139260.2	0.328979324	4.39E-14	postive
MTF1	AL139286.2	0.351196692	5.84E-16	postive
MTF1	AL139287.1	0.464888788	3.55E-28	postive
MTF1	AL139397.1	0.328634579	4.68E-14	postive
MTF1	AL157392.3	0.364570079	3.66E-17	postive
MTF1	AL157400.4	0.339773351	5.62E-15	postive
MTF1	AL157832.1	0.329796347	3.77E-14	postive
MTF1	AL157871.5	0.38242376	7.37E-19	postive
MTF1	AL157932.1	0.404667829	4.00E-21	postive

MTF1	AL158212.3	0.513357063	5.77E-35	postive
MTF1	AL160006.1	0.619672174	2.32E-54	postive
MTF1	AL161729.1	0.32608995	7.50E-14	postive
MTF1	AL161785.1	0.346541961	1.49E-15	postive
MTF1	AL161891.1	0.316711977	4.12E-13	postive
MTF1	AL162231.2	0.397210576	2.40E-20	postive
MTF1	AL162274.1	0.304916928	3.22E-12	postive
MTF1	AL162274.2	0.309805345	1.39E-12	postive
MTF1	AL162311.3	0.32568954	8.08E-14	postive
MTF1	AL162377.1	0.408838946	1.44E-21	postive
MTF1	AL162424.1	0.31637219	4.37E-13	postive
MTF1	AL162431.1	0.473088977	3.00E-29	postive
MTF1	AL162586.1	0.313747022	6.96E-13	postive
MTF1	AL162595.1	0.435211169	1.59E-24	postive
MTF1	AL162727.2	0.300399526	6.92E-12	postive
MTF1	AL353194.1	0.372059503	7.33E-18	postive
MTF1	AL353593.1	0.392571898	7.17E-20	postive
MTF1	AL353625.1	0.462966908	6.28E-28	postive
MTF1	AL353743.1	0.392095502	8.01E-20	postive
MTF1	AL353796.1	0.402034579	7.58E-21	postive
MTF1	AL354696.2	0.322053005	1.57E-13	postive
MTF1	AL354733.3	0.358809522	1.23E-16	postive
MTF1	AL354740.1	0.34432303	2.31E-15	postive
MTF1	AL354760.1	0.312175794	9.18E-13	postive
MTF1	AL354809.1	0.310137428	1.31E-12	postive
MTF1	AL354836.1	0.314477216	6.12E-13	postive
MTF1	AL354892.2	0.352255615	4.71E-16	postive
MTF1	AL354892.3	0.321982019	1.59E-13	postive
MTF1	AL354928.1	0.310586713	1.21E-12	postive
MTF1	AL354989.1	0.310955686	1.14E-12	postive
MTF1	AL355075.2	0.313013136	7.93E-13	postive
MTF1	AL355488.1	0.304640837	3.38E-12	postive
MTF1	AL355574.1	0.427492624	1.24E-23	postive
MTF1	AL355803.1	0.302539967	4.82E-12	postive
MTF1	AL356019.2	0.41026128	1.01E-21	postive
MTF1	AL356481.1	0.486742129	4.20E-31	postive
MTF1	AL356481.3	0.400965037	9.81E-21	postive
MTF1	AL356488.3	0.362580785	5.57E-17	postive
MTF1	AL356599.1	0.382283059	7.60E-19	postive
MTF1	AL357033.3	0.356303197	2.06E-16	postive
MTF1	AL357054.4	0.309066743	1.58E-12	postive
MTF1	AL357079.1	0.40480264	3.88E-21	postive
MTF1	AL357140.2	0.364560124	3.67E-17	postive
MTF1	AL357874.1	0.319430753	2.53E-13	postive
MTF1	AL358074.1	0.332594754	2.22E-14	postive
MTF1	AL358472.2	0.313023046	7.91E-13	postive
MTF1	AL359076.1	0.313527619	7.24E-13	postive
MTF1	AL359091.3	0.312240649	9.08E-13	postive
MTF1	AL359220.1	0.303299353	4.24E-12	postive
MTF1	AL359317.1	0.350062116	7.35E-16	postive

MTF1	AL359532.1	0.335335096	1.32E-14	postive
MTF1	AL359643.2	0.31698891	3.92E-13	postive
MTF1	AL359643.3	0.378444867	1.80E-18	postive
MTF1	AL359715.1	0.316184352	4.52E-13	postive
MTF1	AL359715.2	0.348022308	1.11E-15	postive
MTF1	AL359715.3	0.406457101	2.59E-21	postive
MTF1	AL359921.1	0.403769529	4.98E-21	postive
MTF1	AL360181.2	0.371833787	7.69E-18	postive
MTF1	AL360270.1	0.4009493	9.84E-21	postive
MTF1	AL365330.1	0.342659951	3.20E-15	postive
MTF1	AL390066.2	0.317502305	3.57E-13	postive
MTF1	AL390195.2	0.419468822	9.96E-23	postive
MTF1	AL390195.3	0.326380024	7.11E-14	postive
MTF1	AL390728.4	0.381534365	9.00E-19	postive
MTF1	AL391121.1	0.377948521	2.01E-18	postive
MTF1	AL391244.2	0.389360342	1.51E-19	postive
MTF1	AL391825.1	0.323687542	1.17E-13	postive
MTF1	AL391832.3	0.385286836	3.85E-19	postive
MTF1	AL391834.2	0.320108671	2.24E-13	postive
MTF1	AL445231.1	0.35042542	6.83E-16	postive
MTF1	AL449106.1	0.381890438	8.31E-19	postive
MTF1	AL450263.1	0.317476659	3.59E-13	postive
MTF1	AL450306.1	0.376698367	2.65E-18	postive
MTF1	AL450326.1	0.553483412	1.79E-41	postive
MTF1	AL450998.2	0.395534831	3.57E-20	postive
MTF1	AL450998.3	0.430345023	5.86E-24	postive
MTF1	AL451042.1	0.355371061	2.50E-16	postive
MTF1	AL451042.2	0.381136234	9.84E-19	postive
MTF1	AL512274.1	0.324123637	1.08E-13	postive
MTF1	AL512408.1	0.359133764	1.15E-16	postive
MTF1	AL512791.1	0.405117055	3.59E-21	postive
MTF1	AL512791.2	0.349314336	8.54E-16	postive
MTF1	AL513165.1	0.486183092	5.02E-31	postive
MTF1	AL513165.2	0.315436435	5.17E-13	postive
MTF1	AL513327.1	0.34550046	1.83E-15	postive
MTF1	AL513477.1	0.363398708	4.69E-17	postive
MTF1	AL513534.3	0.350603874	6.59E-16	postive
MTF1	AL513550.1	0.407034786	2.25E-21	postive
MTF1	AL589765.4	0.325657962	8.13E-14	postive
MTF1	AL590133.1	0.300230567	7.11E-12	postive
MTF1	AL590369.1	0.364236958	3.93E-17	postive
MTF1	AL590385.2	0.336372779	1.08E-14	postive
MTF1	AL590617.2	0.353596243	3.59E-16	postive
MTF1	AL590666.1	0.338503737	7.19E-15	postive
MTF1	AL590729.1	0.360522676	8.59E-17	postive
MTF1	AL591684.2	0.426516072	1.61E-23	postive
MTF1	AL592148.3	0.372992491	5.98E-18	postive
MTF1	AL592166.1	0.442189484	2.38E-25	postive
MTF1	AL596202.1	0.386343114	3.02E-19	postive
MTF1	AL596325.2	0.325483464	8.39E-14	postive

MTF1	AL603756.1	0.307786541	1.97E-12	postive
MTF1	AL603832.1	0.354714363	2.86E-16	postive
MTF1	AL603839.2	0.514450413	3.94E-35	postive
MTF1	AL603839.3	0.40739208	2.06E-21	postive
MTF1	AL604028.1	0.520393082	4.81E-36	postive
MTF1	AL606491.1	0.335332676	1.32E-14	postive
MTF1	AL606760.1	0.368688187	1.52E-17	postive
MTF1	AL606760.2	0.363692135	4.41E-17	postive
MTF1	AL606760.3	0.352611555	4.39E-16	postive
MTF1	AL606834.1	0.350142362	7.23E-16	postive
MTF1	AL606834.2	0.421006001	6.72E-23	postive
MTF1	AL627309.5	0.464348837	4.17E-28	postive
MTF1	AL645568.1	0.371799545	7.75E-18	postive
MTF1	AL645728.1	0.451768856	1.62E-26	postive
MTF1	AL645941.1	0.34903338	9.04E-16	postive
MTF1	AL662797.2	0.30577868	2.78E-12	postive
MTF1	AL669831.1	0.528644763	2.42E-37	postive
MTF1	AL671710.1	0.335562327	1.27E-14	postive
MTF1	AL683813.2	0.35727178	1.69E-16	postive
MTF1	AL691432.1	0.340707443	4.69E-15	postive
MTF1	AL691432.2	0.397965939	2.01E-20	postive
MTF1	AL731563.4	0.380810855	1.06E-18	postive
MTF1	AL731566.2	0.327600434	5.67E-14	postive
MTF1	AL731571.1	0.337343383	8.99E-15	postive
MTF1	AL731577.2	0.643509912	8.52E-60	postive
MTF1	AL732314.8	0.344047457	2.44E-15	postive
MTF1	AL732372.2	0.327510312	5.77E-14	postive
MTF1	AL844908.2	0.328275581	5.00E-14	postive
MTF1	AL928654.1	0.379005727	1.59E-18	postive
MTF1	ALG1L13P	0.319054987	2.71E-13	postive
MTF1	ALMS1P1	0.407836216	1.84E-21	postive
MTF1	ALOX12-AS1	0.395189071	3.88E-20	postive
MTF1	AMZ2P1	0.377391796	2.27E-18	postive
MTF1	ANO7L1	0.354703712	2.86E-16	postive
MTF1	ANP32AP1	0.344423966	2.26E-15	postive
MTF1	AOC4P	0.300696618	6.58E-12	postive
MTF1	AP000254.2	0.3275961	5.68E-14	postive
MTF1	AP000317.1	0.303742825	3.93E-12	postive
MTF1	AP000317.2	0.304013547	3.76E-12	postive
MTF1	AP000347.1	0.388614773	1.80E-19	postive
MTF1	AP000442.1	0.486889216	4.01E-31	postive
MTF1	AP000487.1	0.380072092	1.25E-18	postive
MTF1	AP000692.1	0.399434926	1.42E-20	postive
MTF1	AP000759.1	0.56407455	2.42E-43	postive
MTF1	AP000763.3	0.301394589	5.85E-12	postive
MTF1	AP000766.1	0.414370944	3.63E-22	postive
MTF1	AP000781.1	0.316974288	3.93E-13	postive
MTF1	AP000808.1	0.490099246	1.43E-31	postive
MTF1	AP000866.1	0.333886323	1.74E-14	postive
MTF1	AP000873.2	0.304068852	3.72E-12	postive

MTF1	AP000919.4	0.309905326	1.36E-12	postive
MTF1	AP001062.1	0.302281889	5.04E-12	postive
MTF1	AP001107.1	0.524353096	1.16E-36	postive
MTF1	AP001107.4	0.379030504	1.58E-18	postive
MTF1	AP001107.6	0.302766367	4.64E-12	postive
MTF1	AP001160.3	0.422116461	5.04E-23	postive
MTF1	AP001189.3	0.340512876	4.87E-15	postive
MTF1	AP001267.1	0.347381485	1.26E-15	postive
MTF1	AP001318.1	0.345292704	1.90E-15	postive
MTF1	AP001318.2	0.398147616	1.92E-20	postive
MTF1	AP001330.4	0.371237331	8.76E-18	postive
MTF1	AP001372.2	0.412345066	6.03E-22	postive
MTF1	AP001432.1	0.419402995	1.01E-22	postive
MTF1	AP001453.2	0.341368843	4.12E-15	postive
MTF1	AP001453.4	0.386515652	2.91E-19	postive
MTF1	AP001462.1	0.438638715	6.29E-25	postive
MTF1	AP001469.2	0.375853452	3.19E-18	postive
MTF1	AP001486.2	0.433189351	2.74E-24	postive
MTF1	AP001505.1	0.37772666	2.11E-18	postive
MTF1	AP001542.3	0.354659252	2.89E-16	postive
MTF1	AP001793.1	0.329376924	4.07E-14	postive
MTF1	AP001893.1	0.400205072	1.18E-20	postive
MTF1	AP001922.6	0.300710184	6.56E-12	postive
MTF1	AP002490.1	0.370554394	1.02E-17	postive
MTF1	AP002761.4	0.471405851	5.01E-29	postive
MTF1	AP002807.1	0.325960847	7.69E-14	postive
MTF1	AP002840.2	0.441649778	2.76E-25	postive
MTF1	AP002954.1	0.304135641	3.68E-12	postive
MTF1	AP003031.3	0.301760732	5.50E-12	postive
MTF1	AP003068.2	0.315203676	5.38E-13	postive
MTF1	AP003096.1	0.360963893	7.83E-17	postive
MTF1	AP003392.1	0.402797098	6.31E-21	postive
MTF1	AP003392.4	0.330548346	3.27E-14	postive
MTF1	AP003392.5	0.334491467	1.55E-14	postive
MTF1	AP003486.1	0.599288147	4.54E-50	postive
MTF1	AP003498.1	0.310834521	1.16E-12	postive
MTF1	AP003680.1	0.312626209	8.48E-13	postive
MTF1	AP003721.4	0.394116324	4.99E-20	postive
MTF1	AP005061.2	0.32789636	5.37E-14	postive
MTF1	AP005482.4	0.318283792	3.11E-13	postive
MTF1	AP006623.1	0.339058916	6.46E-15	postive
MTF1	ARHGEF35-AS1	0.33287151	2.11E-14	postive
MTF1	ARMCX5-GPRASP2	0.476268218	1.13E-29	postive
MTF1	ARNILA	0.422080804	5.09E-23	postive
MTF1	ARRDC1-AS1	0.403122463	5.83E-21	postive
MTF1	ASB16-AS1	0.460302041	1.38E-27	postive
MTF1	ATF4P4	0.318391917	3.05E-13	postive
MTF1	ATP2B1-AS1	0.388878431	1.69E-19	postive
MTF1	ATP6V0E2-AS1	0.325006586	9.16E-14	postive
MTF1	ATXN1-AS1	0.437483804	8.62E-25	postive

MTF1	BAALC-AS1	0.358911567	1.20E-16	postive
MTF1	BACE1-AS	0.44737071	5.62E-26	postive
MTF1	BAIAP2-DT	0.378933009	1.61E-18	postive
MTF1	BCDIN3D-AS1	0.437457626	8.68E-25	postive
MTF1	BDNF-AS	0.409135789	1.34E-21	postive
MTF1	BEND3P1	0.323653	1.17E-13	postive
MTF1	BHLHE40-AS1	0.332803598	2.14E-14	postive
MTF1	BMS1P1	0.372580335	6.54E-18	postive
MTF1	BMS1P2	0.333065886	2.04E-14	postive
MTF1	BMS1P20	0.534745565	2.52E-38	postive
MTF1	BMS1P4	0.329649886	3.87E-14	postive
MTF1	BNIP3P1	0.3000471	7.34E-12	postive
MTF1	BOLA2P2	0.34675598	1.42E-15	postive
MTF1	BTG3-AS1	0.372440621	6.74E-18	postive
MTF1	BTN2A3P	0.509251788	2.39E-34	postive
MTF1	BX322234.1	0.329478951	4.00E-14	postive
MTF1	BX322557.10	0.588464846	6.49E-48	postive
MTF1	BX322639.1	0.304866259	3.25E-12	postive
MTF1	C10orf25	0.428893062	8.60E-24	postive
MTF1	C1orf147	0.340976496	4.45E-15	postive
MTF1	C1orf220	0.322506854	1.45E-13	postive
MTF1	C1RL-AS1	0.42551926	2.09E-23	postive
MTF1	C21orf62-AS1	0.318371534	3.06E-13	postive
MTF1	C22orf24	0.308511136	1.74E-12	postive
MTF1	C22orf46	0.541957979	1.63E-39	postive
MTF1	C2CD4D-AS1	0.300750029	6.52E-12	postive
MTF1	C5orf66	0.401543034	8.53E-21	postive
MTF1	CA5BP1	0.477525009	7.65E-30	postive
MTF1	CACTIN-AS1	0.38319892	6.18E-19	postive
MTF1	CAPN10-DT	0.331614376	2.68E-14	postive
MTF1	CASC2	0.340938912	4.48E-15	postive
MTF1	CATSPER2P1	0.39593099	3.26E-20	postive
MTF1	CCDC183-AS1	0.418007411	1.45E-22	postive
MTF1	CCDC84-DT	0.44621919	7.75E-26	postive
MTF1	CCNT2-AS1	0.382471042	7.29E-19	postive
MTF1	CCT6P1	0.445326664	9.95E-26	postive
MTF1	CCT6P3	0.369972664	1.15E-17	postive
MTF1	CD27-AS1	0.437116642	9.52E-25	postive
MTF1	CD99P1	0.340678468	4.71E-15	postive
MTF1	CENPBD1P1	0.639257753	8.62E-59	postive
MTF1	CEP250-AS1	0.309632251	1.43E-12	postive
MTF1	CEP83-DT	0.301752052	5.51E-12	postive
MTF1	CERNA1	0.321312709	1.80E-13	postive
MTF1	CFL1P1	0.325309586	8.67E-14	postive
MTF1	CHKB-CPT1B	0.332478454	2.27E-14	postive
MTF1	CHROMR	0.405756235	3.07E-21	postive
MTF1	CIDECP1	0.4048881	3.80E-21	postive
MTF1	CIRBP-AS1	0.35542122	2.47E-16	postive
MTF1	CKMT2-AS1	0.410591594	9.33E-22	postive
MTF1	CLUHP3	0.302669106	4.72E-12	postive

MTF1	COX10-AS1	0.492779941	5.98E-32	postive
MTF1	CROCCP2	0.444994963	1.09E-25	postive
MTF1	CROCCP3	0.36573486	2.86E-17	postive
MTF1	CRYBB2P1	0.42131507	6.20E-23	postive
MTF1	CRYZL2P	0.375622865	3.36E-18	postive
MTF1	CSNK1G2-AS1	0.315876392	4.78E-13	postive
MTF1	CTA-14H9.5	0.305612361	2.86E-12	postive
MTF1	CTA-280A3.1	0.306463309	2.47E-12	postive
MTF1	CTBP1-AS	0.314331356	6.28E-13	postive
MTF1	CTBP1-DT	0.542328344	1.42E-39	postive
MTF1	CTC-205M6.5	0.469608377	8.63E-29	postive
MTF1	CTC-487M23.5	0.488558574	2.35E-31	postive
MTF1	CTC-786C10.1	0.431524617	4.28E-24	postive
MTF1	CTD-2245F17.3	0.543145471	1.03E-39	postive
MTF1	CTD-2521M24.9	0.315767308	4.87E-13	postive
MTF1	CTD-2574D22.4	0.492979912	5.60E-32	postive
MTF1	CUTALP	0.411757589	6.98E-22	postive
MTF1	CYP2F2P	0.338361701	7.39E-15	postive
MTF1	CYP4A22-AS1	0.390201587	1.24E-19	postive
MTF1	DACT3-AS1	0.386095345	3.20E-19	postive
MTF1	DENND10P1	0.332298853	2.35E-14	postive
MTF1	DGCR11	0.347266638	1.29E-15	postive
MTF1	DHRS4-AS1	0.351078711	5.98E-16	postive
MTF1	DLG5-AS1	0.312066736	9.36E-13	postive
MTF1	DNAJC3-DT	0.335012086	1.41E-14	postive
MTF1	DNMBP-AS1	0.383143851	6.26E-19	postive
MTF1	DOCK8-AS1	0.314752716	5.83E-13	postive
MTF1	DPP9-AS1	0.341883262	3.73E-15	postive
MTF1	DPY19L2P3	0.504004974	1.44E-33	postive
MTF1	DSCR9	0.321677288	1.68E-13	postive
MTF1	DSG2-AS1	0.362810423	5.31E-17	postive
MTF1	DSTNP2	0.392610827	7.10E-20	postive
MTF1	DTX2P1-UPK3BP1-PMS2P11	0.41245513	5.87E-22	postive
MTF1	DUBR	0.405242882	3.48E-21	postive
MTF1	EAF1-AS1	0.446165894	7.87E-26	postive
MTF1	EBLN3P	0.542717927	1.22E-39	postive
MTF1	ECI2-DT	0.417013266	1.86E-22	postive
MTF1	EDRF1-DT	0.303373632	4.19E-12	postive
MTF1	EIF1B-AS1	0.32929178	4.14E-14	postive
MTF1	EIF2AK3-DT	0.372999569	5.97E-18	postive
MTF1	EIF3J-DT	0.446556411	7.06E-26	postive
MTF1	ELF3-AS1	0.348858754	9.36E-16	postive
MTF1	ELOA-AS1	0.336155498	1.13E-14	postive
MTF1	EMC1-AS1	0.405062205	3.64E-21	postive
MTF1	EMC3-AS1	0.363986373	4.14E-17	postive
MTF1	ENTPD1-AS1	0.360932292	7.88E-17	postive
MTF1	EP400P1	0.429674072	7.00E-24	postive
MTF1	EPB41L4A-DT	0.48981678	1.56E-31	postive
MTF1	ERVK13-1	0.344295845	2.32E-15	postive
MTF1	FAM111A-DT	0.522801012	2.03E-36	postive

MTF1	FAM157A	0.347736332	1.17E-15	postive
MTF1	FAM157B	0.368156877	1.70E-17	postive
MTF1	FAM157C	0.375178219	3.70E-18	postive
MTF1	FAM160A1-DT	0.448766392	3.79E-26	postive
MTF1	FAM215B	0.368509945	1.58E-17	postive
MTF1	FAM21FP	0.309979519	1.35E-12	postive
MTF1	FAM66C	0.320825917	1.97E-13	postive
MTF1	FAM86C2P	0.357106999	1.75E-16	postive
MTF1	FAM86DP	0.410610726	9.29E-22	postive
MTF1	FAM86EP	0.415995078	2.41E-22	postive
MTF1	FAM86FP	0.305064165	3.14E-12	postive
MTF1	FAM86HP	0.392264142	7.70E-20	postive
MTF1	FAM86JP	0.400790663	1.02E-20	postive
MTF1	FBXL19-AS1	0.308148374	1.85E-12	postive
MTF1	FGD5-AS1	0.559261863	1.74E-42	postive
MTF1	FGF7P3	0.384608041	4.49E-19	postive
MTF1	FLNB-AS1	0.331308643	2.84E-14	postive
MTF1	FOXD2-AS1	0.437263828	9.15E-25	postive
MTF1	FRG1-DT	0.32060161	2.05E-13	postive
MTF1	FRG1HP	0.499952134	5.61E-33	postive
MTF1	FRMD6-AS1	0.318966346	2.75E-13	postive
MTF1	GAS5-AS1	0.30992339	1.36E-12	postive
MTF1	GATA2-AS1	0.318276938	3.11E-13	postive
MTF1	GCC2-AS1	0.341858798	3.74E-15	postive
MTF1	GEMIN7-AS1	0.372977058	6.00E-18	postive
MTF1	GGTA1P	0.347054832	1.34E-15	postive
MTF1	GHET1	0.428161863	1.04E-23	postive
MTF1	GLUD1P3	0.352856609	4.17E-16	postive
MTF1	GMDS-DT	0.328030298	5.24E-14	postive
MTF1	GNG12-AS1	0.337524827	8.68E-15	postive
MTF1	GNRHR2	0.427250012	1.33E-23	postive
MTF1	GOLGA2P5	0.345197081	1.94E-15	postive
MTF1	GOLGA2P7	0.335879148	1.19E-14	postive
MTF1	GPRC5D-AS1	0.375917758	3.15E-18	postive
MTF1	GRHL3-AS1	0.324340214	1.04E-13	postive
MTF1	GS1-124K5.3	0.503751349	1.56E-33	postive
MTF1	GS1-358P8.4	0.548170867	1.46E-40	postive
MTF1	GTF2IP1	0.537992448	7.40E-39	postive
MTF1	GTF2IP4	0.5825447	9.07E-47	postive
MTF1	GTF3C2-AS1	0.320918287	1.93E-13	postive
MTF1	GTSE1-DT	0.326535721	6.91E-14	postive
MTF1	GUSBP1	0.508319389	3.30E-34	postive
MTF1	GUSBP2	0.369326432	1.32E-17	postive
MTF1	GUSBP5	0.317437307	3.62E-13	postive
MTF1	H2BC19P	0.31740231	3.64E-13	postive
MTF1	H2BC20P	0.302331907	5.00E-12	postive
MTF1	H2BP1	0.335278801	1.34E-14	postive
MTF1	HCG11	0.510899391	1.36E-34	postive
MTF1	HCG18	0.577284507	9.03E-46	postive
MTF1	HCG25	0.380007449	1.27E-18	postive

MTF1	HEIH	0.325073725	9.05E-14	postive
MTF1	HERC2P2	0.30843475	1.76E-12	postive
MTF1	HERC2P9	0.393981041	5.15E-20	postive
MTF1	HEXD-IT1	0.313731272	6.98E-13	postive
MTF1	HIPK1-AS1	0.300780451	6.49E-12	postive
MTF1	HM13-IT1	0.378147854	1.92E-18	postive
MTF1	HMGB1P5	0.408886773	1.42E-21	postive
MTF1	HNRNPA1P6	0.326985247	6.36E-14	postive
MTF1	HSP90AA4P	0.302263857	5.05E-12	postive
MTF1	HSP90AA6P	0.32321334	1.27E-13	postive
MTF1	HSP90AB2P	0.369123807	1.38E-17	postive
MTF1	HUNK-AS1	0.302423531	4.92E-12	postive
MTF1	HYI-AS1	0.392230314	7.76E-20	postive
MTF1	IL10RB-DT	0.461885825	8.64E-28	postive
MTF1	ILF3-DT	0.382196716	7.75E-19	postive
MTF1	INE1	0.366276065	2.55E-17	postive
MTF1	INTS4P2	0.305246287	3.05E-12	postive
MTF1	INTS6-AS1	0.314100515	6.54E-13	postive
MTF1	IPO5P1	0.380310157	1.18E-18	postive
MTF1	IQCH-AS1	0.397809549	2.09E-20	postive
MTF1	IRF1-AS1	0.361176258	7.49E-17	postive
MTF1	ITGA9-AS1	0.333595035	1.84E-14	postive
MTF1	JPX	0.329681755	3.85E-14	postive
MTF1	KCNJ2-AS1	0.31304522	7.88E-13	postive
MTF1	KDM4A-AS1	0.427777277	1.15E-23	postive
MTF1	KDM7A-DT	0.415584486	2.67E-22	postive
MTF1	KIAA1671-AS1	0.376557627	2.73E-18	postive
MTF1	KIF9-AS1	0.404889778	3.79E-21	postive
MTF1	KRT8P12	0.428845055	8.71E-24	postive
MTF1	KRTAP5-AS1	0.310961817	1.14E-12	postive
MTF1	L3MBTL2-AS1	0.442324174	2.29E-25	postive
MTF1	LAMC1-AS1	0.306768812	2.35E-12	postive
MTF1	LAMTOR5-AS1	0.36796822	1.77E-17	postive
MTF1	LENG8-AS1	0.359809863	9.96E-17	postive
MTF1	LIFR-AS1	0.385931094	3.32E-19	postive
MTF1	LINC00111	0.30806571	1.88E-12	postive
MTF1	LINC00174	0.306426507	2.49E-12	postive
MTF1	LINC00205	0.463945024	4.70E-28	postive
MTF1	LINC00235	0.325026618	9.13E-14	postive
MTF1	LINC00243	0.307335466	2.13E-12	postive
MTF1	LINC00265	0.391681621	8.82E-20	postive
MTF1	LINC00294	0.408737593	1.48E-21	postive
MTF1	LINC00339	0.422695416	4.34E-23	postive
MTF1	LINC00449	0.365169457	3.22E-17	postive
MTF1	LINC00471	0.363395491	4.69E-17	postive
MTF1	LINC00476	0.339846865	5.54E-15	postive
MTF1	LINC00511	0.333296783	1.95E-14	postive
MTF1	LINC00562	0.306576087	2.43E-12	postive
MTF1	LINC00571	0.312482391	8.70E-13	postive
MTF1	LINC00630	0.468451298	1.22E-28	postive

MTF1	LINC00638	0.35732744	1.67E-16	postive
MTF1	LINC00641	0.408390684	1.61E-21	postive
MTF1	LINC00649	0.315559193	5.05E-13	postive
MTF1	LINC00654	0.44126758	3.06E-25	postive
MTF1	LINC00663	0.393949394	5.19E-20	postive
MTF1	LINC00667	0.438523468	6.49E-25	postive
MTF1	LINC00674	0.438067303	7.35E-25	postive
MTF1	LINC00680	0.32794802	5.32E-14	postive
MTF1	LINC00847	0.304668961	3.36E-12	postive
MTF1	LINC00852	0.358933949	1.20E-16	postive
MTF1	LINC00863	0.502214172	2.63E-33	postive
MTF1	LINC00865	0.323466137	1.22E-13	postive
MTF1	LINC00877	0.396714568	2.70E-20	postive
MTF1	LINC00888	0.328045585	5.22E-14	postive
MTF1	LINC00899	0.35954225	1.05E-16	postive
MTF1	LINC00900	0.314391833	6.22E-13	postive
MTF1	LINC00909	0.495886335	2.16E-32	postive
MTF1	LINC00910	0.301419872	5.83E-12	postive
MTF1	LINC00921	0.381195978	9.71E-19	postive
MTF1	LINC00933	0.330851641	3.09E-14	postive
MTF1	LINC00950	0.347240479	1.29E-15	postive
MTF1	LINC00957	0.3632102	4.88E-17	postive
MTF1	LINC00963	0.50090776	4.07E-33	postive
MTF1	LINC00987	0.428128464	1.05E-23	postive
MTF1	LINC00997	0.333269824	1.96E-14	postive
MTF1	LINC01001	0.378787726	1.66E-18	postive
MTF1	LINC01006	0.341065437	4.37E-15	postive
MTF1	LINC01011	0.365169879	3.22E-17	postive
MTF1	LINC01094	0.306437835	2.48E-12	postive
MTF1	LINC01127	0.34532959	1.89E-15	postive
MTF1	LINC01128	0.646956354	1.27E-60	postive
MTF1	LINC01132	0.329919242	3.68E-14	postive
MTF1	LINC01135	0.375072881	3.79E-18	postive
MTF1	LINC01144	0.354028734	3.29E-16	postive
MTF1	LINC01145	0.387628315	2.25E-19	postive
MTF1	LINC01184	0.405948757	2.93E-21	postive
MTF1	LINC01237	0.310877423	1.15E-12	postive
MTF1	LINC01271	0.372472528	6.69E-18	postive
MTF1	LINC01277	0.313432682	7.36E-13	postive
MTF1	LINC01278	0.513652774	5.20E-35	postive
MTF1	LINC01283	0.302924822	4.52E-12	postive
MTF1	LINC01291	0.305655219	2.84E-12	postive
MTF1	LINC01347	0.407450264	2.03E-21	postive
MTF1	LINC01372	0.300664554	6.62E-12	postive
MTF1	LINC01503	0.373271009	5.62E-18	postive
MTF1	LINC01521	0.440736132	3.54E-25	postive
MTF1	LINC01534	0.434273415	2.05E-24	postive
MTF1	LINC01560	0.427377429	1.28E-23	postive
MTF1	LINC01562	0.323224224	1.27E-13	postive
MTF1	LINC01678	0.328793762	4.54E-14	postive

MTF1	LINC01679	0.357878262	1.49E-16	postive
MTF1	LINC01684	0.30927257	1.52E-12	postive
MTF1	LINC01719	0.308614259	1.71E-12	postive
MTF1	LINC01771	0.303525793	4.08E-12	postive
MTF1	LINC01772	0.38806835	2.04E-19	postive
MTF1	LINC01775	0.342848909	3.08E-15	postive
MTF1	LINC01780	0.334158302	1.65E-14	postive
MTF1	LINC01852	0.389596404	1.43E-19	postive
MTF1	LINC01881	0.602318762	1.09E-50	postive
MTF1	LINC01963	0.555056243	9.54E-42	postive
MTF1	LINC02035	0.562415113	4.80E-43	postive
MTF1	LINC02175	0.301715245	5.54E-12	postive
MTF1	LINC02210	0.486843938	4.07E-31	postive
MTF1	LINC02256	0.379177915	1.53E-18	postive
MTF1	LINC02408	0.335154219	1.37E-14	postive
MTF1	LINC02449	0.348152743	1.08E-15	postive
MTF1	LINC02611	0.360938653	7.87E-17	postive
MTF1	LINC02637	0.329601157	3.91E-14	postive
MTF1	LINC02693	0.543105596	1.05E-39	postive
MTF1	LINC02705	0.377079001	2.43E-18	postive
MTF1	LINC02724	0.370368676	1.06E-17	postive
MTF1	LINC02773	0.309895652	1.37E-12	postive
MTF1	LINC02803	0.330234051	3.47E-14	postive
MTF1	LINC02818	0.400180032	1.18E-20	postive
MTF1	LNCTAM34A	0.308448157	1.76E-12	postive
MTF1	LOH12CR2	0.305734828	2.80E-12	postive
MTF1	LOXL1-AS1	0.303467741	4.12E-12	postive
MTF1	LPP-AS2	0.421557739	5.83E-23	postive
MTF1	LRRC37A16P	0.472912418	3.16E-29	postive
MTF1	LRRC37A17P	0.66155564	3.03E-64	postive
MTF1	LRRC37A4P	0.391506911	9.19E-20	postive
MTF1	LRRC37A6P	0.34948489	8.25E-16	postive
MTF1	LRRC37BP1	0.547257417	2.09E-40	postive
MTF1	LRRC8C-DT	0.364799203	3.49E-17	postive
MTF1	MAFG-DT	0.301998001	5.29E-12	postive
MTF1	MAGI2-AS3	0.310236161	1.29E-12	postive
MTF1	MAN1B1-DT	0.308290446	1.81E-12	postive
MTF1	MAP2K4P1	0.478171504	6.26E-30	postive
MTF1	MAP3K14-AS1	0.357147633	1.73E-16	postive
MTF1	MAP4K3-DT	0.338389306	7.35E-15	postive
MTF1	MAPKAPK5-AS1	0.318548878	2.96E-13	postive
MTF1	MATN1-AS1	0.31382921	6.86E-13	postive
MTF1	MCM3AP-AS1	0.45555682	5.47E-27	postive
MTF1	MCPH1-AS1	0.357363287	1.66E-16	postive
MTF1	MED14OS	0.360875767	7.97E-17	postive
MTF1	MED8-AS1	0.399478776	1.40E-20	postive
MTF1	MFF-DT	0.375013717	3.84E-18	postive
MTF1	MIATNB	0.323351101	1.24E-13	postive
MTF1	MID1IP1-AS1	0.419122124	1.09E-22	postive
MTF1	MIF-AS1	0.369656973	1.23E-17	postive

MTF1	MIPEPP3	0.367338309	2.03E-17	postive
MTF1	MIR22HG	0.377732855	2.10E-18	postive
MTF1	MIR34AHG	0.377775188	2.08E-18	postive
MTF1	MIR4453HG	0.371636753	8.03E-18	postive
MTF1	MIR600HG	0.380195123	1.22E-18	postive
MTF1	MIR762HG	0.315061244	5.52E-13	postive
MTF1	MKLN1-AS	0.379976757	1.28E-18	postive
MTF1	MKNK1-AS1	0.471824987	4.41E-29	postive
MTF1	MMP25-AS1	0.300614381	6.67E-12	postive
MTF1	MORC2-AS1	0.313980872	6.68E-13	postive
MTF1	MRPL20-AS1	0.580621626	2.11E-46	postive
MTF1	MRPS31P4	0.501640892	3.19E-33	postive
MTF1	MRPS31P5	0.441736351	2.69E-25	postive
MTF1	MSL3P1	0.393519425	5.74E-20	postive
MTF1	MTMR9LP	0.310659519	1.20E-12	postive
MTF1	MUC20-OT1	0.441312613	3.02E-25	postive
MTF1	MZF1-AS1	0.418011541	1.44E-22	postive
MTF1	NAIPP3	0.406911523	2.31E-21	postive
MTF1	NARF-AS1	0.303489378	4.11E-12	postive
MTF1	NARF-IT1	0.300089179	7.29E-12	postive
MTF1	NBPF25P	0.581609071	1.37E-46	postive
MTF1	NBPF8	0.429118495	8.10E-24	postive
MTF1	NBR2	0.461793744	8.88E-28	postive
MTF1	NCBP2-AS1	0.353035236	4.02E-16	postive
MTF1	NCF1B	0.310469028	1.24E-12	postive
MTF1	NCK1-DT	0.395885339	3.29E-20	postive
MTF1	NDUFA6-DT	0.387434338	2.36E-19	postive
MTF1	NDUFB2-AS1	0.446556427	7.06E-26	postive
MTF1	NDUFV2-AS1	0.318231675	3.14E-13	postive
MTF1	NFYC-AS1	0.378415107	1.81E-18	postive
MTF1	NIFK-AS1	0.460213643	1.41E-27	postive
MTF1	NINJ2-AS1	0.375246518	3.65E-18	postive
MTF1	NKAPP1	0.397077971	2.48E-20	postive
MTF1	NNT-AS1	0.367480077	1.97E-17	postive
MTF1	NOP14-AS1	0.547518374	1.89E-40	postive
MTF1	NOP53-AS1	0.435912736	1.32E-24	postive
MTF1	NORAD	0.633751976	1.63E-57	postive
MTF1	NPHP3-AS1	0.368731127	1.51E-17	postive
MTF1	NRAV	0.449430189	3.14E-26	postive
MTF1	NRSN2-AS1	0.304161982	3.66E-12	postive
MTF1	NUDT16P1	0.422492638	4.58E-23	postive
MTF1	NUTM2A-AS1	0.507676078	4.11E-34	postive
MTF1	NUTM2B-AS1	0.32443201	1.02E-13	postive
MTF1	NXT1-AS1	0.300896702	6.36E-12	postive
MTF1	ODF2-AS1	0.320196435	2.20E-13	postive
MTF1	OGFR-AS1	0.358765086	1.24E-16	postive
MTF1	OGFRP1	0.424496258	2.72E-23	postive
MTF1	OIP5-AS1	0.572709669	6.44E-45	postive
MTF1	OR2A1-AS1	0.372911994	6.08E-18	postive
MTF1	OTUD6B-AS1	0.442303695	2.30E-25	postive

MTF1	PAN3-AS1	0.348232083	1.06E-15	postive
MTF1	PARGP1	0.455251023	5.97E-27	postive
MTF1	PAXBP1-AS1	0.408586323	1.53E-21	postive
MTF1	PAXIP1-AS2	0.530335837	1.30E-37	postive
MTF1	PCBP1-AS1	0.473129045	2.96E-29	postive
MTF1	PDCD4-AS1	0.301371496	5.87E-12	postive
MTF1	PDIA3P1	0.419003338	1.12E-22	postive
MTF1	PDPK2P	0.393494046	5.78E-20	postive
MTF1	PEF1-AS1	0.335749444	1.22E-14	postive
MTF1	PGAM1P8	0.384966667	4.14E-19	postive
MTF1	PI4KAP2	0.305963509	2.69E-12	postive
MTF1	PICART1	0.415220828	2.93E-22	postive
MTF1	PIK3IP1-AS1	0.363005377	5.10E-17	postive
MTF1	PINK1-AS	0.610947646	1.74E-52	postive
MTF1	PIPSL	0.446892981	6.42E-26	postive
MTF1	PITRM1-AS1	0.372574652	6.55E-18	postive
MTF1	PKD1P6	0.497288818	1.36E-32	postive
MTF1	PKP4-AS1	0.312118453	9.27E-13	postive
MTF1	PLBD1-AS1	0.380851934	1.05E-18	postive
MTF1	PLEKHA8P1	0.536359393	1.37E-38	postive
MTF1	PLEKHM1P1	0.345850662	1.71E-15	postive
MTF1	PMS2CL	0.555795964	7.09E-42	postive
MTF1	PMS2P3	0.479012212	4.82E-30	postive
MTF1	PMS2P4	0.474054224	2.23E-29	postive
MTF1	POLH-AS1	0.346693403	1.44E-15	postive
MTF1	POLR2J4	0.449541411	3.05E-26	postive
MTF1	POM121L9P	0.300305757	7.03E-12	postive
MTF1	PPIEL	0.321811419	1.64E-13	postive
MTF1	PPP1R12A-AS1	0.350989019	6.09E-16	postive
MTF1	PPP1R26-AS1	0.420485763	7.68E-23	postive
MTF1	PPP3CB-AS1	0.392511671	7.27E-20	postive
MTF1	PPP4R1L	0.303338461	4.21E-12	postive
MTF1	PPT2-EGFL8	0.455078616	6.27E-27	postive
MTF1	PRKAR2A-AS1	0.39098094	1.04E-19	postive
MTF1	PRKCZ-AS1	0.311886193	9.66E-13	postive
MTF1	PRR34	0.407289601	2.11E-21	postive
MTF1	PRR7-AS1	0.31551921	5.09E-13	postive
MTF1	PSMA3-AS1	0.363074969	5.02E-17	postive
MTF1	PTENP1	0.617353435	7.42E-54	postive
MTF1	PTMAP5	0.339936895	5.45E-15	postive
MTF1	PTOV1-AS1	0.604954554	3.13E-51	postive
MTF1	PTOV1-AS2	0.331654097	2.66E-14	postive
MTF1	PTPRD-AS1	0.300248973	7.09E-12	postive
MTF1	PTPRG-AS1	0.321420937	1.76E-13	postive
MTF1	RAB30-DT	0.310595907	1.21E-12	postive
MTF1	RAD51-AS1	0.33269096	2.18E-14	postive
MTF1	RALY-AS1	0.307753885	1.98E-12	postive
MTF1	RAP2C-AS1	0.460622674	1.25E-27	postive
MTF1	RARA-AS1	0.350414427	6.84E-16	postive
MTF1	RASAL2-AS1	0.465907831	2.62E-28	postive

MTF1	RBM15-AS1	0.316219932	4.49E-13	postive
MTF1	RBM26-AS1	0.315314173	5.28E-13	postive
MTF1	RBM5-AS1	0.382878908	6.65E-19	postive
MTF1	RFPL3S	0.337817435	8.21E-15	postive
MTF1	RHOA-IT1	0.302981943	4.48E-12	postive
MTF1	RHPN1-AS1	0.356940187	1.81E-16	postive
MTF1	RNASEH2B-AS1	0.304374317	3.53E-12	postive
MTF1	RNF157-AS1	0.308215781	1.83E-12	postive
MTF1	RNF213-AS1	0.326572764	6.86E-14	postive
MTF1	RNF216P1	0.479762615	3.81E-30	postive
MTF1	ROCK1P1	0.349000394	9.10E-16	postive
MTF1	RP11-101E13.5	0.348118238	1.09E-15	postive
MTF1	RP11-10C24.1	0.481785622	2.02E-30	postive
MTF1	RP11-159G9.5	0.588439063	6.57E-48	postive
MTF1	RP11-15H20.6	0.329504212	3.98E-14	postive
MTF1	RP11-15N24.4	0.439595426	4.84E-25	postive
MTF1	RP11-164P12.4	0.573107684	5.44E-45	postive
MTF1	RP11-165J3.6	0.306322263	2.53E-12	postive
MTF1	RP11-16P6.1	0.371198531	8.83E-18	postive
MTF1	RP11-175O19.4	0.60198972	1.28E-50	postive
MTF1	RP11-206L10.3	0.452415769	1.35E-26	postive
MTF1	RP11-206L10.5	0.444748017	1.17E-25	postive
MTF1	RP11-211G3.2	0.335410639	1.30E-14	postive
MTF1	RP11-226L15.5	0.393759029	5.43E-20	postive
MTF1	RP1-122P22.2	0.597104998	1.25E-49	postive
MTF1	RP11-22P4.1	0.30113905	6.11E-12	postive
MTF1	RP11-282O18.3	0.635388173	6.86E-58	postive
MTF1	RP11-327F22.1	0.306776873	2.34E-12	postive
MTF1	RP11-337C18.9	0.377585428	2.17E-18	postive
MTF1	RP11-346C20.4	0.326486872	6.97E-14	postive
MTF1	RP11-435O5.2	0.368758887	1.50E-17	postive
MTF1	RP11-439L18.2	0.50887401	2.73E-34	postive
MTF1	RP11-492E3.51	0.329412719	4.05E-14	postive
MTF1	RP11-524D16__A.3	0.32131076	1.80E-13	postive
MTF1	RP11-529E10.6	0.363928033	4.19E-17	postive
MTF1	RP11-539I5.1	0.436241181	1.21E-24	postive
MTF1	RP11-540B6.6	0.493122737	5.35E-32	postive
MTF1	RP11-58E21.3	0.390170607	1.25E-19	postive
MTF1	RP11-658F2.8	0.395955897	3.24E-20	postive
MTF1	RP11-680F20.6	0.314172414	6.46E-13	postive
MTF1	RP11-69E11.4	0.325148725	8.93E-14	postive
MTF1	RP11-706O15.1	0.388370443	1.90E-19	postive
MTF1	RP11-815I9.4	0.366675762	2.34E-17	postive
MTF1	RP11-86H7.7	0.456882609	3.73E-27	postive
MTF1	RP11-876N24.4	0.404918448	3.77E-21	postive
MTF1	RP11-876N24.5	0.453318724	1.04E-26	postive
MTF1	RP13-36G14.4	0.406681155	2.45E-21	postive
MTF1	RP1-93H18.1	0.437157054	9.42E-25	postive
MTF1	RP3-355L5.5	0.382221266	7.71E-19	postive
MTF1	RP3-368A4.5	0.309693658	1.42E-12	postive

MTF1	RP4-714D9.5	0.332616468	2.22E-14	postive
MTF1	RP5-1039K5.19	0.441515259	2.86E-25	postive
MTF1	RP5-1042I8.7	0.499398822	6.74E-33	postive
MTF1	RP5-1112D6.4	0.317446086	3.61E-13	postive
MTF1	RP5-1125A11.6	0.474030488	2.25E-29	postive
MTF1	RP5-1125A11.7	0.390170223	1.25E-19	postive
MTF1	RP5-1139B12.2	0.368148073	1.71E-17	postive
MTF1	RPL13P5	0.440230702	4.07E-25	postive
MTF1	RPL23AP53	0.46602164	2.53E-28	postive
MTF1	RPL23AP7	0.427932597	1.11E-23	postive
MTF1	RPL23AP82	0.440450151	3.83E-25	postive
MTF1	RPL32P3	0.466597928	2.13E-28	postive
MTF1	RPL39P5	0.312283718	9.01E-13	postive
MTF1	RPS10P7	0.422675652	4.37E-23	postive
MTF1	RPS18P9	0.345683273	1.76E-15	postive
MTF1	RPS4XP16	0.379702306	1.36E-18	postive
MTF1	RPS6KA2-IT1	0.326409972	7.07E-14	postive
MTF1	RRN3P3	0.528925326	2.18E-37	postive
MTF1	RRP7BP	0.451887055	1.57E-26	postive
MTF1	RTCA-AS1	0.421811294	5.46E-23	postive
MTF1	RXYLT1-AS1	0.358329841	1.36E-16	postive
MTF1	SAMD12-AS1	0.335507455	1.28E-14	postive
MTF1	SAP30-DT	0.30989764	1.37E-12	postive
MTF1	SBDSP1	0.535844638	1.67E-38	postive
MTF1	SBF2-AS1	0.330056158	3.59E-14	postive
MTF1	SCAND2P	0.557531746	3.52E-42	postive
MTF1	SDAD1P1	0.385738495	3.47E-19	postive
MTF1	SDCBP2-AS1	0.391747675	8.69E-20	postive
MTF1	SDHAP1	0.489715787	1.62E-31	postive
MTF1	SEC22B4P	0.315501365	5.11E-13	postive
MTF1	SEPSECS-AS1	0.312088257	9.32E-13	postive
MTF1	SEPTIN7-AS1	0.372445215	6.73E-18	postive
MTF1	SEPTIN7P2	0.445695608	8.98E-26	postive
MTF1	SERHL	0.431781056	4.00E-24	postive
MTF1	SGMS1-AS1	0.343392864	2.77E-15	postive
MTF1	SGOL1-AS1	0.32862984	4.68E-14	postive
MTF1	SH3BP5-AS1	0.313728091	6.99E-13	postive
MTF1	SH3GL1P1	0.405122554	3.59E-21	postive
MTF1	SH3RF3-AS1	0.36597942	2.71E-17	postive
MTF1	SHLD2P1	0.354297275	3.11E-16	postive
MTF1	SIGLEC22P	0.424912406	2.44E-23	postive
MTF1	SLC16A1-AS1	0.30367591	3.98E-12	postive
MTF1	SLC25A5-AS1	0.333243112	1.97E-14	postive
MTF1	SLC44A3-AS1	0.367061574	2.15E-17	postive
MTF1	SLC9A7P1	0.341255008	4.21E-15	postive
MTF1	SLFNL1-AS1	0.326135542	7.44E-14	postive
MTF1	SLX1A-SULT1A3	0.32944886	4.02E-14	postive
MTF1	SMG7-AS1	0.509799176	1.98E-34	postive
MTF1	SMIM15-AS1	0.3682834	1.66E-17	postive
MTF1	SNAI3-AS1	0.364696297	3.56E-17	postive

MTF1	SNHG11	0.358459161	1.32E-16	postive
MTF1	SNHG12	0.336743533	1.01E-14	postive
MTF1	SNHG16	0.414916235	3.16E-22	postive
MTF1	SNHG20	0.385227977	3.90E-19	postive
MTF1	SNHG28	0.344831368	2.09E-15	postive
MTF1	SNHG31	0.424388748	2.80E-23	postive
MTF1	SOS1-IT1	0.347444259	1.24E-15	postive
MTF1	SP2-AS1	0.393562076	5.69E-20	postive
MTF1	SPAG5-AS1	0.425387776	2.16E-23	postive
MTF1	SPDYE7P	0.315290565	5.30E-13	postive
MTF1	SREBF2-AS1	0.521988701	2.71E-36	postive
MTF1	SSBP3-AS1	0.434536191	1.91E-24	postive
MTF1	SSR4P1	0.344617092	2.18E-15	postive
MTF1	ST20-AS1	0.475318304	1.51E-29	postive
MTF1	STAG3L1	0.396829286	2.63E-20	postive
MTF1	STAG3L2	0.483093807	1.34E-30	postive
MTF1	STAG3L3	0.429157751	8.02E-24	postive
MTF1	STAG3L5P	0.399692456	1.33E-20	postive
MTF1	STAG3L5P-PVRIG2P-PILRB	0.361717309	6.68E-17	postive
MTF1	STARD4-AS1	0.361503167	6.99E-17	postive
MTF1	STARD7-AS1	0.620100745	1.87E-54	postive
MTF1	STRA6LP	0.33167602	2.65E-14	postive
MTF1	STX18-AS1	0.46025346	1.40E-27	postive
MTF1	STXBP5-AS1	0.341082724	4.36E-15	postive
MTF1	SUCLG2-AS1	0.419236324	1.06E-22	postive
MTF1	SUGT1P4-STRA6LP	0.367307704	2.04E-17	postive
MTF1	SUMO2P17	0.37728817	2.32E-18	postive
MTF1	SUZ12P1	0.362512761	5.65E-17	postive
MTF1	SVIL2P	0.492461288	6.63E-32	postive
MTF1	SVIL-AS1	0.54780877	1.69E-40	postive
MTF1	SYS1-DBNDD2	0.350441014	6.81E-16	postive
MTF1	TBC1D8-AS1	0.324901038	9.34E-14	postive
MTF1	TBX5-AS1	0.36857956	1.56E-17	postive
MTF1	TCAF2P1	0.321928481	1.61E-13	postive
MTF1	TGFB2-AS1	0.304267541	3.60E-12	postive
MTF1	THAP9-AS1	0.475075695	1.63E-29	postive
MTF1	TIMM23B-AGAP6	0.375026218	3.83E-18	postive
MTF1	TMEM147-AS1	0.302848756	4.58E-12	postive
MTF1	TMEM161B-AS1	0.301461194	5.79E-12	postive
MTF1	TMEM198B	0.363749052	4.36E-17	postive
MTF1	TMEM202-AS1	0.376194096	2.96E-18	postive
MTF1	TMEM51-AS1	0.355865432	2.25E-16	postive
MTF1	TMEM9B-AS1	0.398093509	1.95E-20	postive
MTF1	TNFRSF10A-AS1	0.309750937	1.40E-12	postive
MTF1	TNK2-AS1	0.365942196	2.74E-17	postive
MTF1	TNRC6C-AS1	0.409352981	1.27E-21	postive
MTF1	TOB1-AS1	0.320432818	2.11E-13	postive
MTF1	TONSL-AS1	0.374094291	4.70E-18	postive
MTF1	TP73-AS1	0.56706446	6.98E-44	postive
MTF1	TPI1P2	0.390613715	1.13E-19	postive

MTF1	TPM3P9	0.362915976	5.19E-17	postive
MTF1	TPT1-AS1	0.352420854	4.56E-16	postive
MTF1	TPTEP2	0.370143902	1.11E-17	postive
MTF1	TRAF3IP2-AS1	0.411556202	7.34E-22	postive
MTF1	TRAM2-AS1	0.342054099	3.60E-15	postive
MTF1	TRAPPC12-AS1	0.466886701	1.96E-28	postive
MTF1	TSC22D1-AS1	0.318965267	2.75E-13	postive
MTF1	TSPOAP1-AS1	0.364827094	3.47E-17	postive
MTF1	TSPY26P	0.392952494	6.56E-20	postive
MTF1	TSTD3	0.336224296	1.11E-14	postive
MTF1	TTC28-AS1	0.479813525	3.75E-30	postive
MTF1	TTC41P	0.379601982	1.39E-18	postive
MTF1	TTN-AS1	0.420793519	7.09E-23	postive
MTF1	TUBBP1	0.368037906	1.75E-17	postive
MTF1	TUBBP5	0.316828745	4.03E-13	postive
MTF1	TUG1	0.632907472	2.55E-57	postive
MTF1	U47924.2	0.313019685	7.92E-13	postive
MTF1	U73169.1	0.317712313	3.44E-13	postive
MTF1	UBA6-AS1	0.460487321	1.30E-27	postive
MTF1	UBE2D3-AS1	0.349388576	8.41E-16	postive
MTF1	UBE2Q1-AS1	0.312350119	8.90E-13	postive
MTF1	UBE2Q2P2	0.349765072	7.80E-16	postive
MTF1	UGDH-AS1	0.367510836	1.96E-17	postive
MTF1	UPF3AP2	0.341838475	3.76E-15	postive
MTF1	USP27X-AS1	0.447937036	4.79E-26	postive
MTF1	USP46-AS1	0.454210792	8.05E-27	postive
MTF1	UXT-AS1	0.333703904	1.80E-14	postive
MTF1	VASH1-AS1	0.346932997	1.38E-15	postive
MTF1	VDAC1P8	0.459229442	1.88E-27	postive
MTF1	WAC-AS1	0.471179211	5.36E-29	postive
MTF1	WAKMAR2	0.31153844	1.03E-12	postive
MTF1	WARS2-AS1	0.513694304	5.13E-35	postive
MTF1	WASH3P	0.37916843	1.53E-18	postive
MTF1	WEE2-AS1	0.308390966	1.77E-12	postive
MTF1	WHAMMP2	0.333879842	1.74E-14	postive
MTF1	WHAMMP3	0.376449698	2.80E-18	postive
MTF1	WNT5A-AS1	0.456899703	3.71E-27	postive
MTF1	YEATS2-AS1	0.34995726	7.50E-16	postive
MTF1	Z68871.1	0.539473277	4.22E-39	postive
MTF1	Z83851.4	0.356672377	1.91E-16	postive
MTF1	Z92544.2	0.458934282	2.05E-27	postive
MTF1	Z94721.1	0.328268098	5.01E-14	postive
MTF1	Z95114.1	0.314826545	5.76E-13	postive
MTF1	Z95115.1	0.360440632	8.73E-17	postive
MTF1	Z97634.1	0.333673642	1.81E-14	postive
MTF1	Z97989.1	0.353581951	3.60E-16	postive
MTF1	Z98884.2	0.482288878	1.73E-30	postive
MTF1	Z99572.1	0.454505876	7.40E-27	postive
MTF1	ZBED5-AS1	0.306404496	2.50E-12	postive
MTF1	ZBTB11-AS1	0.470034011	7.59E-29	postive

MTF1	ZBTB40-IT1	0.385455604	3.70E-19	postive
MTF1	ZDHHC20-IT1	0.356678075	1.91E-16	postive
MTF1	ZKSCAN7-AS1	0.432260493	3.52E-24	postive
MTF1	ZNF137P	0.580462234	2.26E-46	postive
MTF1	ZNF205-AS1	0.383601728	5.64E-19	postive
MTF1	ZNF213-AS1	0.314872966	5.71E-13	postive
MTF1	ZNF252P	0.554536159	1.18E-41	postive
MTF1	ZNF271P	0.488884343	2.11E-31	postive
MTF1	ZNF286B	0.384152825	4.98E-19	postive
MTF1	ZNF32-AS2	0.347562368	1.21E-15	postive
MTF1	ZNF337-AS1	0.323774131	1.15E-13	postive
MTF1	ZNF341-AS1	0.317181769	3.79E-13	postive
MTF1	ZNF37BP	0.394627755	4.43E-20	postive
MTF1	ZNF436-AS1	0.375633699	3.35E-18	postive
MTF1	ZNF503-AS2	0.366191879	2.59E-17	postive
MTF1	ZNF528-AS1	0.336891547	9.81E-15	postive
MTF1	ZNF529-AS1	0.345793652	1.72E-15	postive
MTF1	ZNF542P	0.422046627	5.14E-23	postive
MTF1	ZNF56	0.381838259	8.41E-19	postive
MTF1	ZNF561-AS1	0.330469158	3.32E-14	postive
MTF1	ZNF582-AS1	0.306520504	2.45E-12	postive
MTF1	ZNF649-AS1	0.343788899	2.56E-15	postive
MTF1	ZNF658B	0.396819087	2.64E-20	postive
MTF1	ZNF667-AS1	0.351303568	5.72E-16	postive
MTF1	ZNF674-AS1	0.314461799	6.14E-13	postive
MTF1	ZNF687-AS1	0.327788901	5.48E-14	postive
MTF1	ZNF767P	0.452325845	1.38E-26	postive
MTF1	ZNF788P	0.331379897	2.80E-14	postive
MTF1	ZNF790-AS1	0.348961571	9.17E-16	postive
MTF1	ZNF815P	0.386206213	3.12E-19	postive
MTF1	ZNNT1	0.358399589	1.34E-16	postive
MTF1	ZNRD1ASP	0.428007539	1.09E-23	postive
MTF1	ZNRD2-AS1	0.433859673	2.29E-24	postive
MTF1	ZNRF2P1	0.382371476	7.45E-19	postive
MTF1	ZNRF2P2	0.462241539	7.78E-28	postive
MTF1	ZSCAN12P1	0.434146626	2.12E-24	postive
PAM	AC000403.1	0.376428931	2.81E-18	postive
PAM	AC004943.2	0.306643729	2.40E-12	postive
PAM	AC005034.6	0.336423248	1.07E-14	postive
PAM	AC005288.1	0.329622004	3.89E-14	postive
PAM	AC005520.4	0.332541124	2.25E-14	postive
PAM	AC005562.1	0.335300382	1.33E-14	postive
PAM	AC005670.3	0.350105752	7.28E-16	postive
PAM	AC006126.4	0.353692387	3.52E-16	postive
PAM	AC006449.2	0.335605877	1.25E-14	postive
PAM	AC007383.2	0.305188366	3.08E-12	postive
PAM	AC008014.1	0.305207015	3.07E-12	postive
PAM	AC008393.1	0.37751776	2.21E-18	postive
PAM	AC008438.1	0.305495659	2.92E-12	postive
PAM	AC008443.1	0.350773645	6.36E-16	postive

PAM	AC008494.3	0.376870447	2.55E-18	postive
PAM	AC008496.2	0.337935632	8.02E-15	postive
PAM	AC008771.1	0.428534778	9.45E-24	postive
PAM	AC008966.1	0.394503676	4.56E-20	postive
PAM	AC009041.3	0.303739156	3.94E-12	postive
PAM	AC009126.1	0.370568536	1.01E-17	postive
PAM	AC009237.14	0.305382489	2.98E-12	postive
PAM	AC009831.1	0.354891136	2.75E-16	postive
PAM	AC010226.1	0.388650341	1.78E-19	postive
PAM	AC010245.2	0.304573465	3.42E-12	postive
PAM	AC010273.2	0.3053809	2.98E-12	postive
PAM	AC010273.3	0.419794973	9.16E-23	postive
PAM	AC010969.2	0.327877818	5.39E-14	postive
PAM	AC012063.1	0.303450137	4.13E-12	postive
PAM	AC012313.5	0.312692748	8.38E-13	postive
PAM	AC012603.1	0.351267651	5.76E-16	postive
PAM	AC015922.2	0.377763438	2.09E-18	postive
PAM	AC015922.3	0.326708666	6.69E-14	postive
PAM	AC016747.1	0.336079984	1.15E-14	postive
PAM	AC018521.5	0.362296082	5.92E-17	postive
PAM	AC018647.2	0.335691235	1.23E-14	postive
PAM	AC018809.2	0.331514492	2.73E-14	postive
PAM	AC020915.3	0.321215616	1.83E-13	postive
PAM	AC021016.2	0.329008667	4.36E-14	postive
PAM	AC026904.2	0.357800726	1.51E-16	postive
PAM	AC027307.3	0.35412201	3.22E-16	postive
PAM	AC044860.1	0.329950295	3.66E-14	postive
PAM	AC060766.7	0.318710195	2.88E-13	postive
PAM	AC067852.2	0.385699371	3.50E-19	postive
PAM	AC068580.1	0.330482569	3.31E-14	postive
PAM	AC068580.3	0.438308599	6.88E-25	postive
PAM	AC080112.2	0.31268879	8.39E-13	postive
PAM	AC083964.1	0.341306497	4.17E-15	postive
PAM	AC087645.2	0.398728887	1.68E-20	postive
PAM	AC090197.1	0.336951487	9.70E-15	postive
PAM	AC090559.1	0.323820567	1.14E-13	postive
PAM	AC092354.2	0.344931228	2.05E-15	postive
PAM	AC092368.3	0.361809579	6.56E-17	postive
PAM	AC092376.2	0.361750998	6.64E-17	postive
PAM	AC092614.1	0.343376531	2.78E-15	postive
PAM	AC093010.3	0.343140595	2.91E-15	postive
PAM	AC093278.2	0.326270109	7.26E-14	postive
PAM	AC093726.1	0.342769689	3.13E-15	postive
PAM	AC093752.1	0.36946621	1.28E-17	postive
PAM	AC096677.1	0.370137145	1.11E-17	postive
PAM	AC096772.6	0.349879232	7.62E-16	postive
PAM	AC098484.1	0.40048909	1.10E-20	postive
PAM	AC099548.2	0.343629364	2.65E-15	postive
PAM	AC100810.1	0.352425393	4.56E-16	postive
PAM	AC100849.1	0.300200839	7.15E-12	postive

PAM	AC100861.1	0.326788552	6.59E-14	postive
PAM	AC104024.2	0.379728344	1.35E-18	postive
PAM	AC104083.1	0.392194802	7.83E-20	postive
PAM	AC104109.2	0.379948312	1.28E-18	postive
PAM	AC104113.1	0.356419184	2.01E-16	postive
PAM	AC105942.1	0.382283231	7.60E-19	postive
PAM	AC106739.1	0.376165843	2.98E-18	postive
PAM	AC106786.2	0.445610619	9.19E-26	postive
PAM	AC106881.1	0.356460385	1.99E-16	postive
PAM	AC107959.1	0.319985672	2.29E-13	postive
PAM	AC109454.2	0.347042136	1.35E-15	postive
PAM	AC110995.1	0.312336866	8.93E-13	postive
PAM	AC112206.2	0.331990396	2.49E-14	postive
PAM	AC115618.2	0.417537585	1.63E-22	postive
PAM	AC124242.1	0.319573302	2.46E-13	postive
PAM	AC125257.1	0.333312883	1.94E-14	postive
PAM	AC136604.3	0.303660652	3.99E-12	postive
PAM	AC137932.1	0.312289695	9.00E-13	postive
PAM	AC138956.2	0.324669397	9.75E-14	postive
PAM	AC139768.1	0.316692813	4.13E-13	postive
PAM	AC139795.2	0.30484177	3.26E-12	postive
PAM	AC141586.1	0.316126745	4.57E-13	postive
PAM	ADGRD1-AS1	0.321564184	1.72E-13	postive
PAM	AF111167.2	0.322560534	1.43E-13	postive
PAM	AL008726.1	0.317670982	3.47E-13	postive
PAM	AL035661.1	0.408997373	1.39E-21	postive
PAM	AL049597.2	0.313770943	6.94E-13	postive
PAM	AL049840.4	0.315352988	5.24E-13	postive
PAM	AL049840.5	0.307574034	2.04E-12	postive
PAM	AL050309.1	0.348649537	9.76E-16	postive
PAM	AL078581.2	0.337902868	8.07E-15	postive
PAM	AL078644.2	0.369542565	1.26E-17	postive
PAM	AL117327.1	0.31450914	6.09E-13	postive
PAM	AL122035.1	0.463998735	4.63E-28	postive
PAM	AL132857.1	0.368961623	1.43E-17	postive
PAM	AL133355.1	0.32531789	8.65E-14	postive
PAM	AL138756.1	0.320262184	2.18E-13	postive
PAM	AL138995.1	0.320302801	2.16E-13	postive
PAM	AL161785.1	0.302244069	5.07E-12	postive
PAM	AL162231.2	0.39295537	6.55E-20	postive
PAM	AL353743.1	0.300577697	6.71E-12	postive
PAM	AL354920.1	0.331276603	2.85E-14	postive
PAM	AL356235.1	0.341989162	3.65E-15	postive
PAM	AL356481.1	0.305860147	2.74E-12	postive
PAM	AL356599.1	0.321020976	1.90E-13	postive
PAM	AL391121.1	0.341912004	3.71E-15	postive
PAM	AL391834.2	0.315574044	5.04E-13	postive
PAM	AL450326.1	0.303063812	4.41E-12	postive
PAM	AL731577.2	0.364185404	3.97E-17	postive
PAM	AMZ2P1	0.323235954	1.27E-13	postive

PAM	AP000317.2	0.338346317	7.41E-15	postive
PAM	AP001189.3	0.307108223	2.21E-12	postive
PAM	AP001330.4	0.314841532	5.74E-13	postive
PAM	AP001816.1	0.375734914	3.27E-18	postive
PAM	AP002840.2	0.32555547	8.28E-14	postive
PAM	AP003486.1	0.374401326	4.39E-18	postive
PAM	ARMCX5-GPRASP2	0.387657328	2.24E-19	postive
PAM	ASB16-AS1	0.307609381	2.03E-12	postive
PAM	ATXN1-AS1	0.324445377	1.02E-13	postive
PAM	BAALC-AS1	0.312373484	8.87E-13	postive
PAM	BAIAP2-DT	0.429149068	8.04E-24	postive
PAM	BDNF-AS	0.358277272	1.37E-16	postive
PAM	BMS1P20	0.346782486	1.42E-15	postive
PAM	BX322557.10	0.317897584	3.33E-13	postive
PAM	C18orf65	0.307478172	2.08E-12	postive
PAM	C21orf62-AS1	0.371037238	9.15E-18	postive
PAM	C22orf46	0.320925036	1.93E-13	postive
PAM	C5orf66	0.326350035	7.15E-14	postive
PAM	CENPBD1P1	0.388545192	1.82E-19	postive
PAM	CHROMR	0.314393266	6.21E-13	postive
PAM	CKMT2-AS1	0.420730836	7.21E-23	postive
PAM	COX10-AS1	0.339152464	6.34E-15	postive
PAM	CRNDE	0.345781697	1.73E-15	postive
PAM	CSPG4P12	0.335260875	1.34E-14	postive
PAM	CTBP1-DT	0.451682671	1.66E-26	postive
PAM	CTC-487M23.5	0.400995073	9.74E-21	postive
PAM	DGCR11	0.402872612	6.19E-21	postive
PAM	DHRS4-AS1	0.343945075	2.49E-15	postive
PAM	DNMBP-AS1	0.309585672	1.44E-12	postive
PAM	EBLN3P	0.38608617	3.21E-19	postive
PAM	EIF1B-AS1	0.322318502	1.50E-13	postive
PAM	EIF3J-DT	0.402621496	6.58E-21	postive
PAM	EPB41L4A-DT	0.402336368	7.05E-21	postive
PAM	ERVE-1	0.316063133	4.62E-13	postive
PAM	FAM111A-DT	0.335865436	1.19E-14	postive
PAM	FAM160A1-DT	0.300711247	6.56E-12	postive
PAM	FAM225B	0.348249346	1.06E-15	postive
PAM	FAM86DP	0.410634084	9.24E-22	postive
PAM	FAM86EP	0.382453884	7.32E-19	postive
PAM	FGD5-AS1	0.435492564	1.48E-24	postive
PAM	FGF7P3	0.324402811	1.02E-13	postive
PAM	FRG1HP	0.383318205	6.02E-19	postive
PAM	GGTA1P	0.317813109	3.38E-13	postive
PAM	GMDS-DT	0.308449978	1.76E-12	postive
PAM	GOLGA2P7	0.351422783	5.58E-16	postive
PAM	GS1-358P8.4	0.332637419	2.21E-14	postive
PAM	GTF2IP4	0.308267873	1.81E-12	postive
PAM	HCG18	0.313270073	7.58E-13	postive
PAM	HECW2-AS1	0.350694776	6.47E-16	postive
PAM	HEIH	0.395739207	3.41E-20	postive

PAM	HLX-AS1	0.315503129	5.10E-13	postive
PAM	IL10RB-DT	0.349268745	8.62E-16	postive
PAM	KIAA1671-AS1	0.301005995	6.25E-12	postive
PAM	LEF1-AS1	0.34432435	2.31E-15	postive
PAM	LINC00160	0.309714501	1.41E-12	postive
PAM	LINC00324	0.314331844	6.28E-13	postive
PAM	LINC00339	0.311197567	1.09E-12	postive
PAM	LINC00398	0.307930827	1.92E-12	postive
PAM	LINC00467	0.317846689	3.36E-13	postive
PAM	LINC00526	0.31820748	3.15E-13	postive
PAM	LINC00571	0.311980392	9.50E-13	postive
PAM	LINC00578	0.341067496	4.37E-15	postive
PAM	LINC00619	0.364022722	4.11E-17	postive
PAM	LINC00667	0.355586366	2.39E-16	postive
PAM	LINC00674	0.303528704	4.08E-12	postive
PAM	LINC00840	0.31337571	7.44E-13	postive
PAM	LINC00847	0.313433615	7.36E-13	postive
PAM	LINC00863	0.416853621	1.94E-22	postive
PAM	LINC00899	0.317739325	3.43E-13	postive
PAM	LINC00909	0.308441954	1.76E-12	postive
PAM	LINC00933	0.336306766	1.10E-14	postive
PAM	LINC00957	0.341928488	3.69E-15	postive
PAM	LINC00963	0.302681323	4.71E-12	postive
PAM	LINC01011	0.307707899	2.00E-12	postive
PAM	LINC01128	0.37787786	2.04E-18	postive
PAM	LINC01135	0.317993398	3.27E-13	postive
PAM	LINC01184	0.490680278	1.18E-31	postive
PAM	LINC01278	0.323901307	1.12E-13	postive
PAM	LINC01352	0.338586318	7.08E-15	postive
PAM	LINC01426	0.307163988	2.19E-12	postive
PAM	LINC01505	0.314680399	5.91E-13	postive
PAM	LINC01614	0.372894756	6.11E-18	postive
PAM	LINC01705	0.317736384	3.43E-13	postive
PAM	LINC01719	0.327055308	6.28E-14	postive
PAM	LINC01852	0.382306551	7.56E-19	postive
PAM	LINC01929	0.301052988	6.20E-12	postive
PAM	LINC02122	0.434581109	1.89E-24	postive
PAM	LINC02198	0.376375535	2.84E-18	postive
PAM	LINC02240	0.301532068	5.72E-12	postive
PAM	LINC02256	0.361642658	6.79E-17	postive
PAM	LINC02391	0.311299403	1.07E-12	postive
PAM	LINC02416	0.31124417	1.08E-12	postive
PAM	LINC02519	0.33305815	2.04E-14	postive
PAM	LINC02544	0.310058009	1.33E-12	postive
PAM	LINC02768	0.320249264	2.18E-13	postive
PAM	LINC02773	0.317995676	3.27E-13	postive
PAM	LOXL1-AS1	0.307742479	1.98E-12	postive
PAM	LRRC37A17P	0.397162753	2.43E-20	postive
PAM	LRRC37BP1	0.358556461	1.29E-16	postive
PAM	LYPLAL1-DT	0.329180612	4.23E-14	postive

PAM	MAGI2-AS3	0.311220659	1.09E-12	postive
PAM	MAP2K4P1	0.403224891	5.69E-21	postive
PAM	MAP4K3-DT	0.352072589	4.89E-16	postive
PAM	MED14OS	0.354065356	3.26E-16	postive
PAM	MID1IP1-AS1	0.327647257	5.62E-14	postive
PAM	MIR100HG	0.372309036	6.94E-18	postive
PAM	MIR22HG	0.404902678	3.78E-21	postive
PAM	MKLN1-AS	0.313106678	7.80E-13	postive
PAM	MMADHC-DT	0.339666353	5.74E-15	postive
PAM	MRPL20-AS1	0.37180841	7.74E-18	postive
PAM	MRPS31P4	0.326778991	6.61E-14	postive
PAM	NBR2	0.309065187	1.58E-12	postive
PAM	NIFK-AS1	0.305143347	3.10E-12	postive
PAM	NOP14-AS1	0.379781311	1.33E-18	postive
PAM	NOP53-AS1	0.333582283	1.85E-14	postive
PAM	NORAD	0.410143698	1.04E-21	postive
PAM	NR2F1-AS1	0.414745556	3.30E-22	postive
PAM	NUDT16P1	0.314973892	5.61E-13	postive
PAM	NUTM2A-AS1	0.362423692	5.76E-17	postive
PAM	OIP5-AS1	0.419738961	9.30E-23	postive
PAM	OTUD6B-AS1	0.41339738	4.64E-22	postive
PAM	PAXIP1-AS2	0.440584918	3.69E-25	postive
PAM	PICART1	0.457133765	3.47E-27	postive
PAM	PINK1-AS	0.326272172	7.26E-14	postive
PAM	PMS2P4	0.358521401	1.30E-16	postive
PAM	PRELID1P1	0.352878099	4.15E-16	postive
PAM	PRKAR2A-AS1	0.308288217	1.81E-12	postive
PAM	PRR34	0.359832646	9.92E-17	postive
PAM	PSMG3-AS1	0.331819998	2.58E-14	postive
PAM	PTENP1	0.368129156	1.71E-17	postive
PAM	PTPRD-AS1	0.351671603	5.31E-16	postive
PAM	RAB11B-AS1	0.325509189	8.35E-14	postive
PAM	RAP2C-AS1	0.489693148	1.63E-31	postive
PAM	RARA-AS1	0.391541199	9.11E-20	postive
PAM	RBM15-AS1	0.300468951	6.84E-12	postive
PAM	RNF216P1	0.343214754	2.87E-15	postive
PAM	RP11-101E13.5	0.321116835	1.86E-13	postive
PAM	RP11-10C24.1	0.310843167	1.16E-12	postive
PAM	RP11-159G9.5	0.367864564	1.81E-17	postive
PAM	RP11-164P12.4	0.331897964	2.54E-14	postive
PAM	RP11-175O19.4	0.305628705	2.85E-12	postive
PAM	RP11-180P8.1	0.337573205	8.60E-15	postive
PAM	RP11-182J1.1	0.330305137	3.42E-14	postive
PAM	RP11-226L15.5	0.305244687	3.05E-12	postive
PAM	RP1-122P22.2	0.459205167	1.90E-27	postive
PAM	RP11-337C18.9	0.320531893	2.07E-13	postive
PAM	RP11-357P18.2	0.300000839	7.39E-12	postive
PAM	RP11-439L18.2	0.31187242	9.68E-13	postive
PAM	RP11-524D16__A.3	0.430329853	5.88E-24	postive
PAM	RP11-539I5.1	0.371766236	7.81E-18	postive

PAM	RP11-86H7.7	0.339499535	5.93E-15	postive
PAM	RP5-1042I8.7	0.33930394	6.16E-15	postive
PAM	RPARP-AS1	0.301056258	6.19E-12	postive
PAM	RPL23AP53	0.389364247	1.51E-19	postive
PAM	RPL23AP7	0.360975668	7.81E-17	postive
PAM	RPL23AP82	0.477721175	7.20E-30	postive
PAM	RPS10P7	0.342410074	3.36E-15	postive
PAM	SAP30-DT	0.30488219	3.24E-12	postive
PAM	SBDSP1	0.356228295	2.09E-16	postive
PAM	SCAMP1-AS1	0.337465057	8.78E-15	postive
PAM	SCAND2P	0.335952896	1.17E-14	postive
PAM	SDAD1P1	0.365938621	2.74E-17	postive
PAM	SEC22B4P	0.36256	5.60E-17	postive
PAM	SEPTIN7-AS1	0.359767847	1.01E-16	postive
PAM	SGMS1-AS1	0.327926527	5.34E-14	postive
PAM	SH3RF3-AS1	0.33965168	5.76E-15	postive
PAM	SLC44A3-AS1	0.360178401	9.23E-17	postive
PAM	SNHG8	0.328451385	4.84E-14	postive
PAM	SPRY4-AS1	0.416576791	2.08E-22	postive
PAM	ST20-AS1	0.314301761	6.31E-13	postive
PAM	STXBP5-AS1	0.303776565	3.91E-12	postive
PAM	SUCLG2-AS1	0.365275785	3.15E-17	postive
PAM	SVIL-AS1	0.399596534	1.36E-20	postive
PAM	SYNPR-AS1	0.331607733	2.68E-14	postive
PAM	TBX5-AS1	0.418660714	1.22E-22	postive
PAM	THAP9-AS1	0.307431328	2.09E-12	postive
PAM	TMEM161B-AS1	0.342756999	3.14E-15	postive
PAM	TNFRSF10A-AS1	0.39027407	1.22E-19	postive
PAM	TP53TG1	0.318584586	2.94E-13	postive
PAM	TP73-AS1	0.390483085	1.17E-19	postive
PAM	TRAM2-AS1	0.38369566	5.53E-19	postive
PAM	TRIM52-AS1	0.356780333	1.87E-16	postive
PAM	TSTD3	0.317004721	3.91E-13	postive
PAM	TTC28-AS1	0.314071784	6.58E-13	postive
PAM	TUG1	0.346604502	1.47E-15	postive
PAM	U52111.1	0.372994494	5.97E-18	postive
PAM	UBA6-AS1	0.392530528	7.24E-20	postive
PAM	UBE2D3-AS1	0.329049326	4.33E-14	postive
PAM	USP27X-AS1	0.39581342	3.35E-20	postive
PAM	VAC14-AS1	0.342355074	3.40E-15	postive
PAM	WAC-AS1	0.319064771	2.70E-13	postive
PAM	WDFY3-AS2	0.356383479	2.03E-16	postive
PAM	ZBTB11-AS1	0.319712252	2.40E-13	postive
PAM	ZNF252P	0.383290619	6.06E-19	postive
PAM	ZNF561-AS1	0.411170547	8.08E-22	postive
PAM	ZNF658B	0.320196258	2.20E-13	postive
PAM	ZNF815P	0.336876933	9.84E-15	postive
PDHA1	A2M-AS1	0.317389037	3.65E-13	postive
PDHA1	AC000068.1	0.404752422	3.92E-21	postive
PDHA1	AC000068.2	0.395168702	3.90E-20	postive

PDHA1	AC002116.2	0.446630849	6.91E-26	postive
PDHA1	AC002310.1	0.460106792	1.46E-27	postive
PDHA1	AC002310.2	0.337230689	9.19E-15	postive
PDHA1	AC002398.1	0.35218661	4.78E-16	postive
PDHA1	AC002401.2	0.37674095	2.62E-18	postive
PDHA1	AC002401.3	0.31894868	2.76E-13	postive
PDHA1	AC002467.1	0.410436655	9.70E-22	postive
PDHA1	AC002470.1	0.489644737	1.65E-31	postive
PDHA1	AC002550.2	0.338722634	6.89E-15	postive
PDHA1	AC003070.2	0.301587545	5.66E-12	postive
PDHA1	AC003101.2	0.395625817	3.50E-20	postive
PDHA1	AC003102.1	0.371137595	8.95E-18	postive
PDHA1	AC003956.1	0.405521895	3.25E-21	postive
PDHA1	AC004067.1	0.314890014	5.69E-13	postive
PDHA1	AC004069.1	0.3111548	1.10E-12	postive
PDHA1	AC004241.1	0.418294104	1.34E-22	postive
PDHA1	AC004241.2	0.300226303	7.12E-12	postive
PDHA1	AC004241.3	0.412987932	5.14E-22	postive
PDHA1	AC004253.1	0.314800987	5.78E-13	postive
PDHA1	AC004263.1	0.308350057	1.79E-12	postive
PDHA1	AC004447.2	0.438083475	7.32E-25	postive
PDHA1	AC004477.1	0.431146776	4.73E-24	postive
PDHA1	AC004477.3	0.39019421	1.25E-19	postive
PDHA1	AC004596.1	0.419106688	1.09E-22	postive
PDHA1	AC004771.2	0.444757131	1.17E-25	postive
PDHA1	AC004771.4	0.359286827	1.11E-16	postive
PDHA1	AC004801.2	0.317941855	3.30E-13	postive
PDHA1	AC004801.6	0.37801974	1.97E-18	postive
PDHA1	AC004803.1	0.409089775	1.35E-21	postive
PDHA1	AC004812.2	0.547498949	1.91E-40	postive
PDHA1	AC004825.2	0.337311563	9.05E-15	postive
PDHA1	AC004893.11	0.465209345	3.23E-28	postive
PDHA1	AC004895.1	0.302934476	4.51E-12	postive
PDHA1	AC004908.2	0.328099962	5.17E-14	postive
PDHA1	AC004943.2	0.419657378	9.49E-23	postive
PDHA1	AC004951.1	0.334875521	1.44E-14	postive
PDHA1	AC004973.1	0.355146504	2.61E-16	postive
PDHA1	AC004980.1	0.372611884	6.49E-18	postive
PDHA1	AC005005.3	0.434133741	2.13E-24	postive
PDHA1	AC005005.4	0.342401066	3.37E-15	postive
PDHA1	AC005034.4	0.470449312	6.69E-29	postive
PDHA1	AC005034.5	0.311290206	1.07E-12	postive
PDHA1	AC005034.6	0.376052125	3.05E-18	postive
PDHA1	AC005037.1	0.300671469	6.61E-12	postive
PDHA1	AC005041.3	0.404570354	4.10E-21	postive
PDHA1	AC005076.2	0.384208942	4.92E-19	postive
PDHA1	AC005089.1	0.300703554	6.57E-12	postive
PDHA1	AC005104.1	0.307658758	2.01E-12	postive
PDHA1	AC005225.2	0.450712123	2.19E-26	postive
PDHA1	AC005229.4	0.474466764	1.97E-29	postive

PDHA1	AC005253.1	0.332087602	2.45E-14	postive
PDHA1	AC005261.4	0.356869569	1.83E-16	postive
PDHA1	AC005288.1	0.453824833	9.00E-27	postive
PDHA1	AC005324.5	0.394801174	4.25E-20	postive
PDHA1	AC005332.1	0.341732382	3.84E-15	postive
PDHA1	AC005332.4	0.339370046	6.08E-15	postive
PDHA1	AC005363.2	0.300186129	7.17E-12	postive
PDHA1	AC005387.1	0.397147051	2.44E-20	postive
PDHA1	AC005387.2	0.33997132	5.41E-15	postive
PDHA1	AC005498.3	0.301376143	5.87E-12	postive
PDHA1	AC005519.1	0.358080426	1.43E-16	postive
PDHA1	AC005520.4	0.356255716	2.08E-16	postive
PDHA1	AC005562.1	0.489104373	1.97E-31	postive
PDHA1	AC005586.1	0.399625015	1.35E-20	postive
PDHA1	AC005670.1	0.330108504	3.55E-14	postive
PDHA1	AC005670.3	0.493631146	4.53E-32	postive
PDHA1	AC005695.1	0.316890682	3.99E-13	postive
PDHA1	AC005696.1	0.468640974	1.16E-28	postive
PDHA1	AC005837.1	0.372793232	6.24E-18	postive
PDHA1	AC005840.4	0.322634819	1.41E-13	postive
PDHA1	AC005841.1	0.362679273	5.46E-17	postive
PDHA1	AC005911.1	0.466869529	1.97E-28	postive
PDHA1	AC005920.1	0.328127239	5.14E-14	postive
PDHA1	AC005920.3	0.310668914	1.19E-12	postive
PDHA1	AC006001.2	0.442783025	2.02E-25	postive
PDHA1	AC006001.3	0.312221672	9.11E-13	postive
PDHA1	AC006064.3	0.350902365	6.20E-16	postive
PDHA1	AC006111.2	0.325661893	8.12E-14	postive
PDHA1	AC006213.1	0.352768955	4.25E-16	postive
PDHA1	AC006252.1	0.474961592	1.69E-29	postive
PDHA1	AC006333.1	0.424200146	2.94E-23	postive
PDHA1	AC006449.2	0.47104431	5.59E-29	postive
PDHA1	AC006449.5	0.391608531	8.97E-20	postive
PDHA1	AC006449.7	0.45149678	1.75E-26	postive
PDHA1	AC006504.5	0.364203891	3.96E-17	postive
PDHA1	AC006547.1	0.435136994	1.63E-24	postive
PDHA1	AC006942.1	0.361342372	7.23E-17	postive
PDHA1	AC007032.1	0.37396447	4.83E-18	postive
PDHA1	AC007036.1	0.364669578	3.58E-17	postive
PDHA1	AC007066.2	0.422061932	5.12E-23	postive
PDHA1	AC007098.1	0.384253591	4.87E-19	postive
PDHA1	AC007114.1	0.41035242	9.91E-22	postive
PDHA1	AC007228.9	0.317498526	3.58E-13	postive
PDHA1	AC007292.1	0.303062054	4.42E-12	postive
PDHA1	AC007292.2	0.304032285	3.75E-12	postive
PDHA1	AC007342.4	0.326018656	7.60E-14	postive
PDHA1	AC007383.2	0.433410041	2.59E-24	postive
PDHA1	AC007405.4	0.314613077	5.98E-13	postive
PDHA1	AC007406.5	0.310388973	1.25E-12	postive
PDHA1	AC007541.1	0.517737353	1.24E-35	postive

PDHA1	AC007663.3	0.374036497	4.76E-18	postive
PDHA1	AC007663.4	0.376930519	2.51E-18	postive
PDHA1	AC007686.3	0.346478422	1.51E-15	postive
PDHA1	AC007881.3	0.335655415	1.24E-14	postive
PDHA1	AC008014.1	0.343147993	2.91E-15	postive
PDHA1	AC008063.1	0.342165403	3.53E-15	postive
PDHA1	AC008105.1	0.351459632	5.54E-16	postive
PDHA1	AC008105.2	0.330770633	3.14E-14	postive
PDHA1	AC008124.1	0.380761884	1.07E-18	postive
PDHA1	AC008267.5	0.535208814	2.12E-38	postive
PDHA1	AC008393.1	0.379886516	1.30E-18	postive
PDHA1	AC008438.1	0.488791546	2.18E-31	postive
PDHA1	AC008443.1	0.537041374	1.06E-38	postive
PDHA1	AC008443.5	0.475912404	1.26E-29	postive
PDHA1	AC008494.3	0.306286605	2.55E-12	postive
PDHA1	AC008543.1	0.308844232	1.64E-12	postive
PDHA1	AC008592.1	0.354115692	3.23E-16	postive
PDHA1	AC008592.4	0.331359566	2.81E-14	postive
PDHA1	AC008608.2	0.41363739	4.36E-22	postive
PDHA1	AC008610.1	0.320200825	2.20E-13	postive
PDHA1	AC008619.1	0.355744815	2.31E-16	postive
PDHA1	AC008622.2	0.425007063	2.38E-23	postive
PDHA1	AC008734.1	0.341430204	4.07E-15	postive
PDHA1	AC008735.1	0.340416823	4.96E-15	postive
PDHA1	AC008735.2	0.38571726	3.49E-19	postive
PDHA1	AC008735.4	0.347190822	1.31E-15	postive
PDHA1	AC008747.1	0.314332793	6.28E-13	postive
PDHA1	AC008764.6	0.341839525	3.76E-15	postive
PDHA1	AC008764.8	0.301478382	5.77E-12	postive
PDHA1	AC008771.1	0.367838949	1.82E-17	postive
PDHA1	AC008781.1	0.311665698	1.00E-12	postive
PDHA1	AC008870.3	0.335704905	1.23E-14	postive
PDHA1	AC008870.5	0.350951648	6.14E-16	postive
PDHA1	AC008915.3	0.451144707	1.93E-26	postive
PDHA1	AC008946.1	0.361457917	7.06E-17	postive
PDHA1	AC008966.1	0.338782951	6.81E-15	postive
PDHA1	AC008969.1	0.300906524	6.35E-12	postive
PDHA1	AC008991.1	0.34743213	1.24E-15	postive
PDHA1	AC009005.1	0.339943989	5.44E-15	postive
PDHA1	AC009041.3	0.312896121	8.09E-13	postive
PDHA1	AC009053.3	0.371351716	8.54E-18	postive
PDHA1	AC009055.2	0.327797042	5.47E-14	postive
PDHA1	AC009061.2	0.527324346	3.93E-37	postive
PDHA1	AC009065.5	0.389459243	1.48E-19	postive
PDHA1	AC009065.9	0.486034177	5.27E-31	postive
PDHA1	AC009113.1	0.445972907	8.31E-26	postive
PDHA1	AC009118.2	0.439016451	5.68E-25	postive
PDHA1	AC009118.3	0.358455314	1.32E-16	postive
PDHA1	AC009121.3	0.399322908	1.45E-20	postive
PDHA1	AC009121.4	0.425905165	1.89E-23	postive

PDHA1	AC009126.1	0.501830949	2.99E-33	postive
PDHA1	AC009159.3	0.344727888	2.13E-15	postive
PDHA1	AC009163.7	0.334154285	1.66E-14	postive
PDHA1	AC009171.2	0.407987882	1.78E-21	postive
PDHA1	AC009237.14	0.367217824	2.08E-17	postive
PDHA1	AC009309.2	0.415894747	2.47E-22	postive
PDHA1	AC009403.1	0.409844175	1.12E-21	postive
PDHA1	AC009506.2	0.407143155	2.19E-21	postive
PDHA1	AC009570.1	0.344033168	2.44E-15	postive
PDHA1	AC009630.2	0.350934037	6.16E-16	postive
PDHA1	AC009690.2	0.500109028	5.32E-33	postive
PDHA1	AC009779.3	0.559296407	1.72E-42	postive
PDHA1	AC009831.1	0.39775847	2.11E-20	postive
PDHA1	AC009948.3	0.314512409	6.08E-13	postive
PDHA1	AC009955.1	0.304734905	3.32E-12	postive
PDHA1	AC009961.1	0.370038587	1.14E-17	postive
PDHA1	AC009988.1	0.344856075	2.08E-15	postive
PDHA1	AC010173.1	0.365268273	3.16E-17	postive
PDHA1	AC010203.2	0.382354301	7.48E-19	postive
PDHA1	AC010205.1	0.38934199	1.52E-19	postive
PDHA1	AC010226.1	0.343245355	2.85E-15	postive
PDHA1	AC010271.2	0.319087032	2.69E-13	postive
PDHA1	AC010273.2	0.324886947	9.37E-14	postive
PDHA1	AC010319.4	0.416160155	2.31E-22	postive
PDHA1	AC010319.5	0.403992874	4.72E-21	postive
PDHA1	AC010320.2	0.351894109	5.07E-16	postive
PDHA1	AC010326.4	0.304455391	3.49E-12	postive
PDHA1	AC010326.5	0.316976474	3.93E-13	postive
PDHA1	AC010331.1	0.346699021	1.44E-15	postive
PDHA1	AC010359.2	0.376937427	2.51E-18	postive
PDHA1	AC010422.2	0.350742429	6.41E-16	postive
PDHA1	AC010442.1	0.447423399	5.53E-26	postive
PDHA1	AC010463.3	0.433286695	2.67E-24	postive
PDHA1	AC010491.1	0.308017098	1.89E-12	postive
PDHA1	AC010503.5	0.32372559	1.16E-13	postive
PDHA1	AC010504.1	0.425077597	2.34E-23	postive
PDHA1	AC010531.6	0.466493152	2.20E-28	postive
PDHA1	AC010536.1	0.318715679	2.88E-13	postive
PDHA1	AC010618.3	0.345108155	1.98E-15	postive
PDHA1	AC010655.4	0.360972304	7.81E-17	postive
PDHA1	AC010761.1	0.376336151	2.87E-18	postive
PDHA1	AC010761.4	0.353929382	3.35E-16	postive
PDHA1	AC010761.6	0.340670117	4.72E-15	postive
PDHA1	AC010809.1	0.341664319	3.89E-15	postive
PDHA1	AC010834.2	0.406160616	2.78E-21	postive
PDHA1	AC010864.1	0.433537183	2.50E-24	postive
PDHA1	AC010883.1	0.354703649	2.86E-16	postive
PDHA1	AC010894.2	0.448414689	4.19E-26	postive
PDHA1	AC010904.2	0.354699997	2.86E-16	postive
PDHA1	AC010913.1	0.456864675	3.75E-27	postive

PDHA1	AC010969.2	0.393697798	5.51E-20	postive
PDHA1	AC010973.2	0.351379662	5.63E-16	postive
PDHA1	AC010997.4	0.445376348	9.81E-26	postive
PDHA1	AC010997.5	0.321128968	1.86E-13	postive
PDHA1	AC010999.2	0.328382182	4.91E-14	postive
PDHA1	AC011450.1	0.387049466	2.57E-19	postive
PDHA1	AC011462.4	0.302008272	5.28E-12	postive
PDHA1	AC011472.1	0.364651001	3.60E-17	postive
PDHA1	AC011474.1	0.338334027	7.43E-15	postive
PDHA1	AC011474.2	0.362010265	6.29E-17	postive
PDHA1	AC011481.1	0.362116787	6.15E-17	postive
PDHA1	AC011487.1	0.314176828	6.46E-13	postive
PDHA1	AC011498.3	0.31013193	1.31E-12	postive
PDHA1	AC011524.1	0.349633396	8.01E-16	postive
PDHA1	AC011603.3	0.374275455	4.51E-18	postive
PDHA1	AC011676.1	0.322813576	1.37E-13	postive
PDHA1	AC011731.1	0.355699376	2.33E-16	postive
PDHA1	AC011921.1	0.36604673	2.67E-17	postive
PDHA1	AC011978.2	0.310065705	1.33E-12	postive
PDHA1	AC012063.1	0.46460428	3.87E-28	postive
PDHA1	AC012073.1	0.394912605	4.14E-20	postive
PDHA1	AC012146.1	0.345684237	1.76E-15	postive
PDHA1	AC012184.3	0.499540867	6.43E-33	postive
PDHA1	AC012186.2	0.348376836	1.03E-15	postive
PDHA1	AC012236.1	0.326884719	6.48E-14	postive
PDHA1	AC012306.2	0.487087621	3.76E-31	postive
PDHA1	AC012313.1	0.438429926	6.66E-25	postive
PDHA1	AC012313.9	0.402628174	6.57E-21	postive
PDHA1	AC012360.3	0.447069801	6.11E-26	postive
PDHA1	AC012442.2	0.3883777	1.90E-19	postive
PDHA1	AC012442.3	0.300900751	6.36E-12	postive
PDHA1	AC012456.2	0.348338837	1.04E-15	postive
PDHA1	AC012467.2	0.437364522	8.90E-25	postive
PDHA1	AC012510.1	0.4125149	5.78E-22	postive
PDHA1	AC012555.1	0.424536785	2.69E-23	postive
PDHA1	AC012615.1	0.396552892	2.81E-20	postive
PDHA1	AC012640.4	0.316281419	4.44E-13	postive
PDHA1	AC012676.1	0.352384316	4.59E-16	postive
PDHA1	AC013400.1	0.330084229	3.57E-14	postive
PDHA1	AC013468.1	0.303978206	3.78E-12	postive
PDHA1	AC013565.3	0.300435762	6.87E-12	postive
PDHA1	AC013724.1	0.354490754	2.99E-16	postive
PDHA1	AC015802.4	0.361056355	7.68E-17	postive
PDHA1	AC015802.5	0.326638673	6.78E-14	postive
PDHA1	AC015819.1	0.368927521	1.44E-17	postive
PDHA1	AC015912.3	0.359296031	1.11E-16	postive
PDHA1	AC015922.2	0.358518504	1.30E-16	postive
PDHA1	AC015922.3	0.343759263	2.58E-15	postive
PDHA1	AC015982.2	0.426919066	1.45E-23	postive
PDHA1	AC016027.1	0.329956825	3.66E-14	postive

PDHA1	AC016065.1	0.5243001	1.18E-36	postive
PDHA1	AC016134.1	0.301679915	5.58E-12	postive
PDHA1	AC016355.1	0.349820998	7.71E-16	postive
PDHA1	AC016394.2	0.484555286	8.43E-31	postive
PDHA1	AC016394.3	0.326586595	6.85E-14	postive
PDHA1	AC016405.3	0.314182587	6.45E-13	postive
PDHA1	AC016722.2	0.33329823	1.95E-14	postive
PDHA1	AC016727.1	0.409526144	1.22E-21	postive
PDHA1	AC016737.1	0.399433381	1.42E-20	postive
PDHA1	AC016747.1	0.31745289	3.61E-13	postive
PDHA1	AC016773.2	0.375887521	3.17E-18	postive
PDHA1	AC016876.2	0.428709128	9.03E-24	postive
PDHA1	AC016876.3	0.495556605	2.41E-32	postive
PDHA1	AC016876.4	0.318435734	3.02E-13	postive
PDHA1	AC016888.1	0.400919478	9.92E-21	postive
PDHA1	AC017083.1	0.426623415	1.56E-23	postive
PDHA1	AC018413.1	0.328228885	5.05E-14	postive
PDHA1	AC018521.5	0.368177965	1.70E-17	postive
PDHA1	AC018521.6	0.314248588	6.37E-13	postive
PDHA1	AC018647.2	0.402262908	7.17E-21	postive
PDHA1	AC018653.3	0.343352788	2.79E-15	postive
PDHA1	AC018690.1	0.400694044	1.05E-20	postive
PDHA1	AC018695.4	0.33096592	3.02E-14	postive
PDHA1	AC018766.4	0.341908306	3.71E-15	postive
PDHA1	AC018809.1	0.311749671	9.89E-13	postive
PDHA1	AC018809.2	0.363937677	4.19E-17	postive
PDHA1	AC018845.3	0.368592839	1.55E-17	postive
PDHA1	AC019069.1	0.327197298	6.11E-14	postive
PDHA1	AC019131.2	0.335612997	1.25E-14	postive
PDHA1	AC019205.1	0.357685303	1.55E-16	postive
PDHA1	AC019294.2	0.415524123	2.71E-22	postive
PDHA1	AC020558.1	0.336013524	1.16E-14	postive
PDHA1	AC020558.2	0.382977197	6.50E-19	postive
PDHA1	AC020663.2	0.413816937	4.17E-22	postive
PDHA1	AC020765.2	0.350220973	7.12E-16	postive
PDHA1	AC020904.2	0.411152755	8.12E-22	postive
PDHA1	AC020907.4	0.348623474	9.81E-16	postive
PDHA1	AC020910.5	0.344853629	2.08E-15	postive
PDHA1	AC020915.2	0.312777004	8.26E-13	postive
PDHA1	AC020915.3	0.471398831	5.02E-29	postive
PDHA1	AC020934.2	0.315511293	5.10E-13	postive
PDHA1	AC021016.3	0.337405255	8.89E-15	postive
PDHA1	AC021148.1	0.313458988	7.33E-13	postive
PDHA1	AC021224.1	0.33827143	7.52E-15	postive
PDHA1	AC021242.3	0.327995026	5.27E-14	postive
PDHA1	AC021755.2	0.311556864	1.02E-12	postive
PDHA1	AC022007.1	0.401708396	8.20E-21	postive
PDHA1	AC022098.1	0.367305704	2.04E-17	postive
PDHA1	AC022150.2	0.401087354	9.52E-21	postive
PDHA1	AC022167.2	0.38692802	2.65E-19	postive

PDHA1	AC022167.4	0.314189521	6.44E-13	postive
PDHA1	AC022211.3	0.414326293	3.67E-22	postive
PDHA1	AC022364.1	0.364527213	3.69E-17	postive
PDHA1	AC022415.2	0.315773235	4.87E-13	postive
PDHA1	AC022784.2	0.314156323	6.48E-13	postive
PDHA1	AC022784.5	0.306394495	2.50E-12	postive
PDHA1	AC022784.6	0.425751883	1.96E-23	postive
PDHA1	AC022915.1	0.328952586	4.41E-14	postive
PDHA1	AC022916.2	0.407704951	1.90E-21	postive
PDHA1	AC023024.1	0.35995371	9.67E-17	postive
PDHA1	AC023043.1	0.304575936	3.41E-12	postive
PDHA1	AC023509.3	0.395757851	3.39E-20	postive
PDHA1	AC023813.3	0.378115194	1.93E-18	postive
PDHA1	AC023906.5	0.38333205	6.00E-19	postive
PDHA1	AC023983.2	0.413989548	4.00E-22	postive
PDHA1	AC024060.2	0.351987657	4.98E-16	postive
PDHA1	AC024243.1	0.400607993	1.07E-20	postive
PDHA1	AC024257.3	0.333628455	1.83E-14	postive
PDHA1	AC024267.7	0.43594828	1.31E-24	postive
PDHA1	AC024361.2	0.420163777	8.34E-23	postive
PDHA1	AC024560.1	0.305780163	2.78E-12	postive
PDHA1	AC024575.2	0.422719084	4.32E-23	postive
PDHA1	AC024896.1	0.31579973	4.84E-13	postive
PDHA1	AC025048.2	0.417255005	1.75E-22	postive
PDHA1	AC025162.2	0.335375991	1.31E-14	postive
PDHA1	AC025171.1	0.344308912	2.31E-15	postive
PDHA1	AC025181.2	0.341101637	4.34E-15	postive
PDHA1	AC025262.1	0.316571842	4.22E-13	postive
PDHA1	AC025265.1	0.364698572	3.56E-17	postive
PDHA1	AC025682.2	0.402363262	7.00E-21	postive
PDHA1	AC025857.2	0.354524528	2.97E-16	postive
PDHA1	AC026191.1	0.311125226	1.10E-12	postive
PDHA1	AC026254.2	0.386293415	3.06E-19	postive
PDHA1	AC026304.1	0.478479116	5.69E-30	postive
PDHA1	AC026367.1	0.382333683	7.52E-19	postive
PDHA1	AC026367.3	0.327941164	5.33E-14	postive
PDHA1	AC026401.3	0.465824773	2.69E-28	postive
PDHA1	AC026471.1	0.511120101	1.26E-34	postive
PDHA1	AC026471.2	0.353073124	3.99E-16	postive
PDHA1	AC026471.4	0.379655798	1.37E-18	postive
PDHA1	AC026691.1	0.455337895	5.82E-27	postive
PDHA1	AC026803.2	0.356446736	2.00E-16	postive
PDHA1	AC026979.2	0.37055768	1.01E-17	postive
PDHA1	AC027097.1	0.427286205	1.31E-23	postive
PDHA1	AC027097.2	0.394794879	4.26E-20	postive
PDHA1	AC027307.2	0.33611688	1.14E-14	postive
PDHA1	AC027559.1	0.344466617	2.24E-15	postive
PDHA1	AC027601.2	0.339702247	5.70E-15	postive
PDHA1	AC027644.3	0.455768545	5.14E-27	postive
PDHA1	AC027682.1	0.320971217	1.91E-13	postive

PDHA1	AC027682.6	0.410889438	8.67E-22	postive
PDHA1	AC027796.4	0.375071563	3.79E-18	postive
PDHA1	AC034102.6	0.358774492	1.24E-16	postive
PDHA1	AC034236.2	0.39776997	2.11E-20	postive
PDHA1	AC036176.1	0.452758163	1.22E-26	postive
PDHA1	AC037459.2	0.378701019	1.70E-18	postive
PDHA1	AC040162.3	0.347841733	1.15E-15	postive
PDHA1	AC040169.1	0.426218241	1.74E-23	postive
PDHA1	AC040169.3	0.350814767	6.31E-16	postive
PDHA1	AC040914.1	0.303007193	4.46E-12	postive
PDHA1	AC040970.1	0.329595744	3.91E-14	postive
PDHA1	AC046134.2	0.556739842	4.85E-42	postive
PDHA1	AC046143.2	0.392080636	8.04E-20	postive
PDHA1	AC048337.1	0.328994507	4.38E-14	postive
PDHA1	AC048382.1	0.329394133	4.06E-14	postive
PDHA1	AC053503.1	0.366940694	2.21E-17	postive
PDHA1	AC053503.4	0.351938062	5.03E-16	postive
PDHA1	AC055855.2	0.356586749	1.94E-16	postive
PDHA1	AC060766.7	0.303965866	3.79E-12	postive
PDHA1	AC060780.1	0.430666535	5.38E-24	postive
PDHA1	AC061992.2	0.301099592	6.15E-12	postive
PDHA1	AC063919.1	0.342845602	3.09E-15	postive
PDHA1	AC063943.2	0.340336991	5.04E-15	postive
PDHA1	AC064807.1	0.551254679	4.34E-41	postive
PDHA1	AC064807.4	0.341661929	3.89E-15	postive
PDHA1	AC064836.3	0.379736922	1.35E-18	postive
PDHA1	AC067750.1	0.44362128	1.60E-25	postive
PDHA1	AC067852.2	0.587123121	1.19E-47	postive
PDHA1	AC068025.1	0.341621213	3.92E-15	postive
PDHA1	AC068189.1	0.37419105	4.60E-18	postive
PDHA1	AC068338.2	0.456026317	4.77E-27	postive
PDHA1	AC068473.5	0.477049257	8.87E-30	postive
PDHA1	AC068580.1	0.301456183	5.79E-12	postive
PDHA1	AC068580.3	0.354332117	3.09E-16	postive
PDHA1	AC068620.1	0.35694622	1.80E-16	postive
PDHA1	AC068620.2	0.30422582	3.62E-12	postive
PDHA1	AC068790.9	0.344419442	2.26E-15	postive
PDHA1	AC068831.2	0.566216829	9.94E-44	postive
PDHA1	AC068870.2	0.369611621	1.25E-17	postive
PDHA1	AC068888.1	0.399061	1.55E-20	postive
PDHA1	AC069185.1	0.305924757	2.71E-12	postive
PDHA1	AC069224.2	0.416227274	2.27E-22	postive
PDHA1	AC069307.1	0.300569593	6.72E-12	postive
PDHA1	AC069544.1	0.449016898	3.53E-26	postive
PDHA1	AC072061.1	0.384433984	4.67E-19	postive
PDHA1	AC073046.1	0.343054178	2.96E-15	postive
PDHA1	AC073073.2	0.384849659	4.25E-19	postive
PDHA1	AC073111.1	0.37230036	6.95E-18	postive
PDHA1	AC073195.2	0.520551599	4.54E-36	postive
PDHA1	AC073254.1	0.335562828	1.27E-14	postive

PDHA1	AC073321.1	0.315194176	5.39E-13	postive
PDHA1	AC073352.1	0.351032366	6.04E-16	postive
PDHA1	AC073370.1	0.329165618	4.24E-14	postive
PDHA1	AC073508.3	0.37029045	1.08E-17	postive
PDHA1	AC073529.1	0.531815632	7.51E-38	postive
PDHA1	AC073569.2	0.428768483	8.89E-24	postive
PDHA1	AC073575.4	0.410064482	1.06E-21	postive
PDHA1	AC073611.1	0.384272496	4.85E-19	postive
PDHA1	AC073842.1	0.328583042	4.73E-14	postive
PDHA1	AC073842.2	0.400187189	1.18E-20	postive
PDHA1	AC073863.1	0.377028034	2.46E-18	postive
PDHA1	AC073869.1	0.480614172	2.92E-30	postive
PDHA1	AC073896.2	0.504921326	1.05E-33	postive
PDHA1	AC073896.3	0.42556394	2.06E-23	postive
PDHA1	AC073896.4	0.401632133	8.35E-21	postive
PDHA1	AC074029.3	0.358355547	1.35E-16	postive
PDHA1	AC074117.1	0.472802564	3.27E-29	postive
PDHA1	AC074212.1	0.346395196	1.53E-15	postive
PDHA1	AC078785.1	0.353511126	3.65E-16	postive
PDHA1	AC078802.1	0.314133715	6.51E-13	postive
PDHA1	AC078846.1	0.375652376	3.33E-18	postive
PDHA1	AC078880.3	0.359145739	1.14E-16	postive
PDHA1	AC078880.4	0.332618493	2.21E-14	postive
PDHA1	AC078880.5	0.35155726	5.43E-16	postive
PDHA1	AC079145.1	0.321765374	1.66E-13	postive
PDHA1	AC079174.2	0.425035932	2.37E-23	postive
PDHA1	AC079209.1	0.367973002	1.77E-17	postive
PDHA1	AC079305.2	0.350486478	6.74E-16	postive
PDHA1	AC079313.2	0.350875597	6.24E-16	postive
PDHA1	AC079414.3	0.398001325	1.99E-20	postive
PDHA1	AC079807.1	0.511758462	1.01E-34	postive
PDHA1	AC079848.1	0.44277425	2.02E-25	postive
PDHA1	AC079907.1	0.342733695	3.15E-15	postive
PDHA1	AC079922.2	0.535541058	1.87E-38	postive
PDHA1	AC080013.5	0.443511626	1.65E-25	postive
PDHA1	AC080112.2	0.428212424	1.03E-23	postive
PDHA1	AC080129.2	0.428057797	1.07E-23	postive
PDHA1	AC082651.1	0.353481871	3.67E-16	postive
PDHA1	AC083798.2	0.360208832	9.17E-17	postive
PDHA1	AC083799.1	0.502838086	2.13E-33	postive
PDHA1	AC083862.2	0.375779019	3.24E-18	postive
PDHA1	AC083880.1	0.455547414	5.48E-27	postive
PDHA1	AC083964.1	0.300310054	7.02E-12	postive
PDHA1	AC084018.1	0.322710176	1.40E-13	postive
PDHA1	AC084036.1	0.301106971	6.14E-12	postive
PDHA1	AC084125.2	0.357123007	1.74E-16	postive
PDHA1	AC084125.4	0.43510228	1.64E-24	postive
PDHA1	AC084757.3	0.346794972	1.41E-15	postive
PDHA1	AC087289.2	0.39743351	2.28E-20	postive
PDHA1	AC087379.2	0.372095879	7.27E-18	postive

PDHA1	AC087392.3	0.318594169	2.94E-13	postive
PDHA1	AC087392.4	0.30065032	6.63E-12	postive
PDHA1	AC087482.1	0.366325524	2.52E-17	postive
PDHA1	AC087500.1	0.335915841	1.18E-14	postive
PDHA1	AC087501.4	0.341392783	4.10E-15	postive
PDHA1	AC087521.3	0.337704385	8.39E-15	postive
PDHA1	AC087623.2	0.325135345	8.95E-14	postive
PDHA1	AC087645.2	0.345783665	1.73E-15	postive
PDHA1	AC087741.1	0.329038003	4.34E-14	postive
PDHA1	AC087741.2	0.470565898	6.46E-29	postive
PDHA1	AC087752.3	0.310974149	1.13E-12	postive
PDHA1	AC090114.2	0.421876771	5.37E-23	postive
PDHA1	AC090193.1	0.303738716	3.94E-12	postive
PDHA1	AC090198.1	0.306330352	2.53E-12	postive
PDHA1	AC090425.2	0.360811267	8.08E-17	postive
PDHA1	AC090425.3	0.321362207	1.78E-13	postive
PDHA1	AC090515.3	0.369672342	1.23E-17	postive
PDHA1	AC090517.2	0.306866172	2.31E-12	postive
PDHA1	AC090527.4	0.30466274	3.36E-12	postive
PDHA1	AC090587.1	0.509631226	2.10E-34	postive
PDHA1	AC090587.2	0.42380696	3.26E-23	postive
PDHA1	AC090589.3	0.31064875	1.20E-12	postive
PDHA1	AC090617.5	0.376044037	3.06E-18	postive
PDHA1	AC090825.1	0.3143585	6.25E-13	postive
PDHA1	AC090912.1	0.408729304	1.48E-21	postive
PDHA1	AC090970.2	0.479331673	4.36E-30	postive
PDHA1	AC091057.1	0.4312223	4.64E-24	postive
PDHA1	AC091114.1	0.36219468	6.05E-17	postive
PDHA1	AC091153.3	0.431724558	4.06E-24	postive
PDHA1	AC091153.4	0.386850031	2.69E-19	postive
PDHA1	AC091271.1	0.421112212	6.53E-23	postive
PDHA1	AC091390.5	0.341027842	4.40E-15	postive
PDHA1	AC091729.3	0.483368493	1.23E-30	postive
PDHA1	AC091965.1	0.307810955	1.96E-12	postive
PDHA1	AC091982.3	0.350697411	6.46E-16	postive
PDHA1	AC092115.4	0.436318983	1.18E-24	postive
PDHA1	AC092118.2	0.366748325	2.30E-17	postive
PDHA1	AC092119.2	0.34972658	7.86E-16	postive
PDHA1	AC092119.3	0.329365261	4.08E-14	postive
PDHA1	AC092123.1	0.312095027	9.31E-13	postive
PDHA1	AC092164.1	0.364366628	3.82E-17	postive
PDHA1	AC092171.2	0.310619172	1.21E-12	postive
PDHA1	AC092171.4	0.323305467	1.25E-13	postive
PDHA1	AC092171.5	0.328434794	4.86E-14	postive
PDHA1	AC092295.2	0.425739608	1.97E-23	postive
PDHA1	AC092301.1	0.321997944	1.59E-13	postive
PDHA1	AC092368.3	0.410370398	9.86E-22	postive
PDHA1	AC092431.1	0.419547027	9.76E-23	postive
PDHA1	AC092614.1	0.333775657	1.78E-14	postive
PDHA1	AC092634.3	0.333766083	1.78E-14	postive

PDHA1	AC092718.2	0.438085633	7.32E-25	postive
PDHA1	AC092718.4	0.464452587	4.04E-28	postive
PDHA1	AC092720.1	0.343289134	2.83E-15	postive
PDHA1	AC092803.1	0.53077234	1.10E-37	postive
PDHA1	AC092903.2	0.319847128	2.35E-13	postive
PDHA1	AC092910.3	0.44079669	3.49E-25	postive
PDHA1	AC092944.1	0.43222222	3.55E-24	postive
PDHA1	AC092969.1	0.365204299	3.20E-17	postive
PDHA1	AC093157.1	0.421527815	5.87E-23	postive
PDHA1	AC093249.2	0.403124005	5.83E-21	postive
PDHA1	AC093249.6	0.5166614	1.81E-35	postive
PDHA1	AC093297.2	0.316973106	3.93E-13	postive
PDHA1	AC093462.1	0.320160237	2.22E-13	postive
PDHA1	AC093484.4	0.33720564	9.23E-15	postive
PDHA1	AC093520.1	0.343246573	2.85E-15	postive
PDHA1	AC093620.1	0.310051903	1.33E-12	postive
PDHA1	AC093627.5	0.387453614	2.34E-19	postive
PDHA1	AC093673.1	0.353274903	3.83E-16	postive
PDHA1	AC093726.1	0.302578359	4.79E-12	postive
PDHA1	AC093752.1	0.333473685	1.88E-14	postive
PDHA1	AC093752.3	0.351297137	5.73E-16	postive
PDHA1	AC096536.2	0.354582581	2.93E-16	postive
PDHA1	AC096540.1	0.324740714	9.62E-14	postive
PDHA1	AC096543.2	0.317337027	3.68E-13	postive
PDHA1	AC096649.2	0.381076405	9.98E-19	postive
PDHA1	AC096677.1	0.414437559	3.57E-22	postive
PDHA1	AC096733.3	0.419598797	9.64E-23	postive
PDHA1	AC096772.6	0.32400031	1.10E-13	postive
PDHA1	AC096992.2	0.317983746	3.28E-13	postive
PDHA1	AC097358.2	0.324932041	9.29E-14	postive
PDHA1	AC097359.2	0.523889999	1.37E-36	postive
PDHA1	AC097376.3	0.364040667	4.10E-17	postive
PDHA1	AC097468.3	0.402427967	6.89E-21	postive
PDHA1	AC097532.2	0.304326681	3.56E-12	postive
PDHA1	AC097641.2	0.309337042	1.51E-12	postive
PDHA1	AC098484.1	0.358784627	1.23E-16	postive
PDHA1	AC098484.2	0.400322485	1.14E-20	postive
PDHA1	AC098487.1	0.341505814	4.01E-15	postive
PDHA1	AC098820.2	0.417035073	1.85E-22	postive
PDHA1	AC098934.1	0.309021719	1.59E-12	postive
PDHA1	AC099343.4	0.518150412	1.07E-35	postive
PDHA1	AC099518.1	0.331847588	2.56E-14	postive
PDHA1	AC099518.4	0.320041097	2.26E-13	postive
PDHA1	AC099522.2	0.462855908	6.49E-28	postive
PDHA1	AC099568.2	0.442078684	2.45E-25	postive
PDHA1	AC099778.1	0.471564927	4.77E-29	postive
PDHA1	AC099791.2	0.349737848	7.84E-16	postive
PDHA1	AC099811.3	0.330849933	3.09E-14	postive
PDHA1	AC099850.2	0.420828546	7.03E-23	postive
PDHA1	AC099850.4	0.415271134	2.89E-22	postive

PDHA1	AC100778.2	0.344530156	2.21E-15	postive
PDHA1	AC100778.3	0.323772749	1.15E-13	postive
PDHA1	AC100786.1	0.357864472	1.49E-16	postive
PDHA1	AC100803.2	0.476026437	1.22E-29	postive
PDHA1	AC100810.1	0.37519817	3.69E-18	postive
PDHA1	AC100861.1	0.413703629	4.29E-22	postive
PDHA1	AC102953.1	0.339436408	6.00E-15	postive
PDHA1	AC103760.1	0.300173597	7.18E-12	postive
PDHA1	AC103770.1	0.353645278	3.55E-16	postive
PDHA1	AC103855.2	0.34733935	1.27E-15	postive
PDHA1	AC103925.1	0.355083984	2.65E-16	postive
PDHA1	AC103974.1	0.303989872	3.77E-12	postive
PDHA1	AC104109.2	0.367658603	1.90E-17	postive
PDHA1	AC104187.1	0.377927519	2.02E-18	postive
PDHA1	AC104435.2	0.301280438	5.96E-12	postive
PDHA1	AC104561.1	0.355686324	2.34E-16	postive
PDHA1	AC104561.3	0.332813216	2.13E-14	postive
PDHA1	AC104564.3	0.339751208	5.65E-15	postive
PDHA1	AC104667.1	0.327033713	6.30E-14	postive
PDHA1	AC104667.2	0.369889671	1.17E-17	postive
PDHA1	AC104777.1	0.343585148	2.67E-15	postive
PDHA1	AC104794.3	0.372129892	7.21E-18	postive
PDHA1	AC105020.6	0.363103482	4.99E-17	postive
PDHA1	AC105036.3	0.424132837	2.99E-23	postive
PDHA1	AC105137.2	0.441232531	3.09E-25	postive
PDHA1	AC105206.2	0.304422719	3.50E-12	postive
PDHA1	AC105219.1	0.312955979	8.01E-13	postive
PDHA1	AC105285.1	0.409658781	1.18E-21	postive
PDHA1	AC106706.1	0.362178043	6.07E-17	postive
PDHA1	AC106779.1	0.338171954	7.66E-15	postive
PDHA1	AC106782.5	0.345645352	1.78E-15	postive
PDHA1	AC106795.1	0.538623487	5.83E-39	postive
PDHA1	AC106820.4	0.303203677	4.31E-12	postive
PDHA1	AC106820.5	0.324394769	1.03E-13	postive
PDHA1	AC106886.3	0.309462279	1.47E-12	postive
PDHA1	AC106897.2	0.315503074	5.10E-13	postive
PDHA1	AC107027.3	0.357782405	1.52E-16	postive
PDHA1	AC107032.2	0.386906566	2.66E-19	postive
PDHA1	AC107068.1	0.389523217	1.46E-19	postive
PDHA1	AC107081.2	0.340965411	4.46E-15	postive
PDHA1	AC107214.1	0.471615783	4.70E-29	postive
PDHA1	AC107214.2	0.337264163	9.13E-15	postive
PDHA1	AC107375.1	0.451830989	1.59E-26	postive
PDHA1	AC108047.1	0.398701205	1.69E-20	postive
PDHA1	AC108488.1	0.462944163	6.32E-28	postive
PDHA1	AC108860.2	0.447087947	6.08E-26	postive
PDHA1	AC109322.1	0.535893233	1.64E-38	postive
PDHA1	AC109347.1	0.491478076	9.13E-32	postive
PDHA1	AC109454.2	0.32140256	1.77E-13	postive
PDHA1	AC109460.1	0.408985496	1.39E-21	postive

PDHA1	AC109460.2	0.374395613	4.40E-18	postive
PDHA1	AC110079.1	0.388000474	2.07E-19	postive
PDHA1	AC110609.1	0.309714726	1.41E-12	postive
PDHA1	AC112220.2	0.388547046	1.82E-19	postive
PDHA1	AC112236.2	0.336662269	1.02E-14	postive
PDHA1	AC112484.1	0.312865844	8.13E-13	postive
PDHA1	AC112491.1	0.323439953	1.22E-13	postive
PDHA1	AC112694.1	0.374957893	3.89E-18	postive
PDHA1	AC112907.3	0.338768913	6.83E-15	postive
PDHA1	AC113382.1	0.359386099	1.09E-16	postive
PDHA1	AC114271.1	0.425935489	1.87E-23	postive
PDHA1	AC114956.2	0.34475192	2.12E-15	postive
PDHA1	AC115618.1	0.531926976	7.20E-38	postive
PDHA1	AC115618.2	0.48891824	2.09E-31	postive
PDHA1	AC116025.2	0.318824003	2.82E-13	postive
PDHA1	AC116036.2	0.350089723	7.31E-16	postive
PDHA1	AC116351.1	0.374599159	4.20E-18	postive
PDHA1	AC116407.2	0.305886859	2.73E-12	postive
PDHA1	AC116913.1	0.458988201	2.02E-27	postive
PDHA1	AC116914.2	0.338951054	6.59E-15	postive
PDHA1	AC118553.1	0.359244992	1.12E-16	postive
PDHA1	AC118755.2	0.31153057	1.03E-12	postive
PDHA1	AC120053.1	0.336312387	1.10E-14	postive
PDHA1	AC120114.1	0.322760177	1.38E-13	postive
PDHA1	AC120498.4	0.368666657	1.53E-17	postive
PDHA1	AC120498.9	0.339944264	5.44E-15	postive
PDHA1	AC121338.2	0.334508822	1.55E-14	postive
PDHA1	AC121761.1	0.320139167	2.23E-13	postive
PDHA1	AC121761.2	0.306915645	2.29E-12	postive
PDHA1	AC123768.2	0.318196929	3.16E-13	postive
PDHA1	AC124016.2	0.495918482	2.14E-32	postive
PDHA1	AC124248.1	0.360442941	8.73E-17	postive
PDHA1	AC124283.2	0.378907383	1.62E-18	postive
PDHA1	AC124784.1	0.318830737	2.82E-13	postive
PDHA1	AC124798.1	0.385907085	3.34E-19	postive
PDHA1	AC124944.2	0.368292643	1.65E-17	postive
PDHA1	AC124947.1	0.306299066	2.54E-12	postive
PDHA1	AC125257.1	0.475868571	1.28E-29	postive
PDHA1	AC125437.1	0.304948779	3.20E-12	postive
PDHA1	AC125494.1	0.461448852	9.83E-28	postive
PDHA1	AC125603.3	0.357593038	1.58E-16	postive
PDHA1	AC126283.1	0.336995694	9.61E-15	postive
PDHA1	AC126755.1	0.313827491	6.87E-13	postive
PDHA1	AC127024.6	0.317950651	3.30E-13	postive
PDHA1	AC127070.1	0.30931571	1.51E-12	postive
PDHA1	AC127537.1	0.308158426	1.85E-12	postive
PDHA1	AC128687.2	0.44140568	2.95E-25	postive
PDHA1	AC129510.1	0.308718526	1.68E-12	postive
PDHA1	AC130343.2	0.306468495	2.47E-12	postive
PDHA1	AC131009.3	0.455208569	6.04E-27	postive

PDHA1	AC131011.2	0.355952035	2.22E-16	postive
PDHA1	AC131159.2	0.310360168	1.26E-12	postive
PDHA1	AC131235.3	0.521817597	2.89E-36	postive
PDHA1	AC131235.4	0.414365423	3.64E-22	postive
PDHA1	AC132008.2	0.589096386	4.89E-48	postive
PDHA1	AC132192.2	0.425552097	2.07E-23	postive
PDHA1	AC132872.1	0.346731652	1.43E-15	postive
PDHA1	AC132872.2	0.327351614	5.94E-14	postive
PDHA1	AC132872.3	0.350363028	6.91E-16	postive
PDHA1	AC132938.3	0.361287596	7.31E-17	postive
PDHA1	AC132938.4	0.451203012	1.90E-26	postive
PDHA1	AC133528.2	0.549869516	7.51E-41	postive
PDHA1	AC133552.2	0.380514662	1.13E-18	postive
PDHA1	AC133552.5	0.359272722	1.11E-16	postive
PDHA1	AC133785.1	0.36415797	4.00E-17	postive
PDHA1	AC134878.2	0.345256684	1.92E-15	postive
PDHA1	AC135048.1	0.352614478	4.38E-16	postive
PDHA1	AC135048.3	0.305258191	3.04E-12	postive
PDHA1	AC135050.1	0.506202307	6.81E-34	postive
PDHA1	AC135050.5	0.358164843	1.40E-16	postive
PDHA1	AC135050.6	0.440928289	3.36E-25	postive
PDHA1	AC135178.4	0.393923499	5.22E-20	postive
PDHA1	AC135178.6	0.363577561	4.52E-17	postive
PDHA1	AC135586.2	0.306653717	2.39E-12	postive
PDHA1	AC136475.2	0.387567675	2.28E-19	postive
PDHA1	AC136604.3	0.333391685	1.91E-14	postive
PDHA1	AC137056.1	0.335477806	1.29E-14	postive
PDHA1	AC137630.1	0.32396227	1.11E-13	postive
PDHA1	AC138028.2	0.316534299	4.25E-13	postive
PDHA1	AC138035.1	0.377116224	2.41E-18	postive
PDHA1	AC138123.1	0.364628659	3.62E-17	postive
PDHA1	AC138207.8	0.300787376	6.48E-12	postive
PDHA1	AC138230.1	0.313469218	7.31E-13	postive
PDHA1	AC138696.2	0.451587282	1.71E-26	postive
PDHA1	AC138932.1	0.341160048	4.29E-15	postive
PDHA1	AC138956.2	0.334920299	1.43E-14	postive
PDHA1	AC139256.2	0.352698476	4.31E-16	postive
PDHA1	AC139256.3	0.391958155	8.27E-20	postive
PDHA1	AC139530.1	0.531985479	7.05E-38	postive
PDHA1	AC139768.1	0.468483571	1.21E-28	postive
PDHA1	AC139795.2	0.336966608	9.67E-15	postive
PDHA1	AC139887.1	0.302942008	4.51E-12	postive
PDHA1	AC139887.2	0.427438514	1.26E-23	postive
PDHA1	AC141586.1	0.431408823	4.41E-24	postive
PDHA1	AC142472.1	0.409882658	1.11E-21	postive
PDHA1	AC144652.1	0.384750531	4.35E-19	postive
PDHA1	AC144836.1	0.34144993	4.06E-15	postive
PDHA1	AC145207.5	0.333560355	1.85E-14	postive
PDHA1	AC145285.2	0.345553681	1.81E-15	postive
PDHA1	AC145285.6	0.424616461	2.64E-23	postive

PDHA1	AC145423.2	0.347909637	1.13E-15	postive
PDHA1	AC145423.3	0.364486914	3.73E-17	postive
PDHA1	AC174065.1	0.319317492	2.58E-13	postive
PDHA1	AC211433.2	0.314324016	6.29E-13	postive
PDHA1	AC231533.2	0.30754554	2.05E-12	postive
PDHA1	AC231981.1	0.413888066	4.10E-22	postive
PDHA1	AC232271.1	0.497962402	1.09E-32	postive
PDHA1	AC233280.1	0.41236203	6.01E-22	postive
PDHA1	AC233728.1	0.428740146	8.96E-24	postive
PDHA1	AC233992.3	0.369152	1.37E-17	postive
PDHA1	AC242426.2	0.329018041	4.36E-14	postive
PDHA1	AC243654.3	0.310409119	1.25E-12	postive
PDHA1	AC243964.3	0.303849398	3.86E-12	postive
PDHA1	AC243965.2	0.34080129	4.60E-15	postive
PDHA1	AC244090.1	0.364298742	3.88E-17	postive
PDHA1	AC244669.1	0.362418207	5.77E-17	postive
PDHA1	AC245052.4	0.355281845	2.54E-16	postive
PDHA1	AC245060.2	0.325663612	8.12E-14	postive
PDHA1	AC245140.1	0.348084631	1.09E-15	postive
PDHA1	AC245140.2	0.527816642	3.28E-37	postive
PDHA1	AC245452.1	0.328571584	4.74E-14	postive
PDHA1	AC253536.6	0.402524464	6.74E-21	postive
PDHA1	ACTN1-AS1	0.321693565	1.68E-13	postive
PDHA1	ACVR2B-AS1	0.520224194	5.11E-36	postive
PDHA1	AD000671.3	0.343220784	2.87E-15	postive
PDHA1	AD001527.1	0.307348136	2.12E-12	postive
PDHA1	ADNP-AS1	0.353701779	3.51E-16	postive
PDHA1	AF064858.11	0.307125193	2.21E-12	postive
PDHA1	AF129075.3	0.342098658	3.57E-15	postive
PDHA1	AF131216.4	0.36936124	1.31E-17	postive
PDHA1	AF196972.1	0.439348765	5.18E-25	postive
PDHA1	AF213884.3	0.310416981	1.25E-12	postive
PDHA1	AF274858.1	0.34085927	4.55E-15	postive
PDHA1	AF279873.4	0.34193438	3.69E-15	postive
PDHA1	AFAP1-AS1	0.300539795	6.76E-12	postive
PDHA1	AFDN-DT	0.311042804	1.12E-12	postive
PDHA1	AFG3L1P	0.487560754	3.23E-31	postive
PDHA1	AHSA2P	0.355421621	2.47E-16	postive
PDHA1	AJ009632.2	0.376417227	2.82E-18	postive
PDHA1	AL008718.2	0.361755045	6.63E-17	postive
PDHA1	AL008721.1	0.318598753	2.94E-13	postive
PDHA1	AL008726.1	0.301758533	5.50E-12	postive
PDHA1	AL008729.1	0.404247081	4.44E-21	postive
PDHA1	AL009179.1	0.40661893	2.49E-21	postive
PDHA1	AL020996.1	0.517162504	1.52E-35	postive
PDHA1	AL021707.3	0.310543946	1.22E-12	postive
PDHA1	AL021878.2	0.410116627	1.05E-21	postive
PDHA1	AL022069.1	0.42793049	1.11E-23	postive
PDHA1	AL022157.1	0.451260589	1.87E-26	postive
PDHA1	AL022311.1	0.348394146	1.03E-15	postive

PDHA1	AL022324.3	0.302433169	4.91E-12	postive
PDHA1	AL022328.2	0.341430211	4.07E-15	postive
PDHA1	AL022328.4	0.309657627	1.42E-12	postive
PDHA1	AL023284.4	0.3879454	2.09E-19	postive
PDHA1	AL023803.1	0.412158635	6.32E-22	postive
PDHA1	AL023803.2	0.300473948	6.83E-12	postive
PDHA1	AL023803.3	0.328402834	4.89E-14	postive
PDHA1	AL024498.1	0.419679246	9.44E-23	postive
PDHA1	AL024507.3	0.53701217	1.07E-38	postive
PDHA1	AL024508.1	0.360768891	8.15E-17	postive
PDHA1	AL031055.1	0.432360619	3.42E-24	postive
PDHA1	AL031123.1	0.313954363	6.71E-13	postive
PDHA1	AL031123.2	0.392815112	6.77E-20	postive
PDHA1	AL031186.1	0.407707744	1.90E-21	postive
PDHA1	AL031282.1	0.324154262	1.07E-13	postive
PDHA1	AL031282.2	0.339912886	5.47E-15	postive
PDHA1	AL031289.1	0.300397347	6.92E-12	postive
PDHA1	AL031429.1	0.355781223	2.29E-16	postive
PDHA1	AL031432.3	0.351766185	5.21E-16	postive
PDHA1	AL031600.1	0.347343909	1.27E-15	postive
PDHA1	AL031714.1	0.386626318	2.83E-19	postive
PDHA1	AL031722.1	0.321326018	1.79E-13	postive
PDHA1	AL031775.1	0.363544823	4.55E-17	postive
PDHA1	AL031775.2	0.322024792	1.58E-13	postive
PDHA1	AL031847.1	0.33822701	7.58E-15	postive
PDHA1	AL031963.2	0.402219052	7.25E-21	postive
PDHA1	AL031963.3	0.454031229	8.48E-27	postive
PDHA1	AL031985.3	0.325616571	8.19E-14	postive
PDHA1	AL033384.2	0.349507305	8.22E-16	postive
PDHA1	AL033527.3	0.361229946	7.40E-17	postive
PDHA1	AL033528.3	0.321170685	1.85E-13	postive
PDHA1	AL035071.1	0.306925481	2.28E-12	postive
PDHA1	AL035252.4	0.345891959	1.69E-15	postive
PDHA1	AL035461.3	0.378885624	1.63E-18	postive
PDHA1	AL035587.1	0.44137516	2.97E-25	postive
PDHA1	AL049597.2	0.373445675	5.41E-18	postive
PDHA1	AL049646.2	0.305399619	2.97E-12	postive
PDHA1	AL049780.1	0.30430198	3.58E-12	postive
PDHA1	AL049794.1	0.362190008	6.05E-17	postive
PDHA1	AL049795.1	0.313843518	6.85E-13	postive
PDHA1	AL049796.1	0.426565727	1.59E-23	postive
PDHA1	AL049840.4	0.372022306	7.38E-18	postive
PDHA1	AL049840.5	0.365806491	2.82E-17	postive
PDHA1	AL049840.6	0.363809241	4.30E-17	postive
PDHA1	AL050309.1	0.476620302	1.01E-29	postive
PDHA1	AL050341.2	0.399980385	1.24E-20	postive
PDHA1	AL078581.2	0.343194043	2.88E-15	postive
PDHA1	AL078587.1	0.321876037	1.62E-13	postive
PDHA1	AL078590.2	0.38442619	4.68E-19	postive
PDHA1	AL078644.2	0.447580807	5.29E-26	postive

PDHA1	AL080250.1	0.350270394	7.05E-16	postive
PDHA1	AL080276.2	0.399480977	1.40E-20	postive
PDHA1	AL096828.3	0.458219325	2.53E-27	postive
PDHA1	AL109811.1	0.372449761	6.73E-18	postive
PDHA1	AL109917.1	0.515171367	3.06E-35	postive
PDHA1	AL109918.1	0.329735692	3.81E-14	postive
PDHA1	AL109945.1	0.303108583	4.38E-12	postive
PDHA1	AL109955.1	0.316643376	4.17E-13	postive
PDHA1	AL110115.2	0.309797416	1.39E-12	postive
PDHA1	AL117209.1	0.379907378	1.30E-18	postive
PDHA1	AL117332.1	0.542107733	1.54E-39	postive
PDHA1	AL117336.2	0.438137806	7.21E-25	postive
PDHA1	AL117379.1	0.322300677	1.50E-13	postive
PDHA1	AL118506.1	0.54217815	1.50E-39	postive
PDHA1	AL118511.1	0.349328316	8.52E-16	postive
PDHA1	AL118516.1	0.409453433	1.24E-21	postive
PDHA1	AL118523.1	0.318417967	3.03E-13	postive
PDHA1	AL118558.1	0.329333988	4.11E-14	postive
PDHA1	AL118558.3	0.555388881	8.35E-42	postive
PDHA1	AL118558.4	0.388643451	1.78E-19	postive
PDHA1	AL121574.1	0.450748357	2.16E-26	postive
PDHA1	AL121601.1	0.476329169	1.11E-29	postive
PDHA1	AL121603.2	0.409844154	1.12E-21	postive
PDHA1	AL121658.1	0.348115555	1.09E-15	postive
PDHA1	AL121832.2	0.511263403	1.20E-34	postive
PDHA1	AL121832.3	0.338790047	6.80E-15	postive
PDHA1	AL121890.4	0.328902252	4.45E-14	postive
PDHA1	AL121894.2	0.313070299	7.85E-13	postive
PDHA1	AL121917.1	0.308222339	1.83E-12	postive
PDHA1	AL121944.2	0.553735996	1.62E-41	postive
PDHA1	AL121970.1	0.378419109	1.81E-18	postive
PDHA1	AL121992.1	0.36912223	1.38E-17	postive
PDHA1	AL122010.1	0.446626423	6.92E-26	postive
PDHA1	AL122023.3	0.350159736	7.20E-16	postive
PDHA1	AL122035.2	0.343908272	2.50E-15	postive
PDHA1	AL132639.2	0.31913649	2.67E-13	postive
PDHA1	AL132639.3	0.391717452	8.75E-20	postive
PDHA1	AL132712.1	0.416784679	1.97E-22	postive
PDHA1	AL132780.1	0.380140122	1.23E-18	postive
PDHA1	AL132780.2	0.336716081	1.01E-14	postive
PDHA1	AL133153.2	0.362920632	5.19E-17	postive
PDHA1	AL133215.2	0.49456344	3.34E-32	postive
PDHA1	AL133216.2	0.375358661	3.56E-18	postive
PDHA1	AL133338.1	0.413734009	4.26E-22	postive
PDHA1	AL133367.1	0.39032204	1.21E-19	postive
PDHA1	AL133406.2	0.324959167	9.24E-14	postive
PDHA1	AL133410.1	0.30841698	1.77E-12	postive
PDHA1	AL133520.1	0.345218215	1.93E-15	postive
PDHA1	AL133523.1	0.302395485	4.94E-12	postive
PDHA1	AL133551.1	0.320351595	2.14E-13	postive

PDHA1	AL135744.1	0.502238407	2.61E-33	postive
PDHA1	AL135791.1	0.348637431	9.78E-16	postive
PDHA1	AL135925.1	0.396777375	2.66E-20	postive
PDHA1	AL135999.1	0.320806578	1.97E-13	postive
PDHA1	AL135999.2	0.317538867	3.55E-13	postive
PDHA1	AL136040.1	0.349529928	8.18E-16	postive
PDHA1	AL136114.1	0.321112706	1.87E-13	postive
PDHA1	AL136162.1	0.343958423	2.48E-15	postive
PDHA1	AL136295.2	0.347347402	1.27E-15	postive
PDHA1	AL136295.6	0.388061265	2.04E-19	postive
PDHA1	AL136295.7	0.415928129	2.45E-22	postive
PDHA1	AL136304.1	0.349283921	8.59E-16	postive
PDHA1	AL136366.1	0.344707673	2.14E-15	postive
PDHA1	AL136531.1	0.387657016	2.24E-19	postive
PDHA1	AL137058.2	0.338783962	6.81E-15	postive
PDHA1	AL137060.3	0.310122345	1.31E-12	postive
PDHA1	AL137127.1	0.365690437	2.89E-17	postive
PDHA1	AL137186.2	0.433447502	2.56E-24	postive
PDHA1	AL137802.2	0.342106101	3.57E-15	postive
PDHA1	AL138724.2	0.36737846	2.01E-17	postive
PDHA1	AL138756.1	0.306477636	2.47E-12	postive
PDHA1	AL138762.2	0.331542923	2.71E-14	postive
PDHA1	AL138831.1	0.390796663	1.08E-19	postive
PDHA1	AL138831.2	0.354402198	3.04E-16	postive
PDHA1	AL138921.1	0.393383259	5.93E-20	postive
PDHA1	AL138921.2	0.301190062	6.06E-12	postive
PDHA1	AL138976.2	0.314816815	5.76E-13	postive
PDHA1	AL139011.1	0.440611305	3.67E-25	postive
PDHA1	AL139035.1	0.458380109	2.41E-27	postive
PDHA1	AL139039.3	0.398532936	1.76E-20	postive
PDHA1	AL139089.1	0.382742848	6.85E-19	postive
PDHA1	AL139123.1	0.327975741	5.29E-14	postive
PDHA1	AL139246.5	0.353875729	3.39E-16	postive
PDHA1	AL139260.1	0.521749655	2.96E-36	postive
PDHA1	AL139274.2	0.338756084	6.85E-15	postive
PDHA1	AL139287.1	0.389691272	1.40E-19	postive
PDHA1	AL139289.1	0.420365951	7.92E-23	postive
PDHA1	AL139383.1	0.336232686	1.11E-14	postive
PDHA1	AL139384.1	0.462680947	6.84E-28	postive
PDHA1	AL139397.1	0.317227339	3.75E-13	postive
PDHA1	AL139423.1	0.350922171	6.18E-16	postive
PDHA1	AL157373.2	0.370610209	1.00E-17	postive
PDHA1	AL157400.2	0.313726336	6.99E-13	postive
PDHA1	AL157832.1	0.311012974	1.13E-12	postive
PDHA1	AL157871.5	0.310496505	1.23E-12	postive
PDHA1	AL157904.1	0.394889499	4.16E-20	postive
PDHA1	AL158154.3	0.304422879	3.50E-12	postive
PDHA1	AL158212.2	0.348389514	1.03E-15	postive
PDHA1	AL158212.3	0.345187655	1.94E-15	postive
PDHA1	AL158835.2	0.358284818	1.37E-16	postive

PDHA1	AL160006.1	0.402276588	7.15E-21	postive
PDHA1	AL160286.2	0.323484691	1.21E-13	postive
PDHA1	AL160291.1	0.333768382	1.78E-14	postive
PDHA1	AL160313.2	0.361035929	7.71E-17	postive
PDHA1	AL161421.1	0.334165222	1.65E-14	postive
PDHA1	AL161452.1	0.434227694	2.08E-24	postive
PDHA1	AL161640.2	0.338978553	6.56E-15	postive
PDHA1	AL161668.3	0.471650533	4.65E-29	postive
PDHA1	AL161719.1	0.312882289	8.11E-13	postive
PDHA1	AL161729.1	0.376579538	2.72E-18	postive
PDHA1	AL161729.3	0.324793641	9.53E-14	postive
PDHA1	AL161729.4	0.352633142	4.37E-16	postive
PDHA1	AL161772.1	0.344035405	2.44E-15	postive
PDHA1	AL161937.1	0.439022491	5.67E-25	postive
PDHA1	AL162231.2	0.344866476	2.07E-15	postive
PDHA1	AL162258.2	0.341650994	3.90E-15	postive
PDHA1	AL162274.2	0.372560006	6.57E-18	postive
PDHA1	AL162377.1	0.448697377	3.87E-26	postive
PDHA1	AL162431.1	0.303038719	4.43E-12	postive
PDHA1	AL162595.1	0.361777157	6.60E-17	postive
PDHA1	AL162632.3	0.309525043	1.46E-12	postive
PDHA1	AL162741.1	0.31084581	1.16E-12	postive
PDHA1	AL163051.1	0.347194535	1.31E-15	postive
PDHA1	AL353194.1	0.44653934	7.09E-26	postive
PDHA1	AL353593.1	0.310792547	1.17E-12	postive
PDHA1	AL353596.1	0.300173315	7.18E-12	postive
PDHA1	AL353625.1	0.370752855	9.73E-18	postive
PDHA1	AL353708.1	0.388411306	1.88E-19	postive
PDHA1	AL353708.3	0.387164332	2.51E-19	postive
PDHA1	AL353743.1	0.453704437	9.31E-27	postive
PDHA1	AL353795.2	0.333271941	1.96E-14	postive
PDHA1	AL353796.1	0.339799146	5.59E-15	postive
PDHA1	AL353801.3	0.308404365	1.77E-12	postive
PDHA1	AL353807.2	0.340327458	5.05E-15	postive
PDHA1	AL354696.1	0.348898216	9.28E-16	postive
PDHA1	AL354696.2	0.403307133	5.57E-21	postive
PDHA1	AL354707.1	0.339600181	5.81E-15	postive
PDHA1	AL354718.3	0.34200157	3.64E-15	postive
PDHA1	AL354733.3	0.338520949	7.17E-15	postive
PDHA1	AL354740.1	0.447379256	5.60E-26	postive
PDHA1	AL354766.2	0.300735214	6.54E-12	postive
PDHA1	AL354824.1	0.343636677	2.64E-15	postive
PDHA1	AL354824.2	0.347438582	1.24E-15	postive
PDHA1	AL354836.1	0.34356335	2.68E-15	postive
PDHA1	AL354864.1	0.31070761	1.19E-12	postive
PDHA1	AL354892.2	0.538366965	6.42E-39	postive
PDHA1	AL354892.3	0.359196216	1.13E-16	postive
PDHA1	AL354920.1	0.388992031	1.65E-19	postive
PDHA1	AL354928.1	0.369609368	1.25E-17	postive
PDHA1	AL355001.2	0.431191213	4.68E-24	postive

PDHA1	AL355297.3	0.392413198	7.44E-20	postive
PDHA1	AL355300.1	0.357644399	1.56E-16	postive
PDHA1	AL355312.2	0.308615368	1.71E-12	postive
PDHA1	AL355312.3	0.338690995	6.93E-15	postive
PDHA1	AL355353.2	0.409859303	1.12E-21	postive
PDHA1	AL355385.1	0.366302909	2.53E-17	postive
PDHA1	AL355388.1	0.335604736	1.26E-14	postive
PDHA1	AL355490.3	0.307939333	1.92E-12	postive
PDHA1	AL355574.1	0.470500563	6.59E-29	postive
PDHA1	AL355607.2	0.341128218	4.32E-15	postive
PDHA1	AL355802.3	0.455386586	5.74E-27	postive
PDHA1	AL356056.2	0.317900154	3.33E-13	postive
PDHA1	AL356235.1	0.326133444	7.44E-14	postive
PDHA1	AL356299.3	0.432934257	2.94E-24	postive
PDHA1	AL356481.1	0.410592063	9.33E-22	postive
PDHA1	AL356481.3	0.32866052	4.66E-14	postive
PDHA1	AL356488.3	0.398603485	1.73E-20	postive
PDHA1	AL356512.1	0.38481517	4.29E-19	postive
PDHA1	AL356599.1	0.378232951	1.88E-18	postive
PDHA1	AL356966.1	0.347530412	1.22E-15	postive
PDHA1	AL357033.2	0.372361689	6.86E-18	postive
PDHA1	AL357033.3	0.312498079	8.68E-13	postive
PDHA1	AL357033.4	0.315396869	5.20E-13	postive
PDHA1	AL357079.1	0.412517997	5.78E-22	postive
PDHA1	AL357140.2	0.410966261	8.50E-22	postive
PDHA1	AL357146.1	0.305333617	3.00E-12	postive
PDHA1	AL357874.1	0.342166601	3.53E-15	postive
PDHA1	AL357992.1	0.302119099	5.18E-12	postive
PDHA1	AL358115.1	0.35999549	9.58E-17	postive
PDHA1	AL358472.2	0.5050054	1.02E-33	postive
PDHA1	AL358472.3	0.38549921	3.67E-19	postive
PDHA1	AL358852.1	0.445210294	1.03E-25	postive
PDHA1	AL358933.1	0.318064441	3.23E-13	postive
PDHA1	AL359091.3	0.369259242	1.34E-17	postive
PDHA1	AL359182.1	0.322791234	1.37E-13	postive
PDHA1	AL359198.1	0.35507885	2.65E-16	postive
PDHA1	AL359317.1	0.324728912	9.64E-14	postive
PDHA1	AL359397.1	0.358775594	1.24E-16	postive
PDHA1	AL359504.2	0.306219904	2.58E-12	postive
PDHA1	AL359513.1	0.396502982	2.84E-20	postive
PDHA1	AL359541.2	0.340525144	4.86E-15	postive
PDHA1	AL359643.3	0.425180144	2.28E-23	postive
PDHA1	AL359711.2	0.409016813	1.38E-21	postive
PDHA1	AL359715.1	0.347808819	1.15E-15	postive
PDHA1	AL359715.3	0.399387677	1.43E-20	postive
PDHA1	AL359878.1	0.345975748	1.66E-15	postive
PDHA1	AL359921.1	0.370529074	1.02E-17	postive
PDHA1	AL359921.2	0.406170317	2.78E-21	postive
PDHA1	AL359962.1	0.446346708	7.48E-26	postive
PDHA1	AL359962.2	0.336418291	1.07E-14	postive

PDHA1	AL360181.2	0.376374784	2.84E-18	postive
PDHA1	AL365330.1	0.371600242	8.09E-18	postive
PDHA1	AL390728.4	0.369352948	1.32E-17	postive
PDHA1	AL390879.1	0.301969774	5.31E-12	postive
PDHA1	AL390955.2	0.41945097	1.00E-22	postive
PDHA1	AL391069.2	0.415439618	2.77E-22	postive
PDHA1	AL391069.3	0.387703234	2.21E-19	postive
PDHA1	AL391121.1	0.318407649	3.04E-13	postive
PDHA1	AL391244.1	0.311687066	1.00E-12	postive
PDHA1	AL391244.2	0.375357222	3.56E-18	postive
PDHA1	AL391684.1	0.376010862	3.08E-18	postive
PDHA1	AL391825.1	0.328634789	4.68E-14	postive
PDHA1	AL391832.2	0.340490181	4.89E-15	postive
PDHA1	AL391832.3	0.375000597	3.85E-18	postive
PDHA1	AL391834.2	0.321639203	1.70E-13	postive
PDHA1	AL392172.1	0.323760928	1.15E-13	postive
PDHA1	AL441992.2	0.508595093	3.00E-34	postive
PDHA1	AL442128.2	0.327395362	5.89E-14	postive
PDHA1	AL442663.3	0.442397806	2.24E-25	postive
PDHA1	AL445222.2	0.328779191	4.56E-14	postive
PDHA1	AL445228.3	0.396083185	3.14E-20	postive
PDHA1	AL445231.1	0.358807992	1.23E-16	postive
PDHA1	AL445645.1	0.337568719	8.61E-15	postive
PDHA1	AL450326.1	0.397790465	2.10E-20	postive
PDHA1	AL450998.2	0.481889528	1.96E-30	postive
PDHA1	AL450998.3	0.320189283	2.21E-13	postive
PDHA1	AL451050.2	0.530354159	1.29E-37	postive
PDHA1	AL451069.3	0.361279677	7.33E-17	postive
PDHA1	AL451074.2	0.301602069	5.65E-12	postive
PDHA1	AL451085.2	0.401575052	8.47E-21	postive
PDHA1	AL451085.3	0.517097392	1.55E-35	postive
PDHA1	AL451165.2	0.462763241	6.67E-28	postive
PDHA1	AL512408.1	0.38361275	5.63E-19	postive
PDHA1	AL512598.1	0.369782869	1.20E-17	postive
PDHA1	AL512625.1	0.317170449	3.79E-13	postive
PDHA1	AL512625.2	0.36986308	1.18E-17	postive
PDHA1	AL512770.1	0.403204743	5.71E-21	postive
PDHA1	AL512791.2	0.322555893	1.44E-13	postive
PDHA1	AL513165.1	0.352411848	4.57E-16	postive
PDHA1	AL513218.1	0.339656374	5.75E-15	postive
PDHA1	AL513320.1	0.331446625	2.76E-14	postive
PDHA1	AL513550.1	0.46571713	2.78E-28	postive
PDHA1	AL583810.1	0.346911197	1.38E-15	postive
PDHA1	AL589765.4	0.356652678	1.92E-16	postive
PDHA1	AL590096.1	0.417741128	1.55E-22	postive
PDHA1	AL590133.1	0.380190906	1.22E-18	postive
PDHA1	AL590369.1	0.437517303	8.54E-25	postive
PDHA1	AL590428.1	0.352495863	4.49E-16	postive
PDHA1	AL590617.2	0.427456581	1.26E-23	postive
PDHA1	AL590705.1	0.314371041	6.24E-13	postive

PDHA1	AL591684.2	0.347213346	1.30E-15	postive
PDHA1	AL592071.1	0.338337197	7.42E-15	postive
PDHA1	AL592166.1	0.447210885	5.87E-26	postive
PDHA1	AL592211.1	0.375085273	3.78E-18	postive
PDHA1	AL592435.1	0.393013971	6.46E-20	postive
PDHA1	AL592494.2	0.413593929	4.41E-22	postive
PDHA1	AL596094.1	0.446271408	7.64E-26	postive
PDHA1	AL596202.1	0.391936336	8.31E-20	postive
PDHA1	AL603832.1	0.322429209	1.47E-13	postive
PDHA1	AL603839.2	0.45403523	8.47E-27	postive
PDHA1	AL603839.3	0.359858001	9.86E-17	postive
PDHA1	AL604028.1	0.403931787	4.79E-21	postive
PDHA1	AL606489.1	0.436061165	1.27E-24	postive
PDHA1	AL606491.1	0.37814777	1.92E-18	postive
PDHA1	AL606760.2	0.393646483	5.57E-20	postive
PDHA1	AL606834.1	0.358379459	1.34E-16	postive
PDHA1	AL606834.2	0.304609016	3.40E-12	postive
PDHA1	AL645568.1	0.458234072	2.52E-27	postive
PDHA1	AL645940.1	0.315227447	5.36E-13	postive
PDHA1	AL645941.1	0.350863903	6.25E-16	postive
PDHA1	AL662791.1	0.316978244	3.93E-13	postive
PDHA1	AL662797.2	0.409517078	1.22E-21	postive
PDHA1	AL662844.4	0.355160476	2.61E-16	postive
PDHA1	AL669831.1	0.339097045	6.41E-15	postive
PDHA1	AL671710.1	0.464980448	3.46E-28	postive
PDHA1	AL691403.2	0.325752933	7.99E-14	postive
PDHA1	AL691432.1	0.357171654	1.72E-16	postive
PDHA1	AL691432.2	0.34177163	3.81E-15	postive
PDHA1	AL731533.2	0.388587814	1.81E-19	postive
PDHA1	AL731566.2	0.358914515	1.20E-16	postive
PDHA1	AL731569.1	0.469608095	8.63E-29	postive
PDHA1	AL731571.1	0.410074687	1.06E-21	postive
PDHA1	AL731577.2	0.428081369	1.07E-23	postive
PDHA1	AL732314.8	0.531926964	7.20E-38	postive
PDHA1	AL732372.2	0.377393146	2.27E-18	postive
PDHA1	AL807752.5	0.310774184	1.17E-12	postive
PDHA1	AL928654.1	0.423309656	3.71E-23	postive
PDHA1	AL928654.2	0.377282093	2.33E-18	postive
PDHA1	ALDH1L1-AS2	0.397004067	2.53E-20	postive
PDHA1	ALG1L13P	0.498758741	8.35E-33	postive
PDHA1	ALMS1-IT1	0.317775026	3.40E-13	postive
PDHA1	ALOX12-AS1	0.493954123	4.07E-32	postive
PDHA1	ALOX12P2	0.303911168	3.82E-12	postive
PDHA1	AMZ2P1	0.471567344	4.77E-29	postive
PDHA1	ANO7L1	0.40623009	2.74E-21	postive
PDHA1	AP000251.1	0.421197553	6.39E-23	postive
PDHA1	AP000253.1	0.410560425	9.41E-22	postive
PDHA1	AP000254.2	0.384334344	4.78E-19	postive
PDHA1	AP000295.10	0.320785247	1.98E-13	postive
PDHA1	AP000350.5	0.362066261	6.21E-17	postive

PDHA1	AP000442.1	0.498346573	9.57E-33	postive
PDHA1	AP000487.1	0.444726503	1.18E-25	postive
PDHA1	AP000525.1	0.340245144	5.13E-15	postive
PDHA1	AP000640.1	0.331389228	2.79E-14	postive
PDHA1	AP000640.2	0.364713982	3.55E-17	postive
PDHA1	AP000704.2	0.35586701	2.25E-16	postive
PDHA1	AP000759.1	0.423672545	3.37E-23	postive
PDHA1	AP000769.1	0.401171561	9.33E-21	postive
PDHA1	AP000781.1	0.37555187	3.41E-18	postive
PDHA1	AP000808.1	0.30153086	5.72E-12	postive
PDHA1	AP000845.1	0.341326052	4.16E-15	postive
PDHA1	AP000873.2	0.362448436	5.73E-17	postive
PDHA1	AP000894.4	0.396124436	3.11E-20	postive
PDHA1	AP001063.1	0.362862243	5.25E-17	postive
PDHA1	AP001107.1	0.443284099	1.76E-25	postive
PDHA1	AP001160.1	0.457706986	2.93E-27	postive
PDHA1	AP001160.2	0.40053588	1.09E-20	postive
PDHA1	AP001160.3	0.496255694	1.91E-32	postive
PDHA1	AP001178.3	0.305748212	2.80E-12	postive
PDHA1	AP001189.5	0.366207264	2.58E-17	postive
PDHA1	AP001267.1	0.360204829	9.17E-17	postive
PDHA1	AP001267.3	0.373670888	5.15E-18	postive
PDHA1	AP001318.2	0.388637746	1.79E-19	postive
PDHA1	AP001351.1	0.315453849	5.15E-13	postive
PDHA1	AP001372.2	0.409729542	1.16E-21	postive
PDHA1	AP001412.1	0.396734202	2.69E-20	postive
PDHA1	AP001453.2	0.426648833	1.55E-23	postive
PDHA1	AP001453.4	0.409304475	1.28E-21	postive
PDHA1	AP001462.1	0.415521975	2.72E-22	postive
PDHA1	AP001469.2	0.32853711	4.77E-14	postive
PDHA1	AP001469.3	0.397326518	2.34E-20	postive
PDHA1	AP001505.1	0.416629279	2.05E-22	postive
PDHA1	AP001527.2	0.461459437	9.80E-28	postive
PDHA1	AP001542.3	0.346829629	1.40E-15	postive
PDHA1	AP001816.1	0.357918307	1.48E-16	postive
PDHA1	AP001830.1	0.314850855	5.73E-13	postive
PDHA1	AP002008.3	0.362512924	5.65E-17	postive
PDHA1	AP002026.1	0.337352724	8.98E-15	postive
PDHA1	AP002360.2	0.480149152	3.38E-30	postive
PDHA1	AP002387.2	0.51610506	2.20E-35	postive
PDHA1	AP002748.4	0.497372189	1.32E-32	postive
PDHA1	AP002807.1	0.409393221	1.26E-21	postive
PDHA1	AP002992.1	0.344449354	2.25E-15	postive
PDHA1	AP003068.1	0.39889899	1.61E-20	postive
PDHA1	AP003068.2	0.412693835	5.53E-22	postive
PDHA1	AP003096.1	0.338115464	7.75E-15	postive
PDHA1	AP003119.2	0.433917454	2.26E-24	postive
PDHA1	AP003352.1	0.40175676	8.11E-21	postive
PDHA1	AP003392.4	0.369228926	1.35E-17	postive
PDHA1	AP003392.5	0.364139249	4.01E-17	postive

PDHA1	AP003419.3	0.312892651	8.10E-13	postive
PDHA1	AP003469.1	0.318761536	2.85E-13	postive
PDHA1	AP003469.2	0.368902417	1.45E-17	postive
PDHA1	AP003469.3	0.302955297	4.50E-12	postive
PDHA1	AP003486.1	0.328016617	5.25E-14	postive
PDHA1	AP003498.1	0.306834343	2.32E-12	postive
PDHA1	AP003680.1	0.313804989	6.89E-13	postive
PDHA1	AP003721.4	0.324358754	1.03E-13	postive
PDHA1	AP004609.5	0.415132842	3.00E-22	postive
PDHA1	AP005061.2	0.334586208	1.52E-14	postive
PDHA1	AP005329.1	0.323864748	1.13E-13	postive
PDHA1	AP005432.2	0.320037476	2.27E-13	postive
PDHA1	AP005482.4	0.37023703	1.09E-17	postive
PDHA1	AP006216.1	0.354282979	3.12E-16	postive
PDHA1	AP006621.2	0.344360113	2.29E-15	postive
PDHA1	AP006621.3	0.556908214	4.53E-42	postive
PDHA1	APTR	0.35681207	1.86E-16	postive
PDHA1	ARHGAP31-AS1	0.348043734	1.10E-15	postive
PDHA1	ARHGAP5-AS1	0.336245945	1.11E-14	postive
PDHA1	ARHGEF35-AS1	0.411914171	6.72E-22	postive
PDHA1	ARMCX5-GPRASP2	0.496851376	1.57E-32	postive
PDHA1	ARRDC1-AS1	0.637188457	2.62E-58	postive
PDHA1	ASB16-AS1	0.509373047	2.30E-34	postive
PDHA1	ASH1L-AS1	0.486782276	4.15E-31	postive
PDHA1	ASMTL-AS1	0.381387297	9.30E-19	postive
PDHA1	ATF4P4	0.359550104	1.05E-16	postive
PDHA1	ATP2A1-AS1	0.419217212	1.06E-22	postive
PDHA1	ATP2B1-AS1	0.346356726	1.54E-15	postive
PDHA1	ATP6V0E2-AS1	0.370126186	1.11E-17	postive
PDHA1	ATXN1-AS1	0.331989926	2.49E-14	postive
PDHA1	ATXN2-AS	0.494602023	3.29E-32	postive
PDHA1	BAALC-AS1	0.352743271	4.27E-16	postive
PDHA1	BACE1-AS	0.426530176	1.60E-23	postive
PDHA1	BAIAP2-DT	0.320448263	2.10E-13	postive
PDHA1	BCAR3-AS1	0.318576499	2.95E-13	postive
PDHA1	BCDIN3D-AS1	0.465439338	3.02E-28	postive
PDHA1	BMS1P20	0.419517277	9.84E-23	postive
PDHA1	BMS1P4	0.302772527	4.64E-12	postive
PDHA1	BOLA2P2	0.300289375	7.04E-12	postive
PDHA1	BOLA3-AS1	0.34639278	1.53E-15	postive
PDHA1	BRWD1-AS2	0.36936341	1.31E-17	postive
PDHA1	BTG3-AS1	0.385959886	3.30E-19	postive
PDHA1	BTN2A3P	0.453714807	9.29E-27	postive
PDHA1	BVES-AS1	0.351882661	5.09E-16	postive
PDHA1	BX284668.5	0.318768799	2.85E-13	postive
PDHA1	BX293535.1	0.310953602	1.14E-12	postive
PDHA1	BX322557.10	0.332902606	2.10E-14	postive
PDHA1	BX322639.1	0.312674366	8.41E-13	postive
PDHA1	BX470209.1	0.395392673	3.70E-20	postive
PDHA1	BX470209.2	0.37294962	6.03E-18	postive

PDHA1	BX537318.1	0.406461161	2.58E-21	postive
PDHA1	BX539320.1	0.340734131	4.66E-15	postive
PDHA1	BX649601.1	0.401126572	9.43E-21	postive
PDHA1	BX649632.1	0.407375459	2.07E-21	postive
PDHA1	C10orf25	0.361526277	6.96E-17	postive
PDHA1	C15orf56	0.31762439	3.50E-13	postive
PDHA1	C1orf147	0.356839192	1.85E-16	postive
PDHA1	C1orf220	0.342715503	3.17E-15	postive
PDHA1	C1orf229	0.464305605	4.23E-28	postive
PDHA1	C1QTNF1-AS1	0.319622911	2.44E-13	postive
PDHA1	C1RL-AS1	0.324512508	1.00E-13	postive
PDHA1	C22orf24	0.392756407	6.86E-20	postive
PDHA1	C22orf46	0.429488817	7.35E-24	postive
PDHA1	C2-AS1	0.333762465	1.78E-14	postive
PDHA1	C2CD4D-AS1	0.342860772	3.08E-15	postive
PDHA1	C2orf48	0.318881714	2.79E-13	postive
PDHA1	C5orf66	0.371912784	7.56E-18	postive
PDHA1	C6orf99	0.420229457	8.20E-23	postive
PDHA1	C9orf147	0.386031651	3.25E-19	postive
PDHA1	C9orf163	0.422743133	4.29E-23	postive
PDHA1	C9orf170	0.303887388	3.84E-12	postive
PDHA1	CA5BP1	0.641821713	2.14E-59	postive
PDHA1	CAHM	0.475430952	1.46E-29	postive
PDHA1	CAMTA1-DT	0.463761457	4.97E-28	postive
PDHA1	CAPN10-DT	0.450497586	2.32E-26	postive
PDHA1	CASK-AS1	0.315328941	5.26E-13	postive
PDHA1	CATIP-AS1	0.301465829	5.78E-12	postive
PDHA1	CATSPER2P1	0.441656951	2.75E-25	postive
PDHA1	CCDC183-AS1	0.407061326	2.23E-21	postive
PDHA1	CCDC84-DT	0.365360015	3.10E-17	postive
PDHA1	CCNT2-AS1	0.445037894	1.08E-25	postive
PDHA1	CCT6P1	0.433156133	2.77E-24	postive
PDHA1	CCT6P3	0.322326061	1.50E-13	postive
PDHA1	CD27-AS1	0.465390896	3.06E-28	postive
PDHA1	CD99P1	0.41692243	1.91E-22	postive
PDHA1	CDC37L1-DT	0.401274846	9.10E-21	postive
PDHA1	CEACAMP10	0.326043145	7.57E-14	postive
PDHA1	CEBPB-AS1	0.466307145	2.33E-28	postive
PDHA1	CENPBD1P1	0.475893111	1.27E-29	postive
PDHA1	CEP83-DT	0.432904027	2.96E-24	postive
PDHA1	CFL1P1	0.310463444	1.24E-12	postive
PDHA1	CHKB-DT	0.383855417	5.33E-19	postive
PDHA1	CHMP1B2P	0.31337749	7.43E-13	postive
PDHA1	CHROMR	0.349465755	8.28E-16	postive
PDHA1	CIDECP1	0.588171448	7.41E-48	postive
PDHA1	CKMT2-AS1	0.444862539	1.13E-25	postive
PDHA1	CNNM3-DT	0.452774763	1.22E-26	postive
PDHA1	COA6-AS1	0.434183715	2.10E-24	postive
PDHA1	COX10-AS1	0.425503582	2.09E-23	postive
PDHA1	CR381653.1	0.369181825	1.37E-17	postive

PDHA1	CRNDE	0.315239436	5.35E-13	postive
PDHA1	CROCCP2	0.365411475	3.06E-17	postive
PDHA1	CRTC3-AS1	0.432762583	3.08E-24	postive
PDHA1	CRYBB2P1	0.499872834	5.76E-33	postive
PDHA1	CRYZL2P	0.430566762	5.52E-24	postive
PDHA1	CRYZL2P-SEC16B	0.308514796	1.74E-12	postive
PDHA1	CSE1L-AS1	0.356029686	2.18E-16	postive
PDHA1	CT66	0.452984622	1.14E-26	postive
PDHA1	CTA-989H11.1	0.446035089	8.16E-26	postive
PDHA1	CTBP1-DT	0.526028706	6.30E-37	postive
PDHA1	CTD-2132N18.4	0.338543582	7.13E-15	postive
PDHA1	CTD-2134A5.3	0.418013545	1.44E-22	postive
PDHA1	CTD-2521M24.9	0.446345976	7.48E-26	postive
PDHA1	CTD-2540M10.1	0.413467389	4.55E-22	postive
PDHA1	CTD-2574D22.4	0.408819155	1.45E-21	postive
PDHA1	CTSLP3	0.313901205	6.78E-13	postive
PDHA1	CUTALP	0.420667312	7.33E-23	postive
PDHA1	CYP4F23P	0.413472244	4.55E-22	postive
PDHA1	CYP51A1-AS1	0.366453996	2.45E-17	postive
PDHA1	DANCR	0.4608695	1.17E-27	postive
PDHA1	DARS-AS1	0.384071677	5.07E-19	postive
PDHA1	DBIL5P	0.496193337	1.95E-32	postive
PDHA1	DCST1-AS1	0.47820043	6.20E-30	postive
PDHA1	DCXR-DT	0.321445431	1.76E-13	postive
PDHA1	DDX11-AS1	0.459152104	1.93E-27	postive
PDHA1	DDX39B-AS1	0.473810131	2.40E-29	postive
PDHA1	DEPDC1-AS1	0.390126211	1.27E-19	postive
PDHA1	DGCR11	0.384920122	4.18E-19	postive
PDHA1	DGUOK-AS1	0.40632754	2.67E-21	postive
PDHA1	DHRS4-AS1	0.475543547	1.41E-29	postive
PDHA1	DICER1-AS1	0.356361783	2.04E-16	postive
PDHA1	DLG5-AS1	0.369953734	1.16E-17	postive
PDHA1	DLGAP1-AS1	0.307902091	1.93E-12	postive
PDHA1	DM1-AS	0.49079732	1.14E-31	postive
PDHA1	DNAH10OS	0.307049586	2.24E-12	postive
PDHA1	DNAH17-AS1	0.306247221	2.57E-12	postive
PDHA1	DNAJC27-AS1	0.318977234	2.74E-13	postive
PDHA1	DNAJC3-DT	0.452359532	1.37E-26	postive
PDHA1	DNM1P35	0.313491514	7.29E-13	postive
PDHA1	DOCK9-DT	0.334327876	1.60E-14	postive
PDHA1	DPY19L2P3	0.389029953	1.63E-19	postive
PDHA1	DSTNP2	0.340384063	4.99E-15	postive
PDHA1	DUXAP8	0.307835166	1.95E-12	postive
PDHA1	DUXAP9	0.35408379	3.25E-16	postive
PDHA1	DYRK3-AS1	0.417896287	1.49E-22	postive
PDHA1	EBLN3P	0.472912646	3.16E-29	postive
PDHA1	ECI2-DT	0.350954953	6.14E-16	postive
PDHA1	EDRF1-DT	0.344836417	2.08E-15	postive
PDHA1	EHMT2-AS1	0.350255673	7.07E-16	postive
PDHA1	EIF1B-AS1	0.379304411	1.48E-18	postive

PDHA1	EIF2AK3-DT	0.447164805	5.95E-26	postive
PDHA1	EIF3J-DT	0.464082435	4.51E-28	postive
PDHA1	ELF3-AS1	0.511481259	1.11E-34	postive
PDHA1	ELOA-AS1	0.449535368	3.05E-26	postive
PDHA1	EMC1-AS1	0.488831054	2.15E-31	postive
PDHA1	EMC3-AS1	0.478656946	5.38E-30	postive
PDHA1	EMSLR	0.373094674	5.84E-18	postive
PDHA1	ENO1-AS1	0.409184993	1.32E-21	postive
PDHA1	ENTPD3-AS1	0.34132177	4.16E-15	postive
PDHA1	EP400P1	0.446861942	6.48E-26	postive
PDHA1	EPB41L4A-DT	0.342034409	3.62E-15	postive
PDHA1	ERICD	0.309893676	1.37E-12	postive
PDHA1	ERVMER61-1	0.347300755	1.28E-15	postive
PDHA1	ETV7-AS1	0.371126048	8.97E-18	postive
PDHA1	EXOC3-AS1	0.379976755	1.28E-18	postive
PDHA1	EXOSC10-AS1	0.436404881	1.15E-24	postive
PDHA1	F10-AS1	0.394851081	4.20E-20	postive
PDHA1	FABP5P3	0.305421659	2.96E-12	postive
PDHA1	FAHD2CP	0.310795759	1.17E-12	postive
PDHA1	FALEC	0.457164403	3.43E-27	postive
PDHA1	FAM111A-DT	0.4994909	6.54E-33	postive
PDHA1	FAM135A-AS1	0.354059251	3.27E-16	postive
PDHA1	FAM157A	0.328172325	5.10E-14	postive
PDHA1	FAM160A1-DT	0.3149751	5.61E-13	postive
PDHA1	FAM201A	0.300951207	6.30E-12	postive
PDHA1	FAM21EP	0.339305552	6.16E-15	postive
PDHA1	FAM21FP	0.431562095	4.24E-24	postive
PDHA1	FAM66D	0.402629963	6.57E-21	postive
PDHA1	FAM83C-AS1	0.343863726	2.53E-15	postive
PDHA1	FAM86B3P	0.435832122	1.35E-24	postive
PDHA1	FAM86C2P	0.519093664	7.64E-36	postive
PDHA1	FAM86DP	0.360878862	7.97E-17	postive
PDHA1	FAM86EP	0.456617224	4.02E-27	postive
PDHA1	FAM86FP	0.317800207	3.39E-13	postive
PDHA1	FAM86HP	0.432458508	3.34E-24	postive
PDHA1	FAM86JP	0.558963946	1.97E-42	postive
PDHA1	FBP2P1	0.358109413	1.42E-16	postive
PDHA1	FBXL19-AS1	0.305573156	2.88E-12	postive
PDHA1	FGD5-AS1	0.48966077	1.64E-31	postive
PDHA1	FLVCR1-DT	0.397363042	2.32E-20	postive
PDHA1	FMR1-AS1	0.326150253	7.42E-14	postive
PDHA1	FO393418.1	0.440954957	3.34E-25	postive
PDHA1	FOXD2-AS1	0.452417706	1.35E-26	postive
PDHA1	FOXD3-AS1	0.365603064	2.94E-17	postive
PDHA1	FOXN3-AS1	0.308497754	1.74E-12	postive
PDHA1	FOXP4-AS1	0.331481639	2.75E-14	postive
PDHA1	FRG1-DT	0.335021138	1.40E-14	postive
PDHA1	FRG1HP	0.480271292	3.25E-30	postive
PDHA1	FZD4-DT	0.387238476	2.46E-19	postive
PDHA1	GAS5-AS1	0.414345254	3.65E-22	postive

PDHA1	GASAL1	0.355731329	2.32E-16	postive
PDHA1	GATA2-AS1	0.410364375	9.88E-22	postive
PDHA1	GBAP1	0.438856218	5.93E-25	postive
PDHA1	GCC2-AS1	0.537218297	9.92E-39	postive
PDHA1	GEMIN7-AS1	0.354849806	2.78E-16	postive
PDHA1	GIHCG	0.413778423	4.21E-22	postive
PDHA1	GLIDR	0.369490782	1.28E-17	postive
PDHA1	GLUD1P3	0.301690812	5.57E-12	postive
PDHA1	GMDS-DT	0.350270181	7.05E-16	postive
PDHA1	GNRHR2	0.483871149	1.05E-30	postive
PDHA1	GOLGA2P5	0.301371697	5.87E-12	postive
PDHA1	GOLGA8EP	0.376940504	2.51E-18	postive
PDHA1	GPR176-DT	0.307357693	2.12E-12	postive
PDHA1	GPRC5D-AS1	0.350671334	6.50E-16	postive
PDHA1	GS1-124K5.3	0.346192067	1.59E-15	postive
PDHA1	GS1-358P8.4	0.412944161	5.19E-22	postive
PDHA1	GSEC	0.338798489	6.79E-15	postive
PDHA1	GTF2IP1	0.482837146	1.45E-30	postive
PDHA1	GTF2IP20	0.31603708	4.64E-13	postive
PDHA1	GTF2IP4	0.390196956	1.25E-19	postive
PDHA1	GTSE1-DT	0.3498461	7.67E-16	postive
PDHA1	GUSBP1	0.33591806	1.18E-14	postive
PDHA1	GUSBP11	0.309928648	1.36E-12	postive
PDHA1	GUSBP2	0.349226655	8.69E-16	postive
PDHA1	GYG2P1	0.325982927	7.65E-14	postive
PDHA1	H1FX-AS1	0.333731452	1.79E-14	postive
PDHA1	H2AZ2P1	0.380127775	1.23E-18	postive
PDHA1	H2BC19P	0.370390367	1.05E-17	postive
PDHA1	H2BC20P	0.346049476	1.64E-15	postive
PDHA1	HCG11	0.317290731	3.71E-13	postive
PDHA1	HCG15	0.316332412	4.40E-13	postive
PDHA1	HCG18	0.504527136	1.20E-33	postive
PDHA1	HCG25	0.529382406	1.85E-37	postive
PDHA1	HCG27	0.340356512	5.02E-15	postive
PDHA1	HCP5	0.339850994	5.54E-15	postive
PDHA1	HDAC11-AS1	0.367046598	2.16E-17	postive
PDHA1	HDHD5-AS1	0.361288209	7.31E-17	postive
PDHA1	HEIH	0.475899533	1.26E-29	postive
PDHA1	HERC2P2	0.312346127	8.91E-13	postive
PDHA1	HERC2P4	0.337736327	8.34E-15	postive
PDHA1	HM13-AS1	0.307489566	2.07E-12	postive
PDHA1	HM13-IT1	0.456065391	4.72E-27	postive
PDHA1	HMGB1P5	0.451949741	1.54E-26	postive
PDHA1	HMMR-AS1	0.413592209	4.41E-22	postive
PDHA1	HOMER3-AS1	0.300273374	7.06E-12	postive
PDHA1	HOXA-AS3	0.317860265	3.35E-13	postive
PDHA1	HPN-AS1	0.369849918	1.18E-17	postive
PDHA1	HSD17B7P2	0.302431574	4.91E-12	postive
PDHA1	HSP90AB2P	0.364252231	3.92E-17	postive
PDHA1	HYI-AS1	0.305309248	3.01E-12	postive

PDHA1	IBA57-DT	0.359582625	1.04E-16	postive
PDHA1	IDH1-AS1	0.434395595	1.99E-24	postive
PDHA1	IDI2-AS1	0.311671176	1.00E-12	postive
PDHA1	IGFL2-AS1	0.303124672	4.37E-12	postive
PDHA1	IL6R-AS1	0.408211392	1.68E-21	postive
PDHA1	ILF3-DT	0.506963427	5.25E-34	postive
PDHA1	INE1	0.398718555	1.68E-20	postive
PDHA1	INGX	0.325408327	8.51E-14	postive
PDHA1	INTS6-AS1	0.36695933	2.20E-17	postive
PDHA1	IPO9-AS1	0.5163142	2.04E-35	postive
PDHA1	IQCH-AS1	0.465975471	2.57E-28	postive
PDHA1	ITFG2-AS1	0.398199357	1.90E-20	postive
PDHA1	JPX	0.447044679	6.15E-26	postive
PDHA1	KB-1572G7.2	0.303061076	4.42E-12	postive
PDHA1	KCNIP2-AS1	0.346839601	1.40E-15	postive
PDHA1	KCNMB2-AS1	0.40537117	3.37E-21	postive
PDHA1	KCNQ1DN	0.376364876	2.85E-18	postive
PDHA1	KCTD21-AS1	0.361518858	6.97E-17	postive
PDHA1	KDM2B-DT	0.408592806	1.53E-21	postive
PDHA1	KDM4A-AS1	0.461351219	1.01E-27	postive
PDHA1	KDM7A-DT	0.355776079	2.30E-16	postive
PDHA1	KIF1C-AS1	0.333386031	1.92E-14	postive
PDHA1	KIF9-AS1	0.334382569	1.58E-14	postive
PDHA1	KMT2E-AS1	0.507511134	4.35E-34	postive
PDHA1	KRT18P55	0.300812782	6.45E-12	postive
PDHA1	KRT8P12	0.563765645	2.75E-43	postive
PDHA1	KTN1-AS1	0.511452365	1.12E-34	postive
PDHA1	L29074.1	0.341220819	4.24E-15	postive
PDHA1	L3MBTL2-AS1	0.333625121	1.83E-14	postive
PDHA1	LAMA5-AS1	0.400840332	1.01E-20	postive
PDHA1	LAMC1-AS1	0.336875162	9.84E-15	postive
PDHA1	LAMTOR5-AS1	0.361710033	6.69E-17	postive
PDHA1	LBX2-AS1	0.471104071	5.49E-29	postive
PDHA1	LENG8-AS1	0.373987836	4.81E-18	postive
PDHA1	LGALS8-AS1	0.477437009	7.86E-30	postive
PDHA1	LGALSL-DT	0.379148144	1.54E-18	postive
PDHA1	LIFR-AS1	0.304041597	3.74E-12	postive
PDHA1	LINC00106	0.383196602	6.19E-19	postive
PDHA1	LINC00115	0.480969601	2.61E-30	postive
PDHA1	LINC00173	0.373102813	5.83E-18	postive
PDHA1	LINC00174	0.377484473	2.22E-18	postive
PDHA1	LINC00205	0.344685238	2.15E-15	postive
PDHA1	LINC00235	0.40304195	5.94E-21	postive
PDHA1	LINC00294	0.358604914	1.28E-16	postive
PDHA1	LINC00323	0.393245313	6.12E-20	postive
PDHA1	LINC00339	0.486200046	4.99E-31	postive
PDHA1	LINC00346	0.42610032	1.79E-23	postive
PDHA1	LINC00449	0.385444712	3.71E-19	postive
PDHA1	LINC00466	0.323989547	1.10E-13	postive
PDHA1	LINC00467	0.406739734	2.41E-21	postive

PDHA1	LINC00471	0.499212766	7.18E-33	postive
PDHA1	LINC00476	0.413946737	4.04E-22	postive
PDHA1	LINC00494	0.35077675	6.36E-16	postive
PDHA1	LINC00511	0.392864269	6.69E-20	postive
PDHA1	LINC00526	0.303462476	4.13E-12	postive
PDHA1	LINC00623	0.303848725	3.86E-12	postive
PDHA1	LINC00630	0.500623471	4.48E-33	postive
PDHA1	LINC00637	0.38352538	5.74E-19	postive
PDHA1	LINC00638	0.338203479	7.62E-15	postive
PDHA1	LINC00652	0.325746081	8.00E-14	postive
PDHA1	LINC00653	0.388684331	1.77E-19	postive
PDHA1	LINC00654	0.351095355	5.96E-16	postive
PDHA1	LINC00659	0.353944641	3.34E-16	postive
PDHA1	LINC00665	0.389222245	1.56E-19	postive
PDHA1	LINC00667	0.419874545	8.98E-23	postive
PDHA1	LINC00680	0.409313586	1.28E-21	postive
PDHA1	LINC00685	0.311757757	9.88E-13	postive
PDHA1	LINC00839	0.309942902	1.36E-12	postive
PDHA1	LINC00847	0.532518965	5.78E-38	postive
PDHA1	LINC00863	0.392158083	7.89E-20	postive
PDHA1	LINC00869	0.357079346	1.76E-16	postive
PDHA1	LINC00884	0.502051439	2.78E-33	postive
PDHA1	LINC00885	0.306805981	2.33E-12	postive
PDHA1	LINC00887	0.470769153	6.07E-29	postive
PDHA1	LINC00888	0.529284169	1.91E-37	postive
PDHA1	LINC00896	0.300066106	7.31E-12	postive
PDHA1	LINC00899	0.312152813	9.22E-13	postive
PDHA1	LINC00906	0.315830568	4.82E-13	postive
PDHA1	LINC00909	0.542445571	1.35E-39	postive
PDHA1	LINC00910	0.397406508	2.30E-20	postive
PDHA1	LINC00921	0.404996937	3.70E-21	postive
PDHA1	LINC00954	0.320471888	2.10E-13	postive
PDHA1	LINC00957	0.353728079	3.49E-16	postive
PDHA1	LINC00963	0.499538449	6.44E-33	postive
PDHA1	LINC01001	0.366653419	2.35E-17	postive
PDHA1	LINC01003	0.372152681	7.18E-18	postive
PDHA1	LINC01004	0.301111812	6.14E-12	postive
PDHA1	LINC01006	0.3114286	1.05E-12	postive
PDHA1	LINC01011	0.478659851	5.38E-30	postive
PDHA1	LINC01023	0.461431713	9.88E-28	postive
PDHA1	LINC01063	0.46714941	1.81E-28	postive
PDHA1	LINC01089	0.364990127	3.35E-17	postive
PDHA1	LINC01093	0.345145399	1.96E-15	postive
PDHA1	LINC01106	0.329330172	4.11E-14	postive
PDHA1	LINC01123	0.315140022	5.44E-13	postive
PDHA1	LINC01128	0.448915024	3.64E-26	postive
PDHA1	LINC01132	0.419794664	9.16E-23	postive
PDHA1	LINC01138	0.393534524	5.72E-20	postive
PDHA1	LINC01144	0.419868059	8.99E-23	postive
PDHA1	LINC01145	0.428063352	1.07E-23	postive

PDHA1	LINC01151	0.35853013	1.30E-16	postive
PDHA1	LINC01184	0.488788231	2.18E-31	postive
PDHA1	LINC01220	0.357015872	1.78E-16	postive
PDHA1	LINC01232	0.478688744	5.33E-30	postive
PDHA1	LINC01270	0.484563381	8.41E-31	postive
PDHA1	LINC01271	0.465384945	3.07E-28	postive
PDHA1	LINC01273	0.551246256	4.36E-41	postive
PDHA1	LINC01275	0.45695669	3.65E-27	postive
PDHA1	LINC01278	0.596597433	1.59E-49	postive
PDHA1	LINC01285	0.454639651	7.12E-27	postive
PDHA1	LINC01293	0.327864695	5.40E-14	postive
PDHA1	LINC01301	0.384383875	4.73E-19	postive
PDHA1	LINC01311	0.458994954	2.02E-27	postive
PDHA1	LINC01315	0.30101612	6.24E-12	postive
PDHA1	LINC01348	0.307982619	1.90E-12	postive
PDHA1	LINC01354	0.373591264	5.24E-18	postive
PDHA1	LINC01366	0.379475637	1.43E-18	postive
PDHA1	LINC01372	0.401770475	8.08E-21	postive
PDHA1	LINC01387	0.362045793	6.24E-17	postive
PDHA1	LINC01410	0.304824659	3.27E-12	postive
PDHA1	LINC01424	0.306567783	2.43E-12	postive
PDHA1	LINC01431	0.353194459	3.90E-16	postive
PDHA1	LINC01497	0.359479649	1.07E-16	postive
PDHA1	LINC01521	0.383458223	5.83E-19	postive
PDHA1	LINC01534	0.359802725	9.98E-17	postive
PDHA1	LINC01560	0.561939185	5.83E-43	postive
PDHA1	LINC01569	0.393503655	5.76E-20	postive
PDHA1	LINC01578	0.317461743	3.60E-13	postive
PDHA1	LINC01585	0.385549206	3.63E-19	postive
PDHA1	LINC01588	0.326667773	6.74E-14	postive
PDHA1	LINC01621	0.38081779	1.06E-18	postive
PDHA1	LINC01637	0.303540541	4.07E-12	postive
PDHA1	LINC01679	0.302867444	4.56E-12	postive
PDHA1	LINC01686	0.395099746	3.96E-20	postive
PDHA1	LINC01693	0.390123041	1.27E-19	postive
PDHA1	LINC01703	0.501532616	3.30E-33	postive
PDHA1	LINC01715	0.325977515	7.66E-14	postive
PDHA1	LINC01719	0.305782267	2.78E-12	postive
PDHA1	LINC01770	0.357408046	1.64E-16	postive
PDHA1	LINC01775	0.379345606	1.47E-18	postive
PDHA1	LINC01806	0.408040359	1.75E-21	postive
PDHA1	LINC01814	0.301843853	5.43E-12	postive
PDHA1	LINC01816	0.354578763	2.94E-16	postive
PDHA1	LINC01842	0.366919505	2.22E-17	postive
PDHA1	LINC01881	0.45965586	1.66E-27	postive
PDHA1	LINC01909	0.33293439	2.09E-14	postive
PDHA1	LINC01952	0.402434046	6.88E-21	postive
PDHA1	LINC01972	0.391341078	9.55E-20	postive
PDHA1	LINC01975	0.303679094	3.98E-12	postive
PDHA1	LINC02004	0.300055022	7.33E-12	postive

PDHA1	LINC02012	0.35598626	2.20E-16	postive
PDHA1	LINC02014	0.361051628	7.69E-17	postive
PDHA1	LINC02021	0.36505365	3.30E-17	postive
PDHA1	LINC02026	0.386932273	2.64E-19	postive
PDHA1	LINC02028	0.325970827	7.67E-14	postive
PDHA1	LINC02062	0.354127775	3.22E-16	postive
PDHA1	LINC02076	0.419870835	8.99E-23	postive
PDHA1	LINC02099	0.345795367	1.72E-15	postive
PDHA1	LINC02130	0.318198177	3.16E-13	postive
PDHA1	LINC02137	0.371091126	9.04E-18	postive
PDHA1	LINC02193	0.361023746	7.73E-17	postive
PDHA1	LINC02210	0.455143827	6.16E-27	postive
PDHA1	LINC02243	0.333765986	1.78E-14	postive
PDHA1	LINC02280	0.34310938	2.93E-15	postive
PDHA1	LINC02321	0.311787782	9.83E-13	postive
PDHA1	LINC02361	0.311462712	1.04E-12	postive
PDHA1	LINC02412	0.352019389	4.95E-16	postive
PDHA1	LINC02449	0.30269375	4.70E-12	postive
PDHA1	LINC02482	0.42408348	3.03E-23	postive
PDHA1	LINC02569	0.309424384	1.48E-12	postive
PDHA1	LINC02585	0.353025747	4.03E-16	postive
PDHA1	LINC02601	0.438905164	5.85E-25	postive
PDHA1	LINC02604	0.398125298	1.93E-20	postive
PDHA1	LINC02611	0.340129982	5.25E-15	postive
PDHA1	LINC02637	0.426851598	1.47E-23	postive
PDHA1	LINC02693	0.433670677	2.41E-24	postive
PDHA1	LINC02707	0.368256444	1.67E-17	postive
PDHA1	LINC02709	0.473458922	2.68E-29	postive
PDHA1	LINC02740	0.317322271	3.69E-13	postive
PDHA1	LINC02749	0.370820221	9.59E-18	postive
PDHA1	LINC02757	0.358133972	1.41E-16	postive
PDHA1	LINC02766	0.322852399	1.36E-13	postive
PDHA1	LINC02777	0.308013413	1.89E-12	postive
PDHA1	LIPE-AS1	0.302265443	5.05E-12	postive
PDHA1	LIX1L-AS1	0.32969541	3.84E-14	postive
PDHA1	LMCD1-AS1	0.303709217	3.96E-12	postive
PDHA1	LNCTAM34A	0.323520285	1.20E-13	postive
PDHA1	LOH12CR2	0.390650136	1.12E-19	postive
PDHA1	LOXL1-AS1	0.312764392	8.28E-13	postive
PDHA1	LPP-AS2	0.460824104	1.18E-27	postive
PDHA1	LRRC37A17P	0.372896902	6.10E-18	postive
PDHA1	LRRC37BP1	0.499588182	6.33E-33	postive
PDHA1	LSP1P4	0.347034088	1.35E-15	postive
PDHA1	MAFG-DT	0.384364492	4.75E-19	postive
PDHA1	MAFTRR	0.310775592	1.17E-12	postive
PDHA1	MAN1B1-DT	0.402167159	7.34E-21	postive
PDHA1	MAP2K4P1	0.318743826	2.86E-13	postive
PDHA1	MAP3K14-AS1	0.377583892	2.18E-18	postive
PDHA1	MAP3K2-DT	0.402364672	7.00E-21	postive
PDHA1	MAP4K3-DT	0.400061013	1.22E-20	postive

PDHA1	MAPK6-DT	0.453692852	9.34E-27	postive
PDHA1	MAPKAPK5-AS1	0.58823267	7.21E-48	postive
PDHA1	MATN1-AS1	0.331353794	2.81E-14	postive
PDHA1	MCM3AP-AS1	0.402371952	6.99E-21	postive
PDHA1	MCPH1-AS1	0.398415942	1.81E-20	postive
PDHA1	MDC1-AS1	0.30725353	2.16E-12	postive
PDHA1	MED14OS	0.404637916	4.03E-21	postive
PDHA1	MED8-AS1	0.345513321	1.82E-15	postive
PDHA1	MELTF-AS1	0.503213125	1.88E-33	postive
PDHA1	MFF-DT	0.302435935	4.91E-12	postive
PDHA1	MHENCR	0.420658709	7.34E-23	postive
PDHA1	MIATNB	0.306470415	2.47E-12	postive
PDHA1	MID1IP1-AS1	0.340777248	4.62E-15	postive
PDHA1	MIF-AS1	0.344370094	2.29E-15	postive
PDHA1	MINCR	0.432394276	3.39E-24	postive
PDHA1	MIPEPP3	0.418572772	1.25E-22	postive
PDHA1	MIR200CHG	0.347211354	1.30E-15	postive
PDHA1	MIR210HG	0.360635148	8.39E-17	postive
PDHA1	MIR3150BHG	0.366482589	2.44E-17	postive
PDHA1	MIR34AHG	0.312128938	9.26E-13	postive
PDHA1	MIR3936HG	0.322829746	1.37E-13	postive
PDHA1	MIR4435-2HG	0.319881912	2.33E-13	postive
PDHA1	MIR4453HG	0.408612115	1.52E-21	postive
PDHA1	MIR4458HG	0.338358306	7.39E-15	postive
PDHA1	MIR762HG	0.520505352	4.62E-36	postive
PDHA1	MIR924HG	0.35959486	1.04E-16	postive
PDHA1	MIS18A-AS1	0.365541897	2.98E-17	postive
PDHA1	MKLN1-AS	0.386656692	2.81E-19	postive
PDHA1	MMP25-AS1	0.301968036	5.31E-12	postive
PDHA1	MNX1-AS2	0.306233193	2.57E-12	postive
PDHA1	MORF4L2-AS1	0.44223623	2.34E-25	postive
PDHA1	MRPL20-AS1	0.551381193	4.13E-41	postive
PDHA1	MRPL45P2	0.402832103	6.25E-21	postive
PDHA1	MRPS31P4	0.393114909	6.31E-20	postive
PDHA1	MSH5-SAPCD1	0.402952504	6.07E-21	postive
PDHA1	MSL3P1	0.414487533	3.53E-22	postive
PDHA1	MT1DP	0.311771905	9.85E-13	postive
PDHA1	MTMR9LP	0.33840337	7.33E-15	postive
PDHA1	MUC20-OT1	0.436797772	1.04E-24	postive
PDHA1	MYLK-AS1	0.395325742	3.76E-20	postive
PDHA1	NACA4P	0.421117874	6.53E-23	postive
PDHA1	NALT1	0.328700487	4.62E-14	postive
PDHA1	NAPA-AS1	0.356469975	1.99E-16	postive
PDHA1	NBPF25P	0.383990603	5.17E-19	postive
PDHA1	NBPF8	0.437460966	8.67E-25	postive
PDHA1	NBR2	0.486381877	4.71E-31	postive
PDHA1	NCBP2-AS1	0.390207683	1.24E-19	postive
PDHA1	NCK1-DT	0.401999567	7.65E-21	postive
PDHA1	NDUFA6-DT	0.341510431	4.01E-15	postive
PDHA1	NDUFB2-AS1	0.440978164	3.32E-25	postive

PDHA1	NDUFV2-AS1	0.365582828	2.95E-17	postive
PDHA1	NEBL-AS1	0.386871491	2.68E-19	postive
PDHA1	NFYC-AS1	0.32487046	9.39E-14	postive
PDHA1	NIFK-AS1	0.585335062	2.64E-47	postive
PDHA1	NINJ2-AS1	0.325895758	7.78E-14	postive
PDHA1	NKAPP1	0.386649116	2.82E-19	postive
PDHA1	NNT-AS1	0.435529998	1.46E-24	postive
PDHA1	NOP14-AS1	0.462668368	6.86E-28	postive
PDHA1	NOP53-AS1	0.341384859	4.11E-15	postive
PDHA1	NORAD	0.422049071	5.13E-23	postive
PDHA1	NPEPPSP1	0.350385266	6.88E-16	postive
PDHA1	NPIPP1	0.386554648	2.88E-19	postive
PDHA1	NRAV	0.444816753	1.15E-25	postive
PDHA1	NRSN2-AS1	0.61813183	5.03E-54	postive
PDHA1	NSMCE1-DT	0.331098537	2.95E-14	postive
PDHA1	NSUN5P1	0.463834	4.86E-28	postive
PDHA1	NSUN5P2	0.477631535	7.40E-30	postive
PDHA1	NUDT16P1	0.534824972	2.44E-38	postive
PDHA1	NUP50-DT	0.458124474	2.60E-27	postive
PDHA1	NUTM2A-AS1	0.47929104	4.42E-30	postive
PDHA1	NUTM2B-AS1	0.32503346	9.12E-14	postive
PDHA1	NXT1-AS1	0.312834522	8.18E-13	postive
PDHA1	OBI1-AS1	0.318816586	2.82E-13	postive
PDHA1	OGFR-AS1	0.328356084	4.93E-14	postive
PDHA1	OGFRP1	0.409128339	1.34E-21	postive
PDHA1	OIP5-AS1	0.430300446	5.93E-24	postive
PDHA1	OLMALINC	0.503708674	1.59E-33	postive
PDHA1	OR2A1-AS1	0.312686284	8.39E-13	postive
PDHA1	OR2A9P	0.308207466	1.83E-12	postive
PDHA1	OR4G11P	0.362375971	5.82E-17	postive
PDHA1	OR4G2P	0.322529179	1.44E-13	postive
PDHA1	OR7E14P	0.319856117	2.34E-13	postive
PDHA1	OR7E161P	0.308981088	1.60E-12	postive
PDHA1	OSER1-DT	0.470960773	5.73E-29	postive
PDHA1	OSGEPL1-AS1	0.368432947	1.61E-17	postive
PDHA1	OTUD6B-AS1	0.477098969	8.73E-30	postive
PDHA1	OXCT1-AS1	0.317294063	3.71E-13	postive
PDHA1	PAN3-AS1	0.307728494	1.99E-12	postive
PDHA1	PARD6G-AS1	0.348471948	1.01E-15	postive
PDHA1	PARGP1	0.341113471	4.33E-15	postive
PDHA1	PAXBP1-AS1	0.311533814	1.03E-12	postive
PDHA1	PAXIP1-AS1	0.469976306	7.72E-29	postive
PDHA1	PAXIP1-AS2	0.350194777	7.15E-16	postive
PDHA1	PCAT6	0.424854983	2.48E-23	postive
PDHA1	PCBP1-AS1	0.453079566	1.11E-26	postive
PDHA1	PCCA-DT	0.353467896	3.68E-16	postive
PDHA1	PCOTH	0.304140428	3.68E-12	postive
PDHA1	PDCD4-AS1	0.364397105	3.80E-17	postive
PDHA1	PDIA3P1	0.469395588	9.21E-29	postive
PDHA1	PGAM1P8	0.394598687	4.46E-20	postive

PDHA1	PI4KAP2	0.361569056	6.90E-17	postive
PDHA1	PIK3IP1-AS1	0.31432669	6.29E-13	postive
PDHA1	PINK1-AS	0.426171264	1.76E-23	postive
PDHA1	PIPSL	0.413264878	4.79E-22	postive
PDHA1	PITPNA-AS1	0.505697819	8.08E-34	postive
PDHA1	PITRM1-AS1	0.368837598	1.47E-17	postive
PDHA1	PKD1P6	0.440468965	3.81E-25	postive
PDHA1	PLEKHA8P1	0.427721758	1.17E-23	postive
PDHA1	PMS2CL	0.518238624	1.04E-35	postive
PDHA1	PMS2P3	0.432099724	3.67E-24	postive
PDHA1	PMS2P4	0.427179846	1.35E-23	postive
PDHA1	POLH-AS1	0.375455339	3.48E-18	postive
PDHA1	POLR2J4	0.457411543	3.20E-27	postive
PDHA1	PPP1R26-AS1	0.403248782	5.65E-21	postive
PDHA1	PPP3CB-AS1	0.372671077	6.41E-18	postive
PDHA1	PPT2-EGFL8	0.505526757	8.57E-34	postive
PDHA1	PRC1-AS1	0.411246449	7.93E-22	postive
PDHA1	PRELID1P1	0.390445667	1.18E-19	postive
PDHA1	PRKAG2-AS1	0.303112828	4.38E-12	postive
PDHA1	PRKAR2A-AS1	0.404964632	3.73E-21	postive
PDHA1	PRMT5-AS1	0.471789031	4.46E-29	postive
PDHA1	PRORSD1P	0.492561628	6.42E-32	postive
PDHA1	PRRT3-AS1	0.322767438	1.38E-13	postive
PDHA1	PSMA3-AS1	0.392149776	7.91E-20	postive
PDHA1	PSMB8-AS1	0.387600205	2.27E-19	postive
PDHA1	PTENP1	0.338143572	7.71E-15	postive
PDHA1	PTMAP5	0.341322085	4.16E-15	postive
PDHA1	PTOV1-AS1	0.501104437	3.81E-33	postive
PDHA1	PTOV1-AS2	0.362361371	5.84E-17	postive
PDHA1	PTPRVP	0.304437663	3.50E-12	postive
PDHA1	PXN-AS1	0.559502225	1.58E-42	postive
PDHA1	RAB11B-AS1	0.307999308	1.90E-12	postive
PDHA1	RAB30-DT	0.515143361	3.09E-35	postive
PDHA1	RABGAP1L-DT	0.376311815	2.88E-18	postive
PDHA1	RAD51-AS1	0.338402482	7.33E-15	postive
PDHA1	RAET1E-AS1	0.330739976	3.16E-14	postive
PDHA1	RALY-AS1	0.401193489	9.28E-21	postive
PDHA1	RAP2C-AS1	0.339393995	6.05E-15	postive
PDHA1	RAP2CP1	0.306605456	2.41E-12	postive
PDHA1	RARA-AS1	0.38552977	3.64E-19	postive
PDHA1	RASA4CP	0.351592682	5.39E-16	postive
PDHA1	RASAL2-AS1	0.363063146	5.04E-17	postive
PDHA1	RASSF1-AS1	0.41349135	4.53E-22	postive
PDHA1	RASSF8-AS1	0.300918453	6.34E-12	postive
PDHA1	RBM15-AS1	0.532228052	6.44E-38	postive
PDHA1	RBM26-AS1	0.412236796	6.20E-22	postive
PDHA1	RDM1P5	0.379011672	1.58E-18	postive
PDHA1	RHPN1-AS1	0.377176217	2.38E-18	postive
PDHA1	RN7SL832P	0.379450755	1.44E-18	postive
PDHA1	RNASEH1-AS1	0.498044852	1.06E-32	postive

PDHA1	RNF139-AS1	0.337871822	8.12E-15	postive
PDHA1	RNF157-AS1	0.334830867	1.46E-14	postive
PDHA1	RNF216P1	0.47620471	1.15E-29	postive
PDHA1	ROCK1P1	0.314449087	6.15E-13	postive
PDHA1	RP11-101E13.5	0.405061261	3.64E-21	postive
PDHA1	RP11-10A14.4	0.394146984	4.96E-20	postive
PDHA1	RP11-10C24.1	0.351130405	5.92E-16	postive
PDHA1	RP11-111M22.4	0.343868725	2.52E-15	postive
PDHA1	RP11-115J16.1	0.363368785	4.72E-17	postive
PDHA1	RP11-141C7.4	0.302047504	5.24E-12	postive
PDHA1	RP11-14C10.5	0.353851134	3.41E-16	postive
PDHA1	RP11-153F5.7	0.345960592	1.67E-15	postive
PDHA1	RP11-15N24.4	0.355767707	2.30E-16	postive
PDHA1	RP11-160O5.1	0.331178878	2.91E-14	postive
PDHA1	RP11-175O19.4	0.395181902	3.88E-20	postive
PDHA1	RP11-199F11.2	0.458809762	2.13E-27	postive
PDHA1	RP11-226L15.5	0.421339871	6.16E-23	postive
PDHA1	RP1-122P22.2	0.396637348	2.75E-20	postive
PDHA1	RP11-254F7.4	0.47672492	9.80E-30	postive
PDHA1	RP11-258C19.7	0.464966093	3.47E-28	postive
PDHA1	RP11-282O18.3	0.378090057	1.94E-18	postive
PDHA1	RP11-337C18.9	0.331091995	2.95E-14	postive
PDHA1	RP11-380L11.4	0.301502054	5.75E-12	postive
PDHA1	RP11-435O5.2	0.524116591	1.26E-36	postive
PDHA1	RP11-439L18.2	0.3066755	2.38E-12	postive
PDHA1	RP11-465N4.5	0.458730369	2.18E-27	postive
PDHA1	RP11-508N22.13	0.31632724	4.41E-13	postive
PDHA1	RP11-529E10.6	0.311989715	9.49E-13	postive
PDHA1	RP11-540B6.6	0.444751173	1.17E-25	postive
PDHA1	RP11-61L19.3	0.363450328	4.64E-17	postive
PDHA1	RP11-658F2.8	0.389053753	1.62E-19	postive
PDHA1	RP11-661A12.4	0.319392333	2.55E-13	postive
PDHA1	RP11-706O15.1	0.488182705	2.65E-31	postive
PDHA1	RP11-876N24.4	0.360074533	9.43E-17	postive
PDHA1	RP11-876N24.5	0.359255778	1.12E-16	postive
PDHA1	RP11-98G7.1	0.392796489	6.80E-20	postive
PDHA1	RP13-476E20.1	0.393050235	6.41E-20	postive
PDHA1	RP4-545L17.11	0.460598338	1.26E-27	postive
PDHA1	RP5-1039K5.19	0.30960737	1.44E-12	postive
PDHA1	RP5-1112D6.4	0.424083228	3.03E-23	postive
PDHA1	RP5-1125A11.6	0.345361533	1.88E-15	postive
PDHA1	RP5-1125A11.7	0.332559931	2.24E-14	postive
PDHA1	RP9P	0.399275204	1.47E-20	postive
PDHA1	RPARP-AS1	0.393660589	5.56E-20	postive
PDHA1	RPL12P25	0.313284392	7.56E-13	postive
PDHA1	RPL13P5	0.350890197	6.22E-16	postive
PDHA1	RPL23AP53	0.434964512	1.70E-24	postive
PDHA1	RPL23AP7	0.436278902	1.19E-24	postive
PDHA1	RPL23AP82	0.449014462	3.54E-26	postive
PDHA1	RPL32P3	0.393685833	5.52E-20	postive

PDHA1	RPL6P27	0.373601729	5.23E-18	postive
PDHA1	RPLP1P6	0.323769751	1.15E-13	postive
PDHA1	RPP38-DT	0.34513808	1.96E-15	postive
PDHA1	RPS10P7	0.391786718	8.61E-20	postive
PDHA1	RPS18P9	0.337588637	8.58E-15	postive
PDHA1	RPS27P25	0.306678462	2.38E-12	postive
PDHA1	RPS4XP16	0.382865435	6.67E-19	postive
PDHA1	RPSAP69	0.417092042	1.83E-22	postive
PDHA1	RRN3P3	0.430653291	5.40E-24	postive
PDHA1	RRP7BP	0.478690662	5.33E-30	postive
PDHA1	RTCA-AS1	0.328903801	4.45E-14	postive
PDHA1	RUSC1-AS1	0.425081209	2.34E-23	postive
PDHA1	SAMD12-AS1	0.313180997	7.70E-13	postive
PDHA1	SAP30-DT	0.388313472	1.92E-19	postive
PDHA1	SBDSP1	0.457293681	3.31E-27	postive
PDHA1	SBF2-AS1	0.419339487	1.03E-22	postive
PDHA1	SBNO1-AS1	0.459999347	1.50E-27	postive
PDHA1	SCAMP1-AS1	0.480894378	2.68E-30	postive
PDHA1	SCAND2P	0.381361501	9.36E-19	postive
PDHA1	SCAT2	0.322256313	1.52E-13	postive
PDHA1	SCGB1B2P	0.309095573	1.57E-12	postive
PDHA1	SDAD1P1	0.377896489	2.03E-18	postive
PDHA1	SDCBP2-AS1	0.365082816	3.28E-17	postive
PDHA1	SDHAP1	0.530697915	1.14E-37	postive
PDHA1	SEC23A-AS1	0.321653977	1.69E-13	postive
PDHA1	SEC24B-AS1	0.428287536	1.01E-23	postive
PDHA1	SEMA3F-AS1	0.317379757	3.65E-13	postive
PDHA1	SEPSECS-AS1	0.460763452	1.20E-27	postive
PDHA1	SEPTIN4-AS1	0.447867989	4.88E-26	postive
PDHA1	SEPTIN7-AS1	0.30257844	4.79E-12	postive
PDHA1	SEPTIN7P2	0.377758375	2.09E-18	postive
PDHA1	SERHL	0.3271785	6.14E-14	postive
PDHA1	SGMS1-AS1	0.308678324	1.69E-12	postive
PDHA1	SH3GL1P1	0.363915223	4.21E-17	postive
PDHA1	SHLD2P1	0.359408179	1.08E-16	postive
PDHA1	SIAH2-AS1	0.329160887	4.24E-14	postive
PDHA1	SLC12A9-AS1	0.347303946	1.28E-15	postive
PDHA1	SLC25A25-AS1	0.321029744	1.89E-13	postive
PDHA1	SLC25A30-AS1	0.36460576	3.63E-17	postive
PDHA1	SLC25A5-AS1	0.46156371	9.50E-28	postive
PDHA1	SLC25A5P1	0.370265709	1.08E-17	postive
PDHA1	SLC2A1-AS1	0.392806342	6.79E-20	postive
PDHA1	SLX1A-SULT1A3	0.345315333	1.90E-15	postive
PDHA1	SMG7-AS1	0.427454314	1.26E-23	postive
PDHA1	SMPD5	0.326362433	7.14E-14	postive
PDHA1	SNAI3-AS1	0.402088033	7.48E-21	postive
PDHA1	SNHG1	0.448191629	4.46E-26	postive
PDHA1	SNHG10	0.520281449	5.00E-36	postive
PDHA1	SNHG11	0.573668916	4.28E-45	postive
PDHA1	SNHG12	0.387988833	2.07E-19	postive

PDHA1	SNHG16	0.418203033	1.38E-22 postive
PDHA1	SNHG17	0.503510942	1.70E-33 postive
PDHA1	SNHG20	0.473684698	2.50E-29 postive
PDHA1	SNHG21	0.542643989	1.25E-39 postive
PDHA1	SNHG28	0.467387224	1.68E-28 postive
PDHA1	SNHG3	0.375902592	3.16E-18 postive
PDHA1	SNHG30	0.51158734	1.07E-34 postive
PDHA1	SNHG32	0.413765441	4.23E-22 postive
PDHA1	SNHG4	0.361833929	6.52E-17 postive
PDHA1	SNHG6	0.374628279	4.18E-18 postive
PDHA1	SNHG7	0.425831944	1.92E-23 postive
PDHA1	SNHG8	0.384042898	5.11E-19 postive
PDHA1	SNHG9	0.309212838	1.54E-12 postive
PDHA1	SOX2-OT	0.334304613	1.61E-14 postive
PDHA1	SP2-AS1	0.340440768	4.94E-15 postive
PDHA1	SPAG5-AS1	0.441000214	3.30E-25 postive
PDHA1	SPATA41	0.374566162	4.23E-18 postive
PDHA1	SPINT1-AS1	0.321006994	1.90E-13 postive
PDHA1	SREBF2-AS1	0.498707189	8.49E-33 postive
PDHA1	SRP14-AS1	0.408064408	1.74E-21 postive
PDHA1	SRP54-AS1	0.337714953	8.37E-15 postive
PDHA1	SSR4P1	0.369704584	1.22E-17 postive
PDHA1	ST20-AS1	0.317944592	3.30E-13 postive
PDHA1	ST3GAL6-AS1	0.364914462	3.40E-17 postive
PDHA1	STAG3L1	0.342559564	3.26E-15 postive
PDHA1	STAG3L2	0.457165647	3.43E-27 postive
PDHA1	STAG3L3	0.453856538	8.92E-27 postive
PDHA1	STAG3L4	0.484946239	7.45E-31 postive
PDHA1	STAG3L5P	0.428782103	8.86E-24 postive
PDHA1	STAG3L5P-PVRIG2P-PILRB	0.457883908	2.79E-27 postive
PDHA1	STAM-AS1	0.380803598	1.06E-18 postive
PDHA1	STARD7-AS1	0.569644713	2.36E-44 postive
PDHA1	STK24-AS1	0.417301247	1.73E-22 postive
PDHA1	STK4-AS1	0.472812729	3.26E-29 postive
PDHA1	STPG3-AS1	0.304925137	3.22E-12 postive
PDHA1	STT3A-AS1	0.33537048	1.31E-14 postive
PDHA1	STX18-AS1	0.462972272	6.27E-28 postive
PDHA1	SUCLA2-AS1	0.445697983	8.97E-26 postive
PDHA1	SUCLG2-AS1	0.333099013	2.02E-14 postive
PDHA1	SUGT1P4-STRA6LP	0.304332043	3.56E-12 postive
PDHA1	SUMO2P17	0.433795266	2.33E-24 postive
PDHA1	SUZ12P1	0.476875137	9.36E-30 postive
PDHA1	SVIL-AS1	0.439488706	4.99E-25 postive
PDHA1	SYNGAP1-AS1	0.307188499	2.18E-12 postive
PDHA1	SYS1-DBNDD2	0.377433702	2.25E-18 postive
PDHA1	TAF1A-AS1	0.388018369	2.06E-19 postive
PDHA1	TAPT1-AS1	0.356331705	2.05E-16 postive
PDHA1	TBC1D8-AS1	0.396854424	2.62E-20 postive
PDHA1	TDRKH-AS1	0.353594669	3.59E-16 postive
PDHA1	TFAP2A-AS1	0.386907935	2.66E-19 postive

PDHA1	THAP7-AS1	0.363450637	4.64E-17	postive
PDHA1	THAP9-AS1	0.461534272	9.58E-28	postive
PDHA1	THUMPD3-AS1	0.330709589	3.17E-14	postive
PDHA1	TIPARP-AS1	0.461531053	9.59E-28	postive
PDHA1	TM4SF19-AS1	0.403546078	5.26E-21	postive
PDHA1	TMC3-AS1	0.488423672	2.45E-31	postive
PDHA1	TMCC1-AS1	0.496541846	1.74E-32	postive
PDHA1	TMCO1-AS1	0.321605125	1.71E-13	postive
PDHA1	TMED2-DT	0.496385844	1.83E-32	postive
PDHA1	TMEM147-AS1	0.404713469	3.96E-21	postive
PDHA1	TMEM161B-AS1	0.378998007	1.59E-18	postive
PDHA1	TMEM198B	0.366519248	2.42E-17	postive
PDHA1	TMEM202-AS1	0.487464141	3.33E-31	postive
PDHA1	TMEM44-AS1	0.56733314	6.24E-44	postive
PDHA1	TMEM99	0.34867676	9.71E-16	postive
PDHA1	TMPO-AS1	0.542346821	1.41E-39	postive
PDHA1	TOB1-AS1	0.540307482	3.07E-39	postive
PDHA1	TOLLIP-AS1	0.439605798	4.83E-25	postive
PDHA1	TONSL-AS1	0.378139174	1.92E-18	postive
PDHA1	TP73-AS1	0.448893142	3.66E-26	postive
PDHA1	TPM3P9	0.427323099	1.30E-23	postive
PDHA1	TPRG1-AS1	0.365107997	3.27E-17	postive
PDHA1	TPT1-AS1	0.379152443	1.53E-18	postive
PDHA1	TPTEP2	0.334014533	1.70E-14	postive
PDHA1	TRAF3IP2-AS1	0.405415992	3.34E-21	postive
PDHA1	TRAM2-AS1	0.47236607	3.74E-29	postive
PDHA1	TRAPPC12-AS1	0.379461868	1.43E-18	postive
PDHA1	TRIM52-AS1	0.473069492	3.02E-29	postive
PDHA1	TSPY26P	0.36118278	7.48E-17	postive
PDHA1	TSTD3	0.441429513	2.93E-25	postive
PDHA1	TTC28-AS1	0.500261989	5.06E-33	postive
PDHA1	TTC41P	0.350165551	7.20E-16	postive
PDHA1	TTTY20	0.353707516	3.51E-16	postive
PDHA1	TUBBP1	0.381061927	1.00E-18	postive
PDHA1	TUBBP9	0.384475841	4.63E-19	postive
PDHA1	TUG1	0.522222771	2.50E-36	postive
PDHA1	TULP3P1	0.334150546	1.66E-14	postive
PDHA1	TYMSOS	0.469241369	9.64E-29	postive
PDHA1	U47924.2	0.377430547	2.25E-18	postive
PDHA1	U62317.2	0.315831029	4.82E-13	postive
PDHA1	U91328.1	0.550887684	5.02E-41	postive
PDHA1	UBA6-AS1	0.493014324	5.54E-32	postive
PDHA1	UBAC2-AS1	0.481308453	2.35E-30	postive
PDHA1	UBE2D3-AS1	0.454649754	7.10E-27	postive
PDHA1	UBE2E1-AS1	0.335331789	1.32E-14	postive
PDHA1	UBE2Q1-AS1	0.397908346	2.04E-20	postive
PDHA1	UBL7-AS1	0.332839669	2.12E-14	postive
PDHA1	UBOX5-AS1	0.304992213	3.18E-12	postive
PDHA1	UBR5-AS1	0.387575283	2.28E-19	postive
PDHA1	UGDH-AS1	0.46059693	1.26E-27	postive

PDHA1	UPF3AP2	0.382235012	7.69E-19	postive
PDHA1	URAHP	0.36036352	8.88E-17	postive
PDHA1	URB1-AS1	0.461166785	1.07E-27	postive
PDHA1	USP27X-AS1	0.531364123	8.87E-38	postive
PDHA1	USP2-AS1	0.334898431	1.44E-14	postive
PDHA1	USP3-AS1	0.304271883	3.60E-12	postive
PDHA1	USP46-AS1	0.476489769	1.05E-29	postive
PDHA1	UVRAG-DT	0.343346876	2.80E-15	postive
PDHA1	UXT-AS1	0.620746427	1.35E-54	postive
PDHA1	VASH1-AS1	0.387789084	2.17E-19	postive
PDHA1	VDAC1P8	0.40329189	5.59E-21	postive
PDHA1	VPS13B-DT	0.478363514	5.90E-30	postive
PDHA1	VPS33B-DT	0.379845116	1.31E-18	postive
PDHA1	VPS9D1-AS1	0.374034096	4.76E-18	postive
PDHA1	WAC-AS1	0.541160638	2.21E-39	postive
PDHA1	WARS2-AS1	0.406763139	2.40E-21	postive
PDHA1	WASH3P	0.533380997	4.19E-38	postive
PDHA1	WASH6P	0.385017693	4.09E-19	postive
PDHA1	WASIR2	0.313316919	7.51E-13	postive
PDHA1	WHAMMP3	0.30911907	1.56E-12	postive
PDHA1	WWC2-AS2	0.315218691	5.37E-13	postive
PDHA1	WWTR1-AS1	0.55088226	5.03E-41	postive
PDHA1	YEATS2-AS1	0.383650078	5.58E-19	postive
PDHA1	YTHDF3-AS1	0.418875811	1.16E-22	postive
PDHA1	Z82185.1	0.328473802	4.82E-14	postive
PDHA1	Z82246.1	0.353403103	3.73E-16	postive
PDHA1	Z83844.2	0.340393219	4.98E-15	postive
PDHA1	Z83851.4	0.426618216	1.56E-23	postive
PDHA1	Z84485.1	0.326535696	6.91E-14	postive
PDHA1	Z92544.1	0.464774964	3.68E-28	postive
PDHA1	Z94721.1	0.336883249	9.82E-15	postive
PDHA1	Z95114.1	0.320283703	2.17E-13	postive
PDHA1	Z95115.1	0.409448128	1.24E-21	postive
PDHA1	Z97055.2	0.351516342	5.48E-16	postive
PDHA1	Z97634.1	0.485548007	6.15E-31	postive
PDHA1	Z98884.2	0.345421464	1.86E-15	postive
PDHA1	Z99572.1	0.487045918	3.81E-31	postive
PDHA1	ZBED3-AS1	0.410593601	9.33E-22	postive
PDHA1	ZBED5-AS1	0.399269383	1.47E-20	postive
PDHA1	ZBTB11-AS1	0.451973022	1.53E-26	postive
PDHA1	ZBTB44-DT	0.351476342	5.52E-16	postive
PDHA1	ZEB1-AS1	0.371826245	7.71E-18	postive
PDHA1	ZFHX2-AS1	0.305437812	2.95E-12	postive
PDHA1	ZFX-AS1	0.326924159	6.43E-14	postive
PDHA1	ZKSCAN2-DT	0.335995208	1.16E-14	postive
PDHA1	ZNF137P	0.365270139	3.16E-17	postive
PDHA1	ZNF205-AS1	0.396121705	3.11E-20	postive
PDHA1	ZNF213-AS1	0.434726027	1.82E-24	postive
PDHA1	ZNF252P	0.45292405	1.16E-26	postive
PDHA1	ZNF252P-AS1	0.407183625	2.17E-21	postive

PDHA1	ZNF271P	0.315274666	5.32E-13	postive
PDHA1	ZNF286B	0.323560222	1.19E-13	postive
PDHA1	ZNF32-AS1	0.30825787	1.82E-12	postive
PDHA1	ZNF32-AS2	0.393275926	6.08E-20	postive
PDHA1	ZNF337-AS1	0.353398135	3.74E-16	postive
PDHA1	ZNF37BP	0.360270716	9.05E-17	postive
PDHA1	ZNF426-DT	0.413117737	4.97E-22	postive
PDHA1	ZNF433-AS1	0.322613702	1.42E-13	postive
PDHA1	ZNF436-AS1	0.367286757	2.05E-17	postive
PDHA1	ZNF460-AS1	0.319771878	2.38E-13	postive
PDHA1	ZNF503-AS2	0.476755993	9.71E-30	postive
PDHA1	ZNF529-AS1	0.421600705	5.76E-23	postive
PDHA1	ZNF561-AS1	0.400068837	1.22E-20	postive
PDHA1	ZNF674-AS1	0.500889157	4.10E-33	postive
PDHA1	ZNF687-AS1	0.494642876	3.25E-32	postive
PDHA1	ZNF767P	0.406511938	2.55E-21	postive
PDHA1	ZNF788P	0.396411785	2.91E-20	postive
PDHA1	ZNF815P	0.310347924	1.26E-12	postive
PDHA1	ZNRD1ASP	0.374048421	4.74E-18	postive
PDHA1	ZNRD2-AS1	0.54649517	2.82E-40	postive
PDHA1	ZNRF2P2	0.314185113	6.45E-13	postive
PDHA1	ZSCAN12P1	0.361553241	6.92E-17	postive
PDHA1	ZSCAN16-AS1	0.481257915	2.39E-30	postive
PDHB	A2M-AS1	0.306246499	2.57E-12	postive
PDHB	ABCA11P	0.317341214	3.68E-13	postive
PDHB	AC000068.1	0.333749652	1.79E-14	postive
PDHB	AC002116.2	0.30346444	4.12E-12	postive
PDHB	AC002310.1	0.440934913	3.36E-25	postive
PDHB	AC002451.1	0.363521478	4.57E-17	postive
PDHB	AC002467.1	0.515033344	3.21E-35	postive
PDHB	AC002470.1	0.312800502	8.23E-13	postive
PDHB	AC004221.1	0.307037888	2.24E-12	postive
PDHB	AC004241.1	0.397726429	2.13E-20	postive
PDHB	AC004241.3	0.37458496	4.22E-18	postive
PDHB	AC004596.1	0.43962684	4.80E-25	postive
PDHB	AC004771.2	0.390375704	1.19E-19	postive
PDHB	AC004801.6	0.384187407	4.94E-19	postive
PDHB	AC004803.1	0.477935235	6.74E-30	postive
PDHB	AC004812.2	0.387982377	2.08E-19	postive
PDHB	AC004893.11	0.34822662	1.06E-15	postive
PDHB	AC004943.2	0.349387895	8.41E-16	postive
PDHB	AC004980.1	0.333504772	1.87E-14	postive
PDHB	AC005005.4	0.316871528	4.00E-13	postive
PDHB	AC005034.4	0.532661747	5.48E-38	postive
PDHB	AC005034.6	0.365195161	3.21E-17	postive
PDHB	AC005076.2	0.352671362	4.33E-16	postive
PDHB	AC005229.4	0.421808486	5.46E-23	postive
PDHB	AC005261.4	0.305291122	3.02E-12	postive
PDHB	AC005288.1	0.487348677	3.46E-31	postive
PDHB	AC005324.5	0.303166597	4.34E-12	postive

PDHB	AC005498.2	0.30060842	6.68E-12	postive
PDHB	AC005520.4	0.400273485	1.16E-20	postive
PDHB	AC005562.1	0.542351385	1.40E-39	postive
PDHB	AC005586.1	0.395380738	3.71E-20	postive
PDHB	AC005670.3	0.4333038	2.66E-24	postive
PDHB	AC005696.1	0.387009622	2.60E-19	postive
PDHB	AC005884.2	0.306265294	2.56E-12	postive
PDHB	AC005920.1	0.321278858	1.81E-13	postive
PDHB	AC006001.2	0.39294373	6.57E-20	postive
PDHB	AC006026.3	0.364634584	3.61E-17	postive
PDHB	AC006122.1	0.367717805	1.87E-17	postive
PDHB	AC006126.4	0.353416637	3.72E-16	postive
PDHB	AC006206.2	0.351315417	5.70E-16	postive
PDHB	AC006213.1	0.35699214	1.79E-16	postive
PDHB	AC006252.1	0.469771305	8.22E-29	postive
PDHB	AC006333.1	0.493404023	4.88E-32	postive
PDHB	AC006449.2	0.598891873	5.46E-50	postive
PDHB	AC006449.7	0.505615357	8.31E-34	postive
PDHB	AC006504.5	0.376701943	2.64E-18	postive
PDHB	AC007114.1	0.317668416	3.47E-13	postive
PDHB	AC007228.9	0.430989234	4.94E-24	postive
PDHB	AC007249.3	0.331668506	2.65E-14	postive
PDHB	AC007342.4	0.357824363	1.51E-16	postive
PDHB	AC007383.2	0.527017127	4.39E-37	postive
PDHB	AC007405.4	0.391776705	8.63E-20	postive
PDHB	AC007406.3	0.349044848	9.01E-16	postive
PDHB	AC007406.5	0.44356729	1.62E-25	postive
PDHB	AC007485.1	0.324641234	9.80E-14	postive
PDHB	AC007541.1	0.351177571	5.87E-16	postive
PDHB	AC007773.1	0.342818567	3.10E-15	postive
PDHB	AC007952.2	0.304019852	3.75E-12	postive
PDHB	AC007998.3	0.338322568	7.45E-15	postive
PDHB	AC008014.1	0.482440093	1.65E-30	postive
PDHB	AC008035.1	0.370367769	1.06E-17	postive
PDHB	AC008105.2	0.340860386	4.55E-15	postive
PDHB	AC008124.1	0.372417597	6.78E-18	postive
PDHB	AC008267.5	0.383489057	5.79E-19	postive
PDHB	AC008280.3	0.370597816	1.01E-17	postive
PDHB	AC008393.1	0.37982351	1.32E-18	postive
PDHB	AC008438.1	0.537530102	8.82E-39	postive
PDHB	AC008443.1	0.499662028	6.18E-33	postive
PDHB	AC008443.5	0.318029603	3.25E-13	postive
PDHB	AC008494.3	0.335936258	1.18E-14	postive
PDHB	AC008592.1	0.309893238	1.37E-12	postive
PDHB	AC008608.2	0.356002828	2.19E-16	postive
PDHB	AC008610.1	0.320157173	2.22E-13	postive
PDHB	AC008622.2	0.33466956	1.50E-14	postive
PDHB	AC008669.1	0.321971268	1.60E-13	postive
PDHB	AC008758.4	0.335988084	1.17E-14	postive
PDHB	AC008771.1	0.460390485	1.34E-27	postive

PDHB	AC008915.3	0.552372354	2.79E-41	postive
PDHB	AC008946.1	0.34488652	2.06E-15	postive
PDHB	AC008966.1	0.472680713	3.40E-29	postive
PDHB	AC009061.2	0.432211943	3.56E-24	postive
PDHB	AC009065.3	0.307489817	2.07E-12	postive
PDHB	AC009065.6	0.303166963	4.34E-12	postive
PDHB	AC009065.9	0.369634115	1.24E-17	postive
PDHB	AC009113.1	0.415903364	2.47E-22	postive
PDHB	AC009118.3	0.350763322	6.38E-16	postive
PDHB	AC009126.1	0.475757854	1.32E-29	postive
PDHB	AC009171.2	0.327936407	5.33E-14	postive
PDHB	AC009237.14	0.346765059	1.42E-15	postive
PDHB	AC009309.2	0.379303468	1.48E-18	postive
PDHB	AC009403.1	0.381536829	9.00E-19	postive
PDHB	AC009506.2	0.43762469	8.29E-25	postive
PDHB	AC009690.2	0.338837328	6.74E-15	postive
PDHB	AC009779.3	0.510016302	1.84E-34	postive
PDHB	AC009812.1	0.321008468	1.90E-13	postive
PDHB	AC009831.1	0.406599261	2.50E-21	postive
PDHB	AC009948.3	0.356275351	2.07E-16	postive
PDHB	AC009955.4	0.300741069	6.53E-12	postive
PDHB	AC009961.1	0.324047063	1.09E-13	postive
PDHB	AC010226.1	0.356614051	1.93E-16	postive
PDHB	AC010273.2	0.346315102	1.56E-15	postive
PDHB	AC010273.3	0.421151813	6.47E-23	postive
PDHB	AC010307.2	0.302900363	4.54E-12	postive
PDHB	AC010307.4	0.329690392	3.84E-14	postive
PDHB	AC010319.4	0.326958666	6.39E-14	postive
PDHB	AC010503.5	0.388216862	1.97E-19	postive
PDHB	AC010504.1	0.345490024	1.83E-15	postive
PDHB	AC010608.1	0.336514724	1.05E-14	postive
PDHB	AC010655.4	0.365408681	3.06E-17	postive
PDHB	AC010761.1	0.3215827	1.71E-13	postive
PDHB	AC010761.6	0.301679511	5.58E-12	postive
PDHB	AC010809.1	0.37780526	2.07E-18	postive
PDHB	AC010894.2	0.324356208	1.03E-13	postive
PDHB	AC010969.2	0.487777877	3.01E-31	postive
PDHB	AC011477.2	0.328686416	4.64E-14	postive
PDHB	AC011731.1	0.340484953	4.90E-15	postive
PDHB	AC011978.2	0.406114368	2.81E-21	postive
PDHB	AC012063.1	0.463549128	5.29E-28	postive
PDHB	AC012146.1	0.351947898	5.02E-16	postive
PDHB	AC012184.3	0.322265685	1.51E-13	postive
PDHB	AC012306.2	0.463754664	4.98E-28	postive
PDHB	AC012313.1	0.361876497	6.46E-17	postive
PDHB	AC012442.2	0.391246649	9.76E-20	postive
PDHB	AC012442.3	0.349411056	8.38E-16	postive
PDHB	AC012467.2	0.5688568	3.29E-44	postive
PDHB	AC012510.1	0.304248188	3.61E-12	postive
PDHB	AC012555.1	0.321248188	1.82E-13	postive

PDHB	AC012603.1	0.417355855	1.71E-22	postive
PDHB	AC012615.1	0.340238219	5.14E-15	postive
PDHB	AC012640.4	0.314457602	6.14E-13	postive
PDHB	AC012676.1	0.318848558	2.81E-13	postive
PDHB	AC015818.3	0.332588778	2.23E-14	postive
PDHB	AC015922.2	0.500458918	4.73E-33	postive
PDHB	AC015922.3	0.534075247	3.23E-38	postive
PDHB	AC015982.2	0.428368892	9.88E-24	postive
PDHB	AC016027.1	0.321651111	1.69E-13	postive
PDHB	AC016065.1	0.475201749	1.57E-29	postive
PDHB	AC016355.1	0.343065371	2.96E-15	postive
PDHB	AC016394.2	0.437257487	9.16E-25	postive
PDHB	AC016727.1	0.477186222	8.50E-30	postive
PDHB	AC016747.1	0.444838547	1.14E-25	postive
PDHB	AC016876.2	0.486968157	3.91E-31	postive
PDHB	AC016876.3	0.519435335	6.77E-36	postive
PDHB	AC016888.1	0.313911568	6.77E-13	postive
PDHB	AC017100.1	0.309522686	1.46E-12	postive
PDHB	AC018413.1	0.414011557	3.97E-22	postive
PDHB	AC018521.5	0.600726589	2.31E-50	postive
PDHB	AC018521.6	0.358380005	1.34E-16	postive
PDHB	AC018647.2	0.501495381	3.35E-33	postive
PDHB	AC018690.1	0.325311924	8.66E-14	postive
PDHB	AC018809.2	0.45217895	1.44E-26	postive
PDHB	AC019131.2	0.356056271	2.17E-16	postive
PDHB	AC020661.1	0.326391192	7.10E-14	postive
PDHB	AC020663.2	0.452703193	1.24E-26	postive
PDHB	AC020915.3	0.485462898	6.32E-31	postive
PDHB	AC021016.2	0.424919789	2.44E-23	postive
PDHB	AC021016.3	0.315147112	5.44E-13	postive
PDHB	AC021028.1	0.333174355	1.99E-14	postive
PDHB	AC021148.1	0.300364286	6.96E-12	postive
PDHB	AC021224.1	0.372369986	6.85E-18	postive
PDHB	AC021483.2	0.311288499	1.07E-12	postive
PDHB	AC021851.1	0.318639124	2.92E-13	postive
PDHB	AC022007.1	0.411527751	7.40E-22	postive
PDHB	AC022098.1	0.335181002	1.36E-14	postive
PDHB	AC022150.2	0.31049854	1.23E-12	postive
PDHB	AC022211.3	0.304761624	3.31E-12	postive
PDHB	AC022306.2	0.357384541	1.65E-16	postive
PDHB	AC022364.1	0.328934365	4.43E-14	postive
PDHB	AC022400.3	0.336322532	1.09E-14	postive
PDHB	AC022613.2	0.418413793	1.30E-22	postive
PDHB	AC022916.2	0.318960156	2.75E-13	postive
PDHB	AC023024.1	0.312654794	8.44E-13	postive
PDHB	AC023043.1	0.357498584	1.61E-16	postive
PDHB	AC023043.4	0.315295273	5.30E-13	postive
PDHB	AC023509.3	0.500485135	4.69E-33	postive
PDHB	AC024060.2	0.352015786	4.95E-16	postive
PDHB	AC024267.7	0.330948726	3.03E-14	postive

PDHB	AC024361.2	0.31621117	4.50E-13	postive
PDHB	AC024575.2	0.465837636	2.68E-28	postive
PDHB	AC024896.1	0.35727015	1.69E-16	postive
PDHB	AC025048.4	0.309410967	1.49E-12	postive
PDHB	AC025265.1	0.302675882	4.71E-12	postive
PDHB	AC025682.2	0.347829729	1.15E-15	postive
PDHB	AC026191.1	0.34163154	3.91E-15	postive
PDHB	AC026202.2	0.324241533	1.05E-13	postive
PDHB	AC026369.2	0.334267354	1.62E-14	postive
PDHB	AC026401.3	0.36949651	1.28E-17	postive
PDHB	AC026412.1	0.302209345	5.10E-12	postive
PDHB	AC026471.1	0.441009662	3.29E-25	postive
PDHB	AC026691.1	0.414597288	3.43E-22	postive
PDHB	AC026801.2	0.343145945	2.91E-15	postive
PDHB	AC027097.1	0.548841192	1.13E-40	postive
PDHB	AC027097.2	0.310355464	1.26E-12	postive
PDHB	AC027228.2	0.320451855	2.10E-13	postive
PDHB	AC027307.2	0.379161218	1.53E-18	postive
PDHB	AC027307.3	0.348154221	1.08E-15	postive
PDHB	AC027601.1	0.318234683	3.14E-13	postive
PDHB	AC027644.3	0.409558824	1.21E-21	postive
PDHB	AC034236.2	0.32374489	1.15E-13	postive
PDHB	AC036176.1	0.482110389	1.83E-30	postive
PDHB	AC036214.2	0.310315184	1.27E-12	postive
PDHB	AC037459.2	0.338052398	7.84E-15	postive
PDHB	AC040970.1	0.356127396	2.14E-16	postive
PDHB	AC040977.1	0.344105455	2.41E-15	postive
PDHB	AC046134.2	0.456531659	4.13E-27	postive
PDHB	AC051619.4	0.340090407	5.29E-15	postive
PDHB	AC051619.6	0.420298209	8.05E-23	postive
PDHB	AC053503.1	0.300246288	7.10E-12	postive
PDHB	AC060780.1	0.354594248	2.93E-16	postive
PDHB	AC061992.2	0.317469562	3.60E-13	postive
PDHB	AC064802.1	0.313053894	7.87E-13	postive
PDHB	AC064807.1	0.435040083	1.67E-24	postive
PDHB	AC064807.4	0.307342767	2.13E-12	postive
PDHB	AC064836.3	0.363309265	4.78E-17	postive
PDHB	AC067750.1	0.341161158	4.29E-15	postive
PDHB	AC067838.1	0.318426623	3.03E-13	postive
PDHB	AC067852.2	0.618307637	4.61E-54	postive
PDHB	AC068338.2	0.464028057	4.59E-28	postive
PDHB	AC068338.3	0.333609847	1.84E-14	postive
PDHB	AC068473.5	0.42669275	1.53E-23	postive
PDHB	AC068580.3	0.303714171	3.95E-12	postive
PDHB	AC068790.8	0.334803209	1.46E-14	postive
PDHB	AC068790.9	0.317275739	3.72E-13	postive
PDHB	AC068831.2	0.377950577	2.01E-18	postive
PDHB	AC068870.2	0.467547006	1.61E-28	postive
PDHB	AC068888.1	0.437668472	8.19E-25	postive
PDHB	AC069185.1	0.41183356	6.85E-22	postive

PDHB	AC069307.1	0.308616225	1.71E-12	postive
PDHB	AC069544.1	0.312547784	8.60E-13	postive
PDHB	AC073073.2	0.339982726	5.40E-15	postive
PDHB	AC073195.2	0.327281759	6.02E-14	postive
PDHB	AC073254.1	0.384668892	4.43E-19	postive
PDHB	AC073508.3	0.430456352	5.69E-24	postive
PDHB	AC073529.1	0.330947893	3.04E-14	postive
PDHB	AC073569.2	0.392726936	6.91E-20	postive
PDHB	AC073611.1	0.396930358	2.57E-20	postive
PDHB	AC073869.1	0.32970653	3.83E-14	postive
PDHB	AC073896.2	0.492985961	5.59E-32	postive
PDHB	AC073896.3	0.340009295	5.37E-15	postive
PDHB	AC073896.4	0.323958226	1.11E-13	postive
PDHB	AC074117.1	0.348678397	9.70E-16	postive
PDHB	AC078785.1	0.319071135	2.70E-13	postive
PDHB	AC078881.1	0.320401228	2.12E-13	postive
PDHB	AC079610.1	0.322844264	1.36E-13	postive
PDHB	AC079807.1	0.402086127	7.49E-21	postive
PDHB	AC079834.2	0.3917863	8.61E-20	postive
PDHB	AC079848.1	0.34292659	3.04E-15	postive
PDHB	AC079922.2	0.303966237	3.79E-12	postive
PDHB	AC080112.2	0.47726252	8.30E-30	postive
PDHB	AC083798.2	0.360969742	7.82E-17	postive
PDHB	AC083799.1	0.451156836	1.93E-26	postive
PDHB	AC083964.1	0.380169159	1.22E-18	postive
PDHB	AC084809.1	0.324247563	1.05E-13	postive
PDHB	AC087500.1	0.305970472	2.69E-12	postive
PDHB	AC087501.4	0.44394618	1.46E-25	postive
PDHB	AC087521.3	0.38903815	1.63E-19	postive
PDHB	AC087645.2	0.30527488	3.03E-12	postive
PDHB	AC087741.2	0.336338441	1.09E-14	postive
PDHB	AC090114.2	0.313780408	6.92E-13	postive
PDHB	AC090198.1	0.307842909	1.95E-12	postive
PDHB	AC090204.1	0.481297904	2.36E-30	postive
PDHB	AC090515.3	0.365249242	3.17E-17	postive
PDHB	AC090587.1	0.409017191	1.38E-21	postive
PDHB	AC090587.2	0.320553034	2.06E-13	postive
PDHB	AC090617.5	0.471361412	5.08E-29	postive
PDHB	AC090912.1	0.314512411	6.08E-13	postive
PDHB	AC091153.3	0.342426537	3.35E-15	postive
PDHB	AC091153.4	0.310982233	1.13E-12	postive
PDHB	AC091271.1	0.357707947	1.54E-16	postive
PDHB	AC091390.5	0.389323866	1.52E-19	postive
PDHB	AC091729.3	0.366403586	2.48E-17	postive
PDHB	AC091965.1	0.316206861	4.50E-13	postive
PDHB	AC091982.3	0.356005329	2.19E-16	postive
PDHB	AC092115.4	0.403495667	5.32E-21	postive
PDHB	AC092171.5	0.339587963	5.83E-15	postive
PDHB	AC092279.2	0.33095638	3.03E-14	postive
PDHB	AC092295.2	0.315629384	4.99E-13	postive

PDHB	AC092354.2	0.347957548	1.12E-15	postive
PDHB	AC092368.3	0.359176342	1.14E-16	postive
PDHB	AC092614.1	0.401624038	8.37E-21	postive
PDHB	AC092803.1	0.304222497	3.63E-12	postive
PDHB	AC092944.1	0.31746594	3.60E-13	postive
PDHB	AC092969.1	0.305490619	2.92E-12	postive
PDHB	AC093157.1	0.419209615	1.06E-22	postive
PDHB	AC093249.6	0.338230465	7.58E-15	postive
PDHB	AC093382.1	0.325060996	9.07E-14	postive
PDHB	AC093484.4	0.363033737	5.07E-17	postive
PDHB	AC093503.1	0.301317707	5.93E-12	postive
PDHB	AC093627.5	0.353740542	3.48E-16	postive
PDHB	AC093673.1	0.32871533	4.61E-14	postive
PDHB	AC093726.1	0.365354741	3.10E-17	postive
PDHB	AC093752.1	0.376905254	2.53E-18	postive
PDHB	AC096536.2	0.403562587	5.24E-21	postive
PDHB	AC096649.2	0.306536489	2.44E-12	postive
PDHB	AC096677.1	0.413906554	4.08E-22	postive
PDHB	AC096733.3	0.325067477	9.06E-14	postive
PDHB	AC096772.6	0.460650562	1.24E-27	postive
PDHB	AC097359.2	0.3707356	9.77E-18	postive
PDHB	AC097376.3	0.319281297	2.60E-13	postive
PDHB	AC097468.3	0.431750352	4.03E-24	postive
PDHB	AC097639.1	0.315855896	4.79E-13	postive
PDHB	AC098484.1	0.518112363	1.08E-35	postive
PDHB	AC098484.2	0.348804223	9.46E-16	postive
PDHB	AC098487.1	0.350462057	6.78E-16	postive
PDHB	AC098820.2	0.474705775	1.83E-29	postive
PDHB	AC098820.4	0.344709718	2.14E-15	postive
PDHB	AC098934.1	0.328522341	4.78E-14	postive
PDHB	AC099343.4	0.389284695	1.54E-19	postive
PDHB	AC099778.1	0.396127672	3.11E-20	postive
PDHB	AC099850.2	0.42042244	7.80E-23	postive
PDHB	AC100803.3	0.374360292	4.43E-18	postive
PDHB	AC100810.1	0.506436836	6.28E-34	postive
PDHB	AC100861.1	0.487728926	3.06E-31	postive
PDHB	AC104109.2	0.435957464	1.30E-24	postive
PDHB	AC104113.1	0.373584553	5.25E-18	postive
PDHB	AC104521.1	0.353127496	3.95E-16	postive
PDHB	AC104561.1	0.301877625	5.39E-12	postive
PDHB	AC104596.1	0.300978532	6.28E-12	postive
PDHB	AC104667.2	0.41033554	9.95E-22	postive
PDHB	AC104794.3	0.300602933	6.68E-12	postive
PDHB	AC105020.6	0.308097727	1.87E-12	postive
PDHB	AC105036.3	0.457335776	3.27E-27	postive
PDHB	AC105285.1	0.392134027	7.94E-20	postive
PDHB	AC105942.1	0.351286133	5.74E-16	postive
PDHB	AC106739.1	0.359359392	1.09E-16	postive
PDHB	AC106786.2	0.341596263	3.94E-15	postive
PDHB	AC106791.1	0.363847891	4.27E-17	postive

PDHB	AC106795.1	0.468958862	1.05E-28	postive
PDHB	AC107027.3	0.36851837	1.58E-17	postive
PDHB	AC107032.2	0.387824763	2.15E-19	postive
PDHB	AC107068.1	0.403434042	5.40E-21	postive
PDHB	AC107214.1	0.384765754	4.33E-19	postive
PDHB	AC107214.2	0.375931213	3.14E-18	postive
PDHB	AC107375.1	0.330569088	3.26E-14	postive
PDHB	AC107959.1	0.305425628	2.95E-12	postive
PDHB	AC107959.3	0.31825519	3.12E-13	postive
PDHB	AC107982.3	0.436043021	1.27E-24	postive
PDHB	AC108488.1	0.358308243	1.36E-16	postive
PDHB	AC108860.2	0.376956651	2.50E-18	postive
PDHB	AC109322.1	0.374681654	4.13E-18	postive
PDHB	AC109347.1	0.406447979	2.59E-21	postive
PDHB	AC109454.2	0.364690898	3.57E-17	postive
PDHB	AC110079.1	0.308096474	1.87E-12	postive
PDHB	AC112220.2	0.518380246	9.85E-36	postive
PDHB	AC112236.2	0.344503806	2.23E-15	postive
PDHB	AC112491.1	0.334416235	1.57E-14	postive
PDHB	AC112694.1	0.323400909	1.23E-13	postive
PDHB	AC114271.1	0.448915422	3.64E-26	postive
PDHB	AC114341.1	0.384742591	4.36E-19	postive
PDHB	AC114810.1	0.508786525	2.81E-34	postive
PDHB	AC115284.1	0.333880821	1.74E-14	postive
PDHB	AC115618.1	0.421080864	6.59E-23	postive
PDHB	AC115618.2	0.516084067	2.22E-35	postive
PDHB	AC116036.2	0.375264556	3.63E-18	postive
PDHB	AC116913.1	0.40008321	1.21E-20	postive
PDHB	AC116914.2	0.30614083	2.61E-12	postive
PDHB	AC120049.1	0.371226985	8.78E-18	postive
PDHB	AC121338.2	0.370342938	1.06E-17	postive
PDHB	AC123768.2	0.392431115	7.41E-20	postive
PDHB	AC123768.3	0.303574909	4.05E-12	postive
PDHB	AC124016.2	0.354779206	2.82E-16	postive
PDHB	AC124242.1	0.481007515	2.58E-30	postive
PDHB	AC124248.1	0.339195476	6.29E-15	postive
PDHB	AC124283.2	0.339578741	5.84E-15	postive
PDHB	AC125257.1	0.548601281	1.24E-40	postive
PDHB	AC125437.1	0.339341529	6.11E-15	postive
PDHB	AC125494.1	0.392345036	7.56E-20	postive
PDHB	AC126365.1	0.346039015	1.64E-15	postive
PDHB	AC127537.1	0.309948014	1.35E-12	postive
PDHB	AC129492.4	0.310011289	1.34E-12	postive
PDHB	AC129507.2	0.328622584	4.69E-14	postive
PDHB	AC129507.3	0.375572511	3.39E-18	postive
PDHB	AC130343.2	0.301094916	6.15E-12	postive
PDHB	AC131009.3	0.420265151	8.12E-23	postive
PDHB	AC131235.3	0.312852654	8.15E-13	postive
PDHB	AC132938.4	0.426581835	1.58E-23	postive
PDHB	AC133528.2	0.421090294	6.57E-23	postive

PDHB	AC133552.2	0.342911905	3.05E-15	postive
PDHB	AC134878.2	0.35546388	2.45E-16	postive
PDHB	AC135048.1	0.306118287	2.62E-12	postive
PDHB	AC135048.3	0.31509304	5.49E-13	postive
PDHB	AC135050.1	0.469494286	8.93E-29	postive
PDHB	AC135050.5	0.3019833	5.30E-12	postive
PDHB	AC135050.6	0.406579279	2.51E-21	postive
PDHB	AC135178.4	0.398107565	1.94E-20	postive
PDHB	AC136475.2	0.319611135	2.45E-13	postive
PDHB	AC136604.3	0.465953155	2.59E-28	postive
PDHB	AC137630.1	0.414052921	3.93E-22	postive
PDHB	AC137630.2	0.338831835	6.75E-15	postive
PDHB	AC137932.1	0.350167566	7.19E-16	postive
PDHB	AC137932.2	0.383955364	5.21E-19	postive
PDHB	AC138207.8	0.3120923	9.32E-13	postive
PDHB	AC138696.2	0.35335163	3.77E-16	postive
PDHB	AC139149.1	0.321401698	1.77E-13	postive
PDHB	AC139530.1	0.326287296	7.24E-14	postive
PDHB	AC139768.1	0.466810422	2.00E-28	postive
PDHB	AC139887.2	0.422460862	4.62E-23	postive
PDHB	AC141586.1	0.401994181	7.66E-21	postive
PDHB	AC142472.1	0.30295923	4.49E-12	postive
PDHB	AC145124.1	0.36642472	2.47E-17	postive
PDHB	AC231981.1	0.504271561	1.31E-33	postive
PDHB	AC233992.3	0.313791698	6.91E-13	postive
PDHB	AC242376.2	0.308989876	1.60E-12	postive
PDHB	AC242426.2	0.337301308	9.07E-15	postive
PDHB	AC243919.1	0.466396193	2.27E-28	postive
PDHB	AC244090.1	0.342454778	3.33E-15	postive
PDHB	AC244669.1	0.40826019	1.66E-21	postive
PDHB	AC245140.2	0.336050922	1.15E-14	postive
PDHB	AC245297.4	0.376888499	2.54E-18	postive
PDHB	ACVR2B-AS1	0.526637317	5.05E-37	postive
PDHB	AD000671.3	0.304039957	3.74E-12	postive
PDHB	ADGRD1-AS1	0.417984637	1.45E-22	postive
PDHB	AF001548.2	0.339660928	5.75E-15	postive
PDHB	AF111167.2	0.321000858	1.90E-13	postive
PDHB	AF131216.4	0.417342888	1.71E-22	postive
PDHB	AFG3L1P	0.34528277	1.91E-15	postive
PDHB	AL008721.1	0.301714632	5.54E-12	postive
PDHB	AL008726.1	0.300322584	7.01E-12	postive
PDHB	AL009179.1	0.329528961	3.96E-14	postive
PDHB	AL020996.1	0.391046928	1.02E-19	postive
PDHB	AL022157.1	0.31643854	4.32E-13	postive
PDHB	AL022311.1	0.325192984	8.85E-14	postive
PDHB	AL023284.4	0.304630463	3.38E-12	postive
PDHB	AL024498.1	0.37965998	1.37E-18	postive
PDHB	AL024507.3	0.345399457	1.86E-15	postive
PDHB	AL031123.2	0.346738183	1.43E-15	postive
PDHB	AL031282.1	0.341198938	4.26E-15	postive

PDHB	AL031282.2	0.331924695	2.53E-14	postive
PDHB	AL031775.1	0.405109753	3.60E-21	postive
PDHB	AL031847.1	0.326187417	7.37E-14	postive
PDHB	AL031963.3	0.316368523	4.38E-13	postive
PDHB	AL031985.3	0.413400117	4.63E-22	postive
PDHB	AL035587.1	0.362468522	5.71E-17	postive
PDHB	AL035661.1	0.401640128	8.34E-21	postive
PDHB	AL049597.2	0.434254899	2.06E-24	postive
PDHB	AL049779.1	0.301868755	5.40E-12	postive
PDHB	AL049796.1	0.419362188	1.02E-22	postive
PDHB	AL049840.4	0.353852575	3.41E-16	postive
PDHB	AL049840.5	0.33602019	1.16E-14	postive
PDHB	AL049840.6	0.317236265	3.75E-13	postive
PDHB	AL050309.1	0.500655411	4.43E-33	postive
PDHB	AL050341.2	0.33110074	2.95E-14	postive
PDHB	AL078581.2	0.37668557	2.65E-18	postive
PDHB	AL078644.2	0.434789853	1.79E-24	postive
PDHB	AL080250.1	0.418805969	1.18E-22	postive
PDHB	AL080276.2	0.322007585	1.59E-13	postive
PDHB	AL109917.1	0.385778506	3.44E-19	postive
PDHB	AL117332.1	0.44761882	5.24E-26	postive
PDHB	AL117336.2	0.328858373	4.49E-14	postive
PDHB	AL118506.1	0.384499435	4.60E-19	postive
PDHB	AL118516.1	0.334935392	1.43E-14	postive
PDHB	AL118558.3	0.404625035	4.05E-21	postive
PDHB	AL121603.2	0.355089136	2.64E-16	postive
PDHB	AL121944.2	0.564355513	2.15E-43	postive
PDHB	AL122010.1	0.409462012	1.24E-21	postive
PDHB	AL122023.3	0.394102893	5.01E-20	postive
PDHB	AL122035.1	0.342443753	3.34E-15	postive
PDHB	AL132639.3	0.302405876	4.93E-12	postive
PDHB	AL132712.1	0.327255559	6.05E-14	postive
PDHB	AL132857.1	0.384549327	4.55E-19	postive
PDHB	AL133153.2	0.363808226	4.30E-17	postive
PDHB	AL133215.2	0.347179788	1.31E-15	postive
PDHB	AL133353.1	0.316617919	4.19E-13	postive
PDHB	AL133355.1	0.35945182	1.07E-16	postive
PDHB	AL133520.1	0.323351971	1.24E-13	postive
PDHB	AL135925.1	0.440569569	3.71E-25	postive
PDHB	AL136038.7	0.31094847	1.14E-12	postive
PDHB	AL136040.1	0.436127128	1.24E-24	postive
PDHB	AL137127.1	0.359064236	1.16E-16	postive
PDHB	AL138756.1	0.342430512	3.35E-15	postive
PDHB	AL138762.2	0.430198044	6.09E-24	postive
PDHB	AL138921.1	0.32554422	8.30E-14	postive
PDHB	AL138921.2	0.33943015	6.01E-15	postive
PDHB	AL139011.1	0.345242254	1.92E-15	postive
PDHB	AL139021.2	0.323102446	1.30E-13	postive
PDHB	AL139035.1	0.344563851	2.20E-15	postive
PDHB	AL139289.1	0.362182934	6.06E-17	postive

PDHB	AL139384.1	0.373522706	5.32E-18	postive
PDHB	AL139397.1	0.342636119	3.22E-15	postive
PDHB	AL139420.2	0.300193422	7.16E-12	postive
PDHB	AL158212.3	0.301780702	5.48E-12	postive
PDHB	AL160006.1	0.339840995	5.55E-15	postive
PDHB	AL160313.2	0.329587983	3.92E-14	postive
PDHB	AL161421.1	0.4296017	7.13E-24	postive
PDHB	AL161729.1	0.370899057	9.43E-18	postive
PDHB	AL161729.3	0.32064935	2.03E-13	postive
PDHB	AL161772.1	0.395397287	3.69E-20	postive
PDHB	AL162171.1	0.330968901	3.02E-14	postive
PDHB	AL162231.2	0.455910481	4.94E-27	postive
PDHB	AL162258.2	0.333164877	2.00E-14	postive
PDHB	AL162377.1	0.484376288	8.92E-31	postive
PDHB	AL162595.1	0.362356833	5.84E-17	postive
PDHB	AL162632.3	0.356179659	2.11E-16	postive
PDHB	AL353194.1	0.380097926	1.24E-18	postive
PDHB	AL353593.1	0.311896452	9.64E-13	postive
PDHB	AL353708.1	0.465576191	2.90E-28	postive
PDHB	AL353708.3	0.317000794	3.91E-13	postive
PDHB	AL353743.1	0.509453072	2.23E-34	postive
PDHB	AL353796.1	0.312027936	9.42E-13	postive
PDHB	AL354696.1	0.394891811	4.16E-20	postive
PDHB	AL354696.2	0.436617564	1.09E-24	postive
PDHB	AL354718.3	0.304569702	3.42E-12	postive
PDHB	AL354892.2	0.498929987	7.88E-33	postive
PDHB	AL354920.1	0.533885929	3.47E-38	postive
PDHB	AL355001.2	0.424798149	2.52E-23	postive
PDHB	AL355574.1	0.337028276	9.55E-15	postive
PDHB	AL355802.3	0.352685359	4.32E-16	postive
PDHB	AL356235.1	0.341187678	4.27E-15	postive
PDHB	AL356481.1	0.411699117	7.09E-22	postive
PDHB	AL356488.3	0.402472002	6.82E-21	postive
PDHB	AL356599.1	0.326209224	7.34E-14	postive
PDHB	AL357140.2	0.341587342	3.95E-15	postive
PDHB	AL358472.2	0.412780012	5.41E-22	postive
PDHB	AL359198.1	0.314242307	6.38E-13	postive
PDHB	AL359232.1	0.373380264	5.49E-18	postive
PDHB	AL359397.1	0.369723853	1.22E-17	postive
PDHB	AL359513.1	0.307271155	2.15E-12	postive
PDHB	AL359541.2	0.357765034	1.52E-16	postive
PDHB	AL359643.3	0.42475166	2.55E-23	postive
PDHB	AL359715.3	0.478043346	6.51E-30	postive
PDHB	AL359921.2	0.330279525	3.44E-14	postive
PDHB	AL365181.3	0.318466062	3.01E-13	postive
PDHB	AL365330.1	0.309521232	1.46E-12	postive
PDHB	AL390198.1	0.367779294	1.85E-17	postive
PDHB	AL390728.4	0.311718852	9.95E-13	postive
PDHB	AL390955.2	0.355082861	2.65E-16	postive
PDHB	AL391069.2	0.392470727	7.34E-20	postive

PDHB	AL391121.1	0.466487856	2.21E-28	postive
PDHB	AL391684.1	0.312746582	8.31E-13	postive
PDHB	AL391832.2	0.313844399	6.85E-13	postive
PDHB	AL391834.2	0.379957494	1.28E-18	postive
PDHB	AL392172.1	0.384821023	4.28E-19	postive
PDHB	AL441992.2	0.435979062	1.30E-24	postive
PDHB	AL442663.3	0.31151841	1.03E-12	postive
PDHB	AL445645.1	0.318273212	3.11E-13	postive
PDHB	AL450326.1	0.366532322	2.41E-17	postive
PDHB	AL450998.2	0.423675314	3.37E-23	postive
PDHB	AL451085.2	0.339705309	5.70E-15	postive
PDHB	AL451085.3	0.453398126	1.02E-26	postive
PDHB	AL451165.2	0.449557301	3.03E-26	postive
PDHB	AL512408.1	0.330392276	3.37E-14	postive
PDHB	AL512625.1	0.42226871	4.85E-23	postive
PDHB	AL513165.1	0.307137952	2.20E-12	postive
PDHB	AL513550.1	0.44458349	1.22E-25	postive
PDHB	AL590617.2	0.351033156	6.04E-16	postive
PDHB	AL590666.2	0.320655976	2.03E-13	postive
PDHB	AL592166.1	0.450204607	2.53E-26	postive
PDHB	AL596202.1	0.353591594	3.59E-16	postive
PDHB	AL603839.2	0.324529648	1.00E-13	postive
PDHB	AL603839.3	0.30384435	3.87E-12	postive
PDHB	AL604028.1	0.348243745	1.06E-15	postive
PDHB	AL606760.1	0.328119457	5.15E-14	postive
PDHB	AL606834.1	0.354047881	3.27E-16	postive
PDHB	AL645568.1	0.401744004	8.13E-21	postive
PDHB	AL645728.1	0.338007686	7.91E-15	postive
PDHB	AL671710.1	0.35233773	4.64E-16	postive
PDHB	AL731566.2	0.36718013	2.10E-17	postive
PDHB	AL731571.1	0.31066423	1.20E-12	postive
PDHB	AL731577.2	0.453020381	1.13E-26	postive
PDHB	AL732314.8	0.421295821	6.23E-23	postive
PDHB	AL807752.5	0.317945185	3.30E-13	postive
PDHB	AL928654.1	0.310888121	1.15E-12	postive
PDHB	ALG1L13P	0.309641917	1.43E-12	postive
PDHB	ALOX12-AS1	0.446981485	6.26E-26	postive
PDHB	ALOX12P2	0.354245262	3.14E-16	postive
PDHB	AMZ2P1	0.448230893	4.41E-26	postive
PDHB	ANO7L1	0.353018373	4.04E-16	postive
PDHB	ANP32AP1	0.357761361	1.52E-16	postive
PDHB	ANP32BP1	0.319763995	2.38E-13	postive
PDHB	AP000254.2	0.36823864	1.67E-17	postive
PDHB	AP000442.1	0.421181922	6.42E-23	postive
PDHB	AP000487.1	0.326816273	6.56E-14	postive
PDHB	AP000688.1	0.325949178	7.70E-14	postive
PDHB	AP000704.2	0.360600064	8.45E-17	postive
PDHB	AP000759.1	0.397175135	2.42E-20	postive
PDHB	AP000894.4	0.328991422	4.38E-14	postive
PDHB	AP001033.3	0.345230713	1.93E-15	postive

PDHB	AP001107.1	0.390348728	1.20E-19	postive
PDHB	AP001160.1	0.301464409	5.78E-12	postive
PDHB	AP001160.3	0.50582498	7.74E-34	postive
PDHB	AP001267.3	0.318170951	3.17E-13	postive
PDHB	AP001318.2	0.340878423	4.53E-15	postive
PDHB	AP001372.2	0.383136408	6.27E-19	postive
PDHB	AP001453.4	0.426084436	1.80E-23	postive
PDHB	AP001462.1	0.323284616	1.26E-13	postive
PDHB	AP001469.3	0.301259735	5.99E-12	postive
PDHB	AP001486.2	0.335546095	1.27E-14	postive
PDHB	AP001505.1	0.305168434	3.09E-12	postive
PDHB	AP001527.2	0.301659404	5.60E-12	postive
PDHB	AP001542.3	0.38446272	4.64E-19	postive
PDHB	AP001816.1	0.485968952	5.38E-31	postive
PDHB	AP001922.5	0.339312252	6.15E-15	postive
PDHB	AP001972.1	0.304681837	3.35E-12	postive
PDHB	AP002360.2	0.360773199	8.15E-17	postive
PDHB	AP002387.2	0.40048079	1.10E-20	postive
PDHB	AP002748.4	0.455785359	5.12E-27	postive
PDHB	AP002840.2	0.370839352	9.55E-18	postive
PDHB	AP003068.1	0.34898602	9.12E-16	postive
PDHB	AP003068.2	0.345390062	1.87E-15	postive
PDHB	AP003352.1	0.405934785	2.94E-21	postive
PDHB	AP003486.1	0.303235262	4.29E-12	postive
PDHB	AP003721.4	0.318041341	3.25E-13	postive
PDHB	AP004609.5	0.349398861	8.40E-16	postive
PDHB	AP005329.1	0.353468963	3.68E-16	postive
PDHB	AP005329.2	0.408388597	1.61E-21	postive
PDHB	AP005432.2	0.31588158	4.77E-13	postive
PDHB	AP005482.4	0.523977121	1.33E-36	postive
PDHB	AP006621.3	0.3157229	4.91E-13	postive
PDHB	APTR	0.371446823	8.37E-18	postive
PDHB	ARF4-AS1	0.477146452	8.60E-30	postive
PDHB	ARHGAP5-AS1	0.398756065	1.66E-20	postive
PDHB	ARMCX5-GPRASP2	0.57468106	2.77E-45	postive
PDHB	ARRDC1-AS1	0.473246961	2.86E-29	postive
PDHB	ASB16-AS1	0.387009764	2.60E-19	postive
PDHB	ASH1L-AS1	0.389437111	1.48E-19	postive
PDHB	ATF4P4	0.419831106	9.08E-23	postive
PDHB	ATP1A1-AS1	0.363544832	4.55E-17	postive
PDHB	ATP2A1-AS1	0.321502621	1.74E-13	postive
PDHB	ATP2B1-AS1	0.364291522	3.88E-17	postive
PDHB	ATP5F1AP2	0.336800664	9.98E-15	postive
PDHB	ATXN2-AS	0.387959057	2.09E-19	postive
PDHB	B4GALT1-AS1	0.305289349	3.02E-12	postive
PDHB	BAALC-AS1	0.424841684	2.49E-23	postive
PDHB	BACE1-AS	0.345352123	1.88E-15	postive
PDHB	BAIAP2-DT	0.47961255	4.00E-30	postive
PDHB	BCAR3-AS1	0.306197828	2.59E-12	postive
PDHB	BCDIN3D-AS1	0.392161156	7.89E-20	postive

PDHB	BDNF-AS	0.333609197	1.84E-14	postive
PDHB	BEND3P1	0.345684024	1.76E-15	postive
PDHB	BHLHE40-AS1	0.420280306	8.09E-23	postive
PDHB	BMS1P20	0.424256412	2.90E-23	postive
PDHB	BNIP3P1	0.334737439	1.48E-14	postive
PDHB	BOLA3-AS1	0.317026286	3.89E-13	postive
PDHB	BTG3-AS1	0.309319368	1.51E-12	postive
PDHB	BTN2A3P	0.332588323	2.23E-14	postive
PDHB	BTNL10	0.393900621	5.25E-20	postive
PDHB	BX284668.5	0.409145922	1.34E-21	postive
PDHB	BX322557.10	0.356123097	2.14E-16	postive
PDHB	BX537318.1	0.388884717	1.69E-19	postive
PDHB	BX539320.1	0.354598607	2.92E-16	postive
PDHB	BX649632.1	0.346261631	1.57E-15	postive
PDHB	C10orf25	0.35592388	2.23E-16	postive
PDHB	C15orf56	0.373547859	5.29E-18	postive
PDHB	C18orf65	0.478205318	6.19E-30	postive
PDHB	C1orf229	0.329132345	4.27E-14	postive
PDHB	C21orf62-AS1	0.321323639	1.80E-13	postive
PDHB	C22orf46	0.375374176	3.55E-18	postive
PDHB	C2CD4D-AS1	0.402932188	6.10E-21	postive
PDHB	C5orf66	0.310919157	1.14E-12	postive
PDHB	C6orf99	0.372789784	6.25E-18	postive
PDHB	C8orf37-AS1	0.347646557	1.19E-15	postive
PDHB	C9orf147	0.318854751	2.80E-13	postive
PDHB	CA5BP1	0.511721641	1.02E-34	postive
PDHB	CARD8-AS1	0.324651955	9.78E-14	postive
PDHB	CATIP-AS1	0.343086964	2.94E-15	postive
PDHB	CATSPER2P1	0.532651286	5.50E-38	postive
PDHB	CCDC183-AS1	0.377876793	2.04E-18	postive
PDHB	CCNT2-AS1	0.551260592	4.33E-41	postive
PDHB	CCT6P1	0.435643394	1.42E-24	postive
PDHB	CCT6P3	0.332948948	2.08E-14	postive
PDHB	CD27-AS1	0.440356811	3.93E-25	postive
PDHB	CD99P1	0.390599558	1.13E-19	postive
PDHB	CDC37L1-DT	0.420685982	7.29E-23	postive
PDHB	CENPBD1P1	0.556058831	6.38E-42	postive
PDHB	CERNA1	0.350601755	6.59E-16	postive
PDHB	CFL1P1	0.367659894	1.89E-17	postive
PDHB	CHKB-DT	0.359389128	1.09E-16	postive
PDHB	CHL1-AS2	0.343377369	2.78E-15	postive
PDHB	CHROMR	0.373833282	4.97E-18	postive
PDHB	CIDECP1	0.620011334	1.96E-54	postive
PDHB	CKMT2-AS1	0.489436848	1.77E-31	postive
PDHB	CNNM3-DT	0.36394523	4.18E-17	postive
PDHB	COA6-AS1	0.337669492	8.44E-15	postive
PDHB	COX10-AS1	0.439346545	5.19E-25	postive
PDHB	CR381653.1	0.395146133	3.92E-20	postive
PDHB	CRNDE	0.453505934	9.86E-27	postive
PDHB	CROCCP2	0.339464586	5.97E-15	postive

PDHB	CRTC3-AS1	0.375620677	3.36E-18	postive
PDHB	CRYBB2P1	0.349090177	8.93E-16	postive
PDHB	CRYZL2P	0.478540285	5.58E-30	postive
PDHB	CTA-14H9.5	0.3209767	1.91E-13	postive
PDHB	CTA-989H11.1	0.390525076	1.15E-19	postive
PDHB	CTBP1-DT	0.566460826	8.98E-44	postive
PDHB	CTD-2134A5.3	0.325726218	8.03E-14	postive
PDHB	CTD-2245F17.3	0.300849363	6.41E-12	postive
PDHB	CTD-2521M24.9	0.30607388	2.64E-12	postive
PDHB	CUTALP	0.300568942	6.72E-12	postive
PDHB	CYTOR	0.34600279	1.65E-15	postive
PDHB	DANCR	0.450327281	2.44E-26	postive
PDHB	DARS-AS1	0.519040402	7.79E-36	postive
PDHB	DBIL5P	0.389355874	1.51E-19	postive
PDHB	DCST1-AS1	0.362768121	5.36E-17	postive
PDHB	DCXR-DT	0.304755941	3.31E-12	postive
PDHB	DENND10P1	0.334067524	1.68E-14	postive
PDHB	DGCR11	0.489223842	1.89E-31	postive
PDHB	DHCR24-DT	0.374075415	4.72E-18	postive
PDHB	DHRS4-AS1	0.600684753	2.36E-50	postive
PDHB	DHRSX-IT1	0.301176818	6.07E-12	postive
PDHB	DLEU1	0.444293581	1.33E-25	postive
PDHB	DLGAP1-AS1	0.480113924	3.42E-30	postive
PDHB	DM1-AS	0.356316224	2.06E-16	postive
PDHB	DNAJC3-DT	0.318876508	2.79E-13	postive
PDHB	DSCAM-AS1	0.316559703	4.23E-13	postive
PDHB	DSCAS	0.30044111	6.87E-12	postive
PDHB	DSG2-AS1	0.340751978	4.65E-15	postive
PDHB	DSTNP2	0.398976716	1.58E-20	postive
PDHB	DYRK3-AS1	0.355555814	2.40E-16	postive
PDHB	EBLN3P	0.537310994	9.58E-39	postive
PDHB	ECI2-DT	0.337476751	8.76E-15	postive
PDHB	EDRF1-DT	0.379867603	1.31E-18	postive
PDHB	EIF1B-AS1	0.508268559	3.36E-34	postive
PDHB	EIF2AK3-DT	0.326091131	7.50E-14	postive
PDHB	EIF3J-DT	0.561741886	6.33E-43	postive
PDHB	ELF3-AS1	0.430628272	5.43E-24	postive
PDHB	ELOA-AS1	0.405899353	2.97E-21	postive
PDHB	EMC1-AS1	0.424521876	2.70E-23	postive
PDHB	EMC3-AS1	0.352409072	4.57E-16	postive
PDHB	EMSLR	0.3169669	3.93E-13	postive
PDHB	ENTPD3-AS1	0.615525587	1.84E-53	postive
PDHB	EP400P1	0.388669628	1.77E-19	postive
PDHB	EPB41L4A-AS1	0.453392022	1.02E-26	postive
PDHB	EPB41L4A-DT	0.373129492	5.80E-18	postive
PDHB	EPCAM-DT	0.310923327	1.14E-12	postive
PDHB	EXOSC10-AS1	0.346249051	1.58E-15	postive
PDHB	FAHD2CP	0.309006412	1.60E-12	postive
PDHB	FAM111A-DT	0.406907919	2.32E-21	postive
PDHB	FAM157B	0.327211837	6.10E-14	postive

PDHB	FAM21FP	0.302698777	4.70E-12	postive
PDHB	FAM66B	0.307573638	2.04E-12	postive
PDHB	FAM66D	0.372504613	6.65E-18	postive
PDHB	FAM86B3P	0.385392627	3.76E-19	postive
PDHB	FAM86C2P	0.585383032	2.58E-47	postive
PDHB	FAM86DP	0.541014759	2.34E-39	postive
PDHB	FAM86EP	0.611506601	1.33E-52	postive
PDHB	FAM86FP	0.392032128	8.13E-20	postive
PDHB	FAM86HP	0.302011099	5.27E-12	postive
PDHB	FAM86JP	0.504239606	1.33E-33	postive
PDHB	FBXL19-AS1	0.313382791	7.43E-13	postive
PDHB	FGD5-AS1	0.673753707	1.98E-67	postive
PDHB	FGF14-AS2	0.32358301	1.19E-13	postive
PDHB	FGF7P3	0.329440926	4.03E-14	postive
PDHB	FLVCR1-DT	0.373695844	5.12E-18	postive
PDHB	FOXP4-AS1	0.352821933	4.20E-16	postive
PDHB	FRG1HP	0.577209889	9.32E-46	postive
PDHB	FTH1P10	0.312207947	9.13E-13	postive
PDHB	FZD4-DT	0.432633021	3.18E-24	postive
PDHB	GAPDHP72	0.306421074	2.49E-12	postive
PDHB	GAPLINC	0.323447608	1.22E-13	postive
PDHB	GAS5	0.438040925	7.40E-25	postive
PDHB	GAS5-AS1	0.333764495	1.78E-14	postive
PDHB	GCC2-AS1	0.367962284	1.78E-17	postive
PDHB	GGTA1P	0.306160472	2.60E-12	postive
PDHB	GLIDR	0.497310227	1.35E-32	postive
PDHB	GLIS2-AS1	0.309419286	1.48E-12	postive
PDHB	GLUD1P2	0.311174456	1.09E-12	postive
PDHB	GMDS-DT	0.396372118	2.93E-20	postive
PDHB	GNRHR2	0.4587141	2.19E-27	postive
PDHB	GOLGA2P7	0.34279904	3.11E-15	postive
PDHB	GPRC5D-AS1	0.40483909	3.84E-21	postive
PDHB	GSEC	0.306627794	2.40E-12	postive
PDHB	GTF2H2B	0.302094666	5.20E-12	postive
PDHB	GTF2IP1	0.451089436	1.96E-26	postive
PDHB	GTF2IP4	0.395748856	3.40E-20	postive
PDHB	GTSE1-DT	0.304109893	3.70E-12	postive
PDHB	GUSBP1	0.311236229	1.08E-12	postive
PDHB	GUSBP2	0.395694104	3.44E-20	postive
PDHB	GYG2P1	0.326506841	6.95E-14	postive
PDHB	H2AZ2P1	0.393020953	6.45E-20	postive
PDHB	H2BC19P	0.32833668	4.95E-14	postive
PDHB	H2BC20P	0.310692269	1.19E-12	postive
PDHB	HCG11	0.340613849	4.77E-15	postive
PDHB	HCG17	0.316469454	4.30E-13	postive
PDHB	HCG18	0.541456907	1.98E-39	postive
PDHB	HCG25	0.430500044	5.62E-24	postive
PDHB	HEIH	0.504490666	1.22E-33	postive
PDHB	HERC2P9	0.343045559	2.97E-15	postive
PDHB	HM13-IT1	0.31272976	8.33E-13	postive

PDHB	HMGB1P5	0.511844994	9.77E-35	postive
PDHB	HNRNPA1P6	0.349801145	7.74E-16	postive
PDHB	HSD17B7P2	0.340371479	5.00E-15	postive
PDHB	HSP90AA4P	0.348096865	1.09E-15	postive
PDHB	HSP90AB2P	0.385432126	3.72E-19	postive
PDHB	IDH1-AS1	0.358973239	1.19E-16	postive
PDHB	IER3-AS1	0.326246896	7.29E-14	postive
PDHB	IL6R-AS1	0.399935595	1.26E-20	postive
PDHB	ILF3-DT	0.440167906	4.14E-25	postive
PDHB	IQCH-AS1	0.473826208	2.39E-29	postive
PDHB	IRAIN	0.329251786	4.17E-14	postive
PDHB	ITFG2-AS1	0.469929163	7.83E-29	postive
PDHB	ITGA9-AS1	0.33741532	8.87E-15	postive
PDHB	ITPR1-DT	0.344501789	2.23E-15	postive
PDHB	JPX	0.469856582	8.01E-29	postive
PDHB	KCTD21-AS1	0.337687719	8.42E-15	postive
PDHB	KIF9-AS1	0.320728548	2.00E-13	postive
PDHB	KMT2E-AS1	0.362795444	5.33E-17	postive
PDHB	KTN1-AS1	0.447972149	4.74E-26	postive
PDHB	LAMTOR5-AS1	0.328169588	5.10E-14	postive
PDHB	LINC00205	0.36760917	1.92E-17	postive
PDHB	LINC00239	0.400841791	1.01E-20	postive
PDHB	LINC00240	0.359795224	9.99E-17	postive
PDHB	LINC002481	0.397167413	2.43E-20	postive
PDHB	LINC00271	0.344195759	2.37E-15	postive
PDHB	LINC00294	0.320843431	1.96E-13	postive
PDHB	LINC00324	0.436436152	1.14E-24	postive
PDHB	LINC00339	0.528481326	2.57E-37	postive
PDHB	LINC00449	0.35193304	5.03E-16	postive
PDHB	LINC00467	0.566765931	7.90E-44	postive
PDHB	LINC00471	0.312146916	9.23E-13	postive
PDHB	LINC00473	0.302452446	4.90E-12	postive
PDHB	LINC00476	0.466547127	2.17E-28	postive
PDHB	LINC00511	0.31887657	2.79E-13	postive
PDHB	LINC00526	0.347171915	1.31E-15	postive
PDHB	LINC00602	0.315752891	4.88E-13	postive
PDHB	LINC00623	0.305737422	2.80E-12	postive
PDHB	LINC00630	0.35129614	5.73E-16	postive
PDHB	LINC00638	0.363338855	4.75E-17	postive
PDHB	LINC00654	0.341929812	3.69E-15	postive
PDHB	LINC00665	0.307412765	2.10E-12	postive
PDHB	LINC00667	0.443280203	1.76E-25	postive
PDHB	LINC00680	0.452911116	1.17E-26	postive
PDHB	LINC00847	0.398087894	1.95E-20	postive
PDHB	LINC00857	0.382991055	6.48E-19	postive
PDHB	LINC00863	0.510477736	1.57E-34	postive
PDHB	LINC00869	0.318111774	3.20E-13	postive
PDHB	LINC00888	0.432200955	3.57E-24	postive
PDHB	LINC00909	0.577114653	9.72E-46	postive
PDHB	LINC00910	0.436059953	1.27E-24	postive

PDHB	LINC00921	0.36301338	5.09E-17	postive
PDHB	LINC00933	0.419130735	1.09E-22	postive
PDHB	LINC00957	0.388639981	1.78E-19	postive
PDHB	LINC00963	0.54154934	1.91E-39	postive
PDHB	LINC01003	0.380372075	1.17E-18	postive
PDHB	LINC01011	0.43507809	1.65E-24	postive
PDHB	LINC01023	0.338061446	7.83E-15	postive
PDHB	LINC01058	0.31683044	4.03E-13	postive
PDHB	LINC01128	0.460885829	1.16E-27	postive
PDHB	LINC01134	0.307294966	2.14E-12	postive
PDHB	LINC01137	0.413518152	4.50E-22	postive
PDHB	LINC01145	0.34680152	1.41E-15	postive
PDHB	LINC01184	0.570695642	1.51E-44	postive
PDHB	LINC01220	0.429204931	7.92E-24	postive
PDHB	LINC01270	0.406819368	2.37E-21	postive
PDHB	LINC01271	0.363417869	4.67E-17	postive
PDHB	LINC01278	0.445340298	9.91E-26	postive
PDHB	LINC01315	0.331952357	2.51E-14	postive
PDHB	LINC01521	0.356462155	1.99E-16	postive
PDHB	LINC01560	0.435467492	1.49E-24	postive
PDHB	LINC01578	0.459579712	1.70E-27	postive
PDHB	LINC01607	0.315883497	4.77E-13	postive
PDHB	LINC01635	0.34257728	3.25E-15	postive
PDHB	LINC01679	0.340157111	5.22E-15	postive
PDHB	LINC01703	0.345116738	1.97E-15	postive
PDHB	LINC01715	0.301587549	5.66E-12	postive
PDHB	LINC01719	0.457565722	3.06E-27	postive
PDHB	LINC01770	0.323360395	1.24E-13	postive
PDHB	LINC01806	0.484466409	8.67E-31	postive
PDHB	LINC01814	0.353264905	3.84E-16	postive
PDHB	LINC01816	0.310621717	1.20E-12	postive
PDHB	LINC01852	0.366675555	2.34E-17	postive
PDHB	LINC01881	0.520046094	5.44E-36	postive
PDHB	LINC01932	0.340766949	4.63E-15	postive
PDHB	LINC02012	0.320204536	2.20E-13	postive
PDHB	LINC02014	0.400772917	1.03E-20	postive
PDHB	LINC02021	0.300696654	6.58E-12	postive
PDHB	LINC02026	0.347580146	1.21E-15	postive
PDHB	LINC02122	0.376303161	2.89E-18	postive
PDHB	LINC02193	0.368263351	1.66E-17	postive
PDHB	LINC02198	0.374958523	3.88E-18	postive
PDHB	LINC02202	0.320335699	2.15E-13	postive
PDHB	LINC02210	0.393952071	5.19E-20	postive
PDHB	LINC02257	0.316110864	4.58E-13	postive
PDHB	LINC02298	0.414446452	3.56E-22	postive
PDHB	LINC02482	0.392262275	7.70E-20	postive
PDHB	LINC02542	0.312312623	8.96E-13	postive
PDHB	LINC02585	0.472826336	3.25E-29	postive
PDHB	LINC02637	0.328001835	5.27E-14	postive
PDHB	LINC02693	0.348613736	9.83E-16	postive

PDHB	LINC02749	0.354281171	3.12E-16	postive
PDHB	LINC02777	0.30334124	4.21E-12	postive
PDHB	LIPE-AS1	0.370801005	9.63E-18	postive
PDHB	LIX1L-AS1	0.303933571	3.81E-12	postive
PDHB	LMCD1-AS1	0.387991871	2.07E-19	postive
PDHB	LNCTAM34A	0.311202541	1.09E-12	postive
PDHB	LOH12CR2	0.36515259	3.24E-17	postive
PDHB	LOXL1-AS1	0.370714508	9.81E-18	postive
PDHB	LRRC37A17P	0.403963903	4.75E-21	postive
PDHB	LRRC37BP1	0.577985588	6.66E-46	postive
PDHB	MAFG-DT	0.396278724	3.00E-20	postive
PDHB	MAN1B1-DT	0.464416315	4.09E-28	postive
PDHB	MAP2K4P1	0.488231285	2.61E-31	postive
PDHB	MAP3K14-AS1	0.448850489	3.70E-26	postive
PDHB	MAP3K2-DT	0.300501806	6.80E-12	postive
PDHB	MAP4K3-DT	0.480779809	2.77E-30	postive
PDHB	MAPKAPK5-AS1	0.667029551	1.18E-65	postive
PDHB	MCM3AP-AS1	0.328442031	4.85E-14	postive
PDHB	MCPH1-AS1	0.304954901	3.20E-12	postive
PDHB	MED14OS	0.357592951	1.58E-16	postive
PDHB	MFF-DT	0.364779228	3.50E-17	postive
PDHB	MIATNB	0.334350218	1.59E-14	postive
PDHB	MID1IP1-AS1	0.359499649	1.06E-16	postive
PDHB	MIF-AS1	0.347764828	1.16E-15	postive
PDHB	MINCR	0.41099433	8.45E-22	postive
PDHB	MIPEPP3	0.405477411	3.29E-21	postive
PDHB	MIR200CHG	0.368740331	1.50E-17	postive
PDHB	MIR210HG	0.316799211	4.05E-13	postive
PDHB	MIR22HG	0.485294475	6.67E-31	postive
PDHB	MIR4435-2HG	0.359028407	1.17E-16	postive
PDHB	MIR4453HG	0.326045819	7.57E-14	postive
PDHB	MIR4458HG	0.335675616	1.24E-14	postive
PDHB	MIR4713HG	0.36283606	5.28E-17	postive
PDHB	MIR497HG	0.336323711	1.09E-14	postive
PDHB	MIR762HG	0.456729078	3.90E-27	postive
PDHB	MKLN1-AS	0.467220439	1.77E-28	postive
PDHB	MMADHC-DT	0.369872173	1.18E-17	postive
PDHB	MRPL20-AS1	0.529535239	1.74E-37	postive
PDHB	MRPL45P2	0.484005778	1.00E-30	postive
PDHB	MRPS31P4	0.433080184	2.83E-24	postive
PDHB	MYO16-AS1	0.314907361	5.67E-13	postive
PDHB	NACA4P	0.451530342	1.73E-26	postive
PDHB	NAPA-AS1	0.449582509	3.01E-26	postive
PDHB	NBPF25P	0.359340675	1.10E-16	postive
PDHB	NBPF8	0.312411116	8.81E-13	postive
PDHB	NBR2	0.439415939	5.09E-25	postive
PDHB	NCK1-DT	0.39988532	1.27E-20	postive
PDHB	NDUFB2-AS1	0.360812134	8.08E-17	postive
PDHB	NDUFV2-AS1	0.378690793	1.70E-18	postive
PDHB	NIFK-AS1	0.630919736	7.25E-57	postive

PDHB	NINJ2-AS1	0.387877865	2.13E-19	postive
PDHB	NKAPP1	0.324836351	9.45E-14	postive
PDHB	NKX2-1-AS1	0.331667409	2.65E-14	postive
PDHB	NNT-AS1	0.346926009	1.38E-15	postive
PDHB	NOP14-AS1	0.523616866	1.51E-36	postive
PDHB	NOP53-AS1	0.426234432	1.73E-23	postive
PDHB	NORAD	0.506342476	6.49E-34	postive
PDHB	NPHP3-AS1	0.309389377	1.49E-12	postive
PDHB	NRAV	0.5843768	4.03E-47	postive
PDHB	NRSN2-AS1	0.589349535	4.36E-48	postive
PDHB	NSUN5P2	0.307724144	1.99E-12	postive
PDHB	NUDT16P1	0.544084701	7.19E-40	postive
PDHB	NUP50-DT	0.323193824	1.28E-13	postive
PDHB	NUTM2A-AS1	0.622362396	5.97E-55	postive
PDHB	NUTM2B-AS1	0.384705024	4.39E-19	postive
PDHB	ODC1-DT	0.328075461	5.19E-14	postive
PDHB	OGFRP1	0.415830332	2.51E-22	postive
PDHB	OIP5-AS1	0.621740664	8.19E-55	postive
PDHB	OLMALINC	0.49658554	1.72E-32	postive
PDHB	OSER1-DT	0.487280837	3.54E-31	postive
PDHB	OSGEPL1-AS1	0.311586718	1.02E-12	postive
PDHB	OTUD6B-AS1	0.554126193	1.38E-41	postive
PDHB	PARD6G-AS1	0.334151331	1.66E-14	postive
PDHB	PARGP1	0.335114982	1.38E-14	postive
PDHB	PAXIP1-AS1	0.329660292	3.86E-14	postive
PDHB	PAXIP1-AS2	0.400570666	1.08E-20	postive
PDHB	PCAT6	0.402481138	6.81E-21	postive
PDHB	PCBP1-AS1	0.475761505	1.32E-29	postive
PDHB	PCCA-DT	0.332252599	2.37E-14	postive
PDHB	PCOTH	0.391089389	1.01E-19	postive
PDHB	PDCD4-AS1	0.355996771	2.19E-16	postive
PDHB	PDCD6IPP1	0.338647429	6.99E-15	postive
PDHB	PDCD6IPP2	0.330758965	3.14E-14	postive
PDHB	PDIA3P1	0.501489102	3.35E-33	postive
PDHB	PINK1-AS	0.412136038	6.35E-22	postive
PDHB	PIPSL	0.435282238	1.56E-24	postive
PDHB	PITPNA-AS1	0.601188337	1.86E-50	postive
PDHB	PKD1P6	0.33165592	2.66E-14	postive
PDHB	PLEKHA8P1	0.406707987	2.43E-21	postive
PDHB	PMS2CL	0.431676392	4.11E-24	postive
PDHB	PMS2P3	0.411636607	7.20E-22	postive
PDHB	PMS2P4	0.476944975	9.16E-30	postive
PDHB	POLH-AS1	0.375027163	3.83E-18	postive
PDHB	POLR2J4	0.387870097	2.13E-19	postive
PDHB	PPP1R14B-AS1	0.319755361	2.38E-13	postive
PDHB	PPP1R26-AS1	0.385784957	3.44E-19	postive
PDHB	PPP3CB-AS1	0.452195462	1.43E-26	postive
PDHB	PPT2-EGFL8	0.309260666	1.53E-12	postive
PDHB	PRELID1P1	0.490976039	1.07E-31	postive
PDHB	PRKAG2-AS1	0.313698074	7.03E-13	postive

PDHB	PRKAR2A-AS1	0.398345784	1.84E-20	postive
PDHB	PRMT5-AS1	0.308006635	1.90E-12	postive
PDHB	PRORSD1P	0.346751309	1.43E-15	postive
PDHB	PRR34	0.324920278	9.31E-14	postive
PDHB	PRR34-AS1	0.415509167	2.72E-22	postive
PDHB	PRRT3-AS1	0.427772578	1.16E-23	postive
PDHB	PSMA3-AS1	0.33645654	1.07E-14	postive
PDHB	PSMG3-AS1	0.308207987	1.83E-12	postive
PDHB	PTENP1	0.432270839	3.51E-24	postive
PDHB	PTMAP5	0.391547739	9.10E-20	postive
PDHB	PTOV1-AS1	0.489509584	1.73E-31	postive
PDHB	PVT1	0.342420178	3.35E-15	postive
PDHB	PXN-AS1	0.613931463	4.04E-53	postive
PDHB	RAB11B-AS1	0.515080208	3.16E-35	postive
PDHB	RAB30-DT	0.389651603	1.41E-19	postive
PDHB	RAD21-AS1	0.380602094	1.11E-18	postive
PDHB	RALY-AS1	0.365767926	2.84E-17	postive
PDHB	RAP2C-AS1	0.40471228	3.96E-21	postive
PDHB	RARA-AS1	0.524379231	1.15E-36	postive
PDHB	RASA4CP	0.402107854	7.45E-21	postive
PDHB	RASSF10-DT	0.34645138	1.51E-15	postive
PDHB	RASSF1-AS1	0.341834514	3.76E-15	postive
PDHB	RBM15-AS1	0.550894787	5.01E-41	postive
PDHB	RBM26-AS1	0.330133689	3.54E-14	postive
PDHB	RHPN1-AS1	0.407957876	1.79E-21	postive
PDHB	RNASEH1-AS1	0.527570165	3.59E-37	postive
PDHB	RNF216P1	0.448147544	4.51E-26	postive
PDHB	ROCK1P1	0.380666591	1.09E-18	postive
PDHB	RP11-101E13.5	0.533624292	3.83E-38	postive
PDHB	RP11-10C24.1	0.483286902	1.26E-30	postive
PDHB	RP11-10C24.2	0.386411625	2.98E-19	postive
PDHB	RP11-159G9.5	0.353761187	3.47E-16	postive
PDHB	RP11-15N24.4	0.318599631	2.94E-13	postive
PDHB	RP11-175O19.4	0.393479362	5.80E-20	postive
PDHB	RP11-226L15.5	0.45822376	2.52E-27	postive
PDHB	RP1-122P22.2	0.469620056	8.60E-29	postive
PDHB	RP11-282O18.3	0.442796557	2.01E-25	postive
PDHB	RP11-2B6.3	0.329770494	3.79E-14	postive
PDHB	RP11-314C16.1	0.384222223	4.90E-19	postive
PDHB	RP11-337C18.9	0.312330829	8.94E-13	postive
PDHB	RP11-435O5.2	0.437783535	7.94E-25	postive
PDHB	RP11-465N4.5	0.309761838	1.40E-12	postive
PDHB	RP11-539I5.1	0.435264097	1.57E-24	postive
PDHB	RP11-61L19.3	0.37441993	4.37E-18	postive
PDHB	RP11-658F2.8	0.318293106	3.10E-13	postive
PDHB	RP11-706O15.1	0.485296247	6.66E-31	postive
PDHB	RP11-815I9.4	0.316061788	4.62E-13	postive
PDHB	RP4-545L17.11	0.413412803	4.62E-22	postive
PDHB	RP5-1125A11.6	0.300932294	6.32E-12	postive
PDHB	RP9P	0.315044487	5.54E-13	postive

PDHB	RPARP-AS1	0.518774354	8.56E-36	postive
PDHB	RPL12P38	0.444212454	1.36E-25	postive
PDHB	RPL13AP3	0.457970139	2.72E-27	postive
PDHB	RPL13AP7	0.506644813	5.85E-34	postive
PDHB	RPL13P5	0.394685957	4.37E-20	postive
PDHB	RPL23AP53	0.498891258	7.99E-33	postive
PDHB	RPL23AP7	0.541394998	2.02E-39	postive
PDHB	RPL23AP82	0.57124384	1.20E-44	postive
PDHB	RPL29P2	0.330503903	3.30E-14	postive
PDHB	RPL29P30	0.350645474	6.53E-16	postive
PDHB	RPL36AP21	0.402373914	6.98E-21	postive
PDHB	RPL37P6	0.362550928	5.61E-17	postive
PDHB	RPL6P27	0.452412678	1.35E-26	postive
PDHB	RPLP1P6	0.328993521	4.38E-14	postive
PDHB	RPS10P7	0.358820617	1.22E-16	postive
PDHB	RPS18P9	0.499273792	7.03E-33	postive
PDHB	RPS27AP16	0.386498679	2.92E-19	postive
PDHB	RPS4XP16	0.394276927	4.81E-20	postive
PDHB	RPS4XP2	0.374880881	3.95E-18	postive
PDHB	RPSAP31	0.378305143	1.85E-18	postive
PDHB	RRN3P3	0.404882726	3.80E-21	postive
PDHB	RRP7BP	0.409739905	1.15E-21	postive
PDHB	RTCA-AS1	0.31869844	2.88E-13	postive
PDHB	SAMD12-AS1	0.346155736	1.61E-15	postive
PDHB	SAPCD1-AS1	0.321226492	1.83E-13	postive
PDHB	SBDSP1	0.520491175	4.64E-36	postive
PDHB	SBF2-AS1	0.481282888	2.37E-30	postive
PDHB	SBNO1-AS1	0.316011954	4.66E-13	postive
PDHB	SCAMP1-AS1	0.527087249	4.28E-37	postive
PDHB	SCAND2P	0.421202627	6.38E-23	postive
PDHB	SDAD1P1	0.467896555	1.45E-28	postive
PDHB	SDCBP2-AS1	0.42341484	3.61E-23	postive
PDHB	SDHAP1	0.343042182	2.97E-15	postive
PDHB	SEC22B4P	0.30082098	6.44E-12	postive
PDHB	SEC24B-AS1	0.365430402	3.05E-17	postive
PDHB	SEMA3B-AS1	0.314822637	5.76E-13	postive
PDHB	SEPSECS-AS1	0.421046671	6.65E-23	postive
PDHB	SEPTIN4-AS1	0.436135854	1.24E-24	postive
PDHB	SEPTIN7P2	0.331367731	2.80E-14	postive
PDHB	SERHL	0.321425421	1.76E-13	postive
PDHB	SGMS1-AS1	0.467448081	1.65E-28	postive
PDHB	SLC12A9-AS1	0.337913026	8.06E-15	postive
PDHB	SLC25A5-AS1	0.31399941	6.66E-13	postive
PDHB	SLC2A1-AS1	0.300118209	7.25E-12	postive
PDHB	SLC44A3-AS1	0.353090982	3.98E-16	postive
PDHB	SMC5-AS1	0.327910207	5.36E-14	postive
PDHB	SMG7-AS1	0.412118309	6.38E-22	postive
PDHB	SNAI3-AS1	0.4479532	4.77E-26	postive
PDHB	SNHG1	0.397877852	2.05E-20	postive
PDHB	SNHG10	0.339971919	5.41E-15	postive

PDHB	SNHG11	0.523481369	1.59E-36	postive
PDHB	SNHG14	0.317591214	3.52E-13	postive
PDHB	SNHG15	0.393610585	5.62E-20	postive
PDHB	SNHG16	0.541700097	1.80E-39	postive
PDHB	SNHG17	0.446189621	7.82E-26	postive
PDHB	SNHG20	0.336176967	1.12E-14	postive
PDHB	SNHG21	0.424942512	2.42E-23	postive
PDHB	SNHG28	0.370921452	9.38E-18	postive
PDHB	SNHG29	0.530581031	1.19E-37	postive
PDHB	SNHG3	0.339592786	5.82E-15	postive
PDHB	SNHG30	0.361737047	6.66E-17	postive
PDHB	SNHG32	0.5358731	1.65E-38	postive
PDHB	SNHG4	0.364360484	3.83E-17	postive
PDHB	SNHG5	0.443919543	1.47E-25	postive
PDHB	SNHG6	0.448169051	4.49E-26	postive
PDHB	SNHG7	0.515325061	2.90E-35	postive
PDHB	SNHG8	0.581461402	1.46E-46	postive
PDHB	SP2-AS1	0.368512512	1.58E-17	postive
PDHB	SPAG5-AS1	0.303890454	3.84E-12	postive
PDHB	SPATA41	0.329680984	3.85E-14	postive
PDHB	SPINT1-AS1	0.432972708	2.91E-24	postive
PDHB	SPRY4-AS1	0.429237991	7.85E-24	postive
PDHB	SREBF2-AS1	0.35987873	9.82E-17	postive
PDHB	SRP14-AS1	0.542256264	1.45E-39	postive
PDHB	SSR4P1	0.328801968	4.54E-14	postive
PDHB	ST20-AS1	0.392910835	6.62E-20	postive
PDHB	STAG3L2	0.427506215	1.24E-23	postive
PDHB	STAG3L3	0.354119687	3.23E-16	postive
PDHB	STAG3L4	0.497835711	1.13E-32	postive
PDHB	STARD7-AS1	0.565911651	1.13E-43	postive
PDHB	STK4-AS1	0.459243317	1.88E-27	postive
PDHB	STX17-AS1	0.542339124	1.41E-39	postive
PDHB	STX18-AS1	0.467155828	1.81E-28	postive
PDHB	SUCLA2-AS1	0.481355371	2.32E-30	postive
PDHB	SUCLG2-AS1	0.544279936	6.67E-40	postive
PDHB	SUMO2P17	0.447587423	5.28E-26	postive
PDHB	SUZ12P1	0.368030882	1.75E-17	postive
PDHB	SVIL-AS1	0.542951331	1.11E-39	postive
PDHB	SYNPR-AS1	0.425057745	2.35E-23	postive
PDHB	SYS1-DBNDD2	0.328880714	4.47E-14	postive
PDHB	TAF1A-AS1	0.470566895	6.46E-29	postive
PDHB	TAPT1-AS1	0.437825894	7.85E-25	postive
PDHB	TBC1D8-AS1	0.336084111	1.15E-14	postive
PDHB	TDRKH-AS1	0.424177336	2.96E-23	postive
PDHB	TEKT4P2	0.316321246	4.41E-13	postive
PDHB	THAP7-AS1	0.304141882	3.68E-12	postive
PDHB	THAP9-AS1	0.435469658	1.49E-24	postive
PDHB	THUMPD3-AS1	0.342311944	3.43E-15	postive
PDHB	TIPARP-AS1	0.389134744	1.59E-19	postive
PDHB	TMC3-AS1	0.375854874	3.19E-18	postive

PDHB	TMED2-DT	0.302370512	4.96E-12	postive
PDHB	TMEM161B-AS1	0.407828164	1.85E-21	postive
PDHB	TMEM198B	0.363440414	4.65E-17	postive
PDHB	TMEM202-AS1	0.403608427	5.18E-21	postive
PDHB	TMEM44-AS1	0.36046708	8.69E-17	postive
PDHB	TMEM99	0.431800942	3.98E-24	postive
PDHB	TNFRSF10A-AS1	0.315537403	5.07E-13	postive
PDHB	TOB1-AS1	0.530081664	1.43E-37	postive
PDHB	TOLLIP-AS1	0.315293124	5.30E-13	postive
PDHB	TP53TG1	0.420242848	8.17E-23	postive
PDHB	TP73-AS1	0.448082521	4.60E-26	postive
PDHB	TPM3P9	0.307894538	1.93E-12	postive
PDHB	TPT1-AS1	0.323993666	1.10E-13	postive
PDHB	TRAF3IP2-AS1	0.340983919	4.44E-15	postive
PDHB	TRAM2-AS1	0.599150843	4.84E-50	postive
PDHB	TRAPPC12-AS1	0.431910326	3.86E-24	postive
PDHB	TRIM52-AS1	0.584549895	3.74E-47	postive
PDHB	TSTD3	0.61383722	4.23E-53	postive
PDHB	TTC28-AS1	0.42038475	7.88E-23	postive
PDHB	TTC41P	0.405986264	2.90E-21	postive
PDHB	TTTY15	0.314141399	6.50E-13	postive
PDHB	TUBBP1	0.36541167	3.06E-17	postive
PDHB	TUBBP5	0.300157412	7.20E-12	postive
PDHB	TUG1	0.468280324	1.29E-28	postive
PDHB	TUSC8	0.303744745	3.93E-12	postive
PDHB	U47924.2	0.37197904	7.45E-18	postive
PDHB	U62317.1	0.362457335	5.72E-17	postive
PDHB	U62317.4	0.303719802	3.95E-12	postive
PDHB	U91328.1	0.465326984	3.12E-28	postive
PDHB	UBA6-AS1	0.559926901	1.33E-42	postive
PDHB	UBAC2-AS1	0.44936883	3.20E-26	postive
PDHB	UBE2D3-AS1	0.594431426	4.31E-49	postive
PDHB	UBL7-AS1	0.377627477	2.15E-18	postive
PDHB	UBR5-AS1	0.537304538	9.61E-39	postive
PDHB	UGDH-AS1	0.489515194	1.72E-31	postive
PDHB	UPF3AP2	0.411615489	7.24E-22	postive
PDHB	URB1-AS1	0.414507887	3.51E-22	postive
PDHB	USP27X-AS1	0.417104139	1.82E-22	postive
PDHB	USP46-AS1	0.420226496	8.20E-23	postive
PDHB	UXT-AS1	0.414110411	3.88E-22	postive
PDHB	VASH1-AS1	0.366772587	2.29E-17	postive
PDHB	VPS13B-DT	0.395840003	3.33E-20	postive
PDHB	VPS33B-DT	0.31536231	5.23E-13	postive
PDHB	WAC-AS1	0.532380642	6.08E-38	postive
PDHB	WARS2-AS1	0.392767644	6.85E-20	postive
PDHB	WASH3P	0.435263464	1.57E-24	postive
PDHB	WDFY3-AS2	0.327487717	5.79E-14	postive
PDHB	WEE2-AS1	0.373905524	4.89E-18	postive
PDHB	WHAMMP2	0.319794853	2.37E-13	postive
PDHB	WHAMMP3	0.333811698	1.77E-14	postive

PDHB	WWC2-AS2	0.318025003	3.26E-13	postive
PDHB	YTHDF3-AS1	0.337635328	8.50E-15	postive
PDHB	Z83851.4	0.417877822	1.49E-22	postive
PDHB	Z92544.2	0.327337421	5.96E-14	postive
PDHB	Z95115.1	0.329386418	4.07E-14	postive
PDHB	Z97634.1	0.336491975	1.06E-14	postive
PDHB	Z99572.1	0.419967059	8.77E-23	postive
PDHB	ZBED3-AS1	0.306368073	2.51E-12	postive
PDHB	ZBED5-AS1	0.457836383	2.83E-27	postive
PDHB	ZBTB11-AS1	0.54268269	1.23E-39	postive
PDHB	ZBTB44-DT	0.418350864	1.33E-22	postive
PDHB	ZFAS1	0.436785661	1.04E-24	postive
PDHB	ZNF205-AS1	0.376956676	2.50E-18	postive
PDHB	ZNF213-AS1	0.381770785	8.53E-19	postive
PDHB	ZNF236-DT	0.379657346	1.37E-18	postive
PDHB	ZNF252P	0.594459542	4.25E-49	postive
PDHB	ZNF271P	0.507458162	4.43E-34	postive
PDHB	ZNF337-AS1	0.413172629	4.90E-22	postive
PDHB	ZNF341-AS1	0.313610348	7.13E-13	postive
PDHB	ZNF433-AS1	0.313007439	7.93E-13	postive
PDHB	ZNF503-AS1	0.333677848	1.81E-14	postive
PDHB	ZNF503-AS2	0.454639745	7.12E-27	postive
PDHB	ZNF529-AS1	0.370574134	1.01E-17	postive
PDHB	ZNF542P	0.342291798	3.44E-15	postive
PDHB	ZNF56	0.378925553	1.61E-18	postive
PDHB	ZNF561-AS1	0.580863614	1.90E-46	postive
PDHB	ZNF582-AS1	0.309844549	1.38E-12	postive
PDHB	ZNF649-AS1	0.300127949	7.24E-12	postive
PDHB	ZNF658B	0.327894034	5.37E-14	postive
PDHB	ZNF687-AS1	0.412057542	6.48E-22	postive
PDHB	ZNF767P	0.308555622	1.72E-12	postive
PDHB	ZNF788P	0.365729903	2.86E-17	postive
PDHB	ZNF790-AS1	0.302436397	4.91E-12	postive
PDHB	ZNF793-AS1	0.341546463	3.98E-15	postive
PDHB	ZNF815P	0.356343698	2.04E-16	postive
PDHB	ZNRD2-AS1	0.407671508	1.92E-21	postive
PDHB	ZSCAN16-AS1	0.534080635	3.23E-38	postive
RNF7	A2M-AS1	0.309704119	1.41E-12	postive
RNF7	ABALON	0.304046401	3.74E-12	postive
RNF7	ABHD11-AS1	0.390380062	1.19E-19	postive
RNF7	AC000068.1	0.300692598	6.58E-12	postive
RNF7	AC002116.2	0.489512918	1.73E-31	postive
RNF7	AC002310.1	0.376187671	2.96E-18	postive
RNF7	AC002310.2	0.353656259	3.55E-16	postive
RNF7	AC002467.1	0.602192999	1.16E-50	postive
RNF7	AC002470.1	0.457473508	3.14E-27	postive
RNF7	AC003965.1	0.313849186	6.84E-13	postive
RNF7	AC004130.2	0.375009685	3.84E-18	postive
RNF7	AC004241.1	0.525104665	8.81E-37	postive
RNF7	AC004241.3	0.443539658	1.64E-25	postive

RNF7	AC004477.1	0.352616555	4.38E-16	postive
RNF7	AC004596.1	0.364692182	3.57E-17	postive
RNF7	AC004707.2	0.310246287	1.29E-12	postive
RNF7	AC004801.6	0.399645186	1.35E-20	postive
RNF7	AC004803.1	0.37495646	3.89E-18	postive
RNF7	AC004812.2	0.421287631	6.25E-23	postive
RNF7	AC004816.1	0.303200404	4.31E-12	postive
RNF7	AC004846.1	0.333720754	1.80E-14	postive
RNF7	AC004854.2	0.378399119	1.81E-18	postive
RNF7	AC004943.2	0.374991394	3.86E-18	postive
RNF7	AC005005.3	0.314508013	6.09E-13	postive
RNF7	AC005034.4	0.365676786	2.89E-17	postive
RNF7	AC005037.1	0.389031375	1.63E-19	postive
RNF7	AC005076.2	0.39691082	2.58E-20	postive
RNF7	AC005083.1	0.310843231	1.16E-12	postive
RNF7	AC005229.4	0.549276359	9.49E-41	postive
RNF7	AC005261.4	0.357219301	1.71E-16	postive
RNF7	AC005280.1	0.349407605	8.38E-16	postive
RNF7	AC005288.1	0.337523159	8.69E-15	postive
RNF7	AC005332.4	0.320626537	2.04E-13	postive
RNF7	AC005476.2	0.327064971	6.27E-14	postive
RNF7	AC005498.2	0.302035277	5.25E-12	postive
RNF7	AC005520.4	0.39393148	5.21E-20	postive
RNF7	AC005562.1	0.46806812	1.37E-28	postive
RNF7	AC005586.1	0.317088191	3.85E-13	postive
RNF7	AC005670.3	0.39234955	7.55E-20	postive
RNF7	AC005829.1	0.31445092	6.15E-13	postive
RNF7	AC005837.1	0.41091228	8.62E-22	postive
RNF7	AC005840.4	0.307297465	2.14E-12	postive
RNF7	AC006001.2	0.363330707	4.76E-17	postive
RNF7	AC006122.1	0.34571665	1.75E-15	postive
RNF7	AC006213.1	0.366345147	2.51E-17	postive
RNF7	AC006252.1	0.337050206	9.51E-15	postive
RNF7	AC006333.1	0.497152055	1.42E-32	postive
RNF7	AC006449.2	0.33066442	3.20E-14	postive
RNF7	AC006449.5	0.388305408	1.93E-19	postive
RNF7	AC006449.7	0.502354223	2.51E-33	postive
RNF7	AC006504.5	0.330725781	3.16E-14	postive
RNF7	AC006547.1	0.301237673	6.01E-12	postive
RNF7	AC007032.1	0.441121052	3.19E-25	postive
RNF7	AC007114.1	0.383258477	6.10E-19	postive
RNF7	AC007228.9	0.324229682	1.06E-13	postive
RNF7	AC007318.3	0.338110773	7.76E-15	postive
RNF7	AC007342.4	0.353528007	3.64E-16	postive
RNF7	AC007383.2	0.364099467	4.05E-17	postive
RNF7	AC007541.1	0.366166927	2.61E-17	postive
RNF7	AC007773.1	0.392096756	8.01E-20	postive
RNF7	AC007823.1	0.363480917	4.61E-17	postive
RNF7	AC007881.3	0.339786399	5.61E-15	postive
RNF7	AC007998.3	0.30736708	2.12E-12	postive

RNF7	AC008014.1	0.421317625	6.20E-23	postive
RNF7	AC008035.1	0.335306027	1.33E-14	postive
RNF7	AC008105.2	0.328997165	4.37E-14	postive
RNF7	AC008267.5	0.483553155	1.16E-30	postive
RNF7	AC008393.1	0.310765974	1.17E-12	postive
RNF7	AC008438.1	0.38480337	4.30E-19	postive
RNF7	AC008443.1	0.428541544	9.44E-24	postive
RNF7	AC008443.5	0.400734109	1.04E-20	postive
RNF7	AC008494.3	0.401751645	8.12E-21	postive
RNF7	AC008608.2	0.476695022	9.89E-30	postive
RNF7	AC008622.2	0.301786229	5.48E-12	postive
RNF7	AC008747.1	0.366959256	2.20E-17	postive
RNF7	AC008771.1	0.448253282	4.38E-26	postive
RNF7	AC008915.3	0.505157434	9.71E-34	postive
RNF7	AC008946.1	0.429978501	6.45E-24	postive
RNF7	AC008966.1	0.415611595	2.66E-22	postive
RNF7	AC009005.1	0.345392044	1.87E-15	postive
RNF7	AC009061.2	0.437899491	7.70E-25	postive
RNF7	AC009118.2	0.434529798	1.91E-24	postive
RNF7	AC009126.1	0.402520431	6.74E-21	postive
RNF7	AC009171.2	0.465962286	2.58E-28	postive
RNF7	AC009226.1	0.332207989	2.39E-14	postive
RNF7	AC009237.14	0.485448368	6.35E-31	postive
RNF7	AC009275.1	0.335492618	1.28E-14	postive
RNF7	AC009309.2	0.457362372	3.24E-27	postive
RNF7	AC009403.1	0.414279357	3.71E-22	postive
RNF7	AC009506.2	0.399694827	1.33E-20	postive
RNF7	AC009630.1	0.306745616	2.36E-12	postive
RNF7	AC009690.2	0.327558938	5.72E-14	postive
RNF7	AC009779.3	0.530019076	1.46E-37	postive
RNF7	AC009831.1	0.419383347	1.02E-22	postive
RNF7	AC009948.3	0.376173139	2.97E-18	postive
RNF7	AC009961.1	0.358092686	1.42E-16	postive
RNF7	AC010173.1	0.492554655	6.43E-32	postive
RNF7	AC010186.2	0.392808804	6.78E-20	postive
RNF7	AC010280.2	0.309793523	1.39E-12	postive
RNF7	AC010319.4	0.304457063	3.48E-12	postive
RNF7	AC010320.2	0.356331609	2.05E-16	postive
RNF7	AC010359.2	0.31450827	6.09E-13	postive
RNF7	AC010442.1	0.391292758	9.66E-20	postive
RNF7	AC010504.1	0.427465731	1.25E-23	postive
RNF7	AC010531.6	0.346078986	1.63E-15	postive
RNF7	AC010595.1	0.318575624	2.95E-13	postive
RNF7	AC010655.4	0.433649395	2.43E-24	postive
RNF7	AC010834.2	0.359822987	9.94E-17	postive
RNF7	AC010894.2	0.462577729	7.05E-28	postive
RNF7	AC010913.1	0.37700986	2.47E-18	postive
RNF7	AC010969.2	0.315280389	5.31E-13	postive
RNF7	AC011290.1	0.309596171	1.44E-12	postive
RNF7	AC011451.1	0.337189668	9.26E-15	postive

RNF7	AC011603.3	0.416320031	2.22E-22	postive
RNF7	AC012063.1	0.408742868	1.48E-21	postive
RNF7	AC012073.1	0.356527378	1.97E-16	postive
RNF7	AC012146.1	0.486342594	4.77E-31	postive
RNF7	AC012184.3	0.371008186	9.20E-18	postive
RNF7	AC012306.2	0.451153036	1.93E-26	postive
RNF7	AC012442.3	0.433807813	2.32E-24	postive
RNF7	AC012467.2	0.34005742	5.32E-15	postive
RNF7	AC012555.1	0.371114152	9.00E-18	postive
RNF7	AC012603.1	0.322764889	1.38E-13	postive
RNF7	AC012640.4	0.538525255	6.05E-39	postive
RNF7	AC012640.6	0.306740118	2.36E-12	postive
RNF7	AC012676.1	0.34365873	2.63E-15	postive
RNF7	AC015912.3	0.34680715	1.41E-15	postive
RNF7	AC015917.2	0.351130623	5.92E-16	postive
RNF7	AC015922.3	0.343451332	2.74E-15	postive
RNF7	AC015982.2	0.483177004	1.30E-30	postive
RNF7	AC016027.1	0.47032648	6.95E-29	postive
RNF7	AC016065.1	0.449469621	3.11E-26	postive
RNF7	AC016355.1	0.42546148	2.12E-23	postive
RNF7	AC016394.2	0.495037373	2.86E-32	postive
RNF7	AC016405.2	0.324303115	1.04E-13	postive
RNF7	AC016405.3	0.423638904	3.40E-23	postive
RNF7	AC016727.1	0.354555938	2.95E-16	postive
RNF7	AC016747.1	0.470076451	7.49E-29	postive
RNF7	AC016876.2	0.416751692	1.99E-22	postive
RNF7	AC016876.3	0.398938798	1.59E-20	postive
RNF7	AC016888.1	0.467898495	1.44E-28	postive
RNF7	AC018521.5	0.523964683	1.33E-36	postive
RNF7	AC018647.2	0.326840261	6.53E-14	postive
RNF7	AC019069.1	0.400335406	1.14E-20	postive
RNF7	AC019294.2	0.330803639	3.12E-14	postive
RNF7	AC020636.1	0.314445537	6.16E-13	postive
RNF7	AC020663.2	0.445875997	8.54E-26	postive
RNF7	AC020765.2	0.30499768	3.18E-12	postive
RNF7	AC020904.2	0.351198943	5.84E-16	postive
RNF7	AC020910.5	0.384554869	4.55E-19	postive
RNF7	AC020915.3	0.421852412	5.40E-23	postive
RNF7	AC021028.1	0.312911275	8.07E-13	postive
RNF7	AC021483.2	0.306767158	2.35E-12	postive
RNF7	AC022007.1	0.312770391	8.27E-13	postive
RNF7	AC022092.1	0.336067052	1.15E-14	postive
RNF7	AC022098.3	0.351615144	5.37E-16	postive
RNF7	AC022167.4	0.450801702	2.13E-26	postive
RNF7	AC022211.3	0.37335859	5.52E-18	postive
RNF7	AC022217.4	0.30485022	3.26E-12	postive
RNF7	AC022382.1	0.347038531	1.35E-15	postive
RNF7	AC022613.2	0.359778077	1.00E-16	postive
RNF7	AC022916.2	0.360632579	8.39E-17	postive
RNF7	AC023043.1	0.424882011	2.46E-23	postive

RNF7	AC023043.4	0.372846857	6.17E-18	postive
RNF7	AC023090.1	0.34503359	2.01E-15	postive
RNF7	AC023509.3	0.382200035	7.75E-19	postive
RNF7	AC023813.3	0.329706712	3.83E-14	postive
RNF7	AC024243.1	0.352257442	4.71E-16	postive
RNF7	AC024267.7	0.312609309	8.51E-13	postive
RNF7	AC024361.2	0.385671487	3.53E-19	postive
RNF7	AC024575.2	0.372118014	7.23E-18	postive
RNF7	AC025048.2	0.381195578	9.71E-19	postive
RNF7	AC025048.4	0.485545168	6.15E-31	postive
RNF7	AC025181.2	0.358996293	1.18E-16	postive
RNF7	AC025287.2	0.348159455	1.08E-15	postive
RNF7	AC025682.2	0.35350549	3.66E-16	postive
RNF7	AC026304.1	0.52260694	2.17E-36	postive
RNF7	AC026401.3	0.403165783	5.77E-21	postive
RNF7	AC026412.1	0.316173543	4.53E-13	postive
RNF7	AC026471.1	0.416498796	2.12E-22	postive
RNF7	AC026691.1	0.339482421	5.95E-15	postive
RNF7	AC026801.2	0.342130782	3.55E-15	postive
RNF7	AC027097.1	0.324508992	1.00E-13	postive
RNF7	AC027237.5	0.340359639	5.02E-15	postive
RNF7	AC027307.2	0.358061453	1.43E-16	postive
RNF7	AC027319.1	0.323559275	1.19E-13	postive
RNF7	AC027559.1	0.370712202	9.81E-18	postive
RNF7	AC027644.3	0.448855397	3.70E-26	postive
RNF7	AC027682.6	0.321069032	1.88E-13	postive
RNF7	AC034102.5	0.31114524	1.10E-12	postive
RNF7	AC034199.1	0.341760772	3.82E-15	postive
RNF7	AC036176.1	0.423001386	4.01E-23	postive
RNF7	AC040169.1	0.326371632	7.12E-14	postive
RNF7	AC040970.1	0.406740874	2.41E-21	postive
RNF7	AC046134.2	0.591835617	1.41E-48	postive
RNF7	AC046143.1	0.434709503	1.82E-24	postive
RNF7	AC046143.2	0.450274354	2.48E-26	postive
RNF7	AC053503.1	0.316628061	4.18E-13	postive
RNF7	AC053503.4	0.380309784	1.18E-18	postive
RNF7	AC060766.7	0.307053661	2.23E-12	postive
RNF7	AC061992.2	0.322611022	1.42E-13	postive
RNF7	AC064807.1	0.504100976	1.39E-33	postive
RNF7	AC064836.3	0.351299816	5.72E-16	postive
RNF7	AC067852.2	0.557191822	4.04E-42	postive
RNF7	AC068338.2	0.562064548	5.54E-43	postive
RNF7	AC068473.5	0.321646102	1.69E-13	postive
RNF7	AC068631.1	0.309679052	1.42E-12	postive
RNF7	AC068790.8	0.342734443	3.15E-15	postive
RNF7	AC068831.2	0.386407662	2.98E-19	postive
RNF7	AC068870.2	0.431781828	4.00E-24	postive
RNF7	AC069213.1	0.351130575	5.92E-16	postive
RNF7	AC069528.1	0.32448593	1.01E-13	postive
RNF7	AC069544.1	0.348559127	9.94E-16	postive

RNF7	AC073195.2	0.379598267	1.39E-18	postive
RNF7	AC073332.1	0.326222174	7.32E-14	postive
RNF7	AC073508.3	0.474426023	1.99E-29	postive
RNF7	AC073529.1	0.477279895	8.25E-30	postive
RNF7	AC073569.2	0.404292506	4.39E-21	postive
RNF7	AC073585.1	0.353419689	3.72E-16	postive
RNF7	AC073611.1	0.529021469	2.11E-37	postive
RNF7	AC073896.2	0.552625844	2.52E-41	postive
RNF7	AC073896.4	0.310860889	1.16E-12	postive
RNF7	AC074044.1	0.342686235	3.18E-15	postive
RNF7	AC074117.1	0.303011574	4.45E-12	postive
RNF7	AC078802.1	0.392667328	7.01E-20	postive
RNF7	AC079145.1	0.303944977	3.80E-12	postive
RNF7	AC079209.1	0.322313927	1.50E-13	postive
RNF7	AC079305.2	0.368427071	1.61E-17	postive
RNF7	AC079610.1	0.351737309	5.24E-16	postive
RNF7	AC079807.1	0.380252172	1.20E-18	postive
RNF7	AC079848.1	0.393687291	5.52E-20	postive
RNF7	AC079921.2	0.315801064	4.84E-13	postive
RNF7	AC079922.2	0.459298365	1.85E-27	postive
RNF7	AC080013.5	0.3343823	1.58E-14	postive
RNF7	AC080037.2	0.328341181	4.94E-14	postive
RNF7	AC080112.2	0.46201251	8.33E-28	postive
RNF7	AC083798.2	0.347649964	1.19E-15	postive
RNF7	AC083799.1	0.684354465	2.51E-70	postive
RNF7	AC083880.1	0.447454171	5.49E-26	postive
RNF7	AC084036.1	0.302570499	4.80E-12	postive
RNF7	AC084125.4	0.380015357	1.27E-18	postive
RNF7	AC084757.3	0.319527394	2.48E-13	postive
RNF7	AC084809.1	0.380774498	1.07E-18	postive
RNF7	AC087482.1	0.310981885	1.13E-12	postive
RNF7	AC087521.3	0.317225369	3.76E-13	postive
RNF7	AC087645.2	0.346833801	1.40E-15	postive
RNF7	AC087741.2	0.335593701	1.26E-14	postive
RNF7	AC090114.2	0.364541609	3.68E-17	postive
RNF7	AC090204.1	0.323938174	1.11E-13	postive
RNF7	AC090409.1	0.357493673	1.61E-16	postive
RNF7	AC090425.2	0.311560321	1.02E-12	postive
RNF7	AC090515.3	0.407748312	1.88E-21	postive
RNF7	AC090587.1	0.46824978	1.30E-28	postive
RNF7	AC090617.5	0.438346969	6.81E-25	postive
RNF7	AC090825.1	0.348359	1.03E-15	postive
RNF7	AC090912.1	0.445834493	8.63E-26	postive
RNF7	AC090970.2	0.43722862	9.23E-25	postive
RNF7	AC091057.1	0.361415728	7.12E-17	postive
RNF7	AC091153.3	0.388544514	1.82E-19	postive
RNF7	AC091271.1	0.4670414	1.87E-28	postive
RNF7	AC091390.5	0.336124598	1.14E-14	postive
RNF7	AC091729.3	0.53902546	5.00E-39	postive
RNF7	AC091982.3	0.454147689	8.20E-27	postive

RNF7	AC092115.4	0.363541926	4.55E-17	postive
RNF7	AC092118.1	0.330019196	3.61E-14	postive
RNF7	AC092295.2	0.359143973	1.14E-16	postive
RNF7	AC092368.3	0.493025486	5.52E-32	postive
RNF7	AC092687.3	0.303932074	3.81E-12	postive
RNF7	AC092718.2	0.303986905	3.77E-12	postive
RNF7	AC092718.4	0.447504709	5.41E-26	postive
RNF7	AC092803.1	0.339190188	6.29E-15	postive
RNF7	AC092903.2	0.352288959	4.68E-16	postive
RNF7	AC092910.3	0.390153498	1.26E-19	postive
RNF7	AC092944.1	0.397276432	2.37E-20	postive
RNF7	AC093157.1	0.467000415	1.89E-28	postive
RNF7	AC093249.6	0.303254359	4.27E-12	postive
RNF7	AC093520.1	0.334032899	1.69E-14	postive
RNF7	AC093627.5	0.316288389	4.44E-13	postive
RNF7	AC093673.1	0.581503322	1.43E-46	postive
RNF7	AC096536.2	0.488166871	2.66E-31	postive
RNF7	AC096649.2	0.353464521	3.69E-16	postive
RNF7	AC096733.3	0.342933018	3.03E-15	postive
RNF7	AC096992.2	0.329495078	3.99E-14	postive
RNF7	AC097358.2	0.360085727	9.41E-17	postive
RNF7	AC097359.2	0.453244533	1.06E-26	postive
RNF7	AC097468.3	0.302047953	5.24E-12	postive
RNF7	AC097639.1	0.319900612	2.32E-13	postive
RNF7	AC098484.1	0.328043009	5.23E-14	postive
RNF7	AC098484.2	0.394297868	4.78E-20	postive
RNF7	AC098487.1	0.430324657	5.89E-24	postive
RNF7	AC098820.2	0.358275153	1.37E-16	postive
RNF7	AC098934.1	0.3138683	6.82E-13	postive
RNF7	AC099066.2	0.309284695	1.52E-12	postive
RNF7	AC099343.4	0.446800261	6.59E-26	postive
RNF7	AC099518.4	0.304820913	3.28E-12	postive
RNF7	AC099522.2	0.343583238	2.67E-15	postive
RNF7	AC099548.2	0.348017161	1.11E-15	postive
RNF7	AC099778.1	0.32551208	8.35E-14	postive
RNF7	AC099850.2	0.498349437	9.56E-33	postive
RNF7	AC099850.4	0.394347168	4.73E-20	postive
RNF7	AC100810.1	0.335769394	1.22E-14	postive
RNF7	AC100847.1	0.398154783	1.92E-20	postive
RNF7	AC100861.1	0.438652951	6.27E-25	postive
RNF7	AC103760.1	0.376032949	3.07E-18	postive
RNF7	AC104109.2	0.350713878	6.44E-16	postive
RNF7	AC104187.1	0.325272631	8.72E-14	postive
RNF7	AC104411.2	0.31190447	9.63E-13	postive
RNF7	AC104653.1	0.536793448	1.17E-38	postive
RNF7	AC104667.2	0.517088854	1.56E-35	postive
[truncated: 557,493 more chars]
